# Akt isoform-specific effects on thyroid cancer development and progression in a murine thyroid cancer model

**DOI:** 10.1038/s41598-020-75529-0

**Published:** 2020-10-27

**Authors:** Motoyasu Saji, Caroline S. Kim, Chaojie Wang, Xiaoli Zhang, Tilak Khanal, Kevin Coombes, Krista La Perle, Sheue-Yann Cheng, Philip N. Tsichlis, Matthew D. Ringel

**Affiliations:** 1 Division of Endocrinology, Diabetes, and Metabolism, The Ohio State University College of Medicine and Arthur G. James Comprehensive Cancer Center, 506 Biomedical Research Tower, 560 West 12th Avenue, Columbus, OH 43210 USA; 2 Division of Endocrinology, University of Pennsylvania, Philadelphia, PA USA; 3 Center for Biostatistics, The Ohio State University College of Medicine and Arthur G. James Comprehensive Cancer Center, Columbus, OH USA; 4 Department of Biostatistics and Bionformatics, The Ohio State University College of Medicine and Arthur G. James Comprehensive Cancer Center, Columbus, OH USA; 5 College of Veterinary Medicine, The Ohio State University, Columbus, OH USA; 6 National Cancer Institute, National Institutes of Health, Bethesda, MD USA; 7 Department of Cancer Biology and Genetics, The Ohio State University College of Medicine and Arthur G. James Comprehensive Cancer Center, Columbus, OH USA

**Keywords:** Cancer, Endocrinology

## Abstract

The Akt family is comprised of three unique homologous proteins with isoform-specific effects, but isoform-specific in vivo data are limited in follicular thyroid cancer (FTC), a PI3 kinase-driven tumor. Prior studies demonstrated that PI3K/Akt signaling is important in thyroid hormone receptor β^PV/PV^ knock-in (PV) mice that develop metastatic thyroid cancer that most closely resembles FTC. To determine the roles of Akt isoforms in this model we crossed Akt1^−/−^, Akt2^−/−^, and Akt3^−/−^ mice with PV mice. Over 12 months, thyroid size was reduced for the Akt null crosses (p < 0.001). Thyroid cancer development and local invasion were delayed in only the PVPV-Akt1 knock out (KO) mice in association with increased apoptosis with no change in proliferation. Primary-cultured PVPV-Akt1KO thyrocytes uniquely displayed a reduced cell motility. In contrast, loss of any Akt isoform reduced lung metastasis while vascular invasion was reduced with Akt1 or 3 loss. Microarray of thyroid RNA displayed incomplete overlap between the Akt KO models. The most upregulated gene was the dendritic cell (DC) marker CD209a only in PVPV-Akt1KO thyroids. Immunohistochemistry demonstrated an increase in CD209a-expressing cells in the PVPV-Akt1KO thyroids. In summary, Akt isoforms exhibit common and differential functions that regulate local and metastatic progression in this model of thyroid cancer.

## Introduction

The Akt proteins are a family of serine-threonine kinases that are critical signaling nodes in the phosphoinositide-3 (OH) kinase (PI3K) signaling cascade. Akt isoforms regulate cellular proliferation, apoptosis, motility, and metabolism^1^. Many cancers develop or are driven by PI3K-Akt signaling, making it an important target for cancer prevention and treatment^1–5^.

Akt1, 2, and 3 are encoded by unique but highly homologous genes^1^. Isoform-specific cellular localization, substrates, and functions have been demonstrated in vitro with results suggesting both isoform and cell type-specific effects^6–9^. Differences in function of Akt isoforms have been also reported in vivo using isoform-specific knockout mouse models. Generalized Akt1 loss results primarily in mitogenic changes leading to small mice; Akt2 null mice are characterized by relatively normal size but develop insulin resistance and diabetes; and Akt3 loss leads to mice of normal size with normal insulin sensitivity but impaired brain development and neurological dysfunction^10–14^.

Akt isoform-specific functions also have been reported in cancer models. For example, in a mouse breast cancer model induced by polyoma middle T (PyMT) or ErbB2/Neu, loss of Akt1 significantly delayed tumor induction while loss of Akt2 accelerated this endpoint. However, invasiveness of the primary tumors was enhanced with Akt1 loss and it was reduced with Akt2 loss^15^. In addition, tissue-specific overexpression of constitutively active Akt1 accelerates breast cancer development induced by Erb2/Neu or PyMT but does not change the frequency of lung metastases. In contrast, constitutively active Akt2 slows breast cancer development but accelerates invasion and recurrence in this model^16,17^. Therefore, Akt2 appears to be a driver of invasiveness and metastasis while Akt1 appears to be a driver of growth in these systems. RPPA studies addressing the expression and phosphorylation of the three Akt isoforms in human cancer cell lines revealed that the relative abundance of expression and the relative level of activation of these isoforms varies between cell lines^9^. The combination of the unique non-redundant biology elicited by the different Akt isoforms, and their unique pattern of expression and activation in different tumors suggests that inhibition of individual isoforms has potential to be a more optimal therapeutic strategy for human cancer than the currently employed generalized Akt inhibition^2,4,5^.

PI3K-Akt signaling has been linked primarily to follicular thyroid cancer tumorigenesis (FTC) although Akt activation contributes to the progression of all forms of the disease^18,19^. FTC is a primary component in Cowden syndrome caused by germline loss of PTEN. In addition, in sporadic thyroid cancer, Akt can be activated by a variety of mechanisms including somatic loss of PTEN expression, mutational activation of PI3K (PIK3CA), Akt1, and the expression of RET fusion proteins and RAS oncogenes^20,21^. An increase in nuclear phosphorylated Akt levels is associated with increased tumor cell invasiveness. Importantly, it correlates with the nuclear localization of Akt1, which is due to impaired nuclear export^22^. PI3K-Akt signaling has been linked to epithelial-to-mesenchymal transition in aggressive thyroid cancers regardless of initiating oncogene^23,24^ and is one of three pathways with enhanced representation in the invasive tumor fronts.

Genetically engineered mouse models with loss of Pten develop goiter and in some cases, thyroid cancers with variable frequencies ranging from 0 to 65% depending on the genetic background^25–27^ in an Akt1^28^ or Akt2-dependent manner^29^. Because this model rarely develops metastases, it is not optimal to assess the impact of Akt in cancer metastatic progression. One model for this purpose may be the thyroid hormone receptor TRß PV knock-in mouse model. TRβ1 is one member of the TR family (TRα1 TRβ1, TRβ2 and TRβ3) that is part of the steroid receptor/retinoic acid nuclear receptor superfamily. The TRβ PV mice were generated by knocking into the TRβ1 locus the inactivating PV mutation that was identified in a patient with generalized thyroid hormone resistance (RTH). The knock-in mice, similar to RTH patients, are characterized by high levels of circulating thyroid hormones and TSH, a predominantly hypothyroid phenotype, and thyroid papillary hyperplasia. They unexpectedly developed invasive follicular-like thyroid carcinomas with well-differentiated distant metastases that can have dedifferentiated cellular features over time. Distant metastases are an uncommon event in endogenous thyroid cancer models induced by a single genetic change^30^.

Similar to human thyroid cancer we demonstrated that the thyroid cancer in these mice are characterized by activated PI3K signaling and nuclear Akt1 expression and activation, which occurs via sequestration of the PI3K regulatory subunit by the mutant TRβ^31–33^. To determine whether Akt1 regulates tumor invasion this model, we crossed TRß PV mice with Akt1 knock out mice^11^ and showed that depletion of Akt1 delayed cancer development and reduced the development of lung metastasis^34^. Thus, Akt1 is critical for cancer induction and metastasis in this model of thyroid cancer. However, the impact of Akt2 or 3 loss had not been tested directly prior to the present study.

The goal of the present study was to compare directly the roles of all three Akt isoforms on the progression of thyroid cancer in TRßPV mice. Our results demonstrated that while all Akt isoforms regulate thyroid growth, Akt1 was the only isoform whose loss delayed tumor induction and local invasion. However, distant metastases were impaired by loss of all three Akt isoforms. The loss of Akt1 and Akt3 had a stronger inhibitory effect than the loss of Akt2 on vascular invasion. Microarray data and subsequent immunohistochemistry (IHC) identified potential Akt1-dependent gene expression differences in tumor-associated dendritic cells (DCs) in the primary tumors. Taken together, these data confirmed the central role of Akt1 in thyroid cancer induction and progression, identified a new role for Akt 2 and 3 in metastasis and uncovered a potential new Akt1-mediated DC suppression pathway in thyroid cancer progression in this model system.

## Materials and methods

### Mice

All mouse studies were performed as part of an approved OSU IACUC protocol and in accordance with relevant guidelines and regulations. Mice heterozygous for Akt1 loss (Akt1^+/−^) were obtained from Jackson Laboratory (stock #004912, Bar Harbor, MI)^11^. Akt2 and Akt3 knock out mice were the generous gifts of M. Birnbaum (University of Pennsylvania, Philadelphia, PA)^12^ and T. Ludwig (Columbia University, NY)^35^, respectively. Homozygous or heterozygous mice were mated with heterozygous of TRßPV (TRß^PV/−^)^36^ to create homozygous TRßPV (TRß^PV/PV^) and TRß^PV/PV^ with isoform-specific Akt^−/−^ (AktKO) mice. We use the term PVPV-AktWT for TRß^PV/PV^, PVPV-Akt1KO for TRß^PV/PV^-Akt1^−/−^, PVPV-Akt2KO for TRß^PV/PV^-Akt2^−/−^, and PVPV-Akt3KO for TRß^PV/PV^-Akt3^−/−^.

For genotyping, crude DNA was isolated from tail biopsies by using DNeasy Blood & Tissue kit (Qiagen, Germantown, MD) and PCR was performed using specific primers (Supplemental Table 1)^11,35,36^. Mice were sacrificed at approximately 3, 6, 9, and 12 months of age, body weight was measured, and the thyroid with trachea, lung, pituitary gland, and liver were harvested, and fixed in 10% zinc formalin (Thermo Fisher, Inc., Waltham, MA) or stored in − 80 freezer for later isolation of protein and RNA. When thyroids were isolated from mice, photographs were taken permitting the longest and the shortest lengths to be recorded later by using enlarged photos, and thyroid volume was calculated as described using [Volume] = [Longest length]  ×  [Shortest length]^2^ × 0.52^34^.

### Human thyroid tissues

Deidentified tissue samples used for IHC staining for Adrenal medullin 2 immunohistochemistry were obtained with written informed consent from patients using an Ohio Sate Univervsity Institutional Review Board-approved protocol in accordance with all relevant guidelines and regulations.

### Cell motility assay

Thyroid cells were isolated as noted in the “Supplemental Materials and Methods” and used for assays within 2–4 days. Cell migration was measured using a modified procedure from previously reported methods^34,37^ using a Boyden chamber. Cell invasion was examined as described^38^. The details of methods are in “Supplemental Materials and Methods”.

### Histology, immunohistochemistry, and immunofluorescence

Thyroid, lung and pituitary glands 3 days after in 10% zinc formalin were embedded in paraffin, and 4-µm-sections were made by the Comparative Pathology & Mouse Phenotyping Shared Resource (CPMPSR) in the Ohio State University Comprehensive Cancer Center. Hematoxylin and eosin (H&E) stained slides were reviewed by a board certified veterinary pathologist (KLP). When metastases were not immediately identified in lung tissues, an additional ten sections were cut and H&E stained to maximize sensitivity. The details of materials and methods for immunohistochemistry (IHC) and immunofluorescence are described in “Supplemental Materials and Methods”, Supplemental Table 2, and previously^34^. Quantitation of IHC staining was performed by using inForm software version 2.3.0, (https://www.perkinelmer.com/Content/LST_Software_Downloads/inFormUserManual_2_3_0_rev1.pdf) or by manual counting of low and high powered fields (for CD209a) in which IHC was considered positive if intensity was 2+ or greater on a scale of 0–3+ and if the staining was positive in > 50% of the cellular volume.

### TSH measurement

Intracardiac blood was drawn during necropsy, and serum was collected after centrifugation of blood at 3000 × *g* for 15 min and stored at − 80 °C until the assay was performed. Serum TSH levels were determined by using Mouse Thyroid Stimulating ELISA (MBS269190, MyBioSource, San Diego, CA). Since high TSH levels occur in PVPV mice^36^, serum was diluted 100× with ELISA buffer.

### RNA isolation and analysis

One thyroid lobe was homogenized in 1 ml of Trizol (Thermo Fisher Scientific, Inc.), and stored at − 80 °C isolation. RNA was isolated according to manufacture protocol (“Supplemental Materials & Methods”). Quantitative PCR using 96 sample plates with cDNA template equivalent to 24 ng of total RNA per 20 μl per well was performed as described in “Supplemental Materials and Methods”.

### Microarray analysis

At least four RNA samples from mouse thyroids of each genotype at 360 days of age were analyzed by Agilent 2100 Bioanalyzer. The three highest quality RNAs from each genotype were selected and gene expression in thyroids from mice of all four genotypes was examined by using Affymetrix GeneChip Mouse Exon 1.0ST Array at the Genomics Shared Resource (GSR) in the Ohio State University Comprehensive Cancer Center. After normalization and background correction, data were analyzed as previously described^39^. Probe sets with two-sided p-value less than 0.001 and ≥ 1.2-fold differences between each genotype groups were considered statistically significant in each comparison and confirmatory studies were performed.

### Protein isolation and immunoblotting

Protein isolation from cells and immunoblot analysis were performed as previously described^39^ and in “Supplemental Materials and Methods”. Primary antibodies for immunoblot are listed in Supplemental Table 2.

### Statistical analysis

Body weight and thyroid volume (normalized to body weight) was assessed by linear modeling and the slopes of body weight or tumor volume change over time were compared among groups. TSH levels were also compared between the groups by linear modeling. Cell proliferation, invasion, migration, and IHC quantification for protein expression were analyzed with ANOVA or when appropriate, paired T tests, or with non-parametric Kruskal–Wallace or Mann–Whitney tests if appropriate. The effect of the presence or absence of each Akt isoform on time-dependent tumor-related endpoints in the mouse experiments were assessed using logistic regression analysis or by Fisher’s exact test if appropriate based on the frequency of events at specific time points. P < 0.05 was considered statistically significant for the primary endpoints after Holm’s adjustment for multiple comparisons.

## Results

### Akt isoform expression in isoform-specific Akt knock out (KO) mouse thyroid

**Figure 1:**
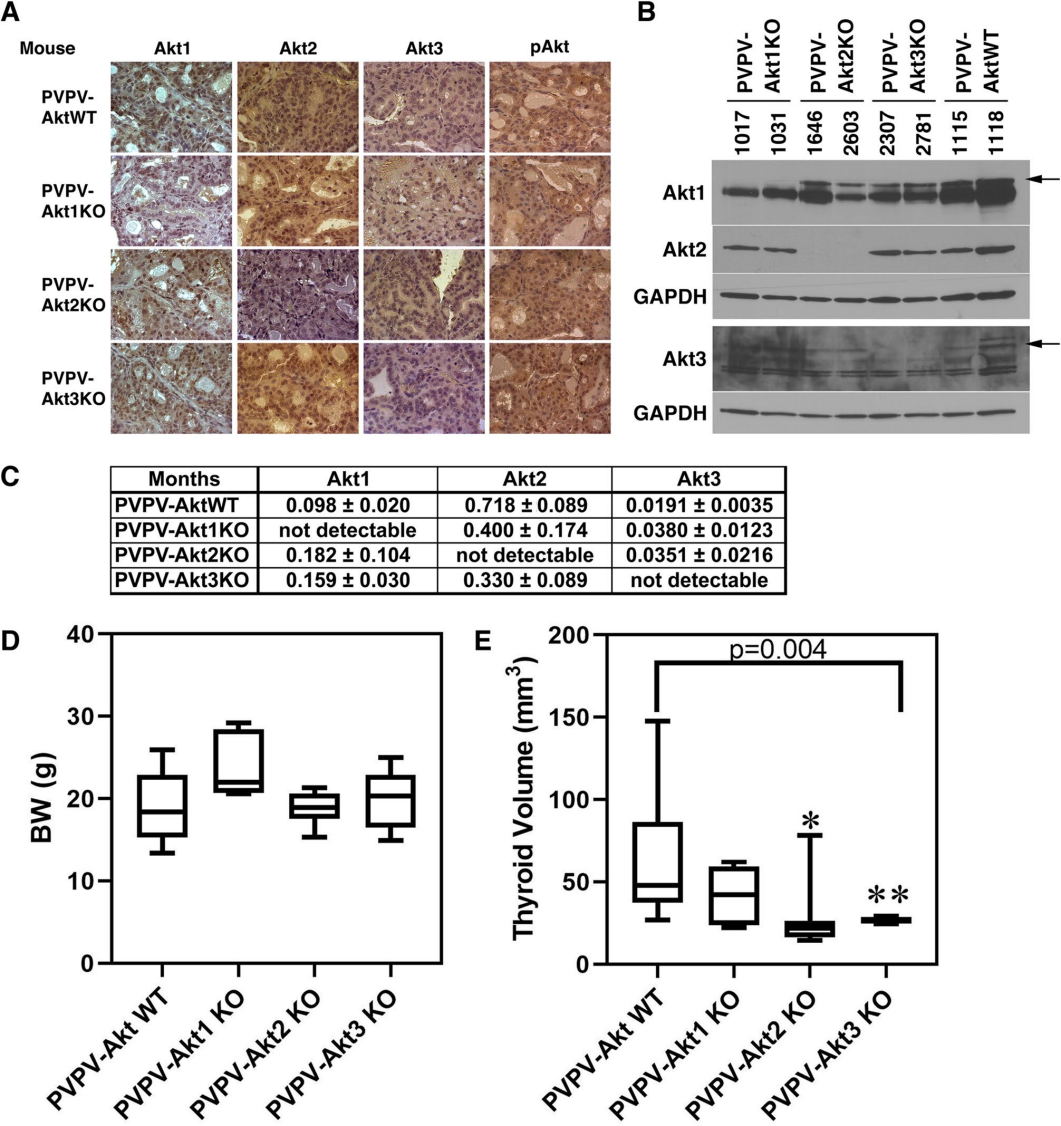
Akt-isoform expression in the thyroid, body weight (BW), and thyroid size in PVPV-AktWT and PVPV-Akt isoform-specific KO mice. Akt isoform protein expression levels were examined by IHC (**A**), immunoblot (**B**), PVPV-AktWT mouse thyroid tissue expressed all Akt isoforms. Akt isoform specific KO mouse thyroids had loss of the expected protein. Separate blots were performed as delineated on the image by the white spaces. Akt 1, 2 and the upper GAPDH are from the same blot; Akt 3 and lower GAPDH are from the same blot. Antibody information is in Supplemental Table 2 and full WB images are Supplemental Fig. 4. (**C**) RNA levels of each Akt isoform from mouse thyroids at 12 months of age were examined by quantitative RT-PCR (n = 4–8). Results are as units calculated by ∆ Ct method normalized to 18S rRNA expressed as mean normalized values. Body Weight (**D**) and thyroid size (E) at ~ 12 months were compared among the 4 groups (n = 12 month group in Fig. 2E). BW was not different between the groups. Thyroid sizes were significantly smaller in PVPV-Akt isoform-specific mice vs. PVPV-AktWT (p = 0.004). For individual groups, *p < 0.002, **p < 0.004; PVPV-Akt1 mice had a p = 0.09. Graph images were created using Graphpad Prism version 8.4.2 (https://graphad.com).

To confirm isoform-specific Akt KO, we examined Akt isoform gene and protein expression by RT-PCR (Fig. 1C), IHC (Fig. 1A), and Western blot (Fig. 1B) using Akt-isoform specific primers and antibodies. All results confirmed isoform-specific depletion. Full Length Western blots are in Supplemental Fig. 5. There was no consistent evidence of compensatory overexpression of other Akt isoforms in the Akt-specific KO mouse thyroids. Levels of pAKT were similar in IHC likely related to continued activation of the remaining isoforms in each of the KO mice as this antibody is not isoform-specific.

### Deletion of each Akt isoform reduces thyroid enlargement in the TRßPVPV mice

We previously showed that PVPV-Akt1WT mice developed goiters beginning at three months of age and deletion of Akt1 reduces thyroid enlargement^34^. We measured thyroid volume every three months in the four mouse lines and performed trend analysis of thyroid volume adjusted by body. At 12 months of age, depletion of each Akt-isoform in the PVPV mice did not change body weight (Fig. 1D) but resulted in reduced thyroid volume (Fig. 1E; p = 0.004). Taken individually Akt2 and 3 KO significantly reduced thyroid size vs PVPV-WT at 12 months (p = 0.002 and 0.004, respectively, while Akt1 KO mice had a trend (p = 0.09). PV/PV Akt 1, 2 and 3 mouse thyroid gland size was not statistically different from each other at 12 months (p = 0.115). Individual Akt KO alone without the PV background did not change thyroid size at 12 months (Supplemental Fig. 1A) and thyroid size over the course of the year are shown (Supplemental Fig. 1B). It is notable that the PVPV-Akt1 KO mice were not smaller by body weight as one would predict. This likely is related to selection of surviving mice as many of these mice were small and did not survive weaning.

Since thyroid cell proliferation and thyroid volume are dependent on TSH and PVPV mice have markedly elevated serum TSH levels^34,40^, we measured serum TSH levels. There was no statistically significant difference in serum TSH among the mouse lines and all had markedly elevated TSH levels. There was a non-significant trend toward a higher TSH in the PVPV-Akt3 KO mice (Supplemental Table 3).

### Deletion of Akt isoforms differentially delays tumor formation, vascular invasion, and distant metastases

We previously reported that at 6 months, approximately 75% of PVPV-Akt1WT mice develop thyroid cancer while only adenomas were identified in the PVPV-Akt1KO mice^34,40^. In that report, thyroid cancer development, local invasion of the primary tumor, and the frequency of lung metastases all were delayed in the PVPV-Akt1KO mice^34^. However, in that report, only Akt1KO was assessed.

**Figure 2:**
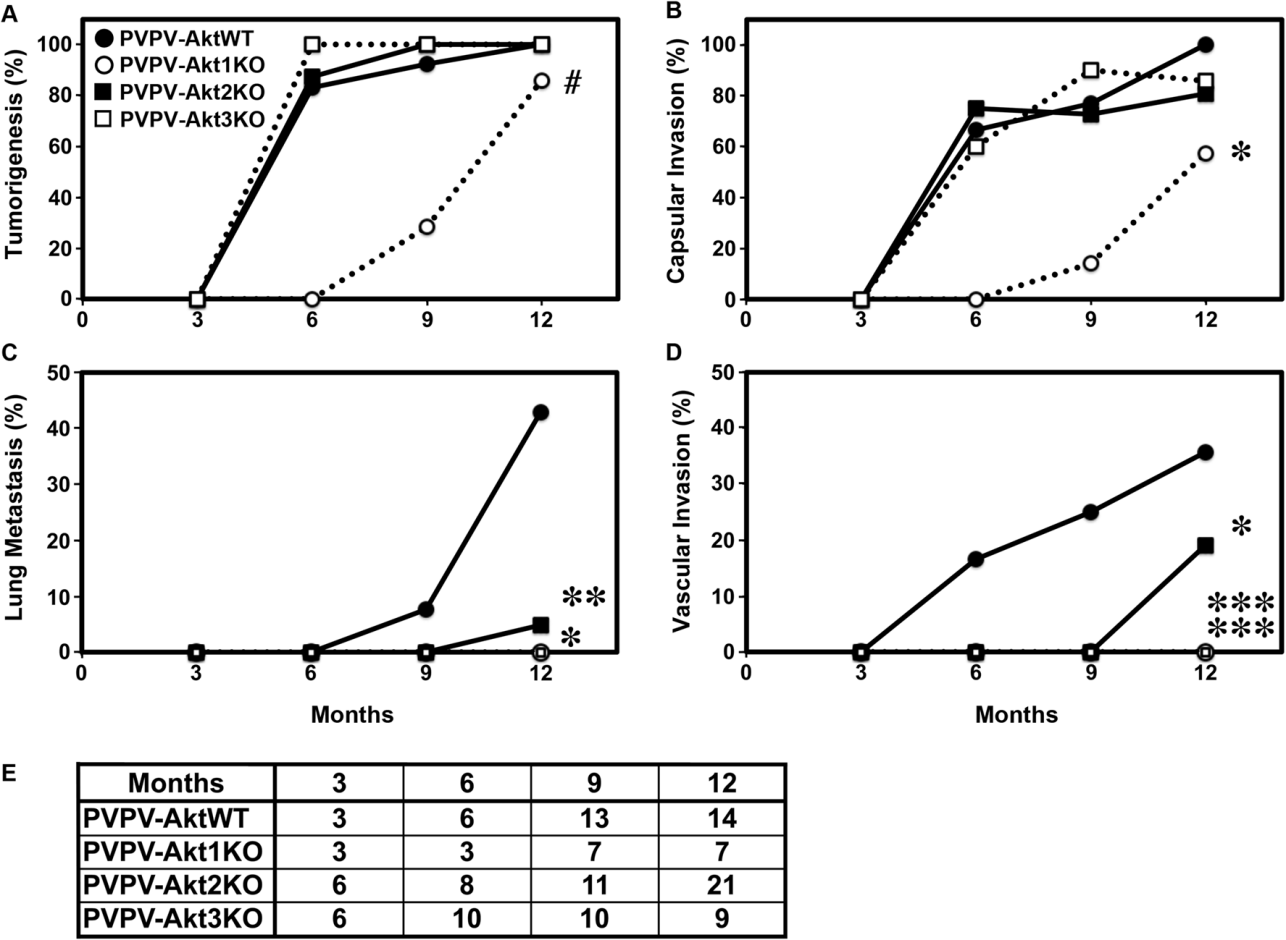
Akt isoform-specific effects on thyroid cancer development and progression. PVPV-AktWT (●), PVPV-Akt1KO (○), PVPV-Akt2KO (**■**), and PVPV-Akt3KO (**□**). A minimum of three mice were sacrificed at 3 months and seven mice at 6, 9, and 12 months for each genotype. Tumor development (**A**; ^#^p = 0.053) and capsular invasion (**B**; *p = 0.012) trended toward or were significantly delayed/reduced only in the PVP-Akt1KO mice, respectively. Vascular invasion (**C**; *p < 0.05; **p = 0.061) and lung metastasis (**D**; *p = 0.018; ***p = 0.004) were decreased with loss of Akt isoforms vs control. (**E**) shows mouse number of each age for each genotype. Graph images were created using Graphpad Prism version 8.4.2 (https://graphad.com).

In the present study, we performed similar analyses of thyroids from PVPV-Akt2KO and PVPV-Akt3KO mice as well as from a new set of contemporaneous PVPV-Akt1KO mice, and compared the results to the PVPV-AktWT mice (Fig. 2). The depletion of Akt2 or Akt3 did not change the tumor incidence or capsular invasion in comparison to PVPV-AktWT mice; however, similar to our prior study using this thyroid cancer model^46^, PVPV-Akt1KO mice had a trend toward a delay in thyroid cancer incidence (p = 0.053, Fig. 2A) and a significant delay in capsular invasion (Fig. 2B, p = 0.012). As above, the PVPV-Akt1KO mice are difficult to breed and often do not survive, numbers are somewhat smaller for that group.

In contrast, Akt 2 and 3 KO significantly decreased the incidence of lung metastasis (Fig. 2C, p 0.009 and 0.048, respectively) with a trend toward significance for Akt1 KO (p = 0.061). At 12 months of age, 46.3% (6/13) of PVPV-AktWT mice developed lung metastasis. However, none of the Akt1- and Akt3-isoform specific KO mice developed lung metastases and only 1 of 21 PVPV-Akt2KO mice had evidence of lung metastasis at this time point.

We also compared vascular invasion between the mouse lines. The incidence of vascular invasion was significantly lower in all of the PVPV-AktKO mouse lines (Fig. 2D). No vascular invasion was identified in PVPV-Akt1 or and Akt3KO mouse thyroids (p = 0.004 vs PVPV-WT), while 19% (4/21) PVPV-Akt2KO mice developed vascular invasion (p = 0.018 vs PVPV-WT).

Because some PVPV-AktKO mice showed invasion at the 12 months of age, we decided to examine mice at the 15 months of age for the presence of distant metastases. At this time point, more than 80% PVPV-AktWT mice died due to local compression and could not be evaluated. Four of 12 (33.3%) 15 month-old PVPV-Akt2KO mice developed lung metastases while only one PVPV-Akt1KO (8.3%, 1/12) and one Akt3KO (7.7%, 1/13) developed lung metastases consistent with trends noted at 12 months. Taken together, the results suggest that tumor development and local invasion are mostly dependent on Akt1 while vascular invasion and distant metastases are dependent on all Akt isoforms but most predominately Akt1 and 3.

### Cell proliferation and apoptosis

**Figure 3:**
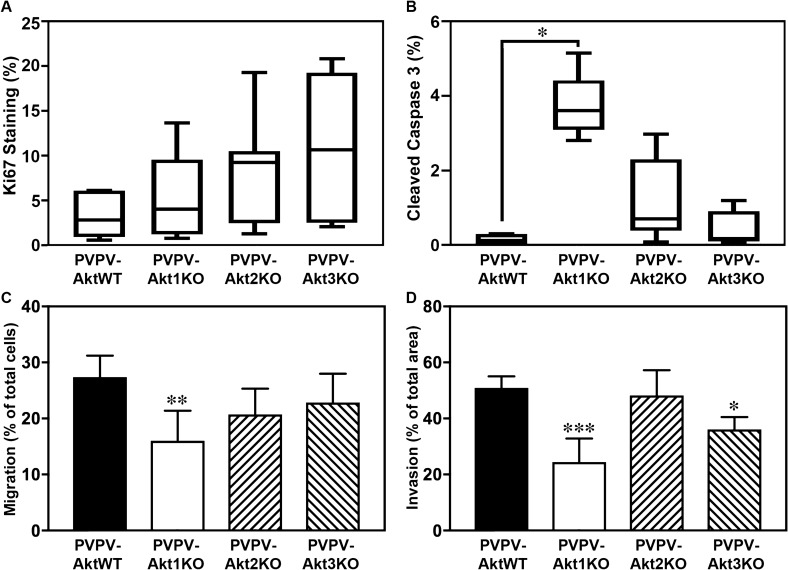
Akt1 loss primarily increase thyroid cell apoptosis and reduces cell motility. Thyroid cell proliferation and apoptosis in vivo were examined by IHC using antibodies against Ki67 (**A**; n = 7 for all genotypes) and cleaved caspase-3 (**B**; n = 5 for all genotypes), respectively. Ki67 was not changed for the any of the KO mice. An increase in apoptosis was identified in the PVPV-Akt1KO thyroid glands vs PVPV-AktWT (*p = 0.008). There was a trend for an increase in PVPV-AKT2KO thyroid glands (p = 0.06). Cell migration (**C**) and invasion (**D**) of primary cultured thyroid cells in vitro were examined using Boyden chambers without or with Matrigel. PVPV-Akt1KO cells had reduced migration and PVPV-Akt1 and Akt3KO cells had reduced invasion. Cells from at least five different thyroids of each genotype mouse were tested. *p < 0.01, **p < 0.005. Graph images were created using Graphpad Prism version 8.4.2 (https://graphad.com). IHC quantitation was performed using InForm software version 2.3.0, (https://www.perkinelmer.com/Content/LST_Software_Downloads/inFormUserManual_2_3_0_rev1.pdf).

Akt isoforms regulate apoptosis and proliferation in vitro and in vivo^41^. Surprisingly, quantitation of Ki67 IHC demonstrated no significant differences (Fig. 3A). To assess apoptosis, we stained for cleaved caspase 3. Figure 3B demonstrates increased cleaved caspase 3 staining increased in thyroids from PVPV-Akt1KO mice compared to the PVPV-AktWT mice while the thyroids from PVPV-Akt2KO and PVPV-Akt3KO had no significant differences although there was a trend for PVPV-Akt2KO (p = 0.06). The quantified percentage is relatively low for apoptosis, thus while the data suggest that decrease of thyroid enlargement in the PVPV-Akt1KO mice is related in part to enhanced apoptosis, it is likely that other factors are involved.

### Cell migration and invasion of primary thyroid cancer cells

As described above, depletion of any Akt isoform decreased local and vascular invasion and lung metastasis, although the effect was greatest for Akt1 and Akt3 loss. We hypothesized that the reduction in local invasion and metastasis may be in part due to cancer cell autonomous effects of Akt. To test this hypothesis, we isolated primary thyroid cells from TRßPVPV mice with either expression of all Akt isoforms or with Akt isoform-specific knock out and compared cell migration and invasion between groups. A significant decrease in cell migration was seen only in the thyroid cells from PVPV-Akt1KO (Fig. 3C). In contrast, cell invasion was decreased in thyroid cells from both PVPV-Akt1KO and PVPV-Akt3KO but not the PVPV-Akt2KO cells, consistent with the in vivo data (Fig. 3D). Experiments are performed in conditions in which growth changes are not identified as described in “Supplemental Materials and Methods”.

### Gene expression in isoform-specific Akt depleted mouse thyroids

**Figure 4:**
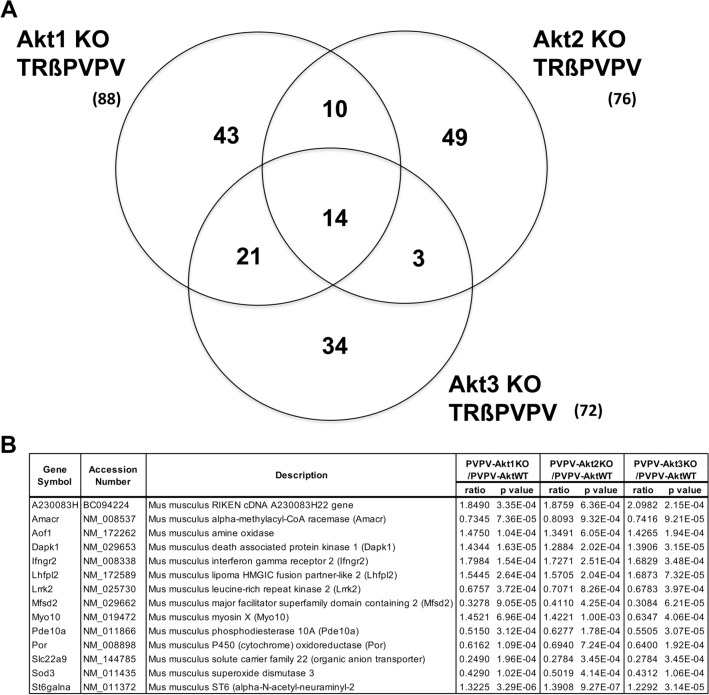
Gene expression in PVPV-AktKO mouse thyroid vs PVPV-AktWT. (**A**) Venn diagram of differentially expressed genes in PVPVAkt isoform KO mouse thyroids vs PVPV-Akt1KO mouse thyroid. (**B**) 14 genes were commonly regulated by KO of each Akt isoform.

To identify isoform-specific Akt pathways responsible for tumor development and progression in an unbiased manner, we examined gene expression in fresh frozen thyroid tissue obtained from PVPV-AktWT and PVPV with isoform-specific Akt KO by Affymetrix mouse microarray. From 11,156 genes analyzed, we broadly selected the statistically significantly altered genes between PVPV-Akt WT and each PVPV-isoform specific Akt KO mouse thyroids for analysis (Supplemental Table 4). Compared to PVPV-AktWT, depletion of Akt1, Akt2, and Akt3 altered expression of 88, 76, and 72 genes, respectively (Fig. 4A). Interestingly, only 14 genes were common for all Akt isoform-specific KO mice (Fig. 4B); 10 genes were common only to PVPV-Akt1KO and PVPV-Akt2KO, 21 were common only to the PVPV-Akt1KO and PVPV-Akt3KO, and 3 were common to the PVPV-Akt2KO and PVPV-Akt3KO mouse thyroids. The majority of the Akt isoform-regulated genes were restricted to individual isoforms (Supplemental Table 4).

**Figure 5:**
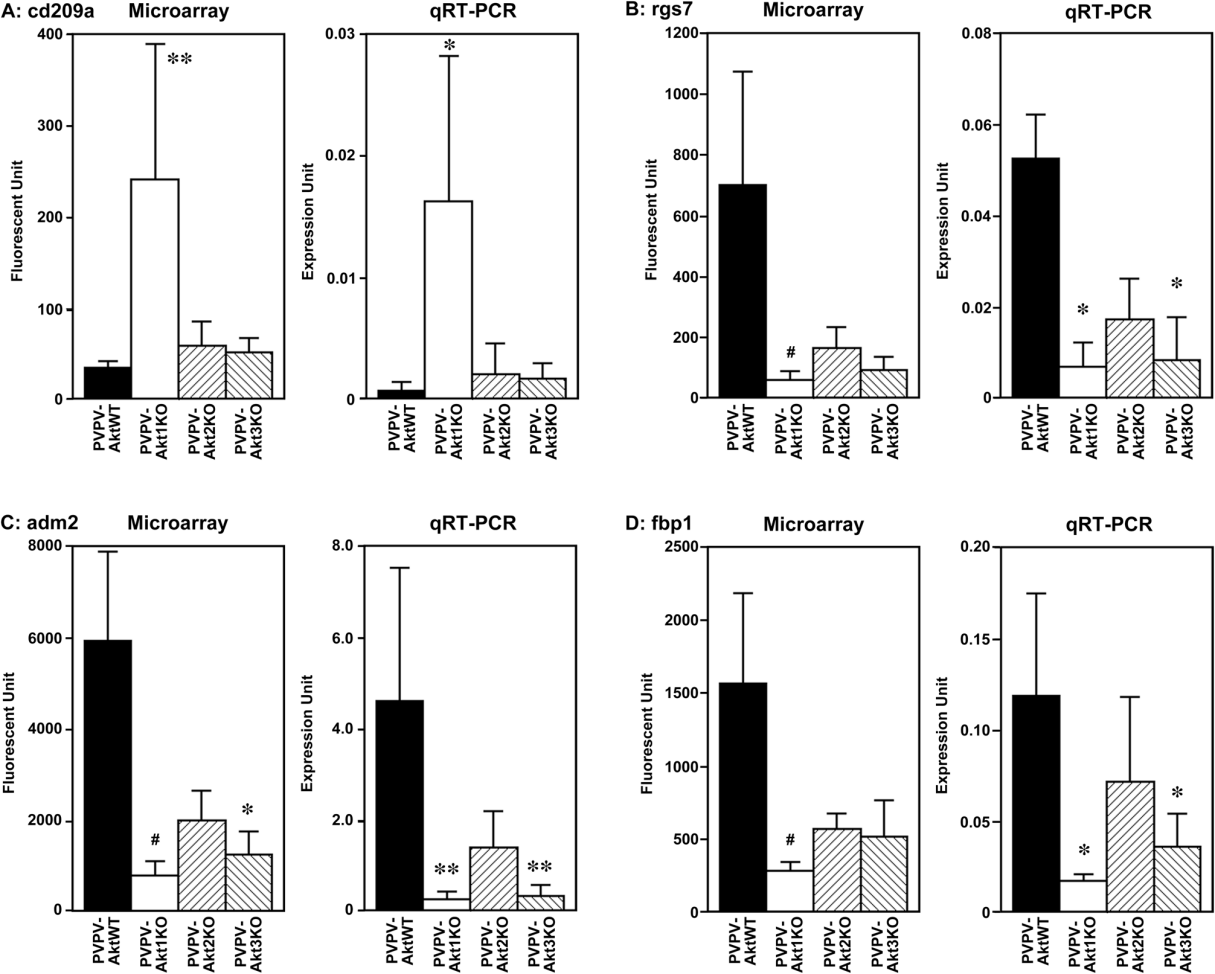
Expression levels of cd209a (**A**), rgs7 (**B**), adm2 (**C**) and fbp1 (**D**). Microarray and confirmatory quantitative RT-PCR expression of these four selected genes respectively. CD209a was specific for Akt1 loss. *p < 0.005, **p < 0.001, ^#^p < 0.0005.

Since Akt1 depletion demonstrated the greatest effects on tumor growth and metastasis in the mouse models, we focused genes altered by depletion of Akt1. Forty-two genes were upregulated and 46 genes were downregulated in the PVPV-KO thyroid versus the PVPV-AktWT mouse thyroid. Among genes significantly altered by Akt1 depletion, some were also changed by Akt2 or Akt3 depletion in the same direction. In order to validate microarray data, we selected 4 genes that demonstrated more than a fivefold difference between the PVPV-Akt WT and PVPV-Akt1 thyroid mRNA and performed quantitative RT-PCR. The most overexpressed gene, the dendritic cell (DC) marker Cd209a was increased only in PVPV-Akt1KO mouse thyroid in the microarray and was confirmed by qRT-PCR (Fig. 5). Regulator of G Protein Signaling 7 (Rgs7), Adrenomedullin 2 (Adm2) and Fructose Biphosphatase 1 (Fbp1) were significantly reduced in PVPV-Akt1KO by microarray and the reduced expression of each of these genes were confirmed by qRT-PCR (Fig. 5). Depletion of Akt2 and 3 also resulted in reduction of these genes in microarray and qRT-PCR analysis suggesting a more general Akt signaling effect; however, the differences were only significant for Akt1 loss. Among these 4 genes, we performed IHC to confirm protein expression patterns for two genes, Adm2 and Cd209a, due to their expression in human tissues and their potential roles in cancer progression. For Adm2, IHC studies confirmed the reduced expression in the PVPV-Akt KO mouse thyroids and an increase in expression with invasion in human thyroid cancer, consistent with potential regulation by Akt signaling (Supplemental Fig. 3). CD209a studies are described in detail below.

### CD209a expression

**Figure 6:**
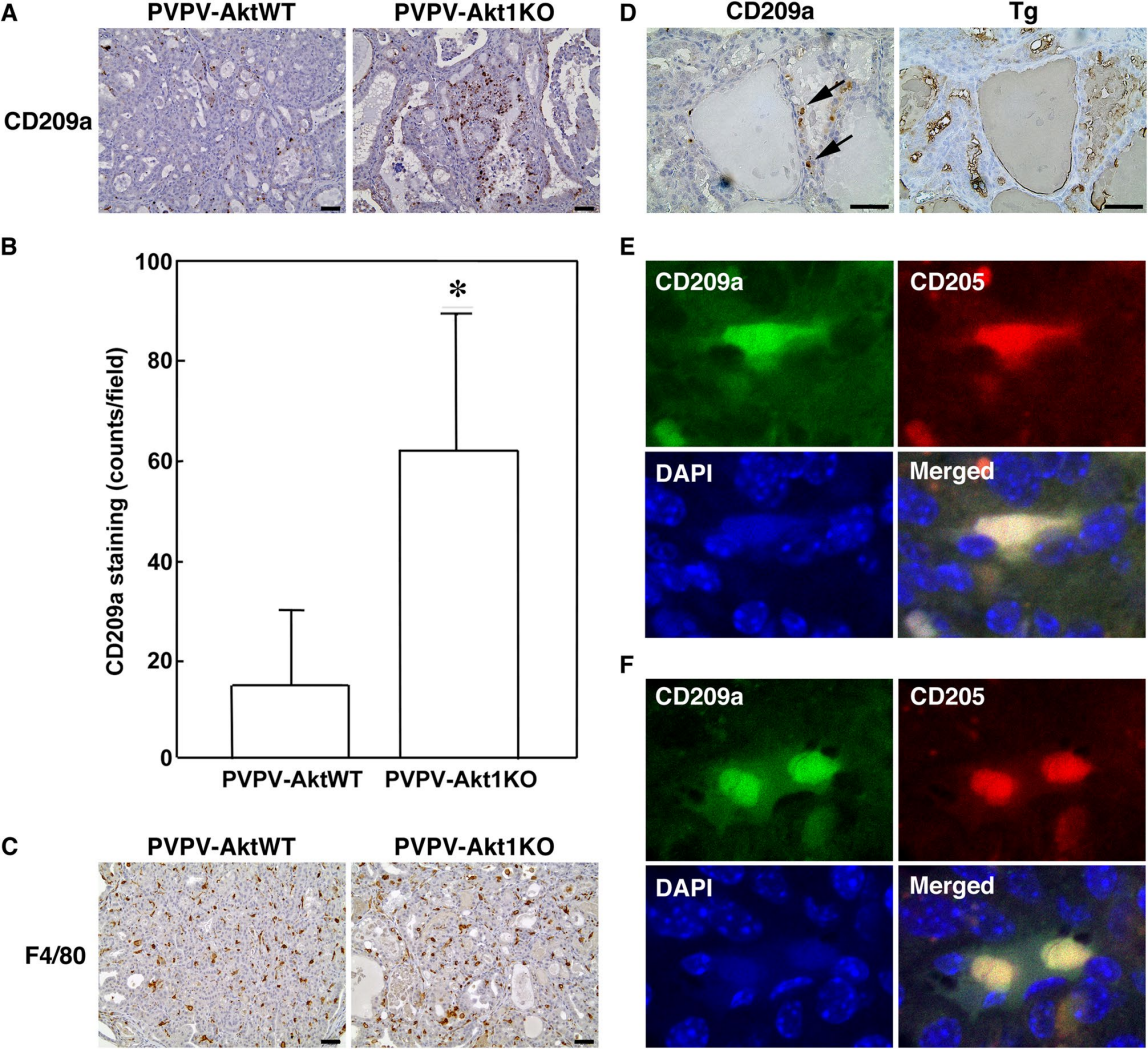
CD209a cells are increased in PVPV-Akt1KO mouse thyroid. (**A**) IHC of CD209a confirmed the presence and increase of expression in PVPV-Akt1KO mouse thyroid by IHC, representative image is shown. (**B**) Quantitation of four pairs of age-matched PVPV-AktWT and PVPV-Akt1KO mice demonstrate an increase of CD209a cells in the Akt1KO mice (62 ± 27 vs 15 ± 15 cells/20 × field; p < 0.05). (**C**) The macrophage marker F4/80, was highly expressed in both the PVPV-AktWT and Akt1KO mouse thyroids and the expression pattern was distinct from CD209a. (**D**) Tg staining was distinct form CD209a staining. (**E**, **F**) Confocal microscope images were taken by 40× objective and digitally enlarged by 5× and show a subset of CD209a cells that co-express CD205 (representative images shown). Primary and secondary antibody negative controls are in Supplemental Fig. 4. Graph images were created using Graphpad Prism version 8.4.2 (https://graphad.com).

CD209a was the most overexpressed gene in the PVPV-Akt1KO model and was unique versus the other Akt KO mice. We examined age-matched thyroids from PVPV-Akt1KO and AktWT mice using IHC to determine if this increase in CD209a mRNA might be due to an increase in a CD209a cell population. We confirmed an increase in CD209a-expressing cells in the PVPV-Akt1 KO mouse thyroids (Fig. 6A,B, p < 0.01) and that they did not express thyroglobulin (Fig. 6D) on serial sections.). Since CD209 in human is expressed mainly in antigen-presenting cells^42–44^, we examined first whether the CD209a positive cells were macrophages or DCs by staining for F4/80 (macrophage marker). In contrast to CD209a, the F4/80 cell population was high in the presence or absence of Akt 1 (Fig. 6C) and there was no discernable overlap with CD209a was on serial sections. These data suggest that CD209a positive cells were primarily a population of DCs. Among DC cells, CD209a is reportedly expressed on conventional tumor suppressive DC. To explore further this possibility, we examined CD205 as a second marker of conventional DCs. A subset of the CD209a positive cells co-expressed CD205 by confocal microscopy, suggesting these to be conventional tumor suppressive DCs (Fig. 6E,F). Taken together, these data suggest that the increase in CD209a is due to an increase in DCs that are suppressed by Akt1 and/or induced by Akt1 loss.

## Discussion

We previously reported that depletion of Akt1 delayed thyroid tumor development and inhibited lung metastasis in the PVPV mouse thyroid cancer model^34^. However, in breast cancer models, loss of Akt2 inhibited, while loss of Akt1 accelerated, cell motility and metastasis in both in vitro and mouse breast cancer models^45–48^. Because Akt is important for oncogenesis and progression of many cancers and pan-Akt inhibitors have been developed and are being studied in clinical trials, we sought to clarify Akt isoform-specificity in regulating thyroid cancer progression^2,4,5,49^. In this present study, we found that depletion of Akt2 or Akt3 did not alter the tumor development or local invasion while we confirmed our prior data with the Akt1 knock out model. In contrast, Akt1 and Akt3 loss delayed the development of vascular invasion and lung metastasis to similar degrees while Akt2 loss had a less impressive effect when considering the 15-month data. This isoform-specific activity was supported by the microarray analysis that showed only modest degrees of overlap between the thyroid Akt isoform-specific KO expression profiles in our model.

Recent reports have suggested that different Akt-isoform specific roles in tumorigenesis and progression may depend not only on isoform but also on tissue origin. For example, Akt2 regulates Snail-mediated induction of EMT in colon cancer cells^50^ and it is anti-apoptotic through phosphorylation of glyceraldehyde-3-phosphate dehydrogenase and decreasing its nuclear translocation in ovarian cancer cells consistent with a tumor promoting effect in these cell types^51^. However, in lung cancer, Akt1 appears to be more responsible for cell motility, tumorigenesis, and tumor progression similar to thyroid cancer^51,52^. In triple-negative breast cancer, activated Akt1, but not Akt2, correlates with reduced disease recurrence-free survival^53^ while knock down of Akt3 significantly inhibited cell growth in spheroid culture and in mouse xenograft^54^. These data suggest that Akt-isoform specific roles in cancer depends on both the tissue of origin and the genetics of the primary tumors within a particular organ-site.

Since Akt stimulates cell proliferation and apoptosis in thyroid cells in vitro^55^, we anticipated that Akt isoform loss both would reduce proliferation and increase apoptosis. Unexpectedly, only changes in apoptosis were detected and this was dependent on Akt1 and not the other isoforms. Based on Akt isoform expression levels, it is unlikely that the absence of an antiproliferative effect was due to compensatory increase of other Akt isoforms, although this has not been excluded entirely. The primary apoptotic response may be consistent with the activation of immune tumor-suppression by DCs induced in the Akt1 KO model. However, it is possible that other mechanisms are involved since the percent of apoptotic cells was modest, particularly since the mouse models utilized generalized Akt isoform KOs.

Depletion of each of the Akt isoforms similarly decreased thyroid size, but tumorigenesis and location invasion were delayed only by Akt1 KO and metastases were decreased by both loss of all isoforms, perhaps to a lesser extent by Akt2 KO. Thyroid size is controlled not only by follicular cell proliferation and apoptosis, but also affected by colloid size. It also may be influenced by non-thyroid factors that could be altered in the generalized Akt KO context. There is no clear explanation of relatively similar degree of decreased thyroid size of the three AktKO mice. Regarding to tumorigenesis and local invasion, in vitro cell motility results were consistent with the in vivo data; only the Akt1 KO thyroid cells showed a significant reduction. By contrast, thyroid cells from both Akt1 KO and Akt3 KO mice showed significantly lower invasion, but not Akt2 KO mice-derived thyroid cells. These results are consistent with the in vivo data in which vascular invasion and distant metastasis were reduced to a lesser extent in Akt2 KO mice compare to the other isoform-specific mice and suggest a component of tumor cell autonomous effects for these endpoints.

It is important to recognize that TSH is markedly elevated in TRβ PV mice^30^ and that TSH cooperates with Akt in inducing cell proliferation^55–57^. Therefore, it is possible that changes in thyroid tumor development are due to reduced TSH levels or signaling. However, depletion of Akt isoforms did not significantly alter serum TSH levels and in all genotypes, the TSH levels were supraphysiological. TSH stimulates TSH receptor, resulting in the cAMP cascade and activation of a number other signaling pathways^34^. Although we did not examine protein levels of TSH receptor and other downstream effectors of this pathway, we did not see any significant changes in the expression of these genes between genotypes or of typical downstream genes in the microarray studies. Thus, while TSHR activation is important in this model, it is not likely to be the primary cause for the findings in this study.

To understand better the Akt-isoform specific effects, we analyzed gene expression in thyroids by microarray. Surprisingly only 14 genes were commonly changed in all Akt isoform-specific KO thyroid glands versus PVPV-AktWT. Akt1 depletion showed the most significant inhibition of tumor development and local invasion. Among 88 genes altered by depletion of Akt1 from PVPV-AktWT, we selected the most highly altered for confirmation by RT-PCR, of which cd209a was further examined due to the high levels of expression, the Akt1 specificity, and the finding of apoptosis in the Akt1KO.

The CD209 family of genes in human also is known as DC-specific intercellular adhesion molecule-3 (ICAM-3) grabbing non-integrin (DC-SIGN). DC-SIGN is a family of membrane-bound receptors expressed on both macrophages and DCs that also includes liver/lymph node-specific ICAM-3 grabbing non-integrin (L-SIGN, also known as DC-SIGNR or CD209L)^42–44^. Mouse CD209a is not a precise homologue of human DC-SIGN, but is considered a DC-SIGN-related protein (SIGNR) that has eight homologs and is one member of the mouse CD209 family. It is expressed in myeloid (or conventional) DCs^42–44^ as well as in tumor-associated macrophages (TAM) that can facilitate cancer progression, including thyroid cancer^58,59^. CD209 and DC-SIGN are reported to localize to both the plasma membrane and intracellularly depending on cell and tissue context, as identified in our experiments^60–62^. DCs are considered a critical factor in antitumor immunity^58,63^ that either can be immunosuppressive or immune activating in function^64^. When considered with the RNA and apoptosis data, the immunohistochemical and immunofluorescence results suggest that at least a proportion of immune activating CD209a DCs are present in the Akt1 KO thyroids. Considering the fact that Akt1 depletion resulted in increased apoptosis, reduced primary tumor size, and a lower frequency of vascular invasion and lung metastasis, it is possible that these DCs might be normally suppressed by Akt1 in cancer and/or immune cells. Further studies are ongoing to determine this potential mechanism for our findings.

The mechanisms by which these DCs are recruited into and/or proliferate in thyroid cancers in this model has yet to be determined. Cancer cells secret growth factors, chemokines, cytokines, and exosome to inhibit tumor immunity^58,65–67^. Akt signaling has been shown by our group to regulate transcription and release of several of these factors, including exosomes^65^, but the isoform dependency of these effects has not been explored. Alternatively, as noted above, since we utilized generalized KO mouse models, it is possible that other stromal cells that also lack Akt isoforms mediate part of this effect^68^. This may be supported by the impact of Akt3 KO despite the lower levels of Akt3 in the thyroid. One previous report showed that Akt1 is essential role for maturation of DCs suggesting that there may be isoform-specific effects on this cell type directly^69^. Further studies using tissue specific knock out models are ongoing.

In conclusion, we have demonstrated, for the first time, Akt isoform-specific effects on thyroid cancer development and progression in the PVPV mouse model of thyroid cancer. While all three Akt isoforms regulate thyroid growth, Akt1 is the primary promoter of thyroid cancer development and local invasion, while vascular invasion and metastatic progression are dependent on Akt1 and 3, and to a lesser extent Akt2. The reduction in cancer development associated with Akt1 loss occurred with an increase in apoptosis and with the recruitment or expansion of a CD209a-expressing dendritic population. These data suggest an isoform-specific immune suppressive role for Akt1 in its ability to promote thyroid cancer progression.

## Supplementary information

Supplementary Figure Legends.

Supplementary Information.
