# Supplementary material for: Akt isoform-specific effects on thyroid cancer development and progression in a murine thyroid cancer model
Source: Sci Rep. 2020 Oct 27;10:18316. doi: 10.1038/s41598-020-75529-0 (PMC7591514; doi:10.1038/s41598-020-75529-0)
Supplement: Supplementary file 1 — Supplementary Figure Legends. [file 41598_2020_75529_MOESM1_ESM.docx]

Supplemental Figure 1. Thyroid volume changes in PVPV-AktWT and Akt-isoform specific depleted mouse thyroids: Panel A. Thyroid volume at 12 months for AktWT (n=8) and Akt1 (n=5), Akt2 (n=7), and Akt3 (n=7) KO mice without TRβ PV background. No significant differences were identified between groups. Panel B. At 3, 6, 9, and 12 months of age, mice were sacrificed and body weight (BW) and thyroid volume were measured. Data are expressed by thyroid volume/gram BW. After 6 months, each of the Akt isoform-specific knock out mice exhibited reduced thyroid volume vs PVPV-AktWT mice. Linear regression demonstrated that all three AktKO groups had significantly smaller thyroids compared to PVPV-AktWT mice (p<0.0001). Graph images were created using Graphpad Prism version 8.4.2 (https://graphad.com).

Supplemental Figure 2. IHC of cleaved caspase-3 in mouse thyroids: Thyroids from PVPV-AktWT showed very few cleaved caspase-3-positive thyroid cells. In PVPV-Akt isoform-specific KO mouse thyroids, clusters of positive cells are identified, particularly in the PVPV-Akt1KO thyroids. Representative data are shown. Bars in photos indicate 20 µm. IHC quantitation was performed using InForm software version 2.3.0, (https://www.perkinelmer.com/Content/LST_Software_Downloads/inFormUserManual_2_3_0_rev1.pdf

Supplemental Figure 3. Immunohistochemical analysis of adrenomedullin 2: Adrenomedullin 2 was strongly expressed in PVPV-AktWT mouse thyroid cells. The intensity was reduced in all of the Akt isoform-specific KO mouse thyroids consistent with the RNA expression data (A). In human follicular cancer (B), the adjacent normal thyroid gland had minimal immune-reactivity but the cancer center showed higher expression that was maintained in both soft tissue metastasis (left) and the invasive fronts of the cancer (left). Photos were taken at 20x and bars indicate 20 µm.

Supplemental Figure 4. Negative Controls for CD209a and CD205 immunofluorescence. Panels 1A and 1B are individual isotype controls using species-specific IgGs for CD209a and CD205, respectively. Panel 1C is secondary antibodies-only controls incubated together.

Supplemental Figure 5: Full images of Western blot for Figure 1B
