# Supplementary material for: Akt isoform-specific effects on thyroid cancer development and progression in a murine thyroid cancer model
Source: Sci Rep. 2020 Oct 27;10:18316. doi: 10.1038/s41598-020-75529-0 (PMC7591514; doi:10.1038/s41598-020-75529-0)
Supplement: Supplementary file 2 — Supplementary Information. [file 41598_2020_75529_MOESM2_ESM.pdf]

Supplementary data for:

Akt isoform- specific effects on Thyroid Cancer Development and Progression in a Murine Thyroid Cancer Model

Motoyasu Saji[1], Caroline S. Kim[2], Chaojie Wang[1], Xiaoli Zhang[3], Tilak Khanal[1], Kevin Coombes [3,4] , Krista La Perle[5], Sheue-Yann Cheng[6] , Philip N. Tsichlis[7], Matthew D. Ringel[1,7]

[1]Division of Endocrinology, Diabetes, and Metabolism, [3]Center for Biostatistics, [4]Department of Biostatistics and Bionformatics, and [7]Department of Cancer Biology and Genetics, The Ohio State University College of Medicine and Arthur G. James Comprehensive Cancer Center, Columbus OH; [2]Division of Endocrinology, University of Pennsylvania, Philadelphia, PA; [5]College of Veterinary Medicine, The Ohio State University, [6]National Cancer Institute, National Institutes of Health, Bethesda, MD,

Table of contents:

SUPPLEMENTAL INFORMATION

Detailed Materials and Methods

Supplemental Figure 1: Adjusted thyroid volumes over time

Supplemental Figure 2: IHC of cleaved caspase 3 in mouse thyroid

Supplemental Figure 3: IHC of adrenomedullin 2 in mouse and human thyroid tissues

Supplemental Figure 4: IF controls for CD209a and CD205

Supplemental Figure 5: Full Length WB for Figure 1

Supplemental Table 1: PCR primers

Supplemental Table 2: Antibodies

Supplemental Table 3: TSH levels

Supplemental Tables 4: Microarray data from mouse thyroids

Sheet 1: All genes examined by microarray analysis

Sheet 2: Up- and Down-regulated genes in PVPV-Akt1KO compared to PVPV-AktWT

Sheet 3: Up- and Down-regulated genes in PVPV-Akt2KO compared to PVPV-AktWT

Sheet 4: Up- and Down-regulated genes in PVPV-Akt3 KO compared to PVPV-AktWT

Sheet 5: Up- and Down-regulated unique genes in PVPV-Akt1KO

Sheet 6: Up- and Down-regulated unique genes in PVPV-Akt2 KO

Sheet 7: Up- and Down-regulated unique genes in PVPV-Akt3KO

Sheet 8: Up-and Down-regulated genes jointly in PVPV-Akt1KO and Akt3KO

Detailed Materials and Methods

Mouse Breeding

Original TRßPV was a mixed strain of C57BL/6J x 129SV. Akt1KO was C57BL/6J. Both Akt2KO and Akt3KO mice were C57BL/6J x CD-1. Current studies have been approved by Institutional Animal Care and Use Committee. All mouse strains were maintained concomitantly in an Association for Assessment and Accreditation of Laboratory Animal (AAALAC)-approved facility and normal rodent chow was used.

Primary Cell Culture:

After isolating the thyroid glands, one lobe of each thyroid was further isolated by eliminating surrounding connective tissues and chopped to 3~5 mm pieces onto a10 cm plate. Pieces were collected in PBS in 50 mL tubes that were centrifuged at 500 g for 3 min at 4 C. After washing with ice-cold PBS, the pieces were placed in a 10x volume of 0.05% Trypsin (Thermo Fisher Scientific Inc., Waltham, MA) overnight at 4 C. Trypsin was blocked by adding equal volume of 6H medium[1] in 10% FBS, 50 µg/mL gentamycin, and 1 µg/mL fungizone. After washing twice with 6H medium, the pieces were transferred onto a sterile cell strainer (100 µm, Thermo Fisher Scientific, Inc.) and passed through the mesh using a 3 mL plastic syringe. The collected medium was centrifuged at 500 x g for 5 min, supernatant was aspirated and pellet was reconstituted with 6H medium and seeded onto a 6-well plate. After 2-4 days, the attached cells were used for experiments.

Cell Motility:

For cell migration, the cell suspension (final concentration of 3×10^4/well) was placed on the top of transwell filter in a Boyden chamber (8 µm pore size, Millipore Sigma, Burlington, MA) in 300 µl 6H medium containing 0.2% FCS. The lower chamber was filled with 400 µl of 6H medium containing 5% FCS. After 24 hours the cells were fixed with 3.7% formaldehyde containing 0.05% crystal violet for 15 min and counted as described[2].

Cell invasion was examined as described[6]. Briefly, cells were seeded similar to cell migration assay, but using Corning BioCoat growth factor-free matrigel coated transwell filters (Thermo Fisher, Inc.). After staining with 0.05% crystal violet and washing chambers with distilled water, cells on the upper chamber were scraped by using a cotton swab. Five areas were randomly selected and images of invaded cells under membrane were taken using an Axiovert 40CFL microscope (Zeiss United States, Richmond, VA). Stained areas representing invasive cells were calculated by using ImageJ software (NIH, Bethesda, MD).

Immunohistochemistry:

The tissue sections were dewaxed by soaking in Xylene twice for 15 min, and hydrated serially in different concentration of alcohol and dH_2O. After microwave treatment in antigen unmasking solution (Vector Laboratory, Inc., Burlingame, CA) for 10 min, endogenous peroxidase activity was inactivated by incubating in 3% hydrogen peroxide for 15 min and washed with PBS 3 times for 5 min. The sections were incubated with 1st antibody in PBS at 4°C overnight (Supplemental Table 2). After washing with PBS, immunostaining was performed using the Vectastain Universal Quick kit and Peroxidase Staining Kit (DAB) according to the manufacturer's instructions (Vector Laboratories, Inc.), and counterstained with Hematoxylin. Antiserum was omitted in the negative control[4, 6].

Immunofluorescence:

Sections were treated and incubated with 1st antibody in PBS at 4°C overnight (Supplemental Table 2). After washing with PBS, fluorescence-conjugated secondary antibodies (Supplemental Table 2) were incubated for 1 hour. After washing slides with PBS twice, they were mounted by using Vectashield containing DAPI (Thermo Fisher Scientific, Inc.). Immunofluorescence photos were taken at the Campus Microscope Imaging Facility using FV3000 confocal laser scanning microscope (Olympus America Co., Center alley, PA).

RNA isolation and RT-PCR analysis:

We isolated RNA and performed RT-PCR similar to previously described[4, 6].

To isolate RNA, after addition of 200 µl of chloroform at room temperature, tubes were shaken, incubated for 2 min at room temperature and centrifuged at 16,000 g for 15 min at 4°C. The supernatant was transferred into a 1.5 ml tube, mixed with 500 µl of isopropanol and 20 µg glycogen, and centrifuged at 16,000 g for 15 min at 4°C. The supernatant was aspirated and the pellets containing RNA were washed using 70% ethanol and air-dried. The RNA was then reconstituted in DEPC-treated water and further purified by using RNA Clean-up and Concentration Micro-Elute Kit (Norgen BIOTEK Co., Thorold, ON, Canada). The concentration was measured by NanoDrop ND-1000 (NanoDrop Technolgies, LLC, Wilmington, DE).

For RT-PCR, 400 - 800 ng of RNA were treated with DNase I (Thermo Fisher) for 15 min and 400 ng of DNase-treated RNA were reversed transcribed using the TaqMan® Reverse Transcription Reagents Kit (Thermo Fisher). Quantitative PCR was performed in 96 sample plates using cDNA equivalent to 24 ng of total RNA per 20 µl per well. As an internal control to

normalize gene expression, 18S ribosomal RNA was amplified using Taqman Ribosomal RNA control reagents kit as described[3]. Specific primers for various genes were listed in Supplemental Table 1, and PCR was performed with SYBR Green Mix (Thermo Fisher Scientific, Inc.) for AKT1 and TaqMan® Universal PCR Master Mix (Thermo Fisher Scientific, Inc.) for other genes using the following parameters: after an initial 2 min incubation at 50°C for inactivation of AmpEASE UNG activity, the cDNA was denatured at 94°C for 10 min and samples were subjected to 40 cycles of a two-step amplification protocol that included 15 seconds at 94°C and a one-minute annealing-elongation step at 60°C. Target gene and 18S were amplified in all samples in duplicate in two separate reactions. Negative controls were included for the RT (RT negative) and PCR (Non-Template Control) reactions. To compare expression levels, difference of threshold (ΔCt) were calculated by subtracting threshold (Ct-18S) of 18S from threshold (Ct-gene) of the target gene, and expression levels were estimated by 2^(-ΔCt).

Protein Isolation and Immunoblotting:

Cells were washed with ice-cold PBS twice, scraped, collected into microcentrifugation tubes, and centrifuged at 300 x g for 5 min at 4 °C. Cell pellets were washed again with ice-cold PBS and lysed with ice-cold M-PER buffer (Thermo Fisher Scientific, Inc.) containing 1 µg/ml leupeptin, 1 µg/ml pepstatin, 1µg/ml aprotinin, 20 µM 4-amidino-phenylmethane-sulfonyl fluoride (APMSF), and 0.3 µM okadaic acid for 10 min on ice. They were centrifuged at 12,000 x g for 10 minutes at 4°C. The supernatants were transferred into fresh tubes and stored at –80 °C. The protein concentration of cell lysates was determined using a micro BCA protein assay reagent kit (Thermo Fisher Scientific, Inc.) [4, 6].

When protein from tissues was isolated, tissue was placed in 5-10 volume of the same buffer as described above and homogenized by hand homogenizer (PowerGen 125, Thermo Fisher Scientific, Inc.).

Twenty μg of total protein lysate were suspended in reduced SDS sample buffer (Thermo Fisher Scientific, Inc.) and boiled for 5 minutes. Protein lysates were subjected to 8% or 4-10% SDS-PAGE, and the separated proteins were transferred to nitrocellulose membranes (0.45 μm pore size, Thermo Fisher Scientific, Inc.) by electrophoretic blotting (Thermo Fisher Scientific, Inc.). Nonspecific binding was prevented by blocking the membrane with TBS-T (0.1% Tween 20 in 20 mM Tris-HCl, pH 7.6 and 137 mM NaCl) containing 5% nonfat dry milk for 2 hr at RT. Immunoblotting was performed as previously described[4-6]. Background measures were obtained from each lane and subtracted from the measured band. Immunoblots of the same protein samples was performed for GAPDH to verify equal loading and for normalization for quantitation between lanes.

References for Supplemental Materials and Methods

1 Ambesi-Impiombato, F.S., Parks, L.A. & Coon HG. Culture of hormone-dependent functional epithelial cells from rat thyroids. *Proc Natl Acad Sci U S A* **77**, 3455-3459 (1980).

2 McCarty, S.K., *et al*. Group I p21-activated kinases regulate thyroid cancer cell migration and are overexpressed and activated in thyroid cancer invasion. *Endocr Relat Cancer* **17**, 989-999 (2010).

3 Ringel, M.D., *et al*. Overexpression and Overactivation of Akt in Thyroid Carcinoma. *Cancer Res* **61**, 6105-6111 (2001).

4 Saji, M., *et al*. Akt1 deficiency delays tumor progression, vascular invasion, and distant metastasis in a murine model of thyroid cancer. *Oncogene* **30**, 4307-4315 (2011).

5 Vasko, V., *et al*. Akt activation and localization correlate with tumor invasion and oncogene expression in thyroid cancer. *J Mol Genet* **41**, 161-170 (2004).

6 Wang, C., *et al*. RCAN1-4 is a thyroid cancer growth and metastasis suppressor. *JCI Insight* **2,** e90651 (2017).

Supplementary Fig 1

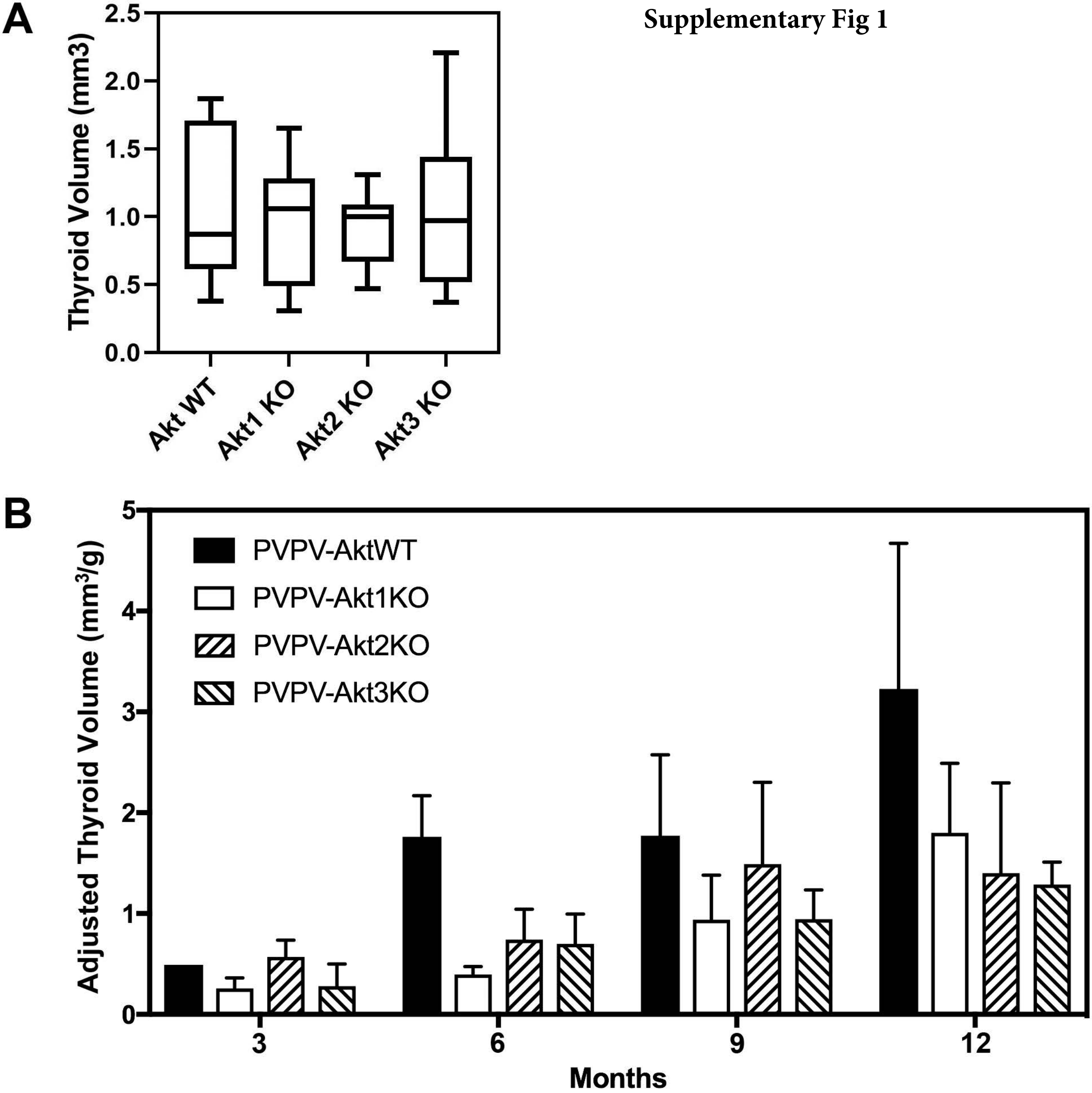

Supplementary Fig 2

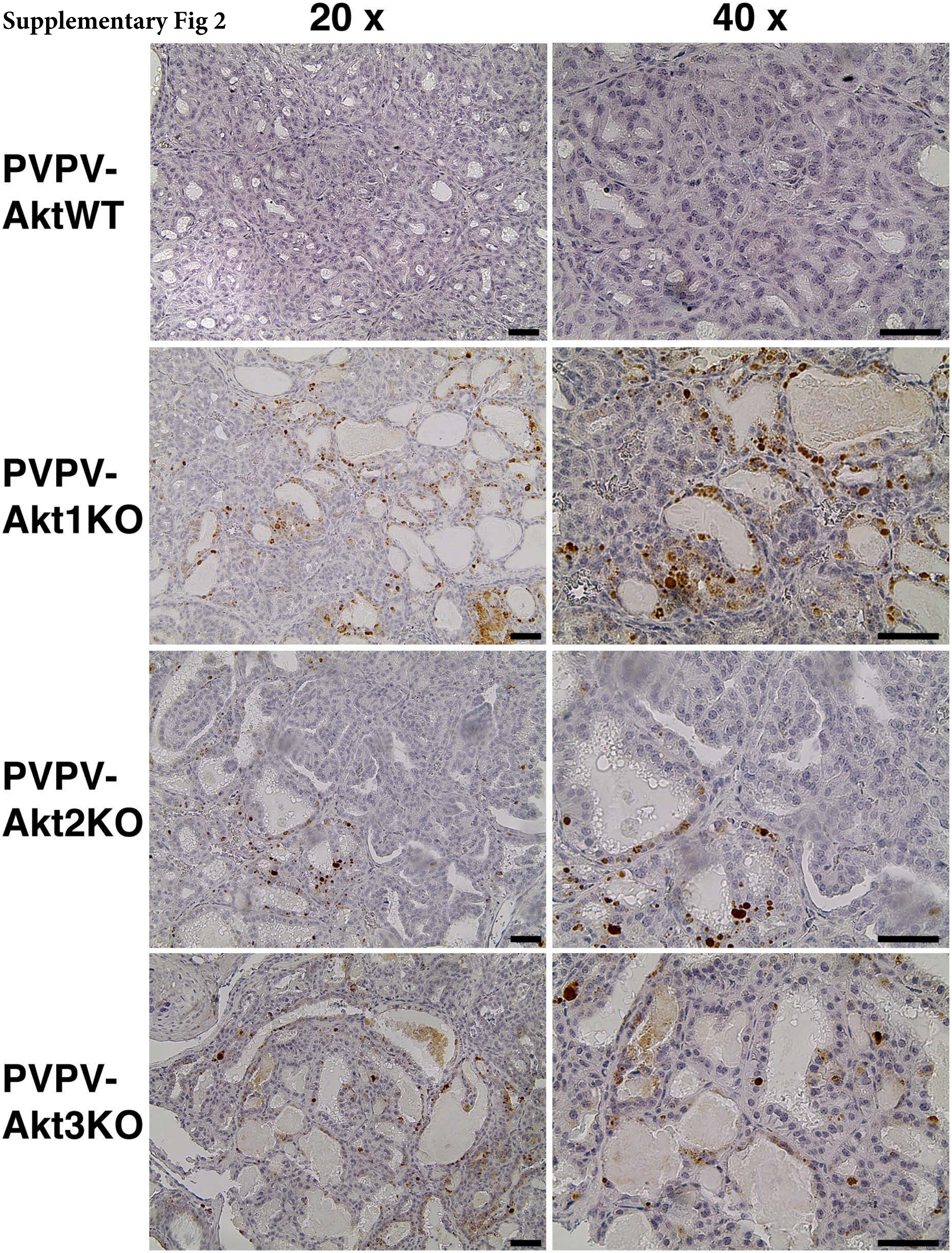

Supplementary Fig 3

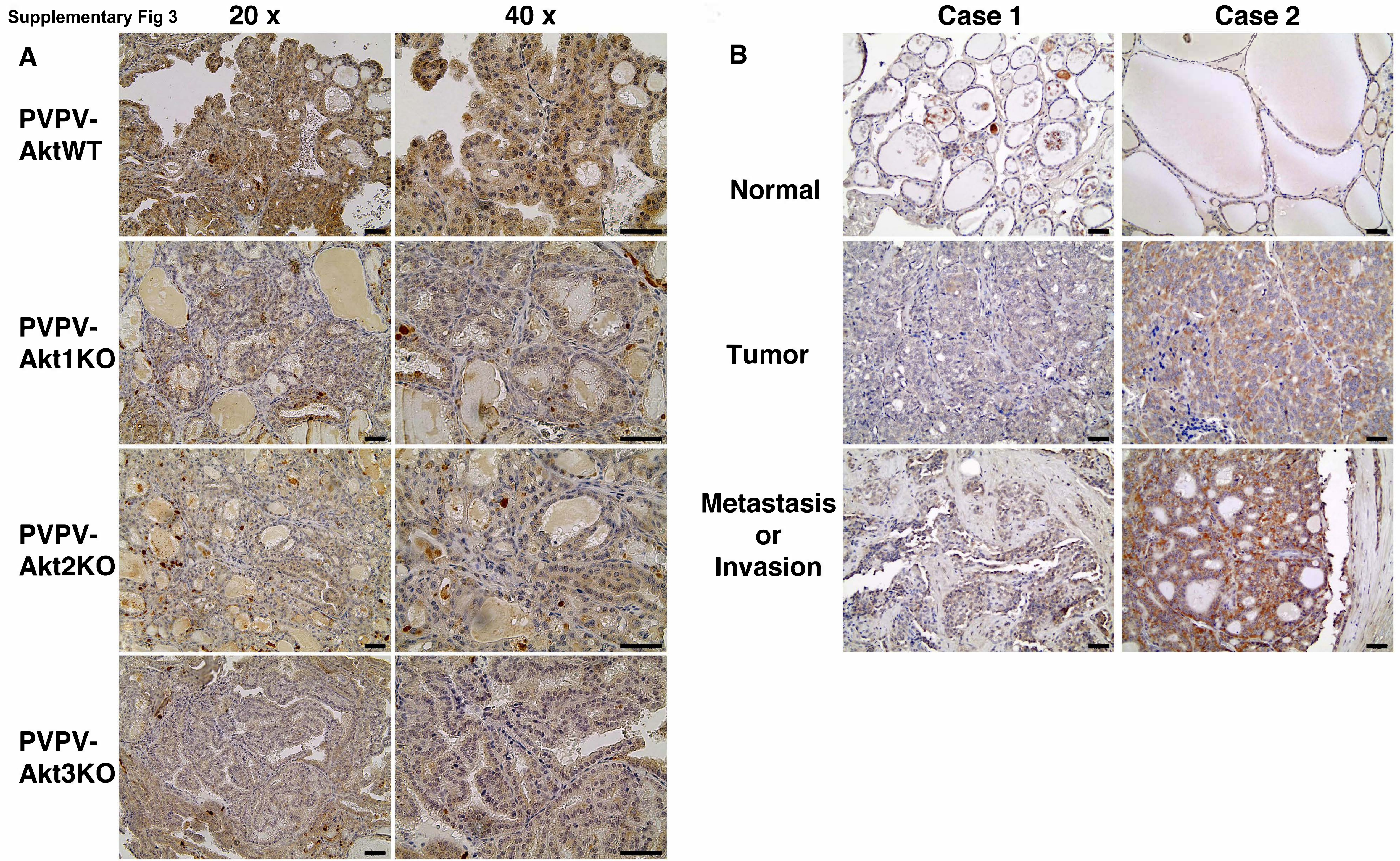

Supplementary Fig 4

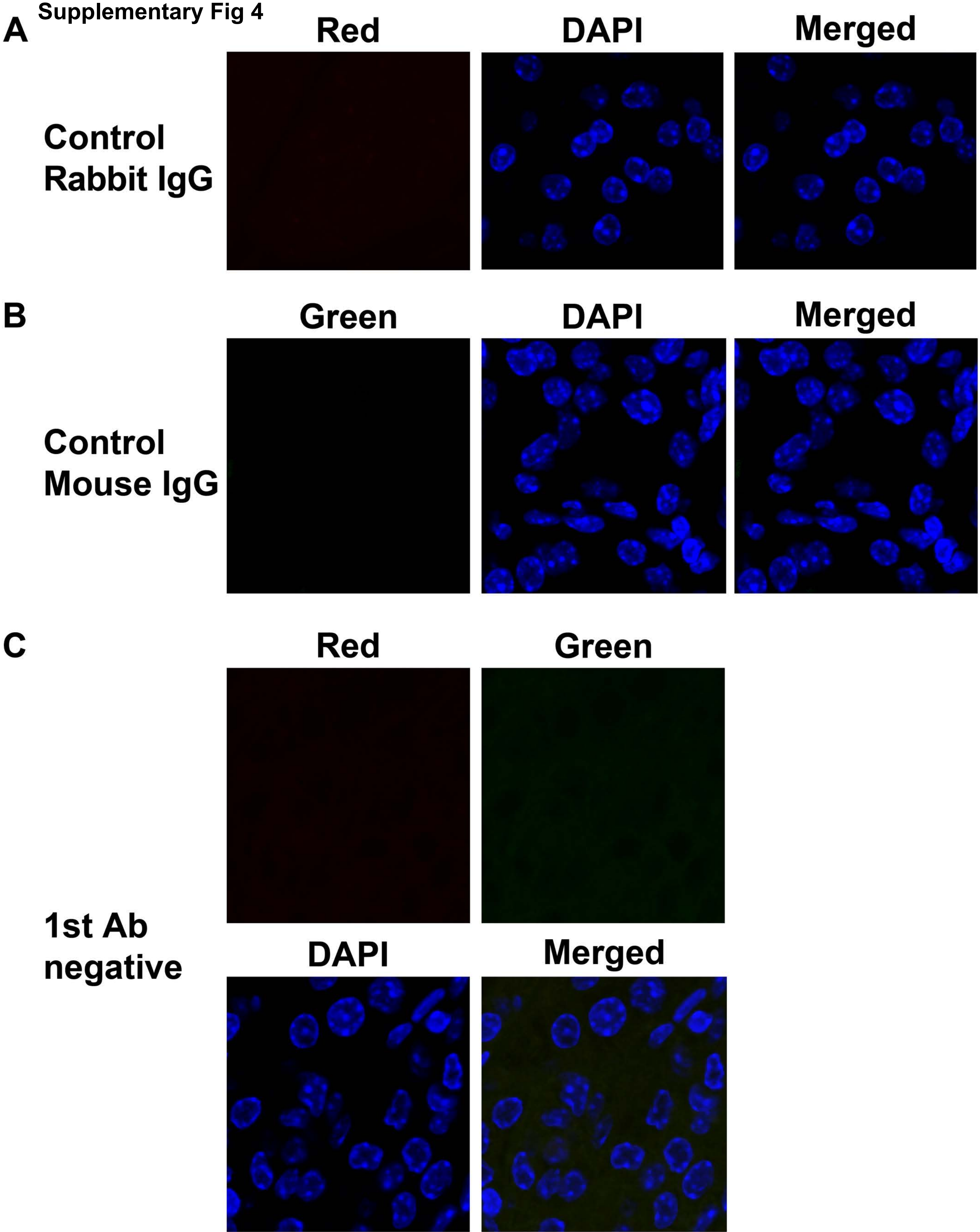

Supplementary Fig 5

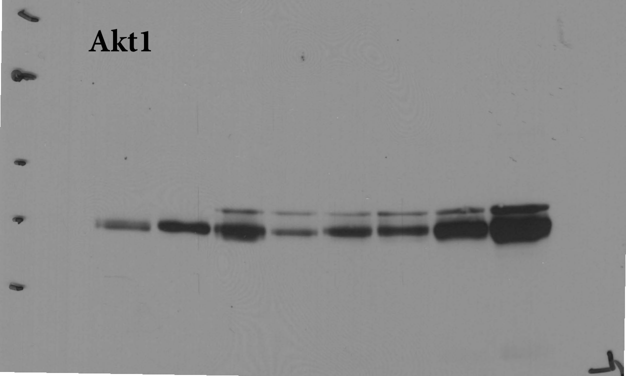

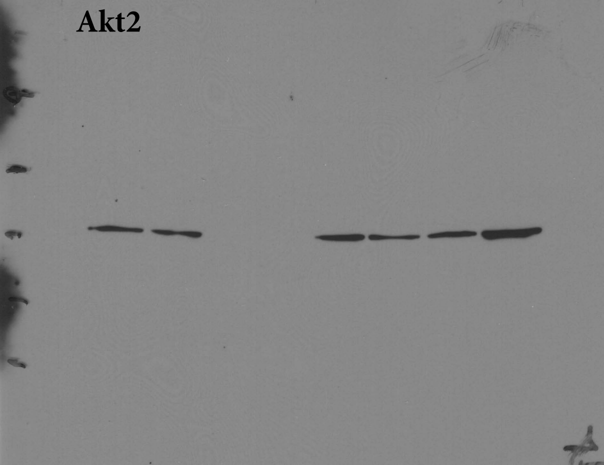
Akt2

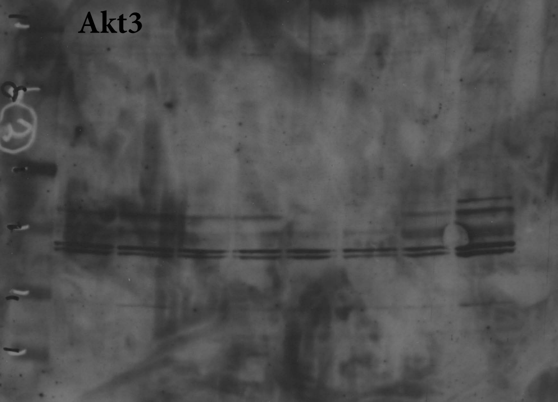
Akt3

GAPDH Akt 1 and 2

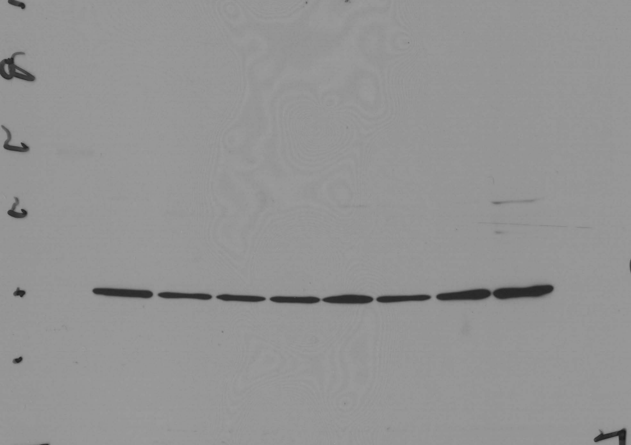

GAPDH Akt3

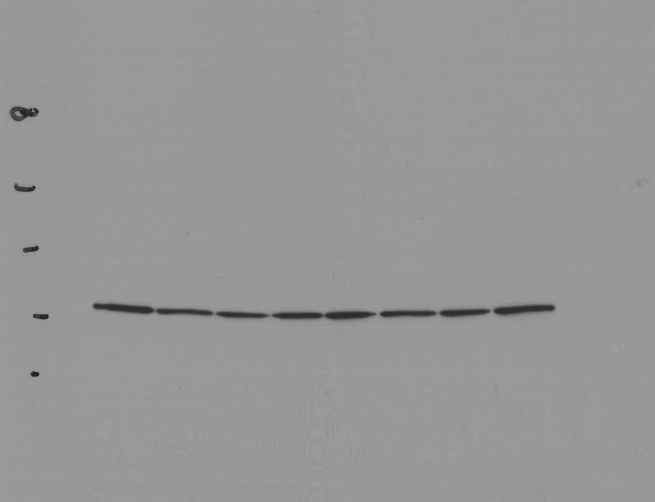

Supplementary Table 1

Genotype Screening

target		Forward primer	Reverse primer	
Akt1	wild-type	CAG CTC TTC TTC CAC CTG TCT C	CAG ACT GTA TCT CAC CTG CGA AAG	144 bp
	KO	GTG GAT GTG GAA TGT GTG CGA G		259 bp
Akt2	wild-type	TAC ACT TCA TTC TCA GTA TTG TTT TGC	TGG ACA ATC TGT CTT CAT GCC AC	110 bp
	KO	ACC AAC CCC CTT TCA GCA CTT G		277 bp
Akt3	wild-type	GGG AGA GAG AAG TGC CAT TGT ATT G	GCA TCC ATC CTT GTT CAC GC	650 bp
	KO		GTG GGG TGG GAT TAG ATA AAT GC	700 bp
TRßPV	wild-type	TCC CCA AGC CAG CAT CCC GAC C	CGG GCA AAT CCT TAC CTG G	452 bp
	KO	TGC AGT GGC GAT GGC ATC	TGC AGT GGC GAT GGC ATC	546 bp

Detection

Akt1	Forward	AGC TCT TCT TCC ACC TGT CTC G
	Reverse	CGG CGT TCC GCA GAA TGT
Akt2	Taqman	Mm00545827_m1
Akt3	Taqman	Mm00442194_m1
Thyroglobulin	Taqman	Mm00447525_m1
TTF-1	Taqman	Mm00657018_m1
Adrenomedulin2	Taqman	Mm00812850_g1
Cd209a	Taqman	Mm00460067_m1
Fbp1	Taqman	Mm00490181_m1
Rgs7	Taqman	Mm01317058_m1
18S	Taqman	cat# 4308329

Supplementary Table 2

	Antibody	company	catalogue number	species	WB	IHC or IF
Primary	Akt (total)	Cell Signaling Technologies	9272	rabbit	2,000 x	
	Akt1	BD Biosciences	610860	mouse	1,000 x	100 x
	Akt2	Upstate Biotechnology	06-606	sheep	1,000 x	100 x
	Akt3	Santa Cruz Biotechnology	sc-11520	goat	1,000 x	100 x
	phospho Ser473-Akt	Santa Cruz Biotechnology	sc-7985-R	rabbit		100 x
	GAPDH	Cell Signaling Technologies	2118	rabbit	2,000 x	
	Ki67	Abcam	ab16667	rabbit		200 x
	Cleaved Caspase-3	Cell Signaling Technologies	9579	rabbit		800 x
	Adrenomedulin 2	Santa Cruz Biotechnology	sc-86272	rabbit		100 x
	CD209a	Invitrogen	MA1-25615	mouse		100 x
	CD205	Origene	TA323022	rabbit		50 x
	CD317	Origene	TA337141	rabbit		100 x
	F4/80	Thermo Fisher, Incorporatio	MF48000	rat		400 x
Secondary for WB	Anti rabbit IgG linked with HRP	Cell Signaling Technologies	7074	mouse	2,000 x	
	Anti mouse IgG linked with HRP	Cell Signaling Technologies	7076	horse	2,000 x	
	Anti goat IgG linked with HRP	Santa Cruz Biotechnology	sc-2768	rabbit	2,000 x	
	Anti sheep IgG linked with HRP	Upstate Biotechnology	12-342	rabbit	2,000 x	
Secondary for IHC	Biotinylated anti rabbit IgG (H+L)	Thermo Fisher, Incorporatio	BA-1000	goat		500 x
	Biotinylated anti mouse IgG (H+L)	Thermo Fisher, Incorporatio	BA-2000	horse		500 x
	Biotinylated anti goat IgG (H+L)	Thermo Fisher, Incorporatio	BA-5000	rabbit		500 x
	Biotinylated anti rat IgG (H+L)	Thermo Fisher, Incorporatio	BA-9401	goat		500 x
Secondary for IF	Alexa Fluor 488 antigoat IgG (H+L	Thermo Fisher, Incorporatio	A110555	donky		2,000 x
	Alexa Fluor 594 antigoat IgG (H+L	Thermo Fisher, Incorporatio	A11037	goat		2,000 x

Supplementary Table 3

genotype	TSH level (compare to the level in PVPV-AktWT)	p-value vs PVPV-AktWT
PVPV-AktWT	1	
PVPV-Akt1KO	1.41 ± 0.19	0.16
PVPV-Akt2KO	1.28 ± 0.20	0.17
PVPV-Akt3KO	1.60 ± 0.07	0.06

Supplemental Table 3. TSH levels in PVPV-AktWT and isoform-specific knock out mice with PVPV gene: TSH levels in mouse serum was measured by using mouse TSH kit and normalized to the average levels in PVPV-AktWT mice. Data were expressed average ± standard deviation. Statistics for difference in each isoform-specific Akt was initially analyzed by one-way ANOVA and significance between groups were examined by non-parametric, Mann Whitney, test.

Table 4: All Probes

Probe_Set	order	Akt1	Akt2	Akt3	AktW	Akt1_WT	p_Akt1WT	Akt2_WT	p_Akt2WT	Akt3_WT	p_Akt3WT	Akt2_3	p_Akt2_3	Akt1_2	p_Akt1_2	Akt1_3	p_Akt1_3	Probe_Set	X1017	X1031	X1109	X1646	X2037	X2603	X2564	X2307	X2780	X1115	X1118	X1060	Gene_Symbol	mRNA_Accession
6747308	1	8.494233	8.542226	8.450806	8.534077	-0.03984	0.660156	0.008149	0.927911	-0.08327	0.367977	0.09142	0.325506	-0.04799	0.597413	0.043427	0.632178	6747308	8.509191	8.378276	8.595231	8.411058	8.605799	8.609821	8.545611	8.444447	8.362359	8.41614	8.548263	8.637829	Lypla1	NM_008866
6747309	2	7.832282	7.946847	7.839986	7.628717	0.203565	0.180354	0.318129	0.050962	0.211269	0.166185	0.106861	0.463152	-0.11456	0.432723	-0.0077	0.957064	6747309	7.986978	7.748579	7.761289	7.944989	8.001403	7.894148	8.011369	7.728848	7.779741	7.414151	7.542768	7.929233	Tcea1	NM_011541
6747314	3	8.277211	8.283995	8.150393	8.440146	-0.16294	0.43091	-0.15615	0.449608	-0.28975	0.178443	0.133602	0.515627	-0.00678	0.973298	0.126819	0.536623	6747314	8.422976	8.162178	8.24648	8.375996	8.149172	8.326817	8.299503	8.257132	7.894543	8.094354	8.367355	8.85873	Atp6v1h	NM_133826
6747343	5	8.233388	8.484023	8.369852	8.331735	-0.09835	0.309842	0.152288	0.131676	0.038117	0.685374	0.114171	0.24362	-0.25063	0.024553	-0.13646	0.170867	6747343	8.085777	8.352393	8.261995	8.373574	8.508552	8.569943	8.46137	8.319905	8.328281	8.219454	8.459518	8.316233	Rb1cc1	NM_009826
6747364	7	9.89663	9.999408	10.03154	9.997929	-0.1013	0.423767	0.001479	0.990482	0.033614	0.786804	-0.03213	0.795932	-0.10278	0.417312	-0.13491	0.294167	6747364	9.85487	9.957383	9.877636	9.886991	9.989443	10.12179	10.24846	9.983746	9.862423	9.868871	9.926656	10.19826	Pcmtd1	NM_183028
6747471	8	7.933488	7.877622	7.900825	8.088712	-0.15522	0.468118	-0.21109	0.330591	-0.18789	0.383524	-0.0232	0.912156	0.055866	0.790931	0.032663	0.87664	6747471	7.848469	7.959668	7.992327	7.709755	7.813577	8.109533	7.826788	7.90558	7.970108	8.456914	7.598595	8.210627	Rrs1	NM_021511
6747472	9	6.443882	6.675383	6.471971	7.065965	-0.62208	0.086218	-0.39058	0.254362	-0.59399	0.09879	0.203412	0.540367	-0.2315	0.487476	-0.02809	0.931801	6747472	6.134744	6.562011	6.634891	7.05863	6.398019	6.569499	6.424033	6.548404	6.443476	6.795094	6.603899	7.798903	Adhfe1	NM_175236
6747497	11	7.0958	6.979269	6.998483	6.465665	0.630135	0.026633	0.513605	0.058141	0.532818	0.051106	-0.01921	0.936161	0.116531	0.62968	0.097317	0.686504	6747497	7.214514	7.012691	7.060195	6.890254	7.19825	6.849304	7.312871	6.846076	6.836501	6.04826	6.942533	6.406201	Sgk3	NM_133220
6747515	13	8.338779	8.335788	8.129769	8.26039	0.07839	0.426563	0.075399	0.443729	-0.13062	0.200321	0.206019	0.058869	0.002991	0.975287	0.20901	0.056006	6747515	8.337455	8.233233	8.44565	8.25795	8.470444	8.278971	7.976404	8.20178	8.211124	8.245715	8.169055	8.366399	Cspp1	NM_026493
6747641	15	7.683186	7.745018	7.185881	6.891996	0.79119	0.099808	0.853022	0.079744	0.293885	0.509004	0.559137	0.224939	-0.06183	0.887974	0.497305	0.275821	6747641	7.619622	8.212584	7.217352	7.642867	8.354205	7.237982	7.678856	6.672298	7.206488	6.75831	7.456565	6.461113	Sulf1	NM_172294
6747786	17	7.90358	7.967006	7.7787	7.722602	0.180978	0.240299	0.244403	0.125065	0.056098	0.704452	0.188305	0.223411	-0.06343	0.668429	0.12488	0.406936	6747786	7.871547	7.924078	7.915115	7.698376	8.12375	8.078891	7.954762	7.574466	7.806873	7.636256	7.609635	7.921916	Terf1	NM_009352
6747805	18	9.798073	9.568034	9.630789	9.357312	0.440761	0.135843	0.210721	0.450759	0.273477	0.33362	-0.06276	0.819282	0.230039	0.411971	0.167284	0.546648	6747805	9.611237	9.796026	9.986956	9.262488	9.882084	9.559529	10.10901	9.141095	9.642262	9.175962	9.264777	9.631198	Rdh10	NM_133832
6747837	19	8.033618	8.02638	8.13901	8.183873	-0.15025	0.232668	-0.15749	0.212901	-0.04486	0.709881	-0.11263	0.361427	0.007238	0.951925	-0.10539	0.391543	6747837	7.782072	8.208173	8.11061	7.95638	8.126415	7.996345	8.148758	8.237759	8.030513	8.054646	8.261362	8.235611	Tmem70	NM_027415
6747839	20	9.106694	8.649774	9.113654	8.957615	0.149078	0.709181	-0.30784	0.447813	0.156039	0.696388	-0.46388	0.263457	0.45692	0.270132	-0.00696	0.986043	6747839	9.186676	9.309758	8.823647	8.872324	9.124195	7.952803	8.588858	9.317153	9.434951	9.150733	9.319798	8.402315	Ly96	NM_016923
6747972	26	5.995576	6.042569	6.193128	6.276299	-0.28072	0.134139	-0.23373	0.202663	-0.08317	0.634725	-0.15056	0.397477	-0.04699	0.78732	-0.19755	0.274579	6747972	6.096198	6.112804	5.777726	6.273231	6.001762	5.852714	6.105161	6.404197	6.070027	6.529078	6.236239	6.063579	Il17a	NM_010552
6747986	27	7.055698	7.170444	7.1154	6.678646	0.377052	0.284396	0.491798	0.172895	0.436754	0.220501	0.055044	0.871138	-0.11475	0.735981	-0.0597	0.860363	6747986	7.132284	6.953485	7.081325	6.871112	7.915843	6.724376	7.447882	6.611611	7.286706	6.653729	6.832895	6.549314	Paqr8	NM_028829
6748011	28	6.405718	6.599054	6.632491	6.738052	-0.33233	0.006315	-0.139	0.163395	-0.10556	0.277374	-0.03344	0.72156	-0.19334	0.065313	-0.22677	0.036718	6748011	6.53071	6.397719	6.288726	6.62075	6.558942	6.617469	6.729312	6.642214	6.525948	6.656498	6.644961	6.912696	Tmem14a	NM_029398
6748020	29	9.071113	9.315781	8.887718	9.463272	-0.39216	0.264948	-0.14749	0.664081	-0.57555	0.116577	0.428062	0.227063	-0.24467	0.47595	0.183395	0.590437	6748020	8.925643	8.913532	9.374165	9.178724	9.344855	9.423763	8.346314	9.716379	8.600462	9.442256	9.312118	9.635442	Gsta3	NM_001077353
6748174	30	6.467291	6.471017	6.748676	6.870341	-0.40305	0.055274	-0.39932	0.05709	-0.12166	0.517682	-0.27766	0.161086	-0.00373	0.983975	-0.28138	0.156201	6748174	6.242949	6.701873	6.457052	6.244442	6.330693	6.837916	6.796279	6.611793	6.837956	6.789991	7.048608	6.772423	B3gat2	NM_172124
6748191	31	6.131641	6.309196	6.230106	6.261318	-0.12968	0.245216	0.047878	0.655688	-0.03121	0.770466	0.07909	0.466314	-0.17755	0.124292	-0.09846	0.368859	6748191	6.153665	6.089301	6.151957	6.213751	6.328437	6.385399	6.404628	6.15245	6.133239	6.188154	6.129926	6.465874	Col9a1	NM_007740
6748213	32	8.950806	9.016889	8.980076	9.095856	-0.14505	0.216265	-0.07897	0.485695	-0.11578	0.315147	0.036813	0.74208	-0.06608	0.557744	-0.02927	0.79331	6748213	9.00209	8.94261	8.907717	9.015176	8.91826	9.117232	9.019315	9.030095	8.890819	8.927529	9.005075	9.354964	Lmbrd1	NM_026719
6748217	33	8.571482	8.609041	8.42371	8.794035	-0.22255	0.306833	-0.18499	0.390762	-0.37032	0.106866	0.185331	0.389942	-0.03756	0.858437	0.147772	0.489264	6748217	8.63557	8.637981	8.440896	8.905919	8.446733	8.474471	8.444964	8.484702	8.341465	8.426457	8.725724	9.229925	Lmbrd1	NM_026719
6748503	37	6.591163	6.804842	6.397065	6.470898	0.120264	0.483183	0.333944	0.075486	-0.07383	0.663697	0.407777	0.037348	-0.21368	0.227738	0.194097	0.269413	6748503	6.56381	6.645514	6.564164	6.451055	7.064598	6.898873	6.534777	6.259222	6.397197	6.283056	6.454692	6.674947	Rab23	NM_008999
6748525	38	7.907372	7.834716	7.735089	7.691994	0.215378	0.262595	0.142722	0.447592	0.043094	0.815521	0.099628	0.592471	0.072656	0.695008	0.172284	0.363284	6748525	7.859222	8.030282	7.832613	8.080112	7.924696	7.499341	7.850011	7.568478	7.786777	7.418998	7.938165	7.71882	Dst	NM_134448
6748534	39	8.407101	8.506406	8.704003	8.566296	-0.1592	0.259905	-0.05989	0.660408	0.137707	0.324899	-0.1976	0.17074	-0.09931	0.471118	-0.2969	0.053609	6748534	8.22206	8.427104	8.57214	8.424599	8.649354	8.445266	8.900406	8.584931	8.626672	8.407932	8.739718	8.551239	Imp4	NM_178601
6748535	40	7.100848	7.384689	7.21936	7.477051	-0.3762	0.014291	-0.09236	0.466082	-0.25769	0.065269	0.165329	0.207941	-0.28384	0.046549	-0.11851	0.354884	6748535	6.994281	7.14338	7.164883	7.458255	7.363111	7.332702	7.297307	7.392089	6.968684	7.433924	7.344652	7.652576	Ptpn18	NM_011206
6748551	44	6.697048	6.691779	6.664418	6.681439	0.015609	0.961589	0.01034	0.974548	-0.01702	0.958116	0.027361	0.932732	0.005269	0.987029	0.03263	0.919827	6748551	6.220959	6.813975	7.056209	6.81884	6.801584	6.454913	6.671004	6.36276	6.959489	6.776812	7.152776	6.114729	C230030N	AK082264
6748553	45	6.619374	6.621404	6.802684	6.704527	-0.08515	0.63632	-0.08312	0.644271	0.098157	0.586605	-0.18128	0.32605	-0.00203	0.99094	-0.18331	0.320992	6748553	6.282598	6.754631	6.820892	6.648177	6.780331	6.435703	6.751539	6.747343	6.909169	6.756626	6.909154	6.4478	Arhgef4	NM_183019
6748557	46	8.396017	8.535398	8.612456	8.547875	-0.15186	0.252635	-0.01248	0.921809	0.064581	0.614289	-0.07706	0.549014	-0.13938	0.290605	-0.21644	0.116958	6748557	8.215275	8.333442	8.639334	8.504985	8.585609	8.515599	8.648173	8.595849	8.593346	8.320401	8.623427	8.699797	Plekhb2	NM_145516
6748605	47	8.81765	8.959019	8.871886	8.770777	0.046873	0.81108	0.188242	0.350153	0.101109	0.608557	0.087133	0.658261	-0.14137	0.477501	-0.05424	0.782236	6748605	8.740138	8.829156	8.883656	9.009698	9.341915	8.525443	8.922292	8.983631	8.709735	8.668724	8.704939	8.938668	Hs6st1	NM_015818
6748620	48	8.590435	8.276982	8.330837	8.17409	0.416344	0.03176	0.102891	0.538939	0.156747	0.356827	-0.05386	0.745554	0.313453	0.086285	0.259597	0.144035	6748620	8.696833	8.549911	8.52456	8.20026	8.473815	8.15687	8.576996	8.006824	8.408692	8.343163	8.183145	7.995963	Arid5a	NM_145996
6748645	49	7.888133	7.813399	7.830534	7.735297	0.152836	0.097277	0.078102	0.36543	0.095237	0.275697	-0.01714	0.838544	0.074734	0.385424	0.057599	0.4993	6748645	7.87567	7.851272	7.937458	7.929451	7.883328	7.627419	7.832866	7.867629	7.791108	7.631047	7.82857	7.746275	Cnnm4	NM_033570
6748646	50	7.309968	7.307288	7.21539	7.377601	-0.06763	0.555932	-0.07031	0.540753	-0.16221	0.178736	0.091898	0.427942	0.00268	0.981169	0.094578	0.415148	6748646	7.112971	7.306593	7.51034	7.326593	7.368989	7.226282	7.096897	7.301846	7.247427	7.274217	7.336924	7.521663	Cnnm3	ENSMUST000000
6748699	55	7.54518	7.44378	7.30894	7.173892	0.371289	0.046133	0.269888	0.124932	0.135048	0.416108	0.13484	0.416798	0.101401	0.537681	0.23624	0.171989	6748699	7.563572	7.479598	7.592371	7.393396	7.672878	7.265065	7.382837	7.078811	7.465172	7.121455	7.441818	6.958402	Inpp4a	NM_030266
6748701	56	8.001554	8.148919	7.985194	8.023941	-0.02239	0.722012	0.124978	0.073629	-0.03875	0.541351	0.163725	0.027261	-0.14736	0.041447	0.01636	0.794476	6748701	8.033443	8.068322	7.902898	8.047117	8.235568	8.164072	8.040213	7.982108	7.933262	7.989299	8.082658	7.999865	Unc50	NM_026123
6748726	57	5.948491	6.004467	6.308476	5.984284	-0.03579	0.787024	0.020183	0.878713	0.324192	0.035218	-0.30401	0.045026	-0.05598	0.673699	-0.35998	0.02284	6748726	5.835757	5.920892	6.088824	5.829697	6.163844	6.01986	6.514363	6.124221	6.286843	6.028164	5.844258	6.080429	Lipt1	NM_001037918
6748729	58	9.295936	9.26557	9.303403	9.171343	0.124593	0.278497	0.094227	0.404903	0.13206	0.252833	-0.03783	0.73319	0.030366	0.7841	-0.00747	0.946164	6748729	9.185081	9.253901	9.448826	9.237568	9.437641	9.1215	9.263417	9.258016	9.388775	9.284691	9.212188	9.01715	Mrpl30	NM_027098
6748736	59	8.829238	8.683327	8.720368	8.60471	0.224528	0.025384	0.078616	0.365184	0.115658	0.195566	-0.03704	0.663061	0.145912	0.112659	0.10887	0.220379	6748736	8.923777	8.72592	8.838018	8.669207	8.743034	8.637739	8.714719	8.754693	8.691693	8.474868	8.787166	8.552097	Eif5b	NM_198303
6748832	61	8.185966	8.199383	8.170499	8.000034	0.185931	0.141856	0.199348	0.118758	0.170465	0.173543	0.028883	0.806549	-0.01342	0.909294	0.015466	0.895528	6748832	8.126516	8.165233	8.266148	8.054925	8.298369	8.244854	8.409678	8.0813	8.02052	7.884165	8.109507	8.006431	Pdcl3	NM_026850
6748837	62	9.038393	8.548588	9.3288	8.536135	0.502258	0.075064	0.012453	0.960801	0.792664	0.012098	-0.78021	0.013055	0.489805	0.081205	-0.29041	0.27094	6748837	9.470399	8.86381	8.78097	8.500113	8.833636	8.312016	9.328553	9.456101	9.201745	8.947129	8.417332	8.243945	Npas2	NM_008719
6748841	63	7.684371	7.819386	7.971492	7.484143	0.200227	0.264607	0.335243	0.079458	0.487349	0.019296	-0.15211	0.388767	-0.13502	0.441954	-0.28712	0.123703	6748841	7.750715	7.528226	7.774171	7.466088	8.087705	7.904366	8.097099	7.897672	7.919705	7.273755	7.549856	7.628819	Rpl31	NM_053257
6748855	64	8.044313	8.038327	8.052999	7.92037	0.123944	0.235849	0.117957	0.257284	0.13263	0.207449	-0.01467	0.88316	0.005986	0.952158	-0.00869	0.930638	6748855	8.043599	8.09138	7.997961	8.022002	8.267361	7.825618	8.024944	8.10224	8.031814	7.875948	7.982991	7.90217	D1Bwg021	BC049091
6748877	65	9.124565	9.278947	8.934837	8.715341	0.409224	0.025849	0.563606	0.005546	0.219497	0.181282	0.344109	0.050823	-0.15438	0.333196	0.189727	0.241248	6748877	9.298595	9.113654	8.961445	9.169694	9.542408	9.124738	8.993734	8.776455	9.034323	8.516644	8.884828	8.74455	Map4k4	NM_008696
6748884	67	9.371545	9.28463	8.914043	8.263764	1.107781	0.003417	1.020866	0.00537	0.650279	0.042554	0.370587	0.206992	0.086915	0.75569	0.457502	0.128511	6748884	9.817344	9.162254	9.135036	9.156234	9.482229	9.215427	9.286444	8.543086	8.912599	7.920242	8.611699	8.25935	Il1r1	NM_008362
6748886	68	6.809983	6.714117	6.313487	6.145132	0.664851	0.112437	0.568984	0.165549	0.168355	0.663624	0.400629	0.313977	0.095867	0.803595	0.496496	0.219715	6748886	6.934111	6.825607	6.670232	6.473698	7.103514	6.565138	6.664112	5.528061	6.748289	5.753435	6.689332	5.99263	Il1rl2	NM_133193
6748889	70	9.617054	9.617792	9.247805	7.632094	1.98496	0.006323	1.985699	0.00631	1.615711	0.017437	0.369988	0.513418	-0.00074	0.998944	0.369249	0.514236	6748889	9.545544	9.723413	9.582204	9.346636	10.01628	9.490461	9.697897	8.24006	9.805457	7.906449	8.390737	6.599095	Il18r1	NM_008365
6748980	73	6.62016	6.661663	6.606451	6.516714	0.103446	0.257915	0.144949	0.126264	0.089737	0.321547	0.055212	0.533851	-0.0415	0.63818	0.013708	0.875773	6748980	6.554523	6.670485	6.635471	6.631785	6.835979	6.517225	6.561791	6.717532	6.540031	6.471915	6.591949	6.486278	Pou3f3	NM_008900
6749009	74	7.572921	7.689704	7.549133	7.599936	-0.02701	0.74676	0.089768	0.298919	-0.0508	0.547108	0.140571	0.120145	-0.11678	0.186437	0.023788	0.775978	6749009	7.540849	7.609076	7.568838	7.670008	7.678933	7.72017	7.68678	7.534532	7.426087	7.575277	7.471957	7.752573	Mrps9	NM_023514
6749044	76	7.677914	7.676266	7.609101	7.450904	0.22701	0.091613	0.225361	0.093599	0.158197	0.218449	0.067164	0.586256	0.001649	0.989236	0.068813	0.577279	6749044	7.696731	7.77725	7.559762	7.580084	7.767672	7.681041	7.781832	7.594422	7.45105	7.233924	7.580704	7.538085	AI597479	BC006931
6749069	77	8.840094	8.583367	8.893525	8.755752	0.084342	0.579059	-0.17239	0.271256	0.137773	0.372591	-0.31016	0.066189	0.256727	0.11647	-0.05343	0.723659	6749069	8.741788	8.727778	9.050716	8.53994	8.683209	8.526951	9.088682	8.650203	8.941691	8.895156	8.835928	8.536172	Nck2	NM_010879
6749073	78	6.305733	6.751252	6.030216	6.510549	-0.20482	0.661954	0.240703	0.608225	-0.48033	0.31817	0.721036	0.148721	-0.44552	0.352387	0.275518	0.558409	6749073	6.371502	6.418344	6.127354	6.088737	7.885746	6.279272	6.51379	5.634047	5.94281	6.673664	6.48945	6.368533	1500015O	NM_024283
6749081	79	8.253793	8.303498	8.378481	8.206486	0.047307	0.751569	0.097012	0.520541	0.171995	0.267669	-0.07498	0.617564	-0.04971	0.739509	-0.12469	0.412924	6749081	8.322536	8.255075	8.183768	8.093683	8.524533	8.292278	8.544434	8.267275	8.323733	8.068163	8.470433	8.080861	Tpp2	NM_009418
6749103	80	7.219801	7.394634	7.363191	7.258906	-0.0391	0.764815	0.135728	0.313976	0.104285	0.43301	0.031443	0.809711	-0.17483	0.203757	-0.14339	0.289215	6749103	7.145934	7.203707	7.309763	7.231268	7.543394	7.409241	7.35799	7.313766	7.417818	7.027521	7.22813	7.521067	Bivm	NM_144558
6749108	81	7.119878	7.204239	7.206647	7.270265	-0.15039	0.069327	-0.06603	0.38428	-0.06362	0.401045	-0.00241	0.974045	-0.08436	0.273409	-0.08677	0.26098	6749108	7.126829	7.229502	7.003302	7.141879	7.244127	7.226711	7.330345	7.157608	7.131988	7.220328	7.255907	7.334561	Ercc5	NM_011729
6749142	83	9.683779	9.241776	8.338158	7.845301	1.838478	0.009861	1.396476	0.033865	0.492857	0.393386	0.903618	0.136764	0.442003	0.441931	1.345621	0.039149	6749142	10.30607	9.822428	8.922839	9.082049	9.998554	8.644726	9.119335	7.688524	8.206615	7.24931	8.316304	7.970288	Col3a1	NM_009930
6749352	86	7.134109	7.498392	7.197349	7.535233	-0.40112	0.039602	-0.03684	0.82723	-0.33788	0.072421	0.301043	0.102609	-0.36428	0.056308	-0.06324	0.708771	6749352	7.07721	7.262807	7.062311	7.54843	7.47051	7.476235	7.534906	6.824689	7.232452	7.388968	7.660251	7.556481	Sdpr	NM_138741
6749376	87	8.736681	8.591584	8.683242	8.195453	0.541228	0.059818	0.396131	0.14745	0.487789	0.083725	-0.09166	0.720222	0.145097	0.573132	0.053439	0.834137	6749376	8.755209	8.804953	8.649881	8.291298	8.967328	8.516127	8.787804	8.341505	8.920418	7.846157	8.610529	8.129673	Stat1	NM_009283
6749444	89	6.444002	6.415509	6.586904	6.434248	0.009753	0.918751	-0.01874	0.844757	0.152656	0.138029	-0.1714	0.101482	0.028493	0.766287	-0.1429	0.161544	6749444	6.604678	6.353765	6.373562	6.505839	6.267701	6.472986	6.606563	6.463109	6.691041	6.404537	6.411076	6.487132	Tmem194b	NM_001142647
6749455	90	6.298127	6.489939	6.304335	6.500293	-0.20217	0.218454	-0.01035	0.947143	-0.19596	0.231602	0.185603	0.255023	-0.19181	0.240754	-0.00621	0.968293	6749455	6.24116	6.315691	6.337531	6.635752	6.372637	6.461427	6.402263	6.24579	6.264953	6.284287	6.33491	6.881683	Hibch	NM_146108
6749471	92	6.800022	6.853373	6.656478	6.490175	0.309847	0.069115	0.363197	0.03934	0.166302	0.292714	0.196895	0.219113	-0.05335	0.727231	0.143545	0.359465	6749471	6.707988	6.88646	6.805619	6.64757	7.036542	6.876006	6.94105	6.625815	6.402568	6.375193	6.586628	6.508705	Ormdl1	NM_145517

6749473	93	6.411613	6.440617	6.486913	6.618841	-0.20723	0.238814	-0.17822	0.305511	-0.13193	0.441185	-0.0463	0.783356	-0.029	0.86303	-0.0753	0.656032	6749473	6.223257	6.295343	6.716238	6.493752	6.421295	6.406805	6.694788	6.328449	6.437502	6.417747	6.577057	6.861718	Osgepl1	NM_028091
6749557	95	8.561286	8.520758	8.291552	8.606235	-0.04495	0.811563	-0.08548	0.651856	-0.31468	0.122782	0.229206	0.244365	0.040529	0.829737	0.269735	0.177471	6749557	8.729457	8.515006	8.439396	8.350733	8.784735	8.426805	8.349049	8.56764	7.957966	8.521386	8.798605	8.498715	Coq10b	NM_001039710
6749572	96	9.624705	9.579304	9.549451	9.639993	-0.01529	0.866561	-0.06069	0.510437	-0.09054	0.334205	0.029853	0.743474	0.045401	0.620293	0.075254	0.417897	6749572	9.624962	9.619083	9.63007	9.460232	9.649475	9.628205	9.530935	9.744543	9.372876	9.608701	9.672312	9.638966	Mobkl3	NM_025283
6749581	97	7.78618	7.625929	7.748529	7.775577	0.010603	0.92893	-0.14965	0.230108	-0.02705	0.820261	-0.1226	0.318274	0.160251	0.20166	0.037651	0.752176	6749581	7.723557	7.687561	7.947421	7.453932	7.737804	7.68605	7.941892	7.695093	7.608601	7.798851	7.845325	7.682555	Mars2	NM_175439
6749676	98	6.429063	6.677483	6.596717	6.478038	-0.04897	0.680106	0.199445	0.119674	0.11868	0.33024	0.080766	0.500559	-0.24842	0.061838	-0.16765	0.181255	6749676	6.376017	6.422899	6.488273	6.58065	6.783384	6.668415	6.765742	6.480187	6.544223	6.322618	6.39902	6.712475	9430016H	BC071241
6749691	99	6.860558	6.923444	6.748807	6.508535	0.352024	0.137641	0.414909	0.087769	0.240272	0.292847	0.174637	0.436858	-0.06289	0.775742	0.111752	0.614706	6749691	6.911244	7.006707	6.663724	6.976044	7.308145	6.486142	6.950075	6.546192	6.750153	6.430307	6.707469	6.387828	2810022L	NM_144882
6749701	100	6.251154	6.521789	6.110361	5.846929	0.404225	0.201064	0.67486	0.048472	0.263432	0.390455	0.411428	0.193951	-0.27064	0.378246	0.140793	0.640515	6749701	6.115094	6.068306	6.570062	6.193435	7.004426	6.367507	6.448713	5.55454	6.32783	5.92765	5.882605	5.730533	Sgol2	NM_199007
6749702	101	8.214419	8.121396	7.843128	7.606656	0.607762	0.044028	0.51474	0.077808	0.236471	0.380096	0.278268	0.306156	0.093023	0.724268	0.371291	0.182801	6749702	8.484587	8.093957	8.064712	8.204103	8.27998	7.880105	8.039455	7.413235	8.076693	7.253453	7.546135	8.020381	Aox1	NM_009676
6749720	105	7.084392	7.101572	7.167761	7.030147	0.054244	0.527952	0.071425	0.410316	0.137613	0.132723	-0.06619	0.444076	-0.01718	0.839706	-0.08337	0.340279	6749720	7.052184	7.126936	7.074055	6.966673	7.191652	7.146391	7.11201	7.289937	7.101335	6.895412	7.08831	7.10672	Nif3l1	NM_022988
6749727	106	10.32934	10.55995	10.37105	10.38609	-0.05674	0.803843	0.173867	0.4541	-0.01504	0.947414	0.188907	0.417522	-0.23061	0.327215	-0.0417	0.855021	6749727	10.31087	10.40413	10.27303	10.59718	10.45768	10.625	10.52818	10.29727	10.28769	9.877478	10.37984	10.90094	Ndufb3	NM_025597
6749734	107	8.642977	8.694169	8.589888	8.651238	-0.00826	0.926368	0.042931	0.633464	-0.06135	0.498869	0.104281	0.263014	-0.05119	0.57082	0.05309	0.556936	6749734	8.765772	8.641603	8.521557	8.607809	8.81928	8.655418	8.598794	8.700678	8.470191	8.574224	8.687274	8.692215	Cflar	NM_207653
6749735	108	8.454353	8.54623	8.188296	8.406297	0.048055	0.797336	0.139933	0.461681	-0.218	0.262839	0.357935	0.083362	-0.09188	0.625417	0.266057	0.179775	6749735	8.551698	8.500531	8.310829	8.434403	8.590981	8.613307	7.987817	8.651012	7.926058	8.313627	8.39805	8.507215	Cflar	NM_207653
6749736	109	10.42851	10.31575	10.38278	10.15218	0.276327	0.055285	0.16357	0.221158	0.230597	0.098309	-0.06703	0.601443	0.112757	0.387085	0.04573	0.72029	6749736	10.51812	10.43173	10.33568	10.22998	10.53076	10.18652	10.20044	10.34262	10.60528	10.07949	10.22797	10.14909	Casp8	NM_009812
6749742	110	7.992075	8.172087	7.799826	7.997189	-0.00511	0.975663	0.174898	0.313172	-0.19736	0.259164	0.372261	0.051195	-0.18001	0.300146	0.192249	0.270737	6749742	7.88496	8.038847	8.052418	8.404204	7.908102	8.203955	7.819121	7.929717	7.650641	7.858666	7.835479	8.297423	Stradb	NM_172656
6749763	111	9.216174	9.147992	9.006891	8.930307	0.285868	0.030147	0.217686	0.080115	0.076584	0.500961	0.141102	0.230303	0.068182	0.547859	0.209284	0.090289	6749763	9.282168	9.267859	9.098496	9.132051	9.222635	9.089291	8.939378	9.143517	8.937777	8.715372	8.953333	9.122215	Fzd7	NM_008057
6749773	113	8.410371	8.381417	8.380095	8.360045	0.050326	0.772008	0.021372	0.901849	0.020051	0.907884	0.001321	0.993913	0.028954	0.867362	0.030276	0.861381	6749773	8.453542	8.449636	8.327935	8.072871	8.733218	8.338161	8.429977	8.392269	8.31804	8.21854	8.618817	8.242777	Nop58	NM_018868
6749785	114	10.90699	10.96952	10.79314	10.66436	0.242627	0.059273	0.305153	0.024555	0.128777	0.277171	0.176377	0.148914	-0.06253	0.5868	0.11385	0.33274	6749785	10.95289	10.97565	10.79243	10.95454	10.97176	10.98225	10.79279	10.8741	10.71253	10.58416	10.93175	10.47718	Bmpr2	NM_007561
6749788	115	9.608951	9.521364	9.51228	9.418342	0.190609	0.131816	0.103022	0.390869	0.093937	0.432162	0.009085	0.938212	0.087587	0.46277	0.096671	0.419426	6749788	9.56144	9.543734	9.721679	9.458707	9.52282	9.582566	9.465498	9.610753	9.460588	9.670975	9.385858	9.198194	Fam117b	NM_001037725
6749804	116	6.530334	6.632532	6.537422	6.596657	-0.06632	0.528554	0.035875	0.73081	-0.05924	0.572523	0.09511	0.372472	-0.1022	0.33978	-0.00709	0.945607	6749804	6.378374	6.569137	6.643492	6.60495	6.630862	6.661783	6.481164	6.443772	6.687329	6.433935	6.61287	6.743166	Carf	NM_139150
6749813	117	8.128402	8.041113	8.044483	8.107064	0.021339	0.849411	-0.06595	0.561236	-0.06258	0.581003	-0.00337	0.976051	0.087289	0.445593	0.083919	0.462722	6749813	8.173854	8.098557	8.112796	8.033397	8.024027	8.065916	8.299833	7.918449	7.915168	8.070873	8.26323	7.987088	Cyp20a1	NM_030013
6749817	118	8.057198	8.085792	8.040971	7.966612	0.090586	0.388426	0.11918	0.264515	0.074359	0.475517	0.044821	0.663792	-0.02859	0.780761	0.016228	0.874275	6749817	8.111558	8.057653	8.002384	8.021704	8.250968	7.984704	8.105717	7.956766	8.060429	7.769063	8.083035	8.047738	Abi2	NM_198127
6749852	121	6.35668	5.906784	6.21158	5.689075	0.667605	0.032591	0.217708	0.4245	0.522505	0.078101	-0.3048	0.272598	0.449897	0.120228	0.1451	0.590252	6749852	6.56722	6.084026	6.418795	6.05142	5.838962	5.829969	6.463425	5.903203	6.268112	5.742406	6.153962	5.170858	Icos	NM_017480
6749911	122	8.615098	8.903202	8.222526	8.141678	0.473421	0.036735	0.761524	0.003803	0.080848	0.680254	0.680676	0.006986	-0.2881	0.166116	0.392573	0.071553	6749911	8.797037	8.490003	8.558255	8.965079	8.89325	8.851276	8.41378	8.033891	8.219906	7.699178	8.410901	8.314954	Nrp2	NM_001077403
6749923	123	11.35226	11.43503	11.40967	11.5237	-0.17145	0.226525	-0.08868	0.517127	-0.11403	0.408946	0.025353	0.851211	-0.08277	0.54472	-0.05742	0.672452	6749923	11.29397	11.33674	11.42606	11.30293	11.55392	11.44823	11.66591	11.44732	11.11579	11.45173	11.60123	11.51815	Eef1b2	NM_018796
6749937	126	6.063357	6.220722	6.246132	6.142968	-0.07961	0.588863	0.077754	0.597425	0.103163	0.486471	-0.02541	0.861863	-0.15737	0.298087	-0.18277	0.232246	6749937	6.066868	6.282529	5.840675	6.160489	6.487808	6.01387	6.336542	6.137021	6.264832	6.106192	6.193784	6.128929	Fastkd2	NM_172422
6750007	127	8.696477	8.729819	8.73059	8.594955	0.101522	0.158507	0.134864	0.072684	0.135635	0.071361	-0.00077	0.990862	-0.03334	0.623314	-0.03411	0.61543	6750007	8.645222	8.777372	8.666837	8.650937	8.855436	8.683083	8.722414	8.784006	8.68535	8.524065	8.677279	8.583521	Creb1	NM_133828
6750009	128	8.663793	8.659392	8.620492	8.497565	0.166227	0.246743	0.161826	0.258438	0.122926	0.382445	0.0389	0.77739	0.004401	0.974417	0.043301	0.753133	6750009	8.575937	8.811515	8.603926	8.478827	8.791564	8.707784	8.715981	8.644789	8.500705	8.25346	8.70131	8.537926	Creb1	NM_133828
6750086	129	7.815867	7.83002	7.829159	7.669212	0.146655	0.14264	0.160808	0.112482	0.159947	0.114132	0.000861	0.992618	-0.01415	0.879211	-0.01329	0.886498	6750086	7.871532	7.792618	7.783451	7.774396	7.981191	7.734473	7.946129	7.793132	7.748216	7.559954	7.819854	7.627827	Pikfyve	NM_011086
6750105	131	6.162635	6.138948	6.257466	6.174642	-0.01201	0.935786	-0.03569	0.811022	0.082824	0.582085	-0.11852	0.435635	0.023687	0.873793	-0.09483	0.529866	6750105	5.980408	6.209748	6.29775	6.039913	6.207548	6.169384	6.407118	6.08592	6.279361	6.213538	6.406802	5.903587	Mtap2	NM_001039934
6750149	133	7.915272	7.99868	7.991844	7.83138	0.083892	0.470154	0.1673	0.169049	0.160464	0.185112	0.006836	0.952263	-0.08341	0.472638	-0.07657	0.508572	6750149	7.871459	7.935137	7.939221	7.916558	8.178885	7.900597	8.185055	7.839355	7.951123	7.683084	7.907925	7.903131	Rpe	NM_025683
6750351	136	8.544328	8.539342	8.356469	8.250409	0.293919	0.025983	0.288933	0.027912	0.10606	0.35399	0.182873	0.12824	0.004986	0.964242	0.187859	0.119552	6750351	8.568919	8.418444	8.645621	8.447132	8.681095	8.489799	8.500808	8.227588	8.341011	8.109712	8.405327	8.236188	Atic	NM_026195
6750413	138	7.328777	7.527582	7.531151	7.405161	-0.07638	0.571193	0.122421	0.371695	0.125991	0.358632	-0.00357	0.978665	-0.19881	0.162909	-0.20237	0.156368	6750413	7.438776	7.330224	7.21733	7.602082	7.617519	7.363145	7.78721	7.390856	7.415388	7.317741	7.335915	7.561826	Xrcc5	NM_009533
6750433	139	7.627702	7.751463	7.713225	7.750369	-0.12267	0.330168	0.001094	0.992849	-0.03714	0.761605	0.038237	0.754848	-0.12376	0.326132	-0.08552	0.490375	6750433	7.482255	7.698954	7.701897	7.756993	7.76912	7.728275	7.583691	7.92441	7.631575	7.568948	7.746323	7.935835	Smarcal1	NM_018817
6750440	140	7.034909	6.864792	6.521596	6.678044	0.356865	0.154774	0.186748	0.43477	-0.15645	0.510417	0.343197	0.169224	0.170117	0.475303	0.513313	0.053719	6750440	6.826579	7.077104	7.201044	6.661761	7.167719	6.764897	6.598368	6.326339	6.64008	6.221567	7.034095	6.77847	Igfbp2	NM_008342
6750532	143	7.55273	7.649212	7.486787	7.718965	-0.16624	0.17656	-0.06975	0.551281	-0.23218	0.072205	0.162425	0.185575	-0.09648	0.414669	0.065943	0.572766	6750532	7.762763	7.477077	7.41835	7.685785	7.638403	7.623448	7.531295	7.504453	7.424614	7.507068	7.764034	7.885793	Pnkd	NM_001039509
6750546	144	8.536929	8.417677	8.680296	7.60872	0.928209	0.016649	0.808957	0.03023	1.071576	0.008291	-0.26262	0.418232	0.119252	0.708465	-0.14337	0.653705	6750546	8.686607	8.57495	8.34923	8.448316	8.670482	8.134233	9.013082	8.194314	8.833492	7.739104	8.061269	7.025787	Slc11a1	NM_013612
6750547	145	9.35395	9.511725	9.456227	9.434237	-0.08029	0.69298	0.077488	0.703065	0.02199	0.913478	0.055498	0.784358	-0.15778	0.444327	-0.10228	0.616112	6750547	9.345718	9.39967	9.316462	9.560394	9.59848	9.376301	9.518948	9.850872	8.998861	9.2412	9.580351	9.481159	Ctdsp1	NM_153088
6750552	147	8.149503	8.249674	8.229862	8.123719	0.025784	0.833639	0.125955	0.320081	0.106143	0.397771	0.019811	0.871718	-0.10017	0.423697	-0.08036	0.51792	6750552	8.132839	8.242397	8.073274	8.163132	8.428415	8.157474	8.418446	8.185218	8.085923	7.943923	8.213652	8.213582	Rqcd1	NM_021383
6750555	148	6.793658	6.65149	6.485067	6.763388	0.030271	0.870839	-0.1119	0.552128	-0.27832	0.161259	0.166423	0.382996	0.142168	0.453141	0.308591	0.125351	6750555	6.96602	6.760818	6.654137	6.52082	6.571487	6.862164	6.402074	6.705078	6.34805	6.51553	6.657328	7.117305	Plcd4	NM_148937
6750557	149	7.764453	7.918741	7.869118	7.859864	-0.09541	0.482806	0.058877	0.661811	0.009254	0.944849	0.049623	0.711881	-0.15429	0.268153	-0.10467	0.442842	6750557	7.565119	7.706083	8.022156	7.76072	8.069798	7.925706	7.875567	7.892542	7.839246	7.691248	7.934252	7.954092	Bcs1l	NM_025784
6750558	150	6.258885	6.27627	6.2937	6.285139	-0.02625	0.733711	-0.00887	0.908194	0.008561	0.911373	-0.01743	0.820939	-0.01738	0.821399	-0.03482	0.65283	6750558	6.19921	6.329705	6.247739	6.29235	6.412181	6.124278	6.229125	6.289316	6.36266	6.335467	6.218946	6.301005	Stk36	NM_175031
6750560	151	7.456866	7.525519	7.479132	7.490469	-0.0336	0.726651	0.035051	0.715472	-0.01134	0.905779	0.046388	0.63062	-0.06865	0.480557	-0.02227	0.816411	6750560	7.425827	7.491946	7.452824	7.429707	7.669236	7.477615	7.294921	7.658023	7.484451	7.469154	7.535432	7.46682	Ttll4	NM_001014974
6750566	152	6.425121	6.231616	6.231002	6.345624	0.079497	0.676993	-0.11401	0.552592	-0.11462	0.550504	0.000614	0.997418	0.193505	0.323499	0.194119	0.322061	6750566	6.522522	6.290337	6.462504	6.414258	6.382802	5.897788	6.338921	6.389757	5.964328	6.160877	6.596228	6.279767	Cyp27a1	NM_024264
6750568	154	6.524847	6.382266	6.603405	6.423252	0.101595	0.410096	-0.04099	0.734927	0.180153	0.161845	-0.22114	0.095164	0.142581	0.257298	-0.07856	0.520487	6750568	6.582012	6.442976	6.549553	6.343642	6.30146	6.501697	6.874074	6.536213	6.399928	6.485226	6.447778	6.336751	Wnt10a	NM_009518
6750570	155	7.077592	7.07557	7.116309	6.993659	0.083933	0.537451	0.081912	0.54706	0.12265	0.374047	-0.04074	0.76251	0.002022	0.987999	-0.03872	0.773903	6750570	7.164828	6.913379	7.154569	7.228136	7.156547	6.842028	7.326984	7.052076	6.969867	7.065274	6.981929	6.933773	Cdk5r2	NM_009872
6750589	156	9.212403	9.147786	9.249639	9.234277	-0.02187	0.536994	-0.08649	0.034158	0.015362	0.662594	-0.10185	0.016986	0.064617	0.093199	-0.03724	0.304165	6750589	9.19575	9.237388	9.204072	9.186191	9.131291	9.125877	9.236783	9.322902	9.189233	9.250649	9.248674	9.203508	Fam134a	NM_170755
6750595	157	7.880238	7.89001	7.895932	8.020991	-0.14075	0.070362	-0.13098	0.088078	-0.12506	0.100831	-0.00592	0.932185	-0.00977	0.888391	-0.01569	0.821845	6750595	7.940421	7.798583	7.90171	7.988058	7.896045	7.785926	7.943061	7.971193	7.773543	8.038313	8.016891	8.007768	Zfand2b	NM_026846
6750600	158	7.063795	7.194651	7.159659	7.18192	-0.11813	0.090922	0.012731	0.841123	-0.02226	0.726664	0.034992	0.584865	-0.13086	0.065951	-0.09586	0.157546	6750600	7.086078	7.068929	7.036377	7.091345	7.144216	7.348392	7.175887	7.126023	7.177067	7.180129	7.130245	7.235386	Ankzf1	NM_026187
6750608	159	6.406304	6.478086	6.423826	6.426431	-0.02013	0.82466	0.051654	0.573043	-0.00261	0.977087	0.054259	0.554273	-0.07178	0.437857	-0.01752	0.846993	6750608	6.401901	6.49594	6.321071	6.448778	6.423054	6.562425	6.348912	6.616364	6.306203	6.351414	6.436825	6.491055	Ankzf1	NM_026187
6750611	160	7.764684	7.686433	7.660549	7.827158	-0.06247	0.53707	-0.14072	0.184409	-0.16661	0.123792	0.025884	0.796089	0.078251	0.442606	0.104136	0.313768	6750611	7.698753	7.749692	7.845608	7.587298	7.931518	7.540483	7.696708	7.67861	7.606328	7.765774	7.864416	7.851283	Stk16	NM_011494
6750616	161	6.832197	6.864125	6.79458	7.355598	-0.5234	0.008132	-0.49147	0.011149	-0.56102	0.00565	0.069545	0.6547	-0.03193	0.83648	0.037617	0.807974	6750616	6.7485	6.792288	6.955803	6.970277	6.699872	6.922226	6.518241	7.04478	6.82072	7.554824	7.209289	7.302681	Dnajb2	NM_178055
6750625	162	8.594041	8.01936	8.140636	8.159262	0.43478	0.716973	-0.1399	0.906781	-0.01863	0.987556	-0.12128	0.919137	0.574682	0.632916	0.453406	0.705508	6750625	7.407638	8.034026	10.34046	9.331412	8.201267	6.5254	9.014331	8.024645	7.382931	6.247042	9.607978	8.622765	Des	NM_010043
6750632	164	6.319224	6.363146	6.325479	6.449509	-0.13028	0.011525	-0.08636	0.062684	-0.12403	0.014585	0.037667	0.3735	-0.04392	0.30371	-0.00626	0.879501	6750632	6.280343	6.297069	6.380261	6.361074	6.350831	6.377534	6.271224	6.325249	6.379965	6.497906	6.382605	6.468017	Accn4	NM_183022
6750639	165	5.985367	5.990286	5.881202	5.992853	-0.00749	0.97153	-0.00257	0.990235	-0.11165	0.597859	0.109084	0.606157	-0.00492	0.98129	0.104165	0.622228	6750639	5.879192	5.896909	6.180001	6.115341	5.701695	6.153822	5.426682	6.112581	6.104343	6.021654	5.945673	6.011232	Inha	NM_010564
6750642	166	6.485326	6.455241	6.450907	6.391774	0.093552	0.053988	0.063466	0.164288	0.059132	0.191559	0.004334	0.919304	0.030085	0.48866	0.034419	0.430432	6750642	6.476136	6.491534	6.488308	6.486488	6.470348	6.408886	6.46994	6.466736	6.416044	6.461723	6.419915	6.293685	Stk11ip	NM_027886
6750643	167	7.309398	7.35456	7.22047	7.344131	-0.03473	0.449923	0.010429	0.817484	-0.12366	0.022216	0.13409	0.015428	-0.04516	0.331891	0.088928	0.076413	6750643	7.352139	7.279057	7.296997	7.410635	7.30911	7.343934	7.207344	7.274562	7.179504	7.272718	7.413701	7.345973	Slc4a3	NM_009208
6750868	170	9.803006	10.07282	9.972722	10.29471	-0.4917	0.030143	-0.22189	0.269214	-0.32199	0.123235	0.100098	0.606827	-0.26981	0.186846	-0.16972	0.390394	6750868	9.740111	9.688703	9.980204	10.25211	9.88981	10.07654	9.888354	10.11933	9.910481	10.57836	9.877241	10.42852	Acsl3	NM_028817
6751063	174	7.867233	7.889196	7.790771	7.758431	0.108802	0.317054	0.130765	0.235563	0.03234	0.759221	0.098425	0.362638	-0.02196	0.834838	0.076462	0.474763	6751063	7.936121	7.816048	7.849531	7.93398	7.887409	7.846199	7.915028	7.568985	7.8883	7.598604	7.81899	7.8577	Rhbdd1	NM_001122685
6751069	175	6.783285	7.132459	6.988233	6.984815	-0.20153	0.401354	0.147643	0.534367	0.003418	0.988377	0.144226	0.54364	-0.34917	0.163218	-0.20495	0.39378	6751069	6.795725	6.838308	6.715822	7.468727	6.87991	7.048739	6.775278	7.282161	6.90726	6.952019	6.621054	7.381373	Col4a3	NM_007734
6751070	176	9.355787	9.409056	9.241177	9.461976	-0.10619	0.434715	-0.05292	0.692701	-0.2208	0.125662	0.16788	0.229786	-0.05327	0.690795	0.11461	0.400684	6751070	9.265758	9.345045	9.456558	9.370064	9.48017	9.376935	9.360058	9.392838	8.970634	9.353383	9.364446	9.668099	Mff	NM_029409
6751082	177	9.56484	9.499794	9.316158	9.398853	0.165987	0.061004	0.100941	0.221827	-0.0827	0.309391	0.183636	0.042499	0.065046	0.418096	0.248682	0.011461	6751082	9.593106	9.507119	9.594296	9.437214	9.606792	9.455376	9.458109	9.293414	9.196951	9.484785	9.329674	9.382101	Agfg1	NM_010472

6751103	178	6.303327	6.265211	5.797954	5.488217	0.815109	0.023235	0.776993	0.028446	0.309737	0.31856	0.467256	0.147273	0.038116	0.899098	0.505372	0.12089	6751103	6.453273	5.836732	6.619975	6.065536	6.607528	6.122568	6.033546	5.336049	6.024268	5.741482	5.565961	5.157209	Ccl20	NM_016960
6751215	180	7.908533	8.150764	8.000659	8.425321	-0.51679	0.003571	-0.27456	0.062431	-0.42466	0.010132	0.150105	0.270802	-0.24223	0.092685	-0.09213	0.488529	6751215	7.838866	7.836821	8.049913	8.296547	8.180868	7.974877	7.910656	8.107039	7.984282	8.398986	8.227041	8.649935	Fbxo36	NM_025386
6751219	181	8.047088	7.808636	7.903575	7.21053	0.836558	0.012374	0.598107	0.050712	0.693045	0.028737	-0.09494	0.724861	0.238451	0.386574	0.143513	0.596588	6751219	8.142446	8.190365	7.808452	7.831471	8.167148	7.42729	8.064993	7.55253	8.093201	6.788349	7.420959	7.422281	Sp100	NM_013673
6751226	182	9.301317	9.46289	9.357167	9.279559	0.021758	0.823748	0.183331	0.088447	0.077608	0.435465	0.105723	0.295878	-0.05585	0.570981	-0.16157	0.125806	6751226	9.253884	9.248791	9.401277	9.436423	9.576853	9.375395	9.512977	9.307752	9.250772	9.336134	9.369724	9.13282	Cab39	NM_133781
6751231	183	10.2875	10.36815	10.33102	10.25498	0.032527	0.772569	0.113173	0.328618	0.076043	0.504366	0.03713	0.741668	-0.08065	0.479699	-0.04352	0.699619	6751231	10.42333	10.29893	10.14025	10.31926	10.34274	10.44245	10.56983	10.26647	10.15676	10.28803	10.23867	10.23823	Itm2c	NM_022417
6751264	185	6.365118	6.391372	6.353153	6.34219	0.022928	0.484672	0.049182	0.154688	0.010963	0.735152	0.038219	0.256746	-0.02625	0.425866	0.011966	0.712153	6751264	6.412418	6.34039	6.342547	6.410311	6.415774	6.348031	6.402988	6.35184	6.30463	6.357852	6.347655	6.321063	Psmd1	NM_027357
6751271	186	7.358028	7.398169	7.444451	7.574437	-0.21641	0.354586	-0.17627	0.446632	-0.12999	0.571351	-0.04628	0.838812	-0.04014	0.859912	-0.08642	0.70501	6751271	7.374418	7.27464	7.425025	7.413158	7.295874	7.485476	7.490622	7.805294	7.037437	7.741378	7.165192	7.816741	B3gnt7	NM_145222
6751300	187	7.502559	7.449307	7.544536	7.321806	0.180753	0.077838	0.127501	0.191652	0.22273	0.037443	-0.09523	0.31788	0.053252	0.567873	-0.04198	0.651217	6751300	7.420933	7.508319	7.578424	7.382966	7.578687	7.386268	7.688231	7.450381	7.494995	7.260251	7.250866	7.454301	Cops7b	NM_172974
6751304	188	7.834145	7.813818	7.736404	7.75243	0.081715	0.32026	0.061388	0.448985	-0.01603	0.84057	0.077414	0.344846	0.020327	0.798764	0.09774	0.240649	6751304	7.771595	7.854104	7.876735	7.832033	7.846996	7.762425	7.789474	7.700618	7.719121	7.558091	7.841892	7.857307	Dis3l2	NM_153530
6751332	192	8.650907	8.56604	8.416783	8.496316	0.154591	0.145313	0.069724	0.48755	-0.07953	0.430569	0.149257	0.157904	0.084868	0.401593	0.234125	0.040343	6751332	8.618567	8.647929	8.686226	8.42152	8.812839	8.46376	8.397859	8.502275	8.350214	8.448021	8.518306	8.522621	Eif4e2	NM_023314
6751338	194	8.398495	8.458455	8.329082	8.372065	0.02643	0.599507	0.08639	0.111766	-0.04298	0.399907	0.129373	0.0281	-0.05996	0.250033	0.069413	0.188994	6751338	8.42588	8.401468	8.368138	8.353578	8.521101	8.500686	8.291454	8.343639	8.352154	8.318223	8.438684	8.359289	Gigyf2	NM_146112
6751344	196	7.958069	7.996805	8.104577	7.336029	0.622039	0.024342	0.660776	0.018675	0.768547	0.009075	-0.10777	0.644274	-0.03874	0.867388	-0.14651	0.532601	6751344	8.221005	7.863196	7.790005	7.976853	8.274174	7.739388	8.368352	7.818952	8.126426	7.394961	7.621875	6.991252	Inpp5d	NM_010566
6751345	197	7.839453	7.900347	7.813182	7.721223	0.11823	0.193173	0.179124	0.063528	0.091958	0.301264	0.087165	0.325505	-0.06089	0.485204	0.026272	0.760308	6751345	7.846452	7.841589	7.830319	7.831864	7.994042	7.875135	7.87766	7.943244	7.618641	7.648133	7.726734	7.788803	Atg16l1	NM_029846
6751349	199	7.120932	7.342274	7.054198	7.252179	-0.13125	0.127918	0.090094	0.277327	-0.19798	0.033567	0.288075	0.005811	-0.22134	0.021029	0.066733	0.413047	6751349	7.04737	7.295131	7.020294	7.362029	7.423593	7.241199	7.063711	7.000016	7.098868	7.206397	7.260645	7.289496	Dgkd	NM_177646
6751362	200	6.63675	6.665121	6.356095	6.134964	0.501787	0.187876	0.530157	0.166695	0.221132	0.543459	0.309025	0.401101	-0.02837	0.937119	0.280655	0.443914	6751362	6.602353	6.488227	6.819671	6.474568	7.075456	6.445338	6.617978	5.8681	6.582208	6.031961	6.808563	5.564367	Ugt1a10	NM_201641
6751430	203	7.603256	7.65665	7.540209	7.5737	0.029556	0.771108	0.08295	0.422818	-0.03349	0.741853	0.116441	0.269744	-0.05339	0.601449	0.063047	0.538817	6751430	7.647342	7.597286	7.565139	7.653626	7.707786	7.608538	7.691206	7.409495	7.519925	7.418293	7.527255	7.775551	Sh3bp4	NM_133816
6751442	204	8.714008	8.829993	8.682042	8.955488	-0.24148	0.094903	-0.1255	0.353902	-0.27345	0.064351	0.147951	0.279433	-0.11599	0.389666	0.031966	0.808392	6751442	8.748081	8.676114	8.717829	8.901731	8.809737	8.778511	8.496777	8.925399	8.623949	8.82847	8.84121	9.196785	Agap1	NM_178119
6751469	205	8.131009	8.511832	8.336257	8.715212	-0.5842	0.004551	-0.20338	0.211719	-0.37896	0.035303	0.175575	0.274997	-0.38082	0.034625	-0.20525	0.207956	6751469	8.050049	8.376295	7.966684	8.293138	8.616015	8.626343	8.257343	8.375124	8.376303	8.712708	8.933883	8.499045	Cxcr7	NM_007722
6751505	206	8.407159	8.436279	8.529034	8.395318	0.011841	0.870508	0.040961	0.576414	0.133716	0.093845	-0.09276	0.223811	-0.02912	0.689778	-0.12188	0.121415	6751505	8.370908	8.448133	8.402435	8.315555	8.603017	8.390265	8.606936	8.519346	8.46082	8.379103	8.421636	8.385215	Cops8	NM_133805
6751520	208	8.369571	8.078098	8.162439	8.882429	-0.51286	0.461249	-0.80433	0.259468	-0.71999	0.308928	-0.08434	0.901869	0.291473	0.671703	0.207132	0.762608	6751520	8.65722	7.737258	8.714235	8.473837	7.793446	7.967012	7.856588	8.707171	7.923557	8.329189	7.833859	10.48424	Mlph	NM_053015
6751525	209	7.711443	7.596271	7.682526	7.704068	0.007375	0.916268	-0.1078	0.151394	-0.02154	0.759397	-0.08626	0.240089	0.115172	0.128613	0.028917	0.681722	6751525	7.586852	7.793401	7.754076	7.565902	7.551192	7.671718	7.77554	7.690651	7.581388	7.664473	7.750624	7.697108	Lrrfip1	NM_008515
6751535	210	6.493639	6.882669	6.240029	6.473736	0.019903	0.953332	0.408932	0.249885	-0.23371	0.49844	0.64264	0.087035	-0.38903	0.271797	0.253611	0.463753	6751535	6.525935	6.217708	6.737275	6.919533	7.291642	6.436831	6.926531	5.967257	5.826298	6.446348	6.689689	6.285172	Ramp1	NM_016894
6751538	212	6.598651	6.503546	6.623615	6.420528	0.178123	0.410229	0.083018	0.696125	0.203087	0.350877	-0.12007	0.574236	0.095105	0.655072	-0.02496	0.906085	6751538	6.599702	6.357987	6.838264	6.187564	6.547278	6.775795	6.686173	6.786379	6.398292	6.41271	6.167672	6.681201	Ube2f	NM_026454
6751544	213	7.337446	7.265132	7.252505	7.30912	0.028326	0.727095	-0.04399	0.589914	-0.05662	0.490555	0.012627	0.875978	0.072314	0.383066	0.084941	0.309946	6751544	7.453712	7.284187	7.274439	7.265857	7.396843	7.132695	7.182458	7.322639	7.252418	7.237259	7.323876	7.366226	Scly	NM_016717
6751549	215	6.446647	6.461286	6.480014	6.468856	-0.02221	0.620023	-0.00757	0.864843	0.011159	0.802096	-0.01873	0.675147	-0.01464	0.742676	-0.03337	0.460751	6751549	6.453094	6.536844	6.350004	6.429116	6.47721	6.477532	6.499415	6.486097	6.454531	6.500358	6.435278	6.470931	Fam132b	NM_173395
6751562	216	6.411578	6.392481	6.394738	6.488202	-0.07662	0.331537	-0.09572	0.232674	-0.09346	0.242907	-0.00226	0.97645	0.019097	0.803207	0.016839	0.825998	6751562	6.440719	6.303533	6.490481	6.501859	6.424762	6.250821	6.428338	6.444784	6.311093	6.501746	6.441327	6.521532	Asb1	NM_001039126
6751617	219	7.396591	7.531593	6.981497	7.381527	0.015064	0.951272	0.150066	0.547437	-0.40003	0.132594	0.550096	0.050277	-0.135	0.587522	0.415094	0.120518	6751617	7.632757	7.437776	7.119239	7.157763	7.443239	7.993777	7.167693	6.891537	6.885261	7.629786	7.107512	7.407282	Olfr1411	NM_146490
6751620	220	6.473739	6.286525	6.366582	6.401517	0.072222	0.676766	-0.11499	0.510485	-0.03494	0.839497	-0.08006	0.644436	0.187214	0.294706	0.107157	0.538965	6751620	6.537735	6.344009	6.539472	6.275761	6.368595	6.215219	6.659838	5.949865	6.490042	6.435017	6.282834	6.486699	Olfr1410	NM_146491
6751623	222	7.923169	7.699564	7.814371	7.377819	0.54535	0.055435	0.321745	0.22288	0.436552	0.110727	-0.11481	0.649847	0.223605	0.385261	0.108798	0.666826	6751623	8.432956	7.867972	7.468578	7.71862	7.829318	7.550753	8.009749	7.512569	7.920794	7.439515	7.514489	7.179453	Gpr35	NM_022320
6751634	223	7.156786	7.032916	6.71024	6.791456	0.36533	0.181617	0.24146	0.36189	-0.08122	0.753346	0.322676	0.232369	0.123871	0.633199	0.446546	0.111541	6751634	7.03166	7.024737	7.413962	7.115379	7.370196	6.613172	6.922419	6.675589	6.532712	6.383374	7.106802	6.884192	Gpc1	NM_016696
6751640	224	6.321815	6.414217	6.426277	6.625334	-0.30352	0.052191	-0.21112	0.151656	-0.19906	0.173445	-0.01206	0.930092	-0.0924	0.507528	-0.10446	0.455514	6751640	6.17749	6.346489	6.441467	6.51227	6.31238	6.418002	6.388805	6.636269	6.253756	6.565754	6.459435	6.850813	Dusp28	NM_175118
6751641	225	8.651314	8.544181	8.602345	8.494697	0.156617	0.278264	0.049484	0.722764	0.107648	0.44708	-0.05816	0.677154	0.107133	0.449176	0.048969	0.725509	6751641	8.55641	8.607086	8.790446	8.691312	8.646179	8.295052	8.504605	8.780903	8.521528	8.337375	8.63665	8.510067	Rnpepl1	NM_181405
6751645	226	6.560274	6.608706	6.628929	6.562376	-0.0021	0.978638	0.04633	0.55962	0.066553	0.407404	-0.02022	0.797202	-0.04843	0.542352	-0.06866	0.393411	6751645	6.499356	6.523458	6.658007	6.521776	6.734776	6.569567	6.688179	6.704969	6.493639	6.563611	6.527666	6.595852	Capn10	NM_011796
6751647	227	6.479754	6.507944	6.361557	6.533213	-0.05346	0.70192	-0.02527	0.855908	-0.17166	0.23843	0.146387	0.308928	-0.02819	0.839506	0.118197	0.405933	6751647	6.542244	6.234486	6.662532	6.552186	6.459235	6.512411	6.57763	6.344868	6.162173	6.563067	6.400009	6.636562	Aqp12	NM_177587
6751684	229	8.17737	7.802277	7.627849	7.640617	0.536753	0.019018	0.161659	0.403371	-0.01277	0.946155	0.174428	0.369001	0.375093	0.074849	0.549521	0.017098	6751684	8.35667	7.989165	8.186275	7.750674	7.997399	7.658757	7.614778	7.590063	7.678705	7.52	8.052999	7.348853	Sned1	NM_172463
6751687	230	8.768544	8.808151	8.795696	8.705452	0.063091	0.641708	0.102699	0.453961	0.090243	0.508785	0.012455	0.926306	-0.03961	0.769237	-0.02715	0.840372	6751687	8.694465	8.819397	8.791769	8.702795	8.940367	8.781291	8.985712	8.559593	8.841782	8.514804	8.706089	8.895464	Ppp1r7	NM_023200
6751698	232	9.978765	10.04613	10.07058	9.792168	0.186597	0.106047	0.25396	0.038186	0.278412	0.026354	-0.02445	0.817353	-0.06736	0.529325	-0.09181	0.39634	6751698	10.01606	9.963606	9.956629	9.875344	10.25214	10.0109	10.2333	9.969468	10.00897	9.726334	9.869839	9.78033	2-Sep	NM_001159719
6751699	233	6.798376	6.759864	6.823019	6.976247	-0.17787	0.056811	-0.21638	0.026834	-0.15323	0.091676	-0.06316	0.452461	0.038512	0.643011	-0.02464	0.765836	6751699	6.820078	6.768727	6.806322	6.824017	6.771297	6.684278	6.74807	7.009639	6.711349	6.908173	6.957808	7.06276	Farp2	NM_145519
6751709	234	7.115815	7.2198	7.035558	7.151865	-0.03605	0.792178	0.067935	0.621529	-0.11631	0.405032	0.184242	0.201277	-0.10398	0.454586	0.080257	0.560966	6751709	7.169565	7.188758	6.989122	7.071923	7.366242	7.221235	7.278658	7.063014	6.765003	7.225372	7.089001	7.141221	Bok	NM_016778
6751712	235	7.975828	8.0194	8.082442	8.022265	-0.04644	0.463612	-0.00286	0.963298	0.060177	0.347767	-0.06304	0.326611	-0.04357	0.490742	-0.10661	0.115194	6751712	8.026069	7.908135	7.993281	7.954001	8.006984	8.097216	8.178584	8.08963	7.979112	7.98433	7.9985	8.083964	Atg4b	NM_174874
6751717	236	7.633362	7.641701	7.699651	7.799702	-0.16634	0.032881	-0.158	0.040204	-0.10005	0.160045	-0.05795	0.395901	-0.00834	0.900476	-0.06629	0.334866	6751717	7.61204	7.649393	7.638653	7.621481	7.560884	7.742739	7.754454	7.671648	7.672852	7.78629	7.689322	7.923495	Ing5	NM_025454
6751763	238	8.236721	8.286427	8.288451	8.23062	0.006101	0.954438	0.055806	0.604396	-0.00177	0.986778	0.057576	0.593192	-0.04971	0.643875	0.00787	0.94125	6751763	8.262452	8.202747	8.244964	8.318346	8.191027	8.349907	8.445887	8.102009	8.138657	8.102386	8.203929	8.385546	Fam174a	NM_026321
6752068	242	7.900673	7.947688	7.956235	7.905747	-0.00507	0.954255	0.041941	0.637831	0.050488	0.572181	-0.00855	0.923039	-0.04702	0.598393	-0.05556	0.535086	6752068	7.956087	7.90193	7.844001	7.876144	8.001923	7.964997	8.080559	7.840918	7.947229	7.733737	7.97864	8.004864	2310035C	NM_173187
6752073	243	6.973633	6.956633	7.044487	6.693512	0.280121	0.007427	0.263121	0.010206	0.350975	0.002118	-0.08785	0.296913	0.017	0.834463	-0.07085	0.394475	6752073	7.105111	6.946373	6.869416	6.941565	7.038361	6.889973	7.168347	6.999212	6.965903	6.770368	6.625669	6.6845	Tnfrsf11	NM_009399
6752083	244	7.290839	7.409976	7.234666	7.361606	-0.07077	0.28158	0.04837	0.452766	-0.12694	0.072111	0.17531	0.021144	-0.11914	0.087835	0.056172	0.386217	6752083	7.271104	7.33077	7.270642	7.424005	7.417996	7.387928	7.27878	7.204175	7.221044	7.206611	7.476823	7.401385	Phlpp	BC059254
6752148	248	3.520366	6.424056	3.142322	4.383021	-0.86265	0.503948	2.041035	0.13642	-1.2407	0.34372	3.281733	0.028724	-2.90369	0.046305	0.378044	0.766952	6752148	3.035483	4.002281	3.523335	7.430891	7.97493	3.866346	3.546033	2.962475	2.918459	3.429959	6.62082	3.098284	Serpinb1	NM_025867
6752165	252	7.485325	7.393211	7.021268	6.618732	0.866592	0.019202	0.774479	0.031018	0.402535	0.211594	0.371943	0.245045	0.092114	0.763968	0.464057	0.15615	6752165	7.195348	7.365367	7.895259	7.479594	7.51458	7.185459	7.00359	6.678391	7.381822	6.553692	7.135316	6.167189	Serpinb8	NM_011459
6752409	254	8.518865	8.44742	8.458655	8.234273	0.284593	0.10536	0.213147	0.208733	0.224382	0.188023	-0.01123	0.94432	0.071445	0.658941	0.060211	0.709403	6752409	8.515407	8.530169	8.51102	8.24044	8.731812	8.370008	8.611572	8.559851	8.204541	8.093989	8.434834	8.173995	Mki67ip	NM_026472
6752425	255	10.67066	10.83376	10.97692	11.37954	-0.70887	0.058798	-0.54578	0.128428	-0.40261	0.246374	-0.14317	0.668286	-0.16309	0.626064	-0.30626	0.369254	6752425	10.66469	10.49809	10.84921	11.31273	10.42527	10.76327	10.61109	11.6132	10.70648	11.43868	11.06409	11.63584	Tcfcp2l1	NM_023755
6752497	256	8.275834	8.210273	8.144748	8.116194	0.15964	0.150366	0.094078	0.376005	0.028554	0.783252	0.065524	0.532156	0.065562	0.531928	0.131086	0.227823	6752497	8.264629	8.343541	8.219333	8.10333	8.34925	8.178238	8.30772	7.947921	8.178604	8.074861	8.058795	8.214927	Tmem185b	NM_146103
6752529	258	6.427346	6.409506	6.437061	6.598264	-0.17092	0.023272	-0.18876	0.01489	-0.1612	0.029764	-0.02755	0.66391	0.01784	0.777685	-0.00971	0.877583	6752529	6.478971	6.483148	6.319918	6.392872	6.343859	6.491786	6.455736	6.49453	6.360916	6.655004	6.539686	6.600101	C1ql2	NM_207233
6752539	259	6.363582	6.328853	6.257773	6.194645	0.168936	0.122383	0.134208	0.207238	0.063127	0.536706	0.07108	0.488089	0.034729	0.731706	0.105809	0.310849	6752539	6.430648	6.267836	6.392261	6.329249	6.223562	6.433748	6.304655	6.217736	6.250927	6.409408	6.138147	6.036381	En1	NM_010133
6752571	260	7.383799	7.533095	7.454935	7.411987	-0.02819	0.701107	0.121108	0.125718	0.042948	0.561147	0.07816	0.301971	-0.1493	0.06814	-0.07114	0.344702	6752571	7.425542	7.398638	7.327218	7.43824	7.661381	7.499664	7.336815	7.482084	7.545907	7.351347	7.440956	7.443659	Ccdc93	NM_001025156
6752730	261	8.766537	9.008534	8.656525	8.583313	0.183224	0.186914	0.42522	0.010085	0.073211	0.57999	0.352009	0.024187	-0.242	0.093058	0.110013	0.411386	6752730	8.676799	8.739446	8.883367	8.907561	9.076417	9.041623	8.940007	8.46541	8.564157	8.475859	8.558602	8.715479	Slc35f5	NM_028787
6752732	262	6.120985	6.337987	6.111961	5.898962	0.222023	0.265423	0.439025	0.045407	0.212999	0.283851	0.226026	0.257568	-0.217	0.27555	0.009024	0.962377	6752732	5.910398	6.017306	6.435251	6.006783	6.55544	6.451739	6.090765	5.919814	6.325304	5.953757	5.845109	5.898021	Gpr39	NM_027677
6752848	263	9.845129	9.726982	9.919584	9.421739	0.42339	0.023113	0.305242	0.078014	0.497845	0.010937	-0.1926	0.23815	0.118148	0.456729	-0.07446	0.635387	6752848	9.916212	9.957893	9.661283	9.589111	9.773155	9.818679	10.13847	9.63362	9.986663	9.224082	9.518957	9.522179	Mgat5	NM_145128
6752869	264	8.640707	8.673227	8.555217	8.598676	0.042031	0.524689	0.074551	0.272009	-0.04346	0.511088	0.11801	0.098802	-0.03252	0.620748	0.08549	0.213108	6752869	8.647622	8.686271	8.588229	8.592192	8.784791	8.642698	8.494308	8.546178	8.625166	8.550974	8.696697	8.548357	Ccnt2	NM_028399
6752873	265	8.061665	8.095427	8.08862	8.151875	-0.09021	0.093564	-0.05645	0.267913	-0.06326	0.218854	0.006807	0.889389	-0.03376	0.496623	-0.02695	0.585257	6752873	8.083782	8.028378	8.072835	8.064938	8.125128	8.096214	8.171001	7.999741	8.095118	8.077135	8.176403	8.202088	Rab3gap1	NM_178690
6752884	266	8.981005	8.878409	8.988548	8.925912	0.055093	0.646233	-0.0475	0.691765	0.062636	0.602503	-0.11014	0.368369	0.102595	0.400465	-0.00754	0.949547	6752884	9.033039	8.99633	8.913645	8.875126	9.026358	8.733744	8.798845	9.210349	8.95645	8.880042	9.050124	8.847569	R3hdm1	NM_181750
6752885	267	9.562674	9.537124	9.637478	9.512183	0.050491	0.550233	0.024941	0.765906	0.125295	0.160315	-0.10035	0.250285	0.02555	0.760402	-0.0748	0.382543	6752885	9.578137	9.633508	9.476376	9.481415	9.661187	9.46877	9.633286	9.682857	9.596291	9.456373	9.671128	9.409047	Ubxn4	NM_026390
6752980	269	6.753339	6.964975	6.867569	7.113705	-0.36037	0.039251	-0.14873	0.339482	-0.24614	0.131276	0.097406	0.524616	-0.21164	0.186365	-0.11423	0.457772	6752980	6.711082	6.662255	6.88668	7.043193	6.702383	7.149349	6.957389	7.008875	6.636444	6.977791	7.256467	7.106858	Yod1	NM_178691

6752988	270	7.431756	7.692871	7.197161	7.56626	-0.1345	0.613085	0.126611	0.63375	-0.3691	0.186814	0.49571	0.088491	-0.26112	0.336984	0.234595	0.385654	6752988	7.046781	7.298987	7.9495	8.080972	7.44345	7.554191	7.078536	7.067534	7.445413	7.67706	7.57197	7.449749	AA986860	NM_177604
6752994	271	5.94879	6.395666	6.22955	5.558186	0.390604	0.348885	0.83748	0.065456	0.671364	0.125607	0.166116	0.683336	-0.44688	0.287926	-0.28076	0.49484	6752994	5.778744	5.862761	6.204865	6.583739	6.35179	6.251469	7.015903	5.344098	6.32865	5.874337	5.65238	5.147842	Fcamr	NM_144960
6753003	272	5.963893	7.576052	6.38254	6.202175	-0.23828	0.761113	1.373877	0.107247	0.180365	0.817763	1.193511	0.153727	-1.61216	0.065946	-0.41865	0.595548	6753003	5.944891	5.793214	6.153574	9.323568	6.705071	6.699516	7.345683	5.547887	6.254051	6.705018	6.280741	5.620766	Pigr	NM_011082
6753033	274	7.676028	7.607286	7.662215	7.435271	0.240757	0.017227	0.172014	0.064859	0.226944	0.022415	-0.05493	0.513833	0.068743	0.417478	0.013813	0.867874	6753033	7.643383	7.645	7.739702	7.587749	7.710553	7.523555	7.79285	7.526261	7.667534	7.348849	7.536239	7.420726	Lgtn	NM_001136070
6753068	277	7.18607	7.032512	7.070648	6.274439	0.91163	0.029818	0.758072	0.059602	0.796208	0.050175	-0.03814	0.914855	0.153558	0.668593	0.115422	0.746992	6753068	7.337169	7.153549	7.067491	7.007054	7.357549	6.732932	7.531069	6.429967	7.250907	6.042087	6.871985	5.909246	5430435G	NM_145509
6753083	279	9.727959	9.760297	9.736516	10.04552	-0.31756	0.101241	-0.28522	0.134891	-0.309	0.109282	0.023782	0.893148	-0.03234	0.855142	-0.00856	0.961433	6753083	9.713614	9.786657	9.683605	9.799136	9.786732	9.695024	9.538461	10.01249	9.658596	9.815588	9.895761	10.42521	Slc41a1	NM_173865
6753084	280	7.352001	7.381977	7.180911	7.122181	0.22982	0.023037	0.259796	0.013189	0.05873	0.493959	0.201066	0.039712	-0.02998	0.723996	0.17109	0.070256	6753084	7.427259	7.327502	7.301242	7.353903	7.519542	7.272487	7.198906	7.116827	7.227	7.058667	7.271063	7.036814	Rab7l1	NM_144875
6753085	281	9.593621	9.679622	9.609579	9.661378	-0.06776	0.482608	0.018244	0.847803	-0.0518	0.588958	0.070043	0.468442	-0.086	0.377398	-0.01596	0.866647	6753085	9.681206	9.631472	9.468186	9.596216	9.662376	9.780275	9.706016	9.530878	9.591843	9.53674	9.622946	9.824449	Nucks1	NM_175294
6753089	282	5.988778	6.074649	5.887404	6.154878	-0.1661	0.391183	-0.08023	0.673093	-0.26747	0.182504	0.187245	0.336759	-0.08587	0.651845	0.101374	0.59523	6753089	5.984038	6.018239	5.964057	6.255269	5.864159	6.10452	6.12775	5.916314	5.618149	6.035008	5.922445	6.50718	Slc45a3	NM_145977
6753091	283	9.557451	9.546078	9.355905	9.282072	0.275379	0.002513	0.264006	0.003205	0.073833	0.279158	0.190173	0.017332	0.011373	0.862522	0.201546	0.013216	6753091	9.573981	9.536336	9.562037	9.523624	9.59949	9.51512	9.242194	9.474664	9.350858	9.205523	9.382139	9.258555	Elk4	NM_007923
6753108	284	7.730496	8.010873	8.172118	7.738043	-0.00755	0.96276	0.27283	0.119774	0.434075	0.02427	-0.16124	0.333482	-0.28038	0.111298	-0.44162	0.022533	6753108	7.611806	7.927617	7.652065	8.10754	8.053003	7.872076	8.036188	8.148292	8.331873	7.412048	7.877867	7.924214	Klhdc8a	NM_144810
6753110	285	8.001126	8.533128	8.312635	8.439353	-0.43823	0.05461	0.093775	0.643149	-0.12672	0.533621	0.220493	0.290489	-0.532	0.025811	-0.31151	0.148481	6753110	7.767115	8.262209	7.974053	8.638062	8.435947	8.525376	8.325076	8.182678	8.430151	8.274499	8.175642	8.867919	Nuak2	NM_028778
6753126	286	7.523626	7.730535	7.549817	7.527987	-0.00436	0.946719	0.202548	0.012564	0.02183	0.738872	0.180718	0.021236	-0.20691	0.011331	-0.02619	0.689669	6753126	7.527273	7.655819	7.387786	7.682382	7.794035	7.715187	7.555204	7.586108	7.508139	7.49773	7.565657	7.520574	Dstyk	NM_172516
6753135	287	7.23216	7.286888	7.203161	7.102287	0.129873	0.098491	0.184602	0.028929	0.100874	0.184558	0.083728	0.262559	-0.05473	0.453528	0.028999	0.687346	6753135	7.195445	7.260842	7.240192	7.17929	7.345203	7.336172	7.169657	7.260109	7.179716	6.991666	7.244207	7.070987	Rbbp5	NM_172517
6753179	290	7.581138	7.479488	7.625886	7.537488	0.04365	0.58072	-0.058	0.466352	0.088398	0.277332	-0.1464	0.08966	0.10165	0.216938	-0.04475	0.571437	6753179	7.645287	7.539329	7.558798	7.622053	7.412301	7.404111	7.647281	7.696858	7.533519	7.562153	7.43244	7.61787	Ppp1r15b	NM_133819
6753185	291	7.028314	6.939799	6.873094	6.732412	0.295901	0.015994	0.207386	0.065524	0.140682	0.186003	0.066704	0.512133	0.088515	0.389301	0.155219	0.149116	6753185	7.040335	6.96402	7.080586	7.078301	6.947143	6.793952	6.909091	6.779763	6.930429	6.623437	6.918907	6.654893	Plekha6	NM_182930
6753189	292	6.048725	6.121294	6.068307	6.079441	-0.03072	0.744383	0.041853	0.657795	-0.01113	0.90563	0.052987	0.576396	-0.07257	0.448179	-0.01958	0.835	6753189	6.125336	6.003371	6.017469	6.142703	6.065116	6.156064	6.049982	6.251049	5.90389	6.123916	5.952552	6.161856	Golt1a	NM_026680
6753247	296	8.231109	9.180975	8.124162	6.607907	1.623202	0.083534	2.573069	0.013958	1.516255	0.102097	1.056813	0.234193	-0.94987	0.28086	0.106947	0.899617	6753247	8.924121	8.326792	7.442414	9.161766	10.06128	8.31988	8.836056	6.517194	9.019236	5.925733	7.611944	6.286043	Chi3l1	NM_007695
6753250	297	6.727303	6.781962	6.728107	6.790036	-0.06273	0.41679	-0.00807	0.914968	-0.06193	0.422535	0.053856	0.483302	-0.05466	0.477008	-0.0008	0.991517	6753250	6.69259	6.642484	6.846835	6.909464	6.690897	6.745526	6.817143	6.720286	6.646891	6.804863	6.760929	6.804317	Mybph	NM_016749
6753268	298	5.899712	5.984925	6.163683	6.046109	-0.1464	0.399119	-0.06118	0.719395	0.117574	0.494781	-0.17876	0.308493	-0.08521	0.618202	-0.26397	0.146962	6753268	6.035141	5.95584	5.708154	6.16512	5.793921	5.995735	6.007493	6.387358	6.096198	6.248374	6.113246	5.776708	Myog	NM_031189
6753273	300	7.75251	7.912572	7.760661	7.617356	0.135154	0.183772	0.295216	0.013036	0.143305	0.161481	0.151911	0.140618	-0.16006	0.123169	-0.00815	0.932235	6753273	7.721157	7.59089	7.945482	7.835492	8.007821	7.894403	7.792097	7.785721	7.704164	7.535482	7.725377	7.591208	Cyb5r1	NM_028057
6753275	301	10.19423	10.2942	10.19925	10.22089	-0.02665	0.714805	0.07331	0.328113	-0.02163	0.766443	0.094943	0.214342	-0.09996	0.193355	-0.00502	0.944898	6753275	10.18839	10.14282	10.25149	10.29088	10.30794	10.28377	10.31054	10.23913	10.04809	10.11642	10.27247	10.27377	Adipor1	NM_028320
6753280	302	8.93284	8.914123	8.889668	8.717981	0.214859	0.143964	0.196142	0.177514	0.171687	0.231698	0.024455	0.858326	0.018717	0.891282	0.043173	0.753192	6753280	8.705616	9.06645	9.026455	8.774345	9.073709	8.894315	8.924624	8.945128	8.799251	8.59072	8.940928	8.622295	Rabif	NM_145510
6753289	303	8.584463	8.732866	8.500391	8.663226	-0.07876	0.320052	0.06964	0.376026	-0.16284	0.059765	0.232475	0.014037	-0.1484	0.080839	0.084072	0.290593	6753289	8.564999	8.615882	8.572509	8.610536	8.916323	8.671739	8.531757	8.536073	8.433343	8.711598	8.671538	8.606542	Kdm5b	NM_152895
6753338	306	9.770716	9.741551	9.687107	9.443232	0.327485	0.03491	0.29832	0.049654	0.243876	0.095604	0.054444	0.684374	0.029165	0.826959	0.083609	0.535418	6753338	9.819635	9.804592	9.687922	9.874021	9.882077	9.468556	9.625262	9.894736	9.541324	9.370903	9.509433	9.449359	Arl8a	NM_026823
6753391	309	11.67202	11.50125	11.56855	12.05293	-0.38091	0.100249	-0.55169	0.027459	-0.48438	0.045773	-0.06731	0.751119	0.170773	0.429034	0.103467	0.627399	6753391	11.55484	11.68878	11.77244	11.71286	11.45864	11.33224	11.72615	11.77271	11.2068	12.04763	11.73331	12.37786	Csrp1	NM_007791
6753394	310	8.46419	8.494375	8.267784	7.976239	0.487951	0.027002	0.518136	0.020866	0.291545	0.145137	0.226591	0.245034	-0.03018	0.871416	0.196406	0.308501	6753394	8.578387	8.297488	8.516695	8.352993	8.892707	8.237424	8.368069	8.16126	8.274023	7.89509	8.206336	7.827291	Phlda3	NM_013750
6753400	312	7.911241	7.989501	7.715323	8.579481	-0.66824	0.021131	-0.58998	0.035497	-0.86416	0.006048	0.274178	0.274266	-0.07826	0.746228	0.195918	0.425988	6753400	7.694898	7.57298	8.465846	7.973346	8.054568	7.940588	7.841973	7.543776	7.76022	8.283285	8.731988	8.72317	Lad1	NM_133664
6753409	314	8.817714	8.976815	8.941096	9.442633	-0.62492	0.004114	-0.46582	0.01815	-0.50154	0.012864	0.035719	0.826145	-0.1591	0.341635	-0.12338	0.45563	6753409	8.950296	8.908083	8.594763	8.999973	8.981497	8.948976	8.998212	9.124772	8.700305	9.462748	9.182734	9.682418	Tmem9	NM_025439
6753412	315	6.454924	6.209195	6.047513	6.022788	0.432135	0.594886	0.186407	0.817215	0.024725	0.975501	0.161682	0.841044	0.245729	0.760908	0.40741	0.615769	6753412	5.314007	5.863552	8.187212	6.926114	6.087045	5.614426	6.651881	5.85135	5.639309	5.172529	6.204676	6.69116	Cacna1s	NM_001081023
6753417	316	6.27444	6.244716	6.442024	6.087692	0.186748	0.076791	0.157024	0.126139	0.354332	0.004859	-0.19731	0.064235	0.029724	0.754835	-0.16758	0.105896	6753417	6.435009	6.261815	6.126495	6.269134	6.325856	6.139158	6.498069	6.44873	6.379274	6.000437	6.22298	6.039659	Kif21b	NM_001039472
6753430	317	6.297014	6.500156	6.456784	6.376321	-0.07931	0.090449	0.123835	0.016931	0.080463	0.086617	0.043373	0.32326	-0.20314	0.001149	-0.15977	0.004691	6753430	6.315136	6.271475	6.30443	6.584905	6.449855	6.465709	6.436223	6.460736	6.473392	6.305897	6.423464	6.399602	Ddx59	NM_026500
6753439	318	8.092548	8.327114	8.095614	8.284083	-0.19153	0.153254	0.043031	0.732136	-0.18847	0.159124	0.231501	0.092949	-0.23457	0.089399	-0.00307	0.980473	6753439	8.095604	8.104225	8.077816	8.167764	8.353346	8.460233	8.161875	8.211109	7.913857	8.264889	8.092005	8.495355	Zfp281	NM_177643
6753546	320	7.950945	8.010303	8.128946	7.967662	-0.01672	0.892818	0.042642	0.73191	0.161284	0.216445	-0.11864	0.352472	-0.05936	0.634658	-0.178	0.176866	6753546	7.890176	7.967879	7.994781	8.066375	8.151701	7.812834	8.153522	8.181458	8.051858	7.737767	8.174038	7.99118	Dennd1b	NM_181347
6753591	322	8.107498	8.11214	7.917365	7.890967	0.21653	0.030268	0.221173	0.027735	0.026398	0.756875	0.194775	0.045671	-0.00464	0.956449	0.190132	0.04987	6753591	8.079935	8.139703	8.102855	7.98133	8.240401	8.114689	7.974951	7.993999	7.783146	7.782754	7.970606	7.919542	Zbtb41	NM_172643
6753706	326	9.232147	9.225089	9.197705	9.367168	-0.13502	0.217643	-0.14208	0.196765	-0.16946	0.131578	0.027384	0.792965	0.007058	0.945955	0.034442	0.741646	6753706	9.285754	9.279883	9.130803	9.157072	9.330208	9.187987	9.334201	9.219659	9.039255	9.226527	9.348568	9.526408	Glrx2	NM_001038592
6753710	327	7.838368	7.869091	7.818197	7.843279	-0.00491	0.965944	0.025812	0.822694	-0.02508	0.827617	0.050894	0.660126	-0.03072	0.789834	0.020171	0.860904	6753710	7.833532	7.836355	7.845218	7.747041	8.069301	7.790931	8.030953	7.62603	7.797608	7.786879	7.888216	7.854741	Uchl5	NM_019562
6753917	329	7.474295	7.126382	6.651779	5.96029	1.514005	0.032783	1.166092	0.08246	0.691488	0.273078	0.474604	0.442594	0.347913	0.570122	0.822517	0.19912	6753917	7.306621	7.560915	7.55535	6.319601	7.85815	7.201396	7.223949	5.811896	6.919491	6.483954	6.532995	4.863922	Ptgs2	NM_011198
6753937	331	9.923168	9.963598	9.938231	9.903804	0.019365	0.752709	0.059795	0.343433	0.034427	0.578031	0.025368	0.680498	-0.04043	0.515181	-0.01506	0.806159	6753937	9.969889	9.910865	9.888751	9.908003	10.04769	9.935102	10.00553	9.89348	9.915682	9.821639	9.871902	10.01787	Tpr	NM_133780
6754014	332	11.22837	11.38816	11.49606	11.20495	0.023423	0.865484	0.183207	0.208443	0.29111	0.061433	-0.1079	0.443635	-0.15978	0.26695	-0.26769	0.08063	6754014	11.0693	11.29576	11.32006	11.20728	11.35594	11.60125	11.65888	11.26504	11.56426	11.15534	11.29877	11.16074	Ivns1abp	NM_054102
6754027	333	8.074517	7.906425	7.681355	7.31994	0.754577	0.015429	0.586485	0.044308	0.361415	0.180088	0.22507	0.387106	0.168092	0.513829	0.393162	0.14876	6754027	8.299584	8.058354	7.865613	7.876143	8.122063	7.721069	7.987264	7.354966	7.701836	7.189722	7.787727	6.982371	Fam129a	NM_022018
6754047	334	6.596905	6.771252	6.304327	6.559847	0.037059	0.771521	0.211405	0.124927	-0.25552	0.072078	0.466925	0.005349	-0.17435	0.195268	0.292579	0.045122	6754047	6.583233	6.688929	6.518554	6.836751	6.748226	6.728778	6.522816	6.034081	6.356083	6.406467	6.671568	6.601505	Glt25d2	NM_177756
6754086	335	9.84206	9.933699	9.839116	9.543792	0.298267	0.138332	0.389906	0.063574	0.295324	0.14175	0.094582	0.61579	-0.09164	0.626645	0.002943	0.987437	6754086	9.888344	9.780354	9.857481	9.74953	10.15525	9.896316	10.2363	9.616279	9.66477	9.397882	9.746339	9.487156	Arpc5	NM_026369
6754087	336	7.617465	7.307629	7.320349	7.087744	0.529721	0.031016	0.219885	0.309789	0.232605	0.28448	-0.01272	0.951519	0.309836	0.165037	0.297115	0.181017	6754087	7.912081	7.558833	7.38148	7.63703	7.251817	7.03404	7.517877	7.396494	7.046677	7.230916	7.088793	6.943523	Ncf2	NM_010877
6754102	337	5.858994	5.992228	5.765062	5.842711	0.016282	0.875918	0.149516	0.177028	-0.07765	0.464076	0.227166	0.054629	-0.13323	0.223602	0.093932	0.379547	6754102	5.687059	5.853478	6.036444	5.892618	6.07435	6.009715	5.853169	5.613564	5.828453	5.830502	5.780295	5.917337	Nmnat2	NM_175460
6754138	339	8.869719	8.226532	8.346229	7.482828	1.386891	0.009864	0.743704	0.108849	0.863401	0.069519	-0.1197	0.778908	0.643187	0.157295	0.52349	0.239783	6754138	9.131872	8.933725	8.543559	7.8713	8.302285	8.506011	8.753663	7.86761	8.417414	7.270626	8.357695	6.820162	Rgs16	NM_011267
6754143	340	6.336143	6.23491	6.486964	5.836706	0.499438	0.083496	0.398204	0.153699	0.650258	0.032953	-0.25205	0.34773	0.101234	0.699177	-0.15082	0.567095	6754143	6.136792	6.446241	6.425397	5.838081	6.25797	6.608678	6.184648	6.46408	6.812164	5.641923	6.211651	5.656543	Rnasel	NM_011882
6754146	341	6.49359	6.440607	6.605134	6.4955	-0.00191	0.986992	-0.05489	0.641812	0.109634	0.362659	-0.16453	0.185472	0.052983	0.65329	-0.11154	0.3548	6754146	6.428885	6.59576	6.456125	6.386735	6.309677	6.625408	6.670039	6.749824	6.39554	6.587753	6.412501	6.486247	EG433365	NM_001008426
6754149	343	8.111337	8.134963	7.890339	8.234908	-0.12357	0.623554	-0.09995	0.690584	-0.34457	0.192481	0.244623	0.341887	-0.02363	0.924665	0.220998	0.388023	6754149	7.912196	7.746392	8.675424	8.214247	8.210017	7.980624	8.154756	7.915667	7.600595	8.239641	8.337593	8.127491	Glul	NM_008131
6754205	344	8.676624	8.699486	8.601244	8.546581	0.130043	0.223174	0.152905	0.159092	0.054662	0.594012	0.098243	0.34767	-0.02286	0.82224	0.07538	0.46597	6754205	8.747982	8.642688	8.639202	8.602917	8.875439	8.620103	8.713754	8.641962	8.448015	8.41923	8.607531	8.612983	Stx6	NM_021433
6754227	345	7.755562	7.802256	7.658124	7.714995	0.040567	0.462394	0.087261	0.135439	-0.05687	0.310756	0.144132	0.025357	-0.04669	0.400233	0.097438	0.100868	6754227	7.732822	7.717165	7.816698	7.796446	7.804345	7.805976	7.780733	7.568304	7.625334	7.751319	7.672215	7.721451	Acbd6	NM_028250
6754252	346	9.298016	9.345274	9.396329	9.139936	0.15808	0.345666	0.205338	0.229282	0.256393	0.142768	-0.05105	0.754518	-0.04726	0.772148	-0.09831	0.55051	6754252	9.083038	9.328311	9.482699	9.05638	9.502756	9.476686	9.443279	9.528727	9.216981	8.980735	9.250875	9.188198	Ifrg15	NM_022329
6754263	347	9.042839	9.136914	8.816109	8.755872	0.286967	0.104737	0.381042	0.04126	0.060237	0.710981	0.320805	0.075078	-0.09408	0.565289	0.22673	0.18636	6754263	9.02769	9.172366	8.928461	9.225816	9.145723	9.039204	9.053691	8.484007	8.91063	8.567529	8.753131	8.946957	Tor1aip2	NM_172843
6754325	349	5.992935	6.208158	5.542866	5.453578	0.539358	0.270418	0.75458	0.136237	0.089288	0.8495	0.665292	0.182313	-0.21522	0.649236	0.450069	0.352113	6754325	5.730036	6.654905	5.593865	6.409239	6.741338	5.473897	5.963251	5.175923	5.489424	4.872709	6.008258	5.479766	Angptl1	NM_028333
6754359	350	6.562175	6.678361	6.603428	6.366031	0.196144	0.298535	0.31233	0.114633	0.237396	0.21533	0.074934	0.682228	-0.11619	0.52868	-0.04125	0.820997	6754359	6.654826	6.495756	6.535944	6.586339	6.656177	6.792568	6.673851	6.789046	6.347386	6.301234	6.061856	6.735004	2810025M	BC055845
6754437	352	6.867509	6.827962	6.868206	6.64854	0.218969	0.270894	0.179422	0.360891	0.219666	0.269487	-0.04024	0.833367	0.039548	0.836202	-0.0007	0.99709	6754437	6.792634	7.000676	6.809218	6.767927	6.975732	6.740226	7.078651	6.760511	6.765456	6.350883	7.073176	6.521561	Rfwd2	NM_011931
6754476	354	7.148013	7.171984	7.216608	7.195258	-0.04725	0.714917	-0.02327	0.856736	0.02135	0.868442	-0.04462	0.729978	-0.02397	0.852497	-0.0686	0.59765	6754476	7.149132	7.17137	7.123536	7.013743	7.112067	7.390143	7.310527	7.285262	7.054035	7.025489	7.165213	7.395072	Mrps14	NM_025474
6754519	356	9.873211	9.076029	9.670967	9.929519	-0.05631	0.847588	-0.85349	0.016837	-0.25855	0.388641	-0.59494	0.069202	0.797182	0.022823	0.202244	0.496091	6754519	9.965795	9.763348	9.890489	8.402281	9.113576	9.71223	9.767937	9.753339	9.491624	9.996738	10.0168	9.775019	Gas5	NR_002840
6754567	360	6.368753	6.471933	6.419487	6.417207	-0.04845	0.73163	0.054726	0.698803	0.00228	0.987077	0.052446	0.710662	-0.10318	0.471122	-0.05073	0.719624	6754567	6.409379	6.510798	6.186082	6.36572	6.733982	6.316097	6.385349	6.446959	6.426153	6.620386	6.315597	6.315639	Pigc	NM_026078
6754604	362	8.296095	8.334111	8.468135	8.207914	0.088181	0.430633	0.126197	0.268996	0.260221	0.039988	-0.13402	0.242694	-0.03802	0.72975	-0.17204	0.144059	6754604	8.251928	8.382756	8.253601	8.143457	8.447886	8.410989	8.511388	8.30377	8.589247	8.129508	8.337168	8.157065	Vamp4	NM_016796
6754666	364	7.818991	7.855412	7.887721	7.64805	0.170941	0.054355	0.207362	0.025749	0.239671	0.013432	-0.03231	0.681518	-0.03642	0.644133	-0.06873	0.391575	6754666	7.739826	7.888915	7.828233	7.815046	7.958304	7.792886	7.954363	7.825861	7.88294	7.628211	7.786153	7.529787	Kifap3	NM_010629

6754669	365	7.692208	7.858131	7.78346	7.638741	0.053467	0.615822	0.21939	0.064591	0.144719	0.195403	0.074671	0.486797	-0.16592	0.143923	-0.09125	0.399001	6754669	7.761498	7.636283	7.678844	7.835359	8.043027	7.696007	7.967169	7.699183	7.684028	7.632386	7.69673	7.587108	Scyl3	NM_028776
6754671	366	5.63869	6.14894	6.359189	5.908412	-0.26972	0.38867	0.240528	0.439825	0.450777	0.16617	-0.21025	0.497569	-0.51025	0.122932	-0.7205	0.040891	6754671	5.04241	5.66675	6.20691	6.016544	6.257824	6.172451	6.493308	6.468593	6.115666	5.511853	6.016006	6.197377	2810422O	BC052693
6754680	367	6.690076	5.848513	6.077063	5.624948	1.065128	0.019163	0.223565	0.556389	0.452115	0.249654	-0.22855	0.547822	0.841563	0.049646	0.613013	0.130863	6754680	7.434578	6.484098	6.151553	5.956962	6.010113	5.578464	6.57079	5.5064	6.154	5.582636	5.736599	5.555609	Sele	NM_011345
6754681	368	6.207169	5.863976	5.988423	5.946467	0.260702	0.210138	-0.08249	0.677749	0.041956	0.831922	-0.12445	0.53365	0.343192	0.110612	0.218746	0.285979	6754681	6.67759	5.926641	6.017275	5.715644	5.966932	5.909353	6.16847	5.87184	5.924958	5.878467	5.90428	6.056654	Sell	NM_011346
6754690	369	8.239226	7.297237	7.074631	5.633562	2.605664	0.006174	1.663675	0.046475	1.441069	0.075907	0.222606	0.760954	0.941989	0.219494	1.164594	0.13817	6754690	9.612606	7.660061	7.44501	7.605494	7.617143	6.669074	7.949172	5.91332	7.361402	5.148389	5.933802	5.818495	Selp	NM_011347
6754691	370	6.700682	7.057943	6.938497	6.708283	-0.0076	0.988903	0.34966	0.527734	0.230214	0.675333	0.119446	0.827249	-0.35726	0.519037	-0.23781	0.665377	6754691	6.859518	6.155281	7.087248	6.45644	7.946874	6.770515	7.451603	6.869489	6.494399	7.207184	5.817848	7.099817	F5	NM_007976
6754696	371	6.960524	6.960077	6.896697	6.932853	0.02767	0.873247	0.027224	0.875272	-0.03616	0.834964	0.06338	0.715756	0.000447	0.997943	0.063827	0.713858	6754696	7.074419	6.871158	6.935994	6.81053	7.194617	6.875084	7.0477	7.002769	6.639622	6.636761	7.100888	7.060911	Slc19a2	NM_054087
6754700	372	7.19888	7.358969	7.266915	7.219599	-0.02072	0.880069	0.13937	0.325331	0.047316	0.731231	0.092054	0.508457	-0.16009	0.263139	-0.06804	0.622805	6754700	7.039599	7.244147	7.312893	7.481083	7.377928	7.217896	7.463842	7.192957	7.143945	7.095535	7.116954	7.446307	4930455F	NM_029115
6754701	373	7.604779	7.633171	7.571473	7.390575	0.214204	0.111561	0.242597	0.077441	0.180899	0.169463	0.061698	0.620453	-0.02839	0.818603	0.033305	0.788045	6754701	7.518209	7.615009	7.681118	7.40184	7.82901	7.668664	7.694818	7.465007	7.554595	7.514459	7.417509	7.239756	Nme7	NM_138314
6754735	374	8.458646	8.150592	7.503541	6.93556	1.523086	0.040036	1.215032	0.086562	0.567981	0.387905	0.647051	0.328679	0.308054	0.633768	0.955105	0.163243	6754735	8.311688	8.336329	8.727921	8.179966	8.974098	7.297712	8.311641	7.313252	6.885729	5.891138	7.916583	6.998958	Dpt	NM_019759
6754798	377	9.909359	9.836305	10.0058	10.15431	-0.24495	0.16387	-0.318	0.081783	-0.14851	0.379926	-0.1695	0.319826	0.073054	0.659731	-0.09644	0.562896	6754798	10.04885	9.986865	9.692361	9.794455	9.9016	9.812859	10.16498	9.912196	9.940229	9.930447	10.02773	10.50475	Creg1	NM_011804
6754842	380	7.277095	7.413412	7.35731	7.213688	0.063408	0.579838	0.199724	0.10669	0.143622	0.227581	0.056102	0.623499	-0.13632	0.249985	-0.08021	0.486282	6754842	7.247355	7.303655	7.280276	7.323714	7.543322	7.373199	7.445344	7.365401	7.261184	6.96219	7.290112	7.388761	Tada1l	NM_030245
6754892	382	8.400862	8.438018	8.397226	8.461378	-0.06052	0.582996	-0.02336	0.830766	-0.06415	0.561036	0.040793	0.709832	-0.03716	0.73448	0.003637	0.973418	6754892	8.322714	8.444139	8.435734	8.360102	8.483358	8.470595	8.610956	8.336729	8.243992	8.31666	8.457359	8.610114	Tmco1	NM_001039483
6754893	383	8.065688	8.120717	8.198116	8.133781	-0.06809	0.262321	-0.01306	0.822841	0.064335	0.287536	-0.0774	0.207698	-0.05503	0.35835	-0.13243	0.047034	6754893	8.045357	8.055309	8.096399	8.103237	8.244981	8.013932	8.154115	8.17814	8.262094	8.089149	8.166897	8.145298	Aldh9a1	NM_019993
6755054	386	10.38115	10.59825	9.771099	9.84702	0.53413	0.068214	0.75123	0.018061	-0.07592	0.772225	0.827151	0.011483	-0.2171	0.416735	0.610051	0.042759	6755054	10.58363	10.41092	10.1489	10.6222	10.68351	10.48904	9.997023	9.430733	9.885542	9.318757	10.28019	9.942113	Rgs5	NM_009063
6755055	387	8.803706	8.758605	7.999106	8.276691	0.527015	0.128957	0.481914	0.160236	-0.27758	0.398631	0.759499	0.040596	0.045101	0.888401	0.8046	0.032396	6755055	9.183063	9.118303	8.109752	8.786537	8.813715	8.675562	8.104407	7.742558	8.150353	7.809616	8.488319	8.532137	Rgs5	ENSMUST000000
6755125	389	7.887299	7.40294	7.269404	6.986427	0.900873	0.02649	0.416513	0.244951	0.282978	0.418705	0.133535	0.697993	0.48436	0.182616	0.617895	0.099692	6755125	8.18843	8.094008	7.37946	7.459315	7.759556	6.989948	7.771162	6.894675	7.142376	6.760231	7.368585	6.830464	Olfml2b	NM_177068
6755146	390	6.781817	7.209317	7.302892	6.292688	0.489129	0.134985	0.916629	0.014325	1.010204	0.008908	-0.09358	0.758589	-0.4275	0.184285	-0.52108	0.114496	6755146	6.51808	6.680551	7.146819	6.926424	7.690603	7.010923	7.699311	6.962893	7.246471	6.214021	6.639798	6.024245	Fcgr4	NM_144559
6755154	391	5.666717	6.597066	5.569047	6.01722	-0.3505	0.285163	0.579846	0.094722	-0.44817	0.181208	1.028019	0.009941	-0.93035	0.016059	0.09767	0.757776	6755154	5.73525	5.436038	5.828864	6.811865	7.10103	5.878303	5.674285	5.771295	5.261561	5.795667	6.091467	6.164527	1700009P	NM_001081275
6755158	392	6.286252	6.009513	6.276784	6.453955	-0.1677	0.152194	-0.44444	0.003022	-0.17717	0.133099	-0.26727	0.035695	0.276739	0.031063	0.009468	0.931008	6755158	6.444109	6.168184	6.246463	5.971803	5.978528	6.078207	6.311107	6.39844	6.120806	6.56568	6.279367	6.516819	Mpz	NM_008623
6755165	394	5.890937	6.473799	6.069291	6.257904	-0.36697	0.004926	0.215895	0.053646	-0.18861	0.083668	0.404508	0.002853	-0.58286	0.000288	-0.17835	0.098745	6755165	5.993598	5.943442	5.73577	6.655522	6.435599	6.330277	5.986138	6.130491	6.091244	6.290012	6.195595	6.288105	Apoa2	NM_013474
6755168	395	6.50163	6.683432	6.401397	6.209456	0.292174	0.176423	0.473976	0.042815	0.191941	0.358542	0.282035	0.190225	-0.1818	0.383182	0.100233	0.624708	6755168	6.859792	6.388064	6.257034	6.409016	7.003684	6.637595	6.431271	6.237631	6.535289	6.057137	6.238083	6.333148	Adamts4	NM_172845
6755170	396	9.41809	9.120011	9.259968	9.842778	-0.42469	0.006917	-0.72277	0.000278	-0.58281	0.001122	-0.13996	0.268705	0.298079	0.035182	0.158122	0.216188	6755170	9.382466	9.335841	9.535963	9.152905	9.159586	9.047543	9.163437	9.42721	9.189257	10.0817	9.657533	9.789101	B4galt3	NM_020579
6755173	397	8.524757	8.505176	8.42431	8.402085	0.122671	0.064012	0.10309	0.108749	0.022225	0.707357	0.080865	0.194589	0.019581	0.740577	0.100446	0.116708	6755173	8.493776	8.431089	8.649405	8.498143	8.536425	8.480959	8.393014	8.494454	8.385463	8.370463	8.458884	8.376909	Dedd	NM_001128609
6755174	398	10.00854	10.1565	9.971814	9.896201	0.112336	0.267653	0.260299	0.024653	0.075613	0.445776	0.184686	0.085835	-0.14796	0.155241	0.036724	0.707093	6755174	9.986835	9.984467	10.05431	10.10816	10.11434	10.247	9.956168	10.12047	9.838803	9.738235	9.892188	10.05818	Pfdn2	NM_011070
6755175	399	7.674614	7.730403	7.591534	7.608213	0.066401	0.616368	0.12219	0.36562	-0.01668	0.899079	0.138869	0.307472	-0.05579	0.673055	0.08308	0.532637	6755175	7.577474	7.779825	7.666542	7.643053	7.762886	7.785271	7.761	7.368555	7.645047	7.382312	7.769536	7.672791	Pvrl4	NM_027893
6755176	400	6.734038	6.552277	6.645497	5.940205	0.793833	0.004413	0.612073	0.01648	0.705292	0.008276	-0.09322	0.657466	0.181761	0.395532	0.088541	0.673443	6755176	7.10816	6.647346	6.446608	6.502901	6.823462	6.330469	6.792372	6.515093	6.629025	5.830121	6.194731	5.795762	Arhgap30	NM_001005508
6755177	401	6.567315	6.802503	6.520514	6.480859	0.086457	0.516	0.321645	0.03537	0.039655	0.763262	0.281989	0.057517	-0.23519	0.101722	0.046801	0.722542	6755177	6.659299	6.570583	6.472064	6.713549	6.999031	6.69493	6.712718	6.443156	6.405668	6.357935	6.401189	6.683452	Usf1	NM_009480
6755179	402	9.374693	9.469274	9.332495	9.566815	-0.19212	0.044951	-0.09754	0.262495	-0.23432	0.020025	0.136778	0.129444	-0.09458	0.276128	0.042197	0.616168	6755179	9.419043	9.346296	9.358739	9.381058	9.600496	9.426267	9.331511	9.410115	9.25586	9.482776	9.494363	9.723305	F11r	NM_172647
6755184	403	7.529589	7.595779	7.378317	7.438914	0.090675	0.548777	0.156865	0.310411	-0.0606	0.686711	0.217462	0.171693	-0.06619	0.659871	0.151273	0.326876	6755184	7.427601	7.655446	7.505721	7.477357	7.741903	7.568077	7.320544	7.673463	7.140943	7.294926	7.436908	7.584909	Refbp2	NM_019484
6755197	405	6.149353	6.102578	6.190363	5.02367	1.125683	0.002749	1.078908	0.003505	1.166693	0.002231	-0.08779	0.748084	0.046775	0.863795	-0.04101	0.88042	6755197	6.359678	5.796511	6.291869	6.040062	6.472576	5.795095	6.476882	5.948427	6.14578	4.977315	5.411816	4.681878	Cd48	NM_007649
6755210	407	7.985356	8.129917	8.226352	7.064756	0.9206	0.01224	1.065162	0.005824	1.161596	0.003619	-0.09643	0.74457	-0.14456	0.626748	-0.241	0.42375	6755210	8.08357	8.042316	7.830181	8.081733	8.457011	7.851008	8.709476	7.662872	8.306708	7.19706	7.292862	6.704345	Cd84	NM_013489
6755222	409	10.53848	10.72662	10.73596	10.63608	-0.0976	0.249679	0.090537	0.282797	0.09988	0.239684	-0.00934	0.908341	-0.18813	0.043673	-0.19748	0.036286	6755222	10.45502	10.49599	10.66444	10.66771	10.80342	10.70872	10.7872	10.73818	10.6825	10.48484	10.7174	10.706	Copa	NM_009938
6755224	410	7.639528	7.884384	7.667952	7.59723	0.042298	0.792531	0.287154	0.10204	0.070722	0.661381	0.216432	0.201502	-0.24486	0.154038	-0.02842	0.859526	6755224	7.429165	7.579785	7.909633	7.829505	7.999065	7.824583	7.771492	7.704466	7.527899	7.418692	7.499089	7.873909	Pex19	NM_023041
6755227	411	9.149967	9.484444	9.06256	9.273653	-0.12369	0.272855	0.210791	0.079688	-0.21109	0.079335	0.421884	0.003861	-0.33448	0.012917	0.087407	0.429495	6755227	9.021337	9.172525	9.256039	9.499158	9.50266	9.451513	9.081143	9.043354	9.063183	9.074052	9.228539	9.518367	Wdr42a	NM_153555
6755233	412	7.388284	7.469721	7.45663	7.152576	0.235709	0.170961	0.317145	0.077601	0.304054	0.088312	0.013091	0.935475	-0.08144	0.617362	-0.06835	0.674272	6755233	7.287377	7.326168	7.551308	7.414709	7.705686	7.288768	7.707764	7.454299	7.207826	7.099178	7.050107	7.308442	Igsf8	NM_080419
6755237	413	6.381988	6.241748	6.739587	6.099705	0.282283	0.114638	0.142042	0.39904	0.639882	0.003879	-0.49784	0.014184	0.140241	0.404779	-0.3576	0.055201	6755237	6.473128	6.574737	6.0981	6.251202	6.338882	6.135159	6.563256	6.859643	6.795862	6.261372	6.206361	5.831383	Kcnj10	NM_001039484
6755238	414	6.877278	7.235599	6.918671	6.904981	-0.0277	0.833784	0.330618	0.032241	0.01369	0.917316	0.316928	0.038093	-0.35832	0.023046	-0.04139	0.754284	6755238	6.738332	6.816448	7.077054	7.091918	7.403824	7.211055	6.941844	6.928356	6.885814	6.684282	7.081244	6.949418	Pigm	NM_026234
6755240	415	7.211584	7.459111	7.341845	6.578076	0.633508	0.075411	0.881035	0.021805	0.763769	0.039186	0.117266	0.71524	-0.24753	0.447933	-0.13026	0.685593	6755240	7.625827	6.966402	7.042522	7.472432	7.631244	7.273657	7.942331	6.781454	7.301749	6.493669	6.887951	6.352607	Slamf9	NM_029612
6755241	416	7.048813	6.896197	6.960909	6.611547	0.437266	0.000707	0.28465	0.008485	0.349362	0.002781	-0.06471	0.453352	0.152616	0.100138	0.087904	0.315607	6755241	7.052158	7.099346	6.994935	6.782216	6.99715	6.909225	6.955846	6.960482	6.9664	6.641739	6.75561	6.437292	Igsf9	NM_033608
6755242	417	11.24687	11.33458	11.14416	11.04903	0.19784	0.183651	0.285547	0.068874	0.095133	0.503867	0.190413	0.198861	-0.08771	0.536855	0.102707	0.471576	6755242	11.16561	11.24371	11.33129	11.27969	11.54456	11.17948	11.25154	11.15425	11.0267	10.91758	11.32151	10.908	Tagln2	NM_178598
6755247	418	6.475573	6.41966	6.583362	6.586036	-0.11046	0.237362	-0.16638	0.090606	-0.00267	0.976093	-0.1637	0.095027	0.055914	0.536074	-0.10779	0.247929	6755247	6.484384	6.413424	6.528912	6.455444	6.256158	6.547377	6.485882	6.539793	6.72441	6.590051	6.644597	6.523459	Vsig8	NM_177723
6755282	424	7.196166	7.915124	7.266471	6.818507	0.377659	0.259272	1.096617	0.007779	0.447965	0.187743	0.648652	0.070502	-0.71896	0.049564	-0.07031	0.826832	6755282	7.181376	7.183115	7.224007	7.92599	7.429313	8.390068	7.328346	6.883315	7.587753	6.274977	7.111897	7.068646	Aim2	NM_001013779
6755294	425	5.513683	6.092279	6.005006	4.402951	1.110732	0.026036	1.689328	0.003232	1.602056	0.004352	0.087272	0.835803	-0.5786	0.193486	-0.49132	0.262454	6755294	5.761095	5.535094	5.244859	6.678277	6.010789	5.58777	6.136515	5.521636	6.356868	3.821774	4.261426	5.125652	Olfr433	NM_146717
6755298	428	5.928516	5.841647	6.176101	5.977216	-0.0487	0.893379	-0.13557	0.710169	0.198885	0.587551	-0.33445	0.369847	0.086868	0.811287	-0.24759	0.501776	6755298	5.912304	5.861126	6.012117	5.023688	6.347356	6.153898	5.841968	6.498149	6.188186	6.245626	5.588894	6.097129	Olfr429	NM_146722
6755451	442	6.723298	6.965174	6.615961	6.543521	0.179777	0.157837	0.421653	0.006454	0.07244	0.547628	0.349213	0.016397	-0.24188	0.069343	0.107337	0.379461	6755451	6.810751	6.635285	6.723858	6.907419	7.02837	6.959732	6.724229	6.464574	6.65908	6.301024	6.743215	6.586323	Sdccag8	NM_029756
6755494	443	8.526399	8.554114	8.383742	8.203937	0.322462	0.009403	0.350177	0.00614	0.179805	0.094819	0.170372	0.110431	-0.02771	0.777749	0.142658	0.17129	6755494	8.515549	8.409434	8.654215	8.485534	8.64778	8.529027	8.537794	8.422056	8.191375	8.182088	8.23689	8.192833	Zfp238	NM_001012330
6755540	444	6.949995	7.021482	6.669941	6.989518	-0.03952	0.724256	0.031964	0.775099	-0.31958	0.018288	0.351541	0.011693	-0.07149	0.52719	0.280054	0.03214	6755540	7.004336	6.96636	6.879288	6.95971	7.173314	6.931422	6.477827	6.864913	6.667082	7.008999	6.875705	7.083849	Pppde1	NM_024282
6755621	448	7.279969	7.237857	7.338675	7.318194	-0.03823	0.814427	-0.08034	0.623921	0.020481	0.899791	-0.10082	0.54017	0.042112	0.79603	-0.05871	0.719146	6755621	7.02382	7.40622	7.409868	7.262116	7.18505	7.266405	7.284626	7.535606	7.195794	7.023865	7.505744	7.424974	9630058J	BC054802
6755630	449	7.774182	8.030728	7.755008	8.507675	-0.73349	0.010489	-0.47695	0.0627	-0.75267	0.009221	0.27572	0.2469	-0.25655	0.278598	0.019174	0.932907	6755630	7.625659	7.933186	7.763701	8.063957	8.10612	7.922106	7.995707	7.686734	7.582582	9.028557	8.345318	8.149151	Sccpdh	NM_178653
6755672	450	7.821083	7.82042	7.836974	7.625265	0.195817	0.016893	0.195155	0.017157	0.211709	0.011685	-0.01655	0.805751	0.000663	0.99213	-0.01589	0.813352	6755672	7.821352	7.788385	7.853511	7.801917	7.839729	7.819614	7.957411	7.673397	7.880115	7.576378	7.675654	7.623764	Parp1	NM_007415
6755687	452	9.250183	9.253358	9.323655	9.335114	-0.08493	0.395132	-0.08176	0.412246	-0.01146	0.906493	-0.0703	0.478317	-0.00317	0.974026	-0.07347	0.459341	6755687	9.224946	9.257039	9.268565	9.141162	9.423823	9.19509	9.321867	9.42592	9.223177	9.318042	9.485337	9.201963	Acbd3	NM_133225
6755700	453	7.765268	7.933908	7.830882	7.840136	-0.07487	0.27806	0.093772	0.18306	-0.00925	0.889185	0.103026	0.147949	-0.16864	0.030587	-0.06561	0.337623	6755700	7.725398	7.858177	7.712229	7.815587	8.009862	7.976274	7.882082	7.800377	7.810187	7.770145	7.918367	7.831895	BC031781	BC031781
6755711	454	7.427354	7.396791	7.380727	7.410564	0.01679	0.861682	-0.01377	0.886307	-0.02984	0.757343	0.016064	0.867593	0.030563	0.75167	0.046627	0.630747	6755711	7.344714	7.430479	7.506869	7.362922	7.425665	7.401786	7.399631	7.540071	7.202479	7.346654	7.329224	7.555815	Pycr2	NM_133705
6755714	456	9.418791	9.234627	9.33292	9.182483	0.236308	0.048365	0.052144	0.621451	0.150437	0.176726	-0.09829	0.361368	0.184165	0.107271	0.085871	0.422288	6755714	9.462232	9.302313	9.491829	9.290689	9.273487	9.139704	9.510796	9.231359	9.256606	9.096008	9.348746	9.102695	Tmem63a	NM_144794
6755729	457	9.386374	9.628152	9.73601	9.662963	-0.27659	0.134367	-0.03481	0.839204	0.073047	0.67167	-0.10786	0.534219	-0.24178	0.183505	-0.34964	0.068358	6755729	9.504974	9.127848	9.5263	9.584874	9.863968	9.435615	9.791779	9.905702	9.510549	9.653522	9.830507	9.504859	Cnih4	NM_030131
6755845	461	8.510455	8.692573	8.656821	9.011984	-0.50153	0.014766	-0.31941	0.084156	-0.35516	0.059725	0.035752	0.83088	-0.18212	0.293584	-0.14637	0.392708	6755845	8.398054	8.675087	8.458223	8.645714	8.619877	8.812128	8.537493	8.871504	8.561466	8.834194	8.842105	9.359653	Trp53bp2	NM_173378
6755856	463	5.91721	6.467411	5.98556	6.048742	-0.13153	0.642068	0.418669	0.162788	-0.06318	0.82237	0.481852	0.11482	-0.5502	0.078034	-0.06835	0.808168	6755856	6.193731	5.769558	5.78834	6.247062	6.925261	6.229911	5.635678	6.191931	6.12907	5.946154	5.740003	6.460069	Susd4	NM_144796
6755896	465	7.050615	6.680087	6.53381	6.588284	0.462331	0.368036	0.091803	0.854479	-0.05447	0.913277	0.146276	0.770485	0.370528	0.466498	0.516805	0.317383	6755896	6.960911	6.656244	7.53469	6.840416	7.051832	6.148012	7.015824	6.414505	6.171102	6.767224	7.378443	5.619184	Dusp10	NM_022019
6755966	466	8.916977	9.00623	8.686984	9.104596	-0.18762	0.180545	-0.09837	0.463907	-0.41761	0.011435	0.319246	0.037158	-0.08925	0.505021	0.229993	0.109827	6755966	8.871426	8.820894	9.058611	9.016706	8.977892	9.024092	8.726864	8.635005	8.699083	9.006035	8.884933	9.42282	Rab3gap2	BC057872
6755975	467	8.331694	8.435039	8.156516	8.640911	-0.30922	0.128971	-0.20587	0.292422	-0.48439	0.029182	0.278523	0.165849	-0.10335	0.587094	0.175178	0.365666	6755975	8.205521	8.209329	8.580233	8.631603	8.454999	8.218516	8.28896	8.167939	8.01265	8.528649	8.410165	8.983918	Bpnt1	NM_011794

6756115	469	7.230407	7.196187	7.164191	7.211597	0.018811	0.816276	-0.01541	0.84896	-0.04741	0.561853	0.031996	0.693671	0.034221	0.673786	0.066217	0.422523	6756115	7.248484	7.142688	7.30005	7.236619	7.304068	7.047873	7.103384	7.244347	7.144841	7.131135	7.303275	7.20038	Gpatch2	NM_026367
6756238	472	9.286431	9.431736	8.957886	8.957905	0.328526	0.016897	0.473831	0.00249	-2E-05	0.999861	0.47385	0.002489	-0.1453	0.220228	0.328546	0.016892	6756238	9.19966	9.255723	9.403911	9.432037	9.558098	9.305073	8.7904	9.124304	8.958953	8.809387	9.040572	9.023757	Ptpn14	NM_008976
6756334	473	8.067013	8.199872	8.141565	8.166839	-0.09983	0.187294	0.033033	0.64602	-0.02527	0.724522	0.058306	0.424127	-0.13286	0.091242	-0.07455	0.312932	6756334	8.066423	8.052421	8.082196	8.179691	8.311437	8.108487	8.252906	8.124724	8.047066	8.099775	8.139106	8.261636	Angel2	NM_021421
6756358	476	7.706648	7.88805	7.801198	7.611863	0.094785	0.427065	0.276187	0.040698	0.189335	0.133217	0.086852	0.465314	-0.1814	0.147994	-0.09455	0.428168	6756358	7.714356	7.748115	7.657303	7.813272	7.926024	7.924855	8.053297	7.730621	7.619676	7.533805	7.774996	7.526789	Tmem206	NM_025864
6756383	477	7.893954	7.925335	7.964186	7.732053	0.161901	0.297936	0.193282	0.220497	0.232133	0.14912	-0.03885	0.796115	-0.03138	0.834562	-0.07023	0.642089	6756383	7.921604	7.806823	7.953434	7.881882	8.010251	7.883871	8.20286	7.620287	8.069412	7.571556	7.756839	7.867764	Ints7	NM_178632
6756386	478	7.53612	7.492345	7.374899	7.256202	0.279918	0.076245	0.236143	0.124332	0.118697	0.413265	0.117446	0.417997	0.043775	0.75843	0.161221	0.274853	6756386	7.423209	7.439691	7.74546	7.344899	7.669204	7.462931	7.454717	7.210528	7.459451	7.075629	7.440853	7.252123	Lpgat1	NM_001134829
6756394	479	5.967947	6.394516	5.858624	5.50177	0.466177	0.154733	0.892746	0.016832	0.356854	0.263408	0.535892	0.108488	-0.42657	0.188409	0.109323	0.722056	6756394	5.997564	5.676816	6.229462	6.102469	6.993924	6.087155	6.118027	5.367068	6.090778	5.490399	5.503439	5.511472	Nek2	NM_010892
6756403	480	8.466105	8.545233	8.284855	8.436832	0.029272	0.796823	0.1084	0.353125	-0.15198	0.204324	0.260377	0.045403	-0.07913	0.492277	0.181249	0.137921	6756403	8.542669	8.482117	8.373528	8.383033	8.699755	8.55291	8.473101	8.244796	8.136669	8.34766	8.413408	8.549429	Slc30a1	NM_009579
6756408	481	7.004264	6.915972	6.940524	7.03068	-0.02642	0.874904	-0.11471	0.500324	-0.09016	0.594225	-0.02455	0.883656	0.088292	0.601738	0.06374	0.705145	6756408	7.153878	6.859105	6.999808	7.015969	6.618515	7.113432	7.039289	7.122658	6.659625	7.080544	6.933558	7.077939	Rd3	NM_023727
6756419	482	5.899334	6.045355	5.828564	5.7768	0.122534	0.423961	0.268555	0.102013	0.051763	0.731103	0.216792	0.17439	-0.14602	0.344762	0.07077	0.639584	6756419	5.811686	5.776094	6.110222	5.999427	6.211712	5.924927	6.011629	5.534914	5.939148	5.816837	5.819266	5.694298	Kcnh1	NM_010600
6756473	483	9.682857	9.654752	9.66542	9.4852	0.197657	0.104049	0.169552	0.154376	0.180221	0.133082	-0.01067	0.923594	0.028105	0.800885	0.017436	0.875507	6756473	9.531525	9.710008	9.807037	9.570506	9.726599	9.66715	9.756279	9.78071	9.459272	9.492887	9.590502	9.37221	Irf6	NM_016851
6756474	484	6.486901	6.57979	6.648983	6.813817	-0.32692	0.037028	-0.23403	0.11148	-0.16483	0.243259	-0.06919	0.611287	-0.09289	0.497921	-0.16208	0.250555	6756474	6.244094	6.648412	6.568196	6.5657	6.574548	6.599122	6.745275	6.396787	6.804886	6.761446	6.762868	6.917137	A130010J	BC055955
6756478	485	9.530795	8.91141	8.808893	7.530582	2.000212	0.001038	1.380827	0.008574	1.27831	0.012562	0.102517	0.80378	0.619385	0.159309	0.721902	0.108113	6756478	9.890554	9.143559	9.558271	9.035668	9.31882	8.379741	9.549105	8.071434	8.806139	7.446769	7.753049	7.391929	Lamb3	NM_008484
6756536	486	8.228907	8.374855	8.994221	8.501686	-0.27278	0.195173	-0.12683	0.529445	0.492535	0.03404	-0.61937	0.012427	-0.14595	0.471114	-0.76531	0.004141	6756536	8.456019	8.134916	8.095787	8.672761	8.320225	8.131579	8.994112	8.868282	9.12027	8.514336	8.800123	8.190599	Plxna2	NM_008882
6756541	487	10.0103	10.10744	9.875786	9.724508	0.285789	0.245479	0.382929	0.131603	0.151278	0.52571	0.231651	0.339415	-0.09714	0.681334	0.13451	0.571545	6756541	10.12936	10.10978	9.791749	10.00243	10.36594	9.95394	10.14961	9.578106	9.899642	9.309879	10.05154	9.812104	Cd34	NM_001111059
6756572	488	6.093575	6.007277	6.066262	6.004127	0.089448	0.4396	0.00315	0.977853	0.062135	0.587606	-0.05899	0.606342	0.086298	0.45528	0.027313	0.810137	6756572	6.110501	6.28908	5.881143	6.092154	5.913418	6.016258	6.193955	5.936893	6.067938	5.981345	6.091714	5.939321	Xkr4	NM_001011874
6756599	490	7.482723	7.438192	7.285462	7.342097	0.140627	0.666194	0.096095	0.767432	-0.05663	0.861376	0.15273	0.639788	0.044532	0.890748	0.197261	0.547442	6756599	7.195977	7.317254	7.934939	7.296192	7.801966	7.216417	7.783579	7.102201	6.970606	7.690203	6.938699	7.397388	Sox17	NM_011441
6756625	491	7.262424	7.45867	7.347263	7.483986	-0.22156	0.104485	-0.02532	0.839516	-0.13672	0.291275	0.111407	0.384158	-0.19625	0.143517	-0.08484	0.503122	6756625	7.135333	7.295164	7.356774	7.404395	7.481601	7.490013	7.384163	7.470798	7.186828	7.4489	7.276883	7.726174	Mrpl15	NM_025300
6756792	495	8.314533	8.372234	8.148805	8.306157	0.008376	0.906299	0.066077	0.365932	-0.15735	0.05189	0.223429	0.011867	-0.0577	0.426942	0.165728	0.042927	6756792	8.36945	8.375278	8.198872	8.314594	8.48408	8.318029	8.074396	8.245234	8.126785	8.265463	8.340395	8.312613	Vcpip1	NM_173443
6756815	497	8.391749	8.497959	8.48347	8.29395	0.097799	0.588225	0.204008	0.273228	0.18952	0.306248	0.014489	0.935465	-0.10621	0.557222	-0.09172	0.61121	6756815	8.267926	8.388072	8.519249	8.418974	8.76907	8.305832	8.72341	8.332896	8.394104	8.067384	8.253347	8.56112	Cops5	NM_013715
6756969	500	9.050192	9.086815	9.220857	9.494106	-0.44391	0.040612	-0.40729	0.055597	-0.27325	0.17163	-0.13404	0.482436	-0.03662	0.845537	-0.17066	0.375801	6756969	8.771932	9.227072	9.151573	9.157051	9.054694	9.048699	8.936071	9.578627	9.147872	9.591488	9.402592	9.688239	Ncoa2	NM_008678
6756985	501	10.96072	10.95807	11.0834	11.07244	-0.11172	0.301985	-0.11437	0.291461	0.01096	0.916478	-0.12533	0.25095	0.002643	0.979814	-0.12268	0.260268	6756985	10.9528	11.00939	10.91996	10.80027	10.94422	11.12973	11.13688	11.1698	10.94352	11.08961	11.19437	10.93334	Tram1	NM_028173
6756989	502	8.88293	8.927647	9.090758	9.132667	-0.24974	0.037054	-0.20502	0.07442	-0.04191	0.686094	-0.16311	0.141418	-0.04472	0.666527	-0.20783	0.071242	6756989	8.729746	9.041411	8.877632	8.915311	8.921887	8.945742	9.181813	9.112929	8.977532	8.999763	9.09255	9.305689	Lactb2	NM_145381
6757120	506	6.859091	6.792746	6.830131	6.710939	0.148152	0.436428	0.081807	0.663056	0.119192	0.528394	-0.03739	0.841406	0.066345	0.723263	0.02896	0.876755	6757120	6.805487	6.734455	7.037332	6.822159	6.803343	6.752737	7.063643	6.797547	6.629204	6.386671	6.665592	7.080555	Stau2	NM_025303
6757127	507	7.792627	7.820627	7.722245	7.731821	0.061446	0.671162	0.089446	0.539209	-0.00894	0.950481	0.098382	0.500545	-0.028	0.845881	0.070382	0.627387	6757127	7.812007	7.882327	7.683547	7.709191	7.965205	7.787486	7.795496	7.822304	7.448936	7.550865	7.751399	7.89128	Ube2w	NM_025773
6757129	508	7.755932	7.766152	7.68625	7.781737	-0.0258	0.705336	-0.01559	0.818825	-0.09549	0.185016	0.079902	0.259507	-0.01022	0.88049	0.069682	0.320787	6757129	7.700678	7.781606	7.785513	7.646354	7.864075	7.788027	7.69691	7.582013	7.779827	7.744529	7.824811	7.775871	Tceb1	NM_026456
6757157	510	7.873584	7.936006	7.766368	8.192884	-0.3193	0.233121	-0.25688	0.329742	-0.42652	0.123178	0.169638	0.512518	-0.06242	0.807267	0.107216	0.676361	6757157	7.495366	7.80491	8.320477	8.132382	7.968685	7.706952	8.004618	7.84013	7.454356	7.984823	8.108186	8.485643	Jph1	NM_020604
6757282	514	7.019315	6.912396	6.977995	6.108066	0.91125	0.085366	0.80433	0.121516	0.86993	0.097919	-0.0656	0.89116	0.106919	0.82369	0.04132	0.931289	6757282	6.92865	6.84181	7.287486	6.59788	7.465012	6.674296	7.784144	5.946975	7.202867	5.973689	6.510542	5.839966	Mcm3	NM_008563
6757301	515	9.413206	9.102609	9.332798	9.020861	0.392345	0.045633	0.081748	0.63548	0.311937	0.096913	-0.23019	0.202779	0.310597	0.098124	0.080408	0.640951	6757301	9.439141	9.546655	9.253822	9.303078	9.323792	8.680957	9.364755	9.325638	9.308	8.922461	9.02889	9.111231	Tram2	NM_133252
6757357	517	6.221994	6.345322	6.014607	6.134039	0.087955	0.708977	0.211283	0.379967	-0.11943	0.613652	0.330714	0.183903	-0.12333	0.60233	0.207386	0.388391	6757357	5.987596	5.943738	6.734647	6.236252	6.300961	6.498752	6.176695	5.903857	5.96327	6.432865	5.904884	6.064367	Rims1	NM_053270
6757370	518	5.842041	6.039606	6.153775	6.096849	-0.25481	0.014349	-0.05724	0.503972	0.056926	0.506264	-0.11417	0.200394	-0.19757	0.042192	-0.31173	0.005161	6757370	5.87459	5.845224	5.806309	6.168491	5.927478	6.02285	6.065275	6.241471	6.15458	6.164859	5.948714	6.176974	4933415F	NM_025746
6757387	519	6.153341	6.202608	6.043317	5.822116	0.331225	0.229949	0.380492	0.173823	0.221201	0.410738	0.159291	0.549393	-0.04927	0.851541	0.110024	0.677375	6757387	6.473135	6.051153	5.935734	5.994239	5.903234	6.710352	6.27995	6.031361	5.81864	5.687999	6.107154	5.671195	Ogfrl1	NM_001081079
6757423	520	9.502593	9.563141	9.545024	9.529691	-0.0271	0.702561	0.03345	0.638213	0.015333	0.828387	0.018117	0.797967	-0.06055	0.402233	-0.04243	0.552608	6757423	9.558113	9.531241	9.418424	9.534611	9.622438	9.532373	9.471539	9.62954	9.533992	9.405593	9.637589	9.545891	Smap1	NM_028534
6757433	522	7.691935	7.774729	7.704373	7.714544	-0.02261	0.902929	0.060185	0.746165	-0.01017	0.956226	0.070356	0.705481	-0.08279	0.65708	-0.01244	0.946489	6757433	7.868279	7.51144	7.696087	7.725104	7.999211	7.599872	7.880616	7.632142	7.600361	7.426148	8.043264	7.674221	Fam135a	BC070446
6757732	527	7.252754	7.490906	7.316075	6.930447	0.322307	0.159071	0.560459	0.027067	0.385628	0.100257	0.174831	0.424083	-0.23815	0.284382	-0.06332	0.768102	6757732	7.183836	7.231783	7.342643	7.274665	7.701301	7.496752	7.726062	6.902549	7.319613	6.710376	7.061808	7.019157	Prim2	NM_008922
6757744	528	6.692023	6.925478	6.732849	6.807254	-0.11523	0.382099	0.118224	0.370466	-0.0744	0.566905	0.192629	0.160678	-0.23345	0.097847	-0.04083	0.751574	6757744	6.484742	6.617642	6.973686	6.772796	7.044687	6.958951	6.734912	6.70428	6.759355	6.696879	6.86516	6.859722	Bag2	NM_145392
6757745	529	7.320019	7.40952	7.38778	7.325358	-0.00534	0.963907	0.084163	0.482716	0.062422	0.600006	0.02174	0.85394	-0.0895	0.456319	-0.06776	0.569808	6757745	7.237382	7.343423	7.379253	7.270439	7.602422	7.3557	7.56664	7.208014	7.388686	7.29524	7.442606	7.238227	Zfp451	NM_133817
6757796	531	6.525514	6.66903	6.643252	6.731688	-0.20617	0.142677	-0.06266	0.63455	-0.08844	0.505367	0.025778	0.844008	-0.14352	0.290579	-0.11774	0.380385	6757796	6.62541	6.410924	6.540207	6.732784	6.831683	6.442623	6.479845	6.754087	6.695823	6.678329	6.613645	6.90309	Ccdc115	NM_027159
6757830	533	9.588559	9.552441	9.363956	9.432646	0.155913	0.258902	0.119795	0.377748	-0.06869	0.606924	0.188485	0.179979	0.036118	0.785453	0.224603	0.118107	6757830	9.750995	9.65139	9.363291	9.581308	9.624623	9.451392	9.13413	9.561873	9.395865	9.469025	9.465356	9.363556	Fam168b	BC043098
6757896	534	9.463982	9.379148	9.537505	9.291418	0.172564	0.121086	0.08773	0.403648	0.246087	0.038515	-0.15836	0.150154	0.084834	0.418669	-0.07352	0.481067	6757896	9.537856	9.460687	9.393403	9.257829	9.421325	9.458289	9.690329	9.524159	9.398027	9.164701	9.451003	9.25855	Ugcgl1	NM_198899
6757898	535	7.997243	7.547203	8.116846	6.983002	1.014241	0.020412	0.564201	0.147416	1.133845	0.012181	-0.56964	0.144039	0.45004	0.23664	-0.1196	0.742615	6757898	8.494398	7.913095	7.584236	7.751016	7.918845	6.971748	8.446919	7.742063	8.161557	6.807662	7.425526	6.715817	Neurl3	NM_153408
6757905	536	7.95051	7.718023	7.957642	8.134747	-0.18424	0.238768	-0.41672	0.020532	-0.1771	0.25588	-0.23962	0.136387	0.232486	0.146862	-0.00713	0.961902	6757905	7.958636	7.927388	7.965505	7.476913	7.763829	7.913328	8.155295	7.799787	7.917845	7.937593	8.11441	8.352238	Lman2l	NM_001013374
6757906	537	8.797762	8.729484	8.773116	8.939634	-0.14187	0.19667	-0.21015	0.070431	-0.16652	0.136918	-0.04363	0.676347	0.068279	0.517005	0.024646	0.81287	6757906	8.839542	8.80741	8.746335	8.788418	8.760446	8.639587	8.627461	9.000974	8.690913	8.834399	8.927969	9.056533	4632411B	NM_172652
6757927	538	7.646571	7.718151	7.813912	7.223899	0.422672	0.531809	0.494252	0.466752	0.590014	0.388362	-0.09576	0.885973	-0.07158	0.914613	-0.16734	0.802399	6757927	6.404397	7.638837	8.896479	8.361575	7.628714	7.164164	8.501159	7.382547	7.558031	6.673912	7.430878	7.566906	Ankrd23	NM_153502
6757928	539	6.038716	6.079489	6.233262	5.996361	0.042355	0.737083	0.083127	0.514326	0.2369	0.087748	-0.15377	0.242456	-0.04077	0.746497	-0.19455	0.148985	6757928	6.055913	6.074008	5.986228	6.032234	5.920087	6.286145	6.057207	6.470487	6.172091	5.910221	6.065349	6.013514	Ankrd39	NM_026241
6757935	541	8.294675	8.308661	8.215131	8.160022	0.134653	0.339578	0.148639	0.294803	0.055109	0.688609	0.09353	0.500589	-0.01399	0.918592	0.079544	0.565169	6757935	8.33402	8.108758	8.441247	8.363994	8.435065	8.126924	8.367306	8.261546	8.016541	8.01057	8.195657	8.273838	Actr1b	NM_146107
6757945	542	9.613164	9.631353	9.587723	9.68811	-0.07495	0.357287	-0.05676	0.480613	-0.10039	0.227091	0.04363	0.585209	-0.01819	0.818565	0.025441	0.748732	6757945	9.663208	9.617788	9.558496	9.589133	9.673906	9.631021	9.449461	9.710526	9.603183	9.560285	9.716016	9.788029	Tmem131	NM_018872
6757971	543	8.816999	8.824486	8.667219	8.715777	0.101223	0.365439	0.108709	0.33297	-0.04856	0.657592	0.157267	0.174393	-0.00749	0.94517	0.14978	0.193475	6757971	8.909152	8.811571	8.730275	8.868993	8.794069	8.810396	8.573366	8.836635	8.591657	8.536539	8.698122	8.912669	6330578E	NM_198006
6757974	544	9.833751	9.83263	9.874027	9.869251	-0.0296	0.858366	-0.03072	0.853072	0.010676	0.948636	-0.0414	0.803107	0.001121	0.9946	-0.04028	0.808315	6757974	9.91845	9.761667	9.821136	9.820899	9.699844	9.977146	10.01887	9.824751	9.77846	9.505236	9.913068	10.17175	6330578E	NM_198006
6757975	545	8.050467	8.220782	8.108285	8.095902	-0.04544	0.577369	0.12488	0.149078	0.012383	0.878146	0.112497	0.188363	-0.17032	0.061134	-0.05782	0.480971	6757975	8.106285	8.037323	8.007793	8.30928	8.262004	8.091063	8.152	8.184187	7.988669	7.989552	8.110194	8.18796	Mgat4a	NM_173870
6757979	546	7.444072	7.716096	7.584496	7.618265	-0.17419	0.232612	0.097831	0.488933	-0.03377	0.808621	0.1316	0.357806	-0.27202	0.078453	-0.14042	0.328274	6757979	7.288739	7.424434	7.619043	7.735417	7.783881	7.62899	7.640242	7.673718	7.439528	7.545714	7.419322	7.889758	2010300C	BC072639
6757982	548	6.826945	6.920876	6.815553	6.652471	0.174475	0.290492	0.268406	0.119834	0.163082	0.320987	0.105323	0.513755	-0.09393	0.559207	0.011392	0.942902	6757982	6.855389	6.796173	6.829274	6.6643	7.113194	6.985135	7.091267	6.633422	6.72197	6.474003	6.668463	6.814946	Mitd1	NM_026913
6757988	549	8.066593	8.249528	8.020204	8.179082	-0.11249	0.458231	0.070446	0.63862	-0.15888	0.303051	0.229324	0.150789	-0.18294	0.240678	0.046388	0.756166	6757988	7.926561	8.058702	8.214515	8.201242	8.123088	8.424254	8.215906	7.9	7.944707	8.085785	8.015821	8.435641	Txndc9	NM_172054
6757994	550	6.886189	7.084658	6.96056	7.045961	-0.15977	0.095317	0.038696	0.659184	-0.0854	0.341776	0.124098	0.180139	-0.19847	0.046778	-0.07437	0.404465	6757994	6.926666	6.800474	6.931426	7.050048	7.126836	7.077089	7.089008	6.93332	6.859351	6.874567	7.137745	7.125572	Rev1	NM_019570
6758027	553	8.624172	8.714349	8.86963	9.344984	-0.72081	0.010026	-0.63063	0.01888	-0.47535	0.057935	-0.15528	0.490591	-0.09018	0.685853	-0.24546	0.286477	6758027	8.709185	8.71164	8.451692	8.947384	8.315702	8.87996	8.709696	9.186651	8.712543	9.37224	9.089267	9.573444	Tbc1d8	NM_018775
6758036	554	6.240154	6.213183	6.330039	6.124963	0.115191	0.206389	0.088219	0.323064	0.205075	0.04006	-0.11686	0.200546	0.026972	0.75573	-0.08988	0.314582	6758036	6.340496	6.161955	6.218012	6.285782	6.227438	6.126328	6.238877	6.231733	6.519506	6.117386	6.145522	6.111982	Creg2	NM_170597
6758261	557	8.020477	7.855898	7.782326	7.422856	0.597621	0.000194	0.433041	0.001561	0.359469	0.004587	0.073572	0.448526	0.164579	0.112504	0.238151	0.032643	6758261	7.917903	8.143333	8.000195	7.818662	7.995625	7.753406	7.753345	7.748374	7.845258	7.538856	7.344907	7.339806	Fhl2	NM_010212
6758325	558	7.864939	7.856006	7.741165	7.788987	0.075953	0.538223	0.06702	0.586033	-0.04782	0.696203	0.114842	0.359423	0.008933	0.941576	0.123775	0.325341	6758325	7.844676	7.918515	7.831627	7.641443	7.94255	7.984026	7.973248	7.695834	7.554412	7.78189	7.826196	7.758874	Uxs1	NM_026430
6758361	559	6.565226	6.684247	6.667763	6.59385	-0.02862	0.866303	0.090397	0.597971	0.073913	0.665405	0.016484	0.922716	-0.11902	0.490346	-0.10254	0.550776	6758361	6.587386	6.605665	6.502626	6.356212	6.929305	6.767224	6.835621	6.422056	6.745612	6.518081	6.775786	6.487682	Kdelc1	NM_023645
6758414	561	7.926698	7.818387	7.071859	6.639334	1.287364	0.004486	1.179053	0.007185	0.432526	0.225459	0.746527	0.053145	0.108311	0.750676	0.854839	0.031824	6758414	8.218467	8.122134	7.439493	7.721416	8.299625	7.434119	7.5289	6.721209	6.965469	6.322016	6.968027	6.627958	Col5a2	NM_007737
6758435	562	6.265548	6.890075	6.415253	6.312591	-0.04704	0.803575	0.577483	0.013469	0.102661	0.590088	0.474822	0.031854	-0.62453	0.009176	-0.1497	0.436916	6758435	6.167855	6.420588	6.208202	6.545753	7.317808	6.806663	6.45603	6.346393	6.443335	6.153769	6.316752	6.467253	Slc40a1	NM_016917

6758450	564	9.957639	10.49483	9.823026	10.19423	-0.23659	0.394251	0.300594	0.285757	-0.37121	0.195488	0.671801	0.033836	-0.53719	0.075188	0.134613	0.62232	6758450	9.840177	10.08632	9.94642	10.44501	10.76815	10.27132	10.0044	9.575307	9.88937	9.969824	9.807993	10.80488	Slc39a10	NM_172653
6758588	565	7.192968	7.503939	7.119742	7.522952	-0.32998	0.203731	-0.01901	0.938398	-0.40321	0.129269	0.384196	0.145753	-0.31097	0.228405	0.073226	0.766589	6758588	7.256038	7.162532	7.160334	7.470267	7.584572	7.456977	6.969096	7.179416	7.210715	7.221877	7.175582	8.171396	Obfc2a	NM_028696
6758595	566	7.451606	7.575618	7.410418	7.279078	0.172528	0.417028	0.296541	0.179535	0.13134	0.533017	0.165201	0.436285	-0.12401	0.555572	0.041188	0.843223	6758595	7.544629	7.416425	7.393764	7.401788	7.756318	7.568749	7.677938	7.092192	7.461123	6.889402	7.538847	7.408984	Myo1b	NM_010863
6758625	567	9.042853	9.049734	9.054459	8.801451	0.241402	0.175284	0.248283	0.164652	0.253008	0.157692	-0.00472	0.977491	-0.00688	0.967223	-0.01161	0.944752	6758625	9.003128	9.014245	9.111185	8.957491	9.128662	9.06305	8.968264	9.226799	8.968315	8.701408	9.193992	8.508953	Nab1	NM_008667
6758633	568	9.007972	8.798402	8.794954	8.738633	0.269339	0.098439	0.059769	0.689103	0.056321	0.706013	0.003448	0.981491	0.209571	0.183792	0.213018	0.177454	6758633	9.101746	8.892471	9.0297	8.868868	8.859913	8.666424	8.540981	8.998694	8.845187	8.641528	8.984093	8.590278	Mfsd6	NM_133829
6758646	569	6.926131	6.859427	6.796146	6.720975	0.205156	0.033096	0.138452	0.120993	0.075171	0.373813	0.063281	0.450697	0.066703	0.427524	0.129985	0.142022	6758646	6.998572	6.824018	6.955802	6.868793	6.938787	6.770702	6.774803	6.683695	6.92994	6.771554	6.769101	6.62227	Inpp1	NM_008384
6758663	572	9.1063	9.158887	8.954403	8.906202	0.200098	0.159995	0.252685	0.086193	0.048201	0.718759	0.204484	0.152111	-0.05259	0.694639	0.151898	0.273476	6758663	8.989192	9.16363	9.166079	9.088703	9.225817	9.16214	9.183877	8.726974	8.952357	8.709236	8.942642	9.066728	Asnsd1	NM_133728
6758691	573	7.783096	7.667901	7.29542	6.8752	0.907896	0.003148	0.792701	0.006631	0.42022	0.090102	0.372481	0.125959	0.115195	0.611616	0.487676	0.055718	6758691	8.028884	7.597134	7.72327	7.827088	7.713358	7.463256	7.552047	7.051158	7.283055	6.912052	7.227539	6.486009	Stk17b	NM_133810
6758694	574	7.33725	7.42736	6.96193	7.541615	-0.20436	0.152587	-0.11425	0.4026	-0.57968	0.002045	0.46543	0.006981	-0.09011	0.505547	0.37532	0.019797	6758694	7.345039	7.297897	7.368815	7.543227	7.500825	7.238029	7.258498	6.827075	6.800218	7.469188	7.538241	7.617417	Hecw2	NM_001001883
6758737	576	10.03651	10.20487	10.15226	10.0182	0.018304	0.879522	0.186664	0.149182	0.134054	0.28488	0.05261	0.6648	-0.16836	0.187996	-0.11575	0.351353	6758737	10.09741	10.0632	9.948912	10.04206	10.31455	10.25799	10.38343	10.01393	10.05941	9.880669	10.06299	10.11095	Sf3b1	NM_031179
6758862	579	5.952819	5.912247	5.93503	6.087797	-0.13498	0.395875	-0.17555	0.276927	-0.15277	0.339687	-0.02278	0.883394	0.040572	0.794262	0.017788	0.908807	6758862	5.820431	6.099723	5.938302	5.850408	5.993876	5.892457	6.050875	6.042767	5.711449	5.889782	5.977457	6.396152	Satb2	NM_139146
6758920	580	6.49038	6.619294	6.561332	6.330638	0.159742	0.104512	0.288656	0.010726	0.230694	0.029532	0.057962	0.525182	-0.12891	0.177802	-0.07095	0.439648	6758920	6.506403	6.589181	6.375555	6.627621	6.689974	6.540286	6.62303	6.38655	6.674415	6.275027	6.40877	6.308117	Kctd18	NM_030211
6758941	581	10.51972	10.69136	10.6288	10.62411	-0.1044	0.602738	0.06725	0.736102	0.004687	0.981192	0.062563	0.753757	-0.17165	0.399055	-0.10908	0.586859	6758941	10.45669	10.53427	10.56819	10.58438	10.4361	11.05361	10.835	10.45435	10.59705	10.54997	10.38822	10.93415	Clk1	NM_001042634
6758943	582	7.411752	7.431283	7.444269	7.41007	0.001682	0.988096	0.021213	0.850891	0.034199	0.762271	-0.01299	0.908316	-0.01953	0.862565	-0.03252	0.77357	6758943	7.313978	7.532338	7.388939	7.213555	7.613358	7.466935	7.548565	7.400009	7.384232	7.294132	7.477662	7.458416	Ppil3	NM_027351
6758949	583	6.848574	6.842442	6.890581	6.892982	-0.04441	0.562	-0.05054	0.510649	-0.0024	0.974711	-0.04814	0.530404	0.006131	0.935491	-0.04201	0.582902	6758949	6.869402	6.766579	6.90974	6.746178	6.970322	6.810827	6.90051	6.99453	6.776702	6.849353	6.929417	6.900175	Orc2l	NM_008765
6758958	584	6.981489	7.113929	7.205746	7.260708	-0.27922	0.023602	-0.14678	0.180819	-0.05496	0.598024	-0.09182	0.385926	-0.13244	0.222466	-0.22426	0.055439	6758958	6.79525	7.101869	7.047348	7.04573	7.157005	7.139052	7.134613	7.369545	7.11308	7.147879	7.311265	7.322979	Fam126b	NM_172513
6758984	586	8.921501	9.089344	8.732388	8.985364	-0.06386	0.713038	0.10398	0.552173	-0.25298	0.169559	0.356956	0.065769	-0.16784	0.345844	0.189113	0.291778	6758984	9.066101	8.863893	8.834509	9.367116	8.848728	9.052187	8.7363	8.88116	8.579703	8.771779	8.926589	9.257723	Trak2	NM_172406
6758995	589	7.532797	7.406663	7.591354	7.853825	-0.32103	0.060573	-0.44716	0.016044	-0.26247	0.112116	-0.18469	0.244578	0.126134	0.415995	-0.05856	0.700901	6758995	7.430699	7.665915	7.501777	7.397355	7.412527	7.410108	7.410045	7.914158	7.449858	7.714811	7.77471	8.071954	Als2	NM_028717
6759021	590	7.320987	7.357215	7.014359	7.098388	0.222599	0.472233	0.258827	0.405963	-0.08403	0.783048	0.342856	0.278747	-0.03623	0.90531	0.306628	0.329111	6759021	7.537405	7.436172	6.989383	7.450298	7.276206	7.345142	7.057232	7.570808	6.415037	7.249602	6.744933	7.300629	Sumo1	NM_009460
6759248	593	8.172549	8.388378	8.176337	8.462469	-0.28992	0.145357	-0.07409	0.690965	-0.28613	0.150013	0.212041	0.271937	-0.21583	0.264112	-0.00379	0.983702	6759248	8.098656	8.049562	8.36943	8.429038	8.306354	8.429742	8.381516	8.111001	8.036493	8.167038	8.363936	8.856432	Ndufs1	NM_145518
6759259	595	5.63316	5.690002	5.74145	5.299496	0.333664	0.272988	0.390507	0.205626	0.441954	0.157586	-0.05145	0.860492	-0.05684	0.846072	-0.10829	0.712391	6759259	6.036549	5.60166	5.226171	5.339744	6.071154	5.659109	5.832267	6.114965	5.277118	5.16231	5.397194	5.338983	Prelid1	NM_025596
6759297	597	8.849476	8.748577	8.752925	8.511029	0.338446	0.019648	0.237547	0.075547	0.241896	0.071288	-0.00435	0.971112	0.100899	0.411215	0.096551	0.430826	6759297	8.959709	8.845897	8.742821	8.766214	8.763772	8.715744	8.702689	8.796059	8.760027	8.342566	8.807837	8.382685	Klf7	NM_033563
6759326	598	6.29722	6.420916	6.357647	6.310078	-0.01286	0.885439	0.110838	0.235675	0.047569	0.597142	0.063269	0.485107	-0.1237	0.190324	-0.06043	0.504341	6759326	6.234406	6.44222	6.215035	6.285381	6.515235	6.462133	6.381166	6.319679	6.372097	6.181616	6.34251	6.406108	Fam119a	NM_025964
6759333	599	7.924296	7.956515	8.023473	8.1548	-0.2305	0.253312	-0.19828	0.320581	-0.13133	0.503001	-0.06696	0.729918	-0.03222	0.867669	-0.09918	0.610755	6759333	8.136665	7.860146	7.776078	8.079308	8.155152	7.635085	7.982728	8.33409	7.753602	8.227782	8.038303	8.198315	Fzd5	NM_022721
6759342	600	8.120624	7.934326	7.906829	7.752171	0.368453	0.00355	0.182155	0.07861	0.154658	0.125404	0.027497	0.76871	0.186298	0.073212	0.213795	0.045563	6759342	8.204989	8.066309	8.090573	8.013176	8.0388	7.751001	7.963104	7.781783	7.9756	7.798947	7.797683	7.797683	Plekhm3	NM_001039493
6759391	607	6.674825	7.070117	5.364265	5.299016	1.375809	0.06246	1.771101	0.023743	0.065249	0.920806	1.705852	0.027825	-0.39529	0.55152	1.31056	0.073272	6759391	7.198698	5.128852	7.696925	6.260949	7.382507	7.566895	5.467408	5.300562	5.324826	5.053412	5.283529	5.560108	D630023F	NM_175293
6759396	608	9.619355	9.857424	8.979861	8.265602	1.353754	0.011985	1.591822	0.005216	0.71426	0.12634	0.877562	0.069321	-0.23807	0.585149	0.639494	0.165106	6759396	9.78568	8.776226	10.29616	9.264861	10.17294	10.13447	9.262276	8.701792	8.975516	7.933366	8.599041	8.264398	Idh1	NM_001111320
6759459	609	8.813699	8.730858	8.962203	9.085785	-0.27209	0.157515	-0.35493	0.076356	-0.12358	0.498876	-0.23134	0.221474	0.082841	0.647628	-0.1485	0.419444	6759459	8.687833	9.141947	8.611317	8.798308	8.929463	8.464804	9.045291	8.898535	8.942782	8.970509	8.978014	9.308833	Acadl	NM_007381
6759466	610	7.523415	7.432142	7.548506	6.701061	0.822355	0.605355	0.731081	0.64537	0.847446	0.594591	-0.11636	0.941209	0.091274	0.953867	-0.02509	0.98731	6759466	4.786597	7.390189	10.39346	9.027866	7.752057	5.516502	8.574691	7.201948	6.86888	5.140893	6.885595	8.076694	Myl1	NM_021285
6759468	611	7.54206	7.655355	7.558871	7.792381	-0.25032	0.187923	-0.13703	0.453372	-0.23351	0.216135	0.096484	0.594133	-0.11329	0.532936	-0.01681	0.925355	6759468	7.394482	7.634153	7.597545	7.563969	7.742061	7.660034	7.727036	7.467314	7.482263	7.544711	7.617033	8.215398	Lancl1	NM_021295
6759549	612	7.299948	7.48436	7.224511	7.197888	0.102059	0.183618	0.286471	0.003507	0.026623	0.714065	0.259849	0.005992	-0.18441	0.030179	0.075437	0.313373	6759549	7.201698	7.379272	7.318873	7.507809	7.438159	7.507111	7.310135	7.065547	7.297851	7.204039	7.221816	7.16781	Ikzf2	NM_011770
6759621	615	11.224	10.98577	10.43606	10.70472	0.51928	0.253169	0.28105	0.523886	-0.26866	0.541891	0.549708	0.228671	0.23823	0.587632	0.787938	0.098654	6759621	11.31836	11.3744	10.97924	10.60722	11.72454	10.62555	11.13023	9.835605	10.34235	11.20219	10.48368	10.42829	Fn1	NM_010233
6759642	616	5.933613	6.026714	5.863942	6.017405	-0.08379	0.735395	0.009309	0.969939	-0.15346	0.539469	0.162772	0.51579	-0.0931	0.707531	0.069671	0.778459	6759642	6.076196	5.748419	5.976224	6.022838	6.16536	5.891943	5.938751	5.697996	5.955079	5.818587	6.613461	5.620167	Mreg	NM_001005423
6759664	619	11.04066	10.97096	11.14264	10.26246	0.778201	0.136527	0.708498	0.170305	0.880174	0.098131	-0.17168	0.724503	0.069703	0.885826	-0.10197	0.833744	6759664	11.0336	11.39648	10.69191	10.50847	11.65404	10.75037	11.61169	10.52989	11.28633	10.44723	10.8802	9.459957	Igfbp5	NM_010518
6759718	622	10.48734	10.45417	10.41121	10.74503	-0.25769	0.2743	-0.29086	0.221862	-0.33383	0.166905	0.042967	0.849734	0.03317	0.883661	0.076137	0.737719	6759718	10.62179	10.48768	10.35256	10.73228	10.345	10.28524	10.19539	10.89454	10.14369	10.7034	10.5786	10.9531	Tns1	NM_027884
6759729	624	8.97409	9.117079	8.854883	9.08175	-0.10766	0.348138	0.03533	0.752025	-0.22687	0.068925	0.262196	0.041385	-0.14299	0.222188	0.119207	0.301894	6759729	8.966749	8.911637	9.043883	9.052525	9.197332	9.101381	9.092765	8.777132	8.694752	8.999188	9.018494	9.227567	Aamp	NM_146110
6759732	625	9.670866	9.64021	9.643133	9.521068	0.149798	0.29276	0.119142	0.396583	0.122065	0.38564	-0.00292	0.983009	0.030656	0.823519	0.027733	0.840059	6759732	9.810611	9.538087	9.6639	9.771199	9.66788	9.481551	9.835871	9.712317	9.38121	9.459339	9.640757	9.463107	Tmbim1	NM_027154
6759742	626	6.90771	7.025889	6.891833	6.916754	-0.00904	0.936362	0.109136	0.34923	-0.02492	0.826099	0.134056	0.256758	-0.11818	0.313045	0.015877	0.888575	6759742	6.92048	6.899004	6.903646	6.834625	7.175002	7.068041	6.931056	7.046858	6.697586	7.029976	6.83553	6.884755	Usp37	NM_176972
6759748	627	7.61708	7.529788	7.582425	7.649309	-0.03223	0.836039	-0.11952	0.450696	-0.06688	0.669	-0.05264	0.735951	0.087292	0.578454	0.034655	0.823936	6759748	7.572907	7.688456	7.589876	7.506946	7.640988	7.44143	7.299915	7.943891	7.503469	7.673582	7.752467	7.521879	Zfp142	NM_029888
6759750	628	7.892313	7.962596	7.984475	7.973724	-0.08141	0.413785	-0.01113	0.909112	0.010751	0.912176	-0.02188	0.822622	-0.07028	0.478064	-0.09216	0.357735	6759750	7.847954	7.960646	7.868338	7.965015	8.124473	7.798301	7.987041	8.102871	7.863514	8.024961	8.031872	7.86434	Rnf25	NM_021313
6759758	629	6.506069	6.443799	6.393901	6.690789	-0.18472	0.139165	-0.24699	0.059377	-0.29689	0.029738	0.049899	0.669069	0.062269	0.59498	0.112168	0.347859	6759758	6.384401	6.427585	6.70622	6.593786	6.366771	6.370841	6.371325	6.360819	6.449558	6.503984	6.784444	6.783938	Prkag3	NM_153744
6759769	633	6.839194	6.763148	6.788457	6.809659	0.029535	0.857071	-0.04651	0.777044	-0.0212	0.897076	-0.02531	0.877315	0.076045	0.644815	0.050737	0.75751	6759769	6.798977	6.891234	6.82737	6.973496	6.650301	6.665648	6.803859	6.937735	6.623776	7.033499	6.466645	6.928832	Ihh	NM_010544
6759773	634	6.945059	6.393323	6.741205	6.332394	0.612665	0.028196	0.060929	0.797034	0.408811	0.11224	-0.34788	0.167437	0.551736	0.042648	0.203854	0.399608	6759773	6.947173	6.941925	6.94608	6.180296	6.582933	6.416741	6.993794	6.498334	6.731487	6.484509	6.698239	5.814434	Slc23a3	NM_194333
6759775	635	8.228008	8.3628	8.159776	8.211682	0.016326	0.788691	0.151118	0.033366	-0.05191	0.403925	0.203024	0.008739	-0.13479	0.051407	0.068232	0.280143	6759775	8.233114	8.252636	8.198275	8.432614	8.299514	8.356273	8.128926	8.24583	8.104572	8.282542	8.097941	8.254564	1810031K	NM_026977
6759778	636	6.586305	6.631603	6.528477	6.620029	-0.03372	0.734577	0.011574	0.90706	-0.09155	0.368417	0.103126	0.314291	-0.0453	0.649804	0.057828	0.563808	6759778	6.639085	6.438385	6.681445	6.634761	6.672058	6.58799	6.6755	6.487886	6.422045	6.523715	6.556522	6.77985	Abcb6	NM_023732
6759779	637	7.998366	7.868568	7.846199	8.007871	-0.0095	0.877514	-0.1393	0.047999	-0.16167	0.026796	0.022369	0.717771	0.129798	0.061526	0.152167	0.034306	6759779	8.053391	7.930539	8.011168	7.909386	7.954597	7.741721	7.781999	7.841801	7.914798	8.010599	7.984079	8.028934	Atg9a	NM_001003917
6759781	638	7.88542	7.832354	7.72344	7.607442	0.277978	0.117522	0.224911	0.193627	0.115997	0.485109	0.108914	0.511372	0.053067	0.746359	0.161981	0.336665	6759781	8.111409	7.803668	7.741184	7.728288	8.026194	7.742579	7.789404	7.738371	7.642544	7.553272	7.359873	7.909182	Glb1l	BC021773
6759782	639	8.852131	8.766702	8.763902	9.173199	-0.32107	0.282496	-0.4065	0.182736	-0.4093	0.180065	0.0028	0.992228	0.085429	0.766991	0.088229	0.75963	6759782	8.58143	8.486163	9.4888	8.870672	8.757699	8.671736	8.824448	8.981447	8.485811	9.227931	9.434336	8.857329	Tuba4a	NM_009447
6759789	640	11.84663	12.08129	12.18657	12.75101	-0.90438	0.011693	-0.66972	0.042702	-0.56444	0.077014	-0.10528	0.714984	-0.23465	0.423516	-0.33993	0.256573	6759789	11.57255	12.05061	11.91674	11.92594	12.02421	12.29371	11.68054	12.64887	12.23029	12.93407	12.98569	12.33327	Ptprn	NM_008985
6759800	642	8.649762	8.74353	8.813475	8.746659	-0.0969	0.339628	-0.00313	0.974648	0.066816	0.503656	-0.06994	0.484491	-0.09377	0.354568	-0.16371	0.124571	6759800	8.570741	8.669394	8.709152	8.753587	8.831593	8.64541	8.910493	8.809234	8.720697	8.878813	8.81732	8.543843	Dnpep	NM_001110831
6759816	643	7.908131	7.765163	7.829327	7.87464	0.033491	0.791408	-0.10948	0.397433	-0.04531	0.720972	-0.06416	0.614513	0.142968	0.276641	0.078804	0.537901	6759816	7.923576	7.884574	7.916243	7.586737	8.00011	7.708642	7.826857	8.019499	7.641624	7.899718	7.952993	7.771208	Chpf	NM_001001565
6759905	645	7.408158	7.400601	7.024378	6.653191	0.754967	0.016675	0.74741	0.01746	0.371188	0.176472	0.376223	0.171323	0.007557	0.97666	0.38378	0.163847	6759905	7.605246	7.144525	7.474703	7.237757	7.813201	7.150845	7.335588	6.617201	7.120346	6.395064	6.717091	6.847417	Epha4	NM_007936
6759949	647	7.765536	7.609525	7.745306	7.67278	0.092756	0.503683	-0.06326	0.645798	0.072525	0.59901	-0.13578	0.335392	0.156011	0.272791	0.02023	0.882412	6759949	7.80555	7.750739	7.740319	7.368975	7.814649	7.64495	7.972911	7.562522	7.700484	7.599857	7.785926	7.632558	Farsb	NM_011811
6760004	650	6.619715	6.602626	5.908315	6.559403	0.060312	0.787692	0.043223	0.846782	-0.65109	0.016904	0.694311	0.012498	0.017089	0.939042	0.7114	0.011105	6760004	6.537851	6.256463	7.064832	6.474392	6.89034	6.443147	6.08333	5.829753	5.811863	6.368425	6.638578	6.671206	Ap1s3	NM_183027
6760006	651	7.731086	7.363897	7.140932	7.620796	0.11029	0.264815	-0.2569	0.023451	-0.47986	0.000806	0.222965	0.041587	0.367189	0.003994	0.590154	0.000206	6760006	7.599864	7.660507	7.932887	7.34359	7.313267	7.434833	7.038535	7.259031	7.125229	7.677769	7.615177	7.569442	Wdfy1	NM_001111279
6760009	652	9.20056	8.963969	8.027493	7.717747	1.482812	0.006065	1.246221	0.0145	0.309746	0.462103	0.936476	0.047776	0.236591	0.571506	1.173067	0.019141	6760009	9.454086	9.305855	8.841738	8.694027	9.584266	8.613613	8.520498	7.712874	7.849107	7.054313	8.283981	7.815848	Serpine2	NM_009255
6760051	654	9.597005	9.623169	9.619349	9.605737	-0.00873	0.913806	0.017433	0.829105	0.013613	0.86607	0.00382	0.96222	-0.02616	0.746432	-0.02234	0.782245	6760051	9.612017	9.599144	9.579854	9.516819	9.694406	9.658283	9.71035	9.650968	9.49673	9.473016	9.625005	9.719189	Cul3	NM_016716
6760177	655	6.988673	6.94953	6.961442	6.921902	0.066771	0.627608	0.027628	0.839909	0.03954	0.772808	-0.01191	0.930517	0.039143	0.775013	0.027231	0.842171	6760177	6.766967	7.208159	6.990892	7.027333	6.993025	6.828231	7.139437	6.92835	6.816538	6.932119	7.051699	6.781888	Irs1	NM_010570
6760194	656	6.985385	7.486373	7.267825	7.400432	-0.41505	0.139957	0.085941	0.743142	-0.13261	0.614822	0.218548	0.413386	-0.50099	0.08334	-0.28244	0.297229	6760194	7.065816	6.956601	6.933737	7.804178	7.251618	7.403323	6.986844	7.669571	7.14706	7.398082	6.988026	7.815187	Col4a4	NM_007735
6760289	660	7.760895	7.327285	7.247791	7.013045	0.74785	0.022879	0.31424	0.27178	0.234746	0.403639	0.079494	0.772865	0.43361	0.142032	0.513104	0.090102	6760289	8.138196	7.712168	7.43232	7.411788	7.635179	6.934888	7.172209	7.461909	7.109255	6.652266	7.38834	6.998528	5033414K	BC080290
6760296	662	9.409031	9.554676	9.364016	9.294491	0.11454	0.062879	0.260185	0.001187	0.069525	0.226382	0.19066	0.007044	-0.14565	0.025237	0.045015	0.420803	6760296	9.470264	9.375669	9.38116	9.482747	9.622802	9.55848	9.417301	9.367265	9.307482	9.204445	9.332623	9.346405	Trip12	NM_133975

6760314	664	6.449148	6.513799	6.247054	6.088422	0.360727	0.063522	0.425377	0.034801	0.158633	0.371551	0.266744	0.150109	-0.06465	0.709711	0.202094	0.262289	6760314	6.684217	6.36029	6.302938	6.700908	6.521869	6.318619	6.57117	5.995203	6.17479	6.036214	6.141985	6.087066	A630001G	BC052931
6760347	666	6.092924	6.315861	6.089168	5.457296	0.635628	0.132982	0.858565	0.053807	0.631872	0.134979	0.226693	0.567359	-0.22294	0.573664	0.003756	0.992356	6760347	6.189264	6.06069	6.028819	6.209204	6.496447	6.241933	6.555785	5.587335	6.124384	5.575057	6.165848	4.630984	Htr2b	NM_008311
6760364	667	11.40193	11.481	11.27287	11.49509	-0.09315	0.298678	-0.01409	0.870702	-0.22222	0.0292	0.20813	0.037924	-0.07907	0.373122	0.129063	0.162164	6760364	11.47628	11.37982	11.3497	11.49361	11.44355	11.50584	11.21237	11.19006	11.41618	11.39427	11.42914	11.66185	Ncl	NM_010880
6760368	668	6.15721	5.893054	6.024026	5.938762	0.218448	0.447205	-0.04571	0.871332	0.085264	0.763035	-0.13097	0.644611	0.264155	0.362088	0.133184	0.639112	6760368	5.517315	6.381364	6.57295	6.130187	5.618886	5.93009	5.870399	6.089839	6.11184	5.724372	5.930989	6.160924	Nmur1	NM_010341
6760385	669	8.268178	8.209044	8.29453	8.047072	0.221107	0.160284	0.161972	0.289721	0.247458	0.121484	-0.08549	0.566136	0.059135	0.6898	-0.02635	0.858248	6760385	8.166029	8.275318	8.363188	8.130378	8.415736	8.081017	8.548152	8.241896	8.093541	8.0627	8.200878	7.877637	Pde6d	NM_008801
6760387	670	7.771254	7.436952	7.376943	7.481669	0.289585	0.025931	-0.04472	0.684674	-0.10473	0.352773	0.060009	0.58738	0.334302	0.013617	0.394312	0.005919	6760387	7.658124	7.802102	7.853537	7.380128	7.290494	7.640234	7.540926	7.255029	7.334873	7.50655	7.515378	7.42308	Nppc	NM_010933
6760446	673	7.001361	7.185255	6.968994	7.336486	-0.33512	0.087845	-0.15123	0.405988	-0.36749	0.065649	0.21626	0.245144	-0.18389	0.317296	0.032367	0.855766	6760446	6.891467	7.117187	6.99543	7.188047	7.076567	7.29115	6.836048	7.213567	6.857368	6.983605	7.387564	7.638288	Ngef	NM_001111314
6760447	674	5.655517	5.827515	5.418871	5.932735	-0.27722	0.363531	-0.10522	0.724078	-0.51386	0.111936	0.408644	0.193319	-0.172	0.566524	0.236645	0.434647	6760447	5.937729	5.61506	5.413761	5.804088	5.451918	6.226539	5.134081	5.990663	5.13187	6.029497	5.728727	6.039981	A530079E	AK080193
6760490	676	7.417933	7.465479	7.670325	7.220035	0.197898	0.658441	0.245444	0.584786	0.45029	0.326823	-0.20485	0.647399	-0.04755	0.914905	-0.25239	0.574414	6760490	6.881044	7.798057	7.574698	7.077682	8.365515	6.953239	7.66802	7.353519	7.989436	7.347071	7.559025	6.754008	6430706D	NM_198652
6760518	678	7.747291	7.608597	7.437162	7.140197	0.607094	0.014777	0.4684	0.043996	0.296965	0.168496	0.171434	0.407593	0.138694	0.499593	0.310129	0.152518	6760518	7.695847	7.86988	7.676146	7.443019	7.820752	7.562019	7.751122	7.071495	7.48887	7.11162	7.407656	6.901315	Arl4c	NM_177305
6760614	679	6.193659	6.1488	6.297544	6.249199	-0.05554	0.53925	-0.1004	0.279763	0.048345	0.59196	-0.14874	0.124209	0.044859	0.618487	-0.10388	0.264631	6760614	6.182388	6.142854	6.255735	6.132145	6.128933	6.185323	6.402481	6.38299	6.10716	6.359775	6.128591	6.259231	Gbx2	NM_010262
6760642	681	7.96847	7.895575	7.306792	7.330238	0.638232	0.085033	0.565337	0.119983	-0.02345	0.944234	0.588783	0.107477	0.072895	0.828071	0.661678	0.076038	6760642	8.356051	7.894818	7.654541	7.595911	8.466361	7.624453	7.535703	7.002172	7.382501	6.915229	7.776189	7.299295	Col6a3	AF064749
6760643	682	5.956386	6.011208	6.226615	6.265839	-0.30945	0.327817	-0.25463	0.416112	-0.03922	0.898172	-0.21541	0.488871	-0.05482	0.858119	-0.27023	0.389398	6760643	5.948442	5.97366	5.947056	6.23701	5.862045	5.93457	6.090746	6.547559	6.04154	6.055142	5.756276	6.9861	Rab17	NM_008998
6760674	683	7.764589	7.832646	7.682051	7.744761	0.019828	0.671202	0.087885	0.08663	-0.06271	0.201006	0.150595	0.01014	-0.06806	0.168946	0.082538	0.104014	6760674	7.720208	7.781199	7.792359	7.776868	7.871427	7.849643	7.7625	7.695755	7.587897	7.720828	7.755579	7.757876	Ilkap	NM_023343
6760678	684	8.047348	8.276111	8.02518	8.507853	-0.4605	0.115747	-0.23174	0.400608	-0.48267	0.101652	0.250931	0.364605	-0.22876	0.406424	0.022168	0.934415	6760678	7.964528	7.992449	8.185068	8.325667	8.320314	8.182351	8.10364	8.150791	7.821109	8.143685	8.183331	9.196544	Hes6	NM_019479
6760680	685	7.987597	8.348414	7.572774	9.35216	-1.36456	0.00482	-1.00375	0.021874	-1.77939	0.001013	0.77564	0.059626	-0.36082	0.337478	0.414823	0.274561	6760680	7.498141	7.855711	8.60894	8.622249	8.171489	8.251504	7.114487	8.209425	7.39441	9.394241	9.116179	9.546061	Per2	NM_011066
6760711	686	9.053297	9.284401	9.152223	9.585145	-0.53185	0.001817	-0.30074	0.032298	-0.43292	0.005846	0.132178	0.288568	-0.2311	0.0821	-0.09893	0.419653	6760711	8.874069	9.187231	9.098592	9.339453	9.186429	9.327322	8.984913	9.37816	9.093596	9.595179	9.499352	9.660905	Hdac4	NM_207225
6760714	687	9.839246	9.995648	9.910252	10.09982	-0.26058	0.092855	-0.10417	0.467541	-0.18957	0.202602	0.085395	0.549255	-0.1564	0.285287	-0.07101	0.617241	6760714	9.75613	9.720358	10.04125	10.00475	9.954163	10.02803	10.05656	9.906818	9.767379	9.914047	10.01163	10.37379	Ndufa10	NM_024197
6760754	692	8.53643	9.092934	8.674992	9.540957	-1.00453	0.012892	-0.44802	0.19318	-0.86597	0.025212	0.417942	0.22166	-0.5565	0.115612	-0.13856	0.672005	6760754	8.684278	8.672865	8.252146	9.601511	8.645103	9.032188	8.251781	9.283341	8.489853	9.652326	9.425757	9.544788	Kif1a	NM_008440
6760762	693	7.090393	7.265714	6.820774	6.93894	0.151453	0.695495	0.326774	0.406743	-0.11817	0.759618	0.444939	0.267309	-0.17532	0.651035	0.269618	0.490571	6760762	6.620014	6.982793	7.668371	7.479474	7.428245	6.889422	6.854887	6.426958	7.180478	6.408113	7.50622	6.902486	2310007B	BC089525
6760771	694	6.54792	6.745778	6.399166	6.472287	0.075633	0.263193	0.273491	0.002439	-0.07312	0.278152	0.346612	0.000563	-0.19786	0.013633	0.148754	0.04546	6760771	6.57187	6.521694	6.550196	6.645984	6.855512	6.735838	6.313275	6.503362	6.380862	6.419336	6.523802	6.473723	Mterfd2	NM_178051
6760777	696	10.47569	10.52899	10.66429	10.5246	-0.04891	0.598221	0.004393	0.961903	0.13969	0.155757	-0.1353	0.167571	-0.0533	0.566425	-0.1886	0.067283	6760777	10.5361	10.41755	10.47342	10.42899	10.61725	10.54074	10.76475	10.77431	10.45381	10.48466	10.57206	10.51708	Hdlbp	NM_133808
6760789	697	9.600212	9.68008	9.608793	9.496347	0.103865	0.286182	0.183733	0.077877	0.112445	0.251107	0.071288	0.455439	-0.07987	0.405163	-0.00858	0.92711	6760789	9.546948	9.573268	9.680421	9.655437	9.722497	9.662307	9.796546	9.591028	9.438804	9.391744	9.601261	9.496037	Stk25	NM_021537
6760794	698	7.292973	7.505036	7.324644	7.448605	-0.15563	0.109489	0.05643	0.532195	-0.12396	0.189477	0.180392	0.070367	-0.21206	0.039739	-0.03167	0.723588	6760794	7.229278	7.306129	7.343512	7.517114	7.562999	7.434994	7.367762	7.391869	7.2143	7.304813	7.408907	7.632096	Thap4	NM_025920
6760802	699	7.719692	7.819247	7.677202	7.681922	0.03777	0.737554	0.137325	0.242638	-0.00472	0.966476	0.142045	0.228193	-0.09956	0.387137	0.04249	0.706468	6760802	7.703975	7.678887	7.776213	7.636167	8.046271	7.775303	7.777932	7.721915	7.531759	7.7867	7.616892	7.642174	Dtymk	NM_001105667
6760804	700	6.033453	5.856666	5.907563	5.95781	0.075643	0.686506	-0.10114	0.590976	-0.05025	0.78801	-0.0509	0.785342	0.176787	0.356548	0.12589	0.505732	6760804	5.911329	5.914841	6.274189	5.916593	5.969785	5.683619	5.962891	5.830179	5.92962	6.217377	6.100006	5.556046	Pdcd1	NM_008798
6760847	701	7.298852	7.210964	7.172127	6.810186	0.488666	0.170862	0.400779	0.25225	0.361941	0.297485	0.038838	0.907769	0.087888	0.793557	0.126725	0.706598	6760847	7.371294	7.402022	7.12324	6.878201	7.377134	7.377558	7.440483	6.76566	7.310237	6.103863	7.319026	7.007668	St8sia4	NM_009183
6760911	704	6.775234	6.925633	6.726462	6.4723	0.302934	0.038538	0.453333	0.006038	0.254162	0.0717	0.199171	0.142636	-0.1504	0.254449	0.048772	0.700949	6760911	6.717421	6.813382	6.794899	6.751992	7.169605	6.855303	6.884027	6.603283	6.692076	6.410148	6.632419	6.374334	D1Ertd62	BC023951
6760915	705	8.101846	8.081695	8.160919	7.360667	0.741179	0.013399	0.721028	0.015257	0.800252	0.009201	-0.07922	0.744244	0.020151	0.933652	-0.05907	0.807513	6760915	8.028443	7.969654	8.30744	7.884923	8.341831	8.018331	8.408874	7.75741	8.316472	6.987336	7.663834	7.430831	Hisppd1	NM_173760
6760917	706	11.22794	11.19543	11.28346	11.17592	0.052023	0.705446	0.01951	0.886823	0.10754	0.441431	-0.08803	0.525998	0.032513	0.812731	-0.05552	0.686875	6760917	11.15384	11.34071	11.18927	11.01547	11.2126	11.35821	11.30713	11.25887	11.28437	11.22622	11.40349	10.89804	Pam	NM_013626
6761155	708	7.855538	7.849151	7.776485	7.658605	0.196933	0.308819	0.190546	0.323765	0.117881	0.53361	0.072666	0.69893	0.006387	0.972748	0.079053	0.674205	6761155	7.887148	7.84952	7.829946	7.672563	8.137817	7.737073	7.938779	7.395305	7.995372	7.517639	7.818436	7.639739	Pign	NM_013784
6761256	709	9.873905	9.67549	9.986116	9.857316	0.016589	0.941098	-0.18183	0.427592	0.1288	0.570222	-0.31063	0.191246	0.198415	0.388468	-0.11221	0.619996	6761256	9.723849	10.06586	9.832006	9.813132	9.465627	9.747711	9.565948	10.34764	10.04476	9.692292	10.14869	9.730966	Bcl2	NM_009741
6761268	710	9.05578	9.315847	9.20004	9.31162	-0.25584	0.010647	0.004227	0.957687	-0.11158	0.186463	0.115807	0.17207	-0.26007	0.009815	-0.14426	0.098676	6761268	9.015118	9.118973	9.033248	9.25122	9.282044	9.414277	9.239948	9.275812	9.084361	9.214904	9.27154	9.448416	Kdsr	NM_027534
6761272	711	10.32901	10.33155	10.34787	10.10713	0.221884	0.022608	0.224424	0.021513	0.240737	0.015676	-0.01631	0.841113	-0.00254	0.975067	-0.01885	0.816863	6761272	10.31919	10.3366	10.33125	10.29171	10.38755	10.3154	10.49162	10.27201	10.27997	9.955698	10.13872	10.22697	Vps4b	NM_009190
6761615	716	8.533269	8.845153	8.90735	8.580947	-0.04768	0.761075	0.264206	0.11938	0.326403	0.063367	-0.0622	0.692232	-0.31188	0.073554	-0.37408	0.038784	6761615	8.531543	8.678379	8.389885	8.677659	9.054845	8.802955	9.141728	8.813111	8.767211	8.358665	8.65655	8.727625	Tsn	NM_011650
6761669	717	9.369661	9.406437	9.406091	9.065956	0.303705	0.038387	0.34048	0.024105	0.340135	0.02421	0.000346	0.997821	-0.03678	0.772012	-0.03643	0.774085	6761669	9.404117	9.359882	9.344984	9.35338	9.575823	9.290107	9.546104	9.297871	9.374298	8.840672	9.290952	9.066245	Ralb	NM_022327
6761675	718	8.289451	8.515377	8.245957	8.110576	0.178875	0.01615	0.4048	0.000128	0.135381	0.050619	0.26942	0.001818	-0.22593	0.004981	0.043494	0.481414	6761675	8.214125	8.332128	8.3221	8.412082	8.559919	8.574129	8.212326	8.29258	8.232965	8.099727	8.034372	8.19763	Epb4.1l5	NM_145506
6761680	719	6.884373	6.886164	6.832566	6.878298	0.006076	0.944244	0.007867	0.927856	-0.04573	0.601814	0.053599	0.542164	-0.00179	0.98355	0.051808	0.555431	6761680	7.020519	6.821976	6.810625	6.899392	6.863248	6.895853	6.955792	6.78515	6.756755	6.787985	6.821014	7.025894	Ptpn4	NM_019933
6761681	720	6.131831	6.252962	6.17256	6.132908	-0.00108	0.993744	0.120054	0.39355	0.039652	0.773429	0.080402	0.562648	-0.12113	0.389518	-0.04073	0.767483	6761681	6.263863	5.967835	6.163794	6.320561	6.298165	6.140159	6.143191	6.322238	6.05225	5.960372	6.038014	6.400337	Tmem177	NM_175106
6761687	721	6.782611	6.808005	6.56024	6.680687	0.101924	0.703043	0.127318	0.634836	-0.12045	0.652963	0.247765	0.364877	-0.02539	0.923995	0.222372	0.413695	6761687	6.500888	6.94912	6.897826	7.165508	6.941737	6.316769	6.663648	6.431141	6.58593	6.566983	6.388605	7.086473	Tmem37	NM_019432
6761691	722	10.25605	9.920521	9.779485	9.842371	0.413679	0.069202	0.07815	0.702276	-0.06289	0.757999	0.141036	0.494875	0.335529	0.127306	0.476565	0.042086	6761691	10.50509	10.06451	10.19855	9.751873	10.12082	9.888871	10.19129	9.650572	9.496594	9.722366	9.841772	9.962976	Dbi	NM_001037999
6761692	723	7.639435	7.162261	6.95073	6.835641	0.803794	0.002508	0.32662	0.116463	0.115089	0.552424	0.211531	0.28735	0.477174	0.033069	0.688705	0.005948	6761692	7.720766	7.488129	7.709409	7.118917	7.526524	6.841341	7.135543	6.813597	6.903049	6.754585	7.071448	6.68089	Steap3	NM_001085409
6761701	724	6.040717	5.923774	5.988967	6.103984	-0.06327	0.429702	-0.18021	0.045316	-0.11502	0.168984	-0.06519	0.416369	0.116943	0.162772	0.05175	0.515515	6761701	6.095438	5.929153	6.09756	5.901908	5.88275	5.986663	5.997842	6.107498	5.86156	6.132861	6.008801	6.170291	Marco	NM_010766
6761733	725	7.85384	7.913433	7.78931	8.011141	-0.1573	0.241288	-0.09771	0.454462	-0.22183	0.112141	0.124123	0.347224	-0.05959	0.644452	0.06453	0.617697	6761733	7.716141	7.843622	8.001756	7.892397	8.020425	7.827476	8.045168	7.733887	7.588874	7.913831	8.093372	8.026219	Insig2	NM_133748
6761914	728	6.279265	6.118624	6.03289	6.163831	0.115434	0.513289	-0.04521	0.795571	-0.13094	0.460131	0.085733	0.625149	0.160641	0.369022	0.246375	0.18244	6761914	6.577417	6.037488	6.22289	6.113812	6.227957	6.014102	6.173424	5.918435	6.006812	6.048726	5.981668	6.461098	Lypd1	NM_145100
6761915	729	6.857819	6.932887	6.862285	7.250503	-0.39268	0.041783	-0.31762	0.085896	-0.38822	0.043618	0.070602	0.67486	-0.07507	0.655825	-0.00447	0.978711	6761915	6.738267	6.931693	6.903498	7.034931	6.886126	6.877604	6.884876	6.959707	6.742271	7.158259	6.949468	7.643782	E030049G	NM_172484
6761916	730	5.8469	5.808419	5.68019	6.2282	-0.3813	0.028068	-0.41978	0.018514	-0.54801	0.004898	0.12823	0.394318	0.038481	0.793893	0.166711	0.275557	6761916	5.794094	5.904282	5.842325	6.100222	5.634734	5.690302	5.743844	5.662508	5.634217	6.173424	6.035183	6.475994	E030049G	NM_001081756
6761964	731	6.293081	6.284997	6.295948	6.522242	-0.22916	0.015002	-0.23725	0.012729	-0.22629	0.015907	-0.01095	0.886444	0.008084	0.916022	-0.00287	0.970155	6761964	6.314842	6.214199	6.350201	6.261169	6.247891	6.345931	6.180743	6.265622	6.441479	6.545854	6.425614	6.595258	Tmem163	NM_028135
6762017	734	7.56103	7.91877	7.872329	6.712291	0.848739	0.032892	1.20648	0.006403	1.160039	0.007859	0.046441	0.891451	-0.35774	0.309456	-0.3113	0.372654	6762017	7.166471	7.68864	7.827978	7.612804	8.246317	7.89719	8.309558	7.480162	7.827268	6.204175	7.216593	6.716104	Mcm6	NM_008567
6762019	735	8.418885	8.527099	8.627387	8.385567	0.033318	0.829941	0.141531	0.373439	0.24182	0.145921	-0.10029	0.522949	-0.10821	0.491577	-0.2085	0.202351	6762019	8.276412	8.603197	8.377046	8.375946	8.670775	8.534575	8.812514	8.498812	8.570835	8.110823	8.479918	8.565961	Dars	NM_177445
6762024	736	7.613743	7.600201	7.925578	7.637243	-0.0235	0.905332	-0.03704	0.851399	0.288335	0.170456	-0.32538	0.127627	0.013542	0.945343	-0.31183	0.141987	6762024	7.635397	7.585515	7.620318	7.481802	7.568738	7.750064	7.633277	8.267047	7.87641	7.398118	7.992168	7.521443	Cxcr4	NM_009911
6762094	738	8.546872	8.992381	8.610218	8.333953	0.212919	0.448105	0.658427	0.038915	0.276265	0.330961	0.382163	0.190125	-0.44551	0.133675	-0.06335	0.818382	6762094	8.444362	8.126876	9.069378	8.991655	9.141897	8.84359	9.054239	8.299943	8.476472	8.205326	8.313083	8.483451	Cd55	NM_010016
6762104	740	6.17631	6.097531	6.132705	4.964626	1.211685	0.092487	1.132906	0.111903	1.168079	0.102799	-0.03517	0.957137	0.078779	0.90422	0.043605	0.946878	6762104	5.019189	5.943051	7.566691	5.56118	6.795494	5.93592	6.60852	5.57164	6.217955	4.675362	4.994291	5.224224	C4bp	NM_007576
6762106	741	8.21008	8.448212	8.063805	8.497522	-0.28744	0.069661	-0.04931	0.728816	-0.43372	0.013425	0.384407	0.023218	-0.23813	0.121109	0.146275	0.31786	6762106	7.998863	8.165792	8.465584	8.598387	8.268009	8.478241	8.053941	8.000134	8.13734	8.536291	8.325375	8.630901	Pfkfb2	NM_008825
6762123	742	6.217803	6.261505	6.153394	6.051407	0.166396	0.581651	0.210098	0.48915	0.101987	0.734002	0.108111	0.718814	-0.0437	0.883872	0.064409	0.829695	6762123	5.816978	6.343063	6.493368	5.68895	6.608944	6.486622	6.274505	5.804767	6.38091	5.989632	6.259398	5.905191	Il24	NM_053095
6762132	745	8.936475	8.992152	8.921204	8.789479	0.146996	0.199753	0.202673	0.090129	0.131725	0.24577	0.070947	0.518956	-0.05568	0.610909	0.015271	0.888147	6762132	8.979774	8.871215	8.958436	9.004392	9.111115	8.860948	9.126269	8.902489	8.734855	8.68164	8.839508	8.847289	Mapkapk2	NM_008551
6762141	746	7.127218	6.947047	7.040101	6.489543	0.637675	0.015891	0.457504	0.06028	0.550558	0.030139	-0.09305	0.668352	0.180171	0.414328	0.087117	0.688146	6762141	7.350392	7.034489	6.996773	6.984943	7.278275	6.577924	7.240031	6.7568	7.123473	6.409293	6.711152	6.348184	Dyrk3	NM_145508
6762147	747	6.45515	6.357942	6.38935	6.117262	0.337888	0.073136	0.24068	0.180091	0.272089	0.135409	-0.03141	0.852775	0.097208	0.569424	0.0658	0.698533	6762147	6.501987	6.486734	6.376729	6.326265	6.582616	6.164944	6.582574	6.200525	6.384952	6.000481	6.43128	5.920024	Rassf5	NM_018750
6762151	748	7.036699	6.986011	6.978252	6.281595	0.755103	0.010653	0.704416	0.014878	0.696656	0.015667	0.007759	0.973677	0.050688	0.829588	0.058447	0.804094	6762151	7.165121	6.933447	7.011528	6.906405	7.217466	6.834162	6.917004	6.699805	7.317946	6.349144	6.640982	5.85466	Ikbke	NM_019777
6762195	749	6.489388	6.678286	6.383294	6.2697	0.219688	0.136089	0.408586	0.015072	0.113594	0.416462	0.294992	0.056728	-0.1889	0.192022	0.106094	0.44667	6762195	6.593176	6.372305	6.502684	6.78213	6.741767	6.510962	6.581907	6.421181	6.146795	6.444352	6.203267	6.161481	Mfsd4	NM_172510
6762197	750	9.917123	10.08187	10.01959	10.15328	-0.23616	0.159679	-0.07141	0.65173	-0.13369	0.405748	0.062275	0.693408	-0.16474	0.311013	-0.10247	0.520121	6762197	9.970616	9.890254	9.890499	10.16891	10.22358	9.853111	9.701905	10.31941	10.03746	10.12286	10.1396	10.19738	Pctk3	NM_008795

6762217	751	6.375011	6.529614	6.406547	6.461753	-0.08674	0.510964	0.067861	0.60511	-0.05521	0.673105	0.123068	0.357663	-0.1546	0.255038	-0.03154	0.808819	6762217	6.326232	6.516447	6.282353	6.830348	6.305206	6.453289	6.340342	6.479244	6.400054	6.447708	6.508818	6.428734	Tmcc2	NM_178874
6762236	753	6.326755	6.33288	6.233532	6.119785	0.20697	0.038191	0.213095	0.034068	0.113747	0.21024	0.099348	0.26824	-0.00613	0.943326	0.093223	0.296645	6762236	6.509396	6.285961	6.184907	6.324265	6.378494	6.29588	6.329653	6.162525	6.208417	6.187385	6.045764	6.126205	Nfasc	NM_182716
6762259	754	8.377654	8.437848	8.2529	8.372077	0.005578	0.945524	0.065771	0.429885	-0.11918	0.170386	0.184948	0.047582	-0.06019	0.468574	0.124754	0.153468	6762259	8.483422	8.358111	8.29143	8.315433	8.51108	8.487031	8.156194	8.347736	8.254771	8.273226	8.434333	8.408671	Mdm4	NM_008575
6762304	755	7.404937	7.47805	7.361646	7.589129	-0.18419	0.180332	-0.11108	0.401873	-0.22748	0.107426	0.116404	0.380737	-0.07307	0.576183	0.04329	0.739002	6762304	7.446526	7.426416	7.341868	7.697047	7.429777	7.307326	7.379202	7.51511	7.190627	7.550562	7.453467	7.763359	Sox13	NM_011439
6762312	756	10.02546	10.10492	9.919431	9.86711	0.158353	0.089995	0.237814	0.02003	0.052322	0.541895	0.185492	0.053853	-0.07946	0.361645	0.106032	0.232763	6762312	9.99727	9.984989	10.09413	9.96257	10.23915	10.11305	9.93548	9.968115	9.854699	9.820749	10.00301	9.77757	Zc3h11a	NM_144530
6762321	758	9.035046	9.092878	8.899543	9.338243	-0.3032	0.239681	-0.24536	0.334032	-0.4387	0.103364	0.193335	0.441366	-0.05783	0.814647	0.135502	0.585811	6762321	8.992929	9.271665	8.840543	9.203816	9.025703	9.049115	9.072244	8.579816	9.04657	9.36213	9.781707	8.870891	Atp2b4	NM_213616
6762328	760	11.2245	10.28914	10.39642	10.28349	0.94101	0.067841	0.005651	0.9902	0.112923	0.806469	-0.10727	0.81594	0.935359	0.069192	0.828087	0.100382	6762328	11.39555	11.41222	10.86574	10.11874	10.97047	9.778222	11.05004	9.602039	10.53717	10.61025	10.43661	9.80362	Prelp	NM_054077
6762345	761	8.885396	8.890515	8.736443	9.410755	-0.52536	0.002815	-0.52024	0.002978	-0.67431	0.000609	0.154071	0.248291	-0.00512	0.968015	0.148953	0.263061	6762345	8.94411	8.70346	9.008618	8.894744	9.002754	8.774046	8.784572	8.734975	8.689783	9.50756	9.153488	9.571218	Btg2	NM_007570
6762353	762	9.134338	9.043183	9.095563	9.093886	0.040452	0.877956	-0.0507	0.847435	0.001677	0.994918	-0.05238	0.842467	0.091155	0.73013	0.038775	0.88297	6762353	9.257881	8.78012	9.365012	9.150107	9.274948	8.704493	9.226509	9.046423	9.013756	9.037854	8.688079	9.555725	Adora1	NM_001008533
6762355	763	8.409604	8.452807	8.371311	8.184197	0.225407	0.071588	0.26861	0.038495	0.187114	0.123166	0.081496	0.474456	-0.0432	0.701148	0.038293	0.733472	6762355	8.419869	8.331653	8.477291	8.400513	8.646205	8.311704	8.532545	8.259695	8.321693	8.089255	8.321828	8.141508	Tmem183a	NM_020588
6762397	766	6.155562	5.987758	6.383082	5.882181	0.273381	0.099765	0.105577	0.492764	0.500901	0.009224	-0.39532	0.027445	0.167804	0.286343	-0.22752	0.160004	6762397	6.191487	6.147333	6.127865	6.057167	6.042445	5.863661	6.696834	6.129811	6.3226	5.674581	6.02278	5.949182	Gpr37l1	NM_134438
6762411	767	9.258232	9.191314	9.368	9.224546	0.033686	0.73663	-0.03323	0.740023	0.143453	0.176339	-0.17669	0.105173	0.066918	0.508619	-0.10977	0.289303	6762411	9.164049	9.321785	9.288863	9.113837	9.392593	9.067512	9.436604	9.451352	9.216043	9.254034	9.193507	9.226098	Elf3	NM_007921
6762413	768	8.761948	8.829118	9.136595	8.606565	0.155383	0.382039	0.222553	0.221822	0.53003	0.013493	-0.30748	0.104582	-0.06717	0.699728	-0.37465	0.056279	6762413	8.538892	8.934314	8.812637	8.723047	9.034169	8.730139	9.250992	9.024026	9.134766	8.277719	8.823625	8.718351	Rnpep	NM_145417
6762420	769	9.709253	9.920163	9.862841	9.691757	0.017496	0.919455	0.228406	0.210196	0.171084	0.337373	0.057322	0.74123	-0.21091	0.243862	-0.15359	0.386392	6762420	9.511707	9.872204	9.743847	9.904554	9.916988	9.938946	9.999498	9.931692	9.657333	9.562847	9.665349	10.02372	Timm17a	NM_011590
6762423	770	9.87696	9.42071	9.667491	9.930738	-0.05378	0.825166	-0.51003	0.062317	-0.26325	0.296267	-0.24678	0.325491	0.456249	0.088813	0.209468	0.399891	6762423	9.888868	9.822346	9.919665	9.791103	9.312416	9.158612	9.68176	9.92988	9.390834	9.620864	9.8083	10.36305	Shisa4	NM_175259
6762425	771	8.207366	8.301929	8.185487	7.959296	0.248069	0.069031	0.342633	0.019913	0.226191	0.091963	0.116442	0.353327	-0.09456	0.446713	0.021878	0.857733	6762425	8.249459	8.191701	8.180937	8.239658	8.491369	8.174761	8.347343	8.040679	8.16844	7.761142	8.094312	8.022435	Ipo9	NM_153774
6762429	772	8.408815	8.419423	8.060513	7.955718	0.453097	0.04324	0.463705	0.039612	0.104795	0.594153	0.35891	0.093915	-0.01061	0.956587	0.348302	0.102384	6762429	8.568185	8.494926	8.163335	8.307878	8.627662	8.322729	7.979506	8.129201	8.072833	7.567119	8.276229	8.023807	Nav1	NM_173437
6762448	773	8.48354	8.219649	7.813206	7.522337	0.961203	0.212336	0.697312	0.354291	0.290869	0.692471	0.406443	0.582325	0.263892	0.719485	0.670334	0.372229	6762448	7.843026	8.477815	9.12978	7.978075	8.498959	8.181912	8.202394	7.047307	8.189917	7.077436	9.139966	6.349609	Pkp1	NM_019645
6762452	774	5.73911	6.622431	6.203337	7.395209	-1.6561	0.007744	-0.77278	0.13824	-1.19187	0.034726	0.419094	0.397908	-0.88332	0.096574	-0.46423	0.351536	6762452	5.922991	5.983999	5.310341	7.170958	6.096547	6.599788	5.779011	7.162281	5.668719	7.083816	7.192866	7.908945	Igfn1	EU429481
6762471	776	6.655379	6.662003	6.64067	6.598448	0.056931	0.512734	0.063555	0.466445	0.042222	0.625174	0.021332	0.803925	-0.00662	0.938441	0.014709	0.863937	6762471	6.635015	6.656682	6.67444	6.594683	6.777914	6.613411	6.657297	6.532652	6.732062	6.443661	6.728567	6.623116	5730559C	BC053100
6762603	778	8.083868	7.803359	8.043998	6.818248	1.26562	0.010056	0.985111	0.03119	1.22575	0.011771	-0.24064	0.541759	0.280509	0.47882	0.039869	0.918514	6762603	8.668591	7.939621	7.643391	7.745417	8.111382	7.553277	8.504829	7.474669	8.152497	6.576279	7.362036	6.516429	Ptprc	NM_001111316
6762612	779	9.127895	9.27183	9.169103	9.101638	0.026257	0.832572	0.170192	0.194585	0.067464	0.590031	0.102728	0.417662	-0.14394	0.265453	-0.04121	0.740595	6762612	9.142063	9.110342	9.131281	9.26976	9.346144	9.199587	9.348518	8.982225	9.176565	8.910262	9.055824	9.338829	Nek7	NM_021605
6762679	784	6.231476	6.091373	5.984993	5.844248	0.387228	0.102665	0.247125	0.273476	0.140745	0.521932	0.10638	0.626402	0.140103	0.523781	0.246483	0.27463	6762679	6.13283	6.018747	6.542852	5.913356	6.40216	5.958603	6.253619	5.826379	5.874981	6.050172	5.913345	5.569227	Ruvbl1	NM_019685
6762765	785	8.880721	8.885816	8.880799	9.039152	-0.15843	0.075269	-0.15334	0.08334	-0.15835	0.075386	0.005018	0.949986	-0.00509	0.949217	-7.7E-05	0.999229	6762765	8.901365	8.950502	8.790297	8.764797	8.904819	8.987833	8.820241	8.991474	8.830681	8.982898	9.138243	8.996316	Cdc73	NM_145991
6762773	786	7.873159	7.983419	8.020381	7.781966	0.091193	0.410562	0.201453	0.091401	0.238415	0.052901	-0.03696	0.734	-0.11026	0.324507	-0.14722	0.198603	6762773	7.81007	7.950419	7.858988	8.016442	7.993025	7.94079	8.121967	8.078152	7.861024	7.552295	7.919842	7.873761	Trove2	NM_013835
6762784	787	7.100502	7.199958	6.968808	6.19399	0.906512	0.008314	1.005968	0.004793	0.774819	0.017739	0.23115	0.400715	-0.09946	0.712514	0.131694	0.626754	6762784	7.358703	7.05707	6.885733	7.165674	7.452298	6.981902	7.495928	6.507421	6.903076	6.058094	6.441128	6.082747	Rgs2	NM_009061
6762796	789	6.398943	5.861645	5.697642	5.06306	1.335883	0.021127	0.798585	0.125609	0.634582	0.211238	0.164003	0.734504	0.537298	0.283124	0.701301	0.171545	6762796	7.029663	5.961536	6.205629	5.879643	6.226139	5.479153	6.374433	4.806575	5.911918	5.408559	5.232175	4.548446	Rgs1	NM_015811
6762944	791	7.752032	7.859683	7.887355	7.060924	0.691108	0.037112	0.798759	0.020323	0.826431	0.01744	-0.02767	0.922819	-0.10765	0.707455	-0.13532	0.638006	6762944	7.519096	7.865787	7.871212	7.429461	8.071717	8.077872	8.364484	7.511745	7.785836	7.064751	7.359918	6.758103	Pla2g4a	NM_008869
6762964	792	8.096172	8.319694	8.326199	8.321034	-0.22486	0.196476	-0.00134	0.993503	0.005165	0.974975	-0.00651	0.968482	-0.22352	0.198884	-0.23003	0.187433	6762964	7.983023	8.235613	8.069881	8.24971	8.14584	8.563531	8.495725	8.128822	8.35405	8.078542	8.33884	8.545721	BC003331	NM_145511
6762967	793	8.505782	8.976691	8.263972	7.472965	1.032817	0.082833	1.503726	0.020348	0.791006	0.167563	0.712719	0.208648	-0.47091	0.392633	0.241811	0.655035	6762967	8.387193	8.78257	8.347584	9.34872	9.675052	7.906301	8.593558	8.157093	8.041264	6.64256	8.177998	7.598338	Prg4	NM_021400
6763011	794	7.108725	7.258118	7.323059	7.312718	-0.20399	0.246727	-0.0546	0.746609	0.01034	0.951044	-0.06494	0.701154	-0.14939	0.386833	-0.21433	0.225575	6763011	6.892339	7.269907	7.16393	6.958265	7.232176	7.583914	7.279704	7.228432	7.46104	7.227387	7.411799	7.298969	1200016B	NM_025819
6763014	795	8.526347	8.767518	8.484422	8.46988	0.056467	0.59041	0.297638	0.018284	0.014542	0.888776	0.283097	0.02282	-0.24117	0.043555	0.041925	0.688175	6763014	8.474648	8.584608	8.519784	8.572741	8.974507	8.755307	8.578328	8.486546	8.388391	8.566153	8.384657	8.45883	Rnf2	NM_011277
6763059	796	9.170459	8.951988	8.474716	8.366596	0.803863	0.011342	0.585392	0.044413	0.10812	0.67163	0.477272	0.088077	0.21847	0.399977	0.695742	0.022119	6763059	9.402299	8.942879	9.166198	8.901752	9.132952	8.821261	8.796551	8.041882	8.585716	8.351287	8.737083	8.011418	1700025G	BC034723
6763078	798	6.568202	6.412778	6.197883	6.400302	0.167901	0.311606	0.012476	0.938005	-0.20242	0.229125	0.214895	0.204232	0.155424	0.346691	0.370319	0.044398	6763078	6.371213	6.417149	6.916245	6.184941	6.525362	6.528031	6.2375	6.130901	6.225248	6.484621	6.280968	6.435316	Tsen15	NM_025677
6763090	799	8.220654	8.128832	8.073107	7.544133	0.676521	0.030775	0.584698	0.053544	0.528973	0.074909	0.055725	0.834699	0.091822	0.731586	0.147547	0.58378	6763090	8.58048	8.228176	7.853306	8.127008	8.375785	7.883702	8.278832	7.830352	8.110136	7.088167	7.743329	7.800904	Rgl1	NM_016846
6763110	800	8.426987	8.459015	8.398631	8.391763	0.035224	0.566942	0.067252	0.287243	0.006868	0.91018	0.060384	0.335966	-0.03203	0.601964	0.028356	0.643612	6763110	8.443159	8.398235	8.439567	8.401006	8.578934	8.397106	8.35127	8.481499	8.363125	8.333976	8.462809	8.378504	Smg7	NM_001005507
6763129	801	9.480211	9.179215	9.319344	8.617052	0.86316	0.003821	0.562163	0.030618	0.702292	0.01129	-0.14013	0.531917	0.300996	0.198164	0.160868	0.474771	6763129	9.546644	9.453983	9.440007	8.904848	9.314041	9.318756	9.639329	8.922251	9.396451	8.340919	8.593659	8.916577	Lamc2	NM_008485
6763132	802	10.65082	10.70833	10.02584	10.3912	0.25962	0.522444	0.317127	0.437605	-0.36536	0.374143	0.682489	0.116772	-0.05751	0.885895	0.624983	0.146058	6763132	11.05512	10.37946	10.51788	10.67028	11.37497	10.07973	10.60473	9.587341	9.885441	10.66582	10.41549	10.09229	Lamc1	NM_010683
6763144	803	9.117095	9.272769	9.079662	8.98216	0.134935	0.227743	0.290554	0.022741	0.097502	0.37283	0.193052	0.098562	-0.15562	0.170342	0.037433	0.726445	6763144	9.273803	9.100564	8.976917	9.188035	9.313195	9.316911	9.200742	8.994714	9.04353	8.801132	9.057655	9.087692	Dhx9	NM_007842
6763146	804	7.730664	7.708384	7.047398	7.146216	0.584448	0.30394	0.562168	0.32154	-0.09882	0.857276	0.660986	0.249283	0.02228	0.967624	0.683265	0.234997	6763146	8.16367	7.360248	7.668073	7.986011	8.392944	6.746197	7.997049	6.542941	6.602205	7.238908	7.438427	6.761313	Npl	NM_028749
6763207	807	7.777562	7.96015	7.850977	7.723389	0.054173	0.781879	0.236761	0.246102	0.127588	0.51905	0.109173	0.579754	-0.18259	0.362733	-0.07341	0.708088	6763207	8.010048	7.79083	7.531808	7.715584	8.19908	7.965786	7.947341	8.017691	7.587898	7.524174	7.696314	7.949678	Ier5	NM_010500
6763208	808	8.71068	8.985377	8.924783	8.922949	-0.21227	0.233523	0.062427	0.714552	0.001834	0.991391	0.060594	0.722517	-0.2747	0.133936	-0.2141	0.229863	6763208	8.575228	8.767881	8.788932	9.054299	9.112869	8.788962	8.921642	8.941002	8.911705	8.652782	8.804728	9.311338	Mr1	NM_008209
6763217	809	9.347809	9.271043	9.265863	9.268024	0.079785	0.438429	0.003019	0.976141	-0.00216	0.982918	0.00518	0.959074	0.076766	0.455307	0.081946	0.426613	6763217	9.322169	9.338767	9.382491	9.195794	9.373012	9.244324	9.065788	9.421349	9.310452	9.134577	9.375115	9.294381	Xpr1	NM_011273
6763231	810	6.160122	6.199546	6.186377	6.273127	-0.113	0.172357	-0.07358	0.357746	-0.08675	0.28319	0.013169	0.865702	-0.03942	0.61527	-0.02625	0.736694	6763231	6.126156	6.238284	6.115926	6.27552	6.135243	6.187824	6.338647	6.074785	6.145698	6.191051	6.286092	6.342238	Lhx4	NM_010712
6763235	811	8.497122	8.719829	8.220567	8.426347	0.070776	0.6865	0.293482	0.120792	-0.20578	0.258227	0.499262	0.018339	-0.22271	0.22422	0.276555	0.140522	6763235	8.666126	8.343969	8.481272	8.799448	8.952225	8.407814	8.290416	8.060946	8.31034	8.280061	8.676281	8.322698	Qsox1	NM_001024945
6763238	812	8.846703	8.87495	8.933891	8.815043	0.03166	0.759367	0.059907	0.565237	0.118848	0.268185	-0.05894	0.571389	-0.02825	0.7845	-0.08719	0.408129	6763238	8.763124	8.942131	8.834855	8.806322	8.932261	8.886267	8.852119	9.111237	8.838318	8.716216	8.993742	8.735172	Cep350	BC089561
6763240	813	8.43863	8.594349	8.520711	8.575027	-0.1364	0.135413	0.019322	0.819952	-0.05432	0.527056	0.073638	0.396198	-0.15572	0.094604	-0.08208	0.346961	6763240	8.39589	8.507529	8.412472	8.512624	8.556254	8.714169	8.590893	8.585668	8.385571	8.48773	8.539958	8.697393	Tor1aip1	NM_144791
6763247	814	6.057456	6.523325	6.160277	6.612163	-0.55471	0.237128	-0.08884	0.842954	-0.45189	0.328312	0.363049	0.427234	-0.46587	0.31448	-0.10282	0.818714	6763247	5.367269	6.267902	6.537197	5.969651	6.408453	7.191872	5.887486	6.257991	6.335353	6.090665	6.540385	7.205439	Fam163a	NM_177838
6763259	815	9.632776	9.580304	9.341742	9.352206	0.28057	0.220159	0.228098	0.311065	-0.01046	0.961652	0.238562	0.290843	0.052472	0.80982	0.291034	0.205015	6763259	9.833462	9.483505	9.581361	9.610172	9.949447	9.181293	9.453152	9.170628	9.401446	9.59299	9.373528	9.0901	Soat1	NM_009230
6763273	816	8.615847	8.535214	8.467014	8.451826	0.164021	0.446348	0.083387	0.694575	0.015188	0.942706	0.068199	0.7477	0.080634	0.704085	0.148833	0.488129	6763273	8.554061	8.590561	8.70292	8.494123	8.763434	8.348084	8.656686	8.321251	8.423106	8.011697	8.509399	8.834383	Tor3a	NM_023141
6763275	817	8.703402	8.701121	8.597665	8.541613	0.161789	0.313862	0.159508	0.320303	0.056052	0.719322	0.103455	0.511387	0.002282	0.988279	0.105737	0.502382	6763275	8.587272	8.582109	8.940826	8.508308	8.895696	8.699358	8.759028	8.510521	8.523447	8.323901	8.683094	8.617844	Fam20b	NM_145413
6763493	821	10.45735	10.46456	10.55298	10.48333	-0.02598	0.854667	-0.01877	0.894659	0.06965	0.625767	-0.08842	0.537734	-0.00721	0.959423	-0.09563	0.505981	6763493	10.44784	10.39053	10.53367	10.23169	10.58556	10.57642	10.61701	10.74221	10.29972	10.41463	10.62606	10.4093	Cacybp	NM_009786
6763495	822	7.965711	7.97027	7.881606	7.704369	0.261342	0.050144	0.265901	0.047095	0.177237	0.156765	0.088664	0.456891	-0.00456	0.968925	0.084105	0.479576	6763495	8.045752	8.010778	7.840603	7.959282	8.085047	7.86648	7.994613	7.727658	7.922547	7.710773	7.885571	7.516763	Rabgap1l	NM_013862
6763517	823	6.861465	7.002891	6.98504	6.957904	-0.09644	0.460952	0.044987	0.727261	0.027136	0.832956	0.017851	0.889559	-0.14143	0.288965	-0.12357	0.350097	6763517	6.879398	6.986938	6.71806	6.94991	6.889913	7.168851	6.994757	6.907838	7.052525	6.846815	6.816945	7.209952	Zbtb37	NM_173424
6763520	824	6.759522	6.975355	6.869175	6.829556	-0.07003	0.608423	0.145799	0.299282	0.039619	0.770646	0.10618	0.442284	-0.21583	0.138996	-0.10965	0.428083	6763520	6.669624	6.713349	6.895594	6.976898	7.103145	6.846022	7.037101	6.757818	6.812606	6.615258	6.809047	7.064364	Dars2	NM_172644
6763524	825	8.1502	8.438736	8.209844	8.021239	0.128962	0.17072	0.417498	0.001236	0.188605	0.058887	0.228892	0.028303	-0.28854	0.009823	-0.05946	0.506091	6763524	8.119601	8.205215	8.125785	8.407213	8.48512	8.423876	8.198087	8.085692	8.345753	7.886015	8.185611	7.99209	Klhl20	NM_001039482
6763537	826	9.661892	9.959729	9.665728	10.0364	-0.37451	0.106916	-0.07667	0.71973	-0.37067	0.110001	0.294001	0.19182	-0.29784	0.186687	-0.00384	0.985616	6763537	9.471243	9.52244	9.991992	10.22829	9.931963	9.718933	9.8209	9.868424	9.307859	10.06344	9.92184	10.12391	Prdx6	NM_007453
6763585	828	6.13739	6.075129	5.947987	5.963433	0.173958	0.148644	0.111696	0.334802	-0.01545	0.89066	0.127141	0.276375	0.062262	0.583	0.189403	0.120006	6763585	6.319133	6.188971	5.904067	6.158622	6.159575	5.907189	6.005602	5.933744	5.904616	5.922288	6.013773	5.954237	Dnm3	NM_001038619
6763591	829	6.347501	6.408627	6.45579	6.409862	-0.06236	0.6774	-0.00124	0.993387	0.045928	0.758705	-0.04716	0.752465	-0.06113	0.683374	-0.10829	0.474998	6763591	6.37514	6.437122	6.23024	6.166327	6.526579	6.532975	6.301493	6.703708	6.36217	6.256643	6.559275	6.413669	Mettl13	NM_144877
6763623	831	8.096262	7.982037	7.761104	7.649399	0.446863	0.263524	0.332638	0.396827	0.111705	0.771372	0.220933	0.568567	0.114225	0.766392	0.335158	0.393424	6763623	8.285341	7.992438	8.011008	8.63854	7.868036	7.439535	8.276686	7.588896	7.417731	7.231493	8.166162	7.550542	Fmo1	NM_010231
6763626	832	6.494877	6.602039	5.909321	7.239616	-0.74474	0.178281	-0.63758	0.242053	-1.33029	0.029905	0.692718	0.207135	-0.10716	0.837157	0.585556	0.279408	6763626	6.627081	6.268947	6.588602	7.209986	6.630666	5.965466	5.948643	6.435779	5.343541	6.768346	8.272638	6.677863	Fmo2	NM_018881

6763646	834	7.696181	7.480503	6.907101	6.856972	0.839209	0.041289	0.623531	0.108804	0.050129	0.888243	0.573402	0.135615	0.215678	0.549897	0.78908	0.051781	6763646	8.016883	7.495428	7.576232	7.590525	7.843782	7.007202	7.283303	6.53247	6.90553	6.27271	7.387074	6.911133	Prrx1	NM_175686
6763652	835	6.400553	6.361334	6.423725	6.39247	0.008083	0.925966	-0.03114	0.721436	0.031256	0.720416	-0.06239	0.480354	0.039219	0.654146	-0.02317	0.790356	6763652	6.375447	6.354876	6.471335	6.19344	6.456173	6.434389	6.414876	6.471519	6.384781	6.254809	6.424468	6.498132	Gorab	NM_178883
6763695	837	7.98472	8.100636	7.85232	8.040703	-0.05598	0.605567	0.059933	0.580846	-0.18838	0.108124	0.248316	0.04427	-0.11592	0.298087	0.1324	0.239399	6763695	7.882598	7.959527	8.112036	8.004377	8.227695	8.069837	7.969609	7.937744	7.649608	7.966721	8.133396	8.021993	Blzf1	NM_025505
6763706	838	12.73985	12.78199	12.64896	13.00005	-0.2602	0.050478	-0.21806	0.090076	-0.35109	0.014593	0.13303	0.273463	-0.04214	0.719216	0.09089	0.444974	6763706	12.80937	12.71193	12.69825	12.82196	12.70899	12.81502	12.67666	12.78531	12.48491	12.94906	12.81571	13.23539	Atp1b1	NM_009721
6763752	841	9.819576	9.756479	10.01823	10.03696	-0.21738	0.076836	-0.28048	0.030676	-0.01872	0.865532	-0.26175	0.040275	0.063097	0.571934	-0.19866	0.100652	6763752	9.795843	9.816251	9.846634	9.669573	9.722731	9.877133	9.998608	10.18387	9.87222	9.893908	9.97966	10.2373	Sft2d2	NM_145512
6763754	842	7.450547	7.446876	7.45506	7.554699	-0.10415	0.336542	-0.10782	0.320798	-0.09964	0.356707	-0.00818	0.937942	0.003671	0.972139	-0.00451	0.965747	6763754	7.437206	7.535225	7.379209	7.295891	7.52053	7.524206	7.441176	7.656881	7.267124	7.551742	7.584918	7.527437	Tiprl	NM_145513
6763777	843	8.547807	8.921762	8.70204	8.753559	-0.20575	0.064763	0.168203	0.118311	-0.05152	0.606616	0.219722	0.051631	-0.37396	0.004611	-0.15423	0.147326	6763777	8.561458	8.481669	8.600294	8.885014	9.030107	8.850166	8.837736	8.752022	8.516362	8.793366	8.616141	8.851171	Mpzl1	NM_001083897
6763810	845	8.31135	8.295138	8.274207	8.281908	0.029442	0.662304	0.01323	0.843641	-0.0077	0.908521	0.020931	0.75546	0.016212	0.809141	0.037143	0.583035	6763810	8.343937	8.307232	8.282881	8.387226	8.270773	8.227415	8.241068	8.244657	8.336895	8.227497	8.197962	8.420264	Pou2f1	NM_198934
6763853	848	8.193727	8.199786	8.23541	8.216645	-0.02292	0.750127	-0.01686	0.814497	0.018766	0.794049	-0.03562	0.622199	-0.00606	0.932688	-0.04168	0.565376	6763853	8.262526	8.24956	8.069094	8.183926	8.1538	8.261632	8.200486	8.1646	8.341145	8.132566	8.272929	8.244439	Pogk	NM_175170
6763894	850	8.814734	9.178985	8.845429	8.747672	0.067061	0.744153	0.431312	0.061523	0.097757	0.635579	0.333556	0.131359	-0.36425	0.103806	-0.0307	0.880926	6763894	8.824084	8.648115	8.972002	8.988209	9.636384	8.912361	8.979871	8.719752	8.836664	8.736929	8.939232	8.566856	Uck2	NM_030724
6763903	851	8.363275	8.451331	8.173551	8.17286	0.190415	0.424191	0.278471	0.2531	0.000691	0.997637	0.27778	0.254184	-0.08806	0.707125	0.189724	0.425806	6763903	8.203439	8.110844	8.775542	8.566393	8.505733	8.281868	8.480133	8.283427	7.757094	8.066354	8.306413	8.145814	Mgst3	NM_025569
6763937	853	9.625615	9.243565	9.222956	9.385971	0.239644	0.096066	-0.14241	0.294997	-0.16301	0.235515	0.020609	0.8752	0.38205	0.016912	0.402659	0.013228	6763937	9.770442	9.562359	9.544044	9.120609	9.354731	9.255355	9.252833	9.251226	9.16481	9.673362	9.189322	9.295228	Pbx1	NM_183355
6763941	854	8.163776	7.249549	7.092806	7.913017	0.25076	0.500574	-0.66347	0.098952	-0.82021	0.049896	0.156743	0.670933	0.914227	0.033034	1.07097	0.016743	6763941	8.428439	7.930227	8.132663	7.365156	7.579795	6.803697	6.462964	7.60394	7.211514	8.410336	7.779626	7.549088	---	BC023710
6763991	856	7.71134	7.561175	7.327524	6.641306	1.070034	0.028365	0.919869	0.050846	0.686218	0.125209	0.233651	0.575951	0.150165	0.717629	0.383816	0.36626	6763991	7.468028	7.491954	8.174037	7.163647	8.066723	7.453155	7.89424	6.775027	7.313304	6.040084	7.019008	6.864826	Rgs4	NM_009062
6764007	857	7.312284	7.358351	7.207663	6.809902	0.502382	0.130013	0.548449	0.102715	0.397762	0.218311	0.150687	0.626422	-0.04607	0.88087	0.104621	0.734369	6764007	7.404484	7.115342	7.417026	7.19389	7.81082	7.070342	7.534237	7.068575	7.020178	6.244835	7.251565	6.933305	Hsd17b7	NM_010476
6764010	858	8.675273	7.516253	7.90081	8.210382	0.464891	0.265724	-0.69413	0.111815	-0.30957	0.448562	-0.38456	0.351254	1.15902	0.017515	0.774463	0.081345	6764010	8.960772	8.288841	8.776206	7.574279	8.031252	6.943227	7.973756	7.918849	7.809826	8.890111	8.235468	7.505567	Ddr2	NM_022563
6764011	859	8.532846	8.549486	8.680096	8.369797	0.16305	0.512477	0.179689	0.471711	0.310299	0.228396	-0.13061	0.597984	-0.01664	0.945955	-0.14725	0.553159	6764011	8.41356	8.461063	8.723916	8.380192	8.999427	8.268839	8.849836	8.83991	8.350541	8.366175	8.645852	8.097363	Uap1	NM_133806
6764016	860	9.006099	9.050097	9.102366	8.933549	0.07255	0.560323	0.116548	0.357639	0.168817	0.195147	-0.05227	0.673163	-0.044	0.72209	-0.09627	0.443439	6764016	9.048344	8.979684	8.990269	8.911531	9.216557	9.022203	9.211551	9.09666	8.998887	8.738838	9.174355	8.887455	Uhmk1	NM_010633
6764033	861	6.496198	6.517778	6.547695	6.443041	0.053157	0.565838	0.074737	0.424263	0.104654	0.272271	-0.02992	0.744756	-0.02158	0.814041	-0.0515	0.577784	6764033	6.461923	6.575972	6.4507	6.527046	6.564865	6.461422	6.592029	6.532513	6.518544	6.255749	6.645774	6.4276	Dusp12	NM_023173
6764034	862	6.000073	6.007724	5.923302	5.768799	0.231274	0.149437	0.238925	0.13807	0.154503	0.317804	0.084422	0.576512	-0.00765	0.959217	0.076771	0.610919	6764034	6.151991	5.975882	5.872346	6.001484	6.171252	5.850437	5.960932	5.882041	5.926933	5.980926	5.450304	5.875167	Fcrlb	NM_001029984
6764038	864	9.572365	9.286442	9.282003	8.217271	1.355094	0.002362	1.069171	0.008661	1.064732	0.008846	0.004439	0.988914	0.285923	0.382842	0.290362	0.375861	6764038	9.939548	9.382383	9.395165	9.299677	9.503276	9.056373	9.626867	9.003321	9.215821	8.630219	8.453248	7.568347	Fcgr2b	NM_001077189
6764040	865	8.22157	8.748556	8.098889	7.292821	0.928749	0.017466	1.455735	0.001584	0.806068	0.032077	0.649667	0.070231	-0.52699	0.128766	0.122682	0.703661	6764040	8.255406	7.997817	8.411488	8.768664	9.007205	8.469799	8.444683	7.65588	8.196103	7.08839	7.915332	6.874741	Fcgr3	NM_010188
6764043	866	9.318018	9.087534	9.219955	9.555146	-0.23713	0.154805	-0.46761	0.014707	-0.33519	0.057111	-0.13242	0.40587	0.230484	0.165262	0.098063	0.534083	6764043	9.277429	9.178781	9.497845	9.210976	9.021989	9.029638	9.207436	9.417417	9.035013	9.464505	9.364422	9.83651	Sdhc	NM_025321
6764046	867	6.849384	7.058319	6.675713	6.565021	0.284363	0.213176	0.493298	0.046946	0.110692	0.612816	0.382606	0.106272	-0.20894	0.349423	0.173671	0.432707	6764046	6.851069	6.607598	7.089486	7.164166	7.109694	6.901098	7.029417	6.21618	6.781542	6.454436	6.690686	6.549941	Pcp4l1	NM_025557
6764048	868	7.48368	7.491331	7.452435	7.388398	0.095282	0.582249	0.102934	0.552977	0.064037	0.710098	0.038897	0.820862	-0.00765	0.964415	0.031245	0.85558	6764048	7.515283	7.372129	7.563628	7.501697	7.532649	7.439648	7.763453	7.356524	7.237327	7.067129	7.559632	7.538432	Tomm40l	NM_001037170
6764049	869	11.07385	10.54696	11.22989	9.810832	1.263022	0.013225	0.736132	0.101991	1.419062	0.007401	-0.68293	0.125021	0.52689	0.222784	-0.15604	0.705684	6764049	11.39457	11.03156	10.79543	10.58186	10.86694	10.19209	11.63217	10.66864	11.38887	9.914201	10.45805	9.060244	Fcer1g	NM_010185
6764050	870	8.181313	8.28928	8.199129	8.373112	-0.1918	0.220182	-0.08383	0.577026	-0.17398	0.262101	0.09015	0.549292	-0.10797	0.475483	-0.01782	0.904725	6764050	8.042925	8.158855	8.34216	8.330882	8.364346	8.172611	8.361759	8.175678	8.059951	8.162476	8.291176	8.665685	Ndufs2	NM_153064
6764053	871	7.844073	8.024506	7.773907	7.921201	-0.07713	0.451806	0.103305	0.320374	-0.14729	0.169376	0.250599	0.033139	-0.18043	0.101437	0.070167	0.492297	6764053	7.728903	7.832476	7.970841	7.931421	8.196101	7.945996	7.62317	7.903462	7.795088	7.921846	7.935346	7.90641	Ppox	NM_008911
6764054	872	8.431667	8.473121	8.367707	8.418123	0.013543	0.909201	0.054998	0.645444	-0.05042	0.67285	0.105414	0.386363	-0.04145	0.727961	0.06396	0.593493	6764054	8.381388	8.381755	8.531857	8.403238	8.73326	8.282865	8.390008	8.378143	8.33497	8.378951	8.562358	8.313061	Usp21	NM_013919
6764056	873	7.560665	7.713039	7.581064	7.576796	-0.01613	0.903944	0.136243	0.323522	0.004268	0.974517	0.131975	0.337993	-0.15237	0.273179	-0.0204	0.87874	6764056	7.46121	7.567335	7.653449	7.746875	7.76805	7.624193	7.850563	7.469587	7.423042	7.405866	7.57062	7.753902	Ufc1	NM_025388
6764068	874	8.760287	8.949313	8.72942	8.918376	-0.15809	0.365336	0.030937	0.855711	-0.18896	0.284522	0.219893	0.218676	-0.18903	0.284354	0.030866	0.856038	6764068	8.503432	8.769391	9.008037	8.98632	8.997972	8.863648	8.828608	8.741055	8.618598	8.643607	8.895065	9.216456	Nit1	NM_012049
6764093	877	6.28602	7.144683	5.869015	5.558651	0.727369	0.094091	1.586032	0.003248	0.310364	0.441172	1.275668	0.010373	-0.85866	0.055256	0.417005	0.307953	6764093	6.270123	5.926385	6.661551	7.108384	7.498517	6.827147	6.042142	5.422761	6.142142	4.915126	6.29113	5.469697	Slamf7	NM_144539
6764118	878	6.673265	7.024503	6.655195	6.736825	-0.06356	0.6377	0.287678	0.057628	-0.08163	0.547181	0.369308	0.021693	-0.35124	0.02689	0.01807	0.892784	6764118	6.846727	6.602347	6.57072	6.861157	7.260884	6.951468	6.760794	6.742189	6.462601	6.643895	6.775479	6.791101	Vangl2	NM_033509
6764123	880	10.44844	10.49955	10.37472	10.42763	0.020813	0.861715	0.071917	0.551525	-0.05291	0.659604	0.12483	0.312091	-0.0511	0.670418	0.073727	0.541791	6764123	10.42583	10.31071	10.60879	10.38986	10.71923	10.38955	10.45834	10.27045	10.39536	10.33433	10.39775	10.55081	Ncstn	NM_021607
6764133	881	9.733281	9.53005	9.790752	9.753079	-0.0198	0.848054	-0.22303	0.056344	0.037674	0.716264	-0.2607	0.031321	0.20323	0.076672	-0.05747	0.581416	6764133	9.858356	9.742586	9.5989	9.496988	9.708876	9.384287	9.859341	9.735138	9.777778	9.649526	9.741886	9.867824	Pea15a	NM_011063
6764134	882	7.389642	6.91469	6.912141	6.636592	0.75305	0.633067	0.278098	0.859152	0.275549	0.860428	0.002549	0.998701	0.474952	0.762297	0.477501	0.761067	6764134	5.062664	6.773592	10.33267	8.130556	7.33518	5.278334	8.450768	6.234934	6.05072	4.772376	7.389787	7.747612	Casq1	NM_009813
6764138	883	7.03618	7.049098	6.742379	6.759805	0.276375	0.764857	0.289293	0.754279	-0.01743	0.984909	0.306719	0.740098	-0.01292	0.988812	0.293801	0.750601	6764138	5.719668	6.489411	8.89946	8.077164	6.841438	6.228693	7.374591	6.663553	6.188993	5.736383	7.33922	7.203812	Atp1a2	NM_178405
6764175	885	6.805989	6.923765	6.770278	5.787339	1.01865	0.012623	1.136426	0.007301	0.98294	0.014952	0.153486	0.642664	-0.11778	0.721047	0.035711	0.913459	6764175	6.572256	6.850914	6.994797	6.863194	7.076738	6.831362	6.957013	6.087375	7.266447	5.713081	6.232419	5.416516	Slamf8	NM_029084
6764182	886	6.127582	6.924963	6.103047	6.638837	-0.51125	0.082685	0.286126	0.299366	-0.53579	0.071337	0.821916	0.012848	-0.79738	0.014832	0.024535	0.926531	6764182	5.58139	6.232591	6.568765	7.14338	6.873076	6.758432	6.037811	6.179363	6.091967	6.349511	6.983354	6.583645	Dusp23	NM_026725
6764209	891	7.201478	6.832661	7.37825	6.444134	0.757344	0.033746	0.388527	0.225805	0.934117	0.013492	-0.54559	0.102595	0.368817	0.248097	-0.17677	0.566958	6764209	7.391722	7.312976	6.899735	7.254499	6.864443	6.37904	7.17185	7.32028	7.642621	6.711751	6.700268	5.920382	Darc	NM_010045
6764211	892	8.923564	8.224958	9.574179	8.298543	0.625021	0.088771	-0.07358	0.82534	1.275635	0.004218	-1.34922	0.003075	0.698606	0.062309	-0.65061	0.078519	6764211	9.001792	9.344428	8.424472	8.474382	8.00296	8.197533	9.382231	9.667954	9.672351	8.849093	8.335556	7.710981	Cadm3	NM_053199
6764243	894	6.283583	5.994604	5.604456	5.050582	1.233002	0.060907	0.944022	0.133728	0.553874	0.356225	0.390148	0.509921	0.28898	0.623273	0.679127	0.264296	6764243	6.846983	5.800448	6.203319	5.435876	7.217649	5.330286	5.862567	5.224571	5.72623	4.427581	5.698153	5.026011	Ifi205	NM_172648
6764246	895	7.707018	7.656968	7.473313	6.66972	1.037298	0.017947	0.987248	0.022354	0.803593	0.050573	0.183655	0.613584	0.05005	0.889697	0.233705	0.522619	6764246	7.983048	7.533362	7.604643	7.278307	8.364248	7.328349	7.882408	7.011862	7.525669	6.291105	6.857129	6.860926	Ifi204	NM_008329
6764274	896	6.795207	6.346434	6.743848	6.10934	0.685867	0.085308	0.237094	0.516622	0.634508	0.106957	-0.39741	0.288358	0.448773	0.235015	0.051359	0.886795	6764274	7.017245	6.637801	6.730576	5.888958	6.790316	6.360029	7.057482	6.071551	7.102512	5.70075	6.473802	6.153468	Ifi203	NM_001045481
6764342	900	6.155422	6.433166	6.150412	6.047884	0.107538	0.362546	0.385282	0.008576	0.102528	0.384194	0.282754	0.034778	-0.27774	0.037302	0.00501	0.965226	6764342	6.216445	6.15056	6.09926	6.532358	6.511953	6.255186	6.060894	6.055137	6.335205	6.068635	5.892006	6.18301	Grem2	NM_011825
6764349	901	5.691198	7.229208	6.411517	9.320248	-3.62905	0.000443	-2.09104	0.010915	-2.90873	0.001789	0.81769	0.233386	-1.53801	0.041545	-0.72032	0.289001	6764349	4.684069	6.460789	5.928737	7.736552	6.357477	7.593594	5.801787	7.134734	6.298031	8.66701	9.163475	10.13026	Rgs7	NM_011880
6764351	902	9.90131	10.35603	10.33896	11.36974	-1.46843	0.000561	-1.01371	0.005163	-1.03078	0.004714	0.017073	0.950412	-0.45472	0.12582	-0.43765	0.138618	6764351	9.61268	10.0875	10.00375	10.2204	10.16681	10.68089	10.23971	10.79411	9.983061	11.49234	10.99611	11.62077	Fh1	NM_010209
6764352	903	6.761194	6.574535	6.687974	6.808234	-0.04704	0.749099	-0.2337	0.138637	-0.12026	0.421929	-0.11344	0.4477	0.186659	0.225366	0.07322	0.620261	6764352	6.762796	6.725079	6.795707	6.612031	6.558659	6.552915	6.902529	6.695877	6.465515	7.094583	6.567438	6.762681	Opn3	NM_010098
6764353	904	7.407086	7.463594	7.350603	7.298499	0.108587	0.469288	0.165095	0.281471	0.052104	0.724945	0.11299	0.452091	-0.05651	0.702967	0.056483	0.703087	6764353	7.313519	7.240252	7.667488	7.313371	7.632807	7.444603	7.53167	7.284527	7.235613	7.155897	7.434601	7.304999	Chml	NM_021350
6764401	906	6.445081	6.493158	6.275413	6.096768	0.348313	0.108962	0.396389	0.074222	0.178645	0.382016	0.217745	0.292223	-0.04808	0.809682	0.169668	0.405268	6764401	6.717769	6.345783	6.271692	6.344862	6.647468	6.487143	6.492861	5.967314	6.366064	5.939598	6.398933	5.951774	Cep170	NM_001099637
6764435	907	9.019482	8.886129	8.688307	8.501167	0.518315	0.000762	0.384962	0.004487	0.18714	0.093924	0.197822	0.07943	0.133353	0.212703	0.331175	0.009889	6764435	9.090664	9.003283	8.964499	8.897964	8.841586	8.918837	8.785915	8.722662	8.556345	8.359277	8.724609	8.419616	Akt3	NM_011785
6764457	908	9.322811	9.435376	9.363912	9.292624	0.030187	0.77069	0.142751	0.191705	0.071288	0.496632	0.071463	0.495606	-0.11256	0.29343	-0.0411	0.692162	6764457	9.322788	9.27327	9.372375	9.307445	9.52114	9.477542	9.532096	9.229552	9.330089	9.138632	9.311337	9.427904	Adss	NM_007422
6764481	909	10.93977	10.99713	11.08305	10.96668	-0.02691	0.672786	0.030447	0.633322	0.116373	0.09465	-0.08593	0.199241	-0.05736	0.377568	-0.14328	0.047894	6764481	10.97708	10.97459	10.86764	10.86974	11.07461	11.04703	11.11823	11.08663	11.0443	10.89078	11.03031	10.97895	Hnrnpu	NM_016805
6764526	910	7.39884	7.490671	7.505606	7.557369	-0.15853	0.066128	-0.0667	0.397026	-0.05176	0.50708	-0.01494	0.846199	-0.09183	0.252945	-0.10677	0.189945	6764526	7.261402	7.483447	7.451671	7.486955	7.503674	7.481384	7.49067	7.523312	7.502836	7.6201	7.401261	7.650745	Smyd3	NM_027188
6764562	911	8.384233	8.432345	8.40913	8.417758	-0.03352	0.683907	0.014587	0.858774	-0.00863	0.916127	0.023215	0.777385	-0.04811	0.561255	-0.0249	0.761816	6764562	8.391341	8.378842	8.382517	8.359791	8.563511	8.373733	8.425723	8.487382	8.314286	8.328191	8.567901	8.357182	Ahctf1	NM_026375
6764578	912	6.799509	6.965779	6.702833	6.659654	0.139855	0.691306	0.306125	0.393726	0.043179	0.901971	0.262947	0.461067	-0.16627	0.637583	0.096677	0.783138	6764578	6.246198	6.552329	7.600001	7.313962	6.741706	6.84167	6.969573	6.619351	6.519574	6.458524	6.668291	6.852147	Cabc1	NM_023341
6764580	913	7.093589	7.141851	7.122617	7.101745	-0.00816	0.969498	0.040106	0.851016	0.020872	0.922068	0.019234	0.928163	-0.04826	0.821279	-0.02903	0.891808	6764580	7.398669	6.951045	6.931054	7.091146	7.346667	6.987741	7.468222	7.002898	6.896732	7.379858	6.918028	7.00735	Psen2	NM_001128605
6764582	914	6.103848	6.110612	6.703576	6.036624	0.067225	0.857222	0.073988	0.84307	0.666952	0.102506	-0.59296	0.139861	-0.00676	0.985543	-0.59973	0.135983	6764582	6.184555	5.626208	6.500782	5.941932	6.564502	5.825401	6.034717	6.871133	7.204877	6.241484	6.125336	5.743051	EG277333	NR_003623
6764650	917	10.36413	10.25416	9.964449	10.26363	0.100493	0.595828	-0.00947	0.959768	-0.29918	0.138736	0.289715	0.149993	0.109963	0.562353	0.399678	0.059307	6764650	10.48831	10.17456	10.42951	10.41675	10.24663	10.09911	10.16787	9.829352	9.896124	10.0958	10.04377	10.65133	Ephx1	NM_010145
6764657	918	7.515983	7.82768	7.838396	7.600933	-0.08495	0.452624	0.226747	0.068178	0.237463	0.058381	-0.01072	0.923129	-0.3117	0.019997	-0.32241	0.017173	6764657	7.567913	7.411399	7.568638	7.713271	8.063591	7.706179	7.876975	7.799831	7.838382	7.473575	7.741229	7.587996	Nvl	NM_026171
6764662	919	9.648904	9.701413	9.39031	9.442374	0.20653	0.064554	0.259039	0.027631	-0.05206	0.60388	0.311103	0.012114	-0.05251	0.60085	0.258595	0.027829	6764662	9.751925	9.676322	9.518466	9.650545	9.858849	9.594845	9.409588	9.470913	9.290428	9.306808	9.51309	9.507224	Wdr26	NM_145514

6764693	920	8.317681	8.544385	8.42262	8.455837	-0.13816	0.295058	0.088548	0.493143	-0.03322	0.794456	0.121766	0.352339	-0.2267	0.10329	-0.10494	0.419511	6764693	8.300749	8.337778	8.314516	8.409809	8.745026	8.478321	8.509343	8.544367	8.214149	8.270188	8.520941	8.576383	Lbr	NM_133815
6764694	921	8.26951	8.348149	8.21637	8.436807	-0.1673	0.278542	-0.08866	0.554982	-0.22044	0.164123	0.131779	0.3866	-0.07864	0.599671	0.05314	0.721521	6764694	8.257797	8.220666	8.330068	8.393706	8.318021	8.332721	8.067361	8.546845	8.034904	8.320719	8.329687	8.660014	Enah	NM_010135
6764705	923	8.803536	9.076071	9.028674	8.928856	-0.12532	0.51067	0.147215	0.44209	0.099818	0.598442	0.047397	0.801166	-0.27253	0.17275	-0.22514	0.251269	6764705	8.890393	8.96184	8.558376	8.904098	9.276031	9.048083	9.198945	9.038791	8.848286	8.709724	9.26312	8.813724	Degs1	NM_007853
6764708	924	7.802702	7.941157	7.976896	7.794171	0.008531	0.911261	0.146986	0.082815	0.182725	0.039093	-0.03574	0.6428	-0.13845	0.098893	-0.17419	0.046778	6764708	7.841588	7.832293	7.734226	7.826828	8.089706	7.906937	7.975491	7.935514	8.019684	7.719205	7.904621	7.758688	Fbxo28	NM_175127
6764721	925	9.550253	9.597272	9.491682	9.15604	0.394213	0.02904	0.441231	0.017833	0.335641	0.053695	0.10559	0.497241	-0.04702	0.759624	0.058572	0.703562	6764721	9.546009	9.493751	9.611	9.576446	9.683257	9.532112	9.773544	9.195522	9.505979	9.07221	9.381973	9.013938	Capn2	NM_009794
6764832	926	7.712665	7.764857	7.770606	7.668106	0.044559	0.814081	0.096751	0.612002	0.1025	0.591399	-0.00575	0.97575	-0.05219	0.783116	-0.05794	0.760057	6764832	7.75274	7.947674	7.437582	7.755634	7.889403	7.649534	7.61574	7.958969	7.73711	7.350861	7.949957	7.703501	Hlx	NM_008250
6764835	928	8.062193	8.389422	8.239022	8.564262	-0.50207	0.052026	-0.17484	0.450004	-0.32524	0.177833	0.1504	0.513797	-0.32723	0.175487	-0.17683	0.445056	6764835	7.908041	8.04965	8.228887	8.407547	8.380261	8.380458	8.536165	8.255951	7.92495	8.317177	8.333826	9.041784	Mosc2	NM_133684
6764838	929	7.157101	7.11285	7.068528	6.942452	0.214649	0.32966	0.170398	0.433845	0.126076	0.559019	0.044322	0.835663	0.044251	0.835923	0.088573	0.679741	6764838	7.100746	7.257662	7.112895	6.970344	6.997588	7.370619	7.340547	6.987945	6.877093	6.612971	6.864314	7.350071	C130074G	NM_178692
6764841	930	6.536333	6.311963	6.309704	6.36551	0.170823	0.359361	-0.05355	0.768304	-0.05581	0.758883	0.002259	0.990054	0.22437	0.237375	0.226629	0.233093	6764841	6.77468	6.453539	6.380781	6.238403	6.578467	6.11902	6.424241	6.335249	6.169622	6.251626	6.181615	6.663289	Mark1	NM_145515
6764854	931	8.55088	8.636524	8.41182	8.710527	-0.15965	0.373659	-0.074	0.673835	-0.29871	0.115928	0.224704	0.221392	-0.08564	0.626864	0.13906	0.435581	6764854	8.534008	8.494457	8.624174	8.737672	8.60079	8.571109	8.59607	8.310213	8.329177	8.469222	8.529984	9.132375	Iars2	NM_198653
6764910	933	7.776963	7.80623	7.820447	7.901323	-0.12436	0.371972	-0.09509	0.490181	-0.08088	0.555615	-0.01422	0.916561	-0.02927	0.82945	-0.04348	0.749374	6764910	7.542113	7.99273	7.796046	7.722738	7.810448	7.885503	7.763515	7.854865	7.842962	7.785644	7.775937	8.142387	Lyplal1	NM_146106
6764953	934	6.077493	6.020864	5.840588	5.627947	0.449545	0.024179	0.392916	0.041602	0.212641	0.226001	0.180276	0.298404	0.056629	0.735858	0.236905	0.18204	6764953	5.948468	6.217152	6.066858	6.033905	6.312728	5.715958	5.995171	5.710318	5.816275	5.540028	5.82635	5.517464	Tgfb2	NM_009367
6765119	937	7.156304	7.160611	7.126443	7.062053	0.094251	0.156356	0.098558	0.140503	0.06439	0.316367	0.034168	0.586185	-0.00431	0.944764	0.029861	0.633477	6765119	7.078676	7.177655	7.21258	7.116555	7.271445	7.093832	7.095382	7.166482	7.117464	6.987934	7.145668	7.052557	Kctd3	NM_172650
6765153	939	7.823451	7.602646	7.580351	7.556632	0.266819	0.077565	0.046014	0.73605	0.023719	0.86168	0.022295	0.869891	0.220805	0.13246	0.2431	0.10239	6765153	7.776542	7.650804	8.043007	7.671435	7.659575	7.476928	7.803833	7.504189	7.43303	7.44736	7.543536	7.678999	Smyd2	NM_026796
6765186	941	6.501183	6.621542	6.502395	6.472646	0.028536	0.605309	0.148895	0.022969	0.029748	0.590352	0.119147	0.054933	-0.12036	0.053009	-0.00121	0.982334	6765186	6.534898	6.512625	6.456025	6.63277	6.705754	6.526101	6.557016	6.45965	6.490518	6.451874	6.5484	6.417665	Rps6kc1	NM_178775
6765199	942	6.530226	6.257552	6.442226	6.22887	0.301355	0.25253	0.028682	0.909464	0.213355	0.408082	-0.18467	0.471507	0.272673	0.296914	0.088	0.728101	6765199	6.671166	6.641702	6.277809	6.234053	6.557327	5.981277	6.625089	6.395089	6.306499	5.795409	6.202693	6.688509	Vash2	NM_144879
6765202	943	8.020466	7.983653	7.958204	8.059562	-0.0391	0.755731	-0.07591	0.549294	-0.10136	0.428102	0.025448	0.839238	0.036813	0.769495	0.062262	0.621984	6765202	7.978922	7.98748	8.094996	7.886731	8.053194	8.011033	7.995555	8.187145	7.691913	8.04462	7.947587	8.186478	Mfsd7b	NM_001081259
6765218	944	8.93745	8.16727	8.275575	7.391436	1.546014	0.001452	0.775834	0.04442	0.884139	0.026481	-0.10831	0.748058	0.77018	0.04564	0.661874	0.076625	6765218	9.365238	8.408254	9.038557	8.182421	8.321084	7.998304	8.663938	7.849076	8.313712	7.537427	7.757843	6.882738	Atf3	NM_007498
6765222	945	8.529923	8.550621	8.26099	8.582769	-0.05285	0.731512	-0.03215	0.834263	-0.32178	0.062422	0.289632	0.087316	-0.2007	0.892747	0.268934	0.108153	6765222	8.600389	8.379712	8.609669	8.59505	8.501324	8.55549	8.558302	8.324422	7.900245	8.606894	8.528036	8.613377	Nenf	NM_025424
6765226	946	9.222818	9.483771	9.319194	9.742064	-0.51925	0.004525	-0.25829	0.088132	-0.42287	0.013023	0.164578	0.251122	-0.26095	0.08545	-0.09638	0.489431	6765226	9.233373	9.271955	9.163126	9.553115	9.362017	9.536182	9.172883	9.525331	9.259367	9.733923	9.504965	9.987304	Ppp2r5a	NM_144880
6765235	947	5.885431	5.948678	5.628278	5.223888	0.661542	0.071965	0.72479	0.05285	0.40439	0.240862	0.3204	0.344916	-0.06325	0.847885	0.257153	0.443778	6765235	5.73493	5.790167	6.131195	5.622338	6.288871	5.934826	6.069465	4.890565	5.924804	5.301145	5.375702	4.994818	Dtl	NM_029766
6765275	948	7.637762	7.571806	7.679405	7.802689	-0.16493	0.3882	-0.23088	0.237298	-0.12328	0.514456	-0.1076	0.568103	0.065956	0.724643	-0.04164	0.823573	6765275	7.266651	7.618107	8.028529	7.504583	7.534231	7.676604	7.652607	7.763673	7.621935	7.602099	7.822823	7.983145	Traf5	NM_011633
6765276	949	6.957986	5.944285	6.815108	7.268442	-0.31046	0.631158	-1.32416	0.06593	-0.45333	0.48693	-0.87082	0.199108	1.013701	0.141837	0.142878	0.8241	6765276	6.950026	6.789757	7.134174	4.366021	6.589595	6.877238	6.523177	7.41342	6.508726	6.975089	7.150344	7.679893	Gstp2	NM_181796
6765281	950	8.24683	8.408579	8.530636	8.40589	-0.15906	0.15857	0.002689	0.97967	0.124746	0.257389	-0.12206	0.266973	-0.16175	0.152482	-0.28381	0.024131	6765281	8.265814	8.324339	8.150337	8.555856	8.418903	8.250978	8.486141	8.632364	8.473402	8.242445	8.549166	8.426058	Rcor3	NM_144814
6765301	951	6.099137	6.162687	5.976566	5.856493	0.242645	0.084622	0.306194	0.037918	0.120073	0.358675	0.186121	0.16962	-0.06355	0.620216	0.122572	0.349302	6765301	6.090752	6.111636	6.095024	5.961996	6.436722	6.089342	6.080408	5.812865	6.036424	5.741447	5.928705	5.899326	Hhat	NM_144881
6765307	953	6.434147	6.524397	6.26483	6.12264	0.311507	0.126119	0.401757	0.058796	0.14219	0.458186	0.259567	0.192604	-0.09025	0.634118	0.169317	0.380515	6765307	6.316629	6.492242	6.493569	6.324834	7.017553	6.230803	6.307244	6.185744	6.301501	6.126981	6.116353	6.124586	Sertad4	NM_198247
6765317	955	6.755165	6.892472	6.780346	6.864825	-0.10966	0.249662	0.027647	0.762327	-0.08448	0.366946	0.112126	0.24005	-0.13731	0.158738	-0.02518	0.782859	6765317	6.687336	6.795807	6.782352	6.763604	7.026954	6.886858	6.694422	6.783245	6.863371	6.840851	7.012449	6.741176	AA408296	NM_145415
6765327	958	7.102295	7.46754	7.441388	7.402104	-0.29981	0.057274	0.065435	0.641151	0.039284	0.778637	0.026151	0.85135	-0.36524	0.026937	-0.33909	0.036387	6765327	7.304557	6.925304	7.077024	7.486018	7.339544	7.577057	7.222391	7.630713	7.471061	7.313239	7.344211	7.548863	G0s2	NM_008059
6765332	959	7.469301	7.852124	8.32303	7.895377	-0.42608	0.368267	-0.04325	0.925273	0.427653	0.366589	-0.47091	0.322761	-0.38282	0.416543	-0.85373	0.092463	6765332	8.080969	7.224839	7.102094	8.413068	7.535115	7.608189	8.188587	9.17759	7.602914	7.672047	7.932345	8.081739	Camk1g	NM_144817
6765443	960	6.017049	5.920325	6.053188	5.981446	0.035603	0.742649	-0.06112	0.575572	0.071743	0.51269	-0.13286	0.240247	0.096724	0.382734	-0.03614	0.738942	6765443	6.039326	6.00237	6.009451	5.911745	5.917483	5.931746	6.219602	6.180669	5.759294	5.977946	5.995562	5.970829	A330023F	AK030377
6765459	962	8.393991	8.306912	8.360834	8.426122	-0.03213	0.714932	-0.11921	0.19788	-0.06529	0.463979	-0.05392	0.543066	0.08708	0.335043	0.033157	0.706322	6765459	8.549368	8.433952	8.198654	8.231457	8.303389	8.385889	8.400628	8.355114	8.326761	8.356709	8.44283	8.478827	Cr1l	NM_013499
6765474	964	7.084401	7.130592	7.129638	7.370176	-0.28578	0.01767	-0.23958	0.037164	-0.24054	0.036593	0.000954	0.992316	-0.04619	0.643226	-0.04524	0.650002	6765474	7.032057	7.142104	7.079041	7.166085	7.157283	7.068407	7.088641	7.237172	7.063101	7.269811	7.238934	7.601784	Cnksr3	NM_172546
6765513	966	6.984806	7.020653	7.024566	7.065388	-0.08058	0.477063	-0.04473	0.689685	-0.04082	0.715354	-0.00391	0.971994	-0.03585	0.748552	-0.03976	0.722391	6765513	6.795708	7.020649	7.138061	6.957758	7.101749	7.002451	7.000331	7.154428	6.918938	6.949952	7.22325	7.022961	Mtrf1l	NM_175374
6765519	967	6.156339	6.563398	6.216657	5.726209	0.43013	0.091437	0.837189	0.005766	0.490449	0.060236	0.34674	0.160707	-0.40706	0.107093	-0.06032	0.794792	6765519	6.206278	6.150518	6.112221	6.297077	7.084784	6.308332	6.520127	5.907377	6.222468	5.680247	5.734433	5.763946	Fbxo5	NM_025995
6765533	968	6.609104	6.390383	6.786641	7.049315	-0.44021	0.10424	-0.65893	0.025333	-0.26267	0.306029	-0.39626	0.137651	0.218721	0.38918	-0.17754	0.481002	6765533	6.670001	6.771387	6.385924	6.768605	6.001247	6.401297	6.731517	6.932342	6.696064	6.615053	7.22964	7.303252	Syne1	NM_153399
6765551	969	7.694041	7.528674	7.544512	7.602183	0.091858	0.550576	-0.07351	0.631469	-0.05767	0.705873	-0.01584	0.917093	0.165367	0.294528	0.149529	0.340146	6765551	7.672984	7.838735	7.570405	7.771076	7.527876	7.287071	7.537668	7.608538	7.487331	7.346328	7.756634	7.703587	Syne1	NM_001079686
6765596	970	8.622553	8.709818	8.835425	8.646837	-0.02428	0.833341	0.062981	0.588326	0.188588	0.129831	-0.12561	0.293418	-0.08726	0.457165	-0.21287	0.093147	6765596	8.508169	8.634037	8.725453	8.573125	8.648949	8.907345	8.877332	8.911482	8.717461	8.551656	8.815834	8.573021	Rmnd1	NM_025343
6765719	972	6.320604	6.441654	6.208451	6.218076	0.102528	0.64134	0.223578	0.322031	-0.00962	0.964871	0.233203	0.302947	-0.12105	0.583378	0.112153	0.610857	6765719	6.3781	6.269178	6.314533	5.966391	6.912706	6.445864	6.369243	6.26453	5.991579	6.253434	6.142284	6.258509	Nup43	NM_145706
6765723	973	8.389683	8.677476	8.386063	8.262271	0.127412	0.366731	0.415205	0.014279	0.123793	0.379811	0.291412	0.060096	-0.28779	0.062696	0.00362	0.978983	6765723	8.42721	8.399759	8.34208	8.390783	8.977107	8.664537	8.426412	8.494384	8.237394	8.262279	8.235247	8.289286	Katna1	NM_011835
6765730	974	8.32566	8.278783	8.011227	8.010058	0.315602	0.146703	0.268725	0.208385	0.001169	0.995395	0.267556	0.210179	0.046877	0.817336	0.314433	0.148015	6765730	8.53989	8.224513	8.212576	7.861467	8.690194	8.284687	8.087759	8.106234	7.839688	7.94991	8.05875	8.021513	Ppil4	NM_026141
6765734	975	6.412418	6.050435	6.205116	5.907786	0.504632	0.014792	0.142649	0.407226	0.29733	0.105734	-0.15468	0.370649	0.361983	0.057215	0.207301	0.239389	6765734	6.773217	6.404537	6.059499	6.089807	6.048708	6.01279	6.395928	6.103041	6.11638	5.870547	5.878409	5.974402	Zc3h12d	NM_172785
6765971	976	7.269822	7.369297	7.318712	7.25291	0.016912	0.754681	0.116387	0.056685	0.065802	0.243765	0.050585	0.361724	-0.09947	0.093651	-0.04889	0.3772	6765971	7.230589	7.341013	7.237865	7.361879	7.386861	7.359151	7.369637	7.28267	7.30383	7.167371	7.365079	7.22628	Shprh	NM_001077707
6765973	977	8.601629	8.641847	8.521762	8.538668	0.062961	0.566999	0.103179	0.356535	-0.01691	0.876613	0.120085	0.287775	-0.04022	0.712859	0.079867	0.470566	6765973	8.44807	8.557792	8.799025	8.556433	8.677137	8.691972	8.523821	8.564425	8.477041	8.365385	8.557965	8.692654	Fbxo30	NM_027968
6766038	979	8.477326	8.30607	8.021107	8.059995	0.417331	0.073858	0.246075	0.260057	-0.03889	0.852867	0.284963	0.198031	0.171256	0.423421	0.456219	0.054806	6766038	8.612763	8.371347	8.447868	8.082959	8.777937	8.057314	8.293281	7.833879	7.936161	8.076732	7.970813	8.13244	Sf3b5	NM_175102
6766046	980	5.952055	6.026069	5.997365	6.256111	-0.30406	0.271354	-0.23004	0.397492	-0.25875	0.344148	0.028704	0.913942	-0.07401	0.780971	-0.04531	0.864624	6766046	5.681813	6.22008	5.954272	6.035239	6.08411	5.958857	5.871195	6.17793	5.94297	6.028152	6.876318	5.863862	Plagl1	NM_009538
6766060	981	9.219867	9.151626	8.980182	8.902402	0.317465	0.030166	0.249224	0.072807	0.07778	0.537339	0.171445	0.193255	0.068241	0.58731	0.239686	0.082301	6766060	9.295026	9.121819	9.242757	9.031082	9.33515	9.088647	9.076552	8.883816	8.980177	8.745745	8.820319	9.141142	Fuca2	NM_025799
6766063	982	6.842086	6.811728	6.567907	6.616544	0.225542	0.21246	0.195184	0.274708	-0.04864	0.77758	0.243821	0.181156	0.030358	0.859825	0.274179	0.138152	6766063	6.929636	6.687547	6.909076	6.731154	7.13591	6.56812	6.777679	6.425634	6.500408	6.427747	6.670458	6.751428	Adat2	NM_025748
6766110	983	8.464682	8.698625	8.678251	8.626028	-0.16135	0.060317	0.072597	0.354174	0.052223	0.499357	0.020374	0.78954	-0.23394	0.013209	-0.21357	0.020099	6766110	8.55329	8.449709	8.391048	8.678926	8.86297	8.553979	8.682122	8.649233	8.703399	8.632464	8.654567	8.591053	Hivep2	NM_010437
6766240	985	9.811666	9.796791	9.956703	9.704476	0.10719	0.495509	0.092315	0.555676	0.252228	0.131433	-0.15991	0.317861	0.014875	0.923505	-0.14504	0.362266	6766240	9.812113	9.760359	9.862526	9.719476	10.08043	9.590466	9.745021	10.16004	9.965049	9.712354	9.858484	9.542589	Cited2	NM_010828
6766250	986	6.837262	6.856746	6.801457	6.740257	0.097005	0.89388	0.116488	0.872765	0.0612	0.932907	0.055289	0.939371	-0.01948	0.978613	0.035805	0.96071	6766250	5.721589	6.461635	8.328562	7.70756	6.541761	6.320916	7.278822	6.684397	6.441152	5.987757	7.201531	7.031484	Txlnb	NM_138628
6766258	987	8.830346	8.816676	8.853993	8.970545	-0.1402	0.221957	-0.15387	0.184151	-0.11655	0.302913	-0.03732	0.733557	0.01367	0.900437	-0.02365	0.828837	6766258	8.890512	8.788252	8.812274	8.914087	8.82818	8.70776	8.748199	9.034867	8.778913	8.92791	8.826031	9.157695	Reps1	NM_009048
6766287	988	7.706209	8.103476	8.000447	7.902619	-0.19641	0.041358	0.200857	0.037961	0.097828	0.261135	0.103029	0.238624	-0.39727	0.001179	-0.29424	0.006621	6766287	7.615659	7.840633	7.662334	8.099911	8.142779	8.067738	8.006086	7.974593	8.020661	7.803676	7.826132	8.078049	Nhsl1	NM_173390
6766301	989	9.922978	9.283926	9.333115	9.535979	0.386999	0.444191	-0.25205	0.61437	-0.20286	0.684225	-0.04919	0.921041	0.639052	0.22051	0.589863	0.254819	6766301	10.1276	9.637445	10.00389	9.36038	9.754204	8.737195	9.529075	9.32553	9.144741	9.211013	10.66968	8.727244	Perp	NM_022032
6766350	991	8.749021	8.656243	8.673184	8.194035	0.554986	0.036872	0.462208	0.070775	0.479149	0.062845	-0.01694	0.941017	0.092778	0.686849	0.075837	0.741319	6766350	9.028149	8.616381	8.602533	8.561168	8.882628	8.524934	9.046969	8.34631	8.626273	8.227666	8.447265	7.907174	Ifngr1	NM_010511
6766368	995	8.563538	8.608014	8.65261	8.932778	-0.36924	0.09401	-0.32476	0.13329	-0.28017	0.187434	-0.0446	0.824281	-0.04448	0.824745	-0.08907	0.65895	6766368	8.485874	8.640167	8.564572	8.616894	8.44249	8.764658	8.562716	8.905148	8.489967	8.884109	8.577929	9.336297	Map3k5	NM_008580
6766372	996	8.234121	8.2825	8.289732	8.329161	-0.09504	0.207642	-0.04666	0.519884	-0.03943	0.585151	-0.00723	0.919483	-0.04838	0.505065	-0.05561	0.445646	6766372	8.211375	8.181448	8.309541	8.260617	8.292	8.294882	8.266436	8.427166	8.175594	8.240667	8.329994	8.416821	Mtap7	NM_008635
6766380	997	8.86914	9.09325	8.938586	8.994837	-0.1257	0.108437	0.098413	0.194923	-0.05625	0.44217	0.154664	0.05691	-0.22411	0.012216	-0.06945	0.347424	6766380	8.917438	8.885267	8.804716	9.004768	9.189399	9.085582	9.047431	8.844926	8.923401	8.948514	8.946796	9.0892	Bclaf1	NM_001025392
6766409	999	6.370708	6.620823	6.611951	6.706795	-0.33609	0.011617	-0.08597	0.429226	-0.09484	0.385205	0.008872	0.933641	-0.25011	0.041705	-0.24124	0.04769	6766409	6.363178	6.396255	6.352692	6.63383	6.716757	6.511882	6.59122	6.77389	6.470743	6.640892	6.901864	6.57763	Ahi1	NM_026203
6766422	1000	7.610687	7.754787	7.640559	7.505543	0.105144	0.354029	0.249244	0.048005	0.135016	0.242053	0.114228	0.316349	-0.1441	0.214491	-0.02987	0.78695	6766422	7.574098	7.646129	7.611835	7.6504	7.901463	7.712499	7.828537	7.447373	7.645768	7.394743	7.628676	7.493211	Hbs1l	NM_019702
6766455	1003	7.630483	7.86064	7.518656	7.802785	-0.1723	0.289737	0.057855	0.713348	-0.28413	0.098476	0.341985	0.054548	-0.23016	0.168384	0.111828	0.482856	6766455	7.657529	7.713471	7.52045	8.151444	7.662326	7.768151	7.786122	7.301851	7.467994	7.851753	7.790254	7.766348	Sgk1	NM_011361
6766491	1005	6.000719	6.7612	6.13518	6.039695	-0.03898	0.854554	0.721505	0.008024	0.095485	0.655143	0.62602	0.016043	-0.76048	0.006095	-0.13446	0.531987	6766491	5.768109	5.891335	6.342712	6.556414	7.149669	6.577516	6.278623	6.125238	6.001678	6.030914	6.217811	5.87036	Slc2a12	NM_178934

6766585	1012	6.342699	6.147782	6.229071	6.155441	0.187258	0.195395	-0.00766	0.955337	0.073631	0.593711	-0.08129	0.556688	0.194917	0.179587	0.113628	0.416207	6766585	6.292555	6.467405	6.268137	6.278355	6.006264	6.158727	6.253597	6.444419	5.989198	5.999197	6.167088	6.300037	Taar5	NM_001009574
6766586	1013	7.958356	7.632113	7.56112	7.921334	0.037022	0.852842	-0.28922	0.172851	-0.36021	0.099313	0.070992	0.72287	0.326243	0.12984	0.397336	0.073861	6766586	8.012877	7.844969	8.017222	7.729414	7.576614	7.59031	7.533981	7.978762	7.170618	7.8867	8.144897	7.732404	Taar7b	NM_001010827
6766595	1017	9.175151	9.256785	9.312989	9.183285	-0.00813	0.920198	0.0735	0.377471	0.129704	0.1378	-0.0562	0.495256	-0.08163	0.329743	-0.13784	0.117839	6766595	9.136094	9.131213	9.258147	9.255271	9.315304	9.19978	9.413289	9.324037	9.201641	9.046988	9.193151	9.309715	Stx7	NM_016797
6766605	1018	9.893915	10.76113	10.59362	11.41641	-1.52249	0.000703	-0.65528	0.050944	-0.82279	0.020498	0.16751	0.573762	-0.69971	0.016162	-0.69971	0.039965	6766605	9.213235	10.41847	10.05004	10.72025	10.76834	10.7948	10.59063	10.88861	10.30162	11.58157	11.3236	11.34406	Moxd1	NM_021509
6766623	1019	12.47025	13.01553	12.22877	13.03011	-0.55986	0.010142	-0.01459	0.932675	-0.80135	0.001375	0.78676	0.001538	-0.54527	0.011549	0.241487	0.186953	6766623	12.06375	12.60741	12.7396	13.05456	12.95038	13.04164	12.12662	12.35203	12.20765	12.96727	12.91848	13.20459	Ctgf	NM_010217
6766649	1020	8.360848	8.406015	8.442297	8.278581	0.082267	0.283019	0.127434	0.11248	0.163717	0.05124	-0.03628	0.625445	-0.04517	0.545105	-0.08145	0.287485	6766649	8.310858	8.418449	8.353236	8.33363	8.55728	8.327134	8.540638	8.370106	8.416148	8.218718	8.317376	8.299648	Med23	NM_027347
6766705	1021	8.451588	8.421797	8.221094	7.645567	0.806021	0.016195	0.77623	0.019219	0.575527	0.062126	0.200703	0.471529	0.029791	0.91346	0.230494	0.410781	6766705	8.595218	8.449989	8.309556	8.371305	8.615778	8.278308	8.58701	7.777157	8.299114	7.219204	8.118813	7.598683	Epb4.1l2	NM_013511
6766772	1022	8.710064	8.504587	8.67868	8.449258	0.260806	0.18221	0.055329	0.76456	0.229422	0.234749	-0.17409	0.358072	0.205477	0.283019	0.031384	0.864835	6766772	8.879908	8.553495	8.696789	8.446437	8.60538	8.461943	8.842081	8.482858	8.711102	8.085065	8.475517	8.787192	Arhgap18	NM_176837
6766835	1023	8.457087	8.732945	8.61903	8.575216	-0.11813	0.358054	0.157729	0.229146	0.043814	0.726963	0.113915	0.374562	-0.27586	0.052303	-0.16194	0.218052	6766835	8.532391	8.506186	8.332683	8.689051	8.847391	8.662392	8.855746	8.449623	8.55172	8.40862	8.629805	8.687223	Ptprk	NM_008983
6766863	1026	8.102534	8.310721	8.594314	8.583394	-0.48086	0.031361	-0.27267	0.177841	0.01092	0.954273	-0.28359	0.162973	-0.20819	0.292035	-0.49178	0.028609	6766863	8.043406	8.289965	7.974232	8.190619	8.351126	8.390419	8.651546	8.817827	8.31357	8.249257	8.624393	8.876533	Echdc1	NM_025855
6767057	1027	6.659826	6.602935	6.523541	6.350076	0.30975	0.780626	0.252859	0.82	0.173465	0.875844	0.079394	0.942956	0.056891	0.959104	0.136285	0.902274	6767057	4.84039	6.292599	8.846489	7.628736	6.756129	5.42394	7.154997	6.361996	6.05363	5.057785	6.86879	7.123652	Trdn	NM_029726
6767084	1029	8.558539	8.635924	8.625247	8.633517	-0.07498	0.47761	0.002407	0.981509	-0.00827	0.936532	0.010677	0.918131	-0.07738	0.464063	-0.06671	0.526097	6767084	8.537242	8.592103	8.546272	8.568905	8.693004	8.645862	8.67022	8.770888	8.434633	8.548882	8.531495	8.820174	Tspyl1	NM_009433
6767088	1030	6.681519	6.768187	7.020633	6.977933	-0.29641	0.089335	-0.20975	0.2086	0.042699	0.787747	-0.25245	0.138362	-0.08667	0.587481	-0.33911	0.057971	6767088	6.834958	6.650599	6.559001	6.885932	6.721102	6.697528	7.168339	6.939309	6.95425	6.760467	6.843897	7.329436	Tspyl4	NM_030203
6767089	1031	6.152179	7.084663	5.633211	6.040394	0.111785	0.872172	1.044268	0.159264	-0.40718	0.561843	1.451452	0.063068	-0.93248	0.203195	0.518968	0.462703	6767089	6.328126	5.745012	6.3834	5.915322	7.741574	7.597092	6.569593	6.082963	4.247077	6.186616	6.17775	5.756817	Col10a1	NM_009925
6767094	1032	8.861245	8.793484	8.872053	8.692421	0.168824	0.327485	0.101063	0.549811	0.179631	0.299386	-0.07857	0.640441	0.067761	0.686531	-0.01081	0.948408	6767094	8.881475	8.836275	8.865985	8.574523	9.125133	8.680797	8.995634	8.679283	8.941241	8.454117	8.79796	8.825187	Frk	NM_001159544
6767155	1033	8.680036	8.794275	8.82432	8.849309	-0.16927	0.214402	-0.05503	0.672655	-0.02499	0.847163	-0.03005	0.816837	-0.11424	0.389352	-0.14428	0.283553	6767155	8.611849	8.671086	8.757172	8.620887	8.953693	8.808244	9.046215	8.833171	8.593575	8.823889	8.764088	8.959951	Hdac2	NM_008229
6767231	1035	8.392966	8.34777	8.033095	7.848671	0.544294	0.130163	0.499098	0.160555	0.184423	0.583373	0.314675	0.358077	0.045196	0.892083	0.359871	0.297168	6767231	8.435616	8.474109	8.269172	8.058027	8.659624	8.325658	8.445365	7.549675	8.104244	7.216726	8.299858	8.02943	Lama4	NM_010681
6767258	1037	8.271691	8.346399	8.138973	8.034737	0.236954	0.309134	0.311662	0.191048	0.104235	0.645636	0.207427	0.369622	-0.07471	0.740881	0.132718	0.559892	6767258	8.351724	8.265711	8.197638	8.239924	8.587436	8.211838	8.206126	7.882328	8.328464	7.648359	8.494049	7.961804	Fyn	NM_001122893
6767270	1038	6.572825	6.555446	6.489672	6.504054	0.068771	0.66148	0.051392	0.742817	-0.01438	0.926596	0.065774	0.675207	0.017379	0.911369	0.083154	0.597552	6767270	6.45742	6.672004	6.589052	6.443151	6.760222	6.462965	6.272071	6.608948	6.587996	6.548643	6.720385	6.243134	Traf3ip2	NM_134000
6767292	1039	7.834662	7.825032	7.910377	7.76585	0.068812	0.232404	0.059182	0.298738	0.144527	0.026503	-0.08534	0.147708	0.00963	0.861004	-0.07572	0.192905	6767292	7.899858	7.783249	7.820879	7.836074	7.838408	7.800614	7.920999	7.948477	7.861655	7.654127	7.780523	7.862901	Rev3l	NM_011264
6767336	1040	8.960283	9.080634	8.886993	8.787505	0.172779	0.236493	0.293129	0.061705	0.099488	0.482232	0.193641	0.189401	-0.12035	0.398718	0.073291	0.602019	6767336	8.8787	8.906349	9.095801	9.069244	9.225044	8.947613	9.123485	8.736664	8.800829	8.809618	8.595568	8.957328	Cdc2l6	NM_198164
6767345	1041	7.367611	7.257494	7.292765	7.596682	-0.22907	0.214757	-0.33919	0.081123	-0.30392	0.111633	-0.03527	0.84083	0.110117	0.535306	0.074846	0.671403	6767345	7.212735	7.500678	7.38942	7.213893	7.13355	7.42504	6.937369	7.396939	7.543986	7.405893	7.644918	7.739235	Ddo	NM_027442
6767384	1044	6.502286	6.727097	6.602124	6.653716	-0.15143	0.234779	0.073381	0.550848	-0.05159	0.673137	0.124973	0.31992	-0.22481	0.092882	-0.09984	0.421538	6767384	6.371045	6.721373	6.414439	6.568519	6.707427	6.905345	6.636547	6.527202	6.642622	6.65338	6.535585	6.772182	Zbtb24	NM_153398
6767385	1045	7.503525	7.677171	7.513652	7.490806	0.012719	0.950754	0.186365	0.377788	0.022846	0.911696	0.163519	0.436382	-0.17365	0.409666	-0.01013	0.960781	6767385	7.490382	7.501206	7.518987	7.432632	7.953083	7.645798	7.850942	7.060073	7.62994	7.510698	7.421057	7.540662	Mical1	NM_138315
6767387	1046	11.57006	11.53654	11.54486	11.60778	-0.03772	0.705864	-0.07124	0.481176	-0.06292	0.532403	-0.00832	0.933364	0.033513	0.737153	0.025193	0.800493	6767387	11.63678	11.48453	11.5888	11.52978	11.654	11.42585	11.6338	11.58936	11.41143	11.57602	11.47507	11.77225	Cd164	NM_016898
6767402	1047	8.909916	9.036779	8.996656	8.851988	0.057928	0.64193	0.184791	0.161821	0.144668	0.262048	0.040124	0.746491	-0.12686	0.320905	-0.08674	0.490009	6767402	9.090955	8.938304	8.70049	8.936217	9.126947	9.047174	9.027769	9.024297	8.937901	8.645284	8.894372	9.016308	Sesn1	NM_001013370
6767460	1048	9.566703	9.423324	9.62344	9.54912	0.017583	0.863022	-0.1258	0.238202	0.07432	0.472983	-0.20012	0.077127	0.143379	0.18434	-0.05674	0.581157	6767460	9.502161	9.593792	9.604155	9.304936	9.522152	9.442883	9.726707	9.620518	9.523095	9.339959	9.649013	9.658387	Snx3	NM_017472
6767463	1049	8.578052	8.667127	8.563942	8.564909	0.013143	0.832588	0.102218	0.12784	-0.00097	0.987582	0.103184	0.124769	-0.08907	0.177111	0.01411	0.820521	6767463	8.513094	8.569021	8.652041	8.591854	8.686724	8.722802	8.633363	8.602946	8.455518	8.501726	8.58148	8.61152	Ostm1	NM_172416
6767464	1050	9.353656	9.422774	9.46045	9.536832	-0.18318	0.014918	-0.11406	0.090659	-0.07638	0.23376	-0.03768	0.54298	-0.06912	0.277399	-0.10679	0.109426	6767464	9.378833	9.402288	9.279847	9.380136	9.370744	9.517442	9.435019	9.530826	9.415504	9.556449	9.604722	9.449326	Sec63	NM_153055
6767468	1051	6.41463	6.467662	6.387953	6.438482	-0.02385	0.732812	0.02918	0.676775	-0.05053	0.475312	0.079709	0.271327	-0.05303	0.454468	0.026677	0.702861	6767468	6.372281	6.439038	6.432572	6.361768	6.630342	6.410877	6.414513	6.39394	6.355407	6.516427	6.392878	6.406141	Scml4	NM_172938
6767519	1053	6.966476	6.946657	6.771001	6.969242	-0.00277	0.978906	-0.02258	0.829309	-0.19824	0.086288	0.175656	0.121419	0.019819	0.849894	0.195475	0.09	6767519	7.023155	6.942901	6.933373	7.169586	6.924817	6.745569	6.663425	6.85527	6.794308	6.938559	6.924369	7.044798	Bend3	NM_199028
6767537	1054	12.98424	13.06395	13.07672	13.09302	-0.10878	0.195712	-0.02907	0.715751	-0.0163	0.83772	-0.01277	0.872481	-0.07971	0.331184	-0.09248	0.264403	6767537	13.00155	13.01888	12.9323	13.05363	13.07557	13.06265	13.09067	13.13097	13.00852	12.90273	13.1397	13.23664	Cd24a	NM_009846
6767563	1055	7.223204	7.135875	7.343417	7.295012	-0.07181	0.641368	-0.15914	0.314749	0.048405	0.75261	-0.20754	0.199421	0.087329	0.572377	-0.12021	0.441242	6767563	7.239431	7.197929	7.232251	7.179741	7.277101	6.950783	7.267306	7.509683	7.253263	7.075637	7.188716	7.620684	Rtn4ip1	NM_130892
6767581	1056	6.947055	6.931073	6.911236	6.881418	0.065637	0.456876	0.049655	0.570584	0.029818	0.731671	0.019837	0.819166	0.015982	0.853778	0.035819	0.680911	6767581	6.973282	7.012504	6.855378	6.896642	7.074754	6.821822	6.925056	6.98264	6.826011	6.911825	6.974644	6.757785	Atg5	NM_053069
6767610	1057	8.944716	8.986201	8.958086	8.857104	0.087611	0.670124	0.129096	0.533024	0.100981	0.62411	0.028115	0.890688	-0.04148	0.839415	-0.01337	0.947865	6767610	9.028073	8.804605	9.001469	8.922727	9.329251	8.706624	9.167542	8.912049	8.794666	8.550694	8.891875	9.128744	Prep	NM_011156
6767631	1059	6.601442	6.695911	6.645592	6.519502	0.081941	0.545825	0.176409	0.211572	0.12609	0.360208	0.050319	0.708629	-0.09447	0.487875	-0.04415	0.742739	6767631	6.509336	6.669538	6.625453	6.628606	6.894413	6.564713	6.747265	6.514191	6.67532	6.413721	6.775411	6.369373	Hace1	NM_172473
6767636	1060	11.23749	11.41494	11.12465	11.28012	-0.04263	0.800975	0.134817	0.433756	-0.15547	0.369761	0.290283	0.11391	-0.17745	0.309648	0.112833	0.509811	6767636	11.35914	11.14738	11.20594	11.6061	11.1872	11.45151	11.25623	11.21843	10.8993	11.27599	11.02668	11.53769	Gpx4	NM_008162
6767760	1061	7.684782	7.57081	7.792023	7.613134	0.071649	0.609364	-0.04232	0.761487	0.17889	0.220949	-0.22121	0.139286	0.113972	0.422235	-0.10724	0.449083	6767760	7.683686	7.72294	7.647721	7.450654	7.805023	7.456754	7.889247	7.756115	7.730708	7.634769	7.844753	7.359879	Ascc3	NM_001146089
6767782	1063	11.33162	11.35278	11.2864	10.00402	1.327598	0.017592	1.348755	0.016364	1.282378	0.02055	0.066377	0.885235	-0.02116	0.963284	0.04522	0.921641	6767782	11.95303	11.33471	10.70712	11.24679	11.50567	11.30587	11.61955	10.58736	11.65211	10.05632	10.62246	9.333286	Lilrb4	NM_013532
6767825	1065	6.603936	6.525761	6.473973	6.409728	0.194208	0.275943	0.116033	0.504591	0.064245	0.708986	0.051788	0.763157	0.078175	0.650438	0.129963	0.456464	6767825	6.388381	6.594714	6.828712	6.532073	6.451741	6.593469	6.430578	6.779051	6.212289	6.542915	6.213111	6.473158	Vgll2	NM_153786
6767828	1066	7.157601	7.179881	6.958924	6.890341	0.26726	0.136198	0.28954	0.11045	0.068583	0.681967	0.220957	0.20803	-0.02228	0.893575	0.198677	0.253119	6767828	7.165259	7.309884	6.997661	7.177862	7.255551	7.106231	7.072459	6.90134	6.902974	6.543639	7.226048	6.901337	Dcbld1	NM_025705
6767845	1068	8.901476	9.026942	8.982479	9.071767	-0.17029	0.094771	-0.04482	0.63143	-0.08929	0.349643	0.044463	0.634144	-0.12547	0.200294	-0.081	0.393828	6767845	8.904944	8.915058	8.884425	8.986017	9.128496	8.966313	9.011069	9.14567	8.790697	8.977592	9.159325	9.078384	Nus1	NM_030250
6767886	1071	7.130989	7.319649	7.220245	7.317698	-0.18671	0.084804	0.001951	0.984109	-0.09745	0.334732	0.099404	0.325719	-0.18866	0.08215	-0.08926	0.374693	6767886	7.1267	7.160771	7.105495	7.289228	7.407226	7.262493	7.409325	7.207057	7.044353	7.206981	7.304104	7.442009	Asf1a	NM_025541
6768005	1072	6.360148	6.271035	6.287734	6.277887	0.082261	0.288177	-0.00685	0.926836	0.009847	0.895042	-0.0167	0.823157	0.089112	0.252773	0.072414	0.345911	6768005	6.31904	6.469282	6.292121	6.197723	6.321648	6.293735	6.390176	6.157375	6.31565	6.270347	6.219588	6.343726	Msl3l2	NR_003618
6768014	1073	9.725312	9.63162	9.406232	8.94209	0.783222	0.161638	0.68953	0.211654	0.464142	0.387525	0.225388	0.66898	0.093692	0.85824	0.31908	0.547374	6768014	9.712654	9.793434	9.669849	9.379415	10.1134	9.402045	9.992858	8.56144	9.664398	9.078597	9.764634	7.98304	Gja1	NM_010288
6768065	1074	6.827991	7.049615	6.911718	6.83291	-0.00492	0.953575	0.216704	0.029426	0.078808	0.364053	0.137897	0.130693	-0.22162	0.02681	-0.08373	0.3365	6768065	6.879342	6.809633	6.794999	7.019113	7.089927	7.039804	7.055748	6.909146	6.77026	6.77908	6.740138	6.979513	Hsf2	NM_008297
6768076	1077	8.601501	8.712542	8.628839	8.893874	-0.29237	0.121111	-0.18133	0.313493	-0.26503	0.154589	0.083702	0.632915	-0.11104	0.528631	-0.02734	0.875204	6768076	8.62195	8.698899	8.483653	8.733545	8.724501	8.679579	8.996906	8.563498	8.326114	8.950314	8.666326	9.064981	Smpdl3a	NM_020561
6768082	1078	8.290357	8.42266	8.391684	8.26451	0.025848	0.763533	0.158151	0.093292	0.127174	0.164151	0.030976	0.718792	-0.1323	0.149738	-0.10133	0.257089	6768082	8.180324	8.297913	8.392835	8.368975	8.553431	8.345575	8.410625	8.420893	8.343534	8.123364	8.316009	8.354156	Gcc2	NM_027375
6768089	1079	7.800225	7.909786	7.825864	7.779767	0.020458	0.80582	0.130019	0.144986	0.046097	0.582666	0.083921	0.327711	-0.10956	0.210661	-0.02564	0.758292	6768089	7.730235	7.806884	7.863557	7.827356	8.057653	7.844348	7.880813	7.76101	7.83577	7.643558	7.867021	7.828722	Gcc2	NM_027375
6768092	1080	9.38067	9.747829	9.519948	9.522265	-0.14159	0.148767	0.225565	0.034428	-0.00232	0.979782	0.227882	0.033056	-0.36716	0.00324	-0.13928	0.154686	6768092	9.288801	9.441685	9.411523	9.645686	9.813965	9.783837	9.586453	9.572784	9.400606	9.385769	9.502128	9.678897	Lims1	NM_026148
6768094	1081	9.308774	9.338274	9.444176	9.445523	-0.13675	0.198031	-0.10725	0.30298	-0.00135	0.989307	-0.1059	0.30869	-0.0295	0.769772	-0.1354	0.202037	6768094	9.379238	9.343864	9.203221	9.211095	9.444951	9.358775	9.448345	9.59507	9.289113	9.429321	9.556969	9.350279	Ranbp2	NM_011240
6768114	1083	7.817722	7.858624	7.986617	7.895682	-0.07796	0.573018	-0.03706	0.787097	0.090935	0.512469	-0.12799	0.362954	-0.0409	0.765742	-0.16889	0.238774	6768114	7.587673	7.960105	7.905388	7.638816	7.803684	8.133373	8.019354	7.957209	7.983288	7.87982	7.92826	7.878965	Ankrd57	NM_172939
6768123	1084	8.380013	8.462478	8.421403	8.65803	-0.27802	0.025885	-0.19555	0.091179	-0.23663	0.048711	0.041075	0.697367	-0.08247	0.441649	-0.04139	0.695184	6768123	8.303297	8.475938	8.360803	8.241893	8.533567	8.611973	8.546652	8.319244	8.398312	8.716084	8.603394	8.654612	P4ha1	NM_011030
6768143	1086	7.777619	7.880174	7.779291	7.877139	-0.09952	0.404145	0.003036	0.979225	-0.09785	0.411743	0.100883	0.398026	-0.10256	0.390611	-0.00167	0.988554	6768143	7.7535	7.695408	7.883949	7.830127	7.990982	7.819414	7.886955	7.696338	7.754581	7.625506	7.97105	8.03486	Cbara1	NM_144822
6768145	1087	8.514691	8.586406	8.454556	8.597899	-0.08321	0.142695	-0.01149	0.827979	-0.14334	0.023184	0.13185	0.032827	-0.07172	0.198778	0.060135	0.273852	6768145	8.469801	8.508191	8.56608	8.612372	8.605356	8.54149	8.452796	8.534071	8.3768	8.559443	8.549664	8.684589	Dnajb12	NM_019965
6768150	1088	7.05297	7.103727	7.158479	7.215622	-0.16265	0.043901	-0.11189	0.138856	-0.05714	0.4256	-0.05475	0.444468	-0.05076	0.477234	-0.10551	0.159758	6768150	6.927698	7.131135	7.100077	7.10404	7.105709	7.101432	7.148987	7.254854	7.071595	7.183136	7.151033	7.312697	Ascc1	NM_026937
6768155	1090	12.60026	12.49417	12.67301	12.63501	-0.03475	0.695957	-0.14084	0.139147	0.038003	0.669385	-0.17884	0.07051	0.10609	0.251115	-0.07275	0.420897	6768155	12.71688	12.50278	12.58113	12.41928	12.49484	12.5684	12.84947	12.58219	12.58738	12.6586	12.56797	12.67846	Psap	NM_001146120
6768157	1091	7.722069	7.639857	7.70157	7.0945	0.627569	0.006477	0.545357	0.013127	0.60707	0.007703	-0.06171	0.728809	0.082212	0.645173	0.020499	0.907992	6768157	7.781516	7.79815	7.58654	7.652997	7.83826	7.428313	7.771112	7.695126	7.638471	7.058011	7.453009	6.772479	4632428N	NM_028732
6768198	1092	7.735117	8.034534	7.618807	7.57571	0.159407	0.560575	0.458823	0.118663	0.043097	0.873683	0.415726	0.151975	-0.29942	0.28709	0.11631	0.669491	6768198	7.477082	7.601805	8.126465	7.578637	8.062154	8.46281	7.650703	7.503657	7.702062	7.234219	7.719254	7.773658	Pcbd1	NM_025273
6768217	1095	6.193084	6.279486	6.279591	6.271287	-0.0782	0.474015	0.008199	0.939155	0.008304	0.938375	-0.00011	0.999217	-0.0864	0.430597	-0.08651	0.430057	6768217	6.302139	6.138644	6.138469	6.391715	6.065516	6.381226	6.341022	6.290757	6.206994	6.236231	6.161438	6.416191	Nodal	NM_013611
6768227	1096	8.202372	8.12365	8.171978	8.167039	0.035333	0.868385	-0.04339	0.838825	0.004939	0.981502	-0.04833	0.82083	0.078722	0.712955	0.030394	0.886624	6768227	8.024977	8.119084	8.463055	8.317584	8.066132	7.987235	8.042358	8.44319	8.030387	7.834305	8.519558	8.147254	Lrrc20	NM_153542

6768232	1097	8.573345	8.48825	8.538386	8.620929	-0.04758	0.786922	-0.13268	0.458155	-0.08254	0.64074	-0.05014	0.775848	0.085095	0.630602	0.034959	0.842414	6768232	8.590896	8.50196	8.627179	8.291867	8.646575	8.526307	8.90836	8.426707	8.280092	8.592979	8.803858	8.465951	Ppa1	NM_026438
6768234	1098	9.852396	9.946567	9.976936	9.951176	-0.09878	0.44445	-0.00461	0.970981	0.025759	0.839107	-0.03037	0.810919	-0.09417	0.465226	-0.12454	0.34023	6768234	9.863302	9.952518	9.741368	9.814781	10.05383	9.97109	10.05633	10.16719	9.707287	9.884462	10.04436	9.924707	Sar1a	NM_009120
6768237	1099	6.335507	6.451699	6.362135	6.430883	-0.09538	0.312805	0.020816	0.820038	-0.06875	0.45982	0.089564	0.341366	-0.11619	0.225823	-0.02663	0.771286	6768237	6.317571	6.266567	6.422384	6.478129	6.420997	6.455971	6.509124	6.40654	6.17074	6.367465	6.380277	6.544907	Aifm2	NM_001039194
6768261	1100	8.827934	8.779886	8.772124	8.745379	0.082555	0.713073	0.034507	0.877395	0.026745	0.904793	0.007762	0.972298	0.048048	0.830036	0.05581	0.803207	6768261	8.808554	8.866623	8.808624	8.870565	8.986065	8.483027	8.792788	8.550485	8.973099	9.136318	8.776736	8.323083	EG432466	NR_002687
6768323	1103	8.155644	8.300682	8.211961	8.559925	-0.40428	0.00475	-0.25924	0.038066	-0.34796	0.010388	0.08872	0.420565	-0.14504	0.202597	-0.05632	0.604616	6768323	8.181199	8.149137	8.136597	8.387782	8.287541	8.226722	8.136383	8.346477	8.153024	8.428812	8.447025	8.803937	Slc25a16	NM_175194
6768326	1105	7.06751	7.294859	7.098062	7.304475	-0.23697	0.06097	-0.00962	0.931715	-0.20641	0.094265	0.196797	0.107969	-0.22735	0.06997	-0.03055	0.785901	6768326	7.161177	7.169969	6.871384	7.308318	7.299547	7.276711	7.143316	7.107868	7.043001	7.082954	7.365828	7.464643	Rufy2	NM_027425
6768332	1106	5.956656	5.894409	6.018462	5.927241	0.029415	0.714614	-0.03283	0.683493	0.091221	0.273757	-0.12405	0.148714	0.062247	0.445814	-0.06181	0.448921	6768332	6.014091	6.023203	5.832673	5.813944	5.830647	6.038636	6.015355	5.978654	6.061376	5.981949	5.829344	5.970429	3110049J	NM_026085
6768336	1108	8.273266	8.408805	8.283911	8.477381	-0.20412	0.081899	-0.06858	0.522791	-0.19347	0.096122	0.124895	0.258271	-0.13554	0.223108	-0.01064	0.919937	6768336	8.342213	8.35824	8.119344	8.408288	8.388025	8.430103	8.34094	8.439886	8.070906	8.450094	8.402271	8.579778	Atoh7	NM_016864
6768339	1109	7.564039	7.562234	7.497997	7.347072	0.216967	0.015092	0.215162	0.01569	0.150925	0.064449	0.064237	0.388314	0.001804	0.980186	0.066042	0.37577	6768339	7.544684	7.535837	7.611595	7.454617	7.671416	7.56067	7.586231	7.433099	7.474661	7.249518	7.449479	7.342219	Herc4	NM_026101
6768351	1110	7.440797	7.6841	7.699966	7.521816	-0.08102	0.515448	0.162284	0.210038	0.17815	0.172984	-0.01587	0.897289	-0.2433	0.075286	-0.25917	0.06119	6768351	7.3023	7.477468	7.542624	7.542638	7.827495	7.682166	7.743734	7.818262	7.537901	7.390196	7.46412	7.711132	Dnajc12	NM_013888
6768450	1113	8.750743	8.813341	8.776699	8.732219	0.018525	0.848485	0.081122	0.412655	0.044481	0.648276	0.036641	0.706481	-0.0626	0.523643	-0.02596	0.789172	6768450	8.713868	8.868124	8.670238	8.639162	8.824571	8.976289	8.781924	8.792372	8.755802	8.64235	8.861668	8.692638	Jmjd1c	NM_207221
6768479	1114	7.766914	7.301169	7.099759	6.399424	1.36749	0.00115	0.901745	0.011693	0.700335	0.035561	0.20141	0.488524	0.465745	0.131697	0.667155	0.04285	6768479	7.828544	7.782951	7.689247	7.07002	7.636016	7.197471	7.177435	6.582359	7.539484	6.363043	6.783718	6.051512	Egr2	NM_010118
6768572	1116	6.151242	6.175807	6.000822	6.020371	0.130871	0.481931	0.155436	0.406647	-0.01955	0.915001	0.174985	0.353016	-0.02456	0.893335	0.15042	0.421306	6768572	6.451144	5.856183	6.1464	6.429993	5.985074	6.112353	6.216882	5.806129	5.979455	6.004022	5.956201	6.10089	Tmem26	NM_177794
6768609	1117	7.616518	7.800488	7.552449	8.348781	-0.73226	0.042479	-0.54829	0.108743	-0.79633	0.030587	0.248039	0.437857	-0.18397	0.561572	0.064069	0.838239	6768609	7.227283	7.595827	8.026443	7.768255	7.44069	8.192518	7.275534	7.556039	7.825774	8.29718	8.791654	7.957509	Ank3	NM_146005
6768618	1118	6.404666	6.344302	6.274869	6.99663	-0.59196	0.060201	-0.65233	0.042507	-0.72176	0.028515	0.069433	0.804023	0.060364	0.829114	0.129797	0.644394	6768618	6.713278	6.352658	6.148062	6.334795	6.470559	6.227553	6.146297	6.480456	6.197855	6.741081	6.613021	7.635787	Slc16a9	NM_025807
6768621	1119	6.514641	6.702948	6.579368	6.287693	0.226948	0.28445	0.415255	0.069038	0.291675	0.17861	0.123581	0.549575	-0.18831	0.369032	-0.06473	0.751925	6768621	6.577901	6.480444	6.485578	7.079183	6.306267	6.723395	6.760213	6.701766	6.276124	6.198666	6.25233	6.412083	Fam13c	NM_001143777
6768693	1120	7.631054	7.774864	7.522278	7.6015	0.029554	0.762892	0.173364	0.104442	-0.07922	0.42699	0.252586	0.028449	-0.14381	0.167235	0.108775	0.283757	6768693	7.524871	7.760792	7.607498	7.750348	7.75586	7.818384	7.692117	7.420311	7.454407	7.504204	7.744979	7.555317	Ipmk	NM_027184
6768783	1121	7.900169	8.078079	7.926074	8.037841	-0.13767	0.111742	0.040238	0.615606	-0.11177	0.18489	0.152005	0.08394	-0.17791	0.049739	-0.0259	0.745329	6768783	8.040491	7.779536	7.880481	8.09306	8.153975	7.987203	7.950062	7.854511	7.973649	8.100547	7.941341	8.071636	Zwint	NM_025635
6768843	1123	7.063524	7.057225	7.112163	7.171766	-0.10824	0.612566	-0.11454	0.592408	-0.0596	0.779097	-0.05494	0.795911	0.006299	0.976291	-0.04864	0.818794	6768843	7.027834	6.962668	7.200069	7.264143	6.778005	7.129526	7.119395	7.105195	7.111899	6.956707	6.904537	7.654055	Gnaz	NM_010311
6768851	1125	8.608618	8.69833	8.65263	8.669277	-0.06066	0.459066	0.029053	0.719169	-0.01665	0.836312	0.0457	0.574037	-0.08971	0.283225	-0.04401	0.587984	6768851	8.699323	8.596146	8.530386	8.695727	8.764359	8.634904	8.688838	8.760805	8.508248	8.580356	8.666986	8.760489	Cytsa	NM_001145826
6768853	1126	5.821375	6.111054	6.080193	5.92605	-0.10468	0.59725	0.185004	0.359374	0.154143	0.441303	0.030861	0.875177	-0.28968	0.166397	-0.25882	0.210839	6768853	6.022875	5.759379	5.681871	6.081992	6.265856	5.985314	6.141587	5.675851	6.423142	5.912792	6.082312	5.783046	Adora2a	NM_009630
6768866	1128	9.612972	9.604225	9.532309	9.433591	0.17938	0.176199	0.170634	0.195846	0.098718	0.437859	0.071916	0.56841	0.008746	0.944107	0.080662	0.523452	6768866	9.662913	9.578945	9.597057	9.442844	9.700367	9.669465	9.777048	9.526496	9.293384	9.341263	9.512688	9.446823	Snrpd3	NM_026095
6768867	1129	9.437407	9.721062	9.393956	9.51402	-0.07661	0.49151	0.207042	0.087244	-0.12006	0.291325	0.327106	0.015166	-0.28365	0.028406	0.043451	0.693379	6768867	9.429302	9.37215	9.510769	9.614861	9.775533	9.772791	9.514012	9.18824	9.479616	9.515469	9.363567	9.663024	Ggt1	NM_008116
6768868	1130	7.005164	6.89337	6.676361	6.302784	0.70238	0.068842	0.590586	0.1153	0.373577	0.296252	0.217009	0.534464	0.111794	0.746685	0.328803	0.354175	6768868	7.268827	6.812759	6.933907	6.616734	7.430336	6.63304	6.992413	6.080981	6.95569	5.99465	6.704059	6.209644	Ggt5	NM_011820
6768891	1131	8.044266	8.154052	8.297141	8.457945	-0.41368	0.207112	-0.30389	0.342806	-0.1608	0.608143	-0.14309	0.647629	-0.10979	0.725088	-0.25288	0.425791	6768891	8.156114	7.954846	8.021838	8.311029	7.477299	8.673827	8.315331	8.406273	8.169819	8.687001	8.666654	8.02018	Derl3	NM_024440
6768897	1132	10.49628	10.23703	10.41556	11.06621	-0.56992	0.007575	-0.82917	0.000868	-0.65065	0.003704	-0.17853	0.299184	0.259252	0.145594	0.080727	0.629192	6768897	10.39627	10.51513	10.57745	10.48071	9.996875	10.23351	10.30568	10.63799	10.303	10.97336	10.90149	11.32377	Chchd10	NM_175329
6768898	1133	5.931322	5.968014	5.841935	6.801343	-0.87002	0.010302	-0.83333	0.012713	-0.95941	0.006237	0.126079	0.641805	-0.03669	0.891613	0.089387	0.740672	6768898	5.69182	6.054236	6.047909	6.125661	5.96694	5.81144	5.741563	5.97235	5.811892	6.624289	6.339387	7.440354	Vpreb3	NM_009514
6768901	1135	7.175646	7.017031	7.043935	7.088603	0.087042	0.452599	-0.07157	0.534425	-0.04467	0.695995	-0.0269	0.813356	0.158615	0.188205	0.13171	0.266454	6768901	7.239557	7.162348	7.125032	6.792434	7.230337	7.028322	7.151223	6.995721	6.984862	6.965176	7.187743	7.112891	Zfp280b	NM_177475
6768910	1137	6.97341	7.377798	7.286235	6.745428	0.227982	0.071659	0.632371	0.000426	0.540808	0.00116	0.091563	0.428794	-0.40439	0.006212	-0.31283	0.021567	6768910	7.134531	6.908111	6.877587	7.290154	7.410345	7.432896	7.467231	7.254628	7.136847	6.659192	6.673582	6.903509	S100b	NM_009115
6768925	1138	5.98567	6.149033	5.627122	5.73497	0.2507	0.137553	0.414063	0.02604	-0.10785	0.497987	0.521911	0.008889	-0.16336	0.313638	0.358548	0.045973	6768925	5.916493	6.103818	5.936698	6.184182	6.362132	5.900784	5.759555	5.755406	5.366404	5.901479	5.606685	5.696746	2610028H	NM_029816
6768926	1139	7.112305	7.172404	7.072358	7.028051	0.084255	0.055685	0.144353	0.004999	0.044307	0.273261	0.100046	0.028972	-0.0601	0.149235	0.039948	0.319826	6768926	7.094256	7.168946	7.073714	7.174871	7.214213	7.128127	7.097014	7.065791	7.054268	6.959678	7.074375	7.050099	Mcm3ap	NM_019434
6768928	1140	7.013865	7.163313	7.196221	7.369626	-0.35577	0.072012	-0.20631	0.263908	-0.17341	0.34212	-0.03291	0.852793	-0.14946	0.409432	-0.18237	0.319215	6768928	6.898994	6.997046	7.145525	7.073787	7.276671	7.139481	7.191688	7.121735	7.275239	7.009239	7.767688	7.331951	Lss	NM_146006
6768933	1142	6.020315	5.991353	5.95862	6.000743	0.019571	0.839446	-0.00939	0.922483	-0.04212	0.664328	0.032733	0.735336	0.028961	0.764689	0.061694	0.527934	6768933	6.029519	6.012173	6.019252	6.00578	5.920264	6.048016	6.045557	6.035011	5.795293	5.994381	5.836336	6.171513	Ftcd	NM_080845
6768951	1143	7.320018	7.562503	7.333638	7.442912	-0.12289	0.12869	0.119591	0.137841	-0.10927	0.170394	0.228865	0.013497	-0.24249	0.01019	-0.01362	0.855739	6768951	7.222181	7.43763	7.300242	7.629215	7.505934	7.552361	7.248278	7.449352	7.303124	7.362942	7.469477	7.496317	Slc19a1	NM_031196
6768978	1144	9.255118	9.26021	9.243238	9.334743	-0.07963	0.388495	-0.07453	0.41818	-0.0915	0.325309	0.016972	0.850741	-0.00509	0.95493	0.01188	0.895148	6768978	9.301434	9.23782	9.226099	9.135093	9.447559	9.197977	9.354938	9.21357	9.161206	9.24185	9.364476	9.397903	Pofut2	NM_030262
6768985	1145	8.645768	8.450834	8.735353	7.30937	1.336398	0.008609	1.141464	0.018355	1.425983	0.006144	-0.28452	0.482784	0.194935	0.627697	-0.08958	0.822572	6768985	8.898539	8.78355	8.255216	8.337297	8.774605	8.240599	9.363068	7.924475	8.918516	7.197049	7.747776	6.983285	Itgb2	NM_008404
6768987	1146	10.10797	10.02373	10.1091	10.17193	-0.06396	0.545607	-0.14821	0.181808	-0.06283	0.552542	-0.08537	0.424064	0.084248	0.429957	-0.00113	0.991403	6768987	10.09037	10.11236	10.12119	9.930537	10.10625	10.03439	10.28879	9.86061	10.1779	10.1622	10.11258	10.24102	Pttg1ip	NM_145925
6768990	1147	7.944112	7.947825	7.937196	7.965706	-0.02159	0.773242	-0.01788	0.81127	-0.02851	0.704209	0.010629	0.886985	-0.00371	0.960374	0.006915	0.926301	6768990	7.936197	7.944976	7.951162	7.980336	7.996909	7.866231	8.029292	7.987451	7.794846	7.960593	8.071783	7.864741	Ube2g2	NM_019803
6769030	1150	7.148996	7.212948	7.138285	6.916824	0.232172	0.117937	0.296124	0.055934	0.221461	0.133299	0.074663	0.588665	-0.06395	0.64238	0.010711	0.93758	6769030	7.077025	7.025307	7.344655	7.138806	7.393932	7.106105	7.370818	7.102871	6.941166	6.853672	6.915052	6.981748	1810043G	NM_026431
6769033	1152	7.626131	7.513839	7.514631	7.175251	0.45088	0.036838	0.338587	0.097095	0.33938	0.096441	-0.00079	0.9966	0.112292	0.550578	0.1115	0.553329	6769033	7.831746	7.360226	7.686421	7.592711	7.613074	7.335731	7.639283	7.202795	7.701815	7.188861	7.364983	6.97191	Icosl	NM_015790
6769080	1153	9.883157	9.934715	9.930414	9.668777	0.21438	0.1524	0.265938	0.085399	0.261637	0.089689	0.004301	0.975464	-0.05156	0.713572	-0.04726	0.736358	6769080	9.916944	9.851068	9.88146	10.00412	10.07193	9.728096	10.16612	9.959591	9.665532	9.54947	9.683467	9.773395	Cstb	NM_007793
6769088	1154	7.531515	7.638818	7.592859	7.577993	-0.04648	0.603162	0.060825	0.498956	0.014866	0.866888	0.045959	0.607142	-0.1073	0.246875	-0.06134	0.495411	6769088	7.548579	7.576578	7.469388	7.6427	7.624108	7.649645	7.732149	7.599596	7.446831	7.502845	7.48759	7.743543	Ilvbl	NM_173751
6769132	1162	7.551743	7.641982	7.655382	7.64193	-0.09019	0.399134	5.13E-05	0.999608	0.013452	0.897604	-0.0134	0.897992	-0.09024	0.398878	-0.10364	0.33605	6769132	7.565812	7.655132	7.434286	7.420976	7.729745	7.775224	7.635866	7.677821	7.652459	7.517866	7.697166	7.710759	Gtrgeo22	NM_148934
6769138	1164	12.81973	12.78607	12.74077	13.00573	-0.186	0.135622	-0.21966	0.085697	-0.26496	0.045685	0.045297	0.696715	0.033663	0.771582	0.07896	0.501144	6769138	12.92574	12.74817	12.78528	12.68689	12.94586	12.72545	12.83965	12.75731	12.62535	13.02952	12.80681	13.18085	Bsg	NM_009768
6769139	1165	6.972424	7.341354	7.041763	8.057164	-1.08474	0.019923	-0.71581	0.092102	-1.0154	0.026507	0.29959	0.446453	-0.36893	0.353025	-0.06934	0.857598	6769139	6.883147	6.983916	7.05021	7.54722	7.280348	7.196493	6.927993	7.572029	6.625268	7.733146	7.521389	8.916958	Hcn2	NM_008226
6769141	1166	7.621768	7.58085	7.519913	7.683287	-0.06152	0.674262	-0.10244	0.488391	-0.16337	0.280165	0.060937	0.677137	0.040919	0.779119	0.101855	0.490791	6769141	7.581112	7.591875	7.692318	7.581138	7.362745	7.798666	7.677416	7.510677	7.371646	7.915067	7.633546	7.501249	Fgf22	NM_023304
6769144	1167	7.410474	7.330376	7.104987	7.292538	0.117936	0.388044	0.037837	0.777095	-0.18755	0.184684	0.225389	0.119245	0.080099	0.552551	0.305487	0.045649	6769144	7.581581	7.27025	7.379592	7.433248	7.37155	7.186329	7.381676	6.971315	6.96197	7.331872	7.262558	7.283185	Fstl3	NM_031380
6769147	1168	7.092504	7.134353	7.022973	6.97697	0.115534	0.229015	0.157383	0.113956	0.046003	0.61808	0.11138	0.244694	-0.04185	0.649691	0.069531	0.455721	6769147	7.08077	7.127683	7.069058	7.182461	7.212933	7.007665	7.203702	6.96806	6.897156	6.885892	7.067301	6.977716	Palm	NM_023128
6769148	1169	6.84491	6.849232	6.900063	6.68401	0.1609	0.287352	0.165221	0.275526	0.216053	0.164431	-0.05083	0.728108	-0.00432	0.976329	-0.05515	0.706223	6769148	6.67182	6.928839	6.934072	7.166528	6.581333	6.799834	6.863954	6.880758	6.955478	6.593275	6.696196	6.76256	9130017N	NM_030218
6769150	1170	10.021	9.895726	9.935276	9.94388	0.077124	0.324517	-0.04815	0.530587	-0.0086	0.909657	-0.03955	0.605013	0.125278	0.126562	0.085728	0.276876	6769150	9.99372	9.993702	10.07559	9.744653	10.03177	9.910756	9.915063	9.880905	10.00986	9.942423	10.01467	9.874548	Ptbp1	NM_001077363
6769159	1173	8.586177	8.651225	8.013328	8.580011	0.006166	0.988534	0.071214	0.868292	-0.56668	0.210132	0.637898	0.163618	-0.06505	0.879587	0.57285	0.20569	6769159	8.083376	8.39955	9.275606	8.543083	8.643127	8.767466	8.652567	7.846437	7.540979	7.98602	9.098941	8.655073	Cfd	NM_013459
6769164	1174	7.138085	7.180379	7.275346	7.33606	-0.19798	0.157312	-0.15568	0.254719	-0.06071	0.645098	-0.09497	0.475609	-0.04229	0.747452	-0.13726	0.310872	6769164	7.111555	7.198812	7.103887	7.465917	7.032452	7.042768	7.168367	7.460706	7.196965	7.298018	7.280371	7.42979	Kiss1r	NM_053244
6769165	1175	7.202289	7.330998	7.284218	7.226396	-0.02411	0.76638	0.104601	0.218974	0.057822	0.481981	0.046779	0.567319	-0.12871	0.139369	-0.08193	0.326677	6769165	7.209991	7.233431	7.163445	7.329488	7.388531	7.274974	7.229901	7.476754	7.146	7.197664	7.193621	7.287904	Arid3a	NM_007880
6769166	1176	7.565341	7.55999	7.654246	7.553658	0.011683	0.923542	0.006332	0.958508	0.100588	0.418597	-0.09426	0.447323	0.005351	0.964929	-0.08891	0.472624	6769166	7.499401	7.692379	7.504243	7.359698	7.702115	7.618157	7.736474	7.46915	7.757115	7.46721	7.68698	7.506785	Wdr18	NM_175450
6769179	1178	10.11881	9.81793	9.969396	9.449269	0.669544	0.003483	0.368661	0.054371	0.520127	0.013043	-0.15147	0.38184	0.300883	0.103326	0.149417	0.388001	6769179	10.14581	10.12868	10.08195	9.881987	9.956027	9.615776	10.15868	9.814328	9.93518	9.548357	9.69931	9.100141	Cnn2	NM_007725
6769180	1179	7.430565	7.390295	7.408585	7.201805	0.22876	0.187922	0.18849	0.269565	0.20678	0.22937	-0.01829	0.911199	0.04027	0.806326	0.02198	0.8934	6769180	7.610222	7.354696	7.326777	7.592829	7.331144	7.246912	7.41864	7.671394	7.135721	7.167755	7.366715	7.070946	Abca7	NM_013850
6769181	1180	7.057724	6.950239	6.922721	6.432797	0.624927	0.000433	0.517441	0.001438	0.489923	0.001999	0.027518	0.80678	0.107486	0.352305	0.135004	0.249987	6769181	7.228166	6.988677	6.95633	7.019955	7.083702	6.747059	7.036811	6.884392	6.846959	6.411198	6.524782	6.362412	Hmha1	NM_001142701
6769189	1181	9.364753	9.442964	9.423528	9.292185	0.072569	0.579047	0.150779	0.263983	0.131344	0.325929	0.019435	0.880775	-0.07821	0.550543	-0.05877	0.652075	6769189	9.329805	9.360847	9.403608	9.436648	9.534817	9.357426	9.659801	9.442604	9.16818	9.119925	9.426247	9.330382	Stk11	NM_011492
6769192	1182	11.31917	11.20385	11.33527	11.63151	-0.31234	0.013271	-0.42766	0.002495	-0.29624	0.016994	-0.13142	0.219502	0.11532	0.276053	-0.0161	0.874429	6769192	11.2638	11.23421	11.4595	11.26947	11.30238	11.0397	11.26455	11.50686	11.23439	11.6219	11.61305	11.65957	Atp5d	NM_025313
6769193	1183	9.531135	9.441937	9.519962	9.543985	-0.01285	0.943595	-0.10205	0.578053	-0.02402	0.894816	-0.07802	0.669325	0.089198	0.626015	0.011173	0.950947	6769193	9.631659	9.524966	9.436779	9.476881	9.725885	9.123045	9.33811	9.826308	9.395467	9.423617	9.553714	9.654625	Midn	NM_021565
6769197	1184	8.278379	8.724835	8.408661	8.697318	-0.41894	0.063178	0.027517	0.890885	-0.28866	0.175701	0.316174	0.142353	-0.44646	0.050656	-0.13028	0.521428	6769197	8.43171	8.291879	8.111547	8.896219	8.623549	8.654737	8.573464	8.369105	8.283414	8.562492	8.387024	9.142437	Cirbp	NM_007705

6769198	1185	6.907722	6.568844	6.684298	6.897839	0.009883	0.954139	-0.32899	0.083658	-0.21354	0.235713	-0.11545	0.507816	0.338878	0.076309	0.223424	0.216612	6769198	6.956219	7.004358	6.762589	6.83574	6.553913	6.31688	6.965773	6.619847	6.467275	7.048759	6.838941	6.805817	1600002K	NM_027207
6769201	1187	7.189057	7.372661	7.206368	7.331829	-0.14277	0.291432	0.040832	0.754963	-0.12546	0.350014	0.166293	0.22478	-0.1836	0.184437	-0.01731	0.894461	6769201	7.081187	7.174729	7.311256	7.391638	7.444519	7.281826	7.430703	7.170584	7.017816	7.164101	7.311192	7.520194	Mum1	NM_023431
6769204	1188	10.63611	10.6767	10.56675	10.93922	-0.30311	0.072434	-0.26252	0.111031	-0.37247	0.034636	0.109943	0.474642	-0.04059	0.78884	0.069353	0.648709	6769204	10.39761	10.60606	10.90465	10.75617	10.71266	10.56126	10.42971	10.72203	10.54852	10.82071	10.85247	11.14448	Ndufs7	NM_029272
6769212	1190	10.84279	11.30253	11.07917	11.45901	-0.61622	0.000914	-0.15649	0.230273	-0.37984	0.013557	0.223353	0.100942	-0.45971	0.005126	-0.23638	0.085451	6769212	10.80279	10.85726	10.86788	11.38805	11.20265	11.31688	10.86524	11.37738	10.9949	11.46303	11.37799	11.53602	Reep6	NM_139292
6769224	1192	6.778693	6.855662	6.707598	6.996408	-0.21772	0.068803	-0.14075	0.211401	-0.28881	0.023652	0.148064	0.190857	-0.07697	0.478809	0.071095	0.512001	6769224	6.860518	6.795521	6.680039	6.79454	6.72938	7.043066	6.726954	6.786304	6.609535	7.014847	6.844684	7.129693	Onecut3	NM_139226
6769232	1193	7.649367	7.430954	7.697656	7.731001	-0.08163	0.281658	-0.30005	0.002826	-0.03335	0.649851	-0.2667	0.005454	0.218413	0.014921	-0.04829	0.513983	6769232	7.682977	7.545694	7.719431	7.339849	7.549963	7.40305	7.761845	7.705709	7.625414	7.805103	7.728153	7.659747	Scamp4	NM_019575
6769235	1194	9.08995	9.2123	9.018472	8.95059	0.13936	0.093241	0.26171	0.007214	0.067881	0.380586	0.193828	0.029273	-0.12235	0.132961	0.071478	0.357134	6769235	9.056538	9.058828	9.154484	9.190772	9.320466	9.125662	9.132481	9.011551	8.911383	8.854902	9.003383	8.993486	Csnk1g2	NM_134002
6769241	1195	7.162825	7.276473	7.340181	7.366547	-0.20372	0.16911	-0.09007	0.522756	-0.02637	0.849783	-0.06371	0.649074	-0.11365	0.423607	-0.17736	0.22467	6769241	7.084652	7.218282	7.18554	7.360278	7.198264	7.270878	7.473766	7.54428	7.002497	7.361797	7.265109	7.472736	9030607L	NM_027829
6769244	1196	7.567284	7.604083	7.597353	7.582091	-0.01481	0.892608	0.021992	0.841197	0.015261	0.88934	0.00673	0.951047	-0.0368	0.738018	-0.03007	0.784363	6769244	7.69862	7.507963	7.495269	7.643199	7.69133	7.47772	7.498665	7.808048	7.485345	7.520622	7.690479	7.535173	Dot1l	NM_199322
6769245	1197	8.374117	8.293493	8.437842	8.287248	0.086868	0.478572	0.006244	0.9587	0.150594	0.233573	-0.14435	0.251832	0.080624	0.509803	-0.06373	0.600443	6769245	8.446034	8.333935	8.342381	8.300336	8.438799	8.141343	8.235655	8.667512	8.41036	8.298496	8.374448	8.188801	Sf3a2	NM_013651
6769249	1200	7.92007	7.829561	7.877283	7.816827	0.103243	0.24608	0.012734	0.881139	0.060456	0.484545	-0.04772	0.578835	0.090509	0.304477	0.042787	0.618015	6769249	7.877382	7.908642	7.974187	7.795352	7.993464	7.699868	7.958477	7.773978	7.899395	7.812541	7.734889	7.903051	3110056O	NM_175195
6769255	1201	7.731316	7.764664	7.413852	7.14111	0.590206	0.071481	0.623554	0.059545	0.272742	0.365323	0.350812	0.252093	-0.03335	0.909484	0.317464	0.296404	6769255	7.829644	7.796813	7.567491	7.598407	8.186781	7.508804	7.796592	6.906804	7.53816	6.905757	7.537221	6.980351	Gadd45b	NM_008655
6769262	1202	7.005884	7.033817	7.010704	7.166346	-0.16046	0.402798	-0.13253	0.486446	-0.15564	0.41646	0.023113	0.901888	-0.02793	0.881593	-0.00482	0.979481	6769262	7.036592	7.023246	6.957813	6.988833	7.234988	6.87763	7.07407	6.869641	7.0884	6.918	7.609142	6.971897	Thop1	NM_022653
6769263	1203	9.306125	9.25878	9.247627	9.365978	-0.05985	0.511238	-0.1072	0.253208	-0.11835	0.211125	0.011153	0.901237	0.047345	0.60142	0.058497	0.520597	6769263	9.262991	9.259336	9.396047	9.19652	9.355046	9.224774	9.317841	9.357476	9.067565	9.279873	9.364351	9.453711	Map2k2	NM_023138
6769264	1204	8.701646	8.596085	8.524484	8.667814	0.033832	0.831652	-0.07173	0.653867	-0.14333	0.379329	0.071602	0.654434	0.105561	0.512513	0.177163	0.283299	6769264	8.763171	8.657531	8.684237	8.65138	8.710912	8.425964	8.243456	8.813686	8.516309	8.503744	8.873218	8.626481	Zbtb7a	NM_010731
6769267	1205	12.60627	12.6681	12.59761	12.70312	-0.09685	0.173484	-0.03502	0.603726	-0.10551	0.142221	0.070493	0.308483	-0.06183	0.368054	0.00866	0.897018	6769267	12.56588	12.6017	12.65123	12.62315	12.70611	12.67505	12.69773	12.58261	12.51249	12.59363	12.6956	12.82014	Eef2	NM_007907
6769268	1206	8.914136	8.847577	8.88847	8.918834	-0.0047	0.9635	-0.07126	0.494234	-0.03036	0.768012	-0.04089	0.691845	0.06656	0.52233	0.025666	0.802938	6769268	8.850325	8.966978	8.925106	8.759132	9.096302	8.687296	8.817471	8.951256	8.896683	8.879105	8.988716	8.88868	Dapk3	NM_007828
6769272	1207	6.488162	6.480418	6.526974	6.499603	-0.01144	0.87851	-0.01918	0.797971	0.027372	0.715563	-0.04656	0.538689	0.007744	0.917555	-0.03881	0.606932	6769272	6.559556	6.417568	6.487362	6.508261	6.40891	6.524082	6.473573	6.683522	6.423828	6.56858	6.460094	6.470134	Matk	NM_010768
6769277	1208	8.370941	8.382348	8.318635	8.349323	0.021617	0.788712	0.033024	0.683189	-0.03069	0.704295	0.063713	0.437708	-0.01141	0.887356	0.052306	0.521403	6769277	8.259112	8.381687	8.472023	8.402812	8.412164	8.332067	8.338999	8.448353	8.168553	8.2834	8.376793	8.387777	Apba3	NM_018758
6769280	1209	8.841383	8.80341	8.784641	8.711048	0.130335	0.301279	0.092361	0.456145	0.073592	0.550045	0.018769	0.877512	0.037973	0.755745	0.056742	0.643346	6769280	8.963306	8.771824	8.789019	8.866194	8.936223	8.607812	8.81951	8.914762	8.61965	8.602049	8.661942	8.869154	Pip5k1c	NM_008844
6769282	1210	7.182776	7.204317	7.051922	7.07646	0.106316	0.478008	0.127857	0.396884	-0.02454	0.867878	0.152395	0.317178	-0.02154	0.883865	0.130854	0.386427	6769282	7.258768	7.415995	6.873564	7.230077	7.149944	7.232931	7.168927	7.039624	6.947215	6.877011	7.166882	7.185487	Tbxa2r	NM_009325
6769286	1211	6.697385	6.65231	6.70919	6.692465	0.004919	0.955167	-0.04016	0.648514	0.016725	0.84858	-0.05688	0.521298	0.045075	0.60951	-0.01181	0.892728	6769286	6.564983	6.760535	6.766636	6.651573	6.605248	6.700108	6.773166	6.709364	6.645041	6.523704	6.72907	6.824622	Dohh	NM_133964
6769304	1212	11.124	11.04497	11.12562	11.3655	-0.2415	0.114268	-0.32052	0.04651	-0.23988	0.116361	-0.08065	0.570278	0.079023	0.577905	-0.00162	0.990786	6769304	11.08139	11.05751	11.23309	11.10805	11.09523	10.93164	11.18273	11.3498	10.84433	11.23994	11.30783	11.54872	Aes	NM_010347
6769305	1213	7.104789	7.358436	7.221624	7.674356	-0.56957	0.008187	-0.31592	0.08885	-0.45273	0.024115	0.136812	0.426073	-0.25365	0.15864	-0.11684	0.494298	6769305	7.08601	7.135051	7.093305	7.375519	7.380444	7.319345	6.997491	7.577766	7.089616	7.515043	7.5501	7.957926	Tle2	NM_019725
6769309	1214	7.061249	7.102001	7.172736	7.40768	-0.34643	0.01201	-0.30568	0.021402	-0.23494	0.059707	-0.07074	0.527767	-0.04075	0.713652	-0.11149	0.328616	6769309	7.002768	7.171227	7.009753	7.144804	6.936217	7.224981	7.065784	7.340088	7.112337	7.396412	7.286964	7.539663	Ankrd24	NM_027480
6769310	1215	6.571723	6.163628	6.774687	6.153048	0.418675	0.20364	0.010579	0.972952	0.621639	0.073881	-0.61106	0.078003	0.408096	0.214162	-0.20296	0.52106	6769310	6.420649	7.004897	6.289624	6.254929	6.351084	5.88487	6.602354	6.782568	6.939139	5.505466	6.460544	6.493135	BC062127	BC062127
6769316	1216	6.295725	6.252421	6.487715	6.287461	0.008264	0.953498	-0.03504	0.80507	0.200254	0.182957	-0.23529	0.125048	0.043304	0.760614	-0.19199	0.199712	6769316	6.2061	6.437605	6.24347	6.171623	6.199149	6.38649	6.321864	6.63526	6.506021	6.034276	6.308263	6.519844	BC062115	NM_001024626
6769357	1218	9.751425	9.733192	9.642639	9.308212	0.443213	0.039726	0.42498	0.046502	0.334426	0.101289	0.090554	0.62969	0.018233	0.922093	0.108786	0.563726	6769357	10.08081	9.576622	9.596843	9.677498	9.97561	9.546469	9.826215	9.517899	9.583802	9.081813	9.455616	9.387208	Txnrd1	NM_001042523
6769358	1219	6.08236	6.031542	5.900216	5.831908	0.250452	0.452853	0.199634	0.546939	0.068308	0.835002	0.131326	0.689946	0.050818	0.876775	0.182144	0.581861	6769358	6.612502	6.27395	5.360628	6.082819	6.248121	5.763686	5.530372	6.06609	6.104186	5.682788	5.836062	5.976875	Eid3	NM_025499
6769366	1220	9.068832	8.634374	9.045102	8.600633	0.468199	0.051333	0.033741	0.873062	0.444469	0.061518	-0.41073	0.079505	0.434459	0.066391	0.02373	0.910493	6769366	9.008704	9.305492	8.892301	8.418172	8.704064	8.780885	9.215422	9.080393	8.839491	8.580872	8.974533	8.246494	Chst11	NM_021439
6769381	1221	8.777901	8.754362	8.658832	8.599873	0.178028	0.306741	0.154489	0.371184	0.058959	0.727047	0.095529	0.574151	0.023539	0.888794	0.119069	0.486119	6769381	8.759754	8.742499	8.83145	8.743399	8.732514	8.787172	8.538125	8.727755	8.710617	8.73833	8.892739	8.16855	D10Wsu10	BC094241
6769445	1222	8.095754	8.242554	8.219154	7.969692	0.126062	0.428203	0.272862	0.108498	0.249462	0.137257	0.0234	0.880734	-0.1468	0.359609	-0.1234	0.437633	6769445	8.074516	8.229522	7.983223	8.00738	8.394654	8.325627	8.352612	8.047865	8.256985	7.82072	7.849751	8.238605	Tcp11l2	NM_146008
6769447	1223	8.20036	8.378701	8.272661	8.199121	0.001239	0.98856	0.17958	0.064306	0.07354	0.405402	0.10604	0.240994	-0.17834	0.065805	-0.0723	0.413013	6769447	8.133594	8.210214	8.257271	8.16137	8.494556	8.480177	8.319343	8.218377	8.280263	8.187479	8.205652	8.204232	Polr3b	NM_027423
6769449	1225	8.164525	8.217072	7.920288	7.88791	0.276615	0.06427	0.329162	0.034041	0.032378	0.808084	0.296784	0.050357	-0.05255	0.694339	0.244237	0.094846	6769449	8.184709	8.146604	8.162261	8.072435	8.525827	8.052954	7.823844	7.908763	8.028256	7.999401	7.921328	7.743001	Ric8b	NM_183172
6769461	1226	7.157029	7.512713	7.405715	7.500215	-0.34319	0.065373	0.012498	0.93996	-0.0945	0.572974	0.106998	0.524527	-0.35568	0.057915	-0.24869	0.160578	6769461	7.032494	7.199396	7.239197	7.260732	7.765356	7.512052	7.433567	7.514467	7.269111	7.409968	7.785607	7.305071	AI597468	NM_001013028
6769509	1228	7.157962	7.241876	7.303552	7.047103	0.110858	0.393236	0.194772	0.151546	0.256449	0.070305	-0.06168	0.629179	-0.08391	0.513884	-0.14559	0.27001	6769509	7.153625	7.13547	7.18479	7.197504	7.494195	7.033928	7.343733	7.244914	7.322009	6.896265	7.247991	6.997054	Pwp1	NM_133993
6769515	1229	7.678802	7.784202	7.731361	7.907141	-0.22834	0.095185	-0.12294	0.33826	-0.17578	0.183426	0.05284	0.673146	-0.1054	0.408016	-0.05256	0.674768	6769515	7.624079	7.738084	7.674244	7.874315	7.826922	7.651368	7.755963	7.710542	7.727579	7.850256	7.675948	8.19522	Fbxo7	NM_153195
6769535	1230	11.40369	11.74521	11.31702	11.81529	-0.4116	0.060015	-0.07008	0.719061	-0.49827	0.029259	0.42819	0.052296	-0.34152	0.106855	0.08667	0.657116	6769535	11.38833	11.51617	11.30657	12.05086	11.62745	11.55732	10.94329	11.57616	11.43161	11.69593	11.9699	11.78003	Timp3	NM_011595
6769559	1232	6.138861	6.261868	5.997682	5.645582	0.49328	0.144075	0.616286	0.077707	0.3521	0.281136	0.264186	0.411104	-0.12301	0.696967	0.14118	0.655417	6769559	5.973819	5.881867	6.560898	6.491876	6.59086	5.702868	6.139507	6.092016	5.761522	6.076829	5.379959	5.479957	Nt5dc3	NM_175331
6769597	1235	8.156878	8.257309	7.752015	9.417276	-1.2604	0.009241	-1.15997	0.013866	-1.66526	0.001992	0.505294	0.208935	-0.10043	0.792797	0.404863	0.305409	6769597	7.669901	8.453298	8.347436	7.73838	8.573181	8.460367	7.826717	7.510521	7.918808	10.09497	9.288677	8.86818	Igf1	NM_010512
6769614	1238	6.877918	6.998638	6.700731	6.958561	-0.08064	0.185705	0.040077	0.492307	-0.25783	0.001691	0.297908	0.000687	-0.12072	0.06208	0.177188	0.012977	6769614	6.808668	6.945156	6.879931	6.913242	6.988361	7.094312	6.766164	6.671902	6.664126	6.972754	6.903485	6.999444	Nup37	NM_028334
6769617	1239	8.051319	8.338245	8.21862	8.702824	-0.65151	0.004138	-0.36458	0.057201	-0.4842	0.018478	0.119625	0.487169	-0.28693	0.118777	-0.1673	0.338191	6769617	7.86653	8.2208	8.066626	8.234715	8.345677	8.434342	8.362255	8.256205	8.037399	8.634036	8.438383	9.036054	Ccdc53	NM_026070
6769634	1240	10.01052	10.06551	10.01857	9.66287	0.347647	0.012175	0.402639	0.00576	0.355695	0.01089	0.046944	0.674865	-0.05499	0.623887	-0.00805	0.942347	6769634	10.00875	10.07104	9.951761	9.941287	10.22694	10.0283	10.16403	9.811045	10.08062	9.58463	9.780486	9.623493	Gnptab	NM_001004164
6769662	1242	6.733419	7.534568	7.269509	8.591432	-1.85801	0.041755	-1.05686	0.205705	-1.32192	0.123222	0.265059	0.738673	-0.80115	0.326961	-0.53609	0.504565	6769662	7.09865	6.488161	6.613447	8.321991	7.189774	7.091939	6.800977	8.227547	6.780004	7.626211	7.819454	10.32863	Slc5a8	NM_145423
6769735	1243	8.351184	8.442413	8.387494	8.451542	-0.10036	0.11891	-0.00913	0.877723	-0.06405	0.297446	0.054919	0.367257	-0.09123	0.151086	-0.03631	0.545146	6769735	8.409027	8.379375	8.26515	8.40077	8.554035	8.372434	8.437179	8.400725	8.324578	8.478313	8.41242	8.463894	Uhrf1bp1	NM_029166
6769747	1244	6.756552	6.944525	6.788878	6.701222	0.05533	0.661821	0.243302	0.080913	0.087655	0.49237	0.155647	0.237276	-0.18797	0.161472	-0.03233	0.797487	6769747	6.831806	6.852766	6.585085	6.817949	7.167171	6.848454	6.656597	6.936701	6.773335	6.721968	6.787733	6.593966	1200009F	NM_026166
6769845	1245	7.742733	7.799691	7.71721	7.565667	0.177066	0.176263	0.234024	0.085585	0.151543	0.239924	0.082481	0.509122	-0.05696	0.646004	0.025523	0.836038	6769845	7.816192	7.709117	7.70289	7.698568	7.998799	7.701706	7.757027	7.683409	7.711195	7.320099	7.759778	7.617125	Pctk2	NM_146239
6769877	1246	7.837058	8.239872	8.06549	8.095904	-0.25885	0.017169	0.143968	0.134176	-0.03041	0.733893	0.174382	0.078235	-0.40281	0.001618	-0.22843	0.02952	6769877	7.83406	7.832113	7.845002	8.145074	8.37293	8.201612	7.988835	8.09846	8.109176	8.117578	8.245894	7.92424	Lta4h	NM_008517
6769878	1247	7.611841	5.618469	6.918114	5.524793	2.087048	6.25E-05	0.093676	0.741493	1.393321	0.000953	-1.29965	0.001467	1.993372	8.65E-05	0.693727	0.035292	6769878	7.891709	7.442249	7.501564	5.437893	5.462702	5.954811	7.078099	6.718747	6.957496	5.116041	5.344496	6.113842	Hal	NM_010401
6769887	1249	6.944978	6.815365	6.824675	6.481182	0.463796	0.020929	0.334183	0.072713	0.343493	0.066488	-0.00931	0.955518	0.129613	0.446166	0.120303	0.478367	6769887	6.978575	7.127357	6.729001	6.901001	6.908731	6.636362	6.670289	7.099379	6.704357	6.389456	6.355965	6.698125	Ntn4	NM_021320
6769917	1251	7.394812	7.311469	7.328014	6.880095	0.514717	0.04463	0.431373	0.081316	0.447918	0.07222	-0.01655	0.940928	0.083344	0.710545	0.066799	0.765426	6769917	7.529809	7.301999	7.352629	7.150761	7.674841	7.108804	7.547987	6.976066	7.459988	6.764502	7.187449	6.688335	Fgd6	NM_053072
6769918	1252	8.496433	8.170866	8.49148	7.986804	0.509629	0.004754	0.184062	0.199941	0.504676	0.005015	-0.32061	0.040982	0.325567	0.038648	0.004952	0.970937	6769918	8.586467	8.614426	8.288405	8.067963	8.362585	8.08205	8.617814	8.282064	8.574563	7.915015	7.941086	8.104311	Nr2c1	NM_011629
6769928	1253	6.935853	6.880888	6.711407	6.767813	0.16804	0.184209	0.113075	0.356745	-0.05641	0.638752	0.169481	0.180877	0.054965	0.64723	0.224446	0.088189	6769928	7.082691	6.766585	6.958283	7.01404	6.968991	6.659633	6.617452	6.716557	6.600212	6.746481	6.704489	6.85247	Tmcc3	NM_172051
6769934	1254	7.012513	7.098702	7.061958	6.751603	0.26091	0.065705	0.347099	0.021988	0.310355	0.035	0.036744	0.771757	-0.08619	0.501461	-0.04944	0.696926	6769934	7.030131	7.022126	6.985281	7.012228	7.28132	7.002558	7.303838	6.845098	7.036937	6.668964	6.871314	6.71453	Ccdc41	NM_029852
6769996	1255	7.220175	7.189008	7.011994	7.059524	0.160651	0.524661	0.129484	0.606463	-0.04753	0.848898	0.177014	0.484536	0.031167	0.90051	0.208181	0.413807	6769996	7.250159	7.446599	6.963767	6.907002	7.316545	7.343478	6.818744	7.329481	6.887758	7.183155	6.619596	7.375822	Ube2n	NM_080560
6770013	1257	8.277115	8.231219	8.258717	8.089407	0.187708	0.227564	0.141812	0.352389	0.16931	0.272347	-0.0275	0.852942	0.045895	0.757502	0.018397	0.90124	6770013	8.442504	8.324763	8.064077	8.298598	8.213362	8.181698	8.425398	8.108196	8.242558	7.871303	8.345392	8.051526	Eea1	NM_001001932
6770030	1258	10.17735	10.18644	10.06062	9.770506	0.406844	0.061057	0.415931	0.056596	0.290117	0.159007	0.125814	0.519599	-0.00909	0.962394	0.116727	0.549458	6770030	10.29564	10.23247	10.00394	9.955791	10.38312	10.2204	10.36004	9.74266	10.07917	9.534907	9.934365	9.842246	Btg1	NM_007569
6770064	1259	11.38751	10.97013	10.5326	9.884553	1.502957	0.027106	1.085577	0.08704	0.648047	0.278014	0.43753	0.454636	0.41738	0.474962	0.85491	0.16324	6770064	11.57641	11.34548	11.24064	10.98316	11.50329	10.42394	11.26777	10.37419	9.955841	8.76116	10.82533	10.06717	Dcn	NM_007833
6770072	1260	8.47182	8.143773	6.925846	6.82546	1.64636	0.065303	1.318313	0.125744	0.100386	0.899647	1.217927	0.152929	0.328047	0.681777	1.545974	0.07994	6770072	8.901783	8.538281	7.975396	7.569652	9.01591	7.845758	8.220445	5.776221	6.780872	5.712848	7.950405	6.813128	Lum	NM_008524
6770148	1264	7.492627	7.509014	7.569864	7.361488	0.131139	0.042378	0.147526	0.02652	0.208376	0.005	-0.06085	0.295538	-0.01639	0.770803	-0.07724	0.193222	6770148	7.511135	7.535748	7.430998	7.554862	7.557082	7.415097	7.641076	7.525909	7.542607	7.3575	7.428561	7.298402	Wdr51b	NM_027740
6770149	1265	6.545074	6.587477	6.464914	6.308009	0.237066	0.268421	0.279468	0.198497	0.156905	0.453878	0.122563	0.555723	-0.0424	0.836863	0.08016	0.698108	6770149	6.727612	6.608794	6.298817	6.468308	6.674432	6.619691	6.853296	6.052296	6.48915	6.250847	6.457822	6.215357	Galnt4	NM_015737

6770160	1266	8.921673	8.935049	8.629208	8.604981	0.316692	0.048134	0.330068	0.04128	0.024227	0.862938	0.30584	0.054523	-0.01338	0.924017	0.292465	0.063568	6770160	8.940022	8.975998	8.848999	8.87256	9.158498	8.774088	8.747234	8.565871	8.57452	8.372248	8.596593	8.846102	Dusp6	NM_026268
6770164	1267	6.361072	5.923896	6.379824	6.219407	0.141666	0.433355	-0.29551	0.123627	0.160417	0.377613	-0.45593	0.029044	0.437176	0.034415	-0.01875	0.915748	6770164	6.105047	6.326924	6.651246	5.81993	5.77986	6.171899	6.157778	6.568386	6.413307	6.332031	6.212954	6.113235	B530045E	ENSMUST000000
6770201	1268	8.046593	8.016425	7.518302	8.18491	-0.13832	0.289734	-0.16849	0.204624	-0.66661	0.000599	0.498123	0.00352	0.030168	0.810923	0.528291	0.002511	6770201	8.144464	7.890562	8.104753	7.916576	8.108975	8.023724	7.479739	7.772472	7.302696	8.123583	8.168143	8.263005	Kitl	NM_013598
6770214	1269	6.726094	6.66	6.591079	6.505638	0.220456	0.109916	0.154362	0.243581	0.085441	0.50568	0.068921	0.589474	0.066094	0.604557	0.135015	0.302894	6770214	6.872473	6.723094	6.582715	6.790788	6.510361	6.678851	6.60316	6.599382	6.570695	6.317945	6.449289	6.74968	Cep290	NM_146009
6770445	1273	7.969862	8.08862	8.125994	7.95434	0.015522	0.856006	0.13428	0.143617	0.171654	0.071948	-0.03737	0.663799	-0.11876	0.189517	-0.15613	0.096148	6770445	7.775058	8.035446	8.099082	8.102204	8.155247	8.00841	8.185943	8.076178	8.11586	7.91813	7.926795	8.018095	Ccdc59	NM_025602
6770646	1275	6.20056	6.232728	6.142287	5.987902	0.212658	0.070812	0.244826	0.043326	0.154385	0.168977	0.09044	0.401585	-0.03217	0.760785	0.058272	0.583865	6770646	6.325848	6.080857	6.194974	6.204529	6.315953	6.177701	6.063992	6.302802	6.060068	5.915625	5.886552	6.161529	E2f7	NM_178609
6770661	1276	8.455625	8.569241	8.346269	8.80628	-0.35066	0.093211	-0.23704	0.233773	-0.46001	0.036975	0.222972	0.260274	-0.11362	0.554184	0.109356	0.568813	6770661	8.321441	8.416101	8.629333	8.755431	8.471915	8.480376	8.204373	8.680358	8.154076	8.737047	8.587102	9.094692	Csrp2	NM_007792
6770693	1277	8.024133	8.026754	8.039547	7.884246	0.139887	0.336917	0.142508	0.328475	0.155301	0.289613	-0.01279	0.927869	-0.00262	0.985199	-0.01541	0.913155	6770693	8.127706	8.048467	7.896227	8.127634	8.131936	7.820693	8.257595	7.919089	7.941958	7.734903	7.839267	8.078568	Osbpl8	NM_175489
6770718	1278	8.295946	8.207538	8.303172	8.10892	0.187026	0.333219	0.098619	0.60191	0.194252	0.316001	-0.09563	0.612764	0.088407	0.639458	-0.00723	0.969237	6770718	8.217838	8.386919	8.28308	7.973046	8.417965	8.231604	8.59869	8.089846	8.220979	7.812034	8.326612	8.188113	Nap1l1	NM_001146707
6770722	1280	9.333635	8.751415	8.995743	8.790803	0.542832	0.110884	-0.03939	0.899751	0.20494	0.517749	-0.24433	0.443203	0.58222	0.090817	0.337892	0.297016	6770722	9.703695	9.145492	9.151718	8.739169	9.194737	8.32034	8.966375	9.467634	8.55322	8.91741	8.91411	8.540888	Phlda1	NM_009344
6770743	1281	8.277923	8.428951	8.447608	8.369393	-0.09147	0.467156	0.059558	0.632529	0.078215	0.532224	-0.01866	0.880123	-0.15103	0.24304	-0.16969	0.194477	6770743	8.141233	8.34308	8.349455	8.284044	8.599136	8.403673	8.628701	8.461348	8.252776	8.351694	8.483685	8.2728	Krr1	NM_178610
6770908	1285	9.354453	9.891889	9.199001	9.42618	-0.07173	0.697468	0.465709	0.030797	-0.22718	0.23756	0.692888	0.004586	-0.53744	0.01656	0.155452	0.407832	6770908	9.520599	9.507639	9.035122	10.17068	9.699124	9.805863	9.118145	9.360892	9.117967	9.347327	9.633042	9.298171	Ptprb	NM_029928
6770923	1286	5.767206	5.974001	6.06848	5.997077	-0.22987	0.067107	-0.02308	0.836995	0.071403	0.529209	-0.09448	0.409526	-0.2068	0.093283	-0.30127	0.024111	6770923	5.826106	5.755211	5.720301	5.838573	5.827948	6.255482	6.139636	6.096346	5.969458	5.996465	5.97413	6.020636	5330438D	AK077360
6771050	1291	5.994056	6.062441	6.093368	6.088415	-0.09436	0.225769	-0.02597	0.727253	0.004953	0.946763	-0.03093	0.678428	-0.06839	0.36936	-0.09931	0.204524	6771050	5.907712	5.974852	6.099603	6.08481	6.134352	5.968162	6.029728	6.198206	6.052171	6.158287	6.006353	6.100605	Mdm1	NM_148922
6771052	1292	5.884263	6.053245	5.985844	6.078605	-0.19434	0.176424	-0.02536	0.851399	-0.09276	0.499207	0.067401	0.620981	-0.16898	0.233328	-0.10158	0.460614	6771052	5.943048	6.084435	5.625306	6.136608	6.102074	5.921054	5.920142	5.867274	6.170117	6.150631	6.108319	5.976866	Ifng	NM_008337
6771139	1294	8.897993	8.91003	8.878377	9.145683	-0.24769	0.032898	-0.23565	0.039976	-0.26731	0.023985	0.031653	0.750603	-0.01204	0.903527	0.019617	0.843527	6771139	8.863115	8.963569	8.867296	8.921011	8.925561	8.883517	8.978521	8.895451	8.761158	8.99577	9.069345	9.371935	Tmbim4	NM_026617
6771141	1295	7.63386	7.475096	7.446136	7.188525	0.445335	0.054278	0.286571	0.185088	0.257612	0.228656	0.02896	0.887123	0.158764	0.444983	0.187724	0.369979	6771141	7.722611	7.444135	7.734834	7.393882	7.799586	7.23182	7.74467	7.472562	7.121177	7.362832	7.065707	7.137035	Llph	NM_025431
6771158	1296	6.153007	6.02091	5.901378	5.967622	0.185385	0.366906	0.053287	0.790366	-0.06624	0.741356	0.119532	0.554621	0.132098	0.514829	0.25163	0.230436	6771158	6.294598	5.943304	6.22112	5.834375	6.348398	5.879956	5.78129	5.73987	6.182973	6.157669	6.02488	5.720318	1700006J	ENSMUST000000
6771224	1298	10.18673	10.15661	10.20159	10.025	0.161725	0.242098	0.131612	0.334041	0.176585	0.205149	-0.04497	0.734459	0.030113	0.81996	-0.01486	0.910461	6771224	10.18507	10.03743	10.33768	10.10563	10.24373	10.12048	10.42768	10.09774	10.07934	10.23022	9.929873	9.914912	Gns	NM_029364
6771264	1300	6.627904	6.578971	6.630107	6.674353	-0.04645	0.650541	-0.09538	0.362236	-0.04425	0.665894	-0.05114	0.618472	0.048933	0.633445	-0.0022	0.98274	6771264	6.729906	6.638551	6.515254	6.705496	6.61126	6.420156	6.618006	6.701698	6.570617	6.71105	6.798537	6.513472	BC048403	BC065058
6771334	1302	8.988889	8.767553	8.84589	8.549844	0.439045	0.015333	0.217709	0.166347	0.296046	0.072169	-0.07834	0.598721	0.221336	0.160209	0.142999	0.346535	6771334	9.073175	9.00088	8.892613	8.720968	9.070631	8.511061	8.801053	8.853182	8.883436	8.709432	8.586915	8.353186	Ppm1h	NM_001110218
6771476	1304	7.551324	7.866149	7.637084	7.924532	-0.37321	0.100249	-0.05838	0.778714	-0.28745	0.190311	0.229065	0.287128	-0.31483	0.155684	-0.08576	0.680685	6771476	7.478311	7.613271	7.56239	7.888278	8.086616	7.623553	7.785788	7.89352	7.231943	7.846401	7.733605	8.19359	Lrig3	NM_177152
6771526	1305	8.702321	8.627991	8.630649	8.61781	0.084511	0.327708	0.010181	0.903167	0.012839	0.878101	-0.00266	0.974653	0.07433	0.386047	0.071673	0.402467	6771526	8.758382	8.743304	8.605278	8.720275	8.73568	8.428018	8.584592	8.637543	8.669811	8.623293	8.595213	8.634924	Ctdsp2	NM_001113470
6771533	1307	7.882543	7.934811	8.238869	8.452269	-0.56973	0.008642	-0.51746	0.013863	-0.2134	0.231837	-0.30406	0.102505	-0.05227	0.75944	-0.35633	0.062758	6771533	7.613618	7.968225	8.065786	7.932158	7.936058	7.936217	7.991185	8.599939	8.125483	8.470034	8.507298	8.379476	Mettl1	NM_010792
6771535	1308	11.78687	11.6945	11.81228	11.41148	0.37539	0.133134	0.283117	0.242922	0.400797	0.112126	-0.11768	0.614452	0.092273	0.691928	-0.02541	0.912708	6771535	11.794	12.00994	11.55667	11.71568	11.73469	11.63342	11.77494	11.62058	12.04131	11.60466	11.70152	10.89246	Cyp27b1	NM_010009
6771538	1309	10.87857	11.02509	10.8917	10.73529	0.14328	0.149999	0.2898	0.012231	0.156407	0.120392	0.133393	0.176528	-0.14652	0.142126	-0.01313	0.887631	6771538	10.83084	10.81898	10.98589	10.93907	11.17736	10.95884	11.00702	10.91345	10.75462	10.6448	10.77884	10.78223	Cdk4	NM_009870
6771546	1310	9.84145	9.560039	9.626602	9.135034	0.706415	0.004151	0.425004	0.044187	0.491568	0.024723	-0.06656	0.718436	0.281411	0.152918	0.214848	0.262362	6771546	10.02776	9.781187	9.715402	9.458226	9.945467	9.276423	9.50737	9.526328	9.846108	9.079541	9.23468	9.090882	B4galnt1	NM_008080
6771558	1311	9.515527	9.500232	9.402761	9.301625	0.213902	0.08458	0.198607	0.105046	0.101136	0.379294	0.097472	0.395974	0.015295	0.891559	0.112767	0.329796	6771558	9.496667	9.480032	9.569883	9.466436	9.667732	9.366529	9.575424	9.416959	9.215899	9.172144	9.364052	9.368679	Dctn2	NM_027151
6771560	1312	7.608814	7.761255	7.663295	7.85245	-0.24364	0.385238	-0.0912	0.739869	-0.18916	0.496072	0.09796	0.721497	-0.15244	0.581314	-0.05448	0.842407	6771560	7.501039	7.655405	7.669999	7.617671	7.76117	7.904929	7.273114	8.324109	7.392661	8.128775	7.781119	7.647457	Ddit3	NM_007837
6771566	1313	6.479459	6.467009	6.46151	6.209894	0.269565	0.029025	0.257115	0.035119	0.251617	0.038211	0.005498	0.958139	0.01245	0.905424	0.017949	0.864069	6771566	6.661865	6.440449	6.336063	6.382481	6.643915	6.37463	6.550744	6.443909	6.389878	6.272167	6.211476	6.146038	Arhgap9	NM_146011
6771568	1314	9.135043	9.213084	9.153557	9.186935	-0.05189	0.659482	0.026149	0.823462	-0.03338	0.776041	0.059527	0.613945	-0.07804	0.510885	-0.01851	0.874389	6771568	9.165182	9.141528	9.098419	9.345318	9.206314	9.087619	8.994934	9.368701	9.097036	9.120715	9.082933	9.357157	R3hdm2	NM_027900
6771575	1316	9.156784	9.177217	9.142986	9.037265	0.119519	0.119131	0.139952	0.075281	0.105721	0.16127	0.034231	0.630681	-0.02043	0.773057	0.013798	0.845367	6771575	9.183886	9.182167	9.1043	9.145789	9.245758	9.140104	9.062145	9.129527	9.237286	8.93893	9.172986	8.999879	Stat6	NM_009284
6771578	1317	6.641407	6.687482	6.515334	6.317859	0.323548	0.080354	0.369623	0.051562	0.197476	0.256675	0.172147	0.31804	-0.04608	0.782886	0.126072	0.457945	6771578	6.676577	6.558712	6.688931	6.501036	6.888526	6.672883	6.761209	6.172342	6.612452	6.393191	6.406429	6.153956	Tmem194	NM_001113211
6771583	1319	6.895458	6.985286	6.904	6.802323	0.093135	0.410042	0.182963	0.126095	0.101677	0.370427	0.081286	0.469826	-0.08983	0.426164	-0.00854	0.938416	6771583	6.816582	6.945117	6.924676	6.950809	7.141779	6.863271	6.922994	7.045409	6.743598	6.673171	6.95787	6.775929	Zbtb39	NM_198035
6771620	1327	7.475664	7.429124	7.221575	6.796847	0.678817	0.0575	0.632277	0.072851	0.424728	0.202919	0.207549	0.517091	0.04654	0.882981	0.254089	0.430801	6771620	7.25444	7.378024	7.794527	7.075147	7.803045	7.409179	7.817076	6.659234	7.188414	6.741149	6.932652	6.716739	Prim1	NM_008921
6771623	1328	6.683776	6.729975	6.570641	6.598418	0.085358	0.346185	0.131557	0.161496	-0.02778	0.753006	0.159334	0.098661	-0.0462	0.602766	0.113135	0.221258	6771623	6.702487	6.608514	6.740327	6.589382	6.751654	6.848889	6.569734	6.462902	6.679288	6.524809	6.557292	6.713152	Ptges3	NM_019766
6771627	1329	11.96069	12.11494	12.00084	12.31201	-0.35132	0.013005	-0.19706	0.112346	-0.31116	0.022627	0.1141	0.331968	-0.15426	0.200208	-0.04016	0.725695	6771627	11.87432	11.92676	12.08098	12.1284	12.04701	12.16942	12.133	11.97767	11.89186	12.14561	12.24647	12.54394	Atp5b	NM_016774
6771628	1330	8.652021	8.667209	8.555164	8.464261	0.18776	0.164205	0.202948	0.136463	0.090903	0.479637	0.112045	0.387505	-0.01519	0.904472	0.096857	0.452315	6771628	8.722205	8.633983	8.599876	8.657907	8.756318	8.587402	8.408668	8.864545	8.392279	8.408864	8.561637	8.422283	Baz2a	NM_054078
6771636	1332	6.17399	6.281789	6.067802	5.987733	0.186257	0.260068	0.294056	0.092024	0.080069	0.616445	0.213987	0.201248	-0.1078	0.502882	0.106188	0.509107	6771636	6.180788	6.087642	6.25354	6.063553	6.472727	6.309088	6.292871	5.783413	6.127123	5.805808	6.081275	6.076117	Timeless	NM_001136082
6771641	1335	8.118923	8.02308	8.074586	7.635632	0.483291	0.009207	0.387447	0.025658	0.438954	0.014704	-0.05151	0.725616	0.095844	0.517805	0.044337	0.762324	6771641	8.068937	8.156023	8.13181	7.788244	8.329143	7.951852	8.07725	7.95958	8.186928	7.569319	7.827663	7.509915	Stat2	NM_019963
6771643	1336	8.013137	8.17673	8.099194	8.088793	-0.07566	0.119439	0.087937	0.077298	0.010401	0.816608	0.077536	0.111812	-0.16359	0.005468	-0.08606	0.082664	6771643	7.953985	8.047082	8.038343	8.197141	8.166224	8.166824	8.11193	8.085485	8.100167	7.995245	8.0955	8.175633	Usp52	NM_133992
6771644	1337	9.410928	9.518775	9.40227	9.646833	-0.2359	0.189505	-0.12806	0.458748	-0.24456	0.175448	0.116505	0.498958	-0.10785	0.530522	0.008658	0.95932	6771644	9.304227	9.441984	9.486574	9.341111	9.531391	9.683824	9.557806	9.436478	9.212527	9.497404	9.445049	9.998045	Cnpy2	NM_019953
6771647	1338	10.3358	10.40971	10.32335	10.51235	-0.17655	0.214374	-0.10264	0.455576	-0.189	0.186787	0.08636	0.527965	-0.07391	0.587831	0.012453	0.926549	6771647	10.42465	10.15763	10.42512	10.39347	10.4605	10.37515	10.49237	10.27453	10.20314	10.26343	10.54839	10.72522	Cs	NM_026444
6771655	1339	8.443085	8.424932	8.25039	8.15918	0.283905	0.053915	0.265752	0.067521	0.09121	0.488922	0.174542	0.202563	0.018153	0.888786	0.192696	0.163968	6771655	8.472502	8.353662	8.503092	8.424241	8.622741	8.227814	8.215293	8.482861	8.053015	8.150117	8.21348	8.113943	Ankrd52	NM_172790
6771658	1340	8.055658	8.260258	8.108454	8.117648	-0.06199	0.625648	0.14261	0.27682	-0.00919	0.941871	0.151804	0.249331	-0.2046	0.132613	-0.0528	0.677127	6771658	8.129207	8.128571	7.909196	8.395075	8.259787	8.125912	8.083678	8.237973	8.00371	8.031529	7.971874	8.349542	Rnf41	NM_026259
6771662	1341	9.991687	10.07444	10.05992	9.928119	0.063567	0.44549	0.146324	0.101933	0.131798	0.134739	0.014526	0.859077	-0.08276	0.326719	-0.06823	0.414145	6771662	10.01199	9.985311	9.977759	10.09745	10.13944	9.98644	10.03665	10.23058	9.912523	9.84343	9.967962	9.972966	Smarcc2	NM_001114097
6771698	1343	8.725799	8.512873	8.747939	8.574671	0.151129	0.081916	-0.0618	0.439598	0.173268	0.052053	-0.23507	0.014811	0.212926	0.023122	-0.02214	0.778188	6771698	8.669936	8.718404	8.789058	8.561028	8.578439	8.399152	8.793009	8.808181	8.642627	8.575258	8.460777	8.687977	Wibg	NM_030100
6771703	1344	7.488944	7.436685	6.96714	7.019507	0.469437	0.088596	0.417178	0.123295	-0.05237	0.834235	0.469545	0.088535	0.052259	0.834572	0.521805	0.063342	6771703	7.762991	7.548174	7.155668	7.43249	7.662931	7.214634	7.007584	6.526144	7.367691	6.828828	7.15146	7.078234	Mmp19	NM_021412
6771704	1345	8.562255	8.605757	8.472497	8.457552	0.104704	0.14351	0.148205	0.050821	0.014945	0.822745	0.13326	0.072896	-0.0435	0.519426	0.089758	0.201901	6771704	8.525334	8.566401	8.595031	8.586015	8.727536	8.50372	8.574313	8.465136	8.378042	8.453129	8.495541	8.423985	Dnajc14	NM_028873
6771718	1346	6.783638	6.680924	6.623196	6.382388	0.40125	0.011916	0.298536	0.042573	0.240808	0.087913	0.057729	0.653752	0.102714	0.431226	0.160443	0.231546	6771718	6.790345	6.9054	6.65517	6.692528	6.753163	6.597082	6.698873	6.454708	6.716006	6.211283	6.632083	6.303798	Itga7	NM_008398
6771862	1368	8.338629	8.541374	8.46078	8.389503	-0.05087	0.862127	0.151871	0.606941	0.071277	0.807939	0.080594	0.783536	-0.20275	0.495101	-0.12215	0.678116	6771862	8.273873	8.721955	8.020059	8.402076	8.739883	8.482163	8.598939	8.380736	8.402664	7.830487	8.945639	8.392382	Myct1	NM_026793
6771884	1369	6.680597	6.472705	6.541971	6.350098	0.330498	0.063467	0.122607	0.447496	0.191873	0.246625	-0.06927	0.663792	0.207891	0.212632	0.138626	0.392848	6771884	6.406767	6.750923	6.8841	6.401281	6.657372	6.359463	6.753199	6.314325	6.558389	6.273583	6.344317	6.432395	Esr1	NM_007956
6771894	1370	6.08749	6.022076	6.023219	6.060861	0.026629	0.773419	-0.03878	0.675907	-0.03764	0.684836	-0.00114	0.990116	0.065414	0.48528	0.064271	0.492701	6771894	6.141704	6.016469	6.104297	6.055625	6.043501	5.967102	5.808448	6.189588	6.07162	6.126139	6.012121	6.044322	1700052N	NM_024261
6771912	1371	7.118324	6.799445	6.56786	6.778857	0.339467	0.021371	0.020588	0.866911	-0.211	0.11408	0.231586	0.087461	0.318879	0.027916	0.550465	0.001695	6771912	6.980748	7.057262	7.316963	6.677124	6.963763	6.757449	6.646986	6.546587	6.510006	6.798971	6.605474	6.932126	Akap12	NM_031185
6771920	1372	6.623366	6.741594	6.337613	6.576005	0.047361	0.831032	0.16559	0.462971	-0.23839	0.299366	0.403981	0.096828	-0.11823	0.597106	0.285753	0.220132	6771920	6.448042	6.320146	7.101909	6.633924	6.910289	6.68057	6.587389	6.123282	6.302168	6.686454	6.644175	6.397385	Mthfd1l	NM_172308
6771927	1373	12.04749	11.99811	11.98204	12.12202	-0.07453	0.522628	-0.1239	0.298672	-0.13998	0.244694	0.016073	0.888926	0.049377	0.669563	0.06545	0.573336	6771927	12.07339	12.1697	11.89938	11.97844	11.81248	12.20342	11.90822	12.06788	11.97002	12.08163	12.14097	12.21525	Iyd	NM_027391
6771954	1374	8.059789	8.216257	8.143921	8.142374	-0.08258	0.527287	0.073883	0.570705	0.001547	0.990424	0.072336	0.578636	-0.15647	0.24592	-0.08413	0.519781	6771954	8.114168	8.027752	8.037448	8.187695	8.251794	8.209281	8.409998	8.125485	7.89628	8.011503	8.101398	8.31422	Pcmt1	NM_008786
6771974	1376	9.882813	9.948066	9.949828	9.977999	-0.09519	0.476468	-0.02993	0.820192	-0.02817	0.830579	-0.00176	0.989307	-0.06525	0.622442	-0.06702	0.613228	6771974	9.88695	9.913479	9.84801	9.81162	9.987248	10.04533	10.19353	9.886169	9.769785	9.82065	9.934017	10.17933	BC013529	NM_145418
6771985	1377	9.841238	9.855608	9.801995	9.745624	0.095614	0.378836	0.109983	0.315193	0.056371	0.597862	0.053613	0.615594	-0.01437	0.892122	0.039243	0.712175	6771985	9.833237	9.798181	9.892296	9.733052	10.06852	9.765251	9.886448	9.879837	9.6397	9.649539	9.79222	9.795114	Map3k7ip	NM_138667
6771995	1378	7.291056	7.124033	7.419132	7.634075	-0.34302	0.163335	-0.51004	0.051876	-0.21494	0.36429	-0.2951	0.223178	0.167023	0.476204	-0.12808	0.582302	6771995	7.346407	7.295819	7.230942	7.28931	6.949832	7.132957	7.462554	7.871926	6.922917	7.597807	7.452587	7.851831	Ust	NM_177387
6772009	1379	10.72053	10.74916	10.68568	10.9337	-0.21317	0.14043	-0.18454	0.194391	-0.24802	0.093435	0.063483	0.639138	-0.02864	0.83153	0.034847	0.795875	6772009	10.74969	10.83831	10.57358	10.86711	10.6674	10.71298	10.41094	10.88607	10.76003	10.99244	10.80655	11.00211	Sash1	NM_175155

6772104	1382	6.428296	6.544834	6.596679	5.76991	0.658386	0.011858	0.774924	0.005128	0.826769	0.003583	-0.05184	0.804991	-0.11654	0.581944	-0.16838	0.431191	6772104	6.556046	6.502534	6.226309	6.675396	6.490863	6.468243	6.854026	6.097058	6.838953	5.705185	5.916059	5.688487	Rab32	NM_026405
6772193	1384	9.159005	9.197546	9.121813	9.131731	0.027274	0.792881	0.065814	0.530749	-0.00992	0.923781	0.075732	0.472519	-0.03854	0.711226	0.037192	0.720821	6772193	9.190206	9.248175	9.038634	9.275093	9.086431	9.231113	9.098861	9.280953	8.985626	8.98609	9.1731	9.236004	Utrn	NM_011682
6772217	1385	7.260893	7.184135	7.399498	7.146119	0.114774	0.503808	0.038016	0.822484	0.253379	0.160879	-0.21536	0.225476	0.076758	0.652202	-0.1386	0.422522	6772217	7.408135	7.179992	7.194553	7.178005	7.424418	6.949983	7.675595	7.300877	7.222022	6.953693	7.28947	7.195194	Ltv1	NM_181470
6772231	1386	8.674909	8.841144	8.884879	8.9057	-0.23079	0.019518	-0.06456	0.438853	-0.02082	0.799391	-0.04374	0.596091	-0.16623	0.06918	-0.20997	0.029272	6772231	8.724976	8.712631	8.58712	8.814785	8.906114	8.802532	9.021416	8.827278	8.805943	8.820959	8.852282	9.043858	Pex3	NM_019961
6772232	1387	8.032901	8.081985	8.124077	8.113011	-0.08011	0.466373	-0.03103	0.774636	0.011067	0.918464	-0.04209	0.698334	-0.04908	0.651873	-0.09118	0.409436	6772232	7.973398	8.145901	7.979403	8.090849	8.06801	8.087097	8.222284	8.189913	7.960035	8.036188	7.974698	8.328146	Aig1	NM_025446
6772261	1389	8.449947	8.410612	8.396784	8.338263	0.111684	0.344461	0.072349	0.533392	0.058521	0.612855	0.013828	0.904074	0.039335	0.732594	0.053163	0.645272	6772261	8.486824	8.388768	8.474248	8.359519	8.623989	8.248327	8.538194	8.224115	8.428043	8.234833	8.364688	8.415267	Vta1	NM_025418
6772382	1391	6.913689	6.913901	6.777103	6.794038	0.119652	0.112613	0.119863	0.112073	-0.01693	0.807244	0.136798	0.07599	-0.00021	0.997562	0.136586	0.076363	6772382	7.021418	6.889938	6.829712	6.920199	6.818055	7.003449	6.853144	6.714918	6.763248	6.828893	6.721961	6.831259	Ccdc28a	NM_144820
6772408	1392	6.448609	6.604881	6.438769	6.21284	0.235769	0.319054	0.392041	0.115275	0.225929	0.33843	0.166112	0.475562	-0.15627	0.501288	0.00984	0.965719	6772408	6.551518	6.243969	6.550341	6.531207	6.656821	6.626615	6.690057	6.318758	6.307493	5.875314	6.737878	6.025329	Hebp2	NM_019487
6772417	1394	7.77252	7.805364	7.997507	7.079363	0.693158	0.038908	0.726002	0.032433	0.918144	0.011401	-0.19214	0.513431	-0.03284	0.909835	-0.22499	0.446453	6772417	8.096042	7.816196	7.405323	7.824071	7.873907	7.718115	8.047994	7.92788	8.016647	7.108074	7.650019	6.479995	Tnfaip3	NM_009397
6772482	1396	7.687665	7.910607	7.821234	7.949863	-0.2622	0.155064	-0.03926	0.820072	-0.12863	0.463321	0.089373	0.607105	-0.22294	0.218645	-0.13357	0.446938	6772482	7.589738	7.628273	7.844985	7.810101	7.90249	8.01923	8.055069	7.830925	7.577709	7.850946	7.729029	8.269614	Pex7	NM_008822
6772550	1397	10.79746	11.27514	10.98989	11.20183	-0.40437	0.037392	0.07331	0.663391	-0.21194	0.227774	0.28525	0.116789	-0.47768	0.018594	-0.19243	0.269651	6772550	10.70381	10.92435	10.76422	11.19118	11.19005	11.44419	10.68722	10.98846	11.29399	11.05695	11.14879	11.39975	Pde7b	NM_013875
6772713	1399	7.121601	7.369248	7.255366	7.142126	-0.02053	0.849505	0.227122	0.061943	0.11324	0.311083	0.113882	0.308523	-0.24765	0.045622	-0.13377	0.237324	6772713	7.040012	7.240211	7.084579	7.234758	7.442904	7.430083	7.448137	7.201791	7.11617	7.021054	7.181345	7.223979	Tbpl1	NM_011603
6772736	1401	5.980261	6.191034	5.917772	5.739699	0.240562	0.386033	0.451335	0.12372	0.178074	0.51649	0.273261	0.328111	-0.21077	0.44501	0.062489	0.817745	6772736	5.697585	6.033773	6.209425	6.278007	6.369119	5.925975	6.218249	5.662873	5.872195	5.331597	6.238844	5.648655	Eya4	NM_010167
6772762	1402	6.114877	5.988701	6.348636	6.084926	0.029951	0.90972	-0.09623	0.7167	0.26371	0.332959	-0.35994	0.19724	0.126177	0.635245	-0.23376	0.38774	6772762	6.141747	5.935607	6.267278	6.051615	5.63709	6.277397	6.190456	6.631729	6.223722	6.329758	5.569905	6.355115	Taar6	NM_001010828
6772802	1406	6.809841	7.277325	6.617089	6.183469	0.626372	0.057005	1.093856	0.004673	0.43362	0.162554	0.660236	0.047259	-0.46748	0.135833	0.192752	0.513436	6772802	6.956325	6.85982	6.613378	6.996475	7.838222	6.997278	6.980943	6.228012	6.642311	6.012635	6.482133	6.055638	Enpp1	NM_008813
6772810	1407	6.830349	7.62591	7.134604	6.598076	0.232273	0.536862	1.027834	0.021313	0.536528	0.174471	0.491306	0.209488	-0.79556	0.058094	-0.30425	0.422588	6772810	6.680199	6.640066	7.170783	7.537094	8.151526	7.189109	7.659075	6.790265	6.954472	6.763632	6.047084	6.983512	Enpp3	NM_134005
6772829	1409	6.892662	7.0127	6.942645	6.904981	-0.01232	0.939687	0.107719	0.51411	0.037663	0.817352	0.070055	0.668835	-0.12004	0.468653	-0.04998	0.759537	6772829	6.766056	7.112653	6.799276	7.093561	6.90037	7.044169	7.232955	6.768904	6.826075	6.814088	6.770071	7.130785	Akap7	NM_018747
6772875	1411	7.080435	7.105578	6.95813	6.987846	0.092589	0.465357	0.117732	0.358245	-0.02972	0.811858	0.147448	0.256937	-0.02514	0.840305	0.122305	0.340899	6772875	7.051357	6.956932	7.233017	6.963386	7.369585	6.983763	7.010023	6.936041	6.928326	6.934146	7.120847	6.908545	L3mbtl3	NM_172787
6772906	1412	6.740938	6.636533	6.119442	6.120631	0.620307	0.026437	0.515902	0.053861	-0.00119	0.995973	0.517091	0.053425	0.104405	0.659803	0.621496	0.026225	6772906	6.932602	6.63822	6.651992	6.756964	6.924371	6.228264	6.362298	5.905252	6.090775	5.780292	6.408206	6.173395	Lama2	NM_008481
6773016	1414	6.190409	6.448628	6.632036	6.703229	-0.51282	0.005227	-0.2546	0.09583	-0.07119	0.61201	-0.18341	0.211057	-0.25822	0.091953	-0.44163	0.011295	6773016	6.067257	6.369045	6.134926	6.376133	6.493023	6.476727	6.82156	6.532558	6.541991	6.440351	6.871017	6.79832	Rnf146	NM_001110197
6773018	1415	7.598787	7.843594	7.855393	7.848894	-0.25011	0.023282	-0.0053	0.95418	0.006499	0.943832	-0.0118	0.898258	-0.24481	0.025514	-0.25661	0.020818	6773018	7.700669	7.60372	7.491971	7.706997	7.905769	7.918016	7.963024	7.819405	7.78375	7.72466	7.861684	7.960338	Rnf146	NM_001110197
6773067	1417	6.094863	6.058032	6.054476	6.077217	0.017646	0.853766	-0.01919	0.84121	-0.02274	0.812385	0.003556	0.970343	0.036831	0.701518	0.040387	0.674584	6773067	6.029612	6.173351	6.081626	5.9908	6.121522	6.061773	6.187613	5.915651	6.060164	5.947257	6.246102	6.038291	Trmt11	NM_028604
6773068	1418	7.740137	7.732732	7.709941	7.858214	-0.11808	0.384869	-0.12548	0.357259	-0.14827	0.281705	0.022792	0.863583	0.007404	0.955448	0.030196	0.820061	6773068	7.636511	7.810673	7.773226	7.77568	7.596025	7.826492	7.506122	7.898313	7.725387	7.706055	7.793072	8.075514	Hint3	NM_025798
6773069	1419	6.955235	6.948511	6.951657	6.967647	-0.01241	0.934107	-0.01914	0.898604	-0.01599	0.915191	-0.00315	0.983277	0.006723	0.964272	0.003577	0.980985	6773069	6.936807	7.101087	6.82781	6.968423	7.045616	6.831495	6.855858	7.155612	6.843502	6.98066	7.21598	6.7063	Ncoa7	NM_172495
6773072	1420	7.247447	7.419078	7.065798	6.657319	0.590128	0.159479	0.761759	0.080242	0.408479	0.314297	0.35328	0.380282	-0.17163	0.663885	0.181649	0.645817	6773072	6.94216	7.485241	7.31494	6.838925	8.236688	7.181621	6.791974	7.236835	7.168585	6.777082	7.036152	6.158724	Hey2	NM_013904
6773080	1421	7.553933	8.020464	7.707661	8.075871	-0.52194	0.013811	-0.05541	0.747471	-0.36821	0.057631	0.312803	0.096655	-0.46653	0.022969	-0.15373	0.38214	6773080	7.460772	7.557522	7.643506	8.047027	8.060404	7.95396	7.847063	7.657102	7.618818	8.0208	7.733446	8.473367	Tpd52l1	NM_009413
6773088	1422	5.536764	6.067889	5.966379	5.569187	-0.03242	0.948622	0.498701	0.336409	0.397192	0.438943	0.10151	0.84031	-0.53112	0.30782	-0.42962	0.404028	6773088	4.960222	5.578559	6.071511	6.346613	5.970108	5.886945	5.793938	6.004651	6.100548	5.888398	6.387253	4.431911	Supt4h1	NM_009296
6773158	1425	7.020778	7.032611	6.970057	6.911946	0.108832	0.19962	0.120665	0.159703	0.058111	0.476712	0.062554	0.444839	-0.01183	0.882939	0.050721	0.532927	6773158	7.034163	7.110998	6.917174	6.900004	7.161412	7.036418	6.973507	7.055653	6.881011	6.876204	6.963623	6.896012	Zufsp	NM_028287
6773164	1426	8.931045	8.981845	8.843225	8.893554	0.037491	0.813815	0.088291	0.582245	-0.05033	0.752237	0.13862	0.394415	-0.0508	0.75001	0.08782	0.58422	6773164	8.914711	8.857848	9.020575	8.875163	9.04471	9.025661	9.128417	8.903051	8.498206	8.718071	9.024024	8.938566	Rwdd1	NM_025614
6773169	1427	6.231185	5.96533	5.772852	5.551818	0.679367	0.041694	0.413512	0.178591	0.221035	0.453334	0.192478	0.511913	0.265855	0.370906	0.458332	0.140844	6773169	6.271238	6.333828	6.088488	5.832067	6.328785	5.735138	6.342987	5.403409	5.572161	5.186172	5.679293	5.789988	Fam26e	NM_178908
6773174	1429	6.33603	6.083331	5.898889	5.174382	1.161647	0.020934	0.908948	0.055158	0.724506	0.111591	0.184442	0.661089	0.252699	0.550246	0.437141	0.312137	6773174	6.5964	6.128622	6.283067	5.768305	6.704409	5.777278	6.50923	5.44949	5.737946	4.755974	5.839216	4.927957	Dse	NM_172508
6773179	1430	7.826461	7.755015	7.786967	7.743927	0.082534	0.321167	0.011088	0.890537	0.04304	0.596377	-0.03195	0.692991	0.071446	0.386727	0.039494	0.626479	6773179	7.909032	7.776093	7.794257	7.777138	7.785524	7.702383	7.864946	7.760165	7.735789	7.589151	7.740198	7.902432	Nt5dc1	NM_176968
6773438	1435	7.269347	7.311479	7.459589	7.336263	-0.06692	0.512157	-0.02478	0.805869	0.123326	0.241768	-0.14811	0.167448	-0.04213	0.677246	-0.19024	0.086987	6773438	7.329165	7.341856	7.137021	7.254506	7.404222	7.275708	7.576003	7.406698	7.396066	7.22934	7.253335	7.526113	Gtf3c6	NM_026113
6773485	1436	8.106847	8.302038	8.267762	8.132446	-0.0256	0.832155	0.169593	0.184915	0.135317	0.280456	0.034276	0.776833	-0.19519	0.133534	-0.16092	0.205968	6773485	8.098423	8.20385	8.018268	8.166037	8.446603	8.293475	8.468913	8.079551	8.254823	7.989336	8.218663	8.189338	Cdc40	NM_027879
6773494	1438	7.970266	8.107939	8.088377	7.856589	0.113677	0.387674	0.25135	0.078075	0.231788	0.099524	0.019563	0.878977	-0.13767	0.300733	-0.11811	0.370337	6773494	7.91269	7.991732	8.006377	8.018438	8.280354	8.025026	8.313875	7.862653	8.088602	7.707492	7.908951	7.953324	Fig4	NM_133999
6773504	1439	8.021085	8.18347	8.084898	8.190199	-0.16911	0.406	-0.00673	0.973015	-0.1053	0.599865	0.098572	0.622991	-0.16238	0.424136	-0.06381	0.749176	6773504	7.801809	7.927689	8.333758	7.967114	8.58598	7.997317	8.083445	8.028621	8.142628	8.132589	8.083758	8.354251	Smpd2	NM_009213
6773545	1441	9.670877	9.682387	9.583984	9.758023	-0.08715	0.453907	-0.07564	0.513811	-0.17404	0.154614	0.098403	0.40006	-0.01151	0.919759	0.086893	0.455174	6773545	9.560268	9.687798	9.764564	9.722036	9.826593	9.498532	9.414325	9.679492	9.658135	9.800304	9.628375	9.84539	Foxo3	NM_019740
6773546	1442	7.015609	6.941795	6.964207	6.992191	0.023418	0.832024	-0.0504	0.649801	-0.02798	0.800034	-0.02241	0.839119	0.073814	0.509262	0.051402	0.643381	6773546	7.002957	6.967882	7.075988	6.901485	7.022106	6.901794	6.91603	7.103596	6.872995	6.821333	6.923653	7.231587	Lace1	NM_145743
6773582	1444	6.327301	6.283785	6.210792	6.276894	0.050407	0.625348	0.00689	0.946373	-0.0661	0.524262	0.072993	0.483204	0.043517	0.672751	0.116509	0.274335	6773582	6.262207	6.242558	6.477139	6.384496	6.289913	6.176945	6.349087	6.258832	6.024457	6.21306	6.325382	6.292241	Sobp	NM_175407
6773622	1445	6.895897	6.857693	6.697233	7.005436	-0.10954	0.593048	-0.14774	0.474344	-0.3082	0.155987	0.16046	0.438499	0.038204	0.850923	0.198664	0.342328	6773622	6.821188	6.812789	7.053715	6.73752	6.917575	6.917984	6.779652	7.085864	6.226183	6.943385	7.137134	6.935788	1700021F	NM_026411
6773629	1446	6.83856	6.896485	6.887851	6.930024	-0.09146	0.138007	-0.03354	0.562431	-0.04217	0.469177	0.008635	0.880233	-0.05793	0.327188	-0.04929	0.400444	6773629	6.799041	6.825811	6.890828	6.861627	6.986849	6.84098	6.842309	6.962341	6.858902	6.844666	6.954479	6.990927	Qrsl1	NM_001081054
6773633	1447	7.622151	7.390531	7.227554	6.876023	0.746128	0.155274	0.514509	0.310796	0.351531	0.480884	0.162978	0.740633	0.231619	0.639268	0.394597	0.430711	6773633	7.763011	7.5236	7.579841	7.113212	7.917987	7.140395	7.706831	6.796476	7.179354	6.523937	7.963298	6.140833	Aim1	NM_172393
6773655	1448	7.434691	7.291618	7.594027	7.386728	0.047963	0.826867	-0.09511	0.665927	0.207299	0.357279	-0.30241	0.191993	0.143073	0.51921	-0.15934	0.47428	6773655	7.497405	7.679429	7.127239	7.263431	7.383019	7.228405	7.448756	7.710765	7.622559	6.947657	7.754996	7.457531	Prdm1	NM_007548
6773940	1452	8.01636	8.090411	7.979863	8.043617	-0.02726	0.738188	0.046794	0.568805	-0.06375	0.441612	0.110548	0.198005	-0.07405	0.374587	0.036497	0.655396	6773940	8.00251	8.074915	7.971655	8.006096	8.207195	8.057942	7.939991	8.090648	7.908949	7.990263	8.179477	7.961111	Gopc	NM_053187
6774006	1454	6.211632	6.109779	6.228527	6.155256	0.056376	0.668219	-0.04548	0.728995	0.073271	0.579066	-0.11875	0.376177	0.101854	0.444795	-0.01689	0.897242	6774006	6.097485	6.390502	6.14691	6.128322	6.170446	6.030568	6.377301	5.964765	6.343514	6.048944	6.283711	6.133113	Mcm9	NM_027830
6774020	1455	7.882122	7.983234	7.761352	7.318222	0.563899	0.075563	0.665012	0.042714	0.443129	0.147417	0.221883	0.445136	-0.10111	0.723882	0.12077	0.673592	6774020	8.240914	7.744659	7.660792	7.876595	8.305189	7.767919	8.059478	7.445887	7.77869	7.034611	7.812661	7.107395	Man1a	NM_008548
6774146	1457	10.53941	10.64664	10.62144	10.59424	-0.05483	0.568478	0.052407	0.58535	0.027203	0.775464	0.025203	0.7915	-0.10724	0.278299	-0.08203	0.399562	6774146	10.49455	10.59225	10.53142	10.5678	10.67714	10.69499	10.82302	10.46472	10.57658	10.47835	10.63993	10.66443	Serinc1	NM_019760
6774226	1459	7.987909	8.120832	7.984069	7.580326	0.407583	0.126656	0.540506	0.053673	0.403743	0.129771	0.136763	0.583038	-0.13292	0.59346	0.00384	0.98758	6774226	7.908989	8.04217	8.012568	7.950728	8.254519	8.157249	8.461919	7.493064	7.997224	7.378085	7.901849	7.461044	10-Sep	NM_001024911
6774264	1461	8.344129	8.960661	8.402352	8.780519	-0.43639	0.040806	0.180141	0.344025	-0.37817	0.067751	0.558309	0.014297	-0.61653	0.0088	-0.05822	0.753491	6774264	8.221558	8.169269	8.641559	8.828091	9.30974	8.744151	8.566014	8.21427	8.426771	8.798615	8.798134	8.744809	Ddit4	NM_029083
6774274	1462	8.430598	8.639245	8.559139	8.65895	-0.22835	0.012588	-0.0197	0.789364	-0.09981	0.199292	0.080106	0.293997	-0.20865	0.019142	-0.12854	0.109212	6774274	8.315139	8.447185	8.529469	8.528659	8.745728	8.643349	8.504789	8.653956	8.518672	8.660666	8.6422	8.673984	D10Ertd6	NM_025514
6774309	1465	9.792859	9.643208	9.8812	9.375663	0.417196	0.009959	0.267545	0.06342	0.505537	0.003587	-0.23799	0.091722	0.149651	0.26278	-0.08834	0.497234	6774309	9.915602	9.715288	9.747688	9.614489	9.797012	9.518123	9.969168	9.965812	9.70862	9.171551	9.391752	9.563686	Slc29a3	NM_023596
6774311	1466	7.48446	7.627237	7.491997	7.241659	0.2428	0.272046	0.385578	0.097915	0.250338	0.258565	0.13524	0.529601	-0.14278	0.507533	-0.00754	0.971685	6774311	7.49523	7.671547	7.286602	7.543518	7.840349	7.497845	7.459716	7.424729	7.591547	6.788499	7.612224	7.324255	Unc5b	NM_029770
6774336	1467	9.869439	10.00559	10.23046	9.773089	0.096351	0.699721	0.232505	0.362844	0.457368	0.094241	-0.22486	0.378021	-0.13615	0.587529	-0.36102	0.172446	6774336	9.979617	9.840385	9.788316	10.28375	9.882081	9.85095	10.21837	10.51692	9.956079	9.484859	9.544027	10.29038	Sgpl1	NM_009163
6774343	1468	8.615443	8.655742	8.591599	8.206968	0.408475	0.000585	0.448774	0.000315	0.38463	0.00086	0.064144	0.414273	-0.0403	0.603266	0.023845	0.757104	6774343	8.683439	8.582163	8.580728	8.614535	8.74843	8.604262	8.68732	8.502026	8.58545	8.115658	8.344788	8.160459	X99384	NM_013753
6774346	1469	8.88233	8.785371	8.777843	8.741376	0.140954	0.302425	0.043996	0.739693	0.036467	0.782765	0.007528	0.954501	0.096958	0.47009	0.104487	0.437557	6774346	8.808053	8.812072	9.026864	8.937335	8.916664	8.502115	8.646334	8.938901	8.748294	8.753512	8.732577	8.738038	Eif4ebp2	NM_010124
6774371	1471	7.290637	7.324153	7.19882	7.422936	-0.1323	0.129089	-0.09878	0.242013	-0.22412	0.020944	0.125333	0.147602	-0.03352	0.679472	0.091817	0.274033	6774371	7.363281	7.344722	7.163908	7.395873	7.233366	7.343221	7.34269	7.109362	7.144408	7.374878	7.436491	7.457438	Col13a1	NM_007731
6774379	1472	7.731242	7.707334	7.752408	7.609383	0.121859	0.125086	0.097951	0.205861	0.143025	0.079228	-0.04507	0.544045	0.023908	0.745471	-0.02117	0.773655	6774379	7.666022	7.694018	7.833686	7.832366	7.597067	7.692569	7.787879	7.783763	7.685581	7.627122	7.531915	7.669112	2010107G	NM_027251
6774384	1473	9.62447	9.596814	9.766197	9.571792	0.052678	0.740557	0.025022	0.874679	0.194405	0.241405	-0.16938	0.302362	0.027656	0.861636	-0.14173	0.383311	6774384	9.585163	9.693537	9.594711	9.674393	9.447732	9.668317	9.641377	9.921558	9.735655	9.222888	9.647477	9.845011	Tspan15	NM_197996
6774391	1474	8.865255	8.635459	8.685416	8.762907	0.102349	0.5907	-0.12745	0.505228	-0.07749	0.682655	-0.04996	0.791455	0.229796	0.243966	0.179839	0.353802	6774391	9.057071	8.696028	8.842667	8.850889	8.679703	8.375786	8.772238	8.689185	8.594826	8.427273	8.800107	9.06134	Hk1	NM_010438
6774395	1476	7.301674	7.374747	7.330374	7.303661	-0.00199	0.979021	0.071086	0.360263	0.026713	0.724818	0.044374	0.561468	-0.07307	0.347715	-0.0287	0.705445	6774395	7.255362	7.273722	7.375937	7.311456	7.498824	7.313962	7.418522	7.346964	7.225635	7.212556	7.375588	7.322839	Supv3l1	NM_181423
6774399	1477	9.003069	9.059763	9.16495	9.019376	-0.01631	0.881668	0.040388	0.713381	0.145574	0.207309	-0.10519	0.350553	-0.05669	0.607647	-0.16188	0.165608	6774399	8.91975	9.066126	9.023332	8.95266	9.174534	9.052096	9.38169	9.126646	8.986514	8.926445	9.119845	9.011837	Vps26a	NM_133672

6774400	1478	11.54633	11.5338	11.45825	11.1572	0.38913	0.080784	0.376597	0.089236	0.301043	0.160783	0.075553	0.708205	0.012533	0.950272	0.088087	0.663099	6774400	11.93953	11.60742	11.09205	11.60095	11.49567	11.50478	11.62766	11.35232	11.39476	11.24939	11.22736	10.99486	Srgn	NM_011157
6774403	1479	8.844419	8.906695	8.881714	8.950948	-0.10653	0.203905	-0.04425	0.581296	-0.06923	0.394849	0.024981	0.753941	-0.06228	0.442041	-0.0373	0.641128	6774403	8.718158	8.857218	8.957882	8.879683	8.941956	8.898447	8.898684	8.99162	8.754839	8.885873	8.931935	9.035036	2510003E	NM_028197
6774404	1480	8.244656	8.170495	8.363458	8.160821	0.083834	0.527148	0.009674	0.941067	0.202636	0.148742	-0.19296	0.166611	0.07416	0.574814	-0.1188	0.376279	6774404	8.353947	8.168118	8.211902	8.089081	8.382079	8.040326	8.484892	8.384829	8.220652	8.008616	8.370024	8.103824	Ddx21	NM_019553
6774405	1481	7.749065	7.925658	7.715535	8.202027	-0.45296	0.003383	-0.27637	0.036448	-0.48649	0.002239	0.210123	0.092916	-0.17659	0.147614	0.03353	0.768622	6774405	7.865365	7.84224	7.539591	7.88557	8.06501	7.826393	7.748914	7.848105	7.549586	8.225667	8.161839	8.218575	Ddx50	NM_053183
6774408	1482	9.199035	9.3042	9.157789	9.114863	0.084171	0.257199	0.189336	0.025279	0.042925	0.551139	0.146411	0.066601	-0.10517	0.165937	0.041246	0.566481	6774408	9.194777	9.173601	9.228726	9.173042	9.460411	9.279146	9.165699	9.166874	9.140793	9.066873	9.207601	9.070116	Ccar1	NM_026201
6774451	1484	7.560578	7.708713	7.536292	7.668646	-0.10807	0.188848	0.040067	0.608838	-0.13235	0.116611	0.172422	0.051138	-0.14814	0.0845	0.024287	0.755133	6774451	7.528206	7.615812	7.537717	7.66108	7.779168	7.685892	7.722631	7.434023	7.452221	7.68421	7.622476	7.699253	Sirt1	NM_019812
6774659	1486	10.15244	10.16264	10.13381	10.16545	-0.01301	0.908924	-0.00281	0.98029	-0.03165	0.781302	0.028837	0.8002	-0.0102	0.928513	0.018633	0.869937	6774659	10.04956	10.21295	10.19481	10.00512	10.16613	10.31668	10.1515	10.12454	10.12538	9.988847	10.38396	10.12355	Reep3	NM_178606
6774676	1487	7.889464	7.887786	7.808794	7.902919	-0.01346	0.926812	-0.01513	0.917722	-0.09413	0.525908	0.078992	0.593098	0.001678	0.990858	0.08067	0.58542	6774676	8.130433	7.613826	7.924133	7.776707	8.047602	7.839049	7.913525	7.666316	7.84654	8.048506	7.800081	7.860171	Nrbf2	NM_001036293
6774684	1488	7.036588	7.051419	7.057392	6.749368	0.28722	0.06716	0.302051	0.056641	0.308024	0.052881	-0.00597	0.965967	-0.01483	0.915656	-0.0208	0.881942	6774684	7.125999	7.025616	6.958149	6.938648	7.053632	7.161976	7.297972	6.999168	6.875035	6.535138	6.760687	6.952278	Ado	NM_001005419
6774719	1490	9.146229	8.260163	8.174893	7.75469	1.39154	0.001212	0.505473	0.113703	0.420204	0.178157	0.085269	0.772165	0.886067	0.014389	0.971336	0.009194	6774719	9.479477	9.087223	8.871988	8.26484	8.560878	7.95477	8.652906	7.680768	8.191006	7.504272	8.006397	7.7534	Arid5b	NM_023598
6774738	1491	7.791704	8.093535	7.744035	9.23165	-1.43995	0.008173	-1.13812	0.02467	-1.48761	0.006904	0.349499	0.421296	-0.30183	0.485062	0.047668	0.910816	6774738	7.755666	7.916649	7.702796	8.228284	7.714354	8.337966	7.113549	8.228993	7.889564	8.827796	8.764514	10.10264	1700040L	BC087900
6774784	1492	7.583771	7.475646	7.405045	7.367121	0.216649	0.063332	0.108524	0.311949	0.037924	0.71588	0.0706	0.502537	0.108125	0.313619	0.178725	0.11341	6774784	7.62079	7.633078	7.497444	7.470642	7.392921	7.563374	7.422859	7.495918	7.296359	7.402802	7.157935	7.540627	A930033H	AK044689
6774794	1493	6.195574	6.440733	5.724929	5.325502	0.870073	0.037468	1.115232	0.01275	0.399428	0.285885	0.715804	0.074592	-0.24516	0.502668	0.470645	0.214764	6774794	6.270001	5.802311	6.514411	5.998787	7.142089	6.181324	6.149829	5.253813	5.771146	5.379042	5.159437	5.438026	Cdc2a	NM_007659
6774875	1494	8.118484	7.749678	7.721411	7.255531	0.862953	0.054043	0.494146	0.232446	0.46588	0.257909	0.028266	0.942903	0.368807	0.363162	0.397073	0.329559	6774875	8.535694	7.953793	7.865966	7.472045	8.221859	7.555129	7.418351	7.830398	7.915485	7.477124	7.829437	6.460033	Phyhipl	NM_178621
6774907	1495	7.947784	7.949443	7.159295	6.776223	1.171561	0.005959	1.17322	0.005914	0.383072	0.259741	0.790148	0.03683	-0.00166	0.995936	0.788489	0.037133	6774907	8.150979	8.097196	7.595176	7.778332	8.425013	7.644984	7.543303	6.812721	7.12186	6.30434	7.18215	6.842178	Bicc1	NM_031397
6774934	1496	6.556186	6.683378	6.571744	6.600216	-0.04403	0.63293	0.083163	0.37585	-0.02847	0.756415	0.111635	0.243624	-0.12719	0.189444	-0.01556	0.865108	6774934	6.561779	6.590604	6.516174	6.615395	6.768845	6.665895	6.740129	6.565465	6.409637	6.480684	6.619741	6.700222	Tfam	NM_009360
6774937	1497	9.537619	9.444457	9.475593	9.263286	0.274333	0.063238	0.181172	0.192411	0.212307	0.133853	-0.03114	0.812899	0.093162	0.485076	0.062026	0.639093	6774937	9.428739	9.453983	9.730135	9.391575	9.676914	9.264883	9.457673	9.47203	9.497076	9.254272	9.423484	9.112101	Ube2d1	NM_145420
6774947	1498	9.072901	9.078595	8.976969	9.38684	-0.31394	0.247893	-0.30825	0.255864	-0.40987	0.142344	0.101626	0.697179	-0.00569	0.98252	0.095932	0.713225	6774947	9.058438	8.845626	9.314639	9.222297	9.038328	8.975159	9.219557	9.238883	8.472466	9.132924	9.249783	9.777813	Cisd1	NM_134007
6774995	1499	6.657411	6.622344	7.072711	6.86328	-0.20587	0.218369	-0.24094	0.156603	0.209432	0.211245	-0.45037	0.019225	0.035067	0.825714	-0.4153	0.027297	6774995	6.618987	6.458685	6.894562	6.452545	6.482217	6.93227	7.196511	6.956974	7.064649	6.780355	6.858952	6.950532	1700049L	NR_003644
6775136	1500	6.528357	6.880764	6.65598	6.260805	0.267552	0.07671	0.619959	0.001528	0.395176	0.017068	0.224784	0.126296	-0.35241	0.028124	-0.12762	0.360968	6775136	6.471435	6.506766	6.60687	6.894207	7.160779	6.587306	6.622073	6.740474	6.605394	6.193686	6.383851	6.204877	AI646023	NM_198860
6775146	1501	9.337672	9.801055	9.072484	8.852099	0.485573	0.231685	0.948957	0.03529	0.220386	0.573122	0.728571	0.08808	-0.46338	0.251829	0.265187	0.499735	6775146	9.266512	9.307273	9.43923	9.724986	10.40662	9.27156	9.740387	8.496513	8.980553	9.002922	9.092519	8.460855	Susd2	NM_027890
6775147	1502	7.937563	7.891399	7.866239	7.927288	0.010275	0.915872	-0.03589	0.713279	-0.06105	0.535297	0.025161	0.796254	0.046163	0.637425	0.071324	0.470893	6775147	7.922647	7.908273	7.981768	7.867665	7.956294	7.850239	7.799743	8.03623	7.762743	7.817241	8.114404	7.850218	Cabin1	NM_172549
6775148	1503	6.953078	7.193253	6.999762	7.169206	-0.21613	0.293897	0.024047	0.903626	-0.16944	0.404191	0.19349	0.344044	-0.24017	0.247233	-0.04668	0.814396	6775148	7.108792	6.74934	7.001101	7.38361	7.096441	7.099707	6.980263	7.280555	6.738469	7.045477	6.955958	7.506183	Ddt	NM_010027
6775149	1504	9.60046	9.687788	9.742319	9.849644	-0.24918	0.222802	-0.16186	0.415586	-0.10733	0.584792	-0.05453	0.779751	-0.08733	0.655554	-0.14186	0.473335	6775149	9.668362	9.554229	9.578788	9.664166	9.844818	9.554381	9.538436	10.05095	9.63757	9.687405	9.623347	10.23818	Gstt3	NM_133994
6775151	1505	9.137722	9.262881	9.275029	9.423349	-0.28563	0.04687	-0.16047	0.223734	-0.14832	0.257577	-0.01215	0.922931	-0.12516	0.333753	-0.13731	0.291831	6775151	9.055696	9.225459	9.132012	9.156322	9.509698	9.122623	9.25127	9.434445	9.139371	9.317482	9.405339	9.547226	Gstt1	NM_008185
6775155	1507	8.986711	9.023958	8.811266	8.903104	0.083607	0.57852	0.120854	0.426911	-0.09184	0.542532	0.212692	0.17898	-0.03725	0.802957	0.175445	0.259	6775155	9.031777	8.847034	9.081321	8.966516	9.132861	8.972497	8.79821	8.857135	8.778452	8.750342	8.69352	9.26545	Gstt2	NM_010361
6775159	1508	9.416504	9.310861	9.394572	9.379929	0.036574	0.600372	-0.06907	0.333192	0.014642	0.832635	-0.08371	0.247243	0.105642	0.153847	0.021932	0.752036	6775159	9.373599	9.383245	9.492667	9.263517	9.436284	9.232783	9.3752	9.398621	9.409894	9.279799	9.482381	9.377608	Smarcb1	NM_011418
6775160	1509	8.5314	8.636882	8.410529	8.127685	0.403715	0.062774	0.509197	0.026064	0.282844	0.168632	0.226353	0.2604	-0.10548	0.587951	0.120871	0.535907	6775160	8.253631	8.691614	8.648956	8.738436	8.774423	8.397787	8.250873	8.483401	8.497314	7.787094	8.341359	8.254603	Mmp11	NM_008606
6775171	1511	8.987477	9.059409	9.009097	8.761592	0.225885	0.031637	0.297817	0.008989	0.247506	0.021531	0.050311	0.578517	-0.07193	0.431769	-0.02162	0.80977	6775171	8.986266	8.933898	9.042267	9.086311	9.179436	8.912479	9.066474	9.100071	8.860747	8.669951	8.837721	8.777103	Prmt2	NM_133182
6775175	1512	7.436156	7.483771	7.415391	7.374372	0.061784	0.104504	0.109399	0.011846	0.041019	0.258827	0.06838	0.077301	-0.04761	0.195942	0.020765	0.555431	6775175	7.398891	7.465528	7.444049	7.454159	7.522538	7.474615	7.449577	7.380378	7.416217	7.36887	7.433903	7.320343	Pcnt	NM_008787
6775182	1514	8.624367	8.549155	8.21834	8.044012	0.580355	0.032764	0.505143	0.055157	0.174328	0.461128	0.330816	0.180025	0.075212	0.74698	0.406028	0.109055	6775182	8.907871	8.548273	8.416958	8.635696	8.768929	8.242841	8.186901	8.462014	8.006104	7.733258	8.39877	8.000008	Col6a2	NM_146007
6775185	1515	9.049494	9.048376	8.522668	8.276792	0.772701	0.044952	0.771584	0.045194	0.245875	0.471623	0.525709	0.144914	0.001117	0.997345	0.526826	0.144171	6775185	9.337124	9.064592	8.746765	8.91364	9.52098	8.710509	8.855309	8.373781	8.338913	7.746556	8.818064	8.265757	Col6a1	NM_009933
6775192	1516	7.114974	7.186478	7.222657	7.349503	-0.23453	0.015007	-0.16302	0.064343	-0.12685	0.133783	-0.03618	0.646893	-0.0715	0.374483	-0.10768	0.194403	6775192	7.067121	7.146258	7.131543	7.301162	7.179047	7.079225	7.121547	7.352938	7.193485	7.371979	7.416692	7.259837	Pcbp3	NM_021568
6775201	1517	8.757311	9.126355	8.794918	9.032078	-0.27477	0.180267	0.094277	0.628032	-0.23716	0.240744	0.331437	0.114535	-0.36904	0.084108	-0.03761	0.845762	6775201	8.954342	8.596092	8.721498	8.991224	9.417636	8.970206	8.989152	8.738328	8.657275	8.970247	8.77913	9.346858	Col18a1	NM_001109991
6775206	1518	6.868701	7.100447	6.736369	6.905787	-0.03709	0.772263	0.194659	0.15471	-0.16942	0.208581	0.364078	0.018732	-0.23175	0.098271	0.132332	0.316547	6775206	6.934274	6.999172	6.672656	7.024174	7.276492	7.000674	6.858325	6.681345	6.669436	6.930571	7.058166	6.728625	Adarb1	NR_004429
6775213	1519	7.530014	7.654516	7.531149	7.494111	0.035903	0.732564	0.160405	0.152511	0.037037	0.724519	0.123367	0.258636	-0.1245	0.254631	-0.00113	0.991353	6775213	7.520528	7.510147	7.559368	7.703716	7.643356	7.616476	7.609485	7.72489	7.259071	7.500253	7.476639	7.505442	1810008A	NM_133998
6775235	1520	7.618852	7.731844	7.688964	7.662999	-0.04415	0.602307	0.068844	0.422211	0.025965	0.757886	0.04288	0.612596	-0.11299	0.202487	-0.07011	0.414071	6775235	7.722165	7.516751	7.617639	7.7036	7.717976	7.773955	7.79531	7.77275	7.498832	7.662735	7.687105	7.639158	Lrrc3	NM_145152
6775236	1521	6.035161	5.707254	5.869563	5.588523	0.446638	0.012936	0.118731	0.422037	0.28104	0.08015	-0.16231	0.280743	0.327907	0.047642	0.165598	0.271822	6775236	6.186676	5.805755	6.113052	5.731089	5.839729	5.550945	6.023278	5.753695	5.831717	5.803797	5.525906	5.435866	Trpm2	NM_138301
6775238	1522	8.038909	8.11383	8.039006	8.342782	-0.30387	0.020183	-0.22895	0.061068	-0.30378	0.020212	0.074824	0.49685	-0.07492	0.496309	-9.7E-05	0.999286	6775238	7.960469	8.097013	8.059244	8.151479	8.079279	8.110732	8.212052	7.993392	7.911573	8.494324	8.130359	8.403663	Pfkl	NM_008826
6775240	1523	6.254808	6.206973	6.274508	6.346085	-0.09128	0.362997	-0.13911	0.179733	-0.07158	0.47109	-0.06754	0.495704	0.047835	0.626829	-0.0197	0.840287	6775240	6.240625	6.171102	6.352698	6.350875	6.093368	6.176676	6.266304	6.311305	6.245916	6.516394	6.188879	6.332982	Aire	NM_009646
6775242	1524	8.109544	8.206089	8.090141	8.51712	-0.40758	0.063796	-0.31103	0.139519	-0.42698	0.054386	0.115948	0.557795	-0.09655	0.624333	0.019402	0.921006	6775242	7.966423	8.075445	8.286763	8.316025	8.183402	8.11884	8.254898	8.290001	7.725525	8.270818	8.457199	8.823343	D10Jhu81	NM_138601
6775246	1525	6.798216	6.763108	6.823487	6.684018	0.114197	0.217492	0.079089	0.381008	0.139469	0.140724	-0.06038	0.499192	0.035108	0.691489	-0.02527	0.774599	6775246	6.768925	6.840902	6.78482	6.688512	6.892783	6.708028	6.891641	6.802258	6.776563	6.515308	6.835715	6.701032	Pwp2	NM_029546
6775250	1526	9.494101	9.62638	9.473976	10.14284	-0.64874	0.01246	-0.51646	0.033965	-0.66887	0.010737	0.152404	0.472656	-0.13228	0.531377	0.020124	0.923177	6775250	9.439891	9.491365	9.551046	9.619679	9.450782	9.80868	9.247895	9.917304	9.25673	9.915121	10.10213	10.41128	Agpat3	NM_053014
6775257	1527	9.745787	9.908476	9.805488	9.824802	-0.07901	0.514765	0.083674	0.491008	-0.01931	0.871822	0.102988	0.400281	-0.16269	0.198132	-0.0597	0.620507	6775257	9.719422	9.719325	9.798615	9.81041	9.994335	9.920683	10.03056	9.784755	9.601149	9.739996	9.73242	10.00199	Rrp1	NM_010925
6775258	1528	9.863318	9.830467	10.10411	10.13602	-0.27271	0.179624	-0.30556	0.138035	-0.03191	0.867655	-0.27365	0.178292	0.032851	0.863798	-0.2408	0.23031	6775258	9.716052	9.933652	9.94025	9.892042	9.655857	9.943502	9.878243	10.5556	9.878496	10.00192	10.18992	10.21623	Pdxk	NM_172134
6775288	1531	7.433075	7.572173	7.596229	7.618557	-0.18548	0.202649	-0.04638	0.737522	-0.02233	0.871473	-0.02406	0.861648	-0.1391	0.328445	-0.16315	0.256966	6775288	7.266686	7.619236	7.413302	7.340453	7.595121	7.780946	7.620819	7.604358	7.563509	7.438072	7.668577	7.749022	2610008E	BC082302
6775298	1532	9.632811	9.346475	9.478617	9.072653	0.560158	0.01724	0.273822	0.181522	0.405964	0.061863	-0.13214	0.500117	0.286336	0.164491	0.154194	0.433796	6775298	9.626974	9.448384	9.823075	9.238118	9.588898	9.21241	9.799772	9.25851	9.377569	8.832766	9.274197	9.110997	Ppap2c	NM_015817
6775299	1533	7.322498	7.354801	7.244271	7.23878	0.083719	0.176737	0.116021	0.074138	0.005492	0.92497	0.110529	0.086187	-0.0323	0.58326	0.078227	0.203633	6775299	7.328162	7.259868	7.379465	7.315552	7.477828	7.271022	7.285142	7.259354	7.188318	7.222462	7.215364	7.278513	Mier2	NM_027422
6775302	1536	6.306334	6.337302	6.250478	6.257551	0.048783	0.610056	0.079751	0.41091	-0.00707	0.94056	0.086824	0.372568	-0.03097	0.744881	0.055856	0.560291	6775302	6.258769	6.363154	6.297079	6.429173	6.345471	6.237261	6.207028	6.428686	6.115719	6.246116	6.150766	6.37577	Shc2	NM_001024539
6775309	1537	7.69299	7.716597	7.680146	7.7178	-0.02481	0.702868	-0.0012	0.985172	-0.03765	0.56503	0.036451	0.577268	-0.02361	0.716516	0.012844	0.842914	6775309	7.693395	7.661734	7.72384	7.705118	7.789361	7.655311	7.694875	7.797164	7.548398	7.70224	7.678151	7.773008	Polrmt	NM_172551
6775310	1538	9.70332	9.59056	9.709038	9.848123	-0.1448	0.397705	-0.25756	0.150688	-0.13908	0.415777	-0.11848	0.485632	0.11276	0.506305	-0.00572	0.972721	6775310	9.768518	9.599604	9.741838	9.704062	9.719454	9.348165	9.503195	10.04586	9.578059	9.691578	9.945638	9.907153	Rnf126	NM_144528
6775319	1540	7.611385	7.586629	7.516921	7.667265	-0.05588	0.535661	-0.08064	0.377713	-0.15034	0.11984	0.069708	0.442842	0.024755	0.781635	0.094463	0.305809	6775319	7.679026	7.613095	7.542033	7.674118	7.651527	7.434243	7.611463	7.554329	7.384972	7.640839	7.590471	7.770485	Med16	NM_198107
6775322	1541	8.673542	8.68838	8.666439	8.623507	0.050035	0.439899	0.064873	0.322796	0.042932	0.505353	0.021941	0.73077	-0.01484	0.815613	0.007103	0.910989	6775322	8.683467	8.599851	8.737309	8.671862	8.819508	8.57377	8.663276	8.644669	8.691372	8.602573	8.592003	8.675946	C030046I	NM_177994
6775329	1542	9.43582	9.413911	9.375943	9.422782	0.013037	0.850413	-0.00887	0.897825	-0.04684	0.503874	0.037968	0.586086	0.021909	0.751814	0.059877	0.397108	6775329	9.463161	9.352576	9.491722	9.365746	9.548137	9.32785	9.386483	9.447447	9.293899	9.378051	9.433884	9.456412	ORF61	NM_001003949
6775335	1543	8.383974	8.435992	8.566938	8.307294	0.076679	0.309984	0.128698	0.106376	0.259644	0.006307	-0.13095	0.101319	-0.05202	0.483141	-0.18296	0.032297	6775335	8.266745	8.391256	8.49392	8.462732	8.44266	8.402585	8.463478	8.570396	8.666941	8.222001	8.368193	8.331689	Polr2e	NM_025554
6775337	1544	8.570064	8.378262	8.33912	7.950977	0.619087	0.005673	0.427284	0.032411	0.388143	0.046875	0.039141	0.818828	0.191802	0.279513	0.230944	0.200043	6775337	8.856228	8.50164	8.352324	8.307121	8.612493	8.215171	8.526914	8.228471	8.261976	7.872956	8.139994	7.839982	Sbno2	NM_183426
6775355	1545	7.070998	7.001736	6.846255	6.529962	0.541036	0.029022	0.471774	0.049282	0.316293	0.159208	0.155481	0.467345	0.069262	0.742679	0.224742	0.302123	6775355	7.016985	6.835555	7.360453	7.056319	7.272405	6.676484	7.016378	6.751766	6.770622	6.34319	6.821046	6.42565	Gamt	NM_010255
6775363	1546	7.30262	7.259337	7.347645	7.299463	0.003157	0.979682	-0.04013	0.74697	0.048182	0.698884	-0.08831	0.483302	0.043283	0.727972	-0.04503	0.71757	6775363	7.173237	7.302383	7.43224	7.067488	7.506082	7.204442	7.389996	7.439404	7.213535	7.381636	7.276136	7.240618	2310011J	NM_025521
6775364	1547	6.381632	6.45063	6.330057	6.599403	-0.21777	0.171154	-0.14877	0.334451	-0.26935	0.100023	0.120573	0.429351	-0.069	0.646586	0.051575	0.731014	6775364	6.441061	6.327607	6.376228	6.512063	6.261953	6.577873	6.28989	6.580456	6.119824	6.772102	6.377944	6.648163	Pcsk4	NM_008793
6775367	1548	7.501201	7.444746	7.280277	7.179295	0.321906	0.039738	0.265451	0.077701	0.100983	0.463668	0.164468	0.245455	0.056455	0.67838	0.220923	0.130752	6775367	7.535732	7.347624	7.620246	7.569389	7.585657	7.179191	7.228794	7.23952	7.372518	7.122414	7.356206	7.059264	Adamtsl5	NM_001113548
6775369	1549	7.226298	7.368417	7.037741	7.117955	0.108343	0.471509	0.250462	0.118788	-0.08021	0.591149	0.330677	0.049969	-0.14212	0.350595	0.188558	0.224903	6775369	7.191192	7.212223	7.27548	7.175602	7.465375	7.464275	6.956954	7.284629	6.871639	7.219003	6.871639	7.263224	Mex3d	NM_198615

6775370	1550	8.60229	8.665176	8.634235	8.654241	-0.05195	0.220972	0.010936	0.786995	-0.02001	0.623011	0.030941	0.451948	-0.06289	0.146733	-0.03194	0.437959	6775370	8.557244	8.626102	8.623524	8.625984	8.664204	8.705341	8.665579	8.67426	8.562866	8.599964	8.674916	8.687842	Mbd3	NM_013595
6775373	1551	8.365999	8.407624	8.395296	8.22672	0.139279	0.400451	0.180904	0.282044	0.168576	0.313814	0.012328	0.93928	-0.04163	0.797419	-0.0293	0.856476	6775373	8.456793	8.337779	8.303425	8.428182	8.482037	8.312654	8.339108	8.751924	8.094856	8.054543	8.34597	8.279648	Tcfe2a	NM_011548
6775377	1553	7.993059	8.039901	8.141642	8.064983	-0.07192	0.595814	-0.02508	0.852064	0.076658	0.572319	-0.10174	0.457129	-0.04684	0.72837	-0.14858	0.286871	6775377	7.975154	7.986804	8.017218	8.014846	8.201643	7.903215	8.026367	8.4513	7.947258	7.983685	8.115646	8.095619	Rexo1	NM_025852
6775379	1554	7.984741	7.988085	7.984465	8.009902	-0.02516	0.767713	-0.02182	0.797724	-0.02544	0.76525	0.00362	0.966004	-0.00334	0.968597	0.000276	0.997404	6775379	8.061745	7.860599	8.03188	8.065992	8.036074	7.86219	7.91878	8.110585	7.92403	8.031453	8.066162	7.93209	Klf16	NM_078477
6775380	1555	7.170768	7.293768	7.150653	7.186624	-0.01586	0.836301	0.107144	0.187149	-0.03597	0.641169	0.143115	0.09017	-0.123	0.136324	0.020115	0.793395	6775380	7.286406	7.121439	7.104458	7.300634	7.404674	7.175996	7.168741	7.224746	7.058471	7.12629	7.214403	7.219178	Fam108a	BC005632
6775389	1556	9.463632	9.432986	9.366049	9.352053	0.111579	0.519107	0.080933	0.637882	0.013996	0.934668	0.066937	0.696419	0.030646	0.857675	0.097583	0.571638	6775389	9.258238	9.425744	9.706914	9.436274	9.627548	9.235137	9.407566	9.284899	9.405683	9.162208	9.241076	9.652875	Mknk2	NM_021462
6775391	1557	8.109725	7.908532	8.119903	7.715969	0.393756	0.099838	0.192563	0.38946	0.403935	0.092717	-0.21137	0.347146	0.201193	0.369594	-0.01018	0.962819	6775391	8.174601	8.048758	8.105816	7.995327	8.218709	7.511559	8.074255	8.197152	8.088303	7.872591	7.971065	7.30425	Mobkl2a	NM_172457
6775392	1558	9.949627	9.970745	10.0245	9.934776	0.01485	0.85496	0.035968	0.659649	0.089726	0.287007	-0.05376	0.513655	-0.02112	0.795133	-0.07488	0.369032	6775392	10.00245	9.969172	9.877258	9.969888	10.0645	9.877846	9.987957	10.18845	9.897099	9.890871	9.933456	9.980002	Ap3d1	NM_007460
6775401	1559	8.256456	8.185993	8.333837	8.370793	-0.11434	0.396323	-0.1848	0.185519	-0.03696	0.779437	-0.14784	0.279955	0.070463	0.595836	-0.07738	0.560988	6775401	8.284687	8.214963	8.269718	8.139504	8.476178	7.942296	8.231372	8.47342	8.296718	8.318571	8.472494	8.321315	Plekhj1	NM_023900
6775402	1560	7.172106	6.985031	7.101466	7.138337	0.033769	0.801546	-0.15331	0.272026	-0.03687	0.783832	-0.11643	0.396444	0.187075	0.187966	0.07064	0.601558	6775402	7.150605	7.047995	7.317719	7.092849	6.780284	7.081961	7.072092	7.050643	7.181663	7.256741	6.889724	7.268547	Jsrp1	NM_028001
6775407	1561	6.477475	6.441207	6.381208	6.49724	-0.01976	0.855988	-0.05603	0.609596	-0.11603	0.303188	0.059999	0.58499	0.036268	0.73976	0.096267	0.387975	6775407	6.614517	6.335272	6.482637	6.410648	6.321906	6.591067	6.393915	6.475948	6.273762	6.391589	6.451678	6.648454	Lingo3	NM_001013758
6775410	1562	7.478994	7.578893	7.438005	7.621815	-0.14282	0.243364	-0.04292	0.714899	-0.18381	0.143691	0.140888	0.249265	-0.0999	0.40403	0.040989	0.727114	6775410	7.446066	7.559481	7.431435	7.470847	7.57061	7.695222	7.615156	7.479615	7.219243	7.559849	7.525361	7.780236	Timm13	NM_013895
6775411	1563	6.607059	6.549663	6.543644	6.594279	0.012779	0.727503	-0.04462	0.243149	-0.05064	0.190564	0.006019	0.869236	0.057396	0.143673	0.063415	0.111069	6775411	6.638749	6.614952	6.567475	6.537721	6.51431	6.596957	6.609408	6.543419	6.478104	6.595922	6.584594	6.602322	Lmnb2	NM_010722
6775412	1564	6.877577	6.800778	6.589252	6.703157	0.174419	0.265195	0.09762	0.521411	-0.11391	0.456526	0.211526	0.184324	0.076799	0.612157	0.288325	0.082998	6775412	6.977727	6.764195	6.890808	6.806431	6.885493	6.710409	6.7174	6.611092	6.439263	6.54527	6.51792	7.045832	Gng7	NM_001038655
6775414	1566	7.348609	7.355338	7.418992	7.41786	-0.06925	0.275932	-0.06252	0.321945	0.001132	0.985218	-0.06365	0.313807	-0.00673	0.91233	-0.07038	0.268742	6775414	7.274784	7.308599	7.462443	7.369384	7.346259	7.350372	7.471897	7.358354	7.426726	7.320343	7.488708	7.44453	Slc39a3	NM_134135
6775415	1567	8.808156	8.763482	8.762488	8.85591	-0.04775	0.730871	-0.09243	0.510004	-0.09342	0.505594	0.000994	0.994267	0.044674	0.747499	0.045668	0.742119	6775415	8.709044	8.757265	8.958159	8.813929	8.83187	8.644646	8.963746	8.6083	8.715418	8.614944	8.922623	9.030164	Sgta	NM_024499
6775430	1569	7.795573	7.703757	7.768263	7.657122	0.138451	0.208495	0.046635	0.657215	0.111141	0.304081	-0.06451	0.541689	0.091816	0.390797	0.02731	0.794107	6775430	7.716436	7.75897	7.911314	7.688997	7.901918	7.520356	7.761645	7.883695	7.659449	7.681005	7.608253	7.682108	Pias4	NM_021501
6775449	1571	8.002444	8.054147	7.801366	8.109903	-0.10746	0.536559	-0.05576	0.746227	-0.30854	0.10087	0.252781	0.167274	-0.0517	0.763993	0.201078	0.261463	6775449	7.989866	7.976366	8.041101	8.076918	8.064318	8.021206	8.058998	7.960301	7.3848	7.974351	8.044253	8.311104	Mrpl54	NM_025317
6775451	1572	8.628627	8.56012	8.669708	8.401037	0.227589	0.146771	0.159083	0.293958	0.268671	0.094431	-0.10959	0.461364	0.068506	0.641593	-0.04108	0.779165	6775451	8.721596	8.531613	8.632671	8.686208	8.566655	8.427498	8.761229	8.857753	8.390143	8.271367	8.320891	8.610854	Tjp3	NM_013769
6775456	1573	6.832644	6.832836	6.839281	6.940843	-0.1082	0.21084	-0.10801	0.211576	-0.10156	0.237484	-0.00644	0.937416	-0.00019	0.998127	-0.00664	0.93555	6775456	6.898993	6.845148	6.75379	6.885208	6.761964	6.851442	6.843939	6.84721	6.826694	6.956384	6.765078	7.101067	Gipc3	NM_148951
6775457	1574	8.219542	8.391073	8.349887	8.285524	-0.06598	0.587396	0.105549	0.392289	0.064363	0.596424	0.041186	0.733327	-0.17153	0.179896	-0.13034	0.296556	6775457	8.298885	8.130278	8.229464	8.418558	8.421906	8.332756	8.451457	8.5401	8.058105	8.195643	8.318521	8.342408	Hmg20b	NM_010440
6775460	1575	8.166023	8.200884	8.111329	8.06237	0.103653	0.301107	0.138514	0.177869	0.04896	0.615719	0.089555	0.367507	-0.03486	0.71971	0.054694	0.575768	6775460	8.113927	8.130432	8.25371	8.098819	8.389507	8.114326	8.185175	8.121496	8.027317	7.937808	8.171886	8.077415	Fzr1	NM_019757
6775465	1576	8.058409	7.932926	7.942303	7.724567	0.333842	0.148393	0.208359	0.347395	0.217736	0.327366	-0.00938	0.965268	0.125484	0.564356	0.116107	0.593244	6775465	8.070122	7.94561	8.159496	8.13453	8.147204	7.517043	7.829573	8.071695	7.92564	7.381214	8.025927	7.76656	Nfic	NM_008688
6775466	1577	8.846098	8.701949	8.596953	8.49232	0.353778	0.157206	0.209629	0.382113	0.104634	0.656655	0.104996	0.655558	0.144149	0.54258	0.249144	0.303681	6775466	8.981085	8.721183	8.836025	8.922116	8.914012	8.269719	8.4569	8.921746	8.412214	8.233761	8.767984	8.475214	Nfic	NM_008688
6775470	1578	7.910788	7.899339	7.889856	7.965153	-0.05437	0.456161	-0.06581	0.370919	-0.0753	0.309736	0.009483	0.89473	0.011448	0.87312	0.020932	0.770735	6775470	7.895765	7.880684	7.955914	7.854428	7.992419	7.851171	7.89122	7.860509	7.917839	7.801802	8.04832	8.045337	Ncln	NM_134009
6775472	1580	6.196321	6.200649	6.206805	5.83636	0.359961	0.140039	0.364289	0.13595	0.370446	0.130323	-0.00616	0.978335	-0.00433	0.984769	-0.01048	0.963115	6775472	6.329098	6.236738	6.023126	6.176462	6.404213	6.021271	6.349064	6.151863	6.119489	5.562992	6.368659	5.577428	Gna15	NM_010304
6775473	1581	9.322156	9.357882	9.320376	9.401614	-0.07946	0.365494	-0.04373	0.611834	-0.08124	0.355418	0.037506	0.662713	-0.03573	0.677623	0.00178	0.983381	6775473	9.224164	9.319077	9.423227	9.318307	9.460498	9.29484	9.399225	9.381934	9.179969	9.323468	9.502122	9.379253	Gna11	NM_010301
6775478	1582	6.442864	6.657771	6.41823	6.587806	-0.14494	0.188546	0.069965	0.507426	-0.16958	0.131137	0.239541	0.044859	-0.21491	0.065668	0.024633	0.813157	6775478	6.31442	6.440715	6.573456	6.77232	6.643995	6.556999	6.505187	6.388784	6.36072	6.429422	6.578592	6.755405	Tle6	NM_053254
6775480	1583	7.729655	7.782254	7.737981	7.607176	0.122479	0.140455	0.175078	0.04751	0.130805	0.118715	0.044273	0.570579	-0.0526	0.502222	-0.00833	0.914185	6775480	7.656595	7.761836	7.770535	7.808743	7.878194	7.659824	7.83617	7.735406	7.642367	7.564673	7.708457	7.548398	Sirt6	NM_181586
6775502	1584	7.711625	7.688159	7.716472	7.897753	-0.18613	0.243383	-0.20959	0.193893	-0.18128	0.254842	-0.02831	0.852851	0.023465	0.877781	-0.00485	0.97464	6775502	7.814609	7.697784	7.622481	7.468998	7.593595	8.001885	7.690591	7.879998	7.578826	7.740636	7.92848	8.024144	B230315N	BC040349
6775521	1588	6.967035	7.137029	7.081411	6.904994	0.062041	0.535999	0.232034	0.041962	0.176416	0.103262	0.055618	0.5781	-0.16999	0.11439	-0.11438	0.267404	6775521	6.917456	6.979262	7.004387	7.047253	7.252937	7.110896	7.204695	7.05602	6.983517	6.721689	7.061716	6.931578	Nfyb	NM_010914
6775559	1589	10.25308	10.00392	10.17538	9.948224	0.304853	0.077972	0.055696	0.721574	0.227161	0.170554	-0.17147	0.28862	0.249157	0.137233	0.077692	0.620489	6775559	10.39595	10.22351	10.13977	10.15496	9.771788	10.08501	10.23256	9.958914	10.33468	9.891092	10.17085	9.782729	Slc41a2	NM_177388
6775561	1590	5.550991	5.996368	5.77567	6.311286	-0.7603	0.000342	-0.31492	0.039049	-0.53562	0.003031	0.220698	0.122422	-0.44538	0.008255	-0.22468	0.116758	6775561	5.511997	5.694204	5.446771	5.861235	5.933119	6.19475	5.756369	5.640157	5.930484	6.31788	6.479269	6.13671	Aldh1l2	NM_153543
6775576	1591	9.95316	10.17261	9.940791	9.75256	0.2006	0.030819	0.420054	0.000588	0.188231	0.039618	0.231823	0.016466	-0.21945	0.021074	0.012369	0.875832	6775576	9.845513	9.949736	10.06423	10.1265	10.13373	10.25761	9.89487	9.979673	9.947829	9.754429	9.625011	9.878239	Appl2	NM_145220
6775625	1592	7.851038	7.752455	7.831295	7.298729	0.552309	0.026895	0.453726	0.057041	0.532567	0.031246	-0.07884	0.709539	0.098583	0.642244	0.019742	0.92537	6775625	7.857165	7.899399	7.796549	7.643988	8.138535	7.474841	7.785142	7.832084	7.87666	6.898648	7.579454	7.418084	Nuak1	NM_001004363
6775674	1595	6.077799	6.117756	6.271669	6.465296	-0.3875	0.074604	-0.34754	0.103383	-0.19363	0.335826	-0.15391	0.439241	-0.03996	0.837933	-0.19387	0.335253	6775674	6.183332	5.940572	6.109492	6.18189	6.21562	5.955759	6.427345	6.415839	5.971823	6.721932	6.586453	6.087502	Cry1	NM_007771
6775741	1596	9.051525	9.14006	9.098547	9.127482	-0.07596	0.421051	0.012578	0.89179	-0.02894	0.754929	0.041514	0.655357	-0.08854	0.351861	-0.04702	0.613819	6775741	8.996525	9.021368	9.136681	9.098681	9.196219	9.125281	9.298449	9.011722	8.985469	9.055568	9.086138	9.240741	D10Wsu52	BC003288
6775750	1598	6.94293	7.060472	6.994546	7.09369	-0.15076	0.218171	-0.03322	0.775897	-0.09914	0.405091	0.065926	0.57505	-0.11754	0.327892	-0.05162	0.659447	6775750	6.828279	6.912905	7.087607	6.998759	7.139433	7.043224	6.7499	7.140609	7.093128	7.097331	7.001135	7.182605	Syn3	NM_013722
6775758	1599	12.50803	12.5244	12.65425	12.63193	-0.1239	0.352453	-0.10753	0.416519	0.022323	0.863252	-0.12985	0.331119	-0.01638	0.899406	-0.14623	0.277552	6775758	12.5752	12.53223	12.41665	12.38747	12.37232	12.81342	12.80624	12.61072	12.5458	12.53897	12.69343	12.66339	Hsp90b1	NM_011631
6775783	1601	7.153257	7.483204	7.273523	7.170125	-0.01687	0.889754	0.313079	0.028988	0.103398	0.405976	0.209681	0.113162	-0.32995	0.023233	-0.12027	0.337472	6775783	7.213311	7.281229	6.96523	7.616777	7.324915	7.50792	7.441916	7.281644	7.097008	7.11504	7.241091	7.154243	Ascl1	NM_008553
6775838	1604	10.36621	10.61581	10.3351	10.51131	-0.1451	0.424628	0.104503	0.561368	-0.17621	0.336847	0.28071	0.142271	-0.2496	0.18587	0.03111	0.861343	6775838	10.30196	10.32719	10.46948	10.27925	10.89006	10.67812	10.59549	10.13142	10.27839	10.61358	10.35995	10.56039	1200002N	NM_027878
6775846	1605	9.635853	9.977099	9.787615	10.40951	-0.77365	0.008009	-0.43241	0.085718	-0.62189	0.022555	0.189484	0.415498	-0.34125	0.160586	-0.15176	0.51106	6775846	9.426672	9.842649	9.638238	10.09141	9.852964	9.986923	9.691812	10.04692	9.624112	10.16147	10.16353	10.90352	Chpt1	NM_144807
6775854	1606	6.903535	6.779499	7.04728	6.819694	0.083841	0.958588	-0.0402	0.980138	0.227586	0.887971	-0.26778	0.868385	0.124036	0.938773	-0.14374	0.929074	6775854	4.291032	6.553222	9.866352	8.318838	7.068202	4.951457	8.404755	6.565762	6.171322	5.019156	8.005642	7.434284	Mybpc1	NM_175418
6775870	1608	6.780264	6.783574	6.844606	6.508933	0.271331	0.092597	0.274641	0.08933	0.335673	0.04579	-0.06103	0.678877	-0.00331	0.981988	-0.06434	0.66271	6775870	6.858477	6.68302	6.799296	6.59288	7.021494	6.736348	7.046667	6.778053	6.709098	6.384639	6.72	6.42216	Utp20	NM_175158
6775888	1612	8.576148	8.614149	8.616849	8.495273	0.080875	0.505878	0.118876	0.335935	0.121576	0.325723	-0.0027	0.98202	-0.038	0.751879	-0.0407	0.735014	6775888	8.605204	8.55783	8.565409	8.387563	8.918593	8.536292	8.578886	8.614656	8.657006	8.428392	8.546984	8.510443	Scyl2	NM_198021
6775891	1613	5.830311	6.088556	5.933261	5.910058	-0.07975	0.386158	0.178498	0.074326	0.023203	0.796454	0.155294	0.112111	-0.25825	0.017921	-0.10295	0.270669	6775891	5.735115	5.943098	5.812719	6.027255	6.201542	6.03687	6.062403	5.923269	5.814112	5.945418	5.98376	5.800996	Actr6	NM_025914
6775977	1614	6.995877	7.133151	6.753903	6.640901	0.354976	0.069384	0.49225	0.019699	0.113002	0.523426	0.379248	0.055499	-0.13727	0.441097	0.241973	0.19096	6775977	7.165855	6.983202	6.838573	7.02898	7.427985	6.942489	6.964894	6.519865	6.776951	6.699231	6.772024	6.451448	Apaf1	NM_001042558
6775983	1615	11.02205	11.09711	11.07779	11.30794	-0.28589	0.005754	-0.21083	0.024915	-0.23015	0.016914	0.01932	0.807139	-0.07505	0.355656	-0.05573	0.487404	6775983	10.97434	10.99396	11.09786	11.05795	11.14292	11.09045	11.08523	11.07694	11.07119	11.18842	11.23293	11.50247	Slc25a3	NM_133668
6775984	1616	8.583341	8.722973	8.631033	8.365125	0.218217	0.180593	0.357849	0.042811	0.265909	0.111676	0.09194	0.553744	-0.13963	0.375402	-0.04769	0.756738	6775984	8.498426	8.533455	8.718143	8.535991	8.939619	8.69331	8.779196	8.441651	8.672253	8.349231	8.59105	8.155093	Tmpo	NM_001080129
6776029	1617	7.613973	7.766405	7.757131	7.542622	0.07135	0.642762	0.223783	0.169101	0.214508	0.185406	0.009275	0.951586	-0.15243	0.333318	-0.14316	0.361885	6776029	7.638694	7.423211	7.780013	7.751598	7.923642	7.623976	7.883708	7.507769	7.879915	7.459006	7.74169	7.427171	Nedd1	NM_008682
6776049	1618	9.425078	9.819159	9.608697	9.286841	0.138237	0.549294	0.532318	0.042691	0.321856	0.183622	0.210462	0.369087	-0.39408	0.112586	-0.18362	0.430415	6776049	9.473788	9.474374	9.327072	9.746243	10.05696	9.654275	9.646956	9.603613	9.575522	9.139879	9.833788	8.886857	Elk3	NM_013508
6776104	1619	9.585944	9.848593	9.527383	9.745315	-0.15937	0.373638	0.103278	0.558397	-0.21793	0.233585	0.32121	0.094096	-0.26265	0.159055	0.058561	0.738096	6776104	9.365335	9.663083	9.729414	9.692825	9.888718	9.964235	9.721704	9.251342	9.609103	9.556517	9.674288	10.00514	Metap2	NM_019648
6776105	1620	7.84229	7.938216	7.910072	7.734339	0.107951	0.28618	0.203876	0.062962	0.175733	0.099883	0.028144	0.773348	-0.09593	0.339605	-0.06778	0.493457	6776105	7.811096	7.772718	7.943056	7.96591	7.969553	7.879184	8.051072	7.705049	7.974095	7.684633	7.849083	7.669302	Vezt	NM_172538
6776152	1622	6.294118	6.945073	6.724067	6.734906	-0.44079	0.009986	0.210167	0.148199	-0.01084	0.936251	0.221006	0.130907	-0.65096	0.001112	-0.42995	0.011291	6776152	6.211223	6.236152	6.434978	7.05268	6.829237	6.953302	6.92029	6.472051	6.779861	6.635904	6.657917	6.910898	4932415G	BC067070
6776156	1623	6.581851	6.612207	6.54118	5.917296	0.664555	0.030419	0.694911	0.025262	0.623884	0.03906	0.071027	0.786177	-0.03036	0.907518	0.040671	0.876356	6776156	6.858065	6.555965	6.331523	6.482856	6.939815	6.41395	6.86109	6.064354	6.698095	5.899155	6.162332	5.690401	Plxnc1	NM_018797
6776173	1624	7.920352	7.7897	7.766249	7.557639	0.362714	0.013827	0.232061	0.079488	0.20861	0.108662	0.023451	0.84424	0.130652	0.290944	0.154104	0.219044	6776173	7.677826	8.052715	8.030516	7.755329	7.830975	7.782796	7.681956	7.705682	7.911108	7.529567	7.705612	7.437737	Cradd	NM_009950
6776185	1625	11.84311	11.4296	11.61227	11.11806	0.725053	0.224426	0.31154	0.587089	0.494217	0.39568	-0.18268	0.74862	0.413513	0.474215	0.230837	0.686113	6776185	11.29294	11.99173	12.24466	10.86685	11.24488	12.17706	11.95992	10.77339	12.10351	11.31121	11.77037	10.27259	Socs2	NM_007706
6776198	1626	8.815033	8.818376	8.372499	8.598061	0.216972	0.516456	0.220315	0.510193	-0.22556	0.500458	0.445878	0.200584	-0.00334	0.991911	0.442534	0.203641	6776198	8.878882	9.045076	8.521141	8.724558	8.94749	8.783081	8.557635	8.254737	8.305124	7.841231	8.707856	9.245097	Nudt4	NM_027722
6776507	1629	7.464849	7.602235	7.568392	7.488413	-0.02356	0.775803	0.113821	0.192555	0.079979	0.346658	0.033843	0.683376	-0.13739	0.124208	-0.10354	0.23162	6776507	7.481704	7.451707	7.461135	7.466829	7.628096	7.711779	7.711627	7.500708	7.492841	7.410662	7.579411	7.475167	4930430F	NM_175128
6776667	1632	6.704119	6.489177	6.580645	6.489596	0.214523	0.182328	-0.00042	0.997794	0.091049	0.552629	-0.09147	0.550845	0.214942	0.181568	0.123474	0.42502	6776667	6.673448	6.601053	6.837857	6.425535	6.595273	6.446724	6.676435	6.54965	6.515851	6.126627	6.656171	6.685991	Tmtc2	NM_177368
6776688	1633	5.968419	5.934929	5.998641	6.036352	-0.06793	0.220573	-0.10142	0.082556	-0.03771	0.481811	-0.06371	0.247941	0.03349	0.5308	-0.03022	0.570748	6776688	6.014507	5.922122	5.968629	5.937489	5.92184	5.945458	5.999102	6.080655	5.916166	5.969422	6.012545	6.127089	BC067068	BC067068

6776784	1636	5.728708	5.154255	5.374266	5.291167	0.437542	0.528873	-0.13691	0.841961	0.0831	0.903597	-0.22001	0.749161	0.574453	0.412639	0.354442	0.608373	6776784	4.801572	5.342685	7.041868	5.958773	4.726731	4.777261	6.11916	5.065056	4.938583	4.954532	6.001178	4.91779	Myf6	NM_008657
6776978	1641	6.665506	6.779282	6.773695	6.791643	-0.12614	0.225516	-0.01236	0.90078	-0.01795	0.856422	0.005587	0.955039	-0.11378	0.270191	-0.10819	0.292657	6776978	6.785597	6.658094	6.552826	6.847805	6.771223	6.718819	6.845957	6.757336	6.717792	6.639036	6.742306	6.993587	Zdhhc17	NM_172554
6777073	1642	7.968553	8.244052	8.356515	7.078426	0.890127	0.030759	1.165626	0.008982	1.278088	0.005555	-0.11246	0.74934	-0.2755	0.441256	-0.38796	0.286896	6777073	8.173137	8.066463	7.666059	8.180703	8.588836	7.962618	8.821692	7.765796	8.482056	6.847785	7.630538	6.756956	Glipr1	NM_028608
6777177	1645	8.698439	8.824494	8.820282	8.755437	-0.057	0.415382	0.069057	0.328477	0.064845	0.357112	0.004212	0.950953	-0.12606	0.094036	-0.12184	0.103669	6777177	8.586767	8.76863	8.73992	8.706172	8.829005	8.938305	8.837645	8.783887	8.839315	8.714247	8.74271	8.809355	Tbc1d15	NM_025706
6777180	1646	9.12397	9.20215	9.071877	9.123831	0.000139	0.997937	0.07832	0.171259	-0.05195	0.347966	0.130274	0.036946	-0.07818	0.171934	0.052093	0.34675	6777180	9.145773	9.136418	9.089718	9.143232	9.247162	9.216057	9.089138	9.119034	9.007458	9.01895	9.145281	9.207261	Rab21	NM_024454
6777181	1647	7.533785	7.50156	7.56811	7.445104	0.088681	0.301072	0.056455	0.501535	0.123005	0.163717	-0.06655	0.430809	0.032226	0.6984	-0.03432	0.680014	6777181	7.435243	7.628017	7.538096	7.423221	7.569452	7.512006	7.656422	7.494475	7.553432	7.296618	7.494407	7.544288	Tmem19	NM_133683
6777182	1648	6.737405	6.744057	6.758124	6.893953	-0.15655	0.378444	-0.1499	0.398067	-0.13583	0.441944	-0.01407	0.935289	-0.00665	0.96937	-0.02072	0.904837	6777182	6.656632	7.079731	6.475851	6.553453	6.789826	6.888891	6.527055	6.900754	6.846564	6.842334	6.94477	6.894756	Thap2	NM_025780
6777190	1649	7.389856	8.583331	7.838228	6.78294	0.606916	0.59674	1.800391	0.140846	1.055288	0.366159	0.745103	0.517893	-1.19348	0.310224	-0.44837	0.694682	6777190	6.8168	8.122285	7.230483	7.5711	10.05144	8.127454	8.61577	5.856908	9.042006	6.469527	8.381723	5.497571	Lgr5	NM_010195
6777251	1651	8.899306	9.070404	9.007778	8.989891	-0.09059	0.32594	0.080513	0.379537	0.017887	0.841457	0.062625	0.490013	-0.1711	0.083496	-0.10847	0.245561	6777251	8.886463	8.979984	8.83147	9.014782	9.128837	9.067592	9.037972	9.147658	8.837705	8.882623	8.99124	9.09581	Cnot2	NM_001037846
6777286	1652	8.914017	9.000621	8.895551	9.117703	-0.20369	0.136627	-0.11708	0.369439	-0.22215	0.108807	0.10507	0.418227	-0.0866	0.501725	0.018466	0.884484	6777286	8.809644	8.896117	9.03629	9.059087	8.937391	9.005386	8.822618	9.099344	8.764692	9.132198	8.905536	9.315374	Rab3ip	NM_001003950
6777296	1654	9.817177	9.888202	9.946103	9.801256	0.01592	0.892688	0.086945	0.468759	0.144847	0.240793	-0.0579	0.626184	-0.07103	0.551709	-0.12893	0.292113	6777296	9.811723	9.781207	9.8586	9.781335	9.96659	9.91668	10.16871	9.712271	9.957328	9.697014	9.767463	9.939292	Cct2	NM_007636
6777297	1655	8.051562	8.22181	8.097135	8.203963	-0.1524	0.104135	0.017847	0.835396	-0.10683	0.234758	0.124675	0.172097	-0.17025	0.074762	-0.04557	0.59853	6777297	7.935702	8.073103	8.14588	8.168426	8.173067	8.323937	8.133028	8.104408	8.05397	8.116199	8.125895	8.369795	Frs2	NM_177798
6777305	1656	8.144788	8.380719	8.127316	8.093307	0.051481	0.586618	0.287412	0.013345	0.03401	0.717967	0.253402	0.023622	-0.23593	0.031815	0.017471	0.852344	6777305	8.149917	8.110037	8.174409	8.306141	8.447824	8.388191	8.326685	8.131526	7.923738	8.032844	8.113796	8.13328	Yeats4	NM_026570
6777309	1658	10.84764	11.08128	11.00607	9.550181	1.297462	0.010973	1.531096	0.004635	1.455892	0.006085	0.075203	0.853383	-0.23363	0.569579	-0.15843	0.698138	6777309	11.22107	10.87121	10.45065	11.10912	11.24498	10.88973	11.35212	10.63051	11.03559	9.912843	10.09067	8.64703	Lyz2	NM_017372
6777310	1659	7.049341	7.80685	7.49988	6.433329	0.616012	0.103254	1.373521	0.003439	1.06655	0.01293	0.30697	0.386322	-0.75751	0.053637	-0.45054	0.215577	6777310	7.604189	6.572597	6.971238	8.310182	7.891135	7.219233	7.615749	7.376373	7.507517	6.645847	6.554522	6.099619	Lyz1	NM_013590
6777315	1660	8.767804	8.658841	8.700603	8.701102	0.066702	0.474679	-0.04226	0.647318	-0.0005	0.995654	-0.04176	0.651157	0.108963	0.255298	0.067201	0.471486	6777315	8.766261	8.772516	8.764635	8.516185	8.836657	8.623682	8.657601	8.714422	8.729785	8.668937	8.853581	8.580789	Cpsf6	NM_001013391
6777346	1661	8.643535	8.601866	8.443773	8.568269	0.075266	0.520061	0.033597	0.7716	-0.1245	0.298115	0.158093	0.195333	0.041668	0.719234	0.199762	0.111999	6777346	8.66775	8.66258	8.600274	8.443685	8.765554	8.59636	8.214012	8.64292	8.474387	8.561759	8.603789	8.539259	Mdm2	NM_010786
6777351	1662	6.199873	6.219715	6.373198	6.310221	-0.11035	0.690945	-0.09051	0.743943	0.062977	0.819884	-0.15348	0.582085	-0.01984	0.942721	-0.17333	0.535378	6777351	6.221697	6.115168	6.262753	6.332543	6.64812	5.678482	6.053689	6.385852	6.680054	6.153131	6.63912	6.138413	Slc35e3	NM_029875
6777353	1663	6.379183	6.52242	6.432961	6.224198	0.154985	0.188146	0.298222	0.024348	0.208763	0.088605	0.089459	0.430334	-0.14324	0.220255	-0.05378	0.631031	6777353	6.26403	6.442339	6.431179	6.372767	6.69336	6.501133	6.616575	6.29439	6.387918	6.159623	6.311296	6.201674	Nup107	NM_134010
6777355	1664	10.82405	10.78124	10.80608	10.7471	0.076953	0.518005	0.034143	0.771821	0.05898	0.618318	-0.02484	0.832713	0.04281	0.716581	0.017973	0.878429	6777355	10.91465	10.90449	10.65301	10.65082	10.95212	10.74078	10.98294	10.75615	10.67914	10.65845	10.82286	10.75998	Rap1b	NM_024457
6777421	1665	8.014703	8.372027	8.205275	8.338637	-0.32393	0.134262	0.03339	0.867911	-0.13336	0.512152	0.166752	0.41604	-0.35732	0.103393	-0.19057	0.355717	6777421	8.031697	7.968707	8.043705	8.460455	8.456075	8.199551	8.207052	8.598846	7.809926	8.184103	8.244533	8.587274	Dyrk2	NM_001014390
6777505	1666	6.659636	6.814639	6.709926	6.512791	0.146844	0.109739	0.301847	0.006062	0.197135	0.04218	0.104713	0.23549	-0.155	0.094137	-0.05029	0.554967	6777505	6.759011	6.577349	6.642547	6.798645	6.867621	6.77765	6.694698	6.650631	6.784449	6.421856	6.694067	6.422451	Helb	NM_080446
6777510	1667	7.724309	7.507021	7.3188	7.047722	0.676587	0.044898	0.459299	0.145576	0.271078	0.369189	0.188221	0.527373	0.217288	0.467518	0.405509	0.192423	6777510	8.044321	7.620443	7.508164	7.707873	7.736837	7.076353	7.629405	6.907718	7.419278	6.799614	7.46114	6.882412	Irak3	NM_028679
6777518	1668	8.597661	8.682691	8.38515	8.428858	0.168803	0.17485	0.253833	0.055527	-0.04371	0.709911	0.297541	0.030445	-0.08503	0.474747	0.212511	0.097749	6777518	8.601625	8.469818	8.721539	8.593457	8.818172	8.636443	8.468517	8.190091	8.496842	8.398451	8.576786	8.311336	Hmga2	NM_010441
6777545	1669	11.18286	11.32238	11.17388	11.01038	0.17248	0.508978	0.311997	0.246502	0.1635	0.530698	0.148497	0.568214	-0.13952	0.591379	0.00898	0.972174	6777545	11.01118	10.91417	11.62323	11.19264	11.35588	11.41861	11.33768	11.02832	11.15564	11.3224	11.19346	10.51528	Msrb3	NM_177092
6777583	1670	8.962215	9.002001	8.772885	8.774129	0.188086	0.224515	0.227872	0.14942	-0.00124	0.993268	0.229116	0.147488	-0.03979	0.787726	0.18933	0.221744	6777583	9.117349	8.887378	8.881917	9.049324	9.053921	8.902758	8.96549	8.805203	8.547962	8.519641	8.837298	8.965447	Rassf3	NM_138956
6777594	1671	8.36409	8.373994	8.257526	8.138412	0.225678	0.031198	0.235582	0.026124	0.119114	0.205862	0.116468	0.215124	-0.0099	0.911679	0.106564	0.253014	6777594	8.464989	8.337747	8.289535	8.296716	8.457765	8.367502	8.402326	8.22984	8.140413	8.051451	8.264227	8.099558	Tbk1	NM_019786
6777614	1672	7.631843	7.83796	7.783628	7.72687	-0.09503	0.500484	0.11109	0.43337	0.056758	0.684542	0.054332	0.697213	-0.20612	0.164449	-0.15179	0.292414	6777614	7.620986	7.717425	7.557118	7.829993	7.980838	7.70305	8.088678	7.601828	7.660378	7.602318	7.762688	7.815604	Tmem5	NM_153059
6777677	1673	9.246004	9.280756	9.292384	9.460265	-0.21426	0.051083	-0.17951	0.091047	-0.16788	0.110211	-0.01163	0.904062	-0.03475	0.719704	-0.04638	0.6331	6777677	9.30034	9.215176	9.222497	9.244617	9.387379	9.210271	9.328499	9.45975	9.088903	9.520322	9.487734	9.372738	Mon2	NM_153395
6777684	1674	8.442862	8.434178	8.464319	8.33738	0.105482	0.246199	0.096798	0.28405	0.126939	0.170549	-0.03014	0.729941	0.008684	0.92049	-0.02146	0.805511	6777684	8.410704	8.360361	8.557521	8.449767	8.537143	8.315624	8.557689	8.455441	8.379827	8.226597	8.443025	8.342518	Usp15	NM_027604
6777879	1676	6.518596	6.57086	6.548712	6.581676	-0.06308	0.088668	-0.01082	0.748241	-0.03296	0.340916	0.022147	0.515514	-0.05226	0.147073	-0.03012	0.381973	6777879	6.497814	6.509758	6.548215	6.605356	6.562705	6.544518	6.493245	6.529245	6.623647	6.596038	6.574233	6.574757	Xrcc6bp1	NM_001159559
6777898	1677	7.367004	7.398558	7.290593	7.349255	0.017749	0.806959	0.049302	0.502809	-0.05866	0.428036	0.107965	0.162979	-0.03155	0.665316	0.076411	0.308526	6777898	7.299515	7.437892	7.363605	7.431187	7.475292	7.289194	7.318926	7.377479	7.175373	7.309818	7.427228	7.31072	Tsfm	NM_025537
6777902	1678	10.60217	10.39288	10.47999	10.67465	-0.07248	0.433957	-0.28178	0.01257	-0.19466	0.057877	-0.08711	0.351168	0.209293	0.044649	0.12218	0.202416	6777902	10.5626	10.60492	10.63899	10.39856	10.55691	10.22316	10.34633	10.54978	10.54386	10.67364	10.73609	10.61423	Tspan31	NM_025982
6777906	1679	10.52062	10.50094	10.48007	10.62106	-0.10044	0.308433	-0.12011	0.229569	-0.14099	0.165337	0.020873	0.826839	0.019677	0.836594	0.04055	0.672193	6777906	10.59631	10.4167	10.54885	10.46536	10.47115	10.56632	10.58396	10.47138	10.38487	10.46629	10.59187	10.80501	Os9	NM_177614
6777915	1680	9.787321	9.943357	10.05845	11.42559	-1.63827	0.030204	-1.48224	0.044615	-1.36715	0.059522	-0.11509	0.858046	-0.15604	0.80856	-0.27112	0.674949	6777915	9.977597	10.29973	9.084637	10.50851	9.17703	10.14453	9.185922	11.32531	9.664106	11.36563	11.01314	11.89801	Slc26a10	BC089587
6777917	1681	7.53096	7.620663	7.473193	7.649557	-0.1186	0.568808	-0.02889	0.88848	-0.17636	0.402682	0.14747	0.481115	-0.0897	0.665054	0.057767	0.779618	6777917	7.692315	7.718716	7.18185	7.783372	7.613956	7.464661	7.448479	7.818055	7.153045	7.548009	7.643158	7.757503	D10Ertd6	NM_028027
6777919	1682	9.797466	9.739368	9.891664	10.18269	-0.38523	0.038304	-0.44333	0.021469	-0.29103	0.098235	-0.1523	0.356172	0.058098	0.718453	-0.0942	0.56154	6777919	9.756941	9.8685	9.766957	9.851556	9.589442	9.777105	9.788176	10.12264	9.764177	10.0005	10.03287	10.51471	Pip4k2c	NM_054097
6777925	1684	8.177148	8.171876	8.202616	8.172851	0.004296	0.981472	-0.00098	0.995792	0.029764	0.872286	-0.03074	0.868145	0.005272	0.977266	-0.02547	0.89057	6777925	8.293222	8.133632	8.104589	8.25714	8.236295	8.022192	7.852546	8.646223	8.109078	8.174427	8.131354	8.212773	Mbd6	NM_033072
6777929	1685	8.083947	8.197517	8.156939	8.092681	-0.00873	0.934468	0.104836	0.338292	0.064258	0.549865	0.040578	0.703744	-0.11357	0.301996	-0.07299	0.498422	6777929	8.143881	7.972817	8.135142	8.110264	8.327476	8.154812	8.301132	8.133167	8.036519	8.02869	8.266583	7.98277	Mars	NM_001003913
6777932	1686	5.938316	5.975087	5.90473	5.901135	0.037181	0.58047	0.073951	0.285078	0.003595	0.956954	0.070356	0.307496	-0.03677	0.584576	0.033586	0.616954	6777932	6.026063	5.885626	5.90326	5.995974	5.987151	5.942135	6.005069	5.874774	5.834348	6.018539	5.84684	5.838027	Gli1	NM_010296
6777955	1689	6.988385	6.860757	6.8566	6.92007	0.068315	0.534662	-0.05931	0.58868	-0.06347	0.563365	0.004157	0.969479	0.127628	0.260073	0.131785	0.246098	6777955	6.97317	6.957672	7.034313	6.790307	7.025905	6.766058	6.898235	6.656073	7.015492	6.80071	6.981423	6.978076	Shmt2	NM_028230
6777957	1691	9.2914	8.907254	8.426806	7.959484	1.331916	0.001958	0.94777	0.012346	0.467322	0.151664	0.480448	0.141887	0.384146	0.228904	0.864594	0.018937	6777957	9.56475	9.352532	8.956918	8.995344	9.298799	8.427619	8.743623	8.270023	8.266771	7.616463	8.390681	7.871308	Lrp1	NM_008512
6777960	1692	9.45873	9.38502	9.252327	9.374786	0.083944	0.582316	0.010234	0.946011	-0.12246	0.427381	0.132693	0.391436	0.07371	0.628356	0.206403	0.196447	6777960	9.400738	9.558715	9.416736	9.434141	9.493883	9.227036	9.103516	9.610834	9.042631	9.328227	9.449053	9.347078	Nab2	NM_008668
6777968	1693	6.690214	7.295613	6.527023	7.180654	-0.49044	0.005781	0.114959	0.407337	-0.65363	0.00109	0.76859	0.000384	-0.6054	0.001743	0.163191	0.249624	6777968	6.913266	6.609193	6.548183	7.430317	7.208746	7.247776	6.560086	6.445792	6.575191	7.27212	7.335352	6.934491	Gpr182	NM_007412
6777994	1696	9.533226	9.650708	9.584888	9.366998	0.166228	0.109207	0.283709	0.015216	0.21789	0.045805	0.065819	0.495795	-0.11748	0.238558	-0.05166	0.590769	6777994	9.488301	9.518235	9.593143	9.608809	9.769382	9.573932	9.403314	9.602641	9.74871	9.269433	9.407475	9.424087	Rbms2	NM_001039080
6777998	1697	7.231133	7.135785	7.115194	7.12507	0.106063	0.513935	0.010715	0.946688	-0.00988	0.950858	0.020591	0.897798	0.095348	0.556305	0.115939	0.476714	6777998	7.014398	7.211933	7.467067	7.083301	7.411572	6.912481	7.236023	7.084465	7.025094	6.989923	7.134805	7.250481	Spryd4	NM_025716
6778027	1699	6.486941	6.514055	6.454435	6.460787	0.026154	0.517453	0.053268	0.205211	-0.00635	0.873454	0.05962	0.161291	-0.02711	0.502619	0.032506	0.42449	6778027	6.529611	6.479505	6.451707	6.517408	6.492318	6.53244	6.466763	6.428316	6.468226	6.540225	6.379381	6.462756	Slc39a5	NM_028051
6778028	1700	9.729793	9.751907	9.750677	9.71737	0.012423	0.943109	0.034537	0.842915	0.033307	0.848426	0.00123	0.994363	-0.02211	0.898957	-0.02088	0.904542	6778028	9.784459	9.633965	9.770956	9.822438	9.905501	9.527781	9.69748	10.10177	9.452781	9.58674	9.717576	9.847794	Obfc2b	NM_027257
6778034	1701	6.092204	6.000144	6.257241	5.974858	0.117346	0.430241	0.025286	0.862387	0.282383	0.080643	-0.2571	0.106255	0.09206	0.532873	-0.16504	0.276323	6778034	6.200612	6.003171	6.072828	6.169058	6.010115	5.821259	6.036543	6.213333	6.521846	6.069251	5.816391	6.038931	Myl6b	NM_172259
6778037	1702	8.73158	8.66927	8.634481	8.034824	0.696756	0.004114	0.634446	0.006825	0.599657	0.009123	0.034789	0.847787	0.06231	0.731693	0.097099	0.595138	6778037	8.932346	8.611018	8.651375	8.670048	8.834945	8.502816	8.893323	8.485729	8.524391	7.99751	8.32708	7.779882	Mbc2	NM_011843
6778040	1703	6.963677	6.825542	6.827362	6.948835	0.014842	0.904283	-0.12329	0.332664	-0.12147	0.339428	-0.00182	0.988233	0.138134	0.281344	0.136315	0.287271	6778040	6.945079	6.938358	7.007593	6.751128	6.87844	6.847059	6.998171	6.979581	6.504333	6.97597	6.967429	6.903106	Zc3h10	NM_134003
6778043	1704	9.453937	9.424628	9.50212	9.4331	0.020836	0.855577	-0.00847	0.940949	0.06902	0.550838	-0.07749	0.504304	0.029309	0.798148	-0.04818	0.675276	6778043	9.474645	9.391995	9.49517	9.306346	9.665311	9.302226	9.585267	9.458094	9.463	9.261464	9.517286	9.520551	Pa2g4	NM_011119
6778044	1705	9.518016	9.617963	9.541544	9.319281	0.198735	0.128637	0.298682	0.034359	0.222263	0.094694	0.076419	0.532962	-0.09995	0.418915	-0.02353	0.846021	6778044	9.348739	9.583061	9.622249	9.523865	9.603144	9.72688	9.694363	9.306849	9.62342	9.352321	9.220246	9.385276	Erbb3	NM_010153
6778047	1706	6.981494	6.98752	6.955394	6.814077	0.167417	0.143868	0.173443	0.131782	0.141318	0.208638	0.032126	0.763836	-0.00603	0.954929	0.0261	0.80697	6778047	6.803204	7.241718	6.89956	7.020733	6.95047	6.991357	6.921498	6.993495	6.95119	6.762352	6.91995	6.759928	Ikzf4	NM_011772
6778052	1707	8.2717	8.251581	8.247482	8.431432	-0.15973	0.246192	-0.17985	0.196541	-0.18395	0.187565	0.004099	0.97517	0.020118	0.87868	0.024218	0.854263	6778052	8.290468	8.207601	8.31703	8.517115	8.079807	8.157822	8.209724	8.353604	8.179118	8.307644	8.352098	8.634553	Suox	NM_173733
6778055	1708	7.924223	8.007988	7.854466	7.837481	0.086742	0.56611	0.170507	0.273287	0.016985	0.909608	0.153522	0.32047	-0.08376	0.579243	0.069757	0.643228	6778055	8.027391	7.811168	7.934111	7.884207	8.29377	7.845986	8.027948	7.908659	7.626791	7.775632	7.782491	7.95432	Cdk2	NM_183417
6778057	1709	10.07691	9.978078	10.01666	9.884445	0.192469	0.202386	0.093633	0.518377	0.132211	0.368078	-0.03858	0.78782	0.098836	0.496069	0.060258	0.675233	6778057	10.11221	9.921922	10.19661	9.96911	10.13297	9.832155	10.26333	9.927289	9.859348	9.743695	9.84928	10.06036	Dgka	NM_016811
6778065	1710	7.773584	7.923982	7.588763	7.805247	-0.03166	0.761277	0.118735	0.272304	-0.21648	0.063829	0.335219	0.01041	-0.1504	0.173712	0.184821	0.103826	6778065	7.863722	7.738369	7.718662	8.02291	8.042795	7.706241	7.577287	7.565626	7.623377	7.714415	7.741606	7.95972	Mmp19	ENSMUST000000
6778068	1711	7.328705	7.403206	7.361918	7.248487	0.080218	0.669994	0.154719	0.418439	0.113431	0.549123	0.041287	0.825636	-0.0745	0.69203	-0.03321	0.859259	6778068	7.099758	7.441893	7.444465	7.166752	7.42466	7.618205	7.268864	7.716365	7.100526	7.331122	7.188967	7.225372	Ormdl2	NM_024180
6778077	1712	6.583872	6.418278	6.461392	6.379327	0.204545	0.070783	0.038951	0.701989	0.082065	0.427566	-0.04311	0.672236	0.165594	0.130213	0.12248	0.247565	6778077	6.514576	6.678071	6.558969	6.627047	6.353661	6.274126	6.454432	6.527632	6.402112	6.331794	6.299325	6.506863	Rdh5	NM_134006
6778113	1719	6.029173	6.034357	6.43651	6.2622	-0.23303	0.117493	-0.22784	0.124669	0.174311	0.225872	-0.40215	0.016378	-0.00518	0.969825	-0.40734	0.015435	6778113	5.956271	5.894083	6.237164	5.86095	6.251264	5.990858	6.305354	6.360725	6.643452	6.258109	6.272735	6.255755	Olfr796	NM_146931

6778278	1740	8.097954	8.107002	8.133139	8.12892	-0.03097	0.762374	-0.02192	0.830285	0.004219	0.967039	-0.02614	0.798387	-0.00905	0.929405	-0.03518	0.731398	6778278	8.115479	8.045993	8.132391	8.067279	8.306526	7.947202	8.169137	8.218458	8.011822	8.02347	8.238432	8.124857	Eif4enif	NM_023743
6778282	1741	8.01808	8.093028	7.871529	8.076676	-0.0586	0.750452	0.016352	0.92906	-0.20515	0.282358	0.221498	0.248556	-0.07495	0.684783	0.146551	0.434154	6778282	7.965942	8.085715	8.002584	8.245142	7.94508	8.088861	7.680151	8.229093	7.705344	7.880095	7.978087	8.371845	Patz1	NM_019574
6778284	1742	8.552652	8.859017	8.528157	8.249644	0.303008	0.160381	0.609374	0.014408	0.278514	0.192761	0.33086	0.129592	-0.30637	0.156345	0.024494	0.903547	6778284	8.468805	8.616172	8.572978	8.895901	8.526085	9.155066	8.615021	8.162589	8.806862	8.119038	8.361977	8.267916	Pik3ip1	NM_178149
6778298	1744	7.425521	7.769713	7.246588	7.469861	-0.04434	0.807816	0.299851	0.127487	-0.22327	0.241101	0.523124	0.017969	-0.34419	0.086754	0.178933	0.339969	6778298	7.46193	7.597072	7.217562	7.761669	7.747677	7.799792	7.676829	7.101864	6.961072	7.388702	7.498766	7.522116	Selm	NM_053267
6778315	1745	7.749343	7.88396	7.795459	7.795824	-0.04648	0.594449	0.088136	0.323855	-0.00037	0.996627	0.088501	0.321975	-0.13462	0.147012	-0.04612	0.597299	6778315	7.679117	7.848698	7.720214	7.816159	7.924899	7.910822	7.707353	7.992476	7.686547	7.754028	7.834601	7.798844	Morc2a	NM_001159288
6778345	1746	7.760795	7.765894	7.508251	7.650528	0.110267	0.474441	0.115367	0.454949	-0.14228	0.361218	0.257643	0.117588	-0.0051	0.973163	0.252544	0.123955	6778345	7.769864	7.725267	7.787254	7.798268	8.026839	7.472576	7.53306	7.647867	7.343826	7.502692	7.619827	7.829064	Dusp18	NM_173745
6778352	1747	7.323098	7.583746	7.440075	7.479165	-0.15607	0.038956	0.104581	0.137049	-0.03909	0.553978	0.143671	0.052884	-0.26065	0.003352	-0.11698	0.101742	6778352	7.395205	7.292095	7.281994	7.514483	7.636053	7.600702	7.504663	7.331077	7.484485	7.381952	7.537644	7.5179	Pes1	NM_022889
6778358	1749	6.045957	6.101595	6.190395	6.499992	-0.45404	0.19746	-0.3984	0.25244	-0.3096	0.365892	-0.0888	0.79035	-0.05564	0.867521	-0.14444	0.666618	6778358	5.606782	6.271312	6.259776	6.548951	5.573886	6.181947	6.017525	6.44253	6.111129	6.257905	6.240279	7.001792	Sec14l4	NM_146013
6778373	1750	7.330999	7.350799	7.262566	7.369058	-0.03806	0.705169	-0.01826	0.855445	-0.10649	0.304416	0.088233	0.389812	-0.0198	0.843426	0.068433	0.500714	6778373	7.365407	7.306148	7.321443	7.279075	7.446154	7.327168	7.447515	7.03465	7.305534	7.327309	7.336502	7.443362	Rnf215	NM_027859
6778375	1751	8.193451	8.175878	8.175337	8.099775	0.093676	0.354609	0.076102	0.447787	0.075562	0.450899	0.00054	0.995617	0.017573	0.858347	0.018114	0.854049	6778375	8.152888	8.254487	8.172978	8.049686	8.364249	8.113698	8.085889	8.309668	8.130455	7.991458	8.188234	8.119634	Sf3a1	NM_026175
6778376	1752	8.23358	8.207495	8.180036	8.168241	0.065339	0.176052	0.039253	0.398601	0.011795	0.795533	0.027458	0.550172	0.026085	0.569854	0.053544	0.258548	6778376	8.249181	8.260018	8.191541	8.194307	8.27084	8.157337	8.225023	8.206181	8.108905	8.165438	8.114573	8.224713	Tbc1d10a	NM_134023
6778383	1753	7.395326	7.453626	7.192328	7.29645	0.098876	0.507306	0.157176	0.301993	-0.10412	0.485716	0.261298	0.103979	-0.0583	0.693116	0.202998	0.192008	6778383	7.37869	7.531716	7.275573	7.659891	7.493682	7.207304	7.269935	6.955774	7.351275	7.317162	7.192225	7.379963	Gatsl3	BC039988
6778410	1755	8.161013	8.150118	8.162166	8.11634	0.044673	0.291299	0.033777	0.417818	0.045825	0.279905	-0.01205	0.768373	0.010896	0.789874	-0.00115	0.977465	6778410	8.124867	8.171725	8.186448	8.139188	8.20004	8.111125	8.093303	8.209143	8.184051	8.106631	8.171156	8.071234	Ascc2	NM_029291
6778414	1756	6.787344	7.074054	6.819004	6.912204	-0.12486	0.351666	0.16185	0.235783	-0.0932	0.481503	0.25505	0.078052	-0.28671	0.052824	-0.03166	0.808326	6778414	6.59078	6.832059	6.939193	7.16596	6.970422	7.085779	6.935648	6.910517	6.610846	6.814069	6.841853	7.08069	Zmat5	NM_026015
6778419	1757	7.775539	7.954894	7.925343	7.936206	-0.16067	0.069625	0.018688	0.813728	-0.01086	0.890929	0.029552	0.710213	-0.17936	0.047627	-0.1498	0.086715	6778419	7.689859	7.790997	7.845761	7.936832	7.956558	7.971293	8.017055	7.786543	7.97243	7.820513	7.932447	8.055658	Nipsnap1	NM_008698
6778420	1758	8.138341	8.144418	8.170196	7.942155	0.196186	0.106455	0.202263	0.09764	0.228041	0.067445	-0.02578	0.81713	-0.00608	0.956457	-0.03185	0.775273	6778420	8.072677	8.177502	8.164845	8.044981	8.28933	8.098943	8.338688	7.934916	8.236985	7.860716	8.014244	7.951506	Thoc5	NM_172438
6778425	1759	8.295474	8.31848	8.321814	8.430359	-0.13489	0.299182	-0.11188	0.384074	-0.10855	0.397736	-0.00333	0.978779	-0.02301	0.854539	-0.02634	0.833801	6778425	8.311371	8.217642	8.35741	8.309243	8.481478	8.164718	8.367647	8.442842	8.154953	8.262174	8.392621	8.636283	Ap1b1	NM_007454
6778426	1760	6.488655	6.422214	6.157245	6.392987	0.095668	0.605407	0.029227	0.873593	-0.23574	0.221763	0.264968	0.174742	0.066442	0.718522	0.33141	0.099515	6778426	6.383105	6.340243	6.742618	6.681526	6.487254	6.097861	6.271636	6.251589	5.948511	6.441728	6.233719	6.503514	Rasl10a	NM_145216
6778432	1761	6.731001	6.702243	6.763319	6.808179	-0.07718	0.339481	-0.10594	0.200748	-0.04486	0.571214	-0.06108	0.444723	0.028758	0.714913	-0.03232	0.6818	6778432	6.791521	6.683665	6.717817	6.823596	6.552615	6.730517	6.806119	6.733701	6.750137	6.811217	6.700853	6.912468	Rhbdd3	NM_177370
6778463	1762	10.1672	10.14911	10.37857	10.20634	-0.03915	0.86496	-0.05723	0.803849	0.172226	0.461971	-0.22946	0.333426	0.018087	0.937325	-0.21137	0.370778	6778463	10.10848	10.18876	10.20435	9.947579	10.43134	10.06841	10.23932	10.62783	10.26856	10.3477	10.54836	9.722973	Xbp1	NM_013842
6778506	1764	8.960586	8.860613	8.984135	8.920874	0.039713	0.563578	-0.06026	0.387376	0.063261	0.36534	-0.12352	0.097841	0.099973	0.167857	-0.02355	0.730168	6778506	8.983362	8.981227	8.91717	8.823793	8.946362	8.811684	8.98633	8.990403	8.975672	8.794311	9.068041	8.900269	Dbnl	NM_001146308
6778521	1765	9.564269	9.288465	8.951757	9.191128	0.373141	0.126636	0.097337	0.66832	-0.23937	0.305956	0.336709	0.16253	0.275804	0.24316	0.612512	0.023256	6778521	9.479145	9.831755	9.381907	9.007246	9.586351	9.271799	9.064192	8.774343	9.016735	9.164948	9.554497	8.85394	Aebp1	NM_009636
6778528	1766	7.954247	8.04329	7.872582	7.877392	0.076855	0.540033	0.165898	0.20445	-0.00481	0.969031	0.170708	0.192917	-0.08904	0.479562	0.081665	0.515632	6778528	8.064294	7.843865	7.954582	8.052691	8.168926	7.908252	8.07068	7.930608	7.616458	7.814489	7.924242	7.893444	Ykt6	NM_019661
6778560	1767	10.99839	10.91933	11.00826	11.31965	-0.32126	0.056318	-0.40032	0.02398	-0.31139	0.062666	-0.08893	0.554237	0.07906	0.598167	-0.00987	0.947065	6778560	11.01984	10.88511	11.09022	11.10258	10.81939	10.83602	11.00784	11.19044	10.8265	11.11908	11.26135	11.57852	Ogdh	NM_010956
6778562	1768	8.810699	8.688715	8.751351	8.593718	0.21698	0.18228	0.094997	0.540418	0.157633	0.319652	-0.06264	0.684411	0.121984	0.435384	0.059347	0.699999	6778562	8.940186	8.773088	8.718822	8.802829	8.818459	8.444857	8.617441	9.033049	8.603564	8.51052	8.733816	8.536819	Zmiz2	NM_028601
6778583	1769	7.377635	7.506437	7.335346	7.257244	0.120391	0.308479	0.249193	0.054479	0.078102	0.500513	0.171092	0.160802	-0.1288	0.278148	0.042289	0.7124	6778583	7.493474	7.258451	7.38098	7.439447	7.745394	7.334471	7.260781	7.385545	7.359711	7.302782	7.325245	7.143705	Ccm2	NM_146014
6778595	1770	7.713726	8.334898	7.814129	8.280965	-0.56724	0.007991	0.053933	0.747337	-0.46684	0.020303	0.520769	0.012236	-0.62117	0.00494	-0.1004	0.551979	6778595	7.713381	7.926847	7.50095	8.350021	8.335599	8.319074	8.0528	7.601971	7.787616	8.440628	8.403318	7.99895	Ramp3	NM_019511
6778719	1772	6.688431	7.039202	6.901798	6.970076	-0.28165	0.096503	0.069126	0.656325	-0.06828	0.660228	0.137404	0.385212	-0.35077	0.047062	-0.21337	0.191619	6778719	6.740497	6.994633	6.330163	6.982439	7.081707	7.053461	6.808978	6.983116	6.913301	6.847638	7.049197	7.013393	Upp1	NM_009477
6778784	1776	7.188756	7.131306	7.120997	6.681849	0.506908	0.049039	0.449458	0.073854	0.439148	0.079459	0.01031	0.963547	0.05745	0.799377	0.06776	0.764547	6778784	7.463127	6.951999	7.151143	7.124015	7.559214	6.71069	7.260108	7.049725	7.053157	6.680957	6.843217	6.521372	Ikzf1	NM_001025597
6778972	1778	8.671793	8.989235	8.805736	9.002028	-0.33023	0.041485	-0.01279	0.92745	-0.19629	0.187338	0.183499	0.214634	-0.31744	0.048037	-0.13394	0.354013	6778972	8.766355	8.673822	8.575202	8.955878	9.092317	8.919511	8.683586	8.946502	8.78712	8.846033	8.839231	9.320819	Egfr	NM_207655
6778984	1780	6.803336	6.595497	6.375957	6.38211	0.421226	0.037294	0.213387	0.242026	-0.00615	0.971829	0.219541	0.229865	0.207839	0.253451	0.427379	0.035237	6778984	6.942428	6.789364	6.678216	6.824957	6.617188	6.344347	6.544573	6.232956	6.350341	6.082273	6.47481	6.589248	Cnrip1	NM_029861
6778992	1781	7.819712	7.973139	7.83662	7.847289	-0.02758	0.730549	0.12585	0.142222	-0.01067	0.89365	0.136519	0.115426	-0.15343	0.082476	-0.01691	0.832368	6778992	7.8101	7.826485	7.822551	7.849526	8.056752	8.013138	7.939245	7.868927	7.701687	7.766341	7.824124	7.951402	Wdr92	NM_178909
6779162	1783	8.529458	8.698981	8.629224	8.802032	-0.27257	0.091707	-0.10305	0.489517	-0.17281	0.259154	0.069756	0.637094	-0.16952	0.267603	-0.09977	0.503053	6779162	8.660042	8.538509	8.389823	8.724845	8.832994	8.539103	8.447229	8.865429	8.575015	8.656007	8.736749	9.01334	Spred2	NM_033523
6779185	1784	8.17724	8.399247	8.19846	8.307133	-0.12989	0.426723	0.092114	0.569063	-0.10867	0.503464	0.200786	0.231671	-0.22201	0.190285	-0.02122	0.894577	6779185	8.159793	8.271601	8.100326	8.396645	8.608932	8.192163	7.944387	8.367097	8.283897	8.066978	8.425979	8.428441	Spred2	BC040462
6779196	1785	10.99057	10.94053	11.09258	10.95632	0.034257	0.638438	-0.01579	0.8276	0.136263	0.088029	-0.15205	0.062074	0.050043	0.495921	-0.10201	0.184017	6779196	11.04082	10.98146	10.94944	10.83832	11.00694	10.97633	11.20117	11.08432	10.99225	10.87919	11.05722	10.93254	Rab1	NM_008996
6779226	1786	8.703098	8.648629	8.649285	8.670801	0.032297	0.742887	-0.02217	0.821502	-0.02152	0.826684	-0.00066	0.994662	0.054469	0.582538	0.053813	0.587002	6779226	8.649307	8.685424	8.774563	8.726377	8.639818	8.579691	8.59843	8.821137	8.528288	8.550394	8.628948	8.83306	Sertad2	NM_021372
6779264	1787	8.686391	8.721404	8.674853	8.625473	0.060918	0.414585	0.095931	0.212437	0.04938	0.505264	0.046551	0.529308	-0.03501	0.634211	0.011538	0.874587	6779264	8.635844	8.656805	8.766524	8.67469	8.790524	8.698999	8.71701	8.709134	8.598416	8.520864	8.771654	8.583901	Peli1	NM_023324
6779270	1788	7.765223	7.868833	7.832747	7.759405	0.005817	0.927186	0.109428	0.113999	0.073342	0.268551	0.036086	0.574676	-0.10361	0.13154	-0.06752	0.305532	6779270	7.686701	7.805083	7.803884	7.788586	7.9616	7.856313	7.877705	7.81025	7.810286	7.649832	7.804986	7.823398	Vps54	NM_139061
6779288	1789	6.071348	6.030672	6.100814	6.070354	0.000994	0.991196	-0.03968	0.661456	0.03046	0.736115	-0.07014	0.444843	0.040676	0.653628	-0.02947	0.744359	6779288	6.002493	6.059407	6.152144	6.035243	5.993951	6.062822	5.887824	6.22834	6.186279	6.004545	6.136273	6.070245	AV249152	NM_145425
6779355	1791	6.979025	7.335582	7.279032	7.417638	-0.43861	0.049836	-0.08206	0.677285	-0.13861	0.486569	0.05655	0.773604	-0.35656	0.097458	-0.30001	0.153052	6779355	6.970685	7.301904	6.664487	7.440334	7.208774	7.357637	7.225996	7.549235	7.061865	7.257828	7.353921	7.641165	Zrsr1	NM_011663
6779359	1792	8.878723	9.138948	9.047498	8.822617	0.056105	0.638698	0.316331	0.025022	0.22488	0.086234	0.091451	0.44939	-0.26023	0.053467	-0.16878	0.180356	6779359	8.833532	8.996737	8.805899	8.976495	9.25281	9.18754	9.238552	8.990376	8.913565	8.667568	8.931103	8.869181	Cct4	NM_009837
6779373	1794	8.860439	8.896686	8.984125	8.727458	0.132981	0.513721	0.169228	0.409882	0.257266	0.222746	-0.08804	0.663019	-0.03625	0.856887	-0.12429	0.540921	6779373	8.697669	9.017634	8.866014	8.545299	9.137015	9.007744	9.022104	8.940131	8.991939	8.519169	9.097308	8.565898	Xpo1	NM_134014
6779392	1795	7.040083	7.165092	7.099879	7.136991	-0.09691	0.255658	0.028101	0.731744	-0.03711	0.651678	0.065213	0.433872	-0.12501	0.152908	-0.0598	0.471627	6779392	6.973909	7.053981	7.09236	7.112408	7.212061	7.170807	7.276854	6.975302	7.047482	7.045242	7.164857	7.200873	Pus10	NM_028956
6779432	1796	6.152345	6.202444	5.905313	6.186254	-0.03391	0.817707	0.01619	0.912248	-0.28094	0.083783	0.297131	0.070307	-0.0501	0.733969	0.247032	0.120885	6779432	6.285157	5.95099	6.220889	6.12258	6.297418	6.187335	6.172173	5.848748	5.695019	6.309565	6.01689	6.232307	Bcl11a	NM_016707
6779588	1797	6.448066	6.490204	6.491892	6.412117	0.035949	0.776967	0.078086	0.542244	0.079775	0.533761	-0.00169	0.989355	-0.04214	0.740103	-0.04383	0.730163	6779588	6.46662	6.547031	6.330547	6.321457	6.608902	6.540252	6.713268	6.380461	6.381948	6.255913	6.462924	6.517515	Fancl	NM_025923
6779672	1798	8.819078	9.373792	9.359529	9.208486	-0.38941	0.228741	0.165306	0.595216	0.151044	0.626832	0.014262	0.963099	-0.55471	0.100469	-0.54045	0.108094	6779672	8.301797	8.899661	9.255776	9.331367	9.63098	9.159028	9.704011	9.441087	8.93349	8.912759	9.1913	9.521398	Efemp1	NM_146015
6779688	1799	7.327767	7.498077	7.395704	7.347368	-0.0196	0.905978	0.15071	0.376017	0.048336	0.771364	0.102373	0.5421	-0.17031	0.32042	-0.06794	0.683762	6779688	7.281044	7.3775	7.324756	7.132127	7.736894	7.625211	7.489796	7.248623	7.448693	7.14469	7.406697	7.490716	Pnpt1	NM_027869
6779694	1800	8.984382	9.051642	9.101381	9.042515	-0.05813	0.649847	0.009127	0.9428	0.058866	0.645791	-0.04974	0.697187	-0.06726	0.600235	-0.117	0.370392	6779694	9.016901	9.097305	8.838939	8.813983	9.200736	9.140208	9.281549	9.017565	9.005029	8.96424	9.04351	9.119795	Smek2	NM_134034
6779714	1801	6.549457	6.552063	6.445222	6.381417	0.16804	0.521635	0.170647	0.515372	0.063806	0.805561	0.106841	0.68128	-0.00261	0.991961	0.104235	0.688564	6779714	6.863216	6.589643	6.195512	6.384907	6.936721	6.334562	6.651532	6.222333	6.461802	6.468617	6.655659	6.019974	Ccdc88a	NM_176841
6779718	1802	7.201075	7.34947	7.315846	7.301543	-0.10047	0.325873	0.047927	0.631027	0.014303	0.885243	0.033624	0.735177	-0.1484	0.160719	-0.11477	0.266088	6779718	7.049594	7.341856	7.211775	7.229856	7.416174	7.402381	7.219497	7.348653	7.379388	7.162886	7.408998	7.332745	Mtif2	NM_133767
6779725	1803	8.549161	8.520359	8.465893	7.968047	0.581113	0.026735	0.552311	0.032927	0.497845	0.048913	0.054466	0.806032	0.028802	0.89653	0.083268	0.708101	6779725	8.44169	8.441664	8.764128	8.496957	8.703212	8.360907	8.839711	8.184387	8.37358	7.695836	8.310501	7.897805	Rtn4	NM_194054
6779787	1804	8.807328	8.892558	8.85713	8.709408	0.09792	0.441288	0.18315	0.168162	0.147722	0.256422	0.035428	0.776893	-0.08523	0.500724	-0.0498	0.691128	6779787	8.846856	8.749639	8.825489	8.780818	9.062115	8.834742	9.078271	8.703035	8.790085	8.553429	8.864435	8.710361	Psme4	NM_134013
6779795	1806	6.396447	6.595448	6.60284	6.100511	0.295936	0.004705	0.494937	0.000192	0.502329	0.000173	-0.00739	0.925267	-0.199	0.031322	-0.20639	0.026955	6779795	6.330441	6.484965	6.373934	6.538156	6.719234	6.528954	6.718151	6.519041	6.571327	6.100134	6.181118	6.02028	Asb3	NM_023906
6779818	1807	9.154211	8.948984	8.912981	9.15499	-0.00078	0.996651	-0.20601	0.28521	-0.24201	0.215383	0.036004	0.846354	0.205226	0.286906	0.24123	0.216724	6779818	9.291386	9.175745	8.995501	9.019718	8.925974	8.901261	8.786974	9.268122	8.683846	8.909024	9.11869	9.437255	Cpeb4	NM_026252
6779827	1809	6.453434	6.270466	6.232472	6.452074	0.00136	0.995095	-0.18161	0.42164	-0.2196	0.335743	0.037994	0.863766	0.182968	0.418317	0.220962	0.332935	6779827	6.462172	6.425739	6.47239	6.051758	6.225045	6.534594	6.293344	6.360022	6.04405	6.482832	6.004262	6.869127	Nsg2	NM_008741
6779832	1810	6.311181	6.625698	6.380484	6.672528	-0.36135	0.006489	-0.04683	0.648775	-0.29204	0.018414	0.245214	0.038273	-0.31452	0.013048	-0.0693	0.50369	6779832	6.340435	6.317279	6.275828	6.593329	6.765719	6.518045	6.49662	6.227897	6.416934	6.545288	6.838144	6.634153	Snrnp25	NM_030093
6779834	1811	6.176455	6.250959	6.091339	6.229016	-0.05256	0.731763	0.021943	0.885844	-0.13768	0.379627	0.15962	0.312415	-0.0745	0.628372	0.085116	0.581166	6779834	6.068735	6.225875	6.234756	6.381456	6.378195	5.993226	6.070493	6.093573	6.109952	5.968801	6.211475	6.506771	Mpg	NM_010822
6779838	1812	6.190394	6.270574	6.193501	6.251576	-0.06118	0.720567	0.018998	0.911226	-0.05808	0.734101	0.077073	0.653069	-0.08018	0.640234	-0.00311	0.985445	6779838	6.331442	6.117076	6.122663	6.368671	6.362583	6.080468	5.975688	6.543854	6.060961	6.443313	6.16273	6.148686	Hba-x	NM_010405
6779843	1814	7.60994	7.827659	7.74936	6.894151	0.715789	0.005239	0.933508	0.001113	0.855209	0.0019	0.078299	0.688598	-0.21772	0.281136	-0.13942	0.480394	6779843	7.568923	7.653883	7.607014	7.676238	8.141763	7.664975	7.757558	7.854619	7.635903	6.860898	7.263263	6.558292	Sh3pxd2b	NM_177364
6779845	1815	8.281837	8.159161	8.131054	8.542153	-0.26032	0.06186	-0.38299	0.012765	-0.4111	0.009004	0.028107	0.820671	0.122676	0.336496	0.150783	0.244318	6779845	8.24851	8.03017	8.566832	8.156905	8.127841	8.192737	8.051759	8.224037	8.117366	8.606062	8.467034	8.553362	Ubtd2	NM_173784
6779855	1816	8.036712	7.835543	7.793975	7.307029	0.729683	0.00633	0.528514	0.02896	0.486946	0.040086	0.041569	0.839715	0.201168	0.34154	0.242737	0.257168	6779855	8.13975	8.156136	7.814249	7.828189	7.970789	7.707652	7.636156	7.830596	7.915172	6.97048	7.753742	7.196865	Stk10	NM_009288

6779857	1817	7.819035	7.89895	7.884142	7.744037	0.074998	0.387775	0.154913	0.095941	0.140105	0.126364	0.014807	0.861384	-0.07991	0.35896	-0.06511	0.450724	6779857	7.816541	7.808605	7.831958	7.855774	8.074981	7.766094	7.906643	7.875996	7.869788	7.612819	7.851641	7.767651	Fbxw11	NM_134015
6779961	1819	8.181579	7.812152	8.260095	7.106906	1.074672	0.012402	0.705245	0.068157	1.153189	0.008749	-0.44794	0.217529	0.369427	0.301743	-0.07852	0.820403	6779961	8.572025	8.182779	7.789932	7.858886	8.297142	7.280427	8.273077	8.173756	8.333453	6.893092	7.680965	6.746662	Lcp2	NM_010696
6780007	1821	9.021267	9.150542	9.065773	9.212118	-0.19085	0.273648	-0.06158	0.714396	-0.14634	0.393772	0.084769	0.615779	-0.12927	0.448932	-0.04451	0.790963	6780007	8.753939	9.148518	9.161345	9.005342	9.131979	9.314304	9.04948	9.209075	8.938763	8.930395	9.402426	9.303532	Pank3	NM_145962
6780266	1822	7.460663	7.590694	7.41937	7.36226	0.098403	0.42525	0.228435	0.086965	0.05711	0.63894	0.171325	0.181711	-0.13053	0.299201	0.041293	0.733549	6780266	7.430247	7.452715	7.499026	7.387765	7.800337	7.583981	7.527781	7.513175	7.217153	7.308477	7.464069	7.314233	Nudcd2	NM_026023
6780368	1826	9.053406	9.249143	9.161952	8.974936	0.07847	0.370858	0.274207	0.010652	0.187016	0.053766	0.087191	0.322923	-0.19574	0.045612	-0.10855	0.226097	6780368	9.007668	9.079032	9.073517	9.14511	9.328449	9.273871	9.317928	9.083642	9.084287	8.881945	8.944396	9.098467	Slu7	NM_148673
6780373	1827	6.177554	6.138945	6.084121	5.93735	0.240205	0.217742	0.201595	0.2941	0.146771	0.437349	0.054824	0.767903	0.03861	0.835122	0.093434	0.616896	6780373	6.435589	6.20015	5.896924	6.23774	6.199266	5.979828	6.163086	6.013942	6.075334	5.581287	6.136549	6.094213	C1qtnf2	NM_026979
6780438	1829	8.654127	8.708777	8.689899	8.897106	-0.24298	0.230002	-0.18833	0.343355	-0.20721	0.300021	0.018878	0.922072	-0.05465	0.77753	-0.03577	0.853058	6780438	8.670295	8.636484	8.655603	8.698106	8.8991	8.529124	8.6021	9.139515	8.328082	8.974086	8.839244	8.877987	Rnf145	NM_028862
6780443	1830	8.258004	8.079951	7.851423	7.631074	0.62693	0.0754	0.448877	0.181803	0.220349	0.493291	0.228528	0.477888	0.178053	0.577856	0.406581	0.22192	6780443	8.386309	8.364512	8.023191	8.104818	8.301809	7.833227	8.140985	7.671762	7.741523	6.927828	8.165804	7.799591	Ebf1	NM_007897
6780527	1832	6.514399	6.477841	6.456481	6.196852	0.317547	0.093807	0.280989	0.131023	0.259629	0.158704	0.02136	0.901399	0.036557	0.832236	0.057918	0.737727	6780527	6.811393	6.416802	6.315001	6.435925	6.760516	6.237083	6.424326	6.507386	6.437731	6.01109	6.258575	6.320891	Adam19	NM_009616
6780544	1834	7.414861	7.562589	7.579726	7.684215	-0.26935	0.110947	-0.12163	0.441883	-0.10449	0.506705	-0.01714	0.912051	-0.14773	0.354546	-0.16487	0.304695	6780544	7.448297	7.642879	7.153408	7.573761	7.662673	7.451334	7.605261	7.78021	7.353708	7.596257	7.619397	7.83699	Med7	NM_025426
6780551	1835	7.461122	7.228296	7.332411	6.797426	0.663696	0.092727	0.43087	0.250436	0.534985	0.16249	-0.10411	0.77226	0.232826	0.521996	0.128711	0.720884	6780551	7.510543	7.546922	7.325901	7.25946	7.58558	6.839848	7.020591	7.071737	7.904904	6.91484	7.299888	6.17755	Havcr2	NM_134250
6780694	1839	11.63342	11.77703	11.69789	11.74706	-0.11363	0.331183	0.029973	0.791873	-0.04916	0.666347	0.079137	0.491783	-0.14361	0.22743	-0.06447	0.57346	6780694	11.5934	11.59659	11.71028	11.62472	11.92623	11.78014	11.90226	11.70457	11.48685	11.72048	11.79946	11.72123	Gnb2l1	NM_008143
6780696	1840	7.03843	6.850941	6.812477	6.435861	0.602569	0.024841	0.415079	0.094225	0.376615	0.12331	0.038464	0.864742	0.187489	0.416159	0.225953	0.331677	6780696	6.932567	6.910885	7.271838	7.05877	7.049983	6.444069	6.814399	6.789146	6.833885	6.577057	6.691272	6.039255	Trim7	NM_053166
6780713	1842	7.004352	7.104905	7.212288	7.240659	-0.23631	0.426601	-0.13575	0.643293	-0.02837	0.922377	-0.10738	0.71341	-0.10055	0.730766	-0.20794	0.482184	6780713	7.115413	6.428607	7.469036	7.116371	7.072597	7.125747	7.391998	7.452588	6.792278	7.310971	7.450499	6.960508	Olfr1395	NM_146877
6780717	1844	8.460068	8.544945	8.463698	8.604797	-0.14473	0.122445	-0.05985	0.495427	-0.1411	0.130747	0.081247	0.360703	-0.08488	0.340809	-0.00363	0.966509	6780717	8.454185	8.58845	8.337568	8.392519	8.602152	8.640164	8.377785	8.533158	8.480151	8.553924	8.650557	8.609909	Zfp62	NM_001024846
6780725	1845	6.707519	6.783262	6.709264	6.701997	0.005522	0.948943	0.081265	0.359361	0.007266	0.932854	0.073998	0.401772	-0.07574	0.391274	-0.00174	0.98386	6780725	6.768904	6.766713	6.586941	6.854417	6.892465	6.602904	6.689366	6.739506	6.698919	6.624423	6.708784	6.772785	Mgat1	NM_001110148
6780746	1856	7.919818	8.547551	7.99492	8.104383	-0.18456	0.023684	0.443168	0.000154	-0.10946	0.136999	0.552631	3.22E-05	-0.62773	1.27E-05	-0.0751	0.289677	6780746	7.934673	7.928241	7.896541	8.611326	8.579988	8.451339	7.981903	7.964246	8.038611	7.968144	8.229831	8.115175	Flt4	NM_008029
6780754	1857	5.886669	9.086557	5.691228	7.427522	-1.54085	0.246994	1.659035	0.21559	-1.73629	0.196968	3.395328	0.024978	-3.19989	0.03193	0.19544	0.878056	6780754	6.001465	5.866168	5.792373	12.07724	7.488574	7.693856	5.474468	6.065995	5.533222	7.907051	8.645133	5.730381	Scgb3a1	NM_170727
6780767	1858	7.672362	7.763871	7.980519	7.039392	0.63297	0.055726	0.724479	0.033654	0.941127	0.010456	-0.21665	0.465954	-0.09151	0.75473	-0.30816	0.307934	6780767	7.750913	7.552804	7.713369	7.789884	7.999113	7.502616	8.069879	7.822237	8.049442	6.980951	7.689836	6.44739	Gfpt2	NM_013529
6780771	1859	8.073251	8.094009	8.155306	8.000555	0.072696	0.439675	0.093455	0.326433	0.154751	0.12171	-0.0613	0.512329	-0.02076	0.82222	-0.08206	0.385554	6780771	8.038259	8.033721	8.147772	8.165354	8.152872	7.963802	8.28055	8.110785	8.074583	7.853563	8.02174	8.126361	Mapk9	NM_016961
6780777	1860	9.14964	9.171069	9.18407	9.154621	-0.00498	0.970144	0.016449	0.90168	0.029449	0.825141	-0.013	0.922201	-0.02143	0.872177	-0.03443	0.796287	6780777	9.289733	9.088679	9.070507	9.365683	9.047387	9.100138	9.235006	9.33326	8.983944	9.032391	9.104933	9.326538	Rnf130	NM_021540
6780779	1862	9.324204	9.371864	9.403765	9.274112	0.050092	0.676553	0.097753	0.422819	0.129654	0.295063	-0.0319	0.789794	-0.04766	0.691266	-0.07956	0.511199	6780779	9.412367	9.336073	9.224171	9.472332	9.408534	9.234727	9.441343	9.596416	9.173537	9.243067	9.19073	9.388538	Tbc1d9b	NM_029745
6780781	1863	6.771343	6.828331	6.695196	6.815038	-0.04369	0.608547	0.013293	0.875217	-0.11984	0.181948	0.133135	0.143055	-0.05699	0.50668	0.076147	0.380166	6780781	6.64754	6.773311	6.893179	6.825605	6.787499	6.871888	6.550297	6.784394	6.750897	6.814342	6.729199	6.901572	Sqstm1	NM_011018
6780782	1864	7.611065	7.816795	7.489041	7.481252	0.129813	0.376231	0.335543	0.04174	0.007789	0.956551	0.327754	0.045567	-0.20573	0.175895	0.122024	0.404172	6780782	7.701925	7.536176	7.595095	7.873677	7.916847	7.659862	7.612344	7.668003	7.186776	7.322566	7.528842	7.592349	Mgat4b	NM_145926
6780797	1865	10.84673	10.93421	10.88314	10.88278	-0.03605	0.733538	0.051423	0.628595	0.000357	0.997302	0.051067	0.630942	-0.08747	0.417195	-0.03641	0.731022	6780797	10.82997	10.87392	10.83631	10.68848	10.98503	11.12911	10.94417	10.92081	10.78444	10.81927	10.94961	10.87947	Hnrnph1	NM_021510
6780811	1866	7.815944	7.697172	7.215565	6.768157	1.047787	0.004842	0.929014	0.009113	0.447408	0.138348	0.481607	0.114339	0.118773	0.673664	0.600379	0.058172	6780811	8.051316	7.767716	7.628801	7.669707	8.166485	7.255323	7.446706	7.086376	7.113613	6.416676	7.180141	6.707655	Adamts2	NM_175643
6780816	1867	5.984473	5.931114	6.003282	5.867593	0.116879	0.209214	0.063521	0.479197	0.135689	0.151536	-0.07217	0.423601	0.053358	0.550351	-0.01881	0.831552	6780816	5.984184	6.063991	5.905243	5.850077	6.023716	5.91955	5.907971	5.909043	6.192833	5.917843	5.876689	5.808248	Grm6	NM_173372
6780855	1873	8.780577	9.025448	8.845868	9.019004	-0.23843	0.1705	0.006444	0.968533	-0.17314	0.305978	0.17958	0.289526	-0.24487	0.160533	-0.06529	0.690884	6780855	8.667763	8.873419	8.80055	8.959752	8.847238	9.269354	8.94771	8.813906	8.775988	8.823275	8.882623	9.351115	Clk4	NM_007714
6780856	1874	6.583943	6.532332	6.611025	6.567418	0.016525	0.75797	-0.03509	0.517449	0.043608	0.424484	-0.07869	0.167338	0.051611	0.348425	-0.02708	0.615384	6780856	6.60832	6.509391	6.634117	6.517086	6.534556	6.545354	6.614273	6.70373	6.515073	6.626955	6.534734	6.540564	Col23a1	NM_153393
6780858	1875	7.382994	7.487164	7.548847	7.558016	-0.17502	0.086217	-0.07085	0.451347	-0.00917	0.920916	-0.06168	0.510138	-0.10417	0.277942	-0.16585	0.100958	6780858	7.377803	7.39293	7.37825	7.631957	7.468182	7.361353	7.51457	7.695645	7.436327	7.503532	7.487895	7.682621	Agxt2l2	NM_028398
6780863	1876	7.602582	7.725598	7.553821	7.410763	0.191819	0.285604	0.314835	0.097196	0.143058	0.418271	0.171777	0.335495	-0.12302	0.484009	0.048761	0.77855	6780863	7.735005	7.546744	7.525998	7.446526	7.965924	7.764345	7.869076	7.428131	7.364257	7.305651	7.517562	7.409077	Nhp2	NM_026631
6780868	1877	7.437176	7.462122	7.509766	7.314175	0.123001	0.115817	0.147947	0.066697	0.195592	0.023043	-0.04764	0.513828	-0.02495	0.729839	-0.07259	0.328403	6780868	7.360058	7.426703	7.524766	7.539971	7.524349	7.322046	7.494672	7.46124	7.573387	7.369596	7.330358	7.24257	D930048N	BC095931
6780871	1878	10.22326	10.14659	10.21488	10.18245	0.040803	0.811727	-0.03586	0.834098	0.03243	0.849742	-0.06829	0.691109	0.076667	0.655979	0.008373	0.960944	6780871	10.05489	10.02602	10.58886	10.16593	10.25832	10.01552	10.37648	10.232	10.03617	10.05369	10.15897	10.3347	Sar1b	NM_025535
6780882	1879	8.680065	8.95562	8.756468	8.636258	0.043806	0.687956	0.319362	0.016141	0.12021	0.28606	0.199152	0.094879	-0.27556	0.030638	-0.0764	0.48823	6780882	8.55203	8.735401	8.752763	8.888225	9.079426	8.899209	8.978805	8.647277	8.643322	8.552962	8.691216	8.664597	Cdkn2aip	NM_029976
6780895	1880	6.222702	6.323646	6.152808	6.264731	-0.04203	0.736207	0.058915	0.637967	-0.11192	0.380079	0.170838	0.193953	-0.10094	0.426439	0.069894	0.577798	6780895	6.291526	6.232826	6.143754	6.333294	6.270434	6.36721	6.216881	6.258103	5.98344	6.185747	6.075033	6.533414	Cdkl3	NM_153785
6780907	1881	11.23798	11.30156	11.20922	11.11488	0.123093	0.282893	0.186677	0.118982	0.094333	0.403382	0.092343	0.41294	-0.06358	0.568512	0.02876	0.794759	6780907	11.23994	11.14926	11.32473	11.21513	11.42963	11.25992	11.37437	11.26617	10.98711	11.05695	11.21904	11.06866	Ppp2ca	NM_019411
6780920	1882	10.56336	10.79155	10.60555	10.73156	-0.1682	0.317583	0.059993	0.713713	-0.12601	0.447616	0.186007	0.272383	-0.2282	0.186174	-0.04219	0.79596	6780920	10.6964	10.72268	10.27099	10.62097	10.85152	10.90217	10.67389	10.6686	10.47415	10.59109	10.61494	10.98865	Skp1a	NM_011543
6780933	1883	10.69652	10.76763	10.57081	10.7112	-0.01468	0.933881	0.056433	0.750476	-0.14039	0.436572	0.19682	0.284118	-0.07111	0.689201	0.12571	0.484328	6780933	10.57242	10.47598	11.04117	10.88653	10.73682	10.67955	10.666	10.67961	10.36683	10.48425	10.76441	10.88494	Vdac1	NM_011694
6780940	1884	9.881537	9.919821	9.806053	9.843404	0.038133	0.760809	0.076418	0.545466	-0.03735	0.765546	0.113768	0.374811	-0.03828	0.759896	0.075484	0.550274	6780940	10.01045	9.797382	9.836779	9.894618	10.02656	9.838286	10.0322	9.758924	9.627036	9.733375	9.780326	10.01651	9530068E	NM_153117
6780945	1885	9.249223	9.118487	9.326822	8.895877	0.353346	0.044683	0.22261	0.172468	0.430945	0.019883	-0.20834	0.198474	0.130737	0.404589	-0.0776	0.615651	6780945	9.49854	9.30519	8.94394	8.992455	9.270069	9.092936	9.265974	9.325687	9.388805	8.700726	8.959793	9.027112	Fstl4	NM_177059
6780961	1886	6.593097	6.511042	6.599897	6.81776	-0.22466	0.161366	-0.30672	0.068213	-0.21786	0.172903	-0.08886	0.558577	0.082056	0.588456	-0.0068	0.963891	6780961	6.460823	6.552342	6.766127	6.531066	6.724768	6.277291	6.558928	6.808452	6.432312	6.73482	6.756885	6.961576	Zcchc10	NM_026479
6780963	1887	10.44991	10.55172	10.43029	10.54747	-0.09757	0.377701	0.004247	0.968573	-0.11719	0.294545	0.121433	0.278612	-0.10181	0.358346	0.01962	0.855715	6780963	10.49804	10.40453	10.44215	10.54427	10.56798	10.54291	10.38196	10.68841	10.22049	10.61679	10.45627	10.56936	Aff4	NM_033565
6780978	1889	6.023135	6.160846	6.232658	6.23983	-0.2167	0.093327	-0.07898	0.507235	-0.00717	0.951288	-0.07181	0.545562	-0.13771	0.260713	-0.20952	0.102823	6780978	6.012403	6.1604	5.896601	6.19857	6.105515	6.178452	6.16018	6.474234	6.06356	6.318737	6.116549	6.284204	Shroom1	NM_027917
6780983	1890	8.894086	8.805596	8.660649	8.397341	0.496745	0.007424	0.408255	0.019172	0.263308	0.096032	0.144947	0.329557	0.08849	0.543909	0.233437	0.133078	6780983	8.994174	8.847896	8.840188	8.787116	8.989903	8.639769	8.747643	8.738393	8.495911	8.212483	8.670119	8.309421	8-Sep	NM_033144
6780986	1891	7.413927	7.445295	7.385019	7.423115	-0.00919	0.862228	0.02218	0.676744	-0.0381	0.478728	0.060276	0.273559	-0.03137	0.557658	0.028907	0.588346	6780986	7.42135	7.4643	7.35613	7.426964	7.506315	7.402606	7.302161	7.471507	7.38139	7.431294	7.367679	7.470373	Kif3a	NM_008443
6780996	1893	8.205351	8.116767	8.320174	7.781252	0.424099	0.026294	0.335515	0.063678	0.538922	0.008631	-0.20341	0.228489	0.088584	0.585676	-0.11482	0.482678	6780996	8.347425	8.32972	7.938909	8.067131	8.280181	8.002989	8.551258	8.143044	8.266219	7.638621	7.964386	7.740749	Irf1	NM_008390
6781012	1894	8.218031	8.362677	8.040003	8.579698	-0.36167	0.003751	-0.21702	0.041689	-0.53969	0.000315	0.322674	0.006964	-0.14465	0.145062	0.178028	0.082126	6781012	8.210667	8.33571	8.107717	8.345864	8.468354	8.273814	8.130564	7.898835	8.090611	8.569198	8.484863	8.685034	P4ha2	NM_001136076
6781032	1896	7.901405	8.107913	8.10179	7.928139	-0.02673	0.699028	0.179774	0.027265	0.173651	0.031439	0.006123	0.929113	-0.20651	0.014748	-0.20039	0.016955	6781032	7.909513	7.997755	7.796947	8.195647	8.010468	8.117624	8.094953	8.15151	8.058908	7.855779	7.921778	8.006861	Rapgef6	NM_175258
6781083	1897	10.38786	10.31242	10.18794	10.07113	0.316728	0.065522	0.241292	0.142879	0.116807	0.454236	0.124485	0.426249	0.075437	0.625198	0.199921	0.215152	6781083	10.31247	10.33183	10.51928	10.26914	10.44044	10.22769	10.44527	10.25675	9.861796	10.08639	9.930335	10.19667	Hint1	NM_008248
6781090	1898	9.357436	9.159798	9.083298	9.860815	-0.50338	0.128608	-0.70102	0.045967	-0.77752	0.030775	0.0765	0.803272	0.197637	0.524561	0.274138	0.383077	6781090	9.614105	9.330163	9.128039	9.215005	9.260047	9.004343	8.985888	9.56366	8.700346	9.551702	9.583984	10.44676	Gpx3	NM_001083929
6781104	1899	10.78277	10.65645	10.65022	10.6149	0.167873	0.435726	0.041553	0.844141	0.03532	0.867238	0.006233	0.976443	0.12632	0.554158	0.132553	0.535244	6781104	11.18273	10.70331	10.46227	10.57107	10.91558	10.4827	10.88867	10.58457	10.47741	10.55577	10.77192	10.517	Gm2a	NM_010299
6781111	1900	8.037315	8.115666	8.0361	8.060303	-0.02299	0.877761	0.055363	0.712092	-0.0242	0.871365	0.079566	0.597571	-0.07835	0.60308	0.001215	0.993507	6781111	8.079004	8.021514	8.011428	8.262469	8.16941	7.915119	7.847729	8.219502	8.041069	7.933089	7.910921	8.3369	Slc36a1	NM_153139
6781138	1901	9.001773	8.848811	8.928646	8.638048	0.363725	0.02174	0.210762	0.138194	0.290597	0.05283	-0.07984	0.550107	0.152962	0.266216	0.073127	0.583409	6781138	9.010752	8.97027	9.024297	8.71903	9.085448	8.741954	9.007036	8.939098	8.839803	8.480596	8.889051	8.544498	G3bp1	NM_013716
6781209	1903	8.901699	8.970422	8.871169	8.728202	0.173497	0.183016	0.24222	0.076243	0.142967	0.264013	0.099252	0.428513	-0.06872	0.579541	0.03053	0.804024	6781209	8.971563	8.930717	8.802818	8.853439	9.073214	8.984612	8.795229	8.898952	8.919327	8.658192	9.001762	8.524652	Mfap3	NM_145426
6781214	1904	7.381362	7.32312	7.11503	7.130339	0.251024	0.096779	0.192781	0.186611	-0.01531	0.911509	0.208089	0.157579	0.058243	0.674088	0.266332	0.081074	6781214	7.485747	7.463408	7.194932	7.314384	7.486165	7.16881	6.925408	7.163988	7.255695	7.073744	7.313473	7.003799	Galnt10	NM_134189
6781240	1905	9.548855	9.479084	9.410131	8.961932	0.586923	0.110969	0.517153	0.153077	0.448199	0.208459	0.068953	0.838554	0.06977	0.836673	0.138724	0.683117	6781240	9.714993	9.613694	9.317877	9.46911	9.391953	9.57619	8.717565	9.929385	9.583443	8.805977	9.469897	8.609921	Larp1	NM_028451
6781243	1906	8.842561	8.988717	8.894959	8.761526	0.081036	0.355391	0.227191	0.025062	0.133434	0.144956	0.093757	0.289298	-0.14616	0.114849	-0.0524	0.543639	6781243	8.733351	8.898569	8.895764	9.008391	8.98137	8.976389	9.016568	8.755434	8.912876	8.718069	8.669214	8.897294	Cnot8	NM_026949
6781248	1908	7.192656	7.146916	7.099915	6.38647	0.806186	0.042077	0.760446	0.052118	0.713445	0.064924	0.047001	0.891444	0.04574	0.894336	0.092741	0.78807	6781248	7.213461	7.584661	6.779845	7.086684	7.420569	6.933494	6.905507	7.043347	7.35089	6.360761	7.024633	5.774016	Igtp	NM_018738
6781252	1909	7.871028	7.952101	8.056099	8.007919	-0.13689	0.227896	-0.05582	0.608865	0.04818	0.658025	-0.104	0.350209	-0.08107	0.461531	-0.18507	0.115479	6781252	7.780449	7.94419	7.888445	7.943419	7.89166	8.021223	7.989714	8.278135	7.900448	7.867172	8.047873	8.108711	Zfp692	NM_182996
6781257	1910	6.522126	6.480041	6.180033	6.559253	-0.03713	0.638081	-0.07921	0.327466	-0.37922	0.001062	0.300008	0.004236	0.042085	0.594651	0.342093	0.001991	6781257	6.525789	6.412255	6.628334	6.555505	6.405214	6.479403	6.137959	6.304218	6.097922	6.475874	6.610984	6.590902	Sh3bp5l	NM_024480
6781305	1921	7.200383	7.283231	7.159152	6.936081	0.264302	0.331819	0.347151	0.211886	0.223072	0.40869	0.124079	0.640711	-0.08285	0.754388	0.04123	0.875975	6781305	7.118692	7.436972	7.045484	7.306229	7.258288	7.285177	6.656435	7.605931	7.215091	7.04132	7.219386	6.547536	Hist3h2a	NM_178218

6781306	1922	6.284423	6.213245	6.247644	6.198077	0.086346	0.313211	0.015168	0.854755	0.049567	0.553882	-0.0344	0.679422	0.071178	0.400876	0.036779	0.658848	6781306	6.318431	6.174166	6.360672	6.213974	6.150829	6.274933	6.142062	6.322866	6.278003	6.239578	6.05464	6.300013	Trim17	NM_031172
6781307	1923	8.002981	8.004054	7.80952	7.768476	0.234505	0.018693	0.235578	0.018313	0.041044	0.620701	0.194534	0.040606	-0.00107	0.989601	0.193462	0.041467	6781307	8.074363	8.042271	7.89231	7.964168	8.176474	7.871519	7.852163	7.767536	7.80886	7.811226	7.780437	7.713765	Trim11	NM_053168
6781321	1924	8.209154	8.206499	8.191391	8.152276	0.056878	0.648651	0.054223	0.663826	0.039115	0.753166	0.015108	0.903059	0.002655	0.982915	0.017763	0.886151	6781321	8.242966	8.099044	8.285453	8.227718	8.093046	8.298734	8.188868	8.434009	7.951297	8.049578	8.208735	8.198516	Mrpl55	NM_026035
6781322	1925	9.218347	9.196063	9.167308	9.298203	-0.07986	0.368541	-0.10214	0.257631	-0.1309	0.156927	0.028755	0.740347	0.022285	0.797028	0.05104	0.55939	6781322	9.252753	9.236082	9.166771	9.136026	9.256868	9.195294	9.186064	9.281194	9.034665	9.178991	9.25651	9.459109	2310033P	BC002181
6781329	1926	5.936039	6.117981	5.951781	5.916796	0.019243	0.714216	0.201185	0.004135	0.034985	0.509775	0.1662	0.011231	-0.18194	0.007107	-0.01574	0.764169	6781329	6.010304	5.944206	5.853608	6.051654	6.167152	6.135137	5.991382	5.955185	5.908777	5.912508	5.856036	5.981845	Wnt9a	NM_139298
6781352	1927	6.508179	6.657656	6.641774	6.67895	-0.17077	0.169007	-0.02129	0.855158	-0.03718	0.750503	0.015882	0.891654	-0.14948	0.222277	-0.13359	0.270857	6781352	6.498173	6.368002	6.658363	6.750231	6.758896	6.463841	6.61746	6.784889	6.522973	6.566036	6.742259	6.728556	Jmjd4	NM_178659
6781372	1929	10.12282	10.264	10.17069	9.84925	0.273566	0.041257	0.414753	0.006194	0.321443	0.021338	0.09331	0.43138	-0.14119	0.245354	-0.04788	0.68195	6781372	10.15018	10.16485	10.05342	10.12961	10.40473	10.25767	10.24254	10.20874	10.0608	9.776803	10.08597	9.684978	Mprip	NM_012027
6781383	1930	7.004946	7.037614	6.954902	7.081441	-0.07649	0.134298	-0.04383	0.367796	-0.12654	0.024837	0.082712	0.109336	-0.03267	0.497036	0.050045	0.307507	6781383	6.941555	7.015759	7.057525	6.947868	7.101843	7.06313	6.934394	6.98386	6.946451	7.098988	7.029202	7.116132	Nt5m	NM_134029
6781396	1931	8.123616	8.22121	8.024312	8.207736	-0.08412	0.41913	0.013473	0.894861	-0.18342	0.100363	0.196897	0.081318	-0.09759	0.352022	0.099304	0.344115	6781396	8.059963	8.129917	8.180968	8.103311	8.185505	8.374813	7.93843	8.034484	8.100023	8.016644	8.341477	8.265088	Med9	NM_138675
6781414	1932	7.719121	7.54256	7.536404	7.679182	0.03994	0.751326	-0.13662	0.294402	-0.14278	0.274699	0.006156	0.960918	0.176561	0.185099	0.182717	0.171857	6781414	7.729965	7.70819	7.719209	7.614091	7.650664	7.362926	7.33777	7.799664	7.471779	7.616567	7.640517	7.780461	Rai1	NM_009021
6781440	1934	8.006104	8.083227	8.055725	8.078524	-0.07242	0.63183	0.004703	0.974991	-0.0228	0.879285	0.027502	0.854688	-0.07712	0.610222	-0.04962	0.741687	6781440	7.838402	8.104409	8.075501	8.125074	8.053868	8.070738	7.973569	8.390638	7.802968	7.979603	8.054378	8.201591	4933439F	BC044901
6781441	1935	8.292514	8.502402	8.388256	8.413787	-0.12127	0.071341	0.088615	0.167399	-0.02553	0.673349	0.114146	0.086211	-0.20989	0.00702	-0.09574	0.139531	6781441	8.25171	8.343472	8.28236	8.509562	8.532753	8.46489	8.320729	8.413713	8.430326	8.28818	8.434847	8.518333	Drg2	NM_021354
6781443	1937	9.526013	9.607993	9.51434	9.752143	-0.22613	0.120919	-0.14415	0.300813	-0.2378	0.105474	0.093653	0.492807	-0.08198	0.546839	0.011674	0.930824	6781443	9.475963	9.507607	9.59447	9.755541	9.547468	9.520969	9.433737	9.786959	9.322323	9.658166	9.672013	9.92625	Alkbh5	NM_172943
6781456	1938	7.384828	7.413121	7.315905	7.176458	0.20837	0.123148	0.236663	0.086033	0.139447	0.282113	0.097216	0.444649	-0.02829	0.820873	0.068922	0.584339	6781456	7.435838	7.391829	7.326816	7.417305	7.67316	7.148899	7.350222	7.391277	7.206217	7.154171	7.266144	7.108819	Llgl1	NM_008502
6781458	1939	6.171161	6.196482	6.116374	6.426498	-0.25534	0.221376	-0.23002	0.266416	-0.31012	0.14589	0.080108	0.688286	-0.02532	0.898613	0.054787	0.783203	6781458	6.052614	6.066705	6.394164	6.372508	6.179665	6.037273	6.025759	6.503006	5.820356	6.238236	6.433991	6.607268	Smcr7	NM_001009927
6781463	1940	8.171124	8.195618	8.133087	8.106629	0.064495	0.492752	0.088989	0.350378	0.026458	0.775613	0.062531	0.505626	-0.02449	0.791788	0.038037	0.682809	6781463	8.292326	8.137901	8.083145	8.23299	8.0488	8.305065	7.978431	8.20095	8.21988	8.099531	8.141993	8.078364	Smcr8	NM_001085440
6781472	1941	8.016838	8.090922	7.936702	8.075895	-0.05906	0.700985	0.015027	0.92181	-0.13919	0.375576	0.15422	0.328949	-0.07408	0.630959	0.080136	0.603788	6781472	7.869031	8.011621	8.169863	7.919139	8.161831	8.191796	8.227676	7.676507	7.905924	8.011264	8.023	8.19342	Dhrs7b	NM_145428
6781487	1942	8.671242	8.771412	8.586869	8.736649	-0.06541	0.72	0.034763	0.848451	-0.14978	0.419806	0.184543	0.325344	-0.10017	0.585128	0.084373	0.644717	6781487	8.495831	8.595028	8.922866	8.794483	8.931367	8.588387	8.628249	8.795814	8.336544	8.472741	8.878902	8.858304	Map2k3	NM_008928
6781515	1946	9.026582	8.777525	8.296772	7.687391	1.339191	0.04738	1.090134	0.093216	0.609382	0.317958	0.480753	0.42519	0.249057	0.674869	0.72981	0.237929	6781515	9.013883	9.402472	8.663391	8.822239	9.521241	7.989095	9.042573	7.794744	8.053	6.65196	8.167666	8.242546	Mfap4	NM_029568
6781516	1947	8.265194	8.290365	8.322135	8.181556	0.083639	0.499574	0.10881	0.384517	0.140579	0.268716	-0.03177	0.79504	-0.02517	0.836802	-0.05694	0.643127	6781516	8.288912	8.240427	8.266244	8.177299	8.371236	8.322561	8.50678	8.361382	8.098243	8.152387	8.023929	8.368351	B9d1	NM_013717
6781525	1948	6.778989	6.987324	6.895605	7.053096	-0.27411	0.065688	-0.06577	0.622922	-0.15749	0.25563	0.091719	0.496077	-0.20834	0.143959	-0.11662	0.391095	6781525	6.691559	6.796295	6.849112	6.82946	7.093968	7.038544	7.003607	6.846462	6.836746	6.809671	7.032801	7.316817	Grap	NM_027817
6781526	1949	5.837816	5.869399	5.926711	5.763764	0.074052	0.566619	0.105635	0.418721	0.162947	0.224938	-0.05731	0.656022	-0.03158	0.805242	-0.0889	0.493531	6781526	5.872783	5.758235	5.88243	5.759468	6.092509	5.75622	6.000519	5.893122	5.886493	5.691319	6.004879	5.595094	Fam83g	NM_178618
6781560	1950	6.886127	6.988179	6.804725	6.645901	0.240225	0.638051	0.342278	0.505812	0.158824	0.754818	0.183454	0.718594	-0.10205	0.840666	0.081401	0.872537	6781560	6.632669	6.345557	7.680154	6.607894	7.846818	6.509826	7.356913	6.693851	6.363412	6.618202	6.281088	7.038414	Adora2b	NM_007413
6781561	1951	7.712183	7.722187	7.639444	7.973864	-0.26168	0.044074	-0.25168	0.050821	-0.33442	0.015815	0.082743	0.472057	-0.01	0.929537	0.072738	0.525694	6781561	7.646811	7.627551	7.862186	7.597704	7.852736	7.716121	7.696088	7.744328	7.477917	7.855167	7.942951	8.123475	Ttc19	NM_028360
6781569	1952	6.69007	6.811743	6.701232	6.77533	-0.08526	0.358876	0.036413	0.688551	-0.0741	0.422162	0.110511	0.242614	-0.12167	0.202251	-0.01116	0.901749	6781569	6.647556	6.61385	6.808805	6.782106	6.934031	6.719091	6.562126	6.775859	6.76571	6.845473	6.671414	6.809103	Pigl	NM_001039536
6781580	1953	6.745114	6.546835	6.641201	6.101691	0.643422	0.016853	0.445144	0.070965	0.53951	0.035664	-0.09437	0.670728	0.198279	0.380997	0.103912	0.640091	6781580	6.999619	6.76765	6.468072	6.411677	6.796805	6.432023	6.954915	6.283517	6.685172	6.176125	6.259826	5.869123	Trpv2	NM_011706
6781590	1954	9.68567	9.570147	9.689766	9.567308	0.118362	0.433177	0.002839	0.984693	0.122458	0.418721	-0.11962	0.428503	0.115523	0.443866	-0.0041	0.97792	6781590	9.570206	9.60335	9.883454	9.592136	9.54962	9.568684	9.973878	9.69485	9.400569	9.544324	9.474926	9.682673	Mmgt2	NM_175002
6781605	1955	7.336685	7.393466	7.58092	7.215572	0.121112	0.444509	0.177893	0.271422	0.365347	0.04146	-0.18745	0.248452	-0.05678	0.715948	-0.24424	0.143506	6781605	7.252661	7.415785	7.341608	7.382476	7.463951	7.33397	7.400065	7.655818	7.686876	6.879032	7.507831	7.259854	Trim16	NM_053169
6781607	1957	8.944994	9.178327	8.979943	9.047354	-0.10236	0.585685	0.130973	0.48816	-0.06741	0.718121	0.198385	0.303065	-0.23333	0.231592	-0.03495	0.851089	6781607	9.015814	8.909451	8.909716	9.064609	9.120861	9.349512	9.304839	9.03262	8.602369	8.968294	8.891276	9.282493	Fam18b	NM_026210
6781612	1960	11.543	11.29666	11.32333	11.23048	0.312523	0.182568	0.06618	0.76518	0.092847	0.676049	-0.02667	0.903969	0.246343	0.28321	0.219677	0.334977	6781612	11.43519	11.47672	11.7171	11.45649	11.15914	11.27435	11.4046	11.10926	11.45612	11.33916	11.60696	10.74532	Pmp22	NM_008885
6781689	1962	7.559571	7.544164	7.861079	7.482796	0.076775	0.749339	0.061368	0.79818	0.378283	0.141831	-0.31692	0.209328	0.015407	0.948711	-0.30151	0.230171	6781689	7.382968	7.627098	7.668646	7.77562	7.485706	7.371166	8.135215	7.823487	7.624536	7.338468	7.972267	7.137654	Hs3st3a1	NM_178870
6781727	1963	7.11281	7.052697	7.032433	7.046725	0.066085	0.389793	0.005972	0.936532	-0.01429	0.849016	0.020264	0.787468	0.060113	0.432171	0.080376	0.300924	6781727	7.178899	7.114786	7.044744	7.062784	7.13263	6.962677	7.086519	7.021151	6.98963	6.901884	7.077684	7.160607	AU040829	NM_001099288
6781795	1964	6.171947	6.130639	6.122612	6.075531	0.096416	0.216081	0.055108	0.464718	0.047081	0.530314	0.008028	0.913712	0.041307	0.580812	0.049335	0.511338	6781795	6.261114	6.133463	6.121263	5.957487	6.243422	6.191009	6.133198	6.08738	6.147257	6.072417	6.049589	6.104587	Zkscan6	NM_026107
6781830	1966	7.034193	7.276884	7.137102	7.315309	-0.28112	0.023346	-0.03843	0.712295	-0.17821	0.114271	0.139782	0.201919	-0.24269	0.042263	-0.10291	0.336028	6781830	7.010323	7.09199	7.000267	7.497152	7.139457	7.194043	7.200523	7.140361	7.070421	7.342689	7.17437	7.428868	Tmem220	NM_177392
6781840	1967	7.636312	7.21289	7.795812	6.086902	1.54941	0.288299	1.125988	0.432451	1.70891	0.245086	-0.58292	0.680008	0.423422	0.763882	-0.1595	0.90968	6781840	5.049013	7.149144	10.71078	8.235988	7.515584	5.887099	8.795781	7.311592	7.280062	5.353845	5.868999	7.037862	Myh8	NM_177369
6781846	1968	6.255027	6.392387	5.281609	6.437842	-0.18281	0.922981	-0.04545	0.980816	-1.15623	0.545632	1.110778	0.56118	-0.13736	0.942084	0.973418	0.609679	6781846	3.694751	6.402621	8.667709	8.591337	6.206178	4.379646	7.411322	4.133903	4.299601	3.740874	6.998008	8.574644	Myh4	NM_010855
6781885	1969	7.885481	7.787742	7.637007	7.76104	0.12444	0.602661	0.026701	0.910302	-0.12403	0.603826	0.150735	0.53	0.097739	0.68161	0.248474	0.310788	6781885	7.93741	7.89487	7.824162	7.998379	7.567356	7.79749	7.508906	8.186974	7.21514	7.703959	7.915137	7.664025	Gas7	NM_001109657
6781889	1970	6.165811	5.972217	6.447094	6.304633	-0.13882	0.20824	-0.33242	0.011224	0.142461	0.197716	-0.47488	0.001577	0.193594	0.092682	-0.28128	0.024162	6781889	6.064106	6.258017	6.17531	5.812988	6.015651	6.088011	6.55657	6.265618	6.519094	6.264932	6.249096	6.399872	Rcvrn	NM_009038
6781909	1972	7.629956	7.666546	7.65358	7.621849	0.008108	0.916378	0.044697	0.566772	0.031732	0.682668	0.012965	0.866733	-0.03659	0.63795	-0.02362	0.760281	6781909	7.661209	7.625652	7.603008	7.679731	7.705421	7.614485	7.703917	7.770374	7.48645	7.539195	7.605264	7.721087	Stx8	NM_018768
6781925	1973	7.865683	7.52672	7.593545	7.163786	0.701897	0.01656	0.362934	0.157014	0.429758	0.101619	-0.06682	0.781022	0.338963	0.182836	0.272138	0.27533	6781925	8.236483	7.807602	7.552964	7.610876	7.820723	7.148562	7.505547	7.530223	7.744864	7.140104	7.437864	6.913391	Pik3r5	NM_177320
6781927	1974	6.868622	6.796831	6.818013	6.644321	0.224301	0.033909	0.152511	0.120548	0.173693	0.083238	-0.02118	0.815405	0.071791	0.437168	0.050609	0.580143	6781927	6.955675	6.869878	6.780313	6.837071	6.889758	6.663665	6.78633	6.738338	6.929372	6.531276	6.77224	6.629446	Pik3r6	NM_001081566
6781928	1975	6.382648	6.08067	5.997697	6.196783	0.185865	0.283299	-0.11611	0.492884	-0.19909	0.252941	0.082973	0.621515	0.301978	0.098608	0.384951	0.044399	6781928	6.47144	6.164618	6.511886	6.115168	5.938745	6.188096	6.31344	5.855586	5.824064	6.326527	6.003281	6.260541	Mfsd6l	NM_146004
6781941	1977	7.612361	7.689995	7.273007	7.928906	-0.31654	0.023248	-0.23891	0.067637	-0.6559	0.000406	0.416988	0.00616	-0.07763	0.51188	0.339354	0.017065	6781941	7.568649	7.72251	7.545925	7.615567	7.756886	7.697532	7.289421	7.316748	7.212853	7.969897	7.666883	8.149939	Myh10	NM_175260
6781954	1978	6.567117	6.635696	6.646981	6.540416	0.026701	0.872135	0.09528	0.569548	0.106565	0.525811	-0.01129	0.945727	-0.06858	0.680754	-0.07986	0.632507	6781954	6.978727	6.376866	6.345757	6.620658	6.619475	6.666954	6.542396	6.754084	6.644463	6.402068	6.645381	6.573799	Rnf222	NM_177060
6781955	1979	6.556555	6.535115	6.601006	6.658466	-0.10191	0.668821	-0.12335	0.605623	-0.05746	0.808642	-0.06589	0.781364	0.02144	0.927879	-0.04445	0.851276	6781955	6.333306	6.631207	6.705153	5.992129	6.641118	6.972099	6.588288	6.563236	6.651493	6.811171	6.485627	6.678599	Rpl26	NM_009080
6781960	1980	9.378618	8.985776	9.389905	8.758073	0.620545	0.00093	0.227703	0.098207	0.631832	0.00083	-0.40413	0.010521	0.392842	0.012084	-0.01129	0.928377	6781960	9.451269	9.386022	9.298563	8.975763	9.01129	8.970274	9.231577	9.464992	9.473145	8.735135	9.021052	8.518031	Slc25a35	NM_028048
6781969	1981	8.699569	8.585809	8.531327	8.478477	0.221092	0.028977	0.107332	0.233265	0.05285	0.543201	0.054482	0.531131	0.11376	0.208892	0.168242	0.07791	6781969	8.714104	8.643821	8.740781	8.57688	8.666779	8.513767	8.57534	8.332875	8.685766	8.467131	8.505749	8.462551	1500010J	NR_026565
6781970	1982	6.519767	6.760882	6.371958	6.082836	0.436931	0.208038	0.678046	0.066276	0.289122	0.391287	0.388924	0.257542	-0.24112	0.471455	0.147809	0.655492	6781970	6.540055	6.254359	6.764886	6.402213	7.349982	6.530451	6.798637	5.792944	6.524292	5.965119	6.155447	6.127942	Aurkb	NM_011496
6781975	1983	6.61574	6.663177	6.793979	6.834123	-0.21838	0.247325	-0.17095	0.357231	-0.04014	0.824309	-0.1308	0.476161	-0.04744	0.793188	-0.17824	0.338215	6781975	6.26658	6.774209	6.68463	6.740452	6.546124	6.702954	6.83151	6.947481	6.602947	6.936502	6.989843	6.576024	Tmem107	NM_028336
6781982	1984	8.854113	8.946452	8.912308	9.029458	-0.17535	0.387411	-0.08301	0.676661	-0.11715	0.558356	0.034143	0.86316	-0.09234	0.64316	-0.0582	0.769352	6781982	8.888391	8.748545	8.925402	9.094619	8.819077	8.925659	8.630303	9.404949	8.701673	9.026152	8.933675	9.128548	Vamp2	NM_009497
6781984	1985	8.506618	8.816563	7.882155	9.250976	-0.74436	0.11515	-0.43441	0.332506	-1.36882	0.0117	0.934409	0.057307	-0.30995	0.4828	0.624463	0.176444	6781984	7.96551	8.195718	9.358625	9.380129	8.277635	8.791926	7.514141	8.045456	8.086867	9.6159	9.088862	9.048165	Per1	NM_001159367
6781986	1986	7.277906	7.255539	7.15975	7.340083	-0.06218	0.537198	-0.08454	0.406285	-0.18033	0.098457	0.095789	0.349733	0.022367	0.822441	0.118155	0.255409	6781986	7.187219	7.291766	7.354732	7.183965	7.169748	7.412904	7.331012	7.092298	7.055941	7.32301	7.262087	7.435152	Hes7	NM_033041
6782016	1991	9.813001	9.831581	9.778642	9.85813	-0.04513	0.776779	-0.02655	0.867307	-0.07949	0.619443	0.052938	0.739699	-0.01858	0.906873	0.034358	0.828915	6782016	9.706884	9.727398	10.00472	9.831472	9.884715	9.778555	9.934679	9.960327	9.440921	9.748359	9.78323	10.0428	Lsmd1	NM_030083
6782031	1992	9.803945	9.589542	9.796078	9.471927	0.332018	0.083631	0.117615	0.50389	0.324151	0.089904	-0.20654	0.254037	0.214403	0.237859	0.007867	0.963815	6782031	9.927625	9.751508	9.732701	9.512017	9.94961	9.306998	9.897105	9.766487	9.724642	9.354943	9.708802	9.352036	Trp53	NM_001127233
6782034	1993	6.268977	6.688145	6.40856	6.488761	-0.21978	0.110668	0.199384	0.142408	-0.0802	0.531202	0.279585	0.051963	-0.41917	0.009084	-0.13958	0.287676	6782034	6.341853	6.369614	6.095464	6.656534	6.606019	6.801882	6.548193	6.362402	6.315085	6.419636	6.327781	6.718867	Sat2	NM_026991
6782037	1994	8.341084	8.424667	8.288716	8.404778	-0.06369	0.477998	0.019889	0.822054	-0.11606	0.212069	0.135951	0.15081	-0.08358	0.357328	0.052367	0.557574	6782037	8.340082	8.348738	8.334431	8.431075	8.47421	8.368716	8.31954	8.470288	8.076321	8.359323	8.424028	8.430982	Fxr2	NM_011814
6782038	1995	7.267661	7.277364	7.371088	6.993715	0.273945	0.245045	0.283649	0.230127	0.377373	0.122209	-0.09372	0.679085	-0.0097	0.965645	-0.10343	0.648397	6782038	7.243475	7.494683	7.064824	7.247866	7.365197	7.219029	7.037864	7.812107	7.263294	6.920153	7.296609	6.764384	Sox15	NM_009235
6782080	1996	8.162258	8.007101	7.878277	7.800337	0.361921	0.007406	0.206765	0.076438	0.07794	0.465372	0.128825	0.240798	0.155157	0.165532	0.283981	0.023451	6782080	8.276576	8.078885	8.131313	7.99858	8.186294	7.83643	7.904243	7.968111	7.762476	7.735222	7.914313	7.751475	Plscr3	NM_023564
6782083	1997	7.826326	7.917147	7.920476	7.932876	-0.10655	0.253178	-0.01573	0.860285	-0.0124	0.889604	-0.00333	0.97025	-0.09082	0.324603	-0.09415	0.308281	6782083	7.760375	7.771315	7.947288	7.921019	8.0527	7.777721	7.880448	8.034489	7.846492	7.893568	7.890765	8.014295	Neurl4	NM_001013414
6782085	1998	6.308789	6.386595	6.311991	6.384425	-0.07564	0.539775	0.002171	0.985785	-0.07243	0.556676	0.074604	0.545186	-0.07781	0.528496	-0.0032	0.979031	6782085	6.372565	6.165171	6.38863	6.422213	6.380566	6.357007	6.556216	6.293555	6.086202	6.442277	6.260311	6.450686	Ybx2	NM_016875
6782087	1999	9.127982	8.892676	8.870065	8.664298	0.463684	0.003262	0.228378	0.075899	0.205767	0.103632	0.022611	0.845122	0.235307	0.068946	0.257918	0.050337	6782087	9.225597	9.018551	9.139799	8.80544	8.916801	8.955786	8.958878	9.012376	8.63894	8.725665	8.755372	8.511857	Cldn7	NM_016887

6782088	2000	8.740296	8.595283	8.549276	8.61163	0.128665	0.389247	-0.01635	0.910773	-0.06235	0.670767	0.046007	0.753143	0.145012	0.334911	0.19102	0.213502	6782088	8.652655	8.788196	8.780036	8.575396	8.660763	8.549691	8.297261	8.878801	8.471766	8.611959	8.758593	8.464339	Dullard	NM_026017
6782090	2001	11.8766	11.78783	11.93362	11.82736	0.04924	0.726293	-0.03952	0.778416	0.10626	0.456455	-0.14578	0.314335	0.088763	0.531668	-0.05702	0.685611	6782090	11.88416	11.91629	11.82934	11.88223	11.87111	11.61016	11.78815	12.26313	11.74957	11.80573	11.89151	11.78483	Gabarap	NM_019749
6782091	2002	7.849723	8.06074	7.829988	7.775899	0.073824	0.532389	0.284841	0.035948	0.054089	0.645405	0.230752	0.075738	-0.21102	0.099163	0.019736	0.865862	6782091	7.896404	7.739747	7.913019	8.00549	8.190872	7.985858	7.901024	8.003861	7.585078	7.764184	7.697256	7.866257	Phf23	NM_030064
6782092	2003	7.284951	7.450652	7.2722	7.299932	-0.01498	0.824613	0.15072	0.050162	-0.02773	0.682798	0.178451	0.025932	-0.1657	0.035095	0.012751	0.850323	6782092	7.271037	7.278601	7.305215	7.554339	7.469305	7.328311	7.227707	7.39747	7.191424	7.298068	7.315729	7.285999	Dvl2	NM_007888
6782093	2004	7.673613	7.81895	7.594118	7.685011	-0.0114	0.92967	0.133939	0.315728	-0.09089	0.488363	0.224832	0.110132	-0.14534	0.278982	0.079495	0.543027	6782093	7.708869	7.725729	7.586241	7.956057	7.854014	7.646779	7.411128	7.873618	7.497609	7.717729	7.623096	7.714208	Dlg4	NM_007864
6782096	2005	8.957228	8.704827	8.495219	9.129916	-0.17269	0.69573	-0.42509	0.347409	-0.6347	0.174449	0.209607	0.635795	0.252401	0.569754	0.462009	0.309572	6782096	9.011167	9.180749	8.679768	8.817884	8.065062	9.231534	8.061917	9.436603	7.987138	9.213663	9.042106	9.133979	Asgr1	NM_009714
6782102	2007	7.365208	6.671427	6.634306	6.296826	1.068382	0.111646	0.374601	0.548267	0.33748	0.587777	0.037121	0.951998	0.69378	0.279176	0.730902	0.256152	6782102	8.050985	7.401386	6.643252	6.56674	7.188156	6.259386	7.096556	5.598241	7.208121	5.53126	6.261112	7.098106	Mgl2	NM_145137
6782105	2008	6.048975	5.679755	5.87106	5.140369	0.908606	0.011236	0.539386	0.087609	0.730692	0.029926	-0.19131	0.509719	0.36922	0.219658	0.177915	0.53902	6782105	6.226013	5.6015	6.319412	5.490466	6.220006	5.328793	5.951751	5.855558	5.805872	4.827408	5.229897	5.363801	Mgl1	NM_010796
6782109	2009	9.120245	9.095834	9.311883	9.373954	-0.25371	0.071972	-0.27812	0.052752	-0.06207	0.625837	-0.21605	0.115618	0.024411	0.846934	-0.19164	0.156145	6782109	9.20273	9.082648	9.075356	9.221881	9.204608	8.861012	9.153803	9.537339	9.244508	9.405128	9.30858	9.408154	Slc16a11	NM_153081
6782125	2010	7.474669	7.424689	7.479976	6.85438	0.620289	0.015753	0.57031	0.022954	0.625596	0.015141	-0.05529	0.792436	0.049979	0.81189	-0.00531	0.979802	6782125	7.76353	7.363863	7.296613	7.561857	7.687895	7.024316	7.638395	7.489136	7.312396	6.786671	7.060642	6.715826	Arrb2	NM_145429
6782127	2011	7.248628	7.337065	7.223699	7.041069	0.20756	0.266018	0.295996	0.126537	0.18263	0.323464	0.113366	0.531997	-0.08844	0.624158	0.024929	0.889351	6782127	7.305117	7.394233	7.046535	7.207858	7.548335	7.255001	7.386044	7.296581	6.988472	6.782931	7.313828	7.026447	Med11	NM_025397
6782132	2014	10.97114	11.05713	10.92971	10.85561	0.115527	0.325465	0.201513	0.105083	0.074097	0.520592	0.127417	0.281285	-0.08599	0.45802	0.04143	0.71693	6782132	10.87701	10.94928	11.08713	10.98344	11.12383	11.06411	11.2025	10.82084	10.76579	10.86118	10.85768	10.84798	Psmb6	NM_008946
6782134	2015	8.18718	8.12337	8.101074	7.93785	0.24933	0.007001	0.18552	0.028044	0.163224	0.046281	0.022296	0.755885	0.06381	0.384053	0.086106	0.249198	6782134	8.183046	8.176808	8.201685	8.129834	8.217766	8.022509	8.127884	8.10729	8.068048	7.932555	8.075163	7.805832	Pld2	NM_008876
6782136	2016	8.949809	9.012084	8.943613	8.922269	0.027539	0.803064	0.089815	0.424878	0.021343	0.846619	0.068472	0.539452	-0.06228	0.575962	0.006196	0.955169	6782136	9.024195	8.916537	8.908694	8.99675	9.102407	8.937096	8.746858	9.205684	8.878296	8.894807	8.965927	8.906074	Mink1	NM_001045959
6782139	2018	8.685349	8.772164	8.877793	9.010408	-0.32506	0.006729	-0.23824	0.028931	-0.13261	0.177379	-0.10563	0.272607	-0.08682	0.361267	-0.19244	0.064136	6782139	8.565763	8.740808	8.749476	8.839	8.703993	8.773499	8.79731	8.936192	8.899877	8.988955	8.855611	9.186657	Rnf167	NM_027445
6782141	2019	8.96553	8.851316	8.71457	8.867545	0.097985	0.936873	-0.01623	0.989532	-0.15298	0.901622	0.136747	0.912005	0.114214	0.92645	0.250961	0.839433	6782141	7.094017	8.565104	11.23747	10.14374	8.824498	7.585711	9.948571	8.276729	7.918409	7.509444	9.683472	9.409719	Eno3	NM_007933
6782147	2020	8.977934	8.968557	8.716942	8.665723	0.312211	0.052787	0.302834	0.058715	0.051219	0.719122	0.251616	0.104567	0.009376	0.947292	0.260992	0.094164	6782147	9.04647	8.798445	9.088886	9.101846	9.035194	8.768632	8.829484	8.792283	8.529058	8.465883	8.795667	8.735619	Kif1c	NM_153103
6782149	2021	6.955032	7.020114	6.828341	6.650819	0.304214	0.047189	0.369295	0.021667	0.177522	0.208713	0.191773	0.177911	-0.06508	0.629685	0.126691	0.35775	6782149	6.917055	6.973784	6.974258	7.047915	6.978225	7.034202	6.918375	6.941064	6.625584	6.374612	6.891485	6.686359	Kif1c	NM_153103
6782153	2022	6.822446	6.951742	6.878345	6.876464	-0.05402	0.672447	0.075279	0.557876	0.001882	0.98818	0.073397	0.567548	-0.1293	0.324313	-0.0559	0.661866	6782153	6.870965	6.837253	6.75912	6.713299	7.092306	7.049622	6.973983	6.978449	6.682604	6.81201	7.021384	6.795997	Zfp3	NM_177565
6782164	2023	8.349968	8.442656	8.388657	8.472542	-0.12257	0.166115	-0.02989	0.719931	-0.08388	0.327579	0.053999	0.520995	-0.09269	0.282538	-0.03869	0.643457	6782164	8.337458	8.397905	8.31454	8.288746	8.518454	8.520769	8.470788	8.379631	8.315553	8.433231	8.382803	8.601591	Rabep1	NM_019400
6782172	2024	7.261956	7.38854	7.289602	7.160897	0.101059	0.270779	0.227643	0.028594	0.128704	0.170339	0.098938	0.280203	-0.12658	0.17668	-0.02765	0.754527	6782172	7.275264	7.315993	7.194611	7.408733	7.309545	7.447342	7.328464	7.349116	7.191225	7.302044	7.202623	6.978025	Rpain	NM_027186
6782177	2025	7.081335	7.250804	7.039188	7.125498	-0.04416	0.833942	0.125306	0.555904	-0.08631	0.683214	0.211616	0.329668	-0.16947	0.42998	0.042148	0.841394	6782177	7.0556	6.987458	7.200948	7.004768	7.550267	7.197376	6.933704	7.426745	6.757114	6.957058	7.34855	7.070885	Mis12	NM_025993
6782201	2026	6.728764	7.031628	6.773648	7.038164	-0.3094	0.05832	-0.00654	0.963955	-0.26452	0.095845	0.257981	0.102962	-0.30286	0.062717	-0.04488	0.75701	6782201	6.643053	6.695666	6.847574	6.932545	7.021275	7.141065	6.464634	6.964985	6.891324	6.869512	7.159159	7.08582	Wscd1	NM_177618
6782246	2029	7.755065	7.678996	7.776904	7.652252	0.102812	0.090217	0.026743	0.629797	0.124652	0.04774	-0.09791	0.103912	0.076069	0.191889	-0.02184	0.693122	6782246	7.73621	7.708462	7.820522	7.799813	7.648507	7.588667	7.741259	7.768821	7.820632	7.63783	7.651655	7.667272	Ggt6	NM_027819
6782248	2030	8.608175	8.700889	8.714921	8.474608	0.133567	0.242763	0.226282	0.065112	0.240313	0.052959	-0.01403	0.897867	-0.09271	0.406838	-0.10675	0.342987	6782248	8.593635	8.611452	8.619438	8.646425	8.810964	8.645279	8.928616	8.507513	8.708633	8.382581	8.606164	8.435078	Mybbp1a	NM_016776
6782255	2031	8.930763	8.993182	8.917984	8.899558	0.031205	0.765655	0.093625	0.381875	0.018426	0.860027	0.075198	0.478621	-0.06242	0.554448	0.012779	0.902617	6782255	8.98449	8.87116	8.936638	8.877863	9.172814	8.92887	9.070357	8.910465	8.77313	8.778494	8.94527	8.974909	Ube2g1	NM_025985
6782256	2032	9.115096	9.130426	9.13055	8.747594	0.367502	0.00536	0.382832	0.004287	0.382956	0.004279	-0.00012	0.99901	-0.01533	0.878504	-0.01545	0.877528	6782256	9.113235	9.137392	9.094662	9.012403	9.322728	9.056146	9.214923	9.034781	9.141946	8.666189	8.909743	8.66685	Ankfy1	NM_009671
6782273	2033	6.552726	6.672799	6.516172	7.204619	-0.65189	0.071103	-0.53182	0.128139	-0.68845	0.059294	0.156627	0.630686	-0.12007	0.711597	0.036554	0.910022	6782273	6.557639	6.574402	6.526136	7.166541	6.535753	6.316103	6.597089	6.786472	6.164955	7.040022	6.765385	7.808449	Atp2a3	NM_016745
6782277	2035	7.287093	7.507053	7.58343	8.015678	-0.72858	0.012538	-0.50863	0.055713	-0.43225	0.09385	-0.07638	0.745607	-0.21996	0.361735	-0.29634	0.228778	6782277	7.085588	7.524633	7.251058	7.643402	7.282324	7.595432	7.511186	7.932919	7.306186	7.732466	7.911556	8.403012	Camkk1	NM_018883
6782278	2036	8.372397	8.453587	8.414582	8.591075	-0.21868	0.147536	-0.13749	0.342953	-0.17649	0.231767	0.039005	0.782161	-0.08119	0.568115	-0.04219	0.765001	6782278	8.370675	8.458458	8.288057	8.348013	8.609829	8.40292	8.32222	8.700335	8.221191	8.470159	8.548888	8.754179	1200014J	NM_025818
6782290	2039	5.922068	6.19729	6.007666	6.168423	-0.24635	0.091032	0.028868	0.827579	-0.16076	0.245494	0.189625	0.177577	-0.27522	0.064209	-0.0856	0.523345	6782290	5.988126	6.036549	5.74153	6.18691	6.124751	6.28021	5.822055	6.186863	6.014079	6.093918	6.032184	6.379166	Shpk	NM_029031
6782368	2050	8.273567	8.196175	8.28654	8.511629	-0.23806	0.038671	-0.31545	0.011291	-0.22509	0.047712	-0.09037	0.375777	0.077392	0.445072	-0.01297	0.89622	6782368	8.290292	8.230298	8.30011	8.277914	8.158171	8.152439	8.132067	8.415411	8.312143	8.327028	8.547906	8.659954	1300001I	BC072573
6782389	2051	7.57203	7.605175	7.592449	7.581792	-0.00976	0.945086	0.023384	0.869047	0.010658	0.940059	0.012726	0.92846	-0.03315	0.815384	-0.02042	0.885499	6782389	7.535895	7.611486	7.568708	7.431371	7.864705	7.51945	7.491865	7.716037	7.569446	7.416715	7.825295	7.503365	Mett10d	NM_026197
6782405	2052	7.436993	7.465738	7.45576	7.511038	-0.07404	0.560514	-0.0453	0.719898	-0.05528	0.662342	0.009978	0.936793	-0.02874	0.819556	-0.01877	0.88149	6782405	7.468999	7.469252	7.372728	7.608241	7.500352	7.28862	7.284611	7.718064	7.364605	7.603641	7.458709	7.470764	Mnt	NM_010813
6782411	2053	7.254476	7.254041	7.351849	7.327432	-0.07296	0.577135	-0.07339	0.574916	0.024417	0.850632	-0.09781	0.458334	0.000434	0.997324	-0.09737	0.460262	6782411	7.188371	7.330884	7.244172	7.057028	7.472976	7.23212	7.415666	7.41202	7.227862	7.176667	7.533983	7.271647	Tsr1	NM_177325
6782414	2054	7.414876	7.373381	7.302817	7.389935	0.024941	0.609086	-0.01655	0.73306	-0.08712	0.100118	0.070564	0.170595	0.041495	0.401797	0.112059	0.043793	6782414	7.415775	7.429444	7.399409	7.423722	7.403112	7.293309	7.258476	7.40025	7.249724	7.372828	7.372403	7.424575	Smg6	NM_001002764
6782422	2055	6.439547	6.338126	6.368584	5.981851	0.457696	0.046407	0.356276	0.104284	0.386733	0.08192	-0.03046	0.879416	0.101421	0.616096	0.070963	0.724615	6782422	6.407479	6.307666	6.603496	6.62842	6.381876	6.004083	6.485575	6.289802	6.330374	5.757298	6.332538	5.855716	Rtn4rl1	NM_177708
6782430	2056	5.995349	5.953761	5.887094	5.942853	0.052497	0.368647	0.010908	0.848016	-0.05576	0.341218	0.066667	0.260875	0.041588	0.472048	0.108256	0.085057	6782430	5.919884	5.988498	6.077666	6.012871	5.867542	5.98087	5.951427	5.842268	5.867586	5.91617	5.90792	6.004468	Smyd4	NM_001102611
6782443	2057	6.988569	7.007045	7.014105	6.748431	0.240139	0.051249	0.258614	0.038928	0.265675	0.035051	-0.00706	0.947968	-0.01848	0.864514	-0.02554	0.813719	6782443	6.87025	7.03369	7.061768	6.881511	7.154466	6.985158	6.897568	6.992255	7.152493	6.757862	6.883764	6.603666	Tlcd2	NM_027249
6782447	2059	9.920104	10.02007	9.985999	9.857225	0.062879	0.387024	0.162848	0.045297	0.128774	0.097873	0.034074	0.633409	-0.09997	0.183903	-0.0659	0.365777	6782447	9.928442	9.956721	9.875149	9.961019	10.10283	9.99637	10.08954	9.942567	9.925891	9.72593	9.93479	9.910955	Prpf8	NM_138659
6782449	2060	6.901657	6.944006	6.922242	7.02521	-0.12355	0.042605	-0.0812	0.152081	-0.10297	0.079616	0.021764	0.682549	-0.04235	0.432985	-0.02058	0.698716	6782449	6.962183	6.920295	6.822493	6.939577	6.933863	6.958578	6.979969	6.909307	6.87745	6.9386	7.022651	7.114379	Scarf1	NM_001004157
6782451	2060	9.75838	9.623128	9.98389	10.41789	-0.65951	0.18688	-0.79476	0.120137	-0.434	0.369977	-0.36076	0.452525	0.135252	0.774748	-0.22551	0.634869	6782451	9.956146	9.809155	9.509839	10.17644	9.299093	9.393851	9.400901	10.66444	9.88633	10.15327	9.835972	11.26442	Slc43a2	NM_173388
6782454	2061	9.505064	9.531984	9.52519	9.406566	0.098498	0.416048	0.125418	0.306614	0.118624	0.331876	0.006794	0.954279	-0.02692	0.820566	-0.02013	0.865246	6782454	9.640201	9.459884	9.415108	9.525374	9.699973	9.370606	9.680469	9.460472	9.43463	9.247545	9.510039	9.462115	Pitpna	NM_008850
6782456	2062	9.476524	9.736367	9.523415	9.539691	-0.06317	0.516156	0.196676	0.067363	-0.01628	0.865416	0.212952	0.051276	-0.25984	0.023411	-0.04689	0.627698	6782456	9.427161	9.479053	9.523357	9.811591	9.728332	9.669179	9.608531	9.534829	9.426885	9.477062	9.388889	9.753122	Inpp5k	NM_008916
6782457	2063	9.211061	9.120926	9.177616	8.92454	0.286522	0.029911	0.196386	0.108459	0.253076	0.048307	-0.05669	0.616146	0.090136	0.431055	0.033445	0.766194	6782457	9.252796	9.16404	9.216348	9.139788	9.281842	8.941147	9.285234	9.152147	9.095467	8.763374	9.108357	8.901888	Myo1c	NM_008659
6782458	2064	8.925107	8.981647	8.883746	8.896337	0.02877	0.837801	0.08531	0.548079	-0.01259	0.928535	0.097901	0.492234	-0.05654	0.688623	0.041361	0.768862	6782458	8.907896	8.935925	8.931501	8.817824	9.180307	8.946811	8.869135	9.121279	8.660824	8.875826	9.06015	8.753035	Crk	NM_133656
6782465	2065	11.38434	11.23798	11.34078	11.33732	0.04702	0.685793	-0.09934	0.401173	0.003457	0.976145	-0.1028	0.385743	0.14636	0.227783	0.043563	0.707576	6782465	11.362	11.35565	11.43537	11.16755	11.4205	11.12589	11.22042	11.57867	11.22324	11.25221	11.38813	11.37162	Ywhae	NM_009536
6782484	2066	6.727472	6.875657	6.812541	6.906317	-0.17885	0.16113	-0.03066	0.797915	-0.09378	0.44155	0.063115	0.600654	-0.14819	0.236604	-0.08507	0.48362	6782484	6.714787	6.764318	6.70331	6.784782	6.950412	6.891776	6.635018	7.037416	6.76519	7.103271	6.774262	6.841419	1700016K	NM_198637
6782498	2068	6.348049	6.574451	6.462092	6.498752	-0.1507	0.282275	0.075699	0.578486	-0.03666	0.786262	0.112359	0.415082	-0.2264	0.12154	-0.11404	0.408429	6782498	6.590543	6.345446	6.108159	6.560688	6.711627	6.451038	6.390378	6.632115	6.363782	6.414163	6.545228	6.536865	Rnmtl1	NM_183263
6782515	2069	7.011884	7.064853	6.966255	6.95334	0.058544	0.651489	0.111514	0.397618	0.012916	0.920115	0.098598	0.452248	-0.05297	0.682411	0.045629	0.724112	6782515	6.9928	7.027795	7.015057	7.033856	7.155005	7.005699	7.257468	6.87942	6.761878	6.799316	6.985866	7.074837	Timm22	NM_019818
6782533	2071	6.624128	6.641083	6.248223	6.503414	0.120714	0.67828	0.137669	0.636729	-0.25519	0.389503	0.392859	0.198891	-0.01696	0.953281	0.375977	0.216994	6782533	6.646735	6.678185	6.547463	6.839145	6.752081	6.332022	5.877389	6.649221	6.21806	5.947137	6.89173	6.671375	Tusc5	NM_177709
6782553	2073	8.5929	8.606888	8.527168	8.832294	-0.23939	0.165874	-0.22541	0.189069	-0.30513	0.087914	0.07972	0.625367	-0.01399	0.931207	0.065732	0.686513	6782553	8.545464	8.583473	8.649762	8.425126	8.787347	8.60819	8.65044	8.542876	8.388187	8.581664	9.176379	8.738838	Blmh	NM_178645
6782554	2074	6.544329	6.747931	6.582419	8.46304	-1.91871	0.001176	-1.71511	0.002315	-1.88062	0.001331	0.165512	0.682933	-0.2036	0.616322	-0.03809	0.924718	6782554	6.358208	6.569624	6.705155	6.900964	6.709268	6.633562	6.57472	6.739805	6.432733	7.890786	7.976707	9.521627	Slc6a4	NM_010484
6782563	2075	8.181008	8.197362	7.811918	7.851599	0.329409	0.035132	0.345763	0.028897	-0.03968	0.768121	0.385444	0.018063	-0.01635	0.903058	0.36909	0.021903	6782563	8.359526	8.080624	8.102874	8.240522	8.377273	7.974291	7.86684	7.753515	7.815399	7.736565	8.058676	7.759557	Ssh2	NM_177710
6782569	2076	7.124805	6.930156	6.855227	7.036322	0.088482	0.831364	-0.10617	0.798455	-0.18109	0.664435	0.074928	0.856832	0.194649	0.641343	0.269577	0.521518	6782569	6.431446	7.002776	7.940192	7.474274	6.797101	6.519092	6.866938	6.985631	6.713113	6.716515	7.427211	6.965241	Coro6	NM_139128
6782572	2077	8.611255	8.657192	8.510189	8.516577	0.094679	0.394795	0.140615	0.218457	-0.00639	0.95311	0.147003	0.200184	-0.04594	0.674162	0.101067	0.365219	6782572	8.636866	8.606007	8.590893	8.741258	8.719587	8.510731	8.562918	8.645839	8.321809	8.348888	8.582056	8.618786	Git1	NM_001004144
6782579	2078	6.241907	6.323698	6.209756	6.273074	-0.03117	0.774988	0.050624	0.643867	-0.06332	0.564639	0.113942	0.311182	-0.08179	0.460067	0.032151	0.76812	6782579	6.317571	6.267574	6.140576	6.195142	6.557486	6.218465	6.30628	6.12083	6.202158	6.375605	6.195862	6.247754	1300007F	NM_026185
6782610	2079	8.211906	8.265286	8.071577	8.193779	0.018127	0.856554	0.071507	0.482502	-0.1222	0.243685	0.193708	0.081147	-0.05338	0.597502	0.140329	0.186403	6782610	8.188345	8.251942	8.195431	8.369478	8.284534	8.141845	8.105381	8.089037	8.020314	7.962843	8.318521	8.299973	Myo18a	NM_011586
6782643	2081	8.390746	8.438205	8.405336	8.365183	0.025563	0.825866	0.073022	0.534288	0.040153	0.730269	0.032869	0.777493	-0.04746	0.684103	-0.01459	0.899971	6782643	8.381562	8.41599	8.374687	8.448545	8.584368	8.281702	8.278797	8.659989	8.277222	8.3316	8.435954	8.327995	Phf12	NM_174852
6782656	2083	8.857823	8.789153	8.730958	8.487331	0.370492	0.028316	0.301822	0.061245	0.243627	0.117082	0.058195	0.68585	0.068669	0.63386	0.126864	0.387124	6782656	8.963074	8.822061	8.788333	8.881683	8.925023	8.560754	8.964695	8.5797	8.64848	8.329975	8.485255	8.646763	Flot2	NM_008028
6782668	2084	7.771808	7.838746	7.70536	7.755662	0.016146	0.910383	0.083085	0.566522	-0.0503	0.726791	0.133387	0.365315	-0.06694	0.642982	0.066448	0.645385	6782668	7.88003	7.785365	7.650028	7.899704	8.019408	7.597127	7.555374	7.967351	7.593354	7.789664	7.788371	7.68895	BC017647	NM_145430

6782679	2085	7.982821	8.20261	8.11174	8.374883	-0.39206	0.012275	-0.17227	0.195066	-0.26314	0.062799	0.090869	0.477091	-0.21979	0.108878	-0.12892	0.320876	6782679	7.848504	8.070115	8.029845	8.272563	8.073649	8.261617	7.973682	8.368521	7.993018	8.335635	8.285345	8.503668	Tlcd1	NM_026708
6782680	2086	7.746204	7.940672	7.686528	7.931802	-0.1856	0.143331	0.00887	0.940096	-0.24527	0.064342	0.254144	0.057016	-0.19447	0.127525	0.059676	0.616	6782680	7.755023	7.820228	7.66336	7.90601	8.076422	7.839584	7.638144	7.88283	7.53861	7.848275	7.829494	8.117638	Rab34	NM_033475
6782682	2087	8.398184	8.538345	8.433675	8.538329	-0.14014	0.065409	1.6E-05	0.999812	-0.10465	0.149738	0.10467	0.149684	-0.14016	0.065384	-0.03549	0.603657	6782682	8.397732	8.358227	8.438593	8.469843	8.542707	8.602485	8.567507	8.427402	8.306117	8.515767	8.598221	8.500999	Sdf2	NM_009143
6782685	2088	10.01545	10.05053	9.987083	9.885538	0.129917	0.150465	0.164992	0.078124	0.101545	0.2491	0.063447	0.459753	-0.03508	0.679023	0.028371	0.737356	6782685	10.03811	9.976914	10.03134	10.05997	10.08898	10.00264	10.12507	10.05642	9.77976	9.817647	9.930526	9.908441	2610507B	NM_001002004
6782694	2090	6.279484	6.462835	6.092004	6.622079	-0.34259	0.097863	-0.15924	0.409188	-0.53008	0.019925	0.370831	0.077093	-0.18335	0.345357	0.18748	0.33522	6782694	6.19154	6.271731	6.375181	6.328666	6.504245	6.555593	6.272247	5.650107	6.353657	6.796482	6.4509	6.618855	Aldoc	NM_009657
6782695	2091	8.436482	8.181018	8.30235	8.377796	0.058687	0.621444	-0.19678	0.12338	-0.07545	0.527671	-0.12133	0.319349	0.255464	0.055816	0.134132	0.27425	6782695	8.463983	8.310766	8.534698	8.0479	8.405135	8.09002	8.378757	8.117033	8.411261	8.416242	8.361017	8.356128	Pigs	NM_201406
6782697	2092	7.588211	7.758581	7.615211	7.664805	-0.07659	0.317762	0.093776	0.228345	-0.04959	0.50978	0.14337	0.081222	-0.17037	0.04525	-0.027	0.71699	6782697	7.597148	7.562581	7.604905	7.913614	7.77698	7.585148	7.593884	7.659436	7.592313	7.615838	7.691905	7.686672	Unc119	NM_011676
6782701	2093	8.844445	8.928664	8.931819	8.73552	0.108926	0.440279	0.193144	0.187875	0.1963	0.181522	-0.00316	0.98181	-0.08422	0.547618	-0.08737	0.533083	6782701	8.878523	8.6807	8.974113	8.678411	9.110505	8.997076	9.015265	9.051735	8.728458	8.663912	8.757463	8.785184	Slc46a1	NM_026740
6782702	2094	5.875506	6.263256	5.903907	5.983478	-0.10797	0.658106	0.279778	0.267919	-0.07957	0.743603	0.35935	0.164721	-0.38775	0.13752	-0.0284	0.906779	6782702	6.21764	5.434075	5.974803	6.2936	6.486547	6.009622	6.165152	5.628153	5.918415	5.760981	6.035828	6.153626	Vtn	NM_011707
6782703	2095	6.442451	6.410156	6.502457	6.592891	-0.15044	0.076748	-0.18274	0.038898	-0.09043	0.256902	-0.0923	0.248	0.032295	0.67438	-0.06001	0.441329	6782703	6.5318	6.428082	6.36747	6.495713	6.399328	6.335426	6.575209	6.348158	6.584003	6.583422	6.637165	6.558087	Sebox	NM_008759
6782704	2096	8.143695	8.363129	8.090723	8.449689	-0.30599	0.046093	-0.08656	0.523497	-0.35897	0.024434	0.272406	0.069015	-0.21943	0.129292	0.052972	0.693816	6782704	8.182774	8.110295	8.138016	8.363474	8.39737	8.328542	8.167621	8.244038	7.86051	8.27947	8.346205	8.723392	Poldip2	NM_026389
6782708	2097	9.763778	9.810441	9.826073	9.935103	-0.17133	0.226061	-0.12466	0.367861	-0.10903	0.428131	-0.01563	0.907693	-0.04666	0.730169	-0.0623	0.646195	6782708	9.737259	9.746628	9.807446	9.612946	9.902994	9.915382	9.724298	10.11903	9.634892	9.901582	10.01843	9.885298	Ift20	NM_018854
6782776	2099	8.930685	9.03723	8.83539	8.886148	0.044537	0.440716	0.151082	0.024989	-0.05076	0.382265	0.20184	0.006252	-0.10654	0.088265	0.095295	0.120832	6782776	8.984684	8.963872	8.8435	8.977337	9.136987	8.997367	8.817179	8.811367	8.877624	8.852238	8.953147	8.853059	Nf1	NM_010897
6782777	2100	7.13182	7.266724	7.193736	7.379233	-0.24741	0.143171	-0.11251	0.481482	-0.1855	0.258249	0.072988	0.644828	-0.1349	0.401899	-0.06192	0.69521	6782777	7.118491	7.080454	7.196516	7.392267	7.219764	7.188142	7.229342	7.251593	7.100274	7.128058	7.24061	7.769032	Rab11fip	NM_175543
6782801	2101	8.634628	8.786839	8.689274	8.73625	-0.10162	0.209266	0.05059	0.515844	-0.04698	0.545524	0.097565	0.226254	-0.15221	0.075065	-0.05465	0.483752	6782801	8.667139	8.716704	8.520041	8.657862	8.81673	8.885926	8.734859	8.710297	8.622667	8.668797	8.722994	8.816958	Suz12	NM_199196
6782808	2102	7.412569	7.540077	7.5937	6.915101	0.497468	0.050373	0.624976	0.020166	0.678599	0.013823	-0.05362	0.81034	-0.12751	0.571576	-0.18113	0.426411	6782808	7.734344	7.378863	7.124499	7.708248	7.636476	7.275507	7.733754	7.365415	7.681931	6.661544	7.253529	6.83023	Adap2	NM_172133
6782811	2103	6.059568	6.194089	6.054721	5.886282	0.173285	0.245368	0.307807	0.056576	0.168438	0.25772	0.139368	0.342848	-0.13452	0.358958	0.004847	0.972886	6782811	5.775516	6.262015	6.141172	6.190611	6.171913	6.219743	6.173264	5.828614	6.162284	5.76565	5.971208	5.921989	Rnf135	NM_028019
6782812	2104	8.53473	8.616198	8.600633	8.541383	-0.00665	0.915051	0.074814	0.25087	0.05925	0.355643	0.015565	0.803274	-0.08147	0.214604	-0.0659	0.307286	6782812	8.49824	8.609981	8.495968	8.524158	8.702749	8.621686	8.593332	8.622117	8.586356	8.439778	8.631794	8.552578	Rhot1	NM_021536
6782814	2105	6.731958	6.674475	6.821975	6.671521	0.060437	0.474362	0.002954	0.971631	0.150454	0.098605	-0.1475	0.104317	0.057483	0.495553	-0.09002	0.295996	6782814	6.790938	6.635951	6.768984	6.756964	6.660737	6.605723	6.690998	6.861657	6.913269	6.716169	6.753951	6.544442	Rhbdl3	NM_139228
6782818	2106	10.44939	10.55068	10.43679	10.39577	0.05362	0.547479	0.154913	0.107153	0.04102	0.643761	0.113893	0.218922	-0.10129	0.269485	0.0126	0.886324	6782818	10.53863	10.44577	10.36377	10.37317	10.70352	10.57536	10.51993	10.44349	10.34695	10.41258	10.41216	10.36257	Zfp207	NM_001130169
6782828	2107	11.15954	11.09225	10.90804	11.01624	0.143303	0.173852	0.07601	0.451331	-0.10819	0.292404	0.184203	0.091287	0.067293	0.503196	0.251497	0.030662	6782828	11.20812	11.17334	11.09716	11.08244	10.94321	11.25109	10.96429	10.95294	10.8069	10.86598	11.15193	11.0308	Psmd11	NM_178616
6782859	2109	9.220019	9.38596	9.148831	8.789303	0.430716	0.021851	0.596657	0.004341	0.359528	0.045271	0.237129	0.156699	-0.16594	0.305915	0.071188	0.651448	6782859	9.001949	9.251989	9.40612	9.304592	9.666107	9.187181	9.235358	8.991095	9.220041	8.687201	8.752796	8.927912	Tmem98	NM_029537
6782861	2110	6.210111	6.262443	6.199293	6.286639	-0.07653	0.504822	-0.0242	0.830808	-0.08735	0.448497	0.06315	0.580348	-0.05233	0.645804	0.010818	0.923804	6782861	6.236994	6.24784	6.1455	6.172115	6.186712	6.428502	6.310075	6.136371	6.151434	6.497588	6.106689	6.255641	Spaca3	NM_029367
6782915	2111	6.997664	6.780575	7.153145	6.124887	0.872776	0.068829	0.655687	0.153122	1.028258	0.038392	-0.37257	0.39597	0.217089	0.615417	-0.15548	0.717917	6782915	7.74551	6.929476	6.318005	6.676719	7.162094	6.502911	7.575727	6.696174	7.187535	5.703278	6.615197	6.056187	Ccl2	NM_011333
6782916	2112	7.289192	6.735448	7.296582	6.291593	0.997853	0.062072	0.444109	0.362984	1.005243	0.060538	-0.56113	0.257636	0.553744	0.263458	-0.00739	0.987587	6782916	7.934458	7.196105	6.737013	6.707169	7.342774	6.156402	7.682854	6.559566	7.647326	5.985323	6.720771	6.167924	Ccl7	NM_013654
6782917	2113	8.38182	8.112717	7.555923	7.624229	0.757591	0.143983	0.488488	0.326878	-0.06831	0.887515	0.556794	0.268044	0.269103	0.580913	0.825897	0.115458	6782917	8.774058	7.672301	8.6991	8.398604	8.800596	7.138951	7.739101	7.392661	7.536006	7.77112	7.919354	7.182212	Ccl11	NM_011330
6782918	2114	7.728946	7.299449	7.95058	6.351977	1.376968	0.055107	0.947472	0.161202	1.598602	0.031381	-0.65113	0.319677	0.429496	0.503866	-0.22163	0.727346	6782918	8.066502	7.584962	7.535373	7.035895	8.241479	6.620974	8.458057	7.3957	7.997982	5.987956	7.574142	5.493834	Ccl12	NM_011331
6782919	2115	8.442033	7.248709	7.223206	6.860911	1.581122	0.020867	0.387798	0.501628	0.362295	0.529409	0.025503	0.964227	1.193325	0.062284	1.218828	0.057949	6782919	8.823906	7.927198	8.574996	7.016171	8.335546	6.394409	7.723431	6.66202	7.284166	6.729861	7.499492	6.35338	Ccl8	NM_021443
6782958	2117	6.964606	7.121364	6.948133	6.947306	0.0173	0.841474	0.174058	0.071248	0.000827	0.992364	0.173231	0.072349	-0.15676	0.098076	0.016473	0.848937	6782958	6.945213	6.826029	7.122575	7.008513	7.181511	7.174068	6.837731	7.0187	6.987967	6.913978	6.968586	6.959354	Zfp830	NM_025884
6782959	2118	7.467454	7.618466	7.497329	7.512102	-0.04465	0.723089	0.106364	0.407355	-0.01477	0.906327	0.121137	0.34846	-0.15101	0.249579	-0.02987	0.812166	6782959	7.463122	7.469475	7.469766	7.501218	7.768507	7.585674	7.465894	7.762192	7.263902	7.435859	7.49624	7.604208	Lig3	NM_010716
6782977	2119	5.944803	5.837435	5.80519	5.961035	-0.01623	0.947432	-0.1236	0.618458	-0.15584	0.531965	0.032246	0.895834	0.107368	0.664657	0.139613	0.574581	6782977	5.607913	5.861215	6.365281	6.178851	5.782263	5.551192	6.060164	5.755017	5.600388	5.738183	6.085677	6.059244	Unc45b	NM_178680
6782979	2120	8.17702	8.051813	7.824399	7.757622	0.419398	0.093079	0.294191	0.21796	0.066777	0.76925	0.227414	0.331556	0.125207	0.58493	0.352621	0.147677	6782979	8.439214	8.175097	7.916749	7.99551	8.260967	7.898962	8.095269	7.47128	7.906648	7.437284	8.005522	7.830059	Slfn5	NM_183201
6782988	2121	8.293023	8.12953	8.259756	7.259454	1.033569	0.024562	0.870076	0.04847	1.000302	0.028185	-0.13023	0.736731	0.163493	0.67363	0.033267	0.931323	6782988	8.565513	8.293833	8.019722	7.897581	8.662815	7.828194	8.779541	7.49755	8.502176	7.32401	7.531423	6.922929	Slfn2	NM_011408
6782989	2122	7.090746	7.096676	7.168784	7.090432	0.000314	0.998396	0.006244	0.968139	0.078352	0.619093	-0.07211	0.646894	-0.00593	0.969742	-0.07804	0.620478	6782989	7.120153	7.162148	6.989937	7.300997	7.106579	6.882452	7.253085	6.892413	7.360853	7.156357	6.907844	7.207094	Slfn1	NM_011407
6783007	2124	6.551671	6.54087	6.406107	6.387315	0.164356	0.344567	0.153556	0.375475	0.018793	0.911393	0.134763	0.434029	0.010801	0.948991	0.145564	0.399635	6783007	6.503816	6.503308	6.647889	6.494557	6.830778	6.297276	6.570968	6.129375	6.517979	6.224574	6.414539	6.522831	Slfn3	NM_011409
6783020	2125	9.972803	9.946282	9.963337	9.596488	0.376315	0.043011	0.349794	0.056021	0.366849	0.047266	-0.01706	0.915976	0.026521	0.86975	0.009466	0.953294	6783020	9.899871	9.825318	10.19322	9.757988	10.22235	9.858507	10.15895	9.747787	9.983274	9.612043	9.672104	9.505317	Ap2b1	NM_001035854
6783029	2127	8.801623	8.872814	8.697706	8.692214	0.109409	0.277702	0.1806	0.090762	0.005493	0.954808	0.175107	0.099311	-0.07119	0.470278	0.103916	0.300789	6783029	8.817171	8.91155	8.676147	8.819342	8.909119	8.88998	8.716961	8.778408	8.59775	8.506934	8.734927	8.83478	Taf15	NM_027427
6783040	2130	8.985704	8.84086	8.956825	7.818406	1.167298	0.02472	1.022454	0.04208	1.138419	0.027473	-0.11597	0.790971	0.144844	0.740924	0.028879	0.947259	6783040	8.958789	8.83489	9.163434	8.651511	9.251762	8.619307	9.536664	8.219825	9.113987	8.313933	8.103129	7.038156	OTTMUSG0	NM_001081957
6783044	2132	8.595762	8.427528	8.258222	8.144614	0.451148	0.003174	0.282913	0.031252	0.113608	0.325665	0.169305	0.157282	0.168235	0.159606	0.33754	0.014424	6783044	8.439843	8.689844	8.6576	8.435311	8.485384	8.361888	8.282473	8.049995	8.442199	8.193532	8.033033	8.207278	1100001G	NM_183249
6783045	2133	8.002473	7.985848	8.009332	7.85907	0.143404	0.194893	0.126779	0.246402	0.150262	0.176542	-0.02348	0.822617	0.016625	0.873797	-0.00686	0.947717	6783045	7.920607	7.997663	8.08915	7.909715	8.180867	7.866963	8.01297	8.015399	7.999627	7.790751	8.041496	7.744962	Heatr6	NM_145432
6783054	2134	6.445947	6.946344	6.693538	6.662453	-0.21651	0.53983	0.283891	0.425462	0.031085	0.929003	0.252806	0.476016	-0.5004	0.177123	-0.24759	0.48487	6783054	5.891186	6.128956	7.317699	6.700865	7.146116	6.992051	6.730547	6.701124	6.648944	6.639543	6.453678	6.894138	Hnf1b	NM_009330
6783056	2135	7.683897	7.760845	7.777912	7.574585	0.109312	0.380185	0.18626	0.152184	0.203327	0.122337	-0.01707	0.888294	-0.07695	0.531591	-0.09402	0.447477	6783056	7.578751	7.702809	7.77013	7.674385	7.918395	7.689755	7.972137	7.613144	7.748454	7.406206	7.688475	7.629073	Ddx52	NM_030096
6783057	2136	8.719893	8.84684	8.571732	8.422662	0.297232	0.081553	0.424179	0.021731	0.14907	0.347089	0.275109	0.102495	-0.12695	0.419685	0.148162	0.349871	6783057	8.449683	8.705801	9.004196	8.640554	9.017178	8.882789	8.710297	8.476776	8.528122	8.44682	8.478949	8.342216	Ap1gbp1	NM_001115009
6783063	2137	9.183389	9.10589	9.418256	9.167792	0.015598	0.864616	-0.0619	0.504509	0.250464	0.022244	-0.31237	0.007777	0.077499	0.407167	-0.23487	0.029201	6783063	9.229338	9.157635	9.163195	8.991694	9.27804	9.047936	9.40418	9.554083	9.296504	9.205951	9.216915	9.080509	Acaca	NM_133360
6783137	2138	7.525161	7.481964	7.499116	7.653242	-0.12808	0.21279	-0.17128	0.107817	-0.15413	0.141931	-0.01715	0.860641	0.043197	0.660097	0.026045	0.790059	6783137	7.554164	7.507693	7.513627	7.515413	7.591827	7.338652	7.362611	7.684782	7.449955	7.547841	7.716187	7.695699	Myo19	BC007156
6783144	2139	6.194647	5.50183	5.806178	6.43469	-0.24004	0.531835	-0.93286	0.034755	-0.62851	0.12549	-0.30435	0.431454	0.692816	0.096044	0.388468	0.321216	6783144	6.378976	6.755242	5.449722	5.667011	5.419999	5.418481	5.813625	6.165422	5.439488	5.935719	6.545343	6.823008	Car4	NM_007607
6783176	2141	7.505474	7.722042	7.585016	7.619507	-0.11403	0.24194	0.102535	0.288752	-0.03449	0.712262	0.137026	0.167387	-0.21657	0.043186	-0.07954	0.403791	6783176	7.49275	7.543639	7.480033	7.620739	7.779636	7.76575	7.77691	7.539548	7.438589	7.513708	7.634681	7.710131	Ppm1d	NM_016910
6783182	2142	8.417204	8.332497	8.31034	8.355073	0.062131	0.501643	-0.02258	0.804659	-0.04473	0.62611	0.022157	0.808202	0.084707	0.365514	0.106864	0.260749	6783182	8.378641	8.4158	8.457171	8.375117	8.451034	8.17134	8.23353	8.259957	8.437533	8.312039	8.27351	8.479671	Bcas3	NM_138681
6783204	2143	7.478384	7.442727	7.451637	7.521371	-0.04299	0.692907	-0.07864	0.475184	-0.06973	0.525161	-0.00891	0.934446	0.035657	0.742837	0.026748	0.805295	6783204	7.464946	7.490581	7.479626	7.533381	7.566434	7.228367	7.458652	7.392436	7.503822	7.328755	7.601997	7.633361	Tbx2	NM_009324
6783225	2144	5.956161	5.858047	5.999848	5.952894	0.003267	0.970878	-0.09485	0.306061	0.046955	0.603058	-0.1418	0.140764	0.098114	0.290807	-0.04369	0.628105	6783225	5.958738	5.862627	6.047118	5.852908	5.790515	5.930718	5.922328	6.175539	5.901678	5.857041	5.961388	6.040252	Tbx4	NM_011536
6783242	2145	8.381902	8.27761	8.230065	8.240194	0.141708	0.348171	0.037416	0.799105	-0.01013	0.944959	0.047546	0.746706	0.104292	0.484239	0.151837	0.31678	6783242	8.317721	8.534321	8.293664	8.311197	8.37806	8.143574	8.443553	8.063344	8.183297	7.978775	8.359561	8.382247	Med13	AK129168
6783255	2146	8.010086	8.01929	8.014215	8.093352	-0.08327	0.30015	-0.07406	0.353321	-0.07914	0.323169	0.005075	0.947826	-0.0092	0.905565	-0.00413	0.957542	6783255	7.919988	8.08509	8.025181	8.0548	8.043215	7.959855	8.090748	7.865055	8.086842	8.006065	8.094275	8.179715	Rnft1	NM_029788
6783259	2147	6.461305	6.431263	6.34943	6.672589	-0.21128	0.13992	-0.24133	0.09816	-0.32316	0.036581	0.081834	0.543362	0.030041	0.82162	0.111875	0.410849	6783259	6.304495	6.486925	6.592494	6.299503	6.623714	6.370573	6.234658	6.535411	6.27822	6.581451	6.847912	6.588404	Tubd1	NM_019756
6783264	2148	6.234264	6.330235	6.190033	6.240657	-0.00639	0.961397	0.089578	0.50399	-0.05062	0.702886	0.140202	0.305375	-0.09597	0.474978	0.044231	0.738658	6783264	6.179137	6.364746	6.158908	6.235873	6.459011	6.295821	6.312275	6.142831	6.114992	6.005202	6.496844	6.219926	Ptrh2	NM_175004
6783296	2149	7.71858	7.79135	7.696991	7.760307	-0.04173	0.838156	0.031044	0.879146	-0.06332	0.757033	0.094359	0.646006	-0.07277	0.722423	0.021589	0.915754	6783296	7.630327	7.955678	7.569735	7.529525	7.895518	7.949008	7.728349	7.445818	7.916807	7.886449	7.430238	7.964233	1110001A	NM_025377
6783297	2150	7.817883	7.924409	7.889601	7.787915	0.029968	0.777503	0.136494	0.21978	0.101686	0.350345	0.034808	0.742978	-0.10653	0.329185	-0.07172	0.504064	6783297	7.765502	7.760152	7.927994	7.842281	8.068011	7.862935	8.104481	7.763492	7.800831	7.72133	7.811477	7.830938	Trim37	NM_197987
6783321	2152	7.529942	7.788322	7.652742	7.314646	0.215296	0.236487	0.473676	0.022645	0.338096	0.079311	0.13558	0.44358	-0.25838	0.163118	-0.1228	0.486239	6783321	7.582051	7.635256	7.372519	7.775565	7.859564	7.729837	7.697384	7.539859	7.720983	6.987598	7.715375	7.240966	4-Sep	NM_011129
6783327	2153	8.37522	8.471321	8.479824	8.560442	-0.18522	0.483237	-0.08912	0.732686	-0.08062	0.757183	-0.0085	0.973905	-0.0961	0.712815	-0.1046	0.688923	6783327	8.334498	8.415436	8.375727	8.392847	8.70034	8.320777	8.194715	9.041323	8.203434	8.636867	8.834858	8.209601	Mtmr4	NM_133215
6783330	2154	7.139397	6.947983	7.011425	7.177322	-0.03792	0.760054	-0.22934	0.092355	-0.1659	0.204176	-0.06344	0.61136	0.191415	0.149337	0.127973	0.317331	6783330	7.012678	7.136561	7.268953	7.015688	7.044483	6.783777	6.864087	7.193845	6.976342	7.216963	7.015194	7.299808	Rnf43	NM_172448
6783337	2155	5.968238	6.000512	6.169622	6.186136	-0.2179	0.05849	-0.18562	0.09708	-0.01651	0.871397	-0.16911	0.125317	-0.03227	0.752302	-0.20138	0.075868	6783337	6.083372	5.852803	5.96854	5.930292	5.912251	6.158994	6.212893	6.236674	6.059299	6.257182	6.035283	6.265944	Bzrap1	NM_172449
6783359	2157	9.652542	9.815198	9.775062	9.695897	-0.04336	0.651029	0.119301	0.232165	0.079165	0.415926	0.040136	0.675105	-0.16266	0.115984	-0.12252	0.220917	6783359	9.650414	9.691629	9.615583	9.635918	9.924282	9.885395	9.888545	9.711442	9.725199	9.577182	9.825153	9.685356	Sfrs1	NM_001078167

6783360	2158	9.026163	8.981206	8.992423	8.939648	0.086515	0.352629	0.041558	0.648145	0.052775	0.563861	-0.01122	0.901356	0.044957	0.621948	0.033741	0.710371	6783360	8.931484	9.061742	9.085264	8.97165	9.06131	8.910659	8.932402	9.173429	8.871437	8.935382	9.031207	8.852355	Vezf1	NM_016686
6783363	2159	7.230792	7.320474	7.197704	7.264337	-0.03354	0.796187	0.056137	0.666793	-0.06663	0.610194	0.122771	0.35701	-0.08968	0.495554	0.033089	0.798886	6783363	7.132002	7.084455	7.47592	7.435807	7.404164	7.121452	7.337473	7.160675	7.094963	7.203829	7.269975	7.319207	Cuedc1	NM_198013
6783367	2160	8.498413	8.569238	8.571882	8.575567	-0.07715	0.468761	-0.00633	0.951791	-0.00368	0.971917	-0.00264	0.979848	-0.07083	0.50487	-0.07347	0.489575	6783367	8.488439	8.440226	8.566573	8.514926	8.617805	8.574984	8.57559	8.762034	8.378022	8.526834	8.47204	8.727826	Mrps23	NM_024174
6783439	2162	7.736419	7.838662	7.82055	7.571446	0.164973	0.058519	0.267216	0.007274	0.249104	0.010388	0.018112	0.814789	-0.10224	0.208898	-0.08413	0.293379	6783439	7.844293	7.655453	7.709511	7.788109	7.973646	7.754231	7.796065	7.861836	7.803748	7.568017	7.66733	7.47899	Trim25	NM_009546
6783484	2164	9.286304	9.759769	8.606911	9.418307	-0.132	0.756693	0.341462	0.430905	-0.8114	0.084219	1.152858	0.023183	-0.47347	0.283322	0.679392	0.137487	6783484	9.199462	9.286265	9.373184	9.737893	9.924531	9.616883	8.59874	9.543481	7.678513	9.248829	9.196163	9.809928	Tmem100	NM_026433
6783489	2165	7.367685	7.601729	7.333486	7.376297	-0.00861	0.959949	0.225432	0.21207	-0.04281	0.803255	0.268243	0.145246	-0.23404	0.19678	0.034199	0.842134	6783489	7.132074	7.561803	7.409177	7.689098	7.726407	7.389683	7.266308	7.382017	7.352133	7.060839	7.611548	7.456504	Mmd	NM_026178
6783500	2166	7.155371	7.19599	7.18389	7.210213	-0.05484	0.647068	-0.01422	0.904874	-0.02632	0.825156	0.012099	0.919014	-0.04062	0.733742	-0.02852	0.810882	6783500	7.21109	7.105007	7.150017	7.117694	7.161976	7.308299	7.286123	7.206375	7.059173	7.05631	7.097508	7.47682	Cox11	NM_199008
6783626	2168	8.259192	8.389559	8.156056	8.26292	-0.00373	0.969788	0.126639	0.221011	-0.10686	0.295167	0.233503	0.040094	-0.13037	0.208964	0.103136	0.311192	6783626	8.257271	8.333041	8.187263	8.282885	8.371679	8.514112	8.238786	7.952303	8.277079	8.242236	8.211075	8.335448	Mbtd1	NM_134012
6783640	2169	8.922873	8.989663	8.774978	8.740614	0.182258	0.079729	0.249049	0.025387	0.034363	0.715064	0.214685	0.045722	-0.06679	0.483186	0.147895	0.142162	6783640	9.007604	8.960667	8.800347	9.014056	9.069562	8.885371	8.85558	8.863844	8.605509	8.661381	8.830325	8.730137	Spag9	NM_027569
6783642	2170	9.692744	9.695141	9.243175	10.10675	-0.41401	0.12749	-0.41161	0.129403	-0.86358	0.007546	0.451966	0.100512	-0.0024	0.992388	0.44957	0.102042	6783642	9.705722	9.674518	9.697993	9.686561	9.518467	9.880395	9.320318	9.568032	8.841174	9.803599	9.916015	10.60064	Tob1	NM_009427
6783654	2171	8.338226	8.36211	8.276319	8.351497	-0.01327	0.92152	0.010613	0.937197	-0.07518	0.580484	0.085791	0.529467	-0.02388	0.859369	0.061907	0.647975	6783654	8.210012	8.31547	8.489197	8.43735	8.395065	8.253915	8.293398	8.400448	8.135111	8.228493	8.203337	8.622662	Ankrd40	NM_027799
6783673	2172	6.357897	7.322012	6.906775	7.328653	-0.97076	0.055145	-0.00664	0.988131	-0.42188	0.358168	0.415236	0.365385	-0.96411	0.056481	-0.54888	0.240331	6783673	6.154164	6.790031	6.129497	8.070274	7.282659	6.613102	7.590925	6.267947	6.861454	7.257162	7.456383	7.272415	Chad	NM_007689
6783674	2173	9.898466	9.809769	10.09355	9.986021	-0.08755	0.484778	-0.17625	0.178572	0.107531	0.394602	-0.28378	0.044956	0.088697	0.479281	-0.19509	0.141305	6783674	9.909257	9.826574	9.959567	9.609466	10.0136	9.806242	10.18809	10.11232	9.980245	9.952958	10.17135	9.833754	Lrrc59	NM_133807
6783685	2175	9.610427	9.240965	8.394201	7.929712	1.680715	0.009617	1.311253	0.029789	0.46449	0.377344	0.846764	0.126828	0.369462	0.478505	1.216226	0.040114	6783685	10.21272	9.757612	8.860949	9.121284	10.04698	8.554631	8.969275	7.998751	8.214578	7.519011	8.370122	7.900002	Col1a1	NM_007742
6783689	2177	7.968923	8.037723	7.845934	7.781011	0.187912	0.268248	0.256712	0.142737	0.064923	0.69182	0.191789	0.259254	-0.0688	0.674637	0.122989	0.458565	6783689	8.143893	7.973449	7.789427	8.019973	8.098155	7.995041	7.975329	8.004128	7.558344	7.555743	8.016335	7.770954	Ppp1r9b	NM_172261
6783694	2178	7.194474	7.038756	7.041899	7.019852	0.174622	0.363735	0.018904	0.919533	0.022047	0.906224	-0.00314	0.986597	0.155718	0.415454	0.152575	0.424537	6783694	7.310362	7.188757	7.084304	7.123704	7.166532	6.826033	6.9484	7.465682	6.711615	7.046354	7.035586	6.977616	Samd14	NM_146025
6783737	2181	8.298928	8.350487	8.230614	8.430873	-0.13195	0.26166	-0.08039	0.482887	-0.20026	0.104166	0.119873	0.304486	-0.05156	0.649592	0.068314	0.549212	6783737	8.268513	8.287704	8.340566	8.360638	8.359171	8.331652	8.228874	8.459176	8.003791	8.362426	8.344258	8.585936	Fam117a	NM_172543
6783740	2182	9.759331	9.758467	9.863308	9.745272	0.014058	0.937048	0.013195	0.940906	0.118035	0.513135	-0.10484	0.560187	0.000864	0.996128	-0.10398	0.563353	6783740	9.939878	9.610389	9.727725	9.636469	9.854227	9.784705	10.03513	10.0684	9.486393	9.81775	9.874887	9.54318	Slc35b1	NM_016752
6783744	2183	9.105906	9.148235	9.032085	9.167673	-0.06177	0.431176	-0.01944	0.800784	-0.13559	0.106312	0.11615	0.15767	-0.04233	0.585575	0.073821	0.350848	6783744	9.091118	9.108563	9.118037	9.154309	9.157302	9.133094	8.866316	9.168187	9.061753	9.066468	9.261052	9.1755	Spop	NM_025287
6783761	2184	7.074248	7.101071	7.067613	7.20006	-0.12581	0.557178	-0.09899	0.642746	-0.13245	0.537068	0.033458	0.874636	-0.02682	0.899318	0.006635	0.975021	6783761	7.22639	6.903567	7.092786	7.274274	7.266127	6.762812	7.087297	6.748487	7.367054	7.270515	6.961614	7.368051	Phb	NM_008831
6783762	2185	8.456607	8.552931	8.422459	8.353966	0.102642	0.258456	0.198965	0.04609	0.068494	0.440392	0.130472	0.160601	-0.09632	0.286621	0.034148	0.696284	6783762	8.450709	8.365366	8.553747	8.638537	8.551497	8.468759	8.388749	8.523106	8.355523	8.243358	8.311503	8.507036	Zfp652	NM_201609
6783784	2186	6.398983	6.443065	5.585046	6.061836	0.337146	0.409837	0.381229	0.354245	-0.47679	0.253703	0.858019	0.057796	-0.04408	0.912273	0.813937	0.068997	6783784	6.209636	5.994488	6.992824	7.080832	6.157948	6.090416	5.893671	5.655939	5.205528	5.564623	6.200896	6.41999	Phospho1	NM_153104
6783810	2189	7.608209	7.657876	7.634631	7.458221	0.149988	0.312818	0.199655	0.189524	0.176409	0.240828	0.023245	0.871561	-0.04967	0.730552	-0.02642	0.854233	6783810	7.542252	7.705066	7.57731	7.542538	7.800014	7.631076	7.623721	7.545166	7.735005	7.126854	7.644578	7.603232	Snf8	NM_033568
6783838	2192	7.706977	7.018216	6.896043	7.497639	0.209338	0.656911	-0.47942	0.321678	-0.6016	0.221608	0.122173	0.794601	0.688761	0.167606	0.810934	0.111796	6783838	7.8729	6.979349	8.268682	6.79885	6.855588	7.40021	6.785452	7.109872	6.792806	8.375238	7.337284	6.780394	Hoxb9	NM_008270
6783840	2193	6.514902	5.982887	5.845197	6.478789	0.036113	0.943589	-0.4959	0.345411	-0.63359	0.236085	0.137691	0.787781	0.532015	0.313466	0.669705	0.212743	6783840	6.146681	5.930841	7.467184	5.889647	5.747645	6.31137	5.505361	6.484144	5.546085	7.193646	6.13716	6.105561	Hoxb8	NM_010461
6783841	2194	6.051477	5.884844	5.69397	6.053522	-0.00205	0.994472	-0.16868	0.571742	-0.35955	0.244299	0.190874	0.523443	0.166634	0.576318	0.357508	0.246767	6783841	5.827538	5.850129	6.476765	5.833965	5.678981	6.141585	5.432809	6.158502	5.490598	6.478881	5.801857	5.879829	Hoxb7	NM_010460
6783842	2195	6.863315	6.774413	6.645351	6.883369	-0.02005	0.929365	-0.10896	0.632565	-0.23802	0.309246	0.129062	0.572306	0.088902	0.695727	0.217964	0.349235	6783842	6.757792	6.618062	7.214092	6.708936	6.841791	6.772512	6.351763	7.032398	6.551892	7.129266	6.89856	6.622282	Hoxb6	NM_008269
6783844	2196	7.048287	6.788923	6.795533	7.020643	0.027644	0.899312	-0.23172	0.305482	-0.22511	0.318587	-0.00661	0.97585	0.259365	0.255285	0.252754	0.266627	6783844	6.875745	6.904565	7.364552	6.680982	6.848941	6.836845	6.414101	7.183963	6.788536	7.233059	6.966937	6.861933	Hoxb5	NM_008268
6783845	2197	6.971714	7.105422	6.837404	7.001406	-0.02969	0.875909	0.104016	0.587687	-0.164	0.399166	0.268018	0.183662	-0.13371	0.488508	0.13431	0.486615	6783845	6.882092	7.139482	6.893567	6.85976	7.415809	7.040696	6.506391	7.121116	6.884705	6.964999	6.950583	7.088636	Hoxb4	NM_010459
6783846	2198	7.583718	7.558446	7.465555	7.521194	0.062525	0.698088	0.037253	0.816652	-0.05564	0.729692	0.092891	0.566699	0.025272	0.8749	0.118163	0.469027	6783846	7.574192	7.656163	7.5208	7.402342	7.757121	7.515876	7.217109	7.804591	7.374965	7.662072	7.465641	7.435868	Hoxb3	NM_001079869
6783847	2199	8.73595	8.652503	8.534757	8.522308	0.213641	0.14383	0.130195	0.352359	0.012449	0.927102	0.117746	0.397924	0.083447	0.54448	0.201192	0.165555	6783847	8.561314	8.908368	8.738167	8.444297	8.829359	8.683853	8.484344	8.563878	8.55605	8.734328	8.44381	8.388787	Hoxb2	NM_134032
6783850	2200	6.843647	6.75675	6.835387	6.656236	0.187411	0.177333	0.100514	0.450448	0.179151	0.195057	-0.07864	0.552071	0.086897	0.512167	0.00826	0.949617	6783850	6.93227	6.59871	6.99996	6.713998	6.625128	6.931124	6.774584	6.936211	6.795365	6.718819	6.503636	6.746252	Hoxb1	NM_008266
6783868	2201	7.655196	7.779272	7.679763	7.614339	0.040857	0.645177	0.164933	0.08956	0.065424	0.465656	0.099509	0.27753	-0.12408	0.184354	-0.02457	0.780927	6783868	7.587005	7.7131	7.665483	7.742011	7.849082	7.746724	7.719823	7.78621	7.533256	7.465027	7.735146	7.642844	Cbx1	NM_007622
6783873	2202	8.516147	8.515702	8.385133	8.706916	-0.19077	0.215099	-0.19121	0.214132	-0.32178	0.052814	0.130569	0.383751	0.000445	0.997573	0.131014	0.382212	6783873	8.519928	8.588794	8.439718	8.500585	8.650805	8.395716	8.330634	8.675703	8.149061	8.659905	8.571554	8.889289	Copz2	NM_019877
6783879	2203	9.285989	9.458485	9.034888	9.324914	-0.03893	0.826627	0.13357	0.459732	-0.29003	0.130226	0.423597	0.039142	-0.1725	0.345261	0.251101	0.182425	6783879	9.132335	9.085147	9.640485	9.634131	9.372508	9.368815	9.307807	8.872217	8.92464	9.381776	9.272792	9.320175	Atad4	NM_146026
6783883	2205	6.63321	6.795396	6.694112	6.597758	0.035452	0.707038	0.197638	0.061663	0.096354	0.320642	0.101284	0.298076	-0.16219	0.112594	-0.0609	0.522219	6783883	6.557876	6.649288	6.692467	6.761282	6.803295	6.821611	6.635337	6.89138	6.55562	6.590596	6.486429	6.716249	Scrn2	NM_146027
6783884	2206	8.191151	8.156862	8.13667	8.235214	-0.04406	0.747267	-0.07835	0.569447	-0.09854	0.476989	0.020192	0.882291	0.034289	0.801733	0.054481	0.690836	6783884	8.138436	8.238411	8.196607	8.251894	8.248548	7.970144	8.133259	8.345863	7.930889	8.057023	8.228709	8.419911	Mrpl10	NM_026154
6783886	2207	8.119155	8.117878	8.079264	8.117256	0.001899	0.97237	0.000622	0.99095	-0.03799	0.495072	0.038614	0.488253	0.001277	0.981416	0.039891	0.474439	6783886	8.100541	8.165741	8.091184	8.115348	8.223195	8.015091	8.068473	8.136666	8.032654	8.1597	8.114099	8.077969	Osbpl7	NM_001081434
6783909	2208	7.162722	7.275528	7.073819	7.332045	-0.16932	0.280467	-0.05652	0.709317	-0.25823	0.115527	0.20171	0.205258	-0.11281	0.462793	0.088903	0.560215	6783909	6.895691	7.263873	7.328602	7.152791	7.405387	7.268407	7.201632	7.220092	6.799732	7.371791	7.295741	7.328602	Mrpl45	NM_025927
6783913	2209	7.358396	7.389368	7.265582	7.242411	0.115985	0.315232	0.146956	0.211664	0.023171	0.835868	0.123786	0.285897	-0.03097	0.782068	0.092814	0.416175	6783913	7.407454	7.299998	7.367737	7.470555	7.419274	7.278274	7.25385	7.487229	7.055667	7.157266	7.211883	7.358085	Socs7	NM_138657
6783914	2210	7.023916	7.22461	6.953095	6.96335	0.060565	0.379658	0.26126	0.003889	-0.01026	0.87879	0.271516	0.003129	-0.20069	0.01509	0.070821	0.308579	6783914	7.073208	7.049689	6.94885	7.22798	7.352837	7.093014	7.027529	6.919814	6.911941	6.957951	6.962441	6.969659	4933428G	NM_021493
6783917	2211	7.805618	7.763202	7.80333	7.812137	-0.00652	0.971115	-0.04893	0.786275	-0.00881	0.960988	-0.04013	0.823904	0.042416	0.814078	0.002288	0.989862	6783917	7.800042	7.894155	7.722657	7.902382	7.754934	7.632291	7.632636	8.245961	7.531394	7.4773	7.887981	7.831131	Mllt6	NM_139311
6783945	2212	10.37185	10.24533	10.28294	9.879569	0.492281	0.036382	0.365763	0.099201	0.403368	0.07376	-0.03761	0.852735	0.126519	0.536961	0.088913	0.66237	6783945	10.47749	10.31698	10.32108	10.28861	10.52299	9.924394	10.21497	10.60464	10.0292	9.718843	10.12072	9.799143	Lasp1	NM_010688
6783989	2213	8.242108	8.147111	8.05842	8.208072	0.034037	0.76981	-0.06096	0.602441	-0.14965	0.219846	0.088691	0.452934	0.094997	0.422683	0.183688	0.140938	6783989	8.211501	8.316045	8.198779	8.090406	8.240827	8.1101	7.838295	8.328005	8.008961	8.235768	8.245728	8.142719	Crkrs	NM_001109626
6783997	2214	9.217955	9.805966	9.673275	10.63159	-1.41363	0.007904	-0.82562	0.074173	-0.95831	0.044349	0.132691	0.749923	-0.58801	0.18186	-0.45532	0.290364	6783997	9.024181	9.53047	9.099214	10.17325	9.414084	9.830565	9.37431	10.42215	9.223365	10.27916	10.32634	11.28927	Ppp1r1b	NM_144828
6783998	2215	7.733071	7.73723	7.563129	7.547652	0.185418	0.055793	0.189578	0.051592	0.015477	0.856603	0.174101	0.069012	-0.00416	0.96123	0.169941	0.074607	6783998	7.728992	7.687694	7.782526	7.6882	7.94649	7.577	7.578567	7.566286	7.544535	7.486922	7.574952	7.581083	Stard3	NM_021547
6784003	2216	7.026692	6.757059	6.509293	6.49269	0.534002	0.558287	0.264369	0.770071	0.016603	0.985314	0.247767	0.784069	0.269633	0.765651	0.5174	0.570325	6784003	6.045047	6.17939	8.85564	7.929534	6.568853	5.772791	7.10564	6.367381	6.054857	5.646927	6.679355	7.151788	Tcap	NM_011540
6784005	2218	8.79233	8.935847	8.840505	8.852594	-0.06026	0.510754	0.083253	0.369542	-0.01209	0.893604	0.095341	0.307925	-0.14352	0.139834	-0.04817	0.59721	6784005	8.677116	8.860023	8.839852	8.84746	9.08073	8.87935	8.780451	8.936547	8.804518	8.726102	8.945553	8.886127	Erbb2	NM_001003817
6784008	2219	8.155113	8.189763	8.138903	8.00353	0.151583	0.172452	0.186233	0.102933	0.135372	0.217676	0.050861	0.628717	-0.03465	0.740813	0.01621	0.876678	6784008	7.987895	8.202599	8.274845	8.157661	8.318077	8.093552	8.240892	8.017277	8.158539	7.974646	7.906844	8.129101	Grb7	NM_010346
6784039	2224	8.53872	8.640169	8.488673	8.372052	0.166668	0.133425	0.268117	0.027631	0.116621	0.276173	0.151496	0.16745	-0.10145	0.339078	0.050046	0.62951	6784039	8.525331	8.486835	8.603993	8.695823	8.763439	8.461246	8.613926	8.427792	8.424302	8.227904	8.504739	8.383513	Psmd3	NM_009439
6784042	2226	8.348921	8.430711	8.363182	8.363143	-0.01422	0.925848	0.067568	0.660317	3.87E-05	0.999798	0.067529	0.660497	-0.08179	0.595813	-0.01426	0.925648	6784042	8.2507	8.374804	8.421259	8.590941	8.354777	8.346415	8.354031	8.621703	8.113812	8.150301	8.392138	8.546991	Thra	NM_178060
6784048	2227	8.881253	8.988325	8.745294	8.779801	0.101453	0.339692	0.208525	0.070369	-0.03451	0.738754	0.243032	0.041067	-0.10707	0.315197	0.13596	0.210732	6784048	8.888664	8.860179	8.894917	8.956591	9.006394	9.001991	8.664138	9.005074	8.566669	8.700826	8.785507	8.853069	Msl1	NM_028722
6784049	2228	7.752702	7.754649	7.781472	7.739949	0.012753	0.900413	0.0147	0.885323	0.041523	0.685134	-0.02682	0.792745	-0.00195	0.984747	-0.02877	0.778152	6784049	7.787582	7.713382	7.757141	7.755664	7.919337	7.588946	7.745965	7.962578	7.635873	7.756371	7.776132	7.687344	Casc3	NM_138660
6784052	2229	8.582512	8.620135	8.609738	8.641618	-0.05911	0.755037	-0.02148	0.909463	-0.03188	0.86606	0.010397	0.956095	-0.03762	0.842276	-0.02723	0.885434	6784052	8.525225	8.635414	8.586897	8.666482	8.656321	8.537603	8.330742	9.114594	8.383879	8.618164	8.629821	8.676868	Wipf2	NM_197940
6784056	2231	8.015086	7.984198	7.869424	7.666542	0.348544	0.098347	0.317656	0.12666	0.202882	0.307981	0.114774	0.555068	0.030888	0.872462	0.145662	0.456919	6784056	8.188673	7.988015	7.86857	8.068884	8.25647	7.62724	7.764693	8.157625	7.685955	7.540251	7.774616	7.68476	Rara	NM_009024
6784062	2232	9.349087	9.264331	8.643298	8.354621	0.994466	0.020554	0.90971	0.030023	0.288678	0.427613	0.621032	0.10994	0.084756	0.812368	0.705788	0.075318	6784062	9.747602	9.302437	8.997221	9.154801	9.712662	8.925529	9.088542	8.178515	8.662838	7.866512	8.752804	8.444546	Igfbp4	NM_010517
6784177	2234	11.62084	11.5558	11.50612	11.64985	-0.02901	0.833826	-0.09405	0.502154	-0.14373	0.314177	0.04968	0.720132	0.065043	0.640012	0.114723	0.416287	6784177	11.59973	11.49519	11.7676	11.68137	11.61809	11.36793	11.34633	11.7736	11.39842	11.61938	11.58708	11.74309	Eif1	NM_011508
6784178	2235	7.306134	7.292396	7.308395	7.324343	-0.01821	0.837092	-0.03195	0.719072	-0.01595	0.857046	-0.016	0.856592	0.013738	0.876654	-0.00226	0.979598	6784178	7.294649	7.316885	7.306867	7.321718	7.243362	7.312108	7.140065	7.301734	7.483387	7.434348	7.329054	7.209628	Gast	NM_010257
6784187	2236	8.281992	8.36026	8.045626	8.407003	-0.12501	0.525751	-0.04674	0.810354	-0.36138	0.091452	0.314634	0.133554	-0.07827	0.688823	0.236366	0.245156	6784187	8.403679	8.382278	8.060019	8.342779	8.726043	8.011958	8.294047	7.926855	7.915976	8.362036	8.407651	8.451322	Fkbp10	NM_010221
6784205	2239	8.336165	8.160641	8.213183	7.675338	0.660826	0.003128	0.485302	0.015548	0.537845	0.009459	-0.05254	0.748815	0.175524	0.300357	0.122981	0.460179	6784205	8.278967	8.210912	8.518615	8.129614	8.334881	8.017427	8.468466	7.915298	8.255786	7.721439	7.791131	7.513445	Cnp	NM_009923
6784207	2240	7.750751	7.774307	7.736663	7.571042	0.17971	0.171522	0.203265	0.127791	0.165621	0.203695	0.037644	0.76111	-0.02356	0.848844	0.014088	0.909175	6784207	7.685355	7.823019	7.74388	7.655364	7.95778	7.709777	7.909169	7.714196	7.586624	7.421101	7.536951	7.755073	Nkiras2	NM_028024

6784218	2241	7.856103	7.757004	7.874027	7.728907	0.127196	0.072012	0.028097	0.659355	0.14512	0.045678	-0.11702	0.09307	0.099099	0.145136	-0.01792	0.77773	6784218	7.891072	7.92425	7.752986	7.851616	7.763197	7.656199	7.88024	7.893022	7.84882	7.710997	7.801333	7.674391	Stat5a	NM_011488
6784236	2242	9.843069	9.83552	9.884769	9.986321	-0.14325	0.410547	-0.1508	0.387433	-0.10155	0.555324	-0.04925	0.772929	0.007549	0.964627	-0.0417	0.806839	6784236	9.892204	9.71867	9.918334	9.809329	10.03579	9.661442	9.830048	10.03416	9.7901	9.739671	9.878911	10.34038	Atp6v0a1	NM_016920
6784237	2243	8.81833	8.799378	8.524566	8.426143	0.392186	0.037286	0.373235	0.044997	0.098422	0.548815	0.274813	0.118659	0.018951	0.907044	0.293764	0.098687	6784237	9.065825	8.673523	8.715641	8.666172	9.041924	8.690039	8.711936	8.459474	8.402287	8.352721	8.299994	8.625715	Naglu	NM_013792
6784239	2245	6.701674	6.859315	6.71579	6.770518	-0.06884	0.528369	0.088797	0.420001	-0.05473	0.614535	0.143525	0.20671	-0.15764	0.169705	-0.01412	0.895842	6784239	6.740209	6.734515	6.630297	6.924479	6.902036	6.751431	6.852532	6.622866	6.671972	6.551459	6.833472	6.926624	Coasy	NM_027896
6784241	2246	8.002676	8.087678	8.145776	8.057638	-0.05496	0.490775	0.03004	0.703381	0.088139	0.280247	-0.0581	0.467168	-0.085	0.296482	-0.1431	0.096878	6784241	8.020011	7.937271	8.050745	8.074049	8.201376	7.987609	8.241812	8.18427	8.011247	7.985716	8.131907	8.05529	Mlx	NM_011550
6784244	2247	9.578892	9.354233	9.865134	9.356348	0.222544	0.30871	-0.00212	0.992011	0.508786	0.037812	-0.5109	0.037208	0.224659	0.304435	-0.28624	0.199629	6784244	9.940682	9.476667	9.319326	9.297242	9.63604	9.129416	9.863939	10.0878	9.643663	9.215456	9.298969	9.554618	Tubg1	NM_134024
6784245	2248	6.876794	6.750772	6.944484	6.721001	0.155793	0.303385	0.02977	0.838788	0.223483	0.153278	-0.19371	0.20863	0.126022	0.399595	-0.06769	0.645523	6784245	7.095628	6.872028	6.662726	6.803705	6.927018	6.521592	7.030255	6.992235	6.810962	6.576594	6.762392	6.824018	Tubg2	NM_134028
6784251	2250	7.544027	7.608159	7.308633	7.793739	-0.24971	0.144526	-0.18558	0.263821	-0.48511	0.013776	0.299526	0.088395	-0.06413	0.688836	0.235394	0.165929	6784251	7.526433	7.505727	7.59992	7.842024	7.774401	7.208052	7.442616	7.16534	7.317942	7.792226	7.806085	7.782906	Ramp2	NM_019444
6784253	2251	10.34219	10.33662	10.33391	10.46059	-0.1184	0.303633	-0.12397	0.282982	-0.12668	0.273365	0.002707	0.980567	0.00557	0.960025	0.008277	0.940636	6784253	10.29601	10.26315	10.4674	10.30186	10.44879	10.2592	10.36733	10.43801	10.19639	10.38779	10.32882	10.66515	Vps25	NM_026776
6784257	2254	9.257142	9.096616	9.281488	9.180111	0.077031	0.61753	-0.0835	0.58886	0.101377	0.513549	-0.18487	0.247813	0.160526	0.31061	-0.02435	0.873676	6784257	9.273389	9.184569	9.313469	8.988439	9.417652	8.883757	9.288841	9.468304	9.087319	9.07063	9.187352	9.282351	Psme3	NM_011192
6784260	2256	6.956567	6.712156	6.582243	6.661042	0.295525	0.261854	0.051114	0.839831	-0.0788	0.755797	0.129913	0.610062	0.244411	0.347342	0.374324	0.164785	6784260	7.018763	6.827425	7.023512	6.908241	6.885816	6.342411	6.533114	6.717917	6.495697	6.162787	7.120117	6.700221	Aoc3	NM_009675
6784266	2258	7.864804	7.771136	7.868205	8.086349	-0.22154	0.02363	-0.31521	0.004137	-0.21814	0.025244	-0.09707	0.256639	0.093669	0.272354	-0.0034	0.96691	6784266	7.836588	7.888589	7.869236	7.759845	7.770997	7.782565	7.91687	7.972529	7.715217	7.933067	8.129587	8.196392	Rundc1	NM_172566
6784270	2259	7.661367	7.735129	7.6535	7.431582	0.229785	0.094244	0.303547	0.036519	0.221918	0.104139	0.081629	0.519139	-0.07376	0.559231	0.007867	0.949781	6784270	7.613378	7.627337	7.743387	7.811549	7.870318	7.52352	7.879133	7.536782	7.544586	7.395452	7.545648	7.353647	Ifi35	NM_027320
6784272	2260	6.482904	6.768682	6.371928	7.185435	-0.70253	0.084086	-0.41675	0.275745	-0.81351	0.051789	0.396754	0.297757	-0.28578	0.445628	0.110976	0.763379	6784272	6.444514	6.451231	6.552968	6.778094	7.059087	6.468866	6.41498	6.597908	6.102897	7.294436	6.356918	7.904952	Rnd2	NM_009708
6784279	2261	9.101985	9.129923	9.179526	9.181978	-0.07999	0.251575	-0.05205	0.444512	-0.00245	0.970706	-0.0496	0.46549	-0.02794	0.677412	-0.07754	0.265221	6784279	8.958334	9.146866	9.200756	9.045074	9.190206	9.15449	9.187731	9.182956	9.167891	9.121083	9.194304	9.230548	Nbr1	NM_008676
6784280	2262	8.66894	8.239772	8.570124	7.972255	0.696685	0.035495	0.267518	0.360543	0.597869	0.062068	-0.33035	0.265349	0.429167	0.158359	0.098816	0.72944	6784280	8.827392	8.598655	8.580772	8.316611	8.672919	7.729787	8.787226	8.234025	8.689121	7.871733	8.363282	7.681749	Tmem106a	NM_144830
6784283	2263	7.546714	7.691594	7.807341	7.917051	-0.37034	0.038749	-0.22546	0.171164	-0.10971	0.485326	-0.11575	0.462429	-0.14488	0.362313	-0.26063	0.120441	6784283	7.146412	7.791684	7.702045	7.721367	7.700413	7.653003	7.745233	7.799908	7.876882	8.013175	7.884165	7.853814	Rdm1	NM_025654
6784290	2264	9.559732	9.935465	9.548924	10.13584	-0.57611	0.13573	-0.20038	0.579831	-0.58692	0.129503	0.386541	0.298029	-0.37573	0.310834	0.010808	0.975937	6784290	9.112282	9.679403	9.887511	10.49808	9.348995	9.95932	9.672994	9.28576	9.688019	10.45578	9.654309	10.29744	Arl4d	NM_025404
6784291	2265	7.789642	7.91257	7.7072	7.744166	0.045476	0.604578	0.168404	0.08103	-0.03697	0.672889	0.20537	0.040945	-0.12293	0.183238	0.082442	0.357142	6784291	7.781093	7.878789	7.709044	7.847901	7.994569	7.895241	7.809988	7.792717	7.518895	7.736398	7.804084	7.692017	Dhx8	NM_144831
6784305	2266	9.472559	9.572117	9.335582	9.805779	-0.33322	0.069246	-0.23366	0.179614	-0.4702	0.018168	0.236535	0.174904	-0.09956	0.548397	0.136977	0.41374	6784305	9.587489	9.56737	9.262819	9.838422	9.407564	9.470366	9.450844	9.119909	9.435993	9.716654	10.00303	9.697652	Cd300lg	NM_027987
6784313	2267	6.103104	6.112843	6.035996	6.202816	-0.09971	0.263698	-0.08997	0.309659	-0.16682	0.079138	0.076847	0.381314	-0.00974	0.909435	0.067109	0.441886	6784313	6.113836	6.029867	6.16561	6.147071	5.957048	6.234409	6.112358	6.089425	5.906204	6.183143	6.154151	6.271155	Nags	NM_145829
6784317	2268	9.792485	9.625138	9.814245	10.03884	-0.24635	0.011038	-0.4137	0.000558	-0.22459	0.017111	-0.18911	0.035546	0.167348	0.055927	-0.02176	0.778812	6784317	9.802674	9.718464	9.856318	9.600294	9.715828	9.559291	9.944767	9.718994	9.778975	10.04257	9.944468	10.12948	G6pc3	NM_175935
6784330	2270	6.095651	5.847109	5.785218	5.85798	0.237671	0.426808	-0.01087	0.970393	-0.07276	0.804194	0.061891	0.832888	0.248542	0.40685	0.310433	0.30603	6784330	5.68976	5.875049	6.722145	6.103504	5.752043	5.68578	5.962249	6.015	5.378405	5.860369	5.783054	5.930518	Asb16	NM_148953
6784331	2271	8.029937	8.162631	8.104573	7.989334	0.040603	0.650588	0.173297	0.079532	0.115239	0.218541	0.058058	0.520092	-0.13269	0.162732	-0.07464	0.412337	6784331	8.020999	8.067105	8.001707	8.158619	8.241085	8.088189	8.233211	7.893404	8.187104	7.933582	8.054626	7.979793	Tmub2	NM_028076
6784343	2272	6.678347	6.729127	6.614004	7.111281	-0.43293	0.024365	-0.38215	0.040343	-0.49728	0.013005	0.115123	0.482693	-0.05078	0.753747	0.064344	0.691569	6784343	6.592704	6.791718	6.65062	6.687891	6.628023	6.871466	6.493423	6.636974	6.711614	7.053269	6.815685	7.46489	Rundc3a	NM_016759
6784345	2273	11.48546	11.13125	11.20063	11.17209	0.31337	0.058489	-0.04083	0.78112	0.028543	0.845799	-0.06938	0.638457	0.354203	0.037353	0.284827	0.079942	6784345	11.5927	11.4236	11.44007	11.13044	11.36304	10.90028	11.14316	11.00124	11.45749	11.09697	11.22183	11.19746	Grn	NM_008175
6784348	2274	6.099874	6.266676	5.773237	6.17784	-0.07797	0.665524	0.088836	0.622948	-0.4046	0.048257	0.493439	0.021811	-0.1668	0.365146	0.326637	0.096906	6784348	6.257164	6.21678	5.825679	5.996374	6.637464	6.16619	5.819304	5.811314	5.689094	6.098129	6.280244	6.155148	Fzd2	NM_020510
6784363	2275	5.925071	5.89216	5.935427	5.981283	-0.05621	0.548954	-0.08912	0.350247	-0.04586	0.623546	-0.04327	0.642991	0.032911	0.723621	-0.01036	0.911071	6784363	6.037726	5.863576	5.873911	5.976789	5.802478	5.897213	6.062216	5.999774	5.744291	6.010373	5.919211	6.014264	Adam11	NM_001110778
6784368	2276	6.29949	6.283675	6.261041	6.398417	-0.09893	0.433148	-0.11474	0.366506	-0.13738	0.284919	0.022634	0.85494	0.015815	0.898299	0.038449	0.756623	6784368	6.211299	6.435506	6.251665	6.442351	6.269225	6.139449	6.073322	6.431699	6.278101	6.274171	6.530825	6.390256	Higd1b	NM_080846
6784396	2278	10.41784	10.42679	10.51433	10.35376	0.064073	0.523803	0.073027	0.469221	0.16057	0.13338	-0.08754	0.389054	-0.00895	0.92808	-0.0965	0.344834	6784396	10.48389	10.32219	10.44743	10.33349	10.57423	10.37265	10.65788	10.35236	10.53276	10.25078	10.40902	10.40149	Nmt1	NM_008707
6784399	2279	6.933183	7.09542	7.032577	7.204963	-0.27178	0.051765	-0.10954	0.384232	-0.17239	0.185493	0.062843	0.611779	-0.16224	0.209921	-0.09939	0.427836	6784399	6.986175	6.894724	6.918651	7.099928	7.17007	7.016263	7.086646	7.084681	6.926405	7.105926	7.007536	7.501428	Acbd4	NM_025988
6784400	2280	7.824558	7.997378	7.631745	7.957002	-0.13244	0.357572	0.040376	0.773597	-0.32526	0.04336	0.365633	0.02729	-0.17282	0.238508	0.192813	0.193068	6784400	8.00881	7.742585	7.722279	7.925814	8.081072	7.985248	7.488075	7.952005	7.455154	7.92398	8.000621	7.946404	Hexim1	NM_138753
6784412	2281	7.311005	7.055779	7.079596	6.439154	0.87185	0.003503	0.616624	0.020194	0.640441	0.017025	-0.02382	0.913865	0.255226	0.26584	0.231409	0.309684	6784412	7.633802	7.241756	7.057456	7.110782	7.295592	6.760962	7.364711	6.939348	6.934728	6.286122	6.700627	6.330714	Fmnl1	NM_019679
6784485	2283	8.234407	8.05714	8.091789	7.857486	0.376921	0.124106	0.199654	0.389385	0.234303	0.316686	-0.03465	0.878421	0.177267	0.442442	0.142618	0.533858	6784485	8.500764	8.064412	8.138045	8.006982	8.374496	7.789943	8.392716	8.144074	7.738577	7.968734	7.970959	7.632766	Arf2	NM_007477
6784494	2284	7.296596	7.104761	6.934626	6.452687	0.843909	0.021625	0.652074	0.05911	0.481939	0.142804	0.170135	0.581948	0.191835	0.535841	0.36197	0.257015	6784494	7.842739	6.90459	7.142458	6.755424	7.454712	7.104146	7.225372	6.706931	6.871574	6.35221	6.802695	6.203156	Crhr1	NM_007762
6784527	2288	6.998577	7.084832	6.88516	7.12017	-0.12159	0.135679	-0.03534	0.642598	-0.23501	0.012487	0.199672	0.026068	-0.08626	0.273052	0.113417	0.16032	6784527	7.053772	7.034071	6.907889	7.048689	7.167518	7.03829	6.848909	6.987989	6.818583	7.249075	7.069295	7.04214	Itgb3	NM_016780
6784553	2290	7.212561	7.322371	7.280705	7.202726	0.009835	0.937943	0.119645	0.357056	0.077979	0.541955	0.041666	0.74237	-0.10981	0.39594	-0.06814	0.593021	6784553	7.212032	7.377258	7.048394	7.176597	7.54839	7.242126	7.401509	7.162877	7.277729	7.116626	7.306623	7.184929	Mettl2	NM_172567
6784558	2291	7.868026	8.059705	7.925127	7.975863	-0.10784	0.197619	0.083842	0.30645	-0.05074	0.527139	0.134578	0.117605	-0.19168	0.037086	-0.0571	0.478156	6784558	7.83674	7.877858	7.889479	8.000505	8.10915	8.06946	8.02828	7.938145	7.808955	7.8891	7.901972	8.136518	Tlk2	NM_001112705
6784564	2292	7.076762	7.002369	6.512683	6.522752	0.55401	0.021202	0.479617	0.038434	-0.01007	0.959843	0.489686	0.035445	0.074393	0.711101	0.564079	0.019577	6784564	7.212208	7.204237	6.813841	6.963427	7.334354	6.709325	6.716474	6.320438	6.501137	6.314897	6.681538	6.57182	Mrc2	NM_008626
6784578	2293	7.314958	7.253461	7.116844	7.541917	-0.22696	0.083487	-0.28846	0.036248	-0.42507	0.006025	0.136617	0.268233	0.061496	0.606817	0.198114	0.12274	6784578	7.295433	7.206313	7.443127	7.408795	7.261721	7.089868	7.069317	7.311017	6.970198	7.517866	7.644236	7.463648	Tanc2	NM_181071
6784587	2294	9.16141	8.8448	8.311567	8.028863	1.132547	0.001517	0.815937	0.009431	0.282703	0.273306	0.533234	0.057303	0.31661	0.224205	0.849843	0.007665	6784587	9.425523	8.972265	9.086442	8.902454	9.157028	8.474919	8.471641	8.241747	8.221312	7.581862	8.307305	8.197423	Ace	NM_207624
6784590	2295	8.970204	8.962068	8.799415	8.830052	0.140152	0.111771	0.132016	0.130845	-0.03064	0.706283	0.162653	0.071808	0.008136	0.91994	0.170789	0.061102	6784590	8.905378	9.013872	8.991361	8.884164	9.129187	8.872853	8.913327	8.780638	8.704279	8.809628	8.874203	8.806324	Wdr68	NM_027946
6784591	2296	7.371418	7.448061	7.299045	7.491297	-0.11988	0.475228	-0.04324	0.793859	-0.19225	0.263958	0.149016	0.378992	-0.07664	0.644795	0.072373	0.663092	6784591	7.181148	7.33569	7.597417	7.514527	7.433977	7.39568	7.31482	7.589533	6.992783	7.370741	7.476904	7.626247	Ccdc44	NM_027346
6784594	2297	9.426777	9.446821	9.397554	9.341411	0.085365	0.406892	0.10541	0.311247	0.056143	0.580644	0.049267	0.627044	-0.02004	0.842284	0.029222	0.772079	6784594	9.419806	9.379446	9.481078	9.32362	9.614235	9.402608	9.515281	9.454254	9.223128	9.34321	9.43057	9.250454	Map3k3	NM_011947
6784605	2298	8.242635	8.369498	8.276069	8.187474	0.055161	0.461195	0.182024	0.03396	0.088595	0.249022	0.093429	0.226254	-0.12686	0.112939	-0.03343	0.651501	6784605	8.287605	8.218991	8.22131	8.256319	8.492575	8.3596	8.387855	8.2321	8.208252	8.103436	8.218618	8.240369	Ddx42	NM_028074
6784606	2299	7.976942	8.047983	8.061199	7.87858	0.098362	0.396448	0.169403	0.161403	0.182618	0.134806	-0.01322	0.907157	-0.07104	0.535701	-0.08426	0.464857	6784606	8.049857	7.848679	8.03229	7.979192	8.05528	8.109478	8.311788	7.986809	7.884999	7.79292	7.942133	7.900688	Psmc5	NM_008950
6784659	2301	6.733284	6.903056	6.772369	6.735835	-0.00255	0.971883	0.167221	0.044246	0.036534	0.61653	0.130687	0.099409	-0.16977	0.041805	-0.03908	0.592579	6784659	6.611837	6.818454	6.769562	6.843151	6.945146	6.920871	6.751994	6.734509	6.830603	6.624887	6.735077	6.84754	Ccdc45	NM_177088
6784709	2302	9.480795	9.449862	9.549157	9.384041	0.096754	0.430257	0.065821	0.587506	0.165115	0.194069	-0.09929	0.41874	0.030933	0.797285	-0.06836	0.573456	6784709	9.444609	9.400656	9.597121	9.330796	9.642066	9.376724	9.744713	9.483602	9.419155	9.309609	9.51433	9.328185	Psmd12	NM_025894
6784714	2303	9.153014	9.20079	9.075548	9.461923	-0.30891	0.036945	-0.26113	0.067525	-0.38638	0.014082	0.125243	0.340458	-0.04778	0.709099	0.077466	0.548179	6784714	9.121346	9.171301	9.166395	9.107369	9.307588	9.187414	9.026819	9.178137	9.021687	9.770042	9.267942	9.347785	Helz	NM_198298
6784761	2305	6.704976	6.599343	6.348389	6.345543	0.359433	0.056402	0.2538	0.154184	0.002846	0.986352	0.250954	0.158287	0.105633	0.530836	0.356587	0.057977	6784761	6.539906	6.858402	6.71662	6.297595	6.839486	6.660948	6.518952	6.220569	6.305645	6.338207	6.524297	6.174124	Ccdc46	NM_029606
6784765	2306	6.419835	6.056339	6.080093	5.928899	0.490935	0.00542	0.127439	0.35571	0.151194	0.278392	-0.02375	0.859576	0.363496	0.02335	0.339741	0.030983	6784765	6.577919	6.461937	6.219648	6.033265	6.299088	5.836663	6.192968	5.995887	6.051425	5.896877	6.000174	5.889647	Axin2	NM_015732
6784783	2307	9.848241	9.861114	9.671175	9.819799	0.028442	0.782487	0.041315	0.689205	-0.14862	0.174019	0.189939	0.092979	-0.01287	0.900358	0.177066	0.11336	6784783	9.791468	9.830412	9.922842	9.73379	9.996183	9.853369	9.595473	9.764275	9.653777	9.936709	9.903183	9.619505	Gna13	NM_010303
6784785	2309	8.220718	8.149456	8.086342	8.315559	-0.09484	0.551614	-0.1661	0.308136	-0.22922	0.171524	0.063114	0.690075	0.071263	0.653	0.134376	0.404277	6784785	7.880941	8.275343	8.505871	7.970861	8.228423	8.249083	8.126629	8.157312	7.975085	8.400681	8.243	8.302995	Amz2	NM_025275
6784787	2310	6.373641	6.516249	6.220967	6.449699	-0.07606	0.50215	0.066551	0.555703	-0.22873	0.067517	0.295282	0.025915	-0.14261	0.224105	0.152674	0.19603	6784787	6.382319	6.352499	6.386105	6.470568	6.424134	6.654046	6.462973	6.06247	6.137459	6.336565	6.490992	6.521539	Arsg	NM_028710
6784810	2311	10.53355	10.65147	10.58812	10.6989	-0.16535	0.124233	-0.04743	0.635525	-0.11078	0.283111	0.063353	0.528994	-0.11792	0.255489	-0.05457	0.586418	6784810	10.5143	10.54195	10.54441	10.58108	10.72814	10.6452	10.72338	10.58764	10.45334	10.51929	10.70225	10.87516	Prkar1a	NM_021880
6784844	2313	10.06872	9.920136	10.0582	10.53312	-0.4644	0.160395	-0.61299	0.075406	-0.47493	0.152237	-0.13806	0.657776	0.148589	0.633873	0.010528	0.972879	6784844	9.787944	10.3484	10.06983	10.08126	9.620037	10.05911	9.374301	10.47829	10.322	10.34396	10.5247	10.73071	Kcnj16	NM_010604
6784845	2314	6.712636	7.638379	7.903456	7.520136	-0.8075	0.114126	0.118243	0.801689	0.38332	0.424378	-0.26508	0.576538	-0.92574	0.076517	-1.19082	0.030892	6784845	6.595456	6.679318	6.863135	7.372468	7.550171	7.992497	7.329851	8.231912	8.148605	6.508328	7.698528	8.353552	Kcnj2	NM_008425
6784940	2315	10.3751	9.853711	10.08106	9.782415	0.592682	0.090196	0.071296	0.822546	0.298645	0.36009	-0.22735	0.481011	0.521386	0.128556	0.294036	0.367161	6784940	10.45986	10.20508	10.46035	9.868	10.20091	9.492223	9.909116	10.73069	9.603375	10.03875	9.831105	9.47739	Sox9	NM_011448
6784990	2317	8.573805	8.630465	8.712037	8.577439	-0.00363	0.94031	0.053026	0.292155	0.134598	0.021077	-0.08157	0.12102	-0.05666	0.262672	-0.13823	0.018722	6784990	8.574157	8.604138	8.543121	8.70593	8.648635	8.53683	8.710203	8.773184	8.652723	8.540315	8.579367	8.612634	Cog1	NM_013581
6784997	2318	7.009548	7.174073	7.012143	6.947997	0.061551	0.57881	0.226076	0.066304	0.064146	0.563243	0.16193	0.166485	-0.16453	0.160575	-0.0026	0.981137	6784997	7.025465	6.983948	7.019231	7.150947	7.376385	6.994887	7.157802	7.025109	6.853518	7.000916	6.993983	6.849093	D11Wsu47	BC048942
6785079	2320	7.626777	7.617406	7.633363	7.149186	0.477591	0.0423	0.46822	0.045544	0.484177	0.04016	-0.01596	0.937722	0.009371	0.963395	-0.00659	0.974271	6785079	7.752419	7.598402	7.529511	7.522089	7.94635	7.38378	7.697013	7.41387	7.789205	6.929745	7.507261	7.010552	Ttyh2	NM_053273

6785104	2322	8.463666	8.428432	8.405744	8.427149	0.036517	0.794312	0.001283	0.992675	-0.0214	0.878366	0.022687	0.871149	0.035234	0.801355	0.057922	0.680233	6785104	8.326221	8.475864	8.588913	8.467501	8.498609	8.319185	8.248982	8.549071	8.41918	8.313753	8.257454	8.710239	Gprc5c	NM_147217
6785106	2323	8.776272	8.967358	8.769069	8.783471	-0.0072	0.971325	0.183887	0.371228	-0.0144	0.942682	0.198289	0.336926	-0.19109	0.353766	0.007202	0.971313	6785106	8.764308	8.719433	8.845074	9.083243	8.834737	8.984094	8.482042	9.023678	8.801488	8.617716	8.532194	9.200503	Gprc5c	NM_001110337
6785111	2324	6.705396	6.511671	6.486665	6.129994	0.575402	0.012116	0.381677	0.06474	0.356671	0.080499	0.025006	0.891947	0.193725	0.308962	0.218731	0.254848	6785111	6.965538	6.588923	6.561727	6.516034	6.865974	6.153004	6.376932	6.489296	6.593767	6.134941	6.096188	6.158852	Cd300a	NM_170758
6785115	2326	8.69788	8.669733	8.607701	8.608416	0.089465	0.351393	0.061318	0.51678	-0.00071	0.993887	0.062032	0.51204	0.028147	0.763518	0.090179	0.347771	6785115	8.64563	8.654255	8.793756	8.637535	8.833234	8.538431	8.717232	8.641671	8.4642	8.547778	8.638678	8.638791	Slc9a3r1	NM_012030
6785130	2329	8.653559	8.676411	8.679065	8.763565	-0.11001	0.441491	-0.08715	0.539108	-0.0845	0.551241	-0.00265	0.984895	-0.02285	0.870585	-0.02551	0.855746	6785130	8.523474	8.669403	8.767799	8.601417	8.78287	8.644947	8.83118	8.780512	8.425502	8.603037	8.70798	8.979679	Ict1	NM_026729
6785133	2330	6.810536	6.80084	6.765941	6.869722	-0.05919	0.68258	-0.06888	0.634771	-0.10378	0.478231	0.0349	0.808777	0.009696	0.946299	0.044596	0.757425	6785133	6.820844	6.775201	6.835564	6.611827	7.003482	6.787212	6.830288	6.915715	6.551819	6.657886	6.889367	7.061914	Kctd2	NM_183285
6785139	2331	7.038967	6.911305	6.970512	6.912577	0.12639	0.176728	-0.00127	0.988468	0.057935	0.516217	-0.05921	0.507304	0.127662	0.172893	0.068455	0.445471	6785139	7.128572	7.037829	6.950501	6.90689	7.028304	6.798721	6.944077	7.013918	6.953541	6.783234	6.884721	7.069776	Armc7	NM_177778
6785147	2332	6.996936	7.086834	6.937168	6.884676	0.11226	0.151349	0.202158	0.021266	0.052492	0.479469	0.149666	0.06737	-0.0899	0.239687	0.059768	0.422916	6785147	6.93716	7.062318	6.991329	6.974427	7.221327	7.064749	6.975089	6.892865	6.94355	6.787231	6.974189	6.892608	Nup85	NM_001002929
6785148	2333	8.429424	8.504449	8.409013	8.540213	-0.11079	0.441303	-0.03576	0.800305	-0.1312	0.365467	0.095436	0.505041	-0.07503	0.598249	0.020411	0.88505	6785148	8.302223	8.479312	8.506737	8.453834	8.720513	8.339001	8.583064	8.45578	8.188195	8.397052	8.537029	8.686557	Mrps7	NM_025305
6785163	2334	9.012151	9.103922	9.087242	8.974361	0.03779	0.599895	0.129561	0.09807	0.112881	0.141494	0.01668	0.815589	-0.09177	0.221404	-0.07509	0.30949	6785163	8.95186	8.993622	9.090972	9.229535	9.002402	9.079828	9.05513	9.14112	9.065475	8.884952	8.973184	9.064947	2310067B	NM_028014
6785168	2335	8.18434	8.135894	7.929466	8.287052	-0.10271	0.512549	-0.15116	0.342756	-0.35759	0.044153	0.206428	0.205752	0.048447	0.754818	0.254875	0.127472	6785168	8.10619	8.111454	8.335377	8.213934	8.341604	7.852143	7.845287	8.091538	7.851572	8.088214	8.32433	8.448612	Llgl2	NM_145438
6785178	2337	8.584914	8.599967	8.42063	8.440372	0.144543	0.157504	0.159596	0.123393	-0.01974	0.836662	0.179337	0.089049	-0.01505	0.875013	0.164284	0.114256	6785178	8.610577	8.635437	8.508729	8.533643	8.766724	8.499535	8.490123	8.304381	8.467387	8.362585	8.373965	8.584565	Sap30bp	NM_020483
6785183	2338	8.378623	8.183696	7.847071	7.683055	0.695568	0.024192	0.500641	0.081048	0.164016	0.53156	0.336625	0.216461	0.194927	0.459484	0.531552	0.066932	6785183	8.502757	8.183291	8.44982	8.175747	8.499866	7.875474	8.187512	7.549547	7.804153	7.577911	8.108871	7.362382	Itgb4	NM_001005608
6785188	2339	7.633627	7.672335	7.661791	7.685492	-0.05186	0.316161	-0.01316	0.793077	-0.0237	0.638234	0.010544	0.833364	-0.03871	0.447908	-0.02816	0.577474	6785188	7.664911	7.662119	7.573851	7.734469	7.718492	7.564044	7.65446	7.623362	7.707552	7.711088	7.689205	7.656182	Unk	NM_172569
6785206	2340	7.455595	7.491192	7.565277	7.377113	0.078482	0.549802	0.11408	0.39066	0.188164	0.172807	-0.07408	0.571898	-0.0356	0.78423	-0.10968	0.40835	6785206	7.365032	7.436102	7.56565	7.447403	7.514363	7.511811	7.696611	7.751454	7.247765	7.343706	7.317469	7.470163	2310004N	BC027639
6785224	2343	7.662671	7.584574	7.321678	7.159257	0.503414	0.238446	0.425317	0.313173	0.162421	0.691845	0.262896	0.524579	0.078097	0.848262	0.340993	0.413302	6785224	8.161079	7.168754	7.658179	7.319989	8.144923	7.28881	7.893547	6.910778	7.160708	7.57861	6.700171	7.19899	Sphk1	NM_011451
6785236	2345	6.233552	6.280476	6.085611	6.352598	-0.11905	0.428996	-0.07212	0.627391	-0.26699	0.098673	0.194865	0.209834	-0.04692	0.751076	0.147941	0.330843	6785236	6.14071	6.364291	6.195654	6.234278	6.516909	6.09024	6.174327	6.266947	5.815558	6.297448	6.327947	6.432399	1810032O	BC055707
6785246	2346	8.570208	8.605572	8.543245	8.577216	-0.00701	0.964843	0.028356	0.858584	-0.03397	0.831041	0.062327	0.696471	-0.03536	0.824251	0.026964	0.865445	6785246	8.770965	8.393434	8.546226	8.626781	8.59574	8.594194	8.754395	8.651765	8.223574	8.737058	8.409891	8.584699	1110005A	BC026936
6785247	2347	8.918857	8.9746	9.132248	8.900627	0.01823	0.835295	0.073973	0.408819	0.231621	0.02588	-0.15765	0.100309	-0.05574	0.529746	-0.21339	0.036126	6785247	8.805905	8.879088	9.071579	8.905761	9.067887	8.950151	9.189898	9.134079	9.072768	8.77126	8.928143	9.002478	Mfsd11	NM_178620
6785259	2349	9.629097	9.798558	9.735764	9.600107	0.028989	0.772479	0.198451	0.074786	0.135657	0.199167	0.062794	0.535195	-0.16946	0.118505	-0.10667	0.303087	6785259	9.553014	9.637823	9.696453	9.811975	9.781945	9.801754	9.704108	9.823049	9.680135	9.35509	9.716086	9.729146	Sec14l1	NM_028777
6785261	2350	8.526947	8.602918	8.317929	8.040788	0.486159	0.023304	0.56213	0.011982	0.277141	0.149492	0.284989	0.13971	-0.07597	0.673606	0.209018	0.263529	6785261	8.589143	8.419862	8.571836	8.445156	8.96232	8.401278	8.47894	8.16013	8.314716	7.866297	8.292276	7.96379	9-Sep	NM_001113486
6785283	2352	7.348261	7.420422	7.368649	7.154974	0.193288	0.288435	0.265448	0.157026	0.213675	0.244224	0.051773	0.768476	-0.07216	0.682395	-0.02039	0.907494	6785283	7.204931	7.487643	7.35221	7.346407	7.638268	7.27659	7.150913	7.544367	7.410667	6.979317	7.47392	7.011684	Tnrc6c	NM_198022
6785294	2353	6.312981	6.410729	6.393636	6.250174	0.062807	0.64779	0.160555	0.259688	0.143462	0.309965	0.017093	0.900428	-0.09775	0.481277	-0.08066	0.559157	6785294	6.534387	6.332894	6.071662	6.582079	6.386025	6.264083	6.359882	6.335138	6.485889	6.30432	6.093489	6.352714	Tmc8	NM_181856
6785299	2354	10.73278	10.65747	10.71542	10.5168	0.21598	0.048487	0.140667	0.168311	0.19862	0.064903	-0.05795	0.549975	0.075313	0.440836	0.01736	0.856367	6785299	10.90858	10.61773	10.67204	10.67341	10.63863	10.66037	10.89094	10.66813	10.5872	10.45793	10.53282	10.55966	Syngr2	NM_009304
6785306	2355	6.880779	6.915488	6.930019	6.952681	-0.0719	0.438235	-0.03719	0.684144	-0.02266	0.803573	-0.01453	0.873139	-0.03471	0.70402	-0.04924	0.591682	6785306	6.811491	6.815768	7.015079	7.011701	6.902372	6.83239	6.90044	7.016667	6.872949	6.998231	6.797487	7.062325	Afmid	NM_027827
6785321	2358	8.97622	8.799065	8.898191	8.768161	0.208059	0.148593	0.030903	0.81829	0.13003	0.347037	-0.09913	0.468157	0.177156	0.210583	0.078029	0.565427	6785321	9.070436	8.958011	8.900214	8.763039	9.053363	8.580792	8.920785	9.024314	8.749475	8.748106	8.911528	8.64485	Pgs1	NM_133757
6785347	2359	7.430378	7.529261	7.365155	6.938611	0.491766	0.182597	0.590649	0.117738	0.426543	0.241211	0.164106	0.63933	-0.09888	0.776657	0.065223	0.851352	6785347	7.572426	7.519447	7.19926	7.198054	8.178265	7.211463	7.728159	6.997426	7.369879	6.676708	7.443296	6.69583	C1qtnf1	NM_019959
6785348	2360	6.478224	6.595382	6.527252	6.495176	-0.01695	0.854125	0.100206	0.294228	0.032076	0.728672	0.06813	0.46728	-0.11716	0.225806	-0.04903	0.597868	6785348	6.41943	6.428807	6.586434	6.593526	6.680607	6.512012	6.580823	6.664433	6.3365	6.444226	6.490279	6.551023	Engase	NM_172573
6785356	2361	6.785785	6.998382	6.803253	6.88495	-0.09917	0.316713	0.113432	0.256644	-0.0817	0.404614	0.195129	0.06879	-0.2126	0.051305	-0.01747	0.855464	6785356	6.807686	6.744969	6.804699	7.210988	6.86714	6.917018	6.854151	6.766345	6.789264	6.856316	6.784067	7.014468	Cbx2	NM_007623
6785364	2362	6.15291	6.335041	6.273811	6.238887	-0.08598	0.290002	0.096154	0.240734	0.034924	0.657588	0.06123	0.443037	-0.18213	0.043161	-0.1209	0.149756	6785364	6.061491	6.147775	6.249464	6.426706	6.360785	6.217631	6.216124	6.352864	6.252445	6.345787	6.15934	6.211534	Ccdc40	NM_175430
6785367	2363	9.854495	9.772447	9.689789	10.19315	-0.33866	0.039666	-0.42071	0.015845	-0.50336	0.006513	0.082658	0.565712	0.082048	0.568524	0.164706	0.266797	6785367	9.803667	9.844253	9.915566	9.714877	9.941458	9.661007	9.689991	9.946608	9.432768	10.1792	10.04939	10.35087	Gaa	NM_008064
6785368	2364	6.036257	5.996922	6.059761	6.023993	0.012264	0.779129	-0.02707	0.539853	0.035768	0.422103	-0.06284	0.175482	0.039335	0.379371	-0.0235	0.593431	6785368	6.075816	6.059558	5.973396	6.051682	5.971562	5.967521	6.011676	6.134253	6.033355	5.987393	6.029762	6.054824	Card14	NM_130886
6785369	2365	6.6257	6.712783	6.598508	6.608807	0.016894	0.848468	0.103977	0.259112	-0.0103	0.907204	0.114275	0.218611	-0.08708	0.338766	0.027192	0.758868	6785369	6.558751	6.552005	6.766345	6.690215	6.885557	6.562578	6.61066	6.621176	6.563689	6.574279	6.654764	6.597377	Slc26a11	NM_178743
6785384	2366	9.124133	9.131743	8.950252	8.634459	0.489674	0.052994	0.497284	0.050156	0.315793	0.181583	0.181491	0.424834	-0.00761	0.972738	0.173881	0.443746	6785384	8.913696	9.170448	9.288254	8.95469	9.523579	8.916959	8.876171	8.952953	9.021631	8.274405	8.971945	8.657027	Rnf213	AK173199
6785385	2367	7.169797	7.411377	7.181778	7.295133	-0.12534	0.454087	0.116244	0.486386	-0.11336	0.496952	0.2296	0.18748	-0.24158	0.167871	-0.01198	0.941899	6785385	7.258221	7.19764	7.053531	7.568441	7.076205	7.589486	7.259906	7.086553	7.198874	7.167311	7.167061	7.551028	A730011L	NM_177394
6785394	2368	8.485937	8.451987	8.311528	8.49408	-0.00814	0.888803	-0.04209	0.47698	-0.18255	0.011957	0.140459	0.037551	0.033949	0.56402	0.174408	0.014862	6785394	8.493195	8.464535	8.50008	8.484529	8.432213	8.43922	8.267633	8.433781	8.233171	8.401606	8.544558	8.536077	4932417H	NM_028898
6785399	2369	8.491369	8.521021	8.457072	8.468133	0.023236	0.858166	0.052888	0.685478	-0.01106	0.93215	0.063949	0.6252	-0.02965	0.819719	0.034297	0.792201	6785399	8.560716	8.415922	8.497469	8.621499	8.628952	8.312613	8.406046	8.665085	8.300085	8.39297	8.372491	8.638938	Baiap2	NM_130862
6785418	2371	6.97726	7.10682	6.958886	6.930648	0.046612	0.517109	0.176172	0.033585	0.028238	0.692193	0.147934	0.06371	-0.12956	0.09638	0.018374	0.796133	6785418	6.948044	7.067441	6.916296	7.158552	7.089656	7.072252	7.015859	7.04564	6.81516	6.891971	6.894151	7.005822	Bahcc1	NM_198423
6785434	2373	5.921623	5.933978	5.875192	5.830903	0.09072	0.570719	0.103075	0.520698	0.044289	0.780215	0.058786	0.711636	-0.01235	0.937807	0.046431	0.769934	6785434	5.977827	5.843612	5.94343	5.9853	6.071157	5.745477	6.031635	6.057206	5.536735	5.872205	5.953709	5.666795	Tspan10	NM_145363
6785437	2374	6.710988	6.743661	6.590004	6.721411	-0.01042	0.881289	0.02225	0.750519	-0.13141	0.087834	0.153657	0.052653	-0.03267	0.641838	0.120984	0.111309	6785437	6.663372	6.791269	6.678324	6.695776	6.711752	6.823455	6.659111	6.660967	6.449935	6.692001	6.784378	6.687855	Ccdc137	NM_152807
6785440	2375	9.198916	9.041941	9.179922	9.024533	0.174383	0.067938	0.017408	0.838489	0.155389	0.096961	-0.13798	0.133657	0.156976	0.094138	0.018994	0.824043	6785440	9.166324	9.190138	9.240287	9.064757	9.142582	8.918483	9.073983	9.225637	9.240146	8.97073	9.177902	8.924967	Hgs	NM_001159328
6785441	2376	8.427324	8.513069	8.336834	8.584208	-0.15688	0.331418	-0.07114	0.651703	-0.24737	0.141692	0.176234	0.278942	-0.08574	0.58751	0.09049	0.567424	6785441	8.422275	8.357886	8.501812	8.512403	8.412292	8.614511	8.392827	8.53589	8.081786	8.338085	8.554335	8.860203	Mrpl12	NM_027204
6785446	2377	8.655274	8.787059	8.676958	9.02395	-0.36868	0.211378	-0.23689	0.408164	-0.34699	0.23689	0.110101	0.695601	-0.13179	0.640274	-0.02168	0.938279	6785446	8.719365	8.502215	8.744242	8.973623	8.78866	8.598895	8.853955	8.789657	8.387262	8.882855	8.537564	9.65143	Slc25a10	NM_013770
6785460	2379	6.761204	6.695801	6.822405	6.732867	0.028337	0.793297	-0.03707	0.732193	0.089538	0.416838	-0.1266	0.260626	0.065403	0.549161	-0.0612	0.57456	6785460	6.571681	6.73338	6.97855	6.748813	6.743501	6.595088	6.850679	6.931039	6.685497	6.736286	6.750349	6.711966	Anapc11	NM_025389
6785467	2381	6.112152	5.997487	6.036783	6.114123	-0.00197	0.99207	-0.11664	0.560819	-0.07734	0.697966	-0.0393	0.84312	0.114665	0.567317	0.075369	0.705229	6785467	6.140681	6.179273	6.016502	6.045747	5.890588	6.056125	6.23254	6.343979	5.533831	6.245816	6.042591	6.053962	Notum	NM_175263
6785468	2382	8.211471	8.252797	8.234169	8.215918	-0.00445	0.969439	0.036879	0.751476	0.018251	0.87515	0.018628	0.872599	-0.04133	0.722901	-0.0227	0.845145	6785468	8.328565	8.104593	8.201254	8.344308	8.335231	8.078852	8.217559	8.41474	8.070208	8.103353	8.237182	8.307218	Aspscr1	NM_026877
6785481	2383	7.366493	7.523063	7.396008	7.405282	-0.03879	0.273522	0.117781	0.007295	-0.00927	0.785775	0.127055	0.004871	-0.15657	0.001454	-0.02952	0.397121	6785481	7.302759	7.417387	7.379333	7.509244	7.553587	7.506359	7.426725	7.351579	7.409721	7.384918	7.391841	7.439088	Lrrc45	NM_153545
6785483	2384	7.036348	7.034518	6.84135	7.411618	-0.37527	0.17564	-0.3771	0.173778	-0.57027	0.053904	0.193168	0.466348	0.001829	0.994398	0.194998	0.462282	6785483	6.811337	6.869643	7.428063	7.100559	7.325832	6.677164	6.98958	7.135712	6.398758	7.465104	7.321919	7.44783	Rac3	NM_133223
6785489	2385	7.024864	6.770417	6.934424	7.055128	-0.03026	0.854085	-0.28471	0.111769	-0.1207	0.470439	-0.16401	0.333437	0.254447	0.148945	0.09044	0.585869	6785489	7.354941	6.83901	6.88064	6.856634	6.682055	6.772561	7.079206	6.96836	6.755705	7.272563	6.95467	6.938151	Gps1	NM_145370
6785508	2386	6.306863	6.373071	6.235575	6.58359	-0.27673	0.323727	-0.21052	0.446831	-0.34802	0.222574	0.137496	0.615492	-0.06621	0.807702	0.071288	0.793332	6785508	6.095819	6.361347	6.463423	6.283601	6.638787	6.196825	6.758215	5.819647	6.128863	6.883573	6.265037	6.602161	Slc16a3	NM_001038653
6785538	2390	7.418387	7.66959	7.383458	7.635473	-0.21709	0.240775	0.034117	0.847132	-0.25202	0.179518	0.286132	0.133458	-0.2512	0.18077	0.034929	0.843547	6785538	7.112818	7.454147	7.688195	7.599582	7.625068	7.78412	7.414878	7.338695	7.3968	7.478909	7.463887	7.963624	Narf	NM_026272
6785549	2391	7.48628	7.605297	7.531441	7.674541	-0.18826	0.071834	-0.06924	0.467551	-0.1431	0.153647	0.073856	0.439493	-0.11902	0.22628	-0.04516	0.632283	6785549	7.375506	7.525188	7.558145	7.589263	7.712257	7.51437	7.462401	7.652085	7.479836	7.574339	7.617498	7.831787	Fn3krp	NM_181420
6785551	2393	8.148874	8.186001	8.003957	8.080567	0.068307	0.608564	0.105434	0.434572	-0.07661	0.566567	0.182044	0.193298	-0.03713	0.779439	0.144917	0.290963	6785551	8.143618	8.121576	8.181428	8.219951	8.250001	8.088051	8.17352	7.759321	8.079031	7.880476	8.066212	8.295013	Tbcd	NM_029878
6785558	2394	6.413545	6.486361	6.397202	6.208473	0.205072	0.171087	0.277888	0.075966	0.188729	0.203803	0.089158	0.531627	-0.07282	0.607925	0.016343	0.907575	6785558	6.411028	6.422633	6.406974	6.325929	6.645062	6.488091	6.457829	6.166412	6.567366	6.296196	6.357891	5.971332	Metrnl	NM_144797
6785576	2395	8.129241	8.115687	8.070219	8.089494	0.039747	0.699711	0.026193	0.798812	-0.01928	0.851073	0.045469	0.659524	0.013554	0.894906	0.059023	0.569054	6785576	7.990819	8.221012	8.175893	8.165895	7.982021	8.199146	8.033628	8.148893	8.028135	8.257709	7.935724	8.075049	Sfi1	NM_030207
6785591	2396	8.516407	8.571154	8.598338	8.523088	-0.00668	0.924239	0.048066	0.500171	0.075251	0.301101	-0.02718	0.700087	-0.05475	0.444503	-0.08193	0.263156	6785591	8.524511	8.506692	8.518019	8.484774	8.743499	8.485188	8.626795	8.530195	8.638025	8.472787	8.542477	8.553999	Drg1	NM_007879
6785601	2397	7.89482	8.102274	7.808931	7.887424	0.007396	0.940208	0.21485	0.054716	-0.07849	0.435225	0.293343	0.015357	-0.20745	0.061736	0.085889	0.395019	6785601	7.907068	7.87442	7.902973	8.115855	8.178288	8.012678	7.894451	7.700062	7.83228	7.703301	8.089942	7.869029	Limk2	NM_173053
6785603	2398	8.657624	8.790879	8.940008	8.86921	-0.21159	0.05671	-0.07833	0.433905	0.070798	0.477811	-0.14913	0.155416	-0.13325	0.198652	-0.28238	0.017873	6785603	8.603907	8.735216	8.63375	8.787863	8.842558	8.742216	8.856854	9.182187	8.780984	8.837438	8.913323	8.85687	Rnf185	NM_145355
6785608	2399	6.392161	6.359438	6.447522	6.329083	0.063078	0.486595	0.030355	0.734666	0.118439	0.208062	-0.08808	0.33826	0.032723	0.715005	-0.05536	0.539992	6785608	6.397905	6.312454	6.466124	6.395149	6.38544	6.297725	6.438513	6.499378	6.404676	6.337596	6.141214	6.508439	Inpp5j	NM_172439
6785609	2400	8.420569	8.904376	8.413787	8.596447	-0.17588	0.346433	0.307929	0.117976	-0.18266	0.329216	0.490589	0.023545	-0.48381	0.02499	0.006782	0.970173	6785609	8.235995	8.598226	8.427486	9.009088	8.866002	8.838037	8.160569	8.635201	8.44559	8.356577	8.506671	8.926092	Smtn	NM_001159284
6785617	2401	9.044667	9.184753	9.038315	9.065517	-0.02085	0.859945	0.119236	0.327848	-0.0272	0.81807	0.146438	0.236484	-0.14009	0.255675	0.006351	0.957096	6785617	9.052254	9.120512	8.961234	8.949018	9.27076	9.334481	9.09446	9.066325	8.954161	8.911315	9.064293	9.220942	Tug1	NR_002322

6785624	2402	6.441193	6.668485	6.262102	6.683445	-0.24225	0.000314	-0.01496	0.719479	-0.42134	5.97E-06	0.406382	7.83E-06	-0.22729	0.000479	0.17909	0.002126	6785624	6.461162	6.493094	6.369322	6.638467	6.716995	6.649992	6.286427	6.291838	6.208042	6.645966	6.679418	6.72495	Osbp2	NM_152818
6785631	2404	7.474632	7.522116	7.352735	7.541056	-0.06642	0.47883	-0.01894	0.83755	-0.18832	0.068295	0.169381	0.09481	-0.04748	0.609832	0.121897	0.209948	6785631	7.599061	7.418734	7.406102	7.475812	7.6785	7.412035	7.329145	7.436022	7.293038	7.556204	7.427346	7.639618	Slc35e4	NM_153142
6785632	2405	12.22849	12.22784	12.379	12.60217	-0.37368	0.013335	-0.37433	0.013225	-0.22316	0.095579	-0.15116	0.236574	0.00065	0.995745	-0.15051	0.238421	6785632	12.40041	12.11034	12.17472	12.08447	12.29082	12.30823	12.43458	12.41394	12.28849	12.71933	12.37476	12.71241	Tcn2	NM_015749
6785641	2406	6.398151	6.370619	6.395299	6.484405	-0.08625	0.661692	-0.11379	0.565556	-0.08911	0.651375	-0.02468	0.899784	0.027532	0.888292	0.002851	0.988386	6785641	6.093212	6.375532	6.725708	6.336563	6.45799	6.317303	6.540687	6.420542	6.224669	6.296248	6.337478	6.819489	1700020C	NM_026443
6785642	2407	6.737852	6.779661	6.836846	6.564817	0.173035	0.248195	0.214844	0.160589	0.272029	0.08592	-0.05718	0.691433	-0.04181	0.771137	-0.09899	0.496381	6785642	6.818634	6.580503	6.814419	6.628156	6.957335	6.753492	6.927273	6.713665	6.8696	6.611281	6.777809	6.30536	Sec14l2	NM_144520
6785644	2408	6.30748	6.353763	6.320539	6.273691	0.033789	0.602497	0.080072	0.234833	0.046848	0.473786	0.033224	0.608466	-0.04628	0.478956	-0.01306	0.839273	6785644	6.296686	6.296493	6.329261	6.395123	6.371012	6.295155	6.324758	6.310369	6.32649	6.410693	6.283127	6.127254	Ccdc157	NM_177616
6785663	2409	8.799849	8.764489	8.765808	8.848066	-0.04822	0.404063	-0.08358	0.165303	-0.08226	0.171292	-0.00132	0.98137	0.03536	0.536361	0.034041	0.551305	6785663	8.740974	8.801533	8.85704	8.807709	8.787493	8.698265	8.676552	8.819319	8.801552	8.806976	8.931019	8.806202	Mtmr3	NM_028860
6785665	2411	8.803815	9.000363	8.888679	9.386525	-0.58271	0.052296	-0.38616	0.169704	-0.49785	0.087587	0.111684	0.674042	-0.19655	0.464488	-0.08486	0.748676	6785665	8.445299	8.919744	9.046401	9.002758	8.956567	9.041763	8.986747	9.081198	8.598091	9.132224	9.093473	9.933879	1110020P	NM_197979
6785669	2412	5.75875	5.929225	5.818577	5.884365	-0.12562	0.448349	0.044859	0.783111	-0.06579	0.687282	0.110648	0.502466	-0.17047	0.310832	-0.05983	0.714062	6785669	5.663527	6.031547	5.581176	5.717775	5.969809	6.10009	5.920365	5.798134	5.737232	5.843144	5.695896	6.114056	Cabp7	NM_138948
6785675	2413	7.756383	7.824747	7.738724	7.701316	0.055067	0.56977	0.123431	0.220671	0.037409	0.697772	0.086023	0.38161	-0.06836	0.482873	0.017658	0.854002	6785675	7.838133	7.750452	7.680563	7.842916	7.918926	7.712399	7.806808	7.638415	7.77095	7.529104	7.854817	7.720026	Nf2	NM_010898
6785694	2415	7.67478	7.691698	7.636669	7.538506	0.136274	0.27081	0.153191	0.220265	0.098163	0.418945	0.055029	0.645667	-0.01692	0.886883	0.038111	0.749282	6785694	7.633876	7.591953	7.798511	7.70378	7.820317	7.550996	7.627839	7.851	7.431168	7.53213	7.468805	7.614584	Gas2l1	NM_144560
6785697	2416	10.86733	10.85139	10.86654	10.72887	0.13846	0.283708	0.12252	0.339004	0.137673	0.286256	-0.01515	0.903025	0.01594	0.898023	0.000787	0.994951	6785697	10.97164	10.83577	10.79457	10.7902	10.92456	10.8394	10.86236	10.92166	10.8156	10.47519	10.70465	11.00676	Ewsr1	NM_007968
6785698	2417	6.1637	6.234692	6.267998	6.48212	-0.31842	0.022461	-0.24743	0.059733	-0.21412	0.094411	-0.03331	0.775455	-0.07099	0.546932	-0.1043	0.382514	6785698	6.213605	6.030419	6.247077	6.194521	6.286047	6.223507	6.28207	6.396377	6.125547	6.320641	6.41248	6.713239	Emid1	NM_080595
6785709	2418	7.944217	7.977279	7.660987	7.645959	0.298258	0.006444	0.33132	0.003631	0.015028	0.858459	0.316292	0.004699	-0.03306	0.695957	0.283231	0.008428	6785709	7.992486	7.931081	7.909085	7.962259	8.081797	7.88778	7.658803	7.684693	7.639464	7.512696	7.833684	7.591497	Kremen1	NM_032396
6785726	2419	7.67701	7.806496	7.704208	7.76154	-0.08453	0.360334	0.044956	0.619801	-0.05733	0.528971	0.102288	0.274127	-0.12949	0.175516	-0.0272	0.762879	6785726	7.571428	7.68686	7.772741	7.717634	7.723251	7.978602	7.736363	7.792507	7.583754	7.743993	7.735497	7.80513	Ccdc117	NM_134033
6785742	2420	8.799193	8.811099	8.822415	8.901059	-0.10187	0.246634	-0.08996	0.301738	-0.07864	0.362791	-0.01132	0.892998	-0.01191	0.887462	-0.02322	0.782923	6785742	8.734377	8.759964	8.903239	8.88772	8.681154	8.864423	8.858317	8.862547	8.74638	8.784922	8.893488	9.024766	Mrps24	NM_026080
6785743	2421	7.382821	7.311181	7.360138	7.26825	0.114572	0.317453	0.042931	0.699968	0.091888	0.417367	-0.04896	0.660799	0.07164	0.523735	0.022684	0.838093	6785743	7.42999	7.424729	7.293745	7.397786	7.198298	7.337459	7.318693	7.545469	7.216251	7.181855	7.448841	7.174053	2010005J	NM_178623
6785745	2422	7.801677	7.204761	7.311842	7.402542	0.399135	0.652086	-0.19778	0.822334	-0.0907	0.917877	-0.10708	0.903126	0.596916	0.50359	0.489835	0.581306	6785745	6.385818	7.425514	9.593699	8.017765	7.193589	6.40293	7.873139	7.215926	6.846462	6.402617	7.81259	7.992419	Pgam2	NM_018870
6785746	2423	6.24736	6.391482	6.426389	6.305601	-0.05824	0.507571	0.08588	0.336357	0.120787	0.188253	-0.03491	0.688552	-0.14412	0.124434	-0.17903	0.065584	6785746	6.178089	6.375961	6.18803	6.379548	6.430534	6.364363	6.454896	6.322708	6.501562	6.150413	6.338396	6.427995	Polm	NM_017401
6785750	2424	7.615481	7.599853	7.90193	7.866058	-0.25058	0.064598	-0.26621	0.05245	0.035872	0.766966	-0.30208	0.032517	0.015629	0.897031	-0.28645	0.040038	6785750	7.400826	7.650312	7.795306	7.570056	7.730118	7.499384	7.894785	7.875347	7.935659	7.900137	8.011867	7.68617	Pold2	NM_008894
6785753	2426	6.193014	6.216265	6.262569	6.412636	-0.21962	0.245273	-0.19637	0.294743	-0.15007	0.416484	-0.0463	0.79818	-0.02325	0.897668	-0.06956	0.701664	6785753	6.238228	6.193413	6.1474	6.342847	6.059886	6.246062	6.39592	6.382841	6.008945	6.408558	6.078491	6.750858	Gck	NM_010292
6785758	2427	6.88651	7.337487	6.892944	6.618549	0.267961	0.348702	0.718938	0.028336	0.274395	0.337903	0.444543	0.137279	-0.45098	0.132422	-0.00643	0.98152	6785758	6.431975	6.680785	7.546771	7.382596	7.160075	7.46979	7.044668	6.832714	6.801449	6.44239	6.868194	6.545063	Camk2b	NM_007595
6785762	2428	8.7391	8.612696	8.708526	8.761956	-0.02286	0.830869	-0.14926	0.187517	-0.05343	0.619894	-0.09583	0.381891	0.126404	0.257042	0.030573	0.775363	6785762	8.581024	8.728208	8.908067	8.597863	8.677135	8.56309	8.837223	8.744337	8.544019	8.64096	8.848581	8.796326	Nudcd3	NM_173748
6785774	2430	7.300496	7.325217	7.337059	7.326885	-0.02639	0.734333	-0.00167	0.982825	0.010174	0.895568	-0.01184	0.878597	-0.02472	0.750433	-0.03656	0.639363	6785774	7.265491	7.244168	7.391829	7.244479	7.362351	7.368822	7.456944	7.328873	7.22536	7.249215	7.433373	7.298067	Ddx56	NM_026538
6785777	2431	10.50157	10.6422	10.55638	10.82924	-0.32766	0.010136	-0.18704	0.092515	-0.27286	0.023683	0.085823	0.406321	-0.14063	0.188886	-0.0548	0.591019	6785777	10.44469	10.48708	10.57295	10.56573	10.6339	10.72697	10.69677	10.58027	10.39209	10.80688	10.68976	10.99107	Tmed4	NM_134020
6785793	2432	8.115177	8.446937	8.25709	8.549448	-0.43427	0.034437	-0.10251	0.564617	-0.29236	0.124992	0.189847	0.298186	-0.33176	0.08776	-0.14191	0.429706	6785793	8.034575	8.175512	8.135444	8.43929	8.430718	8.470803	8.302598	8.502931	7.965741	8.330507	8.415356	8.902482	H2afv	BC147294
6785797	2433	8.292578	8.354664	8.280163	8.408686	-0.11611	0.197439	-0.05402	0.53143	-0.12852	0.158316	0.0745	0.393418	-0.06209	0.473784	0.012415	0.884243	6785797	8.285335	8.299992	8.292408	8.305019	8.434729	8.324243	8.31059	8.425701	8.104199	8.333523	8.518431	8.374104	Purb	NM_011221
6785799	2434	7.051233	6.720745	6.797081	6.399657	0.651576	0.010784	0.321089	0.142074	0.397425	0.07859	-0.07634	0.708737	0.330488	0.132237	0.254152	0.233423	6785799	7.254836	7.027593	6.87127	6.736037	6.885107	6.541092	7.02166	6.371019	6.998565	6.289469	6.599361	6.31014	Myo1g	NM_178440
6785808	2435	7.26239	7.266525	7.218791	7.258522	0.003868	0.975162	0.008003	0.948644	-0.03973	0.749935	0.047734	0.70219	-0.00413	0.97345	0.043599	0.726699	6785808	7.286326	7.239492	7.261353	7.312778	7.348405	7.138393	7.140425	7.502587	7.013361	7.155439	7.347807	7.27232	Tbrg4	NM_134011
6785865	2436	10.90454	11.54839	11.13562	11.62269	-0.71815	0.003279	-0.0743	0.680118	-0.48707	0.023053	0.41277	0.044803	-0.64385	0.005985	-0.23108	0.220082	6785865	10.72453	11.12207	10.86703	11.46071	11.3561	11.82837	11.12895	10.92994	11.34798	11.55591	11.83462	11.47755	Igfbp3	NM_008343
6785935	2437	6.36725	6.506435	6.498146	6.352028	0.015222	0.869035	0.154407	0.122426	0.146118	0.140826	0.008289	0.928408	-0.13919	0.158129	-0.1309	0.181325	6785935	6.356541	6.401552	6.343657	6.443229	6.681356	6.394721	6.51228	6.39717	6.584988	6.2435	6.330821	6.481764	Hus1	NM_008316
6786044	2442	6.144163	6.210668	5.65493	5.280154	0.864009	0.076475	0.930514	0.059948	0.374776	0.403532	0.555738	0.227275	-0.0665	0.879516	0.489234	0.282861	6786044	5.879573	5.830355	6.722562	5.845276	6.788089	5.998638	6.280721	5.058351	5.625717	5.31994	5.708203	4.812319	Fignl1	NM_021891
6786046	2444	8.286733	8.910996	8.571245	8.747215	-0.46048	0.109756	0.16378	0.540202	-0.17597	0.511265	0.339751	0.221077	-0.62426	0.040655	-0.28451	0.298681	6786046	7.94264	8.290889	8.626669	8.832994	9.077785	8.822208	8.842541	8.410872	8.460321	8.867916	9.121128	8.252602	Grb10	NM_010345
6786049	2445	8.588903	8.558067	8.561011	8.344601	0.244302	0.235981	0.213466	0.295384	0.21641	0.289231	-0.00294	0.988056	0.030836	0.87553	0.027892	0.887316	6786049	8.731731	8.448324	8.586654	8.625954	8.879152	8.169094	8.581163	8.673491	8.42838	8.515306	8.43634	8.082157	Cobl	NM_172496
6786249	2446	9.273467	8.887918	9.361907	7.957294	1.316173	0.01658	0.930624	0.065307	1.404613	0.012202	-0.47399	0.308581	0.385549	0.402271	-0.08844	0.844299	6786249	9.805064	9.132314	8.883022	8.872068	9.249555	8.54213	9.996853	8.739316	9.349551	8.035089	8.540862	7.295931	Plek	NM_019549
6786262	2447	7.910953	7.854239	7.941104	7.940491	-0.02954	0.73754	-0.08625	0.340589	0.000613	0.994434	-0.08687	0.337367	0.056715	0.52398	-0.03015	0.732342	6786262	7.948575	7.854839	7.929446	7.719003	8.019705	7.824008	8.035318	7.919454	7.868539	7.823997	8.014456	7.98302	Pno1	NM_025443
6786288	2448	6.463805	6.533248	6.460775	6.341254	0.122551	0.301852	0.191994	0.122074	0.119521	0.313169	0.072473	0.532288	-0.06944	0.549156	0.00303	0.9789	6786288	6.366266	6.532273	6.492875	6.37764	6.723517	6.498587	6.645181	6.414271	6.322872	6.258764	6.328104	6.436894	Etaa1	NM_026576
6786348	2449	6.209859	6.209909	5.986382	6.035201	0.174658	0.30056	0.174708	0.30043	-0.04882	0.764957	0.223527	0.194385	-5E-05	0.999753	0.223477	0.194475	6786348	5.882386	6.30406	6.44313	6.107561	6.248052	6.274114	5.925516	5.994934	6.038696	5.913077	6.300441	5.892084	Meis1	NM_010789
6786463	2450	10.51662	10.56445	10.55719	10.16783	0.34879	0.04648	0.39662	0.028126	0.38936	0.030346	0.00726	0.962142	-0.04783	0.75524	-0.04057	0.791274	6786463	10.57663	10.45774	10.5155	10.4114	10.636	10.64596	10.81806	10.36014	10.49338	9.974961	10.42967	10.09887	Actr2	NM_146243
6786469	2451	7.054082	7.146437	7.102692	7.180653	-0.12657	0.414725	-0.03422	0.821964	-0.07796	0.610623	0.043744	0.773821	-0.09236	0.54773	-0.04861	0.749615	6786469	7.079679	7.031219	7.051347	7.218339	7.242268	6.978703	6.892775	7.428143	6.987159	7.034972	7.151066	7.35592	Cep68	NM_172260
6786473	2452	6.214575	6.306264	6.101447	6.114675	0.099899	0.515486	0.191588	0.228247	-0.01323	0.930429	0.204817	0.200563	-0.09169	0.549732	0.113128	0.463181	6786473	6.335442	6.167851	6.140431	6.357381	6.449391	6.112019	6.322486	6.000454	5.9814	6.171724	6.306671	5.865631	Slc1a4	NM_018861
6786504	2453	8.25777	8.347418	8.323741	8.192642	0.065129	0.474751	0.154776	0.11256	0.131099	0.169579	0.023677	0.792032	-0.08965	0.332126	-0.06597	0.469251	6786504	8.255501	8.268653	8.249157	8.308986	8.342147	8.39112	8.397213	8.431402	8.142607	8.046497	8.315965	8.215463	Aftph	NM_181411
6786505	2454	6.946336	6.707521	6.863628	7.014084	-0.06775	0.760959	-0.30656	0.192108	-0.15046	0.504279	-0.15611	0.488879	0.238816	0.299356	0.082708	0.710748	6786505	6.964825	7.174506	6.699678	6.28256	7.044788	6.795214	6.935245	6.650421	7.005218	6.910439	6.90202	7.229794	---	BC106119
6786516	2455	8.677445	8.677819	8.561886	8.766531	-0.08909	0.724855	-0.08871	0.725952	-0.20464	0.426588	0.115933	0.647836	-0.00037	0.998818	0.115559	0.648879	6786516	8.668018	8.574996	8.789322	8.41532	8.763234	8.854902	8.592052	8.974284	8.119322	8.485139	9.130955	8.683499	1110067D	NM_173752
6786561	2456	8.844943	8.82081	8.705483	8.819652	0.025292	0.897751	0.001159	0.9953	-0.11417	0.565895	0.115328	0.562044	0.024133	0.902407	0.139461	0.4854	6786561	8.644019	8.712237	9.178574	8.933121	8.918582	8.610728	9.008252	8.580457	8.527739	8.617209	8.933415	8.908331	Ugp2	NM_139297
6786572	2458	10.51654	10.66418	10.60096	10.90596	-0.38942	0.058472	-0.24178	0.20806	-0.30499	0.122343	0.063213	0.729572	-0.14764	0.427296	-0.08442	0.645315	6786572	10.40639	10.47485	10.66838	10.64429	10.58352	10.76472	10.80087	10.7078	10.29422	10.70179	10.77128	11.2448	Mdh1	NM_008618
6786586	2460	6.931277	6.891533	6.919117	7.038703	-0.10743	0.076709	-0.14717	0.023823	-0.11959	0.053623	-0.02758	0.616101	0.039744	0.473875	0.012161	0.823909	6786586	6.936	6.896123	6.961709	6.829608	6.880301	6.96469	6.917118	6.968293	6.871939	6.932426	7.076405	7.107277	Ehbp1	NM_153078
6786610	2461	7.626469	7.594966	7.635771	7.507277	0.119192	0.388518	0.087688	0.521234	0.128494	0.354412	-0.04081	0.7629	0.031504	0.815616	-0.0093	0.945017	6786610	7.537736	7.630021	7.711651	7.498001	7.589068	7.697828	7.608112	7.832208	7.466994	7.305089	7.751086	7.465657	Metap2	NM_019648
6786621	2462	6.121526	6.262987	6.296095	6.103826	0.0177	0.843054	0.159161	0.103185	0.192269	0.057032	-0.03311	0.712008	-0.14146	0.140774	-0.17457	0.078403	6786621	5.988043	6.146943	6.229591	6.204092	6.330652	6.254217	6.408508	6.333195	6.146581	6.007504	6.177716	6.126258	Zrsr1	NM_011663
6786659	2463	7.321183	7.477717	7.354045	7.325474	-0.00429	0.955618	0.152243	0.075995	0.02857	0.71218	0.123673	0.136529	-0.15653	0.069509	-0.03286	0.671762	6786659	7.349802	7.333513	7.280235	7.388575	7.613522	7.431055	7.444539	7.346947	7.270648	7.212712	7.354191	7.40952	Ahsa2	NM_172391
6786666	2465	6.993962	7.038545	6.900361	6.673294	0.320668	0.039268	0.36525	0.023092	0.227067	0.119583	0.138183	0.319911	-0.04458	0.741068	0.093601	0.493012	6786666	7.131103	6.883203	6.967581	7.093537	7.061763	6.960334	7.131643	6.795817	6.773624	6.451157	6.846861	6.721865	0610010F	NM_027860
6786668	2466	7.97701	8.106068	8.006632	8.23261	-0.2556	0.006618	-0.12654	0.109451	-0.22598	0.012322	0.099436	0.194819	-0.12906	0.103616	-0.02962	0.684493	6786668	7.881982	7.997717	8.051332	8.04745	8.164879	8.105874	8.010688	8.100098	7.909109	8.154129	8.201539	8.342161	Pex13	NM_023651
6786673	2467	7.86228	7.714606	8.008541	7.659236	0.203044	0.2885	0.055371	0.764464	0.349306	0.086216	-0.29394	0.138429	0.147673	0.432298	-0.14626	0.436538	6786673	8.244966	7.7735	7.568373	7.625113	7.90905	7.609656	8.108171	7.966228	7.951225	7.443061	7.775506	7.75914	Rel	NM_009044
6786674	2468	7.130148	7.224946	7.249372	7.218507	-0.08836	0.337648	0.006439	0.942581	0.030865	0.730872	-0.02443	0.785159	-0.0948	0.305728	-0.11922	0.206082	6786674	7.082147	7.245723	7.062574	7.082547	7.319916	7.272376	7.351985	7.266513	7.129618	7.125974	7.276919	7.252629	Papolg	NM_172555
6786849	2470	6.612976	6.635916	6.768625	6.347102	0.265874	0.187993	0.288814	0.156543	0.421523	0.051905	-0.13271	0.492917	-0.02294	0.904229	-0.15565	0.423896	6786849	6.643712	6.681003	6.514214	6.606017	6.645728	6.656002	6.945088	6.560145	6.800642	5.887931	6.556469	6.596906	Vrk2	NM_027260
6786914	2471	6.400758	6.038905	6.027659	6.308787	0.091971	0.484332	-0.26988	0.063609	-0.28113	0.05531	0.011246	0.930761	0.361852	0.020357	0.373098	0.017746	6786914	6.151579	6.582268	6.468426	6.126412	5.961184	6.02912	6.134426	5.83717	6.111382	6.411912	6.208586	6.305862	Ccdc85a	NM_181577
6786944	2472	7.170424	7.324739	7.35838	7.146066	0.024358	0.809744	0.178673	0.105379	0.212314	0.061908	-0.03364	0.739944	-0.15432	0.153552	-0.18796	0.091085	6786944	7.059588	7.068661	7.383024	7.345181	7.371502	7.257535	7.389453	7.38795	7.297736	7.087038	7.054611	7.296549	Smek2	NM_134034
6786945	2473	8.640512	8.585823	8.540234	8.61869	0.021822	0.859043	-0.03287	0.78937	-0.07846	0.528165	0.045589	0.711584	0.054689	0.658008	0.100278	0.423822	6786945	8.577367	8.564817	8.779351	8.413705	8.755145	8.588618	8.724714	8.427745	8.468242	8.566026	8.52979	8.760253	Ccdc104	NM_025740
6786954	2474	7.383985	7.347782	7.418952	7.375217	0.008768	0.953445	-0.02743	0.855201	0.043735	0.771495	-0.07117	0.638021	0.036203	0.809853	-0.03497	0.816207	6786954	7.467815	7.319778	7.364363	7.197154	7.593568	7.252625	7.692524	7.386909	7.177422	7.314527	7.331908	7.479216	Ncrna001	NM_026465
6786965	2475	7.079713	6.672509	6.762466	7.581719	-0.50201	0.048121	-0.90921	0.002912	-0.81925	0.005212	-0.08996	0.687208	0.407204	0.095363	0.317247	0.179023	6786965	6.940776	7.022281	7.276083	6.6416	6.551836	6.824092	6.97204	6.539311	6.776047	7.655941	7.123326	7.965891	C230094A	NM_146016
6786977	2476	6.49196	6.077188	6.297822	6.893737	-0.40178	0.01043	-0.81655	0.000143	-0.59591	0.001143	-0.22063	0.105114	0.414772	0.008894	0.194138	0.146584	6786977	6.44111	6.480603	6.554168	6.043363	5.893313	6.294889	6.423625	6.34857	6.121271	6.973357	6.73777	6.970083	4931440F	NM_176829

6786978	2477	11.13921	10.95622	10.88181	10.83815	0.301063	0.011227	0.118073	0.234595	0.043663	0.64724	0.07441	0.441315	0.18299	0.081496	0.2574	0.023116	6786978	11.30253	11.11076	11.00434	11.0026	10.95338	10.91268	10.96129	10.87457	10.80957	10.67518	10.91285	10.92641	Spnb2	NM_009260
6786979	2478	7.937535	7.45965	7.300318	7.711342	0.226193	0.062529	-0.25169	0.042743	-0.41102	0.004358	0.159332	0.166162	0.477885	0.001828	0.637216	0.000292	6786979	8.057024	7.955892	7.799688	7.551599	7.505242	7.322109	7.132782	7.300451	7.467722	7.6885	7.798015	7.64751	Spnb2	ENSMUST000000
6786991	2480	7.432557	7.130499	7.254794	7.342221	0.090335	0.727011	-0.21172	0.421341	-0.08743	0.7354	-0.1243	0.632191	0.302058	0.261137	0.177763	0.496952	6786991	7.474579	7.257224	7.565867	7.28806	7.236321	6.867116	7.4621	7.408883	6.893399	7.30541	6.916853	7.804401	Acyp2	NM_029344
6787019	2481	9.643063	9.680698	9.523255	9.725211	-0.08215	0.415794	-0.04451	0.654335	-0.20196	0.067911	0.157443	0.138661	-0.03763	0.70449	0.119809	0.246098	6787019	9.691294	9.606702	9.631194	9.610272	9.681557	9.750265	9.658766	9.474792	9.436206	9.711452	9.54796	9.916222	4933407N	NM_025745
6787020	2482	6.13585	6.090663	6.073554	6.206473	-0.07062	0.684518	-0.11581	0.509045	-0.13292	0.450534	0.017109	0.921191	0.045187	0.794245	0.062296	0.719724	6787020	6.070029	6.041682	6.295839	6.088356	6.314666	5.868967	6.246207	6.20172	5.772736	6.021759	6.224946	6.372714	Chac2	NM_026527
6787035	2483	7.659115	8.525388	8.249102	7.233106	0.426009	0.16248	1.292282	0.001609	1.015996	0.006316	0.276286	0.347595	-0.86627	0.014046	-0.58999	0.065718	6787035	7.460783	8.046594	7.469969	8.357947	8.580984	8.637233	7.882753	8.254572	8.609982	6.973609	7.740791	6.984918	Stc2	NM_011491
6787100	2485	8.025849	8.21363	7.991607	7.865309	0.16054	0.195085	0.348321	0.015398	0.126298	0.298283	0.222023	0.086258	-0.18778	0.136747	0.034242	0.770654	6787100	7.9393	8.03478	8.103468	8.246673	8.368555	8.025663	8.009591	8.08095	7.88428	7.675697	8.018929	7.901302	Rhbdf1	NM_010117
6787102	2486	7.562462	7.603289	7.587362	7.651752	-0.08929	0.297364	-0.04846	0.561949	-0.06439	0.444728	0.015927	0.847357	-0.04083	0.624049	-0.0249	0.763874	6787102	7.654429	7.500702	7.532256	7.597587	7.729829	7.482451	7.630787	7.679316	7.451982	7.615489	7.705455	7.634313	Mare	NM_181569
6787174	2488	6.881708	6.900798	6.640815	6.768634	0.113074	0.493252	0.132164	0.42578	-0.12782	0.440548	0.259983	0.137427	-0.01909	0.906523	0.240894	0.164692	6787174	6.622758	7.031648	6.990719	6.805211	7.119776	6.777407	6.781581	6.738673	6.40219	6.617858	6.886091	6.801954	Fgf18	NM_008005
6787196	2491	7.067911	7.620431	5.764092	5.838638	1.229273	0.204341	1.781793	0.080128	-0.07455	0.93527	1.856339	0.070353	-0.55252	0.551783	1.303819	0.180878	6787196	6.270744	6.803488	8.129502	7.445731	8.98655	6.429013	6.299588	4.781412	6.211276	6.942885	6.016108	4.556921	Gabrp	NM_146017
6787230	2493	7.353275	7.050638	6.941901	6.600128	0.753147	0.021934	0.45051	0.128205	0.341773	0.23404	0.108737	0.692914	0.302637	0.287368	0.411374	0.159917	6787230	7.224357	6.94074	7.202036	7.115816	7.207896	6.828202	7.341566	6.841186	6.642951	6.593483	6.722494	6.484406	Foxi1	NM_023907
6787283	2495	9.246264	9.194121	9.37924	9.020814	0.22545	0.067014	0.173307	0.142114	0.358426	0.009821	-0.18512	0.12018	0.052142	0.637358	-0.13298	0.246848	6787283	9.219383	9.244858	9.27455	9.023541	9.381303	9.17752	9.489608	9.344778	9.303333	8.867755	9.186045	9.008642	Rars	NM_025936
6787285	2496	9.039752	9.130516	9.0242	9.314857	-0.27511	0.120886	-0.18434	0.278401	-0.29066	0.104077	0.106315	0.52133	-0.09076	0.582678	0.015551	0.924266	6787285	9.015945	8.974821	9.128489	9.250814	9.17832	8.962413	8.898822	9.194704	8.979075	9.195944	9.077789	9.670839	Wwc1	NM_170779
6787465	2498	8.301097	8.456201	8.289316	8.386431	-0.08533	0.406508	0.06977	0.494173	-0.09712	0.347932	0.166885	0.124994	-0.1551	0.149955	0.011781	0.906708	6787465	8.233316	8.287003	8.382972	8.409749	8.556893	8.401961	8.418493	8.359786	8.089668	8.280439	8.3733	8.505554	Mat2b	NM_134017
6787474	2500	10.65419	10.49422	10.53555	10.54183	0.112353	0.463387	-0.04761	0.752555	-0.00628	0.966704	-0.04133	0.784173	0.159963	0.30481	0.118637	0.439665	6787474	10.75715	10.56436	10.64105	10.50231	10.59714	10.38322	10.69475	10.57448	10.33742	10.2444	10.60452	10.77658	Ccng1	NM_009831
6787585	2504	7.535849	8.056789	8.019334	6.819385	0.716464	0.073495	1.237404	0.007446	1.199949	0.008719	0.037455	0.916939	-0.52094	0.172776	-0.48348	0.202179	6787585	7.854433	7.348351	7.404764	8.158322	7.991068	8.020977	7.953497	8.360737	7.743767	6.319125	7.665867	6.473163	Pttg1	NM_013917
6787610	2506	8.137947	8.164247	8.102921	8.174758	-0.03681	0.72448	-0.01051	0.919531	-0.07184	0.496379	0.061326	0.559865	-0.0263	0.800782	0.035026	0.737239	6787610	8.166811	8.20922	8.037809	8.062128	8.142962	8.287651	8.142797	8.154675	8.01129	8.039331	8.101503	8.38344	Ttc1	NM_133795
6787614	2507	7.493492	7.477982	7.454244	7.499647	-0.00615	0.97248	-0.02166	0.903391	-0.0454	0.799527	0.023738	0.894206	0.01551	0.930737	0.039248	0.826143	6787614	7.758711	7.26969	7.452076	7.552155	7.670608	7.211184	7.476379	7.455309	7.431044	7.700215	7.574776	7.22395	Adra1b	NM_007416
6787743	2510	6.573883	6.66611	6.680469	6.154555	0.419329	0.146868	0.511555	0.085708	0.525914	0.078711	-0.01436	0.957482	-0.09223	0.732997	-0.10659	0.693763	6787743	6.829916	6.624782	6.266952	6.82053	6.865522	6.312277	6.841541	6.385954	6.813912	6.104754	6.587119	5.771791	Cyfip2	NM_133769
6787830	2514	5.937482	5.700606	5.669591	5.752211	0.185271	0.571142	-0.05161	0.873431	-0.08262	0.798946	0.031015	0.923685	0.236876	0.471887	0.267891	0.418015	6787830	5.592854	5.88451	6.335083	6.163752	5.641922	5.296145	6.09252	5.602921	5.313333	5.425195	5.758614	6.072825	Sgcd	NM_011891
6787889	2515	8.303014	8.323026	8.094743	8.16772	0.135294	0.200596	0.155306	0.14803	-0.07298	0.473407	0.228283	0.046435	-0.02001	0.841706	0.208271	0.064065	6787889	8.387218	8.315023	8.206801	8.377267	8.320923	8.270888	7.860184	8.258759	8.165285	8.142188	8.218451	8.14252	Trim41	NM_145377
6787896	2516	7.956896	8.047651	7.950487	7.554404	0.402492	0.092227	0.493247	0.047173	0.396083	0.096655	0.097165	0.656658	-0.09076	0.677739	0.00641	0.976454	6787896	7.825557	8.10544	7.939692	7.792205	8.574347	7.776402	8.047745	7.798359	8.005356	7.511615	7.712698	7.438899	Irgm1	NM_008326
6787909	2517	6.852571	6.407649	6.685611	5.744957	1.107614	0.014095	0.662692	0.098337	0.940654	0.029029	-0.27796	0.455318	0.444922	0.244623	0.16696	0.650047	6787909	6.834704	7.016737	6.706271	6.106581	6.566329	6.550037	7.182316	5.792883	7.081633	5.586142	6.028289	5.62044	Tgtp	NM_011579
6787918	2518	8.158722	7.815967	8.172837	7.252177	0.906544	0.006989	0.56379	0.055556	0.92066	0.006444	-0.35687	0.194247	0.342755	0.210647	-0.01412	0.95668	6787918	8.004611	8.372303	8.099251	7.751766	7.963645	7.73249	8.527284	7.973629	8.017599	7.136224	7.782291	6.838017	Psme2	NM_011190
6787931	2519	7.088654	6.482175	6.505404	5.038537	2.050317	0.018587	1.443838	0.071849	1.467067	0.068217	-0.02323	0.974206	0.60648	0.409169	0.58325	0.426588	6787931	6.744934	7.31852	7.202509	5.695511	7.115991	6.635022	7.131514	5.040971	7.343728	4.307589	5.928411	4.879218	Tgtp	NM_011579
6787947	2521	8.021193	8.078896	7.775685	7.918075	0.103118	0.737465	0.160821	0.603016	-0.14239	0.64455	0.303212	0.337291	-0.0577	0.850833	0.245509	0.432525	6787947	7.8658	8.402183	7.795597	8.073393	7.941468	8.221828	7.909727	7.313362	8.103965	7.39964	8.345937	8.008649	Btnl9	NM_172793
6787967	2522	8.858387	8.993724	8.787701	8.769297	0.08909	0.369495	0.224427	0.043487	0.018403	0.849174	0.206024	0.059087	-0.13534	0.186594	0.070687	0.472188	6787967	8.898628	8.849941	8.826593	8.822649	9.227015	8.931509	8.829799	8.787457	8.745846	8.721678	8.856924	8.72929	Cnot6	NM_212484
6788014	2523	10.84974	10.79295	10.7441	10.89347	-0.04373	0.422579	-0.10053	0.087959	-0.14937	0.020296	0.048847	0.372781	0.056797	0.304277	0.105643	0.075471	6788014	10.88964	10.83726	10.82233	10.86055	10.721	10.79729	10.76031	10.71078	10.76121	10.87444	10.80904	10.99694	Sqstm1	NM_011018
6788017	2524	7.783145	7.882471	7.932514	7.986953	-0.20381	0.570127	-0.10448	0.769218	-0.05444	0.878253	-0.05004	0.888	-0.09933	0.780249	-0.14937	0.675787	6788017	7.784135	7.807539	7.757761	8.16371	7.794466	7.689236	7.631068	8.340512	7.825963	7.556134	7.590312	8.814412	Ltc4s	NM_008521
6788019	2525	7.097055	7.105592	7.0585	7.070606	0.026449	0.815711	0.034986	0.758175	-0.01211	0.914929	0.047092	0.679336	-0.00854	0.939941	0.038555	0.734565	6788019	7.148653	7.191829	6.950684	7.21894	7.133901	6.963935	6.881096	7.247384	7.047021	7.004672	7.148849	7.058297	Maml1	NM_175334
6788020	2526	11.60002	11.56769	11.72848	11.57192	0.0281	0.767184	-0.00423	0.964352	0.15656	0.126274	-0.16079	0.117736	0.03233	0.733622	-0.12846	0.198982	6788020	11.67966	11.62146	11.49895	11.45555	11.51682	11.73071	11.85748	11.67291	11.65506	11.51976	11.67871	11.5173	Canx	NM_007597
6788025	2527	7.864257	7.863873	7.96419	7.907086	-0.04283	0.494295	-0.04321	0.490553	0.057104	0.367642	-0.10032	0.132004	0.000384	0.995039	-0.09993	0.133288	6788025	7.826483	7.884213	7.882074	7.89446	7.802445	7.894714	8.027952	7.949861	7.914756	7.77641	7.935056	8.009791	Rufy1	NM_172557
6788054	2528	6.485188	6.730699	6.705315	6.551537	-0.06635	0.55362	0.179162	0.133597	0.153777	0.189802	0.025385	0.81897	-0.24551	0.051459	-0.22013	0.074386	6788054	6.410989	6.553826	6.490748	6.638327	6.987813	6.565958	6.798245	6.607021	6.710678	6.596579	6.578128	6.479905	Zfp354c	NM_013922
6788120	2536	10.58281	10.58376	10.55154	10.49027	0.092533	0.366629	0.09349	0.361953	0.061267	0.54404	0.032223	0.747525	-0.00096	0.992349	0.031267	0.754729	6788120	10.5964	10.53742	10.6146	10.4322	10.72507	10.59402	10.69088	10.46136	10.50238	10.34101	10.60044	10.52937	Hnrnpab	NM_001048061
6788122	2537	8.226234	8.397625	8.325599	8.464158	-0.23792	0.041552	-0.06653	0.516895	-0.13856	0.195647	0.072026	0.483913	-0.17139	0.118851	-0.09937	0.340899	6788122	8.019794	8.290244	8.368664	8.319674	8.492031	8.38117	8.415876	8.349904	8.211018	8.398308	8.447487	8.546678	Rmnd5b	NM_025346
6788128	2538	6.922523	6.946263	6.92684	7.026291	-0.10377	0.31203	-0.08003	0.429452	-0.09945	0.331314	0.019424	0.844976	-0.02374	0.811233	-0.00432	0.9653	6788128	6.784444	6.848536	7.13459	6.989216	6.878215	6.971359	6.878181	7.033462	6.868876	6.920497	7.063744	7.094633	C330016O	NM_145974
6788133	2539	7.449138	7.550533	7.743417	7.693002	-0.24386	0.036266	-0.14247	0.18043	0.050415	0.617615	-0.19288	0.082185	-0.1014	0.326832	-0.29428	0.016281	6788133	7.247066	7.55691	7.543437	7.611281	7.51426	7.526057	7.811678	7.840768	7.577806	7.640306	7.704655	7.734045	0610009B	NM_025319
6788134	2540	8.596945	8.460814	8.616392	8.684655	-0.08771	0.705664	-0.22384	0.34701	-0.06826	0.768383	-0.15558	0.507091	0.136132	0.560296	-0.01945	0.932967	6788134	8.559537	8.610507	8.620792	8.417739	8.614202	8.3505	8.31145	9.188368	8.349357	8.757246	8.826257	8.470461	Sec24a	NM_175255
6788141	2541	7.955067	7.881951	7.861759	8.117484	-0.16242	0.23228	-0.23553	0.097763	-0.25573	0.076269	0.020192	0.876329	0.073116	0.576709	0.093308	0.479015	6788141	7.775602	8.10343	7.986168	8.043624	7.743011	7.859218	7.808172	7.870718	7.906387	8.1038	7.920446	8.328207	Phf15	NM_199299
6788147	2542	9.730461	9.746418	9.698036	9.896514	-0.16605	0.128538	-0.1501	0.164048	-0.19848	0.077349	0.048382	0.634688	-0.01596	0.874659	0.032426	0.749168	6788147	9.616559	9.765033	9.809792	9.68212	9.772998	9.784136	9.791344	9.720613	9.58215	9.818458	9.767213	10.10387	Ube2b	NM_009458
6788160	2543	7.308513	7.207563	6.7925	6.739337	0.569176	0.153437	0.468226	0.230676	0.053163	0.886538	0.415062	0.283346	0.10095	0.786798	0.516013	0.19066	6788160	7.484869	6.907684	7.532986	7.508428	7.570264	6.543996	6.96873	6.993412	6.415359	7.179732	6.241667	6.796612	Olfr1372	BC055827
6788172	2544	6.804077	6.800946	6.77482	6.594469	0.209607	0.08678	0.206477	0.090774	0.180351	0.13163	0.026126	0.813935	0.003131	0.97746	0.029256	0.792216	6788172	6.90387	6.767591	6.740769	6.826055	6.805894	6.770889	6.750428	6.785598	6.788435	6.77643	6.691916	6.315062	Tcf7	NM_009331
6788264	2545	9.720591	9.714605	9.754361	9.827328	-0.10674	0.282269	-0.11272	0.258121	-0.07297	0.453367	-0.03976	0.678978	0.005986	0.950041	-0.03377	0.724766	6788264	9.720877	9.709835	9.73106	9.534783	9.859879	9.749153	9.793791	9.854089	9.615202	9.927975	9.808253	9.745756	Hspa4	NM_008300
6788274	2547	8.959787	9.145732	8.984961	9.196651	-0.23686	0.089983	-0.05092	0.689408	-0.21169	0.123148	0.160771	0.226977	-0.18594	0.168581	-0.02517	0.842766	6788274	8.876628	8.983256	9.019477	9.278745	9.032935	9.125516	9.144477	9.082014	8.728391	9.115201	9.11779	9.356962	Uqcrq	NM_025352
6788283	2549	6.018777	5.914227	5.866356	6.202019	-0.18324	0.231948	-0.28779	0.076685	-0.33566	0.045292	0.047871	0.74413	0.10455	0.481605	0.15242	0.313347	6788283	6.146029	5.932471	5.97783	6.120292	5.88921	5.733179	5.778773	6.083976	5.73632	6.197956	6.02008	6.38802	Ankrd43	NM_183173
6788291	2551	5.889003	5.989959	6.011743	6.019015	-0.13001	0.329207	-0.02906	0.822215	-0.00727	0.955083	-0.02178	0.866126	-0.10096	0.44313	-0.12274	0.355402	6788291	6.129226	5.783171	5.754612	5.907959	5.955991	6.105926	6.025991	6.075785	5.933453	6.024673	5.830258	6.202115	Il13	NM_008355
6788293	2552	8.349495	8.46762	8.327484	8.02066	0.328835	0.007686	0.44696	0.001349	0.306823	0.010899	0.140136	0.170462	-0.11813	0.239933	0.022011	0.818939	6788293	8.20418	8.376206	8.468099	8.369658	8.575798	8.457404	8.489882	8.238096	8.254473	7.955478	8.037084	8.069419	Rad50	NM_009012
6788301	2554	7.313144	7.173221	7.202438	6.925293	0.387851	0.004079	0.247928	0.034593	0.277146	0.021752	-0.02922	0.772126	0.139923	0.189277	0.110705	0.289178	6788301	7.372082	7.290999	7.27635	7.068336	7.341495	7.109831	7.35349	7.163607	7.090218	6.787478	7.000284	6.988116	Slc22a5	NM_011396
6788303	2555	9.089745	8.893861	9.218329	8.004392	1.085353	0.001211	0.889469	0.003913	1.213938	0.000596	-0.32447	0.181996	0.195884	0.403314	-0.12858	0.578388	6788303	9.345589	8.977366	8.94628	9.00052	8.831056	8.850006	9.232656	8.996218	9.426114	7.807729	8.505692	7.699754	Slc22a21	NM_019723
6788312	2556	6.059531	6.019411	6.105125	5.768969	0.290562	0.019096	0.250441	0.035668	0.336155	0.009558	-0.08571	0.413083	0.04012	0.696722	-0.04559	0.658286	6788312	5.942967	6.099412	6.136214	5.995592	6.008829	6.053811	6.220668	5.921251	6.173455	5.856939	5.851327	5.598642	Slc22a4	NM_019687
6788314	2557	6.833582	6.76764	6.818194	6.528268	0.305314	0.05287	0.239372	0.112965	0.289926	0.0632	-0.05055	0.71676	0.065942	0.637063	0.015389	0.911719	6788314	6.897367	6.799911	6.803469	6.787523	6.977965	6.537432	7.003588	6.643311	6.807682	6.696124	6.498971	6.389709	Pdlim4	NM_019417
6788368	2560	10.45616	10.37933	10.42414	10.23467	0.22149	0.089405	0.144657	0.2425	0.189463	0.136955	-0.04481	0.706079	0.076833	0.521544	0.032027	0.787024	6788368	10.50878	10.27852	10.58119	10.20471	10.57456	10.35872	10.57001	10.37741	10.32499	10.28896	10.22952	10.18554	Cdc42se2	NM_178626
6788380	2562	8.338056	8.206614	8.382415	8.084169	0.253887	0.039607	0.122445	0.270338	0.298246	0.020376	-0.1758	0.127522	0.131442	0.239382	-0.04436	0.679247	6788380	8.366615	8.401979	8.245575	8.312301	8.25596	8.051582	8.399614	8.376945	8.370687	7.887671	8.279451	8.085386	Tnip1	NM_021327
6788388	2563	11.04921	10.58224	10.88461	10.5094	0.53981	0.070405	0.072837	0.785452	0.375203	0.185045	-0.30237	0.27617	0.466973	0.108731	0.164607	0.542396	6788388	10.90125	10.92062	11.32577	10.34135	10.70342	10.70195	11.47482	10.40148	10.77752	10.49941	10.45153	10.57727	Anxa6	NM_013472
6788401	2566	6.040267	6.074133	5.918413	5.844089	0.196178	0.243432	0.230045	0.178014	0.074324	0.646061	0.15572	0.346791	-0.03387	0.833348	0.121854	0.456634	6788401	5.758237	6.09445	6.268114	6.083453	6.096863	6.042084	6.157722	5.818233	5.779284	5.632549	5.97661	5.923107	Slc36a2	NM_153170
6788410	2567	12.35713	12.3297	11.93535	12.09674	0.260387	0.181402	0.232957	0.226687	-0.1614	0.39075	0.394353	0.057458	0.02743	0.88127	0.421783	0.045167	6788410	12.42311	12.39789	12.25039	12.17125	12.61789	12.19996	12.09288	11.76778	11.94538	11.80763	12.06964	12.41296	Sparc	NM_009242
6788411	2568	9.585662	9.748867	9.759129	9.372362	0.2133	0.159006	0.376505	0.025393	0.386767	0.022628	-0.01026	0.942274	-0.16321	0.26878	-0.17347	0.242138	6788411	9.738342	9.439638	9.579006	9.760002	9.933388	9.553211	9.914295	9.683842	9.679249	9.215758	9.585387	9.315941	Atox1	NM_009720
6788507	2571	8.10681	8.238232	8.072542	8.140071	-0.03326	0.546384	0.098161	0.100131	-0.06753	0.236852	0.16569	0.01386	-0.13142	0.03761	0.034268	0.534596	6788507	8.058062	8.175785	8.086583	8.12559	8.290882	8.298223	8.076997	8.072782	8.067846	8.103621	8.207447	8.109145	Fam114a2	NM_026342
6788536	2572	6.346504	6.338246	6.407295	6.437789	-0.09128	0.478622	-0.09954	0.441137	-0.03049	0.810184	-0.06905	0.589408	0.008258	0.948045	-0.06079	0.63396	6788536	6.454009	6.291329	6.294174	6.323298	6.249266	6.442173	6.638992	6.340422	6.24247	6.29419	6.3902	6.628976	Hand1	ENSMUST000000
6788563	2573	7.634372	7.688248	7.651144	7.64952	-0.01515	0.867677	0.038728	0.671686	0.001624	0.985736	0.037104	0.684553	-0.05388	0.557595	-0.01677	0.853673	6788563	7.57073	7.556715	7.775671	7.596877	7.858119	7.609748	7.747945	7.590499	7.614988	7.59227	7.691272	7.665018	Gemin5	NM_172558
6788580	2574	7.161577	7.28079	7.323554	7.616242	-0.45467	0.077316	-0.33545	0.173299	-0.29269	0.228393	-0.04276	0.853606	-0.11921	0.609668	-0.16198	0.490953	6788580	7.21779	7.265146	7.001794	7.291321	7.085556	7.465492	6.882896	7.793342	7.294424	7.438276	7.826163	7.584288	Zfp672	NM_178761

6788594	2575	6.263505	5.960477	6.1058	6.062804	0.200701	0.311339	-0.10233	0.596696	0.042997	0.822726	-0.14532	0.456459	0.303028	0.141397	0.157705	0.420474	6788594	6.002678	6.387182	6.400655	6.038958	5.798442	6.044031	6.337269	5.893213	6.086919	6.061372	5.769214	6.357825	4930504O	BC048595
6788632	2581	7.127813	7.164915	7.255055	7.556869	-0.42906	0.031538	-0.39195	0.044779	-0.30181	0.104625	-0.09014	0.599582	-0.0371	0.827639	-0.12724	0.462562	6788632	6.952764	7.340654	7.09002	7.129093	7.221555	7.144098	6.894046	7.529971	7.341149	7.500287	7.699094	7.471227	Zfp39	NM_011758
6788635	2582	9.207161	9.212025	9.198135	9.237234	-0.03007	0.770997	-0.02521	0.807079	-0.0391	0.705647	0.01389	0.892823	-0.00486	0.96235	0.009026	0.930209	6788635	9.219887	9.176754	9.224842	9.217566	9.291118	9.127391	9.207996	9.41621	8.970199	9.199935	9.295528	9.21624	Rnf187	AB030190
6788651	2585	6.168277	5.964338	5.914909	5.971228	0.19705	0.666374	-0.00689	0.987899	-0.05632	0.901383	0.049429	0.913386	0.203939	0.655589	0.253368	0.58083	6788651	5.660372	5.676863	7.167597	6.56411	5.703494	5.62541	6.197168	5.983555	5.564005	5.751924	6.13657	6.025189	Obscn	BC060226
6788653	2586	5.967547	6.011716	5.798422	6.098735	-0.13119	0.09903	-0.08702	0.250928	-0.30031	0.002719	0.213294	0.016218	-0.04417	0.547371	0.169125	0.042811	6788653	6.040142	5.867856	5.994642	5.989143	5.926543	6.119462	5.767296	5.782203	5.845767	6.215267	6.049449	6.03149	A230051G	NM_173785
6788657	2589	8.14001	8.188247	8.139794	8.323024	-0.18323	0.264897	-0.13478	0.403064	-0.18323	0.264378	0.048453	0.759075	-0.04824	0.760108	0.000216	0.998907	6788657	8.186091	8.11711	8.116828	8.159413	8.009179	8.396148	8.103399	8.448894	7.867089	8.28852	8.457191	8.223361	Guk1	NM_008193
6788661	2590	10.1117	10.15823	10.36855	10.34396	-0.23226	0.143009	-0.18573	0.230215	0.024597	0.86771	-0.21032	0.179567	-0.04653	0.753253	-0.25685	0.110208	6788661	10.08201	10.3539	9.899194	10.17687	10.1865	10.11132	10.29469	10.56278	10.24819	10.16131	10.31352	10.55704	Arf1	NM_001130408
6788662	2591	5.899843	5.960099	5.951366	5.937641	-0.0378	0.626172	0.022457	0.771146	0.013724	0.858666	0.008733	0.909729	-0.06026	0.44277	-0.05152	0.509476	6788662	5.827663	5.892313	5.979552	5.925553	5.867933	6.08681	5.923345	6.040663	5.890089	5.881348	5.887681	6.043895	Wnt3a	NM_009522
6788672	2592	8.596075	8.770096	8.708234	8.745448	-0.14937	0.127403	0.024648	0.786104	-0.03721	0.682928	0.061862	0.501188	-0.17402	0.082866	-0.11216	0.2374	6788672	8.676387	8.533676	8.578162	8.825705	8.811242	8.673341	8.661156	8.844739	8.618806	8.628677	8.707788	8.899879	Snap47	NM_144521
6788674	2593	6.405198	6.542938	6.476908	6.399498	0.0057	0.938895	0.14344	0.081716	0.07741	0.314066	0.066029	0.386317	-0.13774	0.092339	-0.07171	0.348835	6788674	6.425592	6.348097	6.441906	6.473788	6.685509	6.469516	6.58117	6.375947	6.473608	6.371722	6.461155	6.365617	BC050078	NM_178417
6788676	2594	8.021377	8.146817	8.049431	7.937526	0.083852	0.185623	0.209292	0.006838	0.111905	0.089367	0.097387	0.131106	-0.12544	0.062176	-0.02805	0.641038	6788676	8.036972	8.006038	8.021122	8.285583	8.102417	8.052452	8.112799	8.057335	7.978158	7.924029	7.954132	7.934416	Zkscan17	NM_172941
6788701	2596	8.258194	8.40419	8.301015	8.227146	0.031048	0.709692	0.177045	0.058994	0.073869	0.385503	0.103176	0.235717	-0.146	0.107203	-0.04282	0.609113	6788701	8.16221	8.325998	8.286374	8.424929	8.299271	8.488371	8.351345	8.289143	8.262556	8.229915	8.083597	8.367925	Flcn	NM_146018
6788702	2597	8.582382	8.538853	8.609832	8.483222	0.099161	0.494117	0.055632	0.698263	0.12661	0.387074	-0.07098	0.621945	0.043529	0.761197	-0.02745	0.847744	6788702	8.387437	8.580759	8.778951	8.401511	8.647908	8.567141	8.757559	8.598957	8.47298	8.252405	8.622206	8.575054	Cops3	NM_011991
6788723	2598	7.285721	7.781613	7.948342	7.775186	-0.48947	0.336573	0.006426	0.98962	0.173156	0.726986	-0.16673	0.736662	-0.49589	0.33064	-0.66262	0.203772	6788723	6.046254	7.707029	8.10388	7.605156	7.68295	8.056732	7.745455	8.027147	8.072425	7.745253	8.096127	7.484179	Rasd1	NM_009026
6788725	2599	7.546142	7.958903	7.746338	8.831365	-1.28522	0.00692	-0.87246	0.040059	-1.08503	0.015954	0.212565	0.567372	-0.41276	0.280199	-0.2002	0.589686	6788725	7.266381	7.420614	7.951432	7.911198	8.046696	7.918816	7.783902	8.112745	7.342367	8.497215	8.370761	9.626118	Pemt	NM_008819
6788739	2601	8.882939	9.095624	9.073902	9.477386	-0.59445	0.028862	-0.38176	0.126117	-0.40348	0.10878	0.021722	0.924995	-0.21268	0.369325	-0.19096	0.417887	6788739	8.926277	8.744766	8.977774	9.148642	9.179189	8.95904	9.067835	9.388075	8.765796	9.298196	9.179926	9.954037	Srebf1	NM_011480
6788741	2602	9.218501	9.019782	9.22128	9.193988	0.024513	0.860519	-0.17421	0.233226	0.027292	0.844934	-0.2015	0.174141	0.198719	0.179486	-0.00278	0.984091	6788741	9.217278	9.238726	9.199499	9.137438	8.900187	9.021721	8.898218	9.477778	9.287844	9.150809	9.29518	9.135975	Tom1l2	NM_153080
6788743	2603	7.306487	7.40303	7.412341	7.475756	-0.16927	0.060668	-0.07273	0.375934	-0.06341	0.437323	-0.00931	0.90742	-0.09654	0.248522	-0.10585	0.209531	6788743	7.294866	7.254478	7.370117	7.395949	7.462782	7.35036	7.420676	7.446118	7.37023	7.319297	7.455941	7.652029	Atpaf2	NM_145427
6788784	2604	9.594601	9.408587	9.52259	9.284425	0.310177	0.012741	0.124162	0.237	0.238166	0.039818	-0.114	0.274285	0.186015	0.091819	0.072011	0.479675	6788784	9.720644	9.534631	9.528529	9.521803	9.413894	9.290063	9.474611	9.539628	9.553532	9.298238	9.448754	9.106282	Flii	NM_022009
6788789	2605	6.955656	6.969665	7.057779	6.820149	0.135507	0.048487	0.149516	0.033329	0.23763	0.003542	-0.08811	0.168911	-0.01401	0.816049	-0.10212	0.117745	6788789	6.9717	6.950996	6.944273	6.952896	7.020281	6.935817	7.20068	6.947218	7.02544	6.793896	6.804843	6.861708	Top3a	NM_009410
6788791	2606	6.633825	6.593783	6.378086	6.425215	0.20861	0.276568	0.168567	0.373033	-0.04713	0.798605	0.215697	0.261792	0.040043	0.828274	0.255739	0.190186	6788791	6.472003	6.485771	6.943702	6.370645	6.703274	6.707429	6.54003	6.087336	6.506892	6.486799	6.516429	6.269229	Shmt1	NM_009171
6788794	2607	9.140473	9.233957	9.118654	9.315016	-0.17454	0.199513	-0.08106	0.534234	-0.19636	0.15429	0.115304	0.382582	-0.09348	0.475284	0.021819	0.865561	6788794	9.041563	9.162175	9.217681	8.991954	9.446187	9.263731	9.022042	9.283207	9.050712	9.242149	9.258656	9.444242	Tmem11	NM_173453
6788796	2608	7.880253	7.929685	7.981678	7.970313	-0.09006	0.514405	-0.04063	0.766147	0.011365	0.933518	-0.05199	0.704009	-0.04943	0.717822	-0.10143	0.464421	6788796	7.850884	7.882529	7.907345	7.989148	8.005702	7.794206	8.037197	7.948489	7.959348	7.707514	7.912481	8.290945	Gtlf3b	NM_025294
6788812	2610	10.71057	10.68404	10.52648	10.58862	0.121943	0.184059	0.095417	0.288207	-0.06215	0.479908	0.157563	0.097122	0.026527	0.759896	0.18409	0.059473	6788812	10.63488	10.7159	10.78092	10.63361	10.80215	10.61636	10.63402	10.42517	10.52024	10.57801	10.71763	10.47023	Usp22	NM_001004143
6788815	2611	8.963141	9.044748	9.038859	8.81802	0.145121	0.110581	0.226728	0.023103	0.220839	0.025851	0.005889	0.943752	-0.08161	0.342616	-0.07572	0.376675	6788815	9.052109	9.060642	8.776671	9.045565	9.033631	9.055048	9.12936	9.039119	8.948097	8.80472	8.893284	8.756055	Aldh3a2	NM_007437
6788838	2614	7.32991	7.129915	7.099355	7.113411	0.216499	0.100586	0.016504	0.890998	-0.01406	0.907072	0.03056	0.799978	0.199995	0.124825	0.230555	0.083536	6788838	7.438312	7.332342	7.219076	7.195518	7.133891	7.060337	6.980046	7.143571	7.174448	7.142873	7.329975	6.867385	Mapk7	NM_011841
6788851	2615	8.240942	8.414968	8.368789	8.254835	-0.01389	0.88782	0.160133	0.131776	0.113954	0.266522	0.046179	0.641337	-0.17403	0.105591	-0.12785	0.217042	6788851	8.36392	8.141662	8.217243	8.349356	8.574499	8.32105	8.444719	8.302943	8.358706	8.124475	8.251402	8.388628	Epn2	NM_010148
6788862	2616	8.27215	8.304834	8.239024	8.147163	0.124988	0.169573	0.157672	0.093331	0.091862	0.299434	0.06581	0.449622	-0.03268	0.703329	0.033126	0.699548	6788862	8.248714	8.261794	8.305943	8.339261	8.377915	8.197327	8.337904	8.336937	8.042232	8.146755	8.193636	8.101097	Ulk2	NM_013881
6788866	2617	8.085609	8.133409	8.108921	8.124812	-0.0392	0.546761	0.008597	0.893666	-0.01589	0.805129	0.024487	0.704572	-0.0478	0.465011	-0.02331	0.718005	6788866	8.06146	8.178415	8.016951	8.107071	8.178893	8.114262	8.017325	8.223513	8.085926	8.057779	8.177763	8.138893	Akap10	NM_019921
6788903	2618	10.17362	10.17264	10.11788	10.11088	0.062733	0.231848	0.061753	0.238577	0.006997	0.888837	0.054757	0.291511	0.00098	0.98437	0.055737	0.283565	6788903	10.20572	10.17171	10.14342	10.16864	10.19352	10.15575	10.08898	10.19403	10.07063	10.05397	10.21617	10.06251	Ncor1	NM_011308
6788921	2620	7.378617	7.180916	7.272197	7.15459	0.224027	0.187709	0.026326	0.869786	0.117607	0.471219	-0.09128	0.573449	0.197701	0.239382	0.10642	0.513145	6788921	7.293874	7.163308	7.678668	7.136149	7.301032	7.105567	7.271461	7.479339	7.06579	7.030354	7.305929	7.127487	BC046404	BC053455
6788928	2621	6.02882	5.958738	6.005412	5.897474	0.131345	0.234741	0.061264	0.565507	0.107937	0.321811	-0.04667	0.660081	0.070081	0.512316	0.023408	0.824606	6788928	6.046435	6.120639	5.919385	5.976641	6.126545	5.773029	6.081647	5.921933	6.012655	5.860869	6.031628	5.799926	Zfp287	NM_133208
6788991	2623	6.746556	6.787102	6.611494	6.705042	0.041514	0.801416	0.082061	0.621162	-0.09355	0.574083	0.175608	0.303371	-0.04055	0.805935	0.135062	0.422167	6788991	6.741396	6.579353	6.918919	6.999102	6.892412	6.469793	6.736073	6.72384	6.374569	6.774311	6.668311	6.672503	Hs3st3b1	NM_018805
6788993	2624	7.585846	7.553948	7.488336	7.627698	-0.04185	0.657926	-0.07375	0.441271	-0.13936	0.164311	0.065612	0.491563	0.031898	0.735074	0.09751	0.315328	6788993	7.591137	7.459729	7.706673	7.623124	7.654377	7.384344	7.467104	7.505697	7.492207	7.500801	7.704262	7.67803	Cox10	NM_178379
6789047	2625	7.59915	7.835627	7.489781	7.971255	-0.3721	0.099416	-0.13563	0.516192	-0.48147	0.042439	0.345846	0.121536	-0.23648	0.270326	0.10937	0.598853	6789047	7.491044	7.643255	7.663152	7.901443	7.796279	7.809159	7.48267	7.707912	7.27876	8.186599	7.481151	8.246014	AU040829	NM_175003
6789066	2627	9.259314	9.114396	9.071767	8.839829	0.419485	0.019679	0.274567	0.093574	0.231938	0.146651	0.042628	0.775201	0.144918	0.344653	0.187546	0.229907	6789066	9.213939	9.235427	9.328575	8.938734	9.441888	8.962565	9.276901	8.904486	9.033915	8.775444	8.914292	8.829751	Map2k4	NM_009157
6789119	2628	6.906227	7.017195	7.165664	7.11498	-0.20875	0.25046	-0.09778	0.577637	0.050684	0.771217	-0.14847	0.403904	-0.11097	0.52863	-0.25944	0.162165	6789119	6.916532	7.150906	6.651243	7.073891	6.99222	6.985475	6.879717	7.486135	7.13114	7.123981	7.222339	6.998619	2310004I	NM_025510
6789166	2630	6.162076	6.285571	6.265363	6.047615	0.114461	0.254436	0.237956	0.034046	0.217748	0.047738	0.020208	0.833819	-0.1235	0.221871	-0.10329	0.3001	6789166	6.209227	6.090074	6.186927	6.231225	6.473054	6.152435	6.360494	6.273348	6.162247	5.961273	6.021815	6.159758	Usp43	NM_173754
6789197	2632	7.112212	7.54226	7.024342	7.322311	-0.2101	0.338298	0.219949	0.317477	-0.29797	0.186649	0.517918	0.036345	-0.43005	0.070616	0.08787	0.681392	6789197	7.220291	7.261293	6.855051	7.41776	7.854281	7.354738	7.096227	7.145592	6.831207	7.210273	7.683846	7.072813	Ntn1	NM_008744
6789229	2633	8.599677	8.618258	8.587377	8.574881	0.024796	0.847876	0.043377	0.737817	0.012496	0.922916	0.030881	0.811305	-0.01858	0.88564	0.0123	0.924122	6789229	8.645279	8.593638	8.560115	8.467237	8.786963	8.600575	8.564142	8.728841	8.469149	8.593054	8.786751	8.344838	Ndel1	NM_023668
6789235	2635	7.376443	7.451655	7.275866	7.376994	-0.00055	0.994013	0.074661	0.324554	-0.10113	0.192897	0.175789	0.038628	-0.07521	0.321217	0.100577	0.19508	6789235	7.454067	7.390755	7.284507	7.430567	7.539038	7.38536	7.230149	7.357889	7.23956	7.25369	7.456172	7.421119	Arhgef15	NM_177566
6789268	2636	6.169648	6.175266	6.157051	6.018733	0.150915	0.353061	0.156533	0.336424	0.138318	0.392603	0.018215	0.908215	-0.00562	0.971623	0.012597	0.936437	6789268	6.201593	5.972328	6.335023	6.437419	6.081865	6.006514	5.941204	6.38784	6.14211	6.031074	5.950735	6.074391	Alox8	NM_009661
6789270	2638	6.764651	6.864449	6.794219	6.757619	0.007032	0.910607	0.10683	0.116362	0.0366	0.563125	0.070231	0.280517	-0.0998	0.138673	-0.02957	0.639147	6789270	6.704254	6.821916	6.767782	6.778133	6.994958	6.820257	6.818398	6.744711	6.819547	6.79272	6.688637	6.7915	Cntrob	NM_172560
6789274	2639	8.644192	8.735549	8.545458	8.703965	-0.05977	0.757676	0.031584	0.870205	-0.15851	0.421751	0.190091	0.339615	-0.09136	0.638629	0.098734	0.612206	6789274	8.768349	8.687873	8.476355	8.78656	8.823317	8.596771	8.310494	9.021787	8.304094	8.713576	8.749018	8.649302	Chd3	NM_146019
6789279	2640	8.348121	8.17713	8.190886	7.920877	0.427244	0.034511	0.256253	0.165605	0.270009	0.146597	-0.01376	0.93674	0.170991	0.338457	0.157235	0.376597	6789279	8.525213	8.446944	8.072205	8.151098	8.199247	8.181045	8.260732	8.025488	8.286437	7.577315	8.063707	8.121608	Tmem88	NM_025915
6789311	2643	6.644226	6.696111	6.580533	6.505012	0.139214	0.138215	0.191099	0.053675	0.075521	0.397741	0.115578	0.208739	-0.05188	0.556417	0.063693	0.472775	6789311	6.627963	6.583552	6.721163	6.636553	6.737131	6.714649	6.746577	6.593374	6.401649	6.56891	6.520275	6.425852	Wrap53	NM_144824
6789316	2644	7.689191	7.403284	7.256092	7.326015	0.363176	0.127332	0.077269	0.726774	-0.06992	0.751677	0.147193	0.510033	0.285907	0.217303	0.433099	0.077016	6789316	7.435508	7.502709	8.129356	7.368279	7.437226	7.404348	7.310889	7.372479	7.084907	6.955849	7.499033	7.523163	Atp1b2	NM_013415
6789324	2646	10.40492	10.51184	10.34054	10.54249	-0.13757	0.2266	-0.03064	0.777885	-0.20195	0.090714	0.171307	0.141516	-0.10693	0.338428	0.06438	0.556901	6789324	10.38097	10.43363	10.40015	10.53636	10.43071	10.56846	10.25826	10.58981	10.17354	10.47518	10.48404	10.66824	Mpdu1	NM_011900
6789325	2647	10.2524	9.990684	10.09769	9.082578	1.169819	0.01275	0.908107	0.038201	1.015114	0.024276	-0.10701	0.777675	0.261712	0.495356	0.154705	0.68397	6789325	10.48952	10.16347	10.1042	10.03967	10.30485	9.627533	10.25464	9.490804	10.54763	9.278409	9.549877	8.419447	Cd68	NM_009853
6789328	2648	6.54231	6.624629	6.449368	6.477042	0.065268	0.667726	0.147587	0.343186	-0.02767	0.854865	0.17526	0.265781	-0.08232	0.58954	0.092942	0.543485	6789328	6.658772	6.489177	6.478981	6.428468	6.897069	6.548349	6.569227	6.451868	6.32701	6.281146	6.700834	6.449145	Senp3	NM_030702
6789329	2649	8.023909	7.992159	7.852587	7.488112	0.535796	0.018257	0.504046	0.023893	0.364474	0.079176	0.139572	0.463412	0.03175	0.865301	0.171322	0.372228	6789329	8.177172	7.970721	7.923833	7.920636	8.140678	7.915162	8.112934	7.64607	7.798756	7.402194	7.847621	7.214522	Tnfsf13	NM_023517
6789331	2650	7.94155	7.795176	7.806889	7.52183	0.419721	0.008796	0.273346	0.055277	0.285059	0.047581	-0.01171	0.925839	0.146374	0.264314	0.134662	0.301543	6789331	8.004063	8.006078	7.81451	7.786482	7.923262	7.675784	7.949847	7.761341	7.709478	7.564394	7.711209	7.289886	Tnfsf12-	NM_001034097
6789338	2651	9.27963	9.349817	9.161325	9.13996	0.13967	0.307836	0.209857	0.140401	0.021365	0.871825	0.188492	0.179822	-0.07019	0.599114	0.118305	0.383254	6789338	9.300323	9.320884	9.217683	9.389284	9.383197	9.27697	9.065955	9.492361	8.925659	9.063028	9.165744	9.191109	Polr2a	NM_009089
6789343	2653	9.08313	8.944534	8.911731	8.792875	0.290255	0.115197	0.151659	0.382852	0.118856	0.489927	0.032803	0.846693	0.138596	0.423304	0.171399	0.327227	6789343	9.197824	8.984566	9.066999	8.901068	9.2304	8.702133	9.121405	8.69105	8.922737	8.856033	8.933774	8.588818	Chrnb1	NM_009601
6789344	2654	5.902392	5.916574	5.936118	6.420841	-0.51845	0.022099	-0.50427	0.024902	-0.48472	0.029378	-0.01954	0.917623	-0.01418	0.940166	-0.03373	0.858446	6789344	5.930149	5.804572	5.972456	5.747581	5.996313	6.005827	5.758678	6.39231	5.657365	6.548504	6.322178	6.39184	Fgf11	NM_010198
6789351	2656	7.968519	8.088424	7.808671	7.75209	0.216429	0.066163	0.336334	0.010787	0.056581	0.593481	0.279754	0.025115	-0.11991	0.272626	0.159848	0.154941	6789351	8.080006	7.953771	7.87178	8.093417	8.238504	7.933352	7.725493	7.971884	7.728635	7.818345	7.786661	7.651264	Nlgn2	NM_198862
6789355	2657	8.145388	8.023089	8.185888	8.571208	-0.42582	0.038851	-0.54812	0.013068	-0.38532	0.056036	-0.1628	0.373072	0.122299	0.498616	-0.0405	0.820337	6789355	8.090512	8.132983	8.21267	8.241821	7.827443	8.000003	8.054383	8.555775	7.947506	8.666102	8.384962	8.66256	Tnk1	NM_031880
6789358	2658	6.328861	6.240209	6.241447	6.186448	0.142413	0.492558	0.053761	0.792916	0.054999	0.78828	-0.00124	0.995164	0.088652	0.666295	0.087414	0.670628	6789358	6.407932	6.273498	6.305153	6.08552	6.470217	6.16489	6.465399	6.288243	5.9707	6.079123	6.583162	5.897059	Kctd11	NM_153143
6789360	2660	6.586158	6.776456	6.452591	6.522327	0.063831	0.617469	0.254129	0.072419	-0.06974	0.585896	0.323865	0.029904	-0.1903	0.160022	0.133567	0.308665	6789360	6.571093	6.53737	6.650011	6.97145	6.702665	6.655252	6.686053	6.327244	6.344475	6.610536	6.371596	6.584849	2810408A	BC069874
6789365	2661	7.469164	7.671175	7.483148	7.626546	-0.15738	0.215351	0.044629	0.712735	-0.1434	0.255078	0.188027	0.146613	-0.20201	0.122429	-0.01398	0.907785	6789365	7.398765	7.594059	7.414667	7.647964	7.603411	7.762149	7.393878	7.683383	7.372182	7.649162	7.797042	7.433434	Eif5a	NM_181582
6789367	2662	6.302196	6.136074	5.915851	6.108373	0.193823	0.77545	0.027701	0.967395	-0.19252	0.77691	0.220223	0.746048	0.166122	0.806721	0.386345	0.572646	6789367	5.498335	5.671067	7.737186	6.892973	6.038554	5.476694	6.477483	5.852065	5.418005	5.540878	6.448236	6.336004	Slc2a4	NM_009204

6789369	2663	8.417029	8.411093	8.345267	8.377133	0.039896	0.751365	0.03396	0.787202	-0.03187	0.79998	0.065827	0.60317	0.005936	0.962281	0.071762	0.571538	6789369	8.342813	8.321548	8.586726	8.365698	8.628263	8.239319	8.466375	8.386559	8.182866	8.301025	8.459923	8.370451	Rai12	NM_018740
6789378	2664	8.66075	8.757214	8.62675	8.815253	-0.1545	0.294492	-0.05804	0.684558	-0.1885	0.208304	0.130464	0.371241	-0.09646	0.503541	0.034	0.811232	6789378	8.556894	8.701754	8.723603	8.890517	8.598497	8.782628	8.687468	8.63918	8.553603	8.559386	8.769675	9.116697	Acadvl	NM_017366
6789395	2665	9.121491	9.009469	8.966576	8.684605	0.436887	0.127341	0.324864	0.241501	0.281971	0.304187	0.042893	0.871506	0.112023	0.674237	0.154915	0.563087	6789395	8.883363	9.696992	8.784119	9.052281	9.032324	8.943801	8.799132	9.339105	8.761491	8.505114	8.888192	8.660508	Slc16a13	NM_172371
6789397	2666	7.028411	7.581842	7.010247	7.089462	-0.06105	0.869527	0.49238	0.208519	-0.07921	0.831325	0.571595	0.150946	-0.55343	0.162716	0.018164	0.96099	6789397	6.943281	6.999842	7.142111	7.084423	8.045045	7.616057	7.009038	7.017716	7.003987	6.262149	7.355128	7.651108	Bcl6b	NM_007528
6789401	2667	8.677894	8.693072	8.645862	8.581755	0.096139	0.310349	0.111317	0.245232	0.064106	0.490749	0.047211	0.609279	-0.01518	0.868477	0.032032	0.727549	6789401	8.53086	8.709896	8.792926	8.686808	8.774942	8.617467	8.775062	8.665642	8.496881	8.541933	8.649436	8.553897	0610010K	NM_026757
6789406	2668	10.94089	10.66387	11.01146	11.13116	-0.19027	0.384545	-0.46729	0.053799	-0.1197	0.578734	-0.34759	0.131386	0.277017	0.217296	-0.07057	0.741752	6789406	11.03186	10.79737	10.99343	10.82843	10.75781	10.40537	10.67376	11.48932	10.8713	11.17542	11.02508	11.19297	Rnasek	NM_173742
6789407	2669	6.483039	6.122747	6.572615	6.027676	0.455363	0.084612	0.095071	0.691955	0.544939	0.046314	-0.44987	0.087776	0.360292	0.158066	-0.08958	0.708763	6789407	6.724416	6.415279	6.309422	6.144088	6.412615	5.811537	6.847562	6.215187	6.655095	5.842918	6.351154	5.888956	Alox12	NM_007440
6789409	2670	5.614191	5.93154	6.109547	5.168519	0.445672	0.47951	0.763021	0.239866	0.941028	0.155992	-0.17801	0.774606	-0.31735	0.611763	-0.49536	0.433646	6789409	5.59067	5.315732	5.936171	5.418178	7.049603	5.326839	7.161164	5.07222	6.095258	5.366238	5.169736	4.969584	Alox12e	NM_145684
6789419	2672	7.41147	7.258945	7.301902	7.206986	0.204484	0.05734	0.051959	0.588441	0.094916	0.333301	-0.04296	0.653646	0.152525	0.136606	0.109568	0.268684	6789419	7.42165	7.358504	7.454257	7.193574	7.425427	7.157835	7.23535	7.438498	7.231858	7.152339	7.340488	7.128132	Pelp1	NM_029231
6789420	2673	10.4607	10.13258	10.29295	9.220008	1.240688	0.002155	0.912567	0.011372	1.072943	0.004922	-0.16038	0.581412	0.328122	0.273619	0.167746	0.564531	6789420	10.73895	10.46311	10.18003	10.17289	10.30703	9.917805	10.64399	9.882693	10.35217	9.195701	9.682041	8.782283	Cxcl16	NM_023158
6789443	2675	9.798371	9.944769	9.773006	9.987785	-0.18941	0.337243	-0.04302	0.8225	-0.21478	0.280478	0.171762	0.381713	-0.1464	0.452892	0.025365	0.894653	6789443	9.699764	9.655629	10.03972	9.97118	10.02327	9.839856	9.950077	9.874559	9.494383	9.802122	9.819864	10.34137	Slc25a11	NM_024211
6789444	2676	11.79291	11.59549	11.78873	11.49586	0.297053	0.072374	0.099633	0.507416	0.292877	0.075718	-0.19324	0.215271	0.19742	0.206451	0.004177	0.977508	6789444	11.98957	11.75176	11.6374	11.53424	11.87676	11.37547	11.68648	11.82037	11.85935	11.50256	11.62497	11.36004	Pfn1	NM_011072
6789445	2677	7.134754	7.218045	7.113712	7.261853	-0.1271	0.082686	-0.04381	0.513636	-0.14814	0.049603	0.104334	0.142244	-0.08329	0.230001	0.021043	0.751127	6789445	7.042157	7.155476	7.20663	7.24093	7.228742	7.184464	7.036575	7.255062	7.049498	7.221277	7.302717	7.261566	Spag7	NM_172561
6789448	2678	8.109751	7.976969	8.00026	7.901648	0.208103	0.204922	0.07532	0.63089	0.098611	0.531503	-0.02329	0.88108	0.132783	0.404249	0.109492	0.488487	6789448	8.166502	8.034224	8.128528	8.065257	8.117538	7.748111	7.758491	8.31583	7.926458	7.957249	7.962041	7.785655	Camta2	NM_178116
6789471	2680	7.878175	8.03967	8.009178	7.876513	0.001662	0.990185	0.163157	0.248036	0.132665	0.340674	0.030492	0.821723	-0.1615	0.252488	-0.131	0.346413	6789471	7.847656	7.909548	7.87732	7.910375	8.22145	7.987185	8.19117	8.044804	7.791559	7.695661	8.068271	7.865606	Nup88	NM_172394
6789474	2681	9.201876	9.240946	9.140413	9.473851	-0.27197	0.055126	-0.23291	0.090948	-0.33344	0.025041	0.100533	0.430996	-0.03907	0.755505	0.061463	0.625832	6789474	9.194381	9.340637	9.070611	9.186326	9.226852	9.309659	9.102064	9.383224	8.935951	9.386666	9.421839	9.613049	C1qbp	NM_007573
6789475	2682	7.472767	7.503075	7.498478	7.458952	0.013814	0.869377	0.044123	0.602347	0.039526	0.640099	0.004597	0.956324	-0.03031	0.719158	-0.02571	0.760056	6789475	7.42538	7.50912	7.4838	7.368421	7.665757	7.475047	7.455474	7.544822	7.495138	7.418816	7.588391	7.36965	Dhx33	NM_178367
6789476	2683	7.636985	7.688888	7.807189	7.809484	-0.1725	0.194171	-0.1206	0.35081	-0.00229	0.985419	-0.1183	0.359554	-0.0519	0.681045	-0.1702	0.199558	6789476	7.61815	7.515151	7.777653	7.536297	7.876186	7.65418	7.797489	7.945201	7.678876	7.808806	7.964132	7.655513	Derl2	NM_033562
6789522	2687	6.932006	6.99926	6.950101	6.848787	0.083219	0.281924	0.150473	0.070448	0.101313	0.197771	0.049159	0.514786	-0.06725	0.378427	-0.01809	0.808253	6789522	6.916404	6.956956	6.922659	6.887931	7.170853	6.938996	6.944562	6.881963	7.023777	6.875395	6.884727	6.78624	4933427D	BC043106
6789525	2688	6.237547	6.404107	6.356019	6.335336	-0.09779	0.320877	0.068771	0.478056	0.020683	0.82851	0.048088	0.616902	-0.16656	0.10916	-0.11847	0.235747	6789525	6.245716	6.3231	6.143826	6.381299	6.390346	6.440675	6.236322	6.578115	6.25362	6.311622	6.415312	6.279073	Med31	NM_026068
6789531	2689	5.828842	6.147518	5.930801	6.248327	-0.41949	0.074335	-0.10081	0.635253	-0.31753	0.159058	0.216717	0.320156	-0.31868	0.157734	-0.10196	0.631457	6789531	5.808904	5.826134	5.851488	5.860632	6.309987	6.271935	5.726217	5.906772	6.159414	6.097989	6.067427	5.997565	Slc13a5	NM_001004148
6789538	2690	9.704921	10.10837	10.22919	9.779935	-0.07501	0.796551	0.328438	0.276781	0.449255	0.149064	-0.12082	0.679021	-0.40345	0.189576	-0.52427	0.099476	6789538	9.791945	9.948419	9.3744	9.939447	9.803093	10.58258	10.10221	10.3357	10.24966	10.03807	10.03803	9.263705	Tekt1	NM_011569
6789540	2691	10.1164	10.34411	10.56461	10.20595	-0.08955	0.539021	0.138157	0.35117	0.35866	0.033124	-0.2205	0.152745	-0.22771	0.14139	-0.44821	0.012392	6789540	9.989651	10.39279	9.96677	10.20532	10.43801	10.389	10.56021	10.5815	10.55213	9.99762	10.41781	10.20243	Smtnl2	NM_177776
6789541	2692	8.438025	8.33673	8.258575	7.998185	0.43984	0.150489	0.338545	0.255825	0.260391	0.37409	0.078155	0.784707	0.101295	0.723722	0.179449	0.534695	6789541	8.608355	8.251719	8.454	8.335655	8.762617	7.911918	8.610915	7.936898	8.227913	7.661757	8.38328	7.949517	Spns2	NM_153060
6789584	2695	9.218654	9.355669	9.049808	9.365349	-0.1467	0.222799	-0.00968	0.932642	-0.31554	0.02171	0.305861	0.024834	-0.13702	0.252039	0.168845	0.166675	6789584	9.185913	9.190988	9.27906	9.23105	9.332179	9.503778	9.199785	9.049997	8.899643	9.279255	9.253233	9.563559	Tmem93	NM_025318
6789588	2696	8.564207	8.70248	8.559305	8.534764	0.029443	0.697846	0.167716	0.05104	0.024542	0.745873	0.143174	0.086015	-0.13827	0.095382	0.004902	0.948218	6789588	8.646989	8.552801	8.492831	8.58777	8.772128	8.747541	8.508915	8.640643	8.528358	8.464337	8.484717	8.655237	Ctns	NM_031251
6789684	2715	9.547317	9.625011	9.499618	9.570378	-0.02306	0.831533	0.054633	0.616677	-0.07076	0.519061	0.125393	0.266269	-0.07769	0.480164	0.047698	0.661472	6789684	9.576807	9.556821	9.508322	9.587154	9.642468	9.645411	9.666122	9.543631	9.289102	9.395987	9.594837	9.720311	Pafah1b1	NM_013625
6789693	2716	7.909301	7.930817	7.830212	7.815608	0.093693	0.188783	0.115209	0.115314	0.014604	0.828438	0.100605	0.161524	-0.02152	0.749953	0.079089	0.259869	6789693	7.98584	7.851926	7.890136	7.995173	7.88442	7.912858	7.833559	7.93982	7.717256	7.789516	7.761173	7.896134	Sgsm2	NM_197943
6789696	2717	7.512637	7.595508	7.646254	7.72358	-0.21094	0.027142	-0.12807	0.139992	-0.07733	0.351557	-0.05075	0.534432	-0.08287	0.320057	-0.13362	0.125781	6789696	7.436407	7.604105	7.4974	7.587916	7.612033	7.586576	7.71178	7.719143	7.50784	7.605201	7.71671	7.848829	Srr	NM_013761
6789721	2719	7.591378	7.511009	7.657033	7.577626	0.013753	0.828556	-0.06662	0.310011	0.079408	0.232441	-0.14602	0.044838	0.080369	0.227331	-0.06565	0.316598	6789721	7.552528	7.551101	7.670506	7.474624	7.620391	7.438012	7.741581	7.666715	7.562804	7.556385	7.572051	7.604441	Dph1	NM_144491
6789725	2720	7.610232	7.719742	7.660867	7.340633	0.269599	0.078681	0.379109	0.022037	0.320234	0.04363	0.058875	0.671577	-0.10951	0.436807	-0.05063	0.714958	6789725	7.612002	7.569541	7.649153	7.63995	7.772782	7.746493	7.923245	7.387449	7.671907	7.173278	7.513863	7.334758	Rpa1	NM_026653
6789741	2721	11.35557	11.61302	11.40791	11.73542	-0.37984	0.013797	-0.1224	0.341185	-0.3275	0.026755	0.205103	0.128384	-0.25744	0.065954	-0.05234	0.676643	6789741	11.26897	11.48277	11.31498	11.65508	11.73839	11.44558	11.27217	11.57993	11.37164	11.55496	11.7685	11.88279	Serpinf1	NM_011340
6789789	2724	7.191976	7.048666	7.052435	7.227517	-0.03554	0.704703	-0.17885	0.083458	-0.17508	0.089009	-0.00377	0.967792	0.143309	0.151855	0.13954	0.161566	6789789	7.122226	7.2563	7.197401	6.975626	7.063731	7.106642	7.140634	7.049982	6.96669	7.40379	7.235622	7.043138	Rph3al	NM_029548
6789797	2725	7.862133	7.795621	7.85375	7.816069	0.046064	0.536901	-0.02045	0.781858	0.037681	0.612009	-0.05813	0.439139	0.066512	0.378839	0.008383	0.909433	6789797	7.865921	7.8978	7.822678	7.717089	7.899774	7.769999	7.970115	7.826694	7.764441	7.706526	7.885258	7.856424	Vps53	NM_026664
6789805	2726	7.174894	7.356369	7.01569	7.428098	-0.2532	0.106352	-0.07173	0.620207	-0.41241	0.018054	0.340679	0.040065	-0.18148	0.228506	0.159204	0.285715	6789805	7.104727	7.276743	7.143211	7.42776	7.175326	7.466021	7.08264	7.24568	6.718749	7.380505	7.358963	7.544825	Gemin4	NM_177367
6789808	2727	8.679932	8.810173	8.723285	8.612251	0.06768	0.564964	0.197921	0.117285	0.111034	0.353604	0.086887	0.463109	-0.13024	0.2814	-0.04335	0.710637	6789808	8.647214	8.637547	8.755034	8.731518	8.837646	8.861354	8.853793	8.489189	8.826874	8.624083	8.445512	8.767159	Glod4	NM_026029
6789813	2728	8.221337	8.264209	8.500568	8.378837	-0.1575	0.394397	-0.11463	0.53083	0.121731	0.506369	-0.23636	0.213769	-0.04287	0.812632	-0.27923	0.149238	6789813	8.166527	8.182701	8.314783	8.227084	8.438752	8.126792	8.916949	8.154403	8.430352	8.386724	8.426397	8.32339	Nxn	NM_008750
6789817	2729	8.229292	8.160363	8.316072	7.836127	0.393166	0.0205	0.324237	0.044867	0.479945	0.00789	-0.15571	0.286976	0.068929	0.627186	-0.08678	0.542691	6789817	8.273616	8.161418	8.252843	8.187037	8.320752	7.973301	8.414254	8.013784	8.520177	7.771779	7.923702	7.812899	Abr	NM_198018
6789825	2730	8.958585	9.054544	8.978259	9.027384	-0.0688	0.414976	0.02716	0.743055	-0.04912	0.556367	0.076285	0.368383	-0.09596	0.264805	-0.01967	0.811989	6789825	8.911122	8.960985	9.003647	8.970821	9.144894	9.047917	9.129547	8.821012	8.984219	8.973089	9.003174	9.105888	Gosr1	NM_016810
6789830	2731	9.705918	9.88508	9.702662	9.557037	0.148881	0.302256	0.328043	0.041235	0.145625	0.312252	0.182418	0.213665	-0.17916	0.221172	0.003256	0.981352	6789830	9.638556	9.702227	9.776971	9.843153	10.02888	9.783206	9.945981	9.473535	9.68847	9.349755	9.657875	9.66348	Cpd	NM_007754
6789849	2732	8.683149	8.731172	8.72204	8.731615	-0.04847	0.631121	-0.00044	0.996472	-0.00958	0.923872	0.009132	0.927382	-0.04802	0.634199	-0.03889	0.699258	6789849	8.625021	8.759924	8.664503	8.640049	8.811076	8.742392	8.779648	8.819433	8.567039	8.611742	8.668418	8.914686	Ccdc55	NM_001012309
6789863	2734	7.110053	7.1586	6.994936	7.088515	0.021538	0.771699	0.070084	0.3573	-0.09358	0.228435	0.163664	0.051994	-0.04855	0.517752	0.115118	0.1473	6789863	7.049024	7.088169	7.192967	7.216566	7.170562	7.088671	7.073877	7.082979	6.827951	7.105686	7.079684	7.080176	Ankrd13b	NM_172945
6789872	2735	9.072425	9.239674	9.052333	9.18232	-0.1099	0.397716	0.057353	0.65346	-0.12999	0.321489	0.187341	0.166256	-0.16725	0.211014	0.020092	0.874301	6789872	9.229278	9.153158	8.834839	9.100261	9.33886	9.2799	9.043777	9.130587	8.982635	9.028775	9.35044	9.167746	Taok1	NM_144825
6789875	2736	9.610479	9.703708	9.518706	9.541282	0.069197	0.327075	0.162427	0.039924	-0.02258	0.742206	0.185003	0.023531	-0.09323	0.19725	0.091773	0.203626	6789875	9.605968	9.669415	9.556053	9.670328	9.761602	9.679195	9.563471	9.565343	9.427303	9.417633	9.656699	9.549513	Taok1	NM_144825
6789930	2739	7.414756	7.445639	7.475841	7.421997	-0.00724	0.909691	0.023642	0.712225	0.053844	0.409349	-0.0302	0.638429	-0.03088	0.630989	-0.06109	0.352253	6789930	7.345573	7.4554	7.443295	7.360188	7.573194	7.403535	7.532716	7.443721	7.451086	7.350527	7.438171	7.477292	Eral1	NM_022313
6789939	2740	7.807172	7.883949	7.875835	7.855465	-0.04829	0.753855	0.028483	0.852972	0.02037	0.894503	0.008114	0.957854	-0.07678	0.619844	-0.06866	0.656782	6789939	8.015482	7.62672	7.779314	7.866672	8.179507	7.605667	7.917315	7.951984	7.758206	7.830382	7.839753	7.896261	Traf4	NM_009423
6789941	2741	6.193903	6.295755	6.288743	6.39504	-0.20114	0.055368	-0.09928	0.300859	-0.1063	0.270342	0.007012	0.939659	-0.10185	0.289382	-0.09484	0.321589	6789941	6.215935	6.323434	6.04234	6.35149	6.268917	6.266857	6.221433	6.365007	6.279789	6.446943	6.232465	6.505711	Nek8	NM_080849
6789948	2742	9.319334	9.314297	9.278273	9.272253	0.047081	0.435808	0.042045	0.484747	0.00602	0.919044	0.036024	0.547729	0.005037	0.93223	0.041061	0.494709	6789948	9.33853	9.314049	9.305423	9.238666	9.407019	9.297207	9.19897	9.357536	9.278313	9.211399	9.358383	9.246976	Supt6h	NM_009297
6789958	2743	6.281654	6.31865	6.316903	6.168128	0.113525	0.420151	0.150522	0.292525	0.148775	0.297776	0.001747	0.989885	-0.037	0.788852	-0.03525	0.798562	6789958	6.28536	6.139834	6.419767	6.28844	6.596308	6.071203	6.438015	6.243993	6.268701	6.164266	6.251916	6.088203	Foxn1	NM_008238
6789973	2746	7.288209	7.454696	7.35993	7.227054	0.061155	0.550893	0.227643	0.049114	0.132876	0.213136	0.094766	0.362927	-0.16649	0.128545	-0.07172	0.486135	6789973	7.184356	7.347761	7.33251	7.445346	7.48963	7.429113	7.513378	7.308424	7.257988	7.210674	7.061263	7.409224	Tmem199	NM_199199
6789974	2747	9.397164	9.434078	9.32986	9.363115	0.034049	0.789251	0.070963	0.580419	-0.03326	0.794029	0.104218	0.422131	-0.03691	0.772076	0.067304	0.599728	6789974	9.440603	9.3236	9.427288	9.353778	9.690472	9.257984	9.431235	9.325616	9.232729	9.193762	9.51085	9.384733	Tnfaip1	NM_009395
6789977	2748	5.82538	6.028189	6.013351	6.086151	-0.26077	0.267101	-0.05796	0.797607	-0.0728	0.747684	0.014838	0.947551	-0.20281	0.380692	-0.18797	0.414898	6789977	5.916135	5.945519	5.614485	5.913134	6.473999	5.697435	6.031672	6.230816	5.777565	5.857221	6.240149	6.161084	Tmem97	NM_133706
6789979	2749	8.210654	8.2838	8.248172	8.233676	-0.02302	0.628872	0.050124	0.305793	0.014496	0.759798	0.035628	0.459173	-0.07315	0.149047	-0.03752	0.436571	6789979	8.256896	8.157128	8.217939	8.256646	8.31418	8.280574	8.265137	8.256647	8.222733	8.149851	8.334394	8.216783	Nlk	NM_008702
6789998	2750	6.794795	6.91632	6.828602	7.098254	-0.30346	0.075894	-0.18193	0.256515	-0.26965	0.107715	0.087718	0.572025	-0.12152	0.438013	-0.03381	0.826073	6789998	6.944344	6.947783	6.492259	6.838644	6.883564	7.026752	6.921865	6.948259	6.615683	6.93306	7.189194	7.172509	1810009O	BC027022
6790012	2751	9.274942	9.310863	9.550194	8.487565	0.787377	0.065892	0.823298	0.056635	1.062629	0.020724	-0.23933	0.535664	-0.03592	0.925014	-0.27525	0.478003	6790012	9.322234	9.294419	9.208173	9.195627	9.898311	8.83865	9.971897	8.896068	9.782617	8.328276	8.988546	8.145872	Lgals9	NM_010708
6790015	2752	7.087533	7.34398	6.960818	8.401122	-1.31359	0.003079	-1.05714	0.009871	-1.4403	0.001794	0.383162	0.257447	-0.25645	0.438072	0.126714	0.697333	6790015	7.096557	6.950482	7.215559	7.288132	7.484755	7.259053	7.044769	7.085045	6.752641	8.134583	7.846216	9.222568	Ksr1	NM_013571
6790027	2753	8.542559	8.717689	8.615951	8.256257	0.286302	0.249951	0.461432	0.08061	0.359693	0.157745	0.101739	0.671025	-0.17513	0.469743	-0.07339	0.758639	6790027	8.835864	8.429731	8.362082	8.601429	9.102422	8.449217	8.813515	8.283565	8.750772	8.00012	8.448353	8.320299	Wsb1	NM_019653
6790046	2755	7.593501	7.35916	7.643101	6.545929	1.047572	0.021701	0.813231	0.058328	1.097172	0.017656	-0.28394	0.46305	0.234341	0.542508	-0.0496	0.896234	6790046	8.096519	7.586877	7.097108	7.233414	7.848966	6.9951	8.103748	7.201153	7.624403	6.276196	7.015586	6.346006	Evi2b	NM_146023
6790075	2756	7.389883	7.442429	7.448978	7.408986	-0.0191	0.818914	0.033443	0.689615	0.039992	0.633702	-0.00655	0.937346	-0.05255	0.533422	-0.05909	0.485114	6790075	7.38864	7.363076	7.417932	7.348273	7.586308	7.392705	7.543028	7.421796	7.382109	7.297138	7.541929	7.387891	Utp6	NM_144826
6790079	2757	7.64934	7.666715	7.281202	7.15893	0.490411	0.005462	0.507785	0.004519	0.122272	0.374701	0.385513	0.018038	-0.01737	0.897033	0.368139	0.022138	6790079	7.64639	7.63211	7.669521	7.625651	7.703588	7.670906	7.629936	7.044262	7.169407	7.140058	7.233362	7.103369	Crlf3	NM_018776
6790099	2759	6.983276	7.179485	7.052115	7.144163	-0.16089	0.294714	0.035321	0.811759	-0.09205	0.539143	0.12737	0.400628	-0.19621	0.208683	-0.06884	0.644253	6790099	6.913339	6.967611	7.068878	7.132605	7.136098	7.269751	7.232263	6.819439	7.104642	6.945714	7.051085	7.435691	5730455P	NM_027472

6790124	2760	9.299802	9.327232	9.199388	9.119495	0.180307	0.079314	0.207737	0.049269	0.079892	0.399194	0.127844	0.191992	-0.02743	0.767613	0.100415	0.295521	6790124	9.23913	9.284872	9.375405	9.274422	9.499181	9.208093	9.225531	9.130547	9.242085	9.014588	9.263242	9.080656	Myo1d	NM_177390
6790226	2764	7.734007	7.809658	7.900595	7.97771	-0.2437	0.01577	-0.16805	0.068437	-0.07712	0.362434	-0.09094	0.2877	-0.07565	0.37114	-0.16659	0.070416	6790226	7.625572	7.814428	7.76202	7.891375	7.829791	7.707809	7.863543	8.011631	7.826612	7.917887	7.9196	8.095644	Rffl	NM_001007465
6790228	2765	7.286318	7.396516	7.239372	7.182843	0.103475	0.016548	0.213673	0.000249	0.056529	0.137534	0.157144	0.001785	-0.1102	0.012301	0.046946	0.207791	6790228	7.278717	7.300508	7.279729	7.420835	7.415966	7.352746	7.183099	7.291653	7.243363	7.185019	7.131892	7.231617	Rad51l3	NM_011235
6790231	2766	6.670133	6.678236	6.694879	6.667273	0.002861	0.937498	0.010964	0.764405	0.027606	0.457383	-0.01664	0.650405	-0.0081	0.824471	-0.02475	0.50382	6790231	6.686574	6.654452	6.669374	6.754559	6.65112	6.62903	6.663607	6.753678	6.667351	6.653075	6.67271	6.676033	Nle1	NM_145431
6790239	2767	6.304339	6.243039	6.200876	5.205991	1.098348	0.022401	1.037047	0.028576	0.994884	0.033815	0.042163	0.916382	0.0613	0.878724	0.103463	0.797045	6790239	5.968585	6.323417	6.621015	5.648259	6.88499	6.195867	6.557154	5.604321	6.441152	4.926818	5.643909	5.047247	Slfn9	NM_172796
6790245	2769	6.244446	6.149676	6.240337	4.688706	1.555741	0.003051	1.460971	0.004345	1.551631	0.003097	-0.09066	0.813379	0.09477	0.805118	0.00411	0.991446	6790245	6.012295	6.369116	6.351928	5.960759	6.392509	6.095761	6.392407	5.678695	6.649908	4.267463	5.493363	4.305291	Slfn10	NM_181542
6790264	2770	6.545395	6.667066	6.818413	6.757644	-0.21225	0.030114	-0.09058	0.294032	0.060769	0.472803	-0.15135	0.097455	-0.12167	0.169878	-0.27302	0.009574	6790264	6.615635	6.400711	6.619838	6.706429	6.617589	6.677181	6.84528	6.91943	6.690529	6.711671	6.702799	6.858461	Pex12	NM_134025
6790275	2771	6.858684	6.885185	6.878348	6.584651	0.274033	0.07235	0.300533	0.052973	0.293697	0.057416	0.006837	0.9601	-0.0265	0.846414	-0.01966	0.885655	6790275	6.903369	6.844417	6.828267	6.927858	6.915649	6.812047	7.170495	6.558262	6.906287	6.669098	6.545318	6.539538	Mmp28	NM_080453
6790288	2772	7.083826	6.728007	6.806259	5.399847	1.683979	0.021435	1.32816	0.054664	1.406412	0.044451	-0.07825	0.897864	0.355819	0.56354	0.277567	0.650914	6790288	6.581905	7.712201	6.957372	6.132434	7.525949	6.525639	7.354279	5.622856	7.441641	5.526109	5.762192	4.91124	Ccl5	NM_013653
6790290	2773	9.177357	9.145002	9.066142	7.925005	1.252351	0.010448	1.219997	0.011878	1.141136	0.016296	0.078861	0.839346	0.032354	0.933638	0.111215	0.775242	6790290	9.304967	9.122198	9.104905	8.9377	9.718445	8.778862	9.34616	8.617146	9.235119	8.376265	8.227093	7.171658	Ccl6	NM_009139
6790291	2774	9.642409	9.458199	9.461053	8.19514	1.447269	0.003318	1.263059	0.006976	1.265913	0.006894	-0.00285	0.993707	0.18421	0.613733	0.181356	0.619148	6790291	9.96998	9.542899	9.414348	9.424348	9.92275	9.027499	9.879809	8.97098	9.532371	8.420826	8.533941	7.630653	Ccl9	NM_011338
6790294	2775	8.634386	8.203994	8.798345	7.100483	1.533904	0.033662	1.103511	0.10281	1.697863	0.022037	-0.59435	0.350328	0.430392	0.49306	-0.16396	0.791317	6790294	8.748174	8.989316	8.165669	8.456998	8.449294	7.70569	9.110516	7.758882	9.525638	7.157952	8.037726	6.10577	Ccl3	NM_011337
6790317	2776	9.052234	8.986498	8.648733	8.733742	0.318492	0.330967	0.252756	0.435251	-0.08501	0.78937	0.337765	0.30433	0.065736	0.836195	0.403501	0.226181	6790317	8.811979	8.817323	9.527401	8.662555	9.371849	8.92509	8.923152	8.444864	8.578184	8.37684	9.24957	8.574816	Dusp14	NM_019819
6790318	2777	7.603964	7.778681	7.538525	7.530284	0.07368	0.528688	0.248397	0.057171	0.008241	0.943092	0.240156	0.064127	-0.17472	0.157009	0.065439	0.57475	6790318	7.502168	7.48767	7.822053	7.599747	7.926111	7.810185	7.655694	7.441324	7.518557	7.513415	7.541863	7.535574	Tada2l	NM_172562
6790339	2778	8.062432	8.170814	8.021241	7.910691	0.151741	0.166364	0.260123	0.031126	0.11055	0.299548	0.149573	0.171795	-0.10838	0.30849	0.041192	0.69023	6790339	8.018747	8.006352	8.162198	8.133383	8.270806	8.108253	8.116844	7.813188	8.13369	7.810154	8.026668	7.895251	Aatf	NM_019816
6790356	2780	7.035089	7.09411	7.119035	7.078978	-0.04389	0.649379	0.015132	0.8747	0.040057	0.677848	-0.02493	0.795345	-0.05902	0.543133	-0.08395	0.392792	6790356	6.986137	6.913417	7.205712	7.042653	7.124223	7.115454	7.114396	7.099732	7.142977	6.892806	7.158349	7.185778	Mrm1	NM_145433
6790359	2781	8.177426	8.204885	8.386891	8.749572	-0.57215	0.027517	-0.54469	0.033624	-0.36268	0.126615	-0.18201	0.417123	-0.02746	0.900484	-0.20946	0.353638	6790359	8.272707	8.053095	8.206477	8.286976	8.269176	8.058502	8.314687	8.682756	8.16323	8.543286	8.479371	9.226059	Dhrs11	NM_177564
6790360	2782	9.291698	9.450686	9.304708	9.352118	-0.06042	0.417912	0.098568	0.201023	-0.04741	0.521632	0.145978	0.072969	-0.15899	0.054794	-0.01301	0.858664	6790360	9.307627	9.377045	9.190422	9.384328	9.486962	9.480768	9.349283	9.382099	9.182743	9.278161	9.341229	9.436964	Ggnbp2	NM_153144
6790366	2783	6.011046	6.130941	6.1252	6.004588	0.006458	0.953887	0.126353	0.276706	0.120612	0.297526	0.005741	0.959001	-0.1199	0.300212	-0.11415	0.322421	6790366	6.018226	5.953884	6.061028	6.113407	6.181858	6.097558	6.163008	6.189088	6.023503	5.753326	6.029669	6.230768	Pigw	NM_027388
6790367	2784	6.127334	6.255431	6.247725	6.225272	-0.09794	0.509095	0.030159	0.836808	0.022453	0.878051	0.007706	0.95797	-0.1281	0.392488	-0.12039	0.42032	6790367	5.888535	6.27639	6.217077	5.972557	6.428941	6.364796	6.239527	6.13339	6.370258	6.178851	6.260153	6.236813	Znhit3	NM_001005223
6790374	2785	8.861616	8.98458	8.90421	8.891615	-0.03	0.794299	0.092965	0.427714	0.012595	0.912674	0.08037	0.490707	-0.12296	0.301269	-0.04259	0.711858	6790374	8.837484	8.851109	8.896255	8.762647	9.217875	8.973218	8.898629	8.985869	8.828131	8.763039	9.008828	8.902978	Appbp2	NM_025825
6790478	2787	7.271794	7.274294	7.341752	7.261102	0.010692	0.923644	0.013193	0.905876	0.080651	0.476969	-0.06746	0.54998	-0.0025	0.982111	-0.06996	0.535647	6790478	7.301298	7.228975	7.285108	7.114758	7.480593	7.227532	7.509506	7.282561	7.23319	7.154384	7.372052	7.256869	Ints2	NM_027421
6790500	2788	8.503733	8.510715	8.479858	8.532374	-0.02864	0.646983	-0.02166	0.728333	-0.05252	0.408456	0.030857	0.622119	-0.00698	0.910537	0.023875	0.702049	6790500	8.533145	8.541622	8.436432	8.410762	8.587315	8.534067	8.515401	8.499579	8.424593	8.457964	8.629781	8.509377	Rps6kb1	NM_001114334
6790508	2789	10.13873	10.10325	9.981193	9.960814	0.177919	0.29119	0.142434	0.392065	0.020379	0.900204	0.122055	0.460498	0.035485	0.827331	0.15754	0.34631	6790508	10.30512	10.0629	10.04818	9.925905	10.37056	10.01328	10.20174	9.803101	9.938739	9.902895	9.820027	10.15952	Tmem49	NM_029478
6790513	2790	10.80135	10.78492	10.8573	10.65478	0.146577	0.1038	0.130143	0.141859	0.20252	0.034945	-0.07238	0.391304	0.016433	0.842121	-0.05594	0.503506	6790513	10.8603	10.77187	10.77189	10.66617	10.90989	10.7787	10.94166	10.86271	10.76752	10.53375	10.76219	10.66839	Cltc	NM_001003908
6790517	2791	7.88828	8.044866	7.969893	7.98989	-0.10161	0.211763	0.054976	0.483721	-0.02	0.796145	0.074973	0.345948	-0.15659	0.069853	-0.08161	0.307411	6790517	7.850294	7.923573	7.890974	7.939989	8.171454	8.023156	8.104612	7.890538	7.914529	7.964968	7.936675	8.068028	Dhx40	NM_026191
6790531	2792	8.231206	8.345354	8.170958	8.079406	0.1518	0.269901	0.265948	0.071487	0.091552	0.495014	0.174396	0.210379	-0.11415	0.398772	0.060248	0.650595	6790531	8.295482	8.349446	8.04869	8.174341	8.421232	8.440488	8.351729	7.925131	8.236013	8.126571	8.073927	8.03772	Ypel2	NM_001005341
6790536	2793	7.742782	8.285583	7.926024	8.503054	-0.76027	0.000887	-0.21747	0.179821	-0.57703	0.004546	0.359559	0.041198	-0.5428	0.006323	-0.18324	0.250662	6790536	7.492698	7.855105	7.880544	8.312469	8.182055	8.362225	7.853815	8.092055	7.832201	8.416219	8.324696	8.768248	Gdpd1	NM_025638
6790537	2794	6.587545	6.661332	6.53886	6.411349	0.176196	0.248315	0.249983	0.115286	0.127511	0.393856	0.122471	0.411977	-0.07379	0.616187	0.048685	0.739681	6790537	6.484848	6.593171	6.684616	6.63295	6.738914	6.612131	6.813075	6.590352	6.213154	6.307593	6.537277	6.389177	1200011M	BC020005
6790585	2803	8.828448	8.816862	8.515624	8.933658	-0.10521	0.584515	-0.1168	0.544743	-0.41803	0.053418	0.301238	0.141496	0.011586	0.951513	0.312824	0.128729	6790585	8.870295	8.768894	8.846156	8.876144	8.818017	8.756425	8.422126	8.928365	8.196382	8.77174	8.819511	9.209723	Dynll2	NM_026556
6790621	2804	9.474745	9.584088	9.340161	9.438162	0.036583	0.747881	0.145926	0.221042	-0.098	0.398743	0.243926	0.057302	-0.10934	0.349085	0.134583	0.255732	6790621	9.642704	9.447459	9.334071	9.627694	9.614956	9.509613	9.410513	9.491287	9.118684	9.415094	9.378049	9.521343	Msi2	NM_054043
6790648	2805	7.708496	7.75488	7.544044	7.569742	0.138755	0.452413	0.185138	0.322748	-0.0257	0.887324	0.210836	0.264431	-0.04638	0.798439	0.164452	0.376625	6790648	7.731881	7.853473	7.540135	7.685112	7.66953	7.909997	7.614834	7.834875	7.182423	7.377164	7.601792	7.730269	Akap1	NM_009648
6790655	2806	9.745472	9.801047	9.766852	9.640741	0.10473	0.686165	0.160305	0.539135	0.126111	0.627397	0.034194	0.89454	-0.05557	0.829576	-0.02138	0.933918	6790655	9.698577	9.696925	9.840913	9.593913	10.25029	9.558937	10.09	9.291969	9.918588	9.760379	9.751065	9.41078	Scpep1	NM_029023
6790664	2807	6.464575	6.70708	6.617664	6.44642	0.018155	0.903507	0.26066	0.110129	0.171244	0.27179	0.089415	0.554833	-0.2425	0.133179	-0.15309	0.322177	6790664	6.709578	6.527569	6.156578	6.753292	6.607242	6.760705	6.660042	6.668916	6.524035	6.238353	6.568369	6.532538	Dgke	NM_019505
6790674	2809	5.477664	6.324966	5.598593	7.43003	-1.95237	0.000191	-1.10506	0.00631	-1.83144	0.000295	0.726373	0.042356	-0.8473	0.022706	-0.12093	0.698489	6790674	5.356758	5.603531	5.472703	6.501323	6.19703	6.276544	5.276866	6.317121	5.201791	7.314286	7.163564	7.812241	4932411E	NM_172534
6790684	2810	8.409586	8.598035	7.897299	8.204955	0.204631	0.548311	0.39308	0.263052	-0.30766	0.373649	0.700736	0.064164	-0.18845	0.579713	0.512288	0.155298	6790684	8.120704	8.492994	8.615061	8.369265	8.413737	9.011103	7.568389	8.51393	7.609577	7.908448	8.047659	8.658758	Pctp	NM_008796
6790699	2811	6.073417	6.320752	5.781157	7.201011	-1.12759	0.002688	-0.88026	0.010216	-1.41985	0.000655	0.539594	0.07474	-0.24734	0.375334	0.292259	0.299549	6790699	5.856607	6.072334	6.291309	6.420235	6.065362	6.476658	5.620157	6.078274	5.645041	6.86623	6.957147	7.779657	Hlf	NM_172563
6790734	2812	6.829916	6.87692	6.82365	6.817197	0.012719	0.9071	0.059723	0.587207	0.006454	0.952768	0.053269	0.627552	-0.047	0.668042	0.006266	0.954142	6790734	6.779294	6.850492	6.859962	6.861718	7.052747	6.716294	6.655442	6.944327	6.871182	6.692921	6.830847	6.927822	Stxbp4	NM_011505
6790737	2813	6.887487	6.907654	6.946536	7.008225	-0.12074	0.6687	-0.10057	0.721008	-0.06169	0.826168	-0.03888	0.889799	-0.02017	0.942685	-0.05905	0.833474	6790737	6.903459	6.797422	6.961581	7.199657	7.244388	6.278916	6.642738	7.291074	6.905797	6.798149	7.107291	7.119236	Stxbp4	NM_011505
6790774	2814	9.01451	8.973508	8.956966	8.531052	0.483458	0.030306	0.442456	0.042877	0.425914	0.049336	0.016543	0.930578	0.041001	0.829263	0.057544	0.762494	6790774	8.887359	8.816427	9.339743	8.923951	9.177666	8.818908	9.282216	8.700214	8.888467	8.551181	8.513594	8.528381	Tom1l1	NM_028011
6790902	2816	7.394144	7.43922	7.484723	7.402433	-0.00829	0.964099	0.036787	0.84187	0.08229	0.65707	-0.0455	0.805218	-0.04508	0.807002	-0.09058	0.625534	6790902	7.496142	7.327837	7.358453	7.342731	7.534419	7.440511	7.791778	7.461828	7.200564	7.064942	7.598575	7.543783	Nme1	NM_008704
6790928	2817	7.439051	7.850559	8.1708	9.343502	-1.90445	0.040063	-1.49294	0.091252	-1.1727	0.170138	-0.32024	0.691413	-0.41151	0.611196	-0.73175	0.374445	6790928	6.467303	8.565262	7.284589	8.519393	6.858825	8.17346	7.83192	9.626319	7.05416	9.289851	9.312916	9.42774	Wfikkn2	NM_181819
6790940	2818	9.608311	9.707625	9.68493	9.523485	0.084826	0.407879	0.18414	0.094542	0.161445	0.135013	0.022694	0.821103	-0.09931	0.336417	-0.07662	0.452896	6790940	9.538641	9.635174	9.651118	9.60552	9.703568	9.813786	9.910886	9.523859	9.620046	9.483063	9.557869	9.529523	3300001P	NM_026313
6790944	2819	7.801841	7.657698	7.726801	6.800435	1.001406	0.008552	0.857263	0.018062	0.926366	0.012576	-0.0691	0.817227	0.144143	0.631737	0.07504	0.801901	6790944	8.172604	7.526249	7.706669	7.90674	7.805091	7.261262	8.065231	7.408136	7.707036	6.691018	7.246704	6.463582	Abcc3	NM_029600
6790952	2822	8.531603	8.640422	8.567864	9.152353	-0.62075	0.025139	-0.51193	0.053204	-0.58449	0.032243	0.072558	0.756292	-0.10882	0.642908	-0.03626	0.876451	6790952	8.381866	8.495777	8.717167	8.851404	8.698154	8.371708	8.413404	8.797094	8.493095	8.833113	8.996301	9.627645	Epn3	NM_027984
6790953	2823	5.887696	5.983554	6.026654	6.278017	-0.39032	0.022172	-0.29446	0.065332	-0.25136	0.105912	-0.0431	0.762708	-0.09586	0.506821	-0.13896	0.343292	6790953	5.746941	5.910999	6.005149	6.077271	5.766387	6.107004	6.041107	6.079213	5.959641	6.132776	6.14578	6.555496	Mycbpap	NM_170671
6790960	2824	8.277996	8.111524	7.883785	8.790853	-0.51286	0.189199	-0.67933	0.093849	-0.90707	0.034816	0.227739	0.541776	0.166473	0.653774	0.394211	0.302084	6790960	7.573879	8.613008	8.647102	8.048081	7.994149	8.292341	7.985514	8.218745	7.447096	8.856947	8.300551	9.21506	Acsf2	NM_153807
6790972	2826	7.85074	8.06669	8.002006	8.369806	-0.51907	0.002743	-0.30312	0.037487	-0.3678	0.016507	0.064684	0.609518	-0.21595	0.113921	-0.15127	0.249094	6790972	7.77393	7.973251	7.805039	7.871329	8.032886	8.295856	7.910058	8.015212	8.080749	8.310485	8.253161	8.545773	Xylt2	NM_145828
6791000	2829	6.315398	6.114972	6.068861	6.074951	0.240447	0.572311	0.040022	0.924354	-0.00609	0.98847	0.046111	0.912895	0.200425	0.636811	0.246537	0.562829	6791000	5.802235	5.96162	7.182338	6.510888	6.085051	5.748978	6.43663	5.939466	5.830487	5.63483	6.483241	6.106781	Sgca	NM_009161
6791015	2830	9.554801	9.540302	9.703432	10.19127	-0.63647	0.05359	-0.65097	0.04945	-0.48784	0.121246	-0.16313	0.578105	0.014499	0.960174	-0.14863	0.611741	6791015	9.381097	9.555723	9.727583	9.934977	9.21262	9.473308	9.289647	10.22405	9.596598	10.05025	9.99791	10.52566	Pdk2	NM_133667
6791017	2831	10.80018	10.35871	10.69681	9.825089	0.975091	0.006251	0.533617	0.079041	0.871717	0.011077	-0.3381	0.23817	0.441473	0.134577	0.103373	0.706889	6791017	11.12547	10.56705	10.70802	10.2331	10.81095	10.03207	11.12033	10.36083	10.60926	9.803658	9.990541	9.681069	Itga3	NM_013565
6791020	2832	6.254603	6.239225	6.333506	6.287075	-0.03247	0.763631	-0.04785	0.658772	0.046431	0.668163	-0.09428	0.392674	0.015379	0.886489	-0.0789	0.47127	6791020	6.232068	6.244265	6.287477	6.406692	6.092822	6.21816	6.212718	6.454798	6.333002	6.468987	6.204009	6.188229	Dlx4	NM_007867
6791023	2833	9.067411	9.155834	9.210192	9.050729	0.016682	0.825521	0.105105	0.189139	0.159463	0.0611	-0.05436	0.479146	-0.08842	0.261747	-0.14278	0.087031	6791023	9.004177	9.129032	9.069024	9.032516	9.30313	9.131856	9.225366	9.18349	9.22172	8.954499	9.144185	9.053504	Myst2	NM_177619
6791048	2834	6.208352	6.178341	6.089234	5.918239	0.290112	0.094677	0.260101	0.127731	0.170995	0.296416	0.089107	0.576546	0.030011	0.849465	0.119118	0.458891	6791048	6.374191	6.213303	6.037561	6.075451	6.483851	5.97572	6.178943	5.95645	6.132309	6.097235	5.784314	5.873169	Ngfr	NM_033217
6791063	2836	7.758998	7.548497	7.842818	7.442583	0.316415	0.066785	0.105914	0.498015	0.400235	0.027836	-0.29432	0.084041	0.210501	0.196035	-0.08382	0.589704	6791063	7.946002	7.817584	7.513408	7.600544	7.73425	7.310698	7.956941	7.827186	7.744328	7.29676	7.615181	7.415808	Abi3	NM_025659
6791072	2838	5.892834	7.003714	6.635379	5.706728	0.186106	0.557456	1.296986	0.002739	0.928651	0.015716	0.368335	0.26028	-1.11088	0.006459	-0.74255	0.040421	6791072	6.057939	5.737845	5.882717	7.098994	7.24954	6.662608	6.302525	7.341599	6.262013	5.7032	5.460434	5.95655	Igf2bp1	NM_009951
6791084	2839	9.82429	9.800147	9.75897	9.657594	0.166695	0.08946	0.142552	0.13712	0.101376	0.273825	0.041176	0.645979	0.024143	0.786728	0.065319	0.47075	6791084	9.815392	9.770704	9.886773	9.756399	9.939276	9.704765	9.797749	9.868698	9.610464	9.621924	9.76091	9.589949	Ube2z	NM_172300
6791132	2841	7.181009	7.129051	7.08196	7.074853	0.106157	0.209948	0.054199	0.506153	0.007107	0.929527	0.047092	0.562121	0.051958	0.523414	0.09905	0.239125	6791132	7.2188	7.211478	7.11275	7.125053	7.196041	7.06606	7.063143	7.240845	6.941891	7.01073	7.161223	7.052605	Snx11	NM_028965
6791139	2842	9.76664	9.709862	9.728827	9.769103	-0.00246	0.970446	-0.05924	0.384912	-0.04028	0.549456	-0.01896	0.776067	0.056778	0.404065	0.037813	0.573615	6791139	9.730837	9.814869	9.754213	9.766941	9.671794	9.690851	9.792047	9.793177	9.601257	9.664902	9.833928	9.80848	Nfe2l1	NM_008686
6791150	2843	8.248651	8.235242	8.204255	8.251599	-0.00295	0.974896	-0.01636	0.861508	-0.04734	0.616174	0.030987	0.741672	0.013409	0.88624	0.044396	0.637973	6791150	8.111259	8.24498	8.389715	8.215211	8.318791	8.171724	8.340955	8.208534	8.063276	8.249408	8.18154	8.323849	Cdk5rap3	NM_030248
6791151	2844	9.44366	9.550547	9.443701	9.296276	0.147384	0.140726	0.254271	0.022479	0.147424	0.140632	0.106846	0.269961	-0.10689	0.269794	-4E-05	0.999654	6791151	9.562047	9.39407	9.374864	9.469398	9.670195	9.512048	9.529498	9.445882	9.355722	9.198586	9.455844	9.234399	Kpnb1	NM_008379

6791156	2845	7.625089	7.628199	7.733594	8.045389	-0.4203	0.021396	-0.41719	0.022102	-0.31179	0.067231	-0.1054	0.494767	-0.00311	0.983679	-0.1085	0.482517	6791156	7.516541	7.645587	7.71314	7.617414	7.561662	7.705521	7.743552	8.046721	7.410509	7.996073	7.960812	8.179282	Pnpo	NM_134021
6791157	2846	7.254107	7.23688	7.38016	7.325697	-0.07159	0.539338	-0.08882	0.449298	0.054463	0.638793	-0.14328	0.235329	0.017227	0.881204	-0.12605	0.29163	6791157	7.464321	7.203934	7.094066	7.352542	7.313078	7.04502	7.344053	7.495374	7.301053	7.335194	7.333499	7.308399	Sp2	NM_030220
6791170	2847	6.057022	6.230895	5.97233	6.116912	-0.05989	0.635181	0.113982	0.375462	-0.14458	0.268001	0.258565	0.065904	-0.17387	0.190144	0.084692	0.505361	6791170	5.884649	6.186665	6.099751	6.313849	6.197647	6.181188	6.09866	6.001467	5.816863	6.217972	5.89044	6.242325	Lrrc46	NM_027026
6791171	2848	6.359013	6.307285	6.401796	6.404519	-0.04551	0.668839	-0.09723	0.37059	-0.00272	0.979457	-0.09451	0.38347	0.051728	0.627405	-0.04278	0.687371	6791171	6.364028	6.265578	6.447433	6.407726	6.289261	6.224869	6.445771	6.47935	6.280266	6.465363	6.195173	6.55302	Tbx21	NM_019507
6791174	2849	6.798891	6.767573	6.738194	6.815792	-0.0169	0.836638	-0.04822	0.560199	-0.0776	0.356693	0.029379	0.720775	0.031318	0.703351	0.060697	0.466218	6791174	6.88756	6.874962	6.634152	6.807316	6.781566	6.713837	6.685862	6.875415	6.653305	6.788254	6.838894	6.820229	Tbkbp1	NM_198100
6791175	2850	8.795534	8.816411	8.667457	8.670311	0.125223	0.204572	0.1461	0.145747	-0.00285	0.975661	0.148953	0.139022	-0.02088	0.823664	0.128077	0.195456	6791175	8.737952	8.822153	8.826497	8.742577	8.954015	8.75264	8.644997	8.753335	8.60404	8.752955	8.775713	8.482265	Npepps	NM_008942
6791204	2851	7.244281	7.319253	7.239932	7.556416	-0.31214	0.02865	-0.23716	0.077607	-0.31648	0.027049	0.07932	0.51759	-0.07497	0.540222	0.004348	0.971311	6791204	7.232905	7.274512	7.225425	7.447563	7.230483	7.279712	7.038071	7.502793	7.178933	7.583154	7.436135	7.649959	RP23-157	NM_018873
6791207	2852	6.807277	6.967678	6.903757	7.074124	-0.26685	0.122344	-0.10645	0.510245	-0.17037	0.302133	0.063921	0.689883	-0.1604	0.329446	-0.09648	0.549633	6791207	6.852356	6.818014	6.751462	6.906374	6.857937	7.138724	7.031349	7.087282	6.592641	7.175986	6.831244	7.215141	E130012A	BC055770
6791212	2853	8.146896	8.333419	8.321662	8.290378	-0.14348	0.41819	0.043041	0.804382	0.031284	0.857002	0.011758	0.945956	-0.18652	0.299424	-0.17477	0.328914	6791212	8.08126	8.273155	8.086274	8.436655	8.37259	8.191013	7.991753	8.712843	8.260389	8.238937	8.39712	8.235077	Pcgf2	NM_009545
6791215	2854	8.106159	8.258035	8.218978	7.996706	0.109453	0.361261	0.261329	0.049544	0.222272	0.084822	0.039057	0.73861	-0.15188	0.215944	-0.11282	0.347476	6791215	8.171291	8.083737	8.063448	8.276587	8.359263	8.138255	8.263929	8.389488	8.003516	7.82634	8.062291	8.101487	Pip4k2b	NM_054051
6791219	2855	6.602746	6.65755	6.599999	6.578373	0.024373	0.80095	0.079178	0.421752	0.021627	0.822906	0.057551	0.555356	-0.0548	0.573979	0.002746	0.977289	6791219	6.642988	6.623453	6.541796	6.544645	6.728966	6.69904	6.766311	6.492547	6.54114	6.456527	6.553686	6.724905	Ccdc49	NM_026186
6791222	2857	8.140186	8.40794	8.028213	8.246608	-0.10642	0.58322	0.161331	0.411352	-0.21839	0.274445	0.379726	0.075682	-0.26775	0.188267	0.111973	0.564139	6791222	7.921613	8.431442	8.067504	8.183291	8.517752	8.522776	8.012343	8.348556	7.723741	8.226971	8.201409	8.311445	Rpl23	NM_022891
6791229	2858	6.083775	6.092631	6.027404	5.790855	0.29292	0.118807	0.301777	0.109614	0.236549	0.196027	0.065227	0.707439	-0.00886	0.959173	0.056371	0.745394	6791229	6.252211	5.909858	6.089255	5.9943	6.373927	5.909667	6.173745	5.748261	6.160206	5.855581	5.887436	5.629547	Plxdc1	NM_028199
6791233	2860	7.355416	7.23097	7.100556	7.090173	0.265243	0.306534	0.140797	0.578025	0.010383	0.966945	0.130414	0.605864	0.124446	0.622185	0.25486	0.324632	6791233	7.113875	7.091925	7.860448	7.573755	7.192505	6.92665	7.223715	7.089409	6.988544	6.851516	7.211531	7.207472	Cacnb1	NM_031173
6791238	2862	8.421394	8.401425	8.049562	7.969799	0.451595	0.00065	0.431626	0.000867	0.079763	0.368548	0.351863	0.002982	0.019969	0.817447	0.371832	0.002162	6791238	8.397608	8.559443	8.30713	8.509885	8.305026	8.389364	8.121381	8.030158	7.997146	8.075665	7.866336	7.967396	Fbxl20	NM_028149
6791240	2863	9.111203	9.207434	9.166813	9.25733	-0.14613	0.175206	-0.0499	0.625222	-0.09052	0.383784	0.040621	0.690105	-0.09623	0.355992	-0.05561	0.586872	6791240	9.14364	9.141575	9.048394	9.07201	9.309692	9.2406	8.961623	9.235312	9.303503	9.167824	9.266743	9.337424	Med1	NM_134027
6791257	2865	8.293572	8.066575	8.241268	8.368433	-0.07486	0.686241	-0.30186	0.129597	-0.12717	0.496853	-0.17469	0.356834	0.226997	0.23961	0.052304	0.777169	6791257	8.169718	8.284353	8.426644	8.126493	8.091661	7.981571	8.332448	8.390391	8.000965	8.231565	8.103631	8.770104	1810046J	NM_025559
6791265	2866	7.691764	7.72435	7.413058	7.612228	0.079536	0.589303	0.112122	0.450817	-0.19917	0.196744	0.311292	0.058917	-0.03259	0.823576	0.278706	0.084284	6791265	7.893692	7.460067	7.721534	7.733485	7.69774	7.741826	7.642865	7.426124	7.170185	7.527324	7.552582	7.756779	Ormdl3	NM_025661
6791275	2867	8.160403	8.121393	8.12226	8.053061	0.107343	0.263263	0.068332	0.465681	0.069199	0.460238	-0.00087	0.992486	0.03901	0.673461	0.038144	0.680233	6791275	8.132838	8.080858	8.267514	8.107451	8.273867	7.982861	8.239012	8.011702	8.116065	7.988588	8.118278	8.052316	Med24	NM_011869
6791282	2868	10.57038	10.45335	10.18542	10.63631	-0.06592	0.87337	-0.18296	0.660027	-0.45089	0.293003	0.267928	0.522447	0.117035	0.77761	0.384963	0.364718	6791282	9.978955	10.85242	10.87977	10.67214	10.06029	10.62761	9.828675	10.1685	10.55908	10.0221	10.53414	11.35268	Nr1d1	NM_145434
6791298	2869	7.008295	7.308737	6.442314	5.410329	1.597965	0.047448	1.898408	0.023945	1.031985	0.169255	0.866423	0.240277	-0.30044	0.671669	0.565981	0.431331	6791298	7.111445	6.661814	7.251625	6.763694	8.123599	7.038919	7.364761	4.824431	7.13775	5.04617	5.926616	5.258202	Top2a	NM_011623
6791302	2870	5.92581	5.902641	5.817292	5.893027	0.032784	0.917424	0.009614	0.975737	-0.07573	0.810993	0.085348	0.787647	0.02317	0.941577	0.108518	0.732357	6791302	5.644736	5.913358	6.219337	6.288057	5.670214	5.749651	5.577943	6.460232	5.413702	6.111054	5.907241	5.660785	Gjd3	NM_178596
6791309	2871	5.846842	5.78182	5.699968	6.269496	-0.42265	0.291151	-0.48768	0.228492	-0.56953	0.166285	0.081853	0.832231	0.065022	0.86629	0.146874	0.704771	6791309	5.880949	5.773031	5.886546	5.61448	6.282903	5.448078	5.755395	5.661445	5.683063	6.0936	7.141151	5.573737	Tns4	NM_172564
6791310	2872	6.207772	5.966441	6.098957	5.299342	0.908429	0.009643	0.667099	0.037991	0.799614	0.017733	-0.13252	0.635231	0.24133	0.395456	0.108815	0.696189	6791310	6.51126	5.98866	6.123395	6.273129	6.172797	5.453398	6.204718	5.732624	6.359528	5.034147	5.454207	5.409673	Ccr7	NM_007719
6791316	2873	7.282482	7.291272	7.329005	7.126471	0.156011	0.580637	0.164801	0.559953	0.203434	0.474324	-0.03863	0.89016	-0.00879	0.97492	-0.04742	0.865429	6791316	7.218959	7.288801	7.336677	7.023994	7.554678	7.295143	7.826831	6.879955	7.282928	6.696579	7.295507	7.387327	Smarce1	NM_020618
6791331	2877	5.999491	6.058889	6.026903	6.260219	-0.26073	0.011455	-0.20133	0.035777	-0.23332	0.019261	0.031986	0.699303	-0.0594	0.478368	-0.02741	0.740309	6791331	6.111031	6.020449	5.866994	6.006239	6.021564	6.148864	6.060876	6.116507	5.903325	6.20436	6.336774	6.239523	Krt10	NM_010660
6791332	2878	6.157689	6.228282	6.232636	6.196324	-0.03864	0.640977	0.031958	0.699044	0.036313	0.660905	-0.00435	0.957787	-0.07059	0.40178	-0.07495	0.374733	6791332	6.242212	5.971707	6.259147	6.193228	6.197299	6.294319	6.16134	6.335701	6.200868	6.213476	6.171103	6.204392	Krt12	NM_010661
6791404	2894	6.532689	5.897214	5.704193	8.140944	-1.60826	0.366863	-2.24373	0.218823	-2.43675	0.18534	0.193021	0.911441	0.635475	0.715323	0.828496	0.635458	6791404	5.600341	5.768915	8.228811	5.699925	6.340382	5.651334	5.608598	5.448459	6.055521	6.196426	12.53632	5.690086	Krt13	NM_010662
6791406	2896	10.03086	10.19124	10.12883	10.14638	-0.11553	0.569787	0.044862	0.823758	-0.01755	0.930474	0.062412	0.757039	-0.16039	0.434468	-0.09797	0.628779	6791406	9.828195	9.919025	10.34535	10.18742	10.34517	10.04114	10.40119	9.825494	10.15981	10.33297	9.915465	10.19071	Krt19	NM_008471
6791408	2898	5.762129	6.310331	5.909577	6.960243	-1.19811	0.059972	-0.64991	0.269031	-1.05067	0.091115	0.400754	0.484858	-0.5482	0.345777	-0.14745	0.794404	6791408	5.495152	6.045394	5.74584	5.987774	7.202022	5.741197	5.958441	5.702982	6.067307	7.662029	7.448946	5.769753	Krt14	NM_016958
6791410	2900	5.983566	5.940291	5.56396	5.859197	0.124369	0.592018	0.081094	0.725337	-0.29524	0.221776	0.376331	0.129713	0.043275	0.850853	0.419606	0.096443	6791410	6.047579	5.892978	6.010141	5.63157	6.414623	5.77468	5.673223	5.606292	5.412366	6.19402	5.568882	5.814688	Krt17	NM_010663
6791411	2901	5.975576	6.120662	6.176081	6.007141	-0.03157	0.634914	0.113521	0.113842	0.16894	0.029654	-0.05542	0.411475	-0.14509	0.053027	-0.20051	0.013914	6791411	6.014282	5.883049	6.029396	6.165606	6.115402	6.080977	6.199907	6.113044	6.215291	6.030623	5.882246	6.108554	Krt42	NM_212483
6791418	2902	7.157821	7.309155	7.012641	6.998958	0.158864	0.346384	0.310198	0.086523	0.013683	0.933447	0.296514	0.098811	-0.15133	0.368473	0.14518	0.387291	6791418	7.400589	7.046135	7.02674	7.383429	7.381805	7.162232	6.891044	7.303612	6.843267	7.125652	6.814398	7.056823	Hap1	NM_010404
6791422	2903	10.95399	10.94364	11.01102	11.00548	-0.05148	0.748076	-0.06184	0.700096	0.005543	0.97232	-0.06738	0.674967	0.010353	0.948334	-0.05703	0.722225	6791422	10.97056	10.97477	10.91665	10.99736	11.12863	10.70493	11.00294	11.00712	11.023	10.68036	11.29605	11.04002	Jup	NM_010593
6791423	2904	8.335156	8.326401	8.235069	8.621605	-0.28645	0.063695	-0.2952	0.057491	-0.38654	0.019821	0.091332	0.512237	0.008755	0.949198	0.100088	0.473871	6791423	8.49126	8.40845	8.105759	8.407186	8.385386	8.186631	8.255059	8.434067	8.01608	8.561584	8.590034	8.713198	1110036O	NM_176830
6791424	2905	8.450256	8.636826	8.631678	9.019657	-0.5694	0.014581	-0.38283	0.070364	-0.38798	0.067359	0.005148	0.978299	-0.18657	0.338919	-0.18142	0.351656	6791424	8.049964	8.709372	8.591431	8.537521	8.582228	8.79073	8.642278	8.858178	8.394578	8.92993	9.076994	9.052048	Nt5c3l	NM_001102650
6791427	2906	6.229413	6.457877	6.32629	6.402404	-0.17299	0.190928	0.055472	0.659027	-0.07611	0.547105	0.131587	0.30878	-0.22846	0.095877	-0.09688	0.446743	6791427	6.333654	6.356855	5.997729	6.372022	6.531728	6.46988	6.334658	6.496198	6.148013	6.289401	6.492707	6.425105	Klhl11	NM_172565
6791428	2907	9.842588	9.780437	9.710546	9.357374	0.485214	0.010453	0.423063	0.019904	0.353172	0.041812	0.069891	0.644743	0.062151	0.681359	0.132042	0.391909	6791428	9.847223	9.785464	9.895076	9.622898	9.900111	9.818302	9.899749	9.545514	9.686375	9.198471	9.668502	9.205148	Acly	NM_134037
6791432	2908	9.806896	9.923208	9.865404	9.878661	-0.07177	0.352501	0.044547	0.557069	-0.01326	0.859848	0.057804	0.449514	-0.11631	0.148303	-0.05851	0.444215	6791432	9.780289	9.826936	9.813463	9.820354	9.995931	9.95334	9.808232	9.892158	9.895823	9.802804	9.79018	10.043	Dnajc7	NM_019795
6791436	2909	6.317037	6.349926	6.322669	6.239344	0.077693	0.299379	0.110582	0.152896	0.083325	0.268119	0.027257	0.707212	-0.03289	0.651079	-0.00563	0.937863	6791436	6.351606	6.266478	6.333028	6.42237	6.275331	6.352077	6.34754	6.453569	6.166899	6.242229	6.206514	6.269289	Zfp385c	NM_177790
6791439	2911	7.745112	7.724799	7.688159	7.540024	0.205088	0.102341	0.184775	0.135131	0.148135	0.219476	0.03664	0.75022	0.020313	0.859587	0.056954	0.622337	6791439	7.759266	7.663443	7.812628	7.665818	7.944976	7.563603	7.765263	7.755639	7.543574	7.438598	7.668701	7.512773	Kat2a	NM_001038010
6791440	2912	9.253242	9.288661	9.323781	9.225083	0.028159	0.780292	0.063278	0.530662	0.098398	0.338027	-0.03512	0.725508	-0.07054	0.723285	-0.07054	0.485929	6791440	9.30302	9.286148	9.170559	9.257752	9.382187	9.226045	9.521106	9.28148	9.168758	9.171854	9.155149	9.349146	Rab5c	NM_024456
6791442	2913	5.85223	5.863142	6.040563	5.856955	-0.00473	0.972032	0.006187	0.963388	0.183608	0.197479	-0.17742	0.211459	-0.01091	0.935481	-0.18833	0.187352	6791442	5.961739	5.686952	5.908	5.966009	5.727639	5.895778	5.921119	6.106618	6.093952	6.061926	5.599809	5.909131	Hcrt	NM_010410
6791443	2914	7.420175	7.328948	7.380887	7.597105	-0.17693	0.132693	-0.26816	0.034878	-0.21622	0.075025	-0.05194	0.636356	0.091227	0.413228	0.039288	0.719779	6791443	7.398832	7.351793	7.5099	7.474386	7.328151	7.184307	7.206545	7.464897	7.47122	7.571919	7.482706	7.736689	Ghdc	NM_031871
6791444	2915	7.556981	7.425629	7.386671	7.272703	0.284278	0.041403	0.152927	0.228004	0.113969	0.359075	0.038958	0.747996	0.131352	0.294669	0.17031	0.184044	6791444	7.437389	7.411077	7.822477	7.417945	7.453223	7.40572	7.426635	7.417211	7.316168	7.156285	7.452421	7.209402	Stat5b	NM_011489
6791451	2916	10.44528	10.41447	10.33992	10.07572	0.36956	0.011392	0.338746	0.017191	0.264203	0.047692	0.074543	0.528303	0.030813	0.792159	0.105357	0.378781	6791451	10.50856	10.39413	10.43315	10.28448	10.57116	10.38776	10.47196	10.19597	10.35184	10.05158	10.26857	9.907011	Stat3	NM_213659
6791453	2917	8.762037	8.740435	8.542469	8.413198	0.348839	0.121647	0.327237	0.143017	0.129272	0.539096	0.197966	0.354632	0.021602	0.917264	0.219567	0.307577	6791453	8.653585	8.901471	8.731054	8.86288	8.930488	8.427937	8.782858	8.499495	8.345055	8.118162	8.75704	8.364391	Ptrf	NM_008986
6791465	2918	6.380989	6.873272	6.567454	6.797563	-0.41657	0.238923	0.075709	0.82291	-0.23011	0.502042	0.305818	0.377539	-0.49228	0.171043	-0.18646	0.584583	6791465	6.576133	5.97641	6.590425	6.850913	6.98421	6.784692	7.102582	5.962488	6.637292	6.565904	6.537914	7.288871	Psmc3ip	NM_008949
6791467	2919	8.379893	8.433024	8.307131	8.206668	0.173225	0.145461	0.226356	0.068146	0.100462	0.37699	0.125893	0.274879	-0.05313	0.634138	0.072763	0.517237	6791467	8.550653	8.278837	8.31019	8.323457	8.617775	8.35784	8.450671	8.204019	8.266702	8.179985	8.202416	8.277795	Fam134c	NM_028933
6791470	2920	6.93786	6.950953	6.986631	6.72658	0.21128	0.180827	0.224373	0.158127	0.26005	0.108825	-0.03568	0.810719	-0.01309	0.929848	-0.04877	0.743776	6791470	6.993363	6.855848	6.96437	6.916946	7.14801	6.787904	7.020883	7.237152	6.701857	6.804307	6.782803	6.592631	Plekhh3	NM_146030
6791471	2921	6.048459	6.002308	6.039929	5.828723	0.219736	0.249427	0.173585	0.355276	0.211206	0.266773	-0.03762	0.836928	0.046151	0.800798	0.00853	0.962729	6791471	6.084886	6.133665	5.926826	6.134949	6.080815	5.791159	5.987659	6.155204	5.976924	5.804639	5.478096	6.203434	Ccr10	NM_007721
6791472	2922	9.426992	9.457845	9.458739	9.38401	0.042982	0.700622	0.073835	0.512877	0.074729	0.50793	-0.00089	0.993588	-0.03085	0.782062	-0.03175	0.775946	6791472	9.350933	9.42724	9.502803	9.496157	9.40317	9.474208	9.557937	9.359299	9.458981	9.205992	9.305654	9.640383	Ezh1	NM_007970
6791479	2924	8.899984	8.933627	8.838372	8.810921	0.089063	0.582116	0.122706	0.45229	0.027451	0.864104	0.095255	0.556717	-0.03364	0.833935	0.061612	0.701976	6791479	8.725065	8.827309	9.147577	8.660147	9.089903	9.050831	8.921877	8.968706	8.624533	8.737672	8.819078	8.876012	Ccdc56	NM_026618
6791482	2925	8.761296	8.782056	8.800853	8.715488	0.045808	0.600583	0.066568	0.451175	0.085365	0.3395	-0.0188	0.828632	-0.02076	0.811124	-0.03956	0.650449	6791482	8.718534	8.746854	8.818501	8.635024	8.872082	8.839062	8.912042	8.775623	8.714894	8.59484	8.729672	8.821952	Becn1	NM_019584
6791493	2926	7.698669	7.691278	7.80192	7.789915	-0.09125	0.398559	-0.09864	0.36329	0.012006	0.90949	-0.11064	0.311077	0.007391	0.944191	-0.10325	0.342483	6791493	7.661309	7.653933	7.780764	7.63263	7.721541	7.719662	7.869934	7.962014	7.573813	7.674688	7.783736	7.91132	Aarsd1	NM_144829
6791501	2928	9.581512	9.599546	9.448496	9.315735	0.265777	0.048059	0.283811	0.037548	0.132761	0.277746	0.15105	0.221765	-0.01803	0.878223	0.133016	0.276891	6791501	9.617991	9.514399	9.612146	9.670923	9.666183	9.461533	9.639456	9.412517	9.293515	9.125651	9.458838	9.362717	Vat1	NM_012037
6791520	2930	6.766605	6.417048	6.417168	5.982082	0.784523	0.053181	0.434967	0.24434	0.435086	0.244222	-0.00012	0.999733	0.349556	0.342123	0.349437	0.342278	6791520	6.87898	7.037494	6.38334	6.578573	6.599594	6.072978	6.759831	6.285866	6.205806	5.255286	6.511875	6.179084	Meox1	NM_010791
6791525	2932	5.84061	6.034427	6.105033	6.109773	-0.26916	0.159246	-0.07535	0.67543	-0.00474	0.978866	-0.07061	0.694584	-0.19382	0.296173	-0.26442	0.165832	6791525	6.009042	6.03502	5.477769	6.15986	6.118344	5.825077	6.309939	6.10104	5.904121	6.112955	6.024228	6.192136	Sost	NM_024449
6791528	2933	9.639439	9.783322	9.563781	9.698459	-0.05902	0.572814	0.084863	0.422489	-0.13468	0.216582	0.219541	0.060215	-0.14388	0.189692	0.075657	0.472669	6791528	9.615158	9.519411	9.783747	9.786275	9.86412	9.699572	9.595428	9.45385	9.642066	9.532157	9.707891	9.855329	Dusp3	NM_028207
6791529	2934	6.142249	6.368977	5.933971	6.180203	-0.03795	0.593673	0.188774	0.024544	-0.24623	0.006936	0.435006	0.000216	-0.22673	0.010555	0.208278	0.015846	6791529	6.074192	6.16005	6.192505	6.417641	6.319835	6.369455	5.880766	6.073581	5.847566	6.110075	6.157932	6.272602	Mpp3	NM_007863
6791532	2935	7.507661	7.375903	7.344214	7.987733	-0.48007	0.069293	-0.61183	0.028268	-0.64352	0.022824	0.031689	0.893345	0.131758	0.580808	0.163447	0.495617	6791532	7.564864	7.555871	7.402248	7.448369	7.322864	7.356475	6.948338	7.921628	7.162676	7.88485	8.219449	7.8589	Mpp2	NM_016695

6791533	2936	7.327728	7.37864	7.357379	7.255754	0.071974	0.582974	0.122886	0.357294	0.101625	0.442577	0.021261	0.869996	-0.05091	0.696322	-0.02965	0.819603	6791533	7.399811	7.092158	7.491216	7.300516	7.579331	7.256073	7.431599	7.262334	7.378204	7.366588	7.262438	7.138236	Ppy	NM_008918
6791534	2937	5.889086	6.034869	6.069825	5.804733	0.084353	0.782163	0.230136	0.45773	0.265092	0.39505	-0.03496	0.908588	-0.14578	0.634427	-0.18074	0.557062	6791534	6.258653	5.304392	6.104214	6.188454	6.012922	5.903231	5.644896	6.413348	6.15123	6.108439	5.78402	5.52174	Pyy	NM_145435
6791540	2938	6.16073	6.244422	6.228438	6.020297	0.140433	0.237858	0.224125	0.076152	0.208141	0.095317	0.015984	0.888144	-0.08369	0.468914	-0.06771	0.555607	6791540	5.992855	6.214594	6.274741	6.188398	6.386979	6.157889	6.324604	6.228004	6.132705	5.881176	6.196972	5.982742	Tmem101	NM_029649
6791541	2939	9.032462	9.180632	9.123049	9.060616	-0.02815	0.9038	0.120015	0.609331	0.062433	0.789076	0.057582	0.805053	-0.14817	0.529919	-0.09059	0.698649	6791541	8.953463	9.017985	9.125937	9.179686	9.303121	9.059088	9.344543	9.506964	8.517641	9.017759	9.098575	9.065515	Lsm12	NM_172947
6791543	2940	7.806362	7.569695	7.595481	7.425751	0.380611	0.001576	0.143944	0.114509	0.16973	0.070206	-0.02579	0.759167	0.236667	0.019534	0.210881	0.031886	6791543	7.819024	7.812625	7.787438	7.574039	7.625564	7.509482	7.649652	7.578267	7.558525	7.350333	7.634955	7.291965	Hdac5	NM_001077696
6791558	2941	8.637301	8.761179	8.671269	8.78163	-0.14433	0.306138	-0.02045	0.880739	-0.11036	0.427474	0.08991	0.515099	-0.12388	0.37556	-0.03397	0.803453	6791558	8.534289	8.696529	8.681084	8.659784	8.928503	8.695251	8.580749	8.951779	8.48128	8.686151	8.746111	8.912628	Ubtf	NM_011551
6791565	2943	9.978588	9.873326	9.978071	10.69479	-0.71621	0.001794	-0.82147	0.000767	-0.71672	0.001786	-0.10475	0.52152	0.105262	0.519521	0.000517	0.997441	6791565	10.04694	9.98763	9.901194	9.973204	9.858329	9.788444	9.832519	10.31609	9.785604	10.58275	10.55907	10.94256	Slc25a39	NM_026542
6791569	2944	7.879588	8.099133	7.925861	8.100251	-0.22066	0.04534	-0.00112	0.990716	-0.17439	0.098111	0.173272	0.099937	-0.21955	0.046198	-0.04627	0.632754	6791569	7.815183	7.8855	7.938081	8.094307	8.096208	8.106884	8.007863	8.079792	7.689928	8.020283	8.120597	8.159874	Fam171a2	NM_199200
6791570	2945	6.165304	6.213759	6.128102	6.236835	-0.07153	0.139691	-0.02308	0.611174	-0.10873	0.037375	0.085657	0.085191	-0.04845	0.298943	0.037202	0.418577	6791570	6.114938	6.199539	6.181435	6.157135	6.183788	6.300354	6.170553	6.142052	6.071702	6.211091	6.273078	6.226337	Itga2b	NM_010575
6791626	2947	6.75577	6.949238	6.866461	6.778558	-0.02279	0.841147	0.17068	0.159559	0.087902	0.447549	0.082778	0.473539	-0.19347	0.116838	-0.11069	0.344006	6791626	6.722716	6.721828	6.822767	6.888043	7.198778	6.760894	6.967197	6.918458	6.713727	6.760819	6.771727	6.803129	Ccdc43	NM_025918
6791639	2949	8.998941	9.005134	8.972307	9.150105	-0.15116	0.283499	-0.14497	0.302287	-0.1778	0.213292	0.032827	0.809141	-0.00619	0.963589	0.026634	0.844535	6791639	8.942332	8.929538	9.124953	9.025956	9.153516	8.83593	9.196401	8.78262	8.937899	9.236948	8.976698	9.236668	Eftud2	NM_011431
6791644	2951	6.06207	6.157648	5.841468	5.496497	0.565573	0.047604	0.661151	0.025747	0.344971	0.191774	0.31618	0.22761	-0.09558	0.703154	0.220602	0.388558	6791644	6.108053	5.797801	6.280357	5.910921	6.70399	5.858034	5.894496	5.565793	6.064116	5.527656	5.525433	5.436403	3000004C	BC057614
6791656	2953	8.504372	8.433743	8.455868	8.392995	0.111377	0.463659	0.040748	0.785426	0.062873	0.675453	-0.02212	0.882284	0.070629	0.638624	0.048505	0.746128	6791656	8.52121	8.399545	8.592362	8.344476	8.801096	8.155658	8.399709	8.518761	8.449133	8.344644	8.443758	8.390583	Dcakd	NM_026551
6791666	2954	6.505259	6.632384	6.482809	6.615282	-0.11002	0.193777	0.017101	0.831007	-0.13247	0.126007	0.149574	0.089921	-0.12712	0.139826	0.02245	0.779592	6791666	6.602321	6.420208	6.493248	6.68254	6.723305	6.491306	6.456967	6.552417	6.439044	6.642895	6.511681	6.691271	Plcd3	NM_152813
6791686	2956	7.1898	7.126044	7.104846	7.087348	0.102452	0.271568	0.038696	0.667414	0.017498	0.845198	0.021198	0.813127	0.063756	0.483405	0.084954	0.356165	6791686	7.320903	7.146168	7.10233	7.095397	7.247396	7.035339	6.99979	7.24811	7.066639	7.152547	7.066858	7.04264	Map3k14	NM_016896
6791696	2957	6.559202	6.940848	6.752733	7.065678	-0.50648	0.048623	-0.12483	0.58254	-0.31294	0.188954	0.188115	0.413188	-0.38165	0.118031	-0.19353	0.400458	6791696	6.599991	6.500619	6.576997	7.098894	7.181427	6.542222	6.598606	7.131598	6.527994	6.863196	7.019408	7.314429	Arhgap27	NM_133715
6791727	2958	8.405606	8.484438	8.329143	8.826836	-0.42123	0.079611	-0.3424	0.141401	-0.49769	0.045129	0.155294	0.480435	-0.07883	0.716938	0.076463	0.725024	6791727	8.222102	8.463107	8.53161	8.512102	8.467605	8.473606	8.096587	8.816419	8.074424	8.635416	8.744987	9.100105	Sh3d20	NM_183288
6791729	2959	9.024661	8.848472	8.979845	8.793867	0.230794	0.018897	0.054605	0.507299	0.185978	0.045683	-0.13137	0.13351	0.176189	0.055475	0.044816	0.584564	6791729	9.118958	9.019167	8.935859	8.76726	9.023045	8.755112	8.980196	9.008139	8.9512	8.731094	8.870817	8.77969	Plekhm1	NM_183034
6791757	2961	8.671593	8.657468	8.82831	8.84823	-0.17664	0.166801	-0.19076	0.139129	-0.01992	0.868083	-0.17084	0.179522	0.014125	0.906203	-0.15672	0.214185	6791757	8.675189	8.684229	8.655361	8.619278	8.852328	8.500797	8.86417	8.921701	8.699059	8.782785	9.060305	8.701601	Gosr2	NM_019650
6791765	2963	8.18734	8.17024	8.178182	8.076854	0.110486	0.33653	0.093386	0.412679	0.101328	0.37588	-0.00794	0.943216	0.0171	0.878194	0.009158	0.934552	6791765	8.295871	8.145926	8.120223	8.112677	8.280077	8.117965	8.405581	7.968359	8.160607	8.012736	8.139153	8.078673	Nsf	NM_008740
6791793	2964	9.099979	9.096751	9.037264	9.019224	0.080755	0.269411	0.077527	0.287575	0.01804	0.797649	0.059487	0.407522	0.003228	0.963334	0.062715	0.383709	6791793	9.0623	9.122542	9.115094	9.03858	9.22602	9.025653	8.945274	9.151716	9.014801	8.988647	9.083502	8.985523	1700081L	NM_001081045
6791800	2965	9.343798	9.379637	9.238522	9.13906	0.204738	0.081118	0.240577	0.047088	0.099462	0.3608	0.141115	0.206352	-0.03584	0.735911	0.105276	0.334944	6791800	9.494688	9.2629	9.273805	9.290233	9.551362	9.297316	9.391343	9.184642	9.13958	9.070615	9.222349	9.124216	Cdc27	NM_145436
6791820	2966	7.237521	7.488836	7.612444	7.438058	-0.20054	0.160844	0.050778	0.705792	0.174386	0.215868	-0.12361	0.368716	-0.25131	0.088805	-0.37492	0.020225	6791820	7.221719	7.225815	7.265029	7.650641	7.570912	7.244954	7.772645	7.643945	7.420741	7.449821	7.282305	7.582047	1700052K	BC099431
6791856	2968	8.756103	8.588389	8.516296	8.458172	0.29793	0.005007	0.130216	0.132534	0.058124	0.476155	0.072093	0.380969	0.167714	0.063094	0.239807	0.015025	6791856	8.717793	8.774701	8.775814	8.533335	8.699544	8.532287	8.663931	8.421129	8.463828	8.513349	8.513185	8.347983	Cyb561	NM_007805
6791881	2969	9.223991	9.393588	9.259554	9.716315	-0.49232	0.063463	-0.32273	0.195806	-0.45676	0.080814	0.134034	0.57391	-0.1696	0.479461	-0.03556	0.880254	6791881	9.281951	9.138863	9.25116	9.4566	9.615585	9.10858	9.235416	9.482939	9.060308	9.572794	9.363531	10.21262	Limd2	NM_172397
6791883	2970	8.652963	8.629031	8.658979	8.703315	-0.05035	0.647257	-0.07428	0.503022	-0.04434	0.686562	-0.02995	0.784565	0.023933	0.826919	-0.00602	0.956103	6791883	8.611868	8.624721	8.722301	8.676279	8.708829	8.501984	8.669091	8.730126	8.577721	8.564829	8.596375	8.94874	Strada	NM_028126
6791900	2971	8.472045	8.491659	8.576205	8.414309	0.057736	0.618713	0.07735	0.507633	0.161896	0.184697	-0.08455	0.470179	-0.01961	0.864784	-0.10416	0.3777	6791900	8.486136	8.433267	8.496733	8.300984	8.732445	8.441547	8.585487	8.449209	8.69392	8.32935	8.524721	8.388856	Ftsj3	NM_025310
6791902	2972	7.493495	7.610954	7.444776	7.342893	0.150601	0.205907	0.26806	0.039912	0.101882	0.378932	0.166178	0.167229	-0.11746	0.314265	0.048719	0.667871	6791902	7.538528	7.473176	7.46878	7.434401	7.768085	7.630375	7.658741	7.284721	7.390865	7.273464	7.414894	7.340322	Smarcd2	NM_001130187
6791914	2974	6.950423	7.032674	6.992346	7.153124	-0.2027	0.291635	-0.12045	0.52121	-0.16078	0.396687	0.040327	0.827918	-0.08225	0.659071	-0.04192	0.821244	6791914	7.086747	6.932851	6.831672	7.078077	7.033011	6.986933	7.223891	6.709349	7.043799	6.924792	7.007334	7.527246	Cd79b	NM_008339
6791915	2975	6.336813	6.098332	5.928916	6.051611	0.285202	0.505776	0.046722	0.911955	-0.12269	0.772055	0.169416	0.689887	0.238481	0.576276	0.407897	0.348279	6791915	5.713595	5.940269	7.356575	6.437794	6.025972	5.831231	6.065443	5.996933	5.724372	5.745755	6.084956	6.324121	Scn4a	NM_133199
6791918	2976	7.343508	7.665773	7.14004	7.425439	-0.08193	0.464913	0.240334	0.054488	-0.2854	0.028231	0.525732	0.001158	-0.32227	0.016603	0.203467	0.093154	6791918	7.2445	7.477391	7.308632	7.62861	7.726391	7.642317	7.311295	7.127663	6.981163	7.251135	7.481329	7.543852	Icam2	NM_010494
6791921	2977	9.183898	9.027992	9.171957	9.369184	-0.18529	0.357879	-0.34119	0.110169	-0.19723	0.329459	-0.14397	0.470211	0.155906	0.435521	0.01194	0.951417	6791921	9.245538	9.211208	9.094947	8.994938	8.965395	9.123642	8.987583	9.339949	9.18834	9.70543	9.496211	8.905912	Ern1	NM_023913
6791942	2978	9.038159	9.072527	9.151805	9.210385	-0.17223	0.183641	-0.13786	0.27758	-0.05858	0.633881	-0.07928	0.521748	-0.03437	0.778875	-0.11365	0.364984	6791942	8.977878	8.94969	9.18691	9.109576	9.031132	9.076872	9.027784	9.428227	8.999405	9.159694	9.156766	9.314695	Tex2	NM_198292
6791960	2979	9.87466	10.09808	9.902119	9.925816	-0.05116	0.804724	0.172266	0.414475	-0.0237	0.908674	0.195963	0.35623	-0.22342	0.296723	-0.02746	0.894274	6791960	10.04747	10.0188	9.557711	10.19226	9.980527	10.12146	10.17323	9.652362	9.880765	9.614226	10.19218	9.971043	Pecam1	NM_008816
6791964	2980	5.994001	5.934973	5.918356	5.895417	0.098584	0.455931	0.039556	0.761298	0.022939	0.859883	0.016616	0.898206	0.059028	0.651518	0.075645	0.564383	6791964	5.946532	6.069143	5.966328	5.805584	5.940091	6.059243	5.705876	6.013805	6.035388	5.845488	6.117155	5.723608	Polg2	NM_015810
6791965	2981	11.09001	11.19606	11.22556	11.13236	-0.04235	0.611699	0.063697	0.449867	0.093197	0.27859	-0.0295	0.722472	-0.10605	0.222518	-0.13555	0.129389	6791965	11.048	11.17836	11.04368	11.06067	11.23903	11.28848	11.26626	11.30196	11.10846	11.03387	11.20342	11.1598	Ddx5	NM_007840
6791970	2982	9.13434	9.319546	9.114931	9.151386	-0.01705	0.81813	0.16816	0.047085	-0.03645	0.624994	0.204615	0.021389	-0.18521	0.032509	0.019409	0.793546	6791970	9.074301	9.227319	9.101401	9.231469	9.452056	9.275113	9.187023	9.119492	9.038278	9.104955	9.232836	9.116366	Smurf2	NM_025481
6791989	2984	9.159876	9.167811	9.07814	9.159723	0.000153	0.999145	0.008088	0.954815	-0.08158	0.57169	0.089671	0.53506	-0.00794	0.955669	0.081736	0.570984	6791989	9.18044	9.158429	9.140759	9.183242	9.249769	9.070423	8.868712	9.448115	8.917594	9.120516	9.136593	9.22206	Bptf	NM_176850
6791992	2985	7.279326	7.316227	7.327295	7.240854	0.038473	0.752915	0.075373	0.541093	0.086441	0.485014	-0.01107	0.927622	-0.0369	0.762644	-0.04797	0.69521	6791992	7.299141	7.323049	7.215789	7.044825	7.586734	7.317121	7.366	7.271081	7.344804	7.162948	7.274188	7.285425	Nol11	NM_133702
6791995	2986	7.426125	7.417675	7.293682	7.115597	0.310528	0.05136	0.302078	0.056602	0.178085	0.225716	0.123993	0.387474	0.008449	0.95187	0.132443	0.357558	6791995	7.570651	7.273804	7.433919	7.384769	7.655919	7.212338	7.329687	7.132051	7.419308	6.971259	7.154054	7.221478	Pitpnc1	NM_145823
6792019	2987	8.868538	8.989949	8.94524	9.672789	-0.80425	0.112693	-0.68284	0.168846	-0.72755	0.145716	0.044709	0.923547	-0.12141	0.794783	-0.0767	0.869304	6792019	8.187347	8.869829	9.548438	9.345514	8.659942	8.964392	9.226306	8.155947	9.453468	10.10301	9.608453	9.306903	Cacng1	NM_007582
6792024	2988	10.43812	10.52894	10.25376	10.84511	-0.40699	0.134756	-0.31617	0.232298	-0.59136	0.042031	0.275183	0.29327	-0.09082	0.7201	0.184363	0.47268	6792024	10.11427	10.79104	10.40905	10.52185	10.74372	10.32125	10.20943	10.23749	10.31435	11.15928	11.03778	10.33828	Cacng4	NM_019431
6792031	2990	6.675101	6.879717	6.524272	6.154567	0.520533	0.080361	0.72515	0.023634	0.369705	0.192997	0.355445	0.208936	-0.20462	0.454145	0.150829	0.577959	6792031	6.507298	6.555687	6.962317	6.750391	7.277363	6.611398	6.751257	6.124139	6.69742	5.936114	6.515694	6.011894	Prkca	NM_011101
6792079	2991	6.820924	7.264376	6.987974	7.35665	-0.53573	0.008652	-0.09227	0.568409	-0.36868	0.044787	0.276402	0.112652	-0.44345	0.021198	-0.16705	0.312953	6792079	6.746893	6.780165	6.935713	7.402929	7.02662	7.36358	6.866624	7.100079	6.997219	7.034656	7.519163	7.51613	Rgs9	NM_011268
6792097	2992	7.548314	7.418989	7.570548	7.35581	0.192504	0.347177	0.063179	0.751494	0.214738	0.297598	-0.15156	0.454354	0.129325	0.521153	-0.02223	0.911011	6792097	7.682317	7.591735	7.37089	7.432435	7.546596	7.277936	7.478703	7.886118	7.346822	7.098371	7.708882	7.260176	Slc16a6	NM_001029842
6792100	2993	8.292041	8.064475	8.089022	8.0686	0.223441	0.096548	-0.00412	0.973131	0.020423	0.867669	-0.02455	0.841331	0.227566	0.091516	0.203018	0.125569	6792100	8.347774	8.188588	8.33976	8.207621	8.223281	7.762523	8.098082	8.168655	8.00033	8.057228	8.102755	8.045816	Wipi1	NM_145940
6792107	2994	6.737462	6.596251	6.502915	6.34876	0.388702	0.022418	0.247491	0.110069	0.154155	0.29551	0.093336	0.517094	0.141211	0.335234	0.234547	0.126985	6792107	6.484581	6.975123	6.752683	6.517832	6.786627	6.484295	6.512975	6.422002	6.573768	6.316216	6.504296	6.225769	Fam20a	NM_153782
6792113	2995	7.746524	7.524942	7.306805	6.974926	0.771598	0.060494	0.550016	0.158144	0.33188	0.375048	0.218136	0.554131	0.221582	0.548033	0.439718	0.248517	6792113	7.437125	7.891463	7.910983	7.445189	7.538776	7.59086	7.686645	6.479992	7.753779	6.61833	7.403072	6.903375	Abca8b	NM_013851
6792114	2996	6.871888	6.444583	5.769551	5.884713	0.987176	0.030535	0.55987	0.175253	-0.11516	0.76747	0.675032	0.110695	0.427306	0.289183	1.102338	0.019046	6792114	7.060264	6.860473	6.694928	6.745949	6.632542	5.955257	6.198023	5.743997	5.366632	5.120193	6.41633	6.117615	Abca8a	NM_153145
6792122	2997	7.390159	6.861559	6.681216	6.300114	1.090045	5.9E-05	0.561445	0.00423	0.381102	0.027842	0.180343	0.240069	0.5286	0.005872	0.708943	0.001067	6792122	7.530258	7.321273	7.318947	6.920398	7.060751	6.603529	6.822912	6.587289	6.633447	6.241308	6.512239	6.146796	Abca9	NM_147220
6792129	2999	6.453401	6.743688	7.003062	6.771069	-0.31767	0.011757	-0.02738	0.786689	0.231994	0.045163	-0.25937	0.029209	-0.29029	0.017954	-0.54966	0.0005	6792129	6.520993	6.48031	6.358901	6.857185	6.711897	6.661982	7.123404	6.987891	6.897892	6.635416	6.954443	6.723347	Abca5	NM_147219
6792296	3002	7.534808	7.431716	7.4202	7.388067	0.146741	0.208549	0.043649	0.694769	0.032134	0.772169	0.011516	0.917161	0.103092	0.364712	0.114608	0.316556	6792296	7.644114	7.431377	7.528933	7.431876	7.394267	7.469005	7.450521	7.289031	7.521049	7.196161	7.363401	7.604638	Slc39a11	NM_027216
6792297	3003	8.347513	8.452366	8.180901	8.256722	0.090791	0.486871	0.195644	0.154911	-0.07582	0.559613	0.271465	0.060928	-0.10485	0.424369	0.166612	0.217818	6792297	8.470118	8.175294	8.397127	8.258852	8.491152	8.607095	8.345429	8.081807	8.115468	8.115191	8.277828	8.377148	D11Wsu99	NM_138598
6792310	3006	7.633187	7.520019	7.854852	7.688329	-0.05514	0.592567	-0.16831	0.127349	0.166523	0.130875	-0.33483	0.009584	0.113168	0.285793	-0.22167	0.055399	6792310	7.591851	7.624797	7.682914	7.611709	7.613032	7.335317	7.907657	7.957192	7.699708	7.633576	7.615142	7.81627	Cdc42ep4	NM_020006
6792352	3009	6.943456	6.979973	6.952692	6.917756	0.0257	0.767116	0.062217	0.479424	0.034936	0.687979	0.027282	0.753323	-0.03652	0.674795	-0.00924	0.915032	6792352	6.87219	6.978884	6.979294	6.934215	6.951293	7.054412	7.045035	7.048704	6.764336	6.955194	6.818359	6.979715	Btbd17	NM_028055
6792367	3010	6.680962	6.353123	6.527557	5.762722	0.91824	0.004699	0.590401	0.037383	0.764834	0.01208	-0.17443	0.482536	0.327839	0.20375	0.153405	0.535381	6792367	6.899961	6.711149	6.431776	6.314427	6.774524	5.970419	6.806285	6.272028	6.504357	5.530396	5.800248	5.957523	Cd300lb	NM_199221
6792371	3012	7.678036	7.494904	7.446345	6.795031	0.883005	0.023771	0.699874	0.058375	0.651314	0.074088	0.048559	0.882102	0.183132	0.579533	0.231691	0.485901	6792371	8.2455	7.542397	7.246211	7.489653	7.711568	7.283492	7.504774	7.023628	7.810633	6.410982	7.148934	6.825176	4732429D	NM_145437
6792416	3016	6.319085	6.42285	6.403641	6.533462	-0.21438	0.277562	-0.11061	0.564394	-0.12982	0.500509	0.019209	0.919426	-0.10376	0.588266	-0.08456	0.658089	6792416	6.125482	6.394945	6.436829	6.579701	6.332644	6.356205	6.40879	6.710943	6.091189	6.318727	6.481506	6.800153	Nat9	NM_025400
6792418	3017	6.095165	6.135778	6.229391	6.112389	-0.01722	0.784752	0.02339	0.711267	0.117002	0.09127	-0.09361	0.16326	-0.04061	0.524102	-0.13423	0.058868	6792418	6.137786	5.999104	6.148606	6.148036	6.137602	6.121697	6.230851	6.342858	6.114464	6.070528	6.104615	6.162023	Grin2c	NM_010350
6792419	3018	6.84771	6.822459	6.664754	6.761668	0.086042	0.434828	0.060792	0.577332	-0.09691	0.381535	0.157705	0.170276	0.02525	0.815421	0.182956	0.118574	6792419	6.958885	6.758751	6.825493	6.759092	6.833812	6.874474	6.861384	6.707987	6.424891	6.811471	6.70484	6.768692	Fdxr	NM_007997
6792421	3019	6.592416	6.513196	6.369457	6.565262	0.027153	0.798579	-0.05207	0.626572	-0.1958	0.093606	0.143738	0.20006	0.07922	0.463582	0.222958	0.062173	6792421	6.629334	6.575904	6.572009	6.675069	6.451835	6.412683	6.393451	6.560603	6.154318	6.544561	6.597367	6.553859	Fads6	NM_178035

6792424	3020	6.110454	6.241619	6.333952	6.181278	-0.07082	0.627472	0.060341	0.67863	0.152675	0.308419	-0.09233	0.529138	-0.13116	0.377423	-0.2235	0.149998	6792424	6.090294	5.941572	6.299496	6.2328	6.123127	6.368929	6.449756	6.379991	6.17211	6.39726	5.951318	6.195255	Ush1g	NM_176847
6792427	3021	8.080361	7.922302	8.564024	8.231753	-0.15139	0.562345	-0.30945	0.251749	0.332271	0.221308	-0.64172	0.033554	0.158059	0.545656	-0.48366	0.089609	6792427	8.089731	8.035293	8.116058	8.02089	8.008038	7.737978	8.229197	8.971084	8.491791	8.409694	8.571755	7.71381	C630004H	BC024617
6792441	3022	9.484094	9.393441	9.416323	9.057389	0.426706	0.046533	0.336052	0.101111	0.358934	0.083244	-0.02288	0.902743	0.090653	0.630745	0.067771	0.718435	6792441	9.377867	9.528057	9.546359	9.266228	9.673163	9.240932	9.777741	9.21918	9.252048	8.971847	9.262523	8.937796	Nt5c	NM_015807
6792442	3023	8.95465	8.811898	8.949499	8.928993	0.025657	0.897242	-0.11709	0.559791	0.020506	0.917774	-0.1376	0.494971	0.142751	0.479466	0.005151	0.979304	6792442	9.071979	8.817324	8.974646	8.659918	9.045602	8.730175	9.236856	8.896625	8.715015	8.769125	9.281312	8.736542	Hn1	NM_008258
6792452	3024	7.195502	7.192231	7.241749	7.253284	-0.05778	0.752443	-0.06105	0.739021	-0.01154	0.949633	-0.04952	0.786743	0.003271	0.985706	-0.04625	0.800464	6792452	7.293995	7.144517	7.147995	7.227323	7.267668	7.081702	6.85412	7.662113	7.209014	7.156897	7.312256	7.2907	Gga3	NM_173048
6792455	3025	7.956196	7.942114	7.976487	8.057185	-0.10099	0.548408	-0.11507	0.495571	-0.0807	0.630087	-0.03437	0.836459	0.014082	0.932525	-0.02029	0.902926	6792455	8.071074	8.007783	7.789732	8.03166	8.061018	7.733664	7.853417	8.251324	7.824721	7.919105	7.952643	8.299808	Mif4gd	NM_027162
6792456	3026	8.200615	8.079022	7.987286	7.944057	0.256558	0.201126	0.134965	0.484614	0.043229	0.820334	0.091736	0.631841	0.121593	0.527691	0.213329	0.280174	6792456	8.030718	8.111167	8.45996	7.978924	8.413287	7.844854	8.0231	8.110229	7.828529	7.729929	7.961685	8.140557	Slc25a19	NM_026071
6792458	3027	7.903166	7.961188	7.999095	7.916598	-0.01343	0.904224	0.044589	0.690973	0.082496	0.467509	-0.03791	0.735028	-0.05802	0.606234	-0.09593	0.400989	6792458	7.976057	7.95096	7.78248	7.963892	7.974392	7.945279	8.143911	8.095556	7.757817	7.814131	7.885556	8.050108	Grb2	NM_008163
6792476	3028	7.411268	7.556676	7.424763	7.537805	-0.12654	0.375094	0.018871	0.892067	-0.11304	0.425786	0.131913	0.356184	-0.14541	0.31191	-0.01349	0.922677	6792476	7.384306	7.419771	7.429727	7.676353	7.604588	7.389087	7.329655	7.722461	7.222172	7.39367	7.577606	7.64214	Caskin2	NM_080643
6792477	3029	6.517926	6.601799	6.524692	6.613429	-0.0955	0.100212	-0.01163	0.826661	-0.08874	0.122526	0.077107	0.17194	-0.08387	0.141346	-0.00677	0.898516	6792477	6.594456	6.519802	6.439521	6.573293	6.571106	6.660998	6.516639	6.558892	6.498546	6.567249	6.568274	6.704763	Recql5	NM_130454
6792485	3030	7.391671	7.517045	7.321415	7.37521	0.016461	0.879253	0.141835	0.213532	-0.05379	0.622106	0.195629	0.099322	-0.12537	0.266467	0.070256	0.522082	6792485	7.297882	7.435214	7.441918	7.55704	7.553833	7.440261	7.34717	7.541067	7.076009	7.351783	7.395779	7.378068	Galk1	NM_016905
6792486	3031	9.493667	9.758828	9.585017	9.483203	0.010464	0.95858	0.275625	0.195804	0.101814	0.616225	0.17381	0.399408	-0.26516	0.211567	-0.09135	0.652412	6792486	9.616178	9.725374	9.139449	9.616439	9.747497	9.912547	9.867631	9.315034	9.572387	9.28657	9.516363	9.646676	H3f3b	NM_008211
6792491	3032	6.187217	6.181983	6.337256	6.200458	-0.01324	0.903765	-0.01847	0.86609	0.136798	0.233306	-0.15527	0.181491	0.005234	0.961862	-0.15004	0.195038	6792491	6.256739	6.097695	6.207218	6.369884	6.130413	6.045652	6.449966	6.295697	6.266106	6.31207	6.26107	6.028234	Unc13d	NM_001009573
6792493	3033	9.304341	8.919212	9.232612	9.349067	-0.04473	0.834998	-0.42986	0.072427	-0.11645	0.590603	-0.3134	0.170005	0.385129	0.101004	0.071729	0.738902	6792493	9.358352	9.292113	9.262558	9.233875	8.858102	8.665658	8.87062	9.657516	9.169701	9.232386	9.334812	9.480003	Wbp2	NM_016852
6792494	3034	7.40447	7.256016	7.355895	7.021043	0.383427	0.021662	0.234973	0.119467	0.334852	0.037867	-0.09988	0.479895	0.148454	0.302785	0.048575	0.727909	6792494	7.522292	7.217808	7.473309	7.347939	7.371895	7.048213	7.551887	7.290307	7.225491	6.959076	7.18248	6.921573	Trim47	NM_172570
6792496	3035	7.488239	7.450689	7.328862	7.366558	0.121681	0.435086	0.08413	0.585567	-0.0377	0.805499	0.121827	0.434556	0.03755	0.806234	0.159377	0.313229	6792496	7.47194	7.570609	7.422168	7.473102	7.659975	7.218989	7.038686	7.546867	7.401033	7.440936	7.394751	7.263988	Trim65	NM_178802
6792497	3036	8.207341	8.138498	8.188432	8.237762	-0.03042	0.76967	-0.09926	0.351866	-0.04933	0.636455	-0.04993	0.632388	0.068843	0.512368	0.018909	0.855331	6792497	8.186157	8.008299	8.427568	8.056942	8.201093	8.15746	8.283954	8.183016	8.098327	8.222242	8.289568	8.201475	Mrpl38	NM_024177
6792499	3037	7.025691	7.061032	6.969708	6.995978	0.029713	0.485494	0.065054	0.148047	-0.02627	0.536041	0.091324	0.054779	-0.03534	0.409776	0.055984	0.205582	6792499	7.048412	7.08112	6.947542	7.054581	7.086709	7.041806	6.967244	7.00349	6.938389	6.966661	6.957585	7.063689	Fbf1	NM_172571
6792500	3038	9.509964	9.483101	9.443178	9.58328	-0.07332	0.602593	-0.10018	0.480119	-0.1401	0.33063	0.039923	0.77541	0.026864	0.847538	0.066786	0.634773	6792500	9.523146	9.361795	9.644952	9.558995	9.454799	9.435508	9.558512	9.415671	9.355351	9.329818	9.548347	9.871675	Acox1	NM_015729
6792501	3039	7.103262	7.243951	7.182582	7.184262	-0.081	0.647852	0.059689	0.735641	-0.00168	0.992391	0.061368	0.728548	-0.14069	0.433767	-0.07932	0.654585	6792501	7.133846	7.050344	7.125597	7.28163	7.215199	7.235023	7.260136	7.086443	7.201168	7.053361	7.638219	6.861206	Evpl	NM_025276
6792502	3040	8.404279	8.426107	8.676681	8.496226	-0.09195	0.275651	-0.07012	0.398283	0.180455	0.050757	-0.25057	0.012829	-0.02183	0.788229	-0.2724	0.008487	6792502	8.315792	8.365776	8.531269	8.324898	8.480638	8.472786	8.728751	8.655007	8.646285	8.407928	8.633832	8.446917	Srp68	NM_146032
6792506	3041	8.090742	7.985166	7.870366	7.935723	0.155019	0.138599	0.049443	0.614049	-0.06536	0.507611	0.1148	0.25786	0.105576	0.295087	0.220376	0.047528	6792506	7.952303	8.056092	8.263832	7.953684	8.146884	7.85493	7.813333	7.872688	7.925077	7.873225	7.963103	7.970842	Exoc7	NM_016857
6792516	3043	8.571143	8.561407	8.486373	8.538151	0.032992	0.473148	0.023256	0.61008	-0.05178	0.271368	0.075034	0.125247	0.009736	0.829767	0.08477	0.089142	6792516	8.523653	8.540144	8.649633	8.504744	8.617817	8.561661	8.456154	8.539707	8.463259	8.531949	8.502974	8.57953	Prpsap1	BC029621
6792527	3044	7.226709	7.221881	7.110633	7.217449	0.00926	0.946671	0.004432	0.974459	-0.10682	0.448961	0.111248	0.431083	0.004828	0.972176	0.116077	0.412175	6792527	7.306157	7.184649	7.189322	7.274086	7.312992	7.078565	7.139266	7.367626	6.825006	7.158534	7.143599	7.350214	Ube2o	NM_173755
6792534	3045	7.696085	7.647278	7.693267	7.430153	0.265932	0.099097	0.217125	0.166239	0.263114	0.102151	-0.04599	0.755268	0.048807	0.740894	0.002818	0.984711	6792534	7.89478	7.596247	7.597229	7.584212	7.925407	7.432216	7.721427	7.780849	7.577525	7.356362	7.583316	7.350781	Rhbdf2	NM_172572
6792539	3046	9.222534	9.066478	8.598489	8.648505	0.574029	0.031524	0.417974	0.094758	-0.05002	0.826337	0.467989	0.066693	0.156055	0.499429	0.624045	0.022197	6792539	9.490978	9.188966	8.987657	9.085458	9.224886	8.889091	8.678782	8.532184	8.584501	8.177166	9.049267	8.719081	Cygb	NM_030206
6792544	3047	8.84527	8.443572	8.118111	8.673931	0.171339	0.678286	-0.23036	0.578769	-0.55582	0.200204	0.325461	0.437313	0.401698	0.342521	0.727159	0.105203	6792544	8.470606	8.733751	9.331452	8.089107	8.891593	8.350016	8.851357	7.322453	8.180523	8.64527	8.657927	8.718596	St6galna	NM_009180
6792547	3048	6.243899	6.162705	6.081305	5.895464	0.348435	0.099255	0.267241	0.190598	0.185841	0.349148	0.0814	0.67467	0.081193	0.675441	0.162594	0.409639	6792547	6.098907	6.137568	6.495221	6.331606	6.020431	6.136079	6.182666	5.692423	6.368826	6.03195	5.795312	5.85913	St6galna	NM_011371
6792550	3049	7.851395	7.763807	7.631756	7.596667	0.254728	0.052935	0.16714	0.174783	0.035089	0.762565	0.132052	0.273215	0.087587	0.457651	0.219639	0.086077	6792550	7.887409	7.697215	7.96956	7.656447	7.914743	7.720232	7.816214	7.622768	7.456285	7.542874	7.681458	7.565669	Mxra7	NM_026280
6792553	3050	7.753594	7.779636	7.697958	7.822384	-0.06879	0.549312	-0.04275	0.707807	-0.12443	0.290945	0.081677	0.479177	-0.02604	0.818881	0.055636	0.626787	6792553	7.6325	7.780377	7.847906	7.693997	7.900898	7.744012	7.719858	7.9045	7.469517	7.807874	7.797277	7.862001	Jmjd6	NM_033398
6792554	3051	10.21152	10.29603	10.26119	10.28205	-0.07053	0.534169	0.013977	0.900754	-0.02087	0.85239	0.034843	0.756506	-0.08451	0.458791	-0.04966	0.659553	6792554	10.1763	10.15331	10.30496	10.14075	10.52408	10.22326	10.35794	10.28992	10.1357	10.19203	10.39267	10.26146	Sfrs2	NM_011358
6792595	3052	7.59924	7.647689	7.73113	7.516008	0.083231	0.382544	0.13168	0.181948	0.215121	0.043992	-0.08344	0.381405	-0.04845	0.605335	-0.13189	0.181328	6792595	7.729585	7.564203	7.503931	7.767813	7.640516	7.534737	7.835581	7.659648	7.69816	7.6174	7.537123	7.393502	Tmc6	NM_145439
6792606	3054	6.689655	6.669884	6.648192	6.679708	0.009947	0.911705	-0.00982	0.912786	-0.03152	0.72628	0.021692	0.809197	0.019771	0.825754	0.041463	0.646073	6792606	6.577881	6.639736	6.851347	6.689	6.667244	6.653407	6.715366	6.672107	6.557102	6.61695	6.58947	6.832704	Tha1	NM_027919
6792614	3055	8.312239	8.111701	8.267574	7.121974	1.190265	0.022371	0.989727	0.046839	1.1456	0.026346	-0.15587	0.721155	0.200538	0.646994	0.044665	0.918227	6792614	8.639194	8.295501	8.002021	7.697741	8.578156	8.059205	8.801092	7.653554	8.348075	7.196931	7.740522	6.428469	Socs3	NM_007707
6792638	3056	8.380271	8.558404	8.463848	8.381344	-0.00107	0.99392	0.17706	0.23082	0.082504	0.562379	0.094556	0.508173	-0.17813	0.228256	-0.08358	0.557418	6792638	8.576107	8.382406	8.182299	8.646324	8.498505	8.530383	8.515967	8.557666	8.317912	8.285684	8.220034	8.638314	Cyth1	NM_011180
6792649	3057	10.54288	10.28291	9.811919	9.437051	1.105829	0.024524	0.845859	0.067433	0.374867	0.376197	0.470991	0.272942	0.25997	0.534042	0.730961	0.105119	6792649	10.66548	10.1392	10.82396	10.15905	10.98811	9.70157	10.35289	9.600728	9.482138	9.067227	9.906643	9.337284	Timp2	NM_011594
6792657	3059	10.05194	9.817207	10.01288	9.396014	0.655927	0.162031	0.421193	0.351565	0.61687	0.185465	-0.19568	0.658083	0.234734	0.596515	0.039057	0.929173	6792657	9.911587	9.971487	10.27275	9.674165	10.50912	9.268337	10.30216	9.149202	10.58729	9.273924	9.700508	9.213611	Lgals3bp	NM_011150
6792658	3060	7.725571	7.740379	7.817044	7.664236	0.061335	0.496464	0.076143	0.402281	0.152807	0.113841	-0.07666	0.399208	-0.01481	0.867716	-0.09147	0.31905	6792658	7.872884	7.758258	7.545572	7.716716	7.818303	7.686118	7.840773	7.905425	7.704933	7.647863	7.634612	7.710234	Cant1	NM_029502
6792679	3062	6.807604	6.866025	6.741414	6.606309	0.201295	0.287008	0.259716	0.179313	0.135105	0.465908	0.124611	0.500159	-0.05842	0.749114	0.06619	0.717357	6792679	6.626477	6.870694	6.925641	6.827589	6.974496	6.79599	6.640557	7.085312	6.498373	6.760986	6.327163	6.730777	Cbx8	NM_013926
6792685	3063	7.932397	8.162493	7.925753	8.049318	-0.11692	0.571157	0.113175	0.583304	-0.12356	0.549971	0.23674	0.266075	-0.2301	0.278697	0.006644	0.974056	6792685	7.836573	8.075063	7.885554	8.153284	8.338742	7.995453	7.92719	8.322933	7.527136	8.135451	7.845148	8.167354	Cbx4	NM_007625
6792691	3064	7.240938	7.321353	7.448908	7.492249	-0.25131	0.130272	-0.1709	0.284702	-0.04334	0.778618	-0.12756	0.417017	-0.08041	0.604226	-0.20797	0.200446	6792691	7.198869	7.345027	7.178919	7.50298	7.104871	7.356208	7.357169	7.763038	7.226518	7.44004	7.453382	7.583326	Tbc1d16	NM_172443
6792699	3065	6.243521	6.386813	6.521315	6.241357	0.002165	0.99135	0.145456	0.47383	0.279958	0.186035	-0.1345	0.506745	-0.14329	0.480221	-0.27779	0.189098	6792699	6.334499	6.11241	6.283655	6.394176	6.506803	6.259459	6.848044	6.232887	6.483014	5.876031	6.431319	6.41672	Eif4a3	NM_138669
6792702	3066	8.212843	8.114452	8.162513	8.064233	0.148609	0.139343	0.050219	0.594283	0.098279	0.309325	-0.04806	0.609954	0.09839	0.308814	0.05033	0.593484	6792702	8.368782	8.185111	8.084635	8.10767	8.260513	7.975174	8.144582	8.112071	8.230885	7.995543	8.125475	8.071682	Sgsh	NM_018822
6792708	3067	10.71013	10.80727	10.4706	11.57202	-0.86189	0.06009	-0.76475	0.088133	-1.10142	0.02333	0.336672	0.41755	-0.09714	0.811409	0.239531	0.559969	6792708	9.862085	10.88589	11.38242	10.66343	11.16337	10.59502	9.984803	10.93466	10.49234	11.47107	11.61876	11.62624	Nptx1	NM_008730
6792733	3068	7.785982	7.750876	7.515863	7.128306	0.657675	0.001162	0.62257	0.001626	0.387557	0.019889	0.235013	0.116697	0.035106	0.799425	0.270118	0.077903	6792733	7.88237	7.779143	7.696432	7.712917	7.913808	7.625903	7.81071	7.295067	7.441813	7.16311	7.182868	7.038941	Aatk	NM_007377
6792738	3069	7.419287	7.517193	7.369748	7.472411	-0.05312	0.27101	0.044782	0.348103	-0.10266	0.051673	0.147445	0.011161	-0.09791	0.060952	0.049539	0.30228	6792738	7.481919	7.435774	7.340168	7.498806	7.534917	7.517857	7.414225	7.328312	7.366708	7.428944	7.436788	7.551502	Azi1	NM_009734
6792739	3070	7.751976	7.736869	7.733763	7.609384	0.142592	0.14544	0.127485	0.187285	0.124379	0.19711	0.003106	0.972838	0.015107	0.868564	0.018213	0.841934	6792739	7.816255	7.748423	7.691249	7.69136	7.842524	7.676722	7.701406	7.910281	7.589602	7.626741	7.689223	7.512187	2410002I	NM_183137
6792742	3071	9.529841	9.509416	9.600442	9.540042	-0.0102	0.929982	-0.03063	0.792343	0.0604	0.605949	-0.09103	0.441846	0.020425	0.860449	-0.0706	0.547781	6792742	9.498719	9.582653	9.508151	9.443464	9.399272	9.685513	9.748226	9.503201	9.549898	9.366629	9.729696	9.5238	Slc38a10	NM_024249
6792756	3072	7.707056	7.686391	7.586268	7.514982	0.192074	0.219407	0.171409	0.268487	0.071286	0.634223	0.100123	0.506981	0.020665	0.889553	0.120788	0.426386	6792756	7.751648	7.585256	7.784263	7.711907	7.919274	7.427992	7.613888	7.651873	7.493042	7.472561	7.746485	7.325899	2310003H	NM_027980
6792760	3073	9.481079	9.469047	9.467285	9.406476	0.074603	0.447298	0.062572	0.52158	0.060809	0.533075	0.001762	0.985401	0.012032	0.900636	0.013794	0.886193	6792760	9.528642	9.393698	9.520897	9.468265	9.60528	9.333597	9.453769	9.605593	9.342493	9.319217	9.520432	9.379778	Nploc4	NM_199469
6792769	3075	6.403372	6.467614	6.630657	6.370118	0.033253	0.784328	0.097495	0.430684	0.260539	0.057392	-0.16304	0.202629	-0.06424	0.599423	-0.22729	0.089085	6792769	6.518535	6.265851	6.425729	6.572059	6.523026	6.307756	6.635569	6.661551	6.594852	6.601803	6.18132	6.327232	1810049H	NM_025560
6792770	3076	6.331432	6.216403	6.296262	6.276975	0.054457	0.47645	-0.06057	0.430145	0.019287	0.798036	-0.07986	0.305207	0.115029	0.153251	0.03517	0.642427	6792770	6.365134	6.236153	6.393008	6.137877	6.290302	6.22103	6.193876	6.408623	6.286287	6.268845	6.194972	6.367108	Arl16	NM_197995
6792779	3077	11.50121	11.55292	11.56171	11.56308	-0.06187	0.52015	-0.01016	0.914795	-0.00137	0.988453	-0.00878	0.926274	-0.05171	0.589392	-0.06049	0.529213	6792779	11.56811	11.50154	11.43399	11.45244	11.53932	11.66701	11.74381	11.54115	11.40016	11.51706	11.64255	11.52963	P4hb	NM_011032
6792781	3078	11.28507	11.19786	11.46921	11.13005	0.155023	0.339615	0.06781	0.668685	0.339163	0.057038	-0.27135	0.113391	0.087213	0.583502	-0.18414	0.262208	6792781	11.35929	11.23463	11.26129	11.26259	11.38778	10.9432	11.56815	11.65065	11.18883	11.05457	11.3021	11.03347	Arhgdia	NM_133796
6792784	3079	9.016569	9.19036	9.275385	8.750937	0.265632	0.1655	0.439423	0.035556	0.524448	0.016736	-0.08502	0.638318	-0.17379	0.347297	-0.25882	0.175345	6792784	9.081566	8.836043	9.132098	9.224159	9.175282	9.171639	9.562702	9.415239	8.848213	8.803242	8.617567	8.832002	Thoc4	NM_011568
6792786	3080	7.308786	7.527242	7.413953	7.612977	-0.30419	0.061376	-0.08573	0.55696	-0.19902	0.192599	0.11329	0.441426	-0.21846	0.156982	-0.10517	0.473697	6792786	7.316884	7.248619	7.360856	7.501872	7.658373	7.421482	7.479058	7.63181	7.13099	7.473741	7.543782	7.821407	Pcyt2	NM_024229
6792787	3081	7.565367	7.601857	7.566624	7.45094	0.114428	0.176903	0.150917	0.086568	0.115685	0.172718	0.035232	0.660531	-0.03649	0.649367	-0.00126	0.987419	6792787	7.464881	7.571753	7.659468	7.509704	7.69132	7.604546	7.68094	7.569005	7.449928	7.381275	7.452783	7.518761	Sirt7	NM_153056
6792789	3082	7.473557	7.529012	7.432065	7.350311	0.123246	0.347841	0.178701	0.186194	0.081754	0.526869	0.096947	0.455368	-0.05545	0.66554	0.041493	0.7457	6792789	7.626889	7.231976	7.561807	7.650796	7.461059	7.475181	7.283447	7.629943	7.382804	7.410664	7.352824	7.287445	Mafg	NM_010756
6792792	3083	7.013038	6.93307	6.947698	7.147417	-0.13438	0.189195	-0.21435	0.051343	-0.19972	0.065507	-0.01463	0.879733	0.079969	0.417949	0.06534	0.505092	6792792	6.988857	6.892798	7.15746	6.970071	6.890355	6.938783	7.142353	6.868114	6.832627	7.205102	7.075903	7.161246	Pycr1	NM_144795
6792808	3086	8.585481	8.52176	8.631546	8.546725	0.038756	0.670531	-0.02496	0.78333	0.084821	0.3622	-0.10979	0.24639	0.063721	0.488584	-0.04606	0.613969	6792808	8.369125	8.672774	8.714544	8.487665	8.568712	8.508904	8.691923	8.640346	8.562368	8.545227	8.47916	8.615787	Stra13	NM_016665
6792813	3087	7.821781	7.935867	7.711709	8.364445	-0.54266	0.044698	-0.42858	0.097216	-0.65274	0.021147	0.224158	0.354767	-0.11409	0.630608	0.110073	0.642505	6792813	7.753736	7.746384	7.965224	8.015954	8.043111	7.748535	7.81027	7.737455	7.587401	8.352614	7.862881	8.87784	Dcxr	NM_026428

6792814	3088	8.263969	9.694817	7.679985	9.039621	-0.77565	0.365957	0.655195	0.441633	-1.35964	0.131497	2.014832	0.037559	-1.43085	0.115064	0.583985	0.491133	6792814	8.286117	7.930218	8.575573	10.73739	10.20026	8.1468	8.361152	7.558811	7.119991	10.0908	9.369475	7.658589	Cbr2	NM_007621
6792819	3089	8.129912	8.031419	8.137754	8.255432	-0.12552	0.136558	-0.22401	0.018333	-0.11768	0.159399	-0.10633	0.198541	0.098492	0.230299	-0.00784	0.920202	6792819	8.119877	8.104216	8.165642	8.143508	8.033093	7.917657	8.100565	8.157764	8.154932	8.098866	8.29733	8.370101	Rfng	NM_009053
6792820	3090	6.993333	7.182035	7.129321	7.033856	-0.04052	0.508029	0.14818	0.035067	0.095465	0.141286	0.052714	0.393773	-0.1887	0.012125	-0.13599	0.048539	6792820	6.951301	6.954753	7.073944	7.180514	7.252585	7.113007	7.210199	7.074033	7.103731	6.947124	7.074206	7.080237	Dus1l	NM_026824
6792822	3091	10.00584	9.697075	9.548825	9.276367	0.729472	0.040138	0.420708	0.195892	0.272458	0.387499	0.14825	0.632385	0.308764	0.330655	0.457014	0.16384	6792822	10.18169	9.619547	10.21628	9.461722	10.33548	9.294023	9.800888	9.471369	9.374218	9.123318	9.55492	9.150864	Fasn	NM_007988
6792825	3092	6.041242	6.257032	6.053726	6.150715	-0.10947	0.138493	0.106317	0.148708	-0.09699	0.182999	0.203306	0.015682	-0.21579	0.011816	-0.01248	0.855828	6792825	5.977832	6.051999	6.093894	6.327469	6.187132	6.256495	5.924881	6.069063	6.167233	6.21558	6.131093	6.105473	Ccdc57	NM_027745
6792827	3093	10.53588	10.52774	10.43444	10.39864	0.137243	0.04869	0.129107	0.060362	0.035807	0.561265	0.0933	0.152939	0.008137	0.893862	0.101437	0.12432	6792827	10.57127	10.51252	10.52385	10.43723	10.6815	10.4645	10.47285	10.42997	10.40051	10.38716	10.37923	10.42952	Csnk1d	NM_027874
6792832	3094	6.317741	5.873581	6.326108	5.689359	0.628382	0.028365	0.184222	0.45632	0.636749	0.026844	-0.45253	0.090729	0.44416	0.095838	-0.00837	0.972511	6792832	6.820826	6.053773	6.078623	5.615108	5.979803	6.025831	6.282997	6.306601	6.388726	5.946926	5.757809	5.363341	Cd7	NM_009854
6792848	3098	8.251228	8.385284	8.459154	8.907328	-0.6561	0.00381	-0.52204	0.012551	-0.44817	0.025059	-0.07387	0.662398	-0.13406	0.434587	-0.20793	0.23781	6792848	8.354855	8.220673	8.178157	8.282883	8.379828	8.493141	8.531927	8.6366	8.208936	8.912163	8.604799	9.205023	1110031I	NM_025402
6792849	3099	7.466725	7.468228	7.393382	7.267191	0.199533	0.006561	0.201037	0.006306	0.126191	0.050165	0.074846	0.208958	-0.0015	0.978771	0.073342	0.217361	6792849	7.465632	7.528769	7.405773	7.408333	7.535198	7.461154	7.482698	7.317713	7.379736	7.201755	7.300446	7.299373	BC017643	BC017643
6792866	3100	7.52722	7.699209	7.4317	7.566688	-0.03947	0.678034	0.132521	0.18612	-0.13499	0.178923	0.267509	0.019308	-0.17199	0.097356	0.09552	0.327676	6792866	7.629724	7.506953	7.444983	7.547069	7.814052	7.736505	7.446265	7.571787	7.277047	7.537269	7.593514	7.569281	Wdr45l	NM_025793
6792887	3102	8.010808	8.100776	8.250319	7.883003	0.127805	0.63498	0.217773	0.424904	0.367317	0.193922	-0.14954	0.579593	-0.08997	0.737301	-0.23951	0.382182	6792887	7.863744	8.310493	7.858187	8.153655	8.227992	7.92068	8.124201	8.280149	8.346608	7.280189	8.339051	8.029768	Zfp750	NM_178763
6792903	3103	6.41317	6.346393	6.513908	6.241292	0.171878	0.203111	0.1051	0.42129	0.272616	0.059125	-0.16752	0.21367	0.066777	0.60486	-0.10074	0.440052	6792903	6.388271	6.372051	6.479188	6.343934	6.446865	6.248379	6.446662	6.334953	6.760109	6.236376	6.418289	6.069212	B3gntl1	NM_178664
6792919	3104	6.378917	6.535429	6.444551	6.423445	-0.04453	0.593844	0.111984	0.200054	0.021106	0.799031	0.090878	0.289866	-0.15651	0.086727	-0.06563	0.436735	6792919	6.202565	6.499835	6.43435	6.567289	6.532148	6.506849	6.501707	6.355259	6.476686	6.473996	6.470303	6.326036	1700012B	BC060685
6792931	3105	7.482316	7.616029	7.499097	7.19894	0.283375	0.038415	0.417088	0.006563	0.300157	0.03058	0.116931	0.337025	-0.13371	0.276507	-0.01678	0.887101	6792931	7.456508	7.371097	7.619342	7.532692	7.828857	7.486537	7.680754	7.391291	7.425247	7.145092	7.258071	7.193658	Kif3c	NM_008445
6792935	3106	8.160507	8.249175	8.26485	8.204035	-0.04353	0.290945	0.045141	0.274698	0.060815	0.152822	-0.01567	0.694564	-0.08867	0.050215	-0.10434	0.026641	6792935	8.116395	8.162834	8.202291	8.203421	8.311428	8.232677	8.222671	8.295773	8.276106	8.17048	8.261403	8.180221	Asxl2	NM_172421
6792942	3107	9.343693	9.413794	9.352331	9.500746	-0.15705	0.041714	-0.08695	0.21675	-0.14842	0.051354	0.061463	0.370933	-0.0701	0.31116	-0.00864	0.897316	6792942	9.209284	9.40314	9.418655	9.430546	9.382215	9.428621	9.282882	9.463752	9.310358	9.475934	9.483036	9.543269	Dtnb	NM_007886
6792945	3108	8.433847	8.556944	8.390693	8.650145	-0.2163	0.071114	-0.0932	0.396283	-0.25945	0.037231	0.166251	0.148546	-0.1231	0.270489	0.043154	0.689058	6792945	8.364847	8.476851	8.459843	8.467989	8.646439	8.556405	8.226918	8.653216	8.291945	8.650564	8.626871	8.673001	Dnmt3a	NM_007872
6792953	3109	5.931994	6.162839	6.039539	6.158622	-0.22663	0.121624	0.004217	0.975086	-0.11908	0.38953	0.1233	0.373769	-0.23085	0.115808	-0.10755	0.435092	6792953	5.675727	6.021736	6.098519	6.278667	5.998455	6.211396	6.191761	6.076766	5.85009	6.170677	6.170999	6.13419	Pomc	NM_008895
6792965	3110	6.845199	7.225725	7.05397	7.276847	-0.43165	0.005299	-0.05112	0.665296	-0.22288	0.085949	0.171755	0.169801	-0.38053	0.010191	-0.20877	0.104014	6792965	6.625742	7.084725	6.825129	7.277282	7.109452	7.29044	7.040257	7.031259	7.090394	7.142424	7.33783	7.350287	Dnajc27	NM_153082
6792970	3111	7.545863	8.067979	7.687679	8.13405	-0.58819	0.012825	-0.06607	0.729457	-0.44637	0.041849	0.3803	0.073154	-0.52212	0.022128	-0.14182	0.464068	6792970	7.448543	7.8594	7.329645	7.822989	8.17584	8.205107	7.694319	7.684186	7.684531	8.191168	8.386841	7.82414	Adcy3	NM_001159537
6792991	3112	8.969621	9.048093	9.016874	9.046329	-0.07671	0.241319	0.001764	0.977497	-0.02945	0.64006	0.031218	0.620469	-0.07847	0.231587	-0.04725	0.458099	6792991	9.031813	9.009615	8.867435	9.019436	9.08075	9.044092	8.946157	9.093861	9.010605	8.945465	9.101871	9.09165	Itsn2	NM_011365
6792999	3114	8.643227	8.671265	8.509863	8.484645	0.158581	0.228176	0.18662	0.163154	0.025217	0.84079	0.161402	0.220748	-0.02804	0.823313	0.133364	0.304363	6792999	8.610778	8.790545	8.528357	8.583474	8.819529	8.610792	8.768929	8.348204	8.412455	8.431223	8.532396	8.490317	0610009D	NM_025323
6793002	3115	6.643465	6.712974	6.702379	6.813882	-0.17042	0.086744	-0.10091	0.281133	-0.1115	0.237386	0.010595	0.906408	-0.06951	0.448947	-0.05891	0.518851	6793002	6.641654	6.661872	6.62687	6.630219	6.806744	6.701958	6.585698	6.851033	6.670405	6.919771	6.864726	6.65715	BC068281	NM_173416
6793170	3117	7.125353	7.519848	7.558469	7.61647	-0.49112	0.040945	-0.09662	0.644847	-0.058	0.781067	-0.03862	0.852973	-0.3945	0.086304	-0.43312	0.064119	6793170	6.78213	7.426757	7.167171	7.534436	7.837998	7.187109	7.505617	7.678134	7.491656	7.573461	7.493659	7.782291	Nfyc	NM_008692
6793181	3118	7.611747	7.754963	7.703451	7.763894	-0.15215	0.28204	-0.00893	0.947683	-0.06044	0.658997	0.051511	0.706361	-0.14322	0.309243	-0.0917	0.506619	6793181	7.376165	7.769607	7.689468	7.547815	7.867799	7.849274	7.688156	7.729407	7.692791	7.587589	7.923342	7.78075	1110057K	BC046986
6793186	3119	7.231692	7.144716	7.197971	7.099743	0.131948	0.308055	0.044973	0.720258	0.098227	0.441172	-0.05325	0.672039	0.086976	0.493461	0.033721	0.78792	6793186	7.167544	7.361377	7.166154	7.248659	7.198525	6.986964	7.255148	7.147187	7.191577	7.077297	6.880904	7.341029	Hs1bp3	NM_021429
6793225	3120	9.251964	9.377234	9.274815	9.225843	0.026121	0.703286	0.151392	0.0514	0.048972	0.480322	0.10242	0.160189	-0.12527	0.094915	-0.02285	0.738705	6793225	9.239811	9.321273	9.194808	9.293593	9.422794	9.415316	9.341557	9.324538	9.158349	9.137239	9.297678	9.242611	Pum2	NM_030723
6793226	3121	7.392545	7.768031	7.098275	7.26985	0.122695	0.773828	0.498181	0.261893	-0.17158	0.688551	0.669756	0.143301	-0.37549	0.389535	0.29427	0.496123	6793226	7.566223	7.295902	7.31551	7.494903	8.513402	7.295788	7.542816	6.561583	7.190425	7.106803	7.904323	6.798423	Sdc1	NM_011519
6793249	3122	10.96298	11.01809	10.90777	11.00185	-0.03888	0.662195	0.016237	0.854475	-0.09409	0.304281	0.110323	0.234046	-0.05511	0.53822	0.05521	0.537525	6793249	11.04141	10.95922	10.8883	10.99399	11.01159	11.04869	11.07672	10.92478	10.7218	10.95202	10.96465	11.08889	Laptm4a	NM_008640
6793283	3124	6.880165	6.552693	6.546218	6.210012	0.670153	0.075205	0.342681	0.326483	0.336206	0.335156	0.006475	0.984728	0.327472	0.347136	0.333947	0.338225	6793283	6.753692	6.963317	6.923485	6.684475	7.188271	5.785332	6.65909	6.628505	6.351059	5.92921	6.549285	6.15154	Osr1	NM_011859
6793326	3126	6.565607	6.796738	6.666003	6.978621	-0.41301	0.159805	-0.18188	0.514218	-0.31262	0.274533	0.130735	0.63692	-0.23113	0.411061	-0.1004	0.716186	6793326	6.545865	6.920937	6.230018	6.701393	6.806642	6.882179	7.016793	6.623782	6.357434	6.514513	7.047232	7.374119	Rdh14	NM_023697
6793370	3127	7.812652	7.829319	7.682049	7.492586	0.320065	0.030175	0.336733	0.024409	0.189463	0.158116	0.14727	0.260759	-0.01667	0.894445	0.130603	0.314486	6793370	7.768219	7.839083	7.830653	7.683873	7.97714	7.826945	7.893703	7.538948	7.613496	7.34426	7.686609	7.44689	Smc6	NM_025695
6793416	3128	7.277615	7.353596	7.416998	6.253405	1.02421	0.001795	1.100191	0.00116	1.163593	0.000816	-0.0634	0.783846	-0.07598	0.742625	-0.13938	0.550204	6793416	7.560993	7.164699	7.107153	7.28378	7.65392	7.123087	7.670759	7.059746	7.520488	6.096114	6.543224	6.120876	Fam49a	NM_029758
6793498	3129	7.318122	7.25654	7.519031	7.250559	0.067563	0.450241	0.005981	0.945703	0.268472	0.013515	-0.26249	0.015029	0.061581	0.489996	-0.20091	0.045933	6793498	7.36559	7.314501	7.274274	7.169507	7.280444	7.31967	7.679679	7.496732	7.380681	7.178303	7.37981	7.193564	Nbas	BC057020
6793639	3130	6.739208	6.764762	6.870353	6.725415	0.013792	0.915885	0.039347	0.763775	0.144937	0.285127	-0.10559	0.428216	-0.02555	0.844987	-0.13115	0.330299	6793639	6.769227	6.464732	6.983664	6.819186	6.713988	6.761113	7.013615	6.791743	6.8057	6.838765	6.654284	6.683197	Ntsr2	NM_008747
6793649	3131	7.249625	7.307572	7.279988	6.995307	0.254319	0.104207	0.312266	0.054544	0.284681	0.074336	0.027585	0.847392	-0.05795	0.687235	-0.03036	0.832285	6793649	7.059772	7.187859	7.501245	7.359771	7.326252	7.236694	7.451045	7.255472	7.133446	6.785374	7.06588	7.134666	E2f6	NM_033270
6793652	3132	9.406815	9.388558	9.267014	8.938473	0.468342	0.010816	0.450085	0.013118	0.328541	0.049165	0.121543	0.416329	0.018257	0.900738	0.1398	0.353087	6793652	9.521211	9.338698	9.360535	9.38406	9.475175	9.306438	9.493584	9.08981	9.217649	8.726187	9.209559	8.879673	Rock2	NM_009072
6793672	3133	12.13397	12.07887	12.11323	12.08846	0.045513	0.768192	-0.00959	0.950327	0.024767	0.872322	-0.03436	0.823702	0.055107	0.721545	0.020747	0.892882	6793672	12.20321	12.12843	12.07028	11.79398	12.06522	12.3774	12.31233	12.08826	11.93909	12.08394	12.18384	11.9976	Pdia6	NM_027959
6793674	3134	6.691476	6.800868	6.724672	6.768432	-0.07696	0.489891	0.032436	0.768142	-0.04376	0.691511	0.076196	0.49406	-0.10939	0.333729	-0.0332	0.762899	6793674	6.848059	6.652047	6.574321	6.891641	6.849468	6.661495	6.634313	6.884182	6.655521	6.635397	6.856682	6.813217	Nol10	NM_001008421
6793683	3135	8.87976	9.03142	8.886756	8.881285	-0.00152	0.993064	0.150135	0.40304	0.005471	0.97512	0.144664	0.419654	-0.15166	0.398494	-0.007	0.96819	6793683	8.633421	8.920463	9.085395	9.290707	9.043515	8.760037	8.941082	8.928606	8.79058	8.660407	9.076015	8.907432	Hpcal1	NM_016677
6793961	3137	6.421053	6.467395	6.755593	6.102117	0.318936	0.1031	0.365278	0.068195	0.653477	0.005471	-0.2882	0.135016	-0.04634	0.795996	-0.33454	0.089768	6793961	6.514899	6.386676	6.361584	6.304305	6.85133	6.24655	7.012784	6.523014	6.730982	6.067894	6.089471	6.148985	Cmpk2	NM_020557
6794059	3138	6.97681	7.121522	6.972314	7.028438	-0.05163	0.41977	0.093084	0.163709	-0.05612	0.382229	0.149209	0.03944	-0.14471	0.044272	0.004496	0.942772	6794059	6.979791	6.986434	6.964205	7.054976	7.134754	7.174837	6.886569	7.001045	7.029327	6.948925	6.979877	7.156512	Rnaseh1	NM_011275
6794063	3139	7.798869	8.378605	8.434393	8.156225	-0.35736	0.232525	0.22238	0.444728	0.278168	0.344109	-0.05579	0.84522	-0.57974	0.069419	-0.63552	0.050693	6794063	7.942282	7.784998	7.669328	8.858127	7.923631	8.354056	8.750916	8.160043	8.392221	8.041852	7.862336	8.564487	Adi1	NM_134052
6794073	3140	7.557771	7.424754	7.534023	7.461583	0.096188	0.293204	-0.03683	0.678018	0.07244	0.421477	-0.10927	0.237081	0.133017	0.158376	0.023748	0.788243	6794073	7.555291	7.559619	7.558403	7.357215	7.528602	7.388445	7.726448	7.482584	7.393037	7.421157	7.549051	7.414542	Tssc1	NM_201357
6794152	3142	7.518664	7.735827	7.459567	7.382417	0.136248	0.18491	0.35341	0.005521	0.07715	0.435176	0.27626	0.018666	-0.21716	0.049513	0.059098	0.546729	6794152	7.517624	7.532527	7.505842	7.693299	7.780887	7.733294	7.565794	7.525671	7.287235	7.19348	7.513931	7.439839	Tmem18	NM_172049
6794181	3143	5.982582	6.084608	5.932001	6.10227	-0.11969	0.479082	-0.01766	0.915466	-0.17027	0.321759	0.152608	0.371565	-0.10203	0.5445	0.050581	0.761745	6794181	5.751328	5.963048	6.233369	6.021366	6.23001	6.002449	5.835675	6.029088	5.931239	6.033509	5.874483	6.398817	Sh3yl1	NM_013709
6794202	3145	9.280077	9.929528	9.272489	10.46848	-1.1884	0.000487	-0.53895	0.033775	-1.19599	0.000467	0.657038	0.014273	-0.64945	0.015072	0.007588	0.972159	6794202	9.231062	9.51466	9.094509	9.941712	9.798711	10.04816	9.215635	9.649706	8.952127	10.61388	10.14102	10.65053	Lamb1-1	NM_008482
6794274	3147	6.292539	6.611494	6.37882	6.531762	-0.23922	0.215622	0.079732	0.665927	-0.15294	0.414995	0.232673	0.227264	-0.31895	0.110767	-0.08628	0.640697	6794274	6.319707	6.344678	6.213232	6.271165	6.962032	6.601284	6.583511	6.145212	6.407738	6.555671	6.391145	6.648469	2010109K	BC061478
6794293	3148	7.920609	7.965297	8.033664	8.102373	-0.18176	0.141818	-0.13708	0.25399	-0.06871	0.55499	-0.06837	0.556915	-0.04469	0.699153	-0.11306	0.340427	6794293	7.774383	8.036794	7.950649	7.861721	8.131841	7.902328	8.223531	7.950786	7.926675	8.203217	8.078838	8.025064	Nampt	NM_021524
6794294	3149	6.135274	6.074489	5.776696	6.137028	-0.00175	0.987519	-0.06254	0.580838	-0.36033	0.010614	0.297793	0.025453	0.060785	0.591289	0.358578	0.010872	6794294	6.03837	6.143533	6.223919	6.06706	6.098052	6.058355	5.721104	5.904941	5.704043	6.29458	6.233635	5.882869	Gdap10	BC052902
6794307	3150	8.520756	8.704314	8.742339	8.579538	-0.05878	0.629954	0.124776	0.31873	0.162801	0.202803	-0.03802	0.754246	-0.18356	0.156433	-0.22158	0.095711	6794307	8.391315	8.62594	8.545014	8.61269	8.719725	8.780527	8.88929	8.796619	8.541107	8.394346	8.726688	8.617579	Sypl	NM_013635
6794331	3151	9.157651	9.056881	9.098756	9.228028	-0.07038	0.389648	-0.17115	0.057913	-0.12927	0.133331	-0.04187	0.603125	0.10077	0.229046	0.058895	0.468409	6794331	9.165251	9.101866	9.205836	9.054727	9.036699	9.079217	8.891664	9.195933	9.20867	9.244633	9.237319	9.202132	Atxn7l1	NM_001033436
6794339	3152	7.885366	7.849315	7.993377	7.614278	0.271088	0.047304	0.235037	0.076842	0.379099	0.011281	-0.14406	0.248596	0.036051	0.763464	-0.10801	0.378146	6794339	7.912339	7.844221	7.899537	7.742053	8.080177	7.725714	8.216242	7.88473	7.879159	7.565411	7.643378	7.634044	Twistnb	NM_172253
6794430	3156	8.695742	8.809532	8.864819	8.767175	-0.07143	0.49203	0.042356	0.680699	0.097643	0.353863	-0.05529	0.592606	-0.11379	0.284565	-0.16908	0.126755	6794430	8.712477	8.77094	8.603808	8.725223	8.831746	8.871626	9.009945	8.77511	8.809401	8.578444	8.803789	8.919293	Snx13	NM_001014973
6794483	3158	5.6305	6.626796	5.752538	6.295227	-0.66473	0.349017	0.331569	0.633127	-0.54269	0.440242	0.874259	0.22713	-0.9963	0.174338	-0.12204	0.859642	6794483	5.95125	5.437277	5.502972	7.9305	5.892077	6.057812	5.78362	6.013181	5.460812	7.570336	5.810278	5.505068	Agr2	NM_011783
6794496	3160	8.686749	8.632253	8.634709	8.449093	0.237656	0.070999	0.18316	0.147413	0.185617	0.142738	-0.00246	0.983366	0.054496	0.645985	0.05204	0.660728	6794496	8.607242	8.77307	8.679935	8.493367	8.789547	8.613845	8.782821	8.472458	8.648849	8.266797	8.540827	8.539654	Ankmy2	NM_146033
6794513	3161	9.553808	9.266108	9.445543	9.402623	0.151185	0.728335	-0.13652	0.753622	0.04292	0.921164	-0.17943	0.680646	0.2877	0.512925	0.108265	0.803194	6794513	9.147287	9.647118	9.867018	9.278276	9.585646	8.934402	9.601242	9.36347	9.371916	9.973967	9.86151	8.372392	Sostdc1	NM_025312
6794550	3163	7.496426	7.170613	6.823624	6.725747	0.77068	0.071736	0.444866	0.265413	0.097877	0.798861	0.346989	0.377645	0.325813	0.406063	0.672803	0.107733	6794550	7.786603	7.684731	7.017945	7.220402	7.556456	6.734981	7.093564	6.640927	6.73638	5.995302	7.252825	6.929113	Meox2	NM_008584
6794568	3165	8.343586	8.733443	8.627303	8.744801	-0.40121	0.054648	-0.01136	0.950796	-0.1175	0.528618	0.10614	0.5683	-0.38986	0.060354	-0.28372	0.150408	6794568	8.330276	8.572292	8.128191	8.42558	9.03959	8.735159	8.855503	8.52116	8.505245	8.821729	8.645573	8.767101	Dgkb	NM_178681
6794572	3166	8.415639	8.449959	8.083244	7.833776	0.581863	0.06755	0.616183	0.055624	0.249468	0.391428	0.366716	0.219635	-0.03432	0.903887	0.332396	0.261865	6794572	8.444956	8.352884	8.449078	8.158875	9.075984	8.115019	8.369827	7.646797	8.233107	7.781654	7.949322	7.770352	Etv1	NM_007960
6794757	3168	8.079067	8.110508	8.135615	8.226227	-0.14716	0.043127	-0.11572	0.095746	-0.09061	0.177604	-0.02511	0.692846	-0.03144	0.621872	-0.05655	0.383239	6794757	8.01831	8.09503	8.123862	8.003036	8.160415	8.168072	8.215847	8.120646	8.070353	8.14193	8.278428	8.258323	Pnpla8	NM_026164

6795024	3171	6.823451	7.15367	6.834952	6.899194	-0.07574	0.628684	0.254476	0.129672	-0.06424	0.681051	0.318718	0.067293	-0.33022	0.059747	-0.0115	0.941021	6795024	6.777164	6.918767	6.774421	6.866725	7.268684	7.325602	6.995482	6.657164	6.852211	6.713639	6.881765	7.102178	G2e3	NM_001015099
6795025	3172	8.07239	8.057942	8.354211	8.148779	-0.07639	0.439285	-0.09084	0.361504	0.205432	0.060046	-0.29627	0.013467	0.014448	0.881482	-0.28182	0.017007	6795025	7.991968	8.191957	8.033245	7.90449	8.200102	8.069235	8.391787	8.3144	8.356447	8.026656	8.294006	8.125675	Scfd1	NM_029825
6795041	3174	7.122991	7.127482	7.143671	7.323217	-0.20023	0.077161	-0.19573	0.082794	-0.17955	0.106569	-0.01619	0.873854	-0.00449	0.964843	-0.02068	0.839368	6795041	7.004115	7.190986	7.173873	7.006818	7.34584	7.029789	7.199913	7.169299	7.061802	7.32306	7.241488	7.405103	Ap4s1	NM_021710
6795068	3175	5.213211	6.049202	5.370452	6.005916	-0.7927	0.011204	0.043286	0.862346	-0.63546	0.030243	0.67875	0.022925	-0.83599	0.008592	-0.15724	0.533642	6795068	4.782428	5.302562	5.554644	5.834375	6.50008	5.81315	5.247022	5.540981	5.323353	5.846395	6.060164	6.111188	Nubpl	NM_029760
6795069	3176	8.729033	8.824474	8.954583	8.807963	-0.07893	0.422581	0.016511	0.864068	0.14662	0.155071	-0.13011	0.201066	-0.09544	0.336715	-0.22555	0.042172	6795069	8.718662	8.757212	8.711224	8.755123	8.932056	8.786243	8.99408	9.12602	8.743649	8.730765	8.871245	8.82188	Arhgap5	NM_009706
6795228	3179	10.59313	10.73826	10.55021	10.68551	-0.09238	0.581681	0.052757	0.751436	-0.1353	0.424865	0.188057	0.276177	-0.14514	0.393428	0.04292	0.796426	6795228	10.60052	10.53357	10.64529	10.86213	10.5319	10.82076	10.36306	10.90722	10.38034	10.5531	10.64676	10.85666	Ppp2r3c	NM_021529
6795233	3180	6.999185	7.016615	7.135127	7.14318	-0.14399	0.277373	-0.12656	0.335599	-0.00805	0.949631	-0.11851	0.365511	-0.01743	0.891289	-0.13594	0.30318	6795233	7.015199	7.133529	6.848827	7.025986	6.992614	7.031246	7.364394	6.871865	7.169123	7.107775	7.070164	7.2516	1110008L	NM_025373
6795234	3181	9.060243	9.101551	9.189669	9.065003	-0.00476	0.967991	0.036548	0.758712	0.124666	0.309824	-0.08812	0.46544	-0.04131	0.72868	-0.12943	0.292927	6795234	8.922471	9.140127	9.118131	9.133722	9.163325	9.007607	9.292885	9.353583	8.922539	8.995035	9.097322	9.102652	Psma6	NM_011968
6795260	3182	6.388134	6.41017	6.320813	6.51773	-0.1296	0.187208	-0.10756	0.265563	-0.19692	0.059784	0.089357	0.349127	-0.02204	0.812441	0.06732	0.475185	6795260	6.401547	6.315811	6.447043	6.470695	6.340983	6.418833	6.226496	6.502429	6.233515	6.430122	6.465437	6.65763	Insm2	NM_020287
6795279	3183	7.567696	7.66098	7.532414	7.5526	0.015096	0.89646	0.10838	0.363077	-0.02019	0.861911	0.128567	0.285677	-0.09328	0.430558	0.035282	0.761584	6795279	7.627082	7.616529	7.459476	7.520138	7.852978	7.609825	7.675803	7.567729	7.353709	7.466709	7.66554	7.525551	Brms1l	NM_001037756
6795328	3184	6.941919	7.199831	7.407679	7.712559	-0.77064	0.007976	-0.51273	0.047827	-0.30488	0.202531	-0.20785	0.371679	-0.25791	0.274051	-0.46576	0.066761	6795328	6.760632	7.26934	6.795786	7.044398	7.596607	6.958489	7.44804	7.399811	7.375187	7.648853	8.034906	7.453918	Pax9	NM_011041
6795451	3187	9.09731	9.181049	9.050563	9.056204	0.041105	0.724562	0.124845	0.299806	-0.00564	0.961277	0.130486	0.279989	-0.08374	0.478382	0.046746	0.688973	6795451	9.075175	9.044896	9.171858	8.945518	9.320237	9.277393	8.934375	9.189066	9.028249	8.955187	9.180086	9.03334	Pnn	NM_008891
6795455	3189	9.140835	9.255917	9.364175	9.402273	-0.26144	0.004322	-0.14636	0.058698	-0.0381	0.582055	-0.10826	0.141812	-0.11508	0.121435	-0.22334	0.009901	6795455	9.064259	9.187298	9.170949	9.269883	9.232875	9.264993	9.334942	9.471659	9.285925	9.306193	9.376702	9.523924	Ctage5	NM_146034
6795593	3191	8.545441	8.678078	8.622912	8.539594	0.005847	0.945847	0.138484	0.135495	0.083318	0.347172	0.055166	0.527018	-0.13264	0.150517	-0.07747	0.380239	6795593	8.455249	8.617915	8.563159	8.573998	8.825907	8.634329	8.630234	8.736042	8.50246	8.471066	8.593961	8.553756	Fam179b	BC157958
6795595	3192	8.281853	8.452246	8.320025	8.276568	0.005286	0.955211	0.175678	0.090273	0.043457	0.646507	0.132221	0.185217	-0.17039	0.098699	-0.03817	0.686601	6795595	8.357246	8.328693	8.159621	8.295036	8.497605	8.564097	8.41889	8.27741	8.263774	8.153938	8.323309	8.352456	Prpf39	NM_177806
6795599	3193	6.109957	6.258967	6.107038	6.074734	0.035223	0.540754	0.184233	0.010205	0.032303	0.574075	0.151929	0.024837	-0.14901	0.026956	0.002919	0.959068	6795599	6.114729	6.091233	6.123909	6.215931	6.356194	6.204776	6.14155	6.09688	6.082683	5.967276	6.163503	6.093424	Fancm	NM_178912
6795771	3196	8.45129	8.346768	8.659584	8.616877	-0.16559	0.370217	-0.27011	0.160056	0.042707	0.81273	-0.31282	0.110644	0.104521	0.565574	-0.20829	0.266586	6795771	8.396211	8.474522	8.483136	8.100089	8.599796	8.34042	8.532566	8.897194	8.548993	8.7763	8.774441	8.299891	Mgat2	NM_146035
6795779	3197	5.852908	5.939575	5.825238	5.732939	0.119969	0.419171	0.206636	0.180582	0.092299	0.530708	0.114337	0.440461	-0.08667	0.555485	0.027669	0.849177	6795779	5.610014	5.901896	6.046813	6.021298	5.737423	6.060005	5.91167	5.876177	5.687868	5.62197	5.666626	5.910221	Klhdc1	NM_178253
6795780	3198	8.331909	8.373539	8.28428	8.264623	0.067285	0.546435	0.108916	0.337873	0.019657	0.858617	0.089259	0.427747	-0.04163	0.706984	0.047629	0.66759	6795780	8.282045	8.309019	8.404662	8.349441	8.445093	8.326084	8.385035	8.359778	8.108027	8.082079	8.246707	8.465084	Klhdc2	NM_027117
6795784	3199	10.60658	10.67338	10.50326	10.42524	0.181343	0.105252	0.24814	0.037009	0.078017	0.454711	0.170123	0.125031	-0.0668	0.520116	0.103327	0.328518	6795784	10.77123	10.62226	10.42626	10.66564	10.68833	10.66617	10.5724	10.56895	10.36842	10.29347	10.54013	10.44212	Arf6	NM_007481
6795794	3200	5.98234	6.197524	6.261426	6.237207	-0.25487	0.073377	-0.03968	0.756644	0.024219	0.849694	-0.0639	0.619506	-0.21518	0.120203	-0.27909	0.054094	6795794	5.854435	6.19698	5.895605	6.158805	6.262004	6.171764	6.306439	6.260646	6.217192	5.97963	6.321631	6.410359	Atp5s	NM_026536
6795869	3202	10.09166	10.17232	10.37923	10.45122	-0.35956	0.083535	-0.27889	0.163849	-0.07198	0.702713	-0.20691	0.288356	-0.08067	0.669248	-0.28758	0.152627	6795869	10.08366	10.17461	10.0167	10.04405	10.13621	10.33671	10.57222	10.51788	10.0476	10.18055	10.40829	10.76481	Tmx1	NM_028339
6795881	3203	7.654503	7.496015	7.317357	7.447745	0.206758	0.123209	0.04827	0.698052	-0.13039	0.308912	0.178658	0.174886	0.158488	0.223149	0.337146	0.022866	6795881	7.740474	7.604836	7.6182	7.490951	7.67314	7.323955	7.38868	7.356104	7.207287	7.214067	7.568663	7.560505	Frmd6	NM_028127
6795885	3204	8.149455	8.362013	8.279039	8.208634	-0.05918	0.616474	0.153379	0.213867	0.070405	0.552607	0.082974	0.485931	-0.21256	0.098206	-0.12958	0.286935	6795885	8.102678	8.2038	8.141887	8.340271	8.44686	8.298907	8.462182	8.296704	8.078231	8.005944	8.277057	8.342901	Actr10	NM_019785
6795889	3206	8.442875	8.573476	8.40015	8.548436	-0.10556	0.322411	0.02504	0.808769	-0.14829	0.176764	0.173326	0.121584	-0.1306	0.228252	0.042725	0.680749	6795889	8.537261	8.4245	8.366864	8.369644	8.622799	8.727985	8.47123	8.444589	8.284631	8.545178	8.457968	8.642163	Arid4a	NM_001081195
6795904	3207	6.594947	6.379524	6.139778	5.816538	0.778408	0.017843	0.562986	0.063895	0.32324	0.252315	0.239746	0.386909	0.215422	0.43477	0.455169	0.120541	6795904	6.871083	6.444799	6.468958	6.378223	6.864223	5.896127	6.375837	6.269838	5.773659	5.689707	5.949382	5.810526	Dact1	NM_021532
6795936	3208	7.763541	7.875775	7.858529	7.713817	0.049724	0.780839	0.161958	0.376035	0.144712	0.426627	0.017246	0.922953	-0.11223	0.534197	-0.09499	0.597517	6795936	7.872561	7.779977	7.638086	7.934796	8.100469	7.59206	7.842081	7.998186	7.73532	7.737082	7.985168	7.419202	Daam1	NM_026102
6795947	3209	9.104436	9.263284	9.07699	9.069643	0.034793	0.785475	0.19364	0.155829	0.007347	0.954059	0.186293	0.170182	-0.15885	0.234691	0.027446	0.829836	6795947	9.071455	9.106486	9.135368	9.130474	9.321042	9.338335	9.219837	9.133088	8.878046	8.872351	9.03954	9.297039	1200003C	NM_024205
6795990	3211	8.886121	8.908751	8.773473	8.681845	0.204276	0.243129	0.226906	0.199151	0.091627	0.58741	0.135278	0.428206	-0.02263	0.892427	0.112649	0.506787	6795990	8.738912	8.836458	9.082994	8.860167	9.041416	8.82467	9.107592	8.617337	8.595489	8.710338	8.835673	8.499525	Ppm1a	NM_008910
6796024	3213	7.260676	7.38895	7.246026	7.234322	0.026355	0.808231	0.154629	0.179245	0.011705	0.914028	0.142924	0.210751	-0.12827	0.256818	0.01465	0.892535	6796024	7.154488	7.404348	7.223193	7.306606	7.404628	7.455617	7.365033	7.136225	7.236821	7.042308	7.275447	7.38521	Mnat1	NM_008612
6796027	3214	7.930139	8.147682	7.782303	7.602636	0.327503	0.004611	0.545047	0.000193	0.179667	0.065394	0.36538	0.00248	-0.21754	0.032426	0.147836	0.117185	6796027	8.002459	7.999999	7.787959	8.062602	8.141258	8.239187	7.707249	7.751937	7.887722	7.533251	7.721589	7.553067	Slc38a6	BC157917
6796043	3215	8.252166	8.382464	8.179934	8.227377	0.024788	0.892843	0.155087	0.409645	-0.04744	0.79686	0.20253	0.288785	-0.1303	0.485669	0.072231	0.695951	6796043	8.499359	8.236442	8.020696	8.336576	8.49281	8.318007	8.278755	8.092376	8.168672	7.897737	8.575781	8.208614	Prkch	NM_008856
6796048	3216	11.28367	11.27762	11.43373	10.61516	0.668507	0.001004	0.66246	0.001063	0.81857	0.000268	-0.15611	0.273207	0.006047	0.96477	-0.15006	0.290839	6796048	11.28911	11.35302	11.20887	11.23116	11.2342	11.3675	11.50241	11.44049	11.35829	10.46385	10.95907	10.42256	Hif1a	NM_010431
6796052	3217	7.225491	7.335809	7.307095	7.052707	0.172784	0.290727	0.283102	0.100935	0.254388	0.134377	0.028714	0.855564	-0.11032	0.490706	-0.0816	0.60768	6796052	7.313563	7.240329	7.122581	7.209221	7.564795	7.233411	7.390668	7.425388	7.105229	7.036899	7.305907	6.815314	Snapc1	NM_178392
6796121	3220	8.619473	8.928089	8.565324	8.448576	0.170897	0.481686	0.479513	0.072196	0.116748	0.627805	0.362765	0.155926	-0.30862	0.219416	0.054149	0.821021	6796121	8.766695	8.707083	8.384642	8.744049	9.208296	8.831923	8.871092	8.199219	8.625662	8.334006	8.811444	8.200278	Rhoj	NM_023275
6796158	3221	8.56045	8.373952	8.162254	7.776558	0.783892	0.006434	0.597394	0.023686	0.385696	0.109711	0.211698	0.35234	0.186498	0.409692	0.398196	0.100336	6796158	8.480328	8.503288	8.697734	8.140414	8.679078	8.302365	8.362346	7.835456	8.28896	7.926934	7.995852	7.406889	Syne2	NM_001005510
6796164	3222	7.337617	7.482035	7.284679	7.487091	-0.14947	0.38687	-0.00506	0.976061	-0.20241	0.250377	0.197356	0.261439	-0.14442	0.402389	0.052938	0.754171	6796164	7.191083	7.256595	7.565172	7.40482	7.465354	7.57593	7.460217	7.166942	7.226878	7.237567	7.40709	7.816617	Mthfd1	NM_138745
6796174	3223	8.295889	8.284274	8.25082	8.020801	0.275089	0.115096	0.263474	0.128906	0.230019	0.177649	0.033454	0.83517	0.011615	0.942337	0.045069	0.779482	6796174	8.2725	8.437225	8.177943	8.105613	8.553942	8.193268	8.37174	8.213723	8.166997	7.747492	8.221469	8.093441	Zbtb1	NM_178744
6796184	3224	7.916123	8.087826	7.732953	7.207596	0.708527	0.078306	0.88023	0.036542	0.525357	0.172951	0.354873	0.341741	-0.1717	0.637947	0.18317	0.616018	6796184	7.812445	7.959718	7.976207	7.694586	8.665483	7.903409	7.794394	7.488104	7.916361	7.111307	7.899742	6.611739	Hspa2	NM_008301
6796193	3225	9.273248	9.415717	9.2201	9.462392	-0.18914	0.150591	-0.04668	0.705106	-0.24229	0.076115	0.195617	0.138801	-0.14247	0.265451	0.053148	0.666962	6796193	9.105473	9.418303	9.295968	9.387162	9.327852	9.532136	9.0154	9.261801	9.383099	9.352373	9.447615	9.587188	Plekhg3	NM_153804
6796196	3226	9.468822	9.663384	9.483522	9.71652	-0.2477	0.086319	-0.05314	0.685965	-0.233	0.103203	0.179862	0.193491	-0.19456	0.163177	-0.0147	0.910491	6796196	9.393101	9.579352	9.434012	9.722235	9.594371	9.673545	9.249951	9.641508	9.559107	9.606547	9.595769	9.947243	Plekhg3	NM_153804
6796208	3227	7.799925	7.725363	7.603582	7.805167	-0.00524	0.970121	-0.0798	0.572551	-0.20158	0.175565	0.12178	0.395526	0.074562	0.597551	0.196342	0.1858	6796208	7.754415	7.891026	7.754333	7.668226	7.850764	7.657098	7.739024	7.68876	7.382963	7.761701	7.594951	8.058848	Churc1	NM_206534
6796211	3228	7.27802	7.22263	7.186103	7.245596	0.032423	0.736498	-0.02297	0.811271	-0.05949	0.540461	0.036527	0.704904	0.055389	0.568126	0.091917	0.35218	6796211	7.155835	7.31613	7.362094	7.234027	7.354126	7.079738	7.13463	7.265955	7.157724	7.259701	7.365989	7.111099	Fntb	NM_145927
6796238	3229	9.094244	9.146461	8.952692	8.321619	0.772625	0.009561	0.824842	0.006837	0.631073	0.024469	0.193769	0.420501	-0.05222	0.824752	0.141552	0.55232	6796238	9.260665	9.306818	8.71525	9.072464	9.275574	9.091345	9.195494	8.78439	8.878192	8.36589	8.67859	7.920378	Fut8	NM_016893
6796299	3230	8.260224	8.189194	8.31951	8.312506	-0.05228	0.56097	-0.12331	0.190426	0.007005	0.937228	-0.13032	0.169023	0.07103	0.433794	-0.05929	0.511022	6796299	8.286252	8.253859	8.240561	8.177101	8.241576	8.148906	8.430949	8.284388	8.243194	8.139576	8.300973	8.496968	Gphn	NM_172952
6796305	3232	7.891155	7.960628	7.897949	8.258219	-0.36706	0.033883	-0.29759	0.072023	-0.36027	0.036472	0.062679	0.674101	-0.06947	0.641578	-0.00679	0.963435	6796305	7.716048	8.060536	7.896882	7.846408	7.872803	8.162674	7.920778	7.767617	8.005452	8.254929	8.039039	8.48069	Mpp5	NM_019579
6796331	3233	9.070924	9.285309	9.263894	9.559371	-0.48845	0.214839	-0.27406	0.471391	-0.29548	0.438691	0.021415	0.95435	-0.21439	0.570654	-0.19297	0.609031	6796331	9.222808	8.796947	9.193016	9.3875	9.406278	9.062149	9.199077	9.737465	8.85514	9.43386	8.925553	10.3187	Arg2	NM_009705
6796339	3234	6.421051	7.255731	6.730627	7.138379	-0.71733	0.002632	0.117352	0.502155	-0.40775	0.040456	0.525104	0.013713	-0.83468	0.001055	-0.30958	0.100884	6796339	6.400547	6.3267	6.535907	7.238864	7.287261	7.241067	6.513135	6.937023	6.741722	6.910362	7.519428	6.985346	Rdh12	NM_030017
6796401	3237	7.717891	7.673131	7.802075	7.644229	0.073663	0.563215	0.028903	0.818919	0.157846	0.232395	-0.12894	0.322024	0.04476	0.723569	-0.08418	0.510242	6796401	7.600945	7.769155	7.783574	7.579017	7.701415	7.738962	7.902631	7.625379	7.878214	7.415628	7.855885	7.661173	Exd2	NM_133798
6796403	3238	6.48238	6.20846	6.208845	5.949285	0.533095	0.054036	0.259175	0.30459	0.25956	0.30392	-0.00038	0.99874	0.27392	0.27975	0.273535	0.280377	6796403	6.887946	6.133719	6.425475	6.359979	6.477693	5.787707	6.208806	6.335265	6.082463	5.741215	6.128296	5.978343	Galntl1	NM_001081421
6796415	3239	10.1695	9.93031	9.946066	9.410864	0.75864	0.018534	0.519447	0.078391	0.535202	0.071287	-0.01576	0.952712	0.239193	0.380115	0.223437	0.410823	6796415	10.329	10.11938	10.06013	9.684277	10.31304	9.793614	10.25215	9.439728	10.14632	9.309661	9.711004	9.211926	4933426M	BC040401
6796422	3240	11.11025	11.28547	11.09558	11.11272	-0.00247	0.98406	0.172743	0.187929	-0.01715	0.889903	0.18989	0.152186	-0.17522	0.18235	0.014673	0.905688	6796422	11.19413	11.15337	10.98325	11.15592	11.21511	11.48537	11.28876	10.89012	11.10785	11.15666	11.13246	11.04905	Sfrs5	NM_001079695
6796430	3241	6.163979	6.157261	6.140969	6.117284	0.046695	0.527452	0.039977	0.587216	0.023685	0.746194	0.016292	0.823508	0.006717	0.926631	0.023009	0.753153	6796430	6.253364	6.136034	6.102538	6.191667	6.184936	6.095181	6.047266	6.304954	6.070688	6.130765	6.129663	6.091424	Smoc1	NM_001146217
6796485	3242	8.222622	8.355932	8.209261	8.269117	-0.04649	0.612599	0.086815	0.354073	-0.05986	0.516758	0.146671	0.135101	-0.13331	0.16935	0.013361	0.88341	6796485	8.271164	8.278941	8.117761	8.419964	8.424886	8.222946	8.203936	8.360657	8.063189	8.245263	8.332329	8.229759	Pcnx	NM_018814
6796492	3243	8.223699	8.276471	8.194106	8.30034	-0.07664	0.422743	-0.02387	0.799108	-0.10623	0.275269	0.082365	0.390437	-0.05277	0.576773	0.029593	0.752627	6796492	8.144425	8.207241	8.319432	8.255428	8.447901	8.126083	8.273633	8.216837	8.091848	8.256533	8.250107	8.394379	Sipa1l1	NM_172579
6796525	3245	7.541024	7.590434	7.68215	7.459839	0.081185	0.549739	0.130595	0.344572	0.222311	0.12566	-0.09172	0.500569	-0.04941	0.713816	-0.14113	0.309346	6796525	7.392508	7.558337	7.672227	7.52554	7.730058	7.515703	7.750305	7.437104	7.85904	7.302425	7.518092	7.559	Wdr21	NM_030246
6796532	3246	9.683627	9.693834	9.625515	9.619726	0.063901	0.410631	0.074108	0.343533	0.005789	0.939252	0.068319	0.380502	-0.01021	0.893145	0.058112	0.452611	6796532	9.733173	9.647367	9.670342	9.605605	9.820797	9.655101	9.709538	9.541063	9.625944	9.523903	9.605557	9.729719	Rbm25	NM_027349
6796538	3247	9.454133	9.57374	9.380383	9.36661	0.087523	0.338636	0.20713	0.04262	0.013773	0.876741	0.193357	0.054723	-0.11961	0.201785	0.07375	0.416118	6796538	9.520055	9.506304	9.33604	9.531708	9.535456	9.654055	9.508528	9.230996	9.401625	9.255679	9.407567	9.436584	Rbm25	NM_027349
6796540	3248	8.851907	8.807473	8.914296	8.842056	0.009852	0.932508	-0.03458	0.766847	0.07224	0.539552	-0.10682	0.37107	0.044434	0.703755	-0.06239	0.595088	6796540	8.854468	8.836033	8.865221	8.746875	9.005527	8.670017	8.924809	8.881757	8.936322	8.722035	9.085103	8.719029	Psen1	NM_008943
6796544	3249	6.598259	6.599328	6.556588	6.607653	-0.00939	0.901682	-0.00833	0.912817	-0.05106	0.507857	0.04274	0.577805	-0.00107	0.988775	0.04167	0.587173	6796544	6.626572	6.676124	6.49208	6.571263	6.531156	6.695565	6.610306	6.638969	6.42049	6.626178	6.556024	6.640757	Papln	NM_130887
6796560	3250	6.957557	7.223448	7.273095	7.196512	-0.23895	0.217746	0.026936	0.883864	0.076584	0.679413	-0.04965	0.788098	-0.26589	0.174918	-0.31554	0.115292	6796560	6.866456	6.888062	7.118154	7.223681	7.35319	7.093473	7.266069	7.047889	7.505328	6.916568	7.546195	7.126772	2410016O	BC138094

6796561	3251	11.00071	10.642	10.74707	11.15337	-0.15266	0.568176	-0.51138	0.081306	-0.4063	0.151841	-0.10507	0.692813	0.358717	0.199505	0.253643	0.351685	6796561	10.85426	11.07449	11.07339	10.86164	10.76398	10.30037	10.18437	11.06132	10.99552	11.40301	11.08724	10.96987	Acot2	NM_134188
6796563	3252	8.71677	8.736264	8.78348	8.966853	-0.25008	0.406668	-0.23059	0.442709	-0.18337	0.538721	-0.04722	0.872771	-0.01949	0.947247	-0.06671	0.821148	6796563	8.878363	8.815789	8.456158	9.00368	8.851339	8.353774	8.729575	8.889097	8.731768	9.075205	8.360757	9.464598	Acot1	NM_012006
6796580	3257	6.834767	7.005887	6.886611	6.993267	-0.1585	0.254969	0.01262	0.924623	-0.10666	0.433198	0.119276	0.383104	-0.17112	0.222115	-0.05184	0.698845	6796580	6.799131	6.929633	6.775536	6.881237	7.032706	7.103718	6.659406	7.203811	6.796617	6.988584	6.979666	7.011551	Dnalc1	NM_028821
6796594	3258	8.152205	8.230373	8.188873	8.548218	-0.39601	0.042626	-0.31785	0.089323	-0.35934	0.060367	0.0415	0.80712	-0.07817	0.647244	-0.03667	0.829135	6796594	7.945776	8.194664	8.316175	8.286869	8.197228	8.207021	8.079507	8.28638	8.200732	8.266953	8.456385	8.921316	Ptgr2	NM_029880
6796595	3259	7.069789	7.195933	7.113512	7.121487	-0.0517	0.474965	0.074446	0.311865	-0.00797	0.910797	0.082421	0.26629	-0.12614	0.104796	-0.04372	0.543803	6796595	7.031744	7.115382	7.062241	7.14284	7.31841	7.126549	7.239761	7.098254	7.002522	7.120814	7.156261	7.087386	Zfp410	NM_144833
6796597	3260	6.517195	6.678394	6.57871	6.551827	-0.03463	0.710822	0.126568	0.19786	0.026884	0.773099	0.099684	0.300886	-0.1612	0.111501	-0.06152	0.514199	6796597	6.438669	6.535766	6.577151	6.625908	6.794015	6.61526	6.732485	6.570123	6.433523	6.439171	6.56671	6.649599	Coq6	NM_172582
6796603	3261	5.692483	6.084077	6.088873	5.987362	-0.29488	0.256202	0.096715	0.698878	0.101511	0.684877	-0.0048	0.98462	-0.39159	0.143061	-0.39639	0.138851	6796603	5.744147	5.379413	5.95389	6.031361	6.233425	5.987444	5.846815	6.63563	5.784173	5.822794	6.028409	6.110883	Rnf113a2	NM_025525
6796606	3262	6.67473	6.798785	6.306546	6.905235	-0.23051	0.266341	-0.10645	0.596141	-0.59869	0.014587	0.492239	0.034084	-0.12406	0.53816	0.368184	0.092727	6796606	6.616311	6.438138	6.969741	6.939553	6.836075	6.620727	6.224485	6.672577	6.022575	7.01773	6.790316	6.90766	Lin52	NM_173756
6796632	3264	7.7763	7.653399	7.810081	7.78933	-0.01303	0.944029	-0.13593	0.471474	0.020751	0.910994	-0.15668	0.409077	0.122901	0.513722	-0.03378	0.8557	6796632	7.68422	7.566472	8.078208	7.801939	7.731207	7.427052	7.923182	8.021807	7.485254	7.806942	7.752083	7.808964	Isca2	BC022589
6796645	3265	8.467215	8.430361	8.342609	8.466579	0.000636	0.996301	-0.03622	0.792246	-0.12397	0.378485	0.087752	0.527861	0.036854	0.788701	0.124606	0.376165	6796645	8.432491	8.509969	8.459185	8.424707	8.510786	8.355591	8.073647	8.661807	8.292374	8.467771	8.566631	8.365335	Ylpm1	NM_178363
6796658	3266	9.91402	9.963696	9.892154	10.13002	-0.216	0.147296	-0.16632	0.251726	-0.23787	0.115247	0.071542	0.60958	-0.04968	0.721717	0.021866	0.875009	6796658	9.794547	9.925143	10.02237	9.905998	10.00208	9.98301	9.68272	10.0671	9.926643	9.944789	10.05078	10.39449	Dlst	NM_030225
6796666	3267	7.854423	7.778367	7.804071	7.735506	0.118917	0.413677	0.042861	0.763926	0.068565	0.632473	-0.0257	0.856796	0.076056	0.596396	0.050352	0.724523	6796666	7.758103	7.839636	7.96553	7.727907	7.927629	7.679565	7.872742	7.497543	8.041929	7.801857	7.773011	7.63165	Eif2b2	NM_145445
6796691	3269	8.339687	7.494732	7.153329	7.24229	1.097396	0.015211	0.252442	0.499303	-0.08896	0.809372	0.341404	0.366627	0.844954	0.045365	1.186358	0.010458	6796691	8.153026	7.76158	9.104454	7.260983	7.710773	7.512441	7.472877	6.612513	7.374596	7.372431	7.215581	7.138859	Fos	NM_010234
6796696	3270	5.984973	6.124543	5.604378	6.01171	-0.02674	0.854612	0.112832	0.447564	-0.40733	0.020414	0.520165	0.006204	-0.13957	0.352146	0.380595	0.027333	6796696	6.005701	5.997565	5.951653	6.19389	6.225976	5.953762	5.591273	5.704105	5.517756	6.172352	6.193342	5.669437	Jdp2	NM_030887
6796704	3272	6.647033	6.938443	6.866931	7.273477	-0.62644	0.044437	-0.33503	0.238525	-0.40655	0.16079	0.071512	0.792608	-0.29141	0.300116	-0.2199	0.427421	6796704	6.327941	6.76982	6.843338	7.196685	6.449624	7.16902	6.745594	7.132897	6.722303	7.200322	6.992536	7.627573	Mfsd7c	NM_145447
6796707	3273	7.679111	7.696232	7.598901	7.839269	-0.16016	0.146046	-0.14304	0.188386	-0.24037	0.042083	0.09733	0.3565	-0.01712	0.867624	0.08021	0.443345	6796707	7.647979	7.678822	7.710533	7.75965	7.755635	7.57341	7.513598	7.605257	7.677849	7.632567	7.851777	8.033464	Ttll5	NM_001081423
6796711	3274	8.948157	9.037264	8.986758	8.724331	0.223826	0.051711	0.312933	0.012728	0.262427	0.027994	0.050506	0.620155	-0.08911	0.389687	-0.0386	0.703895	6796711	8.954403	8.932878	8.957191	8.999187	9.152101	8.960504	9.077527	9.069047	8.8137	8.562732	8.733779	8.876482	1700019E	NM_029601
6796719	3275	7.064173	7.023142	6.980723	6.860055	0.204118	0.081297	0.163087	0.149827	0.120668	0.272401	0.042419	0.689516	0.041031	0.699075	0.083449	0.438599	6796719	7.109188	6.991788	7.091542	7.083612	7.21621	6.769604	6.983824	7.022542	6.935804	6.851697	6.930056	6.798412	1700020O	BC048169
6796728	3276	6.49825	6.777988	6.656839	7.643659	-1.14541	0.022824	-0.86567	0.066396	-0.98682	0.041758	0.121149	0.773833	-0.27974	0.511857	-0.15859	0.707328	6796728	6.321711	6.52378	6.64926	7.188221	6.317724	6.828018	6.310459	7.534919	6.125138	7.731346	7.169375	8.030255	Esrrb	NM_001159500
6796744	3277	6.822959	6.910739	6.830085	6.722152	0.100807	0.159844	0.188587	0.019926	0.107933	0.135682	0.080654	0.25019	-0.08778	0.214178	-0.00713	0.915474	6796744	6.894983	6.801027	6.772866	6.789225	6.992287	6.950705	6.906714	6.849922	6.733618	6.675758	6.765298	6.7254	Vash1	NM_177354
6796768	3278	8.153893	8.177484	8.100598	8.370002	-0.21611	0.026203	-0.19252	0.041584	-0.2694	0.009469	0.076887	0.361338	-0.02359	0.773995	0.053295	0.521078	6796768	8.16212	8.075068	8.224491	8.178864	8.201138	8.152451	8.00623	8.296241	7.999322	8.346345	8.431614	8.332046	2310044G	BC026384
6796777	3280	7.784462	8.049755	7.990543	8.520413	-0.73595	0.004433	-0.47066	0.03663	-0.52987	0.022471	0.059212	0.760674	-0.26529	0.195578	-0.20608	0.304549	6796777	7.588645	7.803596	7.961146	8.156928	7.848414	8.143923	7.960467	8.238318	7.772845	8.63928	8.176581	8.745378	Gstz1	NM_010363
6796784	3281	8.619134	8.713668	8.662437	8.764435	-0.1453	0.364708	-0.05077	0.745688	-0.102	0.518958	0.05123	0.743467	-0.09453	0.549247	-0.0433	0.781855	6796784	8.553741	8.577884	8.725778	8.675165	8.676846	8.788992	8.810177	8.735242	8.441893	8.531205	8.667601	9.0945	Ahsa1	NM_146036
6796799	3282	7.974062	8.180112	8.100753	8.093644	-0.11958	0.516278	0.086467	0.636617	0.007109	0.968791	0.079359	0.664221	-0.20605	0.275665	-0.12669	0.492374	6796799	7.929227	8.051449	7.94151	8.077368	8.436129	8.026838	8.381686	8.155114	7.765459	7.961057	8.013577	8.306299	1810035L	NM_026958
6796807	3283	6.792018	6.727852	6.723951	6.88124	-0.08922	0.176843	-0.15339	0.03437	-0.15729	0.031077	0.0039	0.949963	0.064166	0.317876	0.068066	0.291236	6796807	6.746455	6.798734	6.830864	6.673967	6.754009	6.755579	6.71138	6.834769	6.625705	6.873972	6.803059	6.966688	Adck1	NM_028105
6796902	3285	11.34758	11.42795	11.69938	11.85567	-0.50809	0.00261	-0.42772	0.006771	-0.15629	0.22233	-0.27143	0.05063	-0.08037	0.515437	-0.3518	0.017643	6796902	11.39223	11.41769	11.23282	11.2094	11.4556	11.61885	11.64571	11.8834	11.56903	11.82378	11.81201	11.93123	Tshr	NM_011648
6797090	3286	7.396146	7.19281	6.632079	6.427061	0.969085	0.02053	0.765749	0.052458	0.205017	0.559313	0.560731	0.134257	0.203336	0.562468	0.764068	0.052869	6797090	7.465406	7.423513	7.29952	7.031448	7.824795	6.722187	7.115406	6.244383	6.536447	6.026848	6.806202	6.448134	Flrt2	NM_201518
6797196	3287	7.143097	6.977543	7.487873	6.351153	0.791944	0.055304	0.62639	0.114191	1.13672	0.012291	-0.51033	0.186636	0.165555	0.651886	-0.34478	0.357743	6797196	7.234032	7.075712	7.119548	6.89816	7.632826	6.401642	7.8342	7.091271	7.538147	6.510619	6.718319	5.824521	Gpr65	NM_008152
6797241	3289	9.57612	9.64442	9.707022	9.475114	0.101006	0.191657	0.169306	0.04382	0.231908	0.011281	-0.0626	0.402528	-0.0683	0.363103	-0.1309	0.101746	6797241	9.608845	9.608118	9.511396	9.595472	9.682478	9.65531	9.770738	9.776177	9.57415	9.408932	9.41634	9.600069	Zc3h14	NM_029334
6797274	3290	7.347019	7.488095	7.502639	7.155678	0.191342	0.073309	0.332417	0.007192	0.346961	0.005736	-0.01454	0.879425	-0.14108	0.167207	-0.15562	0.132309	6797274	7.245932	7.474024	7.321102	7.553829	7.580695	7.32976	7.477372	7.497141	7.533404	7.056156	7.310383	7.100494	Ttc8	NM_198311
6797387	3292	8.815645	8.864186	8.763327	8.604629	0.211016	0.196658	0.259557	0.121433	0.158698	0.32042	0.100859	0.519797	-0.04854	0.754252	0.052317	0.735956	6797387	8.818578	8.873822	8.754534	8.763615	9.043545	8.785398	9.059822	8.561629	8.668531	8.379448	8.724443	8.709996	Psmc1	NM_008947
6797391	3293	10.91748	10.91538	10.78077	10.6924	0.225083	0.050624	0.222983	0.052347	0.08837	0.393281	0.134613	0.206591	0.0021	0.983419	0.136713	0.200288	6797391	10.95857	10.84744	10.94643	10.85797	10.95007	10.9381	11.00702	10.76958	10.5657	10.64073	10.69481	10.74165	Calm1	NM_009790
6797434	3294	7.814405	7.365931	7.430862	7.210775	0.603629	0.011489	0.155156	0.426084	0.220087	0.268374	-0.06493	0.734718	0.448474	0.041609	0.383543	0.071916	6797434	7.872136	7.722233	7.848845	7.398614	7.739473	6.959706	7.464787	7.232628	7.595171	7.082263	7.273148	7.276915	9030617O	NM_145448
6797474	3296	7.864753	7.896594	7.958754	7.869388	-0.00464	0.967269	0.027206	0.810025	0.089366	0.438013	-0.06216	0.585801	-0.03184	0.778591	-0.094	0.415579	6797474	7.920286	7.827659	7.846313	7.740213	8.068944	7.880625	8.095663	8.002709	7.77789	7.765975	8.008315	7.833874	Cpsf2	NM_016856
6797478	3298	8.550743	8.594075	8.397165	8.641385	-0.09064	0.516362	-0.04731	0.732241	-0.24422	0.10477	0.19691	0.178493	-0.04333	0.753847	0.153578	0.283251	6797478	8.696342	8.438304	8.517584	8.64347	8.769401	8.369354	8.197019	8.616873	8.377604	8.673226	8.575174	8.675754	Rin3	NM_177620
6797484	3299	8.92126	9.116311	9.031074	8.934721	-0.01346	0.872734	0.18159	0.056193	0.096353	0.27044	0.085237	0.325571	-0.19505	0.043408	-0.10981	0.214187	6797484	8.832665	8.930719	9.000395	8.977324	9.190569	9.18104	9.118445	9.085316	8.889462	8.939033	8.879618	8.985512	Golga5	NM_013747
6797496	3300	7.818431	8.355381	7.43592	8.201671	-0.38324	0.794203	0.153709	0.916518	-0.76575	0.604607	0.919461	0.535687	-0.53695	0.715336	0.382511	0.794584	6797496	6.591946	10.20538	6.657966	6.559326	8.939777	9.567039	8.715746	6.603589	6.988425	7.43673	6.682254	10.48603	Chga	NM_007693
6797507	3301	5.934906	6.128118	6.018645	6.035583	-0.10068	0.310199	0.092534	0.348522	-0.01694	0.859904	0.109472	0.272631	-0.19321	0.071206	-0.08374	0.393837	6797507	5.857461	6.035507	5.911749	6.277851	6.101657	6.004845	6.163752	5.899759	5.992425	6.122176	5.964631	6.019943	D230037D	NM_177140
6797510	3302	7.645219	7.885911	7.688393	7.649908	-0.00469	0.969224	0.236003	0.080109	0.038485	0.752295	0.197517	0.132147	-0.24069	0.075314	-0.04317	0.723509	6797510	7.640878	7.611708	7.68307	7.862743	8.005937	7.789052	7.905063	7.46339	7.696727	7.588954	7.545417	7.815353	Ubr7	NM_025666
6797538	3303	6.895181	7.07693	7.20849	7.395805	-0.50062	0.102545	-0.31887	0.27415	-0.18732	0.509921	-0.13156	0.641106	-0.18175	0.522233	-0.31331	0.28199	6797538	7.026827	6.743981	6.914735	7.116986	6.976097	7.137708	6.961427	7.830218	6.833824	7.078014	7.342838	7.766562	Otub2	NM_026580
6797544	3304	8.835996	8.879813	8.612552	8.35347	0.482526	0.025707	0.526343	0.017554	0.259082	0.180371	0.267261	0.16848	-0.04382	0.810216	0.223444	0.2412	6797544	9.021198	8.673307	8.813484	8.792712	8.985716	8.861012	8.97721	8.232697	8.627749	8.250227	8.40762	8.402563	Ifi27l1	NM_026790
6797579	3309	11.23013	10.2674	9.479908	9.628984	1.601152	0.048663	0.638426	0.38134	-0.14907	0.834161	0.787495	0.286206	0.962727	0.199955	1.750222	0.034729	6797579	11.75185	10.89041	11.04813	10.13425	10.4827	10.18526	10.51801	9.29915	8.622564	10.01525	10.68853	8.183153	Serpina3	NM_009252
6797585	3311	6.173832	6.563433	6.78794	6.013995	0.159837	0.688439	0.549438	0.190715	0.773945	0.078828	-0.22451	0.575231	-0.3896	0.340411	-0.61411	0.148754	6797585	6.605557	6.35739	5.558549	6.848871	6.26624	6.575188	7.480261	6.605223	6.278336	5.621134	6.20135	6.219502	Serpina3	NM_001033335
6797707	3312	9.099585	9.429668	9.233263	9.514748	-0.41516	0.029611	-0.08508	0.602927	-0.28149	0.110992	0.196405	0.246633	-0.33008	0.068857	-0.13368	0.419632	6797707	9.207915	8.945644	9.145197	9.38763	9.549702	9.351672	9.311878	9.398864	8.989048	9.464718	9.275669	9.803858	Glrx5	NM_028419
6797742	3318	7.697507	7.880198	7.918406	8.384761	-0.68725	0.004374	-0.50456	0.020412	-0.46635	0.02859	-0.03821	0.832638	-0.18269	0.327033	-0.2209	0.242407	6797742	7.638114	7.836264	7.618144	8.1112	7.55627	7.973124	7.840276	8.239482	7.675461	8.357174	8.431317	8.365793	Bdkrb2	NM_009747
6797744	3319	6.903191	7.675624	7.307232	7.146633	-0.24344	0.438796	0.52899	0.114615	0.160599	0.605555	0.368392	0.252604	-0.77243	0.032356	-0.40404	0.213282	6797744	7.442015	6.869083	6.398474	7.499916	8.120861	7.406094	7.113658	7.11575	7.692288	7.170975	7.128844	7.140081	Bdkrb1	NM_007539
6797747	3320	7.39018	7.432618	7.440139	7.181238	0.208942	0.103875	0.25138	0.058314	0.258901	0.052605	-0.00752	0.948964	-0.04244	0.719082	-0.04996	0.672483	6797747	7.443258	7.270421	7.456861	7.335866	7.660633	7.301355	7.60725	7.355868	7.357299	7.131604	7.276226	7.135885	4933433P	NM_178613
6797758	3321	10.91922	10.96734	10.98856	10.89231	0.026913	0.735895	0.075033	0.358687	0.096253	0.246914	-0.02122	0.789994	-0.04812	0.549691	-0.06934	0.394459	6797758	10.90909	10.9559	10.89268	10.79825	11.0947	11.00908	10.95354	11.0683	10.94385	10.80951	10.97027	10.89715	Papola	NM_011112
6797772	3322	6.76118	6.86713	6.689191	6.509294	0.251886	0.179166	0.357836	0.069851	0.179897	0.323755	0.177939	0.328729	-0.10595	0.552958	0.071988	0.685	6797772	6.76673	6.769453	6.745356	6.75915	7.026684	6.815556	7.047075	6.406791	6.613708	6.307985	6.745564	6.474333	Vrk1	NM_011705
6797880	3323	8.817403	8.886881	8.797319	8.889209	-0.07181	0.582558	-0.00233	0.985636	-0.09189	0.48452	0.089561	0.495319	-0.06948	0.594615	0.020084	0.8767	6797880	8.822291	8.71635	8.913569	8.845235	9.000343	8.815064	8.542933	9.005413	8.843612	8.812531	8.80427	9.050827	Ccnk	NM_009832
6797893	3324	5.89095	5.93281	5.913926	5.966697	-0.07575	0.409284	-0.03389	0.707052	-0.05277	0.560936	0.018884	0.83359	-0.04186	0.643277	-0.02298	0.798373	6797893	5.935474	5.881124	5.856253	6.018248	5.717447	6.062735	5.931273	5.926252	5.884254	5.93554	5.898063	6.066487	Cyp46a1	NM_010010
6797917	3325	8.076849	8.193395	8.101976	8.063713	0.013136	0.878594	0.129682	0.158107	0.038263	0.658194	0.091419	0.304341	-0.11655	0.199312	-0.02513	0.770604	6797917	8.123926	8.160067	7.946555	8.195468	8.219379	8.165337	8.052351	8.233231	8.020346	7.924354	8.123753	8.143032	Evl	NM_007965
6797950	3326	8.387079	8.662518	8.542134	8.474106	-0.08703	0.28267	0.188413	0.037312	0.068028	0.394234	0.120384	0.149768	-0.27544	0.006538	-0.15505	0.074264	6797950	8.471411	8.359849	8.329978	8.574199	8.705601	8.707755	8.559181	8.675179	8.392042	8.431987	8.459723	8.530607	Yy1	NM_009537
6797952	3327	6.608716	6.572853	6.653992	6.916963	-0.30825	0.002827	-0.34411	0.001471	-0.26297	0.006787	-0.08114	0.296497	0.035863	0.634842	-0.04528	0.550514	6797952	6.58321	6.714825	6.528112	6.680781	6.4672	6.570578	6.726564	6.649861	6.58555	6.890352	6.855809	7.004727	AI132487	NM_001012310
6797969	3329	6.858003	6.799137	6.713819	7.42609	-0.56809	0.084107	-0.62695	0.061229	-0.71227	0.038573	0.085317	0.774669	0.058867	0.843202	0.144184	0.630227	6797969	6.825912	6.944337	6.803761	6.904031	6.832966	6.660413	6.411419	6.915831	6.814208	6.827278	7.356019	8.094974	Meg3	NR_003633
6797978	3330	6.370815	6.344521	6.363262	6.436682	-0.06587	0.563897	-0.09216	0.424142	-0.07342	0.521159	-0.01874	0.868275	0.026294	0.816158	0.007553	0.946667	6797978	6.335472	6.380186	6.396786	6.468396	6.218644	6.346523	6.603437	6.248676	6.237672	6.440819	6.54389	6.325338	Dlk1	NM_010052
6798067	3331	9.022545	9.072472	8.980821	8.967853	0.054692	0.573703	0.104619	0.294436	0.012968	0.892834	0.091651	0.354463	-0.04993	0.606924	0.041724	0.666419	6798067	9.079154	9.033547	8.954934	9.028317	9.100594	9.088505	9.168691	8.884885	8.888886	8.811512	9.002221	9.089825	Ppp2r5c	NM_012023
6798090	3332	6.505744	6.326482	5.692496	5.089589	1.416155	0.008412	1.236893	0.016238	0.602907	0.177572	0.633986	0.15866	0.179262	0.67189	0.813248	0.081254	6798090	6.847557	6.775842	5.893833	6.490757	6.589713	5.898975	6.112168	5.176337	5.788983	4.609894	5.751084	4.907789	Dio3	NM_172119
6798108	3333	10.0764	10.16487	9.903318	9.752301	0.324099	0.000961	0.412573	0.000197	0.151017	0.045678	0.261555	0.003465	-0.08847	0.203478	0.173082	0.026687	6798108	10.14342	10.05513	10.03065	10.20429	10.15681	10.13352	10.00232	9.874176	9.833458	9.643393	9.861587	9.751922	Dync1h1	NM_030238
6798115	3334	8.768839	8.822992	8.901469	9.033938	-0.2651	0.062738	-0.21095	0.123899	-0.13247	0.311789	-0.07848	0.540325	-0.05415	0.67065	-0.13263	0.311237	6798115	8.670398	8.973223	8.662895	8.660651	8.821101	8.987224	8.788457	9.101982	8.813968	9.028893	8.991865	9.081055	Wdr20a	NM_027149
6798153	3335	8.766996	8.741738	8.671951	8.744737	0.022259	0.8663	-0.003	0.981886	-0.07279	0.585301	0.069787	0.600568	0.025258	0.848531	0.095045	0.479105	6798153	8.746991	8.781205	8.772793	8.605377	8.972643	8.647195	8.567523	8.885545	8.562786	8.748883	8.895561	8.589768	Rcor1	NM_198023

6798180	3336	7.403107	7.396065	7.4424	6.906872	0.496235	0.0231	0.489193	0.024562	0.535528	0.016443	-0.04634	0.800162	0.007042	0.969247	-0.03929	0.82993	6798180	7.442448	7.299019	7.467854	7.395403	7.670343	7.122448	7.501845	7.501046	7.32431	6.686328	7.257593	6.776695	Traf3	NM_011632
6798197	3337	6.537695	6.504874	6.509197	6.542824	-0.00513	0.942288	-0.03795	0.595564	-0.03363	0.637455	-0.00432	0.951347	0.032821	0.645432	0.028499	0.68901	6798197	6.601002	6.478158	6.533926	6.527749	6.424119	6.562754	6.527135	6.558618	6.441837	6.661591	6.412191	6.554691	Amn	NM_033603
6798212	3338	6.144079	6.079597	6.250629	6.017532	0.126547	0.252453	0.062065	0.562008	0.233097	0.052742	-0.17103	0.134092	0.064482	0.547228	-0.10655	0.329423	6798212	6.2714	6.165767	5.99507	6.029489	6.15306	6.056243	6.336018	6.02735	6.388519	6.030129	6.047272	5.975196	1200009I	NM_028807
6798213	3339	9.425639	9.174583	9.24977	7.896916	1.528723	0.02108	1.277667	0.043709	1.352854	0.035095	-0.07519	0.89153	0.251056	0.650867	0.175869	0.750402	6798213	10.16348	9.35056	8.762877	9.176106	9.477116	8.870526	9.752257	8.359166	9.637888	7.936656	8.602061	7.152031	Tnfaip2	NM_009396
6798216	3340	10.38447	10.46601	10.38444	10.27091	0.113553	0.356803	0.195097	0.131469	0.113523	0.356923	0.081573	0.50233	-0.08154	0.502483	3E-05	0.9998	6798216	10.48083	10.33692	10.33665	10.4165	10.5719	10.40963	10.60189	10.14219	10.40923	10.14508	10.3148	10.35286	Eif5	NM_173363
6798218	3341	8.946053	9.112094	8.879577	8.912898	0.033155	0.730953	0.199196	0.064813	-0.03332	0.729668	0.232517	0.037083	-0.16604	0.112348	0.066476	0.495503	6798218	9.012289	8.91951	8.906359	8.97502	9.234812	9.126449	8.96888	8.926985	8.742865	8.904314	9.048074	8.786305	Mark3	NM_021516
6798223	3342	6.697219	6.107875	6.382655	6.329872	0.367348	0.060621	-0.222	0.223682	0.052783	0.761843	-0.27478	0.141203	0.589344	0.008061	0.314565	0.098566	6798223	6.603233	6.722442	6.765983	6.183454	6.449478	5.690693	6.369267	6.47147	6.307227	6.409951	6.344255	6.235409	Trmt61a	NM_177374
6798229	3343	7.194879	7.000907	6.978538	6.750509	0.444371	0.025564	0.250398	0.16157	0.228029	0.197775	0.022369	0.893818	0.193973	0.266395	0.216342	0.219386	6798229	7.146798	7.213829	7.224011	7.158455	7.219469	6.624796	7.019227	6.971442	6.944944	6.54572	6.724476	6.98133	2810002N	BC071262
6798232	3344	8.947005	8.900402	8.842044	8.677699	0.269306	0.074439	0.222703	0.128377	0.164346	0.246146	0.058358	0.66856	0.046603	0.731874	0.104961	0.447247	6798232	9.033507	8.87992	8.927588	8.979461	9.058763	8.662982	8.883292	8.853322	8.789519	8.486053	8.927205	8.619838	Klc1	NM_001025360
6798234	3345	8.917538	9.250709	9.023121	9.510003	-0.59246	0.033869	-0.25929	0.295796	-0.48688	0.068905	0.227588	0.354969	-0.33317	0.18859	-0.10558	0.660885	6798234	8.587298	9.00193	9.163387	9.453759	9.081031	9.217337	8.96317	9.273986	8.832207	9.384328	9.205121	9.940559	Zfyve21	NM_026752
6798258	3346	6.428027	6.344467	6.457062	6.257583	0.170444	0.105587	0.086884	0.380118	0.199479	0.065521	-0.1126	0.263086	0.08356	0.397745	-0.02904	0.764177	6798258	6.371605	6.345472	6.567005	6.36486	6.375448	6.293094	6.468162	6.559419	6.343606	6.353292	6.078494	6.340963	A530016L	NM_177039
6798269	3347	7.971917	7.841765	7.898386	7.454092	0.517825	0.00218	0.387673	0.010526	0.444294	0.005193	-0.05662	0.640661	0.130152	0.297255	0.073532	0.54633	6798269	8.100589	7.989103	7.82606	7.843401	7.883022	7.798873	7.89718	8.030365	7.767612	7.453434	7.663391	7.245451	2610204M	NM_198411
6798271	3348	7.883555	7.560146	7.654516	6.996754	0.886801	0.056605	0.563392	0.19493	0.657762	0.137251	-0.09437	0.81866	0.323409	0.440289	0.229039	0.581065	6798271	7.609904	7.605447	8.435314	8.1966	7.520072	6.963765	8.175939	7.461178	7.326431	6.627295	7.004026	7.35894	Adssl1	NM_007421
6798272	3349	6.059935	6.114487	6.014013	5.491417	0.568519	0.002853	0.62307	0.001661	0.522596	0.004585	0.100474	0.475526	-0.05455	0.695068	0.045922	0.741048	6798272	5.938021	6.114965	6.12682	6.228345	6.260064	5.855051	6.169766	5.983175	5.889098	5.335461	5.675864	5.414825	Siva1	NM_013929
6798281	3350	8.814726	8.793442	8.66591	8.480967	0.333759	0.039165	0.312474	0.050041	0.184943	0.209544	0.127531	0.374275	0.021284	0.879106	0.148816	0.304158	6798281	8.846007	8.720923	8.877248	8.845574	8.956958	8.577793	8.628514	8.85919	8.510027	8.480042	8.665464	8.297396	AW555464	NM_001024602
6798285	3351	8.466491	8.468777	8.775506	7.696208	0.770284	0.066898	0.772569	0.066247	1.079299	0.017885	-0.30673	0.423234	-0.00229	0.995136	-0.30901	0.41993	6798285	8.617837	8.094271	8.687366	8.147944	8.897984	8.360403	9.253016	8.372993	8.70051	7.455365	8.361354	7.271904	Pld4	NM_178911
6798288	3352	6.609676	6.849094	6.619806	6.943682	-0.33401	0.065084	-0.09459	0.561837	-0.32388	0.071988	0.229289	0.180558	-0.23942	0.164109	-0.01013	0.949914	6798288	6.593621	6.592392	6.643014	6.728282	6.821447	6.997554	6.794461	6.830729	6.234227	6.840575	7.08031	6.91016	BC022687	NM_145450
6798302	3353	7.878557	7.763	7.878327	7.592535	0.286022	0.062675	0.170465	0.23374	0.285792	0.062844	-0.11533	0.408921	0.115557	0.408027	0.00023	0.998656	6798302	7.838163	7.996875	7.800633	7.966297	7.866488	7.456216	7.820366	8.027139	7.787476	7.546313	7.563869	7.667424	Btbd6	NM_201646
6798309	3354	8.412153	8.390123	8.386007	8.35547	0.056683	0.715618	0.034653	0.823275	0.030537	0.843922	0.004116	0.978803	0.02203	0.886984	0.026146	0.866089	6798309	8.413754	8.451394	8.371312	8.524408	8.459895	8.186066	8.168172	8.704243	8.285606	8.233801	8.311923	8.520686	Pacs2	NM_001081170
6798312	3356	8.779418	8.85971	8.738723	8.732426	0.046992	0.641554	0.127283	0.226496	0.006297	0.949909	0.120986	0.248233	-0.08029	0.432487	0.040695	0.686316	6798312	8.80096	8.816288	8.721006	8.83706	9.070582	8.671487	8.670628	8.810374	8.735606	8.662584	8.694855	8.83984	Mta1	NM_054081
6798313	3357	11.27791	11.01283	11.27466	11.29584	-0.01793	0.848649	-0.283	0.014404	-0.02118	0.821691	-0.26182	0.020543	0.265077	0.019446	0.003253	0.972339	6798313	11.20607	11.27549	11.35217	11.11402	11.07366	10.85082	11.39248	11.28507	11.14642	11.18738	11.36228	11.33785	Crip2	NM_024223
6798314	3358	11.65556	11.01571	10.90192	10.76617	0.88939	0.020326	0.249533	0.441535	0.135747	0.671254	0.113787	0.721547	0.639857	0.071535	0.753643	0.040222	6798314	11.55538	11.65715	11.75416	11.25781	11.25105	10.53826	11.2673	11.21455	10.22391	10.73859	10.98725	10.57268	Crip1	NM_007763
6798315	3359	6.604506	6.68945	6.788606	6.812232	-0.20773	0.031073	-0.12278	0.161306	-0.02363	0.774038	-0.09916	0.247867	-0.08494	0.316784	-0.1841	0.049365	6798315	6.570158	6.49847	6.744891	6.611358	6.787344	6.669647	6.742444	6.920366	6.703007	6.789297	6.831759	6.81564	4930427A	NM_134041
6798388	3363	8.425966	8.616234	8.76495	8.38621	0.039756	0.760169	0.230023	0.105005	0.37874	0.01683	-0.14872	0.271271	-0.19027	0.16903	-0.33898	0.027349	6798388	8.36165	8.491384	8.424865	8.561977	8.762376	8.524348	8.931111	8.598414	8.765325	8.137936	8.533827	8.486868	Zfp386	NM_001004066
6798392	3364	5.217495	5.704156	6.602166	5.155317	0.062178	0.752727	0.548839	0.020565	1.446849	6.38E-05	-0.8981	0.001523	-0.48666	0.034057	-1.38467	8.71E-05	6798392	5.246853	5.237805	5.167827	5.870997	5.915416	5.326055	6.7475	6.294157	6.76484	5.240378	5.291445	4.934127	Vipr2	NM_009511
6798399	3365	9.18561	9.20929	9.228487	8.974588	0.211022	0.063735	0.234702	0.043739	0.2539	0.032246	-0.0192	0.849776	-0.02368	0.815388	-0.04288	0.673711	6798399	9.212687	9.187116	9.157027	9.23726	9.324636	9.065973	9.35708	9.275212	9.05317	9.027299	9.061712	8.834752	Fam62b	NM_028731
6798404	3367	5.582824	6.229303	5.954096	6.831762	-1.24894	0.041177	-0.60246	0.274778	-0.87767	0.126035	0.275207	0.606837	-0.64648	0.243858	-0.37127	0.490576	6798404	5.509683	5.486991	5.751797	5.710722	5.534726	7.442462	5.672192	6.594762	5.595334	7.152412	6.926258	6.416615	Ptprn2	NM_011215
6798418	3368	7.856086	8.70927	7.792606	8.22575	-0.36966	0.084001	0.48352	0.032606	-0.43314	0.049582	0.916664	0.001207	-0.85318	0.001867	0.06348	0.74352	6798418	7.880718	8.006405	7.681135	8.499393	8.743024	8.885392	8.028722	7.548952	7.800144	7.893122	8.463671	8.320457	Rapgef5	NM_175930
6798429	3369	8.827041	9.42725	9.460727	7.941131	0.885911	0.105624	1.486119	0.015593	1.519596	0.014048	-0.03348	0.946744	-0.60021	0.25163	-0.63369	0.228306	6798429	8.584353	8.440661	9.45611	9.325546	9.899328	9.056876	9.814037	9.013225	9.554919	7.080338	7.917918	8.825136	Cdca7l	NM_146040
6798513	3371	9.634685	9.615202	9.630493	9.642922	-0.00824	0.929433	-0.02772	0.766271	-0.01243	0.893729	-0.01529	0.869487	0.019483	0.834262	0.004192	0.964042	6798513	9.671233	9.621408	9.611415	9.530885	9.728923	9.585798	9.786532	9.545668	9.55928	9.486446	9.745388	9.696931	Rab10	NM_016676
6798600	3372	6.98593	7.154296	7.052294	7.20909	-0.22316	0.036847	-0.05479	0.55611	-0.1568	0.116854	0.102002	0.28591	-0.16837	0.095807	-0.06636	0.478186	6798600	6.857162	7.073326	7.027302	7.11936	7.183905	7.159623	7.154455	6.932858	7.069569	7.205294	7.065366	7.356611	Cenpo	NM_134046
6798601	3373	9.004232	9.102648	8.994682	9.117762	-0.11353	0.216608	-0.01511	0.862704	-0.12308	0.183947	0.107966	0.237852	-0.09842	0.278398	0.00955	0.912934	6798601	8.913634	9.059537	9.039526	9.093621	9.213243	9.00108	8.893068	9.165364	8.925614	9.05337	9.163431	9.136485	Ncoa1	NM_010881
6798640	3377	9.140207	9.148509	9.202737	9.485306	-0.3451	0.034109	-0.3368	0.03753	-0.28257	0.07013	-0.05423	0.698994	-0.0083	0.952566	-0.06253	0.656203	6798640	9.258551	9.245703	8.916368	9.018697	9.157742	9.269089	9.424696	8.963433	9.220081	9.427035	9.492935	9.535948	Ubxn2a	NM_145441
6798770	3378	6.477966	6.533233	6.466095	6.494443	-0.01648	0.843887	0.03879	0.644829	-0.02835	0.735389	0.067138	0.431209	-0.05527	0.514308	0.011871	0.887104	6798770	6.503181	6.480505	6.450213	6.542922	6.526897	6.52988	6.486237	6.462881	6.449167	6.6395	6.272069	6.57176	Gdf7	NM_013527
6798795	3379	9.322438	9.196501	9.166701	9.130712	0.191726	0.239755	0.065789	0.674494	0.035989	0.817561	0.0298	0.848432	0.125937	0.428345	0.155737	0.332409	6798795	9.408045	9.27381	9.28546	9.46891	9.142863	8.977731	9.190883	9.28872	9.0205	9.236032	9.282953	8.873151	Rhob	NM_007483
6798933	3382	7.107595	7.204905	7.206344	7.14884	-0.04125	0.691074	0.056065	0.590682	0.057504	0.581375	-0.00144	0.988885	-0.09731	0.35937	-0.09875	0.352694	6798933	7.049217	7.093204	7.180363	7.283846	6.994101	7.336769	7.227992	7.256369	7.13467	7.139299	7.285864	7.021357	Kcns3	NM_173417
6798939	3383	6.0052	6.056839	6.194534	6.081687	-0.07649	0.745854	-0.02485	0.915885	0.112847	0.633882	-0.1377	0.56255	-0.05164	0.826472	-0.18933	0.430305	6798939	6.162951	5.701661	6.150988	5.843823	6.20483	6.121863	5.97063	6.663947	5.949025	5.864752	6.111273	6.269035	Msgn1	NM_019544
6799064	3387	9.398888	9.4753	9.459475	9.277319	0.121569	0.409227	0.197981	0.193903	0.182156	0.228241	0.015824	0.912545	-0.07641	0.599078	-0.06059	0.675763	6799064	9.344303	9.37929	9.473072	9.335135	9.661036	9.429728	9.731938	9.300207	9.346281	9.090107	9.327343	9.414507	Ddx1	NM_134040
6799116	3388	6.262774	6.481659	6.150734	6.616769	-0.35399	0.254866	-0.13511	0.652174	-0.46604	0.145028	0.330925	0.284677	-0.21888	0.469959	0.112041	0.70799	6799116	6.045175	6.102078	6.64107	6.157713	7.071486	6.215777	6.129559	6.169785	6.152857	6.882142	6.207228	6.760937	Fam84a	NM_029007
6799173	3389	6.913969	6.966758	6.893083	7.075448	-0.16148	0.300789	-0.10869	0.477825	-0.18237	0.246873	0.073675	0.62737	-0.05279	0.726995	0.020886	0.889766	6799173	6.992951	7.019715	6.72924	7.004474	6.937567	6.958232	6.840662	7.015641	6.822945	6.759183	7.112976	7.354186	Trib2	NM_144551
6799205	3390	7.4244	7.528404	7.149616	7.927362	-0.50296	0.168412	-0.39896	0.264039	-0.77775	0.047297	0.378788	0.287093	-0.104	0.762189	0.274784	0.432047	6799205	6.91423	7.296503	8.062467	7.723048	7.466076	7.396088	7.027931	7.23938	7.181538	7.455825	7.828931	8.497331	Lpin1	NM_015763
6799224	3392	8.917918	8.866131	9.086103	7.82591	1.092008	0.013511	1.040221	0.01695	1.260193	0.006588	-0.21997	0.542914	0.051787	0.884792	-0.16819	0.64013	6799224	8.971795	8.897852	8.884106	8.990097	8.832354	8.775941	9.5464	8.741923	8.969986	7.934178	8.495566	7.047986	Pqlc3	NM_172574
6799246	3394	6.211984	7.546074	7.033436	8.019189	-1.8072	0.002125	-0.47311	0.277098	-0.98575	0.041205	0.512639	0.24192	-1.33409	0.011045	-0.82145	0.077463	6799246	6.203761	6.463717	5.968474	8.280906	7.184575	7.172742	7.034841	7.10643	6.959036	7.685781	7.528465	8.84332	Atp6v1c2	NM_001159632
6799362	3395	5.933517	6.136243	6.118542	5.957771	-0.02425	0.906695	0.178472	0.399344	0.160771	0.445778	0.017701	0.931816	-0.20273	0.341542	-0.18503	0.383065	6799362	6.180049	5.723689	5.896812	6.368186	5.810402	6.230141	6.228	5.802244	6.325381	6.138314	5.91056	5.824438	---	ENSMUST000001
6799462	3397	9.860129	9.524507	9.459991	8.920569	0.93956	0.061484	0.603937	0.199921	0.539422	0.247373	0.064515	0.885055	0.335622	0.459852	0.400138	0.381708	6799462	9.375001	10.45599	9.749396	8.967103	9.982932	9.623485	9.478395	9.372841	9.528738	8.736985	9.735512	8.289211	Id2	NM_010496
6799524	3398	6.855227	6.893151	6.870092	6.839985	0.015242	0.9298	0.053166	0.759289	0.030107	0.861961	0.02306	0.894008	-0.03792	0.826728	-0.01486	0.931536	6799524	6.952059	6.915308	6.698315	6.773942	7.208767	6.696745	6.755986	7.032672	6.821617	6.887756	6.590142	7.042057	Rnf144a	NM_001081977
6799525	3399	6.593882	6.763751	6.642882	6.464904	0.128978	0.688519	0.298847	0.363599	0.177978	0.581935	0.120869	0.706987	-0.16987	0.59893	-0.049	0.878411	6799525	6.53773	6.504211	6.739704	6.327924	7.496417	6.466912	6.551799	6.376124	7.000722	6.440061	6.254683	6.699968	Rsad2	NM_021384
6799578	3400	5.967182	6.088413	5.850572	5.981136	-0.01395	0.936619	0.107277	0.545745	-0.13056	0.464682	0.23784	0.199484	-0.12123	0.49618	0.116609	0.512273	6799578	5.791255	5.717318	6.392972	5.949086	6.186985	6.129167	5.85788	5.819182	5.874655	6.132728	5.957642	5.853037	Sox11	NM_009234
6799651	3403	7.905879	7.909481	7.975251	7.973345	-0.06747	0.320903	-0.06386	0.34585	0.001906	0.976884	-0.06577	0.332467	-0.0036	0.956342	-0.06937	0.308279	6799651	7.887748	7.967172	7.862718	7.89296	7.995254	7.840228	7.952819	8.09803	7.874903	7.980995	7.921177	8.017862	Ttc15	NM_178811
6799771	3404	13.08768	13.03904	13.20228	13.48357	-0.39589	0.003151	-0.44453	0.001593	-0.28128	0.0182	-0.16324	0.124362	0.048637	0.62283	-0.11461	0.262547	6799771	12.96922	13.17518	13.11863	13.04048	12.86674	13.2099	13.17238	13.30526	13.12921	13.4153	13.47803	13.55737	Tpo	NM_009417
6799775	3405	7.407708	7.447784	7.174337	7.335351	0.072357	0.713766	0.112433	0.571078	-0.16101	0.422225	0.273447	0.188804	-0.04008	0.838522	0.233371	0.255087	6799775	7.112539	7.560013	7.550572	7.155394	7.541556	7.646402	7.392481	6.873784	7.256745	7.459968	7.307602	7.238484	Sntg2	NM_172951
6799800	3406	5.915467	5.863541	5.614279	5.831723	0.083744	0.797057	0.031818	0.922021	-0.21744	0.509494	0.249262	0.451543	0.051926	0.873144	0.301188	0.36695	6799800	5.603787	6.045421	6.097192	6.131511	5.773932	5.68518	4.941754	5.746845	6.154238	5.692203	6.16714	5.635826	Acp1	NM_001110239
6799836	3407	8.777018	8.90389	8.872288	9.053843	-0.27682	0.143319	-0.14995	0.405042	-0.18156	0.318293	0.031603	0.857647	-0.12687	0.478338	-0.09527	0.591826	6799836	8.827036	8.815118	8.688901	8.876143	8.844261	8.991267	9.055861	8.819204	8.741798	8.733545	8.970948	9.457035	Dld	NM_007861
6799841	3408	7.820416	7.773622	7.8546	7.741324	0.079092	0.491082	0.032297	0.775746	0.113276	0.331598	-0.08098	0.481123	0.046794	0.680681	-0.03418	0.763098	6799841	7.814669	7.882565	7.764014	7.647985	7.984289	7.688591	7.73431	8.003363	7.826128	7.681411	7.887232	7.65533	Cbll1	NM_134048
6799842	3409	6.912858	6.239718	7.178396	7.643671	-0.73081	0.088407	-1.40395	0.005821	-0.46527	0.251932	-0.93868	0.037451	0.673141	0.111837	-0.26554	0.500977	6799842	6.356461	7.406095	6.976019	6.231218	5.861016	6.626919	7.384593	6.538664	7.611932	7.408352	8.016906	7.505755	Slc26a4	NM_011867
6799849	3410	8.39014	8.489328	8.501361	8.871919	-0.48178	0.013999	-0.38259	0.037745	-0.37056	0.04264	-0.01203	0.939594	-0.09919	0.537253	-0.11122	0.490419	6799849	8.339965	8.443115	8.387339	8.464784	8.437613	8.565586	8.694182	8.347456	8.462444	8.656931	8.716702	9.242123	Bcap29	NM_007530
6799852	3411	6.130931	6.115096	6.224623	6.290889	-0.15996	0.378107	-0.17579	0.335182	-0.06627	0.709184	-0.10953	0.540767	0.015835	0.928678	-0.09369	0.59963	6799852	5.913433	6.255843	6.223518	5.806473	6.350542	6.188273	6.154309	6.413736	6.105824	6.075118	6.414989	6.382561	Dus4l	NM_028002
6799874	3413	8.5553	8.912607	8.778483	8.900503	-0.3452	0.015213	0.012104	0.916771	-0.12202	0.308607	0.134123	0.266282	-0.35731	0.012926	-0.22318	0.081949	6799874	8.481697	8.624433	8.559771	8.870065	8.942143	8.925612	8.887842	8.54196	8.905648	8.88064	8.74701	9.073858	Hbp1	NM_153198
6799879	3414	8.547595	8.815427	8.406437	8.354382	0.193213	0.393509	0.461046	0.063592	0.052056	0.81415	0.40899	0.09269	-0.26783	0.246606	0.141158	0.528509	6799879	8.624918	8.304631	8.713236	8.862474	8.588317	8.995491	8.768212	8.015196	8.435904	8.113135	8.468467	8.481543	Prkar2b	NM_011158
6799897	3415	7.611196	7.795457	7.43871	6.426586	1.18461	0.010778	1.368871	0.005097	1.012124	0.022364	0.356747	0.348737	-0.18426	0.621093	0.172486	0.643244	6799897	7.873091	7.439697	7.5208	7.668561	8.213001	7.50481	7.953197	6.697491	7.665443	6.33632	6.847783	6.095656	Pik3cg	NM_020272
6800020	3416	6.371065	6.651978	6.549691	6.806897	-0.43583	0.019714	-0.15492	0.331862	-0.25721	0.124708	0.102288	0.514501	-0.28091	0.097961	-0.17863	0.267802	6800020	6.280097	6.44552	6.387578	6.448776	6.628458	6.878701	6.625856	6.261054	6.762162	6.795119	6.694172	6.931399	Hdac9	NM_024124

6800060	3417	8.398032	8.178189	8.105888	7.861219	0.536813	0.107433	0.31697	0.315675	0.244668	0.432625	0.072302	0.813254	0.219843	0.479078	0.292145	0.352757	6800060	8.760472	8.487513	7.946112	7.963064	8.658056	7.913448	8.306896	7.957152	8.053615	7.422333	8.143384	8.017941	Ahr	NM_013464
6800079	3418	10.52791	10.71239	10.75147	10.87359	-0.34567	0.018526	-0.16119	0.20671	-0.12212	0.328343	-0.03907	0.747654	-0.18448	0.154486	-0.22355	0.093171	6800079	10.48466	10.55595	10.54313	10.653	10.71644	10.76774	10.90489	10.86078	10.48873	10.70783	11.02695	10.88598	Tspan13	NM_025359
6800082	3419	9.565218	9.425724	9.415256	8.556784	1.008434	0.008666	0.868941	0.017743	0.858473	0.018741	0.010468	0.972291	0.139494	0.64575	0.149962	0.62156	6800082	9.60854	9.368795	9.718319	9.153187	9.856477	9.267509	9.847476	8.981773	9.41652	8.511535	8.965005	8.193811	Bzw2	NM_025840
6800228	3421	7.018697	6.955284	6.886121	6.717441	0.301256	0.034915	0.237842	0.080222	0.16868	0.193356	0.069163	0.576421	0.063413	0.607943	0.132576	0.296762	6800228	7.12042	6.950809	6.984862	7.053503	7.063987	6.748361	7.10735	6.693655	6.857358	6.717622	6.749845	6.684857	Arl4a	NM_001039515
6800229	3422	10.57515	10.56575	10.45956	9.366796	1.208351	0.001892	1.198958	0.001982	1.092765	0.003398	0.106193	0.700153	0.009393	0.972693	0.115586	0.675359	6800229	10.75498	10.44203	10.52843	10.50563	10.88712	10.30451	10.74311	9.925482	10.71009	9.019728	9.461385	9.619274	Scin	NM_001146196
6800233	3423	8.27844	8.6086	8.547713	8.365	-0.08656	0.549481	0.2436	0.116724	0.182713	0.223696	0.060887	0.67192	-0.33016	0.044319	-0.26927	0.087835	6800233	8.435369	8.250002	8.14995	8.685629	8.69241	8.44776	8.720524	8.313217	8.609398	8.223858	8.560035	8.311107	Ifrd1	NM_013562
6800236	3424	6.78851	6.891781	6.864499	6.780979	0.007532	0.949183	0.110803	0.361664	0.08352	0.486665	0.027282	0.817711	-0.10327	0.39357	-0.07599	0.525688	6800236	6.68216	6.824406	6.858965	6.725579	6.984097	6.965668	7.035769	6.706381	6.851347	6.690858	6.700425	6.951653	Zfp277	NM_172575
6800427	3426	11.42262	11.41673	11.86153	11.86802	-0.4454	0.154199	-0.45129	0.149486	-0.00649	0.982267	-0.4448	0.15469	0.005887	0.983915	-0.43891	0.159551	6800427	11.37257	11.62253	11.27275	11.31772	11.448	11.48447	11.3874	12.39684	11.80034	12.13304	12.09639	11.37462	Dnajb9	NM_013760
6800468	3427	7.903722	7.890609	7.988624	7.814519	0.089203	0.496118	0.07609	0.559933	0.174105	0.201513	-0.09802	0.455942	0.013113	0.919106	-0.0849	0.516528	6800468	7.821929	7.942982	7.946254	7.778636	8.161007	7.732183	7.93897	8.079777	7.947124	7.734777	7.704381	8.004398	Stxbp6	NM_144552
6800711	3429	8.588064	8.631708	8.57156	8.61147	-0.02341	0.839903	0.020239	0.86128	-0.03991	0.731153	0.060148	0.606331	-0.04364	0.707317	0.016504	0.886648	6800711	8.518644	8.693184	8.552365	8.540125	8.678195	8.676805	8.611392	8.748761	8.354528	8.446122	8.701558	8.686729	Strn3	NM_052973
6800723	3430	7.845405	7.77239	7.942364	7.672776	0.172629	0.299657	0.099614	0.540107	0.269588	0.121537	-0.16997	0.306651	0.073015	0.651545	-0.09696	0.550704	6800723	7.872621	7.801187	7.862406	7.678088	8.003886	7.635195	8.044702	7.923019	7.85937	7.692165	7.969824	7.356339	Heatr5a	NM_177171
6800726	3431	7.101232	7.21401	7.193818	7.171499	-0.07027	0.368974	0.042511	0.580487	0.022319	0.770073	0.020192	0.791349	-0.11278	0.165049	-0.09259	0.245115	6800726	7.035577	7.116658	7.15146	7.325868	7.141159	7.175002	7.185596	7.214909	7.180948	7.026206	7.18661	7.30168	6530401N	NM_029545
6800859	3433	6.854782	6.683074	6.888531	8.207523	-1.35274	0.010096	-1.52445	0.005434	-1.31899	0.011434	-0.20546	0.624727	0.171707	0.68197	-0.03375	0.935465	6800859	5.917838	6.972346	7.674161	6.994057	6.618203	6.436963	6.853182	7.143873	6.668537	7.931366	8.390794	8.300409	Egln3	NM_028133
6800887	3434	7.362086	7.539638	7.600168	7.496448	-0.13436	0.273413	0.04319	0.715254	0.10372	0.390519	-0.06053	0.610655	-0.17755	0.158794	-0.23808	0.070702	6800887	7.324376	7.421465	7.340417	7.497765	7.733124	7.388026	7.756473	7.644304	7.399726	7.556992	7.558386	7.373966	1110002B	NM_134054
6800890	3435	8.699312	8.769791	8.605761	8.7396	-0.04029	0.775561	0.03019	0.830628	-0.13384	0.355911	0.164029	0.264196	-0.07048	0.619859	0.09355	0.51281	6800890	8.504846	8.833974	8.759115	8.690863	8.862978	8.755531	8.355665	8.697382	8.764237	8.608492	8.922314	8.687995	Eapp	NM_025456
6800894	3436	9.591968	9.609316	9.626664	9.710328	-0.11836	0.583457	-0.10101	0.638914	-0.08366	0.696897	-0.01735	0.93532	-0.01735	0.935318	-0.0347	0.871145	6800894	9.390127	9.522624	9.863153	9.23416	9.938617	9.655172	9.767984	9.577143	9.534866	9.975946	9.640512	9.514527	Snx6	NM_026998
6800906	3437	9.431724	9.383269	9.382452	9.485467	-0.05374	0.652316	-0.1022	0.399524	-0.10302	0.395941	0.000816	0.994503	0.048456	0.684209	0.049272	0.679238	6800906	9.335206	9.341303	9.618664	9.36284	9.454324	9.332642	9.597503	9.3567	9.193154	9.386137	9.54697	9.523295	Cfl2	NM_007688
6800912	3438	6.020426	5.997455	6.147355	6.233489	-0.21306	0.140557	-0.23603	0.107568	-0.08613	0.527048	-0.1499	0.283073	0.022971	0.86441	-0.12693	0.358398	6800912	5.972734	6.045604	6.04294	5.94844	6.178555	5.865371	5.885594	6.272791	6.28368	6.174735	6.403487	6.122245	2700097O	BC056967
6800934	3441	10.71335	10.58308	10.65801	10.23537	0.477978	0.069434	0.347711	0.165937	0.422638	0.101045	-0.07493	0.750991	0.130267	0.583634	0.05534	0.814418	6800934	10.77428	10.66307	10.7027	10.78383	10.49237	10.47305	10.83225	10.53988	10.6019	10.4406	10.6054	9.660116	Nfkbia	NM_010907
6800948	3442	8.881862	8.786123	8.884875	8.92662	-0.04476	0.508488	-0.1405	0.061592	-0.04174	0.5367	-0.09875	0.165303	0.095739	0.177063	-0.00301	0.963977	6800948	8.908597	8.94698	8.790008	8.760333	8.747013	8.851023	8.921309	8.885014	8.848303	8.805805	9.041046	8.93301	Garnl1	NM_001112714
6800968	3443	9.817155	9.911797	9.840797	9.695469	0.121686	0.258395	0.216328	0.062498	0.145318	0.184299	0.07101	0.497871	-0.09464	0.3717	-0.02363	0.819146	6800968	9.728246	9.975822	9.747396	9.75661	9.96034	10.01844	9.96517	9.713444	9.843746	9.611427	9.768977	9.706003	Mbip	NM_145442
6800974	3444	10.12025	10.22848	10.10587	10.14514	-0.02489	0.836729	0.083337	0.49621	-0.03927	0.745571	0.122609	0.324938	-0.10823	0.38166	0.014382	0.905126	6800974	10.03503	10.26822	10.05751	10.12994	10.22943	10.32607	9.932864	10.36208	10.02267	10.10007	10.22308	10.11228	Nkx2-1	NM_009385
6801030	3446	5.849903	6.567765	5.763922	6.060835	-0.21093	0.315135	0.50693	0.032845	-0.29691	0.169862	0.803843	0.003515	-0.71786	0.006521	0.085981	0.673782	6801030	5.798285	5.889763	5.861662	6.748213	6.845797	6.109285	5.761293	5.675456	5.855018	6.084511	6.297452	5.800542	Foxa1	NM_008259
6801076	3447	8.209708	8.491783	8.479718	8.515853	-0.30614	0.311573	-0.02407	0.93441	-0.03613	0.901701	0.012064	0.967092	-0.28207	0.348785	-0.27001	0.368668	6801076	8.372951	8.467483	7.78869	8.687241	8.131715	8.656392	8.940405	8.19957	8.29918	8.203035	8.791925	8.552598	Clec14a	NM_025809
6801102	3448	7.961009	8.072263	7.898239	7.995718	-0.03471	0.758663	0.076545	0.503068	-0.09748	0.397946	0.174024	0.14957	-0.11125	0.337964	0.06277	0.581096	6801102	7.87509	7.957599	8.050338	7.898453	8.289049	8.029287	7.990076	7.975159	7.729482	7.966658	8.057578	7.962918	Sec23a	NM_009147
6801104	3449	8.774026	9.020762	8.774099	8.873141	-0.09911	0.575482	0.147621	0.409948	-0.09904	0.575758	0.246663	0.18437	-0.24674	0.184255	-7.3E-05	0.999667	6801104	8.661268	8.829319	8.831492	8.965004	8.855755	9.241528	8.964212	8.87204	8.486046	8.799629	8.674316	9.145479	Trappc6b	NM_030057
6801324	3450	7.194982	7.352632	7.321984	7.089898	0.105083	0.360223	0.262734	0.041429	0.232086	0.064432	0.030648	0.784325	-0.15765	0.183492	-0.127	0.274562	6801324	7.12857	7.288518	7.167857	7.289999	7.333241	7.434657	7.452466	7.295303	7.218184	6.852919	7.247225	7.169551	Klhl28	NM_025707
6801332	3451	8.350336	8.406321	8.363969	8.429608	-0.07927	0.614873	-0.02329	0.881606	-0.06564	0.676176	0.042351	0.786851	-0.05598	0.721236	-0.01363	0.930488	6801332	8.25776	8.413386	8.379863	8.405749	8.42071	8.392503	8.568202	8.418848	8.104858	8.328074	8.221424	8.739326	Fkbp3	NM_013902
6801451	3454	6.505451	6.624633	6.502365	6.451699	0.053752	0.595302	0.172934	0.113035	0.050666	0.61624	0.122269	0.243813	-0.11918	0.254917	0.003086	0.975445	6801451	6.467542	6.483091	6.565719	6.590933	6.656114	6.626853	6.7117	6.523211	6.272183	6.382303	6.510533	6.462261	1110034A	BC062898
6801459	3456	8.432487	8.476649	8.571809	8.524934	-0.09245	0.228326	-0.04829	0.514879	0.046875	0.526905	-0.09516	0.216181	-0.04416	0.550517	-0.13932	0.084867	6801459	8.430067	8.48505	8.382343	8.394953	8.542538	8.492455	8.563349	8.671062	8.481016	8.461977	8.655417	8.457408	Sdccag1	NM_025441
6801488	3457	6.988112	7.082236	6.908899	7.200073	-0.21196	0.114016	-0.11784	0.352944	-0.29117	0.04077	0.173337	0.18494	-0.09412	0.453576	0.079213	0.526024	6801488	6.963983	7.03381	6.966543	6.863551	7.144532	7.238626	6.895628	6.972355	6.858715	6.962024	7.306411	7.331785	L2hgdh	NM_145443
6801491	3458	7.84103	7.740743	7.544975	7.988235	-0.14721	0.770544	-0.24749	0.625623	-0.44326	0.390114	0.195768	0.698715	0.100287	0.842262	0.296055	0.560771	6801491	7.45861	7.684978	8.379503	7.887937	7.542739	7.791554	7.340338	8.090929	7.203659	7.521068	7.341996	9.101642	Cdkl1	NM_183294
6801493	3459	8.687696	8.891614	8.664341	8.617121	0.070575	0.489041	0.274493	0.022484	0.04722	0.640566	0.227272	0.047779	-0.20392	0.069459	0.023355	0.816398	6801493	8.763021	8.776631	8.523436	8.744741	9.030275	8.899825	8.671671	8.692127	8.629226	8.480658	8.651803	8.718902	Map4k5	NM_201519
6801499	3460	9.043994	8.947951	8.969567	8.645461	0.398534	0.015328	0.302491	0.048102	0.324106	0.037095	-0.02162	0.871854	0.096043	0.480424	0.074428	0.58207	6801499	9.094997	8.948084	9.088902	8.939254	9.201285	8.703315	8.988529	8.929576	8.990595	8.632874	8.827193	8.476315	Sav1	NM_022028
6801500	3461	7.315524	7.329604	7.405544	7.191754	0.12377	0.415074	0.137849	0.366457	0.21379	0.175937	-0.07594	0.612252	-0.01408	0.924516	-0.09002	0.549295	6801500	7.470013	7.392431	7.084128	7.48121	7.191989	7.315612	7.273932	7.626299	7.316401	7.095	7.373077	7.107186	ENSMUSG0	ENSMUST000000
6801506	3462	9.984276	9.856858	9.848461	9.140622	0.843654	0.012391	0.716237	0.025978	0.707839	0.027296	0.008398	0.975279	0.127417	0.640628	0.135815	0.619113	6801506	10.05541	9.599197	10.29822	9.666928	10.25514	9.648507	10.27328	9.482857	9.789245	9.118061	9.053014	9.25079	Pygl	NM_133198
6801560	3464	6.081764	6.091471	6.016752	6.210991	-0.12923	0.102813	-0.11952	0.126946	-0.19424	0.024376	0.074719	0.318067	-0.00971	0.893402	0.065012	0.381324	6801560	6.099443	6.061675	6.084175	6.161676	6.158329	5.954409	5.989097	5.954556	6.106603	6.208743	6.118641	6.305588	Timm9	NM_013896
6801632	3466	6.314162	6.528224	6.209959	6.204608	0.109554	0.376126	0.323616	0.024365	0.005352	0.96461	0.318265	0.026152	-0.21406	0.104467	0.104202	0.398765	6801632	6.269758	6.363123	6.309604	6.697955	6.478151	6.408566	6.31081	6.109587	6.209481	6.034822	6.130606	6.448395	2810055F	NM_026038
6801636	3467	7.072309	7.004188	7.575919	7.384139	-0.31183	0.430541	-0.37995	0.341402	0.19178	0.623459	-0.57173	0.166503	0.06812	0.860608	-0.50361	0.21685	6801636	7.365841	6.977022	6.874063	7.500559	6.652497	6.859509	7.05469	8.440693	7.232373	7.510929	7.265585	7.375902	Rtn1	NM_153457
6801656	3468	8.855306	8.814813	8.81633	8.702818	0.152488	0.253357	0.111995	0.392426	0.113512	0.386347	-0.00152	0.990531	0.040493	0.752178	0.038976	0.761119	6801656	8.706682	8.840986	9.01825	8.82811	8.727451	8.888877	8.993984	8.619543	8.835462	8.622208	8.598797	8.887448	Dhrs7	NM_025522
6801681	3470	6.210519	6.343328	6.058295	6.273036	-0.06252	0.724222	0.070292	0.691904	-0.21474	0.244756	0.285033	0.134204	-0.13281	0.459834	0.152224	0.399475	6801681	5.914061	6.387091	6.330405	6.557018	6.425255	6.047712	6.230092	5.989102	5.955692	6.185855	6.212529	6.420725	Six1	NM_009189
6801691	3471	6.861664	7.179568	6.816924	7.000203	-0.13854	0.403806	0.179365	0.28682	-0.18328	0.277182	0.362645	0.049909	-0.3179	0.077753	0.044741	0.783147	6801691	6.833648	7.030012	6.721333	7.080012	7.436707	7.021986	6.649817	6.870035	6.930919	7.056154	6.75047	7.193986	Six4	NM_011382
6801703	3472	7.336931	7.375783	7.315108	7.165753	0.171178	0.153383	0.21003	0.088993	0.149355	0.20605	0.060675	0.591413	-0.03885	0.729606	0.021822	0.845657	6801703	7.212468	7.402995	7.395329	7.282428	7.580695	7.264227	7.429229	7.135154	7.380942	7.143545	7.129725	7.223989	Trmt5	NM_029580
6801729	3473	8.586336	8.812003	8.84794	8.850411	-0.26407	0.069722	-0.03841	0.768606	-0.00247	0.984852	-0.03594	0.783037	-0.22567	0.11152	-0.2616	0.071877	6801729	8.436571	8.713233	8.609205	8.8216	8.856189	8.758219	9.083831	8.891994	8.567994	8.773557	8.855149	8.922528	Tmem30b	NM_178715
6801823	3476	8.472083	8.526319	8.569579	8.528276	-0.05619	0.464092	-0.00196	0.979288	0.041302	0.587507	-0.04326	0.570281	-0.05424	0.479284	-0.0975	0.21896	6801823	8.504383	8.497756	8.414111	8.448397	8.678706	8.451853	8.581586	8.634311	8.492839	8.431761	8.574522	8.578546	Ppp2r5e	NM_012024
6801838	3477	8.771892	8.979283	9.161475	9.131549	-0.35966	0.001888	-0.15227	0.09056	0.029926	0.715169	-0.18219	0.050306	-0.20739	0.030626	-0.38958	0.00116	6801838	8.789997	8.744233	8.781445	8.935451	9.055567	8.946831	9.202845	9.225362	9.056217	9.181075	8.957401	9.25617	Sgpp1	NM_030750
6801875	3480	6.787641	6.857239	6.937182	6.651627	0.136014	0.311406	0.205612	0.141024	0.285554	0.053015	-0.07994	0.54311	-0.0696	0.595442	-0.14954	0.268914	6801875	6.769611	6.895083	6.69823	6.822351	7.112957	6.636409	7.051009	6.762612	6.997924	6.579596	6.68641	6.688876	Zbtb25	NM_028356
6801902	3481	6.900554	6.888122	6.721665	6.705867	0.194686	0.366999	0.182255	0.396873	0.015797	0.940065	0.166457	0.43731	0.012432	0.952814	0.178889	0.405256	6801902	6.765078	6.707584	7.228999	7.177817	6.689108	6.797441	6.821556	6.675318	6.66812	6.387583	6.732065	6.997954	Spnb1	NM_013675
6801914	3482	9.346243	8.990593	8.965303	7.756823	1.58942	0.006548	1.23377	0.022189	1.208479	0.024264	0.02529	0.955181	0.35565	0.438413	0.38094	0.407873	6801914	10.14714	9.140158	8.751431	8.965921	9.206039	8.799819	9.763303	8.736926	8.395679	7.454307	7.981352	7.834811	Gpx2	NM_030677
6801917	3483	8.159946	8.201714	8.303436	7.962127	0.197819	0.005043	0.239587	0.001678	0.341309	0.000169	-0.10172	0.084647	-0.04177	0.442476	-0.14349	0.024086	6801917	8.124576	8.198867	8.156395	8.20281	8.218996	8.183337	8.246949	8.406776	8.256584	7.900967	7.933639	8.051776	Rab15	NM_134050
6801929	3484	9.103283	9.167613	9.236225	9.354076	-0.25079	0.178196	-0.18646	0.304411	-0.11785	0.507584	-0.06861	0.696944	-0.06433	0.714838	-0.13294	0.456519	6801929	9.071774	9.353189	8.884885	9.264741	9.076124	9.161974	9.231637	9.555807	8.92123	9.262844	9.442283	9.3571	Max	NM_008558
6802023	3485	8.785362	8.959432	8.778736	8.842645	-0.05728	0.698946	0.116787	0.43733	-0.06391	0.666491	0.180697	0.241539	-0.17407	0.257773	0.006627	0.964141	6802023	8.83994	8.782314	8.733833	8.991806	8.960134	8.926357	8.912827	8.918399	8.504981	8.638928	8.767784	9.121224	Atp6v1d	NM_023721
6802026	3486	7.847272	8.019674	7.651778	7.215926	0.631346	0.04596	0.803748	0.016957	0.435852	0.141915	0.367896	0.206346	-0.1724	0.537324	0.195494	0.485774	6802026	7.560414	7.614715	8.366686	7.726003	8.23394	8.099079	7.833611	7.435553	7.686169	7.121513	7.594662	6.931603	Plek2	NM_013738
6802031	3487	6.981509	6.911957	7.025036	6.689892	0.291617	0.17295	0.222065	0.287498	0.335144	0.123819	-0.11308	0.57775	0.069552	0.73042	-0.04353	0.828873	6802031	7.038888	7.02099	6.884649	7.071098	7.12504	6.539733	7.294062	6.710177	7.070868	6.54235	6.877749	6.649577	6330442E	BC066067
6802035	3488	7.197419	7.28779	7.266739	7.280328	-0.08291	0.48012	0.007462	0.948496	-0.01359	0.90638	0.021051	0.855531	-0.09037	0.442873	-0.06932	0.553011	6802035	7.10589	7.279016	7.207351	7.194259	7.315634	7.353477	7.432883	7.367595	6.999739	7.220318	7.251334	7.369333	Pigh	NM_029988
6802040	3489	8.553272	8.640874	8.560647	8.494753	0.05852	0.639925	0.146122	0.25944	0.065894	0.599059	0.080227	0.523886	-0.0876	0.487552	-0.00737	0.952655	6802040	8.580023	8.58248	8.497314	8.589653	8.61994	8.71303	8.731944	8.668858	8.281139	8.328274	8.587718	8.568266	Vti1b	NM_016800
6802041	3490	7.938208	8.56707	8.358417	8.343246	-0.40504	0.079729	0.223823	0.299776	0.015171	0.941942	0.208652	0.331577	-0.62886	0.014336	-0.42021	0.070951	6802041	8.013337	7.939605	7.861681	8.859906	8.359177	8.482126	8.333708	8.401977	8.339567	8.231309	7.999393	8.799037	Rdh11	NM_021557
6802043	3491	7.661624	7.872935	7.493872	7.621897	0.039726	0.813327	0.251038	0.161584	-0.12803	0.454225	0.379063	0.048253	-0.21131	0.230393	0.167752	0.332883	6802043	7.679673	7.693457	7.611741	7.910335	7.938775	7.769696	7.284003	7.640041	7.557572	7.592526	7.299715	7.973451	Zfyve26	NM_001008550
6802088	3492	9.238367	9.210015	9.206369	9.109187	0.12918	0.391492	0.100828	0.499627	0.097182	0.514822	0.003646	0.980231	0.028352	0.847374	0.031997	0.828094	6802088	9.392837	9.323195	8.999068	9.195447	9.485106	8.949492	9.125362	9.251135	9.242611	9.126842	9.128846	9.071873	Zfp36l1	NM_007564
6802098	3493	10.47837	10.44428	10.23883	10.34192	0.136457	0.378445	0.102367	0.504119	-0.10309	0.501215	0.205453	0.198004	0.03409	0.821674	0.239543	0.140339	6802098	10.37645	10.44496	10.61371	10.35383	10.70305	10.27597	10.48039	9.996002	10.2401	10.33839	10.28698	10.40038	Actn1	NM_134156
6802102	3494	8.231962	8.246391	8.206799	8.221647	0.010315	0.920148	0.024744	0.810256	-0.01485	0.885307	0.039592	0.701691	-0.01443	0.888518	0.025163	0.807119	6802102	8.225237	8.185102	8.285548	8.361712	8.394918	7.982544	8.231825	8.236072	8.1525	8.166143	8.258381	8.240417	Wdr22	NM_177267

6802139	3495	6.070203	6.068256	6.081688	6.1345	-0.0643	0.543799	-0.06624	0.531971	-0.05281	0.616664	-0.01343	0.897905	0.001947	0.985156	-0.01149	0.912624	6802139	6.090575	6.037935	6.082098	6.074127	6.014365	6.116276	6.125256	6.188489	5.93132	6.19029	5.911661	6.301548	Gm1568	NM_001008423
6802181	3498	8.068287	8.02715	8.051308	8.165572	-0.09728	0.155262	-0.13842	0.056068	-0.11426	0.102578	-0.02416	0.706968	0.041137	0.525663	0.016979	0.791131	6802181	8.047438	8.090302	8.067122	7.933507	8.067086	8.080857	8.013393	8.126098	8.014433	8.043878	8.200398	8.252441	EG214321	NM_001038995
6802182	3499	6.41065	5.760043	5.89864	6.296452	0.114198	0.569396	-0.53641	0.023681	-0.39781	0.072609	-0.1386	0.492014	0.650607	0.009643	0.51201	0.028811	6802182	6.224005	6.63517	6.372775	5.951634	5.609462	5.719032	5.609194	5.847497	6.23923	6.495393	6.329526	6.064436	Adam4	NM_009620
6802184	3500	7.201769	7.205106	7.303164	7.176388	0.025381	0.83101	0.028718	0.809274	0.126776	0.302769	-0.09806	0.419051	-0.00334	0.977583	-0.1014	0.404075	6802184	7.159735	7.364509	7.081062	7.015468	7.437722	7.162127	7.247202	7.351129	7.311162	7.077999	7.271636	7.179529	Med6	NM_027213
6802197	3502	6.880972	7.047364	6.925745	7.185989	-0.30502	0.080612	-0.13862	0.390104	-0.26024	0.126444	0.121619	0.448373	-0.16639	0.307198	-0.04477	0.776637	6802197	6.830595	6.888385	6.923937	7.24509	7.162047	6.734955	6.798717	7.20767	6.770848	7.128511	7.199448	7.230007	Map3k9	NM_177395
6802262	3503	5.953118	6.086477	6.179927	6.473869	-0.52075	0.017781	-0.38739	0.05791	-0.29394	0.131803	-0.09345	0.60814	-0.13336	0.468245	-0.22681	0.231438	6802262	5.865335	5.953989	6.04003	6.192269	5.953449	6.113713	5.913884	6.517118	6.108779	6.226534	6.452795	6.742279	Dpf3	NM_058212
6802265	3504	8.110674	8.32066	8.156933	8.099783	0.010891	0.922507	0.220878	0.07616	0.057151	0.612644	0.163727	0.169709	-0.20999	0.088965	-0.04626	0.681072	6802265	8.155313	8.179061	7.997648	8.410635	8.329714	8.221632	8.218485	8.176568	8.075747	8.027493	7.92951	8.342345	Zfyve1	NM_183154
6802290	3505	8.786261	8.786998	8.833479	8.703533	0.082728	0.5963	0.083465	0.593092	0.129946	0.411499	-0.04648	0.764552	-0.00074	0.9962	-0.04722	0.760953	6802290	8.648351	8.855216	8.855216	8.744201	8.8463	8.770493	8.644639	9.1368	8.718997	8.705473	8.920524	8.484601	Numb	NM_001136075
6802313	3507	5.931684	6.043395	5.831819	6.126906	-0.19522	0.048037	-0.08351	0.347768	-0.29509	0.007795	0.211576	0.035421	-0.11171	0.218854	0.099864	0.267142	6802313	5.944103	6.006341	5.844607	5.931951	6.016604	6.18163	5.878576	5.838173	5.778709	6.097752	6.014513	6.268452	Fam161b	NM_172581
6802315	3508	8.001288	8.112727	8.087934	8.346692	-0.3454	0.207519	-0.23396	0.380138	-0.25876	0.334343	0.024794	0.924011	-0.11144	0.669902	-0.08665	0.739722	6802315	8.137167	7.885334	7.981362	8.355552	8.039736	7.942894	7.96354	8.551323	7.748938	8.051759	8.211034	8.777284	Entpd5	NM_001026214
6802316	3509	8.140035	8.674163	8.516506	9.225493	-1.08546	0.002792	-0.55133	0.062821	-0.70899	0.024026	0.157658	0.554021	-0.53413	0.069768	-0.37647	0.178521	6802316	7.970792	8.188642	8.26067	8.928734	8.399535	8.694221	8.333575	8.918989	8.296953	9.15383	8.846304	9.676345	Aldh6a1	NM_134042
6802333	3510	7.458638	7.580729	7.416247	7.385799	0.072838	0.135626	0.19493	0.002166	0.030448	0.507539	0.164482	0.005647	-0.12209	0.023872	0.04239	0.36247	6802333	7.4639	7.49142	7.420593	7.638834	7.48779	7.615564	7.418686	7.471852	7.358204	7.370079	7.377737	7.409582	Abcd4	NM_008992
6802349	3511	11.59829	11.61061	11.79762	12.03203	-0.43374	0.101023	-0.42142	0.109505	-0.23441	0.345961	-0.18701	0.447416	-0.01232	0.959317	-0.19933	0.419233	6802349	11.80347	11.50962	11.48178	11.62317	11.48333	11.72533	11.89559	12.1635	11.33377	12.39373	11.92057	11.78179	Npc2	NM_023409
6802352	3512	7.993082	7.92929	7.302043	6.828531	1.164551	0.011546	1.100759	0.015107	0.473512	0.221733	0.627247	0.117279	0.063792	0.862753	0.69104	0.08919	6802352	8.051213	8.468353	7.459681	7.794278	8.460309	7.533283	7.789178	6.852833	7.264117	6.630042	7.103662	6.751889	Ltbp2	NM_013589
6802355	3513	8.78695	8.798844	8.777719	8.659049	0.127901	0.159461	0.139795	0.128442	0.11867	0.188046	0.021125	0.804258	-0.01189	0.888875	0.009232	0.913613	6802355	8.871474	8.715356	8.774021	8.805423	8.961799	8.62931	8.805003	8.793099	8.735054	8.574356	8.712546	8.690245	1110018G	NM_178065
6802364	3514	6.010186	5.929866	6.042246	6.032856	-0.02267	0.817869	-0.10299	0.311111	0.00939	0.923901	-0.11238	0.271972	0.080321	0.423593	-0.03206	0.745101	6802364	5.854975	5.95964	6.215944	6.006927	5.881168	5.901502	6.002914	6.158969	5.964854	5.969253	6.022503	6.106812	Prox2	NM_175198
6802371	3515	7.39103	7.346935	7.277917	7.176046	0.214984	0.046136	0.170889	0.097807	0.101871	0.296366	0.069018	0.470836	0.044095	0.641661	0.113113	0.249968	6802371	7.549949	7.326772	7.29637	7.315883	7.460686	7.264236	7.280303	7.264679	7.288769	7.24345	7.271934	7.012755	Rps6kl1	NM_146244
6802379	3516	8.24004	8.81235	8.266266	8.236191	0.003849	0.975565	0.576159	0.001482	0.030075	0.811193	0.546084	0.002047	-0.57231	0.001544	-0.02623	0.834911	6802379	8.312994	8.396358	8.010768	8.858011	8.923836	8.655203	8.220964	8.153825	8.424009	8.196912	8.170365	8.341296	Pgf	NM_008827
6802382	3517	7.251368	7.336177	7.467102	7.370285	-0.11892	0.205641	-0.03411	0.703079	0.096816	0.294586	-0.13092	0.167821	-0.08481	0.354652	-0.21573	0.036992	6802382	7.109367	7.357562	7.287176	7.392128	7.28612	7.330283	7.561989	7.486351	7.352965	7.235719	7.410525	7.464612	Mlh3	NM_175337
6802384	3518	7.631928	7.729204	7.652494	8.023888	-0.39196	0.071514	-0.29468	0.157127	-0.37139	0.084676	0.07671	0.695124	-0.09728	0.620265	-0.02057	0.915928	6802384	7.534031	7.761826	7.599927	7.822607	7.55538	7.809625	7.750868	7.79751	7.409103	7.894071	7.742753	8.434841	Acyp1	NM_025421
6802386	3519	8.817918	8.867215	8.832406	8.709794	0.108125	0.230906	0.157421	0.095751	0.122612	0.179666	0.034809	0.687342	-0.0493	0.570734	-0.01449	0.866392	6802386	8.809862	8.822639	8.821639	8.821754	8.961725	8.818165	9.012341	8.7241	8.760776	8.592364	8.765385	8.771632	Nek9	NM_145138
6802387	3520	10.1448	10.17467	10.09048	9.985721	0.159081	0.255146	0.188952	0.183508	0.104763	0.442872	0.084189	0.53471	-0.02987	0.823743	0.054318	0.686567	6802387	10.173	10.28479	9.976618	10.03569	10.26354	10.22479	10.36383	9.975419	9.932205	9.923994	10.06654	9.96663	Tmed10	NM_026775
6802433	3521	9.749889	9.892898	9.791207	9.94353	-0.19364	0.049355	-0.05063	0.561862	-0.15232	0.106171	0.101691	0.258872	-0.14301	0.12579	-0.04132	0.634705	6802433	9.675535	9.796966	9.777165	9.937363	9.868591	9.872741	9.86906	9.811244	9.693317	9.77336	9.94779	10.10944	0610007P	BC004591
6802449	3522	7.197781	7.052037	6.916069	6.329583	0.868198	0.021039	0.722454	0.044352	0.586487	0.089122	0.135968	0.665728	0.145744	0.643607	0.281712	0.379999	6802449	7.125988	7.502433	6.964922	7.066574	7.565721	6.523816	7.276005	6.717628	6.754575	5.964811	6.593365	6.430572	Tgfb3	NM_009368
6802485	3523	7.00699	6.967154	7.083986	7.020759	-0.01377	0.791099	-0.0536	0.317391	0.063227	0.243954	-0.11683	0.048609	0.039836	0.450964	-0.077	0.164145	6802485	7.043426	6.94061	7.036934	7.022379	7.000262	6.878822	7.095345	7.031141	7.125471	7.089567	6.987141	6.985569	Angel1	NM_144524
6802491	3524	7.833044	7.911721	7.908994	7.887724	-0.05468	0.793302	0.023997	0.908283	0.021271	0.918658	0.002727	0.989551	-0.07868	0.706831	-0.07595	0.716459	6802491	7.897099	7.813732	7.788301	7.908535	8.185828	7.6408	7.856271	8.313704	7.557008	7.743281	7.882493	8.037397	6430527G	AF525300
6802507	3526	7.691781	7.741457	7.833115	7.920518	-0.22874	0.021136	-0.17906	0.055491	-0.0874	0.306208	-0.09166	0.284824	-0.04968	0.551742	-0.14133	0.115152	6802507	7.685547	7.755411	7.634385	7.675235	7.857902	7.691233	7.791915	7.997822	7.709609	7.900162	7.976426	7.884965	Pomt2	NM_153415
6802525	3527	8.177336	8.41357	8.303239	8.383967	-0.20663	0.036294	0.029602	0.728323	-0.08073	0.355253	0.11033	0.216766	-0.23623	0.020792	-0.1259	0.164494	6802525	8.113586	8.234598	8.183823	8.321012	8.481571	8.438126	8.421175	8.217238	8.271305	8.270742	8.344395	8.536765	AI413782	NM_001142580
6802532	3528	10.44908	10.27266	10.37803	10.25191	0.19717	0.107683	0.02075	0.853575	0.126113	0.280066	-0.10536	0.361442	0.17642	0.143752	0.071057	0.532224	6802532	10.57838	10.39698	10.37189	10.21218	10.37884	10.22697	10.54488	10.29416	10.29504	10.05567	10.36148	10.33859	Sptlc2	NM_011479
6802557	3530	8.579067	8.778068	8.665417	8.61343	-0.03436	0.770492	0.164638	0.186187	0.051987	0.6601	0.112651	0.35144	-0.199	0.118625	-0.08635	0.469955	6802557	8.497399	8.536619	8.703183	8.705157	8.875159	8.753889	8.843225	8.691159	8.461868	8.494312	8.56964	8.776339	Snw1	NM_025507
6802727	3532	7.734972	6.648521	6.673778	5.693715	2.041256	0.00648	0.954806	0.126012	0.980062	0.117657	-0.02526	0.965071	1.08645	0.087867	1.061194	0.094208	6802727	8.199222	8.190465	6.815228	6.698977	7.433877	5.81271	7.485836	6.134942	6.400555	5.635223	5.981332	5.464591	Dio2	NM_010050
6802732	3533	7.448304	7.35596	7.301888	7.537617	-0.08931	0.475241	-0.18166	0.166103	-0.23573	0.083413	0.054073	0.662214	0.092344	0.460892	0.146417	0.254325	6802732	7.54084	7.360514	7.443559	7.132375	7.657434	7.278072	7.349619	7.239386	7.316658	7.547088	7.512843	7.552919	4930534B	NM_181815
6802744	3534	11.65848	11.77708	11.79516	12.05179	-0.39331	0.084565	-0.27472	0.206467	-0.25663	0.234978	-0.01808	0.930117	-0.1186	0.569238	-0.13668	0.513295	6802744	11.53219	11.75245	11.6908	11.56602	11.77216	11.99305	11.68844	12.25956	11.43748	12.08768	11.9829	12.0848	Gtf2a1	NM_031391
6802745	3535	8.430379	8.579115	8.308149	8.368379	0.062001	0.748756	0.210736	0.292458	-0.06023	0.755657	0.270965	0.18539	-0.14874	0.449368	0.12223	0.531699	6802745	8.487566	8.255948	8.547624	8.355491	8.98998	8.391873	8.196198	8.316423	8.411827	8.294788	8.613589	8.196759	Ston2	NM_175367
6802749	3536	10.17978	10.11661	10.48532	10.27285	-0.09307	0.584808	-0.15624	0.367218	0.212471	0.229926	-0.36871	0.054118	0.063175	0.709258	-0.30554	0.098577	6802749	10.14237	10.16668	10.23029	9.876145	10.33231	10.14136	10.5037	10.58492	10.36733	10.33353	10.54412	9.940886	Sel1l	NM_001039089
6802989	3538	8.055515	8.765201	8.574084	9.300172	-1.24466	0.000721	-0.53497	0.051937	-0.72609	0.014738	0.191117	0.438597	-0.70969	0.016389	-0.51857	0.057927	6802989	8.023116	8.219653	7.923775	8.708215	8.497301	9.090086	8.699454	8.825642	8.197155	9.23749	9.009947	9.653079	Galc	NM_008079
6803028	3540	8.08229	8.033203	8.055969	8.001333	0.080958	0.676201	0.03187	0.868774	0.054636	0.777373	-0.02277	0.906012	0.049087	0.799375	0.026321	0.891435	6803028	7.983808	8.058926	8.204137	7.976695	8.179948	7.942966	7.878965	8.536611	7.752331	7.939611	8.015266	8.049121	Ptpn21	NM_011877
6803038	3542	8.448251	8.472515	8.280447	8.25824	0.190011	0.099642	0.214275	0.068976	0.022207	0.833186	0.192068	0.096602	-0.02426	0.818044	0.167804	0.138739	6803038	8.416091	8.548933	8.379729	8.496707	8.59634	8.324498	8.378813	8.344568	8.117961	8.114487	8.3371	8.323134	Foxn3	NM_183186
6803050	3543	8.292041	8.268096	7.832616	8.165062	0.126979	0.524064	0.103035	0.603555	-0.33245	0.119312	0.435481	0.051703	0.023944	0.903138	0.459425	0.042498	6803050	8.246249	8.454134	8.175739	8.441091	8.397341	7.965857	8.070569	7.584064	7.843214	8.039701	8.466562	7.988922	Foxn3	BC029185
6803088	3545	7.028822	7.202176	7.176293	7.027049	0.001772	0.980556	0.175127	0.037843	0.149243	0.067103	0.025883	0.722986	-0.17335	0.039355	-0.14747	0.06978	6803088	7.045298	7.027283	7.013884	7.123711	7.17103	7.311787	7.302413	7.060922	7.165543	7.039475	6.948664	7.093009	BC002230	NM_183155
6803102	3546	8.553349	8.580921	8.48938	7.76584	0.787509	0.00561	0.815081	0.004654	0.72354	0.008732	0.091541	0.674282	-0.02757	0.898733	0.063968	0.76833	6803102	8.748138	8.28386	8.628048	8.40152	8.894449	8.446795	8.803665	8.123227	8.541249	7.770702	7.882798	7.64402	Rps6ka5	NM_153587
6803113	3547	6.368027	6.017314	6.133973	6.010681	0.357347	0.11339	0.006633	0.974489	0.123292	0.556733	-0.11666	0.577708	0.350714	0.119233	0.234054	0.277887	6803113	6.359498	6.231021	6.513563	6.258224	6.056147	5.73757	6.153872	5.786399	6.461648	6.034389	6.196032	5.801621	Gpr68	NM_175493
6803120	3548	7.372022	7.55571	7.383719	7.462416	-0.09039	0.408465	0.093294	0.394265	-0.0787	0.469388	0.171991	0.135558	-0.18369	0.114238	-0.0117	0.912909	6803120	7.325988	7.295276	7.494801	7.501164	7.70386	7.462107	7.420302	7.43795	7.292905	7.416034	7.317762	7.653452	Ccdc88c	NM_026681
6803122	3549	8.393713	8.541396	8.453428	8.424259	-0.03055	0.751493	0.117137	0.244229	0.029169	0.762286	0.087968	0.372828	-0.14768	0.151689	-0.05972	0.539585	6803122	8.361504	8.455577	8.364057	8.354677	8.615719	8.653793	8.436489	8.591557	8.332238	8.337336	8.47343	8.462011	Smek1	NM_211355
6803132	3550	8.880964	9.089278	8.915523	9.43925	-0.55829	0.04473	-0.34997	0.174482	-0.52373	0.05628	0.173755	0.480505	-0.20831	0.400924	-0.03456	0.886646	6803132	8.428468	9.348478	8.865947	8.964504	9.080495	9.222836	8.573578	9.054965	9.118026	9.310513	9.53898	9.468256	Tc2n	NM_001082976
6803133	3551	7.786634	8.32516	6.963042	7.483787	0.302848	0.443557	0.841373	0.0555	-0.52074	0.203197	1.362117	0.006734	-0.53853	0.189707	0.823592	0.059751	6803133	7.741357	8.037069	7.581477	8.052871	8.872672	8.049936	7.319877	6.530584	7.038666	7.033386	8.215662	7.202312	Fbln5	NM_011812
6803136	3552	7.241373	7.395282	7.231868	7.231265	0.010107	0.916001	0.164017	0.115318	0.000602	0.994983	0.163414	0.116463	-0.15391	0.135998	0.009505	0.920986	6803136	7.193898	7.360497	7.169723	7.219112	7.539853	7.426881	7.301543	7.151451	7.242609	7.128463	7.25386	7.311473	Atxn3	NM_029705
6803161	3553	10.73993	11.1047	10.94466	10.65636	0.083563	0.661519	0.44834	0.040644	0.288293	0.155479	0.160047	0.40935	-0.36478	0.082511	-0.20473	0.297777	6803161	10.90652	10.63214	10.68112	11.04009	11.11125	11.16277	11.41029	10.76207	10.66161	10.57287	10.61318	10.78304	Lgmn	NM_011175
6803172	3554	9.435835	9.443443	9.542639	9.416473	0.019362	0.919554	0.026971	0.888154	0.126166	0.516193	-0.0992	0.607866	-0.00761	0.968333	-0.1068	0.581134	6803172	9.427776	9.404076	9.475652	9.737007	9.185908	9.403815	9.587464	9.684395	9.356058	9.121917	9.315448	9.772751	Itpk1	NM_172584
6803173	3555	6.583299	6.765499	6.710244	6.6842	-0.1009	0.41179	0.081299	0.505177	0.026043	0.828764	0.055255	0.648066	-0.1822	0.156571	-0.12695	0.307735	6803173	6.627873	6.636751	6.485272	6.757247	6.79028	6.74897	6.961223	6.593266	6.576242	6.541147	6.650295	6.861159	Moap1	NM_022323
6803178	3556	8.255537	8.337527	8.287579	8.034447	0.22109	0.009942	0.303079	0.001744	0.253132	0.004904	0.049948	0.469664	-0.08199	0.248089	-0.03204	0.639415	6803178	8.217595	8.32424	8.224776	8.258671	8.447768	8.306141	8.308228	8.363057	8.191452	7.973181	8.112623	8.017538	Btbd7	NM_172806
6803192	3557	6.322215	6.308584	6.343737	6.40093	-0.07871	0.465518	-0.09235	0.394917	-0.05719	0.592916	-0.03515	0.741011	0.013631	0.897709	-0.02152	0.839287	6803192	6.233997	6.335971	6.396678	6.282526	6.267612	6.375614	6.340402	6.548285	6.142524	6.376213	6.306039	6.520539	Prima1	NM_133364
6803208	3558	8.978539	8.980068	9.050392	9.036038	-0.0575	0.34975	-0.05597	0.362013	0.014353	0.810455	-0.07032	0.259156	-0.00153	0.979579	-0.07185	0.249764	6803208	8.997682	8.962423	8.975512	8.944033	9.034396	8.961775	9.064051	9.162107	8.925017	8.98024	9.032695	9.09518	Ddx24	NM_001159502
6803210	3559	7.554415	7.698567	7.439199	6.797821	0.756595	0.086535	0.900746	0.048495	0.641378	0.136327	0.259368	0.521937	-0.14415	0.719427	0.115217	0.773685	6803210	6.931255	7.405025	8.326966	7.579974	8.082546	7.43318	7.950702	7.053629	7.313265	6.525858	7.042624	6.82498	Ifi27l2a	NM_029803
6803211	3560	6.268498	6.255803	6.14333	6.287539	-0.01904	0.798207	-0.03174	0.671183	-0.14421	0.08027	0.112473	0.157053	0.012695	0.864483	0.125168	0.120475	6803211	6.212668	6.195695	6.397131	6.217155	6.148558	6.401695	6.13521	6.131609	6.163171	6.312884	6.247833	6.301899	Ifi27l2b	NM_145449
6803249	3565	6.330995	5.400665	5.904562	5.436007	0.894988	0.120741	-0.03534	0.947022	0.468555	0.389937	-0.5039	0.356945	0.93033	0.10876	0.426433	0.432083	6803249	6.676785	5.546136	6.770064	5.278329	5.341949	5.581716	4.934066	6.654284	6.125336	5.884079	5.633492	4.790449	Serpina1	NM_009247
6803297	3570	7.015071	7.136714	7.04123	7.049195	-0.03412	0.472197	0.087518	0.089041	-0.00796	0.864594	0.095483	0.067765	-0.12164	0.027523	-0.02616	0.578935	6803297	7.042305	7.014325	6.988582	7.192005	7.026571	7.191565	7.084722	7.041545	6.997424	7.07578	7.040267	7.031539	Gsc	NM_010351
6803314	3571	8.493287	8.497008	8.419998	8.373163	0.120124	0.245997	0.123845	0.232894	0.046835	0.638597	0.07701	0.445436	-0.00372	0.970021	0.073289	0.466942	6803314	8.566925	8.54529	8.367646	8.474212	8.642623	8.374188	8.408021	8.499607	8.352365	8.289073	8.533777	8.296638	Dicer1	NM_148948
6803319	3572	10.27429	10.18634	10.18881	10.03181	0.242484	0.193167	0.154527	0.391701	0.156997	0.384532	-0.00247	0.988806	0.087957	0.620238	0.085486	0.629941	6803319	10.36861	10.0962	10.35807	10.32418	10.23033	10.0045	10.19409	10.41181	9.960521	10.01711	9.769239	10.30908	Clmn	NM_053155
6803321	3573	6.447179	6.376659	6.267851	6.410366	0.036813	0.454333	-0.03371	0.492022	-0.14252	0.015962	0.108808	0.048601	0.07052	0.170405	0.179328	0.005014	6803321	6.466545	6.441671	6.433321	6.401968	6.35031	6.377699	6.299948	6.357428	6.146176	6.428717	6.40244	6.399942	4831426I	NM_001042699
6803358	3575	7.779288	7.916827	7.804341	7.855769	-0.07648	0.351951	0.061058	0.452859	-0.05143	0.525025	0.112486	0.184145	-0.13754	0.113417	-0.02505	0.754432	6803358	7.797259	7.790137	7.750467	7.83213	7.975923	7.942428	7.790812	7.965906	7.656306	7.940835	7.823651	7.802821	Atg2b	NM_029654

6803364	3576	7.68421	7.76649	7.721326	7.938898	-0.25469	0.018886	-0.17241	0.082275	-0.21757	0.036581	0.045164	0.616965	-0.08228	0.370967	-0.03712	0.68025	6803364	7.751333	7.679022	7.622276	7.750285	7.877496	7.67169	7.616929	7.867561	7.679489	8.061757	7.833793	7.921143	Atg2b	NM_029654
6803524	3577	6.308893	6.329948	6.226094	6.354832	-0.04594	0.800267	-0.02488	0.890834	-0.12874	0.484486	0.103854	0.570641	-0.02106	0.907534	0.082799	0.649925	6803524	6.293911	6.496047	6.13672	6.116393	6.388349	6.485101	6.048857	6.247118	6.382307	6.317997	6.668116	6.078383	Bcl11b	NM_001079883
6803525	3578	6.952181	6.872824	6.864563	6.661748	0.290432	0.444624	0.211076	0.575104	0.202815	0.589864	0.008261	0.982315	0.079356	0.831619	0.087617	0.814455	6803525	6.788316	7.251151	6.817075	6.965142	6.867732	6.785599	6.561274	6.775911	7.256505	6.521692	7.484211	5.979342	A130014H	AK037594
6803527	3579	9.409803	9.519759	9.463817	9.537058	-0.12725	0.177911	-0.0173	0.845871	-0.07324	0.419974	0.055942	0.534325	-0.10996	0.237667	-0.05401	0.548172	6803527	9.366772	9.412651	9.449987	9.54712	9.497748	9.514408	9.478268	9.486412	9.42677	9.393675	9.448429	9.76907	Setd3	NM_028262
6803596	3581	7.684191	7.708048	7.879313	8.029031	-0.34484	0.011923	-0.32098	0.016724	-0.14972	0.197467	-0.17126	0.146525	-0.02386	0.828388	-0.19512	0.104337	6803596	7.521878	7.820629	7.710067	7.585947	7.846741	7.691457	7.837759	8.043471	7.756709	7.941516	8.051573	8.094004	Slc25a29	NM_181328
6803598	3582	8.629366	8.407301	8.774782	8.47412	0.155245	0.170943	-0.06682	0.535489	0.300662	0.019495	-0.36748	0.007394	0.222065	0.063615	-0.14542	0.196509	6803598	8.63057	8.668643	8.588884	8.372697	8.590945	8.25826	8.868016	8.740774	8.715556	8.414556	8.660149	8.347656	Wars	NM_011710
6803770	3586	7.470417	7.715123	8.022673	7.686453	-0.21604	0.135967	0.02867	0.831385	0.33622	0.032621	-0.30755	0.045965	-0.24471	0.09725	-0.55226	0.002847	6803770	7.372739	7.649561	7.388952	7.781346	7.514712	7.849312	7.922644	8.249724	7.895652	7.585836	7.743965	7.729558	Rage	NM_011973
6803780	3587	7.764098	7.786629	7.707808	7.669914	0.094184	0.092162	0.116715	0.045237	0.037894	0.463699	0.078821	0.148131	-0.02253	0.659456	0.05629	0.286059	6803780	7.743961	7.742327	7.806007	7.765757	7.745593	7.848538	7.717818	7.781864	7.623743	7.621989	7.646891	7.740862	2810452K	NM_026048
6803787	3588	6.655347	6.643339	6.515106	6.993595	-0.33825	0.014325	-0.35026	0.012127	-0.47849	0.002265	0.128234	0.271466	0.012008	0.914655	0.140242	0.232511	6803787	6.616391	6.516032	6.833619	6.737499	6.55929	6.633229	6.53941	6.680466	6.325441	6.923611	7.001674	7.0555	Ankrd9	NM_175207
6803821	3589	9.177025	9.091083	9.062058	9.052203	0.124821	0.13047	0.03888	0.613905	0.009854	0.897455	0.029026	0.70541	0.085941	0.279426	0.114967	0.159259	6803821	9.245566	9.108917	9.176591	9.113975	9.194299	8.964976	9.103722	9.071434	9.011017	8.92477	9.133831	9.098009	Cdc42bpb	NM_183016
6803850	3590	9.391975	9.85565	9.604046	11.24805	-1.85607	0.003841	-1.3924	0.016667	-1.644	0.007393	0.251604	0.600652	-0.46368	0.34466	-0.21207	0.65823	6803850	9.587574	9.471855	9.116497	10.08165	9.340351	10.14495	9.393158	10.54895	8.870031	10.75635	11.17884	11.80896	Ckb	NM_021273
6803854	3591	7.748233	7.861452	7.629125	7.929163	-0.18093	0.030798	-0.06771	0.35609	-0.30004	0.00248	0.232327	0.009927	-0.11322	0.140151	0.119109	0.123232	6803854	7.780775	7.815802	7.648123	7.781384	7.869148	7.933823	7.591549	7.710124	7.585701	7.937743	7.824775	8.024971	Bag5	NM_027404
6803862	3592	6.992194	7.065648	7.103709	7.312784	-0.32059	0.35703	-0.24714	0.472795	-0.20907	0.541689	-0.03806	0.910488	-0.07345	0.828427	-0.11152	0.742641	6803862	6.711874	7.117146	7.147561	7.2724	6.997226	6.927317	6.857698	7.345422	7.108007	6.884539	6.930138	8.123675	Xrcc3	NM_028875
6803863	3593	8.562937	8.504251	8.58384	8.33088	0.232057	0.023359	0.173371	0.070176	0.25296	0.015886	-0.07959	0.365748	0.058685	0.49966	-0.0209	0.807535	6803863	8.518763	8.438544	8.731503	8.510148	8.527231	8.475375	8.56167	8.618602	8.571247	8.181565	8.396827	8.414248	Ppp1r13b	NM_011625
6803871	3594	9.224979	9.462876	9.26503	9.458411	-0.23343	0.238539	0.004465	0.981159	-0.19338	0.322182	0.197847	0.311831	-0.2379	0.230448	-0.04005	0.832491	6803871	9.314669	9.269724	9.090545	9.674257	9.361339	9.353033	9.476555	9.229238	9.089296	9.273747	9.250872	9.850614	2010107E	NM_027360
6803891	3596	6.187998	6.263603	6.229318	6.325684	-0.13769	0.033931	-0.06208	0.282643	-0.09637	0.111586	0.034285	0.542465	-0.0756	0.198286	-0.04132	0.465318	6803891	6.1896	6.278486	6.095909	6.361305	6.182945	6.246558	6.250011	6.201245	6.236697	6.306106	6.335693	6.335252	Tmem179	NM_178915
6803895	3597	7.7487	9.810511	9.764483	9.367863	-1.61916	7.69E-07	0.442648	0.005624	0.39662	0.009931	0.046029	0.706757	-2.06181	1.18E-07	-2.01578	1.4E-07	6803895	7.839261	7.750572	7.656266	9.778844	9.966145	9.686545	9.940247	9.7012	9.652001	9.327087	9.561192	9.21531	Akt1	NM_009652
6803912	3598	7.742092	7.88424	7.723252	7.542629	0.199463	0.135296	0.341612	0.021655	0.180624	0.170967	0.160988	0.216875	-0.14215	0.270507	0.018839	0.879227	6803912	7.730531	7.669529	7.826215	7.734977	8.121816	7.795928	7.766648	7.826551	7.576558	7.537295	7.686612	7.403979	Cdca4	NM_028023
6803913	3599	6.302547	6.129671	6.206164	6.035696	0.26685	0.094633	0.093975	0.523239	0.170467	0.260519	-0.07649	0.601716	0.172875	0.254377	0.096383	0.512937	6803913	6.385677	6.223864	6.298099	6.047776	6.250017	6.091221	6.082889	6.097339	6.438263	6.193177	5.752075	6.161837	Gpr132	NM_019925
6803917	3600	7.009545	7.246432	6.949509	7.167415	-0.15787	0.182181	0.079018	0.485518	-0.21791	0.078492	0.296923	0.025151	-0.23689	0.059727	0.060036	0.593709	6803917	7.094081	6.997745	6.936808	7.336566	7.35143	7.051301	7.103278	6.860364	6.884885	7.128173	7.314036	7.060035	Jag2	NM_010588
6803918	3601	7.500376	7.468349	7.527713	7.514887	-0.01451	0.886452	-0.04654	0.649009	0.012826	0.899553	-0.05936	0.563172	0.032027	0.753262	-0.02734	0.788279	6803918	7.494145	7.551698	7.455285	7.556846	7.635829	7.212372	7.550808	7.573546	7.458785	7.5133	7.478392	7.55297	Nudt14	NM_025399
6803922	3602	7.954339	7.978315	7.843098	7.774769	0.17957	0.07142	0.203545	0.046348	0.068329	0.452072	0.135216	0.156407	-0.02398	0.788547	0.111241	0.234139	6803922	8.052784	7.840998	7.969236	8.059202	8.093627	7.782115	7.872038	7.884102	7.773155	7.757026	7.764956	7.802326	Brf1	NM_028193
6803946	3603	5.847099	5.866104	5.813615	5.736147	0.110951	0.553972	0.129957	0.490007	0.077467	0.677669	0.05249	0.777564	-0.01901	0.918344	0.033484	0.856769	6803946	5.891113	5.849537	5.800646	6.027101	6.006557	5.564655	5.835966	6.059671	5.545207	5.465835	5.915495	5.827112	Fusip1	NM_010178
6803978	3604	5.693442	5.799174	5.916491	6.02621	-0.33277	0.316974	-0.22704	0.48726	-0.10972	0.733993	-0.11732	0.716491	-0.10573	0.743245	-0.22305	0.494705	6803978	5.971421	5.128373	5.980531	5.486955	5.659945	6.250623	6.038874	5.783541	5.927058	6.497462	5.81215	5.769019	Igh	BC103785
6804024	3606	6.218418	6.376289	6.302298	6.363368	-0.14495	0.213537	0.012921	0.907088	-0.06107	0.584734	0.073991	0.509813	-0.15787	0.17927	-0.08388	0.456715	6804024	6.267902	6.305382	6.08197	6.384242	6.246596	6.498029	6.257915	6.275799	6.373181	6.166202	6.385425	6.538478	Wdr60	NM_146039
6804033	3607	7.810454	7.940557	7.732683	7.842831	-0.03238	0.697435	0.097725	0.258405	-0.11015	0.207502	0.207873	0.032213	-0.1301	0.143942	0.07777	0.361286	6804033	7.814298	7.760764	7.856299	7.927756	7.874181	8.019733	7.864101	7.611467	7.722482	7.81945	7.733883	7.975161	Wdr60	NM_146039
6804138	3609	7.432118	7.761391	7.847924	7.896441	-0.46432	0.018624	-0.13505	0.416911	-0.04852	0.766308	-0.08653	0.59834	-0.32927	0.070344	-0.41581	0.029923	6804138	7.287806	7.685828	7.32272	7.783818	7.693579	7.806777	7.721093	8.183176	7.639504	7.887635	7.795138	8.00655	Sp4	NM_009239
6804268	3610	8.590194	9.534285	9.05761	8.837973	-0.24778	0.687722	0.696312	0.275089	0.219637	0.721317	0.476675	0.445719	-0.94409	0.150852	-0.46742	0.454281	6804268	7.527407	8.912921	9.330254	9.424496	9.878747	9.299613	8.894146	9.478031	8.800654	9.384609	7.683108	9.446203	Cox7a2l	NM_001159529
6804379	3611	7.352228	7.476985	7.332097	7.267505	0.084723	0.431985	0.20948	0.074993	0.064592	0.545754	0.144888	0.19479	-0.12476	0.257779	0.020131	0.849041	6804379	7.46862	7.354758	7.233306	7.577948	7.459901	7.393107	7.542819	7.239554	7.213918	7.236451	7.204876	7.361188	Klf11	NM_178357
6804405	3612	7.887182	7.778921	8.103868	7.058098	0.829084	0.036959	0.720823	0.061502	1.04577	0.013537	-0.32495	0.355918	0.108261	0.75249	-0.21669	0.531878	6804405	7.814334	7.967049	7.880163	7.785242	7.797756	7.753765	8.394162	7.873739	8.043703	6.832969	7.909054	6.432271	Grhl1	NM_145890
6804486	3613	10.43091	10.4943	10.40756	10.23715	0.19376	0.106098	0.257143	0.042071	0.170403	0.147947	0.08674	0.438563	-0.06338	0.567859	0.023357	0.831754	6804486	10.43814	10.38505	10.46955	10.39354	10.63587	10.45348	10.63304	10.25524	10.33439	10.11938	10.30486	10.28722	Gdi2	NM_008112
6804497	3614	8.227131	8.122523	8.263812	8.244893	-0.01776	0.910613	-0.12237	0.447756	0.018919	0.904823	-0.14129	0.383643	0.104609	0.51425	-0.03668	0.816903	6804497	8.032466	8.32042	8.328508	8.073763	8.208838	8.084967	7.890132	8.483237	8.418066	8.206532	8.270644	8.257503	Asb13	NM_178283
6804582	3623	10.28679	10.18794	9.961055	9.769992	0.516795	0.027747	0.417945	0.061748	0.191064	0.350089	0.226881	0.272505	0.09885	0.62154	0.325731	0.129147	6804582	10.29468	10.08628	10.4794	10.24956	10.15221	10.16204	10.02176	9.956551	9.904855	9.962605	10.06048	9.28689	Klf6	NM_011803
6804608	3624	8.479954	8.592661	8.550418	8.624736	-0.14478	0.201902	-0.03207	0.765948	-0.07432	0.495735	0.042244	0.695635	-0.11271	0.310671	-0.07046	0.517723	6804608	8.421839	8.511636	8.506387	8.557612	8.645377	8.574995	8.727921	8.424675	8.498657	8.448259	8.60337	8.82258	Pitrm1	NM_145131
6804696	3628	10.14092	10.51351	10.24001	10.62399	-0.48307	0.011111	-0.11048	0.474047	-0.38398	0.031089	0.2735	0.099985	-0.37259	0.035069	-0.09909	0.519454	6804696	10.18064	10.15365	10.08848	10.63783	10.38302	10.51969	10.17572	10.28512	10.2592	10.50324	10.37281	10.99593	Larp5	NM_172585
6804698	3629	7.084344	7.014203	6.592482	7.341528	-0.25718	0.144018	-0.32733	0.073238	-0.74905	0.001509	0.421721	0.029009	0.070142	0.670429	0.491862	0.01473	6804698	6.946384	7.086251	7.220398	7.364967	6.756937	6.920704	6.567863	6.457226	6.752357	7.288606	7.271846	7.464133	A130023E	AK037522
6804703	3630	8.223844	8.364597	8.33153	8.128097	0.095747	0.568822	0.236501	0.180393	0.203433	0.242357	0.033067	0.842541	-0.14075	0.407871	-0.10769	0.522783	6804703	8.20327	8.183718	8.284543	8.293186	8.465978	8.334628	8.04077	8.304159	8.649661	8.333803	8.166055	7.884432	U2af2	NM_133671
6804849	3631	8.939164	8.562931	8.58366	7.819067	1.120097	0.023815	0.743864	0.101759	0.764593	0.094012	-0.02073	0.960188	0.376233	0.377254	0.355504	0.402848	6804849	8.904584	9.269343	8.643565	8.11673	9.150299	8.421764	9.054835	7.98737	8.708775	8.03925	8.217082	7.200869	Heatr1	NM_144835
6804893	3632	8.929778	9.068682	8.657331	8.442886	0.486892	0.048826	0.625796	0.017508	0.214444	0.336534	0.411351	0.085514	-0.1389	0.526447	0.272447	0.230172	6804893	9.055937	8.902593	8.830804	9.007164	9.38198	8.816902	8.81764	8.47806	8.676292	8.042504	8.780073	8.506082	Nid1	NM_010917
6804898	3633	7.231759	7.101396	6.961487	6.962907	0.268852	0.179268	0.138489	0.470088	-0.00142	0.993988	0.139909	0.465701	0.130363	0.495708	0.270272	0.177234	6804898	7.540292	7.126605	7.02838	7.008461	7.442379	6.853347	6.906133	6.878494	7.099833	6.865104	7.118789	6.904827	Lyst	NM_010748
6804900	3634	7.827718	7.854701	7.900699	7.925714	-0.098	0.810748	-0.07101	0.862116	-0.02501	0.951173	-0.046	0.910375	-0.02698	0.947338	-0.07298	0.858343	6804900	8.3864	7.475423	7.621331	7.783752	8.379957	7.400393	7.301461	7.88015	8.520485	7.659906	7.877888	8.239347	Gng4	NM_010317
6804901	3635	7.826539	8.076686	7.893654	8.164826	-0.33829	0.000859	-0.08814	0.215363	-0.27117	0.003256	0.183032	0.023414	-0.25015	0.005101	-0.06712	0.335573	6804901	7.746408	7.84415	7.889059	8.06347	8.058582	8.108005	7.846757	7.997434	7.836771	8.286187	8.127349	8.080943	B3galnt2	NM_178640
6804919	3636	8.847165	8.96047	8.814316	9.050726	-0.20356	0.061481	-0.09026	0.363417	-0.23641	0.035573	0.146153	0.157253	-0.1133	0.260886	0.032848	0.734843	6804919	8.799183	8.978205	8.764106	8.860146	8.951538	9.069725	8.729165	8.96052	8.753264	9.176458	9.007038	8.968681	Arid4b	NM_194262
6804938	3637	10.8449	10.8575	10.80776	10.7155	0.1294	0.313611	0.142	0.271907	0.092253	0.465341	0.049747	0.690188	-0.0126	0.91919	0.037147	0.765453	6804938	10.74037	10.86242	10.93192	10.71912	10.89404	10.95935	11.05339	10.6712	10.69868	10.56962	10.76277	10.81412	Psma2	NM_008944
6804940	3638	6.460053	6.609755	6.615281	6.728615	-0.26856	0.123074	-0.11886	0.467462	-0.11333	0.487744	-0.00553	0.972575	-0.1497	0.364807	-0.15523	0.348313	6804940	6.169567	6.671169	6.539422	6.426994	6.612586	6.789684	6.697517	6.520522	6.627805	6.676876	6.566876	6.942094	AW209491	NM_134067
6804996	3640	6.816749	6.804565	6.57578	6.433737	0.383012	0.103541	0.370828	0.11322	0.142043	0.514964	0.228785	0.304471	0.012184	0.954837	0.240969	0.28117	6804996	6.620619	7.192791	6.636837	6.79298	7.100177	6.520538	6.691971	6.399283	6.636087	6.402731	6.663213	6.235267	Inhba	NM_008380
6805058	3641	7.940647	7.864341	7.879983	7.898952	0.041695	0.763847	-0.03461	0.802877	-0.01897	0.891024	-0.01564	0.910033	0.076306	0.58502	0.060664	0.663064	6805058	7.92615	8.001703	7.894088	7.644881	8.091708	7.856433	8.051241	7.761941	7.826766	7.834774	8.100702	7.761381	2810021B	NM_025479
6805096	3642	8.598266	8.579996	8.599986	8.415477	0.182788	0.195186	0.164518	0.238993	0.184509	0.191446	-0.01999	0.880968	0.01827	0.891132	-0.00172	0.989711	6805096	8.593609	8.639266	8.561922	8.518203	8.616742	8.605042	8.871725	8.369086	8.559147	8.22093	8.456969	8.568533	Vps41	NM_172120
6805103	3643	7.411966	7.467175	7.525955	6.809716	0.60225	0.004683	0.657459	0.00286	0.716239	0.001726	-0.05878	0.714863	-0.05521	0.731361	-0.11399	0.483832	6805103	7.708698	7.172512	7.354689	7.48265	7.557478	7.361397	7.747609	7.26765	7.562605	6.844759	6.823987	6.760402	Amph	NM_175007
6805108	3645	6.281125	5.593675	6.628777	5.190088	1.091037	0.036004	0.403587	0.379149	1.438689	0.010566	-1.0351	0.044028	0.68745	0.151486	-0.34765	0.445792	6805108	6.791441	6.207592	5.844341	5.557512	5.919217	5.304295	7.215794	6.191422	6.479114	5.315053	5.843945	4.411266	Otud6b	NM_152812
6805134	3646	8.7412	8.057915	7.590616	6.627341	2.113859	0.036653	1.430574	0.12846	0.963275	0.286661	0.467299	0.594867	0.683285	0.44151	1.150584	0.209852	6805134	9.429086	8.430424	8.36409	7.496334	9.053123	7.624288	8.84634	6.294827	7.630682	5.243826	7.643336	6.994862	Sfrp4	NM_016687
6805158	3647	7.98736	8.204801	8.107094	7.857888	0.129472	0.447093	0.346913	0.064556	0.249206	0.162402	0.097707	0.562994	-0.21744	0.21622	-0.11973	0.480818	6805158	8.047995	8.074628	7.839456	8.085564	8.495927	8.032911	8.383417	7.864012	8.073853	7.882661	7.933686	7.757316	Elmo1	NM_080288
6805180	3648	5.728424	5.956111	6.163985	5.587916	0.140508	0.608728	0.368195	0.200274	0.576069	0.060489	-0.20787	0.453369	-0.22769	0.413162	-0.43556	0.13729	6805180	5.705815	5.876793	5.602664	5.790875	6.522985	5.554473	6.19263	5.977355	6.32197	5.61438	5.910064	5.239305	Aoah	NM_012054
6805194	3650	8.311425	8.412631	8.284362	8.256721	0.054704	0.493106	0.15591	0.074885	0.027641	0.726106	0.128269	0.1307	-0.10121	0.220625	0.027063	0.731567	6805194	8.26742	8.337856	8.329	8.334244	8.580053	8.323596	8.284357	8.343642	8.225088	8.26292	8.347162	8.160082	Trim27	NM_009054
6805206	3653	6.32758	6.6386	6.661371	6.331989	-0.00441	0.971014	0.30661	0.031292	0.329382	0.023188	-0.02277	0.851318	-0.31102	0.029522	-0.33379	0.021887	6805206	6.331085	6.544616	6.107038	6.538579	6.63882	6.7384	6.669274	6.717899	6.596941	6.166648	6.445421	6.383899	Zscan12	NM_016684
6805355	3669	9.580324	9.770021	9.44834	9.513256	0.067068	0.559019	0.256765	0.047867	-0.06492	0.571432	0.321681	0.019174	-0.1897	0.122941	0.131984	0.264578	6805355	9.574288	9.623135	9.543549	9.636537	9.773313	9.900214	9.500003	9.616388	9.22863	9.598934	9.378103	9.562731	Hist1h4h	NM_153173
6805366	3673	11.87211	11.86574	11.90772	11.8043	0.06781	0.464168	0.061437	0.505884	0.103417	0.274797	-0.04198	0.646886	0.006373	0.944179	-0.03561	0.697058	6805366	11.81425	11.75371	12.04837	11.84277	11.87069	11.88375	11.96213	12.01192	11.7491	11.8015	11.75528	11.85612	Hist1h4d	NM_175654
6805370	3674	8.594903	8.759658	8.980804	9.091077	-0.49617	0.091502	-0.33142	0.236202	-0.11027	0.681264	-0.22115	0.417668	-0.16476	0.54215	-0.3859	0.17426	6805370	8.312237	9.211634	8.260837	8.754463	8.685938	8.838573	9.043824	9.187384	8.711203	9.198182	8.833067	9.241982	Hist1h2b	NM_023422
6805380	3676	5.741349	6.294191	5.12447	4.508607	1.232741	0.041667	1.785584	0.007969	0.615863	0.260668	1.169721	0.050553	-0.55284	0.308887	0.616879	0.259944	6805380	5.803214	5.509345	5.911487	5.743338	6.657253	6.481983	5.200403	4.003603	6.169404	4.741386	4.631181	4.153255	Hist1h2b	NM_175664
6805381	3677	7.955579	8.48569	8.359057	8.259646	-0.30407	0.153331	0.226044	0.274643	0.099411	0.61998	0.126633	0.52964	-0.53011	0.02505	-0.40348	0.069665	6805381	8.020182	8.004732	7.841824	8.888215	8.467216	8.101638	8.538774	8.444551	8.093845	8.259411	8.202751	8.316776	Hist1h1c	NM_015786
6805382	3678	7.768344	8.082619	6.918837	6.066456	1.701888	0.083499	2.016163	0.047305	0.85238	0.351205	1.163783	0.213485	-0.31428	0.724582	0.849507	0.352742	6805382	8.079631	7.89219	7.333211	7.509635	8.78721	7.951013	8.258352	4.758133	7.740025	6.241323	6.512998	5.445048	Hist1h2a	NM_175660

6805404	3683	6.117573	6.579398	6.416711	6.763633	-0.64606	0.000266	-0.18424	0.116162	-0.34692	0.010584	0.162687	0.15842	-0.46182	0.002239	-0.29914	0.021142	6805404	6.352508	5.910866	6.089345	6.626377	6.556952	6.554864	6.319531	6.470149	6.460452	6.743783	6.85846	6.688656	Hist1h2a	NM_175658
6805430	3684	7.012529	6.484055	6.209463	5.587202	1.425328	0.005221	0.896854	0.043691	0.622261	0.135511	0.274593	0.484772	0.528474	0.196287	0.803067	0.064552	6805430	7.388756	6.507674	7.141158	6.625323	6.720757	6.106086	6.467105	5.716163	6.44512	5.052402	6.215346	5.493857	Cmah	NM_001111110
6805450	3685	11.22311	11.17871	10.93557	11.01281	0.210307	0.058578	0.165903	0.120212	-0.07724	0.441546	0.243143	0.034241	0.044403	0.654012	0.287547	0.016706	6805450	11.32629	11.28864	11.05441	11.00205	11.24159	11.29249	11.0229	10.84977	10.93403	11.00308	11.05349	10.98185	BC005537	NM_024473
6805452	3686	7.774098	7.97639	7.789907	7.851192	-0.07709	0.436358	0.125198	0.22004	-0.06129	0.533131	0.186483	0.082826	-0.20229	0.063802	-0.01581	0.870753	6805452	7.722267	7.7856	7.814427	7.763805	8.080042	8.085323	7.853034	7.875437	7.64125	7.837735	7.844855	7.870986	Ttrap	NM_019551
6805468	3688	6.488693	7.333681	6.8605	8.1673	-1.67861	0.00792	-0.83362	0.119155	-1.3068	0.025634	0.473181	0.350966	-0.84499	0.114916	-0.37181	0.45882	6805468	6.815599	6.63175	6.01873	7.644206	7.086717	7.27012	6.572825	7.236813	6.771861	7.876879	7.34542	9.2796	Dcdc2a	NM_177577
6805794	3698	6.736446	6.640804	6.588847	6.113802	0.622644	0.092798	0.527003	0.144967	0.475045	0.183539	0.051958	0.877436	0.095642	0.776905	0.147599	0.663046	6805794	6.707984	6.714869	6.786485	6.192159	7.216772	6.513482	6.784754	6.14836	6.833426	6.301697	6.455484	5.584224	Mboat1	NM_153546
6805797	3699	6.274774	5.765104	5.998887	5.796684	0.47809	0.065591	-0.03158	0.891489	0.202203	0.393554	-0.23378	0.327646	0.50967	0.05266	0.275887	0.253539	6805797	6.660792	6.210298	5.953231	6.01919	5.786672	5.48945	6.009264	5.866782	6.120615	5.560193	5.703262	6.126596	Agtr1a	NM_177322
6805878	3701	8.629071	9.026924	8.733508	8.571442	0.057629	0.814332	0.455482	0.091346	0.162066	0.51415	0.293416	0.251593	-0.39785	0.132336	-0.10444	0.67168	6805878	8.538585	8.704077	8.644552	8.88148	9.370264	8.829028	8.991038	8.261766	8.947721	8.420191	8.887042	8.407094	Foxq1	NM_008239
6805887	3702	7.20446	7.667889	8.041501	8.754713	-1.55025	0.000407	-1.08682	0.003619	-0.71321	0.028531	-0.37361	0.200045	-0.46343	0.121435	-0.83704	0.014035	6805887	6.828524	7.674563	7.110293	7.769612	7.669333	7.564722	7.743372	8.510059	7.871071	8.592073	8.624655	9.047412	Foxf2	NM_010225
6805902	3703	7.13291	7.167569	7.136256	7.098076	0.034834	0.735791	0.069493	0.505492	0.03818	0.711704	0.031313	0.761474	-0.03466	0.737057	-0.00335	0.974047	6805902	7.077866	7.16683	7.154033	7.241199	7.32323	6.938277	7.205898	7.149905	7.052964	7.057531	7.213115	7.023582	Foxc1	NM_008592
6806021	3704	7.395272	7.324617	7.460519	7.273643	0.121629	0.246198	0.050974	0.614226	0.186876	0.090772	-0.1359	0.199639	0.070655	0.488039	-0.06525	0.520991	6806021	7.273066	7.55112	7.36163	7.21921	7.425847	7.328794	7.411143	7.423486	7.546927	7.390349	7.315176	7.115404	Wrnip1	NM_030215
6806034	3705	8.683221	8.209042	8.207451	7.85531	0.827911	0.082776	0.353732	0.421672	0.352141	0.423678	0.001591	0.997053	0.474179	0.289138	0.47577	0.287637	6806034	8.717498	8.78265	8.549514	8.180715	8.410828	8.035583	9.003042	7.323976	8.295334	7.542489	7.550735	8.472706	Serpinb6	NM_011454
6806036	3706	8.49394	7.918844	8.243358	7.554784	0.939155	0.021346	0.364059	0.30073	0.688573	0.069743	-0.32451	0.352929	0.575096	0.118651	0.250582	0.468208	6806036	8.374144	8.666174	8.441501	7.905735	8.085617	7.765179	8.693436	7.431536	8.605101	7.329003	7.924651	7.410699	Serpinb9	NM_009256
6806073	3713	7.689069	7.639159	7.680041	7.244185	0.444884	0.084646	0.394974	0.118785	0.435856	0.090032	-0.04088	0.861006	0.04991	0.830818	0.009028	0.969129	6806073	7.722801	7.68004	7.664366	7.52673	8.049738	7.341009	7.444736	7.691176	7.904212	6.870304	7.545284	7.316967	Nqo2	NM_020282
6806078	3714	8.057105	8.055483	7.964361	7.743694	0.313412	0.021582	0.31179	0.022076	0.220668	0.079963	0.091122	0.431829	0.001622	0.988606	0.092744	0.424006	6806078	8.15876	8.002675	8.009881	8.00537	8.223781	7.937299	8.118385	7.869472	7.905227	7.563459	7.842583	7.825039	Ripk1	NM_009068
6806080	3715	7.635565	7.855192	7.731694	7.991953	-0.35639	0.038877	-0.13676	0.371464	-0.26026	0.109259	0.123498	0.417435	-0.21963	0.166884	-0.09613	0.524453	6806080	7.400205	7.796337	7.710154	7.795584	7.961533	7.80846	7.823482	7.665848	7.705753	7.804684	7.884975	8.2862	Bphl	NM_026512
6806162	3717	8.563955	8.667706	8.67285	8.597725	-0.03377	0.722757	0.069981	0.468141	0.075125	0.437253	-0.00514	0.956728	-0.10375	0.291552	-0.10889	0.269972	6806162	8.561766	8.570101	8.559999	8.469483	8.783667	8.749968	8.705482	8.773026	8.540042	8.574441	8.687455	8.53128	Prpf4b	NM_013830
6806219	3720	7.794974	7.80879	7.679315	7.672541	0.122434	0.146627	0.136249	0.111442	0.006775	0.931312	0.129474	0.127579	-0.01382	0.86058	0.115659	0.167374	6806219	7.777471	7.752372	7.85508	7.794592	7.840234	7.791543	7.828167	7.58591	7.623869	7.582352	7.627526	7.807744	Fars2	NM_024274
6806245	3721	9.413327	9.491192	9.869574	8.285632	1.127695	0.036663	1.20556	0.02802	1.583942	0.007865	-0.37838	0.425048	-0.07786	0.866983	-0.45625	0.340521	6806245	9.523435	9.458442	9.258105	9.310909	10.16424	8.998426	10.64293	9.207985	9.757808	8.268559	8.849551	7.738786	Ly86	NM_010745
6806271	3722	8.860979	8.750972	8.76898	8.722207	0.138772	0.223727	0.028765	0.791491	0.046773	0.668453	-0.01801	0.868365	0.110006	0.326372	0.091999	0.407427	6806271	8.874598	8.857509	8.850829	8.77887	8.826522	8.647525	8.583372	8.961061	8.762507	8.56966	8.731089	8.865872	Rreb1	NM_001013392
6806294	3723	7.440872	7.479095	7.426068	7.291258	0.149614	0.336533	0.187836	0.235233	0.13481	0.38389	0.053027	0.72648	-0.03822	0.800548	0.014804	0.921913	6806294	7.412333	7.486125	7.424158	7.343073	7.698464	7.395747	7.469838	7.260588	7.547778	7.021434	7.542277	7.310064	Riok1	NM_024242
6806300	3724	7.619795	7.719186	7.701381	7.553106	0.066689	0.39545	0.16608	0.05576	0.148275	0.080965	0.017805	0.816567	-0.09939	0.217607	-0.08159	0.303943	6806300	7.467698	7.682257	7.70943	7.662089	7.769449	7.726021	7.67447	7.633487	7.796187	7.554688	7.62636	7.478271	Snrnp48	NM_026382
6806435	3725	7.271148	7.494184	6.93126	6.856311	0.414836	0.167824	0.637873	0.048004	0.074949	0.791013	0.562924	0.073579	-0.22304	0.438425	0.339887	0.249185	6806435	7.678045	7.262808	6.87259	7.567047	7.717878	7.197628	7.064948	6.730249	6.998584	6.72748	7.33441	6.507044	Gcnt2	NM_023887
6806441	3726	7.623043	7.736622	7.676985	7.648428	-0.02538	0.872196	0.088194	0.579755	0.028556	0.856426	0.059637	0.706551	-0.11358	0.478621	-0.05394	0.733239	6806441	7.752615	7.491801	7.624714	7.582255	7.990578	7.637033	7.954439	7.46052	7.615995	7.535382	7.679594	7.730309	Pak1ip1	NM_026550
6806444	3727	8.510851	8.66346	8.401885	8.450818	0.060033	0.706582	0.212641	0.204312	-0.04893	0.758597	0.261574	0.127532	-0.15261	0.350304	0.108966	0.498916	6806444	8.502839	8.403213	8.626502	8.482458	8.73526	8.772661	8.681604	8.434175	8.089877	8.323731	8.449799	8.578925	Tmem14c	NM_025387
6806544	3729	9.265751	9.155005	9.2401	9.128391	0.13736	0.240378	0.026614	0.812083	0.111709	0.332525	-0.0851	0.454697	0.110746	0.336466	0.025651	0.818741	6806544	9.344638	9.249027	9.203588	9.272464	9.120735	9.071816	9.167497	9.403573	9.149231	9.088243	9.329906	8.967024	Hivep1	NM_007772
6806566	3730	6.641013	6.749755	6.694518	6.117112	0.523901	0.054324	0.632642	0.026229	0.577406	0.037937	0.055236	0.818219	-0.10874	0.652538	-0.05351	0.823802	6806566	6.415473	6.52727	6.980296	6.727838	6.744308	6.777118	7.090416	6.48337	6.509769	5.929523	6.511351	5.910463	Edn1	NM_010104
6806581	3731	6.433539	6.430514	6.334584	6.235399	0.19814	0.055777	0.195115	0.058828	0.099184	0.295504	0.09593	0.310557	0.003025	0.973605	0.098955	0.296544	6806581	6.477373	6.520667	6.302577	6.479323	6.553513	6.258706	6.329598	6.391425	6.282728	6.324503	6.227493	6.154202	Phactr1	NM_198419
6806610	3732	7.231975	7.299626	7.344819	7.321811	-0.08984	0.328937	-0.02219	0.803871	0.023007	0.796791	-0.04519	0.615173	-0.06765	0.456265	-0.11284	0.227915	6806610	7.303528	7.249214	7.143183	7.446132	7.322663	7.130083	7.248042	7.441584	7.34483	7.384149	7.268104	7.313181	Sirt5	NM_178848
6806618	3733	6.917009	7.04698	6.946366	7.062879	-0.14587	0.320271	-0.0159	0.910903	-0.11651	0.421953	0.100614	0.485719	-0.12997	0.372754	-0.02936	0.83647	6806618	6.735438	7.066336	6.949254	7.032234	7.160603	6.948103	7.220415	6.88884	6.729843	7.069388	6.952725	7.166524	Nol7	NM_023554
6806640	3735	7.083627	7.104427	7.159367	6.187628	0.896	0.001437	0.916799	0.001248	0.97174	0.000868	-0.05494	0.778064	-0.0208	0.914836	-0.07574	0.698284	6806640	7.331437	6.96167	6.957775	6.963274	7.404427	6.94558	7.23298	6.911547	7.333575	6.010762	6.441604	6.110517	Cd83	NM_009856
6806673	3736	7.564016	7.556494	7.339283	7.398584	0.165432	0.107541	0.157911	0.121931	-0.0593	0.534168	0.217212	0.044594	0.007521	0.936362	0.224733	0.039215	6806673	7.713012	7.457761	7.521274	7.537083	7.696633	7.435767	7.255538	7.355849	7.406461	7.296598	7.486323	7.41283	Jarid2	NM_021878
6806701	3737	9.725924	9.776474	9.81257	9.806972	-0.08105	0.103311	-0.0305	0.508668	0.005598	0.902101	-0.0361	0.436655	-0.05055	0.284694	-0.08665	0.084961	6806701	9.675074	9.745809	9.756889	9.794599	9.733763	9.80106	9.75686	9.858212	9.822637	9.811166	9.729664	9.880086	Mylip	NM_153789
6806707	3738	8.784703	9.021122	8.503981	8.43692	0.347782	0.062552	0.584202	0.006661	0.067061	0.687666	0.517141	0.012323	-0.23642	0.17976	0.280722	0.119051	6806707	8.738925	8.631988	8.983195	9.190205	8.898986	8.974176	8.497666	8.211451	8.802826	8.317965	8.539515	8.453281	Gmpr	NM_025508
6806791	3740	6.648115	6.677992	6.524567	6.387961	0.260154	0.734666	0.290031	0.705799	0.136606	0.858365	0.153425	0.841189	-0.02988	0.968836	0.123548	0.871756	6806791	5.401061	6.347463	6.195822	7.485873	6.72218	5.825923	7.176528	6.293798	6.103375	5.824893	6.810949	6.528042	Cap2	NM_026056
6806824	3741	8.12091	7.992141	8.072681	7.560154	0.560756	0.000104	0.431987	0.000605	0.512527	0.000194	-0.08054	0.338929	0.128769	0.142611	0.048229	0.55943	6806824	8.149341	8.028243	8.185146	7.940046	8.013737	8.022639	8.09681	8.040964	8.08027	7.426502	7.747793	7.506167	Aof1	NM_172262
6806831	3742	7.299565	7.243297	7.215915	7.23573	0.063834	0.705682	0.007567	0.964126	-0.01981	0.906282	0.027382	0.870817	0.056267	0.738953	0.083649	0.621836	6806831	7.135466	7.21369	7.549538	7.298487	7.234904	7.196501	7.36308	6.894952	7.389714	7.053483	7.248419	7.405289	Rnf144b	NM_146042
6806882	3743	10.3008	10.41973	10.36381	10.43171	-0.13091	0.404174	-0.01199	0.93771	-0.06791	0.659941	0.05592	0.716555	-0.11892	0.446793	-0.063	0.682846	6806882	10.26663	10.47639	10.15939	10.34016	10.24634	10.67268	10.23989	10.58432	10.26721	10.28201	10.45249	10.56064	Id4	NM_031166
6806933	3746	8.445741	8.40419	8.353795	8.085247	0.360494	0.083208	0.318943	0.118109	0.268548	0.178691	0.050395	0.789085	0.041551	0.825311	0.091946	0.627381	6806933	8.479122	8.30178	8.556321	8.451854	8.571305	8.189412	8.533677	8.325634	8.202075	7.749481	8.43006	8.0762	Ninj1	NM_013610
6806938	3747	7.847166	7.827263	7.841997	7.689099	0.158067	0.026481	0.138164	0.045067	0.152898	0.030387	-0.01473	0.806644	0.019903	0.74133	0.005169	0.931453	6806938	7.790926	7.827968	7.922603	7.799001	7.866731	7.816057	7.879164	7.825184	7.821642	7.58087	7.811559	7.674868	Bicd2	NM_001039179
6806948	3748	6.060493	6.114616	6.050736	6.097548	-0.03705	0.539188	0.017068	0.775172	-0.04681	0.441191	0.06388	0.300966	-0.05412	0.376241	0.009757	0.870066	6806948	6.124891	5.984153	6.072436	6.032782	6.211704	6.099361	6.076209	6.110899	5.9651	6.122672	6.107628	6.062343	Ippk	NM_199056
6806956	3749	6.822476	6.66116	6.385002	6.382157	0.440319	0.135136	0.279003	0.323123	0.002845	0.991697	0.276158	0.327779	0.161316	0.55954	0.437474	0.137339	6806956	6.756821	7.134105	6.576502	6.594726	6.910188	6.478567	6.596431	6.176218	6.382357	5.863138	6.851965	6.431369	Ecm2	NM_001012324
6806958	3750	8.843885	9.030769	8.34721	8.169548	0.674337	0.203974	0.861221	0.11529	0.177662	0.724986	0.683559	0.198465	-0.18688	0.711453	0.496675	0.33812	6806958	8.559562	9.686539	8.285554	8.917061	9.423787	8.75146	9.02661	7.920539	8.094481	7.449193	8.624931	8.43452	Aspn	NM_025711
6806960	3752	7.881523	7.636328	7.230042	6.326337	1.555186	0.018761	1.309991	0.038412	0.903705	0.126136	0.406285	0.464777	0.245195	0.655528	0.651481	0.253338	6806960	7.969134	8.022791	7.652644	7.694628	8.258087	6.956268	7.941882	6.741612	7.006633	5.297806	6.686734	6.994471	Ogn	NM_008760
6806963	3753	9.36432	9.333293	9.509465	9.36296	0.001361	0.991122	-0.02967	0.808677	0.146505	0.251527	-0.17617	0.17552	0.031028	0.800119	-0.14514	0.255598	6806963	9.330659	9.307776	9.454526	9.190068	9.560533	9.249277	9.472854	9.531798	9.523742	9.249784	9.586387	9.252708	Iars	NM_172015
6806991	3754	6.779607	6.754269	6.814506	6.540888	0.23872	0.034143	0.213381	0.052082	0.273618	0.019189	-0.06024	0.537843	0.025338	0.793455	-0.0349	0.718928	6806991	6.777343	6.811844	6.749635	6.75773	6.939473	6.565604	6.816357	6.781474	6.845687	6.460371	6.684772	6.47752	Fbxw17	NM_175401
6806997	3755	9.039638	9.254187	9.154608	9.016674	0.022963	0.830439	0.237513	0.05139	0.137933	0.220504	0.099579	0.365429	-0.21455	0.072546	-0.11497	0.300166	6806997	9.098749	9.017096	9.003068	9.122333	9.451482	9.188746	9.295938	9.161554	9.006331	8.946644	9.135254	8.968125	Spin1	NM_146043
6807007	3757	6.973855	7.136674	6.92638	7.095267	-0.12141	0.70763	0.041407	0.897808	-0.16889	0.603433	0.210294	0.519743	-0.16282	0.616289	0.047475	0.882953	6807007	7.334524	6.899226	6.687814	6.876039	7.833795	6.700187	6.987211	6.593136	7.198792	7.008581	7.073145	7.204074	S1pr3	NM_010101
6807041	3758	7.613823	7.415137	7.329814	7.133531	0.480292	0.264185	0.281606	0.501384	0.196283	0.636799	0.085323	0.836417	0.198686	0.632739	0.284009	0.497847	6807041	7.199988	7.396336	8.245145	7.442471	7.396815	7.406125	7.769359	6.685426	7.534656	7.499524	7.425014	6.476054	Gadd45g	NM_011817
6807074	3759	6.766707	6.631226	6.60253	5.983821	0.782886	0.00911	0.647405	0.02226	0.618709	0.027012	0.028696	0.903381	0.135481	0.570483	0.164177	0.49386	6807074	7.231217	6.503515	6.56539	6.637405	6.870389	6.385885	6.734289	6.455134	6.618168	6.013268	6.2391	5.699095	Syk	NM_011518
6807154	3760	9.116545	9.257398	9.279707	8.959916	0.156629	0.086946	0.297482	0.006006	0.319791	0.00405	-0.02231	0.788216	-0.14085	0.117534	-0.16316	0.076656	6807154	9.145132	9.104221	9.100282	9.140061	9.37212	9.260012	9.340132	9.294619	9.204369	8.798952	9.060525	9.020271	Sfxn1	NM_027324
6807158	3762	7.655669	7.396895	7.83318	7.404061	0.251608	0.220417	-0.00717	0.970727	0.429119	0.053133	-0.43629	0.050084	0.258775	0.208763	-0.17751	0.37579	6807158	7.512976	7.824063	7.629969	7.637062	7.147708	7.405914	7.634058	8.215733	7.64975	7.484541	7.238626	7.489016	Cplx2	NM_009946
6807169	3763	7.409474	7.442124	7.507447	7.472873	-0.0634	0.561147	-0.03075	0.776211	0.034574	0.749424	-0.06532	0.54959	-0.03265	0.762857	-0.09797	0.376237	6807169	7.329523	7.492866	7.406033	7.601566	7.291725	7.433081	7.42466	7.683889	7.413793	7.349105	7.537267	7.532247	4732471D	NM_176987
6807172	3764	7.884341	7.968802	7.884857	8.122955	-0.23861	0.140454	-0.15415	0.321389	-0.2381	0.141203	0.083945	0.580721	-0.08446	0.578443	-0.00052	0.997262	6807172	7.878655	7.824669	7.949699	8.122521	7.9517	7.832185	7.77993	8.189311	7.685331	8.034513	8.009785	8.324568	Arl10	NM_019968
6807175	3765	10.47511	10.36262	10.48097	10.68055	-0.20544	0.140098	-0.31793	0.035002	-0.19958	0.15025	-0.11835	0.373033	0.112493	0.395996	-0.00586	0.963904	6807175	10.41684	10.41145	10.59704	10.39845	10.55321	10.13619	10.36369	10.56345	10.51576	10.66235	10.52329	10.856	Higd2a	NM_025933
6807178	3766	8.720988	8.828157	8.736215	8.706394	0.014594	0.837107	0.121763	0.114315	0.029821	0.675746	0.091942	0.217645	-0.10717	0.157445	-0.01523	0.830168	6807178	8.728536	8.727343	8.707086	8.729379	8.992235	8.762857	8.753081	8.783642	8.671923	8.675629	8.782326	8.661228	Faf2	NM_178397
6807192	3767	8.142435	7.915883	7.923589	7.604511	0.537924	0.004248	0.311373	0.051649	0.319078	0.047285	-0.00771	0.956288	0.226551	0.134945	0.218846	0.146909	6807192	8.234988	8.043065	8.149251	7.952909	7.976763	7.817978	8.161562	7.732816	7.876389	7.408355	7.837989	7.567188	Tspan17	NM_028841
6807201	3768	6.268078	6.191299	6.425986	6.30485	-0.03677	0.677857	-0.11355	0.21991	0.121136	0.193443	-0.23469	0.025033	0.076779	0.394459	-0.15791	0.101357	6807201	6.403305	6.26252	6.138409	6.102207	6.220626	6.251065	6.485041	6.521578	6.271338	6.267696	6.298512	6.348341	Unc5a	NM_153131
6807209	3769	7.329836	7.409073	7.337386	7.400463	-0.07063	0.373201	0.008611	0.911287	-0.06308	0.424055	0.071688	0.366426	-0.07924	0.320884	-0.00755	0.922177	6807209	7.29115	7.379913	7.318446	7.486225	7.370841	7.370154	7.275908	7.513141	7.223108	7.335685	7.422815	7.442888	Zfp346	NM_012017
6807214	3770	6.021056	5.878957	5.980097	5.981441	0.039615	0.371622	-0.10248	0.04003	-0.00134	0.975171	-0.10114	0.042086	0.142099	0.00943	0.040959	0.356455	6807214	6.039843	5.993944	6.02938	5.886106	5.853731	5.897033	5.971684	6.036067	5.93254	5.998608	5.892408	6.053307	Fgfr4	NM_008011
6807223	3771	6.855824	6.573812	6.873672	6.667225	0.188599	0.255728	-0.09341	0.561114	0.206446	0.217067	-0.29986	0.087493	0.282012	0.104566	-0.01785	0.910634	6807223	7.134061	6.766526	6.666886	6.413273	6.775393	6.53277	6.945053	7.040525	6.635437	6.702876	6.60431	6.69449	Prelid1	NM_025596

6807228	3772	6.12956	6.133491	6.057252	6.075943	0.053617	0.524575	0.057548	0.495491	-0.01869	0.822421	0.076239	0.371849	-0.00393	0.962299	0.072308	0.395801	6807228	6.184948	6.121664	6.082069	6.132285	6.117943	6.150244	6.091954	6.058524	6.021278	6.059839	5.898062	6.269928	Rgs14	NM_016758
6807229	3773	7.136358	7.143107	7.125092	6.942989	0.193369	0.122841	0.200118	0.112086	0.182103	0.142956	0.018015	0.876322	-0.00675	0.95347	0.011265	0.922431	6807229	7.219716	7.051834	7.137523	7.216273	7.313557	6.899491	7.20935	7.164731	7.001196	6.906452	7.05331	6.869205	Grk6	NM_001038018
6807230	3774	6.223925	6.091417	6.201053	6.093153	0.130771	0.200835	-0.00174	0.985689	0.1079	0.283304	-0.10964	0.276189	0.132507	0.195518	0.022872	0.813524	6807230	6.202251	6.163878	6.305645	6.190671	6.180727	5.902854	6.099925	6.28702	6.216214	6.178576	6.130078	5.970806	Slc34a1	NM_011392
6807251	3775	9.297125	9.338799	9.310282	9.360246	-0.06312	0.660907	-0.02145	0.880853	-0.04996	0.727808	0.028517	0.842125	-0.04167	0.771323	-0.01316	0.926704	6807251	9.354836	9.282063	9.254475	9.116406	9.360335	9.539655	9.562331	9.237298	9.131217	9.29533	9.510242	9.275167	Tmed9	NM_026211
6807252	3776	6.898781	6.990003	6.804087	6.917534	-0.01875	0.823153	0.072469	0.39818	-0.11345	0.199905	0.185916	0.051294	-0.09122	0.293832	0.094694	0.277122	6807252	6.886136	6.92969	6.880517	6.981048	7.041256	6.947705	6.979815	6.790562	6.641884	6.946903	6.988018	6.81768	B4galt7	NM_146045
6807258	3777	7.562823	7.653277	7.515067	7.481301	0.081522	0.422536	0.171975	0.11242	0.033765	0.735311	0.13821	0.189757	-0.09045	0.37577	0.047756	0.633807	6807258	7.429528	7.579726	7.679215	7.558667	7.749622	7.651541	7.498009	7.616731	7.43046	7.418568	7.650694	7.374642	Caml	NM_007596
6807259	3778	8.907236	9.041618	8.873664	8.738029	0.169207	0.213253	0.303589	0.041439	0.135635	0.309944	0.167954	0.216341	-0.13438	0.314148	0.033572	0.795248	6807259	8.988802	8.8537	8.879206	8.901487	9.102482	9.120884	9.088569	8.712426	8.819997	8.538443	8.756806	8.918837	Ddx46	NM_145975
6807263	3779	10.23036	10.28027	10.20816	10.0689	0.161458	0.113455	0.211371	0.048429	0.139261	0.163871	0.07211	0.450281	-0.04991	0.597748	0.022197	0.813142	6807263	10.23335	10.26912	10.18861	10.20035	10.42447	10.216	10.21455	10.36259	10.04735	9.971647	10.13417	10.10089	B230219D	BC051430
6807265	3780	8.625157	8.659383	8.561179	8.76627	-0.14111	0.310185	-0.10689	0.43561	-0.20509	0.153986	0.098203	0.472463	-0.03423	0.799362	0.063978	0.63647	6807265	8.690392	8.677668	8.507411	8.721296	8.698524	8.558328	8.773209	8.625398	8.284931	8.701702	8.665391	8.931716	Txndc15	NM_175150
6807266	3781	7.697222	7.76668	7.55028	7.841669	-0.14445	0.220851	-0.07499	0.510087	-0.29139	0.027982	0.216399	0.081857	-0.06946	0.540981	0.146942	0.213715	6807266	7.722104	7.615126	7.754436	7.834458	7.705051	7.76053	7.626186	7.70179	7.322865	7.919919	7.673481	7.931607	Pcbd2	NM_028281
6807336	3785	12.36023	12.72761	12.36562	12.46345	-0.10322	0.649192	0.26416	0.2611	-0.09783	0.666147	0.361993	0.136097	-0.36738	0.131128	-0.00539	0.980932	6807336	12.2892	12.42069	12.3708	12.4521	12.96593	12.7648	12.6841	11.99492	12.41783	12.742	12.13468	12.51367	Tgfbi	NM_009369
6807345	3786	8.577646	8.617982	8.537588	8.373793	0.203853	0.055142	0.244189	0.027617	0.163795	0.109139	0.080394	0.402129	-0.04034	0.668888	0.040057	0.671021	6807345	8.570523	8.638927	8.523487	8.521493	8.808969	8.523485	8.593147	8.531164	8.488454	8.248713	8.502016	8.37065	Smad5	NM_008541
6807437	3787	8.210424	8.211459	8.178936	7.920469	0.289955	0.024808	0.29099	0.024434	0.258467	0.039493	0.032523	0.765081	-0.00103	0.992395	0.031488	0.772302	6807437	8.101551	8.268538	8.261184	8.095167	8.370911	8.168298	7.992235	8.310817	8.233756	7.996571	7.953885	7.810951	5133401N	NM_198004
6807467	3788	7.159355	7.331223	7.421292	7.323987	-0.16463	0.125441	0.007236	0.941902	0.097305	0.34148	-0.09007	0.376617	-0.17187	0.111882	-0.26194	0.026155	6807467	7.313146	7.092454	7.072466	7.225899	7.420799	7.34697	7.567142	7.291548	7.405186	7.235062	7.314476	7.422423	Rmi1	NM_028904
6807518	3790	7.817325	7.961923	7.941818	7.919881	-0.10256	0.331432	0.042042	0.68286	0.021937	0.83052	0.020105	0.844445	-0.1446	0.183031	-0.12449	0.244891	6807518	7.679665	7.887662	7.884647	7.930858	8.165385	7.789526	7.939352	7.979276	7.906826	7.825162	7.994397	7.940085	Mak10	NM_030153
6807564	3792	10.38219	10.22735	10.33748	9.861743	0.520444	1.63E-05	0.365604	0.000202	0.475734	3.15E-05	-0.11013	0.088729	0.15484	0.026101	0.04471	0.454264	6807564	10.47216	10.34323	10.33117	10.18502	10.26651	10.23051	10.27559	10.34322	10.39362	9.75841	9.903391	9.923427	Dapk1	NM_029653
6807609	3793	8.468713	8.399993	8.489567	8.427893	0.04082	0.758491	-0.0279	0.833277	0.061673	0.643577	-0.08957	0.504828	0.068721	0.606755	-0.02085	0.874905	6807609	8.517125	8.58642	8.302595	8.524101	8.433526	8.242351	8.66528	8.390968	8.412452	8.218843	8.521683	8.543154	2010111I	NM_028079
6807650	3794	7.077429	7.097727	7.437521	7.066045	0.011384	0.938856	0.031682	0.831168	0.371476	0.032474	-0.33979	0.045777	-0.0203	0.891255	-0.36009	0.036732	6807650	7.21726	7.218399	6.796628	7.086522	7.020734	7.185926	7.487753	7.437464	7.387346	6.874722	6.994112	7.329301	0610007P	NM_023507
6807652	3795	7.002569	7.174454	7.345335	7.175205	-0.17264	0.191089	-0.00075	0.995195	0.17013	0.196938	-0.17088	0.195169	-0.17189	0.192826	-0.34277	0.021958	6807652	7.148995	6.948942	6.90977	7.288355	7.084117	7.150891	7.368053	7.273299	7.394654	6.91761	7.223232	7.384774	0610007P	NM_001013608
6807687	3796	8.577182	8.632983	8.675961	8.225409	0.351773	0.004515	0.407574	0.00194	0.450552	0.001052	-0.04298	0.646093	-0.0558	0.552894	-0.09878	0.304801	6807687	8.55827	8.652211	8.521065	8.699171	8.615277	8.584502	8.730432	8.59522	8.702231	8.108814	8.124567	8.442847	Habp4	NM_019986
6807722	3797	6.589371	6.471282	6.823708	6.463118	0.126253	0.327989	0.008164	0.947948	0.36059	0.017727	-0.35243	0.019651	0.118089	0.358407	-0.23434	0.089228	6807722	6.806042	6.483259	6.478811	6.561505	6.536317	6.316023	6.99171	6.853364	6.62605	6.476332	6.435099	6.477923	Ccrk	NM_053180
6807764	3800	7.224362	7.366252	7.100899	7.24543	-0.02107	0.757368	0.120822	0.104062	-0.14453	0.059605	0.265352	0.003804	-0.14189	0.063447	0.123463	0.097854	6807764	7.155754	7.342633	7.174699	7.286902	7.443727	7.368126	7.014723	7.187615	7.10036	7.24544	7.288398	7.202451	Zfp369	NM_178364
6807790	3801	8.291219	8.335263	8.324478	8.299979	-0.00876	0.900732	0.035284	0.618095	0.024499	0.728119	0.010785	0.877988	-0.04404	0.535553	-0.03326	0.638089	6807790	8.290184	8.256732	8.326741	8.274464	8.476952	8.254373	8.411502	8.291943	8.26999	8.220737	8.309881	8.36932	Ptdss1	NM_008959
6807812	3802	6.773602	6.756345	6.829952	7.274783	-0.50118	0.01651	-0.51844	0.0141	-0.44483	0.027831	-0.07361	0.668926	0.017257	0.919689	-0.05635	0.742774	6807812	6.377257	6.883005	7.060544	6.839404	6.635944	6.793688	6.728651	6.942148	6.819058	7.255983	7.411415	7.156951	Zfp759	NM_172392
6807816	3803	6.271741	6.050459	7.065587	6.29354	-0.0218	0.952303	-0.24308	0.5108	0.772047	0.060324	-1.01513	0.020705	0.221282	0.548467	-0.79385	0.054787	6807816	6.054749	6.779943	5.98053	5.804572	6.701254	5.64555	6.672078	7.000662	7.524022	6.072722	6.495462	6.312437	Rsl1	NM_001013769
6807882	3805	6.221262	6.110297	6.475764	6.022654	0.198608	0.613764	0.087643	0.822579	0.453109	0.265227	-0.36547	0.36223	0.110965	0.776706	-0.2545	0.52	6807882	6.433913	6.094839	6.135035	6.10996	6.190871	6.030061	6.489265	6.279276	6.65875	5.064274	6.197518	6.806171	Zfp273	NM_198322
6807998	3808	8.009733	8.081916	8.213018	8.03465	-0.02492	0.842986	0.047265	0.708052	0.178368	0.181173	-0.1311	0.313073	-0.07218	0.569734	-0.20329	0.133614	6807998	8.144135	8.03679	7.848273	7.941781	8.148763	8.155203	8.345068	8.094458	8.199529	7.859925	8.23637	8.007656	Nsun2	NM_145354
6808026	3809	7.16125	7.300189	7.177855	7.01298	0.14827	0.313206	0.287209	0.070593	0.164875	0.265657	0.122334	0.400463	-0.13894	0.342737	-0.01661	0.907032	6808026	7.205634	7.138555	7.13956	7.23233	7.433219	7.235019	7.373654	7.09715	7.062762	6.717109	7.227427	7.094404	Med10	NM_138596
6808173	3810	7.102798	7.057515	6.924394	7.315376	-0.21258	0.030244	-0.25786	0.012832	-0.39098	0.001297	0.133121	0.138364	0.045283	0.590848	0.178404	0.058442	6808173	7.027421	7.20757	7.073404	7.003543	7.114766	7.054236	7.086644	6.866924	6.819614	7.378536	7.347701	7.219892	Irx2	NM_010574
6808209	3812	7.697209	7.71556	7.683848	7.717827	-0.02062	0.893956	-0.00227	0.988296	-0.03398	0.826287	0.031712	0.83768	-0.01835	0.90555	0.013362	0.931136	6808209	7.73362	7.725277	7.632731	7.700868	7.731527	7.714284	7.810874	7.960194	7.280475	7.655529	7.720303	7.77765	Mrpl36	NM_053163
6808211	3813	8.130919	8.111987	7.990938	7.630864	0.500055	0.029774	0.481122	0.034777	0.360073	0.093964	0.121049	0.540838	0.018933	0.922879	0.139982	0.481224	6808211	8.397305	8.158958	7.836495	8.090014	8.326445	7.919501	8.176394	7.897467	7.898952	7.477095	7.933523	7.481975	Lpcat1	NM_145376
6808214	3815	9.388213	9.395533	9.483359	9.316872	0.071341	0.396677	0.078661	0.352385	0.166488	0.070045	-0.08783	0.302345	-0.00732	0.929049	-0.09515	0.266574	6808214	9.447996	9.375455	9.341187	9.298107	9.382731	9.505761	9.632629	9.410419	9.40703	9.286535	9.414509	9.249571	Clptm1l	NM_146047
6808215	3816	6.547194	6.808964	6.46851	6.451015	0.09618	0.406326	0.35795	0.011494	0.017496	0.877279	0.340454	0.014611	-0.26177	0.044168	0.078684	0.493756	6808215	6.527817	6.594605	6.519161	6.913252	6.80007	6.713571	6.23731	6.555026	6.613195	6.392853	6.349532	6.610659	Tert	NM_009354
6808221	3817	8.80362	8.780457	8.540368	8.455098	0.348523	0.009723	0.325359	0.013597	0.085271	0.43297	0.240089	0.048576	0.023163	0.828173	0.263252	0.03424	6808221	8.839278	8.885986	8.685597	8.870567	8.767166	8.703638	8.682615	8.426972	8.511518	8.273071	8.482054	8.610168	Slc12a7	NM_011390
6808230	3818	7.547681	7.57047	7.578191	7.527077	0.020604	0.789709	0.043393	0.57737	0.051114	0.513219	-0.00772	0.920239	-0.02279	0.768138	-0.03051	0.693726	6808230	7.61596	7.491158	7.535926	7.521006	7.663582	7.526823	7.636572	7.418153	7.679848	7.513367	7.477662	7.590202	Brd9	NM_001024508
6808237	3820	6.839869	6.742722	6.679281	6.797695	0.042174	0.641547	-0.05497	0.545946	-0.11841	0.211476	0.063441	0.487581	0.097147	0.297533	0.160588	0.10275	6808237	6.883481	6.80773	6.828396	6.728157	6.677534	6.822475	6.860321	6.635144	6.542378	6.78647	6.91211	6.694504	Tppp	NM_182839
6808260	3821	8.0938	8.172147	8.118722	8.225156	-0.13136	0.303348	-0.05301	0.668883	-0.10643	0.398804	0.053424	0.666477	-0.07835	0.530197	-0.02492	0.8399	6808260	7.984512	8.148614	8.148273	8.040257	8.414887	8.061296	7.993624	8.270563	8.09198	8.123147	8.345383	8.206938	Ccdc127	NM_024201
6808271	3822	8.492416	8.374371	8.526972	8.322749	0.169667	0.361963	0.051622	0.77613	0.204223	0.278078	-0.1526	0.40988	0.118045	0.520129	-0.03456	0.848812	6808271	8.480713	8.631249	8.365286	8.189266	8.579865	8.353982	8.846098	8.166711	8.568107	8.28265	8.446167	8.239429	Erap1	NM_030711
6808308	3824	8.469245	8.42478	8.516595	8.761188	-0.29194	0.085162	-0.33641	0.053484	-0.24459	0.138536	-0.09182	0.554012	0.044465	0.772493	-0.04735	0.75826	6808308	8.545117	8.490597	8.37202	8.163387	8.705586	8.405367	8.336182	8.692838	8.520765	8.852995	8.828703	8.601867	Ell2	NM_138953
6808314	3825	10.31607	10.61997	10.57361	11.00771	-0.69164	0.00554	-0.38773	0.068064	-0.4341	0.045937	0.046367	0.807315	-0.30391	0.137052	-0.25754	0.198991	6808314	9.893778	10.3792	10.67522	10.64426	10.61226	10.6034	10.5071	10.58462	10.6291	10.96603	11.23281	10.82428	Glrx	NM_053108
6808339	3827	7.109262	6.686528	6.595934	5.558925	1.550338	0.01536	1.127604	0.056075	1.03701	0.074143	0.090594	0.86211	0.422734	0.426904	0.513328	0.339219	6808339	7.946748	6.895998	6.485041	6.835612	7.051423	6.17255	7.257329	5.815493	6.714981	5.035309	5.949609	5.691856	Mctp1	NM_030174
6808363	3828	6.182616	6.168339	6.127502	5.990541	0.192075	0.089172	0.177798	0.111203	0.136962	0.205206	0.040836	0.691739	0.014277	0.889248	0.055114	0.594114	6808363	6.269795	6.225084	6.052969	6.169758	6.243607	6.091651	6.288425	6.017815	6.076267	5.869089	5.956592	6.145941	2210408I	NM_001145676
6808537	3829	7.182126	7.500145	7.475905	7.46303	-0.2809	0.128848	0.037115	0.828573	0.012874	0.940046	0.024241	0.887437	-0.31802	0.09153	-0.29378	0.114524	6808537	7.106123	7.191916	7.248338	7.435286	7.606138	7.459012	7.615803	7.775292	7.036619	7.44219	7.470681	7.47622	Lysmd3	NM_030257
6808544	3830	7.042349	7.02121	7.084214	7.104476	-0.06213	0.586751	-0.08327	0.469667	-0.02026	0.85808	-0.063	0.581583	0.021139	0.852021	-0.04187	0.712709	6808544	7.0951	7.056403	6.975544	6.913841	7.055982	7.093808	7.320097	6.985649	6.946897	6.960475	7.219395	7.133558	Cetn3	NM_007684
6808609	3831	8.399981	8.393434	8.310307	8.045825	0.354156	0.327719	0.347609	0.336205	0.264483	0.458719	0.083126	0.812879	0.006547	0.985098	0.089674	0.798506	6808609	8.203109	8.288082	8.708752	8.66011	8.376634	8.143557	8.743233	7.981735	8.205954	7.32725	8.514717	8.295507	Mef2c	NM_025282
6808617	3833	7.390488	7.273874	7.334731	7.728977	-0.33849	0.068361	-0.4551	0.022125	-0.39425	0.039799	-0.06086	0.714887	0.116614	0.488916	0.055757	0.737679	6808617	7.321844	7.42496	7.42466	7.381146	7.094215	7.346262	7.271483	7.587021	7.145688	7.416115	7.841443	7.929372	10004311	XR_034249
6808638	3835	7.251771	7.280299	7.405052	7.344389	-0.09262	0.190435	-0.06409	0.351206	0.060663	0.376188	-0.12475	0.090151	-0.02853	0.671145	-0.15328	0.045421	6808638	7.19339	7.360489	7.201435	7.22889	7.299167	7.31284	7.449509	7.403267	7.362381	7.218687	7.429358	7.385123	Tmem161b	NM_175187
6808680	3836	8.561305	8.668879	8.679024	8.599957	-0.03865	0.780974	0.068922	0.621941	0.079067	0.572565	-0.01015	0.941682	-0.10757	0.446598	-0.11772	0.406625	6808680	8.463156	8.611924	8.608835	8.534479	8.734724	8.737433	8.725593	8.808593	8.502886	8.342346	8.615439	8.842085	Ccnh	NM_023243
6808773	3837	5.53396	5.805486	5.493346	6.283753	-0.74979	0.240495	-0.47827	0.442051	-0.79041	0.21813	0.31214	0.611946	-0.27153	0.658349	0.040614	0.946932	6808773	4.865561	5.452422	6.283896	4.869267	6.901068	5.646122	5.753635	5.387784	5.338618	7.052934	5.688933	6.109392	Edil3	NM_001037987
6808810	3839	7.657786	7.79708	7.93579	7.759914	-0.10213	0.593418	0.037166	0.844703	0.175876	0.366356	-0.13871	0.471801	-0.13929	0.469999	-0.278	0.168613	6808810	7.777368	7.804847	7.391143	7.57855	8.127464	7.685227	8.042186	7.998049	7.767136	7.749991	7.970601	7.55915	Tmem167	NM_025335
6808876	3840	8.51041	8.60333	8.456729	8.581333	-0.07092	0.434688	0.021998	0.805102	-0.1246	0.186508	0.146601	0.127572	-0.09292	0.312694	0.053681	0.550968	6808876	8.491271	8.57275	8.46721	8.685542	8.569413	8.555036	8.521236	8.424806	8.424146	8.373013	8.657637	8.713348	Ssbp2	NM_024272
6808964	3844	10.95704	10.56818	10.73306	10.47936	0.47768	0.086033	0.08882	0.72535	0.2537	0.328979	-0.16488	0.51837	0.38886	0.149768	0.22398	0.385606	6808964	10.78881	11.0942	10.9881	10.15799	10.9055	10.64104	11.04375	10.25396	10.90146	10.35162	10.55892	10.52753	Serinc5	NM_172588
6808997	3846	6.519631	6.492487	6.492478	6.418352	0.101279	0.34376	0.074135	0.482427	0.074126	0.48248	9.33E-06	0.999928	0.027144	0.79423	0.027153	0.794162	6808997	6.469069	6.454655	6.635169	6.408528	6.711611	6.357323	6.592116	6.496289	6.389029	6.376538	6.488446	6.390072	Homer1	NM_147176
6809030	3848	9.036829	8.996028	8.995849	8.8151	0.221729	0.21962	0.180929	0.30881	0.180749	0.309259	0.000179	0.999167	0.0408	0.812576	0.04098	0.811771	6809030	9.021476	8.818216	9.270794	9.029216	9.101122	8.857747	9.176291	8.953243	8.858013	8.823168	8.5417	9.080431	Arsb	NM_009712
6809032	3849	7.340945	7.590927	7.587048	7.310637	0.030308	0.755093	0.280291	0.017449	0.276411	0.018586	0.00388	0.968049	-0.24998	0.02868	-0.2461	0.03058	6809032	7.487841	7.135117	7.399876	7.648317	7.616296	7.508169	7.508471	7.595091	7.657581	7.331686	7.211572	7.388652	Arsb	NM_009712
6809047	3850	9.090284	9.114417	9.217867	8.46316	0.627124	0.000264	0.651257	0.000204	0.754707	7.32E-05	-0.10345	0.337547	-0.02413	0.81789	-0.12758	0.243853	6809047	9.216584	9.016523	9.037745	9.023988	9.257759	9.061504	9.345815	9.058634	9.249151	8.335999	8.546505	8.506975	Lhfpl2	NM_172589
6809064	3851	8.846909	8.835516	8.923233	8.688301	0.158607	0.092843	0.147215	0.114549	0.234932	0.022291	-0.08772	0.322195	0.011392	0.89439	-0.07632	0.385422	6809064	8.812387	8.876089	8.85225	8.725306	8.950264	8.830979	8.969749	8.810749	8.989202	8.544324	8.811622	8.708958	Ap3b1	NM_009680
6809082	3852	7.094547	7.202049	7.189226	7.220783	-0.12624	0.061751	-0.01873	0.755601	-0.03156	0.602168	0.012822	0.831013	-0.1075	0.101701	-0.09468	0.14216	6809082	7.21174	7.047651	7.024251	7.15968	7.246644	7.199822	7.173845	7.218753	7.175081	7.128164	7.237569	7.296615	Otp	NM_011021
6809083	3853	7.936609	7.959766	8.083906	8.069625	-0.13302	0.179748	-0.10986	0.259323	0.014281	0.878506	-0.12414	0.207317	-0.02316	0.804465	-0.1473	0.142202	6809083	7.941416	7.887802	7.980609	7.925611	8.113187	7.840501	8.133858	8.15658	7.96128	7.946662	8.062241	8.199973	Wdr41	XR_172590
6809115	3854	8.580636	8.755609	8.693899	8.837129	-0.25649	0.055769	-0.08152	0.497483	-0.14323	0.247098	0.06171	0.605236	-0.17497	0.165676	-0.11326	0.352364	6809115	8.454395	8.649678	8.637834	8.850158	8.771944	8.644726	8.71642	8.720958	8.64432	8.679746	8.726653	9.104988	Zbed3	NM_028106
6809223	3856	6.6495	6.855459	6.902263	6.735399	-0.0859	0.376346	0.12006	0.226828	0.166864	0.106329	-0.0468	0.623579	-0.20596	0.054925	-0.25276	0.024821	6809223	6.537931	6.758587	6.651983	6.773758	7.016366	6.776253	6.878091	6.927043	6.901655	6.585283	6.773516	6.847397	1200014M	NM_026173

6809229	3857	8.894065	9.076794	9.115132	9.079767	-0.1857	0.130487	-0.00297	0.979142	0.035365	0.756521	-0.03834	0.736924	-0.18273	0.135906	-0.22107	0.07979	6809229	8.904876	8.864162	8.913156	9.068533	9.003317	9.158532	9.067816	9.297067	8.980512	8.958702	8.971794	9.308804	Col4a3bp	NM_023420
6809266	3858	7.407865	7.445621	7.418	7.539692	-0.13183	0.088388	-0.09407	0.203699	-0.12169	0.111143	0.027622	0.695086	-0.03776	0.593713	-0.01013	0.885149	6809266	7.313207	7.458385	7.452003	7.359418	7.529868	7.447578	7.368468	7.434628	7.450903	7.415052	7.614643	7.58938	Gfm2	NM_177266
6809272	3859	8.090405	8.200847	7.787034	7.85417	0.236235	0.071895	0.346677	0.016012	-0.06714	0.572036	0.413812	0.006673	-0.11044	0.360862	0.303371	0.028712	6809272	8.054371	8.189856	8.026989	8.151702	8.193943	8.256895	7.846189	7.835214	7.6797	7.662332	7.773339	8.126839	Enc1	NM_007930
6809372	3861	6.572059	6.628146	6.577605	6.499229	0.07283	0.439373	0.128917	0.187745	0.078376	0.406744	0.050541	0.587786	-0.05609	0.548366	-0.00555	0.952111	6809372	6.447281	6.698812	6.570083	6.553963	6.681472	6.649002	6.710015	6.517323	6.505477	6.361376	6.547632	6.588678	Ankra2	NM_023472
6809379	3862	6.526102	6.605786	6.423228	6.683516	-0.15741	0.205015	-0.07773	0.514926	-0.26029	0.051961	0.182558	0.14825	-0.07968	0.504715	0.102874	0.39357	6809379	6.59114	6.438949	6.548217	6.603423	6.580981	6.632953	6.691726	6.301288	6.276669	6.708066	6.542418	6.800063	Foxd1	NM_008242
6809436	3863	7.2687	7.229541	7.110727	7.222392	0.046308	0.794395	0.007149	0.967836	-0.11166	0.534055	0.118814	0.5089	0.039159	0.825471	0.157972	0.384857	6809436	7.636276	7.18803	6.981793	7.392259	7.12075	7.175614	6.906066	7.285914	7.140202	7.124575	7.244341	7.298259	Zfp366	NM_001004149
6809447	3864	6.631768	6.542893	6.808131	6.776267	-0.1445	0.200618	-0.23337	0.054356	0.031864	0.766282	-0.26524	0.033645	0.088875	0.415958	-0.17636	0.127123	6809447	6.610141	6.636046	6.649116	6.611821	6.665099	6.351759	6.785274	6.657065	6.982054	6.668531	6.850202	6.810068	Mrps27	NM_173757
6809522	3865	6.126128	6.284723	6.067147	5.918059	0.208069	0.285734	0.366664	0.078567	0.149088	0.436155	0.217575	0.265877	-0.15859	0.408662	0.058981	0.754058	6809522	6.066072	6.310559	6.001753	5.897837	6.706565	6.249766	6.018617	6.066242	6.116583	5.839231	5.979367	5.935579	Serf1	NM_011353
6809524	3866	7.527962	7.609025	7.660493	7.473875	0.054086	0.596077	0.135149	0.20518	0.186617	0.093342	-0.05147	0.613681	-0.08106	0.4321	-0.13253	0.213227	6809524	7.681807	7.452826	7.449252	7.482872	7.804553	7.539649	7.715942	7.695713	7.569823	7.510842	7.511002	7.399782	Smn1	NM_011420
6809550	3867	7.669215	7.747786	7.449641	7.555392	0.113823	0.423464	0.192394	0.191779	-0.10575	0.455816	0.298145	0.058138	-0.07857	0.576438	0.219573	0.142361	6809550	7.577583	7.675138	7.754923	7.543759	7.981034	7.718565	7.647274	7.285187	7.416463	7.430011	7.531264	7.7049	Taf9	NM_027592
6809551	3868	6.407343	6.375411	6.366333	6.579771	-0.17243	0.459972	-0.20436	0.384515	-0.21344	0.364796	0.009078	0.968404	0.031932	0.889261	0.04101	0.858133	6809551	6.292559	6.552569	6.3769	6.325508	6.532489	6.268236	6.195598	6.391546	6.511854	6.327959	6.274232	7.137123	Ccdc125	NM_183115
6809655	3870	8.015928	7.984043	8.615843	6.683283	1.332645	0.040448	1.30076	0.044309	1.93256	0.007608	-0.6318	0.280393	0.031885	0.954844	-0.59992	0.303639	6809655	8.357821	7.918686	7.771276	8.116129	8.356604	7.479395	9.304325	7.708954	8.83425	5.717232	7.50764	6.824976	Cd180	NM_008533
6809806	3871	7.203254	6.937705	7.481263	7.618323	-0.41507	0.084812	-0.68062	0.012157	-0.13706	0.534316	-0.54356	0.032863	0.265549	0.243846	-0.27801	0.224282	6809806	7.417519	7.169596	7.022648	6.943981	6.831496	7.037639	7.862916	7.388451	7.192421	7.82912	7.258637	7.767212	Sgtb	NM_144838
6809810	3872	7.613673	7.832932	7.735851	7.655488	-0.04181	0.696473	0.177444	0.124433	0.080364	0.459334	0.097081	0.375201	-0.21926	0.066741	-0.12218	0.271226	6809810	7.521719	7.668171	7.651128	7.750162	7.811414	7.93722	7.893754	7.631258	7.682542	7.518943	7.600145	7.847375	Trim23	NM_030731
6809851	3874	6.959382	7.143213	7.110436	7.211683	-0.2523	0.090169	-0.06847	0.615218	-0.10125	0.461636	0.032777	0.808657	-0.18383	0.197964	-0.15105	0.281975	6809851	6.88337	6.857719	7.137056	6.960374	7.238628	7.230636	7.322353	6.914926	7.094029	7.084257	7.266919	7.283873	Sfrs12ip	NM_026075
6810063	3878	10.18072	10.45972	10.50315	10.7394	-0.55868	0.068635	-0.27968	0.323236	-0.23625	0.399836	-0.04343	0.874195	-0.279	0.324342	-0.32243	0.259507	6810063	10.23389	10.17369	10.13458	10.59044	10.38458	10.40414	10.14436	10.98275	10.38235	10.34614	10.61131	11.26075	Elovl7	NM_029001
6810067	3880	8.840265	9.170766	8.797	9.496595	-0.65633	0.00014	-0.32583	0.009802	-0.69959	8.95E-05	0.373766	0.004778	-0.3305	0.009126	0.043265	0.6665	6810067	8.658195	9.00916	8.85344	9.038871	9.210165	9.263261	8.821877	8.828952	8.740171	9.494141	9.402896	9.592748	Pde4d	NM_011056
6810166	3882	7.767464	8.282909	8.005716	7.434521	0.332943	0.45601	0.848388	0.08102	0.571195	0.215869	0.277193	0.532594	-0.51545	0.259848	-0.23825	0.590468	6810166	8.011539	7.965049	7.325804	8.124038	8.573011	8.151679	8.049439	7.84414	8.123569	6.368774	7.93516	7.999629	Plk2	NM_152804
6810209	3884	8.468997	8.578144	8.552904	8.386962	0.082035	0.489971	0.191182	0.13025	0.165941	0.181469	0.025241	0.829414	-0.10915	0.363918	-0.08391	0.480434	6810209	8.462709	8.546122	8.398161	8.389905	8.698028	8.6465	8.712887	8.60007	8.345754	8.374741	8.48749	8.298656	Mier3	NM_172593
6810260	3886	10.30148	10.17115	10.14562	9.79386	0.507623	0.032094	0.377293	0.090371	0.35176	0.11039	0.025533	0.899549	0.13033	0.524711	0.155863	0.449353	6810260	10.55403	10.30114	10.04928	10.06875	10.43719	10.00752	10.2328	10.00124	10.20282	9.437263	10.0105	9.933817	Il6st	NM_010560
6810280	3887	8.110416	8.178558	7.995929	7.976722	0.133694	0.213562	0.201836	0.075667	0.019207	0.850906	0.182628	0.102114	-0.06814	0.510463	0.114487	0.28058	6810280	8.158842	8.139287	8.03312	8.069052	8.235296	8.231325	7.939791	7.97091	8.077087	7.762509	8.009273	8.158384	Slc38a9	NM_178746
6810299	3888	8.819305	9.223319	8.544204	9.081748	-0.26244	0.200538	0.141571	0.473316	-0.53754	0.021243	0.679115	0.006885	-0.40401	0.06403	0.275101	0.181819	6810299	8.982329	8.953544	8.522042	9.456689	9.12925	9.084018	8.892845	8.284161	8.455607	9.02367	9.051682	9.169893	Ppap2a	NM_008903
6810317	3889	6.837459	6.691907	6.824367	6.830253	0.007206	0.958583	-0.13835	0.33372	-0.00589	0.966164	-0.13246	0.353507	0.145553	0.310671	0.013092	0.924846	6810317	6.850778	6.947663	6.713937	6.690742	6.79997	6.585008	6.894526	6.912657	6.665919	6.738243	7.11692	6.635597	Dhx29	NM_172594
6810333	3890	9.400049	9.717118	10.19909	9.060979	0.33907	0.420218	0.656139	0.138763	1.138115	0.021416	-0.48198	0.261635	-0.31707	0.449819	-0.79904	0.080234	6810333	9.463676	9.284506	9.451966	9.800465	9.387357	9.963532	9.952152	10.35382	10.29131	8.062257	9.310347	9.810333	Esm1	NM_023612
6810371	3891	7.412842	7.584043	7.496464	7.402164	0.010678	0.950753	0.181879	0.309357	0.094301	0.589012	0.087579	0.615377	-0.1712	0.336824	-0.08362	0.63118	6810371	7.58788	7.482207	7.168438	7.533041	7.709032	7.510056	7.577936	7.43931	7.472147	7.053724	7.689184	7.463583	Arl15	NM_172595
6810421	3893	7.142191	7.3526	7.218056	7.091772	0.050419	0.774727	0.260828	0.164165	0.126284	0.479571	0.134544	0.452288	-0.21041	0.25169	-0.07587	0.667784	6810421	7.128546	7.128835	7.169192	7.291597	7.53716	7.229044	7.473352	7.126726	7.054091	6.747593	7.352736	7.174988	Mocs2	NM_013826
6810548	3894	10.2034	10.19969	10.0486	10.17771	0.025685	0.917265	0.02198	0.929158	-0.12911	0.604614	0.151095	0.545854	0.003705	0.98804	0.1548	0.53629	6810548	10.23869	10.5036	9.867895	10.05781	10.42146	10.1198	10.44634	9.590735	10.10871	10.07806	10.12069	10.33438	Emb	NM_010330
6810620	3897	8.589468	8.757769	8.613356	8.54275	0.046719	0.693344	0.215019	0.096621	0.070606	0.553778	0.144413	0.241833	-0.1683	0.178967	-0.02389	0.839618	6810620	8.521618	8.632173	8.614614	8.629063	8.949074	8.69517	8.743761	8.664952	8.431355	8.395531	8.676398	8.55632	Paip1	NM_145457
6810625	3898	7.850308	8.013699	7.873909	7.845826	0.004482	0.937696	0.167873	0.016528	0.028083	0.626899	0.13979	0.036042	-0.16339	0.018693	-0.0236	0.682211	6810625	7.800182	7.908691	7.84205	7.911972	8.069757	8.059367	7.905437	7.836934	7.879355	7.753852	7.877616	7.90601	4833420G	NM_001113550
6810659	3899	6.919146	6.842212	7.056822	6.774493	0.144653	0.258539	0.067719	0.584713	0.282329	0.044971	-0.21461	0.108799	0.076935	0.535817	-0.13768	0.280409	6810659	6.964847	7.075829	6.716763	6.751269	7.014933	6.760433	7.091957	7.121682	6.956827	6.608173	6.871955	6.843351	BC016423	NM_134063
6810682	3900	9.442845	9.418155	9.301687	9.74457	-0.30172	0.145124	-0.32641	0.118881	-0.44288	0.045279	0.116468	0.550545	0.02469	0.89817	0.141157	0.471759	6810682	9.524462	9.517923	9.286149	9.283608	9.434615	9.536242	9.705544	9.141575	9.057943	9.494797	9.804987	9.933925	Calml3	NM_027416
6810688	3901	9.063742	9.223279	8.993079	9.228778	-0.16504	0.213331	-0.0055	0.965175	-0.2357	0.089584	0.230201	0.096024	-0.15954	0.227519	0.070663	0.578579	6810688	9.04649	9.087078	9.057657	9.287974	9.126639	9.255225	9.08622	9.015136	8.87788	9.161188	9.003653	9.521492	Net1	NM_019671
6810717	3906	7.089117	4.432457	5.611747	6.315529	0.773588	0.230798	-1.88307	0.013454	-0.70378	0.271923	-1.17929	0.083417	2.65666	0.002128	1.47737	0.038297	6810717	6.710183	7.365529	7.191639	5.274677	4.15331	3.869383	6.333364	5.472288	5.029588	6.884263	6.917195	5.14513	Akr1e1	NM_018859
6810782	3907	8.614788	8.556377	8.763007	8.545341	0.069446	0.411637	0.011036	0.893925	0.217666	0.026463	-0.20663	0.032764	0.05841	0.487085	-0.14822	0.101695	6810782	8.658057	8.711649	8.474657	8.564433	8.496909	8.60779	8.823161	8.626329	8.839532	8.488216	8.632891	8.514917	Pfkp	NM_019703
6810874	3908	8.070227	8.100857	8.050937	8.04963	0.020597	0.816106	0.051227	0.566521	0.001307	0.988208	0.04992	0.576256	-0.03063	0.730015	0.01929	0.827546	6810874	8.13769	8.037883	8.035108	8.142281	8.186014	7.974277	8.080134	8.105024	7.967653	7.875319	8.134186	8.139386	Wdr37	NM_001039388
6810893	3909	8.510192	8.709515	8.651334	8.702412	-0.19222	0.084722	0.007102	0.943843	-0.05108	0.615333	0.058181	0.568032	-0.19932	0.075699	-0.14114	0.186629	6810893	8.594947	8.437303	8.498326	8.640439	8.782101	8.706004	8.739428	8.683033	8.53154	8.488626	8.819835	8.798776	Gtpbp4	NM_027000
6810954	3910	9.363703	9.414589	9.327604	9.263921	0.099782	0.139445	0.150669	0.038246	0.063683	0.325585	0.086986	0.190456	-0.05089	0.426978	0.036099	0.56915	6810954	9.372941	9.340752	9.377415	9.371614	9.496519	9.375635	9.356025	9.378169	9.248617	9.15031	9.368325	9.273127	Zmynd11	NM_144516
6811061	3913	7.592053	7.481241	7.512449	7.129021	0.463032	0.714438	0.35222	0.78036	0.383428	0.761574	-0.03121	0.980238	0.110812	0.929931	0.079604	0.949627	6811061	5.769067	7.076553	9.930539	9.062838	7.28594	6.094944	8.618711	7.178543	6.740094	6.053831	8.263158	7.070074	Actn2	NM_033268
6811068	3914	10.55073	10.21825	10.27887	9.748839	0.801891	0.020866	0.469416	0.131593	0.530028	0.094517	-0.06061	0.833756	0.332475	0.268362	0.271863	0.359232	6811068	10.46796	10.77672	10.40751	9.872774	10.50676	10.27523	10.61958	9.708341	10.50868	9.639454	10.069	9.538063	Lgals8	NM_018886
6811073	3915	6.766941	6.377246	6.466492	6.351691	0.415249	0.054145	0.025555	0.89306	0.114801	0.550368	-0.08925	0.640921	0.389695	0.067221	0.300449	0.141417	6811073	6.840762	6.806435	6.653625	6.053885	6.68349	6.394363	6.265048	6.701832	6.432596	6.337023	6.572332	6.145719	Edaradd	NM_133643
6811074	3916	8.952708	8.85133	8.661552	9.151525	-0.19882	0.324144	-0.3002	0.151334	-0.48997	0.032157	0.189777	0.345312	0.101378	0.606733	0.291155	0.162481	6811074	8.970569	9.08481	8.802744	8.764146	9.059632	8.730211	8.8176	8.607788	8.559269	9.257467	8.731608	9.465501	Gpr137b-	NR_003568
6811117	3917	7.998557	8.0187	8.012374	7.805037	0.19352	0.095719	0.213663	0.070617	0.207337	0.077719	0.006326	0.952305	-0.02014	0.849105	-0.01382	0.896097	6811117	8.074011	7.92486	7.9968	7.91646	8.068509	8.071131	8.187631	7.78883	8.060662	7.710592	7.890107	7.814413	Tbce	NM_178337
6811121	3918	7.637256	7.732951	7.883189	7.545304	0.091952	0.590059	0.187646	0.285246	0.337884	0.07313	-0.15024	0.38602	-0.09569	0.575314	-0.24593	0.171785	6811121	7.388372	7.852005	7.671391	7.560424	7.658409	7.980019	8.081007	7.838209	7.73035	7.36123	7.662589	7.612094	Ggps1	NM_010282
6811140	3920	7.033983	7.176756	7.057577	7.114789	-0.08081	0.533892	0.061966	0.631566	-0.05721	0.657603	0.119179	0.365784	-0.14277	0.283943	-0.02359	0.854195	6811140	6.92686	7.147732	7.027356	7.006907	7.225819	7.297541	7.283665	6.808586	7.08048	7.087608	7.114303	7.142457	Mrpl32	NM_029271
6811260	3921	6.931206	6.935273	6.905428	7.197737	-0.26653	0.080566	-0.26246	0.084462	-0.29231	0.059642	0.029844	0.828446	-0.00407	0.976408	0.025778	0.851472	6811260	6.801669	6.84812	7.143829	7.117176	6.676537	7.012105	6.78805	6.914	7.014235	7.289023	7.161686	7.142503	5033411D	BC055852
6811276	3922	8.827475	8.774115	8.679035	8.822825	0.00465	0.963471	-0.04871	0.633922	-0.14379	0.182112	0.095079	0.36226	0.053361	0.602432	0.14844	0.169888	6811276	8.836684	8.826906	8.818836	8.760451	8.896804	8.665089	8.902182	8.616044	8.51888	8.792852	8.773906	8.901718	Rala	NM_019491
6811300	3923	6.125948	6.114055	6.097987	6.078848	0.0471	0.560685	0.035207	0.662085	0.019139	0.811393	0.016068	0.841126	0.011893	0.881981	0.027961	0.727919	6811300	5.972893	6.151475	6.253475	6.170412	6.026402	6.14535	6.091443	6.155833	6.046684	6.102576	5.985719	6.148249	Pou6f2	NM_175006
6811357	3924	7.210518	7.145541	7.099383	7.070188	0.14033	0.193628	0.075353	0.467911	0.029195	0.775328	0.046158	0.653099	0.064977	0.529565	0.111135	0.293652	6811357	7.280242	7.182576	7.168736	7.157089	7.150237	7.129298	7.179724	7.195013	6.923412	7.144048	7.198761	6.867756	Stard3nl	NM_024270
6811366	3925	6.372441	6.36333	6.399927	6.664825	-0.29238	0.261599	-0.30149	0.248195	-0.2649	0.305673	-0.0366	0.883575	0.009111	0.970898	-0.02749	0.912398	6811366	6.229328	6.304179	6.583817	6.268854	6.550368	6.270768	6.82874	6.398832	5.972208	7.041808	6.497308	6.455359	Epdr1	NM_134065
6811487	3932	7.399598	7.528933	7.376608	7.499641	-0.10004	0.327554	0.029292	0.767929	-0.12303	0.235628	0.152324	0.151027	-0.12934	0.21457	0.022989	0.816655	6811487	7.355588	7.502584	7.340621	7.359086	7.60318	7.624532	7.328074	7.492795	7.308956	7.509337	7.618015	7.371571	Zkscan3	ENSMUST000001
6811496	3934	9.213976	9.515312	9.632034	9.590486	-0.37651	0.007566	-0.07517	0.499184	0.041548	0.705876	-0.11672	0.303757	-0.30134	0.021909	-0.41806	0.00432	6811496	9.093966	9.318453	9.229508	9.379156	9.58861	9.578169	9.504903	9.862225	9.528975	9.62734	9.580525	9.563593	Zfp187	NM_001013786
6811512	3936	7.421777	7.585472	7.580768	7.534621	-0.11284	0.241443	0.050851	0.584267	0.046147	0.618911	0.004705	0.959229	-0.1637	0.10381	-0.15899	0.112532	6811512	7.540342	7.45804	7.266948	7.528806	7.603352	7.624259	7.724179	7.508601	7.509523	7.418442	7.592997	7.592424	Zfp192	NM_139141
6811530	3943	6.445209	7.157111	6.432389	5.871289	0.57392	0.163151	1.285822	0.008814	0.5611	0.171638	0.724722	0.088445	-0.7119	0.093251	0.01282	0.973474	6811530	6.293388	6.601071	6.441169	6.825045	7.942344	6.703944	6.801551	6.076779	6.418837	5.758897	6.382699	5.472271	Hist1h1b	NM_020034
6811554	3944	5.568165	5.624973	5.980865	5.979685	-0.41152	0.009245	-0.35471	0.01877	0.00118	0.992441	-0.35589	0.018491	-0.05681	0.650548	-0.4127	0.009112	6811554	5.556326	5.596504	5.551665	5.656466	5.58831	5.630144	6.018118	6.02991	5.894568	6.252102	5.99967	5.687284	Prss16	NM_019429
6811623	3954	5.854929	5.807243	5.741575	5.877844	-0.02292	0.929537	-0.0706	0.785736	-0.13627	0.602165	0.065668	0.800317	0.047686	0.854125	0.113354	0.663697	6811623	5.647964	5.619023	6.297799	5.804859	5.420527	6.196342	5.684783	5.831532	5.70841	6.180065	5.650047	5.80342	V1rh16	NM_134240
6811655	3956	6.426279	6.563147	6.528025	6.57349	-0.14721	0.270874	-0.01034	0.935815	-0.04546	0.724376	0.035122	0.784971	-0.13687	0.303481	-0.10175	0.437337	6811655	6.284428	6.565722	6.428687	6.394635	6.712778	6.582027	6.530277	6.601529	6.452269	6.479343	6.808644	6.432482	Zfp322a	NM_001111107
6811656	3957	6.132864	6.183653	6.341921	6.292179	-0.15931	0.205164	-0.10853	0.374967	0.049742	0.678114	-0.15827	0.207863	-0.05079	0.671806	-0.20906	0.107923	6811656	5.961967	6.082499	6.354127	6.107629	6.280095	6.163235	6.455235	6.314602	6.255925	6.123006	6.379215	6.374315	Abt1	NM_013924
6811663	3958	6.506704	6.093503	5.850686	5.440706	1.065998	0.117082	0.652797	0.31347	0.40998	0.518393	0.242817	0.699564	0.413201	0.515201	0.656018	0.311242	6811663	6.768319	6.05724	6.694553	5.435443	6.740428	6.104639	6.742786	4.433908	6.375364	5.772856	5.371624	5.177639	Btn1a1	NM_013483
6811694	3963	7.125633	7.726551	7.5846	7.417482	-0.29185	0.283008	0.309068	0.257635	0.167117	0.528389	0.141951	0.590953	-0.60092	0.045267	-0.45897	0.107902	6811694	7.280501	7.252196	6.844203	7.721111	8.076378	7.382163	8.0436	7.220222	7.489977	7.613806	7.343945	7.294696	Hfe	NM_010424
6811724	3968	8.404721	8.367372	8.41926	8.467512	-0.06279	0.586128	-0.10014	0.392101	-0.04825	0.674447	-0.05189	0.651772	0.037349	0.744504	-0.01454	0.898752	6811724	8.350379	8.42257	8.441215	8.441844	8.367393	8.292879	8.294075	8.534355	8.429351	8.406527	8.278053	8.717957	Lrrc16a	NM_026825
6811762	3969	5.939822	6.226955	5.724752	5.797796	0.142026	0.232712	0.429159	0.004536	-0.07304	0.525339	0.502203	0.001837	-0.28713	0.031116	0.21507	0.086299	6811762	5.893418	5.91648	6.009568	6.092072	6.195492	6.3933	5.855442	5.733439	5.585374	5.810357	5.955781	5.62725	Gmnn	NM_020567

6811769	3970	8.631323	8.88996	8.640039	8.893429	-0.26211	0.175736	-0.00347	0.984796	-0.25339	0.188921	0.249921	0.194407	-0.25864	0.180883	-0.00872	0.961815	6811769	8.465209	8.61687	8.811891	8.875201	8.732638	9.062041	8.474245	9.001838	8.444035	8.794435	8.78941	9.096442	Acot13	NM_025790
6811776	3971	6.507697	6.768806	6.643566	7.096451	-0.58875	0.001429	-0.32764	0.029333	-0.45288	0.006396	0.12524	0.341029	-0.26111	0.067819	-0.13587	0.304062	6811776	6.470427	6.409319	6.643344	6.7382	6.73547	6.832749	6.61584	6.689965	6.624893	7.003922	6.88585	7.39958	Aldh5a1	NM_172532
6811977	3983	8.075772	8.290781	7.987587	7.66206	0.413712	0.048687	0.628721	0.007728	0.325527	0.104977	0.303194	0.127075	-0.21501	0.261795	0.088185	0.633791	6811977	8.145367	8.086411	7.995538	8.216053	8.59715	8.059139	8.013803	8.069977	7.878981	7.313672	7.922163	7.750344	Sox4	NM_009238
6811997	3984	8.395831	8.359115	8.538155	8.419536	-0.02371	0.846246	-0.06042	0.623491	0.118617	0.345616	-0.17904	0.168825	0.036715	0.76434	-0.14232	0.26357	6811997	8.317863	8.46101	8.408619	8.262076	8.49114	8.32413	8.607774	8.298208	8.708483	8.260566	8.509328	8.488721	Cdkal1	NM_144536
6812035	3985	7.674484	7.828157	7.562262	7.953069	-0.27859	0.274728	-0.12491	0.61334	-0.39081	0.13863	0.265895	0.295577	-0.15367	0.53589	0.112222	0.64932	6812035	7.483813	7.61428	7.925358	7.724043	7.875727	7.884702	7.710709	7.576488	7.399589	7.595853	7.73285	8.530503	Uqcrfs1	NM_025710
6812051	3986	9.645795	9.543731	9.545311	9.130241	0.515553	0.019263	0.413489	0.047217	0.41507	0.046562	-0.00158	0.993075	0.102064	0.578999	0.100484	0.584775	6812051	9.511963	9.827662	9.597759	9.314117	9.717404	9.599671	9.654682	9.260174	9.721077	8.963289	9.398866	9.028569	Exoc2	NM_025588
6812065	3987	7.355062	7.150582	7.092371	7.335977	0.019085	0.939374	-0.18539	0.467732	-0.24361	0.345814	0.058211	0.81684	0.20448	0.424861	0.262691	0.311541	6812065	7.194654	7.412597	7.457935	7.097362	7.191178	7.163207	7.107354	6.940403	7.229356	7.536341	7.766418	6.705171	Hus1b	NM_153072
6812143	3988	7.738547	7.837622	7.811722	7.644057	0.09449	0.515395	0.193565	0.20081	0.167665	0.261728	0.025901	0.856671	-0.09908	0.495807	-0.07317	0.612496	6812143	7.675376	7.682523	7.857742	7.731759	7.832574	7.948534	8.057654	7.640756	7.736755	7.754324	7.779873	7.397974	Gmds	NM_146041
6812212	3992	9.3841	9.434207	9.441581	9.229663	0.154437	0.193614	0.204544	0.096961	0.211918	0.087343	-0.00737	0.947635	-0.05011	0.657449	-0.05748	0.611683	6812212	9.375341	9.402188	9.37477	9.508704	9.407779	9.386137	9.681433	9.400965	9.242345	9.163284	9.380267	9.145438	Serpinb6	NM_009254
6812214	3993	6.926758	6.527889	6.472376	5.554388	1.37237	0.039977	0.973501	0.120507	0.917988	0.139973	0.055513	0.923514	0.398868	0.496767	0.454382	0.440843	6812214	7.004129	7.055152	6.720992	6.601667	6.945451	6.03655	6.968786	5.736551	6.711791	5.433934	6.713305	4.515925	Tubb2a	NM_009450
6812215	3994	9.547744	9.483712	9.334375	9.234132	0.313612	0.077057	0.24958	0.14515	0.100243	0.534929	0.149337	0.362403	0.064032	0.689656	0.213369	0.204926	6812215	9.555494	9.46177	9.625968	9.386155	9.503738	9.561243	9.494257	8.94409	9.564779	9.268571	9.327872	9.105953	Tubb2b	NM_023716
6812250	3995	6.838368	7.01632	6.803718	6.694232	0.144136	0.551948	0.322088	0.20273	0.109485	0.649789	0.212603	0.386535	-0.17795	0.465361	0.034651	0.88504	6812250	6.867161	6.906813	6.741131	7.005435	7.330022	6.713504	7.095825	6.313797	7.001531	6.839633	6.772045	6.471019	1300014I	NM_025831
6812263	3997	7.971485	8.199588	7.940995	7.969805	0.001679	0.992684	0.229783	0.231634	-0.02881	0.875098	0.258593	0.183297	-0.2281	0.234761	0.03049	0.867896	6812263	8.014634	7.994659	7.905161	8.285366	8.281443	8.031956	8.034125	7.9608	7.82806	7.850548	7.65125	8.407618	Peci	NM_011868
6812320	3999	7.980312	8.009176	7.918555	7.912075	0.068238	0.600822	0.097101	0.460589	0.006481	0.960013	0.090621	0.490074	-0.02886	0.823569	0.061757	0.635291	6812320	7.942319	8.129972	7.868646	8.21647	7.923692	7.887366	8.067924	7.860251	7.827491	7.728942	8.039729	7.967553	Lyrm4	NM_201358
6812386	4001	7.877134	7.322676	7.048413	6.877013	1.000121	0.115486	0.445664	0.454131	0.1714	0.769943	0.274264	0.641264	0.554458	0.356373	0.828721	0.181647	6812386	7.905173	7.449154	8.277075	7.409086	7.966512	6.592431	7.520103	7.347508	6.277627	5.876846	7.646678	7.107514	F13a1	NM_028784
6812444	4002	9.632954	9.692084	9.849817	9.916882	-0.28393	0.06779	-0.2248	0.133225	-0.06707	0.631494	-0.15773	0.274676	-0.05913	0.67187	-0.21686	0.14558	6812444	9.711991	9.687607	9.499265	9.507198	9.674282	9.894772	9.999538	9.938435	9.611479	9.931024	10.02613	9.793493	Ssr1	NM_025965
6812505	4004	9.902579	9.938054	10.03785	9.861433	0.041146	0.771184	0.076621	0.590634	0.176419	0.233083	-0.0998	0.486363	-0.03547	0.801876	-0.13527	0.351562	6812505	9.922343	9.89657	9.888825	9.824584	9.936289	10.05329	10.2928	9.929526	9.89123	9.939547	10.03483	9.609922	Txndc5	NM_145367
6812516	4005	6.555527	6.656243	6.652013	6.688294	-0.13277	0.220225	-0.03205	0.756403	-0.03628	0.725711	0.00423	0.967242	-0.10072	0.342592	-0.09649	0.362118	6812516	6.498385	6.68276	6.485435	6.759795	6.714264	6.494669	6.818765	6.573066	6.564207	6.64639	6.636244	6.782248	Muted	NM_139063
6812518	4006	8.916161	8.811441	8.89503	8.689366	0.226795	0.10749	0.122075	0.357853	0.205664	0.13889	-0.08359	0.522937	0.10472	0.426957	0.021131	0.87009	6812518	8.968866	8.886509	8.893108	8.632911	9.088895	8.712518	9.00616	8.953285	8.725644	8.663057	8.80151	8.603531	Eef1e1	NM_025380
6812534	4007	7.660811	7.815558	7.724464	7.530788	0.130023	0.095328	0.284769	0.00325	0.193676	0.022624	0.091093	0.221894	-0.15475	0.054553	-0.06365	0.381737	6812534	7.656213	7.668354	7.657867	7.674565	7.953255	7.818853	7.750417	7.635522	7.787454	7.51356	7.490316	7.588489	Slc35b3	NM_134060
6812642	4012	8.038621	8.341023	8.51654	9.283507	-1.24489	0.003576	-0.94248	0.01506	-0.76697	0.036459	-0.17552	0.58171	-0.3024	0.351611	-0.47792	0.156659	6812642	7.638167	8.336426	8.141271	8.276927	8.343957	8.402184	8.144869	9.2662	8.138552	9.266734	9.217903	9.365883	Elovl2	NM_019423
6812652	4013	9.111083	9.17476	9.122779	8.951318	0.159765	0.254757	0.223442	0.124529	0.171461	0.224418	0.051982	0.70021	-0.06368	0.637976	-0.0117	0.930644	6812652	9.333134	9.072583	8.927532	9.219763	9.248263	9.056255	9.199138	9.26303	8.906168	8.832816	8.965505	9.055633	Nedd9	NM_001111324
6812679	4014	7.359527	7.127427	7.197761	7.372229	-0.0127	0.967075	-0.2448	0.43556	-0.17447	0.57471	-0.07033	0.819505	0.2321	0.458866	0.161766	0.60235	6812679	7.225243	7.146498	7.706839	7.505643	6.609797	7.26684	7.637511	7.130339	6.825432	7.18739	7.645318	7.283978	9530008L	NM_175417
6812770	4015	6.838364	6.960635	6.954907	6.731039	0.107325	0.577682	0.229596	0.249612	0.223869	0.26065	0.005727	0.976054	-0.12227	0.527114	-0.11654	0.54617	6812770	6.85374	6.772349	6.889003	7.135278	7.21713	6.529496	7.031454	6.875172	6.958096	6.577362	6.616205	6.999549	Tbc1d7	NM_025935
6812778	4016	9.031778	9.121532	9.071492	9.143198	-0.11142	0.440875	-0.02167	0.878613	-0.07171	0.615898	0.05004	0.725157	-0.08975	0.531933	-0.03971	0.779902	6812778	9.07806	8.971639	9.045635	9.133651	9.196268	9.034676	9.132623	9.190681	8.891172	8.982738	8.982738	9.466577	Ranbp9	NM_019930
6812871	4017	7.999865	8.177124	8.008076	7.946061	0.053804	0.550725	0.231062	0.028151	0.062015	0.49325	0.169047	0.086067	-0.17726	0.074281	-0.00821	0.926611	6812871	8.010198	8.071757	7.917641	8.181529	8.314334	8.035508	8.16133	7.933489	7.92941	7.96136	7.899548	7.977276	Dtnbp1	NM_025772
6812894	4018	8.601364	8.537096	8.407719	8.535428	0.065936	0.651149	0.001668	0.990812	-0.12771	0.389601	0.129377	0.383733	0.064268	0.659304	0.193645	0.20514	6812894	8.401711	8.731422	8.670959	8.393196	8.697515	8.520578	8.361322	8.379975	8.481861	8.633187	8.716165	8.256933	Atxn1	NM_009124
6812968	4019	8.858568	8.93766	8.884511	8.72677	0.131798	0.188293	0.21089	0.050359	0.157742	0.123479	0.053148	0.577878	-0.07909	0.413197	-0.02594	0.784275	6812968	8.900614	8.891216	8.783874	8.782802	9.058391	8.971786	8.928432	8.999588	8.725514	8.65436	8.809404	8.716545	Nup153	NM_175749
6812973	4020	8.043448	8.14469	7.947192	8.159664	-0.11622	0.240453	-0.01497	0.874271	-0.21247	0.049056	0.197498	0.063296	-0.10124	0.301435	0.096256	0.324306	6812973	8.076085	7.96368	8.090578	8.175796	8.112967	8.145307	8.036242	7.986428	7.818906	8.026918	8.090352	8.361723	Kif13a	NM_010617
6812982	4021	6.859423	6.954623	6.961079	6.997276	-0.13785	0.087282	-0.04265	0.563408	-0.0362	0.622845	-0.00646	0.929555	-0.0952	0.215458	-0.10166	0.188821	6812982	6.807376	7.000468	6.770425	6.846724	7.008172	7.008973	6.961154	6.955094	6.96699	7.086783	6.948666	6.95638	Nhlrc1	NM_175340
6812983	4022	7.133062	7.138252	6.427235	6.287818	0.845244	0.000801	0.850434	0.000771	0.139417	0.414184	0.711017	0.002311	-0.00519	0.975211	0.705827	0.002412	6812983	7.196077	6.986831	7.216279	7.12192	7.070374	7.222462	6.502533	6.09054	6.688632	6.198203	6.520803	6.144449	Tpmt	NM_016785
6812984	4023	9.684186	9.737281	9.759999	9.732952	-0.04877	0.73067	0.004329	0.975529	0.027047	0.848183	-0.02272	0.872207	-0.05309	0.708016	-0.07581	0.594552	6812984	9.642658	9.698307	9.711594	9.659922	9.704132	9.847788	9.869726	9.783183	9.627087	9.512167	9.620659	10.06603	Dek	NM_025900
6813067	4024	7.219127	7.158873	7.241132	7.325783	-0.10666	0.063362	-0.16691	0.009769	-0.08465	0.125687	-0.08226	0.135209	0.060254	0.258298	-0.02201	0.668501	6813067	7.189744	7.322558	7.145079	7.139555	7.170085	7.16698	7.246943	7.308067	7.168386	7.290762	7.35229	7.334298	Zfp169	NM_026450
6813070	4025	6.256178	6.351481	6.304781	6.31126	-0.05508	0.203539	0.040221	0.34157	-0.00648	0.874657	0.0467	0.274169	-0.0953	0.043461	-0.0486	0.256554	6813070	6.257374	6.217341	6.29382	6.414337	6.361425	6.278681	6.248625	6.35294	6.312779	6.286329	6.334755	6.312697	Ptpdc1	NM_207232
6813078	4026	8.730059	8.828563	8.762398	8.786549	-0.05649	0.721453	0.042014	0.790505	-0.02415	0.878441	0.066166	0.676722	-0.0985	0.537565	-0.03234	0.837832	6813078	8.728891	8.738793	8.722494	8.885745	8.92245	8.677495	8.569852	9.159158	8.558183	8.796765	8.849667	8.713215	Phf2	NM_011078
6813084	4027	6.696996	6.579538	6.518325	6.834884	-0.13789	0.559524	-0.25535	0.292228	-0.31656	0.199734	0.061213	0.793788	0.117458	0.618059	0.178671	0.452912	6813084	6.613364	6.662426	6.815197	6.736053	6.227009	6.775551	6.639071	6.801151	6.114753	6.992432	6.520006	6.992213	Wnk2	NM_029361
6813086	4028	8.717977	8.62798	8.542183	8.290131	0.427845	0.004879	0.337849	0.016063	0.252052	0.053056	0.085797	0.462299	0.089996	0.441475	0.175794	0.152348	6813086	8.807235	8.590757	8.755938	8.665404	8.754366	8.464171	8.679283	8.574081	8.373185	8.227178	8.431358	8.211858	1110007C	NM_026738
6813096	4030	6.409513	6.350972	6.32617	6.055134	0.354379	0.096131	0.295838	0.154182	0.271036	0.187322	0.024802	0.898289	0.058542	0.763436	0.083344	0.669244	6813096	6.752538	6.227971	6.248031	6.37762	6.502742	6.172553	6.579112	6.016674	6.382723	5.952167	6.186871	6.026364	Fgd3	NM_015759
6813172	4032	6.040994	5.989344	6.042134	5.936653	0.104341	0.029428	0.052691	0.218205	0.105481	0.028137	-0.05279	0.217422	0.05165	0.226589	-0.00114	0.977637	6813172	6.038582	6.083714	6.000685	6.017639	6.034312	5.916081	6.058134	6.018674	6.049594	5.90303	6.000651	5.906278	Shc3	NM_009167
6813187	4033	8.36514	8.459442	8.482707	8.696309	-0.33117	0.073734	-0.23687	0.179481	-0.2136	0.221264	-0.02326	0.888692	-0.0943	0.574247	-0.11757	0.486128	6813187	8.403767	8.400092	8.291561	8.469492	8.63366	8.275175	8.51067	8.433562	8.503888	8.305114	8.943814	8.84	Sema4d	NM_013660
6813229	4034	6.233633	6.284918	6.174876	6.221608	0.012024	0.945217	0.06331	0.718618	-0.04673	0.789872	0.110042	0.534606	-0.05129	0.770056	0.058757	0.737935	6813229	6.215738	6.359218	6.125942	6.183082	6.482042	6.189631	6.059538	6.396321	6.068769	6.49018	6.283977	5.890668	Diras2	NM_001024474
6813242	4035	7.577011	7.849975	7.728125	7.960717	-0.38371	0.088118	-0.11074	0.590556	-0.23259	0.273027	0.12185	0.554634	-0.27296	0.204543	-0.15111	0.466416	6813242	7.446814	7.657615	7.626605	7.847476	7.670468	8.031981	7.946923	7.729626	7.507825	7.787434	7.703911	8.390807	Auh	NM_016709
6813244	4036	6.351807	6.465585	6.214797	6.351956	-0.00015	0.998681	0.113628	0.231458	-0.13716	0.156652	0.250788	0.021214	-0.11378	0.230898	0.13701	0.15705	6813244	6.495216	6.325031	6.235173	6.406111	6.432239	6.558404	6.318626	6.275679	6.050086	6.375377	6.370283	6.310209	Ror2	NM_013846
6813246	4037	7.459547	7.601032	7.777464	7.183265	0.276282	0.370634	0.417767	0.189408	0.594199	0.075677	-0.17643	0.561488	-0.14148	0.640176	-0.31792	0.306849	6813246	7.579808	7.480715	7.318118	7.391302	8.131982	7.279812	8.199574	7.502143	7.630676	7.590731	7.101091	6.857973	Nfil3	NM_017373
6813253	4038	8.285369	8.416442	8.414438	8.395687	-0.11032	0.209604	0.020755	0.803902	0.018751	0.822436	0.002004	0.980837	-0.13107	0.143673	-0.12907	0.149103	6813253	8.243193	8.342071	8.270842	8.377894	8.483227	8.388204	8.599442	8.351433	8.292439	8.316304	8.479968	8.390788	Sptlc1	NM_009269
6813315	4041	8.804329	8.929881	8.900078	8.823431	-0.0191	0.863174	0.10645	0.350357	0.076647	0.495467	0.029802	0.788313	-0.12555	0.275776	-0.09575	0.398392	6813315	8.802131	8.770918	8.839939	8.838744	9.013079	8.937819	9.111012	8.817023	8.7722	8.637053	8.935547	8.897693	Thoc3	NM_028597
6813317	4042	6.941127	6.916325	6.936442	6.887188	0.053939	0.799499	0.029138	0.890705	0.049255	0.816525	-0.02012	0.924393	0.024802	0.906873	0.004685	0.982363	6813317	6.848418	6.849359	7.125604	7.115381	6.795751	6.837844	6.594059	6.949492	7.265776	6.938869	6.574044	7.14865	Rps24	NM_207635
6813322	4043	11.43612	11.29437	11.38636	11.84783	-0.41171	0.003868	-0.55347	0.000648	-0.46147	0.002001	-0.092	0.395824	0.141757	0.204202	0.04976	0.640495	6813322	11.36715	11.43599	11.50523	11.38709	11.21037	11.28564	11.25055	11.59516	11.31338	11.76108	11.78572	11.9967	4833439L	BC033445
6813326	4044	8.377975	8.340825	8.304836	8.484362	-0.10639	0.251695	-0.14354	0.13409	-0.17953	0.070579	0.035989	0.686976	0.037149	0.677552	0.073139	0.420363	6813326	8.427268	8.335593	8.371063	8.321769	8.474478	8.226229	8.137692	8.441479	8.335337	8.514716	8.422153	8.516217	Nop16	NM_178605
6813327	4045	9.315462	9.12968	9.211622	9.294946	0.020517	0.862652	-0.16527	0.188079	-0.08332	0.488787	-0.08194	0.495806	0.185783	0.14438	0.103841	0.392297	6813327	9.265783	9.250344	9.43026	9.180349	9.158676	9.050014	9.310544	9.214609	9.109712	9.029699	9.389452	9.465686	Cltb	NM_028870
6813332	4046	8.740254	8.775929	8.812337	8.763984	-0.02373	0.888511	0.011946	0.943711	0.048353	0.775582	-0.03641	0.829806	-0.03568	0.833208	-0.07208	0.67185	6813332	8.756147	8.755683	8.708933	8.844366	8.947104	8.536318	8.529117	9.165134	8.742759	8.667556	8.868256	8.756139	Rnf44	NM_001146025
6813348	4047	6.838579	6.908543	7.019865	6.910681	-0.0721	0.425926	-0.00214	0.980766	0.109184	0.239705	-0.11132	0.231413	-0.06996	0.439232	-0.18129	0.067976	6813348	6.725372	6.869936	6.920428	6.756272	7.06776	6.901597	7.001187	7.11628	6.942129	6.912435	6.953901	6.865707	Sncb	NM_033610
6813364	4048	6.999194	7.093065	7.00978	7.086667	-0.08747	0.155745	0.006398	0.911575	-0.07689	0.2057	0.083285	0.174045	-0.09387	0.131144	-0.01059	0.85431	6813364	7.058082	7.087307	6.852192	7.095388	7.108118	7.07569	6.989638	7.01933	7.020372	7.039138	7.107286	7.113578	Uimc1	NM_011307
6813385	4049	8.001249	8.036208	8.06805	7.949547	0.051702	0.391046	0.086661	0.167041	0.118503	0.071308	-0.03184	0.591832	-0.03496	0.556829	-0.0668	0.27509	6813385	8.069601	7.9792	7.954945	7.955964	8.03121	8.121451	8.139952	8.05333	8.010869	7.874894	7.96488	8.008867	Rab24	NM_009000
6813386	4050	6.395049	6.528742	6.46943	6.439952	-0.0449	0.584527	0.08879	0.292621	0.029477	0.718139	0.059312	0.473314	-0.13369	0.128297	-0.07438	0.372963	6813386	6.330949	6.360023	6.494174	6.454051	6.526884	6.605291	6.561625	6.341129	6.505535	6.50432	6.319817	6.49572	Mxd3	NM_016662
6813387	4051	9.866341	9.714482	10.06714	9.941948	-0.07561	0.484994	-0.22747	0.058757	0.125196	0.259985	-0.35266	0.009157	0.151859	0.179637	-0.2008	0.087748	6813387	9.769841	9.941538	9.887645	9.616199	9.784883	9.742365	10.18526	10.04787	9.968303	9.917784	10.14403	9.76403	Lman2	NM_025828
6813396	4054	7.435369	7.75177	7.373684	7.48267	-0.0473	0.659965	0.2691	0.031679	-0.10899	0.32332	0.378086	0.006483	-0.3164	0.015688	0.061685	0.56784	6813396	7.612323	7.420083	7.2737	7.769489	7.867206	7.618614	7.482519	7.397883	7.24065	7.410629	7.547872	7.489508	Dbn1	NM_019813
6813398	4055	8.52805	8.524465	8.269261	8.095097	0.432953	0.043102	0.429368	0.04446	0.174165	0.362381	0.255204	0.194698	0.003585	0.984626	0.258788	0.189144	6813398	8.611488	8.338871	8.63379	8.390108	8.879534	8.303753	8.311595	8.194534	8.301655	8.026276	8.382344	7.87667	Pdlim7	NM_001114088
6813407	4056	6.533348	6.371742	6.517008	6.205905	0.327443	0.056154	0.165837	0.291124	0.311102	0.066798	-0.14527	0.351156	0.161606	0.302756	0.016341	0.914069	6813407	6.709555	6.511811	6.378679	6.434721	6.492623	6.187883	6.7165	6.219127	6.615396	6.238589	6.262777	6.11635	Dok3	NM_013739
6813410	4057	8.030089	8.081296	8.086506	7.897503	0.132586	0.148399	0.183793	0.05744	0.189003	0.052075	-0.00521	0.951429	-0.05121	0.553939	-0.05642	0.51536	6813410	8.042442	8.043327	8.004498	7.910853	8.247475	8.085559	8.115747	8.088745	8.055025	7.886279	8.009784	7.796445	Ddx41	NM_134059
6813411	4058	7.365555	7.422215	7.426172	7.539266	-0.17371	0.102393	-0.11705	0.24919	-0.11309	0.264238	-0.00396	0.967522	-0.05666	0.564155	-0.06062	0.537896	6813411	7.336815	7.439814	7.320036	7.436352	7.376559	7.453733	7.32901	7.657194	7.292311	7.630969	7.520253	7.466576	BC021381	NM_145382

6813470	4061	9.587181	9.476594	9.48465	9.271587	0.315594	0.021093	0.205007	0.100084	0.213063	0.089436	-0.00806	0.943554	0.110588	0.345316	0.102531	0.3797	6813470	9.590673	9.557731	9.61314	9.365672	9.536216	9.527893	9.722811	9.258723	9.472417	9.181284	9.370338	9.263139	H2afy	NM_012015
6813474	4062	6.036153	5.946835	6.075789	5.265737	0.770416	0.053746	0.681098	0.080799	0.810053	0.044827	-0.12895	0.715094	0.089318	0.799957	-0.03964	0.910314	6813474	6.463965	5.954111	5.690383	6.037292	6.308961	5.494252	6.381217	5.631667	6.214484	5.401827	5.647043	4.74834	Tifab	NM_145976
6813480	4064	7.266898	7.153738	7.041806	6.329104	0.937794	0.001044	0.824634	0.002278	0.712702	0.005198	0.111932	0.56662	0.11316	0.562463	0.225092	0.263807	6813480	7.524342	7.055172	7.22118	7.030918	7.463521	6.966775	7.33713	6.832783	6.955505	6.204377	6.385768	6.397168	Cxcl14	NM_019568
6813555	4069	9.047975	9.148428	9.067756	9.137041	-0.08907	0.256331	0.011387	0.879674	-0.06928	0.369475	0.080672	0.300371	-0.10045	0.205297	-0.01978	0.79288	6813555	8.997381	9.045664	9.10088	9.109693	9.199575	9.136017	9.133858	9.143956	8.925455	9.011571	9.20346	9.196092	Ubqln1	NM_152234
6813560	4070	7.359361	7.538696	7.332327	7.48013	-0.12077	0.284803	0.058566	0.593495	-0.1478	0.198248	0.206369	0.085822	-0.17934	0.127135	0.027034	0.803965	6813560	7.274866	7.422721	7.380496	7.481962	7.476643	7.657483	7.300395	7.526208	7.170377	7.523863	7.330369	7.586159	Gkap1	NM_019832
6813570	4072	8.117277	8.333795	7.979744	7.958187	0.15909	0.158312	0.375608	0.006275	0.021558	0.838275	0.35405	0.008531	-0.21652	0.067063	0.137532	0.215452	6813570	8.060571	8.20925	8.082009	8.232665	8.40541	8.363309	8.225878	7.857959	7.855396	7.918514	7.938931	8.017115	Hnrnpk	NM_025279
6813664	4074	8.557714	8.719841	8.543972	9.12858	-0.57087	0.016047	-0.40874	0.061136	-0.58461	0.014361	0.175869	0.376296	-0.16213	0.41297	0.013742	0.943446	6813664	8.445468	8.543637	8.684037	8.953017	8.54409	8.662416	8.62855	8.626349	8.377016	8.997231	8.849318	9.539191	Agtpbp1	NM_023328
6813666	4075	7.703372	7.851384	7.531017	7.162037	0.541335	0.01967	0.689348	0.006023	0.36898	0.082835	0.320368	0.123636	-0.14801	0.449596	0.172355	0.381699	6813666	7.779136	7.721061	7.609918	7.753968	8.079528	7.720657	7.8056	7.131342	7.656108	7.097375	7.375422	7.013313	Golm1	NM_027307
6813676	4079	8.945185	8.952038	8.858125	8.929475	0.01571	0.865138	0.022563	0.807481	-0.07135	0.448725	0.093913	0.325094	-0.00685	0.940898	0.08706	0.359569	6813676	8.980064	8.945375	8.910116	8.939049	8.938603	8.978462	8.916825	8.885241	8.772308	8.738874	8.909106	9.140444	Zcchc6	NM_153538
6813699	4080	7.164953	7.199173	6.926532	7.503257	-0.3383	0.152735	-0.30408	0.193302	-0.57672	0.027339	0.272641	0.23862	-0.03422	0.876973	0.23842	0.297792	6813699	7.03185	7.418525	7.044483	7.216529	7.334266	7.046725	6.882601	7.148247	6.748749	7.192533	7.355114	7.962125	Gas1	NM_008086
6813742	4084	6.056395	6.546434	6.06076	6.34233	-0.28593	0.084252	0.204104	0.19715	-0.28157	0.088262	0.485674	0.01012	-0.49004	0.009679	-0.00436	0.976737	6813742	5.859607	6.428701	5.880877	6.559083	6.456132	6.624087	6.133674	6.127243	5.921363	6.348512	6.360561	6.317916	Ctla2b	NM_001145801
6813795	4095	7.137911	7.368943	7.068817	7.228879	-0.09097	0.752602	0.140064	0.628963	-0.16006	0.581702	0.300126	0.313134	-0.23103	0.431359	0.069095	0.810525	6813795	7.093774	7.495077	6.824883	7.35119	7.146376	7.609262	6.893992	7.275268	7.03719	6.643694	7.450917	7.592026	Fbp2	NM_007994
6813797	4096	8.138473	9.131062	8.915542	10.5305	-2.39202	0.000272	-1.39944	0.00696	-1.61496	0.003179	0.21552	0.594231	-0.99259	0.033913	-0.77707	0.080493	6813797	8.182232	8.411204	7.821983	8.890318	9.073716	9.429151	8.504995	9.646133	8.595498	10.52474	9.950361	11.11639	Fbp1	NM_019395
6813844	4098	8.545004	8.624812	8.748106	8.854645	-0.30964	0.020073	-0.22983	0.063959	-0.10654	0.348521	-0.12329	0.282437	-0.07981	0.477058	-0.2031	0.094215	6813844	8.564782	8.60518	8.465051	8.645802	8.605924	8.622709	8.635142	8.869416	8.73976	8.724666	8.728231	9.111039	Ptch1	NM_008957
6813876	4100	6.207759	6.126344	6.300469	5.688245	0.519514	0.153215	0.438099	0.21996	0.612225	0.09999	-0.17413	0.61123	0.081415	0.810908	-0.09271	0.785393	6813876	6.287797	6.373307	5.962173	5.992115	6.232594	6.154323	6.832455	5.852361	6.216592	5.048077	5.820105	6.196552	Slc35d2	NM_001001321
6813877	4101	7.873985	7.943297	7.869128	7.593874	0.280111	0.138857	0.349423	0.074457	0.275254	0.144927	0.074168	0.674901	-0.06931	0.694874	0.004857	0.97796	6813877	7.986358	7.705474	7.930124	7.918651	7.826668	8.084571	8.252577	7.825986	7.528822	7.533794	7.620227	7.627601	Zfp367	NM_175494
6813880	4102	7.95493	7.86228	7.879803	7.659031	0.295899	0.156403	0.203249	0.313969	0.220772	0.276797	-0.01752	0.928475	0.09265	0.637445	0.075127	0.701651	6813880	7.804832	7.975612	8.084346	7.487554	8.192341	7.906945	7.980956	7.620346	8.038107	7.542634	7.80508	7.629379	Cdc14b	NM_172587
6813881	4103	8.795652	8.904358	8.912612	8.989468	-0.19382	0.016725	-0.08511	0.222303	-0.07686	0.266321	-0.00825	0.901052	-0.10871	0.129462	-0.11696	0.106494	6813881	8.667913	8.928631	8.790413	8.923354	8.823204	8.966516	8.932709	8.944522	8.860605	8.900788	8.971508	8.988712	1110018J	NM_025370
6813887	4104	12.44047	12.61159	12.38294	12.2394	0.201063	0.051741	0.37219	0.002884	0.14354	0.141619	0.22865	0.031743	-0.17113	0.087803	0.057523	0.531786	6813887	12.46094	12.33493	12.52553	12.50339	12.64797	12.68342	12.55161	12.33021	12.26701	12.23142	12.16843	12.31836	Ctsl	NM_009984
6813931	4105	7.968371	8.127796	8.19508	8.025651	-0.05728	0.632116	0.102145	0.400733	0.169429	0.179231	-0.06728	0.574948	-0.15943	0.20342	-0.22671	0.084387	6813931	7.917255	8.099257	7.8886	8.011881	8.299894	8.071613	8.419459	8.119248	8.046534	7.953401	8.080039	8.043513	Hiatl1	NM_133680
6813965	4109	8.355865	8.260626	8.535063	8.480438	-0.12457	0.348235	-0.21981	0.116771	0.054625	0.673722	-0.27444	0.059447	0.095239	0.468057	-0.1792	0.189667	6813965	8.263392	8.406837	8.397366	8.056426	8.435287	8.290166	8.722894	8.486252	8.396042	8.308056	8.561322	8.571935	Mterfd1	NM_025547
6813982	4110	5.685186	5.800546	6.439215	6.047748	-0.36256	0.377372	-0.2472	0.541805	0.391467	0.342496	-0.63867	0.138334	-0.11536	0.773772	-0.75403	0.087859	6813982	5.859847	6.166582	5.029128	5.375395	5.781877	6.244367	6.313666	6.829994	6.173986	5.810831	5.712118	6.620296	Zfp708	NM_001012325
6814012	4111	6.927974	6.509152	6.869584	6.93638	-0.00841	0.975706	-0.42723	0.149	-0.0668	0.809156	-0.36043	0.214873	0.418822	0.156149	0.05839	0.832719	6814012	6.484767	7.085057	7.214097	6.444647	6.681808	6.401	7.008772	7.181225	6.418754	6.580826	7.090541	7.137773	Zfp458	NM_001001152
6814028	4114	6.689262	6.518802	6.873301	6.874069	-0.18481	0.260107	-0.35527	0.048175	-0.00077	0.996101	-0.3545	0.048556	0.17046	0.296066	-0.18404	0.261939	6814028	6.635369	6.839243	6.593175	6.248882	6.553184	6.75434	6.848328	6.897697	6.873877	6.644001	6.858218	7.119989	Zfp595	NM_177622
6814044	4117	5.639644	5.433978	6.18733	6.021443	-0.3818	0.310063	-0.58747	0.133971	0.165887	0.650308	-0.75335	0.064941	0.205666	0.575455	-0.54769	0.15865	6814044	6.052521	5.694238	5.172172	5.34131	4.910319	6.050304	6.203836	6.088587	6.269566	5.75091	5.764163	6.549256	9630025I	ENSMUST000000
6814050	4118	6.305502	6.367646	6.340648	6.42817	-0.12267	0.509061	-0.06052	0.741898	-0.08752	0.635202	0.026998	0.882873	-0.06214	0.735296	-0.03515	0.847978	6814050	6.427331	6.383413	6.105763	6.096393	6.298008	6.708537	6.493595	6.316436	6.211914	6.266535	6.362554	6.655421	Zfp58	NM_001007575
6814055	4119	6.342587	6.346981	6.596185	6.502211	-0.15962	0.280565	-0.15523	0.293062	0.093975	0.514924	-0.2492	0.108445	-0.00439	0.975368	-0.2536	0.103282	6814055	6.474287	6.407588	6.145886	6.26582	6.360863	6.41426	6.58873	6.426656	6.77317	6.277071	6.513287	6.716274	Zfp87	NM_133228
6814070	4121	8.851691	8.777401	8.928346	8.732039	0.119653	0.585913	0.045363	0.83502	0.196308	0.379022	-0.15094	0.494372	0.07429	0.733658	-0.07666	0.72558	6814070	8.606792	8.773961	9.174321	8.589517	8.99305	8.749637	9.181314	8.643952	8.959773	8.455918	8.971232	8.768966	A530054K	NM_183146
6814150	4124	7.505172	7.427888	7.479344	7.313128	0.192044	0.207027	0.11476	0.43574	0.166216	0.268801	-0.05146	0.722519	0.077284	0.595699	0.025828	0.858105	6814150	7.510658	7.495401	7.509457	7.365822	7.677364	7.240478	7.338605	7.73184	7.367588	7.282928	7.46243	7.194027	Pols	NM_198600
6814151	4125	6.237827	6.119571	6.163435	6.076458	0.161369	0.090236	0.043113	0.620715	0.086977	0.329511	-0.04386	0.614739	0.118256	0.195744	0.074392	0.400433	6814151	6.335223	6.115162	6.263096	6.004124	6.146264	6.208324	6.068263	6.312152	6.109889	6.075687	6.037591	6.116096	A530095I	ENSMUST000000
6814157	4126	7.466006	6.71673	8.71735	6.359421	1.106586	0.21415	0.357309	0.674562	2.35793	0.020659	-2.00062	0.040589	0.749276	0.387587	-1.25134	0.165541	6814157	8.301152	7.946862	6.150005	5.912807	6.397317	7.840066	9.197783	8.201621	8.752647	5.425932	5.933419	7.718911	Srd5a1	NM_175283
6814200	4127	7.796695	7.941849	7.870913	7.713155	0.08354	0.325405	0.228694	0.020891	0.157758	0.083248	0.070936	0.399631	-0.14515	0.106188	-0.07422	0.379201	6814200	7.768043	7.842317	7.779724	7.809294	8.083713	7.93254	7.897988	7.95981	7.75494	7.66096	7.8069	7.671605	BC018507	BC026613
6814206	4128	7.103221	8.205492	7.46764	7.893689	-0.79047	0.048495	0.311803	0.385787	-0.42605	0.245433	0.737852	0.061743	-1.10227	0.011826	-0.36442	0.314934	6814206	7.031741	7.072318	7.205603	8.880455	7.789851	7.946169	7.293367	7.839096	7.270457	7.85125	7.433072	8.396744	Adamts16	NM_172053
6814330	4130	8.604038	8.714806	8.681698	8.786896	-0.18286	0.548145	-0.07209	0.810993	-0.1052	0.727663	0.033108	0.912417	-0.11077	0.713981	-0.07766	0.79676	6814330	8.756765	8.522305	8.533045	8.755836	8.808693	8.579888	8.601008	9.162503	8.281583	8.356704	8.626763	9.377221	Ndufs6	NM_010888
6814348	4132	5.763743	5.912809	5.73697	6.140714	-0.37697	0.125139	-0.2279	0.330766	-0.40374	0.103958	0.17584	0.447439	-0.14907	0.517366	0.026774	0.906188	6814348	5.609729	5.795106	5.886395	5.942656	5.629135	6.166637	5.72843	5.780159	5.70232	5.777205	6.634332	6.010605	Slc6a19	NM_028878
6814355	4133	7.532761	7.213865	6.937612	7.856755	-0.32399	0.047731	-0.64289	0.001678	-0.91914	0.000165	0.276254	0.081573	0.318896	0.05055	0.595149	0.002649	6814355	7.625504	7.547507	7.425272	7.171674	7.390775	7.079147	7.0143	7.074922	6.723613	7.665229	8.082394	7.822642	Nkd2	NM_028186
6814382	4135	8.000496	8.042024	8.215912	8.008208	-0.00771	0.947582	0.033815	0.773707	0.207704	0.105124	-0.17389	0.164661	-0.04153	0.724374	-0.21542	0.094723	6814382	7.927209	8.072904	8.001376	8.015964	8.182327	7.92778	8.158056	8.134309	8.355371	7.886016	8.241375	7.897234	Exoc3	NM_177333
6814385	4137	9.036743	9.126989	9.076969	8.978604	0.058139	0.608902	0.148385	0.211264	0.098365	0.394011	0.05002	0.659092	-0.09025	0.432514	-0.04023	0.722156	6814385	8.963673	9.027445	9.119112	9.101783	9.264538	9.014645	9.303849	9.014814	8.912243	8.956752	8.901447	9.077613	Pdcd6	NM_011051
6814387	4138	10.31891	10.33184	10.49972	10.65324	-0.33433	0.17035	-0.3214	0.185565	-0.15352	0.508637	-0.16788	0.47104	-0.01293	0.954978	-0.18081	0.438797	6814387	10.32376	10.27712	10.35586	10.36411	10.12531	10.5061	10.53819	10.56454	10.39643	10.31163	10.42257	11.22553	Sdha	NM_023281
6814407	4139	9.76334	9.65161	9.815893	9.513508	0.249832	0.025689	0.138102	0.169168	0.302385	0.010714	-0.16428	0.109927	0.11173	0.256247	-0.05255	0.581038	6814407	9.809277	9.702437	9.778307	9.594335	9.73982	9.620675	9.988053	9.730049	9.729578	9.359979	9.556137	9.624408	Cast	NM_009817
6814441	4140	6.811045	7.284571	6.635525	7.194942	-0.3839	0.015227	0.08963	0.493197	-0.55942	0.002052	0.649046	0.000823	-0.47353	0.005288	0.17552	0.19735	6814441	6.663823	6.933436	6.835877	7.439021	7.334703	7.07999	6.76556	6.624651	6.516365	7.321689	7.246	7.017136	Rhobtb3	NM_028493
6814448	4141	6.472224	6.77911	6.660643	6.667061	-0.19484	0.127732	0.11205	0.357123	-0.00642	0.956742	0.118467	0.331776	-0.30689	0.028091	-0.18842	0.138984	6814448	6.394017	6.540768	6.481888	6.989577	6.740428	6.607326	6.72326	6.610059	6.64861	6.459143	6.778656	6.763383	Rfesd	NM_178916
6814451	4143	7.402881	7.221268	7.591353	7.294857	0.108024	0.255132	-0.07359	0.427921	0.296497	0.009866	-0.37009	0.002998	0.181613	0.073263	-0.18847	0.064909	6814451	7.366939	7.484978	7.356725	7.284844	7.221878	7.157081	7.7274	7.567194	7.479466	7.15179	7.287159	7.445621	Arsk	NM_029847
6814666	4147	7.044503	6.88643	6.852304	7.286714	-0.24221	0.22229	-0.40028	0.060203	-0.43441	0.045004	0.034126	0.856733	0.158072	0.412927	0.192198	0.324359	6814666	6.827862	7.043964	7.261682	6.840769	6.640102	7.17842	6.855164	6.987458	6.714291	7.235909	7.555718	7.068514	Polr3g	NM_001081176
6814827	4148	9.017918	9.185351	8.977418	8.831905	0.186014	0.097529	0.353447	0.007355	0.145514	0.180445	0.207933	0.069274	-0.16743	0.1298	0.0405	0.693681	6814827	9.007855	9.073627	8.972273	9.028402	9.327544	9.200108	9.090727	8.935719	8.905809	8.666002	8.972526	8.857186	Rasa1	NM_145452
6814956	4149	7.116172	7.128769	6.330999	5.781828	1.334344	0.033079	1.34694	0.031854	0.549171	0.320903	0.797769	0.162823	-0.0126	0.981232	0.785173	0.168777	6814956	7.303278	7.385178	6.660061	7.149913	7.857092	6.379301	7.065123	5.695559	6.232316	5.059035	5.353362	5.933088	Vcan	NM_001081249
6814957	4150	7.886895	7.934448	7.992818	7.965434	-0.07854	0.55471	-0.03099	0.813958	0.027384	0.835184	-0.05837	0.65902	-0.04755	0.718649	-0.10592	0.429846	6814957	7.8466	7.89119	7.922896	7.805715	8.081137	7.916493	8.256069	7.787896	7.934489	7.804824	8.042359	8.049118	Xrcc4	NM_028012
6814996	4151	7.278241	7.410711	7.448761	7.582619	-0.30438	0.155831	-0.17191	0.402064	-0.13386	0.510324	-0.03805	0.849613	-0.13247	0.514597	-0.17052	0.405705	6814996	7.051422	7.358119	7.425181	7.410963	7.268633	7.552537	7.295958	7.618368	7.431958	7.278153	7.468583	8.001122	Atg10	NM_025770
6815021	4152	8.009251	8.066367	7.896165	7.846043	0.161156	0.097837	0.218273	0.034825	0.048071	0.591512	0.170202	0.083183	-0.05712	0.5253	0.113086	0.224996	6815021	8.004049	8.031656	7.992047	7.918823	8.229706	8.050573	7.919022	7.970391	7.799082	7.727228	7.877942	7.939113	Zcchc9	NM_145453
6815022	4153	5.501797	5.796661	5.398721	4.831549	0.670248	0.570589	0.965111	0.419218	0.567172	0.63024	0.397939	0.734567	-0.29486	0.801303	0.103076	0.92977	6815022	3.865851	4.837842	7.801699	7.382907	5.380932	4.626143	6.338243	5.150909	4.707012	3.934833	4.880427	5.679388	Ckmt2	NM_198415
6815027	4154	5.960939	6.065595	5.814929	6.195726	-0.23479	0.132089	-0.13013	0.379863	-0.3808	0.02626	0.250666	0.111189	-0.10466	0.476175	0.14601	0.327514	6815027	6.110892	6.100913	5.671012	6.118698	6.030083	6.048003	5.905975	5.679694	5.859118	6.107311	6.419464	6.060402	Rasgrf2	NM_009027
6815031	4155	6.378163	6.518158	6.429606	6.442244	-0.06408	0.508976	0.075914	0.436571	-0.01264	0.894927	0.088552	0.367443	-0.13999	0.169458	-0.05144	0.594132	6815031	6.31348	6.473418	6.347592	6.477785	6.683866	6.392823	6.531812	6.39834	6.358665	6.322014	6.44758	6.557139	Msh3	NM_010829
6815046	4156	6.395541	6.485988	6.49324	6.522316	-0.12677	0.10148	-0.03633	0.610423	-0.02908	0.682534	-0.00725	0.918323	-0.09045	0.223398	-0.0977	0.191788	6815046	6.420275	6.448351	6.317997	6.427604	6.522973	6.507387	6.481883	6.491453	6.506385	6.381345	6.516675	6.668928	Zfyve16	NM_173392
6815058	4158	8.512353	9.178803	8.545863	7.644491	0.867862	0.200043	1.534312	0.038759	0.901371	0.184944	0.632941	0.338204	-0.66645	0.314766	-0.03351	0.958316	6815058	7.861275	9.498612	8.177173	9.632477	9.441024	8.462909	9.238503	7.813686	8.585399	6.721166	7.962734	8.249574	Thbs4	NM_011582
6815074	4159	6.891214	6.529134	6.567578	6.637915	0.2533	0.789559	-0.10878	0.908577	-0.07034	0.940796	-0.03844	0.967615	0.362081	0.703519	0.323636	0.733487	6815074	5.477544	6.364285	8.831814	7.444055	6.47072	5.672626	7.319001	6.328594	6.05514	5.620064	6.982238	7.311442	Cmya5	ENSMUST000000
6815086	4160	8.791161	8.855222	8.932407	8.809233	-0.01807	0.86485	0.045989	0.666552	0.123174	0.265237	-0.07719	0.474361	-0.06406	0.550618	-0.14125	0.206804	6815086	8.932761	8.871108	8.569613	8.755683	9.002981	8.807001	9.009636	8.883087	8.904497	8.755469	8.792696	8.879534	Papd4	NM_133905
6815111	4161	8.02104	8.218284	8.114802	8.06558	-0.04454	0.734989	0.152703	0.263816	0.049222	0.70858	0.103481	0.438985	-0.19724	0.15919	-0.09376	0.48165	6815111	8.05681	8.053227	7.953082	8.113275	8.414362	8.127214	8.179213	8.294733	7.870461	8.09008	7.927001	8.17966	Jmy	NM_021310
6815188	4163	9.178663	9.265795	9.171939	9.260097	-0.08143	0.218525	0.005698	0.927858	-0.08816	0.186312	0.093856	0.16237	-0.08713	0.190942	0.006724	0.914918	6815188	9.273683	9.157383	9.104923	9.290318	9.230217	9.276849	9.14782	9.292687	9.075309	9.219427	9.261166	9.299697	Scamp1	NM_029153
6815255	4165	8.051892	8.007751	7.966048	8.034545	0.017347	0.859195	-0.02679	0.784378	-0.0685	0.490033	0.041703	0.671278	0.044141	0.653515	0.085844	0.391096	6815255	8.015554	8.187905	7.952217	7.915622	8.135923	7.971708	8.017195	8.049362	7.831586	7.909965	8.115511	8.078159	Aggf1	NM_025630
6815268	4167	6.672056	6.900768	6.898667	6.564286	0.10777	0.695053	0.336482	0.240086	0.334381	0.242783	0.002101	0.993872	-0.22871	0.413459	-0.22661	0.41758	6815268	6.936199	6.390029	6.689941	7.228742	6.866377	6.607184	6.985545	7.259983	6.450473	6.469086	6.341304	6.882468	F2rl1	NM_007974
6815271	4168	9.04668	9.233525	8.869972	9.115757	-0.06908	0.612293	0.117767	0.394919	-0.24579	0.097474	0.363553	0.024104	-0.18684	0.191626	0.176709	0.214305	6815271	9.08091	9.159426	8.899705	9.085909	9.386869	9.227796	8.668865	9.044757	8.896293	9.052637	9.301352	8.993283	F2r	NM_010169

6815291	4169	7.124669	7.25748	7.138822	6.948022	0.176646	0.099889	0.309458	0.011546	0.190799	0.079362	0.118658	0.246765	-0.13281	0.199471	-0.01415	0.885206	6815291	7.124297	7.137694	7.112015	7.228032	7.230595	7.313813	7.248082	7.010452	7.157931	6.770225	6.920926	7.152916	Polk	NM_012048
6815305	4170	7.700013	7.884059	7.790744	7.624624	0.075389	0.68823	0.259436	0.189993	0.16612	0.385942	0.093315	0.620396	-0.18405	0.339378	-0.09073	0.629959	6815305	7.765398	7.484033	7.850607	7.690911	8.156713	7.804554	7.886706	7.823774	7.661752	7.360068	7.944387	7.569416	Hmgcr	NM_008255
6815345	4171	10.68168	10.68008	10.62804	10.25212	0.429553	0.016079	0.42796	0.016357	0.375913	0.028814	0.052047	0.722233	0.001593	0.991281	0.05364	0.714167	6815345	10.85075	10.69967	10.49461	10.62542	10.74022	10.67461	10.89479	10.33408	10.65524	10.18608	10.33154	10.23875	Hexb	NM_010422
6815382	4173	9.68721	9.932241	10.0852	9.615957	0.071253	0.785928	0.316283	0.247735	0.469239	0.101513	-0.15296	0.563234	-0.24503	0.362372	-0.39799	0.155314	6815382	9.763166	9.485712	9.812753	10.10441	9.665392	10.02633	10.20368	10.08735	9.96456	9.340828	9.274694	10.23235	Rgnef	NM_012026
6815390	4174	7.007119	7.085269	7.15017	6.885192	0.121927	0.28381	0.200077	0.096135	0.264978	0.037132	-0.0649	0.557832	-0.07815	0.482546	-0.14305	0.214622	6815390	7.088572	6.969758	6.963027	7.00364	7.316122	6.936045	7.216194	7.132854	7.101461	6.805239	7.039943	6.810393	Utp15	NM_178918
6815391	4175	10.71353	10.66248	10.64072	10.77219	-0.05865	0.572022	-0.10971	0.302516	-0.13147	0.223178	0.02176	0.832459	0.051057	0.621922	0.072817	0.485401	6815391	10.77196	10.70695	10.66169	10.69738	10.54724	10.74281	10.77573	10.7266	10.41982	10.86825	10.68172	10.76659	Btf3	NM_145455
6815433	4177	8.976653	9.033062	9.054509	9.070578	-0.09393	0.013	-0.03752	0.239736	-0.01607	0.601258	-0.02145	0.488472	-0.05641	0.092569	-0.07786	0.029906	6815433	8.999958	8.99529	8.93471	9.026184	9.062731	9.01027	9.095503	9.043186	9.024838	9.033875	9.117767	9.060092	Fcho2	NM_172591
6815437	4178	9.234983	9.363083	9.342033	9.256231	-0.02125	0.810494	0.106852	0.247873	0.085803	0.346197	0.021049	0.812223	-0.1281	0.173465	-0.10705	0.247069	6815437	9.21009	9.219962	9.274897	9.205776	9.43075	9.452722	9.486446	9.351943	9.187711	9.211337	9.261331	9.296024	Tnpo1	NM_001048267
6815479	4179	6.929062	7.008608	7.016643	6.991252	-0.06219	0.559979	0.017356	0.869449	0.025391	0.810171	-0.00803	0.939306	-0.07955	0.459057	-0.08758	0.416695	6815479	6.962209	6.965112	6.859864	7.000046	7.027454	6.998325	6.879677	7.228542	6.941711	6.817378	7.117946	7.038432	Ptcd2	NM_026873
6815490	4180	10.68976	10.77783	10.80333	10.91855	-0.22879	0.274034	-0.14072	0.490687	-0.11522	0.570598	-0.02551	0.899072	-0.08807	0.663247	-0.11358	0.575983	6815490	10.91383	10.39638	10.75906	11.00147	10.62043	10.71158	10.81414	10.86584	10.73002	10.61753	10.85702	11.2811	Mtap1b	NM_008634
6815491	4181	7.956331	7.770017	7.491494	8.112092	-0.15576	0.526297	-0.34207	0.183793	-0.6206	0.029739	0.278523	0.270183	0.186314	0.450982	0.464837	0.083439	6815491	8.13973	7.774835	7.954428	8.006761	7.511802	7.791488	7.135514	7.837979	7.50099	7.989776	7.853611	8.492889	5330431K	AK019926
6815511	4182	6.543686	6.517943	6.521111	6.516245	0.027441	0.816097	0.001697	0.988502	0.004865	0.967052	-0.00317	0.978542	0.025744	0.827251	0.022576	0.848178	6815511	6.589624	6.38802	6.653415	6.448348	6.547525	6.557955	6.608438	6.539947	6.414947	6.618433	6.270483	6.65982	Cartpt	NM_013732
6815518	4183	8.687417	9.019093	8.773322	8.736593	-0.04918	0.712524	0.282499	0.059592	0.036729	0.782744	0.245771	0.092768	-0.33168	0.032852	-0.0859	0.523524	6815518	8.554241	8.660135	8.847876	8.865478	8.998793	9.193007	8.808846	8.79216	8.71896	8.59423	8.626697	8.988853	Mccc2	NM_030026
6815522	4184	6.231048	6.148536	6.102811	5.527985	0.703063	0.016998	0.620551	0.029236	0.574826	0.039618	0.045725	0.850029	0.082512	0.73363	0.128237	0.598832	6815522	6.417934	6.136864	6.138347	6.154181	6.331335	5.960093	6.29413	5.597142	6.417161	5.239077	5.772792	5.572086	Naip2	NM_010872
6815538	4188	6.570505	6.698742	6.654674	6.419606	0.1509	0.220807	0.279136	0.039528	0.235069	0.072367	0.044067	0.708261	-0.12824	0.291783	-0.08417	0.480021	6815538	6.628315	6.550623	6.532578	6.609523	6.839093	6.647609	6.746812	6.497349	6.719862	6.187217	6.558021	6.513579	Gtf2h2	NM_022011
6815539	4189	8.211846	7.727292	7.876002	8.000251	0.211595	0.412573	-0.27296	0.297227	-0.12425	0.625474	-0.14871	0.560392	0.484554	0.083137	0.335843	0.207343	6815539	7.859464	8.376705	8.399368	7.526138	7.626649	8.029089	8.00659	7.799018	7.822399	8.123393	8.352436	7.524924	Ocln	NM_008756
6815542	4190	6.998577	6.934533	6.982587	6.786484	0.212093	0.12353	0.148049	0.263943	0.196103	0.150187	-0.04805	0.706744	0.064044	0.617328	0.01599	0.899958	6815542	6.922987	6.990599	7.082145	6.697801	7.161767	6.944031	7.115104	6.941642	6.891014	6.707654	6.937137	6.714661	Marveld2	NM_001038602
6815543	4191	6.810362	6.870396	6.742869	6.778361	0.032001	0.769127	0.092035	0.407922	-0.03549	0.74494	0.127527	0.260765	-0.06003	0.584533	0.067493	0.53978	6815543	6.766112	6.932233	6.732741	6.806859	6.979387	6.824942	6.900103	6.804543	6.523961	6.683696	6.789932	6.861454	Rad17	NM_011233
6815551	4192	6.6301	6.834381	6.662683	6.772621	-0.14252	0.607397	0.06176	0.822583	-0.10994	0.690838	0.171698	0.537498	-0.20428	0.465448	-0.03258	0.905723	6815551	6.508429	6.465245	6.916625	6.23298	7.02984	7.240323	6.531612	6.927653	6.528783	6.593136	6.933662	6.791064	Mrps36	NM_025369
6815560	4195	9.239359	9.570585	9.479821	9.492169	-0.25281	0.039853	0.078416	0.468858	-0.01235	0.907648	0.090764	0.404475	-0.33123	0.012393	-0.24046	0.048041	6815560	9.098223	9.305972	9.313883	9.440608	9.589565	9.681581	9.395737	9.689917	9.353808	9.48571	9.517703	9.473093	Slc30a5	NM_022885
6815599	4196	10.41438	10.52866	10.26911	10.54093	-0.12656	0.384893	-0.01227	0.931163	-0.27182	0.083802	0.259547	0.09616	-0.11428	0.430602	0.145263	0.322237	6815599	10.43631	10.45964	10.34718	10.29899	10.6006	10.68639	10.05821	10.56029	10.18884	10.50602	10.55082	10.56596	Pik3r1	NM_001077495
6815682	4197	8.574696	8.753326	8.471827	8.701759	-0.12706	0.120084	0.051566	0.500194	-0.22993	0.013634	0.281499	0.004848	-0.17863	0.040197	0.102869	0.196639	6815682	8.597738	8.583525	8.542824	8.691546	8.754332	8.814099	8.374671	8.584411	8.456398	8.685806	8.582973	8.836499	Sfrs12	NM_172592
6815687	4198	9.886302	9.982821	9.995981	10.10112	-0.21482	0.20784	-0.1183	0.472143	-0.10514	0.521353	-0.01316	0.935167	-0.09652	0.555233	-0.10968	0.504015	6815687	9.997788	9.936877	9.724241	10.11845	9.891079	9.938935	10.03524	10.07237	9.880333	9.868062	9.96992	10.46537	Erbb2ip	NM_001005868
6815698	4199	7.143463	7.119018	7.093358	7.014581	0.128882	0.253959	0.104437	0.348432	0.078777	0.474011	0.02566	0.812844	0.024445	0.821518	0.050105	0.645554	6815698	7.212024	7.067472	7.150894	7.209477	7.175962	6.971615	7.198638	7.022967	7.05847	6.801894	7.081225	7.160625	Nln	NM_029447
6815702	4200	8.476523	8.538503	8.463516	8.425867	0.050656	0.540216	0.112636	0.192671	0.037648	0.647157	0.074987	0.371337	-0.06198	0.456303	0.013007	0.873589	6815702	8.460622	8.474801	8.494146	8.428526	8.668753	8.51823	8.544547	8.424181	8.421819	8.274031	8.48226	8.521311	2410002O	NM_025879
6815708	4201	7.937517	7.822419	8.019563	7.895424	0.042093	0.846352	-0.073	0.737433	0.124138	0.571284	-0.19714	0.375961	0.115098	0.599098	-0.08205	0.706614	6815708	7.727118	8.221179	7.864254	7.444168	8.15836	7.86473	7.816278	8.18954	8.05287	8.000402	8.008801	7.67707	Ppwd1	NM_172807
6815726	4202	7.808592	7.868119	7.831427	7.761005	0.047587	0.580494	0.107115	0.230985	0.070422	0.418825	0.036692	0.668751	-0.05953	0.491758	-0.02284	0.789268	6815726	7.784084	7.774999	7.866692	7.895544	7.859023	7.849791	7.873497	7.753844	7.86694	7.65453	7.656774	7.97171	Sdccag10	NM_026072
6815749	4205	6.950599	7.014985	7.000905	6.465484	0.485115	0.022356	0.549501	0.012635	0.535421	0.014297	0.01408	0.936689	-0.06439	0.717543	-0.05031	0.777092	6815749	7.17734	6.753215	6.921242	7.00578	7.135877	6.903298	7.12068	6.80048	7.081555	6.443445	6.771425	6.181582	Rnf180	NM_027934
6815792	4206	7.449559	7.57781	7.523744	7.448494	0.001066	0.99399	0.129316	0.373249	0.07525	0.598135	0.054066	0.703668	-0.12825	0.377003	-0.07418	0.603237	6815792	7.385014	7.522146	7.441518	7.447255	7.729988	7.556187	7.766303	7.284156	7.520772	7.25227	7.573622	7.519589	Ipo11	NM_029665
6815797	4207	7.312323	7.426214	7.22906	7.239636	0.072687	0.525627	0.186578	0.126923	-0.01058	0.925462	0.197154	0.109584	-0.11389	0.328876	0.083263	0.468997	6815797	7.354774	7.20069	7.381505	7.348014	7.692089	7.238538	7.287836	7.248528	7.150816	7.196307	7.252869	7.269731	Kif2a	NM_008442
6815853	4208	8.427346	8.586977	8.318696	8.08375	0.343596	0.043222	0.503227	0.007915	0.234946	0.139505	0.268282	0.097893	-0.15963	0.297342	0.10865	0.46979	6815853	8.528867	8.412485	8.340686	8.432894	8.875686	8.452352	8.426683	8.331064	8.19834	8.051589	8.293461	7.9062	Zswim6	NM_145456
6815870	4209	6.622923	6.801135	6.763276	6.434494	0.188429	0.318788	0.366641	0.0724	0.328782	0.100718	0.037859	0.836211	-0.17821	0.344134	-0.14035	0.451299	6815870	6.546759	6.616989	6.705021	6.663632	6.978308	6.761465	6.875646	6.901756	6.512426	6.264372	6.224233	6.814876	Ndufaf2	NM_001127346
6816060	4212	8.577645	8.727576	8.68389	8.617629	-0.03998	0.658664	0.109947	0.242747	0.066261	0.46899	0.043686	0.629762	-0.14993	0.123754	-0.10625	0.257636	6816060	8.577998	8.590166	8.564771	8.538272	8.827182	8.817274	8.806432	8.647818	8.59742	8.534837	8.697652	8.620399	Gpbp1	NM_028487
6816080	4213	10.08179	10.0435	9.981634	9.515098	0.56669	0.003262	0.528403	0.004819	0.466536	0.009268	0.061868	0.663435	0.038287	0.786895	0.100155	0.485438	6816080	9.962286	10.22565	10.05743	9.803955	10.22648	10.10007	10.11672	9.846517	9.981665	9.577753	9.646598	9.320944	Map3k1	NM_011945
6816087	4214	7.680833	7.759212	7.833763	8.034079	-0.35325	0.046601	-0.27487	0.104728	-0.20032	0.219166	-0.07455	0.633105	-0.07838	0.616033	-0.15293	0.338537	6816087	7.874413	7.627409	7.540676	7.723002	7.894724	7.659909	8.081207	7.614038	7.806043	7.852118	8.228603	8.021515	Ncf2	NM_010877
6816148	4217	7.711634	7.768233	7.82304	7.632898	0.078736	0.474234	0.135336	0.232877	0.190142	0.107346	-0.05481	0.615369	-0.0566	0.604052	-0.11141	0.319056	6816148	7.690785	7.72938	7.714736	7.585025	7.965301	7.754374	7.955108	7.785735	7.728276	7.489679	7.702033	7.706981	Skiv2l2	NM_028151
6816158	4218	8.970401	8.915356	8.611081	8.405089	0.565312	0.013614	0.510267	0.021725	0.205992	0.284316	0.304275	0.128512	0.055045	0.766944	0.35932	0.080305	6816158	8.927069	9.03661	8.947525	8.691725	9.065686	8.988658	8.744646	8.487438	8.60116	8.024984	8.433952	8.756331	Gpx8	NM_027127
6816183	4221	7.956724	8.169491	8.062755	7.709753	0.246971	0.180173	0.459738	0.025706	0.353002	0.069057	0.106736	0.543388	-0.21277	0.241449	-0.10603	0.545989	6816183	8.169333	7.947943	7.752896	8.201893	8.308484	7.998096	8.284554	8.107058	7.796654	7.489313	7.756693	7.883253	Snx18	NM_130796
6816222	4224	7.133999	7.604611	7.466626	7.31251	-0.17851	0.458944	0.292101	0.23873	0.154116	0.520695	0.137985	0.564225	-0.47061	0.074379	-0.33263	0.185179	6816222	7.009666	7.018143	7.374188	7.546201	7.517842	7.74979	7.589133	7.478125	7.33262	6.946014	7.12256	7.868956	Ndufs4	NM_010887
6816226	4225	6.501948	6.611724	6.43558	6.392819	0.109128	0.471815	0.218905	0.168301	0.04276	0.774852	0.176145	0.257616	-0.10978	0.469274	0.066368	0.65828	6816226	6.482731	6.412149	6.610963	6.704804	6.763684	6.366685	6.586833	6.344977	6.374929	6.428205	6.149452	6.600801	Fst	NM_008046
6816247	4226	7.291939	7.968256	6.630989	6.224903	1.067036	0.057528	1.743353	0.006775	0.406086	0.423503	1.337267	0.024022	-0.67632	0.197743	0.66095	0.207084	6816247	7.572969	7.623616	6.679231	7.599837	8.411043	7.893888	7.111369	5.722535	7.059063	6.13914	6.828986	5.706582	Itga2	NM_008396
6816248	4227	7.212612	7.35735	7.249483	7.111387	0.101225	0.346415	0.245962	0.04115	0.138095	0.20947	0.107867	0.317521	-0.14474	0.190471	-0.03687	0.725015	6816248	7.245312	7.202747	7.189778	7.188317	7.538538	7.345194	7.244246	7.400904	7.103298	7.062579	7.059223	7.21236	Itga1	NM_001033228
6816288	4228	8.100762	8.131343	7.657937	7.502101	0.598662	0.321687	0.629242	0.299146	0.155837	0.790314	0.473405	0.427796	-0.03058	0.958293	0.442825	0.457106	6816288	7.872725	8.242768	8.186794	7.757568	8.204917	8.431543	6.311434	8.650597	8.011781	6.878473	7.941894	7.685935	Isl1	NM_021459
6816317	4229	8.746237	8.482754	8.646586	7.963226	0.783011	0.012232	0.519528	0.065107	0.68336	0.022824	-0.16383	0.51942	0.263483	0.31009	0.099651	0.692672	6816317	8.996812	8.784982	8.456917	8.399121	8.825724	8.223416	8.724899	8.48484	8.730018	7.908421	8.394223	7.587033	Parp8	NM_001081009
6816371	4230	7.275575	7.265921	7.313843	7.407357	-0.13178	0.262334	-0.14144	0.231717	-0.09351	0.41707	-0.04792	0.672631	0.009654	0.931784	-0.03827	0.73526	6816371	7.182002	7.418982	7.225741	7.199938	7.452961	7.144865	7.369676	7.307528	7.264325	7.31777	7.593361	7.310941	Mrps30	NM_021556
6816413	4231	8.923313	8.236688	8.357314	9.099035	-0.17572	0.445765	-0.86235	0.004324	-0.74172	0.009572	-0.12063	0.597018	0.686625	0.013942	0.565998	0.032466	6816413	8.775927	9.154934	8.839077	8.274217	8.132866	8.30298	8.265671	8.507529	8.298743	9.217879	9.498729	8.580497	Nnt	NR_003544
6816703	4233	7.481761	7.810905	7.836077	7.532835	-0.05107	0.72536	0.27807	0.082887	0.303242	0.06273	-0.02517	0.862125	-0.32914	0.047035	-0.35432	0.035558	6816703	7.424151	7.378054	7.643078	7.645084	7.912518	7.875114	7.903095	7.58082	8.024315	7.416538	7.711333	7.470634	Abhd6	NM_025341
6816708	4234	6.828069	7.317557	7.041109	6.880901	-0.05283	0.548776	0.436656	0.000849	0.160208	0.094231	0.276448	0.011265	-0.48949	0.000405	-0.21304	0.035578	6816708	6.711697	6.870172	6.902339	7.24436	7.421653	7.286659	7.188758	6.905592	7.028978	6.85387	6.839218	6.949616	Rpp14	NM_025938
6816714	4236	8.436031	8.549266	8.643979	8.340348	0.095683	0.41456	0.208918	0.097078	0.303631	0.025821	-0.09471	0.419109	-0.11324	0.338317	-0.20795	0.098389	6816714	8.543176	8.341733	8.423183	8.498138	8.773919	8.375742	8.615197	8.588385	8.728354	8.312449	8.481647	8.226948	Pxk	NM_145458
6816720	4237	7.582994	8.017846	8.108643	7.79865	-0.21566	0.224444	0.219195	0.217621	0.309993	0.095058	-0.0908	0.594512	-0.43485	0.02904	-0.52565	0.012441	6816720	7.551465	7.477559	7.719959	8.133731	8.160336	7.75947	8.151561	8.342518	7.831851	7.739221	7.666882	7.989848	Kctd6	NM_027782
6816951	4238	8.485914	8.601761	8.293801	8.555795	-0.06988	0.705349	0.045966	0.803072	-0.26199	0.179927	0.307961	0.122407	-0.11585	0.534081	0.192113	0.312694	6816951	8.649881	8.566781	8.24108	8.727938	8.538024	8.539322	8.417135	8.347229	8.117038	8.279685	8.469584	8.918117	Ptprg	NM_008981
6816967	4239	7.392317	7.465702	7.391233	7.424902	-0.03258	0.692903	0.040799	0.621975	-0.03367	0.683329	0.074469	0.376707	-0.07338	0.383355	0.001085	0.989458	6816967	7.336224	7.438463	7.402265	7.416373	7.401699	7.579033	7.515531	7.335907	7.32226	7.358037	7.355256	7.561414	3830406C	NM_146051
6817053	4242	8.821936	8.755115	8.747753	8.68885	0.133085	0.307387	0.066265	0.602056	0.058902	0.64238	0.007362	0.95339	0.066821	0.599067	0.074183	0.560259	6817053	8.956865	8.786857	8.722085	8.795098	8.875708	8.594539	8.608456	9.01025	8.624552	8.667593	8.740355	8.658603	Atxn7	NM_139227
6817060	4244	8.895678	8.768438	8.737237	8.757333	0.138345	0.042135	0.011105	0.851079	-0.0201	0.734735	0.031201	0.600746	0.12724	0.057027	0.158441	0.024427	6817060	8.994666	8.835296	8.857072	8.773586	8.751685	8.780043	8.748382	8.700375	8.762955	8.777604	8.850204	8.64419	Il3ra	NM_008369
6817136	4245	7.13648	7.156449	7.098081	7.10985	0.02663	0.775497	0.046599	0.619658	-0.01177	0.899493	0.058368	0.536	-0.01997	0.830466	0.038399	0.681772	6817136	7.084666	7.176309	7.148465	7.142071	7.165246	7.16203	7.299017	7.023242	6.971985	6.972979	7.139069	7.217501	Ngly1	NM_021504
6817143	4247	8.758399	8.774531	8.782987	8.832986	-0.07459	0.397814	-0.05846	0.503754	-0.05	0.5659	-0.00846	0.921827	-0.01613	0.851627	-0.02459	0.775903	6817143	8.747136	8.79908	8.72898	8.630848	8.866303	8.826441	8.890634	8.722343	8.735985	8.68985	8.881531	8.927577	Top2b	NM_009409
6817217	4248	6.027342	6.142211	6.092706	6.621808	-0.59447	0.001204	-0.4796	0.00425	-0.5291	0.002429	0.049505	0.694339	-0.11487	0.372083	-0.06536	0.605208	6817217	5.975257	6.168622	5.938147	6.114933	6.278286	6.033414	6.023973	6.012489	6.241655	6.459682	6.848853	6.556888	Thrb	NM_001113417
6817229	4249	7.204492	7.21524	7.342388	7.597208	-0.39272	0.083973	-0.38197	0.091278	-0.25482	0.236375	-0.12715	0.540846	-0.01075	0.958262	-0.1379	0.508084	6817229	7.009145	7.197076	7.407254	7.09411	7.368831	7.18278	7.429371	7.648263	6.949529	7.340684	7.688808	7.762132	Nkiras1	NM_023526
6817287	4250	8.328872	8.595515	7.949117	8.207636	0.121236	0.636202	0.38788	0.154405	-0.25852	0.325147	0.646398	0.030598	-0.26664	0.311112	0.379755	0.162154	6817287	8.572238	8.365495	8.048883	8.441795	8.877226	8.467525	8.322165	7.548141	7.977045	7.870265	8.372484	8.380158	Nid2	NM_008695
6817334	4251	7.584963	7.733998	7.70711	7.671241	-0.08628	0.363899	0.062756	0.503637	0.035869	0.699468	0.026887	0.771821	-0.14903	0.134903	-0.12215	0.210035	6817334	7.53336	7.474993	7.746536	7.76453	7.675056	7.762407	7.671727	7.765923	7.683681	7.500562	7.730556	7.782606	Nudt13	NM_026341
6817337	4252	8.067972	8.140212	8.195335	8.180634	-0.11266	0.107668	-0.04042	0.533966	0.0147	0.819102	-0.05512	0.401336	-0.07224	0.27894	-0.12736	0.074775	6817337	7.969924	8.207529	8.026464	8.104799	8.195837	8.119999	8.160758	8.227565	8.197681	8.11917	8.249752	8.172981	Fam149b	NM_172379

6817365	4253	9.326829	9.237885	9.332817	9.221918	0.104911	0.402149	0.015967	0.896222	0.110899	0.37708	-0.09493	0.446539	0.088944	0.474724	-0.00599	0.960972	6817365	9.308619	9.319372	9.352497	9.199298	9.406387	9.10797	9.287006	9.511652	9.199792	9.157418	9.431568	9.076768	Sec24c	NM_172596
6817366	4254	8.552243	8.518884	8.556846	8.484662	0.067581	0.432889	0.034222	0.686843	0.072183	0.403522	-0.03796	0.655141	0.033359	0.694262	-0.0046	0.956545	6817366	8.540949	8.625598	8.490183	8.434176	8.615991	8.506486	8.612473	8.682533	8.375528	8.466137	8.46246	8.525389	Fut11	NM_028428
6817367	4255	6.79365	7.348684	7.028905	7.065239	-0.27159	0.223005	0.283444	0.205291	-0.03633	0.864103	0.319779	0.158437	-0.55503	0.02708	-0.23526	0.28556	6817367	6.832902	7.094168	6.453879	7.372026	7.383343	7.290682	7.18602	7.286712	6.613982	6.9864	7.209814	6.999504	Chchd1	NM_025366
6817368	4256	8.617712	8.737863	8.603105	8.448982	0.16873	0.169916	0.288882	0.032499	0.154124	0.205602	0.134758	0.262783	-0.12015	0.314133	0.014607	0.89934	6817368	8.727824	8.630296	8.495016	8.695376	8.807146	8.711068	8.434177	8.873375	8.501764	8.441679	8.494968	8.410298	2310021P	NM_027996
6817381	4257	7.648919	7.517036	7.619222	6.181135	1.467784	0.003706	1.335901	0.006199	1.438086	0.004154	-0.10219	0.785354	0.131883	0.725639	0.029697	0.936772	6817381	8.060781	7.578507	7.307469	7.293464	8.11389	7.143755	7.928603	7.144212	7.78485	5.718967	6.603991	6.220448	Plau	NM_008873
6817393	4258	9.781489	9.540137	9.691129	9.198421	0.583069	0.016433	0.341716	0.114187	0.492708	0.033845	-0.15099	0.455977	0.241353	0.245877	0.090361	0.651722	6817393	9.816244	9.836294	9.69193	9.41951	9.761604	9.439296	9.834462	9.441247	9.797677	9.214529	9.554439	8.826294	Vcl	NM_009502
6817396	4259	8.723334	8.745887	8.829495	8.752866	-0.02953	0.876826	-0.00698	0.970761	0.07663	0.688878	-0.08361	0.662542	-0.02255	0.905745	-0.10616	0.580924	6817396	8.459572	8.838222	8.872207	8.780071	8.542286	8.915304	9.038601	8.544456	8.905429	8.64712	9.011775	8.599702	Adk	NM_134079
6817403	4260	8.444033	8.524427	8.409137	8.205287	0.238746	0.01293	0.31914	0.002779	0.20385	0.026324	0.11529	0.162778	-0.08039	0.314999	0.034896	0.654113	6817403	8.526638	8.466833	8.338629	8.522549	8.487409	8.563324	8.307949	8.489463	8.429999	8.282478	8.266912	8.066472	Myst4	NM_017479
6817412	4261	7.721119	7.764145	7.714464	7.620275	0.100844	0.376613	0.14387	0.218459	0.094188	0.407422	0.049681	0.65695	-0.04303	0.70005	0.006655	0.952254	6817412	7.771419	7.720672	7.671266	7.754028	7.8601	7.678307	7.852353	7.756388	7.53465	7.413345	7.712939	7.734542	Samd8	NM_026283
6817416	4262	10.45212	10.53612	10.55849	10.73804	-0.28592	0.08614	-0.20192	0.204436	-0.17955	0.254141	-0.02237	0.882137	-0.08399	0.581279	-0.10636	0.487496	6817416	10.37998	10.44307	10.53332	10.5861	10.5376	10.48465	10.64929	10.64552	10.38065	10.63516	10.49287	11.08609	Vdac2	NM_011695
6817611	4264	10.00549	9.876846	10.0741	9.829251	0.17624	0.40123	0.047595	0.816817	0.244847	0.25309	-0.19725	0.35018	0.128645	0.53571	-0.06861	0.738927	6817611	10.16173	10.02176	9.832983	10.00933	10.00695	9.614259	9.774734	10.50727	9.940291	9.730633	9.936447	9.820674	Zmiz1	NM_183208
6817616	4265	6.340181	6.126304	6.06288	6.367492	-0.02731	0.938628	-0.24119	0.50282	-0.30461	0.40141	0.063424	0.858213	0.213876	0.551152	0.2773	0.44318	6817616	6.731994	6.398589	5.889959	6.272983	6.265044	5.840886	5.316762	6.649967	6.221912	6.330513	6.4473	6.324662	4931406H	NM_175264
6817617	4266	7.902163	8.077284	7.846537	8.351441	-0.44928	0.016123	-0.27416	0.100921	-0.5049	0.009173	0.230747	0.157362	-0.17512	0.2704	0.055626	0.716628	6817617	7.878992	7.759837	8.067661	8.163586	8.047896	8.02037	7.661289	8.131385	7.746937	8.142934	8.377605	8.533784	Ppif	NM_134084
6817637	4267	10.07976	10.2657	10.20427	10.52344	-0.44368	0.014279	-0.25775	0.107705	-0.31917	0.055187	0.061427	0.677406	-0.18594	0.227679	-0.12451	0.407125	6817637	10.0536	10.06362	10.12206	10.38637	10.21176	10.19896	10.12871	10.45583	10.02827	10.34182	10.42861	10.7999	Anxa11	NM_013469
6817668	4270	8.311098	8.463352	8.255425	8.314055	-0.00296	0.978176	0.149297	0.19204	-0.05863	0.591119	0.207927	0.082496	-0.15225	0.184292	0.055673	0.609639	6817668	8.400282	8.377664	8.155348	8.368761	8.506952	8.514344	8.361142	8.266964	8.138169	8.173656	8.267974	8.500536	Fam116a	NM_001134465
6817672	4271	11.05593	10.97073	11.14265	11.11913	-0.0632	0.563795	-0.14841	0.195135	0.023517	0.828349	-0.17192	0.140093	0.085207	0.440434	-0.08672	0.432689	6817672	11.11574	11.02372	11.02834	10.88248	11.14807	10.88163	11.04321	11.34077	11.04397	11.0717	11.23618	11.04952	Arf4	NM_007479
6817694	4274	6.806343	7.087681	6.486285	6.341585	0.464758	0.014085	0.746096	0.001028	0.1447	0.358823	0.601396	0.003705	-0.28134	0.095025	0.320058	0.063456	6817694	6.839317	6.998242	6.58147	6.989185	7.285294	6.988565	6.607105	6.214121	6.63763	6.389966	6.356043	6.278746	Il17rd	NM_134437
6817700	4276	8.562489	8.137565	8.65631	7.921505	0.640984	0.011888	0.21606	0.306653	0.734804	0.005926	-0.51874	0.030572	0.424924	0.064024	-0.09382	0.648063	6817700	8.858113	8.430691	8.398664	8.080865	8.322082	8.009748	8.955125	8.318638	8.695166	7.752801	8.094429	7.967236	Arhgef3	NM_027871
6817763	4278	6.863171	6.793652	6.623861	6.411005	0.452166	0.137394	0.382646	0.199958	0.212856	0.459464	0.16979	0.552587	0.069519	0.806051	0.23931	0.407758	6817763	6.982168	6.523333	7.084012	6.591409	7.382171	6.407375	6.713307	6.69703	6.461247	6.718241	6.212288	6.302487	Wnt5a	NM_009524
6817900	4280	6.582963	6.771594	6.869016	6.96078	-0.37782	0.082808	-0.18919	0.35007	-0.09176	0.643154	-0.09742	0.623127	-0.18863	0.351407	-0.28605	0.171872	6817900	6.65231	6.367461	6.729119	6.687894	6.693484	6.933404	6.900397	6.4635	7.243151	6.985437	7.043021	6.853881	Selk	NM_019979
6817902	4281	8.077723	8.232376	8.128648	8.173028	-0.0953	0.261576	0.059348	0.473484	-0.04438	0.589201	0.103728	0.225057	-0.15465	0.085645	-0.05093	0.536715	6817902	7.97603	8.044942	8.212197	8.155359	8.281113	8.260656	8.208109	8.196106	7.981729	8.132919	8.168236	8.217928	Actr8	NM_027493
6817903	4282	9.072104	9.124696	8.96266	8.980242	0.091862	0.685607	0.144454	0.527612	-0.01758	0.937918	0.162036	0.480067	-0.05259	0.816069	0.109445	0.630353	6817903	8.915171	8.93836	9.362782	9.169353	9.177079	9.027656	9.122477	9.029828	8.735674	8.761059	8.715985	9.463682	Chdh	NM_175343
6817926	4283	7.002088	6.938971	7.020987	6.979997	0.022091	0.711233	-0.04103	0.496423	0.040991	0.49678	-0.08202	0.192165	0.063117	0.304922	-0.0189	0.751174	6817926	7.046112	6.970939	6.989213	6.894544	7.056439	6.86593	6.948391	7.096684	7.017887	6.971767	7.031521	6.936702	Dcp1a	NM_133761
6817930	4284	9.440456	9.620552	9.177169	8.96695	0.473505	0.033236	0.653602	0.007569	0.210219	0.287246	0.443383	0.042873	-0.1801	0.357329	0.263286	0.191188	6817930	9.530846	9.216719	9.573802	9.572807	9.680779	9.60807	9.610118	8.978436	8.942954	8.871041	9.138092	8.891718	Tkt	NM_009388
6817940	4285	7.48719	7.461404	7.399203	7.393661	0.093528	0.150443	0.067742	0.282655	0.005542	0.927244	0.0622	0.321133	0.025786	0.672669	0.087986	0.173016	6817940	7.445183	7.593111	7.423275	7.535839	7.484797	7.363575	7.417102	7.450841	7.329667	7.366882	7.406894	7.405708	Rft1	NM_177815
6817944	4286	7.345833	7.522514	7.455143	7.36897	-0.02314	0.838065	0.153544	0.198757	0.086173	0.454324	0.067371	0.555784	-0.17668	0.145573	-0.10931	0.347744	6817944	7.44329	7.29294	7.30127	7.530484	7.628698	7.40836	7.561362	7.580197	7.223871	7.322109	7.288099	7.496703	Sfmbt1	NM_019460
6817951	4287	5.627336	6.221169	6.026214	5.806914	-0.17958	0.319255	0.414125	0.039999	0.2193	0.230827	0.194825	0.282532	-0.5937	0.00795	-0.39888	0.046042	6817951	5.940387	5.452743	5.488877	6.155854	6.139507	6.367756	6.031737	6.015767	6.031139	5.501456	5.852548	6.066738	Sfmbt1	ENSMUST000000
6817952	4288	7.126674	7.279416	7.238706	7.085229	0.041445	0.618962	0.194187	0.041614	0.153478	0.091745	0.04071	0.625099	-0.15274	0.093052	-0.11203	0.19958	6817952	7.14481	7.030645	7.204566	7.28211	7.396585	7.159553	7.317472	7.263705	7.134942	6.982961	7.132429	7.140296	Tmem110	NM_028839
6817954	4289	8.724244	8.381661	8.355179	8.206062	0.518182	0.14776	0.175598	0.601944	0.149117	0.657012	0.026482	0.93675	0.342583	0.32039	0.369065	0.286795	6817954	8.421829	8.599933	9.15097	8.777713	8.47391	7.893359	8.637404	8.389789	8.038344	7.961132	8.709993	7.947062	Mustn1	NM_181390
6817956	4291	7.436819	7.573208	7.517842	7.319916	0.116903	0.0748	0.253292	0.002179	0.197927	0.008486	0.055365	0.360603	-0.13639	0.043941	-0.08102	0.193645	6817956	7.399809	7.478696	7.431952	7.588913	7.592043	7.538667	7.508912	7.479039	7.565576	7.275954	7.458976	7.224817	Nek4	NM_011849
6817958	4292	8.235513	8.33062	8.394095	8.294946	-0.05943	0.614502	0.035674	0.761181	0.099149	0.407531	-0.06347	0.591069	-0.09511	0.426104	-0.15858	0.199634	6817958	8.20419	8.24027	8.26208	8.176175	8.402117	8.413568	8.470281	8.527479	8.184524	8.297335	8.450773	8.13673	Glt8d1	NM_029626
6817960	4293	9.088501	9.16305	9.143081	8.975486	0.113015	0.295715	0.187563	0.100438	0.167594	0.135694	0.019969	0.84823	-0.07455	0.481621	-0.05458	0.60371	6817960	9.101686	9.094567	9.06925	9.174629	9.259573	9.054947	9.356009	9.043348	9.029885	8.895042	9.122797	8.90862	Pbrm1	NM_001081251
6817966	4294	7.927985	8.053105	7.914424	7.935771	-0.00779	0.892673	0.117333	0.069066	-0.02135	0.712511	0.138681	0.038073	-0.12512	0.055592	0.013561	0.814436	6817966	7.937811	7.956294	7.88985	8.024372	8.094719	8.040223	8.006799	7.841301	7.895171	7.826691	7.977459	8.003164	Pbrm1	NM_001081251
6817970	4295	7.855607	8.231772	7.970962	8.597799	-0.74219	0.085417	-0.36603	0.361624	-0.62684	0.136129	0.260809	0.510074	-0.37617	0.349192	-0.11536	0.768211	6817970	7.766641	7.835438	7.964741	8.122963	8.533627	8.038725	7.463389	8.523846	7.925652	8.215984	8.167014	9.410398	Nt5dc2	NM_027289
6817984	4297	7.552533	7.61655	7.532051	7.536385	0.016148	0.875442	0.080165	0.444926	-0.00433	0.966417	0.084499	0.421665	-0.06402	0.539069	0.020482	0.84248	6817984	7.476552	7.544508	7.636538	7.537106	7.812126	7.500418	7.657704	7.542768	7.39568	7.47487	7.502554	7.63173	Bap1	NM_027088
6817989	4298	8.531569	8.606889	8.53735	8.622921	-0.09135	0.31989	-0.01603	0.85699	-0.08557	0.349618	0.069539	0.442851	-0.07532	0.407399	-0.00578	0.948147	6817989	8.555759	8.522361	8.516588	8.493128	8.681297	8.646242	8.636937	8.632594	8.342518	8.540634	8.641498	8.686631	Capn7	NM_009796
6818017	4299	8.235835	8.232251	8.22949	8.15866	0.077175	0.200536	0.073591	0.220126	0.07083	0.236319	0.002761	0.961416	0.003584	0.949942	0.006345	0.911519	6818017	8.264402	8.244533	8.19857	8.268269	8.284758	8.143727	8.269788	8.298062	8.12062	8.158819	8.111789	8.205372	Eaf1	NM_028932
6818029	4300	7.739524	7.688319	7.634359	7.686332	0.053192	0.739397	0.001987	0.990049	-0.05197	0.745118	0.05396	0.7358	0.051205	0.748733	0.105165	0.515106	6818029	7.747353	7.823658	7.647561	7.472692	7.949786	7.642479	7.820604	7.321514	7.760958	7.744312	7.658646	7.656038	Btd	NM_025295
6818051	4302	6.977812	6.788449	7.08397	7.134525	-0.15671	0.2574	-0.34608	0.027369	-0.05055	0.704304	-0.29552	0.050502	0.189364	0.17883	-0.10616	0.43271	6818051	7.073301	6.956185	6.903921	7.014593	6.68681	6.663943	7.04861	7.314268	6.889031	7.15544	7.205328	7.042806	Galntl2	NM_030166
6818053	4303	8.104581	8.154552	8.095431	8.533676	-0.4291	0.185395	-0.37912	0.236349	-0.43825	0.177188	0.059121	0.846749	-0.04997	0.870193	0.00915	0.976109	6818053	8.005054	7.996049	8.31264	8.325301	7.897588	8.240766	7.809742	8.514376	7.962175	8.29761	8.138696	9.164723	Oxnad1	NM_145460
6818064	4305	6.201196	6.558356	6.221663	5.561814	0.639382	0.045697	0.996542	0.006183	0.659849	0.040608	0.336693	0.248476	-0.35716	0.223244	-0.02047	0.941545	6818064	6.102664	6.348074	6.152849	6.517546	6.543089	6.614433	6.171683	6.432326	6.06098	5.907282	4.847	5.93116	Ncoa4	NM_001033988
6818067	4306	7.987375	8.132776	7.961748	7.880132	0.107243	0.103537	0.252644	0.002521	0.081615	0.199674	0.171028	0.019014	-0.1454	0.037494	0.025627	0.672307	6818067	8.047708	7.868248	8.046169	8.065933	8.184351	8.148044	7.995252	8.004938	7.885053	7.913054	7.893313	7.83403	Parg	NM_011960
6818088	4307	6.599865	6.586549	6.615048	6.480743	0.119122	0.409312	0.105805	0.461571	0.134305	0.355039	-0.0285	0.8402	0.013317	0.924859	-0.01518	0.914373	6818088	6.809245	6.430066	6.560285	6.457414	6.533271	6.768961	6.723463	6.536753	6.584929	6.681514	6.284556	6.47616	Prrxl1	NM_001001796
6818104	4308	7.20872	7.144475	6.952424	7.15275	0.05597	0.693911	-0.00827	0.953373	-0.20033	0.182252	0.192051	0.198998	0.064245	0.651982	0.256296	0.098604	6818104	7.328648	7.159482	7.13803	7.141403	7.167897	7.124126	7.102388	6.636848	7.118037	6.990251	7.150821	7.317177	E130203B	NM_178791
6818118	4310	7.403989	7.199531	7.241421	7.191979	0.21201	0.05329	0.007553	0.937667	0.049443	0.611654	-0.04189	0.666338	0.204458	0.060438	0.162568	0.120607	6818118	7.578443	7.394415	7.239109	7.335064	7.145423	7.118107	7.248988	7.265558	7.209718	7.129164	7.146187	7.300585	Arhgap22	NM_153800
6818153	4312	6.708389	6.655018	6.206231	6.197855	0.510534	0.083932	0.457163	0.115231	0.008376	0.974968	0.448787	0.121049	0.053371	0.841729	0.502158	0.088239	6818153	6.972981	6.498214	6.653973	6.661781	7.075154	6.228119	6.355544	6.112347	6.150802	6.037893	5.920875	6.634797	Gdf10	NM_145741
6818186	4316	6.019755	6.889914	5.949261	6.480545	-0.46079	0.38496	0.409369	0.437872	-0.53128	0.320263	0.940653	0.097493	-0.87016	0.120873	0.070494	0.891665	6818186	5.710572	6.497799	5.850895	6.51748	7.800133	6.352129	6.52982	5.605272	5.712691	6.653205	7.046773	5.741657	Anxa8	NM_013473
6818203	4317	8.005323	7.706025	7.698429	6.917051	1.088272	0.006727	0.788973	0.030247	0.781377	0.03146	0.007596	0.98043	0.299298	0.347899	0.306894	0.336491	6818203	8.615121	7.739616	7.661232	7.716393	7.884356	7.517325	7.854928	7.419041	7.821317	6.519024	7.336832	6.895298	Syt15	NM_181529
6818212	4318	8.926311	8.962498	8.895574	8.648428	0.277883	0.088249	0.31407	0.059601	0.247146	0.122616	0.066924	0.652688	-0.03619	0.806855	0.030737	0.835398	6818212	8.94267	8.91412	8.922144	8.935969	9.10139	8.850134	9.128067	8.749949	8.808706	8.358147	8.837003	8.750134	Glud1	NM_008133
6818213	4319	8.283522	8.390543	8.361923	8.157586	0.125936	0.500714	0.232957	0.228359	0.204337	0.285627	0.02862	0.876651	-0.10702	0.565579	-0.0784	0.672274	6818213	8.422059	8.454469	7.974039	8.403996	8.403493	8.364141	8.361319	8.217629	8.506822	7.797007	8.355791	8.319961	Mmrn2	NM_153127
6818240	4320	8.485853	8.54837	8.462342	8.474885	0.010968	0.876488	0.073485	0.313661	-0.01254	0.858957	0.086029	0.243637	-0.06252	0.387098	0.023511	0.739721	6818240	8.495262	8.492754	8.469543	8.472656	8.679358	8.493097	8.439598	8.566504	8.380923	8.386567	8.516308	8.52178	Wapal	NM_001004436
6818499	4327	7.813495	6.573114	7.007575	5.952162	1.861333	0.028327	0.620952	0.398935	1.055413	0.168387	-0.43446	0.550375	1.240382	0.112984	0.80592	0.280879	6818499	8.644043	6.902704	7.893739	6.900134	6.529213	6.289994	6.83189	5.713509	8.477327	6.327937	5.629957	5.898592	Sftpa1	NM_023134
6818523	4329	6.08796	5.959092	5.814677	5.990631	0.09733	0.453312	-0.03154	0.804859	-0.17595	0.192015	0.144415	0.275873	0.128868	0.327188	0.273283	0.057805	6818523	5.916702	6.170873	6.176306	5.891837	6.057441	5.927999	5.88819	5.891889	5.663952	6.134954	6.08948	5.747458	Ptger2	NM_008964
6818553	4330	7.197653	7.251985	7.336131	7.308795	-0.11114	0.514833	-0.05681	0.736593	0.027336	0.871055	-0.08415	0.619865	-0.05433	0.747604	-0.13848	0.420538	6818553	7.328886	7.0042	7.259873	7.316479	7.358325	7.081152	7.366486	7.636904	7.005004	7.390425	7.217862	7.318099	Gpr137c	NM_027518
6818556	4331	9.110359	9.164931	9.188467	8.990629	0.11973	0.366831	0.174302	0.201266	0.197838	0.152652	-0.02354	0.855538	-0.05457	0.674389	-0.07811	0.550009	6818556	9.067445	9.195066	9.068566	9.019364	9.293962	9.181467	9.404051	9.077164	9.084187	8.780646	9.136076	9.055166	Psmc6	NM_025959
6818665	4334	6.681391	6.902866	6.691168	6.929176	-0.24778	0.153669	-0.02631	0.87125	-0.23801	0.168524	0.211698	0.215044	-0.22147	0.196588	-0.00978	0.951943	6818665	6.586887	6.688407	6.76888	6.887663	7.140799	6.680135	6.70217	6.901729	6.469604	6.773605	6.659173	7.15475	Cgrrf1	NM_026832
6818667	4335	7.119374	7.307243	6.88895	6.774227	0.345148	0.05392	0.533016	0.00824	0.114723	0.474494	0.418293	0.025603	-0.18787	0.25404	0.230425	0.170178	6818667	7.106793	7.037165	7.214165	7.376579	7.480819	7.06433	7.209803	6.72655	6.730496	6.691959	6.760406	6.870315	Samd4	NM_001037221
6818686	4336	7.468251	7.565016	7.471668	7.699495	-0.23124	0.160216	-0.13448	0.394294	-0.22783	0.165742	0.093348	0.549448	-0.09677	0.535281	-0.00342	0.982309	6818686	7.641968	7.49323	7.269554	7.333277	7.840365	7.521407	7.498478	7.609916	7.30661	7.604528	7.799323	7.694634	Socs4	NM_080843
6818687	4337	11.19486	11.10727	11.24534	11.1516	0.04326	0.754384	-0.04433	0.748569	0.09374	0.502786	-0.13807	0.331616	0.087587	0.530475	-0.05048	0.715367	6818687	11.19972	11.18236	11.2025	11.09053	11.33121	10.90008	11.01571	11.46879	11.25152	11.0758	11.25756	11.12144	Mapk1ip1	NM_178684
6818696	4338	9.827214	10.08032	9.691159	8.82863	0.998584	0.026488	1.251693	0.009332	0.862529	0.047095	0.389164	0.321063	-0.25311	0.510936	0.136055	0.721132	6818696	9.862509	9.884295	9.734838	10.06472	10.36973	9.806519	10.29467	8.96835	9.810456	9.076531	9.183884	8.225474	Lgals3	NM_001145953
6818707	4339	7.900534	8.009739	8.074355	8.099482	-0.19895	0.038427	-0.08974	0.296691	-0.02513	0.762608	-0.06462	0.444761	-0.10921	0.211399	-0.17382	0.062578	6818707	7.961325	7.819345	7.920931	8.08164	8.018135	7.929442	7.918854	8.202269	8.101942	8.136163	8.004193	8.158091	Fbxo34	NR_027414

6818722	4340	8.753527	8.654809	8.620938	8.54625	0.207276	0.033671	0.108559	0.216889	0.074688	0.383354	0.033871	0.686759	0.098717	0.257562	0.132589	0.140209	6818722	8.780762	8.751717	8.728101	8.636806	8.66895	8.658672	8.740878	8.477025	8.644911	8.406709	8.538928	8.693114	Ktn1	NM_008477
6818742	4341	7.779332	8.024521	7.798126	7.893296	-0.11396	0.399331	0.131225	0.335335	-0.09517	0.478497	0.226395	0.114957	-0.24519	0.091784	-0.01879	0.886917	6818742	7.86151	7.898822	7.577665	8.214101	7.951946	7.907517	7.735453	7.907245	7.751681	7.881814	7.723143	8.074932	Peli2	NM_033602
6818792	4342	7.709177	8.266262	7.836136	7.896643	-0.18747	0.497444	0.36962	0.198696	-0.06051	0.824316	0.430126	0.141594	-0.55709	0.067656	-0.12696	0.643168	6818792	7.406859	7.749351	7.971321	8.180894	8.306143	8.31175	8.378927	7.45098	7.678501	7.658741	7.781642	8.249545	6720456H	NM_172600
6818858	4344	6.996405	7.24058	7.191105	7.14047	-0.14406	0.134225	0.10011	0.280312	0.050636	0.574255	0.049475	0.582889	-0.24418	0.022351	-0.1947	0.054406	6818858	6.918006	7.032401	7.038808	7.131589	7.347963	7.242188	7.226351	7.068589	7.278376	7.046799	7.08725	7.28736	Mudeng	NM_144535
6818863	4345	7.740221	7.969579	7.797016	8.103095	-0.36287	0.010325	-0.13352	0.254823	-0.30608	0.02277	0.172563	0.151533	-0.22936	0.068168	-0.0568	0.615942	6818863	7.67424	7.705271	7.841151	7.858897	7.993464	8.056376	7.836	7.893738	7.66131	8.020424	7.962076	8.326784	Nat12	NM_001081430
6818939	4354	7.952489	8.016381	8.049054	7.872496	0.079993	0.329481	0.143885	0.098764	0.176558	0.051125	-0.03267	0.682664	-0.06389	0.43097	-0.09656	0.245441	6818939	7.921591	7.919987	8.01589	7.968196	8.191817	7.88913	8.134452	7.961372	8.051339	7.88412	7.847822	7.885546	Parp2	NM_009632
6818944	4355	6.583151	6.980742	6.64743	6.356688	0.226463	0.327236	0.624054	0.020656	0.290742	0.217169	0.333312	0.163141	-0.39759	0.104323	-0.06428	0.774639	6818944	6.6963	6.290348	6.762804	6.91959	7.195148	6.827487	7.012415	6.292644	6.63723	6.280584	6.178711	6.610768	Pnp1	ENSMUST000000
6818950	4356	8.086417	8.273804	8.206939	7.886061	0.200356	0.290102	0.387742	0.059708	0.320877	0.107194	0.066865	0.715225	-0.18739	0.320319	-0.12052	0.514841	6818950	8.03342	8.07023	8.155602	8.131704	8.501719	8.187988	8.553785	8.004039	8.062992	7.62418	7.978541	8.055463	Apex1	NM_009687
6818956	4358	9.638634	9.861464	9.635219	9.094956	0.543678	0.076988	0.766508	0.021137	0.540263	0.078525	0.226245	0.423029	-0.22283	0.429779	0.003415	0.990144	6818956	9.818941	9.418463	9.678499	9.802623	10.0578	9.723969	10.16255	9.21472	9.528388	8.910581	9.504069	8.870218	Ang	NM_007447
6819032	4365	7.8223	7.738427	7.606811	7.259977	0.562323	0.013707	0.47845	0.028114	0.346834	0.088397	0.131615	0.482726	0.083873	0.651552	0.215488	0.262603	6819032	7.921695	7.821763	7.723441	7.633927	7.994216	7.587137	7.406112	7.813272	7.60105	7.181248	7.593086	7.005597	E130112L	NM_198249
6819046	4366	5.764569	6.451724	5.949541	5.677706	0.086863	0.792232	0.774019	0.041391	0.271835	0.418786	0.502183	0.153978	-0.68716	0.063311	-0.18497	0.577873	6819046	5.951192	5.369565	5.97295	6.285686	6.355954	6.713533	6.224429	5.337931	6.286263	5.473999	5.422044	6.137074	Rpgrip1	NM_023879
6819075	4368	5.909523	6.04698	6.093812	5.866099	0.043424	0.655858	0.18088	0.090049	0.227713	0.041413	-0.04683	0.631146	-0.13746	0.181119	-0.18429	0.085135	6819075	6.052665	5.789799	5.886106	5.957249	6.210621	5.973069	6.167127	5.958881	6.155429	5.897172	5.874747	5.826379	Tcra	AB183189
6819143	4371	10.76741	10.43194	10.594	10.66455	0.10286	0.364079	-0.23261	0.061227	-0.07055	0.527779	-0.16206	0.167954	0.33547	0.013837	0.17341	0.143391	6819143	10.791	10.82551	10.68573	10.20732	10.67674	10.41177	10.61878	10.5009	10.66233	10.66935	10.69155	10.63276	Abhd4	NM_134076
6819147	4372	9.801397	9.935735	9.867875	10.06252	-0.26113	0.143666	-0.12679	0.453916	-0.19465	0.261414	0.06786	0.684649	-0.13434	0.4285	-0.06648	0.690671	6819147	9.66946	9.845387	9.889343	9.88026	10.01899	9.907955	10.06187	9.586349	9.955406	9.908128	9.901799	10.37764	Oxa1l	NM_026936
6819152	4373	9.520066	9.531664	9.397213	9.502608	0.017458	0.888141	0.029056	0.815118	-0.10539	0.406263	0.134451	0.295911	-0.0116	0.925526	0.122853	0.336782	6819152	9.49794	9.478155	9.584104	9.477304	9.65832	9.459369	9.41956	9.627035	9.145045	9.3712	9.575137	9.561487	Mrpl52	NM_026851
6819153	4374	9.115174	9.439402	8.833156	8.289534	0.82564	0.036365	1.149868	0.008129	0.543622	0.13699	0.606247	0.102555	-0.32423	0.353141	0.282018	0.416174	6819153	9.374243	9.053461	8.917817	9.039439	10.01832	9.260448	9.131358	8.440413	8.927696	8.044779	8.81149	8.012333	Mmp14	NM_008608
6819154	4375	9.81083	9.675139	9.583493	9.760531	0.050299	0.660148	-0.08539	0.460605	-0.17704	0.146753	0.091646	0.429643	0.135691	0.25308	0.227337	0.072975	6819154	10.01266	9.767711	9.65212	9.694617	9.718483	9.612318	9.624744	9.674866	9.450869	9.593706	9.810084	9.877804	Lrp10	NM_022993
6819156	4376	5.941271	5.754805	6.136012	7.29314	-1.35187	0.000676	-1.53833	0.000289	-1.15713	0.00178	-0.18621	0.168983	0.186466	0.480683	-0.19474	0.462077	6819156	5.766573	5.662755	6.171685	5.885556	5.729008	5.625338	5.910069	6.018624	6.401286	5.988126	7.732534	7.41906	6.727825	Rem2	NM_080726
6819177	4378	6.945597	6.982318	7.096051	7.06794	-0.12234	0.311917	-0.08562	0.471688	0.028111	0.810387	-0.11373	0.345073	-0.03672	0.754286	-0.15045	0.221039	6819177	6.785384	7.00458	7.046828	6.749102	7.14555	7.052302	7.151538	7.152051	6.984565	7.001774	7.058293	7.143754	Acin1	NM_023190
6819197	4379	7.080203	7.071657	7.018161	7.137445	-0.05724	0.390122	-0.06579	0.3269	-0.11928	0.094936	0.053495	0.420501	0.008547	0.895443	0.062042	0.35358	6819197	7.066016	7.078424	7.09617	7.018858	7.047041	7.149071	7.154744	6.988703	6.911037	7.079288	7.195789	7.137259	Bcl2l2	NM_007537
6819200	4380	9.345174	9.423765	9.287531	9.259868	0.085258	0.361453	0.163897	0.099837	0.027662	0.76153	0.136235	0.160551	-0.07864	0.398058	0.057596	0.531553	6819200	9.372371	9.369466	9.293542	9.40078	9.485566	9.38495	9.487958	9.28767	9.086964	9.220921	9.262162	9.296522	Pabpn1	NM_019402
6819203	4381	5.890389	5.928472	5.825202	6.018127	-0.12774	0.467886	-0.08966	0.607279	-0.19293	0.28298	0.10327	0.554949	-0.03808	0.825967	0.065187	0.707497	6819203	5.773741	5.979439	5.917987	5.738787	6.039382	6.007247	5.689521	6.199714	5.586371	6.056013	5.853399	6.144969	Il25	NM_080729
6819209	4382	7.805738	7.83246	7.845956	7.763263	0.042474	0.664958	0.069197	0.484826	0.082693	0.406949	-0.0135	0.889944	-0.02672	0.78448	-0.04022	0.681557	6819209	7.808719	7.803885	7.804609	7.742041	7.944909	7.810431	7.766891	8.068958	7.70202	7.685417	7.800083	7.80429	Ngdn	NM_026890
6819214	4383	6.130659	6.263489	6.312885	6.198994	-0.06833	0.62877	0.064495	0.647905	0.113891	0.426511	-0.0494	0.725775	-0.13283	0.35718	-0.18223	0.216953	6819214	6.020353	6.076644	6.294981	6.361346	6.215043	6.214077	6.289487	6.131746	6.517421	6.076169	6.076651	6.444161	Thtpa	NM_153083
6819241	4385	8.227566	8.559825	8.267038	8.342385	-0.11482	0.42071	0.21744	0.146666	-0.07535	0.592801	0.292787	0.06238	-0.33226	0.039567	-0.03947	0.777887	6819241	8.182656	8.101623	8.398419	8.480969	8.672046	8.526461	8.510905	8.167808	8.122402	8.199697	8.287519	8.53994	Dhrs4	NM_030686
6819244	4386	6.230078	6.225728	6.229208	6.281465	-0.05139	0.342228	-0.05574	0.305339	-0.05226	0.334582	-0.00348	0.947171	0.00435	0.933985	0.000871	0.986771	6819244	6.278764	6.210853	6.200618	6.270942	6.125568	6.280674	6.19834	6.255536	6.233747	6.330525	6.197331	6.31654	Cpne6	NM_001136057
6819247	4387	7.508901	7.783093	7.43457	7.478972	0.02993	0.814169	0.304122	0.0388	-0.0444	0.727877	0.348523	0.022187	-0.27419	0.056687	0.074331	0.562995	6819247	7.512989	7.45197	7.561745	7.739625	7.848394	7.761261	7.568327	7.128061	7.607322	7.434041	7.390196	7.612678	Pck2	NM_028994
6819249	4388	10.02415	10.19546	9.99642	10.10725	-0.0831	0.609703	0.088208	0.58828	-0.11083	0.498758	0.199036	0.238968	-0.1713	0.305343	0.027733	0.863687	6819249	9.870652	9.975508	10.2263	10.19328	10.11684	10.27625	10.10822	10.04757	9.833471	9.952083	9.923732	10.44593	Wdr23	NM_133734
6819250	4389	6.340419	6.446594	5.952263	6.016082	0.324336	0.800141	0.430512	0.737245	-0.06382	0.96019	0.494331	0.700394	-0.10618	0.933826	0.388155	0.762115	6819250	4.212192	5.927467	8.881497	7.660728	6.455436	5.223618	7.165064	5.630531	5.061195	4.905384	6.77346	6.369403	Fitm1	NM_026808
6819252	4390	10.98497	11.21444	11.05335	10.80717	0.177793	0.193271	0.40727	0.011637	0.246177	0.084774	0.161093	0.234088	-0.22948	0.10411	-0.06838	0.599749	6819252	10.96121	10.86202	11.13167	11.21486	11.36932	11.05915	11.29694	10.89763	10.96548	10.7218	10.83451	10.86521	Psme1	NM_011189
6819257	4391	7.824912	7.710075	7.72354	7.573218	0.251694	0.047432	0.136858	0.239017	0.150322	0.199826	-0.01346	0.903478	0.114836	0.316884	0.101372	0.373589	6819257	7.784246	7.835271	7.855218	7.726961	7.893631	7.509634	7.602346	7.924654	7.64362	7.554958	7.583745	7.58095	Rnf31	NM_194346
6819258	4392	8.753456	8.690818	8.634761	8.225049	0.528406	0.00599	0.465769	0.011411	0.409712	0.020713	0.056057	0.704461	0.062637	0.672051	0.118695	0.429277	6819258	8.607897	8.813622	8.838848	8.515738	8.907066	8.649651	8.768063	8.339433	8.796787	8.218925	8.201382	8.254841	Irf9	NM_001159417
6819259	4393	7.70894	7.174341	7.002787	8.047593	-0.33865	0.57016	-0.87325	0.165355	-1.04481	0.105182	0.171554	0.77188	0.534599	0.377329	0.706153	0.252028	6819259	6.908486	8.140703	8.077631	6.491385	7.775152	7.256486	6.049684	8.056117	6.902559	8.037254	7.828321	8.277205	Rec8	NM_020002
6819269	4395	7.135866	7.331541	7.311911	7.24128	-0.10541	0.163656	0.090261	0.225527	0.070632	0.334195	0.019629	0.78245	-0.19567	0.021583	-0.17605	0.03358	6819269	7.138923	7.201497	7.067178	7.193517	7.389097	7.412008	7.336491	7.338072	7.261171	7.180568	7.340288	7.202983	Gmpr2	NM_177992
6819274	4396	7.010137	7.126536	7.014554	6.879566	0.130572	0.188885	0.24697	0.026395	0.134988	0.175913	0.111982	0.253041	-0.1164	0.236324	-0.00442	0.962446	6819274	7.050548	6.970649	7.009215	7.071938	7.258372	7.049297	7.177374	6.949541	6.916747	6.749173	6.988719	6.900805	2610027L	NM_026403
6819275	4397	8.334808	8.245954	8.302923	8.52731	-0.1925	0.438065	-0.28136	0.267124	-0.22439	0.369315	-0.05697	0.81523	0.088854	0.716197	0.031885	0.895812	6819275	8.547636	8.343851	8.112938	8.214886	7.748898	8.774078	8.321927	8.428952	8.15789	8.602603	8.502741	8.476587	Ltb4r2	NM_020490
6819276	4398	7.58736	7.288038	7.401023	7.437531	0.149828	0.313908	-0.14949	0.314923	-0.03651	0.800061	-0.11298	0.441191	0.299322	0.064102	0.186337	0.218178	6819276	7.624401	7.526994	7.610684	7.328246	7.328063	7.207805	7.318031	7.229383	7.655654	7.610843	7.540399	7.161352	Ltb4r1	NM_008519
6819284	4399	6.048437	6.346868	5.87544	5.879502	0.168935	0.104348	0.467366	0.000968	-0.00406	0.965951	0.471428	0.000916	-0.29843	0.011949	0.172996	0.097526	6819284	6.161379	6.121896	5.862035	6.378609	6.37776	6.284236	5.877356	5.988266	5.760699	5.779009	5.896348	5.963149	Nfatc4	NM_023699
6819287	4400	6.771722	6.941857	6.750124	6.831115	-0.05939	0.595703	0.110742	0.33307	-0.08099	0.47279	0.191733	0.112336	-0.17013	0.152157	0.021598	0.845778	6819287	6.741816	6.89806	6.67529	6.752716	7.020268	7.052586	6.726325	6.601921	6.922125	6.767182	6.869759	6.856404	9130227C	BC116260
6819311	4405	7.364726	7.404827	7.35001	7.37134	-0.00661	0.945962	0.033487	0.732441	-0.02133	0.827225	0.054816	0.578143	-0.0401	0.682736	0.014715	0.88021	6819311	7.284265	7.424991	7.384921	7.34545	7.465343	7.403687	7.445	7.480264	7.124767	7.285148	7.384695	7.444177	Parp4	NM_001145978
6819328	4406	8.902597	8.969694	8.91191	8.904752	-0.00216	0.983472	0.064942	0.537554	0.007158	0.945151	0.057785	0.582334	-0.0671	0.524486	-0.00931	0.928684	6819328	8.903166	8.92159	8.883035	8.830359	9.113279	8.965445	8.995476	9.05361	8.686643	8.857148	8.927112	8.929996	Zmym2	NM_029498
6819369	4407	7.344939	7.591224	7.435286	7.426076	-0.08114	0.291446	0.165148	0.050561	0.00921	0.901153	0.155938	0.061756	-0.24628	0.008978	-0.09035	0.243996	6819369	7.249403	7.365388	7.420026	7.541868	7.701746	7.530057	7.489265	7.412889	7.403703	7.389574	7.339581	7.549072	Ift88	NM_009376
6819418	4409	5.993787	5.920364	5.969506	6.01594	-0.02215	0.862486	-0.09558	0.462457	-0.04643	0.717456	-0.04914	0.701888	0.073423	0.569649	0.024281	0.849452	6819418	5.870553	5.920967	6.189842	5.956031	5.839159	5.965903	5.984332	6.11284	5.811347	5.927334	6.22999	5.890495	Sap18	NM_009119
6819425	4410	8.612091	8.860628	8.721184	8.874459	-0.26237	0.106149	-0.01383	0.9259	-0.15327	0.318553	0.139443	0.361567	-0.24854	0.122874	-0.10909	0.47074	6819425	8.529181	8.810723	8.496369	8.829388	8.843383	8.909112	8.602515	9.014892	8.546096	8.729923	8.838347	9.055106	Mrp63	NM_026401
6819442	4411	7.117906	7.030147	6.84147	6.928951	0.188955	0.231837	0.101196	0.507983	-0.08748	0.565749	0.188677	0.232463	0.087759	0.564543	0.276436	0.095017	6819442	7.207559	6.963281	7.182877	7.038187	7.055555	6.996699	6.980828	7.078254	6.465328	6.928832	6.906777	6.951244	Fgf9	NM_013518
6819499	4412	9.226671	9.117453	9.052336	9.185708	0.040963	0.73192	-0.06826	0.570728	-0.13337	0.28136	0.065117	0.58822	0.109218	0.371862	0.174335	0.169498	6819499	9.204217	9.092229	9.383568	9.041128	9.202752	9.108479	9.06369	9.053657	9.039661	9.207929	8.94815	9.401046	Rcbtb1	NM_027764
6819510	4413	8.123027	8.237367	8.103927	8.090591	0.032436	0.788643	0.146776	0.245099	0.013336	0.912063	0.13344	0.287092	-0.11434	0.35709	0.0191	0.87438	6819510	8.165052	8.153507	8.050522	8.219649	8.356233	8.136219	8.178161	8.225658	7.907962	7.902317	8.085327	8.284128	Cab39l	NM_026908
6819511	4414	6.923926	6.991265	7.033144	6.821291	0.102635	0.269965	0.169974	0.085295	0.211853	0.040165	-0.04188	0.641635	-0.06734	0.459185	-0.10922	0.242776	6819511	6.797123	6.870606	7.104048	7.028146	7.01265	6.933	7.159172	6.925257	7.015004	6.877105	6.772262	6.814506	Shisa2	NM_145463
6819547	4415	8.834186	8.861048	8.843235	8.660305	0.173881	0.027561	0.200743	0.014581	0.18293	0.022205	0.017813	0.789975	-0.02686	0.688812	-0.00905	0.892184	6819547	8.908768	8.74902	8.844769	8.814722	8.936398	8.832024	8.89348	8.898255	8.73797	8.642215	8.745109	8.593591	Mtmr6	NM_144843
6819573	4418	6.414493	6.412556	6.419134	6.539371	-0.12488	0.097628	-0.12681	0.093355	-0.12024	0.10864	-0.00658	0.923751	0.001936	0.977516	-0.00464	0.946148	6819573	6.439881	6.430273	6.373324	6.392947	6.377282	6.46744	6.357129	6.570464	6.329809	6.461251	6.545935	6.610927	Mipep	NM_027436
6819602	4421	6.930157	6.995125	7.077734	6.26979	0.660367	0.015267	0.725335	0.009701	0.807944	0.005542	-0.08261	0.710643	-0.06497	0.77008	-0.14758	0.511592	6819602	6.881377	7.174011	6.735083	6.860097	7.322635	6.802643	7.271854	6.70018	7.261168	6.256201	6.473286	6.079884	Arl11	NM_177337
6819628	4422	6.759377	6.961314	6.72662	6.65714	0.102237	0.511567	0.304174	0.075248	0.06948	0.653083	0.234694	0.153488	-0.20194	0.211905	0.032757	0.831321	6819628	6.635307	6.75641	6.886414	6.780457	6.981342	7.122143	6.910332	6.536252	6.733277	6.412716	6.690826	6.867878	Trim13	NM_023233
6819662	4424	6.57156	6.745469	6.710862	6.436993	0.134567	0.448136	0.308476	0.104903	0.273869	0.143197	0.034607	0.842602	-0.17391	0.332805	-0.1393	0.432936	6819662	6.536777	6.408588	6.769315	6.676389	6.753177	6.806841	6.914772	6.432243	6.78557	6.143312	6.66115	6.506516	Rnaseh2b	NM_026001
6819683	4425	8.548003	8.460889	8.404654	8.202185	0.345818	0.027116	0.258704	0.078169	0.202469	0.152717	0.056235	0.67235	0.087115	0.515756	0.14335	0.295693	6819683	8.532966	8.59311	8.517934	8.267476	8.659216	8.455974	8.35184	8.432533	8.429588	8.180957	8.449662	7.975936	Wdfy2	NM_175546
6819694	4426	12.76823	12.64641	12.6509	12.25377	0.514457	0.012094	0.39264	0.039099	0.397123	0.037421	-0.00448	0.978247	0.121817	0.466613	0.117333	0.482633	6819694	12.80711	12.63461	12.86297	12.39141	12.84715	12.70068	12.85425	12.34337	12.75507	12.28753	12.33618	12.13761	Ctsb	NM_007798
6819722	4427	7.088515	7.278401	7.076756	7.505451	-0.41694	0.005113	-0.22705	0.071284	-0.42869	0.004391	0.201645	0.102115	-0.18989	0.120361	0.011759	0.91693	6819722	7.183768	7.101754	6.980022	7.447879	7.325397	7.061927	7.037483	7.132528	7.060257	7.552449	7.347513	7.61639	Fam167a	NM_177628
6819737	4429	8.849234	8.61756	8.790543	8.535289	0.313945	0.054323	0.082272	0.571231	0.255254	0.104365	-0.17298	0.249649	0.231674	0.134988	0.058691	0.684719	6819737	8.967377	8.694242	8.886083	8.445602	8.881362	8.525717	8.867956	8.805614	8.698058	8.595862	8.686415	8.323589	2610028A	NM_028228
6819744	4430	6.468327	6.581916	6.757188	6.306191	0.162136	0.552716	0.275725	0.322766	0.450997	0.123062	-0.17527	0.521804	-0.11359	0.675671	-0.28886	0.301695	6819744	6.439221	6.722895	6.242865	6.573228	7.037968	6.134552	6.605125	6.777346	6.889094	5.931632	6.644294	6.342647	Sox7	NM_011446
6819842	4433	8.244016	8.201542	8.306034	8.141573	0.102443	0.268086	0.05997	0.505706	0.164461	0.092427	-0.10449	0.259368	0.042474	0.634945	-0.06202	0.491711	6819842	8.294941	8.21475	8.222357	8.18574	8.343847	8.07504	8.317751	8.214062	8.386289	7.999026	8.251336	8.174356	Ints9	NM_153414
6819860	4434	5.999084	6.180667	6.26605	6.331851	-0.33277	0.020056	-0.15118	0.224919	-0.0658	0.582785	-0.08538	0.478893	-0.18158	0.152868	-0.26697	0.048748	6819860	6.004551	5.879997	6.112704	6.177338	6.168271	6.196393	6.268125	6.430559	6.099466	6.126109	6.354897	6.514548	Fbxo16	NM_015795
6819866	4435	7.380595	7.378987	7.334066	7.216252	0.164343	0.383942	0.162735	0.388388	0.117814	0.527608	0.044921	0.807569	0.001608	0.993031	0.046529	0.800852	6819866	7.138885	7.48105	7.52185	7.499597	7.344286	7.293079	7.399341	7.45251	7.150348	6.896522	7.195787	7.556447	Zfp395	NM_199029

6819877	4436	8.332823	8.276802	8.299812	8.046251	0.286572	0.150371	0.230551	0.236536	0.253561	0.196971	-0.02301	0.901528	0.056022	0.763795	0.033012	0.85918	6819877	8.284594	8.482977	8.230899	7.970821	8.48571	8.373874	8.40155	8.102613	8.395272	7.739506	8.136009	8.263238	Scara5	NM_028903
6819885	4438	8.242582	8.164564	7.990734	7.835877	0.406705	0.030353	0.328687	0.066568	0.154857	0.346602	0.17383	0.294207	0.078018	0.627983	0.251848	0.142539	6819885	8.347968	8.070056	8.309721	7.980656	8.470704	8.042332	7.987872	8.070919	7.913411	7.98911	7.920936	7.597585	Ccdc25	NM_145944
6819887	4439	11.85049	10.98456	11.14385	9.662683	2.18781	0.004144	1.321874	0.043441	1.481167	0.027717	-0.15929	0.780119	0.865937	0.155139	0.706643	0.236113	6819887	12.03923	11.53126	11.98099	10.23838	11.87715	10.83814	11.64015	10.02037	11.77103	9.673406	9.989468	9.325175	Clu	NM_013492
6819901	4441	9.108923	8.87077	8.957361	9.10965	-0.00073	0.994619	-0.23888	0.051569	-0.15229	0.183088	-0.08659	0.431292	0.238152	0.052133	0.151562	0.184962	6819901	9.069496	9.080331	9.176941	8.994401	8.99031	8.6276	9.092399	8.941486	8.838198	9.068045	9.127054	9.13385	Trim35	NM_029979
6819907	4442	5.946988	5.849312	5.947632	5.949523	-0.00253	0.984185	-0.10021	0.442096	-0.00189	0.988203	-0.09832	0.450433	0.097676	0.453297	-0.00064	0.995981	6819907	6.050601	5.786654	6.00371	5.851248	5.900424	5.796264	5.883694	6.135006	5.824197	5.869611	5.795598	6.183359	Stmn4	NM_019675
6819910	4443	6.31626	6.550365	5.370749	6.263857	0.052403	0.882643	0.286508	0.428879	-0.89311	0.031745	1.179616	0.008946	-0.23411	0.515188	0.945511	0.025066	6819910	6.655821	6.181967	6.110992	6.939013	6.785427	5.926656	5.666935	5.619723	4.825589	6.210773	5.976689	6.604109	Adra1a	NM_013461
6819957	4446	7.356857	7.482107	7.31913	7.37766	-0.0208	0.837343	0.104447	0.31803	-0.05853	0.567195	0.162977	0.135178	-0.12525	0.237442	0.037727	0.710534	6819957	7.457227	7.271018	7.342326	7.400548	7.617769	7.428004	7.519246	7.266168	7.171975	7.323944	7.426678	7.382358	Kctd9	NM_001111028
6820013	4449	8.78817	9.834982	8.733602	9.853696	-1.06553	0.008774	-0.01871	0.953253	-1.12009	0.006781	1.10138	0.007404	-1.04681	0.009594	0.054568	0.86439	6820013	8.462071	9.141362	8.761076	9.809457	9.56403	10.13146	8.275841	8.827704	9.097261	9.709923	10.35762	9.493546	Stc1	NM_009285
6820033	4452	9.560399	9.889714	10.3325	9.973017	-0.41262	0.270829	-0.0833	0.817262	0.359483	0.332911	-0.44279	0.240005	-0.32931	0.372782	-0.7721	0.057777	6820033	9.316384	9.321564	10.04325	9.713281	9.470372	10.48549	10.25223	10.53098	10.21429	9.726751	9.650171	10.54213	Entpd4	NM_026174
6820040	4453	6.513575	6.642783	6.259109	5.978704	0.534871	0.038546	0.664079	0.015346	0.280405	0.231005	0.383674	0.114033	-0.12921	0.566802	0.254466	0.273248	6820040	6.662613	6.431299	6.446814	6.601139	7.093757	6.233454	6.499984	6.086086	6.191258	5.785915	6.134484	6.015713	Loxl2	NM_033325
6820047	4454	7.876369	7.841688	7.579027	7.319098	0.557271	0.003615	0.52259	0.005157	0.25993	0.094632	0.262661	0.091773	0.034681	0.806727	0.297341	0.061989	6820047	8.000898	7.807748	7.82046	7.646493	8.139753	7.738818	7.630875	7.514698	7.591509	7.268081	7.508799	7.180413	Tnfrsf10	AB031081
6820057	4457	7.507793	7.617185	7.641523	7.557117	-0.04932	0.525693	0.060067	0.442473	0.084406	0.289103	-0.02434	0.751788	-0.10939	0.179389	-0.13373	0.109756	6820057	7.524302	7.457971	7.541107	7.708439	7.597142	7.545973	7.71359	7.674934	7.536045	7.438749	7.544859	7.687744	Bin3	NM_021328
6820084	4460	7.694056	7.615116	7.469182	7.418593	0.275463	0.098075	0.196523	0.218405	0.050589	0.739838	0.145934	0.350319	0.07894	0.606192	0.224875	0.164943	6820084	7.680974	7.578335	7.82286	7.444975	7.897687	7.502686	7.496007	7.584795	7.326743	7.240653	7.622287	7.392839	Reep4	NM_180588
6820085	4461	7.047898	6.909309	6.799502	6.799095	0.248802	0.178106	0.110213	0.531479	0.000407	0.998132	0.109806	0.532958	0.138589	0.434709	0.248395	0.178739	6820085	7.104478	6.939872	7.099343	6.977207	7.172415	6.578304	6.942758	6.792974	6.662775	6.623412	7.051799	6.722075	Hr	NM_021877
6820088	4462	7.143792	7.145124	7.093861	7.245576	-0.10178	0.516633	-0.10045	0.521989	-0.15171	0.341499	0.051263	0.741384	-0.00133	0.993134	0.049931	0.747828	6820088	7.208557	7.080956	7.141863	7.365112	7.089759	6.980501	7.197369	7.303772	6.780442	7.160311	7.18953	7.386887	Nudt18	NM_153136
6820109	4464	6.016971	5.745195	5.883695	5.615322	0.401649	0.007089	0.129873	0.279235	0.268373	0.043281	-0.1385	0.250893	0.271776	0.041276	0.133276	0.267758	6820109	6.062703	5.982048	6.006161	5.747853	5.87579	5.611942	5.946507	5.665407	6.039171	5.769646	5.522253	5.554067	Dok2	NM_010071
6820216	4466	7.153185	7.225901	7.082918	7.029211	0.123974	0.359133	0.19669	0.161305	0.053707	0.684533	0.142983	0.294413	-0.07272	0.58394	0.070268	0.596427	6820216	7.148334	7.200374	7.110848	7.128495	7.39963	7.149577	7.228653	6.883107	7.136993	6.838341	7.009295	7.239997	Rcbtb2	NM_134083
6820219	4467	7.596209	7.765732	7.883728	7.42203	0.174178	0.376346	0.343701	0.101746	0.461698	0.037945	-0.118	0.543449	-0.16952	0.388613	-0.28752	0.160652	6820219	7.637581	7.4953	7.655745	7.534142	8.032201	7.730852	8.182111	7.632028	7.837045	7.144934	7.604913	7.516244	P2ry5	NM_175116
6820237	4468	6.79304	6.985855	6.890674	6.762891	0.030149	0.824385	0.222965	0.128363	0.127783	0.359569	0.095181	0.489735	-0.19282	0.180683	-0.09763	0.478968	6820237	6.759464	6.847476	6.772179	6.912894	7.09033	6.954342	7.074258	6.547491	7.050273	6.827405	6.765927	6.69534	Med4	NM_026119
6820241	4469	8.689168	8.725923	8.648723	8.716437	-0.02727	0.891835	0.009486	0.962251	-0.06771	0.736406	0.0772	0.701462	-0.03675	0.854644	0.040445	0.840277	6820241	8.595881	8.54868	8.922942	8.835367	8.640635	8.701766	8.895724	8.609997	8.440448	8.460197	8.574795	9.114318	Sucla2	NM_011506
6820325	4474	9.564337	9.451191	9.371367	8.413083	1.151253	0.00342	1.038108	0.006039	0.958284	0.009143	0.079824	0.783223	0.113145	0.69728	0.19297	0.510991	6820325	9.847544	9.569425	9.276041	9.393389	9.684145	9.27604	9.759882	8.806397	9.547822	8.194912	8.767234	8.277104	Lcp1	NM_008879
6820372	4477	7.036945	7.054561	6.971388	6.876023	0.160922	0.121527	0.178539	0.090902	0.095365	0.33475	0.083174	0.396837	-0.01762	0.854346	0.065557	0.500489	6820372	7.065142	6.998445	7.047247	6.884515	7.304053	6.975116	6.958984	6.990407	6.964772	6.864688	6.920357	6.843023	Nufip1	NM_013745
6820381	4478	7.951872	7.914977	7.857124	7.781187	0.170685	0.153071	0.13379	0.251022	0.075937	0.502391	0.057853	0.607147	0.036895	0.741715	0.094748	0.406404	6820381	7.915507	7.893414	8.046696	7.924629	8.078815	7.741488	7.827816	7.866783	7.876774	7.841793	7.9279	7.573868	Tsc22d1	NM_207652
6820409	4479	7.553031	7.725725	7.346698	7.156853	0.396178	0.193864	0.568872	0.076084	0.189846	0.515916	0.379026	0.211861	-0.17269	0.553612	0.206332	0.481223	6820409	7.745966	7.432065	7.481061	7.762301	7.623295	7.791578	7.584188	6.934562	7.521345	6.784764	6.89641	7.789384	Ccdc122	NM_175369
6820472	4481	6.935305	6.742103	6.750772	6.146279	0.789026	0.014479	0.595825	0.046877	0.604493	0.044443	-0.00867	0.973594	0.193202	0.468412	0.184533	0.487956	6820472	6.868756	6.88619	7.050969	6.484171	7.123986	6.618153	7.214176	6.228365	6.809774	6.064792	6.296851	6.077193	Epsti1	NM_029495
6820534	4482	8.586871	8.686578	8.757174	8.922113	-0.33524	0.074426	-0.23553	0.187611	-0.16494	0.342537	-0.0706	0.67726	-0.09971	0.558835	-0.1703	0.327978	6820534	8.679179	8.605807	8.475626	8.763892	8.519194	8.776649	8.730416	8.796295	8.744811	8.740018	8.692854	9.333468	1300010F	NM_027906
6820573	4484	8.912248	8.970861	8.95282	8.743146	0.169101	0.01754	0.227715	0.003863	0.209674	0.006062	0.018041	0.7585	-0.05861	0.331511	-0.04057	0.494635	6820573	8.90391	8.90991	8.922923	8.905626	9.086353	8.920605	8.978877	8.958491	8.921092	8.639025	8.807335	8.783079	Elf1	NM_007920
6820574	4485	9.214424	9.245694	9.269109	9.15703	0.057393	0.614693	0.088664	0.441905	0.112079	0.336397	-0.02342	0.836165	-0.03127	0.782643	-0.05469	0.631228	6820574	9.119046	9.239278	9.284947	9.168403	9.362503	9.206176	9.514717	9.113857	9.178753	9.061614	9.172962	9.236515	Sugt1	NM_026474
6820752	4486	7.174067	7.270081	7.155134	7.346926	-0.17286	0.529561	-0.07685	0.777629	-0.19179	0.486749	0.114947	0.673697	-0.09601	0.724582	0.018933	0.944392	6820752	7.289002	7.098513	7.134685	7.294512	7.289734	7.225997	7.049273	7.226977	7.189151	6.826035	7.170043	8.044701	Pcdh17	NM_001013753
6820896	4487	8.726154	8.995467	8.794205	9.343144	-0.61699	0.016334	-0.34768	0.126232	-0.54894	0.027287	0.201262	0.352059	-0.26931	0.222668	-0.06805	0.746905	6820896	8.5557	8.841832	8.780931	9.178225	8.747219	9.060956	8.723715	9.064725	8.594175	9.236903	9.066081	9.726449	Tdrd3	NM_172605
6821298	4489	5.966749	6.114248	5.800922	5.824863	0.141886	0.366806	0.289386	0.086866	-0.02394	0.875782	0.313326	0.067627	-0.1475	0.349154	0.165826	0.296036	6821298	6.044299	5.927246	5.928701	5.85872	6.394041	6.089984	5.93542	5.553228	5.914119	5.846559	5.719473	5.908556	6720463M	NM_175265
6821301	4490	7.216413	7.218023	7.180986	7.156777	0.059637	0.488081	0.061246	0.476789	0.024209	0.77548	0.037037	0.663714	-0.00161	0.98483	0.035427	0.677328	6821301	7.144117	7.398045	7.107078	7.172872	7.297719	7.183478	7.253346	7.075525	7.214087	7.108973	7.18971	7.171647	Pibf1	NM_029320
6821304	4491	7.789931	7.956585	7.486225	7.69251	0.097421	0.731394	0.264075	0.363425	-0.20628	0.473142	0.47036	0.124408	-0.16665	0.559942	0.303706	0.299933	6821304	7.728207	7.597662	8.043923	8.018201	8.227858	7.623695	7.486508	7.513737	7.458429	7.538929	8.303474	7.235126	Klf5	NM_009769
6821985	4496	7.850809	7.793121	7.792242	7.105153	0.745656	0.049645	0.687968	0.065608	0.68709	0.065887	0.000879	0.997894	0.057688	0.862573	0.058566	0.860505	6821985	7.719451	8.119648	7.713327	7.441781	8.180544	7.757038	7.996339	7.244655	8.135733	6.730212	7.605764	6.979482	Gpc6	NM_001079844
6822073	4497	7.878248	7.908018	7.881782	7.916125	-0.03788	0.660506	-0.00811	0.924649	-0.03434	0.690127	0.026236	0.76019	-0.02977	0.729311	-0.00353	0.967106	6822073	7.810281	7.919108	7.905355	7.839181	8.04393	7.840943	8.027801	7.893704	7.723841	7.883545	7.9377	7.92713	Gpr180	NM_021434
6822127	4499	10.61113	10.62558	10.93954	10.79604	-0.18492	0.319408	-0.17046	0.356425	0.1435	0.433906	-0.31396	0.109141	-0.01445	0.935909	-0.32842	0.096098	6822127	10.66667	10.65831	10.5084	10.43466	10.53715	10.90493	11.1081	11.0747	10.63583	10.90639	10.92625	10.55549	Dnajc3	NM_008929
6822138	4500	5.977782	5.651602	5.905549	5.922741	0.055042	0.769501	-0.27114	0.173688	-0.01719	0.926889	-0.25395	0.199456	0.32618	0.110133	0.072234	0.701445	6822138	5.944096	6.35778	5.631471	5.519462	5.692066	5.743279	5.904406	6.050779	5.761461	5.812682	6.123672	5.831868	Hs6st3	NM_015820
6822167	4502	10.72121	10.87505	10.8922	10.81882	-0.09761	0.202136	0.05623	0.446562	0.073383	0.326723	-0.01715	0.813233	-0.15384	0.059927	-0.17099	0.040938	6822167	10.74531	10.65712	10.7612	10.86999	10.94192	10.81323	10.98787	10.89396	10.79477	10.69293	10.84867	10.91485	Mbnl2	NM_175341
6822173	4503	6.796234	6.98647	6.717125	6.455817	0.340418	0.100636	0.530654	0.020129	0.261309	0.192192	0.269345	0.180278	-0.19024	0.33012	0.079109	0.677723	6822173	6.880626	6.84065	6.667427	6.815804	7.321932	6.821675	6.724498	6.554594	6.872284	6.293146	6.781285	6.293019	Rap2a	NM_029519
6822191	4504	8.777153	8.848409	8.856361	8.753989	0.023164	0.861152	0.09442	0.482591	0.102372	0.447751	-0.00795	0.952075	-0.07126	0.593645	-0.07921	0.55397	6822191	8.762338	8.660892	8.908228	8.723919	9.016579	8.804729	9.082547	8.628922	8.857615	8.644941	8.811236	8.80579	Ipo5	NM_023579
6822216	4505	7.69603	7.317448	7.490231	7.515291	0.180739	0.10142	-0.19784	0.077398	-0.02506	0.803991	-0.17278	0.114876	0.378582	0.004702	0.205799	0.068198	6822216	7.872103	7.680151	7.535836	7.367324	7.220407	7.364612	7.468262	7.550626	7.451804	7.482496	7.666272	7.397105	B930095G	BC096543
6822285	4506	8.174931	8.248449	8.132464	8.223794	-0.04886	0.657071	0.024655	0.821903	-0.09133	0.41395	0.115985	0.305682	-0.07352	0.507559	0.042467	0.699153	6822285	8.222249	8.15301	8.149535	8.268901	8.256637	8.219808	8.183677	8.350385	7.86333	8.196549	8.180487	8.294345	Ubac2	NM_026861
6822296	4507	9.784399	9.877287	9.889555	9.971998	-0.1876	0.053352	-0.09471	0.286004	-0.08244	0.348812	-0.01227	0.885946	-0.09289	0.294723	-0.10516	0.240017	6822296	9.792635	9.832544	9.728019	9.763904	9.844348	10.02361	10.02619	9.842039	9.800436	9.881295	10.02822	10.00648	Tm9sf2	NM_080556
6822297	4508	7.650291	7.944816	7.865458	8.490775	-0.84048	0.000652	-0.54596	0.00805	-0.62532	0.003886	0.079358	0.62442	-0.29453	0.095503	-0.21517	0.204814	6822297	7.452635	7.675291	7.822947	8.109268	7.707351	8.017829	7.728271	8.040682	7.827422	8.430767	8.324652	8.716905	Clybl	NM_029556
6822317	4509	6.168201	6.199526	6.292626	6.152093	0.016108	0.853464	0.047434	0.589678	0.140533	0.134623	-0.0931	0.302285	-0.03133	0.720284	-0.12443	0.178834	6822317	6.045238	6.129343	6.330021	6.174634	6.163106	6.260839	6.35693	6.257335	6.263612	6.158364	6.024941	6.272973	Zic2	NM_009574
6822320	4510	7.698948	7.807745	7.678597	7.8997	-0.20075	0.130962	-0.09196	0.463035	-0.2211	0.100992	0.129147	0.310611	-0.1088	0.388497	0.020351	0.868801	6822320	7.696756	7.703382	7.696706	7.786708	7.771027	7.865499	7.734691	7.64345	7.657651	7.737231	7.734704	8.227165	Pcca	NM_144844
6822367	4511	7.607537	7.689503	6.855898	6.715865	0.891672	0.042885	0.973638	0.030396	0.140033	0.715554	0.833605	0.054757	-0.08197	0.830615	0.751638	0.077245	6822367	7.55852	7.838583	7.425507	7.37886	8.298473	7.391176	7.236401	6.690869	6.640425	5.993304	7.034836	7.119455	Itgbl1	NM_145467
6822443	4512	5.262997	5.575378	6.032207	5.126449	0.136548	0.733524	0.448929	0.279831	0.905758	0.047516	-0.45683	0.272076	-0.31238	0.443234	-0.76921	0.08227	6822443	5.311244	5.201442	5.276305	5.322548	6.201635	5.201952	6.231394	5.366698	6.498529	4.881224	5.700678	4.797445	Dnase1l3	NM_007870
6822459	4513	5.932492	6.206591	6.015881	5.999297	-0.06681	0.593063	0.207294	0.122451	0.016584	0.893529	0.19071	0.150774	-0.2741	0.05179	-0.08339	0.506914	6822459	5.961794	5.785009	6.050672	6.141176	6.117501	6.361096	6.076289	6.05798	5.913373	5.945216	5.826433	6.226241	Pdhb	NM_024221
6822477	4515	5.64425	6.003978	5.806859	5.80657	-0.16232	0.552487	0.197408	0.472423	0.000289	0.999145	0.197119	0.473051	-0.35973	0.206679	-0.16261	0.551795	6822477	5.591166	5.672257	5.669327	6.427557	5.992713	5.591664	5.758543	6.028634	5.6334	6.301444	5.658373	5.459892	Fam107a	NM_183187
6822478	4516	6.288414	6.375992	6.126142	6.385172	-0.09676	0.685072	-0.00918	0.969141	-0.25903	0.292747	0.24985	0.309016	-0.08758	0.713298	0.162271	0.500531	6822478	6.516528	6.119068	6.229645	6.698207	6.576931	5.852838	6.181448	6.06639	6.130589	6.284518	6.671054	6.199945	Oit1	NM_146050
6822485	4517	5.261923	6.039537	6.010861	5.330189	-0.06827	0.657125	0.709348	0.001374	0.680673	0.001765	0.028675	0.851304	-0.77761	0.000773	-0.74894	0.000981	6822485	5.324214	5.094152	5.367404	6.196951	6.174289	5.74737	6.022966	5.952869	6.056749	5.213196	5.207543	5.569827	4930452B	BC064468
6822526	4518	6.564215	7.03126	6.851861	6.747588	-0.18337	0.147467	0.283672	0.038067	0.104273	0.388482	0.179399	0.155317	-0.46704	0.003513	-0.28765	0.036061	6822526	6.598739	6.432932	6.660975	7.071151	7.197595	6.825034	6.754902	6.824581	6.9761	6.755353	6.617886	6.869524	Fhit	NM_010210
6822772	4522	7.937423	8.125519	8.06093	7.87973	0.057693	0.720158	0.245789	0.152482	0.1812	0.277308	0.064589	0.688683	-0.1881	0.260789	-0.12351	0.449799	6822772	7.889063	8.043839	7.879368	8.14791	8.209342	8.019305	8.342187	7.996229	7.844374	7.596102	8.061832	7.981257	Psmd6	NM_025550
6822784	4523	6.864041	6.943079	6.909001	6.882773	-0.01873	0.888417	0.060306	0.653431	0.026228	0.844352	0.034078	0.798823	-0.07904	0.558062	-0.04496	0.737076	6822784	6.680242	7.025738	6.886142	6.807502	7.001328	7.020408	6.925594	6.96015	6.841259	6.678973	6.838461	7.130885	Thoc7	NM_025435
6822852	4525	6.098949	5.809348	5.800109	5.652675	0.446274	0.045597	0.156673	0.430448	0.147434	0.457101	0.009239	0.962149	0.289601	0.16339	0.29884	0.151915	6822852	6.212293	6.078024	6.006529	5.633282	6.108083	5.686679	5.916456	5.388618	6.095253	5.661448	5.653957	5.64262	Lrrc3b	NM_146052
6822879	4526	6.101653	6.182393	6.083352	6.250035	-0.14838	0.457264	-0.06764	0.73101	-0.16668	0.405861	0.09904	0.616262	-0.08074	0.682041	0.0183	0.925629	6822879	5.992581	6.333072	5.979305	6.290783	6.203101	6.053294	5.818076	6.136586	6.295395	5.918367	6.273272	6.558465	Oxsm	NM_027695
6822946	4528	9.510303	9.942168	9.340767	9.989962	-0.47966	0.104341	-0.04779	0.859709	-0.64919	0.038152	0.601401	0.050724	-0.43187	0.137691	0.169536	0.535461	6822946	8.891915	9.792301	9.846693	9.940841	9.820763	10.0649	9.310464	9.163885	9.547951	9.7288	9.978385	10.2627	Nr1d2	NM_011584
6822949	4529	8.141401	8.199748	8.201677	8.25866	-0.11726	0.183719	-0.05891	0.485596	-0.05698	0.49956	-0.00193	0.981492	-0.05835	0.489653	-0.06028	0.47587	6822949	8.056999	8.128513	8.23869	8.08909	8.255772	8.254383	8.234335	8.175363	8.195333	8.09428	8.325531	8.356169	Ube2e1	NM_009455
6822959	4530	7.818967	7.937958	7.66003	7.757547	0.06142	0.69715	0.180411	0.269941	-0.09752	0.539665	0.277928	0.105302	-0.11899	0.456889	0.158937	0.326939	6822959	8.132402	7.783183	7.541316	8.045149	7.932332	7.836393	7.767047	7.770665	7.442378	7.816375	7.687299	7.768968	Ube2e2	NM_144839
6823007	4531	8.613818	8.881762	8.757745	8.999731	-0.38591	0.119703	-0.11797	0.608869	-0.24199	0.306526	0.124017	0.59096	-0.26794	0.261032	-0.14393	0.534132	6823007	8.723712	8.722547	8.395195	9.058643	8.755033	8.831611	8.227682	9.053753	8.9918	8.83419	9.121122	9.04388	Gng2	NM_010315

6823010	4532	7.141745	7.348079	7.469007	7.212375	-0.07063	0.672142	0.135704	0.423269	0.256632	0.149198	-0.12093	0.473612	-0.20633	0.235398	-0.32726	0.076263	6823010	7.149775	6.931034	7.344427	7.276625	7.24039	7.527222	7.470284	7.707335	7.229403	7.046741	7.39678	7.193604	1810063B	NM_026209
6823011	4533	9.653587	9.385453	9.429017	9.280045	0.373542	0.005858	0.105408	0.324321	0.148972	0.176048	-0.04356	0.675733	0.268134	0.0283	0.22457	0.055652	6823011	9.731621	9.58637	9.642769	9.42129	9.531872	9.203196	9.307432	9.393795	9.585824	9.236712	9.228229	9.375193	Kcnk5	NM_021542
6823041	4534	7.799313	7.739098	7.584398	7.180687	0.618626	0.010102	0.558411	0.016493	0.403711	0.060366	0.1547	0.426685	0.060215	0.752832	0.214915	0.278222	6823041	7.799816	7.657742	7.940382	7.652255	7.919081	7.645959	7.85774	7.2394	7.656055	6.956993	7.44595	7.139119	Camk2g	NM_178597
6823051	4535	8.50314	8.466447	8.560051	8.477896	0.025244	0.772086	-0.01145	0.895252	0.082155	0.358032	-0.0936	0.298805	0.036693	0.674675	-0.05691	0.518381	6823051	8.427144	8.562613	8.519663	8.406116	8.564834	8.42839	8.652523	8.498671	8.528959	8.299906	8.56488	8.568903	Ecd	NM_027475
6823058	4536	7.317366	7.512597	7.338111	7.3068	0.010565	0.939498	0.205796	0.165639	0.031311	0.822288	0.174486	0.231958	-0.19523	0.185861	-0.02075	0.881591	6823058	7.262455	7.287808	7.401834	7.422531	7.595377	7.519882	7.558051	7.389004	7.067278	7.101915	7.358946	7.45954	Dnajc9	NM_134081
6823059	4537	7.562818	7.708104	7.666972	7.799865	-0.23705	0.011438	-0.09176	0.241831	-0.13289	0.104554	0.041132	0.586548	-0.14529	0.080362	-0.10415	0.189295	6823059	7.580296	7.633057	7.475101	7.652893	7.666173	7.805246	7.734225	7.708973	7.557719	7.822635	7.696098	7.880861	Mrps16	NM_025440
6823068	4538	9.478283	9.458705	9.543386	9.292054	0.186229	0.213895	0.166651	0.26144	0.251332	0.105886	-0.08468	0.556288	0.019578	0.890629	-0.0651	0.64952	6823068	9.359953	9.448583	9.626314	9.491753	9.396358	9.488004	9.834593	9.361277	9.434288	9.165861	9.481417	9.228884	Anxa7	NM_009674
6823084	4539	9.001368	8.955062	9.035595	8.97104	0.030328	0.764441	-0.01598	0.87429	0.064555	0.527805	-0.08053	0.434171	0.046306	0.648568	-0.03423	0.735428	6823084	8.931996	9.022909	9.049199	8.905553	9.052978	8.906655	9.207669	9.080244	8.818873	8.873676	9.025697	9.013748	Ppp3cb	NM_008914
6823100	4540	8.830888	9.025546	8.712169	9.123467	-0.29258	0.060228	-0.09792	0.485139	-0.4113	0.015254	0.313377	0.047255	-0.19466	0.183791	0.118719	0.400802	6823100	8.79293	8.795887	8.903847	9.176853	9.010201	8.889583	8.478964	8.872293	8.785249	9.11192	8.929675	9.328805	Usp54	NM_030180
6823105	4541	8.400135	8.560097	8.275873	8.686307	-0.28617	0.080541	-0.12621	0.403515	-0.41043	0.020888	0.284225	0.082255	-0.15996	0.296079	0.124263	0.410487	6823105	8.253467	8.333353	8.613586	8.683558	8.428325	8.568409	8.254521	8.380009	8.193088	8.584248	8.505232	8.96944	Usp54	NM_030180
6823114	4542	7.301812	6.922707	6.948261	7.191018	0.110794	0.927903	-0.26831	0.826776	-0.24276	0.843004	-0.02555	0.983346	0.379105	0.757534	0.353551	0.773325	6823114	5.53694	6.702266	9.66623	8.007523	7.037055	5.723543	8.004808	6.673785	6.16619	5.691682	7.823215	8.058157	Myoz1	NM_021508
6823116	4543	6.142838	6.002801	6.005913	5.999289	0.143549	0.450475	0.003512	0.98499	0.006624	0.971695	-0.00311	0.986699	0.140037	0.461236	0.136925	0.470915	6823116	5.960164	5.970168	6.498181	6.292387	5.914821	5.801195	6.171236	6.019194	5.827309	5.935848	6.084638	5.977381	Synpo2l	NM_175132
6823122	4544	7.111104	7.134695	7.095839	6.87726	0.233845	0.026349	0.257435	0.01729	0.218579	0.034703	0.038856	0.663586	-0.02359	0.790918	0.015265	0.863603	6823122	7.150965	7.160486	7.021862	7.098186	7.309555	6.996343	7.127761	7.07113	7.088626	6.783975	6.998331	6.849473	Ndst2	NM_010811
6823149	4545	7.845591	7.8783	7.80676	7.800706	0.044885	0.729058	0.077594	0.552377	0.006054	0.962592	0.07154	0.583153	-0.03271	0.800356	0.038831	0.764211	6823149	7.812416	7.869956	7.854401	7.81736	7.839151	7.97839	7.952744	7.560183	7.907354	7.667017	8.028417	7.706685	Ap3m1	NM_018829
6823213	4546	6.232294	6.177109	6.275395	6.295826	-0.06353	0.532303	-0.11872	0.257388	-0.02043	0.839013	-0.09829	0.342235	0.055185	0.586348	-0.0431	0.669677	6823213	6.304867	6.058263	6.333753	6.244457	6.062469	6.224402	6.426823	6.135906	6.263457	6.348006	6.24379	6.295683	Dusp13	NM_001007268
6823221	4547	7.328312	7.323763	7.367605	7.254904	0.073408	0.53256	0.068859	0.557663	0.112701	0.346005	-0.04384	0.707063	0.004549	0.968752	-0.03929	0.736029	6823221	7.360121	7.27738	7.347436	7.358592	7.436144	7.176554	7.424412	7.47353	7.204872	7.145469	7.145844	7.473399	Comtd1	NM_026965
6823223	4548	7.757584	7.632741	7.736928	7.604676	0.152908	0.336781	0.028065	0.855905	0.132252	0.402602	-0.10419	0.506012	0.124843	0.428345	0.020656	0.893626	6823223	7.653226	7.881312	7.738213	7.540596	7.729838	7.627788	7.684734	8.053045	7.473004	7.47947	7.548141	7.786416	Zfp503	NM_145459
6823302	4551	7.943755	8.476437	7.959483	8.859737	-0.91598	0.003886	-0.3833	0.131722	-0.90025	0.004279	0.516954	0.053381	-0.53268	0.047939	-0.01573	0.946773	6823302	7.814589	8.029474	7.987203	8.369814	8.358591	8.700906	7.732151	8.33989	7.806409	8.645873	8.623439	9.309899	Dlg5	BC021314
6823336	4552	7.584244	7.657564	7.62882	7.570532	0.013713	0.748823	0.087032	0.068549	0.058289	0.196531	0.028743	0.506885	-0.07332	0.114305	-0.04458	0.312699	6823336	7.553336	7.584169	7.615228	7.656101	7.743066	7.573524	7.635924	7.666254	7.584283	7.554204	7.593083	7.564308	Polr3a	NM_001081247
6823414	4553	7.487225	8.214319	7.584056	7.394017	0.093209	0.712194	0.820302	0.009865	0.190039	0.458147	0.630263	0.032361	-0.72709	0.017541	-0.09683	0.701638	6823414	7.576468	7.599219	7.285989	8.36835	8.314759	7.959848	8.124013	7.265929	7.362225	7.134248	7.451243	7.596559	Zcchc24	NM_001101433
6823429	4554	9.741218	11.05996	10.01627	9.930751	-0.18953	0.34217	1.129213	0.000318	0.085518	0.66078	1.043694	0.000534	-1.31875	0.00011	-0.27505	0.180984	6823429	9.54369	9.73731	9.942654	10.95743	11.17148	11.05098	10.47434	9.788945	9.785522	9.883959	9.986562	9.921731	D14Ertd4	NM_025311
6823451	4555	9.250907	9.172161	9.159518	9.05593	0.194977	0.040684	0.116231	0.184178	0.103588	0.231332	0.012643	0.878299	0.078746	0.353617	0.091389	0.286173	6823451	9.302138	9.26349	9.187093	9.154715	9.179117	9.182652	9.266625	9.08172	9.13021	8.880596	9.093958	9.193237	Slmap	NM_032008
6823460	4556	7.961855	7.888037	7.947922	7.931645	0.03021	0.825911	-0.04361	0.751285	0.016277	0.905559	-0.05989	0.664295	0.073818	0.593848	0.013933	0.9191	6823460	8.046632	7.893669	7.945264	7.806204	8.131151	7.726756	8.035435	8.022885	7.785446	7.793008	8.141668	7.860258	E430028B	NM_178668
6823470	4557	8.120762	8.055589	8.06354	7.869403	0.25136	0.039236	0.186187	0.105738	0.194137	0.093821	-0.00795	0.939859	0.065173	0.541187	0.057223	0.590594	6823470	8.135305	8.09836	8.128622	7.969697	8.128967	8.068104	8.222082	8.017883	7.950654	7.723102	8.082884	7.802222	Appl1	NM_145221
6823513	4558	7.422594	7.440009	7.622977	7.426828	-0.00423	0.973082	0.01318	0.916378	0.196149	0.145491	-0.18297	0.170925	-0.01741	0.889694	-0.20038	0.138082	6823513	7.32851	7.561111	7.378161	7.22867	7.633286	7.45807	7.565338	7.652073	7.651521	7.244898	7.590405	7.445182	Ccdc66	NM_177111
6823653	4560	7.915176	7.991516	7.707687	7.499464	0.415712	0.110056	0.492053	0.066101	0.208223	0.39436	0.283829	0.254736	-0.07634	0.749878	0.207489	0.395953	6823653	7.680941	7.946497	8.11809	7.845801	8.473528	7.65522	7.608023	7.935055	7.579983	7.506835	7.271617	7.719939	Il17rb	NM_019583
6823659	4561	6.238884	6.246636	6.200588	6.277829	-0.03894	0.869247	-0.03119	0.895069	-0.07724	0.744693	0.046048	0.845727	-0.00775	0.97384	0.038297	0.871398	6823659	6.659185	6.019279	6.038189	6.372269	6.344884	6.022754	6.290238	6.268829	6.042696	6.278194	5.922984	6.632308	Cacna1d	NM_001083616
6823666	4562	9.007973	8.831892	9.135283	8.288083	0.719889	0.003969	0.543809	0.016586	0.847199	0.001535	-0.30339	0.130623	0.176081	0.356946	-0.12731	0.499779	6823666	9.131761	8.964981	8.927176	8.810017	9.082029	8.60363	9.258978	9.010015	9.136855	8.279494	8.623597	7.961159	Prkcd	NM_011103
6823690	4563	6.879732	8.324833	7.425532	6.107001	0.772732	0.27051	2.217832	0.009397	1.318531	0.078086	0.899301	0.20564	-1.4451	0.057751	-0.5458	0.427378	6823690	6.642728	7.393439	6.60303	7.718818	9.378971	7.876709	7.630698	6.496544	8.149353	5.813209	7.114002	5.393791	Itih3	NM_008407
6823693	4564	6.372431	6.570892	6.689436	6.230279	0.142153	0.449457	0.340614	0.093199	0.459158	0.033208	-0.11854	0.525899	-0.19846	0.299183	-0.317	0.114116	6823693	6.555074	6.285241	6.276979	6.554872	6.677269	6.480536	6.717608	6.744266	6.606435	6.202933	6.632461	5.855442	Itih1	NM_008406
6823695	4565	8.292688	8.346455	8.344721	8.173436	0.119252	0.734161	0.173019	0.623658	0.171284	0.627088	0.001734	0.996044	-0.05377	0.877944	-0.05203	0.881846	6823695	8.063168	8.549274	8.265622	7.992034	8.27519	8.772141	8.699103	8.293188	8.041871	7.700294	7.966787	8.853228	Spcs1	NM_026911
6823696	4566	7.978451	7.999788	8.043433	7.916915	0.061536	0.629656	0.082874	0.518622	0.126518	0.332836	-0.04364	0.731367	-0.02134	0.866318	-0.06498	0.61091	6823696	8.09802	7.962502	7.87483	7.858089	8.278666	7.86261	8.057881	8.094286	7.978132	7.859492	8.061494	7.829758	Gnl3	NM_153547
6823709	4567	8.58489	8.883535	8.611294	8.361894	0.222996	0.106392	0.521641	0.002779	0.249399	0.076318	0.272241	0.057104	-0.29864	0.040801	-0.0264	0.83485	6823709	8.794706	8.59264	8.367324	8.832366	8.988124	8.830115	8.707686	8.466453	8.659742	8.201342	8.466735	8.417606	Stab1	NM_138672
6823710	4568	10.00063	9.998276	10.07289	9.871085	0.129544	0.181588	0.127191	0.188785	0.201808	0.052131	-0.07462	0.423847	0.002354	0.979444	-0.07226	0.43803	6823710	10.05183	9.948958	10.0011	9.882874	10.19893	9.913023	10.07634	10.10751	10.03483	9.760561	9.985305	9.867389	Nisch	NM_022656
6823716	4570	6.175548	6.497579	6.368128	6.440018	-0.26447	0.247042	0.057561	0.792661	-0.07189	0.743006	0.129451	0.557997	-0.32203	0.166852	-0.19258	0.389743	6823716	6.028196	6.360929	6.137518	6.427873	6.550458	6.514407	6.207279	6.288146	6.60896	6.339794	6.918738	6.061523	Dnahc1	ENSMUST000000
6823721	4572	8.637695	9.200824	9.212216	9.353258	-0.71556	0.066296	-0.15243	0.662785	-0.14104	0.686328	-0.01139	0.97384	-0.56313	0.132985	-0.57452	0.126354	6823721	8.594377	8.609793	8.708916	9.211356	9.061003	9.330112	9.040803	9.640866	8.954978	9.152238	8.755006	10.15253	Sh3bp5	NM_011894
6823724	4573	7.742738	7.816996	7.583836	7.714143	0.028595	0.835808	0.102853	0.463336	-0.13031	0.35777	0.23316	0.118971	-0.07426	0.593388	0.158903	0.26821	6823724	7.667885	7.808502	7.751828	7.526474	7.944137	7.980377	7.774954	7.521318	7.455235	7.698822	7.623264	7.820343	Mettl6	NM_025907
6823742	4574	6.592687	6.830253	6.722717	6.773002	-0.18032	0.108867	0.057251	0.58251	-0.05029	0.628437	0.107536	0.313333	-0.23757	0.04476	-0.13003	0.229481	6823742	6.529963	6.632518	6.61558	6.894936	6.73952	6.856303	6.851858	6.600585	6.715708	6.637548	6.696562	6.984897	Hacl1	NM_019975
6823768	4575	8.100302	8.128471	8.275543	8.215205	-0.1149	0.46584	-0.08673	0.579171	0.060338	0.69815	-0.14707	0.355753	-0.02817	0.855775	-0.17524	0.276514	6823768	8.093906	8.062032	8.144969	8.134879	8.172791	8.077743	8.325307	8.592806	7.908516	8.089374	8.261518	8.294724	Dph3	NM_172254
6823849	4579	7.262289	7.373679	7.328197	7.335588	-0.0733	0.335472	0.038092	0.608824	-0.00739	0.920252	0.045482	0.542629	-0.11139	0.15802	-0.06591	0.383777	6823849	7.254045	7.211569	7.321254	7.386353	7.423956	7.310729	7.428154	7.27956	7.276878	7.211962	7.324214	7.470587	Mapk8	NM_016700
6823886	4582	7.072647	7.105785	7.022283	7.969269	-0.89662	0.277552	-0.86348	0.294403	-0.94699	0.253456	0.083503	0.916269	-0.03314	0.966709	0.050364	0.949427	6823886	7.070173	6.987016	7.160752	7.003857	6.962801	7.350698	7.022203	7.389889	6.654756	7.016739	10.08414	6.806927	2200001I	BC147105
6823887	4583	8.110882	8.107409	7.710904	7.774059	0.336823	0.029227	0.33335	0.030493	-0.06315	0.632559	0.396504	0.014222	0.003474	0.978861	0.399978	0.013647	6823887	8.15297	8.120971	8.058706	8.12592	8.128431	8.067875	7.779829	7.6818	7.671084	7.447324	8.03619	7.838663	Sncg	NM_011430
6823889	4584	9.390954	9.531441	9.540034	9.327796	0.063158	0.504358	0.203645	0.054239	0.212238	0.046757	-0.00859	0.926574	-0.14049	0.158588	-0.14908	0.13756	6823889	9.298474	9.484956	9.389433	9.415517	9.650393	9.528414	9.609452	9.616312	9.394338	9.230309	9.435092	9.317988	Bmpr1a	NM_009758
6823891	4585	6.845725	6.590625	6.541051	6.663135	0.18259	0.749699	-0.07251	0.898898	-0.12208	0.830773	0.049574	0.93076	0.2551	0.656806	0.304674	0.596648	6823891	5.984469	6.464359	8.088346	7.187837	6.434523	6.149514	7.018309	6.512319	6.092524	6.270373	6.870607	6.848424	Ldb3	NM_011918
6824000	4587	10.11212	10.09774	10.03733	9.694968	0.417149	0.019853	0.402772	0.023152	0.342359	0.044468	0.060413	0.685419	0.014377	0.922813	0.074789	0.617036	6824000	10.12133	10.16666	10.04836	10.0545	10.21496	10.02376	10.188	9.821802	10.10218	9.386724	9.804436	9.893744	Gcap14	NM_027045
6824007	4590	10.04859	10.034	10.08956	10.23795	-0.18935	0.254231	-0.20395	0.222391	-0.14838	0.363956	-0.05557	0.727833	0.014597	0.926887	-0.04097	0.797138	6824007	9.992764	10.0493	10.10372	9.956172	10.05603	10.08979	10.23193	9.977311	10.05945	9.938206	10.16331	10.61232	Ghitm	NM_078478
6824142	4591	8.64731	8.82344	8.586923	8.514611	0.132698	0.449005	0.308828	0.101084	0.072312	0.675916	0.236516	0.193731	-0.17613	0.321585	0.060386	0.726561	6824142	8.724849	8.482038	8.735042	8.760867	9.0545	8.654952	8.798693	8.389699	8.572378	8.323674	8.425506	8.794654	Tspan14	NM_145928
6824146	4592	9.946663	10.15079	9.797165	10.47496	-0.5283	0.037308	-0.32417	0.164506	-0.67779	0.012625	0.353621	0.133637	-0.20412	0.363511	0.149498	0.500429	6824146	9.966221	9.831338	10.04243	10.20644	10.12297	10.12295	9.311832	10.12258	9.957084	10.53423	10.18188	10.70877	5730469M	BC056635
6824166	4593	8.026342	8.134208	7.543187	6.696976	1.329366	0.0354	1.437232	0.025748	0.846211	0.146326	0.591021	0.293751	-0.10787	0.842637	0.483155	0.38518	6824166	8.120345	7.869525	8.089157	7.890876	8.603013	7.908735	7.809595	6.232777	8.58719	6.716388	6.879767	6.494773	Sftpd	NM_009160
6824181	4594	5.420971	5.310986	4.591043	6.547704	-1.12673	0.040033	-1.23672	0.027605	-1.95666	0.002792	0.719943	0.156336	0.109985	0.817113	0.829928	0.108972	6824181	4.887237	5.430999	5.944677	5.013077	4.553087	6.366795	4.805091	4.245707	4.722332	6.554286	6.648585	6.44024	Ptgdr	NM_008962
6824195	4595	8.522679	8.874418	8.742478	8.755825	-0.23315	0.093344	0.118593	0.361081	-0.01335	0.915873	0.13194	0.312595	-0.35174	0.020733	-0.2198	0.110341	6824195	8.538108	8.523663	8.506266	8.777556	8.967072	8.878626	8.795666	8.737123	8.694644	8.762959	8.472836	9.031679	Txndc16	NM_172597
6824206	4596	9.105302	9.008832	9.295957	9.328564	-0.22326	0.078253	-0.31973	0.020183	-0.03261	0.775661	-0.28712	0.031828	0.096469	0.408551	-0.19066	0.123071	6824206	9.10485	9.058071	9.152984	8.872575	9.132754	9.021168	9.448179	9.356733	9.082958	9.339438	9.189271	9.456984	Ero1l	NM_015774
6824216	4597	6.913536	6.802772	6.634007	6.937416	-0.02388	0.925308	-0.13464	0.600295	-0.30341	0.25392	0.168765	0.513464	0.110764	0.665526	0.279529	0.29024	6824216	6.87255	6.989165	6.878893	6.311445	6.928301	7.168571	7.00662	6.28182	6.613582	7.129662	6.921355	6.761231	Gnpnat1	NM_019425
6824223	4598	8.351206	8.468617	8.208763	8.278347	0.07286	0.56404	0.19027	0.154759	-0.06958	0.581334	0.259854	0.064182	-0.11741	0.360644	0.142443	0.273281	6824223	8.246599	8.430969	8.376051	8.376888	8.657413	8.37155	8.372293	8.209795	8.044201	8.095979	8.348955	8.390106	Fermt2	NM_146054
6824229	4599	8.641063	8.72553	8.543709	8.42948	0.211582	0.092461	0.29605	0.028222	0.114229	0.332509	0.181821	0.139266	-0.08447	0.467532	0.097354	0.404998	6824229	8.588121	8.616613	8.718454	8.558981	8.985542	8.632067	8.649912	8.412069	8.569146	8.38572	8.479486	8.423235	Ddhd1	NM_001042719
6824266	4600	7.612792	7.592141	7.158914	8.236609	-0.62382	0.173411	-0.64447	0.16118	-1.0777	0.032524	0.433227	0.329678	0.020651	0.961754	0.453878	0.308554	6824266	7.270343	8.220744	7.347289	7.163465	8.093185	7.519772	7.272765	7.046973	7.157003	8.57408	8.737615	7.398132	Bmp4	NM_007554
6824286	4601	9.556905	9.60426	9.519263	9.605081	-0.04818	0.724278	-0.00082	0.99518	-0.08582	0.533344	0.084997	0.537173	-0.04735	0.728758	0.037642	0.782506	6824286	9.555583	9.494647	9.620485	9.659307	9.581518	9.571954	9.748945	9.63367	9.175173	9.565135	9.556344	9.693765	Cnih	NM_009919
6824291	4602	8.411862	8.6736	8.492503	8.583589	-0.17173	0.359066	0.090011	0.623832	-0.09109	0.619761	0.181097	0.334887	-0.26174	0.176375	-0.08064	0.659897	6824291	8.362749	8.344371	8.528466	8.551214	8.756649	8.712936	8.827384	8.391846	8.258279	8.416638	8.431601	8.902527	Gmfb	NM_022023
6824321	4603	7.083565	7.28791	7.13858	7.60962	-0.52606	0.008459	-0.32171	0.06669	-0.47104	0.014534	0.14933	0.353634	-0.20435	0.214762	-0.05502	0.726179	6824321	7.351408	6.935569	6.963717	7.276257	7.393977	7.193496	7.039096	7.398387	6.978258	7.523431	7.523389	7.782041	Gch1	NM_008102
6824325	4604	5.934588	6.117536	5.8581	5.688327	0.246261	0.292911	0.42921	0.085386	0.169773	0.460023	0.259437	0.26966	-0.18295	0.427247	0.076488	0.735626	6824325	5.856222	5.827822	6.119719	5.844819	6.377584	6.130206	6.290957	5.457898	5.825444	5.544652	5.741302	5.779026	Wdhd1	NM_172598
6824346	4605	5.873779	6.252509	5.654071	5.138204	0.735575	0.092582	1.114305	0.02011	0.515868	0.217289	0.598437	0.158881	-0.37873	0.354292	0.219708	0.584075	6824346	5.866292	5.495839	6.259206	5.870899	6.914865	5.971762	6.003179	4.923474	6.035561	5.010737	5.213996	5.189878	Dlgap5	NM_001145949

6824363	4606	6.571045	6.769551	6.69728	6.630183	-0.05914	0.588263	0.139368	0.220507	0.067097	0.540177	0.072271	0.510213	-0.19851	0.095001	-0.12623	0.263097	6824363	6.395995	6.700058	6.617082	6.775416	6.776557	6.75668	6.800441	6.485287	6.806111	6.56566	6.59671	6.728178	D14Ertd4	NM_172599
6824477	4608	8.480884	8.591652	8.471684	8.439024	0.04186	0.672252	0.152628	0.148094	0.03266	0.740767	0.119967	0.243788	-0.11077	0.278827	0.0092	0.925504	6824477	8.547938	8.431296	8.463419	8.477112	8.69506	8.602783	8.682457	8.406648	8.325948	8.366013	8.47692	8.474139	Exoc5	NM_207214
6824550	4622	6.588553	6.970026	6.735695	6.445093	0.14346	0.546185	0.524933	0.050025	0.290602	0.237611	0.234331	0.333468	-0.38147	0.132353	-0.14714	0.536183	6824550	6.612444	6.639206	6.514009	7.056786	6.915885	6.937407	6.477324	6.956133	6.773628	6.584554	6.852762	5.897962	Ttc5	NM_177625
6824553	4623	7.361812	7.268612	7.334169	7.100002	0.26181	0.000705	0.16861	0.008942	0.234167	0.001418	-0.06556	0.218928	0.0932	0.094475	0.027643	0.589185	6824553	7.409248	7.346771	7.329417	7.255953	7.308074	7.241809	7.391605	7.220185	7.390717	7.05312	7.134235	7.112651	Tep1	NM_009351
6824557	4625	6.395262	6.395888	6.448796	6.413755	-0.01849	0.892123	-0.01787	0.895747	0.035042	0.797497	-0.05291	0.699238	-0.00063	0.996337	-0.05353	0.695891	6824557	6.425015	6.495608	6.265164	6.336946	6.470726	6.379992	6.402784	6.682157	6.261448	6.520721	6.538219	6.182324	Osgep	NM_133676
6824574	4628	6.120811	5.444397	5.848336	5.567436	0.553375	0.0322	-0.12304	0.580786	0.2809	0.225324	-0.40394	0.095526	0.676414	0.013319	0.272475	0.238281	6824574	6.110171	6.146783	6.105479	5.272439	5.574648	5.486104	5.67187	5.468756	6.404382	5.553046	5.659822	5.48944	Rnase1	NM_011271
6824610	4631	8.711526	9.194072	8.616121	8.976946	-0.26542	0.628329	0.217126	0.691364	-0.36082	0.513186	0.577951	0.305017	-0.48255	0.386946	0.095405	0.860942	6824610	8.076051	8.066025	9.992503	9.612	8.979299	8.990917	9.014768	8.372991	8.460605	8.507738	9.067272	9.355829	Ndrg2	NM_013864
6824620	4632	7.746328	7.833382	7.796923	7.757524	-0.0112	0.890061	0.075857	0.361984	0.039398	0.629121	0.036459	0.654571	-0.08705	0.299474	-0.0506	0.537107	6824620	7.74685	7.765723	7.72641	7.869175	7.962425	7.668545	7.770294	7.925324	7.69515	7.740219	7.764802	7.767552	Zfp219	NM_027248
6824621	4633	6.339275	6.326708	6.434647	6.385125	-0.04585	0.832146	-0.05842	0.787315	0.049521	0.818973	-0.10794	0.620124	0.012568	0.953609	-0.09537	0.660856	6824621	6.588838	6.042798	6.38619	6.439363	6.2735	6.26726	6.673831	6.048395	6.581714	6.550914	6.508826	6.095636	Olfr1514	NM_207576
6824624	4634	8.233674	7.917435	8.270431	7.887789	0.345885	0.122984	0.029646	0.88618	0.382642	0.092919	-0.353	0.116533	0.316239	0.153607	-0.03676	0.859188	6824624	8.362932	8.231874	8.106216	7.61201	8.292868	7.847426	8.393253	8.398996	8.019043	7.619582	8.085857	7.957927	Hnrnpc	NM_016884
6824633	4635	6.758247	7.242385	6.861395	6.83389	-0.07564	0.594619	0.408495	0.017266	0.027505	0.84534	0.38099	0.023519	-0.48414	0.007545	-0.10315	0.471519	6824633	6.748879	6.753781	6.77208	7.229942	7.441276	7.055938	6.807842	7.015983	6.76036	6.622151	6.790433	7.089087	Sall2	NM_015772
6824634	4636	8.942787	9.02943	8.946803	8.719377	0.22341	0.085463	0.310053	0.026161	0.227426	0.080925	0.082627	0.488856	-0.08664	0.468663	-0.00402	0.972739	6824634	8.948542	8.956146	8.923674	8.884424	9.147583	9.056284	9.163154	8.730124	8.947131	8.60687	8.833282	8.717979	Supt16h	NM_033618
6824641	4637	8.248088	8.302877	8.323472	8.198792	0.049296	0.562142	0.104085	0.237499	0.12468	0.164695	-0.02059	0.806925	-0.05479	0.520471	-0.07538	0.382167	6824641	8.299681	8.22558	8.219002	8.272368	8.470204	8.166059	8.321418	8.394487	8.254511	8.169404	8.305656	8.121316	Chd8	NM_201637
6824648	4638	8.404106	8.538639	8.368338	8.38617	0.017936	0.810918	0.152469	0.068712	-0.01783	0.81199	0.1703	0.04683	-0.13453	0.100727	0.035767	0.635171	6824648	8.508511	8.360706	8.3431	8.482037	8.644226	8.489653	8.363874	8.436749	8.304392	8.31484	8.503843	8.339827	Chd8	NM_201637
6824662	4639	7.478464	6.959751	7.290447	7.767673	-0.28921	0.380161	-0.80792	0.031881	-0.47723	0.163906	-0.3307	0.319222	0.518713	0.134302	0.188017	0.562686	6824662	7.379171	7.723845	7.332377	6.785343	7.277	6.816911	6.594556	7.824557	7.452228	7.893181	7.929917	7.479922	Rab2b	NM_172601
6824668	4640	8.115384	8.146018	8.161534	8.071036	0.044348	0.623789	0.074982	0.413587	0.090498	0.32839	-0.01552	0.862806	-0.03063	0.733698	-0.04615	0.609991	6824668	8.019096	8.147633	8.179424	7.992306	8.216207	8.229542	8.176009	8.225586	8.083008	7.954999	8.200129	8.057981	Mettl3	NM_019721
6824679	4644	6.163226	6.253887	6.487018	6.410164	-0.24694	0.209609	-0.15628	0.413043	0.076854	0.682307	-0.23313	0.233739	-0.09066	0.629932	-0.32379	0.111421	6824679	5.873541	6.450889	6.165247	6.095226	6.397578	6.268857	6.316606	6.448143	6.696306	6.573723	6.509943	6.146826	Olfr1508	NM_020513
6824719	4646	13.21325	13.13347	13.28842	13.39729	-0.18404	0.026347	-0.26382	0.004572	-0.10887	0.146578	-0.15495	0.051422	0.079783	0.272609	-0.07517	0.299274	6824719	13.24069	13.30777	13.0913	13.11289	13.16244	13.12508	13.36573	13.3412	13.15833	13.34929	13.42183	13.42076	Dad1	NM_010015
6824728	4649	8.287188	7.974607	8.281436	8.402113	-0.11492	0.710152	-0.42751	0.189819	-0.12068	0.696482	-0.30683	0.333881	0.312581	0.325437	0.005752	0.985091	6824728	8.230602	8.491448	8.139514	7.718467	8.164798	8.040556	8.51615	7.819622	8.508535	7.882613	8.954032	8.369694	Oxa1l	NM_026936
6824738	4650	9.72026	9.703521	9.850139	9.786436	-0.06618	0.652282	-0.08292	0.573804	0.063703	0.664316	-0.14662	0.330129	0.016739	0.90869	-0.12988	0.385227	6824738	9.611862	9.689632	9.859285	9.49598	9.894491	9.720091	9.896072	9.590354	10.06399	9.694892	9.842691	9.821725	Prmt5	NM_013768
6824740	4651	6.251632	6.563362	6.175746	6.111003	0.140629	0.401193	0.452359	0.021423	0.064743	0.693873	0.387616	0.04034	-0.31173	0.084977	0.075886	0.645173	6824740	6.372314	6.025497	6.357086	6.603058	6.585668	6.501361	6.476782	5.938021	6.112436	6.038953	5.973415	6.320642	Haus4	BC011095
6824743	4652	6.845573	7.369042	6.6813	6.32096	0.524613	0.037474	1.048083	0.001085	0.360341	0.12547	0.687742	0.011436	-0.52347	0.037793	0.164273	0.45787	6824743	6.85886	6.922702	6.755157	7.348218	7.748202	7.010707	6.749962	6.657493	6.636446	6.13889	6.718918	6.105071	Jub	NM_010590
6824755	4654	8.697604	8.953587	8.615647	8.650606	0.046998	0.626102	0.302981	0.011427	-0.03496	0.716101	0.33794	0.006563	-0.25598	0.024703	0.081957	0.402774	6824755	8.727858	8.602716	8.762239	8.806236	9.069074	8.985451	8.602532	8.752889	8.491521	8.580803	8.610027	8.760989	Acin1	NM_023190
6824763	4656	7.481296	7.354857	7.683916	6.563635	0.917661	0.010795	0.791222	0.021521	1.120281	0.00377	-0.32906	0.270118	0.126439	0.661042	-0.20262	0.486496	6824763	7.825048	7.389171	7.22967	7.421478	7.828318	6.814775	7.661139	7.566379	7.824229	6.534794	6.878211	6.2779	Slc7a8	NM_016972
6824768	4657	7.39078	7.57737	7.608732	7.632779	-0.242	0.013518	-0.05541	0.490779	-0.02405	0.761989	-0.03136	0.69346	-0.18659	0.041087	-0.21795	0.021795	6824768	7.452561	7.427128	7.292651	7.57507	7.684958	7.472082	7.629445	7.582894	7.613856	7.65303	7.497199	7.748108	Homez	NM_183174
6824779	4658	8.371909	8.434966	8.321316	8.362134	0.009775	0.904214	0.072832	0.381781	-0.04082	0.618019	0.11365	0.186687	-0.06306	0.446113	0.050593	0.538279	6824779	8.376593	8.354476	8.384658	8.363605	8.555902	8.385391	8.249619	8.486702	8.227628	8.439575	8.349732	8.297096	Slc22a17	NM_021551
6824799	4661	7.515776	7.650967	7.596029	7.520332	-0.00456	0.963176	0.130635	0.209127	0.075697	0.451489	0.054938	0.581463	-0.13519	0.195204	-0.08025	0.425766	6824799	7.44344	7.600843	7.503046	7.642039	7.808993	7.501869	7.582201	7.51365	7.692235	7.430918	7.669019	7.461059	Ap1g2	NM_007455
6824800	4662	6.168295	6.138965	6.095135	6.151412	0.016883	0.846129	-0.01245	0.886164	-0.05628	0.522824	0.04383	0.616882	0.029331	0.736644	0.07316	0.410348	6824800	6.300152	6.223871	5.980863	6.069789	6.163362	6.183743	6.0486	6.185596	6.051209	6.218467	6.078443	6.157327	Jph4	NM_177049
6824825	4664	6.664217	6.638502	6.705762	6.702345	-0.03813	0.817815	-0.06384	0.7006	0.003417	0.983501	-0.06726	0.685569	0.025714	0.876423	-0.04154	0.801877	6824825	6.647207	6.663876	6.681567	6.793981	6.5162	6.605326	6.532581	7.06987	6.514834	6.50963	6.876982	6.720422	Fam158a	AB054001
6824826	4665	7.008052	7.070989	7.036114	7.061388	-0.05334	0.705999	0.009601	0.945615	-0.02527	0.857618	0.034875	0.804657	-0.06294	0.656803	-0.02806	0.842146	6824826	7.143029	6.878178	7.00295	7.242344	6.810051	7.160573	7.159652	7.089055	6.859636	6.956797	7.021284	7.206084	Psme2	NM_011190
6824829	4666	8.285983	8.204374	8.243379	8.028732	0.257251	0.036501	0.175642	0.125222	0.214647	0.069757	-0.03901	0.713675	0.081609	0.449261	0.042604	0.688831	6824829	8.319478	8.265422	8.27305	8.262818	8.28938	8.060925	8.457061	8.047325	8.225752	7.968358	8.100602	8.017237	Ipo4	NM_024267
6824830	4667	8.26193	8.266267	8.286271	8.219004	0.042926	0.583861	0.047263	0.547271	0.067267	0.397249	-0.02	0.796998	-0.00434	0.955432	-0.02434	0.754525	6824830	8.306026	8.193946	8.285818	8.236699	8.32295	8.239152	8.39709	8.323647	8.138075	8.117	8.221636	8.318376	Tm9sf1	NM_028780
6824833	4668	7.946739	7.953448	7.902616	7.881885	0.064853	0.452854	0.071563	0.409318	0.020731	0.807235	0.050832	0.553494	-0.00671	0.936949	0.044122	0.606012	6824833	7.963261	7.890489	7.986466	7.794988	8.157877	7.907479	7.871389	7.937582	7.898878	7.858673	7.938354	7.848629	1810034K	NM_023397
6824834	4669	10.12371	10.15807	10.12382	9.963059	0.160654	0.293983	0.195014	0.209929	0.160765	0.293673	0.034249	0.816799	-0.03436	0.816219	-0.00011	0.999402	6824834	10.13831	10.04466	10.18817	10.04836	10.33996	10.0859	10.29906	10.20497	9.867442	9.83188	9.861648	10.19565	Nedd8	NM_008683
6824835	4670	7.680418	7.853675	7.834678	7.766664	-0.08625	0.60365	0.087011	0.6005	0.068014	0.681237	0.018997	0.908189	-0.17326	0.309303	-0.15426	0.362088	6824835	7.780113	7.580313	7.680828	7.550524	8.160342	7.850159	8.063437	7.681703	7.758894	7.657864	7.837315	7.804813	Tinf2	NM_145705
6824838	4671	10.02786	9.855421	9.639562	10.11932	-0.09147	0.741929	-0.2639	0.354083	-0.47976	0.111532	0.215859	0.444302	0.172434	0.538374	0.388293	0.185826	6824838	9.883116	10.16306	10.03739	9.366463	10.19931	10.00049	9.834864	9.198005	9.885818	10.07325	10.41535	9.86937	Tgm1	NM_019984
6824840	4672	8.742501	8.773325	8.812305	8.889804	-0.1473	0.007317	-0.11648	0.02245	-0.0775	0.097335	-0.03898	0.37273	-0.03082	0.476684	-0.0698	0.129355	6824840	8.677858	8.734102	8.815544	8.764456	8.837187	8.718331	8.832117	8.820568	8.78423	8.906924	8.849621	8.912868	Rabggta	NM_019519
6824841	4673	10.64039	10.52901	10.43448	10.68538	-0.04499	0.833751	-0.15637	0.472623	-0.25091	0.26104	0.094538	0.660736	0.11138	0.605973	0.205918	0.350007	6824841	10.71691	10.54179	10.66248	10.35405	10.71183	10.52116	10.81377	9.929235	10.56042	10.56629	10.73838	10.75148	Dhrs1	NM_026819
6824842	4674	7.224445	7.321031	7.256353	7.900727	-0.67628	0.01224	-0.5797	0.024672	-0.64437	0.015394	0.064679	0.765985	-0.09659	0.657847	-0.03191	0.88301	6824842	7.439488	7.180838	7.053008	7.562345	7.154139	7.24661	7.206826	7.135949	7.426283	7.564983	7.799086	8.338111	2610027L	NM_026403
6824844	4675	7.74658	8.033958	7.663838	7.470955	0.275625	0.287489	0.563003	0.048354	0.192883	0.44825	0.37012	0.164528	-0.28738	0.268898	0.082742	0.741122	6824844	7.915751	7.748073	7.575915	8.107156	8.063478	7.931239	7.981849	7.283878	7.725786	6.970679	7.75911	7.683075	Adcy4	NM_080435
6824846	4676	7.827782	7.667493	7.418689	7.009501	0.818281	0.027302	0.657992	0.062132	0.409188	0.214752	0.248804	0.436337	0.160289	0.611941	0.409093	0.214848	6824846	8.055737	7.648675	7.778934	7.424337	8.07868	7.499463	7.803231	6.843366	7.609471	6.961282	7.383621	6.683601	Ripk3	NM_019955
6824850	4678	7.710159	7.647522	7.889538	7.754356	-0.0442	0.801182	-0.10683	0.546712	0.135183	0.44886	-0.24202	0.191828	0.062636	0.721742	-0.17938	0.321555	6824850	7.710165	7.65858	7.761731	7.813256	7.742002	7.387309	7.88028	8.180133	7.608202	7.576389	7.730973	7.955705	Sdr39u1	NM_001082975
6824893	4688	6.233628	6.323141	6.202477	6.173059	0.060569	0.572535	0.150082	0.182997	0.029418	0.782329	0.120664	0.274885	-0.08951	0.409908	0.031151	0.769921	6824893	6.080744	6.398386	6.221755	6.222522	6.265609	6.481293	6.297578	6.080966	6.228887	6.10462	6.150079	6.264479	Cenpj	NM_001014996
6824902	4689	7.396218	7.497048	7.27602	7.522934	-0.12672	0.096666	-0.02589	0.710707	-0.24691	0.006342	0.221028	0.011154	-0.10083	0.172709	0.120199	0.11212	6824902	7.403795	7.43141	7.35345	7.42126	7.557714	7.512169	7.30492	7.35136	7.171779	7.451425	7.467591	7.649786	Pspc1	NM_025682
6824915	4690	8.581945	8.716198	8.535347	8.694559	-0.11261	0.23858	0.021639	0.812835	-0.15921	0.109468	0.180851	0.075063	-0.13425	0.167429	0.046597	0.612513	6824915	8.545621	8.635664	8.564549	8.610841	8.79212	8.745633	8.415824	8.744994	8.445224	8.713635	8.636619	8.733422	Zmym5	NM_144842
6824942	4693	6.725704	6.866588	6.798622	6.920695	-0.19499	0.21545	-0.05411	0.718649	-0.12207	0.424166	0.067966	0.651667	-0.14088	0.359556	-0.07292	0.628491	6824942	6.640525	6.780008	6.756579	6.914265	6.849669	6.835831	6.797394	6.835242	6.76323	6.588264	6.901008	7.272812	Cryl1	NM_030004
6824961	4694	7.163229	7.280729	7.305934	7.303505	-0.14028	0.355353	-0.02278	0.877406	0.00243	0.98686	-0.0252	0.864472	-0.1175	0.435065	-0.14271	0.347536	6824961	7.07909	7.318846	7.091751	7.242333	7.436983	7.162872	7.307158	7.513058	7.097587	7.116442	7.273731	7.520341	N6amt2	NM_026526
6824962	4695	7.371379	7.51357	7.537195	7.526462	-0.15508	0.165898	-0.01289	0.902287	0.010734	0.918567	-0.02363	0.822186	-0.14219	0.19973	-0.16582	0.141753	6824962	7.398448	7.465921	7.249768	7.405138	7.656945	7.478628	7.620915	7.64178	7.348891	7.460391	7.503654	7.61534	Xpo4	NM_020506
6824967	4696	7.753543	7.801204	7.483703	7.607604	0.14594	0.018702	0.1936	0.004541	-0.1239	0.037158	0.317501	0.00021	-0.04766	0.36506	0.269841	0.000619	6824967	7.783513	7.716	7.761117	7.867205	7.785582	7.750825	7.484272	7.524472	7.442364	7.503853	7.675948	7.64301	Lats2	NM_015771
6824980	4698	9.188044	9.325899	9.277389	9.072157	0.115887	0.334065	0.253742	0.054511	0.205232	0.106189	0.04851	0.678351	-0.13785	0.256219	-0.08934	0.45094	6824980	9.113291	9.240353	9.210489	9.126801	9.33089	9.520005	9.41766	9.164332	9.250175	8.930339	9.104409	9.181723	Zdhhc20	NM_029492
6824986	4699	8.21376	8.336173	8.27578	8.213382	0.000378	0.997516	0.122791	0.327313	0.062398	0.610393	0.060393	0.62173	-0.12241	0.328714	-0.06202	0.612521	6824986	8.135063	8.28289	8.223326	8.36032	8.241514	8.406684	8.499309	8.089576	8.238455	8.033468	8.248267	8.35841	Efha1	NM_028643
6825096	4702	10.51799	10.2484	10.3602	10.24137	0.276627	0.285495	0.007037	0.977487	0.118833	0.636164	-0.1118	0.656009	0.26959	0.297059	0.157793	0.532158	6825096	10.42738	10.27412	10.85248	10.18468	10.50254	10.05799	10.12511	10.07306	10.88243	10.28129	10.17834	10.26447	Phf11	NM_172603
6825119	4703	8.219905	8.300366	8.278176	8.313905	-0.094	0.446884	-0.01354	0.911125	-0.03573	0.768866	0.02219	0.854933	-0.08046	0.512896	-0.05827	0.633336	6825119	8.169819	8.305204	8.184691	8.197747	8.382854	8.320498	8.381339	8.072953	8.380236	8.146019	8.272329	8.523366	Cdadc1	NM_027986
6825137	4705	8.54806	8.584241	8.527779	8.369655	0.178405	0.026395	0.214586	0.011402	0.158123	0.042668	0.056462	0.414972	-0.03618	0.59675	0.020281	0.765358	6825137	8.615584	8.495481	8.533115	8.482865	8.720202	8.549656	8.604492	8.510114	8.46873	8.362616	8.420594	8.325756	Nupl1	NM_170591
6825207	4708	6.523081	6.541475	6.57167	6.665539	-0.14246	0.158758	-0.12406	0.212903	-0.09387	0.335772	-0.03019	0.750306	-0.01839	0.845965	-0.04859	0.61045	6825207	6.522775	6.600905	6.445564	6.38126	6.547005	6.696161	6.568371	6.622813	6.523826	6.520817	6.700477	6.775322	Ebpl	NM_026598
6825216	4709	8.887186	8.943709	8.963513	8.762956	0.12423	0.160553	0.180753	0.054548	0.200557	0.037128	-0.0198	0.811466	-0.05652	0.501609	-0.07633	0.369827	6825216	8.848918	8.898701	8.91394	8.91758	9.082598	8.830948	9.050648	8.953901	8.88599	8.632353	8.867924	8.788591	Kpna3	NM_008466
6825223	4710	8.48228	8.542137	8.47612	8.528077	-0.0458	0.637883	0.014059	0.884358	-0.05196	0.594121	0.066017	0.500772	-0.05986	0.54051	0.006161	0.949154	6825223	8.509855	8.544535	8.392451	8.657335	8.470963	8.498112	8.585026	8.559623	8.28371	8.452874	8.502897	8.628461	6330409N	NM_025697
6825255	4711	6.03431	6.3036	6.035357	6.258207	-0.2239	0.056315	0.045394	0.663218	-0.22285	0.057238	0.268244	0.028298	-0.26929	0.027845	-0.00105	0.991941	6825255	6.050659	5.979837	6.072435	6.417756	6.343081	6.149964	6.12984	5.821681	6.154549	6.280008	6.312888	6.181724	Dleu7	NM_173419
6825273	4713	8.236878	8.249175	8.496266	8.080345	0.156533	0.340111	0.16883	0.305787	0.415921	0.027277	-0.24709	0.148009	-0.0123	0.938447	-0.25939	0.131301	6825273	8.255895	8.171422	8.283318	7.8925	8.236211	8.618814	8.566419	8.407117	8.515263	8.075504	8.053697	8.111834	Ints6	NM_008715
6825302	4714	9.31	9.334114	9.431286	9.245052	0.064948	0.748285	0.089062	0.660842	0.186234	0.368719	-0.09717	0.632556	-0.02411	0.904882	-0.12129	0.552291	6825302	9.178778	9.258698	9.492523	9.102348	9.551432	9.348562	9.547526	9.302789	9.443542	9.042007	9.672446	9.020703	Fdft1	NM_010191
6825305	4716	6.454113	6.410605	6.482464	6.545632	-0.09152	0.229976	-0.13503	0.091504	-0.06317	0.395951	-0.07186	0.337437	0.043508	0.553903	-0.02835	0.697819	6825305	6.420032	6.563649	6.378657	6.43374	6.319075	6.478999	6.521921	6.552582	6.372888	6.596782	6.56839	6.471723	Gata4	NM_008092

6825325	4719	8.177927	8.210599	8.067895	7.936221	0.241706	0.023085	0.274377	0.012957	0.131673	0.165262	0.142704	0.136521	-0.03267	0.714619	0.110033	0.237723	6825325	8.226553	8.151278	8.155951	8.247729	8.356297	8.02777	8.129463	8.046643	8.027578	7.90587	8.057179	7.845615	Mtmr9	NM_177594
6825371	4721	7.22068	7.322038	7.230021	7.342388	-0.12171	0.199334	-0.02035	0.820907	-0.11237	0.232515	0.092017	0.32104	-0.10136	0.277511	-0.00933	0.917131	6825371	7.167751	7.217638	7.27665	7.303962	7.274111	7.388041	7.30837	7.347904	7.033788	7.302807	7.27001	7.454348	Msra	NM_026322
6825390	4722	7.598742	7.725701	7.575432	7.704382	-0.10564	0.327242	0.021319	0.838485	-0.12895	0.23853	0.150268	0.176035	-0.12696	0.245235	0.02331	0.823684	6825390	7.641534	7.697402	7.45729	7.707396	7.771748	7.697958	7.465361	7.778522	7.482414	7.572362	7.765882	7.774902	Hmbox1	NM_177338
6825402	4723	8.937258	8.770337	8.782804	8.509423	0.427835	0.007351	0.260914	0.061448	0.273381	0.052256	-0.01247	0.919824	0.166922	0.201765	0.154454	0.234125	6825402	9.045223	8.875595	8.890957	8.628963	8.989715	8.692332	8.848998	8.660329	8.839086	8.556879	8.651735	8.319655	Extl3	NM_018788
6825410	4724	6.798316	6.698764	6.518239	6.826982	-0.02867	0.873664	-0.12822	0.483713	-0.30874	0.114995	0.180525	0.331419	0.099551	0.584226	0.280076	0.147368	6825410	6.86856	6.655665	6.870722	6.659051	6.957429	6.479813	6.788312	6.503253	6.263153	7.055143	6.748756	6.677046	Fzd3	NM_021458
6825429	4726	8.156563	8.166553	8.262774	8.233065	-0.0765	0.470128	-0.06651	0.528341	0.029709	0.77594	-0.09622	0.368249	-0.00999	0.923571	-0.10621	0.323313	6825429	8.03766	8.225009	8.207019	8.107293	8.137095	8.255272	8.449407	8.063223	8.275692	8.144627	8.245438	8.309129	Elp3	NM_028811
6825439	4728	7.271656	7.128039	6.667843	6.473777	0.79788	0.041434	0.654262	0.081817	0.194066	0.571368	0.460196	0.199228	0.143617	0.673842	0.603813	0.103642	6825439	7.55141	7.09858	7.164979	6.951527	7.753994	6.678596	7.006927	6.5205	6.476102	6.050379	6.920684	6.450267	Scara3	NM_172604
6825443	4731	7.207678	6.798318	7.24833	7.371306	-0.16363	0.606475	-0.57299	0.097314	-0.12298	0.697572	-0.45001	0.178613	0.40936	0.216692	-0.04065	0.897333	6825443	7.210237	7.253023	7.159773	7.331438	6.982872	6.080644	6.972298	7.569952	7.202741	7.176015	7.325194	7.612709	Ephx2	NM_007940
6825445	4732	8.668228	8.272967	8.506567	8.197288	0.47094	0.002825	0.075679	0.514579	0.309279	0.023682	-0.2336	0.068432	0.395262	0.007387	0.161661	0.183337	6825445	8.745608	8.57641	8.682667	8.305969	8.430617	8.082314	8.582478	8.445656	8.491568	8.398088	8.115919	8.077857	Ptk2b	NM_172498
6825494	4733	11.46601	11.23527	11.42969	11.40101	0.065003	0.633234	-0.16574	0.2416	0.028683	0.832237	-0.19442	0.176234	0.23074	0.116336	0.03632	0.788703	6825494	11.23335	11.60136	11.56332	11.44307	11.14598	11.11676	11.33821	11.51226	11.4386	11.24974	11.5452	11.40808	Dpysl2	NM_009955
6825509	4734	6.746959	6.69286	6.749281	6.692639	0.05432	0.832183	0.000221	0.999312	0.056642	0.825147	-0.05642	0.825815	0.0541	0.832853	-0.00232	0.992763	6825509	6.400739	6.772613	7.067526	6.869678	6.858807	6.350094	7.224051	6.530998	6.492794	6.69368	6.704236	6.680001	Bnip3l	NM_009761
6825511	4735	8.54573	8.602767	8.510499	8.469174	0.076556	0.369423	0.133593	0.135574	0.041325	0.621563	0.092268	0.284822	-0.05704	0.498719	0.035231	0.673197	6825511	8.563703	8.527788	8.5457	8.48201	8.757577	8.568714	8.590364	8.545204	8.395929	8.416481	8.574669	8.416373	Ppp2r2a	NM_028032
6825553	4737	8.272083	8.14994	8.232996	8.257479	0.014604	0.923032	-0.10754	0.483779	-0.02448	0.871392	-0.08306	0.586231	0.122143	0.428521	0.039087	0.796323	6825553	8.133389	8.188995	8.493865	8.178865	8.112581	8.158374	8.064611	8.530046	8.10433	8.282146	8.093908	8.396384	Dock5	NM_177780
6825573	4738	6.047192	6.0725	6.051737	6.095414	-0.04822	0.65373	-0.02291	0.83035	-0.04368	0.684165	0.020763	0.846025	-0.02531	0.812999	-0.00454	0.966053	6825573	6.025287	6.072096	6.044194	6.031931	6.013742	6.171827	6.113752	6.05724	5.98422	6.279024	5.840069	6.167148	Nefm	NM_008691
6825638	4742	7.593587	7.795438	7.677209	7.843866	-0.25028	0.233946	-0.04843	0.809554	-0.16666	0.416238	0.118229	0.559948	-0.20185	0.329491	-0.08362	0.678445	6825638	7.726561	7.614137	7.440064	7.831486	7.933053	7.621775	7.565722	7.955729	7.510176	7.597938	7.690401	8.243258	Slc25a37	NM_026331
6825653	4744	7.742817	7.904543	7.770162	7.761596	-0.01878	0.722805	0.142947	0.023298	0.008566	0.871042	0.134381	0.030198	-0.16173	0.013301	-0.02734	0.607141	6825653	7.810484	7.635371	7.782597	7.817516	7.940545	7.955568	7.799452	7.768043	7.742991	7.745833	7.779126	7.759829	Chmp7	NM_134078
6825657	4745	7.078256	7.110515	7.015764	7.019994	0.058262	0.586118	0.090521	0.403853	-0.00423	0.968154	0.094752	0.38325	-0.03226	0.761499	0.062492	0.559783	6825657	7.054763	7.102911	7.077094	7.09204	7.234217	7.005289	7.141302	7.118258	6.787731	6.904753	7.061235	7.093995	Rhobtb2	NM_153514
6825678	4746	8.290584	8.288066	8.255806	8.210078	0.080505	0.339656	0.077988	0.354117	0.045728	0.579983	0.03226	0.694767	0.002517	0.97545	0.034778	0.672537	6825678	8.285705	8.270402	8.315644	8.273767	8.408068	8.182364	8.367798	8.268654	8.130966	8.09395	8.274487	8.261798	2610301G	NM_146055
6825679	4747	6.168898	6.519101	6.261629	6.237462	-0.06856	0.632718	0.281639	0.07561	0.024167	0.865355	0.257472	0.099098	-0.3502	0.034857	-0.09273	0.5206	6825679	6.01506	6.032005	6.459629	6.651286	6.537752	6.368265	6.205189	6.220297	6.359401	6.218786	6.398656	6.094944	9930012K	NM_001004155
6825683	4748	8.638188	8.606706	8.433174	8.834331	-0.19614	0.163178	-0.22825	0.111756	-0.40116	0.01379	0.172912	0.212816	0.032102	0.807893	0.205014	0.147142	6825683	8.529828	8.641011	8.743724	8.859262	8.401912	8.557083	8.394984	8.573838	8.3307	8.95822	8.842846	8.701926	Pdlim2	NM_145978
6825684	4749	9.112537	9.098059	9.071905	9.379383	-0.26685	0.105998	-0.28132	0.091061	-0.30748	0.069053	0.026154	0.86274	0.014477	0.923708	0.040632	0.788534	6825684	8.996232	9.119321	9.222057	9.356857	9.035378	8.901943	8.912517	9.225836	9.077362	9.500869	9.158452	9.478828	Sorbs3	NM_011366
6825686	4750	8.655233	8.886095	8.826355	8.826507	-0.17127	0.270427	0.059588	0.691228	-0.00015	0.999187	0.05974	0.690489	-0.23086	0.149196	-0.17112	0.27082	6825686	8.45573	8.754277	8.755692	8.794149	8.669237	9.1949	8.799272	8.775579	8.904214	8.711249	8.808979	8.959293	Ppp3cc	NM_008915
6825688	4751	10.36916	10.51112	10.26772	10.57964	-0.21048	0.146574	-0.06852	0.614904	-0.31192	0.044373	0.2434	0.100066	-0.14196	0.309821	0.10144	0.460736	6825688	10.1795	10.3809	10.54708	10.44844	10.37747	10.70745	10.23336	10.19289	10.37691	10.68633	10.38171	10.67088	Slc39a14	NM_144808
6825696	4753	7.310472	7.348914	7.28683	7.221316	0.089156	0.503244	0.127598	0.345187	0.065514	0.620458	0.062084	0.638598	-0.03844	0.770204	0.023642	0.857179	6825696	7.307507	7.292123	7.331786	7.265185	7.545822	7.235734	7.335144	7.516212	7.009133	7.208789	7.262765	7.192394	Polr3d	NM_025945
6825704	4754	8.266731	8.34997	7.864196	7.583911	0.68282	0.037771	0.766059	0.023603	0.280285	0.337421	0.485774	0.114962	-0.08324	0.769601	0.402535	0.180978	6825704	8.528738	8.232431	8.039024	8.097352	8.953699	7.998858	8.174116	7.692366	7.726105	7.36858	7.786498	7.596655	Bmp1	NM_009755
6825713	4756	8.16326	8.137295	8.178686	7.878177	0.285083	0.279007	0.259117	0.322027	0.300508	0.255737	-0.04139	0.870293	0.025966	0.918369	-0.01543	0.95144	6825713	8.161761	7.929248	8.398772	8.238007	8.078183	8.095694	8.255812	8.31575	7.964495	7.498094	7.674332	8.462106	Epb4.9	NM_013514
6825714	4757	6.37268	6.467478	6.312943	6.484459	-0.11178	0.291867	-0.01698	0.868151	-0.17152	0.121627	0.154535	0.15739	-0.0948	0.366618	0.059737	0.563196	6825714	6.365714	6.369181	6.383145	6.377629	6.401348	6.623457	6.47895	6.169963	6.289915	6.623457	6.455611	6.37431	Fgf17	NM_008004
6825717	4759	8.197103	8.259645	8.27127	8.134077	0.063026	0.583787	0.125568	0.288337	0.137193	0.249213	-0.01163	0.918737	-0.06254	0.586628	-0.07417	0.520675	6825717	8.077919	8.221464	8.291926	8.115402	8.460564	8.202968	8.39951	8.264811	8.149489	8.015279	8.246504	8.140447	Xpo7	NM_023045
6825853	4760	10.92971	10.75785	11.15544	11.39229	-0.46258	0.070174	-0.63444	0.021006	-0.23685	0.316099	-0.39759	0.110375	0.171853	0.460117	-0.22573	0.337942	6825853	10.85953	11.20754	10.72205	10.40705	10.75858	11.10793	11.24144	11.14158	11.0833	11.57116	11.58305	11.02266	Fndc3a	NM_207636
6825875	4762	8.598628	8.427614	8.578639	8.186842	0.411785	0.062372	0.240772	0.241318	0.391796	0.073449	-0.15102	0.450235	0.171014	0.394996	0.019989	0.918975	6825875	8.948971	8.462979	8.383933	8.490262	8.557912	8.234668	8.832327	8.434353	8.469236	7.939569	8.319998	8.30096	Rb1	NM_009029
6825888	4763	11.78513	11.78009	11.7613	11.71341	0.071717	0.499344	0.06668	0.529128	0.047883	0.649274	0.018797	0.857504	0.005037	0.961589	0.023833	0.820021	6825888	11.93142	11.68987	11.7341	11.76016	11.84472	11.7354	11.9303	11.74785	11.60574	11.64717	11.6367	11.85637	Itm2b	NM_008410
6826007	4766	6.23754	6.179942	6.132688	6.115699	0.121841	0.07638	0.064243	0.314792	0.016989	0.783908	0.047254	0.452938	0.057598	0.364437	0.104852	0.118161	6826007	6.26068	6.254999	6.196942	6.179096	6.12798	6.23275	6.192983	6.010371	6.19471	6.182314	6.027706	6.137077	Spert	NM_026457
6826016	4767	7.972531	8.116553	8.183831	8.108099	-0.13557	0.313159	0.008454	0.948133	0.075732	0.564305	-0.06728	0.607758	-0.14402	0.285907	-0.2113	0.131944	6826016	7.99884	8.033978	7.884774	8.064033	8.213385	8.072242	8.148217	8.42307	7.980206	8.046232	8.309905	7.968161	Cog3	NM_177381
6826018	4768	7.702716	7.75881	7.640147	7.440393	0.262323	0.038355	0.318417	0.016933	0.199754	0.096109	0.118663	0.29521	-0.05609	0.61087	0.062569	0.571121	6826018	7.726984	7.724596	7.656568	7.787566	7.798772	7.690093	7.768002	7.388564	7.763875	7.426054	7.326934	7.568192	Slc25a30	NM_026232
6826029	4769	9.475629	9.167592	9.013551	8.81368	0.661949	0.015013	0.353912	0.137726	0.199871	0.378919	0.154041	0.493306	0.308036	0.189098	0.462078	0.06346	6826029	9.378208	9.418014	9.630664	8.897661	9.522532	9.082584	9.128759	8.91941	8.992484	9.058052	9.0058	8.377188	Gtf2f2	NM_026816
6826042	4770	7.89681	8.03943	7.913461	7.781029	0.115781	0.238065	0.258401	0.021621	0.132432	0.182831	0.125969	0.202779	-0.14262	0.154905	-0.01665	0.859067	6826042	7.895725	7.974326	7.820378	7.951563	8.069429	8.097298	7.963505	7.943259	7.833618	7.604048	7.773678	7.965361	1200011I	NM_026177
6826116	4772	8.074481	8.111009	8.117028	7.298007	0.776474	0.003092	0.813002	0.002369	0.819021	0.002269	-0.00602	0.974966	-0.03653	0.849115	-0.04255	0.824704	6826116	8.08379	7.879362	8.260291	7.978384	8.293223	8.061421	8.170773	7.997877	8.182433	6.952251	7.6807	7.26107	9030625A	NM_172488
6826179	4773	7.477039	7.616521	7.558588	7.47057	0.00647	0.928485	0.145951	0.070086	0.088018	0.243172	0.057933	0.430956	-0.13948	0.080922	-0.08155	0.276659	6826179	7.432776	7.403901	7.594441	7.720828	7.537565	7.59117	7.609365	7.525262	7.541136	7.371682	7.497154	7.542873	Dnajc15	NM_025384
6826219	4775	7.099395	7.397901	7.179541	7.425177	-0.32578	0.054937	-0.02728	0.855544	-0.24564	0.128869	0.21836	0.17069	-0.29851	0.073627	-0.08015	0.595725	6826219	7.17066	7.053612	7.073912	7.334771	7.442298	7.416634	7.364101	7.304696	6.869826	7.613779	7.190503	7.471249	Tnfsf11	NM_011613
6826265	4776	9.310606	9.197569	9.030281	9.772789	-0.46218	0.08795	-0.57502	0.04203	-0.74251	0.0142	0.167489	0.501358	0.112837	0.647935	0.280326	0.272489	6826265	9.090903	9.349069	9.491847	9.606481	8.952895	9.033932	9.197293	9.11043	8.783119	10.02711	9.371602	9.919656	1190002H	NM_025427
6826266	4777	6.888807	6.934791	6.859055	6.87116	0.017646	0.739237	0.063631	0.249129	-0.01211	0.819032	0.075737	0.177337	-0.04598	0.395333	0.029752	0.577173	6826266	6.790648	6.93118	6.944592	6.877899	6.988089	6.938386	6.848745	6.9011	6.827319	6.804841	6.930253	6.878387	Narg1l	NM_025832
6826279	4778	8.017735	8.094532	7.957767	8.001354	0.016381	0.881992	0.093179	0.408742	-0.04359	0.694129	0.136765	0.236568	-0.0768	0.492898	0.059967	0.590136	6826279	8.045485	8.100971	7.906748	8.021589	8.177192	8.084816	8.089465	8.035433	7.748404	7.91713	7.927313	8.159618	Wbp4	NM_018765
6826285	4779	8.408536	7.153757	6.798965	8.944703	-0.53617	0.626599	-1.79095	0.129556	-2.14574	0.077524	0.354792	0.746433	1.254779	0.270456	1.609571	0.167351	6826285	7.896883	9.809772	7.518953	6.541504	7.9309	6.988868	7.346967	6.075339	6.974589	10.76752	9.378621	6.687968	Lect1	NM_010701
6826546	4782	7.045176	7.171144	6.922254	6.707602	0.337574	0.223279	0.463543	0.107415	0.214653	0.425566	0.24889	0.358867	-0.12597	0.635491	0.122921	0.643575	6826546	7.091726	6.794426	7.249375	7.162553	7.389315	6.961565	7.235724	6.511225	7.019814	6.657465	6.3417	7.12364	Diap3	NM_019670
6826748	4784	7.76216	7.745683	7.49281	7.617743	0.144417	0.554934	0.12794	0.600068	-0.12493	0.608515	0.252874	0.312112	0.016477	0.945685	0.26935	0.283698	6826748	7.862723	7.692199	7.731558	7.714733	7.588934	7.933383	7.304214	7.415259	7.758956	7.863768	7.059195	7.930267	---	GENSCAN000000
6826916	4786	8.551027	8.739999	8.50163	8.778149	-0.22712	0.100017	-0.03815	0.762776	-0.27652	0.053401	0.23837	0.086789	-0.18897	0.160425	0.049397	0.696508	6826916	8.674999	8.585873	8.392208	8.670744	8.676304	8.87295	8.665877	8.571215	8.267797	8.874899	8.801053	8.658495	2410129H	BC117005
6826927	4788	7.908516	7.91065	7.960397	7.751552	0.156964	0.321733	0.159098	0.315606	0.208845	0.197561	-0.04975	0.746433	-0.00213	0.988893	-0.05188	0.736028	6826927	7.892335	7.895459	7.937754	7.717052	8.176371	7.838528	8.109563	7.786755	7.984872	7.626386	8.006433	7.621837	Dis3	NM_028315
6826975	4789	7.540731	7.281521	7.497133	8.102589	-0.56186	0.041205	-0.82107	0.007498	-0.60546	0.030721	-0.21561	0.378365	0.25921	0.294788	0.043598	0.855136	6826975	7.323356	7.693311	7.605525	7.343695	7.343382	7.157485	7.452065	7.767041	7.272292	8.503023	8.200526	7.604217	Klf12	NM_010636
6827123	4790	9.547763	9.359051	9.381938	8.85298	0.694783	0.039884	0.506071	0.112063	0.528958	0.099028	-0.02289	0.937636	0.188712	0.524338	0.165825	0.574705	6827123	9.701965	9.580155	9.36117	9.075664	9.881286	9.120203	9.529659	9.86857	9.647586	8.980654	9.110802	8.467484	Kctd12	NM_177715
6827132	4791	8.844355	9.075882	9.011983	8.863477	-0.01912	0.75565	0.212404	0.007214	0.148506	0.036866	0.063899	0.313194	-0.23153	0.004547	-0.16763	0.022377	6827132	8.815904	8.860227	8.856935	9.083255	9.003789	9.140601	9.130553	8.999684	8.905712	8.845865	8.819038	8.925529	Fbxl3	NM_015822
6827135	4792	9.108513	9.041478	8.993611	9.019552	0.088961	0.291663	0.021926	0.787889	-0.02594	0.750474	0.047867	0.560419	0.067035	0.419699	0.114902	0.182937	6827135	9.116685	9.116254	9.0926	9.077013	9.067497	8.979924	9.02262	9.031701	8.926511	8.947134	9.219702	8.891819	Mycbp2	NM_207215
6827159	4793	9.393613	10.50107	9.585901	10.31809	-0.92447	0.00389	0.18298	0.450169	-0.73219	0.013062	0.915166	0.004116	-1.10745	0.001347	-0.19229	0.428337	6827159	9.108131	9.594512	9.478195	10.28658	10.32352	10.8931	9.389729	9.528668	9.839306	9.986381	10.53982	10.42806	Ednrb	NM_007904
6827198	4795	5.939822	5.99059	6.009795	6.06155	-0.12173	0.045382	-0.07096	0.204792	-0.05175	0.343492	-0.0192	0.718404	-0.05077	0.352254	-0.06997	0.210536	6827198	5.900361	5.992066	5.927039	6.018943	5.948875	6.003953	5.992613	6.025565	6.011207	6.075152	5.945864	6.163634	Pou4f1	NM_011143
6827203	4796	6.478452	6.579074	6.357654	6.369341	0.109111	0.246728	0.209734	0.04303	-0.01169	0.896822	0.221421	0.034925	-0.10062	0.282397	0.120798	0.203871	6827203	6.432412	6.410336	6.592608	6.486245	6.663854	6.587124	6.379259	6.283733	6.409969	6.34302	6.230666	6.534336	Rnf219	NM_026047
6827239	4797	8.568394	8.567375	8.527936	8.571492	-0.0031	0.966018	-0.00412	0.954845	-0.04356	0.553732	0.039439	0.59106	0.00102	0.988811	0.040459	0.581687	6827239	8.653034	8.559898	8.492251	8.496008	8.672441	8.533675	8.51465	8.582047	8.48711	8.512941	8.699219	8.502316	Rbm26	NM_134077
6827284	4798	7.846541	7.576423	7.154083	7.38337	0.463171	0.186574	0.193053	0.563769	-0.22929	0.49487	0.42234	0.224234	0.270118	0.423993	0.692458	0.062818	6827284	7.64603	8.145303	7.748289	7.488575	7.963984	7.27671	7.361321	6.837844	7.263084	7.656551	7.785278	6.70828	Spry2	NM_011897
6827790	4802	5.909796	5.999979	5.88647	5.812121	0.097675	0.285672	0.187858	0.058959	0.074349	0.409206	0.113509	0.220333	-0.09018	0.321676	0.023326	0.791609	6827790	5.847486	5.973026	5.908875	6.005917	6.089196	5.904823	6.026103	5.870513	5.762794	5.689701	5.923371	5.823291	Tgds	NM_029578
6827801	4803	7.93832	8.053409	8.071644	8.061287	-0.12297	0.154695	-0.00788	0.922773	0.010357	0.897967	-0.01824	0.821572	-0.11509	0.179532	-0.13332	0.126797	6827801	7.925868	7.933051	7.956042	8.152037	8.085397	7.922792	8.196939	8.077205	7.940788	8.059953	8.140387	7.983522	Sox21	NM_177753
6827820	4804	6.414759	6.491043	6.303011	6.148602	0.266157	0.070294	0.342441	0.027677	0.154409	0.260442	0.188032	0.178515	-0.07628	0.566191	0.111748	0.406336	6827820	6.555221	6.354292	6.334764	6.43585	6.652615	6.384664	6.305728	6.163901	6.439404	5.915652	6.317357	6.212798	Dzip1	NM_025943
6827825	4805	7.211371	7.465907	7.078344	7.282508	-0.07114	0.67925	0.183399	0.300882	-0.20416	0.253212	0.387563	0.047625	-0.25454	0.163339	0.133027	0.445603	6827825	7.272011	7.204884	7.157218	7.479862	7.526066	7.391792	6.831089	7.413098	6.990845	7.083273	7.190263	7.573988	Ugcgl2	NM_001081252
6827912	4807	8.121149	8.139177	8.074005	7.862211	0.258938	0.04859	0.276966	0.037748	0.211794	0.093802	0.065172	0.574668	-0.01803	0.875453	0.047143	0.683313	6827912	8.087343	8.127074	8.149029	8.066122	8.326734	8.024675	8.161864	7.988415	8.071737	7.75529	8.090528	7.740815	Stk24	NM_145465

6827925	4809	10.33154	10.5844	10.37578	10.48139	-0.14985	0.082967	0.103013	0.210428	-0.10561	0.200257	0.208623	0.024769	-0.25286	0.010197	-0.04424	0.574858	6827925	10.2066	10.35458	10.43345	10.46593	10.59314	10.69414	10.45191	10.39087	10.28456	10.50888	10.45142	10.48387	Dock9	NM_001081039
6827944	4811	8.239891	7.912377	7.952584	7.099272	1.140619	0.015634	0.813105	0.060881	0.853312	0.051458	-0.04021	0.916817	0.327514	0.405533	0.287307	0.463303	6827944	8.722552	8.041433	7.955689	8.094334	8.248773	7.394024	8.350493	7.409298	8.097962	6.759686	7.625451	6.912679	Gpr183	NM_183031
6827977	4813	6.647574	6.730493	6.73915	6.941231	-0.29366	0.138092	-0.21074	0.271061	-0.20208	0.289764	-0.00866	0.962458	-0.08292	0.65421	-0.09158	0.621324	6827977	6.7505	6.72074	6.471482	6.649354	6.511286	7.030839	6.738574	6.688831	6.790044	7.290874	6.744002	6.788817	EG328479	NM_177817
6827978	4814	6.120676	6.21432	6.130101	6.306801	-0.18612	0.115155	-0.09248	0.405468	-0.1767	0.131899	0.08422	0.446979	-0.09364	0.39985	-0.00942	0.930894	6827978	6.251601	5.958132	6.152356	6.224651	6.256078	6.162232	6.25368	6.175551	5.961071	6.445397	6.16951	6.305496	Zic5	NM_022987
6828001	4815	9.195083	9.322744	9.335088	9.326114	-0.13103	0.473276	-0.00337	0.98503	0.008974	0.96016	-0.01234	0.945221	-0.12766	0.484364	-0.14	0.444576	6828001	8.991354	9.130473	9.463423	9.362274	9.34797	9.257987	9.475966	9.528061	9.001236	9.236848	9.19868	9.542814	A2ld1	NM_145466
6828005	4816	8.078166	8.403845	8.366801	8.135107	-0.05694	0.767486	0.268738	0.186807	0.231693	0.248461	0.037044	0.847218	-0.32568	0.118298	-0.28863	0.159588	6828005	7.789556	7.980912	8.46403	8.490752	8.451651	8.269132	8.545893	8.357101	8.197408	8.005749	8.026312	8.373261	Tmtc4	NM_028651
6828326	4821	10.4038	10.23768	10.1733	10.08591	0.317887	0.012441	0.151767	0.164034	0.087383	0.403424	0.064383	0.533934	0.16612	0.132065	0.230503	0.048391	6828326	10.57558	10.23925	10.39657	10.22162	10.34817	10.14325	10.21264	10.28231	10.02494	10.13216	10.03956	10.08602	Sepp1	NM_009155
6828374	4822	8.071521	8.39516	8.19915	8.092675	-0.02115	0.921518	0.302485	0.184058	0.106475	0.622638	0.19601	0.373711	-0.32364	0.158425	-0.12763	0.55662	6828374	8.080776	7.941804	8.191984	8.43833	8.368378	8.378773	8.498637	8.163294	7.93552	7.629893	8.282616	8.365516	Oxct1	NM_024188
6828417	4825	5.977747	6.341828	6.137598	6.26455	-0.2868	0.102363	0.077278	0.632595	-0.12695	0.43791	0.20423	0.225485	-0.36408	0.047321	-0.15985	0.33405	6828417	6.128589	5.630766	6.173886	6.366498	6.490624	6.168362	6.26872	5.959635	6.184438	6.22401	6.317992	6.251649	Rpl37	NM_026069
6828420	4826	8.936904	9.038548	9.026704	8.813718	0.123186	0.301783	0.22483	0.078724	0.212986	0.09276	0.011844	0.918096	-0.10164	0.389044	-0.0898	0.444288	6828420	8.904138	8.88257	9.024004	8.827538	9.277761	9.010345	9.169886	8.980219	8.930007	8.842623	8.831249	8.767282	Prkaa1	NM_001013367
6828422	4827	7.528093	7.569829	7.492071	7.504488	0.023605	0.853441	0.065341	0.611741	-0.01242	0.922527	0.077758	0.547202	-0.04174	0.744544	0.036022	0.778339	6828422	7.496318	7.566718	7.521244	7.621757	7.483886	7.603845	7.755076	7.515682	7.205455	7.564409	7.395046	7.554009	Ttc33	NM_026213
6828472	4828	9.541256	9.469596	9.260713	9.615901	-0.07464	0.758646	-0.1463	0.550482	-0.35519	0.168725	0.208883	0.399536	0.07166	0.767961	0.280543	0.266284	6828472	9.955194	9.516562	9.152011	9.453197	9.641556	9.314035	9.278528	9.18037	9.32324	9.211018	9.701688	9.934996	Dab2	NM_023118
6828480	4831	7.716762	7.448926	7.957617	6.76457	0.952192	0.00648	0.684357	0.030441	1.193047	0.001813	-0.50869	0.086891	0.267836	0.334419	-0.24086	0.382685	6828480	7.980071	7.592862	7.577353	7.589126	7.647594	7.110059	8.092505	7.672581	8.107765	6.439507	7.285869	6.568333	Fyb	NM_011815
6828492	4832	8.444816	8.554702	8.483455	8.480664	0.036152	0.574118	0.146038	0.045484	0.074791	0.260076	0.071247	0.281575	-0.10989	0.112817	-0.03864	0.548646	6828492	8.398567	8.464892	8.470989	8.403331	8.60114	8.659636	8.515239	8.464023	8.471104	8.38046	8.465495	8.380037	4921505C	NM_030168
6828522	4833	9.008756	9.022361	8.918314	8.890753	0.118003	0.453032	0.131608	0.404714	0.027561	0.858437	0.104047	0.506492	-0.0136	0.929783	0.090442	0.562272	6828522	9.019641	9.060487	8.946141	9.213104	8.787104	9.066876	8.990838	8.85633	8.907774	8.763124	8.695183	9.213953	Lifr	NM_013584
6828573	4835	7.735767	7.875445	7.751273	7.533412	0.202355	0.142221	0.342033	0.025005	0.21786	0.117781	0.124173	0.347107	-0.13968	0.293765	-0.01551	0.903814	6828573	7.708185	7.710635	7.788481	7.708185	8.131551	7.7866	7.877548	7.605319	7.770951	7.373858	7.657867	7.568512	Nup155	NM_133227
6828582	4836	7.168039	7.258153	7.163956	7.07043	0.09761	0.217718	0.187724	0.032973	0.093526	0.235775	0.094197	0.232723	-0.09011	0.251821	0.004083	0.95674	6828582	7.155934	7.194676	7.153508	7.238672	7.326565	7.209223	7.086076	7.209147	7.196645	7.073003	7.221078	6.917208	2410089E	BC058107
6828651	4838	8.438283	8.385332	8.494221	8.652374	-0.21409	0.036748	-0.26704	0.014175	-0.15815	0.101588	-0.10889	0.238684	0.052951	0.553035	-0.05594	0.531407	6828651	8.506948	8.435942	8.371959	8.386179	8.382202	8.387616	8.477477	8.607828	8.397358	8.477656	8.668066	8.811401	Lmbrd2	NM_177178
6828663	4840	7.280004	7.135448	7.155735	6.820364	0.45964	0.274172	0.315084	0.444219	0.335371	0.416603	-0.02029	0.959947	0.144556	0.721553	0.124269	0.75907	6828663	7.247259	7.556317	7.036436	7.398192	7.563953	6.444199	7.790007	6.674154	7.003043	6.456757	6.760847	7.243487	Capsl	NM_029341
6828697	4841	10.89782	10.78657	10.89419	10.52635	0.371473	0.015968	0.260223	0.065556	0.367847	0.016706	-0.10762	0.403557	0.11125	0.388621	0.003627	0.97702	6828697	11.143	10.78078	10.76968	10.74443	10.84338	10.7719	11.02283	10.84077	10.81898	10.33096	10.64579	10.60229	Agxt2	NM_001031851
6828699	4842	9.607123	9.762891	9.670425	9.15952	0.447604	0.040942	0.603371	0.011169	0.510905	0.023988	0.092466	0.628633	-0.15577	0.421578	-0.0633	0.739546	6828699	9.820224	9.610166	9.39098	9.663569	9.743326	9.881777	9.774706	9.528917	9.707651	8.773228	9.481425	9.223906	Prlr	BC096586
6828705	4843	6.077543	6.348987	6.235343	6.229929	-0.15239	0.391778	0.119059	0.499478	0.005414	0.975129	0.113644	0.518638	-0.27144	0.145512	-0.1578	0.375977	6828705	5.912452	6.284072	6.036105	6.216442	6.531485	6.299035	6.35446	6.273779	6.07779	5.990093	6.559933	6.13976	Rad1	NM_011232
6828741	4844	5.754312	5.969107	5.845067	5.316415	0.437897	0.36081	0.652692	0.186551	0.528652	0.275628	0.12404	0.790618	-0.2148	0.647192	-0.09076	0.845807	6828741	5.389473	6.558047	5.315416	5.694131	6.726781	5.486409	6.239527	5.686934	5.608741	4.845919	5.436589	5.666738	C1qtnf3	NM_030888
6828742	4845	7.323585	7.4636	7.337518	7.768765	-0.44518	7.36E-05	-0.30517	0.000932	-0.43125	9.21E-05	0.126082	0.068269	-0.14001	0.047488	-0.01393	0.821798	6828742	7.231173	7.396269	7.343313	7.527757	7.348037	7.515005	7.320185	7.365755	7.326613	7.7355	7.731559	7.839236	Amacr	NM_008537
6828747	4847	6.874664	6.849347	6.588548	6.485108	0.389555	0.054555	0.364239	0.068519	0.10344	0.566706	0.260799	0.170381	0.025317	0.887355	0.286116	0.137002	6828747	7.186872	6.763155	6.673964	6.74866	7.187037	6.612344	6.681612	6.541162	6.54287	6.414782	6.454232	6.586311	Adamts12	NM_175501
6828790	4848	9.390069	9.468758	9.480824	9.49806	-0.10799	0.461777	-0.0293	0.839125	-0.01724	0.904861	-0.01207	0.933302	-0.07869	0.588716	-0.09076	0.534164	6828790	9.293415	9.472281	9.40451	9.414857	9.579005	9.412413	9.285121	9.816906	9.340445	9.469824	9.629524	9.394831	Zfr	NM_011767
6828793	4849	8.097858	8.195039	8.051191	8.096082	0.001776	0.988125	0.098957	0.417036	-0.04489	0.707994	0.143848	0.248735	-0.09718	0.425098	0.046667	0.697112	6828793	8.122836	8.056842	8.113896	8.145006	8.437042	8.00307	7.972325	8.105432	8.075816	7.915768	8.159329	8.21315	Mtmr12	NM_172958
6828799	4850	9.834775	9.926292	10.02438	10.11056	-0.27579	0.005008	-0.18427	0.03365	-0.08619	0.265447	-0.09808	0.210122	-0.09152	0.239311	-0.1896	0.029992	6828799	9.807254	9.866021	9.831051	9.776415	10.00904	9.993421	10.07817	10.07456	9.920399	10.04631	10.09688	10.1885	Golph3	NM_025673
6828862	4851	7.823387	7.841311	7.855633	7.756932	0.066455	0.445531	0.084379	0.338137	0.0987	0.267544	-0.01432	0.867018	-0.01792	0.834088	-0.03225	0.707201	6828862	7.710167	7.87233	7.887665	7.798906	7.994406	7.730622	7.831558	7.872406	7.862934	7.65205	7.872905	7.745842	Rnasen	NM_001130149
6829277	4854	9.366665	9.336565	9.166705	8.82854	0.538125	0.000696	0.508024	0.001002	0.338165	0.010012	0.16986	0.130481	0.030101	0.772858	0.19996	0.082589	6829277	9.47523	9.365962	9.258804	9.325783	9.469641	9.21427	9.208692	9.156935	9.134488	8.634152	8.981264	8.870205	Myo10	NM_019472
6829281	4855	9.477201	9.573045	9.610566	9.546432	-0.06923	0.406219	0.026612	0.744795	0.064134	0.440217	-0.03752	0.647395	-0.09584	0.259489	-0.13337	0.129733	6829281	9.330897	9.534369	9.566337	9.57135	9.496802	9.650982	9.594549	9.58429	9.652859	9.438593	9.529119	9.671585	Fam134b	NM_001034851
6829283	4856	7.472811	7.561888	7.470121	7.563704	-0.09089	0.16641	-0.00182	0.97648	-0.09358	0.155648	0.091767	0.162843	-0.08908	0.174049	0.00269	0.965172	6829283	7.41436	7.502279	7.501793	7.528672	7.604417	7.552574	7.516391	7.507411	7.386561	7.641529	7.438037	7.611545	Zfp622	NM_144523
6829297	4857	6.074272	6.091597	6.079298	6.249564	-0.17529	0.233866	-0.15797	0.279346	-0.17027	0.246373	0.012299	0.930236	-0.01732	0.901873	-0.00503	0.971454	6829297	6.1676	6.111979	5.943238	5.987087	6.036365	6.251339	6.168964	6.258728	5.810203	6.367246	6.085428	6.296019	11-Mar	NM_177597
6829360	4858	11.71255	11.6804	11.44366	11.10808	0.60447	0.017941	0.572317	0.022857	0.335577	0.13809	0.23674	0.278653	0.032153	0.87849	0.268893	0.22334	6829360	11.70253	11.79246	11.64266	11.40466	12.03001	11.60652	11.68184	11.08303	11.5661	11.03215	11.33633	10.95576	Ank	NM_020332
6829361	4859	7.930575	7.2885	6.909513	7.022743	0.907832	0.022365	0.265757	0.432417	-0.11323	0.733793	0.378987	0.272345	0.642075	0.080877	1.021062	0.013078	6829361	7.754592	8.256699	7.780435	6.599156	7.706469	7.559876	6.914835	6.719565	7.09414	6.962537	7.42789	6.677802	Tiaf2	AF075717
6829451	4860	5.782871	6.73028	6.229958	7.426849	-1.64398	0.000128	-0.69657	0.019482	-1.19689	0.001045	0.500322	0.06972	-0.94741	0.004161	-0.44709	0.098396	6829451	5.501648	5.700777	6.146187	6.61079	6.558623	7.021428	5.940893	6.649188	6.099793	7.493379	7.225278	7.561889	Dnahc5	NM_133365
6829495	4861	6.341102	6.390067	6.186303	6.226907	0.114195	0.453154	0.16316	0.292591	-0.0406	0.78632	0.203765	0.197099	-0.04897	0.744004	0.1548	0.316339	6829495	6.504747	6.327259	6.191301	6.181318	6.688253	6.300631	6.301689	6.039206	6.218013	6.308619	6.275059	6.097043	Ctnnd2	NM_008729
6829504	4862	11.16526	11.12762	11.28367	11.69608	-0.53082	0.007468	-0.56846	0.005194	-0.41241	0.024635	-0.15605	0.326679	0.037637	0.807413	-0.11841	0.45078	6829504	11.0218	11.15786	11.31612	11.146	10.90884	11.32803	11.23449	11.43336	11.18317	11.50761	11.63579	11.94484	Dap	NM_146057
6829551	4864	6.600161	6.557426	6.518716	6.657795	-0.05763	0.588208	-0.10037	0.354762	-0.13908	0.210602	0.03871	0.71468	0.042735	0.686795	0.081445	0.448443	6829551	6.625901	6.387424	6.787158	6.636154	6.53826	6.497863	6.472345	6.610828	6.472974	6.53786	6.718471	6.717054	Fam173b	NM_026546
6829591	4866	7.480302	8.214578	7.757811	6.46173	1.018572	0.009179	1.752848	0.000373	1.296081	0.002468	0.456767	0.164394	-0.73428	0.039285	-0.27751	0.379612	6829591	7.541208	7.403754	7.495944	8.380569	8.291304	7.971862	8.239572	7.749063	7.284799	6.558417	6.911659	5.915114	Sema5a	NM_009154
6829598	4867	9.561465	9.485547	9.39914	9.527749	0.033716	0.846843	-0.0422	0.809095	-0.12861	0.468478	0.086407	0.622948	0.075917	0.665186	0.162325	0.364915	6829598	9.403083	9.729432	9.551879	9.345945	9.47531	9.635387	9.638637	9.061704	9.497079	9.339363	9.540621	9.703263	Sdc2	NM_008304
6829612	4868	10.98907	10.99869	11.13474	11.12081	-0.13174	0.230142	-0.12212	0.26296	0.013927	0.894175	-0.13605	0.216614	-0.00962	0.926766	-0.14567	0.188851	6829612	10.98898	11.01456	10.96367	10.89571	10.86688	11.23348	11.13163	11.04475	11.22784	11.05773	11.0621	11.24261	Pgcp	NM_018755
6829647	4869	10.21574	10.28765	10.25545	10.27943	-0.06369	0.492182	0.00822	0.928272	-0.02397	0.793309	0.032193	0.725421	-0.07191	0.439946	-0.03971	0.665482	6829647	10.22383	10.24686	10.17653	10.24366	10.40938	10.2099	10.41501	10.25941	10.09194	10.20389	10.37973	10.25466	Mtdh	NM_026002
6829656	4870	8.432937	8.508643	8.546321	8.486549	-0.05361	0.689192	0.022094	0.868515	0.059771	0.656078	-0.03768	0.778079	-0.07571	0.574201	-0.11338	0.405927	6829656	8.403664	8.504915	8.390232	8.513049	8.529671	8.48321	8.809427	8.612681	8.216854	8.449638	8.444688	8.565322	Laptm4b	NM_033521
6829661	4872	6.056989	6.0196	5.978507	5.880156	0.176833	0.112591	0.139444	0.197559	0.09835	0.350637	0.041093	0.689659	0.037389	0.716118	0.078483	0.451791	6829661	6.152631	6.027987	5.99035	6.119971	5.88874	6.050089	6.145279	5.873953	5.916288	5.843675	5.773929	6.022865	BC030476	BC030476
6829760	4876	6.105492	6.182963	5.967158	6.161966	-0.05647	0.762	0.020997	0.910115	-0.19481	0.311191	0.215806	0.265369	-0.07747	0.67861	0.138334	0.464751	6829760	6.186071	6.016886	6.113518	6.215933	6.281754	6.051203	6.051209	6.027203	5.823061	5.83447	6.047541	6.603888	Spag1	NM_012031
6829871	4877	8.648161	8.689249	8.561789	8.459007	0.189154	0.086373	0.230241	0.044602	0.102781	0.319202	0.12746	0.224276	-0.04109	0.682334	0.086373	0.39816	6829871	8.528537	8.640991	8.774956	8.604285	8.899651	8.56381	8.530043	8.635049	8.520274	8.463817	8.39922	8.513985	Grhl2	NM_026496
6829945	4879	10.16727	10.12628	10.26671	10.00035	0.166923	0.169981	0.125936	0.288157	0.266363	0.042749	-0.14043	0.240234	0.040987	0.72078	-0.09944	0.395226	6829945	10.15596	10.18616	10.15969	9.967638	10.19244	10.21877	10.31733	10.33325	10.14955	9.812999	9.961691	10.22635	Atp6v1c1	NM_025494
6829952	4881	9.837108	10.29874	10.23314	9.648882	0.188226	0.268891	0.649862	0.003429	0.584258	0.006151	0.065603	0.689701	-0.46164	0.019478	-0.39603	0.036962	6829952	9.725307	10.17459	9.611427	10.30984	10.30885	10.27754	10.33499	10.23255	10.13188	9.387667	9.790507	9.768471	Fzd6	NM_008056
6829964	4882	7.447591	7.579053	7.479581	7.272843	0.174748	0.093329	0.30621	0.010275	0.206738	0.054307	0.099472	0.309924	-0.13146	0.18984	-0.03199	0.73636	6829964	7.493288	7.507274	7.342211	7.52985	7.684863	7.522447	7.574914	7.466046	7.397784	7.10637	7.426533	7.285626	Wdsof1	NM_198606
6829967	4883	6.443675	6.395759	5.969732	6.020646	0.423029	0.247225	0.375113	0.300514	-0.05091	0.884296	0.426027	0.244169	0.047916	0.891057	0.473943	0.19951	6829967	5.672747	7.050328	6.607951	6.328703	6.36329	6.495285	5.934013	5.962246	6.012937	6.072722	6.422454	5.566763	Rims2	NM_053271
6830055	4886	9.991887	9.899562	9.963936	10.10692	-0.11504	0.460569	-0.20736	0.199889	-0.14299	0.363538	-0.06437	0.67593	0.092326	0.551198	0.027952	0.855302	6830055	9.971106	10.0257	9.978856	9.73585	10.02432	9.938515	10.26152	9.89283	9.737457	10.20666	9.885501	10.22861	Oxr1	NM_001130166
6830131	4887	9.973978	9.884277	9.898798	9.953192	0.020786	0.906003	-0.06891	0.696745	-0.05439	0.757945	-0.01452	0.934242	0.089701	0.613187	0.07518	0.67102	6830131	10.083	9.719204	10.11973	9.915915	9.893346	9.843571	10.12696	9.911601	9.657833	9.820586	9.78327	10.25572	Ttc35	NM_025736
6830161	4889	8.439901	8.604969	8.568364	8.562708	-0.12281	0.232051	0.042261	0.668109	0.005656	0.953967	0.036605	0.709953	-0.16507	0.120382	-0.12846	0.213137	6830161	8.517738	8.454667	8.347297	8.557906	8.566154	8.690847	8.789206	8.403182	8.512704	8.5974	8.519691	8.571032	Eny2	NM_175009
6830165	4891	7.099626	7.357828	7.189547	7.300062	-0.20044	0.052833	0.057766	0.531199	-0.11051	0.245936	0.168281	0.093044	-0.2582	0.019141	-0.08992	0.338162	6830165	6.964389	7.232666	7.101824	7.282465	7.321968	7.469051	7.303741	7.132703	7.132198	7.189967	7.369759	7.340459	Ebag9	NM_019480
6830354	4893	6.400564	6.222708	6.319863	6.254848	0.145717	0.229286	-0.03214	0.781345	0.065016	0.577404	-0.09716	0.410767	0.177856	0.150798	0.080701	0.491528	6830354	6.390337	6.372648	6.438708	6.047903	6.192091	6.42813	6.329945	6.387943	6.241702	6.200552	6.453901	6.11009	Utp23	NM_030132
6830401	4896	6.661025	6.883523	6.695038	6.745008	-0.08398	0.619433	0.138515	0.418997	-0.04997	0.766423	0.188485	0.27975	-0.2225	0.208336	-0.03401	0.839514	6830401	6.454841	6.770504	6.75773	6.782063	6.811447	7.05706	6.918482	6.463524	6.703109	6.771103	6.50489	6.959031	Med30	NM_027212
6830480	4898	9.985524	10.15163	10.35055	10.61919	-0.63366	0.003326	-0.46755	0.015992	-0.26863	0.118534	-0.19892	0.231567	-0.16611	0.311172	-0.36503	0.044846	6830480	9.764122	10.16996	10.02249	10.05189	10.32028	10.08273	10.338	10.56507	10.14859	10.4759	10.55298	10.82868	Mal2	NM_178920
6830481	4899	8.342486	8.11892	7.532233	7.054791	1.287695	0.043405	1.064129	0.082981	0.477442	0.400145	0.586687	0.306655	0.223566	0.688279	0.810253	0.16998	6830481	8.451707	8.639444	7.936307	7.979088	8.509329	7.868344	8.339812	6.705956	7.550932	6.013809	7.617997	7.532568	Nov	NM_010930
6830505	4900	8.925354	8.896877	8.840347	9.50284	-0.57749	0.079935	-0.60596	0.06856	-0.66249	0.050489	0.05653	0.849318	0.028477	0.923686	0.085007	0.775438	6830505	8.673634	8.934177	9.16825	9.04508	8.733833	8.911717	8.405726	9.450708	8.664606	9.413927	9.215312	9.879282	Depdc6	NM_001037937
6830506	4901	8.166393	7.778238	7.185938	6.561622	1.604771	0.006924	1.216616	0.025701	0.624316	0.198279	0.592301	0.21992	0.388155	0.408539	0.980456	0.058719	6830506	8.691022	8.044098	7.76406	7.564764	8.510436	7.259515	7.781456	6.679614	7.096743	6.023622	6.932974	6.72827	Col14a1	NM_181277

6830510	4902	6.306216	6.443997	6.389359	6.093738	0.212478	0.310213	0.350259	0.111943	0.295621	0.170164	0.054638	0.787631	-0.13778	0.50229	-0.08314	0.682792	6830510	6.345588	6.262244	6.310817	6.317054	6.764522	6.250414	6.748461	6.076978	6.342638	5.873895	6.220189	6.18713	Mtbp	NM_134092
6830619	4903	8.84819	8.925419	8.927764	8.952616	-0.10443	0.402385	-0.0272	0.823657	-0.02485	0.838596	-0.00234	0.984649	-0.07723	0.531511	-0.07957	0.519451	6830619	8.824867	8.871588	8.848115	8.812618	9.166261	8.797379	8.780033	8.995986	9.007272	8.963442	9.099174	8.795231	Zhx2	NM_199449
6830638	4904	6.945083	6.810023	6.944251	6.450244	0.494839	0.064314	0.359779	0.157555	0.494007	0.064676	-0.13423	0.576765	0.13506	0.574453	0.000832	0.997211	6830638	6.720237	7.032248	7.082764	6.426988	7.054755	6.948325	6.922788	6.674227	7.235738	6.224883	6.788214	6.337635	Wdr67	NM_001081396
6830651	4906	7.112639	7.122882	7.150988	6.925004	0.187635	0.167342	0.197878	0.147931	0.225984	0.104799	-0.02811	0.825761	-0.01024	0.935967	-0.03835	0.764208	6830651	7.034248	7.115197	7.188471	6.926277	7.377993	7.064375	7.154757	7.291451	7.006755	6.873763	7.049744	6.851504	Wdyhv1	NM_029734
6830671	4907	9.213527	9.437524	9.34014	9.283052	-0.06953	0.436008	0.154471	0.105978	0.057088	0.519768	0.097384	0.283961	-0.224	0.029639	-0.12661	0.173746	6830671	9.298133	9.234404	9.108043	9.277253	9.564087	9.471231	9.44838	9.313848	9.258192	9.223427	9.301322	9.324408	D15Ertd6	BC070426
6830700	4909	8.601495	8.675342	8.697514	8.621496	-0.02	0.899714	0.053846	0.73523	0.076018	0.634314	-0.02217	0.888911	-0.07385	0.643886	-0.09602	0.549704	6830700	8.417281	8.640547	8.746656	8.690751	8.753801	8.581474	8.952574	8.686951	8.453016	8.401513	8.816428	8.646546	Rnf139	NM_175226
6830710	4910	11.33336	11.42707	11.38753	11.50662	-0.17326	0.310503	-0.07955	0.632472	-0.11909	0.478048	0.039537	0.811081	-0.0937	0.57432	-0.05417	0.743709	6830710	11.1679	11.29585	11.53634	11.44289	11.44106	11.39725	11.44809	11.58907	11.12543	11.3415	11.38657	11.79179	Ndufb9	NM_023172
6830761	4911	6.661642	6.810102	6.890526	6.609986	0.051656	0.876353	0.200115	0.551031	0.28054	0.408372	-0.08042	0.808799	-0.14846	0.656587	-0.22888	0.496803	6830761	6.715125	6.362253	6.907548	6.651464	7.097651	6.68119	7.248841	6.668361	6.754376	6.171666	7.318429	6.339864	Sqle	NM_009270
6830766	4912	7.328169	7.125077	7.307738	7.164637	0.163532	0.260429	-0.03956	0.776999	0.143101	0.320198	-0.18266	0.213129	0.203092	0.170982	0.020431	0.88348	6830766	7.262044	7.448371	7.274092	6.960587	7.307711	7.106933	7.219915	7.336544	7.366755	6.971966	7.446182	7.075764	Nsmce2	NM_026746
6830770	4913	8.396619	8.479089	8.145664	8.603092	-0.20647	0.205428	-0.124	0.431797	-0.45743	0.015737	0.333425	0.056672	-0.08247	0.596995	0.250955	0.13242	6830770	8.312382	8.305052	8.572423	8.696823	8.487989	8.252455	8.147956	8.305775	7.983262	8.718996	8.384066	8.706215	Trib1	NM_144549
6830927	4915	8.78295	8.358581	8.618019	8.557079	0.22587	0.151386	-0.1985	0.200859	0.06094	0.679996	-0.25944	0.105964	0.424369	0.017603	0.16493	0.28021	6830927	8.909318	8.84592	8.593611	8.337521	8.60435	8.133872	8.573587	8.457297	8.823174	8.494086	8.602652	8.5745	Myc	NM_010849
6831120	4916	8.675984	8.800175	8.813801	8.578014	0.09797	0.462696	0.222161	0.118404	0.235787	0.100496	-0.01363	0.917213	-0.12419	0.356838	-0.13782	0.309526	6831120	8.682232	8.686957	8.658763	8.630876	9.015019	8.75463	9.075055	8.68778	8.678567	8.481996	8.625719	8.626327	Efr3a	NM_133766
6831157	4917	9.369014	9.310014	9.17476	9.229622	0.139392	0.119176	0.080392	0.343747	-0.05486	0.51167	0.135255	0.128912	0.059	0.48131	0.194254	0.041106	6831157	9.47496	9.281463	9.350619	9.304905	9.290183	9.334955	9.081614	9.295477	9.147188	9.155262	9.376388	9.157217	Phf20l1	NM_001081409
6831162	4918	13.8014	13.85482	13.85449	13.93565	-0.13425	0.060387	-0.08084	0.224742	-0.08116	0.223062	0.000323	0.99593	-0.05342	0.409964	-0.05309	0.412682	6831162	13.77244	13.85331	13.77845	13.91118	13.70328	13.94999	13.82244	13.91501	13.82603	13.93161	13.92156	13.95379	Tg	NM_009375
6831164	4919	7.744579	7.700651	7.84867	7.199792	0.544787	0.06205	0.500859	0.081417	0.648877	0.032527	-0.14802	0.572155	0.043928	0.865595	-0.10409	0.689641	6831164	7.929206	7.770432	7.534099	7.896347	7.778298	7.427309	8.052626	7.812958	7.680425	6.767763	7.092728	7.738886	Wisp1	NM_018865
6831254	4920	6.950532	6.947224	6.808844	7.01262	-0.06209	0.654977	-0.0654	0.638117	-0.20378	0.16625	0.138379	0.331264	0.003308	0.98088	0.141687	0.320531	6831254	6.945441	6.966369	6.939785	7.02808	6.811268	7.002323	6.968461	6.992426	6.465646	7.031018	6.936742	7.070099	Khdrbs3	NM_010158
6831469	4921	8.191448	8.179662	8.198829	8.160455	0.030993	0.85625	0.019207	0.910557	0.038373	0.822632	-0.01917	0.910742	0.011786	0.945028	-0.00738	0.965555	6831469	8.196779	8.105208	8.272357	8.306095	8.176987	8.055904	8.36809	8.140278	8.088118	7.77528	8.445562	8.260524	Ptp4a3	NM_008975
6831511	4922	6.065915	6.12624	6.161124	6.136572	-0.07066	0.252095	-0.01033	0.861255	0.024553	0.679291	-0.03488	0.559151	-0.06033	0.322729	-0.09521	0.134825	6831511	6.095853	6.047195	6.054696	6.116665	6.018015	6.24404	6.206551	6.173188	6.103634	6.146321	6.074213	6.189181	Bai1	NM_174991
6831538	4926	12.13108	11.61674	11.90836	11.52209	0.608987	0.00707	0.094653	0.591979	0.38627	0.052236	-0.29162	0.123789	0.514333	0.01623	0.222717	0.225451	6831538	12.36632	12.11762	11.90929	11.61553	11.91069	11.32401	11.84232	11.79121	12.09155	11.41716	11.58044	11.56867	Ly6e	NM_008529
6831591	4929	7.905165	8.767548	8.291143	9.210321	-1.30516	0.000376	-0.44277	0.081786	-0.91918	0.003294	0.476405	0.06467	-0.86238	0.004703	-0.38598	0.121023	6831591	7.536411	8.158977	8.020107	8.706512	8.670774	8.925359	7.957373	8.612226	8.30383	9.214285	8.955514	9.461163	Gpihbp1	NM_026730
6831592	4930	6.200097	6.251075	6.012979	5.918417	0.28168	0.25301	0.332658	0.183836	0.094562	0.690091	0.238096	0.328231	-0.05098	0.82918	0.187118	0.436902	6831592	6.124083	5.97007	6.506138	6.000099	6.69561	6.057517	6.195197	5.772873	6.070868	6.040951	6.030904	5.683397	Zfp41	NM_011759
6831600	4931	7.217944	7.132721	7.253797	7.168854	0.04909	0.808601	-0.03613	0.858367	0.084943	0.676274	-0.12108	0.554038	0.085222	0.675281	-0.03585	0.859448	6831600	7.323864	6.984482	7.345485	7.164226	7.037994	7.195944	7.304836	7.623709	6.832846	7.229061	6.991727	7.285774	Rhpn1	NM_008164
6831606	4932	8.147224	8.104251	8.098446	7.500669	0.646555	0.021736	0.603582	0.029104	0.597777	0.03028	0.005805	0.980266	0.042973	0.854868	0.048778	0.835578	6831606	8.249393	8.181189	8.011089	7.951742	8.322682	8.038328	8.29121	7.693286	8.310841	7.40094	7.907028	7.194039	Gsdmd	NM_026960
6831610	4934	7.84904	7.950081	7.845619	7.740294	0.108746	0.387937	0.209787	0.11622	0.105326	0.402344	0.104461	0.406045	-0.10104	0.420928	0.00342	0.977795	6831610	7.653896	8.049158	7.844065	7.827055	8.152542	7.870645	7.915455	7.895459	7.725944	7.677689	7.803972	7.73922	Zfp623	NM_030199
6831637	4937	10.6171	10.65474	10.74294	10.78813	-0.17104	0.176683	-0.13339	0.281184	-0.04519	0.705629	-0.0882	0.466752	-0.03765	0.75268	-0.12584	0.307356	6831637	10.59646	10.62985	10.62498	10.75865	10.71614	10.48944	10.6536	11.01389	10.56133	10.83242	10.76273	10.76925	Grina	NM_023168
6831647	4939	6.33539	6.480147	6.325571	6.271657	0.063733	0.434584	0.20849	0.02746	0.053914	0.506241	0.154576	0.081139	-0.14476	0.098666	0.00982	0.902276	6831647	6.240297	6.312785	6.453089	6.450307	6.608799	6.381335	6.355321	6.340075	6.281316	6.164139	6.34814	6.302692	Exosc4	NM_175399
6831648	4940	8.520948	8.310776	8.534283	8.531064	-0.01012	0.937119	-0.22029	0.114215	0.003219	0.979966	-0.22351	0.109787	0.210172	0.129244	-0.01334	0.917184	6831648	8.567561	8.409092	8.586191	8.230804	8.544162	8.157362	8.75688	8.4793	8.36667	8.542204	8.549088	8.5019	Gpaa1	NM_010331
6831651	4941	8.523988	8.723338	8.650388	9.045588	-0.5216	0.013161	-0.32225	0.085723	-0.3952	0.042969	0.07295	0.669094	-0.19935	0.260022	-0.1264	0.464212	6831651	8.442251	8.443972	8.68574	8.770968	8.71433	8.684715	8.779611	8.848205	8.323348	8.893196	8.917046	9.326521	Cyc1	NM_025567
6831652	4942	8.17539	8.332376	8.166456	8.178728	-0.00334	0.971083	0.153648	0.123529	-0.01227	0.894053	0.16592	0.100141	-0.15699	0.116703	0.008934	0.922748	6831652	8.161383	8.152168	8.212618	8.326643	8.491966	8.17852	8.216682	8.229652	8.053034	8.094021	8.136846	8.305318	Maf1	NM_026859
6831654	4943	6.825136	6.77539	6.831898	6.79957	0.025565	0.622639	-0.02418	0.641325	0.032328	0.535665	-0.05651	0.290744	0.049746	0.348504	-0.00676	0.89566	6831654	6.800098	6.846659	6.82865	6.791799	6.804156	6.730214	6.756837	6.951948	6.78691	6.848724	6.777443	6.772544	Brp16	NM_021555
6831657	4944	9.924013	10.03634	9.988552	10.33107	-0.40705	0.195601	-0.29472	0.336486	-0.34251	0.26881	0.047791	0.87243	-0.11233	0.706931	-0.06454	0.828449	6831657	9.55292	10.17759	10.04153	10.38185	9.792328	9.934852	9.864099	10.18556	9.915997	10.04365	10.02281	10.92674	Scx	NM_198885
6831660	4945	8.878789	8.612326	8.940271	8.80104	0.077749	0.62217	-0.18871	0.248758	0.139231	0.385601	-0.32794	0.062635	0.266463	0.117098	-0.06148	0.695917	6831660	8.808512	8.809646	9.018209	8.650136	8.794256	8.392587	8.672821	9.103553	9.044439	8.821245	8.956142	8.625733	Hsf1	NM_008296
6831665	4946	7.227378	7.117011	7.269244	7.220085	0.007292	0.95504	-0.10307	0.434781	0.049158	0.705205	-0.15223	0.259248	0.110367	0.404328	-0.04187	0.746998	6831665	7.28354	7.128251	7.270342	7.096754	7.26567	6.988609	7.360846	7.456006	6.990879	7.192842	7.153401	7.314013	Gpr172b	NM_029643
6831681	4947	7.83687	7.778995	7.850744	8.365492	-0.52862	0.00778	-0.5865	0.00447	-0.51475	0.008913	-0.07175	0.645066	0.057875	0.709552	-0.01387	0.928547	6831681	7.591892	7.722886	8.195832	7.80177	7.810102	7.725112	7.975801	7.776407	7.800025	8.316561	8.255463	8.524451	Adck5	NM_172960
6831687	4948	6.613705	6.672663	6.702017	6.850151	-0.23645	0.10185	-0.17749	0.202889	-0.14813	0.280445	-0.02935	0.824335	-0.05896	0.657275	-0.08831	0.509666	6831687	6.568788	6.726174	6.546153	6.736629	6.662158	6.619203	6.50508	7.031652	6.569319	6.823874	6.908845	6.817734	Kifc2	NM_010630
6831688	4949	7.470975	7.617862	7.590535	7.491068	-0.02009	0.695479	0.126794	0.033583	0.099468	0.079373	0.027327	0.596032	-0.14689	0.017951	-0.11956	0.042172	6831688	7.46288	7.493352	7.456693	7.672045	7.509497	7.672044	7.627617	7.519465	7.624524	7.500668	7.445615	7.52692	Ppp1r16a	NM_033371
6831690	4951	6.118203	6.039429	6.140455	6.19637	-0.07817	0.456841	-0.15694	0.155128	-0.05592	0.591298	-0.10103	0.341868	0.078774	0.453476	-0.02225	0.829458	6831690	6.056614	6.230934	6.067062	6.086462	6.119108	5.912717	6.140319	5.993567	6.287479	6.257653	6.048684	6.282774	Mfsd3	NM_027122
6831692	4952	7.105093	7.168956	7.074628	7.15736	-0.05227	0.507246	0.011596	0.881422	-0.08273	0.303845	0.094329	0.245677	-0.06386	0.421008	0.030465	0.696375	6831692	7.110086	7.124271	7.080922	7.139961	7.342273	7.024635	7.116906	7.076553	7.030424	7.225862	7.170902	7.075317	Lrrc14	NM_145471
6831703	4953	7.817554	7.760463	7.784727	7.892334	-0.07478	0.698005	-0.13187	0.498212	-0.10761	0.578593	-0.02426	0.899366	0.057092	0.766584	0.032827	0.864211	6831703	8.005245	7.827389	7.620029	7.743622	7.549089	7.988677	7.800371	7.913754	7.640056	7.715878	7.698848	8.262276	Zfp7	NM_145916
6831705	4954	7.062692	7.166482	7.158478	7.243264	-0.18057	0.178039	-0.07678	0.5476	-0.08479	0.507744	0.008004	0.94942	-0.10379	0.420712	-0.09579	0.456037	6831705	7.053694	7.181483	6.952898	7.138606	6.994738	7.366103	7.139969	7.367015	6.96845	7.235773	7.286201	7.207818	Commd5	NM_025536
6831706	4955	10.32897	10.45109	10.3218	10.6386	-0.30964	0.021008	-0.18752	0.121014	-0.31681	0.018976	0.12929	0.265919	-0.12212	0.291325	0.00717	0.948743	6831706	10.2563	10.36575	10.36485	10.25448	10.62993	10.46885	10.44563	10.36485	10.15491	10.53572	10.67543	10.70466	Rpl8	NM_012053
6831709	4956	6.783941	6.83785	6.695297	6.859199	-0.07526	0.284359	-0.02135	0.753152	-0.1639	0.037002	0.142553	0.061487	-0.05391	0.434929	0.088644	0.213498	6831709	6.781019	6.817948	6.752856	6.818494	6.768826	6.926229	6.759303	6.545278	6.781309	6.832307	6.845669	6.89962	1110038F	BC038324
6831762	4957	6.559022	6.694922	6.528434	6.384149	0.174873	0.522441	0.310773	0.268692	0.144285	0.59614	0.166487	0.54208	-0.1359	0.617305	0.030588	0.909755	6831762	6.151996	6.572891	6.95218	6.72994	6.625659	6.729166	6.826651	6.08575	6.672902	6.075026	6.390989	6.686432	Apol10a	NM_177744
6831775	4958	6.198925	6.117333	5.763088	5.968924	0.23	0.28132	0.148408	0.477335	-0.20584	0.331427	0.354245	0.113067	0.081592	0.692688	0.435837	0.06	6831775	6.080228	6.126691	6.389855	6.065372	6.123613	6.163013	6.016872	5.690475	5.581917	6.181761	5.512792	6.21222	Apol9b	NM_173743
6831798	4959	7.199765	7.325116	7.065866	6.501312	0.698453	0.011357	0.823803	0.004831	0.564554	0.029571	0.259249	0.259462	-0.12535	0.573483	0.133899	0.548206	6831798	7.536117	7.12334	6.939839	7.402178	7.563806	7.009363	7.261308	6.902792	7.033499	6.207685	6.687922	6.60833	Ncf4	NM_008677
6831800	4960	8.162616	7.880046	7.809732	6.525727	1.636889	0.008553	1.354319	0.021032	1.284005	0.026452	0.070314	0.885487	0.28257	0.566703	0.352884	0.476917	6831800	8.971181	8.015026	7.501641	7.856256	8.4547	7.329182	8.155313	7.193585	8.080298	6.770063	6.774647	6.03247	Csf2rb	NM_007780
6831806	4961	7.094155	7.284263	6.965385	7.216721	-0.12257	0.537326	0.067543	0.731671	-0.25134	0.222875	0.318879	0.132138	-0.19011	0.346788	0.12877	0.517463	6831806	7.083524	6.994223	7.204717	7.400224	7.369692	7.082874	7.108061	7.142615	6.645478	7.024618	7.047275	7.578269	Mpst	NM_138670
6831821	4962	8.485424	8.313932	8.369825	7.312967	1.172456	0.005454	1.000965	0.012242	1.056858	0.009366	-0.05589	0.861785	0.171492	0.596262	0.115598	0.719662	6831821	8.892052	8.343328	8.220891	8.297254	8.67218	7.972362	8.715385	7.902361	8.49173	7.090478	7.764701	7.083723	Cyth4	NM_028195
6831845	4963	7.710234	7.491948	7.532761	7.632042	0.078191	0.620245	-0.14009	0.382835	-0.09928	0.531243	-0.04081	0.794746	0.218285	0.188189	0.177473	0.275789	6831845	7.837308	7.574692	7.718701	7.526065	7.737826	7.211954	7.573461	7.643503	7.381318	7.429647	7.692393	7.77409	Cdc42ep1	NM_027219
6831847	4964	7.736777	7.700338	7.823222	7.723391	0.013386	0.923294	-0.02305	0.868379	0.099831	0.479852	-0.12288	0.388345	0.036439	0.793637	-0.08645	0.539028	6831847	7.80684	7.713881	7.68961	7.79048	7.818293	7.492242	7.637716	8.131121	7.70083	7.727988	7.737815	7.70437	Gga1	NM_145929
6831851	4966	5.776924	5.972197	6.121668	6.246833	-0.46991	0.315603	-0.27464	0.548955	-0.12516	0.78277	-0.14947	0.74223	-0.19527	0.668201	-0.34474	0.454849	6831851	5.30448	6.025893	6.0004	5.540257	6.734062	5.642273	5.377666	6.246351	6.740986	6.343592	5.939273	6.457633	Nol12	NM_133800
6831852	4967	9.636239	9.292285	9.2855	9.411889	0.22435	0.337931	-0.1196	0.601692	-0.12639	0.581626	0.006785	0.976165	0.343954	0.15678	0.350739	0.149737	6831852	9.674893	9.57019	9.663634	9.389762	9.504342	8.98275	8.885438	9.425036	9.546025	9.27628	9.753163	9.206223	Triobp	NM_001039156
6831858	4968	8.371662	8.75376	8.583343	8.619732	-0.24807	0.224306	0.134028	0.49696	-0.03639	0.851622	0.170418	0.392028	-0.3821	0.077028	-0.21168	0.293678	6831858	8.359439	8.571357	8.184189	8.776059	8.6207	8.864522	8.581614	8.831778	8.336636	8.373493	8.512022	8.973682	H1f0	NM_008197
6831860	4970	6.943744	6.809961	6.87658	6.879452	0.064292	0.601316	-0.06949	0.572804	-0.00287	0.98121	-0.06662	0.588457	0.133783	0.290482	0.067164	0.585472	6831860	6.973242	6.817903	7.040087	6.657584	6.911584	6.860715	6.958512	6.894245	6.776984	6.858339	6.680333	7.099685	Galr3	NM_015738
6831867	4971	9.183893	9.27378	9.255427	9.147292	0.036601	0.675026	0.126487	0.171125	0.108135	0.234637	0.018353	0.832777	-0.08989	0.316506	-0.07153	0.419889	6831867	9.072398	9.150512	9.328769	9.222242	9.428761	9.170336	9.316271	9.280181	9.169829	9.120356	9.148632	9.172889	Eif3l	NM_145139
6831868	4972	7.768968	8.025068	7.573773	7.727198	0.04177	0.614097	0.29787	0.005698	-0.15343	0.090161	0.451295	0.000472	-0.2561	0.012307	0.195195	0.039849	6831868	7.649025	7.735947	7.921931	7.953847	8.060775	8.060582	7.592782	7.653104	7.475432	7.779963	7.633368	7.768262	Micall1	NM_177461
6831869	4973	9.329635	9.389886	9.358685	9.422926	-0.09329	0.578349	-0.03304	0.842577	-0.06424	0.700411	0.031201	0.851211	-0.06025	0.718038	-0.02905	0.861339	6831869	9.174092	9.313992	9.50082	9.287683	9.480242	9.401733	9.488428	9.613325	8.974302	9.399241	9.369642	9.499894	Polr2f	NM_027231
6831891	4974	6.830156	7.049032	6.728196	7.117134	-0.28698	0.151255	-0.0681	0.716316	-0.38894	0.063739	0.320836	0.114017	-0.21888	0.260773	0.101961	0.588394	6831891	6.830889	6.682852	6.976728	7.367582	7.011204	6.768309	6.637669	6.7573	6.789618	7.233938	6.799503	7.317962	Maff	NM_010755
6831919	4975	7.7466	7.981475	7.724347	7.813582	-0.06698	0.788083	0.167893	0.505696	-0.08923	0.720756	0.257128	0.317079	-0.23488	0.358246	0.022253	0.928691	6831919	7.658451	7.733539	7.84781	7.765449	8.431499	7.747477	7.731917	8.037059	7.404065	8.111794	7.807346	7.521605	Kdelr3	NM_134090
6831927	4976	7.882309	7.964111	7.701278	7.845425	0.036884	0.766963	0.118685	0.352715	-0.14415	0.265089	0.262833	0.060395	-0.0818	0.515681	0.181031	0.170758	6831927	7.900586	7.82965	7.916691	7.842789	8.181759	7.867784	7.758943	7.849103	7.495788	7.74589	7.806535	7.983851	Cby1	NM_028634
6831932	4977	8.864295	8.829341	8.829978	8.917196	-0.0529	0.332893	-0.08786	0.125354	-0.08722	0.127731	-0.00064	0.990408	0.034953	0.515145	0.034317	0.522623	6831932	8.935764	8.808075	8.849045	8.878798	8.868625	8.740601	8.869128	8.841196	8.77961	8.855944	8.921062	8.974583	Gtpbp1	NM_013818

6831959	4978	8.997819	8.272078	9.312396	8.691599	0.30622	0.077918	-0.41952	0.024335	0.620796	0.00345	-1.04032	0.000129	0.725741	0.001373	-0.31458	0.071523	6831959	9.038212	8.923924	9.031322	8.222943	8.5017	8.091592	9.416599	9.223723	9.296865	8.511185	9.01817	8.545443	Apobec3	NM_030255
6831982	4980	8.036067	8.134748	8.098921	8.401347	-0.36528	0.019644	-0.2666	0.066563	-0.30243	0.042657	0.035827	0.782708	-0.09868	0.45471	-0.06285	0.630258	6831982	8.179443	7.997509	7.931249	8.219561	7.933683	8.251	8.178421	8.196289	7.922053	8.45381	8.226891	8.523339	Syngr1	NM_207708
6831984	4981	6.435379	6.50142	6.421903	6.328984	0.106395	0.064293	0.172436	0.008367	0.092919	0.097933	0.079518	0.147604	-0.06604	0.219769	0.013476	0.792756	6831984	6.45089	6.384403	6.470844	6.524001	6.490834	6.489426	6.458832	6.301779	6.505097	6.296293	6.358525	6.332134	Map3k7ip	NM_025609
6831993	4983	6.991723	6.992748	7.10996	7.100644	-0.10892	0.589058	-0.1079	0.592502	0.009316	0.962793	-0.11721	0.561607	-0.00194	0.995906	-0.11824	0.558266	6831993	6.832273	7.082771	7.060125	7.089893	7.233803	6.654547	6.822793	7.470783	7.036305	7.066477	7.186572	7.048883	Smcr7l	NM_178719
6831994	4984	9.705934	9.774101	9.669579	9.826021	-0.12009	0.444764	-0.05192	0.737175	-0.15644	0.325662	0.104522	0.504021	-0.06817	0.660345	0.036356	0.813871	6831994	9.727757	9.679264	9.710782	9.602244	9.963932	9.756126	9.656096	9.973433	9.379207	9.943613	9.807221	9.727229	Atf4	NM_009716
6832005	4985	6.54093	6.470829	6.583989	6.255673	0.285257	0.091682	0.215155	0.186426	0.328316	0.058518	-0.11316	0.469022	0.070102	0.650319	-0.04306	0.779773	6832005	6.822673	6.390183	6.409935	6.443994	6.569822	6.39867	6.745177	6.384195	6.622595	6.432827	6.258032	6.076161	Grap2	NM_010815
6832018	4987	9.570057	9.558818	9.393263	9.583116	-0.01306	0.925616	-0.0243	0.862183	-0.18985	0.198866	0.165555	0.256679	0.011239	0.935949	0.176794	0.228372	6832018	9.511723	9.664541	9.533908	9.614142	9.466373	9.595939	9.17874	9.726945	9.274105	9.483764	9.571745	9.693838	Tnrc6b	NM_144812
6832078	4988	9.188168	9.204377	9.005046	9.172351	0.015817	0.922698	0.032026	0.844387	-0.1673	0.32043	0.19933	0.242505	-0.01621	0.920791	0.183122	0.279741	6832078	9.220486	9.230679	9.113339	9.247177	9.109672	9.256281	8.641416	9.362766	9.010957	9.070595	9.194256	9.252202	Tnrc6b	NM_144812
6832079	4989	7.872808	7.83782	7.925233	7.845723	0.027085	0.823998	-0.0079	0.948179	0.07951	0.518918	-0.08741	0.479471	0.034989	0.774119	-0.05242	0.668236	6832079	7.660234	7.845328	8.112863	7.833192	7.978821	7.701446	7.957279	7.834403	7.984016	7.766057	7.919853	7.851259	Adsl	NM_009634
6832086	4990	6.687835	6.701727	6.613139	6.720344	-0.03251	0.730371	-0.01862	0.843138	-0.1072	0.272995	0.088588	0.359205	-0.01389	0.882548	0.074696	0.435888	6832086	6.73166	6.64796	6.683886	6.794487	6.690704	6.61999	6.739744	6.616932	6.482742	6.680303	6.890623	6.590106	Sgsm3	NM_134091
6832105	4993	7.563199	7.498372	7.607648	7.508633	0.054565	0.749167	-0.01026	0.951896	0.099015	0.564737	-0.10928	0.526071	0.064827	0.70443	-0.04445	0.794281	6832105	7.411325	7.619558	7.658713	7.38475	7.857585	7.25278	7.663865	7.753202	7.405877	7.385582	7.589685	7.550633	Xpnpep3	NM_177310
6832107	4994	6.29266	6.256404	6.251046	6.27463	0.01803	0.95654	-0.01823	0.956069	-0.02358	0.943176	0.005358	0.987077	0.036255	0.912766	0.041614	0.899949	6832107	6.42704	6.365151	6.085788	7.087843	5.714602	5.966768	6.412386	6.111887	6.228865	6.286056	6.43166	6.106174	Rbx1	NM_019712
6832115	4995	9.702446	9.731211	9.649288	9.677238	0.025208	0.852364	0.053973	0.691478	-0.02795	0.836565	0.081923	0.549589	-0.02876	0.831885	0.053158	0.695857	6832115	9.73278	9.757503	9.617056	9.709329	9.851672	9.632632	9.462988	9.984354	9.500522	9.665657	9.659193	9.706864	Ep300	NM_177821
6832117	4996	6.907309	6.866097	6.902719	6.833744	0.073565	0.337416	0.032353	0.665521	0.068975	0.366733	-0.03662	0.625192	0.041212	0.583277	0.00459	0.950796	6832117	6.982491	6.88733	6.852106	6.88719	6.928861	6.78224	6.876075	6.839571	6.99251	6.746378	6.783484	6.97137	L3mbtl2	NM_145993
6832132	4997	7.251335	7.735612	6.871243	7.972386	-0.72105	0.010308	-0.23677	0.305342	-1.10114	0.000938	0.864369	0.003962	-0.48428	0.05544	0.380091	0.116808	6832132	6.802492	7.265872	7.68564	7.943848	7.504267	7.758722	6.886647	6.9251	6.801983	7.879901	7.856621	8.180637	Tef	NM_153484
6832142	4998	9.94529	9.981868	9.902458	10.27703	-0.33174	0.066601	-0.29516	0.095696	-0.37457	0.043436	0.079411	0.625165	-0.03658	0.820869	0.042833	0.791024	6832142	9.883865	9.779616	10.17239	10.1042	9.820905	10.0205	9.927865	9.831247	9.948261	10.01022	10.24691	10.57396	Aco2	NM_080633
6832153	5000	7.444859	7.210917	7.597317	7.775063	-0.3302	0.123644	-0.56415	0.018724	-0.17775	0.381457	-0.3864	0.078867	0.233942	0.257578	-0.15246	0.449889	6832153	7.309137	7.253935	7.771504	7.230447	7.276016	7.126287	7.610175	7.798177	7.383599	7.831052	7.448945	8.045192	Xrcc6	NM_010247
6832191	5003	9.980794	10.07992	9.870745	10.01888	-0.03809	0.794889	0.061038	0.678013	-0.14814	0.326381	0.209175	0.178135	-0.09913	0.504033	0.110048	0.459718	6832191	9.899024	9.982297	10.06106	10.05046	10.18039	10.00891	9.974261	10.06093	9.577045	9.832883	9.996192	10.22757	1500032L	BC092006
6832217	5006	7.678	7.858506	7.718066	7.894704	-0.2167	0.169789	-0.0362	0.807377	-0.17664	0.253701	0.14044	0.356811	-0.18051	0.244293	-0.04007	0.787353	6832217	7.653534	7.70369	7.676775	7.859387	7.87688	7.839252	7.705931	7.90963	7.538637	7.837393	7.63043	8.216289	Serhl	NM_023475
6832256	5007	6.923885	6.984424	6.927036	6.904932	0.018953	0.82413	0.079492	0.363667	0.022103	0.795616	0.057389	0.506525	-0.06054	0.484176	-0.00315	0.970486	6832256	7.117287	6.841972	6.812397	6.978694	7.047576	6.927003	6.986295	6.882143	6.912669	6.993913	6.848917	6.871967	Bik	NM_007546
6832259	5008	9.590404	9.523549	9.472035	9.400088	0.190316	0.06928	0.12346	0.210844	0.071947	0.450831	0.051514	0.585907	0.066855	0.482414	0.118369	0.228456	6832259	9.531448	9.561493	9.678271	9.631474	9.562755	9.376417	9.591043	9.413749	9.411313	9.268379	9.417494	9.514452	Tspo	NM_009775
6832276	5009	6.003189	6.024958	6.041756	6.062375	-0.05919	0.398943	-0.03742	0.58869	-0.02062	0.764193	-0.0168	0.806746	-0.02177	0.751538	-0.03857	0.577504	6832276	6.003109	6.011805	5.994652	6.085522	5.982535	6.006818	6.154886	5.935849	6.034533	6.170643	5.956477	6.060005	Mpped1	NM_172610
6832294	5010	6.229621	6.210118	6.297894	6.350113	-0.12049	0.257995	-0.14	0.194814	-0.05222	0.611976	-0.08778	0.400881	0.019503	0.84865	-0.06827	0.509693	6832294	6.242526	6.075479	6.370858	6.246052	6.259389	6.124912	6.410849	6.254552	6.228281	6.27038	6.519597	6.260362	Pnpla3	NM_054088
6832295	5011	8.929446	8.997268	8.981525	9.08734	-0.15789	0.215818	-0.09007	0.465283	-0.10582	0.394055	0.015743	0.896711	-0.06782	0.579618	-0.05208	0.669295	6832295	8.82928	8.920836	9.038223	9.022473	9.032781	8.93655	9.158895	8.962862	8.822819	8.962481	8.97989	9.31965	Samm50	NM_178614
6832300	5012	7.339753	7.365357	7.507684	7.616437	-0.27668	0.248969	-0.25108	0.291926	-0.10875	0.638183	-0.14233	0.540352	-0.0256	0.91124	-0.16793	0.472123	6832300	7.352747	7.336581	7.329931	7.58582	7.413432	7.09682	7.291728	7.957237	7.274086	7.339942	7.59183	7.91754	Parvb	NM_133167
6832302	5013	6.801493	7.049313	6.65053	6.126189	0.675303	0.00907	0.923124	0.001588	0.52434	0.028961	0.398783	0.078011	-0.24782	0.244706	0.150963	0.466327	6832302	7.037049	6.675723	6.691706	7.021616	7.28995	6.836373	6.948121	6.274444	6.729024	6.134978	6.269861	5.973729	Parvg	NM_022321
6832321	5014	7.611215	7.618466	7.450561	7.596725	0.01449	0.890349	0.02174	0.836257	-0.14616	0.189041	0.167905	0.137734	-0.00725	0.944179	0.160654	0.153239	6832321	7.654672	7.549514	7.62946	7.595571	7.807874	7.451952	7.613951	7.312966	7.424766	7.66562	7.582609	7.541947	Prr5	NM_146061
6832324	5015	9.377457	9.352672	9.332271	9.194187	0.18327	0.021876	0.158484	0.039672	0.138084	0.064944	0.0204	0.760151	0.024786	0.711107	0.045186	0.503923	6832324	9.314892	9.35533	9.46215	9.352275	9.380371	9.325369	9.312303	9.30477	9.379741	9.048635	9.237273	9.296654	Arhgap8	NM_028455
6832332	5016	8.909339	8.988952	8.816092	8.811882	0.097457	0.277876	0.17707	0.067318	0.00421	0.961122	0.17286	0.072794	-0.07961	0.369422	0.093247	0.297654	6832332	8.986946	8.860079	8.880993	8.875109	9.173561	8.918186	8.824117	8.907937	8.716222	8.758293	8.840345	8.837008	Nup50	NM_016714
6832343	5018	6.230866	6.331319	6.23277	6.163762	0.067104	0.337291	0.167557	0.034252	0.069008	0.324566	0.098549	0.172274	-0.10045	0.165059	-0.0019	0.977602	6832343	6.204593	6.178196	6.309809	6.30906	6.376736	6.308162	6.330401	6.102992	6.264918	6.078146	6.188174	6.224967	Fam118a	NM_133750
6832351	5019	8.320598	7.730457	7.499448	7.380296	0.940302	0.031125	0.350161	0.359525	0.119152	0.749334	0.231009	0.539299	0.59014	0.14003	0.82115	0.052123	6832351	8.51821	8.392557	8.051026	7.654805	8.285503	7.251064	7.924749	7.157322	7.416273	6.845294	7.933007	7.362587	Fbln1	NM_010180
6832356	5020	9.946172	10.06919	9.998861	9.766793	0.179379	0.118205	0.302394	0.018415	0.232068	0.053369	0.070326	0.512008	-0.12301	0.264385	-0.05269	0.621098	6832356	9.91283	9.909796	10.01589	10.0779	10.11259	10.01707	10.18713	9.820805	9.988647	9.649663	9.710785	9.939931	Atxn10	NM_016843
6832387	5021	6.160954	6.224599	6.177795	6.376657	-0.2157	0.147719	-0.15206	0.291355	-0.19886	0.177831	0.046804	0.737031	-0.06364	0.648971	-0.01684	0.903519	6832387	6.013009	6.185271	6.284581	6.251446	6.142437	6.279913	5.987245	6.505187	6.040953	6.392273	6.426062	6.311635	Ppara	NM_011144
6832394	5022	5.98527	6.174051	6.026246	5.63319	0.35208	0.040235	0.540861	0.005573	0.393056	0.025853	0.147805	0.334681	-0.18878	0.226204	-0.04098	0.783193	6832394	5.997299	5.950233	6.008278	6.017432	6.519533	5.985187	6.054709	5.906492	6.117536	5.610167	5.791409	5.497994	Gtse1	NM_013882
6832395	5023	6.132171	6.198291	6.279827	6.284758	-0.15259	0.121257	-0.08647	0.354741	-0.00493	0.956709	-0.08154	0.381409	-0.06612	0.474091	-0.14766	0.131991	6832395	6.009188	6.172763	6.214561	6.126225	6.26721	6.201438	6.312173	6.382415	6.144894	6.19263	6.422923	6.23872	Trmu	NM_028063
6832412	5024	7.354746	7.289118	7.323029	7.388931	-0.03419	0.789636	-0.09981	0.443818	-0.0659	0.609291	-0.03391	0.791279	0.065627	0.610759	0.031717	0.804445	6832412	7.472772	7.413684	7.177781	7.320951	7.252488	7.293916	7.429896	7.452117	7.087074	7.207653	7.487028	7.472112	Gramd4	NM_172611
6832420	5025	7.898247	7.976929	7.857811	7.895757	0.00249	0.971014	0.081172	0.256452	-0.03795	0.583481	0.119118	0.110662	-0.07868	0.270167	0.040436	0.559546	6832420	7.930083	7.911958	7.8527	8.055305	8.022138	7.853343	7.870387	7.763905	7.939141	7.865493	7.842927	7.978851	Tbc1d22a	NM_145476
6832462	5026	6.502139	6.484534	6.397821	6.391345	0.110793	0.336186	0.093188	0.414528	0.006475	0.953784	0.086713	0.446399	0.017605	0.874885	0.104318	0.363592	6832462	6.426244	6.387273	6.692899	6.628798	6.368223	6.45658	6.437809	6.511613	6.24404	6.472545	6.317842	6.383649	Fam19a5	NM_134096
6832530	5027	10.28004	10.21484	10.74593	10.63647	-0.35642	0.225398	-0.42163	0.158808	0.109463	0.6972	-0.53109	0.086009	0.065207	0.816125	-0.46589	0.124303	6832530	10.3342	10.2312	10.27473	9.84389	10.15452	10.6461	10.90141	10.9304	10.40598	10.80184	10.96371	10.14385	Creld2	NM_029720
6832532	5028	8.503866	8.828641	8.409314	9.539523	-1.03566	0.059182	-0.71088	0.169813	-1.13021	0.04327	0.419327	0.399474	-0.32478	0.51015	0.094551	0.845969	6832532	8.251026	8.346215	8.914356	9.366732	8.109237	9.009955	8.385543	8.614064	8.228336	9.306267	8.816461	10.49584	Pim3	NM_145478
6832540	5031	8.099983	8.191936	8.215553	8.197881	-0.0979	0.62586	-0.00594	0.976197	0.017672	0.929335	-0.02362	0.905681	-0.09195	0.646683	-0.11557	0.566081	6832540	8.096289	8.042165	8.161495	8.141251	8.410172	8.024386	8.182447	8.603817	7.860394	8.254323	8.368807	7.970512	Trabd	NM_026485
6832542	5032	7.47389	7.590685	7.534756	7.419052	0.054838	0.56497	0.171633	0.097117	0.115705	0.240988	0.055929	0.557427	-0.1168	0.23696	-0.06087	0.524022	6832542	7.439304	7.490531	7.491834	7.595535	7.652315	7.524205	7.593163	7.665225	7.345881	7.389001	7.561882	7.306272	1300018J	NM_027905
6832564	5033	7.675265	7.723576	7.667471	7.709478	-0.03421	0.677346	0.014098	0.863227	-0.04201	0.610459	0.056105	0.499073	-0.04831	0.559031	0.007794	0.924075	6832564	7.598532	7.712348	7.714915	7.857565	7.726224	7.586939	7.646434	7.786398	7.569581	7.66069	7.701006	7.766738	Saps2	NM_026813
6832571	5034	9.516372	10.89795	10.20828	12.47369	-2.95732	0.00018	-1.57575	0.008249	-2.26542	0.001038	0.689672	0.165608	-1.38157	0.015672	-0.6919	0.164402	6832571	8.972778	10.16218	9.414159	11.28698	10.29525	11.11161	9.819109	10.83735	9.968366	11.87005	12.68166	12.86937	Adm2	NM_182928
6832573	5036	8.043397	8.029593	8.131801	8.135125	-0.09173	0.37174	-0.10553	0.308034	-0.00332	0.973492	-0.10221	0.322535	0.013804	0.890292	-0.0884	0.388459	6832573	8.053097	7.971019	8.106075	8.244762	7.969704	7.874314	8.162626	8.111412	8.121366	8.05006	8.084955	8.270359	Ncaph2	NM_001115132
6832580	5037	6.247254	6.501311	6.268657	6.201262	0.045992	0.806203	0.300049	0.136636	0.067395	0.719834	0.232654	0.235469	-0.25406	0.198838	-0.0214	0.908968	6832580	6.139129	6.529582	6.073052	6.424431	6.5213	6.326372	6.219684	6.551435	6.034852	6.356041	6.118184	6.129561	Chkb	NM_007692
6832587	5039	7.358094	7.405051	7.208797	7.547909	-0.18981	0.243079	-0.14286	0.370631	-0.33911	0.054433	0.196254	0.228808	-0.04696	0.763182	0.149297	0.35058	6832587	7.536879	7.374557	7.162847	7.505606	7.42953	7.280017	7.178141	7.32726	7.12099	7.336883	7.446032	7.860811	Shank3	NM_021423
6832588	5040	6.174247	6.233606	6.180581	6.089337	0.08491	0.409789	0.144269	0.177735	0.091244	0.377343	0.053025	0.601843	-0.05936	0.560045	-0.00633	0.949863	6832588	6.132161	6.175081	6.215498	6.126844	6.217838	6.356135	6.33026	6.11044	6.101042	6.074858	5.938021	6.255131	Acr	NM_013455
6832713	5041	7.694229	7.759669	7.699764	8.25978	-0.56555	0.000372	-0.50011	0.000826	-0.56002	0.000397	0.059905	0.550987	-0.06544	0.515738	-0.00553	0.955553	6832713	7.718563	7.72307	7.641055	7.624956	7.779141	7.874911	7.661482	7.746341	7.691469	8.38666	8.04264	8.350039	Lrrk2	NM_025730
6832775	5044	8.17163	8.081091	8.002646	7.940202	0.231428	0.064955	0.140889	0.229244	0.062444	0.579849	0.078445	0.489242	0.090539	0.427143	0.168984	0.157078	6832775	8.232816	8.111968	8.170107	7.944138	8.323958	7.975178	8.046682	7.919315	8.041942	7.82286	8.080202	7.917545	Pphln1	NM_146062
6832848	5045	8.137276	8.162849	8.080046	7.669903	0.467373	0.014991	0.492946	0.011625	0.410143	0.026754	0.082803	0.599531	-0.02557	0.870121	0.05723	0.715392	6832848	8.321612	8.070237	8.019979	8.032884	8.389807	8.065857	8.237557	7.872529	8.130053	7.516762	7.887433	7.605515	Irak4	NM_029926
6832857	5046	7.739451	7.789834	7.905732	7.191895	0.547556	0.029929	0.59794	0.02058	0.713838	0.008881	-0.1159	0.592238	-0.05038	0.814501	-0.16628	0.44665	6832857	7.829774	7.673989	7.71459	7.833964	7.610977	7.924562	8.105867	8.024327	7.587003	6.753124	7.346459	7.476101	Tmem117	NM_178789
6832923	5047	9.829776	9.80872	9.75356	9.447215	0.382561	0.013463	0.361504	0.017523	0.306344	0.03538	0.05516	0.661104	0.021057	0.866382	0.076217	0.546955	6832923	9.817876	9.781519	9.889934	9.70541	9.944159	9.77659	9.97381	9.564892	9.721977	9.275319	9.606267	9.46006	Ano6	NM_175344
6832947	5048	9.193363	9.333559	9.077538	9.126696	0.066667	0.261921	0.206863	0.005664	-0.04916	0.399446	0.256022	0.001677	-0.1402	0.034811	0.115826	0.069266	6832947	9.180378	9.190525	9.209187	9.310466	9.399732	9.29048	9.014715	9.216616	9.001282	9.119064	9.119916	9.141109	Arid2	NM_175251
6833005	5049	6.3333	6.378015	6.299307	6.236875	0.096425	0.458582	0.141141	0.287354	0.062432	0.627734	0.078709	0.542782	-0.04472	0.727376	0.033993	0.790649	6833005	6.291224	6.451124	6.257551	6.630465	6.388824	6.114757	6.176719	6.414726	6.306475	6.245936	6.25409	6.210598	Fam113b	NM_172293
6833029	5050	10.69863	10.61501	10.65706	10.69697	0.001663	0.986168	-0.08196	0.403855	-0.03991	0.679153	-0.04205	0.663152	0.08362	0.394812	0.04157	0.66672	6833029	10.7659	10.67702	10.65298	10.63	10.76072	10.45432	10.75243	10.71535	10.50341	10.61539	10.6969	10.77862	Slc48a1	NM_026353
6833057	5051	8.647715	8.537999	8.279945	8.610498	0.037217	0.946805	-0.0725	0.89663	-0.33055	0.557874	0.258054	0.645887	0.109716	0.844241	0.36777	0.515521	6833057	7.840757	8.219131	9.883257	9.072259	8.384744	8.156994	8.830351	8.018961	7.990522	8.326347	8.512132	8.993016	Pfkm	NM_021514
6833100	5057	7.798427	8.251098	7.190538	7.029727	0.7687	0.009019	1.221371	0.000614	0.16081	0.494045	1.060561	0.001491	-0.45267	0.078423	0.60789	0.026715	6833100	8.062564	7.609606	7.723112	8.537869	8.44977	7.765656	7.21569	7.211044	7.144879	7.225444	7.125987	6.737751	Cacnb3	NM_007581
6833151	5063	8.175993	8.55032	8.172989	8.125251	0.050742	0.646657	0.425069	0.00401	0.047738	0.666022	0.377331	0.007606	-0.37433	0.007926	0.003004	0.978199	6833151	8.189318	8.089176	8.249485	8.388301	8.72102	8.541639	8.244868	8.125635	8.148464	8.324044	8.039566	8.012142	Spats2	NM_139140
6833153	5064	10.82982	10.85538	10.94677	10.85379	-0.02398	0.856913	0.001583	0.99049	0.092977	0.490802	-0.09139	0.497993	-0.02556	0.847599	-0.11695	0.390258	6833153	10.74941	10.81431	10.92573	10.95725	10.62638	10.9825	10.90371	11.14921	10.78739	10.76276	10.79115	11.00747	Kcnh3	NM_010601
6833157	5065	7.487001	7.574993	7.486137	7.550273	-0.06327	0.550511	0.02472	0.813764	-0.06414	0.545209	0.088856	0.406988	-0.08799	0.411366	0.000864	0.993419	6833157	7.47811	7.577068	7.405825	7.619886	7.601814	7.503279	7.420758	7.735315	7.302338	7.572538	7.530435	7.547846	Prpf40b	NM_018786

6833161	5066	11.49955	11.54676	11.48039	11.48515	0.014393	0.858356	0.061603	0.45294	-0.00477	0.952826	0.06637	0.420092	-0.04721	0.562234	0.01916	0.812362	6833161	11.57176	11.48339	11.44349	11.55893	11.47566	11.60568	11.53494	11.58387	11.32235	11.39793	11.47484	11.58269	Tmbim6	NM_026669
6833171	5067	6.960607	6.975052	7.003002	7.234268	-0.27366	0.016471	-0.25922	0.021024	-0.23127	0.033903	-0.02795	0.765359	-0.01444	0.877159	-0.04239	0.651998	6833171	7.003467	6.841786	7.036569	6.924127	7.061592	6.939437	7.053267	7.098607	6.857132	7.371227	7.120253	7.211323	Aqp2	NM_009699
6833175	5068	9.149838	9.92123	9.025467	10.21201	-1.06217	0.004949	-0.29078	0.323889	-1.18654	0.002653	0.895763	0.011909	-0.77139	0.023608	0.124371	0.664924	6833175	9.019259	9.324048	9.106208	9.860209	10.25344	9.65004	8.917022	9.34094	8.818439	9.972755	9.861908	10.80136	Aqp5	NM_009701
6833176	5069	5.636311	5.976481	5.810557	6.500698	-0.86439	0.018905	-0.52422	0.113142	-0.69014	0.047273	0.165924	0.588829	-0.34017	0.281676	-0.17425	0.570651	6833176	5.45189	5.720728	5.736314	6.083078	5.690817	6.155548	5.450732	6.531151	5.449788	6.637127	6.260552	6.604415	Aqp6	NM_175087
6833184	5071	7.797892	7.820617	7.707181	7.663571	0.134321	0.089683	0.157045	0.053988	0.04361	0.54832	0.113436	0.141719	-0.02272	0.752378	0.090711	0.228638	6833184	7.780301	7.854135	7.759241	7.802038	7.968925	7.690887	7.727414	7.729371	7.664758	7.578459	7.691937	7.720318	Smarcd1	NM_031842
6833185	5072	9.535413	9.138595	9.00706	9.107377	0.428036	0.107888	0.031218	0.898237	-0.10032	0.682594	0.131534	0.593277	0.396818	0.131865	0.528353	0.055928	6833185	9.293491	9.516908	9.79584	9.002613	9.222182	9.190989	9.269765	8.500825	9.250591	8.811247	9.259747	9.251137	Gpd1	NM_010271
6833186	5073	8.231415	8.449578	8.418239	8.33898	-0.10756	0.561802	0.110598	0.551071	0.079259	0.667451	0.031339	0.864414	-0.21816	0.254521	-0.18682	0.323889	6833186	7.96373	8.255504	8.47501	8.328909	8.579157	8.440668	8.489554	8.631262	8.1339	8.103906	8.486832	8.426201	2310016M	NM_183256
6833216	5074	6.100172	5.999483	6.297497	6.141061	-0.04089	0.851966	-0.14158	0.523324	0.156436	0.481951	-0.29801	0.197714	0.100689	0.647746	-0.19732	0.379516	6833216	6.064457	6.071894	6.164165	6.132563	6.35244	5.513446	6.301965	6.49449	6.096036	5.988654	6.36068	6.07385	Larp4	NM_001024526
6833220	5075	8.126932	8.084499	8.050394	7.737767	0.389165	0.002169	0.346732	0.004202	0.312627	0.00733	0.034105	0.707362	0.042433	0.641293	0.076538	0.408007	6833220	8.252408	8.090666	8.037722	8.045745	8.178712	8.02904	8.121179	8.085754	7.944248	7.665478	7.892994	7.654828	Dip2b	NM_001159361
6833223	5076	9.347868	9.431273	9.285152	9.242237	0.10563	0.485498	0.189035	0.227004	0.042915	0.773972	0.146121	0.341372	-0.0834	0.579564	0.062716	0.675646	6833223	9.270937	9.302417	9.470249	9.407516	9.621774	9.264528	9.065207	9.588118	9.202131	9.145397	9.261851	9.319464	Atf1	NM_007497
6833243	5080	8.590622	8.736823	8.576579	8.660183	-0.06956	0.588135	0.07664	0.55155	-0.0836	0.516899	0.160244	0.229959	-0.1462	0.269782	0.014042	0.91214	6833243	8.546737	8.593091	8.632037	8.817939	8.828915	8.563615	8.600709	8.496843	8.632186	8.448642	8.597731	8.934176	Letmd1	NM_134093
6833259	5081	10.77594	10.79455	10.85381	10.74301	0.032933	0.703099	0.05154	0.553561	0.1108	0.220424	-0.05926	0.497329	-0.01861	0.828959	-0.07787	0.377561	6833259	10.74243	10.71359	10.8718	10.82617	10.90894	10.64853	10.86039	10.84176	10.85927	10.6627	10.67389	10.89243	Dazap2	NM_011873
6833276	5082	7.354572	7.163998	7.256565	7.074862	0.279709	0.003699	0.089136	0.233187	0.181703	0.030223	-0.09257	0.217263	0.190573	0.024775	0.098007	0.193932	6833276	7.442689	7.279205	7.341821	7.18051	7.204205	7.10728	7.404529	7.183045	7.182121	7.052618	7.136106	7.035863	Slc4a8	NM_021530
6833305	5085	8.793087	8.741798	8.755925	8.968594	-0.17551	0.161767	-0.2268	0.081524	-0.21267	0.098726	-0.01413	0.904314	0.051289	0.664323	0.037162	0.752497	6833305	8.832157	8.838779	8.708325	8.949022	8.580983	8.695388	8.785581	8.84334	8.638853	8.783184	9.063298	9.059299	Acvrl1	NM_009612
6833306	5086	8.30615	8.405641	8.224381	8.337511	-0.03136	0.796525	0.06813	0.578412	-0.11313	0.364356	0.18126	0.161912	-0.09949	0.422258	0.081769	0.506665	6833306	8.249877	8.315135	8.353439	8.379387	8.46061	8.376925	8.358705	8.091358	8.22308	8.261962	8.138926	8.611644	Acvr1b	NM_007395
6833308	5087	7.999258	8.019927	7.704613	8.024038	-0.02478	0.840849	-0.00411	0.973386	-0.31943	0.028189	0.315314	0.029735	-0.02067	0.866933	0.294645	0.038921	6833308	8.025621	8.107168	7.864985	8.047252	8.2141	7.798428	7.622942	7.865976	7.624921	8.047993	8.094042	7.93008	Grasp	NM_019518
6833311	5088	8.279763	8.187078	7.494858	7.913534	0.366229	0.151981	0.273544	0.270885	-0.41868	0.107859	0.69222	0.017258	0.092685	0.699104	0.784905	0.009449	6833311	8.056955	8.382141	8.400193	8.219427	8.482668	7.859138	7.230691	7.880963	7.37292	7.684876	8.199882	7.855844	Nr4a1	NM_010444
6833314	5089	7.152893	7.302299	7.037979	6.959885	0.193007	0.12486	0.342414	0.01604	0.078094	0.507599	0.26432	0.046871	-0.14941	0.221173	0.114913	0.33734	6833314	7.12564	7.1684	7.164638	7.292522	7.533694	7.080681	7.18665	7.034709	6.892579	6.905548	7.00421	6.969898	9430023L	BC016463
6833326	5090	9.283615	9.611624	9.669798	9.919234	-0.63562	0.016832	-0.30761	0.18339	-0.24944	0.271543	-0.05817	0.789973	-0.32801	0.159042	-0.38618	0.104901	6833326	9.023179	9.346165	9.481501	9.858639	9.562704	9.413529	9.551998	10.00183	9.455566	9.831217	9.698506	10.22798	Krt7	NM_033073
6833382	5092	10.18144	10.31932	10.03794	10.01005	0.171396	0.307976	0.309276	0.085054	0.027897	0.86375	0.281379	0.111687	-0.13788	0.406645	0.143499	0.388656	6833382	9.97609	10.26734	10.3009	10.19865	10.5811	10.17822	10.3026	9.897421	9.913812	10.00482	9.900102	10.12522	Krt18	NM_010664
6833387	5093	8.837282	8.94279	8.69451	9.190171	-0.35289	0.164468	-0.24738	0.314661	-0.49566	0.063829	0.24828	0.313016	-0.10551	0.659453	0.142773	0.553041	6833387	8.630173	9.085478	8.796196	9.129473	8.82758	8.871317	8.478373	8.977197	8.627959	8.878198	9.028635	9.663679	Tenc1	NM_153533
6833393	5094	7.013001	6.900848	6.700064	6.210442	0.802559	0.058942	0.690407	0.094979	0.489623	0.216277	0.200784	0.596985	0.112152	0.766305	0.312936	0.41583	6833393	7.111612	6.972876	6.954514	6.792558	7.473501	6.436486	7.260798	6.695713	6.143682	5.790431	6.682617	6.158277	Igfbp6	NM_008344
6833398	5096	7.68221	7.882275	7.749917	7.698612	-0.0164	0.832171	0.183663	0.039823	0.051305	0.512757	0.132358	0.115224	-0.20006	0.028328	-0.06771	0.392477	6833398	7.664288	7.694236	7.688107	7.768846	8.0234	7.854578	7.820158	7.632112	7.79748	7.665427	7.642542	7.787866	Zfp740	NM_153194
6833403	5098	8.277476	8.255846	8.319106	8.318906	-0.04143	0.565749	-0.06306	0.388559	0.0002	0.997764	-0.06326	0.387128	0.02163	0.762479	-0.04163	0.563915	6833403	8.216832	8.25484	8.360757	8.255465	8.340439	8.171634	8.348553	8.4252	8.183564	8.297445	8.350209	8.309063	Mfsd5	NM_134100
6833408	5100	7.498401	7.525147	7.504815	7.479801	0.018599	0.859083	0.045345	0.666717	0.025014	0.811438	0.020332	0.846145	-0.02675	0.798703	-0.00641	0.951132	6833408	7.357716	7.438138	7.699348	7.482666	7.659276	7.433498	7.637478	7.454727	7.42224	7.511327	7.503182	7.424895	Myg1	NM_021713
6833414	5101	7.35077	7.523853	7.39003	7.299011	0.05176	0.626439	0.224842	0.059136	0.091019	0.399457	0.133823	0.227045	-0.17308	0.129035	-0.03926	0.711077	6833414	7.337692	7.414165	7.300454	7.496266	7.675681	7.399612	7.311291	7.532947	7.325851	7.303556	7.452897	7.140579	Sp1	NM_013672
6833418	5103	8.562181	8.225858	8.531228	8.20618	0.356002	0.008652	0.019678	0.853369	0.325049	0.013535	-0.30537	0.018085	0.336323	0.011485	0.030953	0.771636	6833418	8.62167	8.522878	8.541996	8.240108	8.455258	7.982208	8.540653	8.581657	8.471375	8.159343	8.216595	8.242601	Prr13	NM_025385
6833420	5104	11.75993	11.79379	11.83204	11.84474	-0.0848	0.083526	-0.05095	0.26917	-0.01269	0.774907	-0.03825	0.398693	-0.03386	0.452851	-0.07211	0.131372	6833420	11.66841	11.82082	11.79057	11.78342	11.82357	11.77438	11.78628	11.89345	11.8164	11.82655	11.83042	11.87724	Pcbp2	NM_001103165
6833430	5105	7.622051	7.786617	7.614545	7.753636	-0.13158	0.351445	0.032981	0.810388	-0.13909	0.326202	0.172072	0.231824	-0.16457	0.251028	0.007507	0.956373	6833430	7.558431	7.678175	7.629548	7.739267	7.852819	7.767765	7.575076	7.936646	7.331912	7.823279	7.666125	7.771503	Tarbp2	NM_009319
6833503	5114	9.133716	9.11693	9.196731	9.222649	-0.08893	0.348827	-0.10572	0.270799	-0.02592	0.779186	-0.0798	0.397964	0.016786	0.855693	-0.06302	0.500759	6833503	9.145554	9.085875	9.169718	9.005462	9.239832	9.105496	9.196481	9.36066	9.033051	9.208691	9.302095	9.15716	Copz1	NM_019817
6833513	5115	8.140516	8.032771	8.148365	7.000781	1.139735	0.006295	1.03199	0.01047	1.147584	0.00607	-0.11559	0.719281	0.107745	0.737475	-0.00785	0.980446	6833513	8.545323	8.043161	7.833063	7.988595	8.294572	7.815145	8.559831	7.663363	8.221901	6.873755	7.474453	6.654135	Nckap1l	NM_153505
6833516	5116	6.425815	6.627058	6.435588	6.291154	0.134662	0.24403	0.335904	0.013872	0.144434	0.21434	0.19147	0.111576	-0.20124	0.097013	-0.00977	0.929529	6833516	6.485389	6.499932	6.292125	6.587028	6.838569	6.455576	6.555929	6.352283	6.398552	6.323524	6.3477	6.202237	Pde1b	NM_008800
6833531	5117	8.467796	8.897954	8.632677	8.749096	-0.2813	0.355146	0.148858	0.617591	-0.11642	0.695274	0.265278	0.381783	-0.43016	0.171823	-0.16488	0.580951	6833531	7.957825	8.835903	8.609659	8.625959	9.061116	9.006788	8.749937	8.241846	8.906247	8.532926	9.127685	8.586678	Ghr	NM_010284
6833599	5118	7.550746	7.633329	7.71251	7.711708	-0.16096	0.119278	-0.07838	0.420388	0.000801	0.993284	-0.07918	0.415851	-0.08258	0.396973	-0.16176	0.117713	6833599	7.472714	7.564701	7.614823	7.576307	7.706078	7.617603	7.833251	7.748524	7.555754	7.556842	7.731733	7.84655	Fbxo4	NM_134099
6833600	5119	8.381945	8.764577	8.656922	8.632155	-0.25021	0.012236	0.132422	0.126734	0.024767	0.75808	0.107655	0.203299	-0.38263	0.001158	-0.27498	0.007632	6833600	8.360352	8.44384	8.341642	8.701228	8.691662	8.900842	8.709614	8.678653	8.5825	8.574614	8.549401	8.772451	AW549877	NM_145930
6833640	5120	7.21918	6.970678	7.098145	6.525847	0.693333	0.009072	0.44483	0.05946	0.572298	0.022358	-0.12747	0.546904	0.248502	0.254988	0.121035	0.566868	6833640	7.501855	7.081344	7.074341	6.845555	7.241394	6.825084	7.253338	6.932692	7.108405	6.336857	6.901136	6.339549	Ptger4	NM_001136079
6833736	5121	9.945057	9.833455	9.756181	8.60874	1.336317	0.028567	1.224715	0.040389	1.147441	0.051373	0.077273	0.881327	0.111602	0.829427	0.188875	0.716174	6833736	10.05227	9.979173	9.803727	9.336749	10.45346	9.710155	10.2513	8.874314	10.14293	8.753547	9.289181	7.783492	Osmr	NM_011019
6833830	5123	7.042862	7.308319	7.094767	7.078517	-0.03565	0.826033	0.229802	0.181406	0.016251	0.920104	0.213552	0.210831	-0.26546	0.129321	-0.05191	0.74942	6833830	7.140996	6.787893	7.199697	7.271693	7.540906	7.112358	7.167105	7.177175	6.940022	6.895531	7.260796	7.079223	Wdr70	NM_001081402
6833842	5124	9.320823	9.348541	9.279299	9.351885	-0.03106	0.625307	-0.00334	0.957749	-0.07259	0.269427	0.069242	0.290447	-0.02772	0.662499	0.041524	0.516408	6833842	9.314376	9.400741	9.247352	9.230963	9.419738	9.394922	9.267784	9.345461	9.224652	9.317769	9.406291	9.331594	Nipbl	NM_027707
6833902	5125	6.795466	6.589744	6.47679	6.768947	0.026519	0.946832	-0.1792	0.654346	-0.29216	0.470162	0.112954	0.776923	0.205722	0.608003	0.318676	0.432289	6833902	6.905929	6.759826	6.720643	6.312123	7.10157	6.355538	6.389632	6.637782	6.402956	7.627236	6.675627	6.003979	Slc1a3	NM_148938
6833919	5126	6.256481	6.394775	6.180898	6.147591	0.108889	0.169724	0.247183	0.009013	0.033306	0.656677	0.213877	0.018035	-0.13829	0.091602	0.075583	0.325496	6833919	6.180943	6.243134	6.345365	6.25149	6.428877	6.503957	6.221545	6.146249	6.174899	6.079628	6.232881	6.130265	Skp2	NM_145468
6833937	5127	7.316206	6.899523	6.993291	5.960991	1.355216	0.009116	0.938532	0.045459	1.0323	0.031447	-0.09377	0.818999	0.416683	0.324017	0.322915	0.438984	6833937	7.582108	7.444765	6.921746	7.2231	7.023181	6.452288	7.14591	6.584574	7.249389	6.287838	6.471214	5.12392	Il7r	NM_008372
6833972	5128	8.548313	8.800705	8.784522	8.60988	-0.06157	0.674761	0.190825	0.214096	0.174642	0.251825	0.016183	0.9117	-0.25239	0.112091	-0.23621	0.133357	6833972	8.599888	8.606592	8.438458	8.573639	8.959617	8.868859	8.928113	8.897816	8.527637	8.446739	8.713422	8.66948	Bxdc2	NM_026396
6833980	5129	8.841842	9.043119	8.784404	8.821036	0.020806	0.867828	0.222083	0.103957	-0.03663	0.769946	0.258715	0.065099	-0.20128	0.135004	0.057438	0.647894	6833980	8.877675	8.986825	8.661027	8.991382	9.212778	8.925197	8.893477	8.742028	8.717708	8.740166	9.015736	8.707207	Rai14	NM_030690
6834013	5131	8.045967	8.06738	8.158235	8.08771	-0.04174	0.729311	-0.02033	0.865766	0.070526	0.561596	-0.09086	0.457787	-0.02141	0.858703	-0.11227	0.363297	6834013	8.032154	8.109907	7.995841	7.921287	8.315869	7.964984	8.276285	8.178504	8.019917	8.051826	8.221829	7.989474	Tars	NM_033074
6834025	5132	7.50863	7.99677	7.579505	8.818804	-1.31017	0.000736	-0.82203	0.010539	-1.2393	0.001046	0.417265	0.130427	-0.48814	0.084146	-0.07087	0.781958	6834025	7.137012	7.83478	7.554099	7.804639	7.914674	8.270998	7.231727	7.796046	7.710743	8.756534	8.550464	9.149414	Npr3	NM_008728
6834031	5133	7.326571	7.885157	7.725035	7.675247	-0.34868	0.036921	0.20991	0.170672	0.049789	0.7303	0.160121	0.284046	-0.55859	0.003919	-0.39846	0.021236	6834031	7.079764	7.460391	7.439558	7.762431	7.920728	7.972311	7.815063	7.606958	7.753085	7.46458	7.661329	7.899831	Sub1	NM_011294
6834063	5134	7.026532	6.977608	6.933853	6.96634	0.060191	0.485591	0.011268	0.894523	-0.03249	0.703456	0.043756	0.609542	0.048923	0.568786	0.092679	0.29294	6834063	6.950806	7.058686	7.070103	6.961938	7.028255	6.942632	6.876495	6.968086	6.956977	6.87204	7.17209	6.854891	6030458C	NM_029998
6834108	5135	6.995147	7.336136	6.744812	6.499184	0.495963	0.180534	0.836953	0.038366	0.245629	0.488197	0.591324	0.118385	-0.34099	0.34267	0.250335	0.480165	6834108	6.653963	6.672995	7.658483	6.885417	7.874654	7.248338	6.445344	7.050559	6.738534	6.632369	6.44945	6.415732	Cdh6	NM_007666
6834291	5136	7.699209	7.544445	7.682077	7.572106	0.127103	0.123223	-0.02766	0.717464	0.109971	0.174407	-0.13763	0.09909	0.154764	0.069184	0.017132	0.822204	6834291	7.66019	7.731659	7.705777	7.510119	7.686362	7.436854	7.695342	7.684486	7.666402	7.609618	7.67011	7.436589	Acot10	NM_022816
6834413	5138	8.589287	8.719071	8.675917	8.561623	0.027665	0.697586	0.157449	0.05103	0.114294	0.134584	0.043154	0.547222	-0.12978	0.095418	-0.08663	0.242635	6834413	8.663073	8.552224	8.552565	8.745946	8.72362	8.687648	8.817029	8.67536	8.535362	8.520753	8.629547	8.534568	Basp1	NM_027395
6834504	5139	5.833377	6.110639	5.887288	5.67688	0.156498	0.203773	0.43376	0.004977	0.210409	0.099835	0.223351	0.083684	-0.27726	0.039825	-0.05391	0.646304	6834504	6.014042	5.921833	5.564257	6.151752	6.141648	6.038518	5.896525	5.785	5.98034	5.591868	5.755909	5.682862	Fbxl7	NM_176959
6834558	5140	6.445427	6.478598	6.320772	6.366337	0.079091	0.297569	0.112261	0.15244	-0.04556	0.538913	0.157826	0.05689	-0.03317	0.652769	0.124655	0.117157	6834558	6.509168	6.403995	6.423119	6.491314	6.584755	6.359725	6.393136	6.279306	6.289874	6.262891	6.369736	6.466383	Fam105b	NM_001013792
6834560	5141	7.078114	7.090627	7.154744	6.025383	1.052731	0.022823	1.065244	0.021679	1.129361	0.016682	-0.06412	0.868333	-0.01251	0.974167	-0.07663	0.843003	6834560	7.573852	6.951679	6.70881	7.1477	7.594402	6.52978	7.41749	6.973397	7.073346	5.552968	6.629365	5.893816	Fam105a	BC052328
6834561	5142	8.054939	8.1628	8.090047	7.88671	0.168229	0.158753	0.27609	0.034142	0.203337	0.097129	0.072753	0.520442	-0.10786	0.348207	-0.03511	0.753998	6834561	8.118763	7.998737	8.047316	8.126916	8.192976	8.168507	8.16704	8.203346	7.899754	7.676105	8.061195	7.92283	Trio	NM_001081302
6834728	5143	6.248022	6.426212	6.371426	6.289174	-0.04115	0.755172	0.137038	0.313839	0.082253	0.536937	0.054785	0.678783	-0.17819	0.199813	-0.1234	0.361487	6834728	6.284905	6.287905	6.171257	6.663709	6.195659	6.419267	6.519492	6.362252	6.232535	6.282436	6.159716	6.425369	Ankrd33b	NM_027496
6834729	5144	8.013	7.882784	7.865701	8.129433	-0.11643	0.559345	-0.24665	0.232976	-0.26373	0.205009	0.017083	0.930985	0.130216	0.514961	0.147299	0.463096	6834729	8.199104	7.89924	7.940657	7.817211	7.669292	8.16185	7.936167	8.147504	7.513433	8.239879	7.949458	8.198961	Ankrd33b	NM_026153
6834745	5146	9.637576	9.718132	9.686353	9.782172	-0.1446	0.144986	-0.06404	0.494802	-0.09582	0.315765	0.031779	0.731817	-0.08056	0.394552	-0.04878	0.60076	6834745	9.589995	9.678202	9.644531	9.68625	9.705148	9.762998	9.7575	9.791018	9.510541	9.67501	9.724201	9.947304	6-Mar	NM_172606
6834752	5147	9.296534	9.409512	9.403228	9.183333	0.113202	0.415564	0.226179	0.124607	0.219895	0.133919	0.006284	0.963153	-0.11298	0.416448	-0.10669	0.4418	6834752	9.318175	9.247334	9.324094	9.254567	9.630208	9.343761	9.663847	9.344026	9.20181	9.081208	9.222415	9.246375	Cct5	NM_007637
6834865	5148	7.061569	6.952817	7.238414	6.967802	0.093767	0.622698	-0.01498	0.936839	0.270612	0.178002	-0.2856	0.157739	0.108752	0.569276	-0.17684	0.362796	6834865	7.052297	7.094735	7.037675	6.837865	6.961552	7.059034	7.244234	7.498593	6.972414	6.702985	7.35807	6.84235	Rpl30	NM_009083
6834870	5149	8.985682	8.692178	8.66725	8.672297	0.313386	0.143859	0.019881	0.920666	-0.00505	0.979823	0.024928	0.900639	0.293505	0.16765	0.318433	0.138331	6834870	9.07875	8.702549	9.175748	8.620231	8.748713	8.707589	8.745865	8.723193	8.532691	8.407915	8.501867	9.107108	Hrsp12	NM_008287

6834877	5150	9.109202	8.9243	9.321119	9.246709	-0.13751	0.212878	-0.32241	0.013122	0.07441	0.484791	-0.39682	0.004506	0.184903	0.106237	-0.21192	0.070454	6834877	9.04222	9.16825	9.117137	9.019757	8.803385	8.949757	9.509897	9.281343	9.172118	9.181901	9.392488	9.165738	Nipal2	NM_145469
6834890	5151	8.515581	8.438454	8.419363	8.436958	0.078623	0.364186	0.001495	0.985849	-0.0176	0.834913	0.019091	0.821155	0.077127	0.372909	0.096218	0.27288	6834890	8.518382	8.50797	8.520391	8.329016	8.542481	8.443864	8.274264	8.400107	8.583717	8.370004	8.502563	8.438308	Stk3	NM_019635
6834951	5152	12.49526	12.57927	12.47395	12.57032	-0.07506	0.616153	0.008947	0.951964	-0.09637	0.52202	0.105317	0.485251	-0.08401	0.575542	0.021307	0.885987	6834951	12.56992	12.71942	12.19643	12.54243	12.4613	12.73407	12.56342	12.48082	12.37761	12.44603	12.52346	12.74147	Cox6c	NM_053071
6834975	5153	9.110729	9.083428	8.837732	9.208507	-0.09778	0.579245	-0.12508	0.480893	-0.37077	0.059799	0.245696	0.184541	0.027301	0.875817	0.272997	0.145314	6834975	9.034366	8.976894	9.320926	9.216752	9.247319	8.786212	9.095124	8.687845	8.730227	9.175381	9.082357	9.367782	Rnf19a	NM_013923
6835001	5154	9.595187	9.811141	9.746667	10.15878	-0.56359	0.018981	-0.34764	0.108276	-0.41211	0.064481	0.064475	0.746051	-0.21595	0.294033	-0.15148	0.453591	6835001	9.370893	9.533033	9.881635	9.97983	9.692649	9.760945	9.863196	9.85654	9.520264	10.04167	9.930028	10.50464	Ankrd46	NM_175134
6835004	5155	5.609858	5.72901	6.00328	5.969005	-0.35915	0.533942	-0.23999	0.675535	0.034275	0.952063	-0.27427	0.633001	-0.11915	0.834673	-0.39342	0.496716	6835004	5.708936	5.444778	5.675859	6.223882	5.409974	5.553175	5.455199	7.112413	5.442228	5.650437	5.338497	6.918081	Snx31	NM_025712
6835005	5156	12.15081	12.17096	12.19826	12.33213	-0.18133	0.066595	-0.16117	0.095964	-0.13387	0.155798	-0.0273	0.757492	-0.02015	0.819459	-0.04746	0.593791	6835005	12.09258	12.15071	12.20913	12.1181	12.24063	12.15415	12.08592	12.24426	12.26461	12.21394	12.26319	12.51927	Pabpc1	NM_008774
6835010	5157	11.52541	11.42444	11.47736	11.42153	0.103877	0.141906	0.00291	0.964714	0.055827	0.406759	-0.05292	0.430622	0.100967	0.151939	0.04805	0.472659	6835010	11.61622	11.48295	11.47706	11.38526	11.53441	11.35366	11.54808	11.49068	11.39332	11.37376	11.47774	11.4131	Ywhaz	NM_011740
6835016	5158	8.339344	8.192195	8.194253	8.341602	-0.00226	0.988914	-0.14941	0.370509	-0.14735	0.376805	-0.00206	0.989891	0.14715	0.377417	0.145091	0.383799	6835016	8.320968	8.224423	8.472642	8.18832	8.299246	8.089018	8.207417	8.331342	8.044	8.130294	8.186852	8.707659	Zfp706	NM_026521
6835045	5159	6.321144	6.587183	6.358389	6.403863	-0.08272	0.504123	0.18332	0.159733	-0.04547	0.710634	0.228794	0.089099	-0.26604	0.054616	-0.03724	0.760883	6835045	6.205922	6.435791	6.32172	6.564276	6.530048	6.667226	6.397313	6.464629	6.213226	6.417083	6.176912	6.617594	Ncald	NM_134094
6835063	5160	7.376796	7.569546	7.500191	7.720829	-0.34403	0.025752	-0.15128	0.263915	-0.22064	0.117815	0.069355	0.596797	-0.19275	0.164341	-0.1234	0.355781	6835063	7.203043	7.537687	7.389659	7.774194	7.477799	7.456645	7.447766	7.610997	7.441811	7.581285	7.682634	7.898567	Rrm2b	NM_199476
6835065	5161	9.092613	9.129554	9.032416	9.020464	0.072148	0.339175	0.10909	0.16288	0.011952	0.870467	0.097138	0.208352	-0.03694	0.616852	0.060197	0.421055	6835065	9.223277	9.028328	9.026233	9.038348	9.205029	9.145286	9.094977	9.04756	8.954711	8.937467	9.067998	9.055928	Ubr5	NM_001081359
6835089	5162	7.615471	7.79093	7.609715	7.610135	0.005336	0.980086	0.180795	0.408376	-0.00042	0.998432	0.181215	0.407336	-0.17546	0.421759	0.005756	0.978519	6835089	7.707215	7.672661	7.466537	7.447922	8.268517	7.65635	7.619976	7.569933	7.639235	7.334725	7.722967	7.772712	Klf10	NM_013692
6835104	5163	9.450422	9.451987	9.499295	9.146789	0.303633	0.098984	0.305198	0.097531	0.352506	0.062149	-0.04731	0.778627	-0.00157	0.992559	-0.04887	0.771541	6835104	9.575263	9.466077	9.309926	9.322081	9.672668	9.361213	9.797644	9.252447	9.447794	8.954899	9.257634	9.227835	Azin1	NM_001102458
6835149	5164	6.911369	7.04548	7.001854	6.943692	-0.03232	0.743	0.101788	0.316265	0.058161	0.558271	0.043627	0.659017	-0.13411	0.196654	-0.09048	0.369791	6835149	6.845093	6.957295	6.931719	6.97427	7.077854	7.084317	7.062652	7.091363	6.851546	6.794328	6.903285	7.133464	Slc25a32	NM_172402
6835222	5166	7.076428	7.115801	7.127321	7.147407	-0.07098	0.665544	-0.03161	0.846618	-0.02009	0.902091	-0.01152	0.943732	-0.03937	0.809703	-0.05089	0.755899	6835222	7.197669	7.139563	6.892051	7.060423	7.440586	6.846394	7.311276	7.087425	6.983262	7.095129	7.117999	7.229093	Lrp12	NM_172814
6835373	5170	8.96016	8.965203	9.099348	9.165668	-0.20551	0.317416	-0.20047	0.328697	-0.06632	0.739637	-0.13414	0.506135	-0.00504	0.979767	-0.13919	0.490755	6835373	9.038504	9.031936	8.81004	8.625511	9.211833	9.058264	9.343295	8.854746	9.100002	8.950878	9.134301	9.411826	Eif3e	NM_008388
6835415	5172	7.56048	7.684504	7.887183	7.445704	0.114775	0.618494	0.2388	0.312594	0.441478	0.081475	-0.20268	0.387102	-0.12402	0.590997	-0.3267	0.178608	6835415	7.561001	7.518948	7.60149	7.4784	7.964368	7.610745	8.132142	7.91046	7.618946	7.492408	7.824711	7.019994	Nudcd1	NM_026149
6835428	5173	6.444437	6.548785	6.463691	6.359087	0.085349	0.203281	0.189698	0.015127	0.104604	0.127911	0.085094	0.2045	-0.10435	0.128715	-0.01925	0.762595	6835428	6.392243	6.55006	6.391007	6.536368	6.63177	6.478218	6.491333	6.374637	6.525103	6.393008	6.306174	6.37808	5730410E	NM_178765
6835429	5174	6.231171	6.348888	6.304505	6.284747	-0.05358	0.746113	0.064141	0.698731	0.019757	0.90468	0.044383	0.788313	-0.11772	0.482498	-0.07333	0.658602	6835429	6.38682	6.068799	6.237893	6.113134	6.258306	6.675224	6.371277	6.458	6.084237	6.215271	6.288517	6.350454	5730410E	NM_176998
6835579	5176	7.907748	7.934935	7.602098	7.224174	0.683574	0.077056	0.710761	0.067978	0.377924	0.29465	0.332837	0.352268	-0.02719	0.937686	0.30565	0.390937	6835579	8.394475	7.911905	7.416864	7.761143	8.292175	7.751487	7.841497	7.190858	7.773938	7.135985	7.729754	6.806783	Trps1	NM_032000
6835634	5177	10.56676	10.70141	10.67191	10.65608	-0.08932	0.461972	0.045327	0.705247	0.015827	0.894494	0.0295	0.80503	-0.13465	0.277706	-0.10515	0.389666	6835634	10.40109	10.61406	10.68513	10.55515	10.83903	10.71004	10.85178	10.62073	10.54321	10.62708	10.56221	10.77895	Eif3h	NM_080635
6835640	5178	9.599824	9.806139	9.528305	9.564396	0.035428	0.573968	0.241743	0.003954	-0.03609	0.566969	0.277834	0.001764	-0.20632	0.00918	0.071519	0.270706	6835640	9.537629	9.647154	9.614688	9.708211	9.88253	9.827677	9.551869	9.582797	9.450249	9.507027	9.533614	9.652547	Rad21	NM_009009
6835692	5179	9.561255	9.393361	9.582295	9.442792	0.118464	0.561228	-0.04943	0.806711	0.139503	0.495656	-0.18893	0.362007	0.167894	0.415312	-0.02104	0.916924	6835692	9.468504	9.625474	9.589788	9.140404	9.783744	9.255936	9.86047	9.259716	9.626698	9.38601	9.574412	9.367953	Ext1	NM_010162
6835706	5180	7.797785	6.752766	7.256044	8.369264	-0.57148	0.140157	-1.6165	0.001684	-1.11322	0.012807	-0.50328	0.187255	1.045019	0.017218	0.541742	0.15919	6835706	7.764118	8.219815	7.409423	6.901285	6.795918	6.561096	7.347139	7.831141	6.589851	8.537458	8.635546	7.934789	Samd12	NM_177225
6835728	5181	6.355358	6.286792	6.242859	6.233221	0.122137	0.59338	0.053571	0.813452	0.009638	0.966076	0.043933	0.846461	0.068566	0.762898	0.112499	0.622365	6835728	6.631606	6.548619	5.885849	6.224535	6.31978	6.316061	6.611404	5.934745	6.182429	6.234185	6.280755	6.184724	Tnfrsf11	NM_008764
6835759	5182	8.116229	7.937945	7.970546	8.093233	0.022996	0.947776	-0.15529	0.660247	-0.12269	0.727753	-0.0326	0.926025	0.178284	0.614501	0.145683	0.67983	6835759	8.607557	8.082565	7.658564	7.674449	8.174945	7.964441	8.278758	7.501865	8.131015	7.725205	8.642905	7.911589	Enpp2	NM_015744
6835806	5184	6.937722	7.116621	7.004755	6.972153	-0.03443	0.78563	0.144468	0.271807	0.032602	0.796711	0.111866	0.387486	-0.1789	0.182009	-0.06703	0.598888	6835806	6.895886	6.854113	7.063167	7.055973	7.197536	7.096354	7.123042	7.041403	6.84982	6.714774	7.046165	7.15552	Mrpl13	NM_026759
6835856	5185	6.727614	6.279329	6.647051	5.115947	1.611667	0.026821	1.163382	0.086539	1.531103	0.033086	-0.36772	0.554119	0.448285	0.473198	0.080564	0.89574	6835856	7.293886	6.753431	6.135526	5.483266	7.431767	5.922954	6.927162	5.786611	7.227379	4.860895	5.591756	4.895191	Has2	NM_008216
6835918	5188	8.809172	8.829134	8.976689	8.912566	-0.10339	0.220355	-0.08343	0.314662	0.064124	0.43354	-0.14756	0.094361	-0.01996	0.803901	-0.16752	0.063381	6835918	8.862028	8.805838	8.759649	8.762248	8.862165	8.86299	9.067908	9.021612	8.840548	8.868713	9.054605	8.814379	Derl1	NM_024207
6835932	5189	6.109938	6.171066	6.255253	5.940613	0.169325	0.292299	0.230453	0.163517	0.31464	0.069508	-0.08419	0.590504	-0.06113	0.69471	-0.14532	0.361662	6835932	6.237117	6.145268	5.947429	6.137077	6.358602	6.017519	6.389924	6.265605	6.11023	5.816865	6.231362	5.773612	9130401M	BC050895
6835933	5190	7.566586	7.623385	7.559904	7.664277	-0.09769	0.642601	-0.04089	0.845086	-0.10437	0.620376	0.063481	0.762051	-0.0568	0.786331	0.006682	0.974497	6835933	7.55458	7.738005	7.407174	7.60604	7.622862	7.641252	7.84217	7.248648	7.588894	7.253845	7.928463	7.810523	Zhx1	NM_009572
6835934	5191	6.297184	6.41722	6.059528	5.596856	0.700328	0.044922	0.820364	0.023865	0.462672	0.155335	0.357692	0.259789	-0.12004	0.694676	0.237657	0.443634	6835934	6.215391	6.198633	6.477529	6.079146	6.809801	6.362712	6.483869	5.48925	6.205464	5.517708	5.942588	5.330272	Atad2	NM_027435
6835938	5192	7.670994	7.673143	7.648509	7.389278	0.281715	0.213361	0.283865	0.21022	0.259231	0.248688	0.024634	0.908807	-0.00215	0.992021	0.022484	0.916729	6835938	7.54029	7.577943	7.894748	7.941229	7.678086	7.400115	7.952233	7.400972	7.592323	7.139412	7.358557	7.669866	Fbxo32	NM_026346
6835945	5193	5.761516	5.64403	5.709418	5.603635	0.157882	0.753346	0.040395	0.935726	0.105784	0.832952	-0.06539	0.896176	0.117487	0.814852	0.052098	0.917176	6835945	5.175989	5.244402	6.864158	6.228271	5.395813	5.308005	6.135343	5.526067	5.466845	5.25332	5.896942	5.660642	Klhl38	NM_177755
6835972	5195	8.73311	8.681619	8.653949	8.733387	-0.00028	0.998914	-0.05177	0.799688	-0.07944	0.697811	0.02767	0.891949	0.05149	0.800733	0.079161	0.698803	6835972	8.528019	8.550367	9.120943	8.720182	8.684824	8.639852	8.697025	8.676323	8.588499	8.806857	9.030877	8.362426	Tmem65	NM_175212
6835973	5196	6.563231	6.357518	6.442176	6.763054	-0.19982	0.038255	-0.40554	0.001016	-0.32088	0.00407	-0.08466	0.324546	0.205713	0.034142	0.121055	0.171763	6835973	6.615236	6.571476	6.50298	6.405556	6.172596	6.494402	6.431511	6.530591	6.364425	6.741809	6.744005	6.803347	Tmem65	NM_175212
6835981	5197	7.005852	7.142793	6.960383	6.941497	0.064355	0.584245	0.201296	0.112383	0.018885	0.87128	0.182411	0.144758	-0.13694	0.259664	0.045469	0.697632	6835981	7.003574	6.998861	7.015121	7.112192	7.208663	7.107525	7.166954	6.78788	6.926314	6.785283	6.885357	7.153852	Tatdn1	NM_175151
6835984	5198	9.035503	8.936042	9.074113	8.583338	0.452165	0.008918	0.352704	0.028027	0.490775	0.005822	-0.13807	0.325173	0.099461	0.471837	-0.03861	0.776889	6835984	8.96519	9.079156	9.062163	8.918727	9.07083	8.818569	8.962412	9.18137	9.078557	8.319526	8.856629	8.57386	Mtss1	NM_001146180
6836006	5199	8.765181	8.784107	8.783223	8.515992	0.249189	0.030072	0.268114	0.022076	0.267231	0.022395	0.000883	0.992784	-0.01893	0.846536	-0.01804	0.853599	6836006	8.741443	8.816034	8.738067	8.726009	8.852895	8.773416	8.96869	8.608994	8.771986	8.373477	8.593972	8.580528	E430025E	BC067035
6836079	5200	7.605031	7.773199	7.709558	7.588328	0.016703	0.914007	0.18487	0.252387	0.12123	0.441963	0.06364	0.682292	-0.16817	0.294371	-0.10453	0.50528	6836079	7.659206	7.453769	7.702118	7.707913	8.027594	7.584089	7.826803	7.876691	7.425181	7.544706	7.56461	7.655669	---	ENSMUST000001
6836149	5201	6.180385	6.192263	6.141043	6.42139	-0.241	0.030704	-0.22913	0.037533	-0.28035	0.015908	0.05122	0.593033	-0.01188	0.900489	0.039343	0.680277	6836149	6.249626	6.138864	6.152666	6.222133	6.138315	6.216341	6.270381	6.059141	6.093606	6.299764	6.337152	6.627255	H2afy3	NR_003523
6836231	5203	8.390662	8.381535	8.516811	8.458049	-0.06739	0.662199	-0.07651	0.620485	0.058762	0.702805	-0.13528	0.38917	0.009127	0.952522	-0.12615	0.420525	6836231	8.555023	8.327383	8.28958	8.338285	8.53479	8.271529	8.861125	8.391899	8.297409	8.434683	8.509005	8.430458	Fam49b	NM_144846
6836237	5204	9.682495	9.501375	9.494782	9.40459	0.277905	0.009752	0.096785	0.273982	0.090193	0.305604	0.006592	0.938205	0.18112	0.059192	0.187712	0.052246	6836237	9.613484	9.685923	9.748077	9.440285	9.646024	9.417815	9.464813	9.49412	9.525414	9.345651	9.563928	9.30419	Asap1	NM_010026
6836260	5205	7.290974	7.312047	7.071463	6.466112	0.824862	0.028509	0.845935	0.025655	0.605351	0.086053	0.240584	0.459081	-0.02107	0.947357	0.219511	0.498079	6836260	7.369524	7.317824	7.185574	7.241602	7.726633	6.967906	7.466507	6.489947	7.257934	6.68361	6.700099	6.014626	Adcy8	NM_009623
6836325	5209	6.005761	5.644947	5.891576	5.416305	0.589456	0.032703	0.228642	0.346554	0.475271	0.071243	-0.24663	0.312143	0.360814	0.15317	0.114185	0.630908	6836325	5.926256	5.914808	6.17622	5.486016	5.474569	5.974257	6.117292	5.539708	6.017729	5.168441	5.802924	5.27755	Tmem71	NM_172514
6836341	5210	9.481831	9.174008	9.158072	9.540201	-0.05837	0.846011	-0.36619	0.243683	-0.38213	0.225495	0.015936	0.957666	0.307822	0.320985	0.323758	0.298154	6836341	9.828869	9.386931	9.229692	9.289956	8.985025	9.247044	8.699449	9.657313	9.117455	9.765585	9.767374	9.087645	Sla	NM_001029841
6836358	5211	11.46049	11.35427	11.28878	10.95205	0.508437	0.120944	0.40222	0.207119	0.336723	0.283709	0.065497	0.828738	0.106217	0.726387	0.171713	0.574041	6836358	11.4575	11.23536	11.68861	11.16728	11.58051	11.31503	11.62391	10.87167	11.37075	11.42714	11.03733	10.39169	Ndrg1	NM_008681
6836366	5212	11.38296	11.17785	11.09331	11.10516	0.2778	0.325088	0.072693	0.790772	-0.01185	0.965416	0.084547	0.75784	0.205107	0.461152	0.289653	0.306151	6836366	11.24045	11.3445	11.56393	10.91986	11.30758	11.30612	11.22791	10.69009	11.36192	11.33183	11.41504	10.56861	St3gal1	NM_009177
6836405	5213	6.929238	6.872487	6.833063	6.935015	-0.00578	0.963915	-0.06253	0.627018	-0.10195	0.433934	0.039424	0.758226	0.056751	0.658754	0.096175	0.459457	6836405	6.876662	6.954247	6.956805	6.637853	7.11315	6.866458	6.652048	7.015557	6.831585	6.916309	6.94906	6.939675	Zfat	NM_001145888
6836584	5216	8.116089	8.090404	7.949645	8.049557	0.066532	0.224103	0.040846	0.441935	-0.09991	0.083212	0.140758	0.023643	0.025686	0.624693	0.166444	0.010918	6836584	8.183962	8.020871	8.143435	8.128056	8.107559	8.035596	7.931621	7.987514	7.929801	8.004751	8.015865	8.128056	Trappc9	NM_180662
6836596	5217	9.044337	9.024356	8.899084	9.086121	-0.04178	0.704675	-0.06177	0.5774	-0.18704	0.116693	0.125272	0.272698	0.019981	0.855653	0.145253	0.209179	6836596	8.97613	8.965605	9.191276	8.983269	9.116579	8.973219	8.742792	9.133037	8.821422	9.033274	9.136046	9.089043	Eif2c2	NM_153178
6836602	5218	8.327916	8.303159	8.408829	8.326535	0.001381	0.987581	-0.02338	0.792644	0.082294	0.366616	-0.10567	0.254077	0.024757	0.780757	-0.08091	0.374301	6836602	8.312878	8.338953	8.331916	8.309198	8.438228	8.162051	8.511939	8.429832	8.284716	8.219133	8.436851	8.32362	Ptk2	NM_007982
6836691	5221	6.334668	6.428194	6.480904	6.478168	-0.1435	0.211311	-0.04997	0.648736	0.002736	0.979965	-0.05271	0.631159	-0.09353	0.401704	-0.14624	0.203565	6836691	6.311138	6.475059	6.217806	6.305803	6.384377	6.594401	6.512072	6.557503	6.373137	6.353929	6.457199	6.623375	Arc	NM_018790
6836694	5223	6.357301	6.360302	6.166956	6.461446	-0.10414	0.726739	-0.10114	0.734253	-0.29449	0.335985	0.193346	0.520498	-0.003	0.991934	0.190345	0.526822	6836694	6.315991	6.176589	6.579324	6.332829	6.424507	6.32357	6.028501	6.413473	6.058894	6.329381	7.154228	5.900729	Slurp1	NM_020519
6836699	5224	7.943133	7.671335	7.5854	7.114199	0.828934	0.029907	0.557136	0.114414	0.471202	0.172433	0.085934	0.791583	0.271798	0.412611	0.357733	0.288248	6836699	7.943472	8.031731	7.854196	7.874669	7.772356	7.366979	7.774076	7.195464	7.786661	6.820743	7.839534	6.682319	Lynx1	NM_011838
6836700	5225	6.727437	7.118197	6.499526	8.600034	-1.8726	0.108426	-1.48184	0.190659	-2.10051	0.077256	0.618671	0.567066	-0.39076	0.715962	0.227911	0.831458	6836700	6.63298	6.798338	6.750992	6.404996	8.699273	6.250321	6.572047	6.404865	6.521666	8.362093	10.84205	6.59596	Ly6d	NM_010742
6836708	5227	6.086533	5.972244	5.995642	5.975294	0.111239	0.421448	-0.00305	0.982032	0.020348	0.880668	-0.0234	0.862976	0.114289	0.409366	0.090891	0.508332	6836708	5.986712	6.017735	6.255152	6.187038	5.760051	5.969643	6.123682	5.971976	5.891269	6.146191	5.91075	5.868942	Ly6k	NM_029627
6836728	5231	9.720545	9.510344	9.484028	9.39617	0.324375	0.334154	0.114174	0.726919	0.087858	0.787797	0.026316	0.935602	0.210201	0.524162	0.236517	0.475101	6836728	9.868982	9.815243	9.477411	9.527337	10.02029	8.983405	9.92734	9.154181	9.370564	9.64321	9.553369	8.991932	Ly6a //	NM_010738
6836767	5234	6.782587	6.805245	6.686978	6.810763	-0.02818	0.770841	-0.00552	0.954387	-0.12379	0.222133	0.118267	0.241535	-0.02266	0.814628	0.095609	0.336467	6836767	6.770874	6.765114	6.811772	6.609998	6.842587	6.96315	6.817478	6.659347	6.584108	6.891672	6.748177	6.792441	Top1mt	NM_028404
6836771	5235	6.384854	6.490309	6.307991	6.525563	-0.14071	0.431202	-0.03525	0.840673	-0.21757	0.235854	0.182318	0.314148	-0.10546	0.551744	0.076863	0.662732	6836771	6.37749	6.470911	6.306161	6.495556	6.54349	6.431881	6.540032	6.52263	5.861312	6.645418	6.416131	6.51514	Mafa	NM_194350

6836778	5236	7.449686	7.418091	7.433676	7.332035	0.117651	0.400361	0.086056	0.534118	0.101641	0.464937	-0.01558	0.909244	0.031595	0.817476	0.01601	0.906778	6836778	7.175819	7.531754	7.641485	7.339391	7.49365	7.421232	7.594155	7.274785	7.432087	7.329137	7.454122	7.212845	Naprt1	NM_172607
6836779	5237	7.070753	7.01795	7.107234	7.23313	-0.16238	0.224778	-0.21518	0.119437	-0.1259	0.337591	-0.08928	0.490066	0.052803	0.680083	-0.03648	0.775089	6836779	7.011575	7.064784	7.1359	6.819007	7.059046	7.175798	7.212257	6.970325	7.139121	7.449528	7.062398	7.187463	Eef1d	NM_029663
6836782	5238	6.561067	6.641392	6.732088	6.798326	-0.23726	0.101874	-0.15693	0.256492	-0.06624	0.619962	-0.0907	0.500097	-0.08032	0.549092	-0.17102	0.219657	6836782	6.381062	6.669414	6.632725	6.721566	6.66216	6.540449	6.70837	6.982656	6.505239	6.819711	6.697915	6.877353	Pycrl	NM_025412
6836784	5239	7.062411	7.244572	7.084157	6.984981	0.077429	0.411692	0.25959	0.019787	0.099176	0.299548	0.160415	0.110528	-0.18216	0.075976	-0.02175	0.81395	6836784	7.105854	7.024004	7.057374	7.172445	7.44891	7.11236	7.18789	7.055177	7.009404	7.033786	6.900004	7.021154	Tsta3	NM_031201
6836791	5240	6.901484	6.943655	6.859697	6.957405	-0.05592	0.21683	-0.01375	0.750115	-0.09771	0.047225	0.083957	0.078921	-0.04217	0.341587	0.041787	0.345751	6836791	6.915966	6.9344	6.854086	6.987105	6.899142	6.944717	6.925935	6.797691	6.855466	6.907502	6.955151	7.009562	2410075B	BC047213
6836792	5241	7.740113	7.479695	7.560339	7.376029	0.364083	0.032179	0.103266	0.483768	0.18431	0.226408	-0.08104	0.580303	0.260817	0.100787	0.179773	0.237011	6836792	7.772789	7.647227	7.800322	7.560494	7.594553	7.282839	7.444223	7.76367	7.473125	7.309272	7.629021	7.189795	Fam83h	NM_134087
6836794	5242	8.341375	8.364497	8.321311	8.185483	0.155892	0.225729	0.179014	0.170173	0.135828	0.285839	0.043186	0.725567	-0.02312	0.850498	0.020064	0.87004	6836794	8.412753	8.249406	8.361967	8.393415	8.526585	8.173491	8.254167	8.556176	8.15359	8.134677	8.21475	8.207022	Scrib	NM_134089
6836795	5243	9.354185	9.360295	9.368508	9.320764	0.033421	0.696196	0.03953	0.644851	0.047744	0.578936	-0.00821	0.923193	-0.00611	0.942818	-0.01432	0.866563	6836795	9.303297	9.275396	9.483862	9.304649	9.522519	9.253716	9.443213	9.328123	9.334188	9.252566	9.358252	9.351475	Puf60	NM_133691
6836797	5244	10.57379	10.59131	10.21256	10.59909	-0.0253	0.854342	-0.00778	0.95491	-0.38653	0.019979	0.378746	0.021853	-0.01751	0.898806	0.361233	0.02676	6836797	10.37463	10.65253	10.69422	10.54851	10.74785	10.47756	9.951121	10.30271	10.38385	10.59617	10.66431	10.53679	Nrbp2	NM_144847
6836805	5246	8.746541	8.718117	8.50684	8.437814	0.308726	0.01161	0.280302	0.018271	0.069026	0.487484	0.211277	0.056499	0.028424	0.772043	0.239701	0.035398	6836805	8.767684	8.666736	8.805202	8.778361	8.726369	8.64962	8.655674	8.466894	8.397952	8.273954	8.601633	8.437856	Plec1	NM_201389
6836806	5247	7.79778	7.601503	7.585025	7.438439	0.359341	0.00437	0.163064	0.112543	0.146586	0.147767	0.016478	0.861543	0.196277	0.064234	0.212755	0.048501	6836806	7.847946	7.734467	7.810927	7.632609	7.746846	7.425055	7.656178	7.470112	7.628786	7.370519	7.554292	7.390506	Plec1	BC024074
6836810	5248	8.759227	8.769937	8.757138	8.991138	-0.23191	0.011749	-0.2212	0.014725	-0.234	0.011246	0.012799	0.862221	-0.01071	0.884508	0.00209	0.977374	6836810	8.612848	8.774939	8.889895	8.861775	8.702039	8.745997	8.81926	8.748557	8.703596	8.957508	9.021046	8.99486	Oplah	NM_153122
6836815	5249	8.644803	8.68539	8.696999	8.549345	0.095459	0.292305	0.136045	0.146823	0.147654	0.119383	-0.01161	0.894345	-0.04059	0.644567	-0.0522	0.554778	6836815	8.599654	8.63717	8.697586	8.626486	8.881681	8.548002	8.80201	8.644567	8.64442	8.527524	8.521272	8.599238	Sharpin	NM_025340
6836827	5250	7.803461	7.803109	7.979625	7.7686	0.034861	0.738459	0.034509	0.740991	0.211025	0.069712	-0.17652	0.118129	0.000353	0.997295	-0.17616	0.118759	6836827	7.850252	7.72296	7.837172	7.781587	7.929605	7.698134	8.097481	8.020777	7.820617	7.606093	7.895884	7.803823	Bop1	NM_013481
6836829	5251	6.843532	7.019526	6.912405	6.997194	-0.15366	0.292134	0.022332	0.873881	-0.08479	0.551099	0.107121	0.45444	-0.17599	0.232565	-0.06887	0.626874	6836829	6.822739	6.764231	6.943627	6.998893	7.164626	6.895059	6.954527	7.023397	6.759292	6.80032	6.904032	7.287229	Dgat1	NM_010046
6836833	5253	6.993298	7.144925	7.158317	7.080138	-0.08684	0.433697	0.064787	0.555695	0.078179	0.47929	-0.01339	0.90199	-0.15163	0.188065	-0.16502	0.155924	6836833	6.979611	6.948473	7.051809	7.088493	7.231825	7.114456	7.295479	7.295877	6.883595	7.061906	7.055963	7.122544	Fbxl6	NM_013909
6836838	5254	7.898873	7.908161	7.918377	7.863119	0.035754	0.622343	0.045042	0.536816	0.055257	0.451415	-0.01022	0.887266	-0.00929	0.897432	-0.0195	0.787018	6836838	7.873293	7.877272	7.946054	7.937365	8.018095	7.769023	7.989608	7.864364	7.901158	7.769942	7.882193	7.937223	Cpsf1	NM_053193
6836839	5255	7.264475	7.087567	7.480064	7.32994	-0.06546	0.817072	-0.24237	0.401938	0.150124	0.598512	-0.3925	0.189681	0.176908	0.536359	-0.21559	0.453818	6836839	7.330506	6.935776	7.527143	7.144151	7.501304	6.617247	7.881881	7.276204	7.282106	7.23524	7.193894	7.560686	Slc39a4	NM_028064
6836840	5256	10.61485	10.72965	10.72471	10.5843	0.03055	0.785831	0.14535	0.217991	0.140413	0.232563	0.004937	0.964894	-0.1148	0.321818	-0.10986	0.341804	6836840	10.58129	10.5266	10.73665	10.67737	10.87517	10.6364	10.92052	10.69038	10.56323	10.57961	10.48704	10.68624	Vps28	NM_025842
6836842	5257	6.329384	6.329705	6.238939	6.223051	0.106334	0.420758	0.106654	0.419416	0.015889	0.902225	0.090766	0.489474	-0.00032	0.998021	0.090445	0.490963	6836842	6.295953	6.182165	6.510035	6.229878	6.591798	6.167439	6.282686	6.178903	6.255229	6.198371	6.132204	6.338577	Nfkbil2	NM_183091
6836843	5258	8.076497	8.150104	8.156805	8.210799	-0.1343	0.194663	-0.06069	0.540259	-0.05399	0.58493	-0.0067	0.945433	-0.07361	0.4602	-0.08031	0.421928	6836843	8.047357	8.111441	8.070694	8.155967	8.105702	8.188643	8.22331	8.290134	7.95697	8.145546	8.113203	8.373647	Cyhr1	NM_019396
6836851	5261	5.746805	5.847968	6.028292	5.950686	-0.20388	0.015097	-0.10272	0.159221	0.077606	0.274648	-0.18032	0.026051	-0.10116	0.164867	-0.28149	0.002786	6836851	5.795844	5.703309	5.741262	5.873343	5.882574	5.787987	6.082069	6.120235	5.882571	5.8705	5.974035	6.007523	Lrrc24	NM_198119
6836852	5262	6.886116	6.833473	6.974106	7.445698	-0.55958	0.010312	-0.61222	0.006509	-0.47159	0.022831	-0.14063	0.42632	0.052643	0.761762	-0.08799	0.614246	6836852	6.910701	6.93661	6.811037	7.008985	6.78174	6.709694	6.978414	7.021836	6.922067	7.211936	7.251661	7.873496	C030006K	NM_176828
6836854	5263	7.28191	7.318824	7.408954	7.193124	0.088786	0.566001	0.1257	0.421362	0.215829	0.183722	-0.09013	0.560265	-0.03691	0.80973	-0.12704	0.416623	6836854	7.269571	7.25756	7.3186	7.307001	7.469332	7.180139	7.170234	7.722404	7.334223	7.261008	7.320654	6.997711	D15Wsu16	BC060637
6836874	5264	6.852772	6.940241	7.007877	7.014473	-0.1617	0.083324	-0.07423	0.390418	-0.0066	0.937675	-0.06764	0.43205	-0.08747	0.315889	-0.1551	0.094377	6836874	6.735297	6.876145	6.946875	6.829661	6.939684	7.051377	7.02398	7.086608	6.913043	6.961225	6.961343	7.120852	Zfp251	NM_001007568
6836875	5265	5.916681	5.936261	6.007199	5.933008	-0.01633	0.854575	0.003253	0.970839	0.074192	0.414721	-0.07094	0.434638	-0.01958	0.826108	-0.09052	0.324624	6836875	5.856662	6.057018	5.836362	5.897677	6.039091	5.872014	5.967648	6.134722	5.919228	5.824715	5.99654	5.977768	Zfp647	NM_172817
6836887	5266	7.845867	7.858518	7.759737	7.390803	0.455064	0.670186	0.467715	0.661686	0.368934	0.729352	0.098781	0.925922	-0.01265	0.990497	0.08613	0.935383	6836887	6.513355	7.336654	9.687592	9.261009	7.906956	6.407589	8.57419	7.779328	6.925694	6.444819	8.371804	7.355787	Mb	NM_013593
6836888	5267	8.260376	8.345863	8.214298	8.239658	0.020718	0.857	0.106205	0.368017	-0.02536	0.825515	0.131566	0.27122	-0.08549	0.464606	0.046078	0.689821	6836888	8.259945	8.400702	8.12048	8.333003	8.300843	8.403744	8.139095	8.241526	8.262272	7.990095	8.394416	8.334463	Rbm9	NM_053104
6836949	5269	6.55114	6.518803	6.436873	6.449629	0.101511	0.658361	0.069174	0.76238	-0.01276	0.955406	0.081931	0.72058	0.032337	0.887337	0.114267	0.61926	6836949	6.761281	6.220604	6.671535	6.513828	6.528405	6.514177	6.360132	6.049206	6.90128	6.312483	6.614358	6.422046	Apol9b	NM_173743
6836954	5270	8.389667	8.143024	8.969707	8.152784	0.236883	0.477803	-0.00976	0.976277	0.816923	0.033235	-0.82668	0.031687	0.246643	0.460465	-0.58004	0.105717	6836954	8.47238	8.426198	8.270424	8.480747	8.559274	7.389052	8.930696	8.623292	9.355133	8.107492	8.353074	7.997786	Apol7b	NM_001024848
6836973	5273	10.87207	10.8943	10.65059	10.43839	0.433683	0.00138	0.455907	0.001013	0.212203	0.047285	0.243703	0.027522	-0.02222	0.81244	0.22148	0.040303	6836973	10.92776	10.82389	10.86457	10.86746	11.00056	10.81487	10.82897	10.54508	10.57773	10.31151	10.46771	10.53595	Myh9	NM_022410
6836979	5274	8.936338	8.704798	8.873689	9.021653	-0.08531	0.50475	-0.31685	0.031933	-0.14796	0.260395	-0.16889	0.204195	0.23154	0.094654	0.062649	0.621938	6836979	9.029811	8.752698	9.026505	8.729937	8.807747	8.576711	9.082938	8.865586	8.672544	8.964674	8.973694	9.12659	Txn2	NM_019913
6836981	5275	6.87483	6.962257	6.516064	7.107035	-0.2322	0.073374	-0.14478	0.234972	-0.59097	0.000781	0.446193	0.004188	-0.08743	0.460317	0.358766	0.012947	6836981	7.009639	6.873804	6.741047	6.943601	7.129649	6.813521	6.563993	6.504478	6.479721	7.293799	6.943465	7.08384	Foxred2	NM_001017983
6836983	5276	9.572473	9.604457	9.570458	9.510264	0.062209	0.36625	0.094193	0.185074	0.060194	0.381192	0.033999	0.614868	-0.03198	0.635666	0.002015	0.976013	6836983	9.514261	9.555686	9.647473	9.483294	9.746629	9.583449	9.599175	9.569651	9.542549	9.479313	9.564207	9.487273	Eif3d	NM_018749
6836999	5278	7.09829	6.923641	7.2693	7.224392	-0.1261	0.433781	-0.30075	0.084947	0.044909	0.776628	-0.34566	0.053818	0.174649	0.286746	-0.17101	0.296197	6836999	7.052178	7.08491	7.157782	7.101232	7.027588	6.642102	7.328638	7.343553	7.13571	6.933809	7.368307	7.371059	Rabl4	NM_025931
6837006	5279	7.43033	7.303678	7.112355	6.623629	0.806701	0.581987	0.680049	0.641654	0.488726	0.737168	0.191323	0.895147	0.126652	0.930455	0.317975	0.826792	6837006	5.373342	6.706127	10.21152	8.460426	7.565392	5.885216	8.387143	6.921551	6.02837	5.486038	5.958269	8.426579	Pvalb	NM_013645
6837008	5280	7.634236	7.082983	6.95916	5.582908	2.051329	0.011049	1.500076	0.042863	1.376253	0.058428	0.123823	0.847611	0.551253	0.402644	0.675076	0.310714	6837008	8.675716	7.469429	6.757564	7.048373	7.658513	6.542064	7.337407	6.416334	7.12374	5.862703	6.332418	4.553602	Csf2rb2	NM_007781
6837013	5281	7.43123	7.78629	7.718933	7.875345	-0.44412	0.13217	-0.08905	0.74539	-0.15641	0.571197	0.067357	0.805705	-0.35506	0.216962	-0.2877	0.309099	6837013	7.307872	7.628685	7.357132	7.94589	7.789439	7.623542	7.736762	8.052781	7.367256	7.586088	7.590142	8.449806	Tst	NM_009437
6837018	5282	6.116666	6.133855	6.095018	6.101617	0.015049	0.820737	0.032238	0.629448	-0.0066	0.92074	0.038837	0.562385	-0.01719	0.795889	0.021649	0.744895	6837018	6.133031	6.099965	6.117003	6.234517	6.145399	6.021648	6.073299	6.193044	5.99871	6.122767	6.060938	6.121146	Tmprss6	NM_027902
6837023	5284	6.226142	6.400031	6.355487	6.555722	-0.32958	0.115573	-0.15569	0.428614	-0.20024	0.314871	0.044544	0.817462	-0.17389	0.378996	-0.12935	0.508127	6837023	6.498829	6.213024	5.966572	6.648891	6.460771	6.09043	6.629574	6.198239	6.238648	6.57443	6.523542	6.569194	Sstr3	NM_009218
6837028	5285	8.484463	8.395539	8.465505	7.401446	1.083017	0.012233	0.994093	0.01826	1.064059	0.013315	-0.06997	0.840393	0.088924	0.798146	0.018957	0.95643	6837028	8.827226	8.320273	8.305889	8.300717	8.908506	7.977394	8.893248	8.016817	8.486451	7.254841	7.872835	7.076662	Rac2	NM_009008
6837045	5287	7.202838	7.157736	7.09938	7.054382	0.148456	0.320109	0.103354	0.481631	0.044998	0.75623	0.058356	0.68788	0.045102	0.755688	0.103458	0.481204	6837045	7.150441	7.342092	7.115981	7.157434	7.22982	7.085955	7.074975	7.163744	7.059421	6.700396	7.208294	7.254457	Mfng	NM_008595
6837046	5288	7.331227	7.436613	7.439446	7.495256	-0.16403	0.227534	-0.05864	0.652771	-0.05581	0.668325	-0.00283	0.982544	-0.10539	0.42545	-0.10822	0.41363	6837046	7.264975	7.333405	7.395301	7.52645	7.362178	7.421211	7.410632	7.669139	7.238567	7.373205	7.397575	7.714989	Card10	NM_130859
6837081	5290	7.510852	7.645668	7.519413	7.594215	-0.08336	0.55611	0.051453	0.714463	-0.0748	0.596569	0.126255	0.379436	-0.13482	0.34962	-0.00856	0.95125	6837081	7.426501	7.50304	7.603014	7.710631	7.692112	7.534261	7.556045	7.796279	7.205914	7.517925	7.60445	7.660271	Ankrd54	NM_144849
6837088	5291	8.333521	8.851717	8.221073	8.558169	-0.22465	0.190299	0.293548	0.098416	-0.3371	0.06404	0.630643	0.003856	-0.5182	0.010843	0.112447	0.49418	6837088	8.269066	8.360661	8.370835	8.899646	8.7882	8.867304	7.914441	8.541134	8.207645	8.797837	8.443843	8.432826	Micall1	NM_177461
6837090	5292	6.259867	6.257615	6.287694	6.46377	-0.2039	0.032085	-0.20616	0.03069	-0.17608	0.055684	-0.03008	0.712304	0.002252	0.977876	-0.02783	0.732822	6837090	6.376958	6.184997	6.217645	6.24119	6.212306	6.319348	6.390325	6.271967	6.200789	6.538587	6.32541	6.527314	Sox10	NM_011437
6837095	5294	6.421529	6.535425	6.4899	6.551309	-0.12978	0.266908	-0.01588	0.88749	-0.06141	0.587768	0.045526	0.686501	-0.1139	0.32556	-0.06837	0.547076	6837095	6.528622	6.247487	6.488479	6.5624	6.452799	6.591077	6.574262	6.488587	6.40685	6.655294	6.333803	6.66483	Baiap2l2	NM_177580
6837096	5295	7.212209	7.389769	7.104295	7.218039	-0.00583	0.939776	0.17173	0.050749	-0.11374	0.16674	0.285474	0.005108	-0.17756	0.044934	0.107914	0.186982	6837096	7.152911	7.232699	7.251018	7.360077	7.479228	7.330003	7.001945	7.106851	7.204089	7.356302	7.155608	7.142207	Pla2g6	NM_016915
6837102	5296	9.812876	9.721982	9.75214	9.890372	-0.0775	0.556721	-0.16839	0.219374	-0.13823	0.30583	-0.03016	0.817369	0.090894	0.49243	0.060736	0.643641	6837102	9.848802	9.786308	9.803517	9.877388	9.831087	9.457471	9.684914	9.857888	9.713618	9.742257	9.83517	10.09369	Tmem184b	NM_172608
6837105	5297	9.737537	9.823883	9.568348	9.722244	0.015293	0.886238	0.101639	0.355048	-0.1539	0.175513	0.255535	0.038839	-0.08635	0.428532	0.169189	0.140905	6837105	9.838724	9.676387	9.6975	9.88961	9.923448	9.65859	9.49864	9.756775	9.449629	9.617878	9.755395	9.793459	Csnk1e	NM_013767
6837113	5299	9.944615	10.05791	10.06551	9.9798	-0.03518	0.458873	0.078115	0.122327	0.085707	0.094619	-0.00759	0.870819	-0.1133	0.036612	-0.12089	0.028202	6837113	9.955961	9.983626	9.894258	9.967443	10.09587	10.11043	10.04033	10.08703	10.06916	9.945568	10.04739	9.946441	Ddx17	NM_199080
6837122	5301	6.785095	6.948285	6.706988	6.633963	0.151132	0.384431	0.314322	0.092022	0.073025	0.668408	0.241297	0.180024	-0.16319	0.349566	0.078107	0.647135	6837122	6.772031	6.864028	6.719227	6.809938	6.980448	7.054469	6.774719	7.037955	6.308291	6.582924	6.611586	6.707379	Josd1	NM_028792
6837127	5302	8.249932	8.251503	8.292674	8.238549	0.011383	0.933882	0.012954	0.92479	0.054125	0.69465	-0.04117	0.764759	-0.00157	0.990865	-0.04274	0.756118	6837127	8.309359	8.259349	8.181089	8.381664	8.268246	8.104599	8.462312	8.387627	8.028083	8.045754	8.307949	8.361944	Unc84b	NM_194342
6837130	5303	6.85983	6.886178	6.807685	6.904205	-0.04437	0.553949	-0.01803	0.808188	-0.09652	0.215951	0.078493	0.306365	-0.02635	0.723312	0.052146	0.488606	6837130	6.832938	6.817576	6.928977	6.835065	6.963077	6.860391	6.776823	6.956651	6.68958	6.867674	6.866101	6.978839	Dnalc4	NM_017470
6837131	5304	7.579436	7.693999	7.414709	7.357064	0.222372	0.120518	0.336935	0.030062	0.057645	0.66439	0.27929	0.060662	-0.11456	0.396873	0.164728	0.234072	6837131	7.533163	7.538718	7.666428	7.760453	7.829674	7.491869	7.37999	7.650626	7.21351	7.34851	7.251616	7.471066	Cbx6	NM_028763
6837143	5305	8.0355	8.106511	8.086803	8.266721	-0.23122	0.208015	-0.16021	0.370408	-0.17992	0.317651	0.019708	0.909945	-0.07101	0.6851	-0.0513	0.768967	6837143	7.83303	8.066483	8.206986	8.289496	7.758473	8.271563	8.087354	8.127145	8.045909	8.289618	8.049473	8.461073	Cbx7	NM_144811
6837144	5306	8.815241	8.804794	8.67702	8.460489	0.354752	0.045319	0.344305	0.050534	0.216531	0.186202	0.127774	0.418334	0.010447	0.946095	0.138221	0.382995	6837144	8.87862	8.861906	8.705197	8.999086	8.854197	8.561099	8.658201	8.788527	8.584332	8.191809	8.698647	8.49101	Pdgfb	NM_011057
6837189	5307	8.095753	8.155484	8.09024	8.145261	-0.04951	0.630063	0.010222	0.920203	-0.05502	0.593112	0.065244	0.527885	-0.05973	0.56252	0.005514	0.956898	6837189	8.065638	8.138759	8.082863	8.153085	8.181789	8.131577	8.150894	8.286674	7.833151	8.120485	8.201508	8.113791	Rps19bp1	NM_175109
6837252	5308	8.368588	8.167292	8.298302	7.901542	0.467046	0.023881	0.265749	0.152195	0.396759	0.04577	-0.13101	0.457766	0.201296	0.26495	0.070286	0.68656	6837252	8.530593	8.193352	8.381819	8.110051	8.509243	7.882581	8.161644	8.450561	8.2827	7.948998	8.009219	7.74641	Mkl1	NM_001082536
6837263	5309	8.652265	8.770344	8.771902	8.767269	-0.115	0.489355	0.003074	0.98502	0.004632	0.977431	-0.00156	0.992408	-0.11808	0.478185	-0.11964	0.472588	6837263	8.687073	8.548079	8.721644	8.720899	8.865689	8.724443	9.039187	8.660476	8.616042	8.481743	8.766268	9.053797	Slc25a17	NM_011399
6837279	5310	9.598986	9.652852	9.641099	9.523349	0.075637	0.58631	0.129503	0.360134	0.11775	0.403195	0.011752	0.931971	-0.05387	0.696973	-0.04211	0.760337	6837279	9.532786	9.56915	9.695021	9.578511	9.666389	9.713655	9.901439	9.579794	9.442065	9.329824	9.514374	9.725849	St13	NM_133726

6837301	5312	8.328719	8.252066	8.284231	8.076391	0.252328	0.077701	0.175674	0.196665	0.20784	0.134217	-0.03217	0.803011	0.076653	0.555927	0.044488	0.730566	6837301	8.371684	8.195871	8.418602	8.108446	8.484715	8.163036	8.424849	8.227007	8.200838	8.079046	8.227432	7.922696	Rangap1	NM_011241
6837318	5313	8.964302	8.912366	8.718454	9.143969	-0.17967	0.322097	-0.2316	0.210793	-0.42551	0.036976	0.193912	0.287654	0.051936	0.768104	0.245848	0.186714	6837318	8.762963	8.86177	9.268173	9.173497	8.691852	8.87175	8.648789	8.881364	8.62521	9.192318	8.974266	9.265323	Tob2	NM_020507
6837320	5314	7.113335	7.276499	7.122995	7.138989	-0.02565	0.844618	0.13751	0.309472	-0.01599	0.902672	0.153505	0.26032	-0.16316	0.233883	-0.00966	0.941107	6837320	7.062381	7.079949	7.197674	7.203573	7.571995	7.05393	7.056629	7.267337	7.045018	7.064809	7.184008	7.168151	Phf5a	NM_026737
6837322	5315	6.702574	6.57326	6.571346	6.682287	0.020286	0.843625	-0.10903	0.305335	-0.11094	0.297488	0.001914	0.985135	0.129314	0.230168	0.131228	0.223956	6837322	6.766397	6.597653	6.743671	6.607343	6.67612	6.436316	6.512383	6.687003	6.514652	6.525539	6.846545	6.674778	Polr3h	NM_030229
6837328	5316	7.610652	7.639971	7.614591	7.643335	-0.03268	0.813782	-0.00336	0.980623	-0.02874	0.835817	0.025381	0.854759	-0.02932	0.83259	-0.00394	0.977312	6837328	7.645069	7.560492	7.626394	7.643735	7.823577	7.452602	7.899547	7.559927	7.384298	7.605203	7.617147	7.707656	Pmm1	NM_013872
6837335	5317	8.524751	8.565694	8.524155	8.642698	-0.11795	0.545222	-0.077	0.690874	-0.11854	0.543238	0.041539	0.829524	-0.04094	0.831929	0.000597	0.997529	6837335	8.667308	8.261963	8.644983	8.569041	8.732826	8.395215	8.489866	8.817086	8.265512	8.90554	8.514513	8.508041	Pppde2	NM_134095
6837368	5318	9.125957	9.47329	9.381627	9.663839	-0.53788	0.000622	-0.19055	0.090489	-0.28221	0.021471	0.091663	0.381648	-0.34733	0.007986	-0.25567	0.032506	6837368	9.099639	9.296218	8.982014	9.472821	9.458205	9.488845	9.413045	9.506915	9.224922	9.670507	9.547664	9.773346	Naga	NM_008669
6837373	5319	8.598135	8.813006	8.616358	8.928508	-0.33037	0.090488	-0.1155	0.520018	-0.31215	0.106524	0.196648	0.285109	-0.21487	0.246053	-0.01822	0.918077	6837373	8.479227	8.523166	8.792013	8.83809	8.77965	8.821277	8.655967	8.866528	8.326579	8.997496	8.630178	9.157851	Ndufa6	NM_025987
6837375	5320	8.205805	8.355407	8.046401	8.331603	-0.1258	0.188506	0.023804	0.792498	-0.2852	0.011543	0.309006	0.007719	-0.1496	0.125722	0.159404	0.105998	6837375	8.120196	8.214193	8.283026	8.433557	8.222166	8.410497	8.063681	7.973742	8.101779	8.427671	8.162844	8.404293	Cyp2d22	NM_019823
6837409	5325	6.761199	6.865649	6.664648	6.803894	-0.04269	0.841438	0.061755	0.772617	-0.13925	0.519301	0.201001	0.359082	-0.10445	0.626782	0.096551	0.652716	6837409	6.978238	6.443522	6.861837	6.693419	6.799084	7.104444	6.582497	6.880024	6.531423	7.154623	6.567304	6.689754	Cyp2d40	NM_023623
6837412	5327	9.644872	9.606808	9.624973	9.600925	0.043947	0.642474	0.005883	0.9501	0.024048	0.798493	-0.01816	0.846944	0.038064	0.687092	0.019899	0.832578	6837412	9.569969	9.663611	9.701035	9.594776	9.58787	9.637778	9.60441	9.701645	9.568863	9.372971	9.743502	9.686302	Tcf20	NM_001114140
6837413	5328	6.714211	6.450324	6.107434	6.566292	0.147919	0.478973	-0.11597	0.576483	-0.45886	0.050193	0.34289	0.123491	0.263887	0.221843	0.606776	0.015915	6837413	6.563645	6.701196	6.877791	6.307909	6.49071	6.552353	5.666014	6.191296	6.464993	6.571901	6.743004	6.38397	Tbrg3	BC095996
6837415	5329	6.508682	6.176176	6.342462	5.781698	0.726984	0.004901	0.394478	0.070316	0.560764	0.01795	-0.16629	0.404591	0.332506	0.11656	0.16622	0.404771	6837415	6.843384	6.4063	6.276362	6.191568	6.350882	5.986077	6.536928	6.158309	6.332149	5.621973	6.055387	5.667733	Nfam1	NM_028728
6837425	5330	7.058726	7.059881	7.171321	7.325688	-0.26696	0.086723	-0.26581	0.087862	-0.15437	0.291724	-0.11144	0.438745	-0.00115	0.993472	-0.11259	0.434202	6837425	7.163969	6.907105	7.105105	7.084552	7.029702	7.065388	7.11404	7.39662	7.003302	7.346559	7.087482	7.543022	Rrp7a	NM_029101
6837426	5331	9.061185	9.143239	9.053999	8.977999	0.083186	0.275415	0.16524	0.048505	0.076	0.316024	0.08924	0.244591	-0.08205	0.281511	0.007186	0.92194	6837426	9.046288	9.075592	9.061674	9.100605	9.242783	9.08633	9.132142	9.135426	8.894429	8.935675	9.045575	8.952747	Poldip3	NM_178627
6837428	5332	12.29545	12.17598	11.9565	12.2856	0.009843	0.964416	-0.10963	0.622065	-0.3291	0.162387	0.219477	0.334782	0.11947	0.591688	0.338947	0.151639	6837428	12.30598	12.25757	12.32279	12.24961	12.27811	12.00021	11.39275	12.3305	12.14625	12.22357	12.32878	12.30446	Cyb5r3	NM_029787
6837435	5333	7.520939	7.339055	6.960083	6.756417	0.764522	0.022333	0.582638	0.063512	0.203666	0.473321	0.378972	0.199029	0.181884	0.520515	0.560856	0.071985	6837435	7.880205	7.69859	6.984022	7.268028	7.708646	7.040492	7.191406	6.951115	6.737729	6.503879	6.864053	6.90132	A4galt	NM_001004150
6837437	5334	9.332669	9.34427	9.610904	9.388796	-0.05613	0.592204	-0.04453	0.669804	0.222108	0.0583	-0.26663	0.029249	-0.0116	0.91105	-0.27824	0.024468	6837437	9.200569	9.390355	9.407084	9.289377	9.388501	9.354931	9.553246	9.793591	9.485876	9.409616	9.514569	9.242204	Arfgap3	NM_025445
6837445	5335	9.630723	9.547837	9.568096	9.659761	-0.02904	0.792532	-0.11192	0.325138	-0.09166	0.415572	-0.02026	0.854232	0.082886	0.459892	0.062627	0.573664	6837445	9.539301	9.562849	9.79002	9.595528	9.632584	9.415399	9.40234	9.691015	9.610933	9.532433	9.760287	9.686563	Pacsin2	NM_011862
6837447	5336	9.061894	9.067727	9.101552	8.881389	0.180505	0.319806	0.186338	0.305404	0.220163	0.231863	-0.03383	0.847406	-0.00583	0.973497	-0.03966	0.821583	6837447	9.174438	9.129766	8.881478	9.075546	9.286111	8.841524	9.028363	9.121202	9.155092	8.585097	8.858519	9.200552	Ttll1	NM_178869
6837453	5337	7.834175	7.902399	7.916686	7.644363	0.189812	0.090925	0.258036	0.031036	0.272324	0.024806	-0.01429	0.888586	-0.06822	0.509359	-0.08251	0.427834	6837453	7.927698	7.752704	7.822123	7.839704	8.040856	7.826637	8.051607	7.754909	7.943543	7.539756	7.771614	7.621718	Ttll12	NM_183017
6837507	5341	6.622467	6.608642	6.756463	7.035065	-0.4126	0.028086	-0.42642	0.024448	-0.2786	0.108361	-0.14782	0.365719	0.013825	0.930749	-0.134	0.410077	6837507	6.638383	6.642491	6.586528	6.579168	6.485903	6.760856	6.75861	6.775857	6.734921	6.798817	6.870705	7.435673	Ldoc1l	NM_177630
6837582	5343	5.922167	5.956177	5.875237	6.168357	-0.24619	0.16917	-0.21218	0.228994	-0.29312	0.109664	0.08094	0.632654	-0.03401	0.839843	0.04693	0.780605	6837582	5.891698	5.962782	5.912021	5.856479	6.164873	5.847179	6.032709	6.052627	5.540375	5.992583	6.389764	6.122723	Wnt7b	NM_009528
6837594	5344	6.06569	6.055111	6.472409	6.428723	-0.36303	0.061724	-0.37361	0.055925	0.043685	0.800504	-0.4173	0.037203	0.010579	0.951107	-0.40672	0.04106	6837594	6.041792	6.29647	5.858809	6.079494	5.969175	6.116664	6.494884	6.612312	6.31003	6.091064	6.52795	6.667156	2210021J	BC025858
6837604	5346	8.61363	9.028549	9.366297	10.07458	-1.46095	0.001662	-1.04603	0.010486	-0.70828	0.054537	-0.33775	0.314528	-0.41492	0.223899	-0.75267	0.043761	6837604	8.785404	9.082208	7.973277	9.314688	8.679426	9.091534	9.338948	9.682416	9.077526	9.828322	10.04463	10.35078	Celsr1	NM_009886
6837614	5347	7.867456	7.884158	7.804993	7.701102	0.166353	0.097176	0.183056	0.072593	0.103891	0.27455	0.079165	0.397517	-0.0167	0.855119	0.062463	0.500684	6837614	7.988619	7.747866	7.865882	7.854959	8.03532	7.762196	7.918494	7.773377	7.723108	7.683801	7.762383	7.657123	Cerk	NM_145475
6837766	5348	8.763284	8.500694	9.013472	9.021319	-0.25803	0.106621	-0.52062	0.006332	-0.00785	0.957272	-0.51278	0.006859	0.26259	0.101504	-0.25019	0.116012	6837766	8.672445	8.72104	8.896367	8.42719	8.644356	8.430536	8.933929	9.042535	9.063952	9.166968	9.21454	8.682449	Alg12	NM_001142357
6837783	5351	7.487167	7.460386	7.527637	7.37992	0.107248	0.249485	0.080466	0.378753	0.147717	0.125565	-0.06725	0.458581	0.026781	0.764414	-0.04047	0.651871	6837783	7.467019	7.557405	7.437078	7.474812	7.462402	7.443944	7.469975	7.450285	7.66265	7.194914	7.506331	7.438514	Hdac10	NM_199198
6837784	5352	7.02041	7.114661	6.794314	6.751582	0.268828	0.071564	0.363079	0.023063	0.042731	0.749888	0.320348	0.038481	-0.09425	0.487469	0.226096	0.118959	6837784	6.872004	6.917751	7.271475	7.172779	7.120811	7.050394	6.933995	6.763718	6.685228	6.549702	6.900519	6.804526	Mapk12	NM_013871
6837785	5353	8.097515	7.888691	7.668794	6.943028	1.154487	0.001907	0.945663	0.005903	0.725766	0.02141	0.219897	0.412666	0.208824	0.435623	0.428721	0.130523	6837785	8.03142	7.997289	8.263836	7.855036	8.248629	7.562407	7.627978	7.440209	7.938195	6.847487	7.413663	6.567933	Mapk11	NM_011161
6837787	5354	10.58756	10.52455	10.5869	10.40988	0.177687	0.03532	0.114677	0.141309	0.177027	0.035841	-0.06235	0.400775	0.06301	0.396034	0.00066	0.992736	6837787	10.68064	10.57365	10.5084	10.52625	10.58982	10.45759	10.62453	10.60091	10.53527	10.27351	10.51808	10.43804	Plxnb2	NM_001159521
6837791	5355	6.949878	7.008393	7.057118	7.060559	-0.11068	0.172616	-0.05217	0.500318	-0.00344	0.963999	-0.04872	0.528234	-0.05851	0.451348	-0.10724	0.184829	6837791	7.034041	6.97584	6.839753	7.008578	7.062448	6.954152	7.144963	7.108771	6.917619	7.043422	7.000165	7.138091	Fam116b	NM_027081
6837805	5356	9.448087	9.790255	9.412127	10.2474	-0.79931	0.009735	-0.45714	0.089809	-0.83527	0.007786	0.378129	0.14918	-0.34217	0.186703	0.035961	0.883125	6837805	9.433213	9.595657	9.315392	10.06568	9.577391	9.727695	8.995601	9.927645	9.313134	10.05945	10.2824	10.40034	Sbf1	NM_001081030
6837809	5357	9.783945	9.459688	9.609234	9.932736	-0.14879	0.16777	-0.47305	0.001317	-0.3235	0.010891	-0.14955	0.165872	0.324257	0.010767	0.174711	0.112755	6837809	9.766899	9.847867	9.737069	9.447236	9.464071	9.467757	9.478522	9.514884	9.834295	9.804907	10.05787	9.935431	Lmf2	NM_178919
6837812	5358	6.924401	6.991031	7.01513	7.09662	-0.17222	0.102429	-0.10559	0.291013	-0.08149	0.408361	-0.0241	0.802908	-0.06663	0.49588	-0.09073	0.359792	6837812	6.881008	6.980349	6.911845	6.994314	6.915837	7.062943	7.050375	7.126487	6.868527	6.981863	7.024028	7.283968	Tymp	NM_138302
6837815	5359	6.190271	6.192098	6.049168	6.072665	0.117606	0.146201	0.119433	0.140821	-0.0235	0.756036	0.14293	0.0862	-0.00183	0.980666	0.141103	0.089593	6837815	6.259047	6.13332	6.178447	6.257492	6.293288	6.025515	6.072013	6.109623	5.965868	6.113297	6.044946	6.059753	Odf3b	NM_001013022
6837818	5360	6.557716	6.478782	6.299499	6.348142	0.209573	0.659248	0.13064	0.782589	-0.04864	0.917987	0.179283	0.705532	0.078933	0.867375	0.258216	0.588144	6837818	5.998849	6.175426	7.498872	7.101214	6.237729	6.097404	6.691546	6.239812	5.96714	5.893146	6.596694	6.554587	Cpt1b	NM_009948
6837819	5361	7.407391	7.466052	7.411525	7.388005	0.019386	0.612756	0.078047	0.066822	0.02352	0.540761	0.054528	0.176841	-0.05866	0.149729	-0.00413	0.913371	6837819	7.372906	7.398942	7.450326	7.434461	7.531163	7.432533	7.382876	7.470113	7.381585	7.374759	7.368307	7.420949	Chkb	NM_007692
6837827	5362	7.474748	7.447714	7.471006	7.46185	0.012898	0.862936	-0.01414	0.849958	0.009156	0.902411	-0.02329	0.755741	0.027034	0.718358	0.003742	0.960021	6837827	7.501145	7.519441	7.403658	7.433377	7.611445	7.29832	7.494446	7.462359	7.456214	7.488427	7.404818	7.492305	Arsa	NM_009713
6837835	5363	7.110982	7.155352	7.639162	7.292532	-0.18155	0.103193	-0.13718	0.202097	0.34663	0.007946	-0.48381	0.001192	-0.04437	0.665027	-0.52818	0.000686	6837835	7.197719	7.005803	7.129425	7.043796	7.237777	7.184484	7.447289	7.724627	7.74557	7.225571	7.41479	7.237236	Rabl2a	NM_026817
6837897	5365	6.51707	6.690027	6.396243	6.316838	0.200232	0.528711	0.373189	0.254599	0.079405	0.800584	0.293785	0.362259	-0.17296	0.585101	0.120827	0.701493	6837897	6.893148	6.356271	6.30179	6.646415	7.065805	6.357862	6.747306	5.897405	6.544017	6.180701	6.717119	6.052694	Cpne8	NM_025815
6837925	5366	8.036425	8.097058	7.988574	8.187506	-0.15108	0.203002	-0.09045	0.430571	-0.19893	0.105334	0.108484	0.348603	-0.06063	0.593124	0.047851	0.672175	6837925	8.051159	7.875622	8.182495	8.123525	8.15696	8.01069	8.099663	8.076555	7.789504	8.149372	8.103066	8.310081	Kif21a	NM_001109040
6838032	5368	8.174961	8.378424	8.465121	8.39311	-0.21815	0.112253	-0.01469	0.907366	0.072012	0.572162	-0.0867	0.498448	-0.20346	0.13469	-0.29016	0.045036	6838032	8.207747	8.154511	8.162626	8.243331	8.517876	8.374066	8.678662	8.542302	8.1744	8.341733	8.422174	8.415422	Yaf2	NM_024189
6838036	5369	9.191412	9.181673	9.235336	9.25505	-0.06364	0.604223	-0.07338	0.551205	-0.01971	0.871415	-0.05366	0.66124	0.009739	0.936224	-0.04392	0.719282	6838036	9.185673	9.225883	9.16268	9.109108	9.237356	9.198554	9.327113	9.285377	9.093519	9.028584	9.212887	9.52368	Zcrb1	NM_026025
6838136	5372	10.39967	10.39329	10.43038	10.14481	0.254863	0.078754	0.24848	0.085152	0.285577	0.053973	-0.0371	0.776839	0.006383	0.961001	-0.03071	0.814315	6838136	10.47603	10.31315	10.40983	10.29994	10.62927	10.25065	10.52289	10.4853	10.28296	9.98404	10.32976	10.12062	Twf1	NM_008971
6838183	5374	6.751312	6.831656	6.783056	7.028204	-0.27689	0.101666	-0.19655	0.225807	-0.24515	0.140301	0.048601	0.75388	-0.08034	0.606239	-0.03174	0.837448	6838183	6.807157	6.707511	6.739269	7.115412	6.540029	6.839528	6.99468	6.754672	6.599815	7.056365	6.919238	7.10901	Dbx2	NM_207533
6838249	5375	8.688001	8.812124	8.767621	8.657622	0.030379	0.66985	0.154502	0.054531	0.109999	0.147776	0.044503	0.535003	-0.12412	0.108232	-0.07962	0.279604	6838249	8.644665	8.666626	8.752712	8.720212	8.865564	8.850595	8.853614	8.77626	8.672988	8.542357	8.694025	8.736483	Sfrs2ip	NM_028148
6838256	5376	9.62081	9.529478	9.298866	9.118548	0.502261	0.044074	0.41093	0.086617	0.180318	0.416431	0.230612	0.305015	0.091332	0.675741	0.321943	0.164566	6838256	9.76384	9.64238	9.456209	9.330589	9.824212	9.433633	9.576519	9.085083	9.234997	9.321061	9.299568	8.735016	Slc38a1	NM_134086
6838257	5377	10.25166	10.47077	10.22204	10.31273	-0.06107	0.683046	0.158043	0.30492	-0.09069	0.546901	0.248731	0.122792	-0.21911	0.167077	0.029618	0.842377	6838257	10.38274	10.15532	10.21692	10.40411	10.60393	10.40428	10.3792	10.42008	9.866847	10.37072	10.27763	10.28984	Slc38a2	NM_175121
6838303	5379	6.148424	6.38403	6.411702	6.210443	-0.06202	0.534194	0.173586	0.106582	0.201259	0.068107	-0.02767	0.779326	-0.23561	0.038861	-0.26328	0.024777	6838303	6.118639	6.308362	6.01827	6.487524	6.470661	6.193904	6.474555	6.422688	6.337864	6.173656	6.222499	6.235175	Amigo2	NM_178114
6838327	5380	6.423642	6.616146	6.79366	6.333984	0.089658	0.530938	0.282162	0.07326	0.459676	0.00997	-0.17751	0.230936	-0.1925	0.197347	-0.37002	0.026967	6838327	6.443267	6.452473	6.375186	6.67213	6.599639	6.57667	6.925398	6.779066	6.676517	5.987628	6.456265	6.558059	Rpap3	NM_028003
6838331	5381	6.279516	6.499775	6.236663	7.026339	-0.74682	0.006158	-0.52656	0.031642	-0.78968	0.004555	0.263112	0.230157	-0.22026	0.308549	0.042853	0.837743	6838331	6.2529	6.4178	6.167848	6.613347	6.28645	6.599527	6.114419	6.301975	6.293594	6.555908	7.398145	7.124963	Pp11r	NM_008902
6838332	5382	7.575057	7.659696	7.409984	7.830045	-0.25499	0.185068	-0.17035	0.361024	-0.42006	0.04393	0.249711	0.193347	-0.08464	0.643161	0.165073	0.375319	6838332	7.602505	7.611461	7.511205	7.815263	7.535932	7.627892	7.272216	7.700333	7.257404	7.535168	7.793978	8.160989	Rapgef3	NM_144850
6838337	5383	8.208554	8.289365	8.027576	8.103269	0.105285	0.364858	0.186096	0.12794	-0.07569	0.509324	0.261789	0.043949	-0.08081	0.481976	0.180978	0.137282	6838337	8.234243	8.254854	8.136565	8.440116	8.310275	8.117704	8.011106	8.233556	7.838065	8.04689	8.139852	8.123065	Hdac7	NM_019572
6838338	5384	8.6113	9.031283	8.712929	8.801357	-0.19006	0.217538	0.229926	0.144059	-0.08843	0.550789	0.318353	0.055256	-0.41998	0.018211	-0.10163	0.494526	6838338	8.639547	8.765477	8.428876	9.130996	8.987985	8.974867	8.710578	8.755766	8.672444	8.657387	8.613976	9.132708	Vdr	NM_009504
6838346	5385	6.610611	6.65915	6.524698	6.657909	-0.0473	0.713366	0.00124	0.99228	-0.13321	0.314951	0.134452	0.310753	-0.04854	0.70626	0.085913	0.508855	6838346	6.686677	6.603659	6.541498	6.649868	6.547469	6.780112	6.705393	6.583889	6.284812	6.74113	6.469029	6.763569	Col2a1	NM_031163
6838349	5386	8.146906	8.102631	8.094815	7.929727	0.217179	0.04368	0.172904	0.093274	0.165088	0.106462	0.007816	0.933501	0.044275	0.638804	0.052091	0.58183	6838349	8.148988	8.076194	8.215536	8.039974	8.276833	7.991085	8.169715	8.106751	8.007979	7.831681	8.064579	7.89292	Senp1	NM_144851
6838355	5387	7.874041	7.835715	7.952339	7.770694	0.103347	0.44366	0.065021	0.62586	0.181644	0.19444	-0.11662	0.389769	0.038326	0.772692	-0.0783	0.55849	6838355	7.773321	7.80095	8.047853	7.881566	7.971176	7.654404	8.063808	7.916186	7.877022	7.691888	7.623475	7.99672	Asb8	NM_030121
6838367	5389	6.599015	6.578624	6.619997	6.762691	-0.16368	0.272429	-0.18407	0.221634	-0.14269	0.334247	-0.04137	0.77336	0.020391	0.886899	-0.02098	0.883651	6838367	6.605594	6.403505	6.787946	6.702812	6.412001	6.621059	6.508512	6.65003	6.701448	6.61375	6.665047	7.009276	Zfp641	NM_173769
6838382	5394	8.51965	8.741025	8.585598	8.796481	-0.27683	0.062579	-0.05546	0.676361	-0.21088	0.138158	0.155427	0.259386	-0.22137	0.122063	-0.06595	0.620426	6838382	8.705879	8.613882	8.239189	8.673517	8.665514	8.884044	8.691324	8.621572	8.443897	8.758844	8.747824	8.882774	2310037I	BC003301
6838383	5395	9.030175	8.994014	8.985417	9.013295	0.01688	0.809873	-0.01928	0.78359	-0.02788	0.692068	0.008598	0.902333	0.03616	0.608699	0.044758	0.528168	6838383	9.054807	9.027113	9.008604	8.938182	9.033502	9.010359	8.828867	9.091967	9.035416	9.081985	9.022687	8.935212	Ccnt1	NM_009833

6838386	5396	8.224632	8.286242	8.192326	8.360092	-0.13546	0.367476	-0.07385	0.616666	-0.16777	0.270799	0.093916	0.52645	-0.06161	0.675463	0.032306	0.825524	6838386	8.101764	8.289656	8.282475	8.353331	8.24135	8.264046	7.985208	8.505027	8.086743	8.248485	8.272414	8.559376	Adcy6	NM_007405
6838390	5397	8.287296	8.368009	8.270143	8.177487	0.109809	0.217362	0.190522	0.048673	0.092656	0.291274	0.097866	0.266906	-0.08071	0.353834	0.017153	0.839547	6838390	8.276166	8.278552	8.307169	8.319047	8.385292	8.399687	8.446936	8.213191	8.150301	8.042	8.247565	8.242895	Ddx23	NM_001080981
6838392	5398	6.789337	6.637448	6.930853	6.184626	0.604711	0.021742	0.452822	0.065975	0.746227	0.007994	-0.29341	0.205225	0.151889	0.495606	-0.14152	0.524686	6838392	7.124856	6.902106	6.34105	6.666598	6.8135	6.432246	7.020077	6.788436	6.984046	5.914005	6.287366	6.352507	Rnd1	NM_172612
6838394	5399	7.810692	7.868391	7.717852	8.222371	-0.41168	0.037501	-0.35398	0.064643	-0.50452	0.015774	0.150539	0.389093	-0.0957	0.736073	0.09284	0.589759	6838394	7.986472	7.831895	7.61371	7.793355	7.733881	8.077936	7.881284	7.725428	7.546844	8.517921	8.113999	8.035192	Fkbp11	NM_024169
6838397	5400	9.22919	9.271081	9.011991	9.07023	0.15896	0.191077	0.20085	0.108782	-0.05824	0.614964	0.25909	0.04832	-0.04189	0.71642	0.217199	0.086789	6838397	9.295484	9.129369	9.262718	9.361804	9.345195	9.106243	8.844609	9.205545	8.985819	8.948218	9.087804	9.174669	Arf3	NM_007478
6838398	5401	6.385644	6.307075	6.127976	6.373339	0.012305	0.92376	-0.06626	0.609295	-0.24536	0.084442	0.179099	0.188536	0.078569	0.545901	0.257668	0.072453	6838398	6.586374	6.393487	6.177072	6.265005	6.255004	6.401217	6.027572	6.366382	5.989974	6.398409	6.393886	6.327723	5830427D	NM_175232
6838399	5402	5.941469	5.898582	5.815246	5.94427	-0.0028	0.986491	-0.04569	0.78297	-0.12902	0.444345	0.083337	0.617382	0.042886	0.795915	0.126223	0.453933	6838399	6.190076	5.897542	5.736788	6.056581	5.894831	5.744335	5.934674	5.934027	5.577036	6.153705	5.880344	5.798761	Wnt10b	NM_011718
6838405	5403	8.95668	8.982565	8.856362	8.783982	0.172698	0.08947	0.198583	0.057061	0.07238	0.441559	0.126203	0.195724	-0.02588	0.779524	0.100318	0.294339	6838405	9.027819	8.952379	8.889842	8.938687	9.080872	8.928135	9.002922	8.911673	8.65449	8.724194	8.789904	8.837848	Prkag1	NM_016781
6838410	5404	5.991478	6.14299	6.100969	6.103831	-0.11235	0.338613	0.039159	0.73198	-0.00286	0.979953	0.042021	0.713387	-0.15151	0.207184	-0.10949	0.350358	6838410	6.070221	5.897167	6.007046	6.088219	5.96061	6.380141	6.154525	6.186136	5.962245	6.163978	6.031185	6.116329	Rhebl1	NM_026967
6838413	5406	7.218145	7.306836	7.126112	7.216714	0.001431	0.9857	0.090122	0.277648	-0.0906	0.275283	0.180724	0.047724	-0.08869	0.284788	0.092033	0.268339	6838413	7.24509	7.192766	7.216579	7.294745	7.439405	7.186358	7.163198	7.233295	6.981844	7.17048	7.213936	7.265727	Lmbr1l	NM_029098
6838417	5407	11.52736	11.05277	11.10991	10.41774	1.109623	0.017921	0.635027	0.127812	0.692167	0.101243	-0.05714	0.882305	0.474597	0.23994	0.417457	0.296548	6838417	11.57779	11.2009	11.8034	10.77273	11.53885	10.84672	11.72796	10.68122	10.92054	10.15642	11.01238	10.08442	Tuba1a	NM_011653
6838450	5408	9.11571	9.13197	8.946864	9.360053	-0.24434	0.042322	-0.22808	0.054377	-0.41319	0.003534	0.185107	0.10498	-0.01626	0.876414	0.168846	0.134014	6838450	9.128903	9.190264	9.027962	9.128328	9.106565	9.161018	8.903099	9.116052	8.82144	9.20385	9.325769	9.550541	Fmnl3	NM_011711
6838455	5409	6.647181	6.701037	6.722446	6.577218	0.069962	0.63385	0.123818	0.406463	0.145227	0.334161	-0.02141	0.883332	-0.05386	0.713044	-0.07526	0.608775	6838455	6.70122	6.710804	6.529518	6.802854	6.734221	6.566035	6.540679	7.047652	6.579006	6.668479	6.624493	6.438683	C230021P	BC072572
6838459	5410	6.968859	6.778948	6.982504	6.876712	0.092147	0.61332	-0.09776	0.592246	0.105792	0.562827	-0.20356	0.278958	0.189911	0.310139	-0.01365	0.939858	6838459	6.865764	6.666412	7.374401	6.717352	6.727039	6.892454	6.974968	7.1551	6.847034	6.728146	6.877787	7.024203	Bcdin3d	NM_029236
6838469	5412	6.811392	7.016294	6.644018	5.797033	1.014359	0.047038	1.219261	0.022534	0.846985	0.085901	0.372276	0.414466	-0.2049	0.64836	0.167374	0.708878	6838469	6.934051	6.553142	6.946984	6.612078	7.740177	6.696626	7.146636	5.726383	7.059035	5.641275	6.036254	5.71357	Racgap1	NM_012025
6838483	5413	8.650602	8.650128	8.663104	8.626614	0.023987	0.819562	0.023514	0.823054	0.03649	0.729174	-0.01298	0.901676	0.000474	0.9964	-0.0125	0.905244	6838483	8.51762	8.762738	8.671447	8.586216	8.835836	8.528332	8.759169	8.64693	8.583213	8.583723	8.751791	8.544329	Lass5	NM_028015
6838492	5414	8.347951	8.384853	8.046203	7.660554	0.687397	0.024483	0.724299	0.019485	0.385649	0.159436	0.33865	0.210243	-0.0369	0.88567	0.301748	0.259448	6838492	8.538156	8.227799	8.277897	8.228296	8.713243	8.21302	8.427494	7.705464	8.005651	7.333342	8.048332	7.599988	Lima1	NM_001113545
6838557	5416	8.264869	8.01006	8.212533	7.968629	0.29624	0.097436	0.041431	0.799619	0.243904	0.160942	-0.20247	0.235575	0.254809	0.145188	0.052336	0.748779	6838557	8.350689	8.182832	8.261087	8.018357	8.215151	7.796673	8.209455	8.078344	8.349801	7.882263	8.285001	7.738624	Slc11a2	NM_008732
6838563	5417	6.492941	6.501178	6.243559	6.284333	0.208608	0.05724	0.216845	0.049924	-0.04077	0.6759	0.257619	0.025419	-0.00824	0.932325	0.249383	0.029113	6838563	6.463271	6.470909	6.544644	6.470553	6.540635	6.492346	6.386314	6.191529	6.152833	6.077157	6.344858	6.430984	Csrnp2	NM_153407
6838564	5418	6.976941	7.070592	6.925094	7.135436	-0.15849	0.193261	-0.06484	0.577155	-0.21034	0.09615	0.145498	0.228515	-0.09365	0.425668	0.051847	0.654562	6838564	6.858756	7.002731	7.069337	7.040666	7.249303	6.921808	6.772235	7.132004	6.871043	7.10264	7.13518	7.168488	Tcfcp2	NM_033476
6838565	5419	6.940189	6.984091	6.898571	6.920292	0.019897	0.813838	0.063799	0.457642	-0.02172	0.797202	0.08552	0.326112	-0.0439	0.605884	0.041618	0.624451	6838565	6.956132	6.952813	6.911622	7.020833	6.909254	7.022185	6.871298	7.07253	6.751884	6.989675	6.812006	6.959194	Pou6f1	NM_010127
6838578	5420	6.802142	6.655495	6.899428	6.764119	0.038023	0.804448	-0.10862	0.485524	0.135309	0.389012	-0.24393	0.139215	0.146648	0.352484	-0.09729	0.530929	6838578	6.91947	6.875956	6.611001	6.753391	6.855998	6.357095	6.985925	6.722444	6.989914	6.870728	6.766561	6.655068	cela1	NM_033612
6838621	5422	6.754082	6.791619	6.673092	6.788845	-0.03476	0.659773	0.002773	0.971806	-0.11575	0.166523	0.118527	0.157757	-0.03754	0.634895	0.08099	0.318022	6838621	6.772395	6.787069	6.702781	6.721807	6.738886	6.914163	6.598696	6.744908	6.675672	6.794705	6.659546	6.912285	4732456N	NM_177717
6838648	5425	6.43086	6.497122	6.472023	6.460387	-0.02953	0.730489	0.036735	0.668902	0.011636	0.891665	0.025099	0.769423	-0.06626	0.446465	-0.04116	0.632309	6838648	6.496214	6.446677	6.349688	6.532206	6.312131	6.647029	6.511552	6.518687	6.38583	6.441812	6.442536	6.496813	Krt82	NM_053249
6838655	5429	6.179945	6.650447	5.980731	7.840234	-1.66029	0.071794	-1.18979	0.17554	-1.8595	0.048712	0.669716	0.427125	-0.4705	0.572939	0.199214	0.809753	6838655	6.046216	6.151158	6.342462	6.462848	7.571899	5.916594	5.933423	5.977333	6.031437	7.719541	9.660741	6.14042	Krt5	NM_027011
6838692	5439	5.607659	5.317049	4.541614	7.572064	-1.96441	0.359441	-2.25501	0.296825	-3.03045	0.172069	0.775435	0.711158	0.29061	0.889199	1.066045	0.612109	6838692	4.594497	4.275783	7.952696	4.761239	6.758292	4.431615	4.575692	4.805511	4.243639	5.704813	12.51425	4.497128	Krt4	NM_008475
6838713	5442	7.691677	7.684383	7.597497	7.68989	0.001787	0.948552	-0.00551	0.842523	-0.09239	0.008786	0.086886	0.011919	0.007294	0.792657	0.09418	0.007967	6838713	7.702251	7.701456	7.671323	7.693755	7.718749	7.640644	7.637994	7.564724	7.589772	7.708648	7.70823	7.652792	Csad	NM_144942
6838716	5443	6.174803	6.065699	6.196031	5.917698	0.257105	0.024227	0.148001	0.149243	0.278334	0.017053	-0.13033	0.197605	0.109104	0.273305	-0.02123	0.824714	6838716	6.324726	6.083807	6.115875	6.109963	6.184728	5.902405	6.269992	6.142594	6.175508	5.917766	6.011134	5.824193	Itgb7	NM_013566
6838717	5444	7.79694	7.759948	7.733686	7.304204	0.492736	0.012011	0.455744	0.017337	0.429482	0.022577	0.026262	0.867488	0.036993	0.814353	0.063255	0.689063	6838717	7.742373	7.852568	7.79588	7.661594	8.001135	7.617114	7.779115	7.889321	7.532621	7.077421	7.560136	7.275055	Rarg	NM_011244
6838730	5445	7.833266	7.723644	7.730483	7.497823	0.335443	0.109027	0.225822	0.259408	0.232661	0.246401	-0.00684	0.971575	0.109621	0.571947	0.102782	0.595702	6838730	7.903205	7.693048	7.903544	7.687821	7.962325	7.520787	7.8896	7.350973	7.950877	7.534587	7.661722	7.297159	Aaas	NM_153416
6838746	5447	7.271742	7.493381	7.239695	7.160473	0.111268	0.240681	0.332908	0.005298	0.079222	0.393238	0.253686	0.020216	-0.22164	0.035562	0.032046	0.72457	6838746	7.364585	7.378701	7.071939	7.55665	7.489704	7.433789	7.292761	7.283381	7.142944	7.076686	7.189413	7.215321	Map3k12	NM_009582
6838747	5448	6.602052	6.74736	6.723212	6.739407	-0.13736	0.199931	0.007952	0.937521	-0.01619	0.87325	0.024147	0.812172	-0.14531	0.177679	-0.12116	0.252812	6838747	6.635914	6.728397	6.441844	6.839487	6.669408	6.733184	6.640166	6.87111	6.658361	6.863741	6.643387	6.711094	Npff	NM_018787
6838750	5449	9.994102	9.939299	9.87947	9.882859	0.111243	0.314444	0.05644	0.600911	-0.00339	0.974719	0.059829	0.579655	0.054803	0.611331	0.114632	0.300878	6838750	9.956918	9.982898	10.04249	9.984209	9.943431	9.890257	9.65196	10.13947	9.84698	9.892181	9.884366	9.872029	Atf7	ENSMUST000000
6838754	5450	10.40895	10.46518	10.29399	10.26274	0.146207	0.34188	0.20244	0.19936	0.03125	0.834421	0.17119	0.270747	-0.05623	0.707696	0.114957	0.449858	6838754	10.2778	10.40524	10.54381	10.59005	10.33443	10.47107	10.2559	10.1824	10.44368	10.0178	10.21665	10.55378	Calcoco1	NM_026192
6838797	5452	10.13742	10.11634	10.05655	10.11348	0.023938	0.820637	0.002858	0.978368	-0.05693	0.592622	0.059787	0.574549	0.02108	0.84169	0.080867	0.451469	6838797	10.1062	10.09054	10.21551	9.985241	10.22943	10.13434	10.01532	10.13601	10.01832	9.938167	10.07844	10.32383	Cbx5	NM_001110216
6838807	5454	5.380995	5.787117	5.817157	4.802255	0.578741	0.423229	0.984862	0.188892	1.014902	0.177159	-0.03004	0.966134	-0.40612	0.570066	-0.43616	0.542532	6838807	6.078192	5.528716	4.536078	5.849659	6.128407	5.383285	5.590426	4.738054	7.122991	4.020463	5.580663	4.805638	Gpr84	NM_030720
6838808	5455	7.678844	7.712636	7.530327	7.219569	0.459275	0.053266	0.493068	0.041064	0.310759	0.163847	0.182309	0.394769	-0.03379	0.871752	0.148517	0.484719	6838808	7.900777	7.370462	7.765293	7.846683	8.007008	7.284218	7.582209	7.579348	7.429425	7.305523	7.292435	7.060748	Zfp385a	NM_013866
6838809	5456	7.840992	8.022763	7.674713	7.386385	0.454607	0.057422	0.636378	0.01458	0.288328	0.197269	0.34805	0.128018	-0.18177	0.401172	0.166279	0.440814	6838809	7.991296	7.836458	7.695223	7.937144	8.33274	7.798406	7.877858	7.554779	7.591502	7.084105	7.769017	7.306034	Itga5	NM_010577
6838979	5461	6.345188	6.219275	6.366872	6.339553	0.005635	0.968506	-0.12028	0.409923	0.027319	0.848373	-0.1476	0.317134	0.125912	0.389321	-0.02168	0.879325	6838979	6.44089	6.393365	6.201308	6.26022	5.912356	6.48525	6.30907	6.406954	6.236237	6.460381	6.32204	Zfp263	NM_148924
6838999	5463	8.741345	8.663206	8.782622	8.709989	0.031356	0.813072	-0.04678	0.724822	0.072632	0.58683	-0.11942	0.379197	0.078139	0.559383	-0.04128	0.755903	6838999	8.7567	8.734868	8.732467	8.702725	8.896278	8.390615	8.83342	8.850017	8.664428	8.572859	8.872554	8.684555	Nat15	NM_029090
6839008	5464	7.270849	7.447995	7.432295	7.34177	-0.07092	0.470899	0.106225	0.289845	0.090525	0.362368	0.0157	0.871113	-0.17715	0.095397	-0.16145	0.123245	6839008	7.110214	7.343359	7.358975	7.407218	7.465889	7.470879	7.534614	7.364702	7.397569	7.168994	7.467156	7.38916	Cluap1	NM_029738
6839052	5466	7.50514	7.648475	7.27619	7.470303	0.034837	0.79741	0.178172	0.211708	-0.19411	0.177444	0.372285	0.021939	-0.14333	0.306631	0.22895	0.119273	6839052	7.5744	7.477014	7.464006	7.749286	7.711424	7.484714	7.158306	7.515533	7.15473	7.328295	7.395798	7.686816	Glis2	NM_031184
6839054	5467	6.527281	6.584394	6.3724	6.921275	-0.39399	0.03463	-0.33688	0.061523	-0.54887	0.007614	0.211994	0.208654	-0.05711	0.722123	0.154881	0.347016	6839054	6.557842	6.55592	6.468081	6.661622	6.609188	6.482373	6.246355	6.760156	6.110689	6.912142	6.800768	7.050914	Vasn	NM_139307
6839057	5468	8.02987	8.158122	8.086735	8.242714	-0.21284	0.000807	-0.08459	0.071895	-0.15598	0.005073	0.071387	0.118346	-0.12825	0.013745	-0.05686	0.200959	6839057	8.045997	7.976522	8.067085	8.124734	8.152958	8.196673	8.122216	8.12062	8.017369	8.186115	8.250512	8.291515	Dnaja3	NM_023646
6839059	5469	9.191643	9.273491	9.269443	9.247167	-0.05552	0.582098	0.026324	0.792602	0.022276	0.823817	0.004049	0.967671	-0.08185	0.42249	-0.0778	0.444896	6839059	9.045314	9.23013	9.299485	9.25033	9.361306	9.208838	9.352109	9.211487	9.244732	9.090596	9.230652	9.420253	Hmox2	NM_001136066
6839078	5470	9.885125	9.803828	9.855888	10.04664	-0.16151	0.076865	-0.24281	0.01578	-0.19075	0.043353	-0.05206	0.531267	0.081297	0.336789	0.029237	0.722805	6839078	9.848225	9.864104	9.943045	9.766779	9.815762	9.828942	9.77222	9.958114	9.837329	9.891985	10.03738	10.21054	Mgrn1	NM_029657
6839080	5471	7.5794	7.673509	7.611496	7.586388	-0.00699	0.905203	0.087121	0.163966	0.025108	0.670451	0.062013	0.307141	-0.09411	0.136457	-0.0321	0.587856	6839080	7.63144	7.483156	7.623604	7.692768	7.670921	7.656838	7.635552	7.627347	7.57159	7.467292	7.629995	7.661878	Nudt16l1	NM_025839
6839102	5472	8.299295	8.394024	8.187943	8.399973	-0.10068	0.35826	-0.00595	0.955487	-0.21203	0.074193	0.206081	0.081125	-0.09473	0.385902	0.111352	0.312448	6839102	8.322352	8.317046	8.258487	8.389277	8.352906	8.439889	8.309433	8.317381	7.937015	8.316936	8.349622	8.53336	Alg1	NM_145362
6839135	5473	6.147048	6.059243	5.973968	5.864389	0.282659	0.57211	0.194854	0.69536	0.10958	0.825109	0.085274	0.86338	0.087805	0.859375	0.17308	0.727689	6839135	5.451046	5.900877	7.089221	6.705086	5.99664	5.476002	6.313081	5.999439	5.609385	5.420771	5.972414	6.199981	A2bp1	NM_021477
6839230	5474	7.844532	7.70037	7.811498	7.63361	0.210922	0.200949	0.06676	0.670835	0.177888	0.273668	-0.11113	0.483777	0.144162	0.368746	0.033034	0.832698	6839230	7.719077	7.692339	8.122179	7.81625	7.774734	7.510125	8.057212	7.704005	7.673277	7.547535	7.648234	7.705061	BC024814	BC024814
6839231	5475	6.523006	6.891056	6.53017	6.739329	-0.21632	0.15236	0.151728	0.299472	-0.20916	0.164696	0.360886	0.029767	-0.36805	0.027445	-0.00716	0.959507	6839231	6.659274	6.541988	6.367757	6.912255	7.136542	6.624372	6.566717	6.599812	6.423982	6.704471	6.633691	6.879824	Abat	NM_172961
6839240	5476	8.577094	8.631619	8.625374	8.620595	-0.0435	0.426843	0.011024	0.837307	0.004779	0.928989	0.006245	0.90731	-0.05452	0.324738	-0.04828	0.380044	6839240	8.54276	8.575887	8.612636	8.613431	8.688432	8.592994	8.564959	8.670939	8.640224	8.537082	8.597142	8.727561	Pmm2	NM_016881
6839334	5479	8.292876	8.180757	8.320184	8.174604	0.118272	0.292427	0.006154	0.954681	0.14558	0.202814	-0.13943	0.220651	0.112118	0.31655	-0.02731	0.801278	6839334	8.344599	8.262568	8.27146	8.022926	8.354734	8.164612	8.51983	8.244562	8.196159	8.098817	8.251887	8.173107	Nubp1	NM_011955
6839340	5480	7.036514	6.721668	6.461311	5.96554	1.070974	0.005256	0.756128	0.02788	0.495771	0.11682	0.260358	0.382916	0.314845	0.296667	0.575203	0.075727	6839340	7.688215	7.058457	6.362869	6.741892	6.82272	6.600393	6.593976	6.346214	6.443742	6.01897	6.024539	5.853111	Ciita	NM_007575
6839380	5481	7.091989	7.309895	7.100067	7.37839	-0.2864	0.141106	-0.0685	0.706321	-0.27832	0.151181	0.209828	0.265785	-0.21791	0.249245	-0.00808	0.964393	6839380	7.113933	7.169142	6.992892	7.184057	7.390958	7.354669	7.148194	7.330998	6.821008	7.381953	7.064443	7.688774	Snn	NM_009223
6839420	5483	7.485509	7.552963	7.228196	7.496166	-0.01066	0.956471	0.056797	0.771728	-0.26797	0.194499	0.324767	0.124467	-0.06745	0.730724	0.257313	0.210999	6839420	7.421032	7.61175	7.423745	7.727611	7.41109	7.520188	6.896852	7.429935	7.3578	7.476564	7.20079	7.811145	Snx29	NM_028964
6839491	5484	7.287132	7.485613	7.352245	7.322899	-0.03577	0.651206	0.162714	0.065169	0.029346	0.710091	0.133368	0.118076	-0.19848	0.03134	-0.06511	0.417521	6839491	7.198128	7.254364	7.408905	7.475098	7.637836	7.343905	7.323125	7.365737	7.367873	7.297381	7.325961	7.345356	Ercc4	NM_015769
6839502	5485	8.055652	8.047345	8.020369	8.104369	-0.04872	0.455287	-0.05702	0.385264	-0.084	0.213091	0.026976	0.675435	0.008307	0.896875	0.035283	0.585459	6839502	8.098003	7.940516	8.128437	7.99637	8.095561	8.050104	7.958065	8.045238	8.057804	8.191306	8.10418	8.017621	Mkl2	NM_181860
6839504	5486	9.629058	9.609747	9.648133	9.552896	0.076163	0.305533	0.056851	0.437531	0.095238	0.208261	-0.03839	0.596221	0.019311	0.788395	-0.01907	0.790912	6839504	9.653299	9.630123	9.603753	9.625426	9.720608	9.483207	9.585727	9.731434	9.627239	9.456321	9.562099	9.640267	Mkl2	NM_153588
6839528	5487	7.283115	7.454116	7.347478	7.307784	-0.02467	0.795911	0.146332	0.151325	0.039694	0.678327	0.106638	0.281028	-0.171	0.100894	-0.06436	0.505115	6839528	7.334022	7.361671	7.153653	7.346628	7.654359	7.36136	7.344114	7.401516	7.296804	7.22316	7.343297	7.356894	Bfar	NM_025976
6839539	5488	8.217688	8.290454	8.432769	8.281984	-0.0643	0.579587	0.00847	0.94124	0.150785	0.212749	-0.14231	0.237111	-0.07277	0.531822	-0.21508	0.089538	6839539	8.225296	8.235626	8.192142	8.274716	8.339032	8.257615	8.494727	8.661003	8.142577	8.274007	8.329966	8.241979	Ntan1	NM_010946

6839543	5489	6.042487	5.997159	6.16894	6.665949	-0.62346	0.083552	-0.66879	0.066849	-0.49701	0.153836	-0.17178	0.60096	0.045329	0.889313	-0.12645	0.699059	6839543	5.931517	6.108303	6.087642	6.151779	6.051005	5.788692	6.091931	6.686923	5.727967	6.451892	6.240005	7.305951	Mpv17l	NM_033564
6839552	5490	7.84829	7.869136	7.849878	7.674081	0.17421	0.252641	0.195055	0.204824	0.175797	0.248695	0.019258	0.894964	-0.02085	0.886374	-0.00159	0.991313	6839552	7.858852	7.766557	7.919462	7.7672	8.20732	7.632888	7.953594	7.807948	7.788091	7.661829	7.803972	7.556441	Nde1	NM_023317
6839557	5491	5.952109	5.97323	5.970541	5.934312	0.017798	0.818997	0.038919	0.619052	0.036229	0.643124	0.002689	0.972369	-0.02112	0.786102	-0.01843	0.81269	6839557	5.839833	6.082922	5.933573	6.004333	5.977921	5.937437	6.092325	5.902642	5.916656	5.864296	5.914844	6.023795	2900011O	NM_144518
6839607	5492	8.525401	8.424954	8.356643	7.875344	0.650058	0.010106	0.54961	0.022119	0.481299	0.038167	0.068311	0.734035	0.100448	0.618885	0.168759	0.410035	6839607	8.671887	8.463333	8.440984	8.464807	8.480613	8.329441	8.568101	8.209343	8.292484	7.934083	8.252396	7.439552	Abcc1	NM_008576
6839624	5493	8.058602	7.842465	7.553452	7.718777	0.339826	0.257539	0.123689	0.669005	-0.16533	0.569511	0.289014	0.330152	0.216137	0.46043	0.505151	0.107536	6839624	7.935792	8.435443	7.804572	7.905443	8.175065	7.446888	7.369427	7.567205	7.723723	7.664111	8.177529	7.31469	Snai2	NM_011415
6839665	5495	6.706572	6.695931	6.674936	6.594727	0.111845	0.229047	0.101204	0.272493	0.080209	0.377658	0.020995	0.813024	0.010641	0.904446	0.031636	0.722153	6839665	6.812389	6.647646	6.659682	6.625579	6.783298	6.678916	6.741608	6.6273	6.6559	6.411338	6.661485	6.711358	Prkdc	NM_011159
6839681	5496	8.933304	9.009272	9.109516	8.990191	-0.05689	0.589588	0.019081	0.855202	0.119325	0.272426	-0.10024	0.351113	-0.07597	0.474532	-0.17621	0.119959	6839681	8.77125	8.863174	9.165488	8.924774	9.108147	8.994896	9.111	9.051404	9.166144	8.974195	8.915056	9.081322	Pkp2	NM_026163
6839685	5497	7.048873	7.030298	7.074347	6.997298	0.051576	0.53631	0.033	0.690178	0.07705	0.362709	-0.04405	0.59615	0.018576	0.821839	-0.02547	0.757813	6839685	7.062938	7.064799	7.018883	6.927434	7.224501	6.938958	7.091617	7.046216	7.085209	6.959322	7.1032	6.929371	Yars2	NM_198246
6839699	5498	8.666154	8.743994	8.713725	8.643775	0.022379	0.637074	0.100219	0.059382	0.06995	0.163915	0.030269	0.525847	-0.07784	0.126499	-0.04757	0.327753	6839699	8.662419	8.612219	8.723824	8.685607	8.849147	8.697229	8.731817	8.707952	8.701406	8.611481	8.656854	8.662991	Top3b	NM_011624
6839703	5499	7.010526	7.214	7.043123	6.987883	0.022643	0.783746	0.226117	0.022001	0.05524	0.508261	0.170877	0.064579	-0.20347	0.034145	-0.0326	0.69355	6839703	7.01118	7.008577	7.011821	7.239221	7.27169	7.131088	7.083845	7.080982	6.964541	6.797368	7.113266	7.053015	Ppm1f	NM_176833
6839714	5500	9.434504	9.548233	9.490838	9.547014	-0.11251	0.111857	0.00122	0.985024	-0.05618	0.398467	0.057395	0.388777	-0.11373	0.108596	-0.05633	0.397191	6839714	9.43652	9.426063	9.440928	9.497664	9.661518	9.485518	9.511235	9.541913	9.419367	9.463191	9.520046	9.657804	Mapk1	NM_001038663
6839718	5501	5.939193	6.13906	5.982778	5.965278	-0.02608	0.82282	0.173782	0.161747	0.017501	0.880475	0.156282	0.203057	-0.19987	0.114169	-0.04359	0.709108	6839718	5.745862	6.118093	5.953624	6.194346	6.213925	6.008909	6.029705	5.950222	5.968408	5.87451	5.866648	6.154675	Ypel1	NM_023249
6839733	5502	6.941257	6.861995	6.774425	6.974143	-0.03289	0.863492	-0.11215	0.56164	-0.19972	0.312352	0.08757	0.648999	0.079263	0.679987	0.166832	0.394046	6839733	6.649831	7.065052	7.108889	6.960492	6.487623	7.137869	6.619765	6.869833	6.833677	7.089522	6.937286	6.895621	Hic2	NM_178922
6839738	5504	8.272946	8.346295	8.356523	8.285357	-0.01241	0.895422	0.060938	0.523993	0.071166	0.458911	-0.01023	0.913714	-0.07335	0.445753	-0.08358	0.387548	6839738	8.312285	8.187586	8.318968	8.285252	8.445591	8.308041	8.268569	8.567943	8.233057	8.289138	8.341286	8.225646	Snap29	NM_023348
6839743	5505	8.524594	8.568694	8.441249	8.488655	0.035939	0.610953	0.080039	0.272329	-0.04741	0.504828	0.127445	0.097344	-0.0441	0.534198	0.083345	0.254514	6839743	8.46896	8.470308	8.634513	8.521839	8.659283	8.52496	8.411874	8.557945	8.353928	8.45189	8.526355	8.48772	Crkl	NM_007764
6839744	5506	6.136624	6.053211	6.077869	6.067817	0.068806	0.384617	-0.01461	0.850078	0.010052	0.896435	-0.02466	0.75017	0.083412	0.297245	0.058754	0.454876	6839744	6.119321	6.156101	6.134449	6.234002	5.970643	5.954989	6.055538	6.069477	6.108593	6.155229	5.977444	6.070779	Aifm3	NM_175178
6839746	5507	8.193521	8.20388	8.25159	8.112983	0.080538	0.403317	0.090897	0.348491	0.138607	0.16736	-0.04771	0.615347	-0.01036	0.912444	-0.05807	0.542427	6839746	8.123637	8.201021	8.255906	8.14836	8.334941	8.128338	8.427981	8.226472	8.100316	8.066234	8.196656	8.076059	Lztr1	NM_025808
6839751	5508	7.072506	6.969978	6.878136	6.631613	0.440893	0.007643	0.338365	0.026445	0.246523	0.083279	0.091842	0.482186	0.102528	0.434497	0.19437	0.157448	6839751	7.121571	7.219148	6.876798	7.073372	6.794353	7.042208	7.011216	6.719613	6.903579	6.536229	6.779953	6.578657	P2rx6	NM_011028
6839754	5509	7.689001	7.661839	7.701811	7.587899	0.101102	0.230481	0.073941	0.370291	0.113912	0.18178	-0.03997	0.621714	0.027161	0.73632	-0.01281	0.873451	6839754	7.686666	7.692086	7.68825	7.648276	7.764915	7.572327	7.701379	7.658603	7.745451	7.430655	7.585542	7.747499	Smpd4	NM_029945
6839769	5510	6.758355	6.865675	6.515152	6.793066	-0.03471	0.565631	0.072609	0.245451	-0.27791	0.00136	0.350522	0.000306	-0.10732	0.101076	0.243203	0.003004	6839769	6.719519	6.78025	6.775297	6.901521	6.939984	6.755519	6.532773	6.550563	6.462121	6.699989	6.810596	6.868612	Klhl22	NM_145479
6839771	5511	6.915171	6.924121	6.564621	6.988114	-0.07294	0.730431	-0.06399	0.762236	-0.42349	0.072024	0.3595	0.116666	-0.00895	0.966147	0.35055	0.124692	6839771	6.994702	6.813255	6.937556	6.867886	7.258369	6.646109	6.639401	6.712509	6.341953	6.700631	6.918576	7.345134	Scarf2	NM_153790
6839788	5515	7.571626	7.682654	7.697356	7.677021	-0.10539	0.053208	0.005633	0.906578	0.020336	0.673492	-0.0147	0.759998	-0.11103	0.044042	-0.12573	0.026931	6839788	7.511803	7.581758	7.621317	7.712355	7.712841	7.622766	7.670159	7.78853	7.63338	7.648058	7.694795	7.688209	Dgcr6	NM_010047
6839796	5516	7.017893	7.043579	7.083951	7.27552	-0.25763	0.241916	-0.23194	0.288168	-0.19157	0.374887	-0.04037	0.84796	-0.02569	0.902843	-0.06606	0.754226	6839796	6.916803	7.047764	7.089111	7.085151	6.973976	7.071611	7.391623	7.335748	6.524483	7.325455	7.23658	7.264524	Rtn4r	NM_022982
6839826	5517	7.464668	7.54058	7.615599	7.487335	-0.02267	0.719581	0.053245	0.407715	0.128264	0.068422	-0.07502	0.253259	-0.07591	0.248115	-0.15093	0.038303	6839826	7.442499	7.565503	7.386002	7.487471	7.634228	7.50004	7.679555	7.600425	7.566817	7.417552	7.506808	7.537644	Trmt2a	NM_008307
6839836	5518	6.672294	6.754149	6.665019	6.732093	-0.0598	0.42886	0.022056	0.766427	-0.06707	0.377311	0.08913	0.249407	-0.08185	0.287018	0.007275	0.921743	6839836	6.701725	6.655104	6.660054	6.738139	6.845313	6.678994	6.778071	6.711576	6.50541	6.774756	6.750242	6.67128	Arvcf	NM_033474
6839839	5519	6.104014	6.149975	6.102367	6.167011	-0.063	0.236383	-0.01704	0.738134	-0.06464	0.225414	0.047607	0.361689	-0.04596	0.377679	0.001647	0.974122	6839839	6.077358	6.155898	6.078787	6.154354	6.160436	6.135134	6.139202	6.151387	6.016513	6.132118	6.107913	6.261003	Txnrd2	NM_013711
6839847	5520	5.91269	5.929916	5.874014	5.912066	0.000625	0.996301	0.017851	0.894667	-0.03805	0.778203	0.055902	0.679933	-0.01723	0.898329	0.038676	0.774679	6839847	5.787905	5.966384	5.983782	5.921897	6.160292	5.70756	6.018635	5.903204	5.700203	6.042327	5.871321	5.822549	Gnb1l	NM_001081682
6839863	5521	6.211824	6.591678	5.671627	5.917759	0.294065	0.422434	0.673919	0.088687	-0.24613	0.49927	0.920051	0.029478	-0.37985	0.306595	0.540197	0.159013	6839863	6.330663	6.271234	6.033576	5.969327	7.16484	6.640867	6.111611	5.604707	5.298563	5.86389	5.530846	6.358542	Cldn5	NM_013805
6839864	5522	9.195883	9.286259	9.231013	9.164219	0.031664	0.683859	0.12204	0.142173	0.066794	0.398915	0.055246	0.482189	-0.09038	0.262413	-0.03513	0.651833	6839864	9.125991	9.182439	9.279218	9.261751	9.39929	9.197735	9.23384	9.124233	9.334966	9.167065	9.240194	9.085397	Ufd1l	NM_011672
6839871	5523	7.652039	7.619566	7.607747	7.559533	0.092507	0.326751	0.060033	0.516962	0.048215	0.600982	0.011819	0.897129	0.032473	0.723361	0.044292	0.63044	6839871	7.716053	7.586442	7.653623	7.637554	7.719909	7.501235	7.766679	7.601641	7.454922	7.469011	7.618404	7.591183	Hira	NM_010435
6839919	5526	9.439908	9.823673	9.46459	9.739511	-0.2996	0.041632	0.084162	0.515245	-0.27492	0.056852	0.359084	0.019756	-0.38377	0.014575	-0.02468	0.846744	6839919	9.346644	9.564226	9.408854	9.824923	9.66172	9.984377	9.552224	9.263434	9.578111	9.724689	9.597116	9.896728	Klhl24	NM_029436
6839928	5527	7.894675	7.972315	7.899131	7.884853	0.009822	0.900641	0.087462	0.284306	0.014279	0.856054	0.073184	0.365064	-0.07764	0.338163	-0.00446	0.954805	6839928	7.905252	7.872611	7.906161	7.905053	8.040087	7.971805	8.072996	7.87529	7.749108	7.819345	7.909974	7.925239	Eif2b5	NM_172265
6839930	5528	9.113995	9.021135	8.84866	9.120859	-0.00686	0.979149	-0.09972	0.705452	-0.2722	0.316106	0.172475	0.517146	0.092861	0.724705	0.265335	0.327699	6839930	9.13187	9.069285	9.140831	9.025575	9.201265	8.836564	8.442696	9.527947	8.575337	9.063821	9.181818	9.116937	Dvl3	NM_007889
6839932	5529	9.280485	9.289005	9.328903	9.353055	-0.07257	0.396733	-0.06405	0.452174	-0.02415	0.773306	-0.0399	0.635764	-0.00852	0.918867	-0.04842	0.566777	6839932	9.439793	9.298671	9.10299	9.328314	9.22256	9.316141	9.368072	9.267945	9.350692	9.400796	9.382936	9.275434	Ap2m1	NM_009679
6839934	5530	7.769586	7.809238	7.891314	7.900557	-0.13097	0.109778	-0.09132	0.245204	-0.00924	0.902126	-0.08208	0.292337	-0.03965	0.600912	-0.12173	0.133121	6839934	7.663577	7.809536	7.835646	7.732276	7.838385	7.857052	7.86792	7.916141	7.889881	7.745427	7.985374	7.970869	Abcf3	NM_013852
6839947	5533	6.378662	6.590642	6.710674	6.595417	-0.21676	0.093935	-0.00478	0.967636	0.115256	0.341877	-0.12003	0.323416	-0.21198	0.100182	-0.33201	0.01957	6839947	6.462219	6.278565	6.395202	6.3366	6.757309	6.678018	6.63455	6.764684	6.732787	6.681644	6.455969	6.648639	Ece2	NM_025462
6839952	5535	9.599362	9.658057	9.731292	9.502681	0.09668	0.435635	0.155376	0.223731	0.22861	0.088289	-0.07323	0.5515	-0.0587	0.631747	-0.13193	0.295286	6839952	9.679526	9.558627	9.559932	9.5495	9.781134	9.643538	9.966805	9.635664	9.591406	9.331913	9.61855	9.557581	Psmd2	NR_027485
6839956	5536	9.146954	9.132435	9.136653	9.036825	0.110129	0.389739	0.095611	0.4526	0.099829	0.433658	-0.00422	0.97307	0.014519	0.907532	0.010301	0.934309	6839956	9.227356	9.036651	9.176855	9.106219	9.273315	9.017772	9.163279	9.32523	8.921451	8.937485	9.199218	8.973771	Eif4g1	NM_145941
6839957	5537	6.37189	6.461673	6.339124	6.514739	-0.14285	0.092487	-0.05307	0.49808	-0.17562	0.046791	0.122549	0.139879	-0.08978	0.264225	0.032766	0.672846	6839957	6.406122	6.431034	6.278514	6.44237	6.426998	6.515651	6.355125	6.39634	6.265906	6.441474	6.424326	6.678418	Fam131a	BC092532
6839959	5538	6.558989	6.828938	6.730594	6.654608	-0.09562	0.732079	0.174331	0.536096	0.075986	0.785275	0.098345	0.724807	-0.26995	0.346136	-0.17161	0.542333	6839959	6.47746	6.692908	6.506598	6.934811	7.156849	6.395155	7.259883	6.665507	6.266391	6.494159	6.736488	6.733176	Polr2h	NM_145632
6839960	5539	6.215983	6.245995	6.182655	6.272249	-0.05627	0.392462	-0.02625	0.684306	-0.08959	0.188025	0.06334	0.338687	-0.03001	0.642632	0.033328	0.606933	6839960	6.265852	6.242807	6.139291	6.188822	6.216434	6.332729	6.279337	6.141951	6.126678	6.360102	6.232244	6.224401	Chrd	NM_009893
6839985	5540	7.635109	7.775458	7.455533	8.026136	-0.39103	0.128766	-0.25068	0.309191	-0.5706	0.038615	0.319925	0.203222	-0.14035	0.560095	0.179577	0.459045	6839985	7.593869	7.520156	7.791303	7.664771	7.94695	7.714653	7.730344	7.519682	7.116572	8.407414	7.568736	8.102257	Ephb3	NM_010143
6840004	5541	7.103229	7.124522	7.17966	6.927769	0.17546	0.091587	0.196753	0.06385	0.251891	0.024999	-0.05514	0.563649	-0.02129	0.821916	-0.07643	0.428001	6840004	7.086642	7.077438	7.145607	7.109646	7.260262	7.003658	7.355112	7.113677	7.070191	6.831845	7.018465	6.932996	Vps8	NM_001081366
6840019	5542	8.110732	8.166736	8.17898	8.102521	0.008211	0.940933	0.064215	0.566405	0.076459	0.496715	-0.01224	0.912036	-0.056	0.616141	-0.06825	0.542838	6840019	8.182719	8.016449	8.133028	8.099216	8.397327	8.003666	8.200941	8.278658	8.057342	8.01019	8.121314	8.17606	Senp2	NR_027488
6840052	5543	7.650587	7.754416	7.890018	7.625691	0.024896	0.917248	0.128725	0.594448	0.264327	0.287873	-0.1356	0.57529	-0.10383	0.666597	-0.23943	0.3326	6840052	7.741086	7.2619	7.948775	8.002718	7.828222	7.432308	8.013286	8.121914	7.534853	7.618384	7.50155	7.757138	Gng5	NM_010318
6840086	5544	8.980067	8.979033	9.265663	9.010111	-0.03004	0.893963	-0.03108	0.890343	0.255552	0.275548	-0.28663	0.225703	0.001033	0.99634	-0.2856	0.227229	6840086	9.052432	8.93624	8.951528	8.745191	9.116954	9.074955	9.359537	9.531967	8.905484	9.091557	9.331243	8.607533	Dnajb11	NM_026400
6840094	5546	7.446595	7.561732	7.031121	7.200132	0.246463	0.726028	0.3616	0.608823	-0.16901	0.809704	0.530611	0.457042	-0.11514	0.869562	0.415475	0.557602	6840094	7.538567	7.192763	7.608456	7.296371	8.28504	7.103784	7.089035	6.628344	7.375983	6.873627	8.810086	5.916683	Fetub	NM_021564
6840101	5548	6.029135	6.477324	5.821838	5.54078	0.488355	0.251909	0.936544	0.045373	0.281058	0.497461	0.655486	0.136005	-0.44819	0.289897	0.207297	0.614358	6840101	5.69436	5.732885	6.66016	5.937152	6.818029	6.676791	6.050707	5.276277	6.138531	5.829321	5.754157	5.038863	Kng1	NM_001102411
6840103	5549	9.066307	9.166275	9.279693	9.310286	-0.24398	0.151025	-0.14401	0.376115	-0.03059	0.847168	-0.11342	0.481567	-0.09997	0.533584	-0.21339	0.202397	6840103	8.983336	9.287546	8.928039	9.014688	9.130829	9.353308	9.527386	9.04597	9.265724	9.260486	9.214104	9.456268	Eif4a2	NM_001123038
6840111	5550	6.71443	6.578928	6.08626	6.567726	0.146704	0.706053	0.011202	0.976917	-0.48147	0.235412	0.492668	0.225642	0.135502	0.727389	0.62817	0.132679	6840111	6.034476	6.705646	7.403168	6.334071	6.7826	6.620114	6.402233	5.963152	5.893396	6.001003	6.940647	6.761529	Adipoq	NM_009605
6840115	5552	7.143888	7.309526	6.7986	7.828893	-0.685	0.256	-0.51937	0.380774	-1.03029	0.103041	0.510925	0.388189	-0.16564	0.774903	0.345288	0.554599	6840115	7.743727	6.879451	6.808486	7.727988	7.633468	6.567121	6.928806	7.19324	6.273755	7.80201	6.857542	8.827127	St6gal1	NM_145933
6840129	5554	7.102198	7.112249	7.304413	6.631812	0.470386	0.147683	0.480437	0.140292	0.672601	0.051138	-0.19216	0.53102	-0.01005	0.973521	-0.20222	0.510332	6840129	6.997713	7.189831	7.11905	6.913514	7.780964	6.642269	7.54599	6.88273	7.484519	6.49777	6.775515	6.622152	Rtp4	NM_023386
6840175	5555	10.10237	9.920519	9.820734	9.830413	0.27196	0.016216	0.090106	0.344256	-0.00968	0.916672	0.099786	0.29798	0.181854	0.077023	0.28164	0.013769	6840175	10.00995	10.15948	10.13769	9.820755	9.986805	9.953998	9.730012	9.816779	9.91541	9.758205	10.01295	9.720085	Lpp	NM_178665
6840199	5556	6.7377	6.260748	6.435732	6.668288	0.069412	0.758744	-0.40754	0.09899	-0.23256	0.318008	-0.17498	0.446122	0.476952	0.060485	0.301968	0.204116	6840199	6.555871	6.938215	6.719013	6.045255	6.512226	6.224763	6.868722	6.107135	6.331339	6.435493	6.778804	6.790567	Lpp	BC005613
6840223	5558	7.816987	7.844373	7.944238	7.705256	0.111731	0.605295	0.139117	0.521892	0.238982	0.283173	-0.09986	0.643562	-0.02739	0.898366	-0.12725	0.557151	6840223	7.937668	7.639178	7.874115	7.952294	8.187381	7.393445	7.962781	7.764027	8.105906	7.482431	7.850413	7.782924	Trp63	NM_001127259
6840261	5560	8.23467	8.131253	8.247083	8.161181	0.073489	0.789533	-0.02993	0.913272	0.085902	0.755242	-0.11583	0.675031	0.103417	0.707838	-0.01241	0.963958	6840261	8.081681	7.922411	8.699918	7.721787	8.509344	8.162627	8.574108	8.050571	8.11657	8.272243	8.004121	8.207178	Il1rap	NM_001159318
6840284	5562	11.74157	11.80845	11.79811	11.54142	0.200147	0.02487	0.267027	0.00626	0.256683	0.007697	0.010343	0.890308	-0.06688	0.384196	-0.05654	0.458848	6840284	11.80071	11.58686	11.83714	11.80457	11.84794	11.77284	11.84133	11.7085	11.84449	11.46176	11.5469	11.61561	Ccdc50	NM_026202
6840369	5564	8.6894	8.82037	8.781028	8.784987	-0.09559	0.386069	0.035383	0.743083	-0.00396	0.970642	0.039342	0.715749	-0.13097	0.244515	-0.09163	0.405134	6840369	8.702909	8.71776	8.64753	8.805372	8.825111	8.830626	8.861292	8.685963	8.795829	8.584398	8.725107	9.045456	Opa1	NM_133752
6840400	5565	9.375303	9.449163	9.156839	9.546418	-0.17111	0.326668	-0.09725	0.569047	-0.38958	0.04464	0.292324	0.11212	-0.07386	0.663988	0.218464	0.21897	6840400	9.170049	9.529824	9.426037	9.277106	9.615849	9.454535	9.161348	9.192371	9.116798	9.476608	9.276608	9.886039	Hes1	NM_008235
6840432	5566	7.93357	8.815351	7.87482	8.35917	-0.4256	0.107386	0.456181	0.087892	-0.48435	0.07298	0.940532	0.003913	-0.88178	0.005573	0.058751	0.808679	6840432	7.591372	8.008005	8.201334	9.056422	8.358907	9.030725	7.941606	7.779064	7.903789	8.660089	8.165341	8.252081	Fam43a	NM_177632
6840507	5568	9.825239	9.818237	9.712379	9.839415	-0.01418	0.893762	-0.02118	0.84197	-0.12704	0.251723	0.105857	0.33338	0.007003	0.947377	0.11286	0.30433	6840507	9.894827	9.747688	9.833203	9.880932	9.741643	9.832135	9.700876	9.840643	9.595619	9.769345	9.689529	10.05937	Dlg1	NM_007862

6840508	5569	7.097819	6.880826	6.836907	6.735408	0.362411	0.34568	0.145418	0.69817	0.101499	0.786117	0.043919	0.906346	0.216993	0.565141	0.260912	0.491217	6840508	7.212432	6.747503	7.333521	6.667428	7.231723	6.743326	7.676409	6.517385	6.316926	6.681747	6.528526	6.995951	Mfi2	NM_013900
6840516	5570	7.693315	7.166833	7.324077	7.305137	0.388178	0.26599	-0.1383	0.681287	0.01894	0.954901	-0.15724	0.64107	0.526481	0.143475	0.369238	0.288243	6840516	7.764321	7.689328	7.626295	7.230794	7.365712	6.903994	8.038943	7.119897	6.813391	7.770368	7.116828	7.028214	Pigz	NM_172822
6840517	5571	8.031326	7.900289	7.84846	7.984923	0.046403	0.758027	-0.08463	0.576922	-0.13646	0.375893	0.051829	0.730974	0.131037	0.394247	0.182866	0.244412	6840517	7.927944	8.275856	7.890178	7.659904	8.050191	7.990771	7.924542	7.886632	7.734205	7.807386	8.01133	8.136052	Ncbp2	NM_026554
6840527	5572	6.883306	7.01604	6.95099	6.919726	-0.03642	0.729601	0.096313	0.371479	0.031263	0.76645	0.06505	0.540412	-0.13273	0.228254	-0.06768	0.524558	6840527	6.862439	6.917943	6.869537	6.981614	7.045149	7.021356	7.032298	7.009702	6.810969	6.693741	6.94895	7.116488	1500031L	NM_025892
6840532	5573	6.497036	6.824424	6.551219	6.543332	-0.0463	0.814196	0.281092	0.178503	0.007887	0.968006	0.273205	0.189644	-0.32739	0.124181	-0.05418	0.783415	6840532	6.252719	6.664863	6.573526	6.557652	6.93521	6.98041	6.921986	6.470944	6.260728	6.563818	6.585656	6.480522	Wdr53	NM_026898
6840539	5575	8.136529	8.096177	8.019139	8.042067	0.094462	0.256694	0.05411	0.503967	-0.02299	0.774423	0.077038	0.348355	0.040351	0.615971	0.11739	0.167519	6840539	8.101406	8.070986	8.237194	7.93005	8.217351	8.141131	7.99923	8.033168	8.025019	7.995221	8.128289	8.002691	Rnf168	NM_027355
6840565	5577	6.929729	6.863816	6.914883	6.678597	0.251132	0.135783	0.18522	0.256032	0.236286	0.157253	-0.05107	0.744593	0.065912	0.674839	0.014846	0.924306	6840565	6.953659	6.859545	6.975982	6.933712	7.140022	6.517715	6.872425	7.041663	6.830561	6.536606	6.8247	6.674484	Pcyt1a	NM_009981
6840579	5578	7.980638	8.064863	8.272042	8.208305	-0.22767	0.131381	-0.14344	0.320623	0.063737	0.650589	-0.20718	0.164735	-0.08422	0.551457	-0.2914	0.063686	6840579	7.964249	8.081564	7.896102	8.023004	7.994668	8.176916	8.330854	8.404182	8.081091	7.960141	8.462121	8.202653	Tfrc	NM_011638
6840586	5579	7.899187	7.953366	7.89335	7.72009	0.179097	0.099637	0.233276	0.041514	0.173259	0.109348	0.060017	0.55004	-0.05418	0.588698	0.005838	0.953096	6840586	7.965568	7.844707	7.887287	8.01564	7.990888	7.853571	7.79025	8.044591	7.845208	7.590327	7.901569	7.668375	Tnk2	NM_016788
6840589	5580	8.589881	8.865637	8.393978	9.451378	-0.8615	0.052408	-0.58574	0.160358	-1.0574	0.023435	0.471659	0.248011	-0.27576	0.487092	0.195903	0.618788	6840589	7.746855	8.719624	9.303164	8.81799	8.914283	8.864638	8.841485	7.951745	8.388703	9.499327	9.228381	9.626425	Muc4	NM_080457
6840598	5581	8.034008	8.288934	8.134329	8.110839	-0.07683	0.366679	0.178095	0.057371	0.02349	0.777324	0.154605	0.090365	-0.25493	0.013102	-0.10032	0.246854	6840598	8.071072	8.051639	7.979312	8.169447	8.354765	8.34259	8.205376	8.132694	8.064917	7.953423	8.143877	8.235217	Fyttd1	NM_027226
6840603	5582	6.391566	6.583384	6.568443	6.4839	-0.09233	0.646705	0.099484	0.621801	0.084544	0.674382	0.014941	0.940479	-0.19182	0.351554	-0.17688	0.388367	6840603	6.533193	6.619013	6.022493	6.711255	6.527717	6.51118	6.860012	6.572344	6.272974	6.609842	6.31494	6.526917	Rpl35a	NM_021338
6840604	5583	6.752207	6.77977	6.74241	6.704037	0.048171	0.553507	0.075733	0.359406	0.038373	0.635516	0.03736	0.644335	-0.02756	0.732603	0.009798	0.90301	6840604	6.640681	6.793514	6.822427	6.819073	6.788659	6.731578	6.723216	6.725477	6.778536	6.598007	6.881486	6.632617	Lmln	NM_172823
6840612	5584	7.70928	7.770263	7.471781	7.558305	0.150975	0.053015	0.211958	0.012904	-0.08652	0.229782	0.298482	0.002043	-0.06098	0.386298	0.237499	0.007309	6840612	7.794538	7.584692	7.74861	7.710612	7.852213	7.747963	7.565676	7.465339	7.384328	7.583588	7.562665	7.528661	Osbpl11	NM_176840
6840613	5585	9.188251	9.290822	9.114739	9.121413	0.066838	0.513697	0.169409	0.121512	-0.00667	0.947274	0.176083	0.109514	-0.10257	0.324976	0.073512	0.47384	6840613	9.209635	9.095442	9.259675	9.225415	9.412661	9.234391	9.314186	8.979744	9.050287	9.082223	9.057747	9.224269	Snx4	NM_080557
6840618	5586	9.318386	9.435893	9.396749	9.533885	-0.2155	0.071851	-0.09799	0.373377	-0.13714	0.223536	0.039144	0.716249	-0.11751	0.290987	-0.07836	0.472482	6840618	9.344198	9.378015	9.232945	9.372824	9.444461	9.490395	9.483815	9.522072	9.18436	9.403627	9.506163	9.691864	Zfp148	NM_011749
6840623	5587	6.697355	6.687067	6.439222	6.27063	0.426725	0.042221	0.416437	0.046237	0.168592	0.36808	0.247845	0.198427	0.010288	0.955011	0.258133	0.182289	6840623	6.935835	6.508336	6.647893	6.639584	6.455936	6.96568	6.450058	6.407241	6.460367	6.304986	6.520999	5.985905	Slc12a8	NM_134251
6840629	5588	8.835208	8.754956	8.690228	8.476252	0.358957	0.205403	0.278704	0.315748	0.213976	0.435072	0.064728	0.80997	0.080253	0.765827	0.14498	0.592948	6840629	9.11939	8.995325	8.39091	9.043797	8.818101	8.402969	8.801775	8.672605	8.596304	8.305241	8.903276	8.220238	Heg1	NM_175256
6840631	5589	9.984429	10.09822	9.738945	9.52026	0.464169	0.108144	0.577963	0.054412	0.218685	0.418973	0.359278	0.199138	-0.11379	0.669253	0.245484	0.366855	6840631	10.26869	10.06053	9.624067	10.32082	10.17491	9.798938	10.0252	9.544237	9.647397	9.232368	9.961278	9.367133	Heg1	NM_175256
6840637	5591	10.01801	9.993243	10.03971	9.715903	0.302109	0.062209	0.27734	0.081979	0.323812	0.048801	-0.04647	0.747565	0.024769	0.863467	-0.0217	0.880206	6840637	10.04825	10.00786	9.997926	9.901027	10.20009	9.878612	10.33049	9.775524	10.01313	9.627829	9.781369	9.738511	Itgb5	NM_001145884
6840677	5593	5.923658	6.140938	6.081685	5.968492	-0.04483	0.707144	0.172447	0.172576	0.113193	0.354346	0.059254	0.620721	-0.21728	0.095847	-0.15803	0.20715	6840677	5.988475	5.909818	5.87268	6.130953	6.217158	6.074704	6.289578	5.871078	6.084398	5.806224	5.963854	6.135397	Ccdc14	NM_172824
6840694	5594	9.535638	9.568939	9.452658	9.269238	0.2664	0.059935	0.299701	0.039099	0.18342	0.170051	0.116281	0.367138	-0.0333	0.79122	0.08298	0.514431	6840694	9.580444	9.474556	9.551914	9.322331	9.765091	9.619395	9.379854	9.608737	9.369383	9.345633	9.341496	9.120584	Mylk	NM_139300
6840699	5595	12.25634	12.30827	12.03296	12.46124	-0.2049	0.393105	-0.15297	0.519397	-0.42828	0.095923	0.27531	0.25981	-0.05193	0.824807	0.223383	0.353921	6840699	12.45387	12.3336	11.98156	12.26236	11.97363	12.68882	12.11924	12.23211	11.74753	12.48389	12.21577	12.68406	Ptplb	NM_023587
6840705	5596	6.039728	6.043615	5.976673	6.059651	-0.01992	0.693738	-0.01604	0.750839	-0.08298	0.127418	0.066942	0.207294	-0.00389	0.938464	0.063055	0.232308	6840705	6.064119	5.972362	6.082704	6.102959	5.994571	6.033315	6.024473	6.019707	5.885839	6.062277	6.018619	6.098058	Adcy5	NM_001012765
6840725	5598	7.124521	7.358243	7.04282	7.159623	-0.0351	0.740074	0.198621	0.087847	-0.1168	0.286083	0.315424	0.014965	-0.23372	0.051492	0.081701	0.44708	6840725	7.11191	7.176327	7.085325	7.24067	7.53492	7.29914	7.011224	7.232199	6.885036	7.234923	7.089295	7.15465	Hspbap1	NM_175111
6840734	5599	7.922395	7.91018	7.830721	7.51195	0.410445	0.028578	0.39823	0.032324	0.318771	0.072246	0.079459	0.619847	0.012215	0.938729	0.091674	0.568122	6840734	7.829889	7.9307	8.006596	7.792099	8.134832	7.803608	7.992257	7.500092	7.999814	7.412534	7.646227	7.477089	Parp9	NM_030253
6840735	5600	7.896407	7.926684	8.075608	7.922466	-0.02606	0.819701	0.004218	0.970518	0.153142	0.203662	-0.14892	0.215154	-0.03028	0.791265	-0.1792	0.14393	6840735	7.845218	7.814781	8.029222	7.847013	8.148639	7.7844	8.124454	8.148184	7.954185	7.808857	8.013412	7.945128	Kpna1	NM_008465
6840737	5601	6.099653	6.374846	6.336	6.141088	-0.04144	0.832268	0.233758	0.252051	0.194912	0.333434	0.038846	0.84258	-0.27519	0.184211	-0.23635	0.247264	6840737	6.029225	6.216686	6.053047	6.176268	6.288971	6.659298	6.667504	6.37077	5.969725	5.987844	6.192877	6.242543	Wdr5b	NM_027113
6840766	5603	9.775154	9.403363	9.759956	9.33802	0.437134	0.056501	0.065343	0.747699	0.421936	0.063753	-0.35659	0.106703	0.371791	0.09474	0.015198	0.940166	6840766	9.801463	9.3642	10.1598	9.474281	9.487898	9.24791	10.00254	9.570127	9.707201	9.394356	9.256123	9.363582	Ildr1	NM_134109
6840775	5604	6.705927	6.838906	6.750005	6.839742	-0.13381	0.181486	-0.00084	0.992929	-0.08974	0.355128	0.088901	0.359387	-0.13298	0.183932	-0.04408	0.642673	6840775	6.607694	6.76583	6.744258	6.776945	6.906281	6.833492	6.842733	6.633022	6.77426	6.717392	6.774655	7.027179	Iqcb1	NM_177128
6840778	5605	7.687456	7.655888	7.740837	7.190916	0.49654	0.055592	0.464972	0.069391	0.549921	0.03819	-0.08495	0.71177	0.031568	0.89037	-0.05338	0.815911	6840778	8.035279	7.662236	7.364853	7.593519	7.792681	7.581463	7.957787	7.313048	7.951676	7.035932	7.383035	7.153781	Hcls1	NM_008225
6840805	5607	7.022295	7.031231	7.167648	7.057405	-0.03511	0.777409	-0.02617	0.832892	0.110243	0.385386	-0.13642	0.288784	-0.00894	0.942501	-0.14535	0.260611	6840805	7.069152	7.008507	6.989625	7.058486	7.252025	6.783181	7.260695	7.247612	6.994638	6.971266	7.065915	7.135035	Rabl3	NM_001042499
6840818	5609	9.717788	9.893361	9.272684	8.596667	1.121121	0.06882	1.296694	0.041193	0.676017	0.240818	0.620677	0.278262	-0.17557	0.750572	0.445104	0.428388	6840818	9.889527	9.94518	9.318656	9.630871	10.4348	9.614412	9.907029	8.546844	9.364178	7.909391	9.678574	8.202036	Fstl1	NM_008047
6840819	5610	8.937099	9.080075	8.909984	8.906802	0.030297	0.768297	0.173273	0.119463	0.003182	0.975247	0.170091	0.125411	-0.14298	0.188269	0.027115	0.791926	6840819	9.025779	8.942961	8.842558	8.974807	9.192808	9.072611	8.82635	8.95694	8.946663	8.996902	9.027824	8.695681	Lrrc58	NM_177093
6840828	5612	10.19113	10.24114	10.07953	10.11502	0.076108	0.330536	0.126122	0.124375	-0.03549	0.642044	0.161607	0.059027	-0.05001	0.515254	0.111593	0.167269	6840828	10.25068	10.20655	10.11615	10.16663	10.33797	10.21882	10.03007	10.17547	10.03306	9.983745	10.16878	10.19253	Gsk3b	NM_019827
6840837	5613	9.122172	9.153513	9.096497	9.279568	-0.1574	0.221488	-0.12606	0.319299	-0.18307	0.161608	0.057016	0.643877	-0.03134	0.798446	0.025675	0.83418	6840837	8.995856	9.202815	9.167845	9.141934	9.077882	9.240723	9.208502	8.996885	9.084103	9.134737	9.155746	9.548222	Cox17	NM_001017429
6840849	5615	6.209183	5.617125	5.401233	4.9633	1.245884	0.010179	0.653825	0.11741	0.437933	0.273695	0.215892	0.578289	0.592058	0.150761	0.807951	0.061993	6840849	6.924971	6.068278	5.634301	5.592828	6.163343	5.095204	5.12862	5.309859	5.765219	4.899293	5.069345	4.921261	Setd8	NM_030241
6840860	5616	8.506622	8.510892	8.642961	8.751167	-0.24454	0.29148	-0.24027	0.299404	-0.10821	0.630735	-0.13207	0.558844	-0.00427	0.984749	-0.13634	0.546494	6840860	8.447009	8.511025	8.561831	8.333653	8.764138	8.434885	8.372449	9.125209	8.431225	8.855822	8.908272	8.489406	Tmem39a	NM_026407
6840877	5617	8.137589	8.133642	8.076853	8.427739	-0.29015	0.224558	-0.2941	0.218889	-0.35089	0.150107	0.056789	0.803207	0.003947	0.986153	0.060736	0.7899	6840877	7.810995	8.319975	8.281796	7.92489	8.290421	8.185614	8.284709	7.61141	8.334439	8.384426	8.555569	8.343223	B4galt4	NM_019804
6840887	5618	6.905972	7.23	6.840173	6.848025	0.057947	0.632546	0.381975	0.011251	-0.00785	0.94796	0.389827	0.010177	-0.32403	0.023953	0.065799	0.587977	6840887	7.054685	6.818087	6.845143	7.142979	7.410666	7.136354	6.930916	6.968521	6.621082	6.920465	6.809042	6.814567	Igsf11	NM_170599
6841019	5621	9.990689	9.905265	9.659088	10.04852	-0.05783	0.575538	-0.14325	0.186243	-0.38943	0.004354	0.246177	0.03784	0.085424	0.413698	0.331601	0.010129	6841019	9.98918	10.06786	9.915026	9.90445	9.854199	9.957145	9.431928	9.795972	9.749363	10.02498	9.956593	10.16398	Zbtb20	NM_019778
6841097	5623	8.818197	8.86005	8.785631	8.790633	0.027563	0.838727	0.069416	0.610829	-0.005	0.970495	0.074419	0.585835	-0.04185	0.757711	0.032566	0.810073	6841097	8.788865	8.810401	8.855324	8.822442	8.964616	8.793091	8.984053	8.745473	8.627366	8.509268	8.897243	8.965389	Nat13	NM_028108
6841110	5624	6.534711	6.681722	6.648359	6.630505	-0.09579	0.319655	0.051217	0.586078	0.017854	0.84817	0.033363	0.721317	-0.14701	0.142106	-0.11365	0.243597	6841110	6.413178	6.60153	6.589425	6.611915	6.894368	6.538884	6.664179	6.593184	6.687714	6.642361	6.628893	6.620261	Ccdc52	NM_144550
6841132	5625	6.763116	6.897491	6.805043	6.702509	0.060607	0.518175	0.194983	0.061396	0.102534	0.285917	0.092448	0.332717	-0.13438	0.172378	-0.04193	0.652571	6841132	6.823177	6.731245	6.734926	6.705946	7.086697	6.899831	6.855231	6.833481	6.726417	6.650203	6.778499	6.678824	BC027231	BC027231
6841160	5630	7.759746	7.469706	6.686678	6.458383	1.301363	0.012648	1.011323	0.037796	0.228295	0.590136	0.783027	0.090513	0.29004	0.496245	1.073068	0.029849	6841160	8.004157	7.960674	7.314407	7.18198	8.19469	7.032447	7.238243	6.283748	6.538044	5.983746	6.872276	6.519127	Ccdc80	NM_026439
6841168	5631	8.097596	7.993588	8.003014	8.122225	-0.02463	0.811601	-0.12864	0.234153	-0.11921	0.267237	-0.00943	0.927193	0.104009	0.328566	0.094582	0.371784	6841168	8.066308	8.228476	7.998005	7.856018	8.079453	8.045292	8.018504	8.040631	7.949908	7.927365	8.192321	8.24699	Atg3	NM_026402
6841362	5638	5.14685	5.993866	5.416159	4.773847	0.373003	0.760757	1.220019	0.332881	0.642312	0.602201	0.577707	0.638638	-0.84702	0.494649	-0.26931	0.825745	6841362	4.554852	3.555422	7.330275	5.835777	7.388976	4.756845	7.062276	4.240239	4.945963	3.931468	5.57373	4.816343	Retnla	NM_020509
6841399	5641	6.926976	7.002042	6.8499	6.862522	0.064454	0.587659	0.13952	0.256239	-0.01262	0.914645	0.152142	0.219144	-0.07507	0.529108	0.077077	0.518421	6841399	6.964368	6.982285	6.834276	7.081652	7.052714	6.87176	6.987088	6.762545	6.800066	6.649035	6.869218	7.069313	Ift57	NM_028680
6841410	5642	10.69666	10.50702	10.58094	10.46675	0.22991	0.036995	0.040277	0.67311	0.114197	0.249656	-0.07392	0.444902	0.189633	0.073214	0.115713	0.24394	6841410	10.73302	10.73718	10.61977	10.40345	10.6124	10.50522	10.52674	10.63723	10.57886	10.50935	10.62154	10.26935	Cd47	NM_010581
6841701	5643	7.033066	7.114926	7.244441	7.113835	-0.08077	0.206632	0.001091	0.98564	0.130607	0.056986	-0.12952	0.05866	-0.08186	0.201163	-0.21138	0.007018	6841701	6.955625	7.122565	7.021007	7.077877	7.0932	7.173701	7.250215	7.259609	7.2235	7.018739	7.0992	7.223565	Senp7	NM_025483
6841712	5645	8.564054	8.28048	7.673742	7.365522	1.198532	0.035338	0.914958	0.089685	0.30822	0.53376	0.606738	0.236408	0.283574	0.566223	0.890312	0.097165	6841712	8.737047	8.827406	8.127709	8.354436	9.006885	7.480119	8.22886	7.234652	7.557715	6.728628	7.918439	7.4495	Abi3bp	NM_001014423
6841739	5646	8.475168	8.54934	8.499739	8.413987	0.061181	0.601431	0.135354	0.263378	0.085752	0.467852	0.049601	0.671	-0.07417	0.528271	-0.02457	0.8326	6841739	8.535205	8.463632	8.426666	8.475143	8.70314	8.469738	8.65093	8.447606	8.400681	8.191018	8.511857	8.539085	Tomm70a	NM_138599
6841849	5647	7.613069	7.598228	7.489346	7.47295	0.140119	0.353167	0.125278	0.403873	0.016396	0.91102	0.108882	0.465715	0.014841	0.919423	0.123723	0.409476	6841849	7.756853	7.588008	7.494345	7.666644	7.783774	7.344265	7.558522	7.607829	7.301687	7.439343	7.644295	7.335212	Dcbld2	NM_028523
6841862	5648	7.661614	7.731826	7.745575	7.871613	-0.21	0.096791	-0.13979	0.245954	-0.12604	0.291692	-0.01375	0.905033	-0.07021	0.546994	-0.08396	0.473613	6841862	7.552677	7.643768	7.788397	7.664545	7.811211	7.719722	7.623517	7.926471	7.686738	7.904082	8.025238	7.685518	Cpox	NM_007757
6841865	5649	9.775543	9.565725	9.950763	9.805225	-0.02968	0.840261	-0.2395	0.131451	0.145539	0.337156	-0.38504	0.02703	0.209818	0.179269	-0.17522	0.253933	6841865	9.562296	9.764112	10.00022	9.366149	9.851606	9.479419	9.940024	9.99815	9.914116	9.748081	9.905067	9.762526	Cldnd1	NM_171826
6841882	5653	9.346447	9.098642	9.350211	9.094351	0.252095	0.100472	0.00429	0.975569	0.25586	0.09628	-0.25157	0.10107	0.247805	0.10546	-0.00376	0.978562	6841882	9.40304	9.234609	9.401691	9.027086	9.130628	9.138211	9.502603	9.201054	9.346977	8.804349	9.346981	9.131724	Mina	NM_025910
6842009	5655	8.71132	8.833373	8.612204	8.304576	0.406744	0.150612	0.528797	0.072627	0.307627	0.263674	0.22117	0.412587	-0.12205	0.646143	0.099116	0.708611	6842009	8.789209	8.728555	8.616196	8.737549	9.096028	8.666543	9.07457	8.282557	8.479484	7.85005	8.616742	8.446937	Pros1	NM_011173
6842085	5656	7.687656	7.827476	7.719432	7.715797	-0.02814	0.864022	0.111679	0.502685	0.003635	0.982336	0.108045	0.5163	-0.13982	0.405189	-0.03178	0.846705	6842085	7.69321	7.70261	7.667148	7.668161	8.002892	7.811376	7.813593	7.997624	7.347078	7.596921	7.757089	7.793381	Cggbp1	NM_178647
6842273	5658	7.799734	7.751495	7.767409	8.022771	-0.22304	0.174394	-0.27128	0.107392	-0.25536	0.126272	-0.01591	0.917915	0.048239	0.755414	0.032325	0.834364	6842273	7.753509	7.762566	7.883128	7.8518	7.853324	7.549361	8.012168	7.754064	7.535996	7.905079	7.904869	8.258364	Gbe1	NM_028803
6842326	5659	6.061423	6.788696	5.843676	5.984384	0.077039	0.828164	0.804311	0.047302	-0.14071	0.692823	0.94502	0.025014	-0.72727	0.067111	0.217747	0.543834	6842326	6.346768	5.960023	5.877478	6.177377	7.592819	6.595891	5.809646	5.917791	5.80359	5.915807	6.348476	5.68887	Robo1	NM_019413
6842503	5661	9.832421	9.763582	9.969986	10.07125	-0.23883	0.006783	-0.30767	0.001616	-0.10127	0.163359	-0.2064	0.014048	0.068839	0.32727	-0.13757	0.070557	6842503	9.748342	9.839485	9.909435	9.764235	9.780677	9.745834	10.03652	10.00432	9.869117	9.963945	10.17746	10.07235	Usp25	NM_013918

6842552	5662	7.948016	7.878783	8.003888	7.703704	0.244312	0.068681	0.175079	0.170334	0.300184	0.032455	-0.12511	0.31306	0.069234	0.567791	-0.05587	0.643533	6842552	7.778091	7.98993	8.076028	7.704434	8.01993	7.911984	8.141315	7.912859	7.957491	7.779257	7.779329	7.552526	Cxadr	NM_001025192
6842800	5665	9.637368	9.578999	9.362278	9.297164	0.340204	0.230783	0.281834	0.313945	0.065113	0.810202	0.216721	0.432619	0.058369	0.829478	0.27509	0.324934	6842800	9.730508	9.714528	9.467068	9.701277	9.589955	9.445764	9.915933	8.831177	9.339723	9.01468	9.580444	9.296369	Jam2	NM_023844
6842803	5666	8.486497	8.628501	8.59819	8.584752	-0.09825	0.138567	0.043749	0.484765	0.013438	0.827622	0.030311	0.625491	-0.142	0.044712	-0.11169	0.098391	6842803	8.392847	8.5384	8.528245	8.58428	8.661822	8.6394	8.668885	8.578197	8.547487	8.477993	8.664102	8.61216	Gabpa	NM_008065
6842926	5667	6.712711	6.578098	6.845041	6.578114	0.134597	0.308246	-1.6E-05	0.9999	0.266927	0.063	-0.26694	0.062987	0.134613	0.308192	-0.13233	0.315938	6842926	6.680856	6.728825	6.728825	6.505064	6.724238	6.504992	6.825099	6.988177	6.721848	6.308349	6.663793	6.7622	N6amt1	NM_026366
6842933	5668	8.403299	8.359713	8.477409	8.340866	0.062433	0.609771	0.018847	0.876595	0.136543	0.2789	-0.1177	0.346039	0.043585	0.720432	-0.07411	0.545986	6842933	8.459828	8.388443	8.361625	8.325089	8.528566	8.225485	8.484766	8.619738	8.327724	8.141247	8.512975	8.368376	Usp16	NM_024258
6842940	5670	8.21103	8.367806	8.253549	8.469876	-0.25885	0.016389	-0.10207	0.266847	-0.21633	0.035278	0.114257	0.218287	-0.15678	0.104118	-0.04252	0.632431	6842940	8.181792	8.25528	8.196019	8.365165	8.278663	8.45959	8.142007	8.390601	8.228039	8.535028	8.314618	8.559981	Bach1	NM_007520
6843099	5677	7.945337	7.774563	7.610277	7.540085	0.405252	0.100072	0.234478	0.313447	0.070192	0.755704	0.164286	0.472651	0.170774	0.455945	0.33506	0.162829	6843099	7.688651	7.734079	8.41328	7.901489	7.953131	7.469068	7.829272	7.402234	7.599324	7.525701	7.485121	7.609433	Hunk	NM_015755
6843130	5678	6.066242	5.867166	5.764662	5.887542	0.1787	0.469071	-0.02038	0.933074	-0.12288	0.615422	0.102504	0.674413	0.199076	0.421803	0.30158	0.235558	6843130	6.059252	5.56958	6.569893	5.997554	5.824924	5.779019	5.883887	5.902204	5.507895	5.802795	6.045474	5.814357	Mrap	NM_029844
6843133	5679	7.090089	6.956142	7.011285	6.862317	0.227772	0.003793	0.093825	0.135558	0.148968	0.029939	-0.05514	0.357914	0.133947	0.045288	0.078804	0.200823	6843133	7.012581	7.149094	7.108593	6.991305	6.928402	6.94872	7.11437	6.967824	6.951662	6.941697	6.844254	6.801	4931408A	BC132305
6843170	5680	6.473714	6.614985	6.560584	6.520056	-0.04634	0.711516	0.094929	0.455032	0.040528	0.746118	0.054401	0.664723	-0.14127	0.276313	-0.08687	0.492944	6843170	6.500936	6.354281	6.565924	6.630323	6.410403	6.80423	6.553355	6.691265	6.437132	6.524428	6.373568	6.662172	Olig2	NM_016967
6843171	5681	6.501062	6.49681	6.396838	6.487344	0.013717	0.892491	0.009466	0.925669	-0.09051	0.38422	0.099973	0.339013	0.004251	0.966571	0.104224	0.320082	6843171	6.439494	6.476024	6.587667	6.450078	6.452686	6.587667	6.475224	6.551307	6.163982	6.433769	6.474325	6.553939	Olig1	NM_016968
6843177	5682	10.11166	9.996328	9.957147	9.709295	0.402368	0.010819	0.287033	0.046258	0.247852	0.076352	0.039181	0.756011	0.115335	0.371548	0.154516	0.240389	6843177	10.25578	9.982788	10.09642	9.798314	10.08751	10.10316	10.13848	9.769951	9.963009	9.65192	9.803916	9.672049	Ifnar2	NM_010509
6843178	5683	10.60688	10.63998	10.69343	10.51026	0.096613	0.158137	0.12972	0.069992	0.18317	0.018384	-0.05345	0.41416	-0.03311	0.608212	-0.08656	0.200606	6843178	10.59542	10.65045	10.57476	10.5208	10.77897	10.62018	10.72582	10.71515	10.63933	10.47601	10.48844	10.56634	Il10rb	NM_008349
6843179	5684	8.914936	8.863689	8.915049	8.516937	0.397999	0.042165	0.346752	0.068487	0.398112	0.04212	-0.05136	0.763259	0.051247	0.763759	-0.00011	0.999471	6843179	9.036522	8.915513	8.792774	8.731391	9.202923	8.656753	8.960799	8.781579	9.002769	8.831961	8.766138	8.393013	Ifnar1	NM_010508
6843184	5685	9.916496	9.858163	9.820724	9.069815	0.846681	0.000154	0.788348	0.000251	0.750909	0.000348	0.037439	0.77486	0.058333	0.657069	0.095772	0.470823	6843184	10.00102	9.875806	9.872663	9.834496	9.984409	9.755584	9.93323	9.687118	9.841825	8.918911	9.35655	8.933985	Ifngr2	NM_008338
6843194	5686	9.459333	9.478509	9.462058	9.45297	0.006363	0.919982	0.025539	0.688278	0.009088	0.885952	0.016451	0.79546	-0.01918	0.762706	-0.00272	0.965675	6843194	9.49982	9.526207	9.351972	9.406953	9.557742	9.470831	9.438441	9.491596	9.456137	9.354256	9.506108	9.498546	Son	NM_178880
6843198	5687	7.101531	7.007314	6.965824	6.915917	0.185614	0.034273	0.091397	0.244996	0.049907	0.512619	0.04149	0.584595	0.094217	0.231956	0.135707	0.099469	6843198	7.062165	7.053458	7.188971	7.014887	7.108322	6.898733	7.031074	6.937167	6.929231	6.824256	7.036233	6.887262	Itsn1	NM_010587
6843207	5688	10.49932	10.51387	10.3973	10.58259	-0.08327	0.577215	-0.06872	0.644398	-0.18529	0.232114	0.11657	0.43951	-0.01455	0.921646	0.102023	0.496748	6843207	10.4452	10.40572	10.64705	10.66449	10.35774	10.51938	10.47737	10.44569	10.26884	10.50545	10.36459	10.87773	Mrps6	NM_080456
6843208	5689	10.99313	10.83271	10.81461	11.0779	-0.08476	0.606352	-0.24519	0.159443	-0.26328	0.13433	0.018097	0.911668	0.160423	0.339834	0.17852	0.291432	6843208	10.96843	10.99142	11.01955	11.01538	10.5377	10.94505	10.64635	10.97872	10.81877	11.22232	10.80705	11.20432	Slc5a3	NM_017391
6843222	5691	8.411918	8.532509	8.581967	8.581897	-0.16998	0.142622	-0.04939	0.649251	7E-05	0.999482	-0.04946	0.648793	-0.12059	0.282003	-0.17005	0.142479	6843222	8.298743	8.485394	8.451618	8.49396	8.581048	8.52252	8.586122	8.795341	8.364439	8.544258	8.521853	8.679581	1190017O	NM_138743
6843335	5693	8.048152	8.084578	8.090601	7.922784	0.125368	0.119808	0.161794	0.054803	0.167817	0.048095	-0.00602	0.935385	-0.03643	0.626543	-0.04245	0.571732	6843335	8.059858	8.022171	8.062427	8.068731	8.137338	8.047665	8.06179	8.221468	7.988545	7.923453	7.803251	8.041649	Cbr1	NM_007620
6843338	5694	7.257579	7.065016	6.667848	6.172821	1.084758	0.016007	0.892195	0.036813	0.495027	0.202465	0.397168	0.297669	0.192564	0.603858	0.589731	0.136734	6843338	7.589366	6.771258	7.412114	7.035193	7.304142	6.855712	7.125459	6.10716	6.770925	6.15738	6.689702	5.671381	Cbr3	NM_173047
6843340	5695	8.218287	7.929648	7.93094	7.78103	0.437257	0.018784	0.148618	0.34733	0.14991	0.343388	-0.00129	0.993284	0.288639	0.08848	0.287347	0.089674	6843340	7.956418	8.13806	8.560383	7.957351	7.983199	7.848393	7.968467	7.889668	7.934686	7.873371	7.889311	7.580408	Dopey2	NM_027293
6843343	5696	6.457177	6.539865	6.329864	5.695168	0.762009	0.027854	0.844697	0.017799	0.634695	0.05598	0.210002	0.481003	-0.08269	0.778457	0.127314	0.666008	6843343	6.392619	6.274766	6.704147	6.388445	6.846909	6.384242	6.82496	5.715333	6.449298	5.463521	5.886991	5.734993	Chaf1b	NM_028083
6843378	5698	6.938843	7.04742	7.00455	7.070457	-0.13161	0.090096	-0.02304	0.744538	-0.06591	0.362757	0.042869	0.547658	-0.10858	0.15051	-0.06571	0.364132	6843378	6.980375	6.944218	6.891936	7.066269	7.053144	7.022846	6.896622	7.095313	7.021716	6.988504	7.213315	7.009551	Ripply3	NM_133229
6843384	5699	8.880787	8.959744	8.941901	8.943035	-0.06225	0.438143	0.016709	0.83212	-0.00113	0.988508	0.017842	0.820953	-0.07896	0.33095	-0.06111	0.446214	6843384	8.786951	9.010059	8.84535	8.869158	9.024866	8.985207	8.945727	8.922072	8.957905	8.837634	8.916223	9.075248	Ttc3	NM_009441
6843393	5700	9.927732	9.996127	9.987586	10.07953	-0.1518	0.147416	-0.08341	0.403898	-0.09195	0.359772	0.008541	0.930313	-0.0684	0.490491	-0.05985	0.544861	6843393	9.883062	9.913445	9.986688	9.995215	10.09283	9.900336	9.825226	10.18524	9.952291	9.995337	10.06691	10.17635	Dyrk1a	NM_007890
6843419	5701	9.995987	9.971153	9.983964	10.28063	-0.28464	0.19848	-0.30948	0.165913	-0.29667	0.182067	-0.01281	0.951231	0.024834	0.905657	0.012023	0.954228	6843419	9.863828	9.988943	10.13519	10.06594	10.0025	9.845018	9.546702	10.36833	10.03686	10.39699	10.03568	10.40922	Kcnj15	NM_019664
6843433	5702	9.872317	10.0709	10.09057	10.09406	-0.22174	0.039466	-0.02315	0.80395	-0.00349	0.970093	-0.01966	0.832934	-0.19859	0.058904	-0.21825	0.041921	6843433	9.890765	9.926561	9.799626	9.848469	10.1811	10.18314	10.17044	10.06229	10.03897	10.12205	10.03597	10.12415	Ets2	NM_011809
6843487	5703	8.10889	8.184144	8.215269	8.009249	0.099641	0.320929	0.174896	0.100367	0.206021	0.060139	-0.03113	0.749507	-0.07525	0.447303	-0.10638	0.29137	6843487	8.02417	8.067364	8.235135	8.036167	8.342446	8.17382	8.283143	8.076608	8.286057	7.986593	8.070108	7.971045	Wrb	NM_207301
6843494	5705	10.42107	10.10635	10.15874	10.13303	0.28804	0.094585	-0.02668	0.864991	0.025707	0.869844	-0.05238	0.739157	0.314717	0.072081	0.262333	0.122521	6843494	10.64062	10.36506	10.25753	10.08564	10.18678	10.04664	10.32246	10.26385	9.889902	10.35422	9.9726	10.07227	Igsf5	NM_028078
6843500	5706	7.626204	7.662925	7.367981	7.664232	-0.03803	0.720496	-0.00131	0.990147	-0.29625	0.020269	0.294943	0.020669	-0.03672	0.729646	0.258222	0.035973	6843500	7.508031	7.57247	7.79811	7.814669	7.512419	7.661686	7.500118	7.25765	7.346176	7.670247	7.616243	7.706205	Pcp4	NM_008791
6843543	5707	7.969636	8.654754	8.128363	9.618458	-1.64882	0.000274	-0.9637	0.007041	-1.4901	0.000536	0.526391	0.085233	-0.68512	0.033899	-0.15873	0.570209	6843543	8.095862	8.099375	7.713672	8.673927	8.442189	8.848147	7.990104	8.268515	8.12647	9.010236	9.713038	10.1321	Bace2	NM_019517
6843614	5710	6.256673	6.323688	6.216042	6.332313	-0.07564	0.452837	-0.00862	0.930524	-0.11627	0.259768	0.107647	0.294038	-0.06702	0.504322	0.040632	0.682841	6843614	6.215707	6.262395	6.291918	6.474306	6.237429	6.25933	6.159697	6.426764	6.061664	6.346781	6.29929	6.350868	1700037C	NM_028484
6843624	5711	7.212242	7.337898	7.205425	7.331425	-0.11916	0.387141	0.006499	0.961436	-0.12597	0.361902	0.132473	0.339008	-0.12566	0.363051	0.006817	0.959551	6843624	7.160025	7.163764	7.312937	7.342834	7.390581	7.280278	7.095724	7.399218	7.121332	7.498397	7.043137	7.452661	Btbd12	NM_177472
6843629	5712	8.709354	8.908811	8.847226	9.022522	-0.31317	0.054327	-0.11371	0.437041	-0.1753	0.242831	0.061585	0.669486	-0.19946	0.189252	-0.13787	0.350341	6843629	8.59481	8.720051	8.8132	8.905299	8.806153	9.01498	8.919676	8.77801	8.843991	8.825243	8.878654	9.36367	Trap1	NM_026508
6843634	5713	7.214438	7.154353	7.067261	7.323128	-0.10869	0.325553	-0.16878	0.142536	-0.25587	0.038981	0.087092	0.425713	0.060085	0.57854	0.147177	0.193882	6843634	7.115286	7.300901	7.227126	7.115047	7.16435	7.183662	7.156731	7.188974	6.856077	7.218627	7.488225	7.262533	Adcy9	NM_009624
6843642	5714	6.443182	6.482542	6.033023	6.153012	0.290169	0.776622	0.329529	0.747481	-0.11999	0.906399	0.449519	0.661442	-0.03936	0.969221	0.410159	0.689159	6843642	5.197453	5.731515	8.400577	7.663452	6.200192	5.583981	7.077325	5.780737	5.241007	5.07884	6.843195	6.537002	Srl	NM_175347
6843643	5715	6.294716	6.261212	6.366131	6.274011	0.020706	0.893495	-0.0128	0.934021	0.09212	0.55572	-0.10492	0.503594	0.033504	0.82865	-0.07141	0.646346	6843643	6.144404	6.441513	6.298232	6.483555	6.3749	5.925181	6.390658	6.271367	6.436368	6.227193	6.424142	6.170697	Tcfap4	NM_031182
6843653	5716	7.809804	7.709357	7.612568	7.456786	0.353018	0.032578	0.252571	0.102035	0.155782	0.287719	0.096789	0.499297	0.100447	0.483719	0.197236	0.187308	6843653	7.992394	7.692935	7.744083	7.90239	7.817191	7.40849	7.549566	7.628089	7.660048	7.32733	7.55472	7.488307	Coro7	NM_030205
6843657	5717	6.177984	6.622255	6.355564	6.327691	-0.14971	0.310449	0.294564	0.065739	0.027873	0.845261	0.266691	0.089869	-0.44427	0.012362	-0.17758	0.234943	6843657	6.164636	6.019116	6.3502	6.34933	6.880143	6.637292	6.270523	6.401142	6.395027	6.241426	6.445189	6.296458	Nmral1	NM_026393
6843663	5718	8.746972	8.804575	8.747759	9.073639	-0.32667	0.073215	-0.26906	0.127978	-0.32588	0.073781	0.056816	0.729237	-0.0576	0.725665	-0.00079	0.996159	6843663	8.80669	8.636981	8.797244	8.932802	8.737632	8.743291	8.599877	9.140488	8.502911	9.011147	9.013783	9.195987	5730403B	NM_025670
6843674	5719	7.517314	7.622219	7.590774	7.552426	-0.03511	0.677455	0.069793	0.415941	0.038348	0.649982	0.031445	0.709208	-0.10491	0.233287	-0.07346	0.39295	6843674	7.519876	7.492869	7.539198	7.569716	7.7684	7.528542	7.542272	7.759245	7.470806	7.567066	7.545095	7.545117	Anks3	NM_028301
6843680	5721	9.775404	9.56347	9.736456	9.799587	-0.02418	0.858212	-0.23612	0.109311	-0.06313	0.642952	-0.17299	0.223419	0.211934	0.144552	0.038948	0.773919	6843680	9.812961	9.674238	9.839014	9.720307	9.594697	9.375407	9.581796	9.956596	9.670977	9.754624	9.664238	9.979899	Rogdi	NM_133185
6843681	5722	10.20943	10.24327	10.26757	10.20136	0.00807	0.923924	0.041907	0.622659	0.066207	0.442196	-0.0243	0.774227	-0.03384	0.69032	-0.05814	0.497919	6843681	10.15659	10.201	10.27071	10.16551	10.36776	10.19654	10.39294	10.11456	10.29521	10.19408	10.13375	10.27626	3930401K	NM_001079814
6843685	5723	9.762412	9.806603	9.701083	9.941611	-0.1792	0.311358	-0.13501	0.439123	-0.24053	0.184979	0.10552	0.54234	-0.04419	0.796603	0.061329	0.721106	6843685	9.604044	9.873111	9.810081	9.811538	9.818811	9.789461	9.665764	9.721853	9.715633	9.750156	10.37869	9.695988	Ppl	NM_008909
6843690	5724	8.20708	7.928815	8.24672	8.125377	0.081703	0.599469	-0.19656	0.224832	0.121343	0.440276	-0.31791	0.066062	0.278265	0.099607	-0.03964	0.79751	6843690	8.203985	8.199454	8.2178	7.889023	8.057099	7.840322	8.360258	7.894764	8.485138	8.030718	8.304745	8.040668	Nagpa	NM_013796
6843694	5725	8.887616	8.859904	9.06214	9.332755	-0.44514	0.007067	-0.47285	0.005124	-0.27061	0.060528	-0.20224	0.141365	0.027712	0.828669	-0.17452	0.196726	6843694	8.637052	9.037139	8.988657	8.980514	8.714434	8.884763	8.991361	9.201632	8.993428	9.223637	9.334434	9.440193	Fam86	NM_027446
6843830	5727	7.46611	7.652306	7.665164	7.628378	-0.16227	0.03386	0.023928	0.716046	0.036786	0.578242	-0.01286	0.844557	-0.1862	0.018916	-0.19905	0.013902	6843830	7.541477	7.309382	7.547471	7.575526	7.676874	7.704518	7.649673	7.686515	7.659304	7.603291	7.623545	7.658297	Tmem186	NM_025708
6843833	5728	8.747892	9.02402	8.684615	8.653072	0.09482	0.685683	0.370948	0.139155	0.031542	0.892391	0.339406	0.171333	-0.27613	0.256311	0.063277	0.786472	6843833	8.929618	8.599998	8.71406	8.722736	9.447987	8.901338	9.002386	8.318521	8.732937	8.556448	8.799523	8.603246	Carhsp1	NM_025821
6843834	5729	9.203631	9.360276	9.231006	9.286868	-0.08324	0.439268	0.073408	0.493321	-0.05586	0.59982	0.12927	0.24182	-0.15665	0.164145	-0.02737	0.795729	6843834	9.24573	9.234418	9.130746	9.223261	9.44612	9.411448	9.234909	9.380262	9.077847	9.122817	9.404968	9.332819	Usp7	NM_001003918
6843880	5731	11.42558	11.27478	11.33887	11.25837	0.167203	0.247241	0.016403	0.905558	0.080497	0.564518	-0.06409	0.645114	0.1508	0.292899	0.086707	0.535571	6843880	11.47615	11.23664	11.56394	11.19537	11.47495	11.15401	11.53043	11.30814	11.17804	11.24246	11.39493	11.13773	Emp2	NM_007929
6843889	5733	8.994617	9.024379	8.702415	8.939099	0.055518	0.837778	0.085281	0.753593	-0.23668	0.393552	0.321965	0.254848	-0.02976	0.912518	0.292202	0.297945	6843889	9.026722	9.02372	8.933409	9.012402	9.290677	8.770059	8.75972	9.225091	8.122433	8.767371	9.143994	8.905931	Dexi	NM_021428
6843913	5734	8.060172	8.0317	8.296152	7.970233	0.089939	0.669731	0.061467	0.769964	0.325919	0.147352	-0.26445	0.22928	0.028472	0.892017	-0.23598	0.278931	6843913	8.203404	8.12521	7.851901	7.826028	8.411718	7.857353	8.328924	8.596685	7.962848	8.037551	7.957085	7.916063	Socs1	NM_009896
6843914	5735	6.377526	6.223121	6.122941	6.5114	-0.13387	0.423315	-0.28828	0.106755	-0.38846	0.040042	0.100179	0.545399	0.154405	0.358978	0.254585	0.147261	6843914	6.473758	6.14082	6.518	6.073734	6.295755	6.299873	6.425818	5.860221	6.082785	6.610121	6.404597	6.519483	Tnp2	NM_013694
6843915	5736	6.094115	6.246733	6.187757	6.117917	-0.0238	0.922411	0.128815	0.601296	0.06984	0.775565	0.058976	0.809603	-0.15262	0.537306	-0.09364	0.702861	6843915	6.416748	6.031032	5.834565	6.307933	6.108097	6.324168	6.009725	5.858177	6.695369	6.252836	6.197526	5.90339	Prm3	NM_013638
6843916	5737	6.814907	6.866071	6.91917	6.831293	-0.01639	0.861628	0.034778	0.712374	0.087877	0.362642	-0.0531	0.575763	-0.05116	0.589479	-0.10426	0.285171	6843916	6.670564	7.007484	6.766674	6.891758	6.853569	6.852887	6.845084	6.983308	6.929117	6.837983	6.708712	6.947184	Prm2	NM_008933
6843923	5739	10.19321	10.25846	10.29704	9.978849	0.214361	0.266478	0.279611	0.157813	0.318194	0.114135	-0.03858	0.83514	-0.06525	0.72557	-0.10383	0.578767	6843923	10.39065	10.12392	10.06506	10.01723	10.61303	10.14512	10.58943	10.13513	10.16657	10.00096	9.966863	9.968725	Litaf	NM_019980
6843928	5740	8.043979	7.945125	8.19024	8.229015	-0.18504	0.331124	-0.28389	0.151107	-0.03877	0.83379	-0.24511	0.207751	0.098854	0.595557	-0.14626	0.43716	6843928	8.024652	8.087357	8.019929	7.922175	8.157624	7.755576	7.964635	8.523129	8.082955	8.377558	8.370481	7.939005	Txndc11	NM_029582
6843943	5741	8.407289	8.382943	8.508814	8.421937	-0.01465	0.930613	-0.03899	0.816964	0.086877	0.608565	-0.12587	0.462236	0.024347	0.884975	-0.10153	0.550764	6843943	8.489892	8.339999	8.391977	8.175322	8.582055	8.391451	8.430671	8.717653	8.378119	8.517688	8.642332	8.105791	Zc3h7a	NM_145931
6843951	5742	8.70989	8.764694	8.8126	8.650819	0.059072	0.588084	0.113875	0.308438	0.161781	0.160884	-0.04791	0.659443	-0.0548	0.61486	-0.10271	0.355332	6843951	8.658188	8.727891	8.743592	8.630237	8.970396	8.693448	8.929663	8.806829	8.701307	8.537244	8.798361	8.616851	Rsl1d1	NM_025546

6843953	5743	10.09562	10.05996	10.19012	10.06755	0.028063	0.789022	-0.00759	0.942178	0.122569	0.261338	-0.13016	0.235276	0.035653	0.734258	-0.09451	0.378674	6843953	10.16294	10.06775	10.05616	9.962849	10.22784	9.989202	10.31521	10.16386	10.0913	9.942422	10.24046	10.01978	Gspt1	NM_146066
6844000	5744	8.785441	8.797446	8.881337	9.042751	-0.25731	0.040015	-0.24531	0.047825	-0.16141	0.163059	-0.08389	0.447705	-0.012	0.911859	-0.0959	0.388119	6844000	8.721836	8.667689	8.966798	8.850644	8.806082	8.735611	8.979806	8.895535	8.768671	8.908387	8.99747	9.222397	Cpped1	NM_146067
6844072	5745	7.810584	8.018025	7.807007	7.883914	-0.07333	0.251747	0.134111	0.053783	-0.07691	0.231243	0.211018	0.007456	-0.20744	0.008143	0.003577	0.953432	6844072	7.69873	7.966435	7.766586	8.017021	8.001378	8.035676	7.837065	7.771694	7.812262	7.891117	7.86159	7.899035	Parn	NM_028761
6844081	5747	5.636835	6.137166	6.112359	5.879256	-0.24242	0.397369	0.257909	0.369341	0.233102	0.414957	0.024807	0.92935	-0.50033	0.102208	-0.47552	0.117546	6844081	5.751982	5.525576	5.632947	6.265472	6.095952	6.050073	6.494599	5.370075	6.472402	5.927537	5.838541	5.871691	LOC62275	XR_004927
6844093	5748	9.196406	9.305275	9.286364	9.309854	-0.11345	0.081614	-0.00458	0.937912	-0.02349	0.690947	0.018911	0.748477	-0.10887	0.092405	-0.08996	0.152999	6844093	9.167664	9.173403	9.24815	9.234736	9.316767	9.364321	9.379387	9.289148	9.190557	9.289614	9.257245	9.382703	Pdxdc1	NM_053181
6844110	5751	8.090512	8.041323	8.105117	8.028656	0.061855	0.442738	0.012667	0.872765	0.076461	0.347441	-0.06379	0.429127	0.049188	0.538759	-0.01461	0.853537	6844110	7.965615	8.249355	8.056565	7.998816	8.041444	8.08371	8.016809	8.170794	8.127749	8.064156	8.082728	7.939085	4921513D	NM_001081154
6844112	5752	7.388631	7.276399	7.304303	6.998842	0.389789	0.117196	0.277558	0.246541	0.305461	0.206128	-0.0279	0.903078	0.112231	0.626811	0.084328	0.713942	6844112	7.824653	7.162343	7.178896	7.48577	7.195845	7.147583	7.46572	7.52081	6.926379	6.892855	7.109217	6.994453	Myh11	NM_013607
6844118	5753	7.899509	7.904001	7.762358	7.650331	0.249178	0.07847	0.25367	0.074166	0.112027	0.391087	0.141643	0.284789	-0.00449	0.971889	0.137151	0.299259	6844118	7.811325	7.783193	8.104009	7.911747	8.025592	7.774665	7.872406	7.827589	7.587079	7.485184	7.748973	7.716836	0610037P	BC011300
6844176	5755	7.867871	7.880388	7.715778	7.763344	0.104527	0.567613	0.117044	0.523274	-0.04757	0.793068	0.164611	0.375363	-0.01252	0.944848	0.152093	0.411032	6844176	7.855505	7.976863	7.771245	7.777547	8.186697	7.676921	7.751946	7.826653	7.568734	7.43119	7.905525	7.953318	Ube2v2	NM_023585
6844177	5756	7.397658	7.354944	7.341036	6.814478	0.583181	0.037087	0.540466	0.049336	0.526558	0.054143	0.013908	0.953966	0.042714	0.859407	0.056622	0.814504	6844177	7.288296	7.329179	7.5755	7.119072	7.678872	7.266888	7.701948	6.86891	7.45225	6.697388	7.033667	6.712378	Mcm4	NM_008565
6844184	5757	6.670258	6.947093	6.630982	6.834827	-0.16457	0.275054	0.112265	0.447218	-0.20385	0.184765	0.316111	0.054523	-0.27684	0.084227	0.039276	0.786862	6844184	6.629294	6.630646	6.750833	6.898477	6.983224	6.959577	6.914598	6.273813	6.704534	6.900775	6.761555	6.842152	Fam128b	NM_029354
6844187	5758	7.266324	7.315603	7.317109	7.287166	-0.02084	0.848093	0.028436	0.794032	0.029942	0.783441	-0.00151	0.988943	-0.04928	0.652404	-0.05078	0.642647	6844187	7.226892	7.279461	7.29262	7.237453	7.414438	7.294917	7.371895	7.167201	7.41223	7.345682	7.451269	7.064548	2310008H	NM_146068
6844196	5759	9.514074	9.520193	9.517612	9.48428	0.029794	0.712685	0.035913	0.657766	0.033333	0.680683	0.002581	0.974441	-0.00612	0.939452	-0.00354	0.96496	6844196	9.538124	9.461385	9.542712	9.435712	9.59173	9.533137	9.575467	9.508268	9.469102	9.318045	9.499165	9.635629	Dnm1l	NM_152816
6844210	5760	7.261763	7.296939	7.271283	7.390545	-0.12878	0.102001	-0.09361	0.216361	-0.11926	0.125611	0.025656	0.722499	-0.03518	0.627577	-0.00952	0.894791	6844210	7.173873	7.269834	7.341582	7.398827	7.231911	7.252879	7.232909	7.395994	7.184946	7.342183	7.398201	7.43125	Fgd4	NM_139232
6844249	5764	9.31169	9.335421	9.374101	9.306518	0.005172	0.931035	0.028903	0.631187	0.067583	0.276863	-0.03868	0.52303	-0.02373	0.692756	-0.06241	0.312642	6844249	9.234524	9.376086	9.32446	9.278048	9.420361	9.307853	9.456144	9.270211	9.395947	9.304186	9.326568	9.288799	Ppil2	NM_144954
6844253	5766	9.567293	9.58333	9.81308	9.7274	-0.16011	0.523995	-0.14407	0.565418	0.085679	0.73066	-0.22975	0.367041	-0.01604	0.94843	-0.24579	0.336339	6844253	9.664692	9.456504	9.580683	9.381431	9.432842	9.935718	9.906399	10.08798	9.44486	9.997795	9.869629	9.314777	Sdf2l1	NM_022324
6844255	5768	9.022307	8.958761	9.071909	8.94247	0.079837	0.536377	0.016291	0.898387	0.129439	0.325555	-0.11315	0.386683	0.063546	0.621032	-0.0496	0.69867	6844255	9.063379	8.970737	9.032804	8.884809	9.165113	8.82636	9.15521	9.068951	8.991567	8.68705	9.101758	9.038602	Ube2l3	NM_009456
6844269	5769	8.335325	8.352737	8.369627	8.335688	-0.00036	0.99492	0.017049	0.766011	0.033938	0.556923	-0.01689	0.768118	-0.01741	0.761199	-0.0343	0.5528	6844269	8.376904	8.355776	8.273294	8.417519	8.355092	8.2856	8.325352	8.425787	8.357741	8.237999	8.419977	8.349089	Pi4ka	NM_001001983
6844278	5770	7.725532	7.74566	7.823304	7.765268	-0.03974	0.718972	-0.01961	0.858617	0.058036	0.600935	-0.07764	0.487096	-0.02013	0.854917	-0.09777	0.385794	6844278	7.719657	7.692062	7.764877	7.816524	7.87666	7.543796	7.805769	7.941957	7.722187	7.803592	7.894219	7.597993	Thap7	NM_026909
6844283	5771	7.217559	7.19511	7.326084	7.066688	0.150871	0.175657	0.128422	0.241593	0.259397	0.033939	-0.13097	0.233158	0.022449	0.830574	-0.10853	0.316381	6844283	7.310084	7.200949	7.141644	7.245862	7.168517	7.17095	7.381304	7.336275	7.260674	6.984212	6.898592	7.317259	Slc7a4	NM_144852
6844291	5772	8.638944	8.493104	8.512647	8.434214	0.20473	0.167227	0.05889	0.673696	0.078432	0.576621	-0.01954	0.888296	0.14584	0.310736	0.126297	0.376123	6844291	8.690756	8.642186	8.58389	8.48033	8.672968	8.326015	8.256681	8.708308	8.572951	8.347678	8.606194	8.348771	Med15	NM_033609
6844301	5773	6.893096	6.421963	6.446199	9.838447	-2.94535	0.00184	-3.41648	0.000734	-3.39225	0.000768	-0.02424	0.970962	0.471133	0.486173	0.446897	0.508214	6844301	6.229989	6.685904	7.763394	6.281821	6.425778	6.55829	6.54211	6.573803	6.222684	8.838929	9.302581	11.37383	Car15	NM_030558
6844302	5774	8.543474	8.454444	8.420586	8.423565	0.119909	0.190223	0.030879	0.721993	-0.00298	0.972502	0.033859	0.696685	0.08903	0.318934	0.122889	0.180587	6844302	8.465652	8.503198	8.661573	8.364666	8.617666	8.381001	8.517948	8.414674	8.329135	8.411523	8.382659	8.476513	Dgcr2	NM_010048
6844306	5775	8.084649	8.18975	8.071925	8.115765	-0.03112	0.716142	0.073985	0.39648	-0.04384	0.609942	0.117825	0.191502	-0.1051	0.238886	0.012724	0.881366	6844306	8.065656	8.036537	8.151754	8.237766	8.197369	8.134115	8.197182	8.034164	7.984428	8.085849	7.983935	8.27751	Dgcr14	NM_022408
6844310	5776	9.389331	9.352427	9.215671	9.284851	0.10448	0.399728	0.067576	0.580926	-0.06918	0.57216	0.136756	0.277888	0.036904	0.761435	0.17366	0.17757	6844310	9.42054	9.208534	9.538919	9.433017	9.361463	9.262801	9.340262	9.328286	8.978465	9.228883	9.25913	9.366541	Slc25a1	NM_153150
6844316	5777	7.188761	7.18546	7.136871	7.326055	-0.13729	0.09049	-0.14059	0.084246	-0.18918	0.029173	0.048589	0.515047	0.003302	0.964221	0.051891	0.487742	6844316	7.119819	7.208281	7.238184	7.271392	7.075401	7.209586	7.211401	7.160141	7.03907	7.35889	7.219804	7.39947	Prodh	NM_011172
6844321	5778	6.934011	7.01429	7.009824	6.832305	0.101707	0.124629	0.181985	0.015366	0.17752	0.017231	0.004465	0.941818	-0.08028	0.212768	-0.07581	0.23688	6844321	6.966389	6.806363	7.029282	7.011332	7.058196	6.973341	7.093561	6.970172	6.96574	6.799562	6.851902	6.84545	D16H22S6	BC005445
6844324	5779	7.815334	7.872877	7.694601	7.572635	0.242699	0.065376	0.300242	0.02971	0.121966	0.314794	0.178276	0.155608	-0.05754	0.626509	0.120733	0.319403	6844324	7.799366	7.797201	7.849434	8.010015	7.959158	7.649459	7.695476	7.866009	7.522318	7.563482	7.672338	7.482085	Zdhhc8	NM_172151
6844327	5780	9.723969	9.782472	9.750335	9.520531	0.203438	0.191692	0.261941	0.10366	0.229804	0.145878	0.032137	0.827417	-0.0585	0.692503	-0.02637	0.85797	6844327	9.771709	9.655643	9.744555	9.690281	9.963258	9.693878	10.03696	9.747239	9.466807	9.458633	9.650276	9.452684	Ranbp1	NM_011239
6844329	5781	7.441119	7.432865	7.501191	7.449008	-0.00789	0.948705	-0.01614	0.895321	0.052182	0.672257	-0.06833	0.581197	0.008253	0.946345	-0.06007	0.6269	6844329	7.441646	7.418872	7.462838	7.314542	7.719587	7.264467	7.532127	7.578482	7.392963	7.481067	7.542084	7.323874	Dgcr8	NM_033324
6844334	5782	9.599224	9.891467	9.433054	9.44308	0.156144	0.059751	0.448388	0.000234	-0.01003	0.891564	0.458413	0.000202	-0.29224	0.003427	0.16617	0.047971	6844334	9.654629	9.68629	9.456753	9.921616	9.96117	9.791616	9.485097	9.395597	9.418469	9.442456	9.37307	9.513713	Comt1	NM_001111062
6844346	5783	6.523243	6.703798	6.390337	6.587297	-0.06405	0.642425	0.116501	0.405821	-0.19696	0.176249	0.313461	0.045895	-0.18055	0.210951	0.132906	0.346135	6844346	6.439052	6.510071	6.620607	6.752851	6.787561	6.570982	6.552366	6.52064	6.098005	6.473209	6.743145	6.545536	Tbx1	NM_011532
6844348	5784	6.564973	6.760492	6.693885	6.741017	-0.17604	0.516298	0.019475	0.941967	-0.04713	0.860272	0.066607	0.803738	-0.19552	0.472392	-0.12891	0.632416	6844348	6.583941	6.566398	6.544579	6.847799	6.912024	6.521654	6.745975	6.837322	6.498358	6.477841	7.398362	6.346848	5-Sep	NM_213614
6844359	5785	6.079365	6.11731	5.99641	5.857092	0.222274	0.080555	0.260218	0.047337	0.139318	0.245458	0.1209	0.308415	-0.03794	0.741618	0.082956	0.476835	6844359	6.024098	5.998727	6.215271	5.942285	6.201691	6.207954	6.213522	5.929222	5.846485	5.866474	5.853752	5.851049	Cdc45l	NM_009862
6844362	5786	7.709883	7.831691	7.855194	7.619562	0.090321	0.614556	0.212128	0.253493	0.235631	0.20888	-0.0235	0.894933	-0.12181	0.49992	-0.14531	0.423795	6844362	7.533814	7.776255	7.81958	7.774916	7.914522	7.805634	7.853062	7.942402	7.770117	7.204695	7.714174	7.939818	Mrpl40	NM_010922
6844442	5796	6.071325	5.890445	5.75743	6.397869	-0.32654	0.260961	-0.50742	0.096962	-0.64044	0.045081	0.133015	0.635449	0.18088	0.52171	0.313894	0.278443	6844442	6.212574	5.608071	6.393329	5.961193	5.83391	5.876232	5.432209	6.016594	5.823488	5.96813	6.420922	6.804555	Map6d1	NM_198599
6844443	5797	8.600037	8.686689	8.713417	8.738131	-0.13809	0.370558	-0.05144	0.732924	-0.02471	0.869401	-0.02673	0.858881	-0.08665	0.568107	-0.11338	0.458456	6844443	8.557842	8.530576	8.711693	8.683467	8.821994	8.554606	8.872717	8.810744	8.456791	8.524276	8.722328	8.967788	Parl	NM_001005767
6844447	5799	7.547451	7.553658	7.463062	7.597948	-0.0505	0.222248	-0.04429	0.279192	-0.13489	0.007673	0.090596	0.044935	-0.00621	0.874799	0.084389	0.057924	6844447	7.5314	7.588739	7.522214	7.501317	7.592797	7.566861	7.477042	7.464954	7.44719	7.674549	7.583859	7.535437	Abcc5	NM_013790
6844468	5800	9.024323	8.629054	8.999478	8.918341	0.105982	0.45885	-0.28929	0.066395	0.081136	0.567804	-0.37042	0.026252	0.395269	0.01982	0.024846	0.85978	6844468	9.180514	8.937799	8.954657	8.439303	8.844047	8.603812	9.147785	8.756888	9.09376	8.835036	8.997597	8.922391	Alg3	NM_145939
6844480	5801	6.771842	6.974329	6.749353	6.839047	-0.06721	0.485416	0.135282	0.17917	-0.08969	0.357597	0.224976	0.040039	-0.20249	0.058662	0.022488	0.812822	6844480	6.735852	6.75167	6.828003	7.138096	6.851677	6.933215	6.795717	6.800788	6.651555	6.788255	6.732756	6.99613	Clcn2	NM_009900
6844530	5803	7.695862	7.965599	7.909699	7.746302	-0.05044	0.515513	0.219257	0.018224	0.163397	0.058645	0.05586	0.472796	-0.2697	0.006613	-0.21384	0.020382	6844530	7.702423	7.615179	7.769984	7.996763	7.985929	7.913985	7.97218	8.001407	7.755511	7.72035	7.679363	7.839193	2510009E	NM_001001881
6844531	5804	5.984129	6.098956	6.114776	6.211577	-0.22729	0.124957	-0.11262	0.420518	-0.0968	0.486326	-0.01582	0.908002	-0.11467	0.412485	-0.13049	0.354043	6844531	5.798729	6.071243	6.082895	6.286244	5.992278	6.018345	6.122499	6.15995	6.061878	6.031561	6.138888	6.464282	Ehhadh	NM_023737
6844542	5805	7.978856	8.163383	8.201818	8.860542	-0.88169	0.016767	-0.69716	0.044443	-0.65872	0.054551	-0.03844	0.898784	-0.18453	0.546053	-0.22296	0.468135	6844542	7.918016	7.992462	8.026091	8.587383	7.879822	8.022943	8.066138	8.542426	7.99689	8.646727	8.468774	9.466126	Tmem41a	NM_025693
6844549	5807	8.030572	8.22569	8.21164	7.915203	0.115369	0.550439	0.310487	0.131972	0.296437	0.147921	0.01405	0.941356	-0.19512	0.322601	-0.18107	0.356599	6844549	7.791415	8.142007	8.158293	8.018522	8.454925	8.203623	7.953035	8.537874	8.144011	7.975688	8.036394	7.733527	Igf2bp2	NM_183029
6844553	5808	7.029148	7.058383	6.968903	7.14146	-0.11231	0.458261	-0.08308	0.580188	-0.17256	0.265479	0.08948	0.551981	-0.02923	0.844325	0.060245	0.686943	6844553	7.177288	7.067068	6.843088	6.884517	7.118086	7.172545	7.021621	6.843666	7.041422	6.970259	7.031154	7.422966	Tra2b	NM_009186
6844558	5809	7.693381	7.049985	7.239049	7.156834	0.536547	0.10033	-0.10685	0.721066	0.082215	0.783172	-0.18906	0.531154	0.643396	0.056535	0.454332	0.154411	6844558	7.890458	7.548938	7.640748	6.841398	7.778411	6.530146	7.320574	7.107797	7.288776	7.300883	7.220928	6.948692	Etv5	NM_023794
6844567	5810	11.37637	10.83156	10.67035	10.4714	0.904967	0.006699	0.36016	0.186707	0.19895	0.448066	0.16121	0.536126	0.544807	0.060441	0.706017	0.022121	6844567	11.44093	11.14041	11.54776	10.91103	11.02604	10.55761	10.65224	10.3227	11.03611	10.78074	10.58083	10.05263	Dgkg	NM_138650
6844571	5811	6.820884	6.711268	6.62571	6.532826	0.288057	0.424199	0.178442	0.61605	0.092883	0.792861	0.085559	0.808802	0.109615	0.756845	0.195174	0.583967	6844571	6.606743	6.597163	7.258745	6.668748	7.036341	6.428716	6.683642	6.464284	6.729203	7.298448	6.220265	6.079766	Crygs	NM_009967
6844575	5812	7.572606	7.56651	7.722514	7.632978	-0.06037	0.6589	-0.06647	0.627426	0.089536	0.515848	-0.156	0.270258	0.006096	0.964221	-0.14991	0.288016	6844575	7.498446	7.579853	7.639518	7.460201	7.809966	7.429362	7.617512	7.983527	7.566503	7.618484	7.690126	7.590325	Tbccd1	NM_001081368
6844598	5813	5.995274	6.279193	6.185287	5.889579	0.105695	0.701811	0.389614	0.181589	0.295708	0.29906	0.093906	0.733469	-0.28392	0.317455	-0.19001	0.495793	6844598	5.859669	5.925841	6.200313	5.844635	6.83326	6.159684	6.531776	5.879914	6.144172	5.950048	6.025326	5.693364	Rfc4	NM_145480
6844632	5815	6.33846	5.854749	5.827837	5.821693	0.516766	0.001327	0.033055	0.765879	0.006143	0.955743	0.026912	0.808263	0.483711	0.00198	0.510623	0.001428	6844632	6.443243	6.3838	6.188336	6.024625	5.912874	5.626747	5.840474	5.886598	5.756438	5.770706	5.793671	5.900703	Masp1	NM_008555
6844653	5818	7.88396	8.120346	8.049541	7.363649	0.520311	0.276366	0.756697	0.127765	0.685892	0.162141	0.070805	0.877633	-0.23639	0.610023	-0.16558	0.719729	6844653	8.620312	7.593459	7.43811	8.253601	8.784713	7.322725	8.364885	8.191917	7.591821	7.559174	7.457355	7.074418	Bcl6	NM_009744
6844806	5819	8.220587	8.362519	8.207948	8.112641	0.107946	0.367507	0.249878	0.058006	0.095307	0.423587	0.154571	0.208623	-0.14193	0.244637	0.012639	0.913715	6844806	8.402613	8.121514	8.137634	8.329111	8.539515	8.21893	8.166822	8.247658	8.209363	8.058834	8.285814	7.993274	Leprel1	NM_173379
6844819	5820	7.288219	7.467742	6.45314	5.300088	1.98813	0.021679	2.167654	0.014648	1.153052	0.137672	1.014602	0.184735	-0.17952	0.803811	0.835079	0.26647	6844819	7.291888	7.462667	7.110101	6.741711	8.350917	7.310598	6.850156	5.211772	7.297492	5.397882	6.262242	4.240141	Cldn1	NM_016674
6844937	5824	7.145137	7.316911	6.981141	7.175282	-0.03015	0.827765	0.141628	0.321738	-0.19414	0.185707	0.335769	0.036715	-0.17177	0.236085	0.163995	0.256142	6844937	7.02767	7.292738	7.115002	7.389994	7.471705	7.089033	6.941357	7.200269	6.801798	7.17821	7.083595	7.264042	1600021P	NM_177718
6845042	5829	6.445375	6.494268	6.397627	6.420135	0.02524	0.833573	0.074133	0.541536	-0.02251	0.851321	0.096641	0.429963	-0.04889	0.685167	0.047748	0.692086	6845042	6.579855	6.541274	6.214997	6.594666	6.453791	6.434348	6.551217	6.409375	6.23229	6.463537	6.318881	6.477988	Tmem44	NM_172614
6845044	5830	7.997612	8.125503	8.051485	7.996626	0.000986	0.993489	0.128877	0.303153	0.054859	0.651984	0.074018	0.545015	-0.12789	0.306621	-0.05387	0.657761	6845044	8.018278	8.00047	7.974088	8.093208	8.253093	8.030208	8.325159	7.949847	7.879449	7.874818	8.049767	8.065293	Lsg1	NM_178069
6845060	5831	6.913594	6.782408	6.871751	6.990392	-0.0768	0.354609	-0.20798	0.028764	-0.11864	0.167522	-0.08934	0.286073	0.131186	0.131791	0.041843	0.606972	6845060	6.927679	6.91186	6.901244	6.785121	6.901227	6.660877	6.91469	6.772974	6.927589	6.851014	7.049955	7.070208	AI480653	BC031419
6845079	5832	9.285084	8.994266	8.219019	8.710829	0.574255	0.172839	0.283437	0.481175	-0.49181	0.235801	0.775247	0.07799	0.290818	0.470203	1.066065	0.023978	6845079	9.431401	9.030596	9.393255	9.150815	9.632739	8.199245	8.440676	8.561743	7.654639	8.445136	8.925557	8.761795	Apod	NM_007470
6845139	5833	7.197032	7.317744	7.368787	7.405536	-0.2085	0.294755	-0.08779	0.64949	-0.03675	0.848286	-0.05104	0.790684	-0.12071	0.53448	-0.17175	0.382741	6845139	7.196788	6.933832	7.460477	7.330247	7.36226	7.260724	7.474098	7.632783	6.99948	7.274043	7.353234	7.589331	0610012G	BC021536
6845141	5834	7.242024	7.395169	7.185837	7.111281	0.130743	0.379903	0.283889	0.078312	0.074556	0.610534	0.209333	0.175081	-0.15315	0.308045	0.056187	0.700061	6845141	7.318572	7.199728	7.207771	7.211865	7.573373	7.40027	7.237078	7.353863	6.966569	7.114345	7.315114	6.904383	Senp5	NM_177103

6845146	5835	9.579755	9.497797	9.552682	9.362634	0.217121	0.0464	0.135163	0.180992	0.190048	0.073318	-0.05488	0.568273	0.081958	0.400182	0.027073	0.776604	6845146	9.570177	9.549798	9.61929	9.461973	9.667865	9.363554	9.62524	9.634612	9.398194	9.332502	9.461701	9.2937	Pak2	NM_177326
6845147	5836	7.063713	7.418612	7.049476	7.180083	-0.11637	0.336682	0.238529	0.069491	-0.13061	0.284507	0.369136	0.011844	-0.3549	0.014296	0.014237	0.903583	6845147	6.990016	7.098111	7.103011	7.246611	7.58838	7.420845	7.144076	7.169676	6.834676	7.066358	7.230866	7.243026	Pigx	NM_024464
6845154	5837	6.694349	6.605804	6.513059	6.485807	0.208542	0.345553	0.119996	0.579988	0.027252	0.899027	0.092744	0.667598	0.088546	0.681641	0.18129	0.40897	6845154	7.053108	6.589015	6.440925	6.502349	6.858591	6.456471	6.733728	6.330466	6.474984	6.31479	6.357763	6.784869	Lrrc33	NM_146069
6845159	5838	7.998924	8.104679	8.035524	7.951931	0.046993	0.652154	0.152749	0.166564	0.083593	0.429119	0.069156	0.510329	-0.10576	0.322848	-0.0366	0.724839	6845159	7.903046	8.072565	8.02116	7.956086	8.164163	8.193789	8.102644	8.120418	7.883509	7.821675	8.094406	7.939711	Fbxo45	NM_173439
6845193	5840	8.437503	8.553047	8.385842	8.217711	0.219792	0.621161	0.335336	0.455529	0.168131	0.704465	0.167205	0.706002	-0.11554	0.79385	0.051661	0.906823	6845193	8.668687	8.037223	8.6066	8.297207	9.093852	8.268083	8.778673	8.228584	8.15027	7.588453	7.947301	9.11738	Muc20	NM_146071
6845195	5841	8.119625	8.142631	8.149763	7.990148	0.129477	0.032659	0.152484	0.016126	0.159616	0.013007	-0.00713	0.890539	-0.02301	0.658938	-0.03014	0.564885	6845195	8.112261	8.13921	8.107403	8.127142	8.180274	8.120478	8.07695	8.247091	8.125249	7.922716	8.075397	7.97233	1700021K	BC060601
6845274	5843	7.521767	7.439705	7.547492	7.140282	0.381485	0.03896	0.299423	0.08897	0.40721	0.030077	-0.10779	0.50571	0.082062	0.610209	-0.02573	0.872057	6845274	7.381608	7.505003	7.678689	7.316512	7.662782	7.33982	7.810366	7.2986	7.53351	6.99931	7.268566	7.15297	Umps	NM_009471
6845348	5844	7.35161	7.457167	7.430093	7.427936	-0.07633	0.343217	0.029231	0.709681	0.002157	0.977984	0.027074	0.730068	-0.10556	0.201028	-0.07848	0.330537	6845348	7.329924	7.396166	7.328741	7.441186	7.532637	7.397678	7.502099	7.535144	7.253036	7.35367	7.448241	7.481897	Sec22a	NM_133704
6845366	5845	9.49053	10.09736	9.23731	9.625251	-0.13472	0.568622	0.472104	0.07076	-0.38794	0.125287	0.860045	0.005274	-0.60683	0.028026	0.253219	0.29627	6845366	9.559595	9.269505	9.642489	9.959346	10.16181	10.17091	9.138976	9.556445	9.01651	10.05653	9.220275	9.598948	Dirc2	NM_153550
6845375	5846	8.543635	8.368275	8.450041	7.552478	0.991157	0.000312	0.815797	0.001098	0.897563	0.000599	-0.08177	0.632049	0.17536	0.316895	0.093593	0.584487	6845375	8.481156	8.601716	8.548032	8.177801	8.664473	8.26255	8.538629	8.218499	8.592996	7.388068	7.806278	7.463088	Parp14	NM_001039530
6845377	5847	8.689841	8.629909	8.686158	8.103166	0.586675	0.016638	0.526743	0.026711	0.582992	0.017124	-0.05625	0.779741	0.059931	0.765809	0.003682	0.985356	6845377	8.571331	8.716632	8.781559	8.445422	8.9395	8.504806	8.871331	8.316781	8.870363	7.930469	8.323085	8.055944	Dtx3l	NM_001013371
6845382	5848	7.364086	7.565402	7.374107	7.680226	-0.31614	0.01707	-0.11482	0.307599	-0.30612	0.019741	0.191295	0.107015	-0.20132	0.092471	-0.01002	0.92658	6845382	7.387841	7.32816	7.376258	7.483182	7.638326	7.574697	7.547759	7.469234	7.105328	7.728177	7.702022	7.610479	Fam162a	NM_027342
6845419	5850	6.755299	6.562298	6.450633	6.721631	0.033668	0.935367	-0.15933	0.702445	-0.271	0.519575	0.111665	0.788403	0.193001	0.644283	0.304666	0.470621	6845419	6.159788	7.521425	6.584684	6.401313	6.301562	6.984019	6.500877	6.615156	6.235865	6.453035	6.347916	7.363943	Casr	NM_013803
6845435	5851	6.146975	5.901594	6.004327	5.310352	0.836623	0.040493	0.591242	0.122783	0.693975	0.077467	-0.10273	0.772002	0.245381	0.494368	0.142648	0.688188	6845435	6.526523	5.956102	5.958299	5.970469	6.304873	5.429439	6.514587	5.444623	6.05377	5.245057	5.67512	5.010878	Cd86	NM_019388
6845444	5852	7.670364	8.506785	7.906985	8.197546	-0.52718	0.366635	0.309239	0.589959	-0.29056	0.612221	0.5998	0.307986	-0.83642	0.16744	-0.23662	0.678894	6845444	7.516087	7.138206	8.356799	8.392565	8.770133	8.357657	8.524673	6.993883	8.202399	9.063718	8.171544	7.357375	Slc15a2	NM_021301
6845471	5855	6.732864	6.720885	6.834653	6.698806	0.034058	0.800422	0.022079	0.869666	0.135847	0.327674	-0.11377	0.408085	0.011979	0.929016	-0.10179	0.45723	6845471	6.730574	6.821997	6.646021	6.733798	7.014471	6.414385	6.856003	6.862111	6.785844	6.675518	6.668138	6.752762	Gtf2e1	NM_028812
6845551	5857	6.948913	6.60293	6.449918	6.279271	0.669642	0.024043	0.323658	0.2164	0.170647	0.499273	0.153012	0.543472	0.345983	0.189288	0.498995	0.07232	6845551	6.901268	6.796389	7.149082	6.523764	6.856488	6.428537	6.879926	6.180168	6.28966	5.874077	6.510751	6.452986	Pla1a	NM_134102
6845553	5858	8.016709	7.709231	7.957665	7.985416	0.031293	0.808937	-0.27619	0.058459	-0.02775	0.830146	-0.24843	0.082511	0.307479	0.039579	0.059044	0.649823	6845553	7.93682	8.072402	8.040906	7.658331	7.767829	7.701532	8.008034	7.898054	7.966907	7.793201	7.846608	8.316439	Adprh	NM_007414
6845559	5859	8.554708	8.492133	8.680696	8.760249	-0.20554	0.161181	-0.26812	0.078791	-0.07955	0.566654	-0.18856	0.194393	0.062575	0.650873	-0.12599	0.371663	6845559	8.414157	8.600511	8.649457	8.50669	8.332651	8.637058	8.825191	8.650217	8.566681	8.559923	8.718651	9.002173	4930455C	NM_024273
6845562	5860	7.779555	7.855186	7.980131	7.804638	-0.02508	0.859626	0.050549	0.722368	0.175493	0.237129	-0.12494	0.389521	-0.07563	0.596872	-0.20058	0.182283	6845562	7.765605	7.88741	7.685651	7.750177	7.950805	7.864577	8.277046	7.818794	7.844553	7.637325	7.814593	7.961995	Ktelc1	NM_172380
6845569	5861	9.968486	9.661994	9.700555	9.84346	-0.17497	0.088769	-0.18147	0.079428	-0.1429	0.152325	-0.03856	0.680734	0.006493	0.944468	-0.03207	0.731772	6845569	9.635086	9.832929	9.537444	9.746661	9.536392	9.702928	9.617025	9.824997	9.659643	9.828203	9.892877	9.809299	Cdgap	NM_020260
6845800	5863	6.669272	6.735122	6.536263	6.757789	-0.08852	0.294558	-0.02267	0.781241	-0.22153	0.022949	0.198859	0.035821	-0.06585	0.428266	0.133009	0.130404	6845800	6.718245	6.742798	6.546772	6.754558	6.685966	6.764841	6.63764	6.601429	6.36972	6.699732	6.765207	6.808428	Gap43	NM_008083
6845909	5864	7.251636	7.283943	7.288856	7.337825	-0.08619	0.303523	-0.05388	0.51128	-0.04897	0.549568	-0.00491	0.951562	-0.03231	0.69105	-0.03722	0.647601	6845909	7.257237	7.291253	7.206417	7.170276	7.456234	7.22532	7.327434	7.279305	7.25983	7.268206	7.457358	7.28791	Qtrtd1	NM_029128
6845911	5865	7.020396	6.757171	6.947052	7.341399	-0.321	0.213236	-0.58423	0.039236	-0.39435	0.135214	-0.18988	0.446833	0.263225	0.299668	0.073344	0.765218	6845911	7.024531	6.67537	7.361287	6.627637	6.709913	6.933964	6.625173	7.443182	6.772801	7.259888	7.366838	7.397472	Zdhhc23	NM_001007460
6845912	5866	6.44382	6.622595	6.738065	6.774304	-0.33048	0.065492	-0.15171	0.356192	-0.03624	0.82095	-0.11547	0.477443	-0.17877	0.281879	-0.29425	0.094103	6845912	6.291333	6.581618	6.45851	6.551291	6.675361	6.641133	6.734103	6.824546	6.655547	6.409981	7.066044	6.846886	Gramd1c	NM_153528
6845914	5867	9.458479	9.6753	9.727306	10.09131	-0.63283	0.037342	-0.41601	0.139866	-0.36401	0.189473	-0.05201	0.842781	-0.21682	0.417847	-0.26883	0.320497	6845914	9.571951	9.467246	9.336239	9.920263	9.490003	9.615633	9.605676	10.0837	9.492542	9.809426	9.825267	10.63924	Atp6v1a	NM_007508
6845943	5869	8.876578	8.590664	8.873103	8.717545	0.159033	0.133627	-0.12688	0.21964	0.155558	0.141167	-0.28244	0.018023	0.285914	0.017047	0.003475	0.971798	6845943	8.810867	9.047148	8.771719	8.645604	8.67533	8.451059	8.82733	8.993509	8.79847	8.731468	8.631877	8.789289	Boc	NM_172506
6845957	5870	7.263195	7.411767	7.386365	7.526512	-0.26332	0.036174	-0.11474	0.305264	-0.14015	0.21775	0.025403	0.814509	-0.14857	0.193891	-0.12317	0.27351	6845957	7.104129	7.463916	7.22154	7.399513	7.323726	7.512063	7.423963	7.271124	7.464007	7.401842	7.554372	7.623322	Gtpbp8	NM_025332
6845971	5871	7.638357	7.493803	7.731089	7.74547	-0.10711	0.410658	-0.25167	0.075734	-0.01438	0.910098	-0.23729	0.090703	0.144554	0.275122	-0.09273	0.473881	6845971	7.508502	7.753874	7.652695	7.578959	7.491064	7.411385	7.839678	7.679594	7.673996	7.601056	7.606403	8.028951	Slc35a5	NM_028756
6845978	5872	8.599674	9.039108	8.745965	8.878356	-0.27868	0.16373	0.160752	0.402233	-0.13239	0.487118	0.293143	0.145425	-0.43943	0.041991	-0.14629	0.44413	6845978	8.755632	8.809855	8.233536	8.976443	9.19173	8.949152	8.880446	8.537467	8.819982	8.753577	9.125303	8.756188	Cd200	NM_010818
6846000	5876	6.029845	6.054746	6.05249	6.191873	-0.16203	0.297965	-0.13713	0.373721	-0.13938	0.366309	0.002256	0.988012	-0.0249	0.868415	-0.02264	0.880225	6846000	6.075762	6.061948	5.951826	6.108506	5.901924	6.153808	6.225243	5.768492	6.163734	6.224707	5.969951	6.380961	Tagln3	NM_019754
6846003	5877	6.237609	6.335178	6.27965	6.340892	-0.10328	0.395193	-0.00571	0.961577	-0.06124	0.608698	0.055528	0.642021	-0.09757	0.420704	-0.04204	0.724069	6846003	6.137555	6.242275	6.332997	6.376586	6.418087	6.210862	6.198423	6.426716	6.213811	6.318168	6.149671	6.554838	Abhd10	NM_172511
6846006	5878	8.742144	9.094395	8.849947	9.197494	-0.45535	0.09751	-0.1031	0.682213	-0.34755	0.190081	0.244448	0.343384	-0.35225	0.184778	-0.1078	0.668718	6846006	8.193622	8.955578	9.077232	8.76069	9.186989	9.335506	8.707309	9.041121	8.801412	9.253996	9.113588	9.224899	Phldb2	NM_153412
6846029	5880	6.587823	7.421225	6.535301	6.418899	0.168924	0.648191	1.002327	0.022762	0.116402	0.752352	0.885924	0.037774	-0.8334	0.047538	0.052522	0.88649	6846029	6.704536	6.920477	6.138456	6.874672	8.083124	7.30588	6.649723	6.522805	6.433375	6.124856	6.949741	6.182099	Pvrl3	NM_021496
6846197	5883	8.814096	8.939804	8.748946	8.815167	-0.00107	0.991601	0.124637	0.241995	-0.06622	0.520936	0.190859	0.089062	-0.12571	0.238315	0.06515	0.527525	6846197	8.740187	8.897566	8.804535	8.822662	9.054668	8.942083	8.588609	8.830249	8.827979	8.655992	8.914271	8.875239	Bbx	NM_027444
6846375	5886	7.335438	7.369439	7.299266	6.811589	0.523849	0.050432	0.557849	0.039949	0.487676	0.064611	0.070173	0.765827	-0.034	0.885001	0.036172	0.877723	6846375	7.652533	7.123817	7.229964	7.371961	7.561172	7.175183	7.575099	6.933949	7.388749	6.725486	7.139775	6.569507	Nfkbiz	NM_030612
6846386	5887	5.935451	5.950859	6.0357	5.864225	0.071226	0.530126	0.086634	0.44784	0.171475	0.152821	-0.08484	0.456947	-0.01541	0.890633	-0.10025	0.382726	6846386	5.915057	5.959352	5.931944	5.791416	6.000406	6.060754	5.869262	6.269823	5.968016	5.931431	5.88791	5.773334	Cep97	NM_028815
6846388	5888	6.264115	6.364917	6.571873	6.623804	-0.35969	0.215059	-0.25889	0.360873	-0.05193	0.850711	-0.20696	0.460791	-0.1008	0.715733	-0.30776	0.282561	6846388	6.630979	6.135896	6.025469	6.60522	6.364246	6.125286	6.414751	7.118743	6.182125	6.642622	6.78324	6.44555	2310061J	BC017620
6846393	5889	7.7066	7.754188	7.714756	7.645935	0.060665	0.703599	0.108253	0.501546	0.068821	0.666444	0.039432	0.804148	-0.04759	0.76494	-0.00816	0.959014	6846393	7.570654	7.845655	7.703491	7.593051	7.780987	7.888526	7.986572	7.79069	7.367006	7.629128	7.667823	7.640855	Rg9mtd1	NM_029092
6846437	5890	9.864438	9.881168	9.94539	9.94603	-0.08159	0.590566	-0.06486	0.667787	-0.00064	0.996601	-0.06422	0.670835	-0.01673	0.911353	-0.08095	0.593429	6846437	9.891971	9.713332	9.98801	9.769081	10.15761	9.716813	9.887338	10.12064	9.828192	9.943727	10.10799	9.786373	Tfg	NM_019678
6846455	5894	6.626432	6.708101	6.670291	6.531197	0.095235	0.42897	0.176904	0.160334	0.139094	0.258378	0.03781	0.749333	-0.08167	0.495288	-0.04386	0.711222	6846455	6.496887	6.759392	6.623016	6.733953	6.822743	6.567606	6.796455	6.447471	6.766947	6.439392	6.552128	6.602071	Nit2	NM_023175
6846457	5895	8.237236	8.612502	8.290043	8.371968	-0.13473	0.159329	0.240534	0.024287	-0.08192	0.373025	0.322459	0.005924	-0.37527	0.002539	-0.05281	0.559933	6846457	8.308414	8.209525	8.193768	8.483717	8.703521	8.650268	8.437355	8.192413	8.24036	8.285023	8.339914	8.490966	Tbc1d23	NM_026254
6846463	5896	6.762987	7.150708	6.846472	6.938463	-0.17548	0.055835	0.212245	0.026921	-0.09199	0.274986	0.304236	0.004705	-0.38772	0.001137	-0.08348	0.318615	6846463	6.816618	6.6168	6.855543	7.185426	7.122434	7.144263	6.928284	6.91653	6.694602	6.987211	6.934851	6.893326	2610528E	NM_025599
6846468	5897	8.255455	8.127739	7.189473	6.884325	1.37113	0.015632	1.243414	0.024181	0.305148	0.515368	0.938266	0.069742	0.127716	0.783006	1.065982	0.044729	6846468	8.507478	8.633272	7.625614	7.871994	8.719093	7.79213	7.769423	6.901272	6.897724	6.193275	7.40458	7.05512	Col8a1	NM_007739
6846497	5898	8.738101	8.09543	8.761606	8.26643	0.471671	0.016155	-0.171	0.303011	0.495175	0.01285	-0.66618	0.002657	0.642671	0.003267	-0.0235	0.883481	6846497	8.733014	8.705198	8.776091	8.113214	8.027845	8.145231	9.100414	8.540507	8.643896	8.181966	8.522484	8.094841	St3gal6	NM_018784
6846571	5918	8.748872	8.555491	8.795338	8.735277	0.013595	0.914593	-0.17979	0.181416	0.06006	0.637974	-0.23985	0.086625	0.193381	0.154035	-0.04647	0.715047	6846571	8.820271	8.725999	8.700347	8.528379	8.653759	8.484335	8.760905	8.74088	8.884228	8.425567	8.861231	8.919034	Crybg3	NM_174848
6846575	5919	7.028307	6.93982	7.041505	7.016025	0.012281	0.922583	-0.07621	0.551075	0.02548	0.840376	-0.10169	0.430421	0.088486	0.490524	-0.0132	0.916825	6846575	7.097498	6.919858	7.067564	6.842933	7.080209	6.896319	7.104846	6.952472	7.067198	6.815579	6.945936	7.286561	Arl6	NM_019665
6846646	5921	8.272711	8.240365	8.27367	8.433533	-0.16082	0.48252	-0.19317	0.40222	-0.15986	0.485051	-0.0333	0.882567	0.032346	0.88592	-0.00096	0.996605	6846646	8.035984	8.421268	8.360881	8.408665	8.345988	7.966442	7.974502	8.359987	8.48652	8.048738	8.564412	8.687448	Nsun3	NM_178925
6846647	5922	8.10375	8.186237	8.098712	7.903411	0.200339	0.012237	0.282826	0.001884	0.195301	0.013823	0.087525	0.197121	-0.08249	0.221495	0.005037	0.937457	6846647	8.005033	8.18194	8.124276	8.10254	8.254888	8.201284	8.199006	8.010591	8.08654	7.893327	7.901425	7.915481	Arl13b	NM_026577
6846694	5924	6.942748	7.152608	7.173316	7.14915	-0.2064	0.213115	0.003457	0.982477	0.024166	0.87808	-0.02071	0.895393	-0.20986	0.206277	-0.23057	0.169194	6846694	6.878057	7.154632	6.795554	7.082	7.208278	7.167545	7.196687	7.362567	6.960694	6.922632	7.117898	7.406921	Zfp654	NM_028059
6846729	5926	8.740362	8.869442	8.777471	8.677808	0.062554	0.523147	0.191634	0.07505	0.099663	0.318509	0.091971	0.355039	-0.12908	0.20561	-0.03711	0.702411	6846729	8.634256	8.859406	8.727424	8.887154	8.963992	8.757179	8.927481	8.658889	8.746042	8.579058	8.673759	8.780606	Chmp2b	NM_026879
6847184	5931	8.671916	8.69415	9.149677	8.988935	-0.31702	0.100694	-0.29478	0.122821	0.160742	0.374444	-0.45553	0.028559	-0.02223	0.899697	-0.47776	0.023354	6847184	8.718009	8.786634	8.511106	8.404838	8.860326	8.817287	9.169242	9.299512	8.980277	9.069571	9.195148	8.702085	Hspa13	NR_027492
6847198	5933	8.853806	9.133453	8.872726	8.793455	0.060351	0.674495	0.339997	0.039613	0.07927	0.582753	0.260727	0.096491	-0.27965	0.07813	-0.01892	0.894703	6847198	8.830887	8.890602	8.839929	8.828558	9.424084	9.147716	8.977672	8.77051	8.869995	8.911101	8.798607	8.670658	Nrip1	NM_173440
6847238	5934	5.907839	5.878871	5.996441	6.065742	-0.1579	0.27519	-0.18687	0.20311	-0.0693	0.621108	-0.11757	0.408566	0.028968	0.835239	-0.0886	0.52949	6847238	5.771077	5.948228	6.004213	5.700607	5.952881	5.983125	6.220836	5.987772	5.780715	6.23565	5.987042	5.974535	---	GENSCAN000000
6847324	5935	6.092754	6.270504	6.493501	6.337815	-0.24506	0.457781	-0.06731	0.835694	0.155686	0.633501	-0.223	0.497948	-0.17775	0.587019	-0.40075	0.237849	6847324	6.122888	5.970987	6.184387	6.240329	6.249175	6.322009	6.33561	7.290132	5.854762	6.533464	6.364624	6.115358	Btg3	NM_009770
6847338	5936	6.594378	6.845307	6.703947	6.6497	-0.05532	0.852048	0.195606	0.515036	0.054247	0.854885	0.141359	0.635792	-0.25093	0.407727	-0.10957	0.712752	6847338	6.393652	6.960574	6.428909	6.622163	6.732953	7.180804	6.477357	6.888775	6.74571	6.374612	7.241253	6.333236	D16Ertd4	NM_025967
6847517	5938	8.722933	8.781221	8.727165	8.680929	0.042004	0.645139	0.100292	0.286343	0.046236	0.612732	0.054056	0.555191	-0.05829	0.525415	-0.00423	0.962742	6847517	8.66848	8.753494	8.746826	8.716525	8.951147	8.675991	8.745882	8.732469	8.703143	8.520583	8.714365	8.807839	Mrpl39	NM_017404
6847525	5939	8.560681	8.567147	8.57053	8.705018	-0.14434	0.351006	-0.13787	0.371847	-0.13449	0.383111	-0.00338	0.98205	-0.00647	0.9657	-0.00985	0.94778	6847525	8.423187	8.522087	8.73677	8.564754	8.603987	8.532701	8.82419	8.592322	8.295079	8.531578	8.704775	8.8787	Atp5j	NM_016755
6847540	5940	12.2245	12.24637	12.262	12.16023	0.064273	0.523838	0.086147	0.397754	0.101773	0.322078	-0.01563	0.875287	-0.02187	0.826249	-0.0375	0.707516	6847540	12.3579	12.15538	12.16022	12.2685	12.26621	12.20441	12.41826	12.14508	12.22266	12.14697	12.02135	12.31236	App	NM_007471
6847553	5941	10.63477	10.44746	10.39533	10.54715	0.087623	0.705949	-0.09969	0.668161	-0.15182	0.517149	0.052127	0.821875	0.187313	0.427412	0.23944	0.316405	6847553	10.87724	10.78655	10.24052	10.77199	10.40921	10.16117	10.46663	10.29791	10.42145	10.28531	10.84882	10.50731	Cyyr1	NM_144853
6847556	5942	8.295155	8.690472	7.915913	7.962146	0.333009	0.37871	0.728326	0.075919	-0.04623	0.900263	0.77456	0.062081	-0.39532	0.30082	0.379242	0.319604	6847556	8.348125	8.137712	8.399627	8.379152	9.057867	8.634398	8.250504	7.469261	8.027973	7.261363	8.627584	7.997491	Adamts1	NM_009621

6847559	5943	7.161326	7.958445	6.664651	6.825666	0.335661	0.374894	1.132779	0.013172	-0.16101	0.664136	1.293794	0.006764	-0.79712	0.056167	0.496675	0.201864	6847559	7.437006	6.940572	7.106401	7.35706	8.420334	8.09794	7.05059	6.546716	6.396647	6.22065	7.240586	7.015761	Adamts5	NM_011782
6847627	5944	5.656355	6.005206	6.101093	5.999158	-0.3428	0.036733	0.006048	0.965853	0.101934	0.477889	-0.09589	0.503593	-0.34885	0.034292	-0.44474	0.011734	6847627	5.532779	5.698816	5.737471	5.913239	6.254623	5.847756	6.223228	6.241225	5.838825	5.966237	5.99444	6.036798	Rwdd2b	NM_016924
6847632	5945	9.251389	9.260279	9.242826	8.982063	0.269326	0.070746	0.278216	0.063565	0.260763	0.078409	0.017453	0.895942	-0.00889	0.946862	0.008563	0.948813	6847632	9.242509	9.212398	9.299261	9.102508	9.454044	9.224286	9.482675	9.032537	9.213267	8.878869	9.118295	8.949026	Cct8	NM_009840
6847680	5948	7.520972	7.937556	7.478754	7.297853	0.223119	0.645552	0.639703	0.207903	0.180901	0.70855	0.458802	0.354596	-0.41658	0.398348	0.042218	0.93018	6847680	6.276973	8.055619	8.230325	7.944263	7.761761	8.106644	7.189801	7.592262	7.654199	7.094228	7.52404	7.275291	Cldn8	NM_018778
6847748	5963	8.597831	8.933281	8.505592	8.877994	-0.28016	0.184127	0.055286	0.781515	-0.3724	0.089416	0.427688	0.057267	-0.33545	0.119958	0.092239	0.645043	6847748	8.614031	8.659457	8.520006	8.933846	8.806056	9.05994	8.256817	8.855857	8.404103	8.523386	8.955504	9.155093	Tiam1	NM_009384
6847765	5964	8.489361	8.459857	8.521012	8.455835	0.033526	0.838825	0.004022	0.980505	0.065177	0.693626	-0.06115	0.711493	0.029504	0.8579	-0.03165	0.847702	6847765	8.545185	8.537393	8.385504	8.51411	8.607107	8.258354	8.298644	8.889633	8.374759	8.418489	8.56181	8.387205	Sfrs15	NM_178923
6847805	5965	6.622551	6.996928	6.421017	6.286058	0.336493	0.126745	0.71087	0.00698	0.134959	0.51359	0.575912	0.019387	-0.37438	0.094538	0.201534	0.337274	6847805	6.757504	6.615985	6.494163	6.839806	7.39881	6.752169	6.610271	6.16259	6.490189	6.238263	6.505122	6.114789	2610039C	NM_025642
6847819	5966	7.503326	7.590174	7.433374	7.490615	0.012711	0.931384	0.099559	0.506198	-0.05724	0.699546	0.1568	0.304966	-0.08685	0.560645	0.069953	0.637991	6847819	7.576058	7.663476	7.270445	7.506684	7.745561	7.518276	7.678936	7.439236	7.181949	7.503403	7.470909	7.497533	1110004E	BC019533
6847823	5967	7.476435	7.738531	7.484366	7.623747	-0.14731	0.213604	0.114784	0.323177	-0.13938	0.236935	0.254165	0.048067	-0.2621	0.042907	-0.00793	0.943795	6847823	7.410862	7.503234	7.51521	7.636739	7.601468	7.977386	7.392701	7.418319	7.642079	7.714209	7.577721	7.579311	1810007M	NM_026110
6847824	5968	7.393556	8.001998	7.712199	7.940558	-0.547	0.064253	0.06144	0.815652	-0.22836	0.396629	0.289799	0.288634	-0.60844	0.044119	-0.31864	0.246751	6847824	6.984199	7.436452	7.760017	7.909105	7.792399	8.304489	7.523641	7.965876	7.647079	8.329597	7.771349	7.720727	4932438H	NM_028905
6847834	5969	8.968214	8.739586	8.995563	8.798754	0.169459	0.28797	-0.05917	0.701455	0.196809	0.222746	-0.25598	0.123873	0.228627	0.163181	-0.02735	0.85882	6847834	8.892118	9.05211	8.960413	8.62834	8.744995	8.845424	8.683022	8.982008	9.32166	8.670559	8.880738	8.844966	H3f3a	NM_008210
6847876	5970	10.57681	10.52135	10.92176	10.99575	-0.41894	0.062997	-0.47441	0.04034	-0.07399	0.713034	-0.40041	0.073083	0.055463	0.782381	-0.34495	0.11351	6847876	10.55939	10.56499	10.60605	10.30806	10.87076	10.38522	10.7153	11.27479	10.77519	11.08843	11.12729	10.77154	Tmem50b	NM_030018
6847878	5971	6.719614	6.881046	6.828298	6.820266	-0.10065	0.632875	0.06078	0.771944	0.008032	0.969363	0.052748	0.801272	-0.16143	0.448803	-0.10868	0.606434	6847878	6.452025	6.787344	6.919474	6.978335	6.855017	6.809785	6.780644	6.733654	6.970596	6.636538	6.538213	7.286046	Dnajc28	NM_001099738
6847880	5972	8.151422	8.14341	8.194023	8.132854	0.018568	0.860462	0.010556	0.920343	0.061169	0.566364	-0.05061	0.634028	0.008012	0.939486	-0.0426	0.688003	6847880	8.091465	8.101359	8.261443	8.050945	8.259804	8.119481	8.225051	8.159784	8.197233	7.965878	8.359001	8.073683	Gart	NM_010256
6847899	5973	6.189237	6.279112	6.261814	6.145108	0.044129	0.726252	0.134004	0.302806	0.116706	0.365585	0.017297	0.890474	-0.08987	0.481249	-0.07258	0.567361	6847899	6.364964	6.219309	5.983438	6.161858	6.342067	6.33341	6.441543	6.291512	6.052388	6.12818	6.205551	6.101593	Donson	NM_021720
6847901	5974	7.738046	7.91427	7.810564	7.7823	-0.04425	0.739627	0.13197	0.334825	0.028264	0.831545	0.103706	0.443315	-0.17622	0.207806	-0.07252	0.588276	6847901	7.697694	7.752205	7.764238	7.7528	8.093533	7.896478	8.012399	7.615109	7.804185	7.669504	7.698419	7.978977	Cryzl1	NM_133679
6847928	5975	8.565809	8.778474	8.648922	8.968407	-0.4026	0.109947	-0.18993	0.421045	-0.31949	0.191549	0.129553	0.578864	-0.21267	0.370127	-0.08311	0.720192	6847928	8.638073	8.422834	8.63652	8.773238	8.816518	8.745667	8.714277	9.045864	8.186624	8.845046	8.738292	9.321883	Atp5o	NM_138597
6847948	5977	5.964497	5.917416	6.285195	7.054394	-1.0899	0.084796	-1.13698	0.074336	-0.7692	0.202599	-0.36778	0.525605	0.047081	0.934389	-0.3207	0.578749	6847948	5.899918	6.074808	5.918764	6.179909	5.862257	5.710082	6.332427	6.532121	5.991038	6.369359	6.23525	8.558574	Kcne1	NM_008424
6847952	5978	7.934672	7.696347	7.541759	7.368374	0.566298	0.011625	0.327973	0.096196	0.173385	0.348236	0.154588	0.400259	0.238325	0.208022	0.392913	0.053893	6847952	8.018764	7.970216	7.815035	7.746865	7.911816	7.430359	7.867165	7.264407	7.493704	7.217885	7.412867	7.474369	Rcan1	NM_001081549
6847972	5979	9.020705	8.595991	8.23274	7.456373	1.564332	0.003646	1.139618	0.01826	0.776367	0.078824	0.363251	0.373675	0.424715	0.302668	0.787965	0.07523	6847972	9.385095	8.920681	8.75634	8.359063	9.202292	8.226617	8.342114	7.810351	8.545755	7.457637	8.05678	6.854702	Runx1	NM_001111023
6848040	5981	6.067684	5.997787	6.125787	6.137006	-0.06932	0.537675	-0.13922	0.232073	-0.01122	0.919573	-0.128	0.268578	0.069896	0.534396	-0.0581	0.604116	6848040	6.19642	5.951448	6.055183	6.145659	5.812073	6.03563	6.061084	6.044798	6.271479	6.239721	6.045187	6.12611	Cldn14	NM_019500
6848062	5982	6.964274	7.000912	6.89481	6.848685	0.115589	0.218552	0.152227	0.116726	0.046125	0.608643	0.106102	0.255197	-0.03664	0.683281	0.069463	0.445505	6848062	6.999321	6.980976	6.912524	7.093541	7.01605	6.893145	6.888318	7.030572	6.765541	6.725093	6.850366	6.970596	Hlcs	NM_139145
6848080	5983	8.611007	8.690856	8.696299	8.846279	-0.23527	0.144431	-0.15542	0.316457	-0.14998	0.332646	-0.00544	0.97107	-0.07985	0.598012	-0.08529	0.573787	6848080	8.458053	8.683928	8.691041	8.683167	8.497576	8.891826	8.728275	8.841539	8.519082	8.842931	8.638887	9.057018	Pigp	NM_001159617
6848087	5984	7.889995	8.041553	7.974178	7.735864	0.154131	0.128471	0.305689	0.009899	0.238313	0.030595	0.067375	0.479846	-0.15156	0.13408	-0.08418	0.381574	6848087	7.803491	7.818621	8.047873	8.029978	8.132477	7.962204	8.052181	8.032058	7.838294	7.62431	7.777005	7.806278	Dscr3	NM_007834
6848127	5986	7.876922	8.035638	7.583071	7.916059	-0.03914	0.796872	0.119579	0.439679	-0.33299	0.053366	0.452567	0.015177	-0.15872	0.311941	0.293851	0.080749	6848127	7.983008	7.897733	7.750025	8.097025	7.942068	8.067821	7.449227	7.511927	7.78806	7.616054	7.970725	8.161399	Erg	NM_133659
6848146	5987	7.703579	7.861318	7.846671	7.807935	-0.10436	0.474408	0.053383	0.711018	0.038736	0.787616	0.014647	0.918692	-0.15774	0.289425	-0.14309	0.333508	6848146	7.695437	7.782438	7.632863	7.790511	7.861769	7.931675	8.036942	7.816613	7.686458	7.566028	7.75544	8.102338	Psmg1	NM_019537
6848147	5988	8.277646	8.283334	8.150482	8.295571	-0.01792	0.874265	-0.01224	0.913935	-0.14509	0.222557	0.132852	0.260467	-0.00569	0.959922	0.127164	0.279836	6848147	8.241843	8.298373	8.292723	8.267076	8.326406	8.256521	7.951227	8.420706	8.079514	8.309873	8.39231	8.184531	Brwd1	NM_145125
6848177	5989	6.29127	6.08436	6.090387	6.220021	0.071248	0.512296	-0.13566	0.227993	-0.12963	0.247471	-0.00603	0.955172	0.20691	0.081617	0.200883	0.089274	6848177	6.304889	6.365497	6.203423	6.095134	6.019465	6.138481	6.092763	6.291887	5.88651	6.35022	6.180397	6.129447	Itgb2l	NM_008405
6848203	5993	9.937734	9.881801	9.79135	9.310046	0.627688	0.000516	0.571755	0.000939	0.481304	0.002664	0.090451	0.443785	0.055934	0.631788	0.146384	0.228601	6848203	9.897386	9.864617	10.0512	9.758979	10.02103	9.865393	10.02316	9.738489	9.612401	9.380589	9.293328	9.256221	Tmprss2	NM_015775
6848209	5994	7.390125	7.381795	7.379352	7.53767	-0.14755	0.166067	-0.15588	0.146105	-0.15832	0.14068	0.002443	0.980489	0.00833	0.933558	0.010773	0.914152	6848209	7.450135	7.210524	7.509715	7.314022	7.398361	7.433001	7.40721	7.492923	7.237922	7.481571	7.656446	7.474994	Ripk4	NM_023663
6848212	5995	6.392869	6.353032	6.228067	6.262118	0.130751	0.391462	0.090914	0.546337	-0.03405	0.819424	0.124965	0.411811	0.039837	0.78954	0.164802	0.286557	6848212	6.348444	6.514787	6.315375	6.221034	6.600564	6.237497	6.314828	6.280358	6.089015	6.379308	6.409563	5.997483	Prdm15	NM_144789
6848214	5996	7.674595	7.657907	7.622529	7.590807	0.083788	0.317645	0.067099	0.418217	0.031722	0.697158	0.035377	0.664665	0.016688	0.837206	0.052066	0.526424	6848214	7.664175	7.605887	7.753723	7.702171	7.6097	7.661849	7.679839	7.533628	7.654121	7.439481	7.744723	7.588218	C2cd2	NM_174847
6848215	5997	7.614619	7.609204	7.531342	7.574876	0.039743	0.383963	0.034328	0.44925	-0.04353	0.342555	0.077862	0.108808	0.005415	0.903235	0.083277	0.089727	6848215	7.597443	7.595972	7.650441	7.613947	7.616838	7.596827	7.490244	7.605914	7.497867	7.64576	7.493198	7.58567	Zfp295	NM_175428
6848361	5998	8.99877	8.999359	8.967473	8.946962	0.051809	0.500654	0.052397	0.495944	0.020511	0.78716	0.031886	0.675705	-0.00059	0.993802	0.031298	0.681297	6848361	9.001229	9.01001	8.985072	8.971626	9.094368	8.932083	8.866164	9.128845	8.907409	8.896366	9.028652	8.915867	Rbm16	BC075621
6848479	6000	8.026489	7.758999	7.720339	7.434135	0.592355	0.007084	0.324864	0.084491	0.286205	0.121038	0.038659	0.820638	0.267491	0.143643	0.30615	0.100631	6848479	7.974352	7.954392	8.150724	7.713093	7.995742	7.568161	7.819978	7.422808	7.918232	7.568731	7.515139	7.218534	Zdhhc14	NM_146073
6848504	6001	9.209892	9.471287	8.863666	9.297905	-0.08801	0.614171	0.173382	0.331741	-0.43424	0.032233	0.607621	0.006775	-0.2614	0.157933	0.346225	0.073003	6848504	9.092402	9.346763	9.19051	9.446752	9.469812	9.497297	8.851108	8.965793	8.774098	9.19859	8.979907	9.715218	Snx9	NM_025664
6848505	6002	8.797885	8.916899	8.640112	8.897796	-0.09991	0.497211	0.019103	0.895208	-0.25768	0.103981	0.276787	0.084335	-0.11901	0.421559	0.157774	0.294017	6848505	8.7333	8.798289	8.862067	8.933623	8.977138	8.839936	8.567072	8.866195	8.487068	9.099729	8.599711	8.993949	Synj2	NM_001113353
6848511	6003	9.240363	9.26283	9.230257	9.222955	0.017408	0.864018	0.039875	0.696019	0.007302	0.942683	0.032572	0.749209	-0.02247	0.82518	0.010106	0.920754	6848511	9.201135	9.199004	9.32095	9.158982	9.413886	9.215621	9.42141	9.182872	9.08649	9.218391	9.150389	9.300085	Gtf2h5	NM_181392
6848513	6004	9.395224	9.408991	9.179459	9.299248	0.095976	0.554539	0.109744	0.500684	-0.11979	0.463552	0.229533	0.178446	-0.01377	0.931676	0.215765	0.202998	6848513	9.320669	9.4585	9.406502	9.474861	9.463819	9.288294	9.127017	9.495135	8.916224	9.068356	9.35416	9.475227	Tulp4	NM_054040
6848553	6005	6.683817	6.796235	6.711415	6.279167	0.40465	0.094428	0.517068	0.041603	0.432248	0.077312	0.08482	0.701323	-0.11242	0.612509	-0.0276	0.900258	6848553	6.65609	6.470847	6.924513	6.858644	6.888794	6.641268	7.103517	6.310684	6.720045	6.371413	6.431046	6.035042	Tagap	NM_145968
6848568	6007	11.24878	11.15816	11.30238	11.31227	-0.06349	0.546111	-0.15411	0.164583	-0.00989	0.924205	-0.14422	0.190125	0.09062	0.394604	-0.0536	0.609136	6848568	11.29193	11.13042	11.324	11.05474	11.22425	11.1955	11.42897	11.18722	11.29095	11.48576	11.2934	11.15765	Ezr	NM_009510
6848572	6008	7.482304	7.59094	7.44414	7.317059	0.165245	0.013397	0.273882	0.000786	0.127082	0.041196	0.1468	0.022943	-0.10864	0.071404	0.038163	0.486345	6848572	7.559719	7.436346	7.450846	7.543805	7.691305	7.537711	7.448122	7.429902	7.454397	7.306463	7.25871	7.386003	Fgfr1op	NM_201230
6848581	6010	7.381675	7.277287	7.354537	7.298137	0.083538	0.452159	-0.02085	0.848556	0.056399	0.608162	-0.07725	0.485761	0.104388	0.352314	0.027138	0.803867	6848581	7.358138	7.249708	7.537179	7.250208	7.20961	7.372043	7.152743	7.521215	7.389652	7.22555	7.332302	7.33656	Sft2d1	NM_134114
6848583	6011	6.093835	6.20781	6.144027	6.380902	-0.28707	0.012356	-0.17309	0.088654	-0.23688	0.029174	0.063784	0.495497	-0.11398	0.237772	-0.05019	0.589581	6848583	6.103982	6.181427	5.996096	6.302152	6.04743	6.273849	6.006365	6.163877	6.261838	6.318172	6.424749	6.399785	Prr18	NM_178774
6848594	6014	6.085632	6.371079	6.181718	7.043027	-0.9574	1.42E-05	-0.67195	0.000178	-0.86131	3.07E-05	0.189361	0.102044	-0.28545	0.023805	-0.09609	0.376234	6848594	5.883453	6.232888	6.140554	6.214986	6.379905	6.518346	6.1503	6.139046	6.255807	6.980504	7.061038	7.08754	Pde10a	NM_011866
6848677	6016	6.617231	6.817157	6.56356	6.686221	-0.06899	0.709213	0.130935	0.484191	-0.12266	0.511413	0.253597	0.1932	-0.19993	0.295215	0.053671	0.771337	6848677	6.391501	6.77167	6.688521	6.868914	6.699142	6.883414	6.491558	6.68696	6.512162	6.634384	6.355481	7.068799	Park2	NM_016694
6848688	6017	8.793268	8.7607	8.640676	8.948432	-0.15516	0.171827	-0.18773	0.106957	-0.30776	0.017694	0.120024	0.279176	0.032568	0.76083	0.152592	0.178233	6848688	8.605808	8.762703	9.011293	8.754507	8.705922	8.821671	8.572294	8.787577	8.562158	8.943169	8.898833	9.003293	Agpat4	NM_026644
6848718	6020	9.120784	8.995614	8.986659	9.038732	0.082052	0.754335	-0.04312	0.86908	-0.05207	0.842281	0.008955	0.97267	0.12517	0.634531	0.134124	0.610884	6848718	9.133582	9.208942	9.019827	8.910632	9.230778	8.845432	8.489255	9.172685	9.298038	9.026028	9.424397	8.665771	Airn	NR_002853
6848721	6021	7.062306	6.90943	7.044823	7.03604	0.026265	0.91777	-0.12661	0.621404	0.008783	0.972451	-0.13539	0.597832	0.152875	0.552403	0.017482	0.945202	6848721	7.049111	7.156048	6.981758	6.768616	7.196496	6.763179	6.718562	7.173738	7.24217	6.854556	7.561931	6.691634	D17Ertd6	ENSMUST000001
6848736	6022	7.579496	7.286975	7.126627	7.591831	-0.01234	0.917626	-0.30486	0.02981	-0.4652	0.003812	0.160349	0.202717	0.29252	0.035191	0.452869	0.004427	6848736	7.450081	7.635577	7.652829	7.2532	7.180359	7.427367	7.083074	7.243842	7.052964	7.539284	7.815052	7.421157	C230029D	AK049490
6848761	6024	9.115928	8.742153	8.915523	9.695416	-0.57949	0.171127	-0.95326	0.038517	-0.77989	0.077647	-0.17337	0.66479	0.373775	0.360573	0.200405	0.617179	6848761	9.311686	8.741683	9.294415	8.928364	8.576473	8.721621	8.873427	9.299595	8.573546	9.430713	9.073245	10.58229	Smoc2	NM_022315
6848806	6025	6.67408	6.851192	6.747375	6.510191	0.163889	0.092121	0.341001	0.004061	0.237184	0.024358	0.103817	0.260188	-0.17711	0.072541	-0.0733	0.417163	6848806	6.606877	6.575865	6.839498	6.771898	6.959553	6.822125	6.819454	6.753955	6.668717	6.411128	6.531983	6.587463	1600012H	NM_001083882
6848813	6026	8.813258	9.211227	8.34557	8.837723	-0.02447	0.930494	0.373504	0.206668	-0.49215	0.107786	0.865657	0.012905	-0.39797	0.181315	0.467688	0.123628	6848813	8.333097	9.257082	8.849596	9.245703	9.22676	9.161218	8.214358	8.388009	8.434344	8.864114	8.363088	9.285968	10004162	NM_001123372
6848854	6027	7.693827	7.671154	7.628199	7.6791	0.014727	0.771496	-0.00795	0.875247	-0.0509	0.329439	0.042955	0.406399	0.022673	0.65601	0.065627	0.21741	6848854	7.677435	7.678846	7.725199	7.620851	7.754296	7.638315	7.528044	7.664938	7.691616	7.668579	7.709508	7.659212	Fam120b	NM_024203
6848859	6028	8.154426	8.255274	8.057656	8.139906	0.01452	0.831454	0.115368	0.118732	-0.08225	0.248129	0.197618	0.017257	-0.10085	0.165198	0.09677	0.180931	6848859	8.077258	8.197874	8.188147	8.156426	8.363253	8.246143	7.953576	8.103583	8.115809	8.180203	8.080612	8.158904	Tbp	NM_013684
6848877	6029	8.415963	8.590967	8.367658	8.421828	-0.00587	0.955092	0.169139	0.13236	-0.05417	0.606126	0.223309	0.057887	-0.175	0.121212	0.048305	0.645085	6848877	8.387016	8.481477	8.379395	8.425625	8.7183	8.628976	8.447413	8.407553	8.248007	8.268141	8.417673	8.57967	Chd1	NM_007690
6848939	6030	6.169578	6.141544	6.228077	6.239216	-0.06964	0.701119	-0.09767	0.592071	-0.01114	0.950816	-0.08653	0.634298	0.028034	0.876713	-0.0585	0.746788	6848939	6.282856	6.27036	5.955517	5.954709	6.459998	6.009924	6.517991	6.027045	6.139196	6.162346	6.321813	6.233489	Zfp97	NM_011765
6848940	6031	7.514744	7.57465	7.628628	7.508305	0.006439	0.924253	0.066344	0.34161	0.120323	0.104066	-0.05398	0.434586	-0.05991	0.387983	-0.11388	0.120876	6848940	7.463634	7.537907	7.542691	7.552988	7.647153	7.523808	7.70669	7.638687	7.540507	7.380833	7.595497	7.548586	Riok2	NM_025934
6848966	6033	7.802925	7.861956	7.643209	7.705934	0.096991	0.589401	0.156022	0.392245	-0.06272	0.725609	0.218746	0.240497	-0.05903	0.741054	0.159715	0.381666	6848966	7.89963	7.771025	7.738119	8.025306	7.955723	7.604838	7.278352	7.877033	7.774243	7.749574	7.813001	7.555226	4930546H	BC171982
6849044	6040	11.01132	10.89348	11.14509	11.03724	-0.02592	0.790154	-0.14376	0.165479	0.10785	0.285315	-0.25161	0.028311	0.117837	0.246279	-0.13377	0.193338	6849044	11.07035	10.87462	11.08898	10.87051	11.05991	10.75002	11.2586	11.13506	11.0416	11.0457	11.08619	10.97982	Ppp2r1a	NM_016891
6849048	6041	6.958347	7.029588	7.090442	6.97688	-0.01853	0.869085	0.052708	0.641328	0.113563	0.327482	-0.06085	0.591554	-0.07124	0.531314	-0.1321	0.259696	6849048	6.875337	7.050841	6.948864	7.035148	7.043139	7.010476	7.250086	6.804117	7.217124	6.993215	7.002292	6.935132	Zfp160	NM_145483

6849063	6046	7.365532	7.075824	7.677283	7.37254	-0.00701	0.967175	-0.29672	0.109943	0.304743	0.102053	-0.60146	0.006551	0.289708	0.117297	-0.31175	0.095607	6849063	7.52396	7.090408	7.482229	6.801071	7.057202	7.3692	7.776113	7.592133	7.663603	7.265408	7.516052	7.33616	4930432O	NM_001025373
6849064	6047	6.492133	6.543248	6.642669	6.493818	-0.00168	0.991437	0.049431	0.753661	0.148851	0.35668	-0.09942	0.531898	-0.05112	0.745604	-0.15054	0.351557	6849064	6.419527	6.743754	6.313117	6.537785	6.653258	6.438702	6.611932	6.613768	6.702306	6.550026	6.73419	6.197237	Zfp677	NM_172486
6849068	6048	7.021691	6.975742	7.192855	7.146405	-0.12471	0.182248	-0.17066	0.080671	0.04645	0.601265	-0.21711	0.034562	0.045948	0.605128	-0.17116	0.07994	6849068	7.028152	7.033925	7.002995	6.926421	6.962145	7.038661	7.255455	7.292553	7.030557	7.015489	7.126831	7.296896	Zfp54	NM_011760
6849070	6049	6.65093	6.644739	6.882338	6.632214	0.018716	0.944729	0.012525	0.962994	0.250124	0.367079	-0.2376	0.39036	0.006192	0.9817	-0.23141	0.402258	6849070	6.354486	6.684794	6.913511	6.719567	7.061327	6.153322	6.966217	6.994925	6.685872	6.445297	6.982736	6.468609	Zfp51	NM_009558
6849073	6050	7.099209	7.23513	7.005404	7.122248	-0.02304	0.825816	0.112882	0.297571	-0.11684	0.282098	0.229726	0.053107	-0.13592	0.21658	0.093805	0.381605	6849073	7.040941	7.097926	7.158759	7.056734	7.218437	7.430219	6.945157	6.988422	7.082632	7.013465	7.272671	7.080609	Zfp53	NM_013843
6849091	6051	6.560748	6.008723	7.128842	6.17315	0.387598	0.129489	-0.16443	0.493803	0.955692	0.003135	-1.12012	0.001218	0.552025	0.042708	-0.56809	0.038287	6849091	6.600102	6.349226	6.732916	5.833607	6.059483	6.13308	7.419672	6.934142	7.032712	6.219644	6.580926	5.71888	Zfp52	NM_144515
6849098	6052	7.111584	7.029792	7.067387	7.047401	0.064183	0.57392	-0.01761	0.876223	0.019986	0.85971	-0.03759	0.740187	0.081791	0.476446	0.044197	0.697046	6849098	7.062266	7.239278	7.033207	6.915711	7.10857	7.065096	7.081473	7.066824	7.053863	6.81644	7.258075	7.067688	BC049807	BC076634
6849109	6053	7.194308	7.34532	7.231835	7.107811	0.086497	0.710634	0.237509	0.321915	0.124025	0.596481	0.113484	0.627541	-0.15101	0.520956	-0.03753	0.871654	6849109	6.910417	7.631516	7.04099	7.161214	7.465939	7.408806	7.403929	7.120469	7.171108	6.730993	7.275111	7.317328	Zfp760	NM_001008501
6849110	6054	6.035709	6.084931	5.588071	5.852904	0.182805	0.289636	0.232027	0.188015	-0.26483	0.139044	0.49686	0.015068	-0.04922	0.767905	0.447638	0.024043	6849110	5.927929	6.228348	5.950851	5.951196	6.181722	6.121875	5.29813	5.540756	5.925328	5.722081	5.890174	5.946458	---	ENSMUST000000
6849162	6055	7.357382	7.411712	7.636061	7.590481	-0.2331	0.192853	-0.17877	0.307255	0.04558	0.788037	-0.22435	0.208346	-0.05433	0.748853	-0.27868	0.127569	6849162	7.216774	7.412466	7.442907	7.308464	7.428708	7.497963	7.780045	7.676756	7.451381	7.335531	7.473005	7.962906	Zfp758	NM_145484
6849214	6057	6.269039	6.242881	6.443708	6.502917	-0.23388	0.179188	-0.26004	0.140302	-0.05921	0.719053	-0.20083	0.24177	0.026158	0.873301	-0.17467	0.303542	6849214	6.418233	6.002767	6.386117	6.338079	6.225	6.165565	6.477995	6.554933	6.298196	6.348375	6.814801	6.345575	Ccdc64b	NM_153784
6849215	6058	7.639026	7.455477	7.626205	7.579711	0.059315	0.57343	-0.12423	0.253876	0.046494	0.657712	-0.17073	0.129614	0.183549	0.10686	0.012821	0.902173	6849215	7.587846	7.524301	7.804931	7.52613	7.561397	7.278905	7.627963	7.578146	7.672505	7.680773	7.606902	7.451458	Hcfc1r1	NM_181821
6849216	6059	5.833995	6.393847	5.792057	7.240983	-1.40699	0.074504	-0.84714	0.252113	-1.44893	0.067753	0.60179	0.406137	-0.55985	0.438267	0.041938	0.952774	6849216	6.687285	5.122506	5.692195	7.104481	6.382425	5.694636	5.711665	6.738736	4.925771	7.477457	6.209777	8.035714	Cldn6	NM_018777
6849221	6060	6.842875	6.646331	6.863923	6.557938	0.284937	0.141861	0.088393	0.62684	0.305985	0.11826	-0.21759	0.24859	0.196543	0.293615	-0.02105	0.907157	6849221	6.937066	6.610563	6.980995	6.562078	6.982307	6.394609	6.890077	6.903111	6.798582	6.59937	6.752402	6.322042	Pkmyt1	NM_023058
6849231	6061	11.56094	11.53813	11.55137	11.52852	0.032417	0.804066	0.00961	0.941263	0.022853	0.861019	-0.01324	0.919135	0.022807	0.861299	0.009563	0.941548	6849231	11.64042	11.54891	11.49348	11.56874	11.61485	11.4308	11.25219	11.7993	11.60263	11.56639	11.56707	11.4521	Srrm2	NM_175229
6849241	6064	8.831706	7.734667	12.30793	12.8831	-4.05139	0.074374	-5.14843	0.031297	-0.57517	0.778307	-4.57327	0.049274	1.097039	0.593799	-3.47623	0.116454	6849241	9.993199	6.007958	10.49396	12.22275	4.945232	6.036019	12.55572	13.12259	11.24549	13.75074	13.13404	11.76452	Dcpp1	NM_019910
6849244	6065	6.029193	5.469403	9.918243	10.86816	-4.83897	0.035633	-5.39876	0.022666	-0.94991	0.633698	-4.44884	0.048939	0.55979	0.777797	-3.88905	0.077114	6849244	7.07809	3.500242	7.509246	9.84215	3.32062	3.245438	10.04941	11.09895	8.60637	11.97457	10.9463	9.683604	Dcpp2	NM_001039238
6849251	6067	7.054285	7.076626	7.054206	7.291453	-0.23717	0.414622	-0.21483	0.45822	-0.23725	0.414474	0.02242	0.937174	-0.02234	0.937394	7.87E-05	0.999779	6849251	7.060991	7.016019	7.085844	6.827334	7.404295	6.99825	6.990746	6.946928	7.224944	6.968136	7.968959	6.937265	Prss27	NM_175440
6849285	6068	9.336072	9.419002	9.555436	9.909402	-0.57333	0.004211	-0.4904	0.009609	-0.35397	0.040484	-0.13643	0.374256	-0.08293	0.583057	-0.21936	0.168749	6849285	9.325385	9.309318	9.373513	9.569594	9.21372	9.473692	9.34841	9.898763	9.419136	9.865396	9.934048	9.928761	Abca3	NM_013855
6849287	6069	7.815896	8.019648	8.071151	7.937928	-0.12203	0.433906	0.08172	0.596234	0.133223	0.394715	-0.0515	0.73704	-0.20375	0.206253	-0.25526	0.123144	6849287	7.925294	7.737901	7.784492	7.921315	8.262552	7.875076	8.286588	7.897871	8.028994	7.731007	7.963125	8.119652	Rnps1	NM_009070
6849288	6070	8.318289	8.266733	8.478368	8.436749	-0.11846	0.48672	-0.17002	0.325936	0.041619	0.804288	-0.21163	0.22895	0.051556	0.759117	-0.16008	0.353339	6849288	8.076933	8.34898	8.528955	8.185801	8.232676	8.381723	8.425857	8.795229	8.214017	8.325411	8.509884	8.474952	Dci	NM_010023
6849294	6071	8.195837	8.231723	8.099142	8.487586	-0.29175	0.029027	-0.25586	0.048275	-0.38844	0.007675	0.132581	0.26207	-0.03589	0.752367	0.096695	0.404532	6849294	8.209627	8.154127	8.223757	8.29494	8.319784	8.080444	8.092992	8.281252	7.923182	8.3202	8.545233	8.597326	Pgp	NM_025954
6849297	6073	6.34607	6.330107	6.297964	6.342287	0.003783	0.978782	-0.01218	0.931775	-0.04432	0.756082	0.032143	0.821507	0.015963	0.910679	0.048106	0.736153	6849297	6.497096	6.392102	6.149012	6.48864	6.257564	6.244117	6.241157	6.534379	6.118355	6.398811	6.190776	6.437274	Caskin1	NM_027937
6849303	6074	7.848283	7.770489	7.65646	7.682551	0.165731	0.203773	0.087937	0.483732	-0.02609	0.832984	0.114029	0.36889	0.077794	0.534155	0.191823	0.147875	6849303	7.963259	7.837215	7.744374	7.796865	7.892663	7.621938	7.66212	7.815541	7.491718	7.723249	7.8285	7.495905	Pkd1	NM_013630
6849307	6075	6.178332	6.283587	6.201785	6.147436	0.030896	0.714411	0.136151	0.13326	0.054349	0.523516	0.081803	0.34478	-0.10526	0.232481	-0.02345	0.78078	6849307	6.213061	6.037771	6.284163	6.190578	6.319032	6.341152	6.249136	6.207995	6.148223	6.142673	6.029205	6.27043	Nthl1	NM_008743
6849310	6076	7.750903	7.687401	7.811636	7.591484	0.159419	0.446433	0.095917	0.642884	0.220153	0.300982	-0.12424	0.550014	0.063502	0.757939	-0.06073	0.768121	6849310	7.866004	7.618492	7.768212	7.786277	7.956267	7.319659	7.620396	8.139285	7.675228	7.596526	7.768881	7.409044	Zfp598	NM_183149
6849312	6077	6.907983	6.594134	7.219317	6.178442	0.729542	0.03524	0.415693	0.187348	1.040876	0.006881	-0.62518	0.06198	0.313849	0.308065	-0.31133	0.311705	6849312	7.368864	6.5168	6.838286	6.707778	6.809409	6.265216	7.485311	6.763753	7.408888	6.310802	6.356523	5.868	Noxo1	NM_027988
6849316	6079	9.181301	9.031849	8.986862	9.378896	-0.19759	0.502869	-0.34705	0.252885	-0.39203	0.201444	0.044987	0.877061	0.149452	0.610104	0.194439	0.50952	6849316	9.117588	9.056971	9.369344	9.270952	9.163933	8.660662	9.093256	9.113211	8.754118	9.311377	8.866552	9.958759	Sepx1	NM_013759
6849317	6080	7.54477	7.988862	7.116053	8.084418	-0.53965	0.20075	-0.09556	0.81122	-0.96836	0.036835	0.872809	0.054145	-0.44409	0.284388	0.428716	0.300211	6849317	7.124656	7.833952	7.675701	8.068529	8.201894	7.696164	7.19736	7.656086	6.494714	8.092022	7.489554	8.671679	Hs3st6	NM_001012402
6849319	6081	8.271882	8.262226	8.451449	8.310524	-0.03864	0.686916	-0.0483	0.615485	0.140924	0.165886	-0.18922	0.074855	0.009656	0.91938	-0.17957	0.087998	6849319	8.359383	8.17764	8.278622	8.308947	8.349468	8.128263	8.586809	8.504065	8.263472	8.323439	8.276329	8.331805	Hagh	NM_001159626
6849325	6083	7.23863	7.181435	7.257231	7.379668	-0.14104	0.186388	-0.19823	0.076693	-0.12244	0.245015	-0.0758	0.459672	0.057196	0.573963	-0.0186	0.85358	6849325	7.223766	7.219408	7.272717	7.218063	7.257535	7.068706	7.338957	7.40024	7.032496	7.280667	7.416056	7.44228	Spsb3	NM_027141
6849326	6084	8.076412	8.250158	8.146711	8.313242	-0.23683	0.039442	-0.06308	0.531012	-0.16653	0.122183	0.103447	0.314294	-0.17375	0.109007	-0.0703	0.486435	6849326	8.082097	7.875985	8.271154	8.223868	8.306097	8.22051	8.037616	8.24065	8.161868	8.310077	8.253165	8.376484	Mrps34	NM_023260
6849327	6085	8.195689	8.577871	8.164466	8.660779	-0.46509	0.053697	-0.08291	0.697557	-0.49631	0.042372	0.413406	0.079385	-0.38218	0.100333	0.031224	0.883145	6849327	7.864405	8.407334	8.315329	8.469744	8.665155	8.598715	8.155911	8.334482	8.003004	8.545058	8.369119	9.068159	Nme3	NM_019730
6849339	6086	6.77974	6.893159	6.763468	6.764896	0.014844	0.844296	0.128263	0.117686	-0.00143	0.984903	0.129692	0.114238	-0.11342	0.159696	0.016272	0.829576	6849339	6.766866	6.748468	6.823885	6.890231	7.019999	6.769248	6.710796	6.829313	6.750294	6.750606	6.666908	6.877174	Ift140	NM_134126
6849342	6087	8.407605	8.27815	8.476551	8.268708	0.138897	0.042671	0.009443	0.874048	0.207843	0.006956	-0.1984	0.008838	0.129455	0.055091	-0.06895	0.266287	6849342	8.517496	8.330138	8.375181	8.321597	8.280807	8.232047	8.427908	8.542906	8.458838	8.191432	8.287956	8.326735	Clcn7	NM_011930
6849345	6088	6.507484	6.531714	6.435414	6.567223	-0.05974	0.784786	-0.03551	0.870848	-0.13181	0.550537	0.096299	0.661018	-0.02423	0.911624	0.072069	0.742091	6849345	6.676785	6.590259	6.255407	6.547475	6.404438	6.643228	6.66367	6.681634	5.960939	6.679348	6.34953	6.672791	Unkl	NM_028789
6849347	6089	7.869509	8.072355	7.791846	8.022694	-0.15319	0.32061	0.049662	0.740252	-0.23085	0.149245	0.28051	0.088503	-0.20285	0.198487	0.077663	0.606009	6849347	7.744442	7.882312	7.981772	8.043275	8.101632	8.072159	8.041435	7.561947	7.772155	7.949347	7.838614	8.28012	0610007P	NM_026676
6849361	6090	5.889203	5.832071	5.949905	5.947843	-0.05864	0.536701	-0.11577	0.238324	0.002061	0.982454	-0.11783	0.230787	0.057132	0.546979	-0.0607	0.522847	6849361	6.065929	5.725464	5.876216	5.84201	5.771163	5.883041	5.89207	5.889646	6.067998	5.923926	5.880274	6.03933	Prss34	NM_178372
6849383	6096	9.908265	9.84442	9.931262	10.20283	-0.29457	0.039227	-0.35841	0.017202	-0.27157	0.052946	-0.08684	0.488721	0.063845	0.608182	-0.023	0.8524	6849383	9.863446	9.851589	10.00976	9.87883	9.818855	9.835575	10.14298	9.887702	9.763103	10.04214	10.14056	10.4258	Lmf1	NM_029624
6849388	6098	6.787078	6.842155	6.828276	6.867688	-0.08061	0.277314	-0.02553	0.721542	-0.03941	0.584368	0.013879	0.845934	-0.05508	0.448747	-0.0412	0.567803	6849388	6.78518	6.839745	6.73631	6.871172	6.847569	6.807725	6.723124	6.841235	6.920469	6.820482	6.775307	7.007274	Rpusd1	NM_028009
6849391	6100	6.656712	6.733425	6.697765	6.726928	-0.07022	0.368623	0.006498	0.931914	-0.02916	0.702677	0.03566	0.641456	-0.07671	0.328359	-0.04105	0.59274	6849391	6.60195	6.778552	6.589633	6.768654	6.743995	6.687627	6.800889	6.676723	6.615684	6.610336	6.760918	6.809529	Narfl	NM_026238
6849397	6101	7.40109	7.436407	7.476945	7.371287	0.029803	0.67843	0.06512	0.374774	0.105658	0.165766	-0.04054	0.574608	-0.03532	0.623992	-0.07585	0.305449	6849397	7.34105	7.497477	7.364744	7.44802	7.544999	7.316203	7.497952	7.401775	7.531109	7.309096	7.369342	7.435423	Wdr24	NM_173741
6849398	6102	10.31125	10.27354	10.42553	10.53542	-0.22416	0.056229	-0.26187	0.031322	-0.10989	0.305985	-0.15199	0.168858	0.03771	0.717211	-0.11428	0.288354	6849398	10.2482	10.28589	10.39967	10.08498	10.43915	10.2965	10.55121	10.41836	10.30702	10.46579	10.50674	10.63372	Jmjd8	NM_028101
6849406	6103	8.940618	9.081273	8.936201	9.153633	-0.21302	0.159197	-0.07236	0.612291	-0.21743	0.151746	0.145073	0.321309	-0.14066	0.335365	0.004418	0.975107	6849406	8.849704	8.949584	9.022567	9.040308	9.101774	9.101738	9.035595	9.151212	8.621795	9.104526	9.020358	9.336016	0610011F	NM_026686
6849416	6104	6.155072	5.936457	6.090908	6.202514	-0.04744	0.63764	-0.26606	0.025251	-0.11161	0.282777	-0.15445	0.1497	0.218615	0.054094	0.064164	0.526566	6849416	6.066065	6.207349	6.191803	5.903299	6.046772	5.859301	6.040305	6.306422	5.925998	6.165932	6.180244	6.261365	Solh	NM_015830
6849432	6105	8.223512	8.113248	8.227922	8.273078	-0.04957	0.838821	-0.15983	0.517151	-0.04516	0.852957	-0.11467	0.6399	0.110264	0.652646	-0.00441	0.985542	6849432	8.256452	8.196988	8.217097	8.462657	7.844065	8.033023	8.102374	8.67633	7.905063	8.202186	8.047495	8.569553	Tmem8	NM_021793
6849433	6106	6.940627	7.081163	6.92024	6.999821	-0.05919	0.447096	0.081342	0.303901	-0.07958	0.313796	0.160924	0.061489	-0.14054	0.094245	0.020387	0.790028	6849433	6.993791	6.888231	6.939858	7.124567	6.984232	7.134691	6.948266	7.038608	6.773845	6.931017	6.997216	7.071231	Mrpl28	NM_024227
6849434	6107	7.852153	7.989027	7.775835	7.802997	0.049156	0.654283	0.186029	0.116444	-0.02716	0.803676	0.213192	0.078422	-0.13687	0.231456	0.076319	0.490829	6849434	7.789304	7.91599	7.851166	7.892775	8.150091	7.924214	7.860275	7.91812	7.549109	7.731475	7.829333	7.848184	Axin1	NM_009733
6849435	6108	7.69536	7.821076	7.790619	7.942345	-0.24712	0.198654	-0.12141	0.510594	-0.15186	0.414169	0.030457	0.867156	-0.12572	0.49614	-0.09526	0.603749	6849435	7.665305	7.775485	7.645597	7.955009	7.751776	7.756444	7.630833	8.207152	7.533873	8.072857	7.725777	8.028817	Rgs11	NM_001081069
6849438	6109	7.983513	8.218117	8.012891	8.045145	-0.06163	0.470273	0.172972	0.066106	-0.03225	0.702025	0.205226	0.035611	-0.2346	0.020361	-0.02938	0.727273	6849438	7.92036	8.028183	8.001997	8.076813	8.372317	8.205222	8.043897	8.096124	7.898652	8.086692	7.971463	8.07728	Luc7l	NM_028190
6849444	6110	6.687693	6.72975	6.435172	6.596949	0.090744	0.39398	0.132802	0.223875	-0.16178	0.146939	0.294579	0.019163	-0.04206	0.687281	0.252521	0.036549	6849444	6.745465	6.719579	6.598035	6.854105	6.826385	6.508761	6.404516	6.375798	6.525201	6.721212	6.534797	6.534837	EG240055	NM_001081656
6849456	6111	10.92267	10.72516	10.66565	10.53309	0.389577	0.006979	0.19207	0.113806	0.132557	0.255399	0.059513	0.597332	0.197507	0.105393	0.25702	0.044873	6849456	10.99241	10.81664	10.95895	10.6697	10.85985	10.64593	10.87562	10.62914	10.49218	10.42247	10.55911	10.61769	Ergic1	NM_026170
6849458	6112	10.48772	10.45849	10.5381	10.63149	-0.14377	0.246129	-0.173	0.170522	-0.09339	0.439807	-0.07961	0.507932	0.02923	0.805581	-0.05036	0.672584	6849458	10.52799	10.35796	10.5772	10.46253	10.531	10.38193	10.72058	10.5506	10.34312	10.59324	10.49713	10.80409	Atp6v0e	NM_025272
6849466	6113	8.387579	8.6959	8.511136	8.833889	-0.44631	0.007512	-0.13799	0.304348	-0.32275	0.033279	0.184764	0.17988	-0.30832	0.039795	-0.12356	0.354524	6849466	8.225753	8.51931	8.417673	8.730673	8.619659	8.737368	8.642395	8.425149	8.465863	8.897753	8.574236	9.029678	A930001N	NM_029870
6849467	6114	6.830031	6.924681	6.925659	6.904253	-0.07422	0.403801	0.020429	0.814423	0.021406	0.805751	-0.00098	0.991024	-0.09465	0.293606	-0.09563	0.288995	6849467	6.783246	6.826301	6.880545	6.75748	7.031912	6.984652	6.960465	7.032505	6.784006	6.958059	6.888572	6.866127	Bnip1	NM_172149
6849471	6115	6.268137	5.943666	6.031197	6.297048	-0.02891	0.93422	-0.35338	0.328307	-0.26585	0.456092	-0.08753	0.80303	0.324471	0.367141	0.23694	0.504963	6849471	6.584593	6.035951	6.183866	5.870918	6.24492	5.71516	5.544975	6.14551	6.403106	5.959432	6.973111	5.958601	Kifc1	NM_053173
6849474	6116	8.396034	8.39411	8.478354	8.487647	-0.09161	0.425857	-0.09354	0.416604	-0.00929	0.934273	-0.08424	0.462608	0.001924	0.986374	-0.08232	0.472544	6849474	8.378952	8.38492	8.42423	8.500669	8.437914	8.243747	8.353544	8.739559	8.34196	8.438751	8.509942	8.514249	Phf1	NM_009343
6849476	6117	6.406719	6.417078	6.388337	6.447638	-0.04092	0.736779	-0.03056	0.801472	-0.0593	0.627567	0.028741	0.813019	-0.01036	0.931953	0.018382	0.879624	6849476	6.434542	6.325026	6.46059	6.476271	6.370186	6.404777	6.381803	6.63777	6.145439	6.536019	6.312146	6.494748	Ggnbp1	NM_027544
6849481	6118	8.820974	8.786675	8.670498	8.206908	0.614067	0.00144	0.579767	0.002037	0.46359	0.007105	0.116177	0.394828	0.034299	0.797367	0.150476	0.277726	6849481	8.921093	8.692097	8.849733	8.840801	8.851263	8.667961	8.755989	8.587201	8.668304	8.179211	8.481789	7.959723	Itpr3	NM_080553
6849507	6119	11.08937	11.172	11.0988	10.99895	0.09042	0.466751	0.17305	0.181789	0.09985	0.423306	0.0732	0.553399	-0.08263	0.504802	-0.00943	0.938443	6849507	11.11176	11.15077	11.00559	11.23277	11.24695	11.03629	11.116	11.33082	10.84959	10.92777	10.9836	11.08549	Hmga1	NM_016660
6849525	6121	8.101696	8.142491	8.012726	8.003203	0.098494	0.205675	0.139288	0.087263	0.009523	0.897335	0.129765	0.107101	-0.04079	0.583986	0.08897	0.248616	6849525	8.024369	8.171163	8.109557	8.187677	8.186937	8.052859	7.930403	8.124501	7.983274	7.903092	8.01286	8.093656	Anks1	NM_181413

6849530	6122	8.569982	8.436505	9.16867	7.45608	1.113902	0.013671	0.980425	0.024317	1.712591	0.001292	-0.73217	0.072433	0.133477	0.71595	-0.59869	0.129273	6849530	8.960633	8.164073	8.585239	8.218295	8.988222	8.102997	9.419214	9.007821	9.078976	7.658186	7.886586	6.823467	Scube3	NM_001004366
6849532	6123	7.734747	7.759793	7.727556	7.691136	0.04361	0.639196	0.068657	0.465119	0.03642	0.69478	0.032237	0.728073	-0.02505	0.786726	0.00719	0.937952	6849532	7.848763	7.655869	7.699608	7.83203	7.6744	7.77295	7.787733	7.843731	7.551205	7.74712	7.592091	7.734198	Zfp523	NM_172617
6849534	6124	6.727509	6.720612	6.733661	6.595232	0.132277	0.170643	0.12538	0.19145	0.138429	0.153814	-0.01305	0.885625	0.006896	0.939371	-0.00615	0.945902	6849534	6.775434	6.703825	6.703267	6.861874	6.780303	6.51966	6.777843	6.634239	6.7889	6.513515	6.650268	6.621913	Def6	NM_027185
6849536	6125	7.324053	7.285362	7.172852	7.50392	-0.17987	0.202972	-0.21856	0.1305	-0.33107	0.034055	0.11251	0.411009	0.038691	0.773092	0.151201	0.277338	6849536	7.329415	7.366871	7.275874	7.353065	7.354873	7.148148	7.0767	7.449213	6.992644	7.332081	7.530258	7.649422	Ppard	NM_011145
6849539	6126	6.632841	6.736411	6.737678	6.674326	-0.04148	0.469796	0.062086	0.289056	0.063352	0.280028	-0.00127	0.98209	-0.10357	0.094819	-0.10484	0.091495	6849539	6.637058	6.633212	6.628253	6.686459	6.8288	6.693975	6.739501	6.772628	6.700904	6.560973	6.75505	6.706954	Fance	BC016538
6849543	6127	8.033589	8.136481	7.948058	8.050671	-0.01708	0.924155	0.08581	0.634914	-0.10261	0.571378	0.188423	0.310091	-0.10289	0.570355	0.085531	0.635998	6849543	8.185363	7.791356	8.124047	7.95136	8.486401	7.971681	7.9236	8.132308	7.788265	8.098444	8.139754	7.913815	Rpl10a	NM_011287
6849567	6130	8.432197	8.366577	8.356533	8.162809	0.269388	0.072847	0.203768	0.157001	0.193724	0.175934	0.010044	0.940536	0.06562	0.628589	0.075664	0.577966	6849567	8.430469	8.405191	8.46093	8.320929	8.540917	8.237884	8.539661	8.344473	8.185465	8.003599	8.404817	8.08001	Mapk14	NM_011951
6849571	6131	8.616538	8.572962	8.543873	8.094719	0.521818	0.020681	0.478242	0.029965	0.449154	0.03845	0.029088	0.876661	0.043576	0.816325	0.072664	0.699413	6849571	8.541965	8.545831	8.761817	8.370483	8.789597	8.558805	8.632268	8.366388	8.632964	8.068233	8.445096	7.770829	Mapk13	NM_011950
6849572	6132	6.899388	6.944669	6.844754	6.736968	0.16242	0.092609	0.207702	0.040451	0.107786	0.24074	0.099916	0.273919	-0.04528	0.608955	0.054634	0.538649	6849572	6.978608	6.80503	6.914526	6.972806	7.016876	6.844326	6.907075	6.867655	6.759531	6.907465	6.657507	6.645931	Brpf3	NM_001081315
6849581	6133	8.179796	8.389226	8.259651	8.045211	0.134584	0.194876	0.344014	0.00682	0.214439	0.054218	0.129575	0.210294	-0.20943	0.05886	-0.07985	0.425611	6849581	8.122908	8.24912	8.167359	8.268915	8.595145	8.303617	8.359608	8.21838	8.200964	7.95735	8.157993	8.020291	Kctd20	NM_025888
6849589	6134	7.340065	7.295302	7.358384	7.191542	0.148523	0.429354	0.10376	0.576922	0.166842	0.377141	-0.06308	0.732832	0.044763	0.808243	-0.01832	0.92076	6849589	7.340268	7.431993	7.247935	6.900223	7.592837	7.392846	7.337447	7.514762	7.222942	7.013581	7.381194	7.179851	Sfrs3	NM_013663
6849595	6135	9.294921	8.818038	9.057786	8.818278	0.476643	0.133205	-0.00024	0.999349	0.239508	0.425397	-0.23975	0.424952	0.476883	0.133036	0.237135	0.429812	6849595	9.763399	8.926192	9.195172	8.602375	9.147423	8.704315	9.071397	8.997646	9.104314	9.110264	9.065248	8.279321	Cdkn1a	NM_007669
6849612	6137	9.930538	9.977274	9.837285	10.05061	-0.12007	0.510052	-0.07333	0.684788	-0.21332	0.255459	0.13999	0.444732	-0.04674	0.795213	0.093253	0.606897	6849612	9.952027	9.88982	9.949766	9.996309	10.02073	9.914784	10.08832	9.94327	9.480264	9.899342	9.882019	10.37046	BC004004	BC058575
6849621	6138	7.432134	7.170773	6.769789	6.517962	0.914172	0.019927	0.652811	0.072221	0.251827	0.447609	0.400983	0.239262	0.261361	0.43126	0.662345	0.068905	6849621	7.627295	7.53269	7.136417	7.222356	7.653211	6.636751	7.300723	6.55708	6.451565	6.254182	6.602978	6.696726	Pi16	NM_023734
6849622	6139	7.123733	7.114774	7.100195	7.037784	0.085948	0.239251	0.07699	0.287652	0.062411	0.382837	0.014579	0.834633	0.008959	0.897832	0.023537	0.73666	6849622	7.265604	7.024351	7.081243	7.124519	7.10242	7.117383	7.181047	7.033413	7.086126	6.950255	7.087676	7.075422	Fgd2	NM_001159538
6849626	6140	8.687918	8.154198	9.002823	8.003101	0.684817	0.102592	0.151097	0.694955	0.999722	0.027484	-0.84863	0.051772	0.53372	0.188854	-0.3149	0.421386	6849626	9.17345	8.542414	8.347891	7.961512	8.485485	8.015598	9.456176	8.37784	9.174454	7.980512	8.510422	7.51837	Pim1	NM_008842
6849645	6141	7.676446	7.641663	7.512019	7.536056	0.140391	0.265427	0.105608	0.394041	-0.02404	0.842688	0.129644	0.300983	0.034783	0.77428	0.164427	0.19839	6849645	7.6434	7.689001	7.696938	7.603275	7.84379	7.477925	7.380482	7.740917	7.414659	7.45535	7.627444	7.525373	Tbc1d22b	NM_198647
6849653	6142	6.951867	6.965551	6.945405	6.823975	0.127892	0.332899	0.141576	0.286943	0.12143	0.356494	0.020146	0.875067	-0.01368	0.914911	0.006461	0.959753	6849653	6.942586	7.063719	6.849295	6.860503	7.181952	6.854199	7.021643	7.077231	6.737342	6.740201	6.780945	6.950779	Rnf8	NM_021419
6849665	6143	8.331207	8.28229	8.426093	8.218064	0.113143	0.337537	0.064226	0.578485	0.208029	0.097565	-0.1438	0.230938	0.048917	0.670861	-0.09489	0.417161	6849665	8.281842	8.345366	8.366413	8.164217	8.425283	8.257369	8.587018	8.208322	8.48294	8.073195	8.309253	8.271744	1300018I	NM_028791
6849698	6144	9.023909	9.092672	8.971403	8.874028	0.149881	0.198377	0.218644	0.074989	0.097375	0.38885	0.121269	0.289347	-0.06876	0.537954	0.052506	0.636411	6849698	9.021944	9.042032	9.00775	9.09074	9.26799	8.919286	9.004699	9.11712	8.79239	8.755123	8.927701	8.93926	Zfand3	NM_148926
6849766	6148	7.815638	7.852177	8.038573	7.535903	0.279734	0.168397	0.316274	0.125224	0.502669	0.026199	-0.1864	0.342486	-0.03654	0.848129	-0.22294	0.261953	6849766	8.057142	7.669659	7.720112	7.808396	8.119966	7.628169	8.078372	7.934927	8.102419	7.501148	7.852181	7.254381	Abcg1	NM_009593
6849775	6150	7.98283	7.987823	8.014643	7.946213	0.036617	0.820054	0.041609	0.796127	0.06843	0.672058	-0.02682	0.867567	-0.00499	0.975217	-0.03181	0.843269	6849775	7.907422	8.126162	7.914907	8.178549	7.854654	7.930265	8.052774	7.895555	8.095601	7.830501	7.720686	8.287453	Slc37a1	NM_153062
6849779	6151	5.740347	5.811266	6.162106	5.780394	-0.04005	0.848348	0.030872	0.882747	0.381712	0.096508	-0.35084	0.121807	-0.07092	0.73553	-0.42176	0.071094	6849779	6.164771	5.504822	5.551447	6.092534	5.801334	5.539929	6.339083	6.178884	5.968351	5.781338	5.783273	5.77657	Pde9a	NM_008804
6849785	6152	9.456303	9.514683	9.354128	9.62382	-0.16752	0.238815	-0.10914	0.431007	-0.26969	0.074601	0.160555	0.257226	-0.05838	0.669088	0.102174	0.459877	6849785	9.551544	9.45078	9.366584	9.528374	9.370134	9.645542	9.427358	9.414363	9.220664	9.569094	9.405016	9.89735	Ndufv3	NM_030087
6849791	6153	6.924152	6.969602	6.950687	6.865114	0.059038	0.520316	0.104488	0.268194	0.085573	0.358345	0.018915	0.834845	-0.04545	0.618754	-0.02653	0.770221	6849791	6.877305	6.981006	6.914145	6.883183	7.09903	6.926592	6.88359	6.989537	6.978934	6.731696	7.048921	6.814724	Pknox1	NR_027493
6849797	6154	6.399134	6.380391	6.454864	6.287926	0.111208	0.272909	0.092465	0.356307	0.166938	0.115161	-0.07447	0.453188	0.018743	0.847664	-0.05573	0.571488	6849797	6.359938	6.329093	6.508372	6.460675	6.282193	6.398306	6.376408	6.368943	6.61924	6.405523	6.15501	6.303245	Cryaa	NM_013501
6849845	6155	7.117968	7.26919	7.181726	7.223955	-0.10599	0.200887	0.045235	0.568381	-0.04223	0.593858	0.087464	0.28329	-0.15122	0.081949	-0.06376	0.426126	6849845	7.125752	7.138833	7.08932	7.223378	7.450819	7.133374	7.179028	7.215396	7.150755	7.312633	7.161643	7.19759	Rrp1b	NM_028244
6849891	6157	8.606305	8.646062	8.442937	8.383343	0.222962	0.041637	0.262719	0.021303	0.059595	0.535317	0.203125	0.058293	-0.03976	0.67708	0.163368	0.113717	6849891	8.788527	8.496121	8.534267	8.700218	8.717216	8.520752	8.500618	8.385729	8.442465	8.286234	8.374411	8.489383	Cyp4f17	NM_001101445
6849923	6160	6.206664	6.342556	6.235744	6.415583	-0.20892	0.073722	-0.07303	0.492631	-0.17984	0.114592	0.106812	0.323715	-0.13589	0.217717	-0.02908	0.781925	6849923	6.080621	6.240954	6.298417	6.375316	6.310031	6.342322	6.357269	6.233239	6.116724	6.204436	6.507418	6.534894	C920016K	NM_001045559
6849932	6161	8.401674	8.563049	8.371204	8.425713	-0.02404	0.891402	0.137337	0.443976	-0.05451	0.757473	0.191845	0.293295	-0.16138	0.371775	0.030469	0.862661	6849932	8.142846	8.299833	8.762342	8.545296	8.422317	8.721535	8.534616	8.324579	8.254418	8.237434	8.500806	8.538898	10004346	NM_001142957
6849950	6163	7.355401	7.459639	7.062329	7.141256	0.214145	0.167122	0.318383	0.053795	-0.07893	0.590785	0.39731	0.022523	-0.10424	0.480662	0.293073	0.071166	6849950	7.441171	7.420884	7.204149	7.410079	7.658562	7.310275	7.039037	7.192386	6.955563	6.886744	7.186471	7.350553	Adamts10	NM_172619
6849951	6164	7.474486	7.235357	7.435019	6.548669	0.925817	0.00401	0.686688	0.01818	0.88635	0.005096	-0.19966	0.414661	0.239129	0.332984	0.039468	0.869186	6849951	7.757595	7.464193	7.201671	7.247066	7.500114	6.958892	7.706169	7.101848	7.497039	6.431006	6.868138	6.346863	Myo1f	NM_053214
6849952	6165	8.288932	8.159364	8.326554	8.250295	0.038638	0.719754	-0.09093	0.407172	0.076259	0.484098	-0.16719	0.146398	0.129568	0.24783	-0.03762	0.726769	6849952	8.347545	8.27407	8.245182	8.225388	8.323542	7.929162	8.188631	8.398995	8.392035	8.2169	8.336064	8.19792	Zfp414	NM_026712
6849968	6167	10.70403	10.67587	10.77976	9.385184	1.318846	0.017072	1.290683	0.018827	1.394576	0.01315	-0.10389	0.819118	0.028163	0.950492	-0.07573	0.867509	6849968	11.18192	10.80344	10.12673	10.74543	10.87739	10.40478	11.2242	10.20827	10.90681	9.745097	9.873857	8.536598	H2-Eb1	NM_010382
6849970	6168	8.820596	9.147537	8.714204	9.309096	-0.4885	0.029939	-0.16156	0.408873	-0.59489	0.012442	0.433333	0.047599	-0.32694	0.115806	0.106392	0.581801	6849970	8.560653	8.963415	8.937719	9.198706	9.113779	9.130126	8.373656	9.085359	8.683597	9.298271	9.153568	9.475449	Kank3	NM_030697
6849973	6169	5.497328	5.966562	5.88567	6.108539	-0.61121	0.084843	-0.14198	0.660017	-0.22287	0.493807	0.080893	0.801266	-0.46923	0.169618	-0.38834	0.246888	6849973	5.327985	5.894683	5.269315	6.286216	6.101935	5.511536	6.007623	6.340646	5.30874	6.223142	5.95356	6.148915	Ndufa7	NM_023202
6849974	6170	7.000421	7.112076	7.019102	7.52974	-0.52932	0.026876	-0.41766	0.065365	-0.51064	0.031165	0.092974	0.647429	-0.11166	0.583978	-0.01868	0.9263	6849974	6.983522	6.873531	7.144209	7.034536	7.137855	7.163836	7.039944	7.154586	6.862775	7.288412	7.274517	8.026292	Cd320	NM_019421
6849986	6171	7.721877	7.824393	7.691771	7.685955	0.035922	0.728076	0.138438	0.202602	0.005816	0.954934	0.132622	0.220319	-0.10252	0.334133	0.030106	0.770488	6849986	7.719276	7.717661	7.728693	7.672913	8.071038	7.729228	7.712203	7.648045	7.715065	7.561233	7.7602	7.736432	Daxx	NM_007829
6849988	6172	8.145304	8.105116	8.05842	8.107334	0.03797	0.654965	-0.00222	0.979037	-0.04891	0.566493	0.046696	0.583865	0.040188	0.636492	0.086884	0.319288	6849988	8.268748	8.156037	8.011127	8.171021	8.161055	7.983272	8.16127	8.048992	7.964999	8.049533	8.125002	8.147467	Zbtb22	NM_020625
6849990	6173	10.94561	10.78149	10.85492	10.65922	0.28639	0.099928	0.122263	0.450069	0.195693	0.239449	-0.07343	0.646185	0.164127	0.317556	0.090697	0.572083	6849990	10.99124	10.86831	10.97729	10.67149	10.93979	10.73318	11.17966	10.52759	10.8575	10.5453	10.67226	10.76011	Tapbp	NM_001025313
6849991	6174	7.734261	7.873887	7.730754	7.553738	0.180522	0.137865	0.320149	0.019188	0.177016	0.144657	0.143133	0.227509	-0.13963	0.238077	0.003507	0.975239	6849991	7.786324	7.747355	7.669103	7.85817	8.073376	7.690116	7.78208	7.830862	7.57932	7.427548	7.658593	7.575074	Rgl2	NM_009059
6849992	6175	8.606387	8.346778	8.532959	8.600989	0.005397	0.974054	-0.25421	0.152665	-0.06803	0.683476	-0.18618	0.280464	0.259609	0.145196	0.073428	0.660169	6849992	8.564741	8.597455	8.656964	8.297758	8.562747	8.179828	8.293253	8.504972	8.800651	8.576875	8.834868	8.391225	Wdr46	NM_020603
6849994	6176	8.285869	8.217938	8.308762	8.264108	0.021761	0.780855	-0.04617	0.558467	0.044654	0.571183	-0.09082	0.264114	0.067931	0.39528	-0.02289	0.769832	6849994	8.270418	8.300602	8.286588	8.276009	8.29692	8.080886	8.234466	8.324766	8.367054	8.125378	8.362951	8.303996	Vps52	NM_172620
6850000	6177	7.15214	7.269884	7.245884	7.183459	-0.03132	0.714172	0.086424	0.325577	0.062424	0.471065	0.024	0.778579	-0.11774	0.191475	-0.09374	0.288848	6850000	7.115117	7.200117	7.141185	7.229051	7.35676	7.22384	7.288407	7.390675	7.058569	7.177093	7.120794	7.252491	Rxrb	NM_011306
6850001	6178	6.444923	6.460493	6.448771	6.467162	-0.02224	0.799681	-0.00667	0.939225	-0.01839	0.833674	0.011722	0.893435	-0.01557	0.858837	-0.00385	0.964906	6850001	6.51036	6.39438	6.43003	6.427293	6.387225	6.566962	6.516327	6.553225	6.276762	6.501926	6.364664	6.534897	Col11a2	NM_009926
6850011	6179	6.219277	5.980324	5.751875	5.67472	0.544557	0.137346	0.305603	0.381272	0.077155	0.820924	0.228449	0.508156	0.238953	0.489426	0.467402	0.194207	6850011	6.957127	5.935054	5.76565	6.013412	6.312705	5.614854	5.870807	5.502696	5.882122	5.97184	5.482785	5.569536	H2-Oa	NM_008206
6850012	6180	8.038772	7.634798	7.773598	7.33118	0.707592	0.012658	0.303618	0.20729	0.442418	0.080607	-0.1388	0.547985	0.403975	0.105353	0.265174	0.265087	6850012	8.17323	8.33173	7.611357	7.508794	7.91577	7.479829	7.968161	7.504475	7.848158	7.300545	7.526712	7.166283	H2-DMa	NM_010386
6850017	6181	8.674937	8.58218	8.406284	7.964282	0.710655	0.020444	0.617898	0.0366	0.442002	0.1108	0.175896	0.495898	0.092757	0.716558	0.268653	0.307637	6850017	8.919212	8.834736	8.270863	8.449639	8.817512	8.479389	8.823857	7.942569	8.452426	8.02266	7.913525	7.956662	H2-DMb2	NM_010388
6850019	6182	8.397209	8.133965	8.28694	7.518552	0.878656	0.00731	0.615412	0.036979	0.768388	0.014215	-0.15298	0.551705	0.263244	0.316215	0.110269	0.666153	6850019	8.383516	8.471083	8.337027	8.03473	8.44215	7.925014	8.483348	7.858168	8.519304	7.177054	7.932649	7.445954	Tap1	NM_013683
6850021	6183	9.521394	9.372708	9.321908	8.620157	0.901237	0.006493	0.752551	0.015871	0.701751	0.021739	0.0508	0.842129	0.148686	0.563718	0.199485	0.442484	6850021	9.408438	9.558868	9.596875	9.215261	9.657545	9.245318	9.714547	8.866404	9.384774	8.533847	8.989474	8.33715	Psmb8	NM_010724
6850022	6184	8.126457	7.971773	8.069018	7.683509	0.442948	0.008357	0.288264	0.053492	0.385508	0.016407	-0.09724	0.467179	0.154684	0.259292	0.05744	0.664047	6850022	8.115189	8.235373	8.02881	7.89698	7.991944	8.026396	8.225061	7.86292	8.119072	7.473493	7.908421	7.668614	Tap2	NM_011530
6850025	6185	7.65942	7.915893	7.669161	7.878334	-0.21891	0.02004	0.037559	0.632741	-0.20917	0.024434	0.246732	0.011475	-0.25647	0.009473	-0.00974	0.900672	6850025	7.664536	7.772475	7.541248	8.010963	7.909273	7.827443	7.61039	7.779561	7.617532	7.812151	7.916576	7.906274	Notch4	NM_010929
6850033	6186	11.41242	11.32778	11.33393	10.36765	1.044762	0.012882	0.960129	0.01906	0.966272	0.018522	-0.00614	0.985512	0.084633	0.802857	0.07849	0.816852	6850033	11.80688	11.5252	10.90517	11.29941	11.49569	11.18825	11.68061	10.89797	11.4232	10.49976	10.78738	9.815823	H2-Ab1	NM_207105
6850055	6189	7.281615	7.110612	7.011343	6.990467	0.291148	0.142146	0.120145	0.520596	0.020876	0.909943	0.099269	0.593987	0.171003	0.366936	0.270272	0.169136	6850055	7.392156	7.248919	7.20377	7.154573	7.09294	7.084322	6.579737	7.265491	7.1888	7.08658	7.123	6.76182	Gpsm3	NM_134116
6850056	6190	7.421628	7.574137	7.515204	7.428255	-0.00663	0.943445	0.145882	0.145773	0.086949	0.364984	0.058933	0.533329	-0.15251	0.130572	-0.09358	0.331561	6850056	7.485891	7.366707	7.412286	7.406747	7.73561	7.580054	7.502407	7.593742	7.449462	7.312314	7.430636	7.541815	Pbx2	NM_017463
6850058	6191	11.8624	11.72849	11.87296	11.95016	-0.08776	0.401744	-0.22166	0.055725	-0.0772	0.458437	-0.14446	0.183055	0.133907	0.213631	-0.01056	0.917796	6850058	11.94726	11.91078	11.72916	11.75786	11.78588	11.64174	12.00745	11.87723	11.73419	11.79597	11.97306	12.08144	Ager	NM_007425
6850060	6192	9.314794	9.177964	9.330102	9.374987	-0.06019	0.541712	-0.19702	0.070441	-0.04488	0.647311	-0.15214	0.145872	0.13683	0.185431	-0.01531	0.875261	6850060	9.272369	9.3038	9.368214	9.232147	9.326607	8.975138	9.297025	9.470919	9.222362	9.420938	9.337966	9.366057	Agpat1	NM_018862
6850062	6193	5.981062	5.939663	6.001339	5.977274	0.003788	0.972117	-0.03761	0.729573	0.024066	0.824538	-0.06168	0.573298	0.041398	0.703799	-0.02028	0.851738	6850062	5.838441	5.966146	6.138598	5.931053	6.013496	5.874441	6.086619	6.076191	5.841208	6.123208	5.96256	5.846053	Prrt1	NM_030890
6850063	6194	7.145171	7.330994	7.29667	7.319102	-0.17393	0.456844	0.011892	0.95868	-0.02243	0.922169	0.034324	0.881209	-0.18582	0.427812	-0.1515	0.515111	6850063	7.09769	7.034473	7.303349	7.086991	7.679945	7.226046	6.964949	7.756038	7.169024	7.228452	7.285635	7.443218	Fkbpl	NM_019873
6850064	6195	7.871311	7.819699	7.985193	7.923508	-0.0522	0.717442	-0.10381	0.477188	0.061685	0.669428	-0.16549	0.268607	0.051612	0.720453	-0.11388	0.437012	6850064	7.945393	7.856964	7.811575	7.720214	8.022666	7.716217	8.102633	8.181623	7.671323	7.873547	8.007047	7.88993	Atf6b	NM_017406
6850066	6196	7.614659	7.186398	6.999419	6.841235	0.773424	0.037396	0.345163	0.298356	0.158183	0.624019	0.186979	0.56353	0.428261	0.204935	0.61524	0.082734	6850066	7.791308	7.230406	7.822263	7.317319	7.535832	6.706043	7.25764	7.031031	6.709585	6.390233	7.298069	6.835404	Tnxb	NM_031176

6850070	6197	7.267105	7.255149	7.286721	7.092953	0.174152	0.266731	0.162196	0.298477	0.193768	0.220712	-0.03157	0.83407	0.011956	0.936693	-0.01962	0.896352	6850070	7.250992	7.116332	7.433991	7.183642	7.579873	7.001933	7.299962	7.366143	7.194057	7.108801	7.169828	7.00023	Dom3z	NM_033613
6850071	6198	7.776742	7.95582	7.97347	7.832399	-0.05566	0.574853	0.12342	0.230911	0.141071	0.17661	-0.01765	0.857505	-0.17908	0.096707	-0.19673	0.072593	6850071	7.765088	7.710942	7.854196	7.85842	8.0928	7.916239	7.945218	8.118736	7.856457	7.693316	7.945735	7.858147	Rdbp	NM_001045864
6850073	6199	7.102919	7.585301	7.468836	7.402412	-0.29949	0.101723	0.182889	0.291709	0.066424	0.692595	0.116465	0.492722	-0.48238	0.017676	-0.36592	0.053859	6850073	6.816046	7.155619	7.337093	7.582623	7.705247	7.468032	7.440442	7.631814	7.334252	7.149781	7.58239	7.475065	Zbtb12	NM_198886
6850074	6200	8.327401	8.461782	8.278918	8.222352	0.105049	0.084696	0.23943	0.002045	0.056566	0.320374	0.182863	0.009027	-0.13438	0.035997	0.048482	0.390431	6850074	8.342987	8.339101	8.313046	8.461581	8.521241	8.402523	8.286637	8.353643	8.196475	8.127298	8.274903	8.264855	Ehmt2	NM_145830
6850077	6201	8.019405	8.238138	8.228763	8.854988	-0.83558	0.034024	-0.61685	0.096225	-0.62622	0.092073	0.009375	0.977852	-0.21873	0.522773	-0.20936	0.540296	6850077	7.966853	7.884185	8.207178	8.534712	8.09408	8.085623	8.314032	8.647541	7.724717	8.750906	8.339411	9.474646	Slc44a4	NM_023557
6850080	6202	7.933562	8.082961	8.072853	8.56053	-0.62697	0.006012	-0.47757	0.02246	-0.48768	0.02049	0.010108	0.953853	-0.1494	0.403246	-0.13929	0.434467	6850080	7.959278	7.999467	7.841941	8.121418	7.981658	8.145806	8.143727	8.223723	7.851108	8.467107	8.271997	8.942485	Neu1	NM_010893
6850090	6204	6.265447	6.093299	6.140621	6.144461	0.120986	0.261476	-0.05116	0.623213	-0.00384	0.970348	-0.04732	0.649152	0.172148	0.123916	0.124826	0.247829	6850090	6.300143	6.20329	6.292908	6.005733	6.34119	5.932975	6.156552	6.169925	6.095385	6.068361	6.119883	6.245139	Lsm2	NM_030597
6850092	6205	7.863382	7.806794	7.749854	7.609664	0.253718	0.025652	0.19713	0.066319	0.14019	0.169202	0.05694	0.556419	0.056588	0.558805	0.113528	0.25587	6850092	7.904295	7.735596	7.950254	7.795111	7.939305	7.685965	7.812454	7.801722	7.635386	7.62651	7.713705	7.488777	Vars	NM_011690
6850097	6207	10.27876	10.1845	10.07505	9.599251	0.679505	0.010092	0.585252	0.020366	0.475794	0.047038	0.109458	0.604224	0.094253	0.654626	0.203712	0.344745	6850097	10.30798	10.15317	10.37512	10.10943	10.39035	10.05373	10.4937	9.701755	10.02968	9.535273	9.831384	9.431097	Clic1	NM_033444
6850103	6208	7.761483	7.84243	7.446604	7.381871	0.379612	0.020466	0.460559	0.008128	0.064733	0.636369	0.395825	0.016955	-0.08095	0.556	0.314879	0.04385	6850103	7.681986	7.749131	7.853332	7.946269	7.922071	7.658949	7.618819	7.480699	7.240295	7.214613	7.348816	7.582184	Ddah2	NM_016765
6850107	6209	6.397605	6.192638	6.077663	7.687552	-1.28995	0.254608	-1.49491	0.192742	-1.60989	0.16414	0.114974	0.915589	0.204967	0.850248	0.319942	0.768593	6850107	6.214473	6.209554	6.768788	6.091978	6.436425	6.04951	6.14506	6.063467	6.024463	6.532586	10.60509	5.924979	Ly6g6c	NM_023463
6850111	6210	7.263075	7.27555	6.973429	7.109495	0.15358	0.266961	0.166054	0.233038	-0.13607	0.321311	0.302121	0.046877	-0.01247	0.925175	0.289647	0.054533	6850111	7.400793	7.121324	7.267109	7.330667	7.187562	7.30842	7.196088	6.8889	6.835298	6.960441	7.323177	7.044868	Ly6g6e	NM_027366
6850114	6211	8.790539	8.75019	8.697207	8.773695	0.016844	0.795596	-0.0235	0.71829	-0.07649	0.258538	0.052984	0.423961	0.040348	0.539071	0.093332	0.176066	6850114	8.819591	8.720262	8.831763	8.82508	8.728365	8.697126	8.731285	8.739118	8.621217	8.718413	8.707005	8.895667	Bat5	NM_178592
6850118	6214	7.11824	7.02764	6.959767	7.088481	0.029759	0.684735	-0.06084	0.414294	-0.12871	0.106001	0.067874	0.364912	0.090599	0.235694	0.158473	0.055193	6850118	7.110978	7.083879	7.159862	7.079562	6.948699	7.05466	6.841212	7.093287	6.944801	7.073407	7.182175	7.00986	D17H6S53	NM_033477
6850120	6215	9.216433	9.142902	9.159412	9.117716	0.098718	0.281934	0.025186	0.775989	0.041697	0.639123	-0.01651	0.851807	0.073531	0.415164	0.057021	0.523915	6850120	9.247841	9.146736	9.254723	9.181119	9.216666	9.030921	9.052623	9.351485	9.074129	9.067255	9.172294	9.113598	Bat3	NM_057171
6850128	6216	6.585187	6.30007	6.539778	6.240224	0.344963	0.109181	0.059846	0.762534	0.299554	0.156216	-0.23971	0.245819	0.285117	0.174667	0.045409	0.818438	6850128	7.028886	6.473375	6.253299	6.303708	6.311075	6.285427	6.555872	6.436183	6.627279	5.989319	6.423575	6.307778	Ltb	NM_008518
6850131	6217	7.851398	7.902811	7.975569	8.018114	-0.16672	0.137383	-0.1153	0.286589	-0.04254	0.684645	-0.07276	0.491762	-0.05141	0.624428	-0.12417	0.253802	6850131	7.862565	7.975451	7.716178	8.026923	7.894819	7.786692	7.927812	8.107019	7.891877	7.965679	7.924019	8.164644	Atp6v1g2	NM_023179
6850132	6218	9.681852	9.646794	9.758453	9.63317	0.048682	0.469036	0.013624	0.836879	0.125283	0.086196	-0.11166	0.119453	0.035058	0.59908	-0.0766	0.265971	6850132	9.68675	9.686929	9.671877	9.586636	9.797964	9.555782	9.755044	9.764662	9.755652	9.546855	9.636991	9.715664	Bat1a	NM_019693
6850135	6219	11.91465	11.69886	11.88063	11.11754	0.797107	0.008411	0.58132	0.035118	0.763087	0.010472	-0.18177	0.451281	0.215787	0.374697	0.03402	0.885847	6850135	11.98062	12.02093	11.74239	11.55899	11.91849	11.6191	12.09322	11.4943	12.05436	11.06037	11.52247	10.76978	H2-D1	NM_010380
6850155	6222	8.150441	7.712247	7.856402	7.148374	1.002068	0.00844	0.563873	0.086629	0.708029	0.039817	-0.14416	0.631065	0.438194	0.167622	0.294039	0.338354	6850155	7.994945	8.512011	7.944368	7.534436	8.044451	7.557854	7.919431	7.404222	8.245554	6.922711	7.578351	6.944059	H2-gs10	NM_001143689
6850175	6224	6.579129	6.80273	6.522814	6.67209	0.037238	0.784593	0.13064	0.350376	-0.14928	0.289947	0.279916	0.066315	-0.0934	0.498439	0.186515	0.194542	6850175	6.553674	6.747229	6.827083	6.874633	6.727412	6.806146	6.665927	6.333959	6.568556	6.535254	6.551403	6.929614	Cchcr1	NM_146248
6850177	6226	6.0687	5.809777	6.29616	6.503447	-0.43475	0.30934	-0.69367	0.121524	-0.20729	0.618777	-0.48638	0.259227	0.258923	0.536075	-0.22746	0.585674	6850177	6.217786	5.99531	5.993005	5.815825	5.86301	5.750497	6.435982	6.384335	6.068163	5.864325	7.595412	6.050603	Cdsn	NM_001008424
6850191	6228	9.652361	9.299806	8.956688	9.081736	0.570625	0.022147	0.21807	0.310973	-0.12505	0.552384	0.343117	0.127216	0.352555	0.118493	0.695672	0.008691	6850191	9.780926	9.617453	9.558703	9.212358	9.527708	9.159351	9.212392	8.475086	9.182587	8.942451	9.103674	9.199082	Ier3	NM_133662
6850192	6229	8.968488	8.879316	8.787341	8.568795	0.399693	0.013788	0.310521	0.040559	0.218546	0.124245	0.091975	0.490426	0.089172	0.503345	0.181147	0.192411	6850192	9.077263	8.786422	9.04178	8.954335	8.944849	8.738764	8.952612	8.546179	8.863233	8.551446	8.683823	8.471116	Flot1	NM_008027
6850196	6230	8.016017	7.927486	8.082759	7.822179	0.193839	0.181507	0.105308	0.449536	0.26058	0.084699	-0.15527	0.27482	0.088531	0.522707	-0.06674	0.62793	6850196	8.088018	7.943526	8.016508	7.864412	8.078566	7.839481	7.892863	8.31334	8.042074	7.824569	8.014191	7.627776	Mdc1	NM_001010833
6850198	6231	7.475656	7.473237	7.455494	6.933053	0.542603	0.017913	0.540184	0.018279	0.522441	0.021212	0.017743	0.925062	0.00242	0.989763	0.020162	0.914886	6850198	7.532296	7.264072	7.630601	7.400537	7.67167	7.347503	7.791039	7.171771	7.403672	7.130351	6.91904	6.749768	Nrm	NM_134122
6850200	6232	7.753219	7.758617	7.695284	7.622433	0.130786	0.046538	0.136184	0.039996	0.072851	0.226527	0.063334	0.287678	-0.0054	0.925066	0.057936	0.327928	6850200	7.696187	7.740131	7.82334	7.708632	7.865393	7.701827	7.686275	7.695309	7.704267	7.57607	7.709856	7.581373	Dhx16	NM_026987
6850204	6233	8.850497	8.868473	9.013799	8.732648	0.117848	0.347055	0.135824	0.282828	0.28115	0.044318	-0.14533	0.252961	-0.01798	0.88266	-0.1633	0.203657	6850204	8.90232	8.865891	8.783279	8.899986	8.879942	8.82549	9.214531	9.007729	8.819136	8.613514	8.623291	8.96114	Ppp1r10	NM_175934
6850250	6238	7.78703	7.788712	7.740947	7.729579	0.057451	0.5905	0.059133	0.579882	0.011368	0.914423	0.047765	0.653653	-0.00168	0.98731	0.046083	0.66495	6850250	7.743179	7.795139	7.822772	7.831058	7.957113	7.577965	7.753236	7.80057	7.669035	7.612146	7.691626	7.884964	Trim26	NM_001025599
6850265	6241	7.221945	7.270007	7.283098	7.249755	-0.02781	0.859551	0.020252	0.897421	0.033343	0.832065	-0.01309	0.933566	-0.04806	0.760229	-0.06115	0.698332	6850265	7.131989	7.327066	7.206779	7.393208	7.299403	7.117411	7.144906	7.545987	7.158402	7.199271	7.038717	7.511277	1700022C	BC027007
6850268	6242	6.565758	6.586321	6.528041	6.709258	-0.1435	0.535617	-0.12294	0.594413	-0.18122	0.437402	0.05828	0.799303	-0.02056	0.928386	0.037717	0.869145	6850268	6.572367	6.340769	6.784138	6.346061	7.018476	6.394427	6.564258	6.429355	6.59051	6.961595	6.806451	6.359727	Zfp57	NM_001013745
6850271	6243	7.621721	7.696144	7.555381	7.596506	0.025214	0.857384	0.099638	0.484253	-0.04113	0.769837	0.140763	0.330458	-0.07442	0.598776	0.066339	0.638443	6850271	7.57253	7.650383	7.642249	7.655963	7.801101	7.631369	7.220201	7.807436	7.638507	7.624282	7.674973	7.490264	Gabbr1	NM_019439
6850308	6249	5.574614	5.763927	6.162275	5.063327	0.511287	0.48162	0.700601	0.341519	1.098948	0.151363	-0.39835	0.581128	-0.18931	0.791586	-0.58766	0.420979	6850308	6.319806	5.351914	5.052121	5.82909	5.916393	5.546299	5.852871	5.747305	6.886649	5.415542	6.270827	3.503611	Olfr111	NM_001005485
6850421	6263	8.159402	8.429721	8.173311	8.718288	-0.55889	0.02158	-0.28857	0.179772	-0.54498	0.024069	0.25641	0.227802	-0.27032	0.205811	-0.01391	0.945254	6850421	8.012634	8.226051	8.239521	8.464583	8.393727	8.430854	8.130868	8.332376	8.056689	8.388643	8.548572	9.21765	Mut	NM_008650
6850502	6264	7.349732	7.305223	7.086715	7.132398	0.217333	0.219593	0.172824	0.320473	-0.04568	0.786608	0.218508	0.217342	0.044509	0.791943	0.263017	0.14566	6850502	7.326773	7.292829	7.429593	7.373865	7.430998	7.110805	7.315199	6.968059	6.976886	6.989748	7.47035	6.937097	Tnfrsf21	NM_178589
6850534	6266	10.52212	10.97992	10.75837	10.98571	-0.46359	0.153515	-0.00579	0.98478	-0.22734	0.461634	0.221553	0.472727	-0.4578	0.158079	-0.23625	0.444903	6850534	10.91296	10.33337	10.32004	10.98311	11.16634	10.79032	10.82534	10.9634	10.48637	10.96809	10.43932	11.54972	Pla2g7	NM_013737
6850537	6267	8.124499	8.387235	8.307165	8.601212	-0.47671	0.037606	-0.21398	0.29641	-0.29405	0.163331	0.08007	0.68695	-0.26274	0.207435	-0.18267	0.368224	6850537	7.774354	8.233383	8.365759	8.25193	8.451254	8.458521	8.484881	8.179661	8.256952	8.473941	8.395089	8.934605	Cyp39a1	NM_018887
6850552	6269	10.10522	10.11481	10.07963	10.33301	-0.22779	0.232122	-0.2182	0.250625	-0.25338	0.188317	0.035179	0.846712	-0.00958	0.957958	0.025597	0.888075	6850552	10.20579	10.12722	9.982664	10.21806	9.970931	10.15543	9.958427	10.33262	9.947836	10.25135	10.05153	10.69615	Enpp5	NM_032003
6850564	6270	8.21038	8.468938	7.511713	8.725003	-0.51462	0.155813	-0.25606	0.458085	-1.21329	0.006097	0.957225	0.019464	-0.25856	0.453875	0.698667	0.066097	6850564	7.47735	8.463799	8.68999	8.830353	8.078462	8.497999	7.658034	7.365782	7.511322	8.423668	8.863743	8.887597	Clic5	NM_172621
6850594	6271	7.370544	7.472788	7.595196	7.516129	-0.14559	0.155306	-0.04334	0.652906	0.079067	0.41895	-0.12241	0.223626	-0.10224	0.302572	-0.22465	0.041786	6850594	7.191939	7.540232	7.37946	7.610978	7.403206	7.40418	7.683922	7.519292	7.582375	7.518169	7.522375	7.507844	Supt3h	NM_178652
6850623	6272	6.174558	6.319496	6.374303	6.271273	-0.09672	0.567481	0.048222	0.773812	0.10303	0.543036	-0.05481	0.744135	-0.14494	0.397638	-0.19975	0.253119	6850623	5.983992	6.239354	6.300328	6.003248	6.424327	6.530912	6.465508	6.540068	6.117334	6.304408	6.244595	6.264817	B230354K	ENSMUST000000
6850637	6273	6.395441	6.350626	6.451913	6.340002	0.055439	0.318836	0.010624	0.843672	0.111911	0.064204	-0.10129	0.088075	0.044815	0.415209	-0.05647	0.310473	6850637	6.422146	6.378975	6.385202	6.416342	6.329442	6.306095	6.523194	6.508021	6.324523	6.328967	6.354559	6.33648	Aars2	NM_198608
6850645	6275	8.116757	7.845573	8.10958	7.091352	1.025405	0.013766	0.754221	0.049614	1.018228	0.014231	-0.26401	0.441947	0.271184	0.430118	0.007177	0.982994	6850645	8.541921	7.956919	7.851431	7.807044	8.213546	7.516129	8.290969	7.974428	8.063343	6.858693	7.765151	6.650213	Nfkbie	NM_008690
6850646	6276	7.744309	7.765775	7.830554	7.735006	0.009303	0.942725	0.030768	0.812487	0.095548	0.468273	-0.06478	0.619672	-0.02147	0.868432	-0.08624	0.51135	6850646	7.904236	7.713413	7.615279	7.668829	7.877556	7.750939	8.028776	7.909167	7.553719	7.716084	7.721695	7.76724	Slc35b2	NM_028662
6850661	6277	7.36871	7.372595	7.221523	7.285475	0.083236	0.387333	0.08712	0.366653	-0.06395	0.502349	0.151072	0.135644	-0.00388	0.967016	0.147188	0.144625	6850661	7.240301	7.362614	7.503216	7.402002	7.36652	7.349262	7.346491	7.13488	7.183197	7.364678	7.124612	7.367134	Mrpl14	NM_026732
6850678	6278	8.197211	8.080568	8.197985	8.193808	0.003403	0.980938	-0.11324	0.435774	0.004177	0.976601	-0.11742	0.419713	0.116643	0.422659	-0.00077	0.995662	6850678	8.252593	8.130515	8.208524	8.145439	8.092535	8.00373	8.162944	8.534777	7.896234	8.173556	8.156874	8.250994	Mrps18a	NM_026768
6850681	6279	8.100293	8.012727	8.14187	8.04975	0.050543	0.654664	-0.03702	0.742421	0.092119	0.421812	-0.12914	0.269323	0.087566	0.44421	-0.04158	0.712333	6850681	8.256783	8.011168	8.032928	7.951021	8.188545	7.898614	8.247803	8.158976	8.01883	8.042129	8.177297	7.929825	Gtpbp2	NM_019581
6850683	6280	8.025579	8.154473	8.107085	7.962883	0.062696	0.575282	0.19159	0.112132	0.144202	0.216025	0.047387	0.670583	-0.12889	0.264204	-0.08151	0.469466	6850683	8.052693	8.030073	7.993971	8.025723	8.423327	8.014368	8.16511	8.14176	8.014386	7.88346	8.054624	7.950565	Xpo5	NM_028198
6850684	6281	7.518347	7.32786	7.495302	7.565509	-0.04716	0.784488	-0.23765	0.191933	-0.07021	0.684816	-0.16744	0.344714	0.190487	0.286347	0.023045	0.893497	6850684	7.700881	7.462083	7.392077	7.307752	7.344212	7.331615	7.187417	7.796594	7.501894	7.50878	7.806147	7.381601	Yipf3	NM_145353
6850688	6282	6.756915	6.800052	6.727862	6.86083	-0.10391	0.022701	-0.06078	0.138404	-0.13297	0.006974	0.07219	0.086331	-0.04314	0.276372	0.029053	0.454088	6850688	6.799647	6.767139	6.70396	6.841107	6.731329	6.827721	6.722394	6.77011	6.691082	6.878471	6.872262	6.831758	Dlk2	NM_207666
6850695	6283	7.304376	7.423326	7.150148	7.213987	0.090389	0.378385	0.20934	0.062834	-0.06384	0.528675	0.273178	0.02256	-0.11895	0.254671	0.154227	0.150272	6850695	7.155237	7.34305	7.41484	7.341006	7.537266	7.391707	6.968674	7.268773	7.212998	7.259558	7.22276	7.159642	Zfp318	NM_207671
6850705	6285	6.151962	6.090244	6.147219	6.263224	-0.11126	0.366007	-0.17298	0.174614	-0.11601	0.347012	-0.05697	0.636822	0.061718	0.60948	0.004744	0.968413	6850705	6.122623	6.040673	6.292591	6.190136	6.194738	5.885858	6.074771	6.150737	6.216148	6.153258	6.180478	6.455935	BC048355	NM_207161
6850711	6286	8.287876	8.380758	8.277962	8.329937	-0.04206	0.557032	0.05082	0.480227	-0.05198	0.47063	0.102796	0.172611	-0.09288	0.212994	0.009914	0.88873	6850711	8.246502	8.287163	8.329962	8.339771	8.458166	8.344336	8.380534	8.206005	8.247346	8.279696	8.246354	8.463762	Mrpl2	NM_025302
6850721	6287	6.667908	6.913954	6.763426	6.687952	-0.02004	0.780023	0.226002	0.011578	0.075473	0.308409	0.150528	0.06188	-0.24605	0.007554	-0.09552	0.20594	6850721	6.787839	6.609916	6.605969	6.874333	6.940987	6.926542	6.816951	6.80958	6.663746	6.576138	6.738873	6.748846	Cul7	NM_025611
6850723	6288	7.821693	7.903288	7.973539	7.921649	-0.09996	0.24017	-0.01836	0.821557	0.05189	0.528593	-0.07025	0.398547	-0.0816	0.330603	-0.15185	0.090063	6850723	7.813772	7.806829	7.844479	7.834908	7.994643	7.880314	8.058943	7.965858	7.895817	7.747831	7.982046	8.035071	Mea1	NM_010787
6850729	6289	7.368101	7.319414	7.398414	7.52858	-0.16048	0.040537	-0.20917	0.012982	-0.13017	0.083119	-0.079	0.26397	0.048686	0.480234	-0.03031	0.657088	6850729	7.335523	7.374525	7.394254	7.266158	7.358517	7.333568	7.37222	7.559971	7.263051	7.518887	7.521434	7.54542	Pex6	NM_145488
6850732	6290	7.182165	7.247951	7.31446	7.202786	-0.02062	0.916974	0.045165	0.819634	0.111674	0.576189	-0.06651	0.737546	-0.06579	0.740275	-0.1323	0.509581	6850732	7.441251	6.833992	7.271253	7.387171	7.23711	7.119573	7.564503	7.416582	6.962296	7.2081	7.276198	7.124061	2310039H	NM_025966
6850749	6291	6.228787	6.661371	6.337929	6.378797	-0.15001	0.382632	0.282574	0.120032	-0.04087	0.807647	0.323442	0.081554	-0.43258	0.028635	-0.10914	0.52046	6850749	6.275175	6.155711	6.255475	6.554695	6.987002	6.442417	6.331769	6.474951	6.207068	6.139462	6.601022	6.395908	Tbcc	NM_178385
6850763	6293	7.424012	7.455422	7.188447	7.558465	-0.13445	0.291911	-0.10304	0.412391	-0.37002	0.014556	0.266975	0.0554	-0.03141	0.798772	0.235565	0.083471	6850763	7.388607	7.489882	7.393548	7.521404	7.532959	7.311903	6.950049	7.461854	7.153438	7.551029	7.55777	7.566597	Trerf1	NM_001097623
6850779	6294	6.150126	6.170095	6.224569	6.245129	-0.095	0.33607	-0.07503	0.442296	-0.02056	0.830268	-0.05447	0.573515	-0.01997	0.83507	-0.07444	0.445769	6850779	6.170895	6.042961	6.236523	6.088506	6.047396	6.374382	6.256253	6.277154	6.1403	6.179382	6.237797	6.318209	Guca1b	NM_146079
6850780	6295	6.923648	7.247782	7.103014	7.177965	-0.25432	0.181605	0.069817	0.698461	-0.07495	0.677723	0.144767	0.429106	-0.32413	0.099214	-0.17937	0.332319	6850780	6.85673	6.688248	7.225966	7.255112	7.067546	7.420687	7.365571	6.987692	6.95578	7.005375	7.276011	7.252508	Mrps10	NM_001146212

6850784	6296	6.714689	6.553541	6.746452	6.912737	-0.19805	0.364178	-0.3592	0.119149	-0.16629	0.442565	-0.19291	0.376113	0.161148	0.456269	-0.03176	0.881196	6850784	6.517155	6.823205	6.803707	6.893742	6.583202	6.183679	6.659172	6.967829	6.612354	6.630559	7.019772	7.087879	AI661453	NM_145489
6850786	6297	9.158716	9.11269	9.140903	9.281511	-0.1228	0.418	-0.16882	0.274181	-0.14061	0.356833	-0.02821	0.849358	0.046026	0.757129	0.017813	0.904476	6850786	9.06641	9.160649	9.249088	9.081778	9.167212	9.08908	9.030168	9.133797	9.258743	8.915878	9.452936	9.475719	Ccnd3	NM_001081636
6850789	6298	6.988547	7.07744	7.101927	7.026183	-0.03764	0.534858	0.051257	0.402912	0.075744	0.228156	-0.02449	0.684201	-0.08889	0.164155	-0.11338	0.086521	6850789	7.057122	6.937314	6.971206	7.015803	7.093708	7.122808	7.061148	7.172899	7.071733	6.928823	7.124065	7.02566	Med20	NM_020048
6850791	6299	8.37153	8.219874	8.474302	8.272616	0.098914	0.566655	-0.05274	0.75817	0.201687	0.257766	-0.25443	0.162832	0.151656	0.386351	-0.10277	0.551957	6850791	8.33295	8.35375	8.42789	8.129984	7.944635	8.585003	8.47722	8.449525	8.496162	8.326518	8.47061	8.020719	Usp49	NM_198421
6850793	6300	6.889377	6.892616	6.918523	6.875259	0.014118	0.882104	0.017357	0.855379	0.043264	0.651453	-0.02591	0.785873	-0.00324	0.972839	-0.02915	0.760029	6850793	6.849713	6.871777	6.946642	7.006887	6.988323	6.682639	6.875354	7.051043	6.829172	6.858985	6.920499	6.846294	Frs3	NM_144939
6850798	6302	6.893388	6.949097	7.042071	7.022676	-0.12929	0.322081	-0.07358	0.564718	0.019395	0.878125	-0.09297	0.469656	-0.05571	0.661371	-0.14868	0.259476	6850798	6.83968	6.935144	6.905339	6.96133	7.119396	6.766566	7.223224	7.121121	6.781868	6.959921	7.063269	7.044837	Tcfeb	NM_011549
6850817	6304	6.16661	6.090248	6.209421	5.956853	0.209757	0.304858	0.133395	0.505441	0.252568	0.223344	-0.11917	0.550724	0.076361	0.700264	-0.04281	0.828561	6850817	6.532677	5.875607	6.091545	6.282207	6.195255	5.793283	6.253201	6.103872	6.271189	6.122078	5.771518	5.976963	Trem1	NM_021406
6850834	6308	7.869796	7.518662	8.245708	7.205648	0.664148	0.03729	0.313014	0.27362	1.04006	0.004509	-0.72705	0.025839	0.351134	0.223809	-0.37591	0.195754	6850834	8.289458	7.483377	7.836554	7.473196	7.73298	7.349811	8.737672	7.852346	8.147107	7.293551	7.283885	7.039508	Trem2	NM_031254
6850836	6309	6.172358	6.304493	6.198907	6.212092	-0.03973	0.765813	0.092401	0.493955	-0.01318	0.921064	0.105586	0.436529	-0.13213	0.335411	-0.02655	0.841996	6850836	6.237979	6.106954	6.172141	6.345147	6.216853	6.351478	6.20989	6.47291	5.913921	6.191897	6.116556	6.327822	Treml1	NM_027763
6850841	6310	7.813466	7.987967	8.093691	8.068925	-0.25546	0.085741	-0.08096	0.551911	0.024767	0.854071	-0.10572	0.440874	-0.1745	0.217547	-0.28023	0.063842	6850841	7.713819	7.779484	7.947096	8.000615	7.950919	8.012367	8.132915	8.339119	7.80904	8.063224	7.949173	8.194377	AI314976	NM_207219
6850877	6313	5.904829	5.951232	5.88678	5.964912	-0.06008	0.626537	-0.01368	0.911131	-0.07813	0.529051	0.064452	0.602093	-0.0464	0.706177	0.018049	0.882958	6850877	5.751133	6.049999	5.913356	5.950402	5.918196	5.985099	6.139229	5.870698	5.650414	5.938379	5.952671	6.003685	Kif6	NM_177052
6850937	6315	7.061101	6.958778	7.006047	6.924406	0.136695	0.129185	0.034372	0.681791	0.081641	0.341925	-0.04727	0.5747	0.102323	0.241051	0.055054	0.514923	6850937	7.136674	6.970261	7.076367	7.007715	7.016109	6.852509	7.04674	6.995873	6.975528	6.752806	6.997394	7.023017	Plcl2	NM_013880
6851095	6316	9.851127	9.792829	9.941017	9.946449	-0.09532	0.435387	-0.15362	0.222326	-0.00543	0.963826	-0.14819	0.237606	0.058298	0.629085	-0.08989	0.461032	6851095	10.00924	9.780296	9.763845	9.713167	9.871109	9.794212	10.1023	9.977005	9.743746	9.88936	10.11732	9.832668	Rab5a	NM_025887
6851103	6317	7.614919	7.852767	7.615084	7.846508	-0.23159	0.234366	0.006259	0.973124	-0.23142	0.234669	0.237683	0.223357	-0.23785	0.223066	-0.00016	0.999294	6851103	7.622173	7.660072	7.562513	7.776735	7.906023	7.875543	7.713011	7.690058	7.442182	7.688858	7.542763	8.307904	Kat2b	NM_020005
6851187	6321	6.093313	5.790843	5.75706	6.185387	-0.09207	0.706215	-0.39454	0.132626	-0.42833	0.106652	0.033783	0.889557	0.30247	0.235248	0.336253	0.191466	6851187	6.039546	6.021958	6.218436	5.287564	5.930639	6.154326	6.067965	5.431334	5.771882	6.322925	6.173616	6.059621	BC011426	NM_145490
6851191	6322	6.205525	5.85789	6.106246	6.002223	0.203302	0.158864	-0.14433	0.302086	0.104023	0.449568	-0.24836	0.094257	0.347635	0.028952	0.099279	0.469784	6851191	6.445823	5.990642	6.18011	5.972468	5.98083	5.620373	6.155866	6.018327	6.144546	5.957603	6.052803	5.996263	Ebi3	NM_015766
6851192	6323	6.464502	6.549721	6.606141	6.584376	-0.11987	0.037861	-0.03466	0.493086	0.021765	0.663934	-0.05642	0.275971	-0.08522	0.115378	-0.14164	0.018846	6851192	6.436234	6.53366	6.423612	6.485765	6.602376	6.561023	6.626351	6.669334	6.522738	6.623055	6.571933	6.558141	Ccdc94	NM_028381
6851193	6324	6.069943	6.184134	6.236816	6.191398	-0.12146	0.326301	-0.00726	0.951674	0.045418	0.705997	-0.05268	0.662209	-0.11419	0.354359	-0.16687	0.188755	6851193	6.132631	5.993839	6.083358	6.366096	6.22402	5.962287	6.247474	6.301808	6.161166	6.153384	6.04328	6.377529	Shd	NM_001159523
6851198	6325	6.880671	6.789939	6.85969	6.891944	-0.01127	0.842168	-0.10201	0.099755	-0.03225	0.572431	-0.06975	0.238885	0.090732	0.136428	0.020981	0.711832	6851198	6.914241	6.877849	6.849922	6.805827	6.722016	6.841974	6.857688	6.764596	6.956785	6.940791	6.914102	6.82094	Fsd1	NM_183178
6851200	6326	7.851536	8.022212	7.887694	7.878781	-0.02725	0.806208	0.14343	0.218613	0.008913	0.935922	0.134517	0.245915	-0.17068	0.150813	-0.03616	0.745115	6851200	7.845911	7.813811	7.894887	8.019142	8.072209	7.975284	8.039208	7.88789	7.735985	7.658638	7.912037	8.065669	Mpnd	NM_026530
6851204	6327	6.545523	6.525066	6.41052	6.359979	0.185543	0.182839	0.165087	0.230581	0.050541	0.701561	0.114546	0.394225	0.020457	0.876242	0.135002	0.319603	6851204	6.560461	6.439598	6.636509	6.535851	6.620821	6.418526	6.613862	6.101425	6.516274	6.29783	6.389713	6.392395	Chaf1a	NM_013733
6851207	6328	7.502794	7.58839	7.497609	7.47388	0.028914	0.691167	0.11451	0.141406	0.023729	0.743981	0.09078	0.231934	-0.0856	0.257341	0.005185	0.942924	6851207	7.592807	7.428668	7.486907	7.572946	7.664145	7.528078	7.520605	7.606523	7.3657	7.474938	7.52743	7.419272	Hdgfrp2	NM_008233
6851210	6329	6.703512	6.636706	6.783095	6.405712	0.297799	0.221496	0.230994	0.33384	0.377383	0.131436	-0.14639	0.532837	0.066806	0.773716	-0.07958	0.732258	6851210	7.045753	6.766104	6.298678	6.701429	6.804174	6.404578	6.830932	6.544488	6.973866	6.112423	6.621893	6.482821	Tnfaip8l	NM_025566
6851227	6330	7.907738	7.799537	7.733954	7.797939	0.109798	0.603584	0.001598	0.993916	-0.06398	0.760847	0.065583	0.7551	0.1082	0.608773	0.173783	0.417188	6851227	7.784553	7.796056	8.142604	8.004563	7.837636	7.556413	7.697295	8.065083	7.439485	7.625603	8.067929	7.700286	Fem1a	NM_010192
6851232	6331	7.156869	7.003639	6.908898	6.198555	0.958313	0.026914	0.805084	0.052767	0.710343	0.079996	0.094741	0.795996	0.15323	0.67692	0.24797	0.503989	6851232	6.978658	6.995842	7.496106	6.696556	7.424728	6.889633	7.294043	6.347796	7.084856	5.695492	6.747001	6.153173	Uhrf1	NM_010931
6851234	6332	7.455043	7.460725	7.451288	7.501029	-0.04599	0.612723	-0.0403	0.656673	-0.04974	0.584545	0.009437	0.916594	-0.00568	0.949716	0.003756	0.966747	6851234	7.441254	7.444181	7.479695	7.486413	7.511857	7.383905	7.364178	7.671997	7.317688	7.560318	7.502845	7.439925	Kdm4b	NM_172132
6851269	6334	7.550491	7.537781	7.57613	7.708405	-0.15791	0.384089	-0.17062	0.348921	-0.13228	0.462706	-0.03835	0.828665	0.012711	0.942738	-0.02564	0.884863	6851269	7.666937	7.356459	7.628078	7.495498	7.452368	7.665476	7.727814	7.815117	7.185458	7.772881	7.801352	7.550982	Ndufa11	NM_027244
6851273	6335	7.393074	7.479785	7.506544	7.536557	-0.14348	0.012493	-0.05677	0.240236	-0.03001	0.52131	-0.02676	0.566405	-0.08671	0.088662	-0.11347	0.034947	6851273	7.447009	7.382288	7.349924	7.493661	7.466727	7.478967	7.514019	7.574072	7.43154	7.54658	7.466786	7.596304	Dus3l	NM_144858
6851291	6336	7.263054	7.465822	7.468418	7.548425	-0.28537	0.053902	-0.0826	0.531732	-0.08001	0.544388	-0.0026	0.98412	-0.20277	0.147313	-0.20536	0.14285	6851291	7.213817	7.362363	7.212982	7.516372	7.546259	7.334836	7.61958	7.614007	7.171666	7.505446	7.480927	7.658901	Clpp	NM_017393
6851292	6337	6.762817	6.883961	7.014719	7.069981	-0.30716	0.092059	-0.18602	0.279991	-0.05526	0.73955	-0.13076	0.438938	-0.12114	0.472112	-0.2519	0.155265	6851292	6.561733	7.114359	6.612358	6.873739	6.852384	6.925761	7.005686	7.059247	6.979224	6.861022	7.014844	7.334077	Alkbh7	NM_025538
6851303	6338	8.294717	8.435026	8.280272	8.394427	-0.09971	0.458883	0.040599	0.759477	-0.11415	0.399003	0.154754	0.261653	-0.14031	0.305397	0.014445	0.913025	6851303	8.085172	8.272039	8.52694	8.494438	8.477876	8.332764	8.295919	8.445702	8.099196	8.349907	8.317973	8.5154	Crb3	NM_177638
6851320	6340	8.607405	8.636171	8.48177	8.408323	0.199082	0.026378	0.227848	0.014459	0.073447	0.345541	0.1544	0.068185	-0.02877	0.704902	0.125635	0.124781	6851320	8.562411	8.576038	8.683767	8.605846	8.794001	8.508665	8.52017	8.517531	8.40761	8.455007	8.416494	8.353468	Trip10	NM_134125
6851322	6341	6.568758	6.4022	6.696242	5.608876	0.959882	0.020029	0.793325	0.043645	1.087366	0.011187	-0.29404	0.400965	0.166558	0.628904	-0.12748	0.710586	6851322	7.108097	6.391867	6.20631	6.417538	6.707989	6.081074	7.059777	6.23421	6.794738	5.374426	6.065637	5.386564	Vav1	NM_011691
6851324	6342	8.309341	8.106785	8.344299	7.252169	1.057173	0.023054	0.854616	0.053157	1.09213	0.019991	-0.23751	0.546276	0.202556	0.605706	-0.03496	0.928405	6851324	8.47331	8.228722	8.225992	8.102206	8.521454	7.696695	8.762286	7.785887	8.484724	6.911268	7.990361	6.854877	Emr1	NM_010130
6851605	6343	7.455342	7.473503	7.563729	7.596	-0.14066	0.231808	-0.1225	0.292479	-0.03227	0.774142	-0.09023	0.430623	-0.01816	0.871469	-0.10839	0.347947	6851605	7.337624	7.45435	7.574052	7.36104	7.573193	7.486276	7.603941	7.578716	7.508531	7.553833	7.821484	7.412683	Fert2	NM_001037997
6851669	6344	8.903951	9.103651	9.025641	9.089734	-0.18578	0.195863	0.013917	0.918413	-0.06409	0.639419	0.07801	0.569836	-0.1997	0.167756	-0.12169	0.382328	6851669	9.011793	9.001343	8.698716	8.921255	9.135602	9.254096	8.988297	9.134568	8.954059	9.276257	9.08932	8.903626	Man2a1	NM_008549
6851734	6345	8.693414	8.661559	8.622595	8.594738	0.098676	0.395812	0.066821	0.560328	0.027857	0.806438	0.038964	0.732289	0.031855	0.779458	0.07082	0.537651	6851734	8.722777	8.664808	8.692658	8.619343	8.878907	8.486427	8.718136	8.684808	8.46484	8.535808	8.725906	8.5225	Ppp4r1	NM_146081
6851763	6346	7.12153	7.247938	7.255398	7.029055	0.092475	0.21603	0.218883	0.013007	0.226343	0.011057	-0.00746	0.916375	-0.12641	0.103645	-0.13387	0.087724	6851763	7.154505	7.034914	7.175171	7.233636	7.325683	7.184494	7.148692	7.386117	7.231384	6.965413	7.062094	7.059657	ORF19	NM_026833
6851764	6347	6.266057	6.254551	6.251178	6.129757	0.1363	0.068415	0.124794	0.090109	0.121421	0.097636	0.003373	0.959735	0.011506	0.863385	0.014879	0.824031	6851764	6.228818	6.273638	6.295716	6.143956	6.355392	6.264306	6.222291	6.177245	6.353999	6.094985	6.205502	6.088784	Ddx11	NM_001003919
6851897	6351	6.086441	5.93008	6.078731	5.671909	0.414532	0.138569	0.258171	0.335546	0.406821	0.145083	-0.14865	0.57152	0.156361	0.552173	0.00771	0.976339	6851897	6.213496	6.164218	5.881609	5.943192	6.26289	5.584159	6.446254	5.965448	5.82449	5.448136	6.0834	5.484192	Epb4.1l3	NM_013813
6851907	6352	6.476608	6.526795	6.570298	6.635701	-0.15909	0.320238	-0.10891	0.488887	-0.0654	0.674621	-0.0435	0.779368	-0.05019	0.746763	-0.09369	0.549978	6851907	6.546995	6.588422	6.294407	6.445005	6.572018	6.563362	6.532677	6.882091	6.296126	6.491063	6.755868	6.660172	Zfp161	NM_009547
6852031	6354	6.245011	6.163373	6.048988	6.233886	0.011125	0.990234	-0.07051	0.938176	-0.1849	0.839021	0.114384	0.899907	0.081638	0.928451	0.196023	0.829508	6852031	5.192573	5.676888	7.865572	7.225749	6.019423	5.244946	6.884729	5.841802	5.420434	5.520977	7.172318	6.40023	Myom1	NM_010867
6852034	6355	7.684901	7.693161	7.674068	7.62188	0.063021	0.71123	0.071281	0.675797	0.052188	0.758843	0.019093	0.910331	-0.00826	0.961127	0.010833	0.949037	6852034	7.852055	7.691434	7.511213	7.917687	7.668681	7.493115	7.635187	7.845881	7.541135	7.366126	7.631858	7.867655	Lpin2	NM_022882
6852056	6357	7.115762	6.878327	6.982999	6.535133	0.580629	0.01256	0.343194	0.094975	0.447866	0.038683	-0.10467	0.579566	0.237435	0.226655	0.132762	0.48487	6852056	7.199433	7.161125	6.986727	6.8006	7.053133	6.781248	6.986867	6.685245	7.276886	6.468579	6.833901	6.302919	Fam179a	NM_177087
6852068	6358	11.59348	11.51143	11.32745	10.3972	1.196277	0.003703	1.114223	0.005476	0.93025	0.013671	0.183973	0.551086	0.082053	0.788406	0.266027	0.394504	6852068	11.6791	11.65104	11.4503	11.25105	11.79428	11.48895	11.60992	10.6236	11.74884	10.28209	10.67478	10.23474	Clip4	NM_030179
6852130	6359	9.60339	9.654951	9.520099	9.437928	0.165462	0.195317	0.217023	0.10093	0.082171	0.502708	0.134852	0.28269	-0.05156	0.671327	0.08329	0.497079	6852130	9.619821	9.59879	9.591558	9.598401	9.714664	9.651788	9.80871	9.390056	9.361532	9.294031	9.488694	9.531059	Ypel5	NM_027166
6852144	6360	10.13446	10.03841	10.04669	10.06688	0.067577	0.658627	-0.02847	0.851589	-0.02019	0.894378	-0.00828	0.95657	0.096042	0.532696	0.087765	0.567785	6852144	10.16799	10.18859	10.04679	9.991346	10.16232	9.961577	10.31835	9.803654	10.01807	9.82306	10.22293	10.15465	Lbh	NM_029999
6852181	6361	8.07258	7.569149	7.698939	7.57568	0.496899	0.136867	-0.00653	0.983195	0.123259	0.692485	-0.12979	0.677257	0.50343	0.132456	0.373641	0.248991	6852181	8.770127	7.635495	7.812117	7.550761	7.870342	7.286345	7.612985	7.564936	7.918896	7.633239	7.757566	7.336236	Ehd3	NM_020578
6852214	6362	7.752826	7.893819	7.789954	7.67103	0.081797	0.461293	0.22279	0.068123	0.118924	0.293186	0.103865	0.354588	-0.14099	0.218984	-0.03713	0.734482	6852214	7.602465	7.760442	7.895572	7.765078	8.033611	7.882769	7.958845	7.763908	7.647109	7.619525	7.721449	7.672115	Spast	NM_016962
6852215	6363	7.934274	7.933245	7.98275	7.972468	-0.03819	0.466956	-0.03922	0.455462	0.010282	0.842241	-0.0495	0.351219	0.001029	0.984093	-0.04848	0.360774	6852215	7.890231	7.918904	7.993687	7.902803	8.038946	7.857987	7.99504	7.923588	8.029622	7.95305	7.974131	7.990223	Slc30a6	NM_144798
6852220	6364	8.382824	8.581558	8.380219	8.582962	-0.20014	0.180597	-0.0014	0.99204	-0.20274	0.175603	0.201339	0.178279	-0.19873	0.183341	0.002605	0.985233	6852220	8.412474	8.482984	8.253015	8.45356	8.600394	8.69072	8.587392	8.469799	8.083466	8.560499	8.477541	8.710847	Yipf4	NM_026417
6852222	6365	9.302029	9.269913	9.217674	9.14618	0.155849	0.060435	0.123733	0.121077	0.071494	0.345625	0.052239	0.484925	0.032116	0.664542	0.084355	0.270998	6852222	9.353159	9.301486	9.251442	9.220273	9.377883	9.211584	9.287621	9.210665	9.154737	9.078609	9.286332	9.073599	Birc6	NM_007566
6852225	6366	6.734745	6.835818	6.876373	6.733347	0.001398	0.991094	0.102471	0.423148	0.143026	0.272596	-0.04056	0.746921	-0.10107	0.429245	-0.14163	0.27696	6852225	6.803962	6.785451	6.614822	6.732029	6.990277	6.785148	7.048283	6.688177	6.89266	6.685856	6.91409	6.600095	Ttc27	NM_152817
6852229	6367	7.726487	7.463216	7.611666	7.575908	0.150579	0.698759	-0.11269	0.771624	0.035759	0.926436	-0.14845	0.702774	0.263271	0.502886	0.11482	0.767458	6852229	7.683376	7.653551	7.842533	7.553331	7.801137	7.035179	7.959681	7.849036	7.026282	7.535031	6.947738	8.244954	Ltbp1	NM_019919
6852235	6368	9.110966	9.233009	9.085517	9.077614	0.033351	0.809648	0.155394	0.279462	0.007902	0.954404	0.147492	0.30288	-0.12204	0.388868	0.025449	0.854051	6852235	9.083982	9.082249	9.166666	9.063581	9.124799	9.510646	9.167376	8.951999	9.137175	8.870831	9.150799	9.211213	Rasgrp3	NM_207246
6852361	6369	6.408157	5.970273	5.73472	5.596601	0.811556	0.137305	0.373672	0.468903	0.138119	0.785829	0.235553	0.644583	0.437884	0.398974	0.673437	0.207848	6852361	5.255902	7.12251	6.846059	5.639081	6.02415	6.247589	5.665724	5.426579	6.111857	6.110556	5.483539	5.195708	Vit	NM_028813
6852382	6370	7.358562	7.574373	7.438133	7.343782	0.01478	0.936668	0.230591	0.236674	0.094351	0.61487	0.136241	0.471434	-0.21581	0.265487	-0.07957	0.670593	6852382	7.060072	7.598336	7.417277	7.252874	7.851972	7.618274	7.484102	7.493258	7.337038	7.20234	7.33654	7.492466	Ccdc75	NM_181649
6852389	6371	7.343684	7.554375	7.463734	7.438515	-0.09483	0.279815	0.11586	0.19445	0.025219	0.765758	0.090641	0.300079	-0.21069	0.032852	-0.12005	0.180441	6852389	7.230141	7.402355	7.398556	7.539474	7.552825	7.570825	7.539266	7.468419	7.383516	7.285799	7.433239	7.596507	2410091C	NM_028611
6852399	6372	5.696431	5.962352	5.781281	6.841361	-1.14493	0.072404	-0.87901	0.150942	-1.06008	0.091799	0.181071	0.751969	-0.26592	0.643794	-0.08485	0.881966	6852399	6.020536	5.695874	5.372884	5.795474	6.015426	6.076155	5.957339	5.444955	5.941548	8.285187	5.867903	6.370994	Qpct	NM_027455
6852438	6373	5.952392	5.9391	5.944036	6.217093	-0.2647	0.118435	-0.27799	0.103568	-0.27306	0.10887	-0.00494	0.974785	0.013292	0.932176	0.008357	0.957323	6852438	6.202702	5.7464	5.908075	5.946048	6.187356	5.683896	6.014875	5.912891	5.904341	6.31549	6.07055	6.265239	Fam82a1	NM_201361
6852471	6374	7.768354	7.855369	7.710909	8.030422	-0.26207	0.315456	-0.17505	0.494751	-0.31951	0.227968	0.14446	0.571275	-0.08701	0.731354	0.057445	0.820307	6852471	7.57927	7.609448	8.116343	7.935767	7.831751	7.798588	7.906993	7.725705	7.500028	8.009776	7.570364	8.511126	Galm	NM_176963

6852475	6375	6.106954	6.115917	5.958072	5.826184	0.28077	0.226169	0.289733	0.213027	0.131888	0.555061	0.157845	0.482091	-0.00896	0.967638	0.148882	0.506565	6852475	6.07938	6.16956	6.071922	6.092392	5.952871	6.302488	6.294757	6.131016	5.448442	5.784791	5.650906	6.042854	Gemin6	NM_026053
6852483	6376	8.073569	8.010559	8.060441	8.130389	-0.05682	0.741898	-0.11983	0.492537	-0.06995	0.685704	-0.04988	0.772301	0.06301	0.715168	0.013128	0.939142	6852483	7.953442	8.059813	8.207452	7.987031	7.951789	8.092856	7.746368	8.414933	8.020023	8.045301	8.010016	8.33585	Morn2	NM_194269
6852542	6377	6.777835	6.695589	6.451265	5.925965	0.85187	0.083221	0.769624	0.111643	0.5253	0.25716	0.244325	0.585943	0.082246	0.853257	0.326571	0.469886	6852542	6.304988	7.019356	7.009162	6.270571	7.131219	6.684978	6.832428	5.838828	6.682538	6.059819	6.536376	5.1817	Tmem178	NM_026516
6852655	6378	7.372682	6.880095	7.136846	7.175823	0.196859	0.687039	-0.29573	0.54768	-0.03898	0.936096	-0.25675	0.600619	0.492587	0.326314	0.235836	0.630136	6852655	7.367716	6.883011	7.86732	6.789592	7.383181	6.467512	7.607981	7.29511	6.507448	8.033799	6.751139	6.742532	Pkdcc	NM_134117
6852676	6379	7.723721	7.77212	7.61164	7.533381	0.19034	0.020157	0.238739	0.006718	0.078259	0.268605	0.16048	0.040705	-0.0484	0.483207	0.112081	0.127057	6852676	7.844336	7.680089	7.646739	7.722536	7.887951	7.705874	7.639257	7.612419	7.583245	7.465714	7.588708	7.545721	Eml4	NM_001114361
6852695	6380	10.29586	10.29235	10.20645	10.06756	0.228297	0.004826	0.22479	0.00525	0.138883	0.046923	0.085907	0.184686	0.003507	0.954205	0.089413	0.169284	6852695	10.32963	10.31456	10.24339	10.33002	10.33535	10.21169	10.14495	10.22095	10.25344	9.95618	10.08323	10.16328	Mta3	NM_054082
6852751	6382	6.552192	6.754165	6.494256	6.695392	-0.1432	0.371983	0.058772	0.70804	-0.20114	0.220717	0.259909	0.124414	-0.20197	0.21898	0.057936	0.711974	6852751	6.418442	6.597541	6.640592	6.728421	6.768453	6.76562	6.644136	6.22707	6.611562	6.519442	6.568501	6.998234	Dync2li1	NM_172256
6852767	6384	6.037023	5.94619	5.92809	6.207449	-0.17043	0.076523	-0.26126	0.01431	-0.27936	0.010351	0.018099	0.83448	0.090833	0.310182	0.108933	0.230023	6852767	5.982046	5.944074	6.184949	5.814533	6.029712	5.994324	5.998498	5.963537	5.822236	6.195728	6.156083	6.270535	Ppm1b	NM_001159496
6852820	6386	7.518021	7.59702	7.230253	7.55571	-0.03769	0.633427	0.04131	0.601698	-0.32546	0.002685	0.366767	0.001315	-0.079	0.32916	0.287768	0.00535	6852820	7.447347	7.590792	7.515925	7.532374	7.640386	7.618301	7.185523	7.185832	7.319404	7.453074	7.718462	7.495594	Prkce	NM_011104
6852836	6387	9.683006	9.862951	9.461863	9.805631	-0.12262	0.694291	0.05732	0.853657	-0.34377	0.28627	0.401088	0.219229	-0.17994	0.566342	0.221143	0.483335	6852836	9.900085	9.768708	9.380226	10.26483	9.79908	9.524943	9.015068	10.07276	9.297762	9.6492	9.993199	9.774494	Epas1	NM_010137
6852845	6388	7.62676	7.729659	7.584685	7.878387	-0.25163	0.054079	-0.14873	0.219146	-0.2937	0.03004	0.144973	0.229919	-0.1029	0.383267	0.042075	0.715843	6852845	7.620555	7.589144	7.670581	7.779879	7.891764	7.517333	7.596481	7.729887	7.427688	7.817171	7.807584	8.010406	Rhoq	NM_145491
6852849	6389	10.72454	10.84377	10.91826	10.87799	-0.15344	0.255757	-0.03421	0.791823	0.04027	0.756259	-0.07448	0.568817	-0.11923	0.36939	-0.19371	0.160858	6852849	10.68132	10.69435	10.79796	10.78684	11.10019	10.64429	11.01548	11.02317	10.71612	10.83237	10.96071	10.84088	Cript	NM_019936
6852856	6390	7.811056	7.866319	7.735253	8.11968	-0.30862	0.140117	-0.25336	0.215675	-0.38443	0.075679	0.131066	0.506438	-0.05526	0.776793	0.075803	0.698033	6852856	7.780158	7.872567	7.780444	7.850222	7.852502	7.896233	7.80564	7.990981	7.409138	7.903474	7.934258	8.521307	Socs5	NM_019654
6852874	6391	7.927445	7.6673	7.738986	7.776274	0.151171	0.311901	-0.10897	0.458927	-0.03729	0.796796	-0.07169	0.622598	0.260144	0.100332	0.188458	0.215333	6852874	7.998116	7.787731	7.996487	7.647197	7.835701	7.519003	7.830606	7.876143	7.51021	7.556151	7.846737	7.925934	Ttc7	NM_028639
6852882	6392	10.88799	11.04632	10.98737	11.07204	-0.18405	0.177745	-0.02572	0.841563	-0.08467	0.515812	0.058953	0.648637	-0.15833	0.239374	-0.09938	0.447974	6852882	10.79107	10.81421	11.05868	10.95891	11.12933	11.05072	11.17374	10.99745	10.79091	11.01445	10.94393	11.25774	Epcam	NM_008532
6852887	6393	7.146083	7.480729	7.467984	6.825615	0.320469	0.062445	0.655114	0.002218	0.642369	0.002489	0.012745	0.933548	-0.33465	0.053786	-0.3219	0.061511	6852887	7.008536	7.330052	7.099662	7.436219	7.578957	7.42701	7.608715	7.201045	7.594192	6.5854	6.933343	6.958101	Msh2	NM_008628
6852902	6394	6.868324	7.030164	6.919426	6.83621	0.032115	0.79929	0.193955	0.151034	0.083216	0.514995	0.110739	0.391179	-0.16184	0.221845	-0.0511	0.686737	6852902	6.773576	6.886862	6.944535	6.761304	7.261436	7.067753	6.975299	6.912382	6.870596	6.692805	6.921683	6.894141	Msh6	NM_010830
6852924	6395	7.37935	7.299325	7.233761	7.071595	0.307755	0.006404	0.22773	0.026736	0.162166	0.089937	0.065564	0.458027	0.080025	0.369142	0.145589	0.121639	6852924	7.496489	7.318229	7.323332	7.128346	7.42255	7.347079	7.26221	7.288924	7.150149	7.014087	7.070694	7.130005	Foxn2	NM_180974
6852929	6396	7.844645	8.073894	7.953059	7.792852	0.051793	0.581499	0.281042	0.0143	0.160207	0.113526	0.120835	0.217042	-0.22925	0.034585	-0.10841	0.263627	6852929	7.752731	7.897048	7.884156	8.083472	8.188945	7.949265	7.985804	8.07548	7.797892	7.691506	7.829133	7.857917	Klraq1	NM_028658
6852934	6397	6.758859	6.997796	6.801592	6.574257	0.184602	0.162994	0.423539	0.007789	0.227335	0.095125	0.196204	0.141121	-0.23894	0.081949	-0.04273	0.731294	6852934	6.671482	6.785633	6.819461	7.004312	7.209596	6.779479	6.642704	6.915789	6.846282	6.711619	6.511288	6.499863	Ston1	NM_029858
6853197	6400	7.162725	7.195447	7.359242	7.341665	-0.17894	0.301946	-0.14622	0.393599	0.017577	0.916361	-0.1638	0.342068	-0.03272	0.845136	-0.19652	0.260179	6853197	6.945556	7.242363	7.300257	6.881586	7.466983	7.237771	7.357649	7.196247	7.523831	7.285026	7.296884	7.443086	Mettl4	NM_176917
6853248	6401	6.639095	6.617026	6.971599	6.925955	-0.28686	0.27253	-0.30893	0.240126	0.045644	0.855946	-0.35457	0.183364	0.02207	0.929996	-0.3325	0.209161	6853248	6.342744	6.700568	6.873974	6.442416	7.013395	6.395266	6.851506	7.14375	6.919542	6.888418	7.318077	6.571371	Tfb1m	NM_146074
6853388	6403	8.035457	8.237866	7.650322	8.147814	-0.11236	0.656429	0.090052	0.720818	-0.49749	0.075048	0.587544	0.042139	-0.20241	0.429452	0.385135	0.151985	6853388	7.776023	8.213614	8.116733	8.169664	8.366546	8.177388	7.775456	7.552056	7.623453	8.10088	7.646842	8.695719	5730437N	NM_027457
6853467	6405	6.464547	6.532571	6.473257	6.534367	-0.06982	0.28045	-0.0018	0.976984	-0.06111	0.34066	0.059314	0.354233	-0.06802	0.292109	-0.00871	0.88876	6853467	6.414112	6.53684	6.44269	6.522865	6.5145	6.560349	6.423287	6.507263	6.489221	6.608163	6.392249	6.602688	Serac1	NM_001111017
6853661	6408	9.1564	9.223274	9.08838	8.998403	0.157997	0.111579	0.224871	0.034453	0.089977	0.338368	0.134894	0.165388	-0.06687	0.470876	0.068021	0.463559	6853661	9.168084	9.144516	9.156601	9.146447	9.392297	9.131078	9.172142	9.00583	9.087167	8.871711	9.140944	8.982554	Qk	NM_021881
6853762	6410	7.52424	7.510115	7.526037	7.375165	0.149074	0.062314	0.13495	0.08569	0.150871	0.05983	-0.01592	0.82295	0.014125	0.842601	-0.0018	0.97982	6853762	7.469481	7.49259	7.610648	7.519361	7.581303	7.429681	7.599218	7.481308	7.497584	7.285808	7.502514	7.337174	Map3k4	NM_011948
6853780	6413	9.228645	9.1975	9.15268	9.017898	0.210747	0.049793	0.179603	0.084673	0.134782	0.178057	0.04482	0.636618	0.031145	0.741757	0.075965	0.429454	6853780	9.229466	9.252716	9.203753	9.09409	9.313708	9.184703	9.204618	9.07814	9.175282	8.816171	9.166878	9.070644	Igf2r	NM_010515
6853805	6414	5.838757	5.707888	6.055414	5.989263	-0.15051	0.566318	-0.28137	0.295948	0.066151	0.799289	-0.34753	0.204616	0.130869	0.6171	-0.21666	0.41431	6853805	5.822201	5.803576	5.890495	5.971756	5.192933	5.958975	5.663885	6.35717	6.145186	6.244792	5.92268	5.800317	Tcte2	NM_022311
6853849	6415	7.563678	7.606071	7.829027	7.756038	-0.19236	0.16479	-0.14997	0.267407	0.072989	0.577777	-0.22296	0.114303	-0.04239	0.744811	-0.26535	0.067962	6853849	7.562197	7.542772	7.586064	7.678041	7.550994	7.589178	7.920504	7.969849	7.596729	7.647002	7.609934	8.011179	Dact2	NM_172826
6853899	6417	7.547557	7.421376	7.219086	6.596329	0.951229	0.024122	0.825048	0.042733	0.622757	0.106815	0.20229	0.57143	0.126181	0.722374	0.328471	0.366052	6853899	7.708404	7.733622	7.200646	7.216856	8.106894	6.940379	7.608237	7.080415	6.968606	6.199199	6.885465	6.704322	Thbs2	NM_011581
6853910	6419	7.926247	8.018772	7.898784	7.876882	0.049364	0.640229	0.141889	0.200251	0.021902	0.834786	0.119987	0.271719	-0.09252	0.389273	0.027462	0.793859	6853910	7.953218	7.886845	7.938677	7.960616	8.220485	7.875214	7.797045	8.091684	7.807624	7.888565	7.890881	7.851201	Phf10	NM_024250
6853911	6420	6.460074	6.74774	6.334952	6.487998	-0.02792	0.91627	0.259742	0.342413	-0.15305	0.568504	0.412788	0.147407	-0.28767	0.296123	0.125121	0.639892	6853911	6.229949	6.549438	6.600834	6.824739	6.771173	6.647309	6.376729	6.496619	6.131509	6.319817	6.030869	7.113309	Tcte3	NM_011560
6853934	6421	6.543604	6.870447	6.378701	6.516486	0.027118	0.888411	0.353961	0.095328	-0.13779	0.482765	0.491746	0.030336	-0.32684	0.118988	0.164904	0.404097	6853934	6.627268	6.614101	6.389444	6.67883	7.13898	6.793531	6.431322	6.267654	6.437126	6.215419	6.907477	6.426562	Dll1	NM_007865
6853944	6422	9.855207	9.991829	9.873513	9.834954	0.020253	0.854748	0.156875	0.181224	0.038559	0.728197	0.118316	0.301479	-0.13662	0.237953	-0.01831	0.868554	6853944	9.789173	9.787146	9.989302	9.951558	10.04867	9.975258	10.09432	9.883529	9.642689	9.787836	9.850475	9.866551	Psmb1	NM_011185
6853948	6423	7.902426	7.998396	7.887743	7.945383	-0.04296	0.55517	0.053013	0.469109	-0.05764	0.432623	0.110653	0.151369	-0.09597	0.206211	0.014684	0.838555	6853948	7.794136	7.964736	7.948407	7.909853	8.008181	8.077154	7.92208	7.972741	7.768407	7.994226	7.908374	7.933548	Pdcd2	NM_008799
6853951	6424	6.692025	6.767323	6.561527	6.717264	-0.02524	0.75733	0.050058	0.543644	-0.15574	0.083936	0.205796	0.031259	-0.0753	0.36802	0.130498	0.136859	6853951	6.670482	6.687314	6.718278	6.75806	6.707038	6.83687	6.45553	6.688877	6.540173	6.689136	6.597296	6.865361	Prdm9	NM_144809
6853960	6425	7.814021	7.977588	7.87372	7.791362	0.022659	0.819319	0.186227	0.088336	0.082358	0.415861	0.103869	0.310749	-0.16357	0.126779	-0.0597	0.551294	6853960	7.788037	7.778377	7.875648	8.04303	7.981024	7.908711	7.96079	8.010051	7.650318	7.765225	7.707774	7.901086	Rgmb	NM_178615
6854031	6426	9.249372	9.257097	9.200383	9.137208	0.112164	0.09825	0.119889	0.08053	0.063175	0.322743	0.056714	0.371816	-0.00772	0.900656	0.048989	0.437471	6854031	9.239685	9.361622	9.14681	9.192884	9.265595	9.312811	9.229078	9.213417	9.158654	9.058666	9.194148	9.15881	Lnpep	NM_172827
6854108	6435	5.919695	5.806697	5.990669	5.746026	0.173669	0.613351	0.060672	0.858853	0.244644	0.480137	-0.18397	0.592836	0.112997	0.741128	-0.07097	0.835265	6854108	6.122429	5.822811	5.813844	5.689058	5.687109	6.043925	6.801263	5.510689	5.660056	6.076522	5.542558	5.618997	V1rf5	NM_134202
6854188	6438	10.69988	10.85749	10.72311	10.71546	-0.01558	0.927316	0.142033	0.415667	0.00765	0.964259	0.134383	0.440211	-0.15761	0.368732	-0.02323	0.891839	6854188	10.49492	10.90821	10.69652	10.72106	10.8525	10.99892	10.97823	10.61153	10.57957	10.6073	10.55881	10.98027	6330416L	NM_176962
6854231	6439	7.510951	7.524876	7.508444	7.459545	0.051406	0.634731	0.065331	0.547794	0.048899	0.651108	0.016433	0.878491	-0.01393	0.896896	0.002507	0.981375	6854231	7.33418	7.653027	7.545646	7.558326	7.525101	7.491202	7.712654	7.40748	7.405197	7.392492	7.438899	7.547244	A630033E	NM_001110254
6854239	6440	7.019255	6.963643	7.38781	7.032229	-0.01297	0.938668	-0.06859	0.68574	0.355581	0.061246	-0.42417	0.031831	0.055612	0.742382	-0.36855	0.054112	6854239	7.318194	6.864226	6.875345	6.948945	6.988647	6.953337	7.654451	7.231672	7.277307	6.870467	7.251764	6.974457	Zfp40	NM_009555
6854256	6441	7.456185	7.428374	7.547955	7.407308	0.048877	0.539847	0.021066	0.789545	0.140647	0.102624	-0.11958	0.155825	0.02781	0.725036	-0.09177	0.263616	6854256	7.444497	7.462194	7.461863	7.462687	7.371525	7.450911	7.675383	7.442134	7.526348	7.488738	7.250533	7.482653	Zfp13	NM_011747
6854259	6442	7.413222	7.537408	7.528725	7.463215	-0.04999	0.624541	0.074192	0.471737	0.06551	0.523614	0.008683	0.931741	-0.12419	0.241749	-0.1155	0.273477	6854259	7.500309	7.382017	7.35734	7.448794	7.638123	7.525306	7.684904	7.498106	7.403165	7.306209	7.478857	7.60458	Thoc6	NM_001008425
6854260	6443	8.296045	7.783643	8.004844	8.032215	0.26383	0.401087	-0.24857	0.427702	-0.02737	0.928966	-0.2212	0.478461	0.512402	0.123332	0.2912	0.356375	6854260	8.572854	8.242777	8.072503	7.490604	8.405097	7.455227	8.364312	8.007059	7.643162	8.080709	7.796622	8.219313	Tnfrsf12	NM_013749
6854270	6446	8.021814	7.966196	8.307496	8.296197	-0.27438	0.363955	-0.33	0.280389	0.011299	0.969353	-0.3413	0.265455	0.055618	0.850168	-0.28568	0.345593	6854270	8.183687	7.722416	8.15934	8.269666	7.7956	7.833323	8.513144	8.47331	7.936034	8.00266	8.016088	8.869843	Paqr4	NM_023824
6854271	6447	6.173114	6.057211	6.087886	6.229416	-0.0563	0.533468	-0.1722	0.08174	-0.14153	0.140534	-0.03067	0.732108	0.115902	0.2172	0.085227	0.353466	6854271	6.22295	6.138945	6.157446	6.124316	5.870099	6.177219	6.198078	6.09988	5.965701	6.227785	6.181218	6.279246	Kremen2	NM_028416
6854273	6448	7.458242	7.194732	7.57732	7.446851	0.011391	0.917927	-0.25212	0.046408	0.130469	0.257909	-0.38259	0.007275	0.263511	0.039312	-0.11908	0.298557	6854273	7.652032	7.296381	7.426314	7.329432	7.207085	7.047678	7.700118	7.555873	7.47597	7.456001	7.501642	7.382911	Flywch1	NM_153791
6854276	6449	6.834217	6.771811	6.884385	6.819865	0.014352	0.937838	-0.04805	0.794411	0.06452	0.726897	-0.11257	0.545515	0.062407	0.735438	-0.05017	0.785626	6854276	6.988075	6.79248	6.722097	6.821319	7.074228	6.419885	6.83769	6.790244	7.025222	6.703128	6.685052	7.071415	Flywch2	NM_029798
6854284	6450	6.786204	6.587382	7.096165	6.811407	-0.0252	0.931524	-0.22402	0.453316	0.284758	0.345755	-0.50878	0.111237	0.198822	0.504067	-0.30996	0.307241	6854284	7.237986	6.384332	6.736294	6.93348	6.72563	6.103037	6.895967	7.090642	7.301885	6.816363	7.078571	6.539287	Tceb2	NM_026305
6854287	6452	6.564368	6.798823	6.762645	6.750917	-0.18655	0.172419	0.047906	0.710429	0.011728	0.927267	0.036178	0.778764	-0.23445	0.096439	-0.19828	0.149924	6854287	6.471518	6.634596	6.586991	6.711309	6.991837	6.693323	6.830495	6.769907	6.687534	6.99004	6.531774	6.730938	4931440B	NM_027644
6854314	6456	7.991246	7.998839	7.923149	7.927886	0.06336	0.574664	0.070952	0.530776	-0.00474	0.966186	0.075689	0.504451	-0.00759	0.945834	0.068097	0.54704	6854314	8.019247	7.978685	7.975807	7.802925	8.241261	7.95233	8.04223	7.842717	7.884501	7.894396	7.853966	8.035297	Kctd5	NM_027008
6854320	6457	9.99926	10.10391	9.986576	10.16778	-0.16852	0.217742	-0.06387	0.62582	-0.1812	0.188242	0.117334	0.37886	-0.10465	0.430192	0.012684	0.922271	6854320	10.00285	10.01476	9.98017	10.13202	10.08872	10.09099	9.795794	10.30941	9.854524	10.0728	10.12303	10.3075	Pdpk1	NM_011062
6854335	6458	7.66938	7.622377	7.761846	7.506053	0.163328	0.078061	0.116324	0.188198	0.255793	0.013323	-0.13947	0.122829	0.047004	0.57703	-0.09247	0.285863	6854335	7.671575	7.56037	7.776196	7.659956	7.694989	7.512185	7.82748	7.745851	7.712206	7.381585	7.513232	7.623341	Amdhd2	NM_172935
6854340	6459	6.679474	6.83727	6.639378	7.055408	-0.37593	0.125687	-0.21814	0.350213	-0.41603	0.09512	0.197892	0.394402	-0.1578	0.493393	0.040096	0.859842	6854340	6.866928	6.703213	6.468281	6.983089	6.891263	6.637459	6.430117	7.158587	6.329431	6.980861	6.997861	7.187502	Tbc1d24	NM_173186
6854341	6460	7.533677	7.649331	7.474744	8.777686	-1.24401	0.019191	-1.12835	0.029182	-1.30294	0.015538	0.174588	0.692369	-0.11565	0.792668	0.058933	0.893274	6854341	7.353503	7.482846	7.764682	7.965461	7.383138	7.599395	7.582296	7.666801	7.175134	8.351277	8.124781	9.857	Dnase1l2	NM_025718
6854345	6461	6.064747	6.014735	6.031156	6.103784	-0.03904	0.716823	-0.08905	0.416228	-0.07263	0.504242	-0.01642	0.87831	0.050011	0.643077	0.033591	0.754694	6854345	6.143796	6.063062	5.987382	5.930637	6.057982	6.055587	6.052266	6.122188	5.919013	6.223599	5.865294	6.222458	Ntn3	NM_010947
6854346	6462	6.657216	6.879313	6.84362	6.624357	0.032859	0.649162	0.254956	0.006341	0.219263	0.013533	0.035693	0.621617	-0.2221	0.012727	-0.1864	0.027897	6854346	6.80753	6.510927	6.653191	6.901254	6.90192	6.834765	6.839545	6.88418	6.807136	6.696607	6.598556	6.577908	1600002H	BC072612
6854366	6464	7.890493	7.939968	7.932641	8.137622	-0.24713	0.000398	-0.19765	0.001643	-0.20498	0.001316	0.007327	0.867409	-0.04947	0.277918	-0.04215	0.350378	6854366	7.842896	7.932916	7.895668	8.001437	7.901853	7.916613	7.888347	7.997243	7.912333	8.10973	8.106339	8.196796	E4f1	NM_007893
6854369	6465	7.138494	7.30918	7.183712	7.229806	-0.09131	0.502845	0.079373	0.558888	-0.04609	0.732379	0.125468	0.363282	-0.17069	0.226106	-0.04522	0.737243	6854369	7.137315	7.125442	7.152726	7.311332	7.586731	7.029476	7.257858	7.196376	7.096902	7.137739	7.171614	7.380066	Gbl	NM_019988
6854371	6466	8.641152	8.615575	8.634268	8.362826	0.278326	0.043351	0.252749	0.061138	0.271442	0.047555	-0.01869	0.876073	0.025577	0.831144	0.006884	0.954168	6854371	8.676436	8.600402	8.646619	8.561303	8.864033	8.421389	8.736898	8.571917	8.593989	8.274	8.526687	8.287791	Traf7	NM_153792

6854378	6467	8.959608	8.837948	8.928728	8.865682	0.093925	0.233948	-0.02773	0.713729	0.063045	0.41267	-0.09078	0.248612	0.12166	0.133962	0.03088	0.683254	6854378	9.013468	8.830232	9.035123	8.782767	8.988547	8.742529	8.92177	8.972174	8.892239	8.882634	8.858527	8.855886	Tsc2	NM_011647
6854381	6468	8.211588	8.295769	8.357854	8.632512	-0.42092	0.004982	-0.33674	0.015393	-0.27466	0.036804	-0.06208	0.587139	-0.08418	0.465132	-0.14627	0.219362	6854381	8.187514	8.213976	8.233275	8.37795	8.33426	8.175096	8.361753	8.540454	8.171354	8.561762	8.817987	8.517788	Slc9a3r2	NM_023055
6854383	6469	7.022063	6.521577	7.0105	6.719472	0.302591	0.228929	-0.19789	0.419009	0.291028	0.245622	-0.48892	0.068418	0.500486	0.063316	0.011563	0.961517	6854383	7.404608	6.556034	7.105547	6.467542	6.423776	6.673413	7.341723	6.696431	6.993345	6.684326	6.861414	6.612676	Syngr3	NM_011522
6854384	6470	6.792682	6.643504	7.040384	6.628573	0.16411	0.263601	0.014932	0.915581	0.411812	0.016631	-0.39688	0.019656	0.149178	0.306226	-0.2477	0.1071	6854384	6.948308	6.557981	6.871758	6.643213	6.756778	6.530522	7.26827	6.881128	6.971755	6.538325	6.769032	6.578361	Gfer	NM_023040
6854386	6471	7.81162	7.501253	7.870701	7.421567	0.390054	0.113194	0.079686	0.725753	0.449134	0.074742	-0.36945	0.130552	0.310368	0.194728	-0.05908	0.794441	6854386	8.123791	7.471556	7.839514	7.510944	7.716043	7.276771	8.14925	7.537072	7.925781	7.436135	7.607149	7.221416	Tbl3	NM_145396
6854389	6473	7.467903	7.482318	7.856349	7.796807	-0.3289	0.186656	-0.31449	0.20463	0.059542	0.800343	-0.37403	0.139122	-0.01442	0.951081	-0.38845	0.126447	6854389	7.536515	7.239388	7.627806	7.417474	7.487724	7.541756	7.721642	8.381426	7.465978	7.571302	7.840517	7.978602	Ndufb10	NM_026684
6854393	6474	5.697376	5.832857	6.121085	6.192186	-0.49481	0.050128	-0.35933	0.132781	-0.0711	0.749058	-0.28823	0.216343	-0.13548	0.545675	-0.42371	0.083921	6854393	5.709545	5.922771	5.459812	6.106649	5.874316	5.517607	6.014936	6.438102	5.910217	6.051716	6.057469	6.467373	Fahd1	NM_023480
6854398	6475	8.331308	8.411417	8.381225	8.369504	-0.0382	0.728729	0.041912	0.703761	0.011721	0.914945	0.030192	0.78367	-0.08011	0.472809	-0.04992	0.651282	6854398	8.371211	8.261507	8.361205	8.373436	8.531482	8.329332	8.507058	8.284826	8.351791	8.295685	8.217259	8.595569	Nubp2	NM_011956
6854400	6476	8.029856	8.126313	7.767129	8.183137	-0.15328	0.277998	-0.05682	0.677509	-0.41601	0.013418	0.359183	0.025951	-0.09646	0.484814	0.262726	0.081149	6854400	7.905413	8.158198	8.025956	8.160231	8.088454	8.130253	7.62148	7.96493	7.714978	8.085936	8.01228	8.451196	Mapk8ip3	NM_013931
6854401	6477	9.714414	9.827522	9.990555	10.04614	-0.33173	0.070124	-0.21862	0.205888	-0.05559	0.735326	-0.16303	0.334612	-0.11311	0.496533	-0.27614	0.120234	6854401	9.450754	9.737484	9.955004	9.76781	9.946961	9.767796	10.03736	10.22949	9.704814	9.958802	10.11094	10.06869	Hn1l	NM_198937
6854403	6478	7.989989	8.104297	7.658018	8.148582	-0.15859	0.250412	-0.04428	0.738257	-0.49056	0.004997	0.446279	0.008235	-0.11431	0.397857	0.331971	0.031923	6854403	7.909726	7.947252	8.112989	8.164439	8.107041	8.041411	7.450656	7.85849	7.664907	8.125083	7.958436	8.362226	Cramp1l	NM_020608
6854404	6479	8.874283	8.94166	8.910765	9.00303	-0.12875	0.529251	-0.06137	0.761943	-0.09226	0.650033	0.030894	0.878523	-0.06738	0.739608	-0.03648	0.856814	6854404	8.867731	9.002432	8.752686	8.884325	8.952433	8.988221	9.212523	8.566205	8.953568	8.832178	9.378056	8.798856	Tmem204	NM_001001183
6854405	6480	6.614954	6.612912	6.716262	6.562873	0.052081	0.408361	0.050038	0.426209	0.153388	0.033144	-0.10335	0.121624	0.002042	0.973544	-0.10131	0.128098	6854405	6.642365	6.536498	6.665999	6.5625	6.629299	6.646936	6.789359	6.776347	6.583079	6.600425	6.559444	6.528751	Telo2	NM_027880
6854414	6481	8.807503	8.876271	8.844119	9.133574	-0.32607	0.048957	-0.2573	0.104585	-0.28945	0.073469	0.032153	0.824826	-0.06877	0.63784	-0.03662	0.801083	6854414	8.838917	8.87086	8.712732	8.906088	8.903747	8.818979	9.032295	8.912151	8.58791	9.077887	8.929144	9.39369	Gnptg	NM_172529
6854416	6482	8.279354	8.604956	8.499214	8.494157	-0.2148	0.146329	0.110799	0.430692	0.005058	0.970712	0.105742	0.451224	-0.3256	0.040651	-0.21986	0.138242	6854416	8.25978	8.540044	8.038237	8.754237	8.537448	8.523183	8.479271	8.647916	8.370456	8.397873	8.563774	8.520823	Ube2i	NM_011665
6854430	6486	6.252156	6.216456	6.135453	6.085132	0.167024	0.057907	0.131324	0.120201	0.050321	0.523896	0.081002	0.314684	0.035701	0.648994	0.116703	0.160804	6854430	6.244029	6.201061	6.311379	6.167916	6.307126	6.174325	6.270346	5.980707	6.155307	6.144983	6.023772	6.086641	Chtf18	NM_145409
6854433	6488	7.243726	7.496724	7.178874	7.401787	-0.15806	0.228126	0.094937	0.455669	-0.22291	0.102942	0.317851	0.030434	-0.253	0.070119	0.064852	0.606866	6854433	7.309339	7.239603	7.182235	7.476045	7.493762	7.520366	7.40809	7.16343	6.965101	7.270522	7.321514	7.613326	Haghl	NM_026897
6854435	6489	8.672991	8.79663	8.561168	8.65742	0.015571	0.890169	0.13921	0.238261	-0.09625	0.403904	0.235462	0.063212	-0.12364	0.290446	0.111823	0.335908	6854435	8.593561	8.559845	8.865566	8.745179	8.828267	8.816444	8.698497	8.600242	8.384765	8.524908	8.673235	8.774117	Fam173a	BC096050
6854438	6490	6.164446	6.052157	5.77929	5.985493	0.178953	0.216745	0.066664	0.630834	-0.2062	0.160877	0.272868	0.075116	0.112288	0.424535	0.385156	0.020317	6854438	6.140324	6.15961	6.193403	6.152792	5.780738	6.222942	5.951914	5.833467	5.552488	6.014942	5.887655	6.053882	Metrn	NM_133719
6854444	6491	8.861667	8.970913	8.902293	8.996254	-0.13459	0.23933	-0.02534	0.816839	-0.09396	0.400669	0.068621	0.535013	-0.10925	0.33228	-0.04063	0.71116	6854444	8.843332	8.842619	8.899051	8.95356	8.997478	8.961702	9.107327	8.898421	8.70113	8.857199	8.966353	9.165209	Stub1	NM_019719
6854446	6492	6.709516	6.724387	6.713018	6.783992	-0.07448	0.600842	-0.0596	0.674435	-0.07097	0.617778	0.01137	0.935776	-0.01487	0.916076	-0.0035	0.980199	6854446	6.753428	6.531411	6.84371	6.643975	6.950379	6.578808	6.815428	6.76268	6.560945	6.677187	6.69458	6.980208	Rhbdl1	NM_144816
6854447	6493	8.584567	8.550371	8.590274	8.559154	0.025414	0.782646	-0.00878	0.923876	0.03112	0.735815	-0.0399	0.666039	0.034197	0.711035	-0.00571	0.950487	6854447	8.616793	8.552791	8.584118	8.562466	8.572258	8.516388	8.498722	8.791889	8.480211	8.421977	8.596091	8.659393	Rhot2	NM_145999
6854448	6494	6.113092	6.187826	6.193655	6.125225	-0.01213	0.743081	0.062602	0.118058	0.06843	0.091964	-0.00583	0.874546	-0.07473	0.069983	-0.08056	0.054283	6854448	6.101168	6.15475	6.083358	6.15058	6.231323	6.181576	6.214943	6.192902	6.173119	6.191177	6.061762	6.122735	Wdr90	BC043315
6854449	6495	6.637636	6.735566	6.701274	7.227439	-0.5898	0.003473	-0.49187	0.009181	-0.52616	0.006484	0.034292	0.817899	-0.09793	0.515974	-0.06364	0.670482	6854449	6.613265	6.627044	6.6726	6.899576	6.678785	6.628338	6.654161	6.959807	6.489855	7.053791	7.16148	7.467046	9530058B	BC024332
6854453	6496	7.331975	7.294328	7.262182	7.27238	0.059595	0.531448	0.021948	0.815714	-0.0102	0.913644	0.032147	0.733361	0.037646	0.690352	0.069793	0.465711	6854453	7.379892	7.337981	7.278051	7.34286	7.358888	7.181237	7.371988	7.341275	7.073282	7.153922	7.336049	7.327169	Rab40c	NM_139154
6854458	6497	7.517614	7.560672	7.491118	7.571931	-0.05432	0.648999	-0.01126	0.924347	-0.08081	0.501756	0.069554	0.561681	-0.04306	0.71756	0.026496	0.823393	6854458	7.55875	7.472858	7.521235	7.523934	7.645418	7.512665	7.278224	7.79167	7.403461	7.559557	7.566411	7.589824	Solh	NM_015830
6854460	6498	7.86098	7.873373	7.865694	7.904456	-0.04348	0.693847	-0.03108	0.777839	-0.03876	0.725346	0.007678	0.9443	-0.01239	0.910243	-0.00471	0.965783	6854460	7.87055	7.736602	7.975789	7.977646	7.779686	7.862786	7.836286	8.067315	7.693482	7.813042	7.905892	7.994433	Rab11fip	BC037132
6854462	6499	8.25308	8.099339	8.462596	8.208579	0.0445	0.687684	-0.10924	0.336012	0.254016	0.044547	-0.36326	0.009312	0.153741	0.187701	-0.20952	0.085256	6854462	8.21666	8.081503	8.461076	8.109789	8.156184	8.032043	8.341889	8.503348	8.54255	8.103702	8.349553	8.172483	Decr2	NM_011933
6854463	6500	6.041341	6.090056	5.855678	6.008495	0.032846	0.710713	0.081561	0.3678	-0.15282	0.111526	0.234378	0.025341	-0.04871	0.584276	0.185663	0.06156	6854463	6.011473	6.109111	6.00344	6.046731	6.024767	6.19867	6.009576	5.88978	5.667679	5.989253	6.049911	5.986321	Nme4	NM_019731
6854467	6501	7.329442	7.297521	7.341861	7.456824	-0.12738	0.388879	-0.1593	0.287502	-0.11496	0.434751	-0.04434	0.759257	0.031921	0.82513	-0.01242	0.931404	6854467	7.352821	7.440608	7.194898	7.372766	7.266822	7.252975	7.41345	7.611446	7.000688	7.469848	7.434109	7.466516	Arhgdig	NM_008113
6854468	6502	9.587664	9.562658	9.51796	9.676102	-0.08844	0.592656	-0.11344	0.495114	-0.15814	0.348277	0.044698	0.785396	0.025006	0.878723	0.069704	0.672178	6854468	9.556548	9.705201	9.501243	9.620966	9.698053	9.368955	9.538625	9.527306	9.487949	9.576926	9.405799	10.04558	Itfg3	NM_207217
6854487	6503	11.15214	10.77631	10.42836	11.29362	-0.14148	0.579198	-0.51731	0.067537	-0.86526	0.007681	0.347947	0.193006	0.37583	0.163273	0.723777	0.018241	6854487	11.01272	10.85558	11.58812	11.2451	10.48278	10.60105	10.5906	10.46867	10.22582	11.3964	11.2318	11.25267	Dusp1	NM_013642
6854506	6504	7.013299	7.171213	7.076212	7.094111	-0.08081	0.365716	0.077102	0.387024	-0.0179	0.837127	0.095001	0.292328	-0.15791	0.097853	-0.06291	0.476746	6854506	7.059071	7.006001	6.974825	7.210147	7.053216	7.250276	7.101295	7.220819	6.906521	7.128263	7.014445	7.139624	Nkx2-5	NM_008700
6854509	6505	9.60121	9.502113	9.605533	9.628717	-0.02751	0.812308	-0.1266	0.29139	-0.02318	0.841295	-0.10342	0.383143	0.099097	0.40241	-0.00432	0.970176	6854509	9.573585	9.57704	9.653004	9.468814	9.704646	9.332878	9.667739	9.74667	9.40219	9.571247	9.60332	9.711585	Cuta	NM_026307
6854514	6506	7.411273	7.358808	7.16265	6.861395	0.549878	0.019652	0.497413	0.030157	0.301255	0.149801	0.196159	0.329914	0.052465	0.788471	0.248624	0.22502	6854514	7.526557	7.286017	7.421246	7.241588	7.731751	7.103086	7.061568	7.26634	7.160041	7.018625	7.031518	6.534042	Bak1	NM_007523
6854528	6509	7.537313	7.715204	7.523933	7.650902	-0.11359	0.324504	0.064302	0.568758	-0.12697	0.274381	0.191271	0.115094	-0.17789	0.138794	0.01338	0.904642	6854528	7.610759	7.538467	7.462713	7.673666	7.723722	7.748224	7.716106	7.561158	7.294536	7.52146	7.642895	7.788351	Lemd2	NM_146075
6854540	6511	8.660649	8.460832	8.805166	8.461117	0.199532	0.327898	-0.00029	0.998849	0.34405	0.110122	-0.34433	0.109871	0.199817	0.327249	-0.14452	0.472079	6854540	8.642947	8.540336	8.798663	8.685482	8.596099	8.100914	8.45868	9.072315	8.884504	8.485395	8.517245	8.38071	AI413582	NM_001002895
6854541	6512	8.093669	8.145538	7.95306	7.968855	0.124814	0.11396	0.176683	0.036289	-0.01579	0.827986	0.192477	0.025608	-0.05187	0.48201	0.140609	0.080681	6854541	8.169128	7.952744	8.159135	8.164034	8.214854	8.057725	8.000461	7.996098	7.862622	7.931659	8.021656	7.95325	Nudt3	NM_019837
6854546	6513	7.14099	7.224472	7.212269	7.867399	-0.72641	0.012023	-0.64293	0.021136	-0.65513	0.019446	0.012203	0.958033	-0.08348	0.719966	-0.07128	0.759262	6854546	7.432897	6.812081	7.177991	7.269383	7.173359	7.230673	7.35839	7.344678	6.933738	8.175603	7.441415	7.98518	Spdef	NM_013891
6854562	6514	7.190047	7.362453	7.180499	7.068059	0.121988	0.427397	0.294394	0.078347	0.11244	0.46309	0.181954	0.247661	-0.17241	0.271302	0.009548	0.949434	6854562	7.160348	7.216219	7.193573	7.173885	7.368784	7.544691	7.477101	7.181574	6.882822	7.077331	7.128152	6.998694	Taf11	NM_026836
6854572	6515	6.458683	6.467132	6.536452	6.389343	0.06934	0.397872	0.077789	0.345718	0.147108	0.094728	-0.06932	0.398002	-0.00845	0.916022	-0.07777	0.345835	6854572	6.51249	6.387778	6.475781	6.639276	6.405196	6.356924	6.595941	6.586063	6.427351	6.37427	6.408276	6.385484	Tcp11	NM_013687
6854596	6517	7.922995	7.924296	7.854484	7.820526	0.102469	0.363512	0.10377	0.357773	0.033958	0.757673	0.069812	0.529972	-0.0013	0.990539	0.068511	0.537476	6854596	7.901632	7.913409	7.953944	7.912502	8.0962	7.764186	7.660833	8.017752	7.884868	7.725698	7.880687	7.855193	Tead3	NM_011566
6854604	6519	7.410229	7.615694	7.062525	7.981005	-0.57078	0.127952	-0.36531	0.308815	-0.91848	0.025755	0.553169	0.138473	-0.20546	0.558021	0.347704	0.331227	6854604	7.47011	7.191256	7.569321	8.065548	7.384579	7.396955	7.014009	7.230879	6.942687	8.768591	7.59271	7.581714	Fkbp5	NM_010220
6854614	6520	6.112254	6.137149	6.269161	6.169256	-0.057	0.612472	-0.03211	0.77414	0.099905	0.382659	-0.13201	0.257009	-0.0249	0.823734	-0.15691	0.184909	6854614	6.013726	6.147969	6.175066	6.197496	6.177377	6.036573	6.25983	6.508051	6.039601	6.15738	6.177197	6.17319	Clps	NM_025469
6854616	6521	8.621118	8.505598	8.542115	8.273707	0.347411	0.044242	0.231891	0.150153	0.268408	0.102704	-0.03652	0.808421	0.11552	0.450738	0.079003	0.602438	6854616	8.664713	8.509068	8.689572	8.348722	8.714621	8.453451	8.771398	8.310639	8.544307	8.162792	8.470548	8.18778	Srpk1	NM_016795
6854653	6524	7.604598	7.424247	7.58861	6.283565	1.321033	0.007859	1.140681	0.016101	1.305045	0.008365	-0.16436	0.6731	0.180352	0.643802	0.015988	0.967074	6854653	7.81291	7.439989	7.560896	7.113198	7.545458	7.614084	7.802454	6.709364	8.254012	6.312917	6.591779	5.946	4930539E	NM_172450
6854654	6525	6.117842	6.194868	6.232398	6.0634	0.054443	0.729174	0.131468	0.411736	0.168998	0.297971	-0.03753	0.810977	-0.07703	0.6256	-0.11456	0.472145	6854654	6.192336	6.266262	5.894929	6.109743	6.247897	6.226963	6.545633	6.067101	6.08446	5.974103	5.987735	6.228361	Pxt1	NM_153390
6854658	6526	8.872662	8.914162	8.818828	8.686725	0.185937	0.078978	0.227438	0.03922	0.132104	0.190645	0.095334	0.332337	-0.0415	0.665229	0.053834	0.576175	6854658	8.902938	8.798714	8.916334	8.855765	9.088685	8.798037	8.993698	8.739381	8.723406	8.664123	8.701923	8.694128	Stk38	NM_134115
6854680	6527	5.838822	6.011341	5.705623	5.871769	-0.03295	0.807047	0.139572	0.316049	-0.16615	0.23872	0.305718	0.047222	-0.17252	0.222727	0.133199	0.337284	6854680	5.677128	6.016796	5.822541	6.214367	5.938375	5.881281	5.820046	5.675197	5.621626	5.864694	5.699698	6.050915	Cpne5	NM_153166
6854690	6528	7.941362	7.961077	7.771835	7.789538	0.151825	0.254677	0.171539	0.203033	-0.0177	0.889766	0.189242	0.16467	-0.01971	0.877352	0.169528	0.207854	6854690	7.941895	7.919008	7.963184	7.763201	8.147796	7.972234	7.945048	7.771366	7.59909	7.614195	7.913438	7.84098	Ppil1	NM_026845
6854694	6529	9.773521	9.911468	9.843634	9.788798	-0.01528	0.920854	0.12267	0.43418	0.054836	0.72239	0.067834	0.66101	-0.13795	0.381601	-0.07011	0.650505	6854694	9.810716	9.648379	9.861467	9.930092	10.01763	9.786682	10.01253	10.0463	9.472072	9.806286	9.83621	9.723898	Mtch1	NM_019880
6854735	6530	8.928739	8.87759	8.517654	8.815589	0.11315	0.31752	0.062001	0.575227	-0.29793	0.02295	0.359935	0.009485	0.05115	0.642783	0.411085	0.004721	6854735	8.689758	8.973095	9.123365	8.843908	8.966208	8.822653	8.491482	8.477899	8.583582	8.711669	8.908877	8.826221	Btbd9	NM_027060
6854760	6531	7.53786	7.799849	7.617341	7.408083	0.129777	0.352889	0.391766	0.017673	0.209258	0.150432	0.182508	0.202859	-0.26199	0.081642	-0.07948	0.562557	6854760	7.323699	7.523324	7.766557	7.589376	7.89062	7.919551	7.562277	7.641035	7.64871	7.314233	7.566225	7.343791	Glo1	NM_025374
6854790	6534	5.928254	6.183359	5.955885	5.985196	-0.05694	0.608674	0.198163	0.100848	-0.02931	0.790849	0.227474	0.065968	-0.25511	0.044074	-0.02763	0.802529	6854790	5.779649	6.108916	5.896196	6.333659	6.141124	6.075294	5.938952	5.993315	5.935388	6.058007	5.816086	6.081495	Tff1	NM_009362
6854813	6536	6.347688	6.411997	6.344571	6.377436	-0.02975	0.581273	0.034561	0.523116	-0.03286	0.5432	0.067426	0.228945	-0.06431	0.249262	0.003116	0.953469	6854813	6.384701	6.3824	6.275962	6.446106	6.322992	6.466894	6.316016	6.4203	6.297398	6.36424	6.343442	6.424627	Wdr4	NM_021322
6854826	6537	6.114068	6.075259	6.104537	6.403028	-0.28896	0.084801	-0.32777	0.056242	-0.29849	0.076694	-0.02928	0.847036	0.03881	0.798365	0.009531	0.949873	6854826	6.027451	5.913644	6.40111	6.022012	5.978626	6.225138	6.136547	6.177027	6.000037	6.377669	6.220474	6.61094	Cbs	NM_144855
6854844	6538	9.388239	9.345863	9.566967	9.925316	-0.53708	0.037405	-0.57945	0.027555	-0.35835	0.134941	-0.2211	0.334949	0.042377	0.849024	-0.17873	0.430988	6854844	9.461624	9.308938	9.394156	9.608114	9.209143	9.220331	9.415932	9.757401	9.527569	9.91443	9.493928	10.36759	Sik1	NM_010831
6854864	6539	8.123597	7.943699	7.663227	7.534651	0.588946	0.020184	0.409047	0.079577	0.128576	0.545618	0.280472	0.205951	0.179899	0.403026	0.46037	0.053768	6854864	8.251579	8.108276	8.010937	8.071153	8.05689	7.703053	7.708086	7.640821	7.640774	7.253465	8.036001	7.314488	Notch3	NM_008716
6854868	6540	8.792327	8.789444	8.727641	8.638729	0.153598	0.357937	0.150716	0.366553	0.088913	0.587813	0.061803	0.704966	0.002883	0.985843	0.064686	0.692025	6854868	8.833247	8.762227	8.781507	8.766648	8.971474	8.630211	8.525422	9.100767	8.556735	8.696871	8.713285	8.50603	Brd4	NM_020508
6854871	6541	8.682007	8.814704	8.731094	8.717674	-0.03567	0.531274	0.09703	0.112953	0.013421	0.811728	0.083609	0.163626	-0.1327	0.040928	-0.04909	0.394155	6854871	8.701906	8.6473	8.696814	8.704196	8.897098	8.842817	8.772911	8.764292	8.65608	8.667404	8.772633	8.712984	Akap8	NM_019774
6854872	6542	8.09722	8.335141	8.180589	8.284501	-0.18728	0.052863	0.05064	0.556335	-0.10391	0.243288	0.154552	0.097867	-0.23792	0.020379	-0.08337	0.341772	6854872	8.027372	8.169013	8.095275	8.312555	8.273388	8.41948	8.027371	8.353053	8.161342	8.27593	8.231855	8.345717	Akap8l	NM_017476

6854874	6543	7.646852	7.69569	7.662539	7.620072	0.02678	0.814113	0.075618	0.511994	0.042467	0.710015	0.033151	0.771223	-0.04884	0.669383	-0.01569	0.890323	6854874	7.584524	7.732997	7.623034	7.771372	7.808304	7.507393	7.561372	7.885752	7.540493	7.615911	7.672226	7.572078	Wiz	NM_212438
6854893	6544	8.752667	8.837509	8.652108	8.782425	-0.02976	0.761492	0.055084	0.576982	-0.13032	0.206272	0.185401	0.086076	-0.08484	0.396674	0.100559	0.319511	6854893	8.745584	8.854139	8.658279	8.742886	8.883626	8.886016	8.45077	8.770322	8.735233	8.68792	8.819393	8.839962	9030612M	NM_172458
6854895	6545	6.441925	6.458112	6.463154	6.509884	-0.06796	0.671232	-0.05177	0.745819	-0.04673	0.769692	-0.00504	0.974731	-0.01619	0.919019	-0.02123	0.893954	6854895	6.287507	6.748259	6.290009	6.457847	6.464041	6.452449	6.377672	6.746258	6.265532	6.45677	6.452652	6.620231	Zfp799	NM_177359
6854907	6547	5.914961	6.034406	6.054421	6.009579	-0.09462	0.487524	0.024827	0.85332	0.044842	0.739068	-0.02001	0.88147	-0.11945	0.38512	-0.13946	0.314725	6854907	5.911142	5.891918	5.941824	5.970676	6.101824	6.030719	5.942039	6.016137	6.205087	5.687698	6.189985	6.151055	BC066107	NM_207245
6854910	6549	8.604513	8.597558	8.620117	8.839151	-0.23464	0.079823	-0.24159	0.072788	-0.21903	0.098074	-0.02256	0.851897	0.006956	0.954049	-0.0156	0.89719	6854910	8.470224	8.660224	8.683092	8.642893	8.526284	8.623496	8.517463	8.550637	8.792252	8.727243	8.714349	9.07586	Cyp4f13	NM_130882
6854930	6553	5.602446	6.001442	5.932654	5.736975	-0.13453	0.168335	0.264467	0.017666	0.19568	0.05871	0.068788	0.460934	-0.399	0.002023	-0.33021	0.005891	6854930	5.660217	5.524895	5.622227	5.863529	6.046389	6.094408	5.95632	5.764685	6.076958	5.676228	5.759907	5.774789	Zfp101	NM_009542
6854944	6554	9.687749	9.688678	9.803857	9.6733	0.014449	0.876343	0.015378	0.86848	0.130557	0.184658	-0.11518	0.23619	-0.00093	0.992014	-0.11611	0.232761	6854944	9.607563	9.764269	9.691416	9.556986	9.836351	9.672697	9.866626	9.703112	9.841833	9.617957	9.813296	9.588647	Hnrnpm	NM_029804
6854954	6555	7.884695	7.865078	7.961908	7.842132	0.042564	0.738414	0.022946	0.856756	0.119776	0.359011	-0.09683	0.454148	0.019617	0.877324	-0.07721	0.54795	6854954	7.859148	7.871656	7.923282	7.914249	7.938465	7.74252	8.033623	8.085583	7.766518	7.615966	7.851934	8.058495	2-Mar	NM_145486
6854962	6556	7.592973	7.813056	7.857441	7.791688	-0.19872	0.260733	0.021368	0.899671	0.065753	0.699304	-0.04439	0.793759	-0.22008	0.216944	-0.26447	0.145915	6854962	7.815033	7.643964	7.319923	7.720391	8.091204	7.627574	7.898081	8.023792	7.650451	7.771906	7.757164	7.845995	Rab11b	NM_008997
6854971	6557	7.022332	6.990188	6.406708	6.447842	0.574489	0.181754	0.542346	0.20473	-0.04113	0.91918	0.58348	0.175749	0.032144	0.936794	0.615623	0.155704	6854971	7.542459	7.180765	6.343771	6.799767	7.561522	6.609275	6.537648	5.865098	6.817379	6.514183	6.188749	6.640595	Angptl4	NM_020581
6854983	6558	9.022677	9.132868	9.155558	9.064912	-0.04223	0.672619	0.067956	0.50048	0.090646	0.374173	-0.02269	0.819677	-0.11019	0.285666	-0.13288	0.205023	6854983	8.895872	8.970042	9.202118	9.082163	9.17126	9.145181	9.304305	9.117196	9.045172	8.978547	9.04064	9.175548	H2-Ke2	NM_010385
6854988	6560	6.223805	6.217106	6.273887	5.825956	0.397849	0.215638	0.39115	0.222736	0.447931	0.168528	-0.05678	0.852605	0.006699	0.982492	-0.05008	0.869796	6854988	6.327513	6.304873	6.03903	5.955863	6.428064	6.267392	6.263034	6.365648	6.192979	5.323916	6.572317	5.581635	Rps18	AK018004
6854990	6561	10.98454	10.65433	10.89918	10.1186	0.865938	0.004893	0.535735	0.044511	0.780578	0.008462	-0.24484	0.308307	0.330203	0.180491	0.08536	0.714341	6854990	11.11365	11.04299	10.79697	10.50556	10.8053	10.65214	11.05756	10.52141	11.11856	10.04411	10.53223	9.779456	H2-K1	NM_001001892
6854999	6562	7.024793	7.036422	7.090909	7.061786	-0.03699	0.651594	-0.02536	0.756034	0.029123	0.721552	-0.05449	0.509258	-0.01163	0.886436	-0.06612	0.426253	6854999	6.964473	6.968402	7.141503	7.032535	7.18621	6.890522	7.159488	7.061969	7.05127	7.03213	7.044566	7.108662	Ring1	NM_009066
6855000	6563	7.840043	8.020773	7.993886	8.160843	-0.3208	0.029967	-0.14007	0.283242	-0.16696	0.207569	0.026887	0.830773	-0.18073	0.176047	-0.15384	0.242016	6855000	7.788497	7.878457	7.853174	8.02293	7.997283	8.042106	8.069455	8.190365	7.721837	8.042327	8.091223	8.348978	H2-Ke6	NM_013543
6855003	6564	8.30535	8.218412	8.369412	8.393476	-0.08813	0.265161	-0.17506	0.04454	-0.02406	0.751942	-0.151	0.074171	0.086938	0.271156	-0.06406	0.409155	6855003	8.363626	8.333079	8.219346	8.159513	8.307983	8.18774	8.348881	8.46462	8.294735	8.323353	8.524685	8.33239	Slc39a7	NM_008202
6855006	6565	9.153574	9.196388	9.005137	9.011133	0.142441	0.279981	0.185255	0.170231	-0.006	0.962289	0.191252	0.158366	-0.04281	0.736613	0.148438	0.261718	6855006	9.298951	9.183229	8.978543	9.151115	9.326885	9.111165	8.993962	9.218861	8.802587	8.909468	9.06424	9.059692	Brd2	NM_010238
6855013	6566	8.044342	7.857768	7.906341	7.321992	0.72235	0.012221	0.535776	0.043921	0.584349	0.031342	-0.04857	0.833949	0.186574	0.429573	0.138001	0.555417	6855013	7.883897	8.229578	8.019552	7.85485	8.024849	7.693605	8.185003	7.376078	8.157942	7.255862	7.527233	7.182881	Psmb9	NM_013585
6855018	6567	11.66876	11.59411	11.68454	10.42067	1.248094	0.01309	1.173441	0.017457	1.263867	0.012324	-0.09043	0.823822	0.074653	0.854097	-0.01577	0.968974	6855018	12.07555	11.77921	11.15153	11.64273	11.78404	11.35556	12.08257	11.21153	11.75951	10.68215	10.93145	9.648408	H2-Aa	NM_010378
6855037	6570	8.233611	8.255908	8.252124	8.747105	-0.51349	0.005819	-0.4912	0.007353	-0.49498	0.007065	0.003784	0.978765	-0.0223	0.875474	-0.01851	0.896451	6855037	8.278538	8.390594	8.0317	8.223634	8.093194	8.450896	8.343165	8.170853	8.242353	8.517945	8.837613	8.885756	Rnf5	NM_019403
6855040	6571	6.593099	6.550641	6.403266	6.595359	-0.00226	0.976099	-0.04472	0.557774	-0.19209	0.030309	0.147375	0.078591	0.042459	0.577432	0.189834	0.0318	6855040	6.641348	6.541621	6.596329	6.587834	6.535546	6.528542	6.491035	6.476369	6.242393	6.517096	6.567598	6.701384	Egfl8	NM_152922
6855041	6572	7.772578	7.87596	7.743596	7.729315	0.043264	0.72495	0.146645	0.251725	0.014281	0.907202	0.132364	0.297183	-0.10338	0.409163	0.028982	0.813251	6855041	7.800948	7.814194	7.702593	7.874396	8.075574	7.677909	7.601588	7.909554	7.719646	7.800566	7.578198	7.80918	Ppt2	NM_019441
6855051	6573	8.537951	7.830992	7.663456	7.361564	1.176387	0.013458	0.469428	0.243273	0.301892	0.44129	0.167536	0.664937	0.706959	0.09438	0.874495	0.046919	6855051	8.878675	8.164727	8.57045	7.938024	8.3529	7.202051	7.983752	7.656586	7.350029	6.857386	7.88597	7.341336	C4b	NM_009780
6855057	6575	6.26474	6.512554	6.584004	6.529607	-0.26487	0.093171	-0.01705	0.90538	0.054397	0.705758	-0.07145	0.621121	-0.24781	0.112463	-0.31926	0.050718	6855057	6.352413	6.072917	6.368891	6.692743	6.388389	6.456529	6.691221	6.724564	6.336228	6.632919	6.569714	6.386189	Stk19	NM_019442
6855060	6576	8.503566	8.499547	8.489009	8.437787	0.065779	0.085679	0.06176	0.10303	0.051222	0.165495	0.010538	0.761567	0.004019	0.907639	0.014557	0.675948	6855060	8.535034	8.463636	8.512028	8.460706	8.542955	8.49498	8.545767	8.468371	8.452888	8.397455	8.463251	8.452654	Skiv2l	NM_021337
6855061	6577	7.549089	7.479009	6.653799	6.174571	1.374518	0.023808	1.304437	0.029656	0.479228	0.360291	0.82521	0.133287	0.070081	0.890667	0.89529	0.107429	6855061	8.133171	7.158963	7.355134	7.913399	7.908095	6.615532	7.349047	5.926498	6.685852	5.813611	6.540426	6.169677	Cfb	NM_008198
6855062	6578	6.469153	6.420017	6.326691	6.21686	0.252293	0.136868	0.203157	0.219771	0.109831	0.492173	0.093326	0.557793	0.049136	0.755711	0.142461	0.377843	6855062	6.769262	6.397284	6.240912	6.412912	6.51875	6.328389	6.328388	6.348118	6.303568	6.328453	6.378136	5.943991	C2	NM_013484
6855067	6579	8.039846	7.840388	7.808672	7.92025	0.119595	0.743462	-0.07986	0.826673	-0.11158	0.760005	0.031716	0.930611	0.199458	0.587507	0.231174	0.530881	6855067	7.85204	8.265119	8.002378	7.838131	7.78885	7.894182	7.732724	7.859115	7.834176	8.421857	8.382078	6.956816	Hspa1a	NM_010479
6855068	6580	10.05891	9.952571	9.788725	9.001318	1.057587	0.009357	0.951253	0.015621	0.787407	0.035168	0.163846	0.612634	0.106334	0.741252	0.27018	0.410336	6855068	10.20885	9.689196	10.27867	9.595616	10.46159	9.800508	10.21739	9.24379	9.904995	9.111329	9.071623	8.821003	D17H6S56	L78788
6855076	6583	10.4252	10.39001	10.42165	10.41508	0.010113	0.928439	-0.02507	0.824039	0.00657	0.953468	-0.03164	0.779211	0.035187	0.755382	0.003543	0.974892	6855076	10.41223	10.31308	10.55028	10.35799	10.5617	10.25034	10.51101	10.45387	10.30008	10.25041	10.4938	10.50104	Csnk2b	NM_009975
6855083	6585	8.844159	8.783011	8.765426	8.696162	0.147997	0.466611	0.086848	0.665669	0.069264	0.729809	0.017584	0.929872	0.061148	0.760233	0.078733	0.694949	6855083	8.933031	8.778128	8.821318	8.776981	9.010372	8.561679	8.453992	9.215842	8.626445	8.687826	8.792843	8.607818	Bat2	NM_020027
6855084	6586	6.820383	6.835971	7.087011	6.26754	0.552843	0.071457	0.568431	0.065235	0.819471	0.01515	-0.25104	0.373232	-0.01559	0.95474	-0.26663	0.345837	6855084	6.910187	6.777204	6.773759	6.702959	7.12345	6.681503	7.548752	6.578906	7.133376	6.017752	6.663393	6.121475	Aif1	NM_019467
6855087	6587	6.186595	6.215624	6.443262	5.276262	0.910333	0.073255	0.939362	0.066138	1.167	0.029621	-0.22764	0.620239	-0.02903	0.949215	-0.25667	0.57719	6855087	6.744293	6.2133	5.602193	6.159782	6.83485	5.652239	6.785908	6.295447	6.248432	5.474561	5.788619	4.565606	Tnf	NM_013693
6855088	6588	8.388527	8.08785	8.38324	7.978801	0.409726	0.06426	0.109049	0.583725	0.40444	0.06709	-0.29539	0.160563	0.300677	0.154087	0.005287	0.978597	6855088	8.611519	8.449523	8.104539	8.053185	8.315324	7.89504	8.2293	8.488279	8.432142	7.775172	8.319621	7.841609	Lta	NM_010735
6855089	6589	7.453227	7.530186	7.524944	7.552534	-0.09931	0.4027	-0.02235	0.847353	-0.02759	0.81227	0.005242	0.963944	-0.07696	0.512857	-0.07172	0.541242	6855089	7.473919	7.387311	7.49845	7.708039	7.396074	7.486446	7.602407	7.651426	7.321	7.486969	7.477616	7.693017	Nfkbil1	NM_010909
6855116	6590	7.125696	7.407383	7.154516	6.889499	0.236197	0.332434	0.517883	0.053565	0.265017	0.280466	0.252866	0.301523	-0.28169	0.253538	-0.02882	0.902937	6855116	7.035314	6.957791	7.383983	7.409351	7.61271	7.200087	7.545025	6.777236	7.141288	6.630259	6.869038	7.169201	Tcf19	NM_025674
6855121	6591	7.213812	7.270287	7.277749	7.291	-0.07719	0.046363	-0.02071	0.545149	-0.01325	0.696671	-0.00746	0.825656	-0.05648	0.123259	-0.06394	0.086964	6855121	7.205104	7.166934	7.269397	7.251616	7.271188	7.288057	7.330964	7.277073	7.225211	7.309501	7.262204	7.301296	Vars2	NM_175137
6855122	6592	6.61051	6.614012	6.694952	6.619342	-0.00883	0.905117	-0.00533	0.942636	0.07561	0.322995	-0.08094	0.29221	-0.0035	0.962288	-0.08444	0.273296	6855122	6.712294	6.632396	6.48684	6.585428	6.576734	6.679874	6.783985	6.722974	6.577896	6.686133	6.580617	6.591276	Gtf2h4	NM_010364
6855123	6593	10.01576	9.718785	9.784459	9.679678	0.336081	0.001884	0.039107	0.611173	0.104781	0.194134	-0.06567	0.400276	0.296974	0.003857	0.2313	0.014043	6855123	10.07253	9.974106	10.00064	9.824471	9.768365	9.563519	9.79527	9.819579	9.738527	9.694581	9.769359	9.575094	Ddr1	NM_007584
6855134	6594	10.85247	10.77303	11.05	10.44789	0.404583	0.1917	0.325143	0.284913	0.602116	0.066593	-0.27697	0.357544	0.07944	0.786584	-0.19753	0.506005	6855134	10.91767	10.8169	10.82284	10.44414	11.16954	10.70541	11.34059	10.66266	11.14676	10.53421	10.87087	9.938581	Tubb5	NM_011655
6855144	6595	8.106574	7.990522	8.026743	7.935025	0.171549	0.389309	0.055497	0.775903	0.091718	0.639562	-0.03622	0.852386	0.116052	0.555164	0.079831	0.683036	6855144	8.126675	8.028452	8.164596	7.907645	8.181668	7.882253	7.578581	8.412726	8.088923	7.994	7.939729	7.871346	2610110G	NM_001142744
6855145	6596	8.549881	8.390812	8.620886	8.604061	-0.05418	0.739699	-0.21325	0.212727	0.016825	0.917553	-0.23007	0.18218	0.159069	0.342051	-0.071	0.664065	6855145	8.541141	8.539096	8.569406	8.260324	8.676326	8.235785	8.57759	8.754568	8.530499	8.572873	8.889015	8.350294	Mrps18b	NM_025878
6855149	6597	8.867872	8.864566	8.817231	8.795049	0.072823	0.386239	0.069518	0.407168	0.022182	0.787217	0.047336	0.567856	0.003306	0.967838	0.050641	0.541748	6855149	8.916035	8.737278	8.950303	8.803761	8.965815	8.824123	8.88203	8.810316	8.759346	8.668777	8.893602	8.822767	Abcf1	NM_013854
6855150	6598	6.705608	6.830926	6.729212	6.804796	-0.09919	0.34717	0.02613	0.799122	-0.07558	0.468456	0.101714	0.335726	-0.12532	0.242553	-0.0236	0.818111	6855150	6.815181	6.621523	6.680119	6.771657	6.920725	6.800395	6.775041	6.832793	6.579801	6.78321	6.657045	6.974133	Prr3	NM_145487
6855155	6599	6.522661	7.564938	6.000231	6.043234	0.479427	0.095932	1.521704	0.000328	-0.043	0.869827	1.564707	0.000272	-1.04228	0.003431	0.52243	0.073842	6855155	6.19263	6.354183	7.02117	7.542197	7.383492	7.769125	6.144156	6.194273	5.662265	6.036549	5.780715	6.312437	H2-T10	NM_010395
6855158	6600	6.254127	5.955124	5.983552	5.933077	0.32105	0.140145	0.022046	0.913236	0.050474	0.803327	-0.02843	0.888296	0.299004	0.165737	0.270576	0.204886	6855158	6.38688	6.440083	5.935419	5.808979	6.216588	5.839804	5.893553	5.789462	6.26764	5.902679	5.752605	6.143948	EG667977	NM_001081032
6855176	6602	7.420203	7.518256	7.391852	7.531204	-0.111	0.37682	-0.01295	0.915776	-0.13935	0.273906	0.126404	0.317739	-0.09805	0.432461	0.028352	0.817122	6855176	7.266535	7.51173	7.482345	7.588851	7.511453	7.454463	7.496361	7.556816	7.122378	7.623276	7.469991	7.500344	Rpp21	NM_026308
6855177	6603	7.118007	7.079145	7.248954	7.218854	-0.10085	0.192278	-0.13971	0.083998	0.0301	0.682034	-0.16981	0.043335	0.038862	0.598176	-0.13095	0.101642	6855177	7.079845	7.164539	7.109637	7.054115	7.104636	7.078685	7.161887	7.425736	7.159239	7.169921	7.292051	7.19459	Trim39	NM_024468
6855202	6609	8.174245	8.424249	8.272372	8.309961	-0.13572	0.423728	0.114287	0.497951	-0.03759	0.821254	0.151877	0.373116	-0.25	0.159064	-0.09813	0.559104	6855202	8.025798	8.218266	8.27867	8.435413	8.676239	8.161094	8.335177	8.465784	8.016155	8.16289	8.339653	8.427341	Ppp1r11	NM_029632
6855219	6611	7.233809	7.337725	7.456044	7.40277	-0.16896	0.281416	-0.06505	0.668359	0.053275	0.725158	-0.11832	0.442	-0.10392	0.497659	-0.22224	0.167197	6855219	7.093533	7.271332	7.336562	7.194633	7.673079	7.145462	7.418352	7.637537	7.312244	7.391259	7.40672	7.41033	Znrd1	NM_023162
6855225	6613	5.989899	5.941831	5.951236	5.822157	0.167742	0.187692	0.119674	0.334154	0.129078	0.299868	-0.0094	0.937616	0.048068	0.690605	0.038663	0.748402	6855225	6.091143	5.919787	5.958767	5.844498	5.986035	5.994961	6.093872	5.745079	6.014756	5.948714	5.902803	5.614955	Mog	NM_010814
6855227	6614	6.808829	6.642468	6.557683	6.943873	-0.13504	0.592656	-0.3014	0.248873	-0.38619	0.149748	0.084785	0.735514	0.16636	0.511878	0.251145	0.330421	6855227	6.998862	6.39903	7.028594	6.464328	6.868215	6.594862	6.081648	6.784423	6.806979	6.853972	7.076449	6.901197	Olfr90	NM_146477
6855228	6615	5.861964	6.027778	6.226747	5.924907	-0.06294	0.762159	0.102871	0.622595	0.30184	0.171549	-0.19897	0.351186	-0.16581	0.433279	-0.36478	0.107074	6855228	6.142433	5.674879	5.768579	6.266592	5.610849	6.205893	6.001369	6.298347	6.380524	5.933355	5.820439	6.020927	Olfr91	NM_182714
6855246	6622	6.209876	6.960596	7.014512	6.641455	-0.43158	0.241711	0.319141	0.377188	0.373057	0.306268	-0.05392	0.878413	-0.75072	0.059066	-0.80464	0.046162	6855246	6.574536	6.231787	5.823305	7.184587	6.536987	7.160214	6.584624	7.366755	7.092157	7.188426	6.570785	6.165154	H2-M2	NM_008204
6855304	6631	11.3805	11.58443	11.41961	11.65316	-0.27266	0.198294	-0.06873	0.7328	-0.23355	0.263908	0.164827	0.421106	-0.20394	0.324763	-0.03911	0.845561	6855304	11.43602	11.41696	11.28851	11.39958	11.30173	12.05199	11.46195	11.57007	11.2268	11.54027	11.59318	11.82603	AY036118	AY036118
6855400	6638	10.51465	10.56561	10.41159	10.41651	0.098133	0.455311	0.149093	0.267424	-0.00493	0.969546	0.15402	0.253152	-0.05096	0.694384	0.10306	0.433842	6855400	10.61789	10.37765	10.5484	10.51835	10.61207	10.5664	10.11117	10.62515	10.49844	10.39489	10.49319	10.36146	Cd2ap	NM_009847
6855478	6641	6.782371	6.943071	6.665313	7.070534	-0.28816	0.0424	-0.12746	0.317222	-0.40522	0.009482	0.277758	0.048573	-0.1607	0.215538	0.117058	0.355948	6855478	6.612712	6.847672	6.886728	6.886973	6.943841	6.998399	6.468225	6.858822	6.668891	6.933776	7.047188	7.230638	Slc25a27	NM_028711
6855526	6642	7.438069	7.528985	7.306999	7.433437	0.004632	0.977264	0.095548	0.560968	-0.12644	0.445375	0.221987	0.196448	-0.09092	0.579719	0.13107	0.429524	6855526	7.351643	7.539567	7.422996	7.534015	7.604157	7.448784	7.258599	7.424075	7.238322	7.199263	7.263594	7.837454	Enpp4	NM_199016
6855567	6643	6.460733	6.414371	6.470335	6.215767	0.244966	0.091433	0.198604	0.158603	0.254568	0.081404	-0.05596	0.672888	0.046362	0.726046	-0.0096	0.941923	6855567	6.62909	6.530067	6.223041	6.400215	6.552418	6.29048	6.496894	6.404119	6.509992	6.332271	6.306715	6.008315	Runx2	NM_001146038
6855603	6644	8.210754	8.377546	8.301659	8.214579	-0.00382	0.971185	0.162967	0.150962	0.087081	0.420817	0.075887	0.480775	-0.16679	0.142771	-0.09091	0.401578	6855603	8.206505	8.215849	8.209908	8.222399	8.454353	8.455886	8.502318	8.113553	8.289107	8.308118	8.193385	8.142233	Cdc5l	NM_152810
6855608	6645	6.104916	6.324548	6.207326	6.263621	-0.15871	0.279777	0.060927	0.668087	-0.05629	0.691715	0.117222	0.416769	-0.21963	0.147315	-0.10241	0.475839	6855608	6.228544	6.212902	5.873302	6.423857	6.269762	6.280025	6.256496	6.423646	5.941837	6.283977	6.185801	6.321086	Tmem151b	NM_001013749

6855610	6646	12.33892	12.32902	12.33476	12.23103	0.10789	0.245131	0.097983	0.287599	0.103727	0.2623	-0.00574	0.948402	0.009907	0.911145	0.004163	0.962582	6855610	12.35477	12.22871	12.43329	12.30858	12.4027	12.27577	12.50034	12.21697	12.28697	12.14642	12.226	12.32068	Hsp90ab1	NM_008302
6855615	6647	7.768791	8.035152	7.673079	7.564847	0.203944	0.126229	0.470304	0.004319	0.108231	0.391489	0.362073	0.016301	-0.26636	0.05636	0.095712	0.446236	6855615	7.858654	7.781131	7.666588	8.116299	8.140932	7.848224	7.895488	7.56725	7.556498	7.511743	7.69493	7.487869	Slc29a1	NM_022880
6855625	6649	9.55126	9.418118	9.178738	9.250277	0.300983	0.028136	0.167841	0.174098	-0.07154	0.542635	0.23938	0.066036	0.133142	0.270631	0.372522	0.010682	6855625	9.741302	9.44991	9.462567	9.442191	9.565486	9.246677	9.203555	9.290663	9.041996	9.202488	9.347289	9.201054	Tmem63b	NM_198167
6855659	6650	12.29568	12.38038	12.25042	12.43421	-0.13852	0.207261	-0.05382	0.608422	-0.18379	0.106167	0.129967	0.233956	-0.0847	0.425832	0.045267	0.66577	6855659	12.18959	12.43522	12.26224	12.3682	12.25326	12.51969	12.11505	12.3599	12.2763	12.30847	12.50191	12.49224	Vegfa	NM_001025250
6855669	6651	8.685458	8.56748	8.647285	9.102613	-0.41715	0.080892	-0.53513	0.033554	-0.45533	0.060888	-0.07981	0.712378	0.117979	0.587703	0.038173	0.859546	6855669	8.403289	8.93433	8.718756	8.497682	8.630464	8.574293	9.075708	8.64364	8.222507	9.041183	9.098545	9.16811	Rsph9	NM_029338
6855670	6652	7.041507	7.063566	7.009339	7.015407	0.0261	0.816189	0.048159	0.66931	-0.00607	0.956831	0.054226	0.631126	-0.02206	0.844174	0.032168	0.774705	6855670	6.905695	7.069301	7.149525	6.943312	7.118594	7.128791	7.177388	6.988905	6.861725	7.012431	6.878061	7.155729	Mad2l1bp	NM_025649
6855672	6653	6.61974	6.447313	6.752835	6.413353	0.206387	0.410979	0.03396	0.890034	0.339482	0.191482	-0.30552	0.235059	0.172427	0.489297	-0.1331	0.591218	6855672	6.913138	6.477354	6.468728	6.287191	6.768129	6.286618	6.987324	6.648478	6.622703	6.297335	6.853992	6.088731	Polh	NM_030715
6855680	6654	7.337544	7.246923	7.166437	7.111522	0.226022	0.094292	0.135401	0.288495	0.054915	0.657011	0.080485	0.518231	0.090621	0.468555	0.171106	0.188742	6855680	7.241144	7.300817	7.47067	7.244073	7.503323	6.993372	7.121881	7.212236	7.165195	7.143182	7.151483	7.039901	Tjap1	NM_028751
6855684	6655	6.845877	6.797255	6.702836	6.865722	-0.01984	0.833118	-0.06847	0.474126	-0.16289	0.111784	0.094419	0.330614	0.048622	0.608273	0.143041	0.155261	6855684	6.753733	6.969113	6.814786	6.80077	6.735221	6.855774	6.547087	6.893789	6.667633	6.831219	6.837985	6.927963	Abcc10	NM_145140
6855705	6657	7.107637	7.229283	7.083721	7.120896	-0.01326	0.907096	0.108388	0.353637	-0.03717	0.744271	0.145562	0.222602	-0.12165	0.301242	0.023916	0.833443	6855705	7.062896	7.137364	7.12265	7.261741	7.229504	7.196605	7.363971	6.979248	6.907944	7.075469	7.05216	7.235058	Cul9	NM_001081335
6855706	6658	7.568515	7.495183	7.389725	7.40924	0.159276	0.280802	0.085943	0.549948	-0.01951	0.890814	0.105458	0.465789	0.073333	0.608837	0.17879	0.230355	6855706	7.723975	7.509163	7.472408	7.531172	7.6472	7.307176	7.439032	7.600294	7.129849	7.333235	7.510164	7.38432	Srf	NM_020493
6855707	6659	7.30513	7.384975	7.280942	7.106922	0.198207	0.219291	0.278052	0.098443	0.17402	0.2756	0.104033	0.504027	-0.07985	0.60593	0.024188	0.874827	6855707	7.451305	7.210548	7.253536	7.394856	7.530268	7.2298	7.590059	7.152905	7.099862	7.118452	7.246377	6.955938	Ptk7	NM_175168
6855708	6660	7.321424	7.345737	7.308464	7.340414	-0.01899	0.796512	0.005323	0.942259	-0.03195	0.665651	0.037273	0.61495	-0.02431	0.741634	0.01296	0.86015	6855708	7.379888	7.227618	7.356766	7.318301	7.299487	7.419424	7.429239	7.194026	7.302128	7.272299	7.327516	7.421427	Klc4	NM_029091
6855712	6661	7.508656	7.550466	7.332595	7.439702	0.068953	0.31998	0.110763	0.126943	-0.10711	0.138185	0.21787	0.010079	-0.04181	0.538277	0.17606	0.026773	6855712	7.485773	7.472233	7.567961	7.447073	7.69549	7.508834	7.369947	7.383173	7.244666	7.427279	7.448531	7.443297	BC011248	BC011248
6855716	6662	8.596819	8.601993	8.471692	8.525183	0.071636	0.616435	0.076811	0.591656	-0.05349	0.707372	0.130301	0.370989	-0.00517	0.970898	0.125126	0.389348	6855716	8.540797	8.454452	8.795207	8.55508	8.861613	8.389287	8.579521	8.311495	8.524061	8.494586	8.478181	8.602781	Klhdc3	NM_027910
6855717	6663	8.711042	8.712061	8.603079	8.622962	0.088079	0.215948	0.089099	0.211181	-0.01988	0.769401	0.108982	0.135002	-0.00102	0.987975	0.107962	0.1382	6855717	8.679119	8.708277	8.745729	8.722708	8.826082	8.587393	8.683372	8.547322	8.578544	8.542441	8.643202	8.683244	Ppp2r5d	NM_009358
6855722	6665	8.796893	8.592579	8.942796	8.82853	-0.03164	0.822708	-0.23595	0.122471	0.114266	0.427277	-0.35022	0.033486	0.204313	0.173203	-0.1459	0.316768	6855722	9.058938	8.731775	8.599965	8.55324	8.657756	8.566742	9.112288	9.002059	8.714041	8.895759	8.880184	8.709647	Cnpy3	NM_028065
6855724	6666	6.912953	6.965794	6.872778	6.81033	0.102623	0.371182	0.155463	0.189155	0.062447	0.580139	0.093016	0.415499	-0.05284	0.638778	0.040176	0.720352	6855724	7.039834	6.967098	6.731928	7.05205	6.848172	6.997159	7.027014	6.913414	6.677905	6.834562	6.763196	6.833233	Ptcra	NM_011195
6855725	6667	9.084537	9.073764	9.116087	9.185155	-0.10062	0.243786	-0.11139	0.201123	-0.06907	0.412901	-0.04232	0.610994	0.010773	0.896166	-0.03155	0.703489	6855725	9.061755	9.159185	9.03267	8.95051	9.187963	9.082819	9.059302	9.216369	9.072591	9.098213	9.30965	9.147601	Rpl7l1	ENSMUST000000
6855727	6668	6.302491	6.045054	6.115013	6.373973	-0.07148	0.680823	-0.32892	0.085182	-0.25896	0.160698	-0.06996	0.687189	0.257437	0.16288	0.187478	0.295521	6855727	6.252085	6.189155	6.466234	5.978914	6.359292	5.796956	6.385335	5.987086	5.972618	6.343058	6.29277	6.486091	A330017A	ENSMUST000000
6855737	6669	8.813607	8.886102	8.830185	8.858176	-0.04457	0.458743	0.027926	0.638833	-0.02799	0.638063	0.055917	0.357382	-0.0725	0.241097	-0.01658	0.779537	6855737	8.752885	8.891685	8.796251	8.831588	8.948442	8.878276	8.749135	8.943696	8.797724	8.829418	8.889732	8.855378	Ubr2	NM_146078
6855759	6672	7.164106	7.085112	7.168359	7.245011	-0.0809	0.111381	-0.1599	0.007664	-0.07665	0.128499	-0.08325	0.102894	0.078994	0.11879	-0.00425	0.92738	6855759	7.187493	7.194372	7.110453	7.127738	7.113627	7.013971	7.092256	7.249061	7.16376	7.240461	7.238682	7.25589	Taf8	NM_022015
6855772	6673	8.006234	7.617976	7.877077	7.998981	0.007253	0.965156	-0.38101	0.045408	-0.1219	0.470436	-0.2591	0.146031	0.388258	0.042324	0.129157	0.445375	6855772	8.052232	7.95716	8.00931	7.403219	7.923282	7.527427	7.924088	8.050363	7.656779	7.929022	8.221785	7.846137	Bysl	NM_016859
6855785	6674	7.642839	7.539101	7.721645	7.810878	-0.16804	0.418162	-0.27178	0.204765	-0.08923	0.662395	-0.18254	0.380914	0.103739	0.612519	-0.07881	0.699407	6855785	7.802124	7.55162	7.574774	7.619332	7.635458	7.362512	7.360064	8.175282	7.62959	7.838341	7.926956	7.667337	Tomm6	NM_025365
6855797	6675	6.269836	6.194512	6.245159	6.088259	0.181577	0.201527	0.106254	0.439036	0.1569	0.263571	-0.05065	0.708036	0.075323	0.579642	0.024677	0.85471	6855797	6.349651	6.330495	6.129361	6.050646	6.467558	6.065333	6.240324	6.305357	6.189795	6.180621	5.895307	6.188848	Mdfi	NM_010783
6855801	6676	7.755118	7.792162	7.851164	7.671896	0.083221	0.590408	0.120266	0.441275	0.179267	0.261677	-0.059	0.70142	-0.03704	0.809225	-0.09605	0.535743	6855801	7.848979	7.737605	7.678769	7.830283	8.010959	7.535244	7.738787	8.123883	7.690821	7.613242	7.791446	7.611001	Foxp4	NM_001110824
6855829	6679	9.455901	9.463038	9.367672	9.303415	0.152487	0.466123	0.159623	0.446258	0.064257	0.755376	0.095366	0.645055	-0.00714	0.972309	0.08823	0.669673	6855829	9.200048	9.600592	9.567064	9.221925	9.748023	9.419166	9.126324	9.555101	9.42159	9.374325	9.525519	9.0104	Nfya	NM_001110832
6855833	6680	6.79946	6.463577	6.427608	6.87987	-0.08041	0.937853	-0.41629	0.687982	-0.45226	0.662923	0.035969	0.972173	0.335883	0.745467	0.371852	0.719516	6855833	5.204314	6.232213	8.961853	7.161192	6.338201	5.891338	7.609882	6.040631	5.632311	5.966176	7.638969	7.034464	Apobec2	NM_009694
6855876	6681	6.885636	6.936769	6.737312	7.20196	-0.31632	0.086906	-0.26519	0.14062	-0.46465	0.02098	0.199457	0.253647	-0.05113	0.760589	0.148324	0.387108	6855876	6.677716	7.064983	6.91421	6.639044	6.964822	7.206442	6.594808	6.874732	6.742396	7.346171	7.188113	7.071595	Daam2	NM_001008231
6855891	6682	7.616414	7.352856	7.207858	6.922345	0.694069	0.003961	0.430511	0.038124	0.285513	0.138676	0.144998	0.427839	0.263558	0.167463	0.408556	0.046439	6855891	7.851328	7.677587	7.320327	7.317688	7.577129	7.163751	7.30678	7.051629	7.265165	6.752011	7.160463	6.854562	Rftn1	NM_181397
6855935	6684	8.274712	8.339848	8.319554	8.36687	-0.09216	0.303299	-0.02702	0.755295	-0.04732	0.587693	0.020293	0.814691	-0.06514	0.459233	-0.04484	0.607019	6855935	8.321143	8.350552	8.152442	8.393676	8.37697	8.248897	8.299734	8.335392	8.323537	8.187724	8.459069	8.453818	Tbc1d5	NM_028162
6855981	6685	6.397518	6.416942	6.117327	6.238287	0.159231	0.540813	0.178655	0.493939	-0.12096	0.640502	0.299616	0.263742	-0.01942	0.9398	0.280191	0.293587	6855981	6.406685	6.20279	6.583078	6.615208	6.394351	6.241268	6.169152	6.438136	5.744692	5.799642	6.271289	6.64393	Satb1	NM_009122
6856182	6693	6.702882	6.980701	6.865723	6.839584	-0.1367	0.511107	0.141117	0.497954	0.026139	0.898637	0.114978	0.578922	-0.27782	0.199794	-0.16284	0.436423	6856182	6.854598	6.587276	6.666771	6.792913	6.813122	7.336068	7.219793	6.575197	6.802179	6.828929	6.716599	6.973225	Zfp119	NM_144546
6856192	6695	9.143598	9.206961	9.236371	8.959357	0.184241	0.126653	0.247605	0.05119	0.277014	0.033488	-0.02941	0.79243	-0.06336	0.573863	-0.09277	0.415663	6856192	9.135803	9.038569	9.256422	9.200877	9.371969	9.048038	9.388098	9.099295	9.221719	8.8388	9.021451	9.017819	Stap2	NM_145934
6856196	6696	8.046716	8.115854	8.000847	7.99616	0.050555	0.70628	0.119693	0.382118	0.004687	0.972001	0.115007	0.400151	-0.06914	0.607731	0.045869	0.732198	6856196	8.044415	8.007489	8.088243	8.032554	8.434541	7.880466	8.06682	8.029281	7.90644	7.946347	8.110623	7.931511	Sh3gl1	NM_013664
6856197	6697	8.059097	8.164835	8.109418	8.120093	-0.061	0.454238	0.044742	0.579867	-0.01067	0.893925	0.055416	0.495204	-0.10574	0.209881	-0.05032	0.534607	6856197	7.944075	8.084446	8.148769	8.167199	8.21571	8.111595	8.162475	8.215599	7.950181	8.062546	8.134378	8.163355	Ubxn6	NM_024432
6856201	6698	6.552725	6.460439	6.293075	6.458563	0.094162	0.727943	0.001876	0.994448	-0.16549	0.544252	0.167364	0.539807	0.092286	0.733114	0.25965	0.34954	6856201	6.253681	6.298051	7.106444	6.779939	6.430003	6.171376	6.362195	6.368656	6.148375	6.178195	6.70544	6.492055	S3-12	NM_020568
6856202	6699	6.641567	6.705474	6.641418	6.712456	-0.07089	0.439064	-0.00698	0.938045	-0.07104	0.438139	0.064056	0.482889	-0.06391	0.483875	0.000149	0.998676	6856202	6.745147	6.530037	6.649517	6.835078	6.519391	6.761952	6.702155	6.62652	6.595579	6.735258	6.75726	6.644851	2310076L	NM_001077348
6856203	6700	8.858349	8.204535	7.644274	7.203654	1.654696	0.013625	1.000881	0.093308	0.44062	0.426116	0.560261	0.317485	0.653815	0.248663	1.214075	0.049669	6856203	9.594991	8.692858	8.287199	8.545276	8.499254	7.569074	8.111964	6.715542	8.105316	7.381921	7.59901	6.63003	Lrg1	NM_029796
6856204	6701	7.627808	7.593806	7.535197	7.42644	0.201368	0.104128	0.167366	0.166103	0.108757	0.351131	0.058609	0.60815	0.034002	0.764816	0.092611	0.423662	6856204	7.793579	7.591762	7.498084	7.761349	7.660688	7.359381	7.562706	7.552726	7.490159	7.440837	7.354882	7.483602	Sema6b	NM_013662
6856208	6702	8.368577	8.301183	8.482552	8.445634	-0.07706	0.627498	-0.14445	0.37195	0.036918	0.815081	-0.18137	0.269095	0.067394	0.670711	-0.11397	0.47688	6856208	8.465516	8.180447	8.459769	8.083349	8.580421	8.239779	8.654918	8.540564	8.252174	8.446707	8.520849	8.369346	D17Wsu10	NM_080837
6856210	6703	8.096508	7.991543	8.108365	8.058626	0.037881	0.735259	-0.06708	0.552447	0.049739	0.657922	-0.11682	0.311683	0.104965	0.360338	-0.01186	0.915423	6856210	8.109805	8.004217	8.175501	7.940844	8.189382	7.844403	8.059619	8.287905	7.977571	8.00589	8.140932	8.029057	Dpp9	NM_172624
6856215	6704	7.260776	7.064501	7.451769	7.300663	-0.03989	0.890744	-0.23616	0.425526	0.151106	0.605752	-0.38727	0.205884	0.196276	0.505092	-0.19099	0.516303	6856215	7.459783	7.250011	7.072535	7.071234	7.321607	6.800661	7.112962	8.126208	7.116138	7.433904	7.355779	7.112307	Ticam1	NM_174989
6856221	6705	9.119729	9.109599	9.201803	8.958801	0.160928	0.048761	0.150798	0.061253	0.243002	0.008004	-0.0922	0.220047	0.010131	0.887397	-0.08207	0.270299	6856221	9.018895	9.129068	9.211225	9.149444	9.011059	9.168293	9.232321	9.249358	9.12373	8.884809	9.053963	8.937631	M6prbp1	NM_025836
6856231	6706	8.551831	8.463949	8.003055	7.961613	0.590218	0.003074	0.502336	0.007414	0.041443	0.776538	0.460893	0.011438	0.087882	0.55088	0.548775	0.004624	6856231	8.744734	8.549973	8.360785	8.397911	8.656429	8.337506	8.0959	7.832002	8.081264	7.883675	8.166226	7.834937	Ptprs	NM_011218
6856238	6708	8.190125	8.441227	8.412219	8.626152	-0.43603	0.038665	-0.18492	0.325319	-0.21393	0.259998	0.029009	0.873511	-0.2511	0.192576	-0.22209	0.243697	6856238	8.200308	8.187325	8.182743	8.531443	8.410627	8.381612	8.500417	8.730632	8.005607	8.587647	8.440215	8.850594	2410015M	NM_153152
6856239	6709	7.825729	7.951987	7.822408	7.949704	-0.12397	0.175138	0.002283	0.978818	-0.1273	0.165134	0.129579	0.158559	-0.12626	0.168206	0.003321	0.969184	6856239	7.720519	7.775575	7.981094	7.962331	7.907012	7.986617	7.855786	7.820461	7.790977	7.883241	7.853426	8.112445	Lonp1	NM_028782
6856245	6710	7.684677	7.831054	7.677785	7.77504	-0.09036	0.203686	0.056014	0.415844	-0.09726	0.174614	0.153269	0.046842	-0.14638	0.055234	0.006893	0.918513	6856245	7.624611	7.717381	7.71204	7.877067	7.77365	7.842445	7.661211	7.795745	7.576398	7.874103	7.703327	7.747691	AI662250	NM_178926
6856254	6711	6.279078	6.24706	6.148055	6.428903	-0.14983	0.234338	-0.18184	0.157125	-0.28085	0.042439	0.099005	0.420063	0.032018	0.790384	0.131023	0.293281	6856254	6.297914	6.238921	6.300398	6.288035	6.27004	6.183105	6.272662	6.200026	5.971476	6.528551	6.167181	6.590976	Nrtn	NM_008738
6856257	6712	7.036216	7.036407	7.000732	7.159061	-0.12285	0.333238	-0.12265	0.333949	-0.15833	0.221059	0.035675	0.772519	-0.00019	0.998757	0.035484	0.773701	6856257	7.096235	6.979069	7.033343	7.125106	7.139069	6.845047	6.938302	7.230736	6.833158	7.165062	7.265454	7.046668	Rfx2	NM_027787
6856262	6713	8.200754	8.274008	8.187218	8.252708	-0.05195	0.64869	0.0213	0.850995	-0.06549	0.567309	0.08679	0.451998	-0.07325	0.523385	0.013537	0.904908	6856262	8.134297	8.270096	8.19787	8.293097	8.267207	8.26172	8.062559	8.466216	8.032878	8.144815	8.315937	8.297371	Mllt1	NM_022328
6856269	6716	8.560524	8.571077	8.614759	8.473107	0.087417	0.259396	0.09797	0.210773	0.141652	0.084728	-0.04368	0.560937	-0.01055	0.887117	-0.05423	0.472946	6856269	8.58754	8.572655	8.521376	8.473455	8.704867	8.534908	8.696083	8.572162	8.576031	8.369464	8.575131	8.474725	Gtf2f1	NM_133801
6856270	6717	9.419819	9.396896	9.453863	9.270395	0.149424	0.294378	0.126501	0.369956	0.183468	0.205598	-0.05697	0.68009	0.022923	0.867606	-0.03404	0.804672	6856270	9.372045	9.380562	9.506849	9.375812	9.587669	9.227206	9.353334	9.706266	9.301989	9.202667	9.430777	9.17774	Khsrp	NM_010613
6856276	6719	8.904298	8.955045	9.088958	9.288001	-0.3837	0.132423	-0.33296	0.184119	-0.19904	0.410161	-0.13391	0.574897	-0.05075	0.830208	-0.18466	0.443426	6856276	8.919243	8.945688	8.847962	9.135628	8.973929	8.755579	8.810841	9.598323	8.857711	9.072306	9.181804	9.609892	Slc25a23	NM_025877
6856279	6721	6.511271	6.558506	6.370926	6.371677	0.139594	0.457249	0.186829	0.326408	-0.00075	0.996749	0.187581	0.324584	-0.04724	0.798233	0.140345	0.454917	6856279	6.773475	6.307676	6.452662	6.45928	6.667073	6.549166	6.50041	6.429152	6.183215	6.102718	6.711141	6.301172	Tubb4	NM_009451
6856290	6724	12.52088	12.33163	12.2117	11.29566	1.22522	0.018478	1.03597	0.037361	0.916037	0.058611	0.119933	0.780236	0.18925	0.660945	0.309183	0.478181	6856290	12.78034	12.37456	12.40774	12.03455	12.71703	12.24331	12.44869	11.65754	12.52886	11.13273	12.15905	10.5952	C3	NM_009778
6856292	6725	9.15092	9.25037	9.217716	9.243773	-0.09285	0.2702	0.006597	0.934995	-0.02606	0.748123	0.032654	0.687944	-0.09945	0.240221	-0.0668	0.418926	6856292	9.202456	9.194481	9.055823	9.188983	9.196174	9.365954	9.344483	9.197542	9.111123	9.178011	9.223041	9.330267	Gpr108	NM_030084
6856367	6728	7.337923	7.321009	7.467802	7.488917	-0.15099	0.211059	-0.16791	0.16903	-0.02111	0.853954	-0.14679	0.222811	0.016914	0.882726	-0.12988	0.275899	6856367	7.16031	7.410332	7.443127	7.405387	7.372191	7.185448	7.293283	7.483608	7.626515	7.394359	7.573214	7.499178	Nudt12	NM_026497
6856480	6729	7.717633	7.745119	7.544082	7.393057	0.324575	0.067724	0.352062	0.051247	0.151025	0.354656	0.201037	0.227277	-0.02749	0.862538	0.17355	0.291623	6856480	7.602287	7.716722	7.833889	7.684094	7.939729	7.611534	7.624574	7.303969	7.703704	7.239757	7.279384	7.660031	Efna5	NM_207654
6856584	6731	9.019405	9.094509	9.230381	9.275865	-0.25646	0.028524	-0.18136	0.096088	-0.04548	0.648931	-0.13587	0.195466	-0.0751	0.457384	-0.21098	0.059599	6856584	8.958562	9.052138	9.047516	9.161276	9.12786	8.99439	9.140946	9.444009	9.106188	9.16773	9.370306	9.289558	Pja2	NM_001025309
6856674	6733	9.351623	9.449533	9.443443	9.439998	-0.08837	0.350631	0.009535	0.917472	0.003445	0.970129	0.00609	0.947221	-0.09791	0.304105	-0.09182	0.333228	6856674	9.361617	9.389646	9.303607	9.490098	9.431483	9.427017	9.497234	9.597747	9.235347	9.338334	9.53301	9.44865	Vapa	NM_013933

6856676	6735	8.102581	8.066757	7.764855	7.522031	0.58055	0.023573	0.544726	0.030772	0.242824	0.276896	0.301902	0.184925	0.035824	0.867604	0.337726	0.143288	6856676	8.304774	8.221066	7.781903	8.042897	8.347159	7.810215	7.991917	7.618265	7.684384	7.286993	7.80475	7.474351	Rab31	NM_133685
6856682	6736	9.721334	9.938644	9.767834	10.11013	-0.38879	0.019852	-0.17148	0.236529	-0.34229	0.033947	0.17081	0.23821	-0.21731	0.143537	-0.0465	0.73754	6856682	9.592384	9.837831	9.733786	10.03315	9.725402	10.05738	9.66589	9.968043	9.66957	9.978366	10.05259	10.29942	Ralbp1	NM_009067
6856689	6737	8.536645	8.665679	8.464717	8.7578	-0.22115	0.121087	-0.09212	0.49061	-0.29308	0.050599	0.200962	0.153686	-0.12903	0.341215	0.071928	0.588169	6856689	8.561034	8.554605	8.494297	8.655378	8.69725	8.644408	8.536195	8.56628	8.291676	8.53465	8.681225	9.057526	Twsg1	NM_023053
6856695	6738	9.774603	9.856839	9.869721	10.00999	-0.23539	0.122127	-0.15315	0.293313	-0.14027	0.333083	-0.01288	0.926957	-0.08224	0.562613	-0.09512	0.504634	6856695	9.677536	9.827806	9.818466	9.869073	9.798618	9.902827	9.968762	10.02207	9.618331	9.929402	9.83039	10.27018	Ndufv2	NM_028388
6856710	6739	6.802362	7.100062	6.929487	7.131765	-0.3294	0.081797	-0.0317	0.852903	-0.20228	0.256528	0.170576	0.332969	-0.2977	0.109837	-0.12712	0.464598	6856710	6.540523	6.979899	6.886665	7.181501	6.969244	7.149442	6.724783	7.158107	6.90557	7.031729	6.974735	7.38883	1110012J	NM_001114098
6856756	6740	9.360437	9.641812	9.208504	9.611673	-0.25124	0.173572	0.030139	0.862231	-0.40317	0.043364	0.433308	0.032798	-0.28138	0.132857	0.151933	0.392711	6856756	9.116735	9.426741	9.537834	9.612118	9.543521	9.769797	9.124161	9.186702	9.314649	9.508282	9.361415	9.965321	Ptprm	NM_008984
6856796	6741	7.168238	6.827718	7.177594	7.039975	0.128263	0.268313	-0.21226	0.084527	0.137619	0.237631	-0.34988	0.01179	0.34052	0.013432	-0.00936	0.932982	6856796	7.23623	7.245648	7.022836	6.753604	6.871679	6.857871	7.114989	7.101316	7.316478	6.924226	7.256422	6.939277	Arhgap28	NM_172964
6857022	6742	8.453167	8.283703	8.513684	8.16229	0.290877	0.080564	0.121413	0.428137	0.351394	0.042136	-0.22998	0.152536	0.169464	0.27759	-0.06052	0.688332	6857022	8.485594	8.466805	8.407103	8.360234	8.448969	8.041906	8.332561	8.415216	8.793276	8.094912	8.321376	8.070582	Tgif1	NM_009372
6857030	6743	11.48283	11.37267	11.40967	11.10258	0.380247	0.02246	0.270087	0.080054	0.307087	0.052217	-0.037	0.790659	0.11016	0.437443	0.07316	0.602085	6857030	11.53966	11.40321	11.50561	11.39687	11.57093	11.1502	11.48232	11.39717	11.34951	10.83564	11.19988	11.27222	Myl12b	NM_023402
6857037	6744	6.80047	6.764663	6.781554	6.577116	0.223354	0.466668	0.187547	0.539005	0.204438	0.50406	-0.01689	0.95533	0.035807	0.905511	0.018916	0.949982	6857037	6.763526	6.528636	7.109248	6.597591	7.381466	6.314932	6.686763	6.657137	7.000762	6.47244	6.351235	6.907672	2900073G	NM_026064
6857065	6745	7.518488	7.558658	7.44346	8.025254	-0.50677	0.099369	-0.4666	0.12451	-0.58179	0.064818	0.115198	0.682983	-0.04017	0.886213	0.075028	0.789603	6857065	7.876226	7.400318	7.278919	7.707779	7.38041	7.587784	7.322911	7.79965	7.207818	7.847535	7.673217	8.555011	Emilin2	NM_145158
6857075	6746	8.253558	8.244897	8.286455	8.208465	0.045093	0.536455	0.036432	0.615935	0.07799	0.2964	-0.04156	0.568145	0.00866	0.904343	-0.0329	0.650091	6857075	8.147691	8.27198	8.341002	8.099236	8.331278	8.304178	8.317621	8.283287	8.258457	8.15581	8.259132	8.210453	Smchd1	NM_028887
6857078	6747	5.918029	6.009145	5.947407	5.101917	0.816112	0.035605	0.907228	0.023005	0.84549	0.030913	0.061738	0.85335	-0.09112	0.78528	-0.02938	0.929849	6857078	5.879065	5.818819	6.056203	5.495755	6.547501	5.984179	6.297161	5.408245	6.136815	5.204366	4.729908	5.371477	Ndc80	NM_023294
6857183	6751	7.721802	7.590885	7.347838	7.469124	0.252678	0.425371	0.121762	0.696291	-0.12129	0.697408	0.243048	0.442532	0.130916	0.674957	0.373964	0.249062	6857183	7.965931	7.494034	7.70544	7.676836	7.921117	7.174703	7.606473	6.986352	7.450688	7.729875	7.77156	6.905936	Xdh	NM_011723
6857212	6753	9.23277	9.326311	9.420726	9.447334	-0.21456	0.112609	-0.12102	0.344272	-0.02661	0.830645	-0.09442	0.455549	-0.09354	0.459587	-0.18796	0.157145	6857212	9.156027	9.180995	9.361289	9.268978	9.410054	9.299901	9.604113	9.497491	9.160575	9.354475	9.399547	9.587979	Dpy30	NM_001146222
6857223	6754	8.978453	9.239756	8.931495	9.104931	-0.12648	0.47198	0.134825	0.444274	-0.17344	0.3309	0.30826	0.103087	-0.2613	0.1575	0.046958	0.786396	6857223	8.876192	8.975748	9.083419	9.11834	9.574515	9.026412	9.047888	8.760025	8.986573	8.880648	9.321143	9.113002	10004388	XR_034625
6857310	6755	9.277698	9.24323	9.325263	9.320294	-0.0426	0.685597	-0.07706	0.469223	0.00497	0.962127	-0.08203	0.442073	0.034468	0.742764	-0.04757	0.651648	6857310	9.396606	9.21498	9.221508	9.124308	9.369578	9.235805	9.322089	9.506319	9.147382	9.260921	9.315593	9.384367	Fam98a	NM_133747
6857415	6756	8.369558	8.415866	8.348711	8.437398	-0.06784	0.526423	-0.02153	0.838767	-0.08869	0.411834	0.067155	0.530506	-0.04631	0.663235	0.020847	0.843823	6857415	8.3315	8.367532	8.409641	8.419253	8.407826	8.420518	8.428776	8.311941	8.305416	8.166864	8.531749	8.613582	Fez2	NM_199448
6857431	6757	7.922789	8.050678	7.948251	7.775022	0.147767	0.080148	0.275656	0.005734	0.17323	0.04682	0.102427	0.202458	-0.12789	0.121234	-0.02546	0.73889	6857431	8.036069	7.922315	7.809983	8.031595	8.124874	7.995565	7.904236	8.020967	7.919551	7.677497	7.889101	7.758467	Strn	NM_011500
6857434	6759	7.962712	8.008142	7.973294	8.06315	-0.10044	0.404972	-0.05501	0.643072	-0.08986	0.454244	0.034848	0.768139	-0.04543	0.701298	-0.01058	0.928483	6857434	7.923913	8.066116	7.898108	7.953047	8.108036	7.963342	7.946898	8.019919	7.953065	7.785902	8.144063	8.259484	Heatr5b	NM_001081179
6857435	6760	8.461505	8.418475	8.344912	8.198437	0.263069	0.110789	0.220039	0.172147	0.146476	0.347439	0.073563	0.629669	0.04303	0.776813	0.116593	0.449822	6857435	8.470804	8.417872	8.49584	8.247967	8.577349	8.43011	8.175139	8.273922	8.585676	7.935526	8.278471	8.381313	Eif2ak2	NM_011163
6857440	6761	8.004894	8.024668	8.085279	8.040182	-0.03529	0.574394	-0.01551	0.803386	0.045097	0.475767	-0.06061	0.344071	-0.01977	0.751287	-0.08038	0.219049	6857440	8.003297	8.006208	8.005178	7.948422	8.133325	7.992258	8.127009	8.059386	8.069441	7.924995	8.132114	8.063436	Cebpz	NM_001024806
6857441	6762	7.480275	7.832175	7.481321	7.382865	0.09741	0.578709	0.44931	0.028392	0.098456	0.574721	0.350854	0.07063	-0.3519	0.06995	-0.00105	0.995194	6857441	7.713289	7.498841	7.228695	7.652436	8.033077	7.811012	7.674444	7.364345	7.405174	7.192898	7.615751	7.339945	Prkd3	NM_029239
6857461	6763	8.553909	8.821141	8.480367	8.351039	0.20287	0.089664	0.470102	0.002074	0.129328	0.253416	0.340774	0.011831	-0.26723	0.034557	0.073542	0.503897	6857461	8.41636	8.497531	8.747836	8.857005	8.852024	8.754395	8.615105	8.337332	8.488665	8.260147	8.483248	8.309722	Cdc42ep3	NM_026514
6857483	6764	7.795072	7.242472	6.989243	6.577349	1.217723	0.015602	0.665124	0.13328	0.411894	0.331041	0.253229	0.542436	0.552599	0.202495	0.805829	0.077526	6857483	8.485588	7.777985	7.121642	7.346162	7.645125	6.73613	7.376876	6.720903	6.86995	6.513061	6.996015	6.22297	Cyp1b1	NM_009994
6857501	6765	9.159166	9.203465	9.108287	9.254734	-0.09557	0.489826	-0.05127	0.707938	-0.14645	0.29961	0.095178	0.49154	-0.0443	0.745882	0.05088	0.710034	6857501	9.005857	9.111446	9.360196	9.287865	9.154943	9.167587	9.171056	9.211201	8.942603	9.162977	9.103576	9.497648	Atl2	NM_178050
6857512	6766	7.62847	7.784436	7.575749	7.586107	0.042363	0.653604	0.198329	0.060682	-0.01036	0.91208	0.208687	0.050798	-0.15597	0.124527	0.052721	0.577874	6857512	7.641877	7.663327	7.580206	7.718989	7.845587	7.788732	7.764699	7.425436	7.537111	7.47726	7.570452	7.710609	Hnrpll	NM_144802
6857523	6767	8.788753	9.071426	8.872497	8.806912	-0.01816	0.852492	0.264514	0.023289	0.065585	0.507559	0.198929	0.068528	-0.28267	0.017347	-0.08374	0.401637	6857523	8.730302	8.798957	8.837	8.834886	9.234504	9.144889	8.923344	8.905756	8.788392	8.823316	8.832841	8.764579	Sfrs7	NM_146083
6857526	6768	7.563728	7.542687	7.495694	7.365268	0.19846	0.042539	0.177419	0.063373	0.130426	0.15196	0.046992	0.583976	0.021041	0.804809	0.068034	0.432742	6857526	7.546516	7.562514	7.582154	7.473553	7.697149	7.457358	7.529506	7.393193	7.564384	7.42151	7.446092	7.228202	Dhx57	NM_198942
6857544	6769	8.506653	8.562084	8.494666	8.46272	0.043933	0.760372	0.099363	0.495627	0.031945	0.824242	0.067418	0.64113	-0.05543	0.700887	0.011987	0.933489	6857544	8.617544	8.54926	8.353155	8.472359	8.621564	8.592328	8.286014	8.77091	8.427073	8.294117	8.634424	8.45962	Sos1	NM_009231
6857639	6771	6.810716	6.955087	6.791345	6.133493	0.677223	0.016596	0.821593	0.006382	0.657852	0.018934	0.163742	0.48631	-0.14437	0.537909	0.019371	0.933317	6857639	6.859573	6.879422	6.693153	6.74773	7.131045	6.986485	6.983181	6.481606	6.909248	6.242377	6.493614	5.664489	Slc8a1	NM_011406
6857769	6774	6.486122	6.518097	6.57153	6.35767	0.128452	0.30642	0.160426	0.209571	0.213859	0.106432	-0.05343	0.66158	-0.03197	0.792551	-0.08541	0.488301	6857769	6.624338	6.442367	6.391662	6.40918	6.631404	6.513706	6.767035	6.508581	6.438973	6.34103	6.525769	6.206212	Haao	NM_025325
6857797	6775	9.058702	8.998646	8.826471	8.601251	0.457451	0.007632	0.397395	0.015248	0.22522	0.11962	0.172175	0.219569	0.060056	0.654602	0.23223	0.110131	6857797	9.204459	9.057381	8.914265	8.986971	9.242571	8.766396	8.919138	8.767164	8.793112	8.49117	8.738241	8.574342	Zfp36l2	NM_001001806
6857799	6776	7.430261	7.238647	7.310763	7.258704	0.171557	0.169715	-0.02006	0.864341	0.052058	0.659187	-0.07212	0.543558	0.191614	0.130379	0.119498	0.3239	6857799	7.416555	7.487887	7.386341	7.324484	7.33831	7.053148	7.165517	7.466488	7.300283	7.194856	7.442888	7.138369	Thada	EF222207
6857810	6778	7.575927	7.707242	7.595112	7.733823	-0.1579	0.243103	-0.02658	0.837297	-0.13871	0.300423	0.11213	0.396936	-0.13132	0.325231	-0.01918	0.882095	6857810	7.546517	7.56312	7.618145	7.671307	7.790317	7.660103	7.674687	7.550759	7.55989	7.454153	7.718278	8.029037	Lrpprc	NM_028233
6857834	6779	8.54108	8.626141	8.604524	8.781954	-0.24087	0.091321	-0.15581	0.249747	-0.17743	0.195297	0.021617	0.867571	-0.08506	0.517197	-0.06344	0.626957	6857834	8.578322	8.480345	8.564574	8.617018	8.674115	8.58729	8.623013	8.735451	8.455107	8.646807	8.612434	9.08662	Prepl	NM_145984
6857876	6781	6.187508	6.40595	6.189815	6.251982	-0.06447	0.629047	0.153968	0.264715	-0.06217	0.641194	0.216135	0.130763	-0.21844	0.127256	-0.00231	0.986101	6857876	6.153605	6.137656	6.271263	6.428051	6.41483	6.374969	6.334824	6.390372	5.84425	6.250572	6.30233	6.203043	Six2	NM_011380
6857963	6784	7.547446	7.805901	7.69702	7.914506	-0.36706	0.017038	-0.1086	0.400442	-0.21749	0.113244	0.108881	0.399296	-0.25846	0.067527	-0.14957	0.256128	6857963	7.341187	7.606295	7.694856	7.758237	7.934122	7.725345	7.804504	7.750183	7.536373	7.831396	7.821892	8.090229	Pigf	NM_008838
6857984	6785	8.157186	7.95359	7.994008	8.267473	-0.11029	0.334024	-0.31388	0.019122	-0.27347	0.034227	-0.04042	0.716161	0.203597	0.094293	0.163179	0.166748	6857984	8.216969	8.156784	8.097806	7.796782	7.896455	8.167532	8.149824	8.016744	7.815455	8.248222	8.285977	8.268221	Mcfd2	NM_176808
6858033	6786	6.080755	6.133493	5.955163	6.283376	-0.20262	0.143143	-0.14988	0.26413	-0.32821	0.030189	0.17833	0.190905	-0.05274	0.683744	0.125592	0.343737	6858033	6.093935	6.105654	6.042676	6.099174	5.972729	6.328577	5.974996	6.169951	5.720542	6.292078	6.1844	6.373649	Kcnk12	NM_199251
6858344	6792	9.61648	9.76497	9.712152	10.10176	-0.48528	0.034495	-0.33679	0.115464	-0.38961	0.075386	0.052818	0.788886	-0.14849	0.458716	-0.09567	0.629496	6858344	9.596205	9.557472	9.695762	9.890362	9.597572	9.806977	9.770277	9.91052	9.45566	9.793756	10.00067	10.51086	Sod2	NM_013671
6858456	6793	7.586458	7.636295	7.667561	7.54872	0.037738	0.686579	0.087575	0.359883	0.118841	0.224002	-0.03127	0.73773	-0.04984	0.595567	-0.0811	0.394682	6858456	7.652342	7.588169	7.518863	7.55371	7.755973	7.599203	7.790374	7.678132	7.534177	7.401925	7.642446	7.60179	Cul2	NM_029402
6858465	6794	7.933844	8.247691	7.985996	8.173991	-0.24015	0.355936	0.0737	0.77134	-0.188	0.465153	0.261695	0.316861	-0.31385	0.236298	-0.05215	0.836839	6858465	7.430423	7.84228	8.528829	8.209566	8.122588	8.410919	8.052505	7.971352	7.934132	8.24008	7.986677	8.295217	Bambi	NM_026505
6858520	6796	7.723465	7.739943	7.79641	7.820106	-0.09664	0.329893	-0.08016	0.414553	-0.0237	0.805629	-0.05647	0.561221	-0.01648	0.864001	-0.07295	0.456168	6858520	7.743553	7.707073	7.719769	7.652072	7.927156	7.6406	7.921687	7.678635	7.788908	7.704392	7.854392	7.901535	Mtpap	NM_026157
6858563	6797	8.721923	8.800831	8.703075	8.397786	0.324137	0.00258	0.403045	0.000678	0.305288	0.003636	0.097757	0.229835	-0.07891	0.324715	0.018848	0.808415	6858563	8.68925	8.680504	8.796015	8.764272	8.955854	8.682368	8.746142	8.691501	8.671581	8.311105	8.495625	8.386629	Svil	NM_153153
6858575	6798	8.093263	8.365992	7.980679	7.914011	0.179252	0.436577	0.451981	0.072822	0.066668	0.768478	0.385313	0.116413	-0.27273	0.248056	0.112584	0.620939	6858575	8.122372	8.113384	8.044034	8.254135	8.596052	8.247789	8.093129	7.666997	8.181912	7.482594	8.304682	7.954758	Zeb1	NM_011546
6858637	6800	8.76219	8.749461	8.658351	8.700857	0.061333	0.483742	0.048604	0.576668	-0.04251	0.624564	0.091111	0.307131	0.012729	0.882651	0.10384	0.249002	6858637	8.811842	8.815085	8.659644	8.680566	8.773012	8.794806	8.704449	8.71051	8.560093	8.529663	8.753853	8.819056	Rab18	NM_181070
6858694	6801	10.32759	10.35629	10.34838	10.29894	0.028647	0.700251	0.057343	0.447401	0.049437	0.510447	0.007907	0.914997	-0.0287	0.699758	-0.02079	0.779447	6858694	10.38166	10.38774	10.21337	10.2778	10.4627	10.32836	10.29514	10.47088	10.27912	10.27539	10.32652	10.29492	Wac	NR_027465
6858743	6802	7.360339	7.420692	7.440334	7.083318	0.27702	0.136015	0.337374	0.078233	0.357016	0.065173	-0.01964	0.909345	-0.06035	0.72737	-0.08	0.645013	6858743	7.4179	7.522446	7.14067	7.427943	7.577376	7.256758	7.65918	7.460097	7.201725	6.959437	6.948218	7.3423	Fzd8	NM_008058
6858773	6803	7.498482	7.40815	6.93401	6.88198	0.616502	0.027691	0.52617	0.051108	0.05203	0.826387	0.47414	0.072756	0.090332	0.704243	0.564472	0.039392	6858773	7.705464	7.534266	7.255715	7.349918	7.688946	7.185585	7.371482	6.624454	6.806094	6.740392	7.131614	6.773933	Colec12	NM_130449
6858784	6804	7.421518	7.571889	7.521471	7.363084	0.058435	0.630884	0.208805	0.112124	0.158387	0.212787	0.050418	0.677872	-0.15037	0.234636	-0.09995	0.41775	6858784	7.471334	7.485862	7.307359	7.361988	7.704584	7.649095	7.654005	7.483099	7.427308	7.249971	7.541834	7.297446	Thoc1	NM_153552
6858819	6806	8.903684	9.103843	8.898963	8.87606	0.027624	0.674902	0.227783	0.007097	0.022903	0.727563	0.20488	0.012094	-0.20016	0.013526	0.004721	0.94253	6858819	8.898673	8.95914	8.853239	9.027265	9.175683	9.108581	8.852373	9.00752	8.836995	8.78117	8.910313	8.936697	Mib1	NM_144860
6858842	6807	6.648088	6.655866	6.592615	6.563337	0.084751	0.275799	0.092529	0.237411	0.029278	0.696747	0.063251	0.408129	-0.00778	0.917157	0.055473	0.465899	6858842	6.708107	6.582629	6.653528	6.69744	6.752354	6.517804	6.71028	6.508646	6.558919	6.523181	6.59975	6.567081	Gata6	NM_010258
6858868	6808	7.360478	7.424652	7.443684	7.070292	0.290186	0.082088	0.35436	0.041392	0.373392	0.033783	-0.01903	0.899506	-0.06417	0.671907	-0.08321	0.5844	6858868	7.41153	7.413514	7.25639	7.283722	7.613172	7.377061	7.531035	7.298743	7.501273	6.973365	7.379371	6.85814	Rbbp8	NM_001081223
6858871	6809	5.823942	6.160687	5.767384	5.927327	-0.10338	0.558649	0.23336	0.205699	-0.15994	0.37278	0.393303	0.048811	-0.33674	0.082078	0.056558	0.747092	6858871	5.689899	5.990924	5.791004	6.253351	6.133584	6.095126	5.534299	6.113243	5.654611	5.925177	5.707922	6.148883	Cables1	NM_001146287
6858892	6810	8.419682	8.461037	8.480234	8.235347	0.184335	0.153918	0.22569	0.08996	0.244888	0.069769	-0.0192	0.873784	-0.04136	0.732967	-0.06055	0.618905	6858892	8.521814	8.381851	8.35538	8.490016	8.524344	8.368751	8.700007	8.491699	8.248997	8.086934	8.316062	8.303044	Riok3	NM_024182
6858895	6811	7.048921	7.066428	6.984163	6.995503	0.053418	0.596135	0.070925	0.484661	-0.01134	0.909628	0.082265	0.42012	-0.01751	0.860982	0.064759	0.522337	6858895	7.004874	7.071563	7.070327	7.012815	7.196045	6.990424	7.095679	7.104189	6.75262	6.955812	6.990189	7.040508	3110002H	NM_029623
6858910	6812	8.844072	8.686079	8.526847	8.817788	0.026284	0.914613	-0.13171	0.594385	-0.29094	0.25545	0.159232	0.521486	0.157992	0.524649	0.317225	0.218439	6858910	8.840312	8.698688	8.993215	8.278336	9.016911	8.762991	8.618649	8.360415	8.601477	9.095654	8.990679	8.367031	Ttc39c	NM_028341
6858936	6814	8.672614	8.768015	8.732018	8.735217	-0.0626	0.24178	0.032798	0.52641	-0.0032	0.950081	0.035997	0.488027	-0.0954	0.090224	-0.0594	0.264649	6858936	8.708796	8.731021	8.578024	8.81855	8.669658	8.815837	8.705598	8.743392	8.747063	8.74632	8.727431	8.731899	Impact	NM_008378
6859279	6817	6.263518	6.11139	6.235852	6.83792	-0.5744	0.264427	-0.72653	0.167505	-0.60207	0.243897	-0.12446	0.8014	0.152128	0.758741	0.027666	0.955323	6859279	6.555138	6.156317	6.0791	6.069332	5.799145	6.465693	6.182014	6.506007	6.019535	6.28928	8.066036	6.158443	Dsg1b	NM_181682
6859285	6820	9.369066	9.479459	9.471	9.403351	-0.03429	0.72987	0.076108	0.450168	0.067649	0.500436	0.008459	0.931858	-0.11039	0.28277	-0.10193	0.318694	6859285	9.302302	9.473663	9.331232	9.387045	9.414102	9.637229	9.52441	9.472899	9.41569	9.252345	9.390438	9.56727	Dsg2	NM_007883

6859304	6822	7.602489	8.084283	8.150713	7.657284	-0.0548	0.839663	0.426999	0.142017	0.493428	0.096589	-0.06643	0.806358	-0.48179	0.103401	-0.54822	0.069894	6859304	7.569178	7.775561	7.462727	7.885735	8.296285	8.070829	8.586535	7.94035	7.925253	7.76625	8.042566	7.163037	Rnf125	NM_026301
6859305	6823	6.69764	6.741957	6.765986	6.646717	0.050922	0.506759	0.09524	0.229874	0.119269	0.142235	-0.02403	0.751379	-0.04432	0.562054	-0.06835	0.378236	6859305	6.665466	6.70717	6.720283	6.625316	6.892453	6.708103	6.799541	6.829814	6.668604	6.572397	6.718669	6.649086	Rnf138	NM_207623
6859406	6825	7.039434	7.320445	6.641445	7.010571	0.028863	0.840851	0.309874	0.056553	-0.36913	0.029123	0.678999	0.001224	-0.28101	0.078101	0.397988	0.021139	6859406	6.912654	6.954515	7.251132	7.412604	7.34644	7.20229	6.848948	6.353982	6.721406	6.989165	7.085737	6.956811	Dtna	NM_207650
6859411	6826	8.006558	7.796391	7.965442	7.71138	0.295178	0.168015	0.085011	0.673961	0.254062	0.22826	-0.16905	0.41059	0.210167	0.311909	0.041116	0.838048	6859411	8.253306	7.943017	7.823352	7.935613	8.008741	7.44482	8.011882	8.080379	7.804066	7.42218	7.884024	7.827936	Mapre2	NM_153058
6859415	6827	7.914471	8.075116	8.005643	7.961169	-0.0467	0.595307	0.113946	0.214093	0.044473	0.612658	0.069473	0.434436	-0.16065	0.093579	-0.09117	0.311686	6859415	7.855978	7.969701	7.917733	8.024304	8.095349	8.105694	7.972936	8.158243	7.885749	7.805436	8.03567	8.042402	Zfp397	NM_027007
6859419	6828	6.159035	6.360184	6.334276	5.921578	0.237457	0.298173	0.438605	0.073913	0.412698	0.0892	0.025908	0.906372	-0.20115	0.373516	-0.17524	0.435362	6859419	6.07586	6.356477	6.044769	6.08419	6.6333	6.363061	6.332811	6.594174	6.075843	5.766598	6.287566	5.710571	Zfp35	NM_011755
6859428	6829	9.464761	9.312473	9.424906	9.137321	0.32744	0.143344	0.175152	0.410716	0.287585	0.191965	-0.11243	0.592678	0.152288	0.472103	0.039855	0.848369	6859428	9.51731	9.399556	9.477418	9.153817	9.422821	9.360782	9.912736	9.071315	9.290668	8.93594	9.237705	9.238318	Galnt1	NM_013814
6859471	6831	8.788112	8.867321	8.944313	8.74474	0.043373	0.700704	0.122581	0.292736	0.199573	0.104073	-0.07699	0.499435	-0.07921	0.487543	-0.1562	0.189189	6859471	8.728259	8.781373	8.854705	8.747128	9.005855	8.848979	9.095439	8.71658	9.02092	8.649679	8.847086	8.737454	Elp2	NM_021448
6859474	6832	6.5535	6.422914	6.20195	5.873225	0.680274	0.038333	0.549689	0.0804	0.328725	0.265719	0.220964	0.444447	0.130586	0.647247	0.351549	0.236519	6859474	6.313744	6.453847	6.892908	6.020882	7.006859	6.241001	6.375811	5.85076	6.37928	5.872206	5.839859	5.907611	Fhod3	NM_175276
6859707	6833	7.951907	7.934339	7.854794	7.830299	0.121609	0.705707	0.104041	0.746332	0.024495	0.939095	0.079546	0.804387	0.017568	0.956294	0.097114	0.762594	6859707	7.500235	7.578414	8.777073	7.816782	8.083281	7.902955	7.954505	7.679163	7.930713	7.997567	7.811481	7.681848	Pik3c3	NM_181414
6859775	6834	9.279906	9.218603	9.248221	9.214917	0.064988	0.544872	0.003685	0.972278	0.033304	0.754251	-0.02962	0.780563	0.061303	0.567406	0.031685	0.76577	6859775	9.249303	9.22296	9.367454	9.245991	9.258277	9.15154	9.030069	9.431843	9.282751	9.169806	9.343713	9.131233	Sap130	NM_172965
6859778	6835	8.241337	8.320142	8.09468	8.109451	0.131886	0.265027	0.210691	0.091877	-0.01477	0.896538	0.225462	0.074644	-0.07881	0.494286	0.146657	0.219336	6859778	8.350226	8.161194	8.212591	8.283169	8.475161	8.202097	7.873588	8.265057	8.145396	8.174198	8.069714	8.084442	Ammecr1l	NM_153515
6859779	6836	7.664787	7.819078	7.562483	7.539652	0.125135	0.47393	0.279426	0.131889	0.022831	0.894345	0.256596	0.161935	-0.15429	0.381297	0.102305	0.556071	6859779	7.611459	7.639493	7.74341	7.766304	7.972851	7.71808	7.922299	7.527527	7.237622	7.365775	7.576916	7.676265	Polr2d	NM_027002
6859781	6837	8.207718	8.318293	8.185479	8.097274	0.110444	0.16773	0.221018	0.016164	0.088205	0.260245	0.132814	0.105552	-0.11057	0.167284	0.022239	0.767803	6859781	8.202078	8.321596	8.09948	8.241916	8.386025	8.326937	8.273514	8.147463	8.13546	8.023984	8.06821	8.199629	Wdr33	NM_028866
6859790	6838	7.985787	7.932641	7.96431	8.351513	-0.36573	0.033085	-0.41887	0.018584	-0.3872	0.026176	-0.03167	0.829415	0.053146	0.718421	0.021477	0.883737	6859790	7.988556	8.125293	7.843512	8.278725	7.783034	7.736164	8.055419	7.982137	7.855374	8.384047	8.347727	8.322764	Lims2	NM_144862
6859800	6839	7.547266	7.546083	7.480523	7.454477	0.09279	0.123727	0.091606	0.127908	0.026046	0.642151	0.06556	0.258886	0.001183	0.983036	0.066744	0.251071	6859800	7.537955	7.551804	7.55204	7.489366	7.60248	7.546403	7.508016	7.456291	7.477261	7.335132	7.567412	7.460886	Iws1	ENSMUST000000
6859809	6840	8.437965	8.593215	8.331668	8.310412	0.127552	0.130983	0.282803	0.005786	0.021256	0.786305	0.261547	0.008696	-0.15525	0.074763	0.106296	0.19848	6859809	8.313326	8.44727	8.553298	8.540956	8.611499	8.627191	8.430215	8.282832	8.281958	8.191359	8.370657	8.369221	Map3k2	NM_011946
6859811	6841	8.732487	8.685409	8.727575	8.692943	0.039544	0.58361	-0.00753	0.916042	0.034633	0.630418	-0.04217	0.559433	0.047077	0.515753	0.004911	0.945194	6859811	8.720872	8.737784	8.738804	8.673641	8.835403	8.547184	8.77423	8.709676	8.69882	8.602704	8.742947	8.733177	Ercc3	NM_133658
6859814	6842	8.380605	8.28182	8.273692	8.092317	0.288289	0.293036	0.189504	0.480571	0.181375	0.499017	0.008129	0.975462	0.098785	0.709816	0.106914	0.687382	6859814	8.155045	8.077607	8.909164	8.320478	8.345911	8.179072	8.68408	8.030008	8.106987	7.849315	8.263946	8.163689	Bin1	NM_009668
6859836	6844	7.728098	7.709344	7.865603	7.628816	0.099282	0.512557	0.080527	0.593549	0.236787	0.140838	-0.15626	0.312254	0.018754	0.900204	-0.13751	0.370377	6859836	7.761113	7.769335	7.653846	7.537548	7.936732	7.653751	8.035829	7.607672	7.953309	7.488987	7.814416	7.583046	Wdr36	NM_144863
6859837	6845	7.355777	7.292127	7.431728	7.147898	0.20788	0.183587	0.144229	0.342096	0.28383	0.082096	-0.1396	0.356939	0.063651	0.66763	-0.07595	0.609293	6859837	7.386686	7.354525	7.326121	7.263353	7.552618	7.060409	7.492358	7.334805	7.468021	6.954295	7.40268	7.086718	Wdr36	NM_001110015
6859935	6847	9.774933	9.791408	9.775245	9.628189	0.146744	0.26299	0.163219	0.217303	0.147056	0.262057	0.016163	0.897769	-0.01647	0.895811	-0.00031	0.998022	6859935	9.746732	9.8603	9.717768	9.653796	10.0053	9.715129	9.986032	9.732514	9.607189	9.535201	9.741476	9.607891	Apc	NM_007462
6859937	6848	7.241738	7.28898	7.267747	7.1923	0.049438	0.671853	0.09668	0.414975	0.075446	0.521189	0.021233	0.854943	-0.04724	0.685479	-0.02601	0.822907	6859937	7.202971	7.067108	7.455135	7.190465	7.482076	7.194399	7.293803	7.288759	7.220678	7.234803	7.249288	7.09281	Srp19	NM_025527
6859953	6851	6.476933	6.752045	6.19747	5.806448	0.670485	0.062586	0.945596	0.01584	0.391022	0.242869	0.554574	0.111527	-0.27511	0.400886	0.279463	0.393818	6859953	6.513791	6.270459	6.646549	6.348925	7.441172	6.466037	6.469714	5.709366	6.413331	5.767354	5.805852	5.846139	Kif20a	NM_009004
6859967	6852	7.440601	7.468989	7.349679	7.359791	0.08081	0.592903	0.109198	0.473375	-0.01011	0.946166	0.119309	0.434861	-0.02839	0.849803	0.090921	0.548469	6859967	7.574734	7.421566	7.322561	7.439991	7.713354	7.253621	7.31627	7.572042	7.160726	7.313566	7.271633	7.494174	Fam53c	BC057111
6859971	6853	7.064343	7.06092	6.798928	6.535281	0.529063	0.021926	0.525639	0.022555	0.263647	0.1952	0.261992	0.197731	0.003424	0.985804	0.265416	0.192527	6859971	7.309289	6.796573	7.087168	7.026299	7.372532	6.783928	6.686545	6.665631	7.044607	6.635667	6.531593	6.438582	Reep2	NM_144865
6859972	6854	8.187191	7.957631	7.887311	7.480975	0.706216	0.006067	0.476656	0.037207	0.406336	0.066073	0.07032	0.722319	0.22956	0.263806	0.29988	0.155069	6859972	8.19224	7.994796	8.374536	7.702111	8.270645	7.900136	7.849704	7.863744	7.948485	7.195189	7.812145	7.43559	Egr1	NM_007913
6860001	6855	10.54486	10.48421	10.54688	10.14726	0.397597	0.017645	0.336947	0.035586	0.399617	0.017243	-0.06267	0.651277	0.06065	0.661672	-0.00202	0.988298	6860001	10.58082	10.50137	10.55238	10.3789	10.58948	10.48424	10.84835	10.28881	10.50347	10.02545	10.26655	10.14978	Ctnna1	NM_009818
6860034	6856	9.537095	9.750315	9.791023	9.721736	-0.18464	0.091308	0.028579	0.774056	0.069287	0.492042	-0.04071	0.683438	-0.21322	0.057576	-0.25393	0.029778	6860034	9.510069	9.643306	9.457909	9.618067	9.779322	9.853556	9.952174	9.728621	9.692273	9.604272	9.738318	9.822618	Matr3	NM_010771
6860035	6857	8.07382	7.852511	8.012851	8.24297	-0.16915	0.409726	-0.39046	0.079536	-0.23012	0.270632	-0.16034	0.433531	0.221309	0.288027	0.060969	0.761897	6860035	7.748857	8.161419	8.311184	7.591372	8.120095	7.846067	7.833561	8.258865	7.946126	8.420205	8.161022	8.147683	Pnet-ps	AY223547
6860038	6858	10.99752	11.01464	10.93631	11.09306	-0.09554	0.400754	-0.07842	0.487133	-0.15674	0.183507	0.078323	0.487654	-0.01712	0.877617	0.061207	0.58528	6860038	10.95231	11.00293	11.03732	10.88251	11.08988	11.07152	11.03623	11.05356	10.71915	11.01226	11.01431	11.2526	Paip2	NM_026420
6860049	6859	8.498559	8.320699	8.312305	8.39831	0.10025	0.473199	-0.07761	0.576139	-0.086	0.536525	0.008395	0.95129	0.17786	0.21849	0.186255	0.199532	6860049	8.609147	8.570376	8.316155	8.393459	8.323196	8.245443	8.355602	8.465813	8.115499	8.229163	8.334292	8.631474	Ube2d2	NM_019912
6860068	6860	7.777498	8.044109	7.971436	7.631181	0.146317	0.112623	0.412928	0.001016	0.340255	0.003237	0.072673	0.401952	-0.26661	0.011755	-0.19394	0.045825	6860068	7.672784	7.887174	7.772535	7.999044	8.116216	8.017068	7.9712	8.028981	7.914128	7.521484	7.798466	7.573593	Cxxc5	NM_133687
6860102	6863	7.671578	7.870559	7.73256	7.819039	-0.14746	0.38341	0.05152	0.755568	-0.08648	0.603368	0.137999	0.413262	-0.19898	0.248588	-0.06098	0.712866	6860102	7.677525	7.633239	7.70397	7.932587	8.082099	7.596992	7.770376	7.986619	7.440685	7.683331	7.918999	7.854787	Pura	NM_008989
6860111	6864	9.221524	9.195069	9.201895	9.334016	-0.11249	0.413855	-0.13895	0.318157	-0.13212	0.341057	-0.00683	0.95957	0.026456	0.844438	0.019629	0.884182	6860111	9.060708	9.244348	9.359517	9.24672	9.077169	9.261317	9.351228	9.269036	8.985422	9.231012	9.22984	9.541197	0610010O	NM_001081365
6860119	6866	7.311241	7.28576	7.231024	7.211631	0.09961	0.508912	0.074129	0.620814	0.019393	0.896255	0.054736	0.713908	0.02548	0.864024	0.080217	0.592931	6860119	7.165509	7.25216	7.516053	7.268806	7.412396	7.176079	7.073908	7.295206	7.323958	7.321749	7.378443	6.934701	Ankhd1	NM_175375
6860121	6867	9.094013	9.077448	9.283555	9.107836	-0.01382	0.908577	-0.03039	0.801018	0.17572	0.170314	-0.20611	0.115169	0.016565	0.890566	-0.18954	0.142771	6860121	9.100147	9.104391	9.077501	9.032206	9.254852	8.945287	9.348724	9.320105	9.181837	8.951277	9.358047	9.014183	Slc35a4	NM_026404
6860127	6868	6.407957	6.400158	6.456804	6.428492	-0.02053	0.7644	-0.02833	0.68001	0.028312	0.680253	-0.05665	0.417184	0.0078	0.90914	-0.04885	0.481792	6860127	6.248805	6.561889	6.413178	6.394651	6.373093	6.432729	6.449873	6.485547	6.434992	6.444328	6.423437	6.417712	Tmco6	NM_028036
6860128	6869	9.583696	9.763753	9.793204	9.753019	-0.16932	0.077601	0.010734	0.901082	0.040185	0.643874	-0.02945	0.733929	-0.18006	0.063568	-0.20951	0.036707	6860128	9.54799	9.669638	9.533461	9.639389	9.792293	9.859578	9.823354	9.835628	9.72063	9.595736	9.865349	9.797973	Ik	NM_011879
6860129	6870	8.120096	8.176857	8.251167	7.851773	0.268323	0.192709	0.325084	0.12313	0.399394	0.067115	-0.07431	0.703928	-0.05676	0.771169	-0.13107	0.506844	6860129	8.047834	8.032236	8.280218	7.930614	8.459118	8.140838	8.443282	7.925697	8.384523	7.612595	7.985687	7.957037	Wdr55	NM_026464
6860133	6871	6.76028	6.859071	6.8497	6.760627	-0.00035	0.99512	0.098444	0.110927	0.089073	0.143617	0.009371	0.868794	-0.09879	0.109862	-0.08942	0.142264	6860133	6.748832	6.818632	6.713377	6.851097	6.965525	6.760592	6.902103	6.792732	6.854265	6.721671	6.756627	6.803583	Hars2	NM_080636
6860135	6872	8.829748	8.862869	8.918401	8.852093	-0.02234	0.762118	0.010776	0.883666	0.066308	0.379819	-0.05553	0.45871	-0.03312	0.654812	-0.08865	0.249154	6860135	8.878133	8.863441	8.74767	8.86855	8.830112	8.889945	9.048302	8.902263	8.804637	8.74784	8.940521	8.867917	Zmat2	NM_025594
6860138	6873	6.785404	6.680011	6.723031	6.874323	-0.08892	0.242345	-0.19431	0.024721	-0.15129	0.063974	-0.04302	0.558298	0.105393	0.172935	0.062373	0.401689	6860138	6.76159	6.907127	6.687495	6.634471	6.691855	6.713708	6.586103	6.788313	6.794676	6.862121	6.842711	6.918137	Pcdha6	NM_007767
6860167	6877	6.944609	7.017781	6.864666	6.807911	0.136697	0.432385	0.209869	0.240055	0.056755	0.740276	0.153115	0.381553	-0.07317	0.669843	0.079943	0.64173	6860167	7.125093	7.087655	6.621078	6.954775	7.036507	7.06206	7.10579	6.574588	6.91362	6.870359	6.69423	6.859145	Pcdhb4	NM_053129
6860224	6896	6.192273	6.284629	6.26661	6.30147	-0.1092	0.102467	-0.01684	0.783369	-0.03486	0.572381	0.018019	0.76871	-0.09236	0.157537	-0.07434	0.244867	6860224	6.289096	6.15404	6.133684	6.360765	6.285056	6.208066	6.251525	6.19031	6.357994	6.268268	6.325603	6.310539	Rell2	NM_153793
6860252	6897	7.724513	7.728234	7.811405	7.995785	-0.27127	0.038014	-0.26755	0.040086	-0.18438	0.130136	-0.08317	0.468566	-0.00372	0.973681	-0.08689	0.449605	6860252	7.738777	7.728318	7.706445	7.831363	7.734738	7.618601	7.806555	7.998497	7.629164	7.897755	7.907885	8.181715	0610009O	NM_024179
6860254	6898	9.081951	9.022556	9.159359	8.993322	0.088629	0.387008	0.029234	0.770526	0.166037	0.124894	-0.1368	0.195604	0.059395	0.556837	-0.07741	0.447336	6860254	9.024907	9.091581	9.129364	9.093479	9.111647	8.862542	9.269011	9.201975	9.007092	8.848645	9.069674	9.061647	Rnf14	NM_020012
6860256	6899	10.29545	10.38267	10.37706	10.4303	-0.13485	0.187857	-0.04763	0.624771	-0.05324	0.585329	0.005607	0.95373	-0.08722	0.378939	-0.08161	0.408899	6860256	10.38986	10.29025	10.20623	10.36498	10.39826	10.38476	10.55867	10.20684	10.36567	10.40823	10.32959	10.55307	Ndfip1	NM_022996
6860259	6900	8.646231	8.5444	8.489797	8.830607	-0.18438	0.204956	-0.28621	0.064575	-0.34081	0.03414	0.054603	0.693515	0.101831	0.467862	0.156434	0.275382	6860259	8.863007	8.555435	8.520251	8.663697	8.551782	8.41772	8.422103	8.710073	8.337215	8.890168	8.67614	8.925513	Arhgap26	NM_175164
6860411	6902	6.189592	6.185297	5.970787	6.102195	0.087397	0.81673	0.083103	0.825558	-0.13141	0.728075	0.214511	0.572835	0.004294	0.990898	0.218805	0.565339	6860411	5.879789	5.954858	6.734128	6.421248	6.273766	5.860878	6.092332	5.911388	5.90864	5.548195	6.878639	5.87975	Sh3rf2	NM_001146299
6860424	6903	8.76918	8.773137	8.757127	8.827517	-0.05834	0.622994	-0.05438	0.646422	-0.07039	0.554468	0.016011	0.891884	-0.00396	0.973187	0.012054	0.918474	6860424	8.820255	8.858206	8.62908	8.712436	8.911422	8.695554	8.681257	9.002597	8.587526	8.816978	8.866843	8.79873	Rbm27	NM_172626
6860430	6905	8.860768	9.00318	8.875981	9.207227	-0.34646	0.060113	-0.20405	0.233555	-0.33125	0.069815	0.127199	0.44502	-0.14241	0.394725	-0.01521	0.925823	6860430	8.606315	8.978878	8.99711	8.945616	9.038623	9.025302	8.888922	8.707274	9.031748	8.983873	9.507086	9.130722	LOC10004	XM_001479156
6860436	6906	8.32082	8.251991	8.264471	8.07732	0.243499	0.087708	0.174671	0.200527	0.187151	0.173359	-0.01248	0.923056	0.068829	0.597534	0.056348	0.664638	6860436	8.409693	8.270363	8.282403	8.084317	8.464855	8.206801	8.403656	8.122346	8.267412	7.883152	8.222447	8.126362	Tcerg1	NM_001039474
6860476	6907	7.775593	8.22346	7.717657	7.123098	0.652495	0.205117	1.100362	0.048455	0.594559	0.244255	0.505804	0.316157	-0.44787	0.371472	0.057937	0.905543	6860476	7.98787	8.042138	7.296772	7.93813	7.924366	8.807885	7.912375	7.018463	8.222132	7.129379	7.843194	6.396721	Stk32a	NM_178749
6860507	6908	4.414264	6.785194	4.414294	5.346581	-0.93232	0.256091	1.438613	0.095819	-0.93229	0.256105	2.3709	0.014437	-2.37093	0.014436	-3E-05	0.999969	6860507	4.389303	4.395675	4.457814	7.947661	6.732479	5.675443	4.774631	4.101729	4.366523	5.495286	6.707634	3.836824	Scgb3a2	NM_054038
6860520	6911	7.230834	7.342593	7.303853	7.236554	-0.00572	0.996945	0.106039	0.943411	0.067299	0.964064	0.03874	0.979308	-0.11176	0.940366	-0.07302	0.961012	6860520	4.91748	6.794983	9.98004	8.880771	7.296983	5.850025	8.427711	7.359284	6.124564	5.448735	8.185276	8.075651	Myot	NM_001033621
6860576	6912	7.380733	6.764921	7.205769	6.847042	0.533691	0.018655	-0.08212	0.6628	0.358727	0.083378	-0.44085	0.041194	0.615812	0.009436	0.174964	0.363055	6860576	7.635341	7.247226	7.259633	6.753229	7.063835	6.477699	7.347555	7.274699	6.995053	7.033682	6.799047	6.708398	Kcnn2	NM_080465
6860635	6913	8.367436	8.30612	8.31895	8.042519	0.324917	0.089036	0.263601	0.155098	0.27643	0.138336	-0.01283	0.940975	0.061316	0.724464	0.048486	0.780114	6860635	8.386316	8.396267	8.319725	8.121318	8.654737	8.142306	8.466809	8.301238	8.188802	8.133363	8.221688	7.772507	Eif1a	NM_010120
6860666	6914	7.60488	7.730757	7.81633	7.862343	-0.25746	0.058828	-0.13159	0.293081	-0.04601	0.704228	-0.08557	0.485166	-0.12588	0.313095	-0.21145	0.108171	6860666	7.600763	7.519243	7.694633	7.69338	7.778086	7.720805	7.812713	7.990915	7.645361	7.743128	7.743212	8.100689	Commd10	NM_178377
6860778	6916	8.391892	8.405778	8.405538	8.497072	-0.10518	0.099563	-0.09129	0.144641	-0.09153	0.143725	0.000239	0.996722	-0.01389	0.811976	-0.01365	0.815146	6860778	8.387445	8.412662	8.37557	8.358232	8.472584	8.386517	8.394511	8.51753	8.304574	8.50164	8.556319	8.433256	Dmxl1	NM_001081371
6860780	6917	8.084312	8.689949	8.568271	9.018684	-0.93437	0.01188	-0.32874	0.287319	-0.45041	0.156966	0.121678	0.684202	-0.60564	0.068938	-0.48396	0.131849	6860780	8.015295	8.305855	7.931786	8.471234	8.868262	8.73035	8.560816	8.96366	8.180337	8.500758	9.022243	9.533051	Tnfaip8	NM_134131

6860787	6918	9.062999	9.133134	9.154916	9.044262	0.018737	0.876287	0.088872	0.4677	0.110654	0.370279	-0.02178	0.856424	-0.07014	0.564052	-0.09192	0.45312	6860787	9.111934	8.980011	9.097051	9.14693	9.151084	9.101389	9.366934	9.018667	9.079147	8.844707	9.038902	9.249177	Hsd17b4	NM_008292
6860892	6920	6.226426	6.285362	6.465413	6.289686	-0.06326	0.552077	-0.00432	0.967197	0.175776	0.12288	-0.1801	0.115202	-0.05894	0.579003	-0.23904	0.047024	6860892	6.435246	6.036632	6.207399	6.228346	6.259644	6.368096	6.423457	6.418784	6.554147	6.39768	6.187698	6.283681	Ftmt	NM_026286
6860903	6921	7.232606	7.330217	7.308017	7.359736	-0.12713	0.169747	-0.02952	0.735107	-0.05172	0.55635	0.0222	0.798826	-0.09761	0.280041	-0.07541	0.396876	6860903	7.279908	7.228654	7.189257	7.155465	7.444551	7.390636	7.335849	7.382318	7.205885	7.262333	7.446038	7.370838	Srfbp1	NM_026040
6860925	6926	9.491804	9.545061	9.651062	9.512025	-0.02022	0.82433	0.033036	0.71759	0.139037	0.153412	-0.106	0.263584	-0.05326	0.562498	-0.15926	0.108465	6860925	9.46376	9.442034	9.569617	9.540019	9.650553	9.444612	9.792568	9.627319	9.533299	9.418757	9.471453	9.645829	Snx2	NM_026386
6860929	6927	7.185359	7.56978	7.443714	7.079909	0.10545	0.554573	0.489871	0.020993	0.363804	0.066036	0.126066	0.482018	-0.38442	0.05472	-0.25835	0.169251	6860929	7.09449	7.085734	7.375853	7.468246	7.794532	7.446562	7.727379	7.086099	7.517663	7.013811	7.11302	7.112897	Snx24	NM_029394
6860952	6928	8.369252	8.496341	8.422424	8.3329	0.036352	0.806408	0.163442	0.287677	0.089524	0.550094	0.073918	0.62041	-0.12709	0.401651	-0.05317	0.720605	6860952	8.332382	8.440682	8.334691	8.27554	8.709005	8.504479	8.59555	8.157797	8.513924	8.176657	8.425537	8.396505	Csnk1g3	NM_152809
6860967	6929	8.993275	8.948663	8.921277	8.82111	0.172164	0.119032	0.127552	0.232017	0.100166	0.339555	0.027386	0.788309	0.044612	0.663052	0.071998	0.486222	6860967	9.057778	8.953096	8.96895	8.820951	9.082663	8.942374	8.913584	8.804973	9.045273	8.655857	8.959129	8.848345	Csnk1g3	NM_152809
6861135	6930	7.318873	7.386319	7.416167	7.489309	-0.17044	0.197091	-0.10299	0.419948	-0.07314	0.562718	-0.02985	0.811586	-0.06745	0.592922	-0.09729	0.445094	6861135	7.234275	7.31551	7.406835	7.35351	7.532805	7.272643	7.303323	7.686161	7.259017	7.412615	7.47026	7.585051	Gramd3	NM_026240
6861136	6931	7.693348	7.824223	7.621536	7.552052	0.141296	0.169881	0.27217	0.019722	0.069484	0.47943	0.202687	0.062419	-0.13087	0.1999	0.071812	0.465316	6861136	7.601931	7.752632	7.725482	7.709797	7.984411	7.77846	7.734537	7.605567	7.524504	7.445157	7.528165	7.682835	Phax	NM_019996
6861175	6934	8.509087	8.440223	8.503974	8.671038	-0.16195	0.296187	-0.23081	0.149876	-0.16706	0.282252	-0.06375	0.671635	0.068864	0.647341	0.005113	0.972717	6861175	8.482817	8.55701	8.487435	8.372382	8.597737	8.35055	8.344812	8.847672	8.319438	8.674705	8.798733	8.539676	Prrc1	NM_028447
6861200	6935	9.3238	9.581604	9.164331	8.918234	0.405566	0.038492	0.66337	0.003705	0.246097	0.171752	0.417273	0.03444	-0.2578	0.154503	0.159469	0.359223	6861200	9.279987	9.371618	9.319796	9.493616	9.577591	9.673606	9.473978	8.777994	9.241022	8.734814	8.991889	9.028	Slc12a2	NM_009194
6861260	6936	8.440544	8.696185	8.576537	8.345435	0.095108	0.384048	0.35075	0.009415	0.231102	0.055633	0.119648	0.280084	-0.25564	0.038398	-0.13599	0.224396	6861260	8.401592	8.521462	8.398577	8.548092	8.69248	8.847984	8.660073	8.589131	8.480408	8.153514	8.413457	8.469335	Isoc1	NM_025478
6861308	6940	6.677045	6.233912	6.174215	5.37016	1.306884	0.015414	0.863751	0.077196	0.804055	0.095858	0.059697	0.892041	0.443133	0.328746	0.50283	0.271856	6861308	6.653786	6.972265	6.405083	5.658143	6.865028	6.178564	6.187646	5.588262	6.746737	4.924313	5.989694	5.196474	EG240327	NM_001033767
6861314	6941	6.100637	5.797458	5.5334	4.882853	1.217784	0.056099	0.914605	0.132162	0.650548	0.267238	0.264057	0.641336	0.303179	0.593584	0.567236	0.328847	6861314	5.709843	6.56339	6.028677	5.425808	6.072411	5.894154	5.092468	4.751337	6.756396	4.502397	5.551283	4.594878	BC023105	BC023105
6861321	6942	9.100158	9.181101	9.238456	8.9738	0.126358	0.293812	0.207301	0.102546	0.264655	0.046454	-0.05735	0.623849	-0.08094	0.492206	-0.1383	0.253765	6861321	9.018914	9.082148	9.199413	9.065815	9.400765	9.076723	9.417466	9.115949	9.181952	8.933246	9.064314	8.923841	Dctn4	NM_026302
6861322	6943	8.537224	8.64129	8.541277	8.431529	0.105695	0.277296	0.209761	0.049418	0.109748	0.260657	0.100013	0.302071	-0.10407	0.284227	-0.00405	0.965444	6861322	8.529689	8.576907	8.505077	8.567191	8.872812	8.483867	8.598102	8.458237	8.567492	8.452352	8.403149	8.439086	Rbm22	NM_025776
6861340	6944	12.19953	12.22433	12.2665	12.44193	-0.2424	0.110736	-0.21759	0.146173	-0.17543	0.230587	-0.04217	0.76309	-0.0248	0.858997	-0.06697	0.63366	6861340	12.11357	12.3047	12.18032	12.15854	12.18013	12.33433	12.46277	12.40334	11.93339	12.45119	12.35427	12.52032	Rps14	NM_020600
6861341	6945	12.29174	12.14172	12.29283	11.37315	0.918593	0.012956	0.768577	0.028696	0.919687	0.012882	-0.15111	0.614837	0.150017	0.617357	-0.00109	0.997071	6861341	12.58792	12.36423	11.92307	12.23989	12.28193	11.90335	12.468	12.06849	12.34201	11.65415	11.72412	10.74117	Cd74	NM_001042605
6861354	6947	9.064054	8.854004	8.617416	8.421524	0.64253	0.060531	0.432481	0.179843	0.195892	0.52433	0.236588	0.444632	0.210049	0.495641	0.446638	0.167543	6861354	9.38299	9.144973	8.664198	8.769679	9.148912	8.643422	8.770264	8.460861	8.621123	7.851842	8.927544	8.485185	Pdgfrb	NM_001146268
6861358	6948	9.407477	9.24492	9.301755	8.134327	1.27315	0.005719	1.110593	0.011509	1.167428	0.008982	-0.05684	0.8716	0.162558	0.645902	0.105722	0.764164	6861358	9.820145	9.228336	9.173951	9.259528	9.592626	8.882605	9.765077	8.895986	9.244202	7.80533	8.70962	7.888031	Csf1r	NM_001037859
6861389	6950	10.52701	10.57569	10.44995	10.48964	0.037373	0.574399	0.086053	0.214593	-0.03969	0.551463	0.12574	0.084392	-0.04818	0.467624	0.07706	0.261874	6861389	10.53545	10.50796	10.53762	10.48811	10.66305	10.57591	10.50388	10.45866	10.38731	10.35763	10.55338	10.5579	Csnk1a1	NM_146087
6861408	6952	7.118579	7.27542	7.058029	7.164697	-0.04612	0.680039	0.110723	0.334364	-0.10667	0.351347	0.217391	0.078441	-0.15684	0.18373	0.06055	0.589672	6861408	6.98408	7.077819	7.293837	7.392152	7.285135	7.148973	7.194971	7.00039	6.978725	7.113802	7.073971	7.306317	Sh3tc2	NM_172628
6861437	6953	6.271866	6.120849	6.281638	6.286931	-0.01507	0.884795	-0.16608	0.137751	-0.00529	0.959378	-0.16079	0.149047	0.151017	0.172142	-0.00977	0.925087	6861437	6.242383	6.467093	6.106121	6.008899	6.169878	6.183769	6.215006	6.345598	6.284311	6.395882	6.161644	6.303267	Htr4	NM_008313
6861468	6956	7.648238	7.694624	7.666788	7.621455	0.026783	0.808095	0.073169	0.512169	0.045333	0.682073	0.027836	0.800733	-0.04639	0.675183	-0.01855	0.866274	6861468	7.725559	7.542197	7.676958	7.636699	7.80205	7.645124	7.857037	7.628581	7.514746	7.464189	7.740711	7.659465	Napg	NM_028017
6861551	6958	5.921744	5.83578	5.800073	6.089033	-0.16729	0.095525	-0.25325	0.021145	-0.28896	0.011468	0.035707	0.697318	0.085964	0.360052	0.121671	0.206668	6861551	5.843639	6.014548	5.907045	5.906682	5.805295	5.795362	5.885069	5.907702	5.607448	6.190452	6.027806	6.048842	Onecut2	NM_194268
6861576	6959	10.27372	10.3873	10.37309	10.02425	0.249469	0.150646	0.363047	0.049462	0.34884	0.056966	0.014207	0.930108	-0.11358	0.489928	-0.09937	0.544346	6861576	9.947806	10.33848	10.53488	10.26713	10.44257	10.4522	10.15432	10.37477	10.59019	10.02237	10.01248	10.03791	Nedd4l	NM_031881
6861627	6960	11.20314	10.89919	10.76412	9.804112	1.399032	0.012411	1.095082	0.036193	0.960005	0.058704	0.135077	0.764488	0.30395	0.505224	0.439027	0.343174	6861627	11.432	11.17648	11.00095	10.54275	11.36586	10.78897	11.0862	10.01815	11.188	9.688369	10.55751	9.166456	Malt1	NM_172833
6861628	6961	8.13584	8.2764	7.886218	7.975034	0.160806	0.221878	0.301366	0.037963	-0.08882	0.485271	0.390183	0.012347	-0.14056	0.280314	0.249622	0.073786	6861628	8.044121	8.15269	8.210709	8.166092	8.494995	8.168114	7.749735	7.834169	8.074749	7.883224	8.124233	7.917646	Zfp532	NM_207255
6861632	6963	10.07654	9.779198	10.11874	10.67403	-0.59749	0.059497	-0.89483	0.011077	-0.55529	0.075724	-0.33954	0.247626	0.297345	0.306568	-0.0422	0.880675	6861632	10.19487	10.05015	9.984611	9.94777	9.39356	9.996265	10.18781	10.45462	9.713787	10.75639	10.21431	11.0514	Sec11c	NM_025468
6861639	6964	10.60068	9.34529	10.9852	12.74706	-2.14638	0.024575	-3.40177	0.002354	-1.76186	0.053081	-1.63991	0.067795	1.25539	0.144795	-0.38452	0.63396	6861639	10.13663	10.94754	10.71787	9.79312	7.68244	10.56031	10.73457	12.07304	10.148	12.9654	12.17225	13.10353	Grp	NM_175012
6861662	6965	7.597797	7.448888	7.565326	6.719324	0.878473	0.005014	0.729564	0.012974	0.846002	0.00614	-0.11644	0.62535	0.148909	0.534336	0.032471	0.890908	6861662	7.806392	7.384329	7.602669	7.218176	7.757466	7.371071	7.958737	7.273449	7.463792	6.896164	6.843426	6.418381	Pmaip1	NM_021451
6861689	6967	8.424625	8.521824	8.424431	8.243863	0.180762	0.308102	0.277961	0.132724	0.180568	0.308588	0.097393	0.573751	-0.0972	0.5745	0.000194	0.999095	6861689	8.356264	8.4671	8.450511	8.472181	8.794149	8.299141	8.744895	8.381485	8.146912	8.148547	8.326339	8.256702	Chmp1b	NM_024190
6861700	6968	6.149257	6.226962	6.051146	6.068362	0.080896	0.421735	0.158601	0.135471	-0.01722	0.861478	0.175816	0.102995	-0.0777	0.439545	0.098111	0.334487	6861700	6.168684	6.187392	6.091696	6.266861	6.134021	6.280005	6.112917	5.959947	6.080575	5.839982	6.189666	6.175437	Impa2	NM_053261
6861707	6970	8.522763	8.133522	8.030094	7.78766	0.735103	0.033995	0.345862	0.26397	0.242434	0.424192	0.103428	0.728699	0.389241	0.213343	0.492669	0.12539	6861707	8.669868	8.492803	8.405618	7.791099	8.664953	7.944514	8.475415	7.529455	8.085411	7.80753	7.965251	7.590199	Tubb6	NM_026473
6861711	6971	6.914206	6.725519	6.214535	6.088314	0.825891	0.006865	0.637204	0.023686	0.126221	0.59605	0.510983	0.0559	0.188687	0.433211	0.69967	0.01559	6861711	7.032975	6.71741	6.992232	6.453858	6.980473	6.742225	6.25305	5.793965	6.596591	6.353109	5.957021	5.954813	Slmo1	NM_144867
6861722	6972	7.425047	7.609051	7.46489	7.194746	0.230302	0.008398	0.414305	0.000246	0.270144	0.003561	0.144161	0.061401	-0.184	0.024099	-0.03984	0.564519	6861722	7.299452	7.441246	7.534444	7.545868	7.670482	7.610803	7.568649	7.405186	7.420834	7.176183	7.194327	7.213727	Psmg2	NM_134138
6861746	6973	9.308921	9.214223	9.35548	9.024163	0.284758	0.19238	0.19006	0.369853	0.331317	0.136247	-0.14126	0.500123	0.094699	0.648563	-0.04656	0.821793	6861746	9.383053	9.207856	9.335855	8.957193	9.347304	9.338171	9.715873	8.889046	9.461522	8.976287	9.083099	9.013103	Seh1l	NM_001039088
6861747	6974	7.344927	7.232898	7.096582	6.589879	0.755048	0.02135	0.64302	0.041171	0.506703	0.091787	0.136317	0.62031	0.112029	0.683125	0.248345	0.375359	6861747	7.40136	7.186869	7.446552	6.928264	7.733415	7.037016	7.288182	6.661105	7.340458	6.69577	6.778906	6.29496	Cep192	BC064462
6861751	6975	8.803196	8.901348	8.636343	8.390862	0.412334	0.038798	0.510486	0.015673	0.245482	0.179855	0.265004	0.151282	-0.09815	0.573	0.166853	0.347089	6861751	8.814758	8.850775	8.744055	8.794278	9.194154	8.715611	8.74512	8.316732	8.847178	8.296309	8.550762	8.325514	D18Ertd6	NM_172631
6861774	6976	6.724462	6.865046	6.771822	6.663656	0.060806	0.506504	0.201389	0.050224	0.108166	0.251161	0.093224	0.317479	-0.14058	0.146556	-0.04736	0.602832	6861774	6.70929	6.766122	6.697974	6.762911	7.063743	6.768483	6.903367	6.715365	6.696734	6.650854	6.711722	6.628393	Rnmt	NM_026440
6861850	6979	9.51375	9.551753	9.513423	9.327931	0.185819	0.236373	0.223823	0.161663	0.185493	0.237126	0.03833	0.798411	-0.038	0.800083	0.000326	0.998261	6861850	9.550159	9.470013	9.521077	9.409033	9.822057	9.42417	9.58348	9.427756	9.529034	9.112674	9.605637	9.265481	Tcf4	NM_013685
6861868	6980	7.060057	7.451105	7.287929	7.493985	-0.43393	0.102965	-0.04288	0.860204	-0.20598	0.407595	0.163176	0.508454	-0.39105	0.135775	-0.22787	0.362089	6861868	6.917742	7.197449	7.064979	7.593143	7.267303	7.492869	7.323609	7.382149	7.158029	7.187068	7.198085	8.096802	Ccdc68	NM_201362
6861892	6982	11.05101	11.19538	11.20461	11.08706	-0.03605	0.794072	0.108327	0.440819	0.117553	0.404474	-0.00923	0.946623	-0.14438	0.311244	-0.1536	0.283365	6861892	11.02766	11.20701	10.91835	11.00273	11.12788	11.45554	11.35107	11.10651	11.15625	11.01484	11.22569	11.02064	Mbd2	NM_010773
6862085	6984	8.644769	8.628951	8.613979	8.565639	0.07913	0.238329	0.063311	0.337779	0.04834	0.458703	0.014971	0.815545	0.015818	0.805354	0.03079	0.633347	6862085	8.685013	8.619093	8.630201	8.592077	8.727931	8.566844	8.650992	8.595366	8.59558	8.433151	8.618047	8.64572	Cxxc1	NM_028868
6862088	6985	8.351938	8.276414	8.32752	8.078286	0.273652	0.006	0.198129	0.02782	0.249234	0.009722	-0.05111	0.50857	0.075523	0.336464	0.024418	0.749447	6862088	8.340146	8.342417	8.37325	8.250328	8.282681	8.296234	8.471663	8.280148	8.230749	7.969388	8.050591	8.214878	Mbd1	NM_013594
6862094	6986	9.18128	9.464716	9.719889	9.927147	-0.74587	0.003899	-0.46243	0.037775	-0.20726	0.2976	-0.25517	0.207415	-0.28344	0.166126	-0.53861	0.02004	6862094	9.209318	8.987448	9.347074	9.468973	9.236473	9.688702	9.740318	10.02451	9.394839	10.08554	9.774839	9.921062	Myo5b	NM_201600
6862102	6987	9.131352	9.236664	9.000895	9.569026	-0.43767	0.057499	-0.33236	0.130847	-0.56813	0.020603	0.235769	0.266687	-0.10531	0.608308	0.130457	0.527393	6862102	8.970996	9.3067	9.11636	9.312013	9.182505	9.215473	8.724287	9.187022	9.091375	9.303667	9.404046	9.999364	Acaa2	NM_177470
6862126	6988	8.3624	8.435847	8.37136	8.412248	-0.04985	0.748236	0.023599	0.87891	-0.04089	0.792117	0.064487	0.678666	-0.07345	0.637594	-0.00896	0.953841	6862126	8.421752	8.291118	8.374329	8.38808	8.367984	8.551476	8.378459	8.646482	8.089139	8.269652	8.317393	8.649698	BC031181	BC016084
6862130	6989	8.946454	8.930582	8.831303	9.203143	-0.25669	0.122265	-0.27256	0.103874	-0.37184	0.036765	0.099279	0.522748	0.015872	0.917542	0.115151	0.460545	6862130	8.842305	8.932298	9.064759	9.10224	8.813862	8.875643	8.607385	9.094818	8.791705	9.203619	9.01249	9.39332	Dym	NM_027727
6862133	6990	7.483146	7.470231	7.349106	7.226991	0.256156	0.22848	0.24324	0.250704	0.122115	0.551454	0.121125	0.554614	0.012916	0.949188	0.13404	0.514263	6862133	7.408654	7.613981	7.426804	7.498496	7.430263	7.481933	7.625339	7.531001	6.890978	6.950706	7.385921	7.344345	Smad7	NM_001042660
6862188	6991	6.543238	6.517186	6.569445	6.259616	0.283623	0.039979	0.25757	0.056797	0.309829	0.028116	-0.05226	0.663754	0.026053	0.82763	-0.02621	0.826631	6862188	6.575793	6.564029	6.489893	6.588918	6.637492	6.325147	6.674849	6.416681	6.616805	6.143889	6.464604	6.170354	Zbtb7c	NM_145356
6862195	6992	7.777758	7.82028	7.766556	7.626542	0.151216	0.205256	0.193738	0.11528	0.140014	0.23751	0.053724	0.637364	-0.04252	0.708324	0.011203	0.92115	6862195	7.740714	7.799456	7.793105	7.753236	7.937233	7.770371	7.765234	7.792366	7.742067	7.514049	7.907876	7.457701	Smad2	NM_010754
6862247	6993	9.689822	9.590124	9.637251	9.861078	-0.17126	0.23266	-0.27095	0.075319	-0.22383	0.12995	-0.04713	0.73152	0.099698	0.473744	0.052571	0.702178	6862247	9.753834	9.670835	9.644796	9.476992	9.6236	9.66978	9.62636	9.848845	9.436547	9.751713	9.714261	10.11726	Ier3ip1	NM_025409
6862251	6994	8.433281	8.563525	8.541021	8.882994	-0.44971	0.000645	-0.31947	0.004971	-0.34197	0.003405	0.022504	0.793807	-0.13024	0.156416	-0.10774	0.231809	6862251	8.420817	8.480735	8.39829	8.588397	8.540907	8.56127	8.447223	8.69656	8.47928	8.742095	8.876447	9.03044	Hdhd2	NM_001039201
6862258	6995	8.930014	9.086545	8.957036	9.090061	-0.16005	0.068395	-0.00352	0.964248	-0.13302	0.118293	0.129509	0.126894	-0.15653	0.073493	-0.02702	0.731466	6862258	8.933579	8.883006	8.973456	9.051311	9.13109	9.077233	8.889703	9.112989	8.868415	9.009878	9.041863	9.218441	Pias2	NM_008602
6862305	6997	7.530314	7.68996	7.481926	7.77206	-0.24175	0.190484	-0.0821	0.640153	-0.29013	0.124371	0.208033	0.253319	-0.15965	0.372514	0.048388	0.781921	6862305	7.647002	7.512857	7.431083	7.648906	7.556833	7.86414	7.808832	7.409872	7.227075	7.879976	7.524741	7.911462	4930465K	BC061021
6862316	6998	10.63313	10.86605	10.74588	11.05226	-0.41913	0.02361	-0.1862	0.25051	-0.30638	0.075861	0.120173	0.447061	-0.23293	0.159802	-0.11275	0.474629	6862316	10.53409	10.61574	10.74955	10.90669	10.68958	11.00189	10.83338	10.76919	10.63507	10.79411	10.98673	11.37593	Atp5a1	NM_007505
6862318	6999	6.30689	6.269757	6.324165	5.976687	0.330203	0.021403	0.29307	0.035171	0.347478	0.017032	-0.05441	0.650914	0.037133	0.756612	-0.01728	0.885072	6862318	6.389599	6.202271	6.3288	6.229149	6.298801	6.28132	6.27643	6.557689	6.138376	6.051387	6.081169	5.797505	Pstpip2	NM_013831
6862459	7001	7.917896	8.02952	8.024489	7.976446	-0.05855	0.531121	0.053074	0.569343	0.048044	0.605809	0.005031	0.95653	-0.11162	0.247357	-0.10659	0.267548	6862459	7.858537	7.899231	7.995921	7.902737	8.20147	7.984353	8.144319	8.032739	7.89641	7.933731	8.046825	7.948781	Txnl4a	NM_025299
6862462	7002	7.505633	7.564609	7.603075	7.180955	0.324678	0.12373	0.383654	0.076556	0.42212	0.055745	-0.03847	0.843609	-0.05898	0.762693	-0.09744	0.619657	6862462	7.47647	7.402005	7.638424	7.346432	7.756124	7.59127	7.963609	7.302519	7.543096	7.010375	7.417531	7.114959	Pqlc1	NM_025861
6862586	7003	6.390084	6.296546	6.305824	6.367963	0.022121	0.768268	-0.07142	0.353859	-0.06214	0.416735	-0.00928	0.901422	0.093538	0.233417	0.08426	0.279052	6862586	6.356602	6.471836	6.341815	6.275446	6.235313	6.37888	6.266376	6.432729	6.218367	6.337213	6.29572	6.470957	Mbp	NM_010777

6862627	7004	7.779094	7.757557	7.99034	7.738837	0.040257	0.797357	0.01872	0.904797	0.251503	0.135795	-0.23278	0.163311	0.021537	0.890572	-0.21125	0.20109	6862627	7.952073	7.594775	7.790434	7.769201	8.00543	7.49804	7.981086	8.155697	7.834238	7.597638	7.784022	7.834851	Zfp516	NM_183033
6862693	7005	8.00559	8.062292	8.024384	7.993278	0.012312	0.917732	0.069014	0.566688	0.031107	0.794502	0.037908	0.751187	-0.0567	0.636668	-0.01879	0.874775	6862693	8.072135	7.91416	8.030475	8.161206	8.17469	7.85098	8.00224	8.109236	7.961677	7.807132	7.996867	8.175834	Zadh2	NM_146090
6862751	7007	9.204438	9.251082	9.236548	9.087214	0.117223	0.548978	0.163867	0.407287	0.149334	0.448446	0.014534	0.940076	-0.04664	0.809674	-0.03211	0.868182	6862751	9.303617	9.158153	9.151543	9.317239	9.244845	9.191161	9.448148	9.476588	8.784908	8.864443	9.104599	9.292601	Cyb5	NM_025797
6862973	7013	8.524661	8.622369	8.608746	8.556662	-0.032	0.850413	0.065707	0.699655	0.052084	0.759336	0.013624	0.935947	-0.09771	0.568448	-0.08408	0.622602	6862973	8.588222	8.687035	8.285934	8.319678	8.742931	8.804499	8.856371	8.53169	8.438176	8.492494	8.571843	8.605649	Tmx3	NM_198295
6863002	7014	7.238752	7.287933	7.039462	7.309599	-0.07085	0.666648	-0.02167	0.894616	-0.27014	0.126623	0.248471	0.155496	-0.04918	0.764205	0.19929	0.243964	6863002	7.053877	7.271995	7.390384	6.949864	7.454788	7.459146	7.146564	6.946153	7.025669	7.349893	7.445605	7.1333	Crem	NM_001110859
6863048	7015	6.949974	6.625113	6.716866	6.267541	0.682433	0.039105	0.357572	0.232827	0.449325	0.14346	-0.09175	0.748991	0.324861	0.274647	0.233108	0.424511	6863048	7.346426	6.835937	6.66756	6.671217	6.662223	6.5419	6.969482	6.352886	6.82823	6.359341	6.690238	5.753044	Map3k8	NM_007746
6863096	7016	5.905962	5.970159	5.928362	5.964764	-0.0588	0.540348	0.005394	0.954654	-0.0364	0.702515	0.041797	0.661487	-0.0642	0.50483	-0.0224	0.813654	6863096	5.766618	6.030599	5.92067	6.019073	5.936378	5.955025	5.84934	6.082598	5.853148	5.92803	5.871777	6.094486	Zfp438	NM_178722
6863156	7017	8.253842	8.327869	8.279554	8.220351	0.033491	0.699987	0.107518	0.235575	0.059203	0.500117	0.048315	0.580247	-0.07403	0.402979	-0.02571	0.766902	6863156	8.095802	8.249987	8.415736	8.213002	8.408788	8.361817	8.31742	8.251893	8.269348	8.211128	8.295003	8.154922	Arhgap12	NM_001039692
6863158	7018	9.984796	9.967072	9.980774	9.921342	0.063454	0.363355	0.04573	0.506964	0.059432	0.393029	-0.0137	0.840327	0.017724	0.794575	0.004022	0.952788	6863158	10.03946	9.946654	9.968273	9.971858	10.03126	9.898097	9.989273	10.05374	9.899309	9.790768	9.966787	10.00647	Kif5b	NM_008448
6863167	7019	8.005813	8.08947	7.986296	8.071794	-0.06598	0.220588	0.017676	0.731083	-0.0855	0.123418	0.103174	0.07137	-0.08366	0.130544	0.019517	0.704569	6863167	7.976076	8.017591	8.023771	8.081963	8.074963	8.111484	8.034199	7.956114	7.968575	7.983683	8.037336	8.194364	Epc1	NM_007935
6863281	7022	9.287776	9.452716	9.343442	9.366667	-0.07889	0.266309	0.086049	0.228682	-0.02322	0.734076	0.109274	0.13647	-0.16494	0.037032	-0.05567	0.423608	6863281	9.26428	9.283631	9.315418	9.48123	9.418053	9.458865	9.483397	9.35507	9.191859	9.302315	9.397959	9.399726	Ccny	NM_026484
6863299	7024	8.13044	8.083414	8.160688	7.946413	0.184026	0.113429	0.137001	0.222373	0.214274	0.072302	-0.07727	0.47686	0.047026	0.661789	-0.03025	0.777628	6863299	8.160501	8.090445	8.140373	7.963313	8.249487	8.037442	8.314143	8.082952	8.084968	7.883455	8.120498	7.835287	Usp14	NM_021522
6863301	7025	9.571451	9.611128	9.548892	9.429915	0.141536	0.13634	0.181213	0.066818	0.118977	0.201413	0.062236	0.487303	-0.03968	0.654877	0.022559	0.798512	6863301	9.611318	9.60412	9.498914	9.527584	9.661717	9.644083	9.734339	9.5142	9.398137	9.34875	9.492015	9.448979	Rock1	NM_009071
6863323	7026	7.239321	7.379799	7.08768	7.326556	-0.08724	0.648828	0.053243	0.780144	-0.23888	0.231341	0.29212	0.15185	-0.14048	0.468074	0.151641	0.434744	6863323	7.068875	7.42105	7.228038	7.105179	7.347081	7.687138	6.801673	7.248133	7.213233	7.389352	7.143921	7.446396	Esco1	NM_001081222
6863326	7027	6.412228	6.46162	6.376052	6.591684	-0.17946	0.146463	-0.13006	0.277367	-0.21563	0.089394	0.085568	0.465219	-0.04939	0.669762	0.036176	0.754111	6863326	6.611715	6.386908	6.23806	6.354635	6.495659	6.534566	6.341998	6.339595	6.446562	6.684934	6.403235	6.686884	Abhd3	NM_134130
6863417	7028	6.191696	6.364098	6.354664	6.44331	-0.25161	0.01209	-0.07921	0.339242	-0.08865	0.288322	0.009434	0.90665	-0.1724	0.05791	-0.16297	0.069924	6863417	6.320775	6.179444	6.07487	6.38669	6.381905	6.323699	6.347433	6.312641	6.403919	6.386096	6.596069	6.347765	6030446N	BC094382
6863434	7029	8.581738	8.542001	8.542204	8.624378	-0.04264	0.686846	-0.08238	0.442561	-0.08217	0.443644	-0.0002	0.99846	0.039737	0.706955	0.039534	0.70837	6863434	8.638591	8.555048	8.551575	8.611532	8.670206	8.344266	8.632829	8.571374	8.42241	8.7583	8.489408	8.625427	Npc1	NM_008720
6863467	7030	6.99595	7.086319	6.887015	6.837289	0.158661	0.151542	0.24903	0.037619	0.049726	0.632647	0.199304	0.081586	-0.09037	0.392915	0.108936	0.308074	6863467	6.938982	7.006825	7.042044	7.130909	6.982166	7.145881	7.023605	6.774244	6.863195	6.634142	6.89158	6.986145	Osbpl1a	NM_207530
6863540	7031	8.466822	8.719302	8.397748	8.413322	0.0535	0.660281	0.30598	0.031134	-0.01557	0.897595	0.321555	0.025341	-0.25248	0.063421	0.069075	0.571995	6863540	8.320459	8.710741	8.369267	8.707525	8.849899	8.600483	8.494859	8.257416	8.440968	8.33659	8.410672	8.492705	Zfp521	NM_145492
6863571	7032	9.357485	9.506255	9.401973	9.188199	0.169286	0.230999	0.318056	0.040845	0.213774	0.140261	0.104282	0.4476	-0.14877	0.287563	-0.04449	0.742119	6863571	9.336397	9.338413	9.397645	9.318527	9.660379	9.53986	9.546411	9.504802	9.154707	9.011127	9.311956	9.241514	Ss18	NM_009280
6863582	7033	9.687995	9.595759	9.584229	9.997411	-0.30942	0.106088	-0.40165	0.045683	-0.41318	0.041093	0.01153	0.947563	0.092236	0.602035	0.103766	0.558349	6863582	9.460418	9.940529	9.663037	9.527806	9.562143	9.697328	9.306244	9.770628	9.675814	10.15595	10.08561	9.750673	Kctd1	NM_001142731
6863645	7036	6.203667	6.342594	6.135099	6.084979	0.118689	0.544229	0.257615	0.206547	0.05012	0.795911	0.207495	0.300421	-0.13893	0.479729	0.068569	0.723959	6863645	6.358898	6.177107	6.074997	6.140755	6.659959	6.227068	6.229284	6.060591	6.115421	5.879343	6.459012	5.916581	Cdh2	NM_007664
6863758	7038	8.033847	7.981017	7.944731	7.252374	0.781474	0.279445	0.728644	0.31091	0.692358	0.334099	0.036286	0.958361	0.05283	0.939414	0.089116	0.898017	6863758	7.715917	8.19394	8.191685	7.501055	8.237836	8.204161	8.109247	7.268441	8.456506	7.158109	8.746498	5.852514	Dsc2	NM_013505
6863783	7040	9.536905	9.800351	9.791449	9.517347	0.019558	0.87076	0.283004	0.041149	0.274102	0.046365	0.008902	0.940927	-0.26345	0.053485	-0.25454	0.060261	6863783	9.677344	9.455685	9.477685	9.760776	9.923986	9.716291	9.705574	9.860141	9.808631	9.696037	9.583925	9.272078	B4galt6	NM_019737
6863929	7043	8.246518	8.372768	8.409672	8.269637	-0.02312	0.873929	0.103131	0.485729	0.140035	0.350087	-0.0369	0.800301	-0.12625	0.397083	-0.16315	0.280964	6863929	8.147579	8.236341	8.355334	8.246664	8.615242	8.256398	8.63486	8.387229	8.206926	8.134992	8.268373	8.405545	Zfp191	NM_021559
6863934	7044	8.361666	8.24851	8.340941	8.070335	0.291331	0.137014	0.178175	0.341743	0.270606	0.163323	-0.09243	0.614267	0.113156	0.538898	0.020725	0.909309	6863934	8.350062	8.342604	8.392333	8.239271	8.360955	8.145304	8.737495	7.967165	8.318163	7.890657	8.193304	8.127044	Ino80c	NM_172625
6863965	7045	8.318089	8.365344	8.301515	8.177665	0.140424	0.175938	0.187679	0.082506	0.12385	0.226752	0.06383	0.518801	-0.04725	0.630803	0.016575	0.865249	6863965	8.3977	8.360311	8.196257	8.246511	8.55738	8.292142	8.234312	8.339289	8.330943	8.122995	8.296494	8.113506	Rprd1a	NM_144861
6863973	7046	7.541293	7.564912	7.456884	7.278775	0.262518	0.265358	0.286138	0.228069	0.17811	0.440002	0.108028	0.635386	-0.02362	0.916847	0.084408	0.710227	6863973	7.618756	7.558866	7.446256	7.406756	7.864257	7.423724	7.595859	7.427259	7.347535	7.128923	7.778491	6.92891	Slc39a6	NM_139143
6864037	7047	6.513521	6.540795	6.563378	6.407946	0.105575	0.56254	0.132849	0.468997	0.155431	0.399784	-0.02258	0.900382	-0.02727	0.879859	-0.04986	0.782691	6864037	6.502093	6.591969	6.446501	6.434332	6.732612	6.455441	6.482387	6.916894	6.290852	6.296738	6.658508	6.268593	5730494M	NM_001004361
6864062	7048	6.223916	6.424504	6.231728	6.55653	-0.33261	0.004923	-0.13203	0.165625	-0.3248	0.005599	0.192776	0.056511	-0.20059	0.049084	-0.00781	0.930286	6864062	6.180965	6.227698	6.263084	6.279174	6.434782	6.559557	6.167496	6.205907	6.321782	6.504297	6.460134	6.705158	Brunol4	NM_001146292
6864330	7051	8.600484	8.670649	8.549336	8.749895	-0.14941	0.173318	-0.07925	0.450744	-0.20056	0.079702	0.121314	0.259467	-0.07017	0.502591	0.051148	0.622665	6864330	8.66613	8.625051	8.51027	8.654351	8.677759	8.679838	8.46162	8.669377	8.51701	8.540149	8.76178	8.947755	Slc25a46	NM_026165
6864404	7055	6.589072	6.645697	6.328663	6.273615	0.315457	0.061556	0.372082	0.033504	0.055048	0.714443	0.317033	0.060523	-0.05662	0.706712	0.260409	0.110632	6864404	6.65303	6.472244	6.641943	6.684594	6.855323	6.397173	6.542907	6.154971	6.288112	6.232582	6.444072	6.144191	Gypc	NM_001048207
6864444	7056	6.623665	6.811267	6.507095	6.389185	0.234479	0.181935	0.422081	0.030115	0.117909	0.483283	0.304172	0.094507	-0.1876	0.275842	0.11657	0.48811	6864444	6.657508	6.531722	6.681764	6.558995	7.010841	6.863964	6.718788	6.278013	6.524483	6.239711	6.634837	6.293008	Stard4	NM_133774
6864456	7057	6.556444	6.858662	6.350964	6.03767	0.518774	0.101456	0.820992	0.019058	0.313294	0.296288	0.507698	0.107787	-0.30222	0.31254	0.20548	0.484587	6864456	6.63723	6.597324	6.434779	6.51324	7.473781	6.588965	6.548087	6.312376	6.192429	5.819903	5.819871	6.473236	D0H4S114	NM_053078
6864466	7058	7.180684	7.601614	7.032486	7.364643	-0.18396	0.488294	0.236971	0.376789	-0.33216	0.226041	0.569128	0.054794	-0.42093	0.135057	0.148198	0.574546	6864466	6.892933	6.895523	7.753597	7.706776	7.465951	7.632116	6.935417	7.098582	7.06346	7.754302	7.216456	7.123172	Epb4.1l4	NM_013512
6864497	7059	9.900668	9.905096	9.985071	10.00224	-0.10157	0.698424	-0.09714	0.710852	-0.01716	0.947544	-0.07998	0.75987	-0.00443	0.986457	-0.0844	0.747102	6864497	9.998158	9.755172	9.948673	9.939157	10.03523	9.7409	9.978967	10.55051	9.425737	10.01864	9.826507	10.16156	Reep5	NM_007874
6864509	7060	7.716115	7.701209	7.641355	7.528382	0.187732	0.092464	0.172827	0.116663	0.112973	0.283484	0.059854	0.559351	0.014906	0.883188	0.074759	0.468609	6864509	7.751623	7.763159	7.633562	7.504635	7.896622	7.70237	7.645555	7.697574	7.580937	7.55561	7.616782	7.412755	Pkd2l2	NM_016927
6864520	7062	7.981025	8.221292	8.063798	7.995713	-0.01469	0.845026	0.225579	0.014646	0.068085	0.376703	0.157494	0.062305	-0.24027	0.010814	-0.08277	0.288119	6864520	7.938402	7.978173	8.026499	8.118064	8.280476	8.265335	8.207205	7.945184	8.039004	7.966188	7.951501	8.06945	Brd8	NM_030147
6864522	7063	7.886929	7.989063	7.978263	7.90569	-0.01876	0.794548	0.083373	0.265745	0.072573	0.328077	0.0108	0.880667	-0.10213	0.180876	-0.09133	0.226304	6864522	7.860643	7.901701	7.898442	7.886857	8.148543	7.931788	8.016556	7.942232	7.976001	7.809102	7.928528	7.97944	Cdc23	NM_178347
6864564	7066	9.13862	9.196839	9.161158	9.043492	0.095128	0.354148	0.153348	0.151518	0.117667	0.25844	0.035681	0.721767	-0.05822	0.56388	-0.02254	0.82159	6864564	9.161344	9.103205	9.15131	9.016577	9.38361	9.190331	9.273726	9.079957	9.129792	8.920629	9.107538	9.102308	Etf1	NM_144866
6864565	7067	10.54237	10.72277	10.65102	10.84866	-0.30629	0.030106	-0.12589	0.310957	-0.19764	0.127939	0.071747	0.554751	-0.1804	0.15975	-0.10866	0.377862	6864565	10.59809	10.48437	10.54464	10.73464	10.67765	10.75602	10.78344	10.65522	10.51441	10.64598	10.7842	11.1158	Hspa9	NM_010481
6864583	7068	6.184382	6.015729	5.999561	5.824864	0.359518	0.219337	0.190865	0.499334	0.174697	0.535391	0.016168	0.953679	0.168653	0.549265	0.184821	0.512629	6864583	5.701002	6.351763	6.50038	5.993684	6.342317	5.711185	6.431045	5.680006	5.887631	5.878515	5.856033	5.740044	Lrrtm2	NM_178005
6864585	7069	9.261494	9.213709	9.250199	9.49402	-0.23253	0.138815	-0.28031	0.082824	-0.24382	0.123039	-0.03649	0.802938	0.047785	0.744184	0.011295	0.938313	6864585	9.370039	9.173766	9.240678	9.028528	9.167719	9.444881	9.470483	9.084633	9.195481	9.491844	9.334818	9.655399	Sil1	NM_030749
6864593	7070	10.24592	10.04966	9.97291	10.27537	-0.02945	0.876185	-0.22571	0.252606	-0.30246	0.137111	0.076746	0.686088	0.19626	0.314972	0.273006	0.174232	6864593	10.0146	10.10281	10.62034	9.92899	10.11406	10.10592	10.05626	9.869782	9.992689	10.35597	9.972001	10.49813	Slc23a1	NM_011397
6864597	7071	6.447367	6.381245	6.28101	6.523606	-0.07624	0.602901	-0.14236	0.341534	-0.2426	0.123159	0.100234	0.496727	0.066122	0.651133	0.166357	0.27129	6864597	6.333892	6.390615	6.617594	6.61147	6.267457	6.264807	6.390761	6.338892	6.113378	6.402113	6.430026	6.738679	2010001M	NM_027222
6864604	7072	7.28456	7.47132	7.415129	7.65709	-0.37253	0.027811	-0.18577	0.217753	-0.24196	0.119607	0.05619	0.696347	-0.18676	0.21554	-0.13057	0.374605	6864604	7.222515	7.261491	7.369673	7.514819	7.489635	7.409505	7.423057	7.632782	7.189549	7.475391	7.566436	7.929442	5133400G	NM_027733
6864607	7073	8.176651	8.171106	8.140826	8.218735	-0.04208	0.330643	-0.04763	0.27487	-0.07791	0.091494	0.03028	0.477476	0.005544	0.894841	0.035825	0.403685	6864607	8.13541	8.238334	8.156208	8.132444	8.17523	8.205645	8.171942	8.157265	8.093271	8.156589	8.219046	8.280569	Dnajc18	NM_029669
6864609	7074	9.810187	9.335121	9.237665	8.416497	1.393689	0.009807	0.918624	0.057155	0.821168	0.08244	0.097456	0.819699	0.475065	0.284047	0.572522	0.203807	6864609	10.15823	9.849557	9.422773	9.099278	9.907879	8.998207	9.704397	8.486116	9.522482	8.472261	8.84454	7.932691	Tmem173	BC046640
6864678	7075	7.788079	7.86589	7.781351	7.75794	0.030139	0.821023	0.10795	0.426699	0.023411	0.860416	0.084539	0.530383	-0.07781	0.562849	0.006728	0.959658	6864678	7.786774	7.783763	7.7937	7.804329	8.008553	7.784787	7.958611	7.74171	7.643732	7.639196	7.598699	8.035925	Pfdn1	NM_026027
6864680	7076	7.709849	7.687154	7.426813	7.373164	0.336685	0.06119	0.31399	0.076876	0.053649	0.737669	0.260341	0.130871	0.022695	0.886985	0.283036	0.10468	6864680	7.579759	7.693014	7.856774	7.483606	7.892397	7.685458	7.302224	7.425696	7.552519	7.089952	7.433701	7.595838	Hbegf	NM_010415
6864695	7077	8.17884	8.359017	8.233072	8.276484	-0.09764	0.180067	0.082533	0.249573	-0.04341	0.532043	0.125945	0.094756	-0.18018	0.026643	-0.05423	0.438229	6864695	8.108052	8.250487	8.177982	8.214916	8.376996	8.485138	8.224369	8.266754	8.208092	8.22607	8.290665	8.312716	Sra1	NM_025291
6864696	7078	6.822711	6.822497	6.750142	6.82036	0.002351	0.977833	0.002137	0.97985	-0.07022	0.416728	0.072355	0.403303	0.000214	0.997982	0.072569	0.401975	6864696	6.808595	6.86946	6.790078	6.671316	6.960014	6.836161	6.648285	6.830278	6.771863	6.846635	6.899364	6.715082	Apbb3	NM_146085
6864698	7079	6.192275	6.154418	6.20361	6.155215	0.037059	0.56181	-0.0008	0.989934	0.048395	0.452126	-0.04919	0.444988	0.037856	0.553615	-0.01134	0.857745	6864698	6.24135	6.258187	6.077287	6.193218	6.188855	6.081182	6.134733	6.301044	6.175053	6.170924	6.175128	6.119594	E230025N	NM_172831
6864700	7080	7.768911	7.719814	7.239774	6.470424	1.298487	0.005622	1.24939	0.006897	0.76935	0.057002	0.48004	0.203024	0.049097	0.890743	0.529137	0.164974	6864700	8.288755	7.822068	7.195909	7.777865	7.994422	7.387154	7.592663	6.778631	7.348027	6.764373	6.614331	6.032568	Cd14	NM_009841
6864702	7081	8.969143	9.034625	9.1046	9.158629	-0.18949	0.282749	-0.124	0.472676	-0.05403	0.751073	-0.06997	0.681857	-0.06548	0.701071	-0.13546	0.434238	6864702	8.865149	8.897877	9.144402	8.991986	9.069004	9.042885	9.389892	9.170023	8.753884	8.960236	9.319128	9.196524	Ndufa2	NM_010885
6864709	7083	8.31762	8.397545	8.233339	8.24563	0.07199	0.480077	0.151915	0.156693	-0.01229	0.902491	0.164207	0.129615	-0.07993	0.434733	0.084281	0.411136	6864709	8.383259	8.30588	8.263721	8.303008	8.532197	8.357431	8.450542	8.173719	8.075755	8.211352	8.26546	8.260078	Hars	NM_008214
6864760	7086	8.43756	8.356846	8.350198	8.162917	0.274643	0.019242	0.19393	0.073014	0.187281	0.08148	0.006649	0.945349	0.080713	0.415525	0.087362	0.379887	6864760	8.475299	8.344864	8.492516	8.469753	8.41891	8.181876	8.439088	8.378552	8.232953	8.090514	8.287065	8.111171	Diap1	NM_007858
6864761	7087	8.574105	8.661527	8.529105	8.49791	0.076195	0.604357	0.163617	0.280238	0.031195	0.830784	0.132422	0.376046	-0.08742	0.553278	0.045001	0.758254	6864761	8.412115	8.553114	8.757087	8.54387	8.906341	8.53437	8.734388	8.51371	8.339216	8.430949	8.485218	8.577563	Hdac3	NM_010411
6864762	7088	6.46247	6.478031	6.357991	6.540742	-0.07827	0.267399	-0.06271	0.367512	-0.18275	0.023806	0.12004	0.104933	-0.01556	0.818619	0.104479	0.150237	6864762	6.479733	6.456415	6.451262	6.512758	6.393115	6.528221	6.505843	6.331264	6.236867	6.544556	6.499629	6.578042	Fchsd1	NM_175684
6864763	7089	7.678282	7.681503	7.669392	7.592787	0.085495	0.429632	0.088716	0.413169	0.076605	0.477391	0.012111	0.909103	-0.00322	0.975764	0.00889	0.9332	6864763	7.793773	7.681444	7.559629	7.685975	7.751369	7.607166	7.671769	7.700664	7.635744	7.35499	7.743072	7.6803	Arap3	NM_139206
6864776	7090	6.459796	6.410939	6.467196	6.420269	0.039527	0.691306	-0.00933	0.924957	0.046927	0.638049	-0.05626	0.573982	0.048858	0.624497	-0.0074	0.940446	6864776	6.549833	6.362363	6.467193	6.285222	6.500485	6.447109	6.513139	6.347398	6.541051	6.245411	6.506821	6.508575	Pcdh1	NM_029357

6864783	7091	6.874163	7.113595	6.96105	6.905129	-0.03097	0.808489	0.208466	0.130164	0.055921	0.662974	0.152545	0.252164	-0.23943	0.088738	-0.08689	0.502018	6864783	6.974013	6.761883	6.886592	7.176606	7.202649	6.961531	7.01783	7.092497	6.772824	6.698487	7.061316	6.955584	Pcdh12	NM_017378
6864789	7092	7.453905	7.704882	7.410908	7.233603	0.220302	0.224155	0.47128	0.022549	0.177306	0.319945	0.293974	0.11679	-0.25098	0.171767	0.042997	0.803564	6864789	7.40085	7.569919	7.390946	7.669389	7.515037	7.930221	7.332336	7.403757	7.496632	7.078587	7.013124	7.609097	Gnpda1	NM_011937
6864813	7093	7.578951	7.533048	7.210592	7.692114	-0.11316	0.490572	-0.15907	0.339567	-0.48152	0.015249	0.322456	0.073506	0.045903	0.77693	0.368359	0.046551	6864813	7.676444	7.644712	7.415696	7.712069	7.754388	7.132687	7.176599	7.206028	7.249149	7.66806	7.636868	7.771413	Spry4	NM_011898
6864837	7094	8.777105	8.637356	9.177909	8.631053	0.146051	0.455418	0.006302	0.973829	0.546855	0.018799	-0.54055	0.019799	0.139749	0.474433	-0.4008	0.063525	6864837	8.807041	8.664765	8.859508	8.84385	8.646689	8.421528	9.098772	9.400147	9.034807	8.239961	8.847551	8.805648	Fgf1	NM_010197
6864895	7095	8.487692	8.631805	8.513478	8.295596	0.192096	0.168677	0.336209	0.029325	0.217882	0.124429	0.118327	0.378548	-0.14411	0.289137	-0.02579	0.844104	6864895	8.376948	8.564219	8.521909	8.555994	8.683966	8.655455	8.518652	8.556243	8.46554	7.981986	8.534187	8.370615	Nr3c1	NM_008173
6864926	7096	9.376442	9.363039	9.551804	9.50387	-0.12743	0.253925	-0.14083	0.211377	0.047935	0.656119	-0.18876	0.106112	0.013403	0.900324	-0.17536	0.129188	6864926	9.290574	9.460787	9.377965	9.171433	9.549669	9.368016	9.610652	9.654287	9.390474	9.489091	9.547212	9.475306	Yipf5	NM_023311
6865022	7097	7.479305	7.599255	7.383029	7.302116	0.177189	0.219424	0.297139	0.05591	0.080913	0.559766	0.216226	0.142608	-0.11995	0.393403	0.096276	0.489707	6865022	7.54623	7.479186	7.412498	7.521192	7.664936	7.611636	7.701632	7.136637	7.310817	7.179843	7.325498	7.401007	Lars	NM_134137
6865100	7100	7.738079	7.800224	7.457222	6.815868	0.922211	0.002067	0.984356	0.001395	0.641354	0.014383	0.343003	0.134518	-0.06214	0.770628	0.280858	0.209953	6865100	8.200912	7.658415	7.354911	7.946586	7.741342	7.712745	7.475639	7.679967	7.216059	6.816785	6.789386	6.841433	Dpysl3	NM_009468
6865221	7103	5.960757	6.368568	6.184746	7.407972	-1.44722	0.008184	-1.0394	0.036519	-1.22323	0.018403	0.183821	0.669197	-0.40781	0.35404	-0.22399	0.603678	6865221	6.051966	5.963617	5.866687	6.68744	5.914082	6.504181	5.97433	6.778111	5.801798	7.662782	6.546438	8.014696	Trim36	NM_178872
6865222	7104	8.627416	8.837437	8.720122	8.623115	0.0043	0.963435	0.214322	0.046155	0.097007	0.317132	0.117315	0.232985	-0.21002	0.049692	-0.09271	0.337737	6865222	8.706887	8.66937	8.50599	8.814768	8.950107	8.747436	8.703272	8.704026	8.753068	8.434797	8.728325	8.706224	Pggt1b	NM_172627
6865229	7105	6.247128	6.308973	6.120238	5.837714	0.409414	0.031897	0.47126	0.017434	0.282525	0.111192	0.188735	0.265953	-0.06185	0.705367	0.12689	0.444593	6865229	6.527287	5.99928	6.214817	6.332068	6.464963	6.129889	6.056566	6.189483	6.114666	5.861338	5.612322	6.039481	Ticam2	NM_173394
6865231	7106	9.358906	9.245987	9.431534	9.242921	0.115985	0.391078	0.003067	0.981462	0.188613	0.178606	-0.18555	0.184991	0.112919	0.403149	-0.07263	0.585792	6865231	9.402782	9.206485	9.467451	9.083924	9.419924	9.234114	9.638778	9.454169	9.201655	9.235884	9.303014	9.189864	Fem1c	NM_173423
6865238	7107	6.342278	6.655238	6.069386	6.261626	0.080651	0.846336	0.393612	0.357204	-0.19224	0.646021	0.585852	0.183997	-0.31296	0.459639	0.272891	0.517304	6865238	5.497491	6.275301	7.254041	6.486129	7.123071	6.356514	6.210752	5.919619	6.077788	6.152439	6.342377	6.290063	Cdo1	NM_033037
6865240	7108	7.466093	7.719156	7.670428	7.447959	0.018134	0.865448	0.271197	0.030803	0.222468	0.064113	0.048728	0.650775	-0.25306	0.040449	-0.20433	0.08413	6865240	7.456278	7.406082	7.535919	7.533542	7.885846	7.73808	7.68524	7.72641	7.599633	7.287132	7.455729	7.601017	Atg12	NM_026217
6865267	7109	8.060855	8.350776	7.820645	7.916685	0.14417	0.522775	0.434091	0.079018	-0.09604	0.667999	0.530131	0.039486	-0.28992	0.215861	0.240209	0.297857	6865267	8.279015	7.955911	7.947638	8.230165	8.744733	8.07743	7.775695	7.920178	7.766063	7.703087	8.305691	7.741276	Sema6a	NM_018744
6865551	7111	9.384598	9.915423	9.374721	9.700326	-0.31573	0.183776	0.215097	0.350618	-0.32561	0.171893	0.540703	0.037427	-0.53083	0.040179	0.009877	0.964813	6865551	9.225833	9.492824	9.435136	9.855758	10.0677	9.822812	9.814486	9.341945	8.967731	9.927743	9.748824	9.424412	Ppic	NM_008908
6865573	7112	7.982748	8.152601	8.041413	7.889468	0.09328	0.351787	0.263133	0.023599	0.151945	0.145969	0.111188	0.272478	-0.16985	0.109515	-0.05867	0.551393	6865573	7.810199	7.944179	8.193867	8.077368	8.252543	8.127892	8.062415	8.076534	7.985291	7.945646	7.912249	7.810509	Cep120	NM_178686
6865643	7113	7.934306	8.230367	7.749378	7.214118	0.720188	0.010501	1.01625	0.001565	0.53526	0.03875	0.48099	0.057274	-0.29606	0.209151	0.184928	0.418391	6865643	7.94711	7.929439	7.926369	7.98614	8.672435	8.032527	7.785147	7.466001	7.996985	6.924468	7.376709	7.341176	Zfp608	NM_175751
6865709	7114	8.38502	8.390557	8.486633	8.475495	-0.09048	0.334829	-0.08494	0.36357	0.011138	0.902586	-0.09608	0.307572	-0.00554	0.951462	-0.10161	0.282381	6865709	8.292418	8.316358	8.546284	8.360694	8.389085	8.421893	8.617154	8.418885	8.423861	8.455538	8.371577	8.59937	Aldh7a1	NM_001127338
6865723	7115	7.291756	7.331555	7.115841	7.389471	-0.09771	0.556884	-0.05792	0.725765	-0.27363	0.124379	0.215713	0.212958	-0.0398	0.809125	0.175914	0.301849	6865723	7.304973	7.214395	7.355899	7.270123	7.239182	7.485359	7.34393	6.971594	7.032	7.703569	7.356544	7.108299	3-Mar	NM_177115
6865902	7117	8.92693	8.909428	8.992077	8.850292	0.076637	0.665787	0.059135	0.73829	0.141785	0.430873	-0.08265	0.641672	0.017502	0.920964	-0.06515	0.713021	6865902	8.857002	9.077975	8.845812	8.744335	9.184037	8.799911	9.160336	8.948009	8.867887	8.652611	8.728501	9.169765	2010002N	NM_134133
6865926	7118	9.754711	9.989883	9.874312	9.775783	-0.02107	0.883399	0.2141	0.162514	0.098528	0.499073	0.115572	0.430382	-0.23517	0.129536	-0.1196	0.415141	6865926	9.899824	9.883208	9.481101	10.06634	10.066	9.83731	9.849806	10.00925	9.763879	9.61527	9.76822	9.94386	Ndst1	NM_008306
6865949	7119	7.558969	7.54229	7.535154	7.406113	0.152855	0.096967	0.136177	0.132564	0.129041	0.15123	0.007136	0.932231	0.016678	0.842627	0.023815	0.777098	6865949	7.521016	7.577971	7.577919	7.442558	7.710869	7.473444	7.610704	7.439273	7.555485	7.305791	7.499204	7.413345	Tcof1	NM_011552
6865958	7121	6.207981	6.276037	6.180396	6.267156	-0.05917	0.535493	0.008881	0.924985	-0.08676	0.370296	0.09564	0.325964	-0.06806	0.477822	0.027585	0.770498	6865958	6.252561	6.197324	6.174059	6.293653	6.183396	6.351061	6.331436	6.256176	5.953577	6.230724	6.269853	6.30089	Cdx1	NM_009880
6865967	7122	7.793281	7.748865	7.701566	7.778562	0.014719	0.892433	-0.0297	0.785365	-0.077	0.48609	0.047299	0.665642	0.044416	0.684673	0.091715	0.409745	6865967	7.814673	7.810077	7.755094	7.705709	7.926306	7.61458	7.720551	7.883478	7.50067	7.807457	7.812987	7.715243	Hmgxb3	NM_178277
6865971	7123	7.17762	7.300059	7.330744	7.464815	-0.28719	0.45359	-0.16476	0.663363	-0.13407	0.722628	-0.03069	0.934998	-0.12244	0.745655	-0.15312	0.685563	6865971	7.337945	7.358272	6.836644	7.566392	7.346747	6.987037	6.837468	8.206016	6.948748	7.296681	7.709892	7.387872	Slc26a2	NM_007885
6865980	7124	6.325159	6.316492	6.297529	6.599213	-0.27405	0.168677	-0.28272	0.157126	-0.30168	0.134317	0.018963	0.919186	0.008667	0.963004	0.02763	0.88252	6865980	6.31529	6.30486	6.355327	6.572578	6.154727	6.222171	6.098439	6.572691	6.221457	6.364572	6.507009	6.926057	Ppargc1b	NM_133249
6865983	7125	6.302604	6.205959	6.262907	7.06575	-0.76315	0.016836	-0.85979	0.009497	-0.80284	0.013281	-0.05695	0.827957	0.096645	0.713066	0.039697	0.879496	6865983	6.267663	6.199224	6.440924	6.642078	5.898258	6.07754	6.213332	6.539717	6.035672	7.512842	6.775702	6.908705	4933429F	NM_177828
6866002	7126	6.689303	6.647524	6.668889	6.611556	0.077747	0.463421	0.035968	0.730882	0.057333	0.585728	-0.02137	0.837707	0.041779	0.689903	0.020414	0.844823	6866002	6.698716	6.6467	6.722493	6.615798	6.656721	6.670053	6.881544	6.649623	6.475501	6.482765	6.746158	6.605746	Pcyox1l	NM_172832
6866003	7127	6.399559	6.456158	6.493566	6.24311	0.156449	0.280241	0.213048	0.153443	0.250456	0.100869	-0.03741	0.788877	-0.0566	0.686271	-0.09401	0.506228	6866003	6.341835	6.295499	6.561343	6.485091	6.615895	6.267488	6.708108	6.323403	6.449187	6.135703	6.402805	6.190823	Grpel2	NM_021296
6866004	7128	8.42565	8.497602	8.299076	8.736958	-0.31131	0.094988	-0.23936	0.183632	-0.43788	0.028687	0.198526	0.261838	-0.07195	0.673301	0.126574	0.463618	6866004	8.222772	8.436077	8.6181	8.514816	8.441728	8.536263	8.358667	8.330696	8.207865	8.46059	8.636518	9.113765	Afap1l1	NM_178928
6866006	7129	5.871863	6.092766	6.130491	6.126264	-0.2544	0.092627	-0.0335	0.807839	0.004227	0.975469	-0.03772	0.784266	-0.2209	0.13592	-0.25863	0.088203	6866006	5.813589	5.85099	5.951011	6.241534	6.008306	6.028459	5.9334	6.204107	6.253965	6.124996	6.361448	5.892347	Ablim3	NM_198649
6866021	7130	6.302775	6.236961	5.685934	5.915822	0.386953	0.201134	0.321139	0.281035	-0.22989	0.431929	0.551028	0.082597	0.065814	0.818681	0.616842	0.057143	6866021	6.148534	5.816954	6.942838	6.403549	6.160105	6.14723	5.904535	5.462202	5.691064	6.195077	5.780714	5.771676	Adrb2	NM_007420
6866039	7131	8.041723	8.181948	8.098556	8.05429	-0.01257	0.862676	0.127658	0.107156	0.044266	0.546776	0.083392	0.269913	-0.14023	0.081383	-0.05683	0.442562	6866039	8.100994	8.017241	8.006935	8.126654	8.260316	8.158875	8.22721	7.999609	8.06885	7.965189	8.047794	8.149888	Fbxo38	NM_134136
6866054	7133	6.85751	6.850639	6.532384	6.307047	0.550464	0.06622	0.543592	0.069012	0.225337	0.409523	0.318255	0.253967	0.006872	0.979478	0.325126	0.244688	6866054	7.093305	6.956652	6.522574	6.764587	7.062136	6.725193	6.821738	6.119957	6.655457	5.869401	6.563536	6.488203	Fam38b	BC147606
6866068	7134	8.679934	8.796983	8.831734	8.780274	-0.10034	0.460701	0.016709	0.900517	0.05146	0.701464	-0.03475	0.795206	-0.11705	0.39245	-0.1518	0.274811	6866068	8.588783	8.724886	8.726133	8.72579	8.916293	8.748865	9.010813	8.886662	8.597728	8.617337	8.998615	8.72487	Txnl1	NM_016792
6866112	7135	7.527052	7.709664	7.449967	7.654906	-0.12785	0.241185	0.054758	0.602489	-0.20494	0.076971	0.259697	0.033066	-0.18261	0.108223	0.077085	0.46725	6866112	7.533785	7.565447	7.481925	7.795614	7.642889	7.69049	7.499295	7.360435	7.490171	7.430301	7.669968	7.86445	Fech	NM_007998
6866116	7136	9.529216	9.583741	9.679144	9.587467	-0.05825	0.547727	-0.00373	0.96896	0.091676	0.352185	-0.0954	0.334053	-0.05453	0.573071	-0.14993	0.144878	6866116	9.504767	9.467639	9.615241	9.542528	9.65654	9.552156	9.886122	9.630933	9.520376	9.551408	9.680515	9.530479	Nars	NM_001142950
6866118	7137	9.313648	9.605167	9.516255	9.943974	-0.63033	0.009044	-0.33881	0.103035	-0.42772	0.048695	0.088912	0.642116	-0.29152	0.152023	-0.20261	0.303178	6866118	9.172245	9.600691	9.168007	9.546028	9.402873	9.8666	9.438532	9.811392	9.298841	9.876058	9.870383	10.08548	Atp8b1	NM_001001488
6866233	7140	10.43891	10.45586	10.68153	10.70656	-0.26765	0.031289	-0.2507	0.04046	-0.02503	0.813562	-0.22567	0.059187	-0.01695	0.872973	-0.24262	0.045744	6866233	10.4102	10.47624	10.4303	10.25607	10.48156	10.62996	10.86185	10.59978	10.58297	10.65283	10.7399	10.72695	Lman1	NM_027400
6866238	7141	6.473785	6.353065	6.080835	6.205029	0.268756	0.065639	0.148036	0.274129	-0.12419	0.353483	0.272229	0.062882	0.12072	0.366385	0.392949	0.014304	6866238	6.368894	6.53816	6.5143	6.335009	6.461322	6.262863	6.366223	5.81405	6.062233	6.208807	6.182913	6.223366	Ccbe1	NM_178793
6866257	7142	7.331257	7.76015	8.749672	7.576364	-0.24511	0.52189	0.183786	0.629075	1.173308	0.012501	-0.98952	0.026917	-0.42889	0.274954	-1.41842	0.004704	6866257	6.9237	7.492033	7.578039	7.370034	8.487831	7.422585	9.100242	8.728457	8.420318	7.526194	8.003444	7.199455	Mc4r	NM_016977
6866289	7143	6.980112	7.116989	7.027147	7.103475	-0.12336	0.144932	0.013514	0.863951	-0.07633	0.346885	0.089842	0.273288	-0.13688	0.110878	-0.04704	0.555117	6866289	6.989301	7.004358	6.946676	7.179151	7.15993	7.011887	7.101285	7.103016	6.87714	7.137356	6.997148	7.175921	Mppe1	NM_172630
6866299	7144	7.957688	7.953934	7.813332	8.058995	-0.10131	0.291302	-0.10506	0.275049	-0.24566	0.025463	0.140603	0.155515	0.003754	0.967632	0.144357	0.14609	6866299	7.966328	7.899091	8.007646	7.994576	7.921255	7.945972	7.864503	7.759732	7.81576	7.950687	7.933418	8.29288	Afg3l2	NM_027130
6866305	7145	7.42717	7.398898	7.290098	7.120728	0.306442	0.02519	0.27817	0.037335	0.16937	0.167481	0.1088	0.35804	0.028272	0.80635	0.137072	0.254148	6866305	7.576827	7.287224	7.41746	7.421498	7.428736	7.346461	7.518612	7.156152	7.195531	7.143675	7.00142	7.21709	Spire1	NM_194355
6866307	7146	10.31581	9.878891	10.0146	9.053045	1.262761	0.011178	0.825845	0.064222	0.961558	0.037052	-0.13571	0.733515	0.436916	0.289199	0.301204	0.456447	6866307	10.48904	10.16966	10.28872	9.551147	10.30087	9.784655	10.52904	9.224329	10.29044	9.139235	9.486953	8.532948	Ptpn2	NM_001127177
6866354	7147	8.330117	8.492963	8.40056	8.630316	-0.3002	0.055244	-0.13735	0.33498	-0.22976	0.124502	0.092403	0.509631	-0.16285	0.258554	-0.07044	0.613074	6866354	8.126079	8.408079	8.456193	8.455724	8.459033	8.564133	8.247355	8.625149	8.329176	8.445039	8.641048	8.80486	4933403F	NM_153794
6866486	7149	7.214431	7.514718	7.227829	7.304018	-0.08959	0.799438	0.2107	0.553888	-0.07619	0.828829	0.286889	0.424673	-0.30029	0.40428	-0.0134	0.969627	6866486	7.184205	7.501718	6.957371	7.521031	7.411872	7.611251	7.232695	7.057468	7.393324	6.459902	7.501222	7.95093	Rab27b	NM_001082553
6866504	7150	6.357832	6.684129	6.541849	6.240135	0.117697	0.672733	0.443994	0.136809	0.301714	0.29373	0.14228	0.610567	-0.3263	0.258916	-0.18402	0.512491	6866504	6.527522	6.527447	6.018526	6.331907	6.754284	6.966197	6.734451	6.200326	6.690771	5.905579	6.672069	6.142757	4930503L	NM_172967
6866511	7151	7.212733	7.368372	7.43212	7.412051	-0.19932	0.179539	-0.04368	0.755472	0.02007	0.885927	-0.06375	0.650612	-0.15564	0.283933	-0.21939	0.14412	6866511	6.905776	7.360021	7.372403	7.287982	7.31904	7.498095	7.430698	7.481132	7.384531	7.247914	7.430291	7.557947	Poli	NM_011972
6866631	7153	9.813239	9.936446	9.801859	9.747506	0.065733	0.031921	0.18894	7.23E-05	0.054353	0.064295	0.134587	0.000719	-0.12321	0.001253	0.01138	0.665293	6866631	9.833853	9.80792	9.797945	9.951734	9.912789	9.944815	9.78995	9.853354	9.762273	9.732803	9.781978	9.727738	Smad4	NM_008540
6866635	7154	6.979137	7.12566	6.952249	7.152397	-0.17326	0.147315	-0.02674	0.810702	-0.20015	0.100969	0.173411	0.147008	-0.14652	0.211908	0.026888	0.809659	6866635	7.008383	7.056931	6.872097	7.343757	7.011761	7.021462	7.06227	7.01952	6.774958	7.174536	7.114851	7.167804	Elac1	NM_053255
6866643	7155	8.719512	8.903641	8.88608	8.829326	-0.10981	0.423848	0.074315	0.584119	0.056754	0.674674	0.017561	0.896125	-0.18413	0.195341	-0.16657	0.236982	6866643	8.786507	8.50029	8.87174	8.962167	8.759365	8.98939	8.830145	9.10507	8.723025	8.850163	8.723742	8.914072	Me2	NM_145494
6866653	7156	7.332919	7.310692	7.414847	7.220512	0.112407	0.557582	0.09018	0.636668	0.194334	0.320994	-0.10415	0.586274	0.022227	0.906678	-0.08193	0.667426	6866653	7.260956	7.505658	7.232143	7.586338	7.055039	7.2907	7.374015	7.732345	7.13818	7.089113	7.367173	7.205251	Mapk4	NM_172632
6866747	7159	6.256441	6.15539	6.145469	6.180848	0.075593	0.363206	-0.02546	0.753733	-0.03538	0.663799	0.009921	0.902432	0.101051	0.233467	0.110972	0.194683	6866747	6.218654	6.281523	6.269145	6.296583	6.138532	6.031054	6.042979	6.242531	6.150897	6.092593	6.270965	6.178985	Gm672	NM_201354
6866838	7161	7.473042	7.625047	7.472376	7.597495	-0.12445	0.18686	0.027553	0.757469	-0.12512	0.184762	0.152672	0.114538	-0.15201	0.115894	0.000666	0.994026	6866838	7.484989	7.469826	7.46431	7.531238	7.730933	7.612971	7.589964	7.439198	7.387965	7.420947	7.686024	7.685513	8030462N	NM_178670
6866839	7162	5.756144	6.066795	5.904129	5.829884	-0.07374	0.526008	0.236911	0.065805	0.074245	0.523248	0.162666	0.181775	-0.31065	0.023456	-0.14799	0.220054	6866839	5.641161	5.843151	5.784121	6.115345	6.112308	5.972732	6.109095	5.717988	5.885305	5.695692	5.827716	5.966244	Haus1	NM_146089
6866856	7164	11.57463	11.37415	11.58651	11.67346	-0.09883	0.33883	-0.2993	0.015102	-0.08695	0.396937	-0.21235	0.060323	0.200477	0.072966	-0.01188	0.905721	6866856	11.67096	11.38379	11.66914	11.24734	11.46053	11.41459	11.63725	11.45332	11.66895	11.68119	11.61364	11.72554	Slc14a2	NM_207651
6866862	7165	8.488525	8.602736	8.577644	8.713579	-0.22505	0.043893	-0.11084	0.273082	-0.13594	0.186937	0.025092	0.796661	-0.11421	0.259872	-0.08912	0.371749	6866862	8.496524	8.519956	8.449095	8.70796	8.550355	8.549893	8.518626	8.792215	8.42209	8.676115	8.656772	8.807851	Setbp1	NM_053099
6866918	7166	8.332134	8.376273	8.286214	8.178307	0.153827	0.185771	0.197966	0.099487	0.107907	0.339625	0.090059	0.421362	-0.04414	0.688797	0.04592	0.67707	6866918	8.337739	8.46944	8.189222	8.265429	8.442932	8.420457	8.362193	8.402124	8.094325	8.28132	8.073995	8.179606	Adnp2	NM_175028
6866919	7167	6.913391	6.998958	6.817457	6.821753	0.091639	0.437235	0.177205	0.15251	-0.0043	0.970365	0.181501	0.144014	-0.08557	0.467102	0.095934	0.416914	6866919	6.900413	6.934726	6.905035	7.078066	7.152732	6.766076	6.942575	6.711909	6.797887	6.749769	6.733341	6.982148	1110032A	BC064066

6866936	7168	7.268768	7.320369	7.241416	7.323829	-0.05506	0.547165	-0.00346	0.969462	-0.08241	0.374352	0.078953	0.393761	-0.0516	0.572079	0.027352	0.762849	6866936	7.296824	7.247143	7.262336	7.395949	7.436306	7.128853	7.147893	7.269598	7.306757	7.275867	7.44188	7.253741	Ctdp1	NM_026295
6866955	7169	7.72692	7.570958	7.620945	7.549238	0.177682	0.153284	0.02172	0.851874	0.071708	0.542092	-0.04999	0.668903	0.155962	0.203492	0.105974	0.374252	6866955	7.707886	7.792072	7.680801	7.715683	7.662489	7.334701	7.65391	7.746984	7.461942	7.515042	7.476013	7.656658	Nfatc1	NM_198429
6866960	7170	7.99281	8.018818	8.02829	7.94025	0.052561	0.477999	0.078569	0.298209	0.08804	0.24778	-0.00947	0.896622	-0.02601	0.722217	-0.03548	0.628921	6866960	8.01422	7.972976	7.991235	7.950052	8.116256	7.990147	8.188454	7.945955	7.95046	7.880035	7.96729	7.973424	Atp9b	NM_015805
6867165	7172	7.683678	7.898604	7.610435	7.710848	-0.02717	0.843922	0.187756	0.197542	-0.10041	0.473826	0.288169	0.06309	-0.21493	0.146344	0.073242	0.598503	6867165	7.467934	7.669485	7.913614	8.049145	7.835352	7.811315	7.76734	7.570915	7.493051	7.574551	7.857941	7.700053	Tshz1	NM_001081300
6867193	7173	6.736096	7.139878	7.000719	7.694173	-0.95808	0.091563	-0.5543	0.299663	-0.69345	0.202747	0.139159	0.787755	-0.40378	0.442536	-0.26462	0.610879	6867193	5.961299	6.783768	7.46322	7.117256	6.915231	7.387146	7.359695	7.324852	6.31761	7.50801	7.079456	8.495053	Cndp1	NM_177450
6867197	7174	8.450472	8.514542	8.636434	8.481066	-0.03059	0.90556	0.033476	0.896719	0.155368	0.551333	-0.12189	0.638724	-0.06407	0.804079	-0.18596	0.477971	6867197	8.256481	8.33915	8.755786	8.499545	8.496064	8.548018	9.036955	8.665433	8.206914	8.232024	8.318073	8.893101	Cndp2	NM_023149
6867222	7175	7.073276	7.476868	7.241689	7.058717	0.014559	0.934286	0.418151	0.040328	0.182972	0.316126	0.235179	0.206577	-0.40359	0.046054	-0.16841	0.353827	6867222	6.863897	7.172574	7.183356	7.415103	7.584548	7.430952	7.384791	7.101645	7.23863	6.685338	7.148472	7.342341	1700034H	NM_025969
6867363	7176	8.933813	9.060033	8.82956	8.686488	0.247325	0.221755	0.373546	0.080361	0.143073	0.465391	0.230473	0.25196	-0.12622	0.517969	0.104252	0.59177	6867363	8.75797	8.666262	9.377206	8.849075	9.059403	9.271622	8.955939	8.755627	8.777115	8.644218	8.745327	8.669918	Socs6	NM_018821
6867379	7177	8.203913	8.710326	8.295224	8.849023	-0.64511	0.071314	-0.1387	0.666863	-0.5538	0.112256	0.415102	0.217917	-0.50641	0.141453	-0.09131	0.776127	6867379	7.848516	8.471762	8.291461	8.428453	8.56078	9.141745	7.78169	8.292609	8.811373	8.551988	8.988132	9.006948	9330132A	BC098197
6867566	7179	9.186873	9.213987	9.124187	9.071517	0.115357	0.626334	0.14247	0.549228	0.05267	0.823002	0.0898	0.703808	-0.02711	0.908217	0.062686	0.790208	6867566	9.366049	9.239299	8.955272	9.452283	9.363536	8.826142	9.0343	9.191813	9.146448	8.859864	8.842188	9.512498	Cpt1a	NM_013495
6867593	7181	8.69977	8.525556	8.595441	8.762076	-0.06231	0.590686	-0.23652	0.066146	-0.16663	0.172456	-0.06989	0.547294	0.174214	0.155896	0.104329	0.375684	6867593	8.778156	8.686569	8.634585	8.448163	8.716629	8.411875	8.623936	8.743278	8.41911	8.761395	8.882193	8.642639	1810055G	NM_028077
6867598	7182	8.348431	8.493226	8.291486	8.468901	-0.12047	0.197177	0.024325	0.783607	-0.17742	0.072059	0.20174	0.04628	-0.1448	0.129371	0.056945	0.524818	6867598	8.282455	8.385571	8.377266	8.384614	8.418269	8.676795	8.352198	8.316047	8.206212	8.441259	8.388813	8.576631	Suv420h1	NM_144871
6867609	7183	7.748131	7.976945	7.7965	8.661215	-0.91308	0.003856	-0.68427	0.0168	-0.86471	0.005204	0.180446	0.45016	-0.22881	0.343553	-0.04837	0.836803	6867609	7.597875	7.847398	7.799121	8.024758	7.768118	8.13796	7.557175	8.19472	7.637604	8.524832	8.380065	9.078747	Chka	NM_013490
6867615	7184	8.877899	8.726858	8.746145	8.255841	0.622057	0.004449	0.471016	0.018023	0.490304	0.014983	-0.01929	0.906376	0.151041	0.369629	0.131754	0.43102	6867615	8.985084	8.77622	8.872392	8.700035	8.980456	8.500082	8.962658	8.513343	8.762434	8.181452	8.458843	8.127229	Unc93b1	NM_019449
6867618	7185	5.689582	5.811735	5.726175	5.763376	-0.07379	0.836914	0.04836	0.89261	-0.0372	0.917269	0.08556	0.811456	-0.12215	0.733919	-0.03659	0.918612	6867618	5.517036	6.114965	5.436744	5.598578	6.081889	5.754739	4.995993	5.75148	6.431053	5.802216	5.832753	5.655158	Aldh3b1	NM_026316
6867622	7186	7.313339	7.154997	6.943443	7.202387	0.110952	0.558852	-0.04739	0.801055	-0.25894	0.192426	0.211554	0.278381	0.158342	0.409438	0.369896	0.076464	6867622	7.389484	7.223747	7.326786	7.3646	7.179277	6.921113	6.939438	6.943211	6.947679	7.081773	6.900722	7.624667	Acy3	NM_027857
6867626	7188	6.753454	6.933998	6.75925	6.857375	-0.10392	0.33973	0.076623	0.475572	-0.09812	0.365844	0.174748	0.12619	-0.18054	0.11579	-0.0058	0.956236	6867626	6.84929	6.551237	6.859835	7.059533	6.937229	6.805233	6.732081	6.892676	6.652993	6.837875	6.895415	6.838835	Nudt8	NM_025529
6867627	7189	7.25683	7.275253	7.166923	7.278416	-0.02159	0.79509	-0.00316	0.969578	-0.11149	0.202878	0.10833	0.214707	-0.01842	0.824481	0.089907	0.295845	6867627	7.347064	7.246579	7.176847	7.278228	7.261675	7.285856	6.982122	7.266916	7.251732	7.293677	7.344622	7.196949	Doc2g	NM_021791
6867632	7190	6.236021	6.320104	6.260908	6.387037	-0.15102	0.129985	-0.06693	0.47591	-0.12613	0.19638	0.059196	0.526893	-0.08408	0.374932	-0.02489	0.787988	6867632	6.394672	6.128626	6.184764	6.36322	6.20607	6.391023	6.179449	6.339087	6.264188	6.508848	6.295599	6.356665	Cabp2	NM_013878
6867637	7191	8.587675	8.43059	8.660897	8.53917	0.048505	0.710342	-0.10858	0.413976	0.121726	0.362364	-0.23031	0.10503	0.157085	0.247835	-0.07322	0.577213	6867637	8.532337	8.51416	8.716528	8.30481	8.529906	8.457055	8.598506	8.843079	8.541105	8.669999	8.649834	8.297678	Cdk2ap2	NM_026373
6867638	7192	7.742823	7.749087	7.68967	7.628639	0.114184	0.226428	0.120447	0.204247	0.061031	0.503529	0.059416	0.514569	-0.00626	0.944462	0.053153	0.558788	6867638	7.800565	7.783433	7.644472	7.757655	7.845808	7.643797	7.695696	7.841889	7.531426	7.589705	7.596047	7.700166	Pitpnm1	NM_008851
6867642	7193	9.296944	9.287059	9.203559	9.188295	0.108648	0.23041	0.098763	0.271882	0.015263	0.85983	0.0835	0.347656	0.009885	0.908894	0.093385	0.296909	6867642	9.280843	9.234685	9.375303	9.301832	9.309425	9.249919	9.361271	9.249119	9.000286	9.163036	9.235991	9.165859	Tmem134	NM_001078649
6867644	7195	9.692785	9.613616	9.742617	9.345762	0.347023	0.052902	0.267854	0.117869	0.396855	0.031824	-0.129	0.423298	0.079169	0.618582	-0.04983	0.752824	6867644	9.699224	9.639673	9.739457	9.575604	9.785783	9.479461	10.10902	9.480092	9.638739	9.260594	9.415833	9.360859	Coro1b	NM_011778
6867646	7196	6.050831	6.003073	6.153648	5.96945	0.081381	0.54005	0.033623	0.798136	0.184198	0.185475	-0.15058	0.27032	0.047758	0.71698	-0.10282	0.442122	6867646	5.968359	6.208603	5.975531	5.978065	6.127497	5.903656	6.179911	5.999644	6.281389	6.192666	5.945014	5.77067	Ptprcap	NM_016933
6867650	7197	10.79785	10.79794	10.69929	10.52328	0.27457	0.066524	0.274657	0.066455	0.176007	0.210662	0.09865	0.467514	-8.7E-05	0.999482	0.098563	0.467892	6867650	10.77898	10.69437	10.92021	10.67421	10.92262	10.79699	10.96153	10.60956	10.52678	10.37263	10.55848	10.63874	Ppp1ca	NM_031868
6867653	7198	7.816966	7.600098	7.780379	7.451792	0.365174	0.104644	0.148305	0.478622	0.328587	0.138253	-0.18028	0.392689	0.216868	0.308794	0.036587	0.85909	6867653	7.666064	7.692727	8.092107	7.528738	7.946939	7.324616	7.91445	7.879713	7.546975	7.551148	7.583137	7.221092	Clcf1	NM_019952
6867655	7199	7.763661	7.75442	7.631235	7.798248	-0.03459	0.778006	-0.04383	0.721327	-0.16701	0.196753	0.123185	0.329368	0.009242	0.939807	0.132426	0.296618	6867655	7.656379	7.782768	7.851837	7.792757	7.863332	7.60717	7.794642	7.371878	7.727185	7.774819	7.738708	7.881218	Pold4	NM_027196
6867677	7200	7.758818	7.564285	7.184777	7.128571	0.630247	0.062255	0.435714	0.172743	0.056206	0.851677	0.379508	0.228512	0.194533	0.52269	0.574041	0.084035	6867677	7.777414	7.476763	8.022277	7.337649	7.79993	7.555276	7.718992	6.575639	7.259701	6.875653	7.288003	7.222058	Pcx	NM_008797
6867685	7201	6.694949	6.870029	6.701727	6.768602	-0.07365	0.376306	0.101427	0.2331	-0.06688	0.419757	0.168302	0.064726	-0.17508	0.056587	-0.00678	0.933419	6867685	6.558132	6.795789	6.730925	6.909379	6.839988	6.860719	6.726115	6.678316	6.70075	6.630627	6.914298	6.760881	Spnb3	NM_021287
6867686	7202	6.432658	6.684763	6.566233	6.742628	-0.30997	0.082319	-0.05786	0.720492	-0.17639	0.291213	0.11853	0.469443	-0.2521	0.144972	-0.13357	0.417057	6867686	6.128315	6.436872	6.732787	6.565074	6.639024	6.85019	6.460507	6.727509	6.510682	6.614056	6.827363	6.786464	Rbm4b	NM_025717
6867701	7204	7.742146	7.946639	7.87552	8.375609	-0.63346	0.000507	-0.42897	0.005265	-0.50009	0.00221	0.071119	0.546645	-0.20449	0.10794	-0.13337	0.271777	6867701	7.800188	7.769218	7.657033	7.994592	7.919646	7.925679	7.921332	7.89634	7.808887	8.323238	8.149483	8.654107	Ctsf	NM_019861
6867702	7205	7.445528	7.420791	7.470847	7.474507	-0.02898	0.678572	-0.05372	0.448467	-0.00366	0.958025	-0.05006	0.478915	0.024737	0.723122	-0.02532	0.716938	6867702	7.405499	7.485482	7.445602	7.290855	7.481066	7.490451	7.391809	7.580553	7.440178	7.445137	7.437097	7.541286	Zdhhc24	NM_027476
6867706	7206	7.464103	7.553538	7.414087	7.490753	-0.02665	0.743095	0.062786	0.447117	-0.07667	0.357569	0.139451	0.113708	-0.08944	0.287746	0.050015	0.542018	6867706	7.511203	7.387806	7.493299	7.571102	7.617127	7.472386	7.517078	7.426367	7.298817	7.568258	7.349213	7.554787	Mrpl11	NM_025553
6867710	7208	6.235206	6.485106	6.359551	6.442177	-0.20697	0.094569	0.042928	0.704425	-0.08263	0.470837	0.125554	0.283327	-0.2499	0.051338	-0.12435	0.287657	6867710	6.127243	6.185858	6.392518	6.598987	6.350247	6.506083	6.548467	6.301178	6.229009	6.365485	6.418787	6.54226	B3gnt1	NM_175383
6867711	7209	7.381348	7.490777	7.490923	7.41946	-0.03811	0.687778	0.071316	0.45788	0.071463	0.456989	-0.00015	0.998759	-0.10943	0.265665	-0.10958	0.265075	6867711	7.320848	7.454578	7.368618	7.495702	7.498642	7.477986	7.736057	7.380931	7.355782	7.432581	7.403216	7.422584	Brms1	NM_134155
6867715	7210	6.867446	6.677661	6.615791	6.548811	0.318635	0.005046	0.128849	0.160407	0.06698	0.444479	0.061869	0.478783	0.189786	0.052162	0.251655	0.016514	6867715	6.80708	6.927735	6.867524	6.705061	6.82532	6.502601	6.64935	6.553415	6.644609	6.483927	6.654005	6.508502	Rin1	NM_145495
6867720	7211	6.926832	6.690465	6.608842	6.393375	0.533457	0.092687	0.29709	0.318771	0.215467	0.462856	0.081623	0.777659	0.236367	0.422235	0.31799	0.288097	6867720	7.14872	6.913033	6.718744	6.632173	7.298815	6.140408	6.789547	6.434708	6.602272	6.134505	6.571989	6.473631	Cd248	NM_054042
6867728	7212	8.153249	8.02486	8.169544	8.314513	-0.16126	0.200535	-0.28965	0.036623	-0.14497	0.24524	-0.14468	0.246089	0.128388	0.299007	-0.0163	0.891385	6867728	8.06756	8.258728	8.133458	7.984622	8.222921	7.867038	8.212555	8.300631	7.995447	8.204185	8.305825	8.433528	Yif1a	NM_026553
6867742	7213	6.530877	6.583743	6.680552	6.748597	-0.21772	0.310374	-0.16485	0.435953	-0.06805	0.74372	-0.09681	0.643023	-0.05287	0.799225	-0.14967	0.477864	6867742	6.65063	6.602516	6.339486	6.562976	6.604826	6.583426	6.656486	7.131125	6.254045	6.689047	6.64201	6.914735	Gal3st3	NM_001024717
6867748	7215	7.413243	7.525175	7.430468	7.407441	0.005802	0.965062	0.117734	0.385906	0.023027	0.862109	0.094707	0.481757	-0.11193	0.408666	-0.01722	0.896579	6867748	7.355063	7.379217	7.505449	7.533316	7.656733	7.385477	7.37723	7.424442	7.489732	7.131924	7.662647	7.427753	Eif1ad	NM_027236
6867753	7216	8.150162	8.20281	8.183707	8.117754	0.032408	0.817282	0.085056	0.548332	0.065953	0.640046	0.019103	0.891549	-0.05265	0.708206	-0.03355	0.811014	6867753	8.10282	8.25253	8.095135	8.038581	8.419887	8.149961	8.075143	8.330399	8.145578	8.367354	8.003242	7.982665	AI837181	NR_024189
6867755	7217	6.854298	6.865196	6.942822	6.518982	0.335316	0.028346	0.346214	0.024783	0.423839	0.009708	-0.07763	0.553639	-0.0109	0.932972	-0.08852	0.50083	6867755	7.077669	6.804012	6.681214	6.793137	6.98482	6.817632	6.95446	6.941691	6.932314	6.742162	6.478469	6.336316	Fosl1	NM_010235
6867758	7218	7.998282	8.015294	8.006342	7.91807	0.080213	0.493963	0.097225	0.410329	0.088273	0.45303	0.008952	0.938215	-0.01701	0.882953	-0.00806	0.944359	6867758	8.172475	7.93579	7.886582	8.009832	8.234512	7.801539	8.070153	7.994689	7.954185	7.912075	7.8834	7.958734	Fibp	NM_021438
6867761	7219	9.360915	9.156251	9.071576	8.980753	0.380161	0.0234	0.175497	0.23309	0.090823	0.52318	0.084674	0.551003	0.204664	0.170895	0.289338	0.066123	6867761	9.414012	9.273458	9.395274	9.088256	9.388159	8.992337	9.296183	9.019797	8.898749	8.831421	9.121486	8.989353	Efemp2	NM_021474
6867775	7221	7.580354	7.682352	7.666723	7.500316	0.080039	0.238693	0.182036	0.020023	0.166407	0.029387	0.015629	0.809925	-0.102	0.143351	-0.08637	0.206734	6867775	7.540524	7.553576	7.646963	7.642817	7.787273	7.616965	7.788112	7.603423	7.608634	7.47293	7.531238	7.496779	Rnaseh2c	NM_026616
6867776	7222	10.12383	9.955071	9.901293	9.805233	0.318593	0.042044	0.149838	0.288541	0.096059	0.48693	0.053778	0.693979	0.168756	0.236319	0.222534	0.129835	6867776	10.23715	10.0596	10.07473	9.930315	10.18579	9.749108	9.814013	10.04789	9.841975	9.772335	9.992852	9.650513	Rela	NM_009045
6867780	7223	8.520359	8.437485	8.449882	8.359257	0.161101	0.272854	0.078228	0.583195	0.090624	0.526362	-0.0124	0.930034	0.082874	0.561497	0.070477	0.620409	6867780	8.543739	8.537889	8.479448	8.524969	8.664494	8.122992	8.319834	8.650745	8.379066	8.32729	8.364853	8.385629	Map3k11	NM_022012
6867785	7225	9.947964	10.05884	9.805033	10.17931	-0.23135	0.24401	-0.12048	0.530915	-0.37428	0.076319	0.253805	0.205027	-0.11087	0.563402	0.142931	0.459542	6867785	10.07867	10.03978	9.725442	10.03145	10.24406	9.901004	9.685212	10.17898	9.550907	10.16471	10.02534	10.34789	Ltbp3	NM_008520
6867806	7226	7.924919	8.005391	7.923563	7.930257	-0.00534	0.974125	0.075134	0.650239	-0.00669	0.96756	0.081828	0.621863	-0.08047	0.627556	0.001355	0.99343	6867806	7.823945	7.920315	8.030496	7.989628	7.958079	8.068466	8.12201	7.514465	8.134215	7.91803	7.821845	8.050896	Slc25a45	NM_134154
6867823	7227	8.482762	8.346389	8.604234	8.727886	-0.24512	0.560772	-0.3815	0.372576	-0.12365	0.767325	-0.25785	0.541088	0.136373	0.74434	-0.12147	0.771289	6867823	8.442519	8.420359	8.585407	8.165195	8.672823	8.201149	8.071255	9.431539	8.309908	9.022279	9.126686	8.034693	Syvn1	NM_028769
6867825	7228	7.220576	7.310428	7.362511	7.266912	-0.04634	0.773483	0.043517	0.786856	0.0956	0.556077	-0.05208	0.746476	-0.08985	0.579568	-0.14194	0.3884	6867825	7.141789	7.08224	7.437698	7.21883	7.471588	7.240867	7.490139	7.444601	7.152794	7.005744	7.46682	7.328171	Znhit2	NM_013859
6867840	7231	6.663154	6.624926	6.756141	6.743999	-0.08085	0.405371	-0.11907	0.231883	0.012142	0.898309	-0.13122	0.191821	0.038228	0.68882	-0.09299	0.341954	6867840	6.707002	6.714041	6.568419	6.644345	6.662695	6.567737	6.924229	6.687491	6.656704	6.875015	6.593335	6.763648	Batf2	NM_028967
6867845	7232	6.435657	6.390716	6.372259	6.292342	0.143315	0.538743	0.098374	0.671043	0.079917	0.72955	0.018457	0.936122	0.044941	0.84544	0.063398	0.783575	6867845	6.506239	6.331952	6.46878	6.354181	6.399661	6.418306	6.240396	6.376134	6.500246	6.535975	5.693084	6.647967	Gpha2	NM_130453
6867846	7233	7.841372	7.78111	7.754409	7.733935	0.107437	0.103775	0.047175	0.443588	0.020474	0.73554	0.026701	0.660418	0.060262	0.333368	0.086963	0.175682	6867846	7.8969	7.816345	7.810872	7.797291	7.86954	7.676499	7.66348	7.832223	7.767525	7.694145	7.770901	7.736759	Atg2a	NM_194348
6867849	7234	8.677227	8.51928	8.653255	8.394537	0.28269	0.218371	0.124743	0.571831	0.258718	0.256306	-0.13398	0.544355	0.157947	0.476818	0.023971	0.912607	6867849	8.986198	8.534259	8.511223	8.582011	8.848023	8.127805	8.791136	8.570546	8.598084	8.400585	8.61486	8.168166	Ehd1	NM_010119
6867852	7235	7.953942	7.821374	7.839835	7.87102	0.082922	0.364377	-0.04965	0.580612	-0.03118	0.726975	-0.01846	0.835828	0.132568	0.162746	0.114107	0.222294	6867852	7.938105	7.818977	8.104744	7.849957	7.857934	7.756231	7.80849	7.9575	7.753515	7.879547	7.96481	7.768702	Men1	NM_008583
6867853	7236	7.794295	7.752079	7.774345	7.768642	0.025653	0.802637	-0.01656	0.871646	0.005703	0.955602	-0.02227	0.82817	0.042216	0.681892	0.01995	0.845762	6867853	7.796008	7.732077	7.8548	7.824444	7.798097	7.633697	7.661164	8.014647	7.647225	7.754568	7.74126	7.810099	Map4k2	NM_009006
6867854	7237	9.476608	9.449142	9.482768	9.523355	-0.04675	0.744619	-0.07421	0.606949	-0.04059	0.777127	-0.03363	0.814437	0.027466	0.847876	-0.00616	0.965648	6867854	9.494158	9.396338	9.539329	9.448728	9.589508	9.30919	9.255692	9.797972	9.394639	9.533266	9.622746	9.414053	Sf1	NM_001110791
6867859	7238	8.54793	8.301777	8.400585	9.189317	-0.64139	0.613323	-0.88754	0.487652	-0.78873	0.536044	-0.09881	0.937437	0.246153	0.845131	0.147345	0.906845	6867859	6.631681	8.092809	10.9193	9.59626	8.171714	7.137356	9.218825	8.65242	7.33051	7.699375	9.709926	10.15865	Pygm	NM_011224
6867860	7239	5.634356	5.516765	5.64689	6.104269	-0.46991	0.000957	-0.5875	0.000221	-0.45738	0.001133	-0.13012	0.197416	0.11759	0.23965	-0.01253	0.89563	6867860	5.761058	5.593253	5.548756	5.46955	5.449401	5.631345	5.49184	5.809545	5.639285	6.173573	6.062879	6.076354	Rasgrp2	NM_011242
6867861	7240	6.600039	6.627783	6.441367	6.468412	0.131627	0.292148	0.159371	0.209301	-0.02704	0.822593	0.186416	0.148926	-0.02774	0.818103	0.158672	0.211112	6867861	6.688594	6.66989	6.441633	6.807711	6.437732	6.637905	6.300166	6.618768	6.405167	6.510956	6.418353	6.475926	Nrxn2	NM_020253

6867872	7241	8.574769	8.405215	8.472087	8.674565	-0.0998	0.427677	-0.26935	0.054138	-0.20248	0.128485	-0.06687	0.590896	0.169555	0.193509	0.102682	0.414984	6867872	8.505662	8.499487	8.719159	8.302938	8.565558	8.347148	8.504405	8.647023	8.264833	8.764439	8.547021	8.712235	0610038D	NM_026306
6867879	7242	7.185067	7.192619	7.058609	7.019853	0.165214	0.174553	0.172766	0.157822	0.038756	0.735715	0.13401	0.261329	-0.00755	0.947371	0.126458	0.287077	6867879	7.216215	7.244305	7.094682	7.189946	7.144278	7.243634	7.030678	7.20921	6.93594	7.098639	6.777825	7.183095	Bad	NM_007522
6867883	7243	7.665349	7.774284	7.702468	7.978198	-0.31285	0.031229	-0.20391	0.127578	-0.27573	0.050586	0.071816	0.565955	-0.10894	0.390359	-0.03712	0.764898	6867883	7.660348	7.660537	7.675162	7.893135	7.615006	7.814711	7.683348	7.91044	7.513617	7.950293	7.832531	8.151771	Trpt1	NM_153597
6867885	7244	7.376183	7.452642	7.401124	7.725337	-0.34915	0.095413	-0.27269	0.178134	-0.32421	0.117323	0.051518	0.787408	-0.07646	0.689824	-0.02494	0.895937	6867885	7.248591	7.293257	7.586752	7.678947	7.24503	7.433948	7.425717	7.602806	7.174849	7.577863	7.551838	8.046309	Macrod1	NM_134147
6867917	7246	7.102014	7.265098	7.082326	7.113484	-0.01147	0.900973	0.151614	0.127994	-0.03116	0.736175	0.182771	0.074901	-0.16308	0.105255	0.019688	0.831038	6867917	7.131756	7.109288	7.064998	7.235573	7.322236	7.237484	7.156657	7.231349	6.858973	7.031619	7.134114	7.174719	2700081O	NM_175381
6867925	7247	9.159071	9.212071	9.178283	8.912759	0.246312	0.042515	0.299312	0.019044	0.265524	0.031723	0.033788	0.749456	-0.053	0.618112	-0.01921	0.855581	6867925	9.236297	9.161942	9.078975	9.124126	9.343213	9.168874	9.353327	9.084034	9.097489	8.795055	9.069949	8.873274	Atl3	NM_146091
6867930	7248	8.242642	8.391444	8.18485	8.269493	-0.02685	0.884023	0.121952	0.513313	-0.08464	0.647673	0.206594	0.279996	-0.1488	0.428177	0.057792	0.754153	6867930	8.049308	8.287411	8.391206	8.41159	8.414454	8.348289	8.519513	8.002191	8.032846	8.125445	8.098986	8.584047	Pla2g16	NM_139269
6867955	7253	8.322363	8.287645	8.502004	8.396474	-0.07411	0.552656	-0.10883	0.389372	0.10553	0.403244	-0.21436	0.110801	0.034718	0.778947	-0.17964	0.171432	6867955	8.309981	8.211947	8.445162	8.163642	8.562639	8.136655	8.556855	8.534818	8.41434	8.4091	8.485984	8.294339	Wdr74	NM_134139
6867956	7254	8.925213	9.01915	9.081128	9.132177	-0.20696	0.166613	-0.11303	0.4301	-0.05105	0.717188	-0.06198	0.660755	-0.09394	0.509349	-0.15591	0.284815	6867956	8.826798	8.914669	9.034172	8.875312	9.134971	9.047167	8.883104	9.37252	8.987759	9.209977	9.201771	8.984783	Stx5a	NM_019829
6867957	7255	9.175813	9.2949	9.312083	9.28619	-0.11038	0.279961	0.008711	0.929385	0.025893	0.792629	-0.01718	0.861331	-0.11909	0.246544	-0.13627	0.190413	6867957	9.207607	9.204073	9.115758	9.185457	9.447931	9.251313	9.158995	9.510712	9.266542	9.314451	9.273018	9.2711	Nxf1	NM_016813
6867959	7256	9.536598	9.846737	9.703962	9.767348	-0.23075	0.261369	0.079388	0.688507	-0.06339	0.748446	0.142775	0.476022	-0.31014	0.142978	-0.16736	0.406301	6867959	9.34203	9.364598	9.903167	9.810778	9.94216	9.787272	9.706473	9.962099	9.443314	9.623498	9.671727	10.00682	Tmem223	NM_025791
6867963	7257	6.403181	6.502754	6.360718	6.562842	-0.15966	0.083967	-0.06009	0.479018	-0.20212	0.03708	0.142036	0.117317	-0.09957	0.253498	0.042464	0.614008	6867963	6.40691	6.403686	6.398948	6.445801	6.463959	6.598502	6.423507	6.493507	6.165139	6.576427	6.60369	6.50841	Zbtb3	NM_001098237
6867966	7258	8.480414	8.40309	8.410153	8.414845	0.065569	0.716437	-0.01176	0.947859	-0.00469	0.979173	-0.00706	0.968654	0.077325	0.668923	0.070261	0.697298	6867966	8.689651	8.323242	8.42835	8.447338	8.583664	8.178267	8.661414	8.343489	8.225556	8.261369	8.303244	8.679923	Hnrnpul2	NM_001081196
6867972	7259	7.55233	7.749901	7.63871	7.640289	-0.08796	0.264657	0.109612	0.173332	-0.00158	0.983355	0.111191	0.167915	-0.19757	0.027316	-0.08638	0.272664	6867972	7.551202	7.588821	7.516968	7.724787	7.920096	7.60482	7.705339	7.604514	7.606278	7.588246	7.683276	7.649344	Ubxn1	NM_146093
6867973	7260	6.414695	6.573834	6.450628	6.56718	-0.15249	0.282818	0.006654	0.961162	-0.11655	0.404484	0.123206	0.379429	-0.15914	0.263868	-0.03593	0.793012	6867973	6.374801	6.415814	6.45347	6.457327	6.546447	6.717728	6.628327	6.514492	6.209065	6.41022	6.501817	6.789504	1810009A	BC047099
6867975	7261	7.574489	7.651768	7.706034	7.626867	-0.05238	0.599055	0.024902	0.801253	0.079167	0.432026	-0.05427	0.586197	-0.07728	0.442663	-0.13154	0.20649	6867975	7.627471	7.560059	7.535938	7.759354	7.752172	7.443779	7.692081	7.844951	7.58107	7.685355	7.608074	7.587171	Ints5	NM_176843
6867976	7262	10.04634	10.06547	10.17054	10.17155	-0.12521	0.317994	-0.10609	0.393274	-0.00102	0.993313	-0.10507	0.397615	-0.01913	0.874817	-0.1242	0.321693	6867976	10.12283	10.05813	9.958059	9.862007	10.14714	10.18725	10.34622	10.02959	10.1358	10.02216	10.20263	10.28987	Ganab	NM_008060
6867978	7263	8.575051	8.452973	8.671774	8.671116	-0.09606	0.469724	-0.21814	0.123161	0.000658	0.995978	-0.2188	0.122193	0.122078	0.363136	-0.09672	0.466788	6867978	8.475286	8.607257	8.64261	8.511296	8.444887	8.402736	8.615443	8.948393	8.451487	8.642247	8.541831	8.82927	B3gat3	NM_024256
6867980	7264	7.640121	7.736921	7.550396	7.458721	0.1814	0.05144	0.2782	0.007977	0.091675	0.280957	0.186525	0.046503	-0.0968	0.256905	0.089725	0.290576	6867980	7.743632	7.691715	7.485016	7.70497	7.832801	7.672992	7.546329	7.537336	7.567523	7.378338	7.581177	7.416648	Eml3	NM_144872
6867983	7265	8.836309	8.929001	8.794444	8.596749	0.23956	0.06322	0.332252	0.017343	0.197695	0.11314	0.134557	0.260537	-0.09269	0.428448	0.041865	0.716185	6867983	8.882422	8.790733	8.835773	8.833365	9.166484	8.787154	8.896004	8.815657	8.671672	8.504738	8.742258	8.543252	Mta2	NM_011842
6867984	7266	7.402391	7.400005	7.489567	7.458169	-0.05578	0.591425	-0.05816	0.575973	0.031398	0.761043	-0.08956	0.395575	0.002386	0.981507	-0.08718	0.407716	6867984	7.32841	7.422132	7.45663	7.263873	7.513217	7.422924	7.441876	7.669914	7.356911	7.527503	7.521399	7.325605	Tut1	NM_197993
6867987	7267	6.016875	5.959718	5.889993	5.376415	0.64046	0.020591	0.583302	0.030617	0.513577	0.049889	0.069725	0.762091	0.057157	0.803814	0.126882	0.584284	6867987	6.137202	5.880651	6.032772	5.984876	6.117437	5.77684	6.001117	5.766887	5.901974	4.855947	5.821392	5.451907	Ahnak	NM_009643
6868013	7268	13.22834	13.25681	13.21814	13.26567	-0.03733	0.444544	-0.00886	0.853431	-0.04752	0.335874	0.038667	0.428997	-0.02847	0.556677	0.010197	0.831626	6868013	13.27668	13.27324	13.1351	13.27985	13.23875	13.25183	13.16496	13.2333	13.25617	13.19537	13.30302	13.29861	Fth1	NM_010239
6868017	7269	7.006232	6.978376	6.956028	6.195934	0.810298	0.009087	0.782442	0.010819	0.760094	0.012459	0.022348	0.92717	0.027856	0.909305	0.050204	0.837485	6868017	7.348114	6.881668	6.788915	6.819171	7.261913	6.854045	7.244864	6.648605	6.974615	5.979022	6.553314	6.055466	Rab3il1	NM_144538
6868018	7270	8.481738	8.644677	8.086412	8.13483	0.346908	0.25926	0.509847	0.112154	-0.04842	0.869626	0.558265	0.086441	-0.16294	0.584106	0.395327	0.203808	6868018	8.482958	8.445016	8.517241	8.336919	9.066758	8.530354	8.248028	7.686749	8.324458	7.770229	8.669934	7.964327	Fads3	NM_021890
6868021	7271	8.626395	8.94151	8.528328	8.417746	0.208649	0.225646	0.523764	0.010931	0.110582	0.506267	0.413182	0.031629	-0.31512	0.082699	0.098067	0.554357	6868021	8.759414	8.640697	8.479073	8.78802	9.142641	8.893868	8.801875	8.245604	8.537504	8.288023	8.575166	8.390048	Fads1	NM_146094
6868023	7272	6.997789	7.059086	7.180348	7.000686	-0.0029	0.986948	0.0584	0.742446	0.179662	0.325839	-0.12126	0.499963	-0.0613	0.73025	-0.18256	0.318577	6868023	6.969223	6.971327	7.052818	6.813542	7.1147	7.249017	7.038138	7.549405	6.953502	7.043961	7.118452	6.839646	1810006K	BC024923
6868038	7274	7.561417	7.614962	7.649041	7.53903	0.022387	0.82745	0.075933	0.466841	0.110012	0.300555	-0.03408	0.740543	-0.05355	0.604755	-0.08762	0.403725	6868038	7.622805	7.580337	7.481108	7.593503	7.744537	7.506847	7.585356	7.853415	7.508353	7.483799	7.500121	7.633169	Cpsf7	NM_172302
6868041	7275	7.362083	7.379435	7.370277	7.096538	0.265545	0.023296	0.282897	0.017599	0.273739	0.020399	0.009158	0.92552	-0.01735	0.859515	-0.00819	0.933339	6868041	7.450556	7.296525	7.339168	7.350155	7.551154	7.236995	7.379328	7.310855	7.420647	6.975442	7.248492	7.06568	Cybasc3	NM_201351
6868048	7276	9.978534	10.06549	10.05636	9.819917	0.158617	0.117224	0.245568	0.026329	0.236442	0.030792	0.009126	0.922031	-0.08695	0.364029	-0.07783	0.414089	6868048	10.00757	9.95204	9.975992	9.992865	10.14876	10.05483	10.26154	9.948878	9.95866	9.706045	9.903608	9.850099	Ddb1	NM_015735
6868053	7278	8.176752	8.060329	8.238947	8.09995	0.076802	0.648854	-0.03962	0.813367	0.138997	0.416882	-0.17862	0.303313	0.116422	0.493773	-0.0622	0.71167	6868053	8.144184	8.172988	8.213083	8.148408	8.143112	7.889468	8.198358	8.617682	7.900801	8.088088	8.178489	8.033272	Vps37c	NM_181403
6868058	7280	7.911543	7.642225	7.773913	6.791256	1.120287	0.003302	0.850969	0.013879	0.982657	0.006764	-0.13169	0.640426	0.269318	0.349955	0.13763	0.625649	6868058	8.230866	7.993755	7.510007	7.585357	7.948867	7.392451	7.955241	7.359235	8.007263	6.52674	7.135036	6.711991	Slc15a3	NM_023044
6868066	7281	9.355556	9.382901	9.296886	9.234631	0.120925	0.453	0.14827	0.361814	0.062255	0.695339	0.086015	0.590134	-0.02735	0.862872	0.05867	0.711925	6868066	9.326562	9.304845	9.435261	9.272113	9.637661	9.23893	9.443703	9.404838	9.042118	9.040541	9.232256	9.431096	Prpf19	NM_134129
6868098	7285	8.338453	8.158853	8.243082	7.288466	1.049987	0.021566	0.870387	0.045934	0.954616	0.032176	-0.08423	0.825055	0.1796	0.639292	0.095371	0.802451	6868098	8.638268	8.004528	8.372563	8.084213	8.530756	7.861591	8.543618	7.68259	8.503038	7.597679	7.673069	6.59465	Ms4a6c	NM_028595
6868105	7286	7.022872	6.863652	6.952407	6.05222	0.970652	0.014099	0.811432	0.030972	0.900187	0.019918	-0.08876	0.78226	0.15922	0.621959	0.070465	0.826163	6868105	7.238803	6.751346	7.078468	6.8823	7.24658	6.462076	7.303687	6.695925	6.85761	6.268692	6.421896	5.466072	Ms4a6b	NM_027209
6868110	7287	6.762687	6.338365	6.239002	5.773403	0.989283	0.005412	0.564961	0.063089	0.465599	0.113366	0.099363	0.714297	0.424322	0.14389	0.523685	0.080596	6868110	7.040101	6.496548	6.751411	6.56168	6.621985	5.831429	6.528827	5.955311	6.232868	5.574078	6.05187	5.694262	Ms4a4d	NM_025658
6868128	7289	6.831606	6.937084	6.927437	7.00424	-0.17263	0.095973	-0.06716	0.484028	-0.0768	0.425728	0.009647	0.91865	-0.10548	0.282388	-0.09583	0.325657	6868128	6.834906	6.801083	6.858829	6.970171	6.913392	6.927688	6.951927	6.932057	6.898327	6.782529	7.010496	7.219695	Mrpl16	NM_025606
6868149	7291	8.176727	8.14857	8.123857	8.011569	0.165158	0.134206	0.137001	0.204257	0.112288	0.29006	0.024713	0.809382	0.028157	0.783556	0.05287	0.608234	6868149	8.25656	8.135221	8.138401	8.161563	8.295419	7.988728	8.043509	8.310265	8.017797	7.938612	8.065358	8.030737	Patl1	NM_172635
6868176	7298	7.770096	7.863406	7.649847	7.316058	0.454037	0.196399	0.547348	0.127749	0.333789	0.33046	0.213559	0.526034	-0.09331	0.779457	0.120248	0.718656	6868176	7.661257	7.490561	8.158469	7.370563	8.197738	8.021918	7.853251	7.148484	7.947807	6.962839	7.660268	7.325068	4632417K	NM_026640
6868193	7304	6.649336	6.727863	6.817171	6.179552	0.469784	0.056975	0.548311	0.031924	0.637619	0.016654	-0.08931	0.683806	-0.07853	0.719929	-0.16784	0.450132	6868193	6.806023	6.675696	6.466288	6.86502	6.966857	6.351712	7.071362	6.544724	6.835427	6.329454	6.313399	5.895802	Lpxn	NM_134152
6868356	7325	9.284608	9.246399	9.173102	9.081561	0.203047	0.022467	0.164838	0.051268	0.091541	0.239202	0.073298	0.338355	0.038209	0.609968	0.111507	0.159958	6868356	9.305232	9.222286	9.326307	9.18231	9.333103	9.223785	9.144189	9.26525	9.109866	8.973427	9.216648	9.054608	Gnaq	NM_008139
6868386	7327	7.238849	7.259701	7.421233	6.352091	0.886757	0.000739	0.90761	0.000636	1.069141	0.000215	-0.16153	0.363583	-0.02085	0.904096	-0.18238	0.308402	6868386	7.287911	7.176712	7.251923	7.164267	7.405769	7.209068	7.54866	7.380347	7.334691	6.57201	6.558354	5.92591	A230083H	BC094224
6868404	7328	9.63988	9.442406	9.432658	9.395658	0.244222	0.022065	0.046748	0.60246	0.037	0.67915	0.009748	0.91277	0.197474	0.05124	0.207222	0.042951	6868404	9.79569	9.520904	9.603045	9.442907	9.571477	9.312834	9.442073	9.513387	9.342514	9.372137	9.393085	9.421751	Rfk	NM_019437
6868511	7329	5.978178	6.083781	5.864374	6.065	-0.08682	0.740135	0.018781	0.942606	-0.20063	0.450347	0.219407	0.410755	-0.1056	0.687149	0.113803	0.664558	6868511	5.654293	5.794439	6.485801	5.834352	6.0834	6.333591	5.86963	5.825279	5.898214	6.3739	5.686707	6.134393	BC016495	NM_145497
6868524	7331	8.131873	8.328153	7.90528	7.809333	0.322539	0.247177	0.518819	0.079509	0.095947	0.720006	0.422873	0.140318	-0.19628	0.469218	0.226593	0.40603	6868524	8.065019	8.451991	7.878608	8.372112	8.458035	8.154311	7.972856	7.477039	8.265945	7.543045	7.662756	8.222199	Trpm6	NM_153417
6868582	7333	6.249092	7.348019	6.62611	7.173767	-0.92467	0.104403	0.174252	0.738893	-0.54766	0.30965	0.721909	0.190628	-1.09893	0.061186	-0.37702	0.476589	6868582	5.538826	5.949859	7.258592	8.05803	7.236687	6.749341	6.915108	6.629944	6.333279	7.422078	7.442729	6.656494	Aldh1a1	NM_013467
6868618	7334	8.790543	8.926234	8.88139	8.80958	-0.01904	0.795986	0.116654	0.140037	0.07181	0.342796	0.044844	0.546442	-0.13569	0.093188	-0.09085	0.237856	6868618	8.841062	8.738554	8.792013	8.900151	8.868658	9.009892	8.959778	8.780061	8.90433	8.677017	8.850294	8.901428	Zfand5	NM_009551
6868649	7335	7.503272	7.690384	7.630416	7.602626	-0.09935	0.507637	0.087758	0.557183	0.02779	0.851032	0.059968	0.686539	-0.18711	0.227842	-0.12714	0.400726	6868649	7.456394	7.462408	7.591015	7.469887	7.900692	7.700573	7.869951	7.573489	7.447808	7.59095	7.76277	7.454158	Fam108b	NM_146096
6868650	7336	7.902747	8.250261	7.780902	7.83469	0.068057	0.621427	0.415571	0.013888	-0.05379	0.695456	0.469359	0.007598	-0.34751	0.030534	0.121844	0.384732	6868650	8.037705	7.903647	7.766888	8.32407	8.408829	8.017884	7.888662	7.749146	7.704899	7.639342	8.007908	7.86222	Tmem2	NM_031997
6868652	7337	7.589498	7.97228	7.585131	7.809854	-0.22036	0.371646	0.162426	0.505193	-0.22472	0.36273	0.387149	0.134933	-0.38278	0.138797	0.004367	0.985496	6868652	7.525529	7.758141	7.484823	8.037959	7.996275	7.882605	8.104869	7.137362	7.513162	7.529054	7.955141	7.945367	Ldhb	NM_008492
6868654	7338	7.064972	7.455828	7.033483	8.165955	-1.10098	0.012166	-0.71013	0.071176	-1.13247	0.0106	0.422345	0.251256	-0.39086	0.285488	0.031489	0.9288	6868654	6.467494	7.149852	7.577569	7.355244	7.620063	7.392177	7.25972	7.055712	6.785017	8.395942	7.532013	8.569909	Trpm3	NM_001035240
6868676	7339	10.19883	10.24473	10.04205	10.39808	-0.19925	0.195823	-0.15335	0.308998	-0.35603	0.0357	0.202678	0.189016	-0.04589	0.753454	0.156785	0.299006	6868676	10.0221	10.06043	10.51397	10.42665	10.07495	10.23258	9.964005	10.16173	10.00041	10.40375	10.3443	10.44619	Klf9	NM_010638
6868809	7341	6.196425	6.82126	6.371388	7.343301	-1.14688	0.032491	-0.52204	0.27359	-0.97191	0.060062	0.449872	0.340716	-0.62483	0.197079	-0.17496	0.703896	6868809	6.499608	5.822671	6.266996	7.402775	6.654975	6.406029	6.378757	6.458688	6.276718	8.327429	7.098709	6.603764	E030010A	NM_183160
6868830	7342	7.914866	7.826028	7.814248	7.683941	0.230925	0.298206	0.142087	0.512963	0.130307	0.54763	0.01178	0.956133	0.088838	0.679933	0.100618	0.640838	6868830	8.147666	7.967358	7.629574	7.834268	7.868086	7.77573	7.965489	7.773478	7.703777	7.249975	7.735034	8.066813	Dock8	NM_028785
6868833	7343	8.915893	8.803917	8.916634	9.016745	-0.10085	0.4631	-0.21283	0.142566	-0.10011	0.466281	-0.11272	0.414168	0.111976	0.417115	-0.00074	0.995619	6868833	8.927187	8.889889	8.930603	8.909689	8.870533	8.631528	8.848845	9.19795	8.703108	8.897087	9.011854	9.141294	Kank1	NM_181404
6868839	7345	6.10497	6.088109	6.228637	6.090941	0.014029	0.889731	-0.00283	0.977656	0.137696	0.197705	-0.14053	0.18957	0.016861	0.867694	-0.12367	0.242615	6868839	6.246247	5.967371	6.101292	6.132018	6.066264	6.066044	6.374671	6.094382	6.216858	6.223927	6.085601	5.963295	Dmrt3	NM_177360
6868884	7347	8.942367	9.067535	8.834132	9.161745	-0.21938	0.155488	-0.09421	0.519657	-0.32761	0.047282	0.233404	0.133793	-0.12517	0.397062	0.108236	0.46137	6868884	8.963214	8.976736	8.887152	9.131378	8.943296	9.127932	8.523086	9.067156	8.912153	8.98337	9.283871	9.217995	Smarca2	NM_011416
6868899	7348	8.423831	9.517977	9.217823	9.853079	-1.42925	0.000401	-0.3351	0.210283	-0.63526	0.032509	0.300154	0.257196	-1.09415	0.002147	-0.79399	0.012106	6868899	8.207746	8.631424	8.432323	9.686061	9.413924	9.453945	9.034497	9.728515	8.890456	9.785386	9.582932	10.19092	Vldlr	NM_013703
6869013	7351	10.12481	10.30484	9.930367	9.702416	0.422393	0.413536	0.602424	0.253615	0.227952	0.654021	0.374473	0.466451	-0.18003	0.722708	0.194442	0.701733	6869013	10.27399	9.756227	10.34421	9.87396	10.9374	10.10316	10.61171	9.149892	10.0295	10.06972	8.902327	10.1352	Slc1a1	NM_009199
6869015	7352	7.224973	7.254438	7.127604	7.37483	-0.14986	0.365996	-0.12039	0.463518	-0.24723	0.152564	0.126834	0.440807	-0.02946	0.855245	0.097369	0.550866	6869015	7.046856	7.142636	7.485427	7.390005	7.236884	7.136425	7.020328	7.251264	7.111221	7.296074	7.657263	7.171153	Ppapdc2	NM_028922
6869016	7353	7.858125	7.825138	7.785783	8.000826	-0.1427	0.100866	-0.17569	0.051869	-0.21504	0.023421	0.039355	0.622957	0.032987	0.679547	0.072342	0.374799	6869016	7.828428	7.887466	7.858481	7.772102	7.841938	7.861373	7.840612	7.871964	7.644772	7.940463	7.910255	8.151759	Cdc37l1	NM_025950

6869021	7354	7.084453	7.225796	7.093595	7.191525	-0.10707	0.417843	0.034271	0.791469	-0.09793	0.457145	0.132201	0.322387	-0.14134	0.292173	-0.00914	0.943653	6869021	7.140458	7.040808	7.072093	7.170446	7.275992	7.23095	7.002024	7.343883	6.934877	6.993962	7.397494	7.183119	Rcl1	NM_021525
6869027	7355	9.043672	9.012644	8.839096	8.915044	0.128627	0.457397	0.0976	0.569907	-0.07595	0.657053	0.173548	0.322894	0.031028	0.855295	0.204576	0.24947	6869027	8.960674	9.167425	9.002916	8.757159	8.994732	9.286041	8.989726	8.59592	8.931642	8.701733	9.059209	8.984191	Jak2	NM_008413
6869032	7356	7.587315	7.34402	7.4866	6.555273	1.032042	0.043137	0.788747	0.103891	0.931327	0.062174	-0.14258	0.748675	0.243296	0.586968	0.100715	0.82066	6869032	7.669404	7.974294	7.118248	7.207186	7.591803	7.23307	7.276741	6.837224	8.345835	6.682281	7.002035	5.981503	Cd274	NM_021893
6869038	7358	6.27935	6.181665	5.946442	6.304826	-0.02548	0.81829	-0.12316	0.284209	-0.35838	0.010231	0.235223	0.059716	0.097684	0.389226	0.332908	0.014609	6869038	6.494061	6.116691	6.227297	6.15696	6.140877	6.247159	5.781683	6.111322	5.946321	6.286782	6.286381	C030046E	NM_001081319
6869068	7359	7.158701	6.951491	6.309758	6.345015	0.813686	0.241766	0.606476	0.373658	-0.03526	0.957661	0.641733	0.347963	0.20721	0.755763	0.848943	0.223698	6869068	7.59882	6.798979	7.078304	6.881386	7.568382	6.404705	7.0055	5.974295	5.94948	5.383411	7.786091	5.865543	Il33	NM_133775
6869070	7361	8.299595	8.439451	8.262295	8.109311	0.190284	0.371459	0.330141	0.139074	0.152984	0.468377	0.177156	0.403765	-0.13986	0.506211	0.0373	0.857384	6869070	8.157203	8.293974	8.447607	8.145966	8.765065	8.407323	8.535866	7.961648	8.289371	8.205141	8.247886	7.874905	Uhrf2	NM_144873
6869128	7363	6.810308	7.0522	6.914173	6.809397	0.000911	0.994511	0.242804	0.095299	0.104776	0.438142	0.138027	0.313764	-0.24189	0.096346	-0.10386	0.441984	6869128	6.812464	6.968382	6.650078	6.975892	7.133015	7.047694	7.156858	6.772215	6.813446	6.651132	6.825045	6.952013	Cstf2t	NM_031249
6869212	7365	6.781818	6.613685	6.802579	6.692693	0.089125	0.611511	-0.07901	0.651938	0.109886	0.532941	-0.18889	0.295184	0.168133	0.347979	-0.02076	0.905063	6869212	6.715647	6.952476	6.677331	6.793541	6.797209	6.250304	7.053219	6.678115	6.676402	6.638191	6.727501	6.712386	Minpp1	NM_010799
6869216	7366	10.97374	10.76252	10.98014	11.12878	-0.15505	0.549718	-0.36626	0.178414	-0.14864	0.565956	-0.21762	0.406328	0.211213	0.419684	-0.0064	0.980057	6869216	11.11845	10.79398	11.00878	10.90382	10.66576	10.71799	10.43138	11.53035	10.97869	11.10054	10.98651	11.2993	Papss2	NM_011864
6869222	7367	9.18329	9.231541	9.105497	9.172615	0.010674	0.829253	0.058926	0.253609	-0.06712	0.198763	0.126044	0.030123	-0.04825	0.343303	0.077792	0.143047	6869222	9.231091	9.149881	9.168897	9.172325	9.289298	9.233	9.18537	9.070501	9.060621	9.10318	9.218158	9.196508	Pten	NM_008960
6869223	7368	6.314524	6.229883	6.077132	6.396468	-0.08194	0.664731	-0.16658	0.387144	-0.31934	0.117654	0.152751	0.42602	0.084641	0.654533	0.237392	0.228717	6869223	6.466006	6.538378	5.939188	6.07505	6.239187	6.375412	6.24431	5.895983	6.091103	6.183245	6.57301	6.433148	B430203M	NM_177096
6869294	7370	9.238228	9.674974	9.382924	6.825199	2.413029	0.017543	2.849774	0.007824	2.557725	0.013376	0.29205	0.727499	-0.43675	0.604065	-0.1447	0.862524	6869294	9.773178	9.168618	8.772888	8.99453	10.58114	9.449251	10.40146	7.791558	9.955754	6.667537	7.923599	5.884462	Lipk	NM_172837
6869305	7372	6.985805	6.783142	7.247851	6.816801	0.169004	0.20339	-0.03366	0.789632	0.43105	0.007698	-0.46471	0.005171	0.202663	0.135275	-0.26205	0.063994	6869305	6.977667	6.881087	7.09866	6.748464	7.033506	6.567455	7.161211	7.262973	7.319368	6.678468	6.924047	6.847888	Stambpl1	NM_029682
6869310	7373	7.226615	7.213616	7.165395	6.961946	0.264669	0.278539	0.25167	0.30112	0.203449	0.39762	0.04822	0.837563	0.012999	0.95587	0.061219	0.794818	6869310	7.254378	7.424933	7.000533	7.186638	7.253243	7.200966	7.373135	6.61492	7.508131	7.151334	6.941087	6.793417	Fas	NM_007987
6869334	7376	5.734508	6.668196	5.879009	5.35195	0.382558	0.613619	1.316246	0.108327	0.527058	0.489859	0.789187	0.310096	-0.93369	0.235722	-0.1445	0.847669	6869334	5.489965	6.093616	5.619944	5.936448	7.794091	6.274049	6.7013	4.360686	6.57504	4.712384	5.921403	5.422064	Ifit1	NM_008331
6869407	7378	7.496481	7.506459	7.530997	7.245557	0.250924	0.047645	0.260902	0.041213	0.28544	0.028879	-0.02454	0.824965	-0.00998	0.928249	-0.03452	0.756102	6869407	7.456829	7.518022	7.514592	7.383874	7.706518	7.428984	7.742155	7.438732	7.412105	7.222195	7.315887	7.198589	Rpp30	NM_019428
6869414	7379	7.63934	7.570532	7.849989	7.630421	0.008919	0.953131	-0.05989	0.694549	0.219568	0.173822	-0.27946	0.093961	0.068808	0.652387	-0.21065	0.189973	6869414	7.504143	7.645239	7.768638	7.599302	7.671318	7.440977	8.152296	7.791496	7.606174	7.46403	7.691263	7.735969	Pcgf5	NM_029508
6869503	7381	6.026149	6.356902	5.576634	4.94727	1.078879	0.049838	1.409632	0.016669	0.629364	0.215069	0.780267	0.133619	-0.33075	0.499292	0.449515	0.364382	6869503	6.119698	5.834936	6.123813	5.870133	7.096912	6.10366	6.051651	4.528488	6.149764	4.768072	5.130453	4.943285	Kif11	NM_010615
6869509	7382	9.555879	9.6716	9.314502	9.808415	-0.25254	0.319373	-0.13682	0.580976	-0.49391	0.071506	0.357098	0.171678	-0.11572	0.639658	0.241378	0.339914	6869509	9.420027	9.839388	9.408223	9.702951	9.578533	9.733315	8.831771	9.785706	9.326028	9.758867	9.625858	10.04052	Hhex	NM_008245
6869517	7383	8.530068	8.462312	8.568128	8.440891	0.089177	0.429072	0.021421	0.846422	0.127237	0.268774	-0.10582	0.351958	0.067756	0.544512	-0.03806	0.731432	6869517	8.531897	8.384484	8.673824	8.594389	8.294797	8.497751	8.628557	8.546219	8.529608	8.277772	8.481127	8.563775	Exoc6	NM_175353
6869537	7385	6.035605	6.300533	5.655028	4.948685	1.08692	0.031725	1.351848	0.012039	0.706343	0.129847	0.645505	0.161452	-0.26493	0.544276	0.380577	0.389611	6869537	6.095711	5.728656	6.282448	5.824193	7.117966	5.95944	6.027065	4.924362	6.013658	4.788957	5.242198	4.814901	Cep55	NM_028760
6869561	7389	7.619241	7.948254	7.461712	7.685156	-0.06591	0.643858	0.263098	0.09151	-0.22344	0.142124	0.486543	0.00756	-0.32901	0.043344	0.15753	0.284168	6869561	7.559983	7.535426	7.762315	7.681051	7.988997	8.174715	7.635227	7.343133	7.406775	7.602461	7.645282	7.807725	Tmem20	NM_175507
6869570	7390	7.081959	7.984177	6.414726	7.634709	-0.55275	0.208301	0.349468	0.412061	-1.21998	0.016539	1.569451	0.004635	-0.90222	0.055949	0.667233	0.13711	6869570	6.909217	7.617905	6.718755	8.077961	7.965383	7.909188	7.142034	5.929518	6.172626	7.225326	7.381647	8.297154	Plce1	NM_019588
6869573	7391	7.615115	7.696991	7.693281	7.578453	0.036662	0.74189	0.118538	0.302289	0.114827	0.316675	0.00371	0.973316	-0.08188	0.468187	-0.07817	0.487934	6869573	7.531639	7.649599	7.664107	7.539704	7.909374	7.641895	7.719334	7.739157	7.621351	7.550008	7.744499	7.440853	Tbc1d12	NM_145952
6869577	7392	6.316592	6.206583	5.965542	5.283377	1.033215	0.051666	0.923207	0.075472	0.682165	0.169824	0.241041	0.608467	0.110009	0.813903	0.35105	0.459868	6869577	6.191955	5.969447	6.783064	5.787691	6.693401	6.138658	6.497353	5.055232	6.344041	5.087934	5.80874	4.953456	Hells	NM_008234
6869635	7399	9.547108	9.937513	9.513631	9.186016	0.361092	0.395925	0.751497	0.0989	0.327615	0.439303	0.423882	0.323129	-0.3904	0.360557	0.033477	0.935769	6869635	9.557236	9.780916	9.303173	9.941882	10.15673	9.713927	10.2997	8.587342	9.653852	9.316785	9.445576	8.795688	Entpd1	NM_009848
6869656	7400	6.651612	6.73129	6.626766	6.658724	-0.00711	0.9454	0.072566	0.49146	-0.03196	0.758983	0.104524	0.329414	-0.07968	0.451409	0.024846	0.811241	6869656	6.595234	6.73129	6.628311	6.696279	6.724052	6.773538	6.523421	6.742834	6.614043	6.620292	6.880307	6.475573	Ccnj	NM_172839
6869724	7402	8.422341	8.617814	8.374334	8.479932	-0.05759	0.477236	0.137882	0.112073	-0.1056	0.208758	0.24348	0.013552	-0.19547	0.035216	0.048007	0.551546	6869724	8.452881	8.328421	8.48572	8.562481	8.520157	8.770803	8.408581	8.380047	8.334373	8.367716	8.543991	8.528088	Lcor	NM_172154
6869736	7403	6.107644	6.221341	6.107946	5.894179	0.213466	0.378815	0.327163	0.191215	0.213768	0.378174	0.113395	0.634003	-0.1137	0.633114	-0.0003	0.998981	6869736	6.27306	6.465577	5.584296	6.289855	6.288315	6.085854	5.920162	6.167788	6.235889	5.616	6.002436	6.0641	AI606181	BC116839
6869763	7405	7.342208	7.393228	7.366371	7.152538	0.18967	0.093396	0.240689	0.042105	0.213833	0.064122	0.026857	0.794295	-0.05102	0.622384	-0.02416	0.814455	6869763	7.438008	7.291744	7.296873	7.327811	7.56669	7.285182	7.529842	7.338492	7.230779	7.241564	7.135327	7.080724	Zdhhc16	NM_023740
6869766	7406	8.733414	8.876138	8.706656	8.740334	-0.00692	0.965358	0.135804	0.404844	-0.03368	0.83283	0.169482	0.304381	-0.14272	0.382428	0.026758	0.866746	6869766	8.764818	8.545448	8.889977	8.760027	9.175289	8.693098	8.739506	8.864646	8.515816	8.610309	8.828525	8.782169	Ubtd1	NM_145500
6869783	7407	6.607685	6.578776	6.61479	6.426823	0.180862	0.235228	0.151953	0.312299	0.187967	0.218938	-0.03601	0.804721	0.028909	0.842571	-0.00711	0.961021	6869783	6.374074	6.528445	6.920536	6.713187	6.523428	6.499714	6.706566	6.614658	6.523146	6.522892	6.275142	6.482435	Ankrd2	NM_020033
6869795	7410	8.600187	8.306699	8.519917	8.622457	-0.02227	0.870636	-0.31576	0.044253	-0.10254	0.461047	-0.21322	0.146072	0.293488	0.057534	0.08027	0.561247	6869795	8.766086	8.532172	8.502304	8.399085	8.443018	8.077994	8.488782	8.73672	8.33425	8.603481	8.575665	8.688225	Pi4k2a	NM_145501
6869796	7411	9.383582	9.437005	9.520481	9.002527	0.381055	0.03108	0.434478	0.017679	0.517954	0.007519	-0.08348	0.583039	-0.05342	0.723811	-0.1369	0.37567	6869796	9.486621	9.437233	9.226892	9.584659	9.405366	9.32099	9.63671	9.558805	9.365929	8.786916	9.300958	8.919707	Marveld1	BC054384
6869798	7412	7.62213	7.551988	7.613169	7.693691	-0.07156	0.263613	-0.1417	0.04449	-0.08052	0.213083	-0.06118	0.334058	0.070142	0.272468	0.008961	0.884056	6869798	7.710625	7.592805	7.56296	7.585404	7.593699	7.476862	7.582921	7.691006	7.565581	7.627903	7.671418	7.781753	Zfyve27	NM_177319
6869813	7414	7.004788	7.075087	6.919589	7.081636	-0.07685	0.330553	-0.00655	0.931829	-0.16205	0.060454	0.155498	0.069362	-0.0703	0.371071	0.085199	0.283964	6869813	7.006558	6.945792	7.062014	7.036474	7.108251	7.080537	7.04873	6.787972	6.922066	7.046428	6.997264	7.201216	D19Ertd3	NM_177464
6869885	7417	7.632905	7.600667	7.467192	7.617183	0.015722	0.948455	-0.01652	0.945855	-0.14999	0.542307	0.133475	0.58672	0.032238	0.894587	0.165712	0.501951	6869885	7.702703	7.515966	7.680045	7.512775	7.867241	7.421985	7.188944	7.995646	7.216987	7.594379	7.868457	7.388713	Entpd7	NM_053103
6869932	7421	11.43246	11.60183	11.49187	11.8176	-0.38514	0.060243	-0.21576	0.255429	-0.32573	0.101588	0.109967	0.549802	-0.16937	0.364413	-0.05941	0.744589	6869932	11.51266	11.24115	11.54357	11.62647	11.62324	11.55579	11.44571	11.72408	11.30581	11.58324	11.66956	12.19999	Scd2	NM_009128
6869951	7424	8.653354	8.85244	8.469532	8.754623	-0.10127	0.524295	0.097817	0.538176	-0.28509	0.09777	0.382909	0.035961	-0.19909	0.226941	0.183822	0.261372	6869951	8.369903	8.730727	8.859432	8.68761	8.822356	9.047355	8.469491	8.544172	8.394932	8.903225	8.541522	8.819123	Hif1an	NM_176958
6869973	7426	7.167805	7.265785	7.194967	7.053463	0.114342	0.280772	0.212322	0.06399	0.141505	0.190184	0.070817	0.494132	-0.09798	0.350636	-0.02716	0.790453	6869973	7.190163	7.16481	7.148442	7.102994	7.277892	7.416468	7.040745	7.390149	7.154008	7.045516	7.098517	7.016355	Fam178a	NM_001081225
6869975	7428	6.232111	6.474963	6.371372	6.369073	-0.13696	0.07321	0.105891	0.149672	0.002299	0.973242	0.103591	0.157596	-0.24285	0.006448	-0.13926	0.069376	6869975	6.111112	6.238468	6.346753	6.466992	6.430305	6.527593	6.410711	6.322384	6.381021	6.319448	6.31454	6.47323	Peo1	NM_153796
6869976	7429	7.979445	8.015771	8.023229	7.925307	0.054138	0.61952	0.090464	0.413282	0.097922	0.377586	-0.00746	0.945032	-0.03633	0.737887	-0.04378	0.687177	6869976	8.011353	7.871926	8.055056	8.142844	8.032014	7.872456	8.079478	8.15893	7.831279	7.951193	7.819631	8.005097	Lzts2	NM_145503
6869979	7430	8.886063	8.839315	8.791359	8.477995	0.408068	0.0161	0.361319	0.02748	0.313363	0.047913	0.047956	0.730267	0.046749	0.736753	0.094705	0.500715	6869979	8.783623	8.88538	8.989187	8.831182	9.035378	8.651384	8.923339	8.576281	8.874456	8.427506	8.656683	8.349817	Sfxn3	NM_053197
6869984	7432	5.972407	6.319144	6.109664	6.193169	-0.22076	0.052116	0.125975	0.229562	-0.08351	0.413661	0.20948	0.0625	-0.34674	0.007186	-0.13726	0.194163	6869984	5.834064	5.925215	6.157943	6.353459	6.405823	6.198149	6.140378	6.188308	6.000305	6.252474	6.230867	6.096166	Tlx1	NM_021901
6869997	7433	7.814162	8.074336	7.818741	7.976573	-0.16241	0.081745	0.097763	0.265199	-0.15783	0.089152	0.255595	0.01397	-0.26017	0.012837	-0.00458	0.956635	6869997	7.744768	7.810482	7.887237	8.12467	8.116502	7.981835	7.739527	7.81597	7.900725	7.931708	7.856088	8.141923	Btrc	NM_001037758
6870010	7434	7.842799	7.898333	7.77141	7.895432	-0.05263	0.623687	0.002901	0.978257	-0.12402	0.263657	0.126923	0.253522	-0.05553	0.605008	0.071389	0.508514	6870010	7.84453	7.841289	7.842579	7.854445	7.889024	7.95153	7.930072	7.872931	7.511228	7.914507	7.78807	7.98372	5330431N	NM_172639
6870024	7435	6.796081	6.853308	6.981722	6.725822	0.070259	0.463874	0.127486	0.200312	0.2559	0.023139	-0.12841	0.197382	-0.05723	0.548423	-0.18564	0.076567	6870024	6.840029	6.784559	6.763654	6.863671	7.030518	6.665734	6.957422	7.077606	6.910138	6.648098	6.708409	6.820959	Hps6	NM_176785
6870025	7436	6.995742	6.908909	7.018635	6.7942	0.201542	0.503784	0.114709	0.700758	0.224435	0.458128	-0.10973	0.713056	0.086833	0.770662	-0.02289	0.938579	6870025	7.071801	6.999034	6.91639	6.736506	7.303877	6.686343	6.800719	7.537717	6.717469	6.869519	7.163554	6.349526	Pprc1	NM_001081214
6870027	7437	9.202759	9.114481	9.270257	9.169833	0.032927	0.753056	-0.05535	0.599034	0.100424	0.34972	-0.15578	0.16199	0.088278	0.408081	-0.0675	0.523223	6870027	9.279561	9.109287	9.21943	8.988335	9.290014	9.065094	9.167796	9.359217	9.283757	9.19618	9.296267	9.017051	Nolc1	NM_053086
6870029	7439	8.799273	8.761224	8.944243	8.876403	-0.07713	0.592752	-0.11518	0.42965	0.06784	0.637337	-0.18302	0.222803	0.038049	0.790448	-0.14497	0.325723	6870029	8.833684	8.800687	8.763448	8.689229	8.97964	8.614804	8.819275	9.209929	8.803525	8.775813	9.053393	8.800003	Gbf1	NM_178930
6870063	7440	8.38036	8.27382	8.371669	7.628449	0.751911	0.005032	0.645371	0.011095	0.74322	0.00536	-0.09785	0.631783	0.10654	0.602324	0.008691	0.965793	6870063	8.619623	8.273167	8.24829	8.226577	8.539186	8.055697	8.361897	8.332663	8.420447	7.703001	7.940639	7.241708	Nfkb2	NM_019408
6870065	7441	6.01304	6.169211	6.080365	6.222763	-0.20972	0.09187	-0.05355	0.638037	-0.1424	0.229801	0.088846	0.440762	-0.15617	0.19177	-0.06733	0.555866	6870065	6.136653	5.88075	6.021716	6.231382	6.256006	6.020246	6.072803	6.199526	5.968766	6.39611	6.082479	6.1897	Fbxl15	NM_133694
6870068	7442	7.747435	7.724627	7.723432	7.632809	0.114626	0.334927	0.091818	0.434982	0.090623	0.440754	0.001195	0.991726	0.022807	0.843343	0.024003	0.835269	6870068	7.536331	7.829769	7.876204	7.754566	7.840743	7.578573	7.686491	7.704776	7.779029	7.510622	7.596552	7.791253	Tmem180	NM_029186
6870069	7443	6.155411	6.645778	6.195937	6.61652	-0.46111	0.019547	0.029258	0.858037	-0.42058	0.029006	0.449841	0.021803	-0.49037	0.014752	-0.04053	0.804501	6870069	6.001379	6.304434	6.160421	6.702923	6.599731	6.63468	6.220619	6.2672	6.099993	6.324279	6.531837	6.993445	Sfxn2	NM_053196
6870070	7444	7.66734	7.758555	7.766386	7.619202	0.048138	0.622568	0.139353	0.176676	0.147184	0.156202	-0.00783	0.935688	-0.09122	0.360493	-0.09905	0.323025	6870070	7.688022	7.707254	7.606745	7.826912	7.836568	7.612186	7.699707	7.879343	7.720107	7.438863	7.717289	7.701454	Sufu	NM_015752
6870093	7445	8.710793	8.569382	8.792512	8.64477	0.066022	0.734611	-0.07539	0.699017	0.147742	0.454748	-0.22313	0.269485	0.141411	0.47364	-0.08172	0.67539	6870093	8.879964	8.736326	8.516088	8.629827	8.703091	8.375228	8.626493	9.197332	8.553712	8.48875	8.642028	8.803533	Trim8	NM_053100
6870097	7446	9.013189	9.160901	8.910522	9.09204	-0.07885	0.471907	0.068861	0.528207	-0.18152	0.120415	0.250378	0.043359	-0.14771	0.194993	0.102667	0.354395	6870097	9.131475	8.904318	9.003775	9.247832	9.21233	9.02254	8.878011	9.09943	8.754126	9.044931	9.04114	9.190048	D19Wsu16	BC058949
6870102	7447	5.89111	6.149976	5.945468	5.913003	-0.02189	0.896185	0.236973	0.18298	0.032465	0.846674	0.204508	0.243812	-0.25887	0.14992	-0.05436	0.746661	6870102	5.605274	6.021193	6.046862	6.1721	6.077594	6.200233	6.147692	6.084892	5.60382	5.836187	5.953149	5.949672	2010012O	NM_025563
6870104	7448	8.100319	8.468714	8.330008	8.587919	-0.4876	0.035901	-0.1192	0.555221	-0.25791	0.219539	0.138706	0.49413	-0.36839	0.093581	-0.22969	0.269541	6870104	7.914069	8.278703	8.108186	8.293056	8.474955	8.638131	8.382223	8.450065	8.157735	8.35564	8.391055	9.017061	As3mt	NM_020577
6870107	7449	7.502958	7.462392	7.544222	7.406427	0.096531	0.288035	0.055965	0.527922	0.137795	0.142919	-0.08183	0.362933	0.040566	0.645268	-0.04126	0.639674	6870107	7.568229	7.51282	7.427824	7.460673	7.545017	7.381485	7.500376	7.627069	7.50522	7.567211	7.409323	7.242747	Cnnm2	NM_033569
6870130	7452	7.456052	7.436319	7.424697	7.41944	0.036613	0.600501	0.016879	0.807889	0.005258	0.939522	0.011622	0.866911	0.019733	0.776364	0.031355	0.653036	6870130	7.462319	7.474349	7.431489	7.369128	7.533569	7.40626	7.387457	7.54329	7.343345	7.355258	7.522317	7.380744	Pdcd11	NM_011053

6870134	7453	7.109731	6.876333	6.901118	6.894723	0.215008	0.092522	-0.01839	0.87428	0.006396	0.956084	-0.02479	0.831242	0.233398	0.071845	0.208612	0.100972	6870134	7.042047	7.005241	7.281904	7.029747	6.853297	6.745955	6.944584	7.02118	6.737591	6.773008	6.938577	6.972583	Neurl1a	NM_021360
6870166	7455	9.209409	9.228015	9.144759	8.961803	0.247606	0.05977	0.266212	0.046225	0.182946	0.144043	0.083265	0.482175	-0.01861	0.873279	0.06466	0.582829	6870166	9.259048	9.219422	9.149757	9.155098	9.309529	9.219417	9.338969	9.011312	9.083967	8.800262	9.17794	8.907207	Slk	NM_009289
6870172	7456	9.499849	9.611599	9.616646	9.475456	0.024393	0.83709	0.136143	0.269801	0.141189	0.2538	-0.00505	0.966022	-0.11175	0.35897	-0.1168	0.338867	6870172	9.389712	9.543228	9.566607	9.511601	9.774091	9.549105	9.835055	9.488353	9.526529	9.370333	9.600808	9.455228	6330577E	NM_026377
6870181	7457	6.582844	8.565753	6.582902	8.435159	-1.85232	0.039447	0.130594	0.866728	-1.85226	0.039452	1.982851	0.030126	-1.98291	0.030122	-5.8E-05	0.99994	6870181	6.800498	6.46567	6.482364	9.255506	9.291818	7.149935	6.952171	6.234653	6.561883	8.997887	9.379253	6.928337	Gsto1	NM_010362
6870375	7460	6.435472	6.350705	6.286685	6.259313	0.176159	0.498359	0.091393	0.722473	0.027372	0.91497	0.064021	0.803119	0.084766	0.741706	0.148787	0.565756	6870375	6.57797	6.363482	6.364963	6.881249	6.301281	5.869586	6.381084	5.981171	6.497799	6.39958	6.284688	6.09367	Ins1	NM_008386
6870424	7461	9.248503	9.594439	9.555249	10.10853	-0.86002	0.004021	-0.51409	0.044311	-0.55328	0.033383	0.03919	0.860346	-0.34594	0.147424	-0.30675	0.192787	6870424	9.299011	9.336576	9.109921	9.757052	9.349283	9.676981	9.475785	9.985062	9.2049	9.848446	10.13814	10.33899	Add3	NM_013758
6870429	7462	7.363443	7.632387	7.46485	7.517066	-0.15362	0.110781	0.115321	0.21528	-0.05222	0.559207	0.167537	0.086313	-0.26894	0.013838	-0.10141	0.270632	6870429	7.241237	7.38311	7.465982	7.719742	7.474668	7.702752	7.589014	7.393125	7.412412	7.506269	7.496559	7.548371	Mxi1	NM_001008542
6870470	7463	8.75306	8.888849	8.885034	8.740275	0.012785	0.889264	0.148574	0.133391	0.144759	0.142283	0.003815	0.966842	-0.13579	0.165365	-0.13197	0.176165	6870470	8.738756	8.781245	8.739178	8.721042	9.013043	8.932461	8.981099	8.930724	8.743279	8.635113	8.804266	8.781446	Smc3	NM_007790
6870489	7464	10.56185	10.56632	10.2924	10.76179	-0.19994	0.244208	-0.19547	0.25402	-0.46938	0.018397	0.273913	0.123352	-0.00447	0.978285	0.269447	0.128713	6870489	10.3144	10.77165	10.5995	10.46228	10.61119	10.62548	10.07769	10.33055	10.46897	10.60695	10.65769	11.02072	Pdcd4	NM_011050
6870497	7465	9.407527	9.437013	9.412182	9.374339	0.033188	0.810148	0.062675	0.651635	0.037843	0.784258	0.024831	0.857237	-0.02949	0.830919	-0.00465	0.97307	6870497	9.389746	9.363673	9.469162	9.402775	9.618952	9.289313	9.414193	9.606228	9.216125	9.28061	9.598635	9.243771	Shoc2	NM_019658
6870498	7466	7.647759	7.54405	7.29249	7.189051	0.458708	0.172757	0.354999	0.280045	0.103439	0.744366	0.25156	0.435433	0.103709	0.743727	0.355269	0.279706	6870498	8.258408	7.401502	7.283366	7.456916	7.862641	7.312593	7.301747	7.051553	7.524169	6.878638	7.610085	7.07843	Adra2a	NM_007417
6870566	7468	9.780037	9.775806	9.715908	9.571088	0.208948	0.202733	0.204718	0.211114	0.144819	0.364404	0.059898	0.701231	0.004231	0.978277	0.064129	0.681462	6870566	9.623999	9.86507	9.851041	9.548271	9.825596	9.953551	9.855911	9.45683	9.834982	9.39855	9.611825	9.70289	Acsl5	NM_027976
6870569	7469	8.811555	8.783658	8.800868	8.821677	-0.01012	0.928114	-0.03802	0.735587	-0.02081	0.85298	-0.01721	0.878139	0.027897	0.803958	0.010687	0.924111	6870569	8.733044	8.872983	8.828639	8.778433	8.811255	8.761286	8.839038	8.581638	8.981929	8.643077	8.920012	8.901941	Vti1a	NM_016862
6870580	7470	8.204907	8.415882	8.273229	7.918001	0.286906	0.172344	0.497881	0.031584	0.355228	0.100618	0.142653	0.4775	-0.21098	0.302495	-0.06832	0.73041	6870580	8.035702	8.249179	8.32984	8.435357	8.570091	8.242199	7.908327	8.54702	8.364341	7.824328	8.199037	7.730638	Tcf7l2	NM_001142918
6870583	7471	6.137506	5.968529	5.874484	5.944015	0.193492	0.370579	0.024514	0.907299	-0.06953	0.741967	0.094045	0.657021	0.168977	0.431438	0.263022	0.233247	6870583	6.1322	6.255023	6.025296	6.022926	6.123544	5.759117	5.429361	6.14269	6.051401	6.201485	5.839405	5.791154	Ppnr	X65588
6870614	7473	7.750354	7.933843	7.740785	7.571776	0.178578	0.420514	0.362066	0.123461	0.169008	0.444855	0.193058	0.385491	-0.18349	0.408392	0.009569	0.964825	6870614	7.706246	7.904378	7.640438	7.821295	8.065022	7.915211	8.098004	7.380866	7.743484	7.393811	7.37954	7.941978	Casp7	NM_007611
6870617	7475	7.898045	7.897188	7.897086	7.905779	-0.00773	0.910462	-0.00859	0.900603	-0.00869	0.899434	0.000102	0.99882	0.000857	0.990057	0.000958	0.988878	6870617	7.825732	7.853909	8.014493	7.85624	7.960197	7.875127	7.858315	8.014471	7.818473	7.889561	7.866162	7.961613	Nhlrc2	NM_025811
6870622	7476	6.34311	6.18122	6.438951	6.437427	-0.09432	0.718741	-0.25621	0.340442	0.001524	0.995338	-0.25773	0.337744	0.16189	0.539775	-0.09584	0.714432	6870622	6.107797	6.368986	6.552548	6.200123	6.435269	5.908269	6.863527	6.194051	6.259274	6.208529	6.848756	6.254996	Adrb1	NM_007419
6870675	7479	8.458982	8.600969	8.455961	8.478286	-0.0193	0.789025	0.122683	0.116704	-0.02232	0.75716	0.145008	0.071275	-0.14199	0.076234	0.003021	0.966517	6870675	8.428454	8.516514	8.431978	8.575415	8.67982	8.547671	8.469024	8.579531	8.319327	8.428319	8.557433	8.449105	Fam160b1	NM_145505
6870680	7480	6.797187	6.96214	6.721758	6.96315	-0.16596	0.089394	-0.00101	0.990905	-0.24139	0.022822	0.240382	0.023239	-0.16495	0.091031	0.075429	0.405415	6870680	6.881362	6.852221	6.657978	7.030969	6.894207	6.961244	6.672653	6.735539	6.757082	6.882541	6.869151	7.137758	Trub1	NM_028115
6870697	7481	8.385364	9.115247	8.486486	8.604202	-0.21884	0.396083	0.511045	0.069592	-0.11772	0.642496	0.628761	0.032807	-0.72988	0.017319	-0.10112	0.689519	6870697	7.89413	8.724702	8.53726	8.916609	9.336562	9.09257	8.532479	8.19286	8.734118	8.360255	8.665415	8.786936	Atrnl1	NM_181415
6870880	7492	6.619937	6.799318	6.816961	6.721226	-0.10129	0.348793	0.078091	0.464968	0.095734	0.374439	-0.01764	0.866686	-0.17938	0.116015	-0.19702	0.088924	6870880	6.527431	6.54085	6.79153	6.756119	6.784446	6.857388	6.71306	6.935214	6.802608	6.764389	6.546651	6.852639	Nanos1	NM_178421
6870891	7493	8.8891	8.617508	8.950838	8.751775	0.137325	0.262026	-0.13427	0.271967	0.199062	0.118383	-0.33333	0.019028	0.271592	0.044097	-0.06174	0.602262	6870891	8.929385	8.82369	8.914225	8.461347	8.838472	8.552704	9.135109	8.850562	8.866842	8.798374	8.821077	8.635875	1810055E	BC013514
6870897	7494	8.873165	9.293725	9.117828	9.682665	-0.8095	0.007391	-0.38894	0.125462	-0.56484	0.037835	0.175897	0.461326	-0.42056	0.101475	-0.24466	0.313197	6870897	8.633947	9.145884	8.839663	9.363945	9.189417	9.327812	8.994006	9.449995	8.909482	9.434571	9.483413	10.13001	Grk5	NM_018869
6870919	7495	6.839013	6.865109	6.860176	6.856832	-0.01782	0.75807	0.008277	0.88595	0.003344	0.95376	0.004933	0.931852	-0.0261	0.653068	-0.02116	0.714841	6870919	6.829261	6.961592	6.726187	6.888803	6.866297	6.840226	6.851039	6.841921	6.887568	6.909972	6.869379	6.791144	Ighmbp2	NM_009212
6870929	7497	9.363729	9.563903	9.399335	9.610047	-0.24632	0.017999	-0.04614	0.59374	-0.21071	0.03489	0.164568	0.082897	-0.20017	0.04252	-0.03561	0.679487	6870929	9.309214	9.401735	9.380238	9.533193	9.588535	9.569982	9.375868	9.432771	9.389366	9.496307	9.500558	9.833275	Saps3	NM_029456
6870931	7498	9.376386	9.311933	9.22423	9.379953	-0.00357	0.974475	-0.06802	0.546586	-0.15572	0.18752	0.087703	0.440476	0.064453	0.567351	0.152156	0.19675	6870931	9.426894	9.357156	9.345108	9.321115	9.414262	9.200423	9.36307	9.326433	8.983187	9.335936	9.295906	9.508017	Lrp5	NM_008513
6870956	7499	8.187732	8.204764	8.249252	7.668627	0.519105	0.004875	0.536138	0.00408	0.580626	0.002592	-0.04449	0.749885	-0.01703	0.902583	-0.06152	0.660291	6870956	8.305838	8.162176	8.095182	8.19526	8.386682	8.032351	8.224353	8.098167	8.425237	7.6092	7.888492	7.508188	Tcirg1	NM_016921
6870957	7500	10.3749	10.56069	10.45726	10.48008	-0.10518	0.409341	0.080617	0.523374	-0.02281	0.854929	0.10343	0.416844	-0.1858	0.16264	-0.08237	0.514639	6870957	10.27095	10.34283	10.51091	10.51497	10.58342	10.58369	10.64858	10.53159	10.19162	10.35406	10.49223	10.59394	Ndufs8	NM_144870
6870958	7501	7.642806	7.742392	7.358731	7.266021	0.376785	0.115608	0.476371	0.056193	0.09271	0.675597	0.383661	0.110064	-0.09959	0.653344	0.284074	0.220022	6870958	7.8901	7.490182	7.548135	7.790562	7.909434	7.52718	7.797825	6.939303	7.339066	7.234173	7.225214	7.338676	Aldh3b1	NM_026316
6870964	7502	5.705447	6.073255	5.943373	5.436937	0.26851	0.279997	0.636318	0.025219	0.506436	0.060351	0.129882	0.590502	-0.36781	0.151131	-0.23793	0.334594	6870964	5.348549	5.866724	5.901068	6.046554	6.42444	5.748771	6.079957	5.821342	5.928821	5.397517	5.150594	5.7627	4833408A	AK079024
6870967	7503	8.676754	8.842397	8.664352	8.794827	-0.11807	0.371492	0.04757	0.712822	-0.13047	0.326061	0.178045	0.191258	-0.16564	0.220769	0.012402	0.923237	6870967	8.527758	8.612038	8.890465	8.949971	8.773466	8.803753	8.754471	8.728454	8.510131	8.609778	8.81881	8.955893	Ndufv1	NM_133666
6870969	7504	11.1291	10.86014	10.97052	11.11041	0.01869	0.905194	-0.25026	0.138365	-0.13989	0.384431	-0.11038	0.488537	0.268953	0.114859	0.158577	0.32743	6870969	11.32084	11.01824	11.04821	10.78248	10.91399	10.88396	10.81878	11.29039	10.80239	10.92011	11.25222	11.15889	Gstp2	NM_181796
6870975	7506	8.158105	8.151631	8.181284	8.050063	0.108042	0.212206	0.101568	0.238254	0.131221	0.138255	-0.02965	0.719485	0.006474	0.937245	-0.02318	0.778561	6870975	8.11532	8.221182	8.137813	8.192266	8.211986	8.05064	8.239141	8.299632	8.005078	7.995432	8.04895	8.105806	Aip	NM_016666
6870978	7508	7.998829	7.860348	7.87589	7.949395	0.049435	0.740763	-0.08905	0.554363	-0.0735	0.624263	-0.01554	0.916891	0.138481	0.365364	0.122939	0.419045	6870978	8.075611	7.84758	8.073297	7.862599	8.002481	7.715965	7.614981	8.132536	7.880153	7.875629	8.113607	7.858948	Rps6kb2	NM_021485
6870980	7510	6.385698	6.446732	6.378656	6.311207	0.074491	0.345904	0.135525	0.10591	0.067449	0.390987	0.068076	0.386808	-0.06103	0.435645	0.007042	0.926902	6870980	6.460805	6.398551	6.297738	6.411613	6.530536	6.398048	6.357852	6.377461	6.400656	6.456325	6.308321	6.168975	Tbc1d10c	NM_178650
6870982	7511	8.251961	8.151391	8.264078	8.025382	0.226579	0.120504	0.126009	0.362202	0.238697	0.104572	-0.11269	0.412678	0.10057	0.462753	-0.01212	0.928252	6870982	8.167736	8.188001	8.400146	8.004636	8.37266	8.076877	8.256594	8.278858	8.256783	8.115793	8.183016	7.777336	Rad9	NM_011237
6870989	7512	8.262622	8.252818	8.274397	8.035572	0.22705	0.052667	0.217246	0.061379	0.238825	0.043817	-0.02158	0.834394	0.009804	0.924239	-0.01178	0.909081	6870989	8.263231	8.201676	8.32296	8.225036	8.4102	8.123219	8.328742	8.241091	8.253359	8.067546	8.198521	7.840649	Ssh3	NM_198113
6870991	7513	6.144852	6.210542	6.061056	5.958306	0.186545	0.00631	0.252235	0.001104	0.10275	0.077885	0.149485	0.018685	-0.06569	0.232316	0.083795	0.137855	6870991	6.176701	6.090094	6.16776	6.254704	6.224658	6.152263	6.073411	6.114621	5.995137	5.879491	6.043736	5.951692	Ankrd13d	NM_026720
6870993	7514	8.848937	8.86106	8.747713	8.548534	0.300402	0.020301	0.312526	0.016988	0.199179	0.091959	0.113347	0.307777	-0.01212	0.910125	0.101224	0.359183	6870993	8.903908	8.811694	8.831208	8.864592	8.985774	8.732815	8.79823	8.842393	8.602516	8.440262	8.749351	8.45599	Adrbk1	NM_130863
6870996	7515	8.954737	8.995863	8.888752	8.832589	0.122148	0.134818	0.163275	0.056884	0.056163	0.466357	0.107112	0.182799	-0.04113	0.590782	0.065985	0.395137	6870996	8.981477	8.955322	8.927412	8.904659	9.145778	8.937153	8.890448	8.996585	8.779222	8.821799	8.889026	8.786941	Kdm2a	NM_001001984
6871002	7516	7.932125	7.994743	7.881164	7.862864	0.069261	0.647908	0.131879	0.39277	0.0183	0.903347	0.113579	0.458999	-0.06262	0.679321	0.050961	0.736069	6871002	7.953362	7.81706	8.025952	7.960807	8.193003	7.83042	7.821523	8.082334	7.748928	7.61868	8.067722	7.90219	Rhod	NM_007485
6871009	7518	6.075853	5.990572	5.957836	6.026496	0.049357	0.730086	-0.03592	0.801371	-0.06866	0.632502	0.032736	0.81861	0.085281	0.554126	0.118017	0.417725	6871009	6.061496	6.019823	6.146239	5.918061	6.016248	6.037407	5.8476	6.094323	5.931584	6.240464	6.156568	5.682455	2010003K	BC021951
6871019	7519	7.289715	7.245048	7.144649	7.047757	0.241958	0.07614	0.197291	0.135459	0.096892	0.438481	0.1004	0.42274	0.044666	0.716791	0.145066	0.256951	6871019	7.386215	7.220829	7.2621	7.267514	7.349611	7.11802	7.298853	7.159814	6.975279	7.014909	6.873446	7.254916	Lrfn4	NM_153388
6871021	7520	7.163544	7.229717	7.181209	7.02559	0.137954	0.13143	0.204127	0.037749	0.155619	0.094645	0.048508	0.570964	-0.06617	0.44357	-0.01766	0.835038	6871021	7.181724	7.133156	7.175752	7.108406	7.415794	7.164951	7.300293	7.124648	7.118685	7.058231	7.048454	6.970084	Rce1	NM_023131
6871044	7521	6.972453	6.81499	6.81645	6.905838	0.066615	0.520422	-0.09085	0.386117	-0.08939	0.393435	-0.00146	0.988607	0.157463	0.150764	0.156003	0.154114	6871044	6.864369	7.144396	6.908593	6.904482	6.800229	6.740258	6.848948	6.955083	6.645318	6.967446	6.916718	6.833349	Rbm4	NM_009032
6871051	7522	8.081891	7.98394	8.020546	8.124867	-0.04298	0.686448	-0.14093	0.206997	-0.10432	0.339198	-0.03661	0.730584	0.097951	0.367865	0.061346	0.566585	6871051	8.068975	8.091713	8.084986	7.947788	8.072978	7.931054	7.810025	8.243594	8.008018	8.112494	8.230198	8.031909	Rbm14	NM_019869
6871056	7523	7.61088	7.838187	7.617715	7.833433	-0.22255	0.087564	0.004754	0.967865	-0.21572	0.096015	0.220471	0.090058	-0.22731	0.082112	-0.00684	0.953812	6871056	7.493894	7.575697	7.763049	7.894127	7.714656	7.905777	7.663098	7.660015	7.530033	7.786456	7.655545	8.058297	Ccs	NM_016892
6871057	7524	8.098008	7.511565	7.567173	7.974717	0.123291	0.922532	-0.46315	0.715985	-0.40754	0.748619	-0.05561	0.965007	0.586443	0.645886	0.530835	0.677092	6871057	6.236442	7.616452	10.44113	8.297225	7.971227	6.266242	8.672585	7.205542	6.823392	6.467381	7.943777	9.512993	Actn3	NM_013456
6871058	7525	8.957787	8.926351	9.031774	8.852881	0.104906	0.474305	0.07347	0.613299	0.178892	0.236336	-0.10542	0.472203	0.031436	0.827645	-0.07399	0.610847	6871058	8.954031	8.92265	8.99668	8.907718	9.136477	8.734859	9.223457	8.841986	9.029878	8.633146	9.013682	8.911816	Bbs1	NM_001033128
6871060	7526	6.830942	7.059282	6.878343	7.286322	-0.45538	0.031616	-0.22704	0.231011	-0.40798	0.048207	0.180939	0.331779	-0.22834	0.228587	-0.0474	0.793517	6871060	6.78736	6.863413	6.842052	7.265297	6.828554	7.083995	6.854044	7.230428	6.550557	7.41072	7.141884	7.306363	Peli3	NM_172835
6871077	7530	10.4402	10.26546	10.54046	10.51815	-0.07794	0.421502	-0.25269	0.02519	0.022313	0.814468	-0.275	0.017357	0.174747	0.094054	-0.10026	0.307562	6871077	10.45722	10.39241	10.47098	10.28045	10.46377	10.05215	10.55329	10.56207	10.50602	10.44131	10.52249	10.59064	Rab1b	NM_029576
6871078	7531	7.106404	7.045479	7.24765	7.125969	-0.01956	0.857511	-0.08049	0.467453	0.121681	0.282104	-0.20217	0.091668	0.060925	0.57954	-0.14125	0.217474	6871078	7.115694	7.066992	7.136526	7.11064	7.144386	6.881411	7.218873	7.440012	7.084064	7.012729	7.238603	7.126574	Klc2	NM_008451
6871085	7532	7.659761	7.685957	7.605482	7.506656	0.153105	0.113436	0.179301	0.070976	0.098826	0.284472	0.080475	0.377574	-0.0262	0.768833	0.054279	0.546231	6871085	7.704308	7.724047	7.550928	7.730555	7.800161	7.527154	7.601369	7.707909	7.507168	7.426855	7.570265	7.522848	Pacs1	NM_153129
6871091	7533	8.425014	8.595053	8.481449	8.410757	0.014257	0.841365	0.184296	0.028244	0.070692	0.335271	0.113604	0.13807	-0.17004	0.038957	-0.05644	0.436808	6871091	8.443005	8.503448	8.328589	8.586102	8.68361	8.515448	8.543814	8.538973	8.361561	8.353082	8.424359	8.45483	Sf3b2	NM_030109
6871095	7534	7.180988	6.687795	7.143046	6.629538	0.55145	0.044549	0.058257	0.807822	0.513508	0.057521	-0.45525	0.085027	0.493193	0.065944	0.037941	0.873991	6871095	7.426234	6.7729	7.343829	6.923591	6.535028	6.604766	7.442512	7.077984	6.908643	6.346274	6.636996	6.905344	Cst6	NM_028623
6871097	7535	9.816732	9.652468	9.790851	9.647429	0.169303	0.316766	0.00504	0.975422	0.143422	0.392115	-0.13838	0.408209	0.164264	0.330489	0.025881	0.874383	6871097	9.754746	9.721642	9.973808	9.620498	9.925591	9.411316	9.945327	9.800562	9.626664	9.417332	9.763713	9.761241	Banf1	NM_001038231
6871102	7536	7.598331	7.630872	7.613673	7.581598	0.016733	0.770941	0.049273	0.400987	0.032075	0.579579	0.017199	0.764788	-0.03254	0.574199	-0.01534	0.789428	6871102	7.623702	7.584663	7.586629	7.592441	7.736697	7.563477	7.653522	7.659107	7.52839	7.509073	7.622024	7.613698	Sart1	NM_016882
6871109	7537	10.40919	10.47027	10.4297	10.30492	0.10427	0.45916	0.165353	0.252508	0.12478	0.379287	0.040573	0.769919	-0.06108	0.66082	-0.02051	0.882216	6871109	10.46053	10.37021	10.39683	10.53801	10.4581	10.41471	10.59586	10.59052	10.10272	10.20733	10.23342	10.47401	Drap1	NM_024176
6871114	7538	8.117092	8.320032	8.063385	8.213113	-0.09602	0.33307	0.106919	0.284499	-0.14973	0.146864	0.256647	0.024927	-0.20294	0.061126	0.053707	0.58033	6871114	8.232055	8.079874	8.039346	8.310745	8.387204	8.262147	8.070275	8.243625	7.876254	8.218243	8.147021	8.274076	Ccdc85b	NM_198616
6871117	7539	6.341069	6.23279	6.415035	5.453653	0.887417	0.005971	0.779138	0.0116	0.961382	0.003855	-0.18224	0.46818	0.108279	0.662947	-0.07397	0.765155	6871117	6.346991	6.401048	6.275169	5.927362	6.609311	6.161698	6.720721	6.148474	6.375909	5.233821	5.880275	5.246862	Ctsw	NM_009985

6871119	7540	6.812244	6.697892	6.796322	6.733457	0.078787	0.44165	-0.03557	0.724277	0.062864	0.536436	-0.09843	0.341479	0.114352	0.273822	0.015922	0.874111	6871119	6.77124	6.850064	6.815428	6.626025	6.81745	6.6502	6.828448	6.987543	6.572974	6.741385	6.704525	6.754462	Mus81	NM_027877
6871121	7541	6.873835	7.065293	6.838366	6.895213	-0.02138	0.837216	0.17008	0.129763	-0.05685	0.587948	0.226927	0.054308	-0.19146	0.093838	0.035469	0.733821	6871121	7.003721	6.864319	6.753465	7.179475	7.135653	6.880752	6.760543	6.991021	6.763535	6.940911	6.863749	6.88098	Snx32	NM_001024560
6871122	7542	6.211336	6.229986	6.230481	6.216697	-0.00536	0.963	0.013289	0.908477	0.013785	0.905085	-0.0005	0.99658	-0.01865	0.871886	-0.01915	0.86852	6871122	6.286794	6.263342	6.083872	6.226863	6.330122	6.132973	6.314507	6.030881	6.346056	6.378244	6.076592	6.195254	1700020D	NM_023781
6871125	7543	6.550369	6.305993	6.594167	6.666104	-0.11573	0.565026	-0.36011	0.098812	-0.07194	0.718821	-0.28817	0.173452	0.244376	0.240727	-0.0438	0.826034	6871125	6.609114	6.52535	6.516644	6.290264	6.437214	6.190502	6.700739	6.85783	6.223932	6.481112	7.024101	6.493098	Ovol1	NM_019935
6871128	7544	8.158968	8.294775	8.258011	8.229373	-0.0704	0.406082	0.065402	0.438863	0.028637	0.730544	0.036765	0.659186	-0.13581	0.129191	-0.09904	0.252351	6871128	8.123478	8.194146	8.159281	8.333682	8.318533	8.232111	8.40815	8.171376	8.194506	8.121223	8.190656	8.376241	Kat5	NM_178637
6871134	7545	8.361617	8.383019	8.400339	8.197565	0.164052	0.145748	0.185454	0.10598	0.202774	0.081545	-0.01732	0.869134	-0.0214	0.838746	-0.03872	0.713583	6871134	8.402847	8.363116	8.318888	8.30162	8.596745	8.250692	8.294633	8.463136	8.443247	8.105399	8.34691	8.140386	Sipa1	NM_011379
6871136	7546	8.831737	8.804677	8.733937	8.636523	0.195214	0.133672	0.168154	0.188477	0.097414	0.429093	0.07074	0.562076	0.02706	0.82285	0.0978	0.427343	6871136	8.911211	8.819061	8.764938	8.819919	8.967956	8.626155	8.627674	8.958468	8.615669	8.608223	8.744416	8.556929	Pcnxl3	NM_144868
6871138	7547	7.097264	6.961859	6.981597	6.804331	0.292933	0.025541	0.157528	0.179179	0.177266	0.136175	-0.01974	0.858238	0.135406	0.241316	0.115668	0.311205	6871138	7.087364	6.977928	7.226501	7.02142	7.02652	6.837636	7.141679	6.902641	6.90047	6.639928	6.932382	6.840683	Ehbp1l1	NM_001114597
6871139	7548	8.353935	8.352228	8.261914	8.322672	0.031263	0.835816	0.029555	0.844642	-0.06076	0.688257	0.090314	0.553415	0.001707	0.990957	0.092021	0.546115	6871139	8.273886	8.265106	8.522813	8.39716	8.547567	8.111956	8.208618	8.520827	8.056296	8.278417	8.342595	8.347005	Sssca1	NM_020491
6871144	7549	9.298587	9.268102	9.285437	9.481158	-0.18257	0.049592	-0.21306	0.027193	-0.19572	0.038247	-0.01733	0.8318	0.030484	0.70962	0.01315	0.871916	6871144	9.304612	9.304165	9.286983	9.319265	9.278833	9.206209	9.290486	9.42737	9.138454	9.377328	9.461776	9.604369	Scyl1	NM_023912
6871156	7550	8.384485	8.29308	8.314817	8.02122	0.363265	0.001723	0.27186	0.008655	0.293598	0.005794	-0.02174	0.789465	0.091405	0.279109	0.069667	0.402024	6871156	8.401942	8.27523	8.476282	8.331829	8.4083	8.13911	8.390945	8.261058	8.292449	7.977818	8.083021	8.00282	Frmd8	NM_026169
6871161	7552	9.669925	9.67199	9.638233	9.645988	0.023937	0.732524	0.026002	0.710642	-0.00776	0.911529	0.033757	0.631106	-0.00206	0.976393	0.031693	0.651842	6871161	9.62711	9.677473	9.705193	9.655188	9.804538	9.556244	9.586431	9.750996	9.577271	9.616277	9.655243	9.666444	Dpf2	NM_011262
6871163	7553	6.781131	6.851749	6.602624	6.740534	0.040597	0.8192	0.111216	0.535682	-0.13791	0.445484	0.249126	0.185228	-0.07062	0.691936	0.178507	0.329339	6871163	6.802891	6.678354	6.862148	6.678645	7.262004	6.614599	6.712088	6.694862	6.400921	6.627287	6.828174	6.76614	Cdc42ep2	NM_026772
6871164	7554	6.753646	6.83067	6.789606	6.609084	0.144562	0.203248	0.221586	0.066413	0.180522	0.121815	0.041064	0.70415	-0.07702	0.481429	-0.03596	0.739219	6871164	6.685393	6.725932	6.849614	6.710909	7.034862	6.746238	6.853786	6.645117	6.869914	6.497393	6.701307	6.628552	Pola2	NM_008893
6871168	7556	9.673614	9.59748	9.717956	9.555482	0.118132	0.224931	0.041998	0.652595	0.162474	0.108108	-0.12048	0.216697	0.076134	0.421327	-0.04434	0.634838	6871168	9.608534	9.626868	9.785439	9.665763	9.72412	9.402556	9.737058	9.747703	9.669106	9.464554	9.56111	9.640782	Capn1	NM_001110504
6871177	7557	8.299001	8.274659	8.339402	8.368836	-0.06984	0.451576	-0.09418	0.317048	-0.02943	0.747313	-0.06474	0.484121	0.024342	0.789676	-0.0404	0.659273	6871177	8.184263	8.331135	8.381604	8.181575	8.434022	8.20838	8.404438	8.367435	8.246334	8.301335	8.320509	8.484665	Mrpl49	NM_026246
6871181	7558	7.098907	7.053391	7.021324	7.089814	0.009092	0.912613	-0.03642	0.662087	-0.06849	0.41837	0.032068	0.700004	0.045515	0.58628	0.077583	0.362126	6871181	7.10223	7.067689	7.126801	7.040077	7.164214	6.955883	7.069404	7.123977	6.87059	7.025211	7.045397	7.198835	Tm7sf2	NM_028454
6871189	7559	7.82367	7.793474	7.903852	7.877983	-0.05431	0.569005	-0.08451	0.382491	0.025868	0.784474	-0.11038	0.261948	0.030196	0.749754	-0.08018	0.406194	6871189	7.75102	7.814648	7.905341	7.823655	7.924633	7.632133	7.786522	8.040178	7.884855	7.845528	7.964588	7.823834	Zfpl1	NM_024231
6871192	7560	6.780006	6.841796	6.789301	6.774585	0.005421	0.966923	0.067211	0.610211	0.014716	0.910406	0.052496	0.689538	-0.06179	0.638876	-0.00929	0.943325	6871192	6.818646	6.707328	6.814044	6.669194	7.015942	6.840253	6.804053	6.898338	6.665511	6.86117	6.938573	6.524012	Sac3d1	NM_133678
6871194	7561	7.704108	7.658476	7.759486	7.867457	-0.16335	0.171116	-0.20898	0.090622	-0.10797	0.34943	-0.10101	0.37971	0.045632	0.68553	-0.05538	0.62401	6871194	7.637518	7.634836	7.83997	7.682678	7.86203	7.430719	7.739234	7.838785	7.70044	7.806824	7.944471	7.851075	Snx15	NM_026912
6871199	7562	9.162732	9.018898	9.1708	9.443341	-0.28061	0.132563	-0.42444	0.035101	-0.27254	0.142524	-0.1519	0.391178	0.143834	0.415693	-0.00807	0.962782	6871199	9.026659	9.242447	9.21909	9.11871	9.020597	8.917388	9.123779	9.430883	8.957737	9.441316	9.149895	9.738812	Arl2	NM_019722
6871206	7563	8.38344	8.267789	8.172771	8.061944	0.321496	0.108061	0.205845	0.280175	0.110827	0.550256	0.095017	0.607436	0.115651	0.533457	0.210668	0.269884	6871206	8.639163	8.126319	8.384837	8.283318	8.503796	8.016252	8.286592	8.287997	7.943725	7.881433	8.171412	8.132987	Ppp2r5b	NM_198168
6871240	7564	6.772428	6.777214	6.693839	6.728034	0.044394	0.65148	0.04918	0.617321	-0.0342	0.727179	0.083376	0.403955	-0.00479	0.960902	0.07859	0.430328	6871240	6.874823	6.698036	6.744426	6.880651	6.755537	6.695455	6.77898	6.784226	6.51831	6.859924	6.646735	6.677443	Slc22a12	NM_009203
6871254	7565	7.490657	7.502108	7.488215	7.351274	0.139384	0.197846	0.150834	0.167086	0.136941	0.205018	0.013893	0.892145	-0.01145	0.911001	0.002442	0.980972	6871254	7.566045	7.389784	7.516143	7.468931	7.675622	7.36177	7.620718	7.456722	7.387205	7.240438	7.449302	7.364081	Rps6ka4	NM_019924
6871258	7567	8.881849	9.018776	8.914353	9.264129	-0.38228	0.057599	-0.24535	0.192865	-0.34978	0.077218	0.104423	0.561856	-0.13693	0.450377	-0.0325	0.855281	6871258	8.883546	8.787436	8.974564	9.106062	8.968395	8.98187	9.015709	9.137445	8.589905	9.103655	9.095012	9.59372	Prdx5	NM_012021
6871259	7568	6.700288	6.739671	6.730761	6.943961	-0.24367	0.113999	-0.20429	0.175267	-0.2132	0.159239	0.00891	0.949873	-0.03938	0.781622	-0.03047	0.829996	6871259	6.656693	6.70397	6.740202	6.851589	6.596888	6.770536	6.770421	6.911774	6.510088	6.688385	7.008368	7.135131	Esrra	NM_007953
6871264	7570	6.124681	6.108944	6.150429	6.205344	-0.08066	0.098774	-0.0964	0.056127	-0.05492	0.239286	-0.04148	0.364938	0.015737	0.725034	-0.02575	0.567571	6871264	6.140291	6.071385	6.162368	6.14824	6.130214	6.048379	6.139768	6.16506	6.146458	6.28652	6.133223	6.196289	Kcnk4	NM_008431
6871265	7571	7.558451	7.513222	7.550184	7.396881	0.161569	0.062948	0.116341	0.158748	0.153303	0.074746	-0.03696	0.634739	0.045228	0.562451	0.008266	0.91479	6871265	7.630159	7.488825	7.556368	7.520374	7.594125	7.425168	7.551542	7.69536	7.403651	7.40548	7.38168	7.403484	Gpr137	NM_207220
6871267	7572	8.489939	8.492737	8.553234	8.27968	0.210258	0.121265	0.213057	0.117085	0.273554	0.054131	-0.0605	0.63139	-0.0028	0.98216	-0.0633	0.615944	6871267	8.532502	8.470078	8.467236	8.540061	8.592082	8.346068	8.568656	8.663006	8.428041	8.180944	8.550006	8.108091	Plcb3	NM_008874
6871270	7573	10.46649	10.45876	10.40547	10.71656	-0.25007	0.271262	-0.2578	0.257853	-0.31109	0.179754	0.053292	0.807522	0.007727	0.971769	0.061019	0.780415	6871270	10.53145	10.45921	10.40881	10.31743	10.20794	10.85092	10.66138	10.58777	9.967263	10.67805	10.72704	10.74459	Fkbp2	NM_008020
6871273	7574	9.212752	9.248068	9.333881	9.623346	-0.41059	0.014531	-0.37528	0.021848	-0.28946	0.059943	-0.08581	0.534418	-0.03532	0.796104	-0.12113	0.386284	6871273	9.146721	9.243876	9.24766	9.304661	9.178892	9.260651	9.179129	9.663131	9.159384	9.609545	9.50394	9.756552	Vegfb	NM_011697
6871274	7575	8.920625	9.034813	9.01324	9.12414	-0.20352	0.076941	-0.08933	0.399085	-0.1109	0.300951	0.021573	0.835062	-0.11419	0.287817	-0.09262	0.382764	6871274	8.774724	8.962815	9.024335	8.998748	9.05297	9.052721	9.058184	9.075998	8.905537	9.070874	8.973142	9.328404	Dnajc4	NM_020566
6871275	7576	7.380296	7.385555	7.531025	7.415123	-0.03483	0.736654	-0.02957	0.775025	0.115902	0.279924	-0.14547	0.18388	-0.00526	0.959358	-0.15073	0.170208	6871275	7.256135	7.354298	7.530456	7.503878	7.405789	7.246997	7.473243	7.422718	7.697115	7.465243	7.416114	7.364013	Nudt22	NM_026675
6871276	7577	7.07157	6.924544	7.132007	6.545295	0.526275	0.051385	0.379249	0.137726	0.586712	0.034105	-0.20746	0.393328	0.147026	0.540473	-0.06044	0.799338	6871276	7.372113	6.983294	6.859303	6.967184	7.174416	6.632032	7.350559	6.765458	7.280005	6.359827	6.845383	6.430675	Fermt3	NM_153795
6871277	7578	9.537352	9.581698	9.723633	9.534088	0.003265	0.977622	0.04761	0.684116	0.189545	0.131431	-0.14194	0.243812	-0.04435	0.70452	-0.18628	0.137292	6871277	9.570266	9.465953	9.575838	9.49089	9.807252	9.446951	9.782587	9.813843	9.574469	9.385138	9.626395	9.59073	Stip1	NM_016737
6871283	7579	8.266585	8.252684	8.246104	8.298969	-0.03238	0.868079	-0.04628	0.812524	-0.05286	0.786594	0.00658	0.973054	0.013901	0.94312	0.020481	0.916295	6871283	8.318948	8.389002	8.091804	8.263858	8.201658	8.292536	7.898697	8.670666	8.168949	8.274053	8.494992	8.127862	Flrt1	NM_201411
6871295	7580	9.654729	9.594255	9.594612	9.659773	-0.00504	0.94817	-0.06552	0.408953	-0.06516	0.411402	-0.00036	0.996325	0.060475	0.444482	0.060117	0.447077	6871295	9.67545	9.685635	9.603103	9.613868	9.697001	9.471895	9.683413	9.514459	9.585964	9.547606	9.667763	9.763949	Otub1	NM_134150
6871297	7581	7.01492	7.229552	7.181845	7.049133	-0.03421	0.751962	0.180419	0.12279	0.132713	0.240129	0.047706	0.660408	-0.21463	0.074249	-0.16693	0.149135	6871297	7.045035	7.032081	6.967643	7.187101	7.405046	7.096508	7.28158	7.258779	7.005177	6.907425	7.107395	7.132578	Nat11	BC020020
6871298	7582	9.030417	8.973392	9.138658	9.004308	0.026109	0.858491	-0.03092	0.832858	0.13435	0.371117	-0.16527	0.277369	0.057025	0.698089	-0.10824	0.467151	6871298	9.077411	9.058407	8.955432	9.022342	9.100928	8.796905	8.933825	9.46776	9.014389	8.917982	8.992918	9.102024	Mark2	NM_007928
6871314	7583	10.48668	10.41961	10.54611	10.54044	-0.05375	0.562775	-0.12083	0.211837	0.005677	0.950731	-0.1265	0.193181	0.067073	0.472866	-0.05943	0.523275	6871314	10.49681	10.41194	10.5513	10.32997	10.56481	10.36405	10.69411	10.52289	10.42134	10.44168	10.60451	10.57512	Rtn3	NM_001003934
6871325	7584	6.704339	6.790408	6.345668	6.769232	-0.06489	0.826318	0.021176	0.942836	-0.42356	0.177161	0.444739	0.158812	-0.08607	0.771294	0.358671	0.245522	6871325	6.225968	6.598852	7.288198	6.597606	7.055216	6.718401	6.205251	6.393092	6.438662	7.007154	6.945318	6.355223	Lgals12	NM_019516
6871332	7585	5.88109	5.841328	6.042139	7.887091	-2.006	0.000196	-2.04576	0.000171	-1.84495	0.000345	-0.20081	0.535824	0.039762	0.901241	-0.16105	0.617946	6871332	5.405944	6.293746	5.94358	5.614155	5.760458	6.149371	6.439316	6.264778	5.422323	7.82198	7.866937	7.972355	Slc22a9	NM_144785
6871361	7586	11.44453	11.59014	11.56308	11.67473	-0.23021	0.097178	-0.0846	0.509595	-0.11166	0.388936	0.02706	0.830799	-0.14561	0.268911	-0.11855	0.361766	6871361	11.41595	11.65552	11.26211	11.45819	11.52441	11.78781	11.56753	11.48806	11.63364	11.69895	11.54147	11.78378	Slc3a2	NM_008577
6871372	7587	9.35059	9.377465	9.592735	9.561882	-0.21129	0.096049	-0.18442	0.138399	0.030853	0.790014	-0.21527	0.090936	-0.02687	0.816471	-0.24214	0.062678	6871372	9.303904	9.377123	9.370744	9.327682	9.559249	9.245465	9.613914	9.748233	9.416058	9.517961	9.450285	9.717399	Tmem179b	NM_026325
6871374	7588	7.05875	7.170467	7.253201	7.177219	-0.11847	0.159134	-0.00675	0.931659	0.075982	0.348534	-0.08273	0.309849	-0.11172	0.181336	-0.19445	0.034266	6871374	6.9454	7.137877	7.092974	7.1862	7.295652	7.029548	7.273052	7.296231	7.19032	7.105986	7.214103	7.211569	Taf6l	NM_146092
6871377	7589	8.285251	8.51925	8.313415	8.359402	-0.07415	0.568605	0.159848	0.235887	-0.04599	0.721921	0.205835	0.137496	-0.234	0.097492	-0.02816	0.827014	6871377	8.249586	8.175901	8.430266	8.396653	8.636438	8.524659	8.435093	8.42522	8.079933	8.25684	8.296587	8.524779	Polr2g	NM_026329
6871380	7590	8.946858	8.935329	8.986235	8.947713	-0.00086	0.995081	-0.01238	0.928894	0.038521	0.781825	-0.05091	0.714893	0.011529	0.933791	-0.03938	0.777132	6871380	8.747274	9.01998	9.07332	8.764767	9.076406	8.964814	9.14308	8.735517	9.080107	8.882335	9.021148	8.939657	Ttc9c	NM_027412
6871393	7592	8.921087	9.069815	8.881679	9.05219	-0.1311	0.056518	0.017625	0.77223	-0.17051	0.019982	0.188136	0.012679	-0.14873	0.035424	0.039408	0.521997	6871393	8.880703	8.931315	8.951242	9.032587	9.029347	9.14751	8.940096	8.823276	8.881665	9.068431	8.93766	9.150479	AI462493	AY941793
6871425	7594	3.401795	6.173855	3.373856	6.085206	-2.68341	0.170736	0.088649	0.961565	-2.71135	0.16683	2.799999	0.154962	-2.77206	0.158617	0.027938	0.987882	6871425	3.072877	3.424267	3.70824	9.595763	3.869275	5.056527	3.187108	4.157409	2.777052	7.546992	8.135176	2.57345	Scgb1a1	NM_011681
6871429	7595	7.067187	7.269758	6.997772	7.18163	-0.11444	0.552424	0.088129	0.645751	-0.18386	0.348295	0.271987	0.178759	-0.20257	0.304285	0.069415	0.716592	6871429	6.987002	6.949345	7.265214	7.520358	7.228675	7.060242	7.014166	7.216198	6.762951	7.265315	6.887647	7.391927	Asrgl1	NM_025610
6871457	7596	6.719269	6.763224	6.681434	6.372692	0.346576	0.021493	0.390532	0.012406	0.308742	0.034792	0.08179	0.520222	-0.04396	0.727156	0.037835	0.763691	6871457	6.865644	6.700738	6.591424	6.630979	6.958082	6.700611	6.800573	6.474529	6.7692	6.386878	6.452223	6.278976	Incenp	NM_016692
6871471	7598	7.852197	8.356553	7.491238	7.226959	0.625238	0.054715	1.129594	0.003624	0.264278	0.369778	0.865315	0.014412	-0.50436	0.107299	0.36096	0.230471	6871471	8.163994	7.570226	7.822372	7.996354	8.977774	8.095531	7.678659	7.243534	7.55152	7.026544	7.271099	7.383235	Fads2	NM_019699
6871476	7599	6.200291	6.221781	6.183563	5.927097	0.273194	0.211809	0.294684	0.1814	0.256466	0.238438	0.038218	0.85416	-0.02149	0.917614	0.016727	0.935821	6871476	6.196085	6.046529	6.358258	6.118239	6.63858	5.908525	6.475647	5.954402	6.120641	5.838619	5.942965	5.999707	Fen1	NM_007999
6871479	7600	6.99689	6.981277	6.894553	7.207661	-0.21077	0.207324	-0.22638	0.178827	-0.31311	0.075904	0.086724	0.58789	0.015613	0.921554	0.102337	0.524072	6871479	6.917104	7.01591	7.057657	6.972783	7.119341	6.851708	6.67462	7.110042	6.898997	6.904188	7.31513	7.403664	Dagla	NM_198114
6871490	7602	7.261596	7.282237	7.230624	7.228136	0.03346	0.629578	0.054101	0.44097	0.002488	0.971168	0.051612	0.461501	-0.02064	0.76499	0.030972	0.65492	6871490	7.161204	7.329799	7.293786	7.259604	7.318847	7.268259	7.236716	7.271394	7.183763	7.096929	7.23941	7.348069	0610038F	NM_025333
6871493	7603	6.743447	6.783939	7.051499	6.897378	-0.15393	0.152131	-0.11344	0.277038	0.154121	0.151686	-0.26756	0.025014	-0.04049	0.688097	-0.30805	0.013244	6871493	6.762889	6.713311	6.754142	6.621399	6.899162	6.831257	7.160655	7.065567	6.928275	6.798776	7.065598	6.827759	Tmem216	NM_026798
6871496	7604	6.083458	6.126036	6.102038	5.990898	0.09256	0.492211	0.135138	0.324082	0.11114	0.412668	0.023998	0.856626	-0.04258	0.749106	-0.01858	0.888705	6871496	6.011083	6.176204	6.063087	5.999309	6.383893	5.994907	6.140738	6.264818	5.900559	5.901134	6.079487	5.992074	Tmem138	NM_028411
6871499	7605	6.994957	7.104083	7.101459	7.10684	-0.11188	0.061206	-0.00276	0.958545	-0.00538	0.919212	0.002624	0.960543	-0.10913	0.066539	-0.1065	0.072037	6871499	7.047177	7.002111	6.935583	7.106334	7.122192	7.083722	7.011232	7.197142	7.096002	7.175145	7.085679	7.059695	Dak	NM_145496
6871509	7607	6.176856	6.085022	6.150845	6.091581	0.085275	0.265164	-0.00656	0.928842	0.059264	0.429194	-0.06582	0.382114	0.091834	0.233003	0.026011	0.72425	6871509	6.256083	6.127091	6.147394	6.048848	6.138807	6.067412	6.159309	6.091866	6.201361	6.104958	6.22682	5.942966	Cd5	NM_007650
6871514	7609	7.349159	7.463026	7.214026	7.196831	0.152328	0.087819	0.266195	0.009403	0.017195	0.831815	0.249	0.013046	-0.11387	0.184274	0.135133	0.122912	6871514	7.352957	7.313728	7.380793	7.412919	7.592722	7.383438	7.326372	7.235422	7.080284	7.118802	7.289012	7.18268	Tmem132a	NM_133804
6871515	7610	8.540519	8.490327	8.367204	8.359104	0.181415	0.401871	0.131224	0.539882	0.0081	0.969442	0.123124	0.56462	0.050191	0.812695	0.173315	0.422304	6871515	8.476068	8.57429	8.571198	8.479575	8.424456	8.566951	8.643511	8.306057	8.152043	8.176822	8.844729	8.05576	Tmem109	NM_134142

6871519	7612	6.729945	6.569558	6.664621	6.648923	0.081023	0.353382	-0.07936	0.36279	0.015698	0.853371	-0.09506	0.281067	0.160387	0.086961	0.065324	0.449931	6871519	6.64757	6.614002	6.928264	6.63991	6.52698	6.541785	6.726166	6.674818	6.592879	6.698992	6.649082	6.598694	Ccdc86	NM_023731
6871545	7618	7.977037	8.137636	8.128585	6.978051	0.998986	0.010103	1.159585	0.00463	1.150535	0.004833	0.009051	0.976541	-0.1606	0.60501	-0.15155	0.625176	6871545	8.173364	7.84022	7.917527	8.178669	8.454988	7.779251	8.346242	7.672512	8.367002	6.732202	7.533143	6.668807	Ms4a7	NM_027836
6871566	7619	7.706181	7.701373	7.713072	6.489229	1.216952	0.031268	1.212145	0.031773	1.223843	0.030559	-0.0117	0.980619	0.004807	0.992035	-0.00689	0.988583	6871566	7.997886	7.738098	7.382558	7.518803	8.100355	7.484962	8.196939	7.301633	7.640643	6.920459	7.138701	5.408526	Ms4a6d	NM_026835
6871580	7623	8.305717	8.225811	8.373966	7.992345	0.313372	0.000943	0.233466	0.005302	0.381621	0.00026	-0.14815	0.042779	0.079906	0.230581	-0.06825	0.299931	6871580	8.335003	8.211517	8.370632	8.238591	8.268004	8.170839	8.36283	8.386218	8.37285	8.117904	7.966102	7.89303	Stx3	NM_152220
6871627	7633	8.276172	8.343071	8.329503	7.981105	0.295066	0.082342	0.361966	0.040838	0.348398	0.047091	0.013568	0.929499	-0.0669	0.664536	-0.05333	0.728979	6871627	8.420187	8.091748	8.31658	8.344697	8.585918	8.098598	8.373376	8.389082	8.226051	7.88746	7.853925	8.201931	Dtx4	NM_172442
6871645	7634	7.836222	7.923858	7.785694	7.767634	0.068588	0.426716	0.156224	0.092932	0.01806	0.831025	0.138164	0.130141	-0.08764	0.315892	0.050528	0.554479	6871645	7.765182	7.757736	7.985748	7.856501	7.985192	7.929882	7.861588	7.752949	7.742545	7.641239	7.776245	7.885419	Zfp91-cn	NR_024093
6871700	7644	7.689317	7.765088	7.780739	7.659471	0.029847	0.834443	0.105617	0.466734	0.121269	0.40587	-0.01565	0.912633	-0.07577	0.598526	-0.09142	0.52693	6871700	7.724247	7.66287	7.680835	7.746349	8.062154	7.48676	7.798681	7.922104	7.621433	7.581285	7.642219	7.754908	Tle4	NM_011600
6871768	7645	6.016922	6.431396	6.071404	5.641841	0.375081	0.133732	0.789555	0.007931	0.429563	0.092378	0.359991	0.147921	-0.41447	0.102424	-0.05448	0.81458	6871768	5.987331	5.930285	6.13315	6.115488	7.020736	6.157963	6.087744	5.958211	6.168258	5.595805	5.800641	5.529077	Psat1	NM_177420
6871771	7646	6.416967	6.50827	6.300893	6.241592	0.175374	0.177684	0.266678	0.054793	0.059301	0.630711	0.207377	0.118663	-0.0913	0.463746	0.116074	0.356635	6871771	6.369581	6.387243	6.494076	6.431859	6.757324	6.335627	6.332036	6.26573	6.304913	6.121977	6.440216	6.162584	Cep78	NM_198019
6871837	7647	6.891728	6.895544	6.833207	6.828595	0.063133	0.495841	0.066948	0.470991	0.004612	0.959714	0.062337	0.501132	-0.00382	0.966665	0.058521	0.526973	6871837	6.908564	6.877367	6.889254	6.835416	6.960936	6.890279	6.948907	6.716339	6.834375	6.714039	7.025185	6.746562	Vps13a	NM_173028
6871839	7648	6.120889	6.16016	6.254756	6.250595	-0.12971	0.344501	-0.09043	0.503511	0.004161	0.975082	-0.0946	0.484673	-0.03927	0.768761	-0.13387	0.330134	6871839	6.265761	6.011478	6.085427	6.422325	6.068545	5.989611	6.356014	6.290762	6.117491	6.379233	6.233164	6.139388	Foxb2	NM_008023
6871874	7649	7.037984	6.355748	6.857194	5.371833	1.666151	0.00225	0.983915	0.031344	1.485361	0.004335	-0.50145	0.220839	0.682235	0.108438	0.18079	0.644916	6871874	7.036191	6.662384	7.415376	6.276567	6.506492	6.284186	7.2049	6.265747	7.100935	5.72507	5.776687	4.613742	Gcnt1	NM_173442
6871879	7650	6.528211	6.646384	6.304557	5.990111	0.538099	0.057386	0.656272	0.026875	0.314446	0.231128	0.341827	0.196554	-0.11817	0.639295	0.223654	0.383607	6871879	6.669582	6.669675	6.245375	6.481882	7.213405	6.243864	6.324546	6.255682	6.333443	5.993308	6.178566	5.79846	Pcsk5	BC013068
6871903	7651	9.017727	9.118249	8.982276	8.991154	0.026573	0.79681	0.127096	0.238684	-0.00888	0.931322	0.135973	0.210258	-0.10052	0.343407	0.035451	0.731664	6871903	8.975685	8.934896	9.1426	9.038138	9.304577	9.012033	9.139463	8.916508	8.890858	9.034309	8.932512	9.00664	Ostf1	NM_017375
6872010	7653	12.1635	11.64941	11.60732	10.63059	1.532913	0.003629	1.01882	0.027116	0.976737	0.032237	0.042083	0.913978	0.514093	0.210336	0.556177	0.178868	6872010	12.33629	11.87485	12.27936	11.46238	12.03271	11.45313	12.27995	10.85327	11.68875	10.78127	10.94329	10.1672	Anxa1	NM_010730
6872029	7654	5.619082	6.393043	5.893711	6.704314	-1.08523	0.021637	-0.31127	0.438076	-0.8106	0.066285	0.499332	0.226839	-0.77396	0.076959	-0.27463	0.492013	6872029	5.428709	5.068461	6.360075	6.256844	6.482517	6.439768	6.229417	5.812678	5.639039	6.39907	7.35927	6.354603	Aldh1a7	NM_011921
6872051	7656	7.341918	7.192483	7.05799	6.994329	0.347589	0.138708	0.198154	0.375956	0.063662	0.770956	0.134493	0.542366	0.149435	0.499657	0.283927	0.216049	6872051	7.81988	7.117847	7.088026	7.17638	7.432593	6.968476	6.90562	7.168216	7.100135	6.95975	7.163277	6.859959	Gda	NM_010266
6872063	7657	7.39928	7.514067	7.464074	7.180399	0.218881	0.2067	0.333668	0.069521	0.283675	0.112821	0.049993	0.76168	-0.11479	0.491692	-0.06479	0.694863	6872063	7.406214	7.411509	7.380117	7.403347	7.726496	7.412357	7.62378	7.399502	7.36894	6.859218	7.194763	7.487215	1110059E	BC023385
6872203	7658	7.832848	7.892489	7.757552	7.540366	0.292482	0.126304	0.352123	0.073992	0.217186	0.240731	0.134937	0.453799	-0.05964	0.736836	0.075296	0.672056	6872203	7.846768	7.86658	7.785197	7.750503	8.016547	7.910418	8.087417	7.37037	7.81487	7.356027	7.627276	7.637795	Smc5	NM_153808
6872250	7661	9.293528	9.209048	9.29229	8.779149	0.514379	0.003988	0.429899	0.010274	0.51314	0.004042	-0.08324	0.536252	0.08448	0.530357	0.001239	0.992564	6872250	9.280587	9.388467	9.211531	9.161449	9.436288	9.029408	9.444498	9.182095	9.250276	8.680623	8.979109	8.677716	Tjp2	NM_011597
6872256	7662	6.962726	6.914229	6.757915	7.023914	-0.06119	0.603592	-0.10969	0.360999	-0.266	0.046715	0.156314	0.204715	0.048498	0.679679	0.204811	0.108043	6872256	6.942525	6.905863	7.039791	6.837806	7.054415	6.850465	6.894702	6.675751	6.703292	6.979201	6.842281	7.250261	Fxn	NM_008044
6872262	7663	8.259622	8.529045	8.354368	8.987334	-0.72771	0.040664	-0.45829	0.163175	-0.63297	0.066726	0.174676	0.574474	-0.26942	0.393009	-0.09475	0.759009	6872262	8.206278	8.130245	8.442343	8.806433	8.340971	8.43973	8.150278	8.923167	7.98966	9.011751	8.529929	9.420322	Pip5k1b	NM_008846
6872264	7664	6.991525	6.883838	7.052547	7.085957	-0.09443	0.523658	-0.20212	0.191362	-0.03341	0.819428	-0.16871	0.267677	0.107687	0.468827	-0.06102	0.67792	6872264	7.175936	6.976145	6.822495	7.037851	7.042249	6.571415	7.044762	7.054997	7.057883	6.951539	7.10717	7.199161	Fam122a	NM_026520
6872292	7666	7.167653	7.383127	7.213302	7.164676	0.002978	0.978633	0.218451	0.077204	0.048627	0.663806	0.169824	0.153705	-0.21547	0.080583	-0.04565	0.683019	6872292	7.153962	7.249275	7.099723	7.241522	7.536774	7.371084	7.375447	7.145502	7.118958	7.066085	7.092063	7.335879	Cbwd1	NM_146097
6872426	7668	8.585032	8.564357	8.58988	8.389241	0.195791	0.07856	0.175116	0.109052	0.200639	0.072703	-0.02552	0.799354	0.020675	0.836755	-0.00485	0.961408	6872426	8.678632	8.509069	8.567394	8.471901	8.717922	8.503248	8.671639	8.47347	8.624532	8.284377	8.552489	8.330857	D19Bwg13	NM_177474
6872444	7669	6.449582	6.590153	6.574365	6.501494	-0.05191	0.677968	0.088659	0.482861	0.072871	0.562085	0.015788	0.898986	-0.14057	0.276955	-0.12478	0.330675	6872444	6.363421	6.543805	6.441521	6.518901	6.753582	6.497976	6.336101	6.655792	6.731201	6.45253	6.639946	6.412005	Rfx3	NM_011265
6872462	7670	8.862008	9.505045	9.034186	9.754866	-0.89286	0.004839	-0.24982	0.312134	-0.72068	0.014401	0.470859	0.076468	-0.64304	0.024044	-0.17218	0.47844	6872462	8.6276	9.125731	8.832692	9.626478	9.33509	9.553568	8.692938	9.416901	8.99272	9.46314	9.700859	10.1006	Glis3	NM_175459
6872496	7672	9.124078	9.273421	9.283614	9.413711	-0.28963	0.060849	-0.14029	0.321813	-0.1301	0.356125	-0.01019	0.940728	-0.14934	0.29355	-0.15954	0.26415	6872496	9.008856	9.132798	9.23058	9.361758	9.309734	9.148771	9.264337	9.490026	9.096478	9.18777	9.463968	9.589396	Ak3	NM_021299
6872530	7675	7.679322	7.670911	7.606914	7.33507	0.344252	0.071528	0.335841	0.07739	0.271844	0.139727	0.063997	0.70957	0.00841	0.960788	0.072408	0.673869	6872530	7.632564	7.689468	7.715933	7.673573	7.833841	7.50532	7.860182	7.357349	7.603211	7.061344	7.601263	7.342603	5033414D	NM_026362
6872543	7676	9.463302	9.388992	9.673036	9.680518	-0.21722	0.134956	-0.29153	0.056171	-0.00748	0.955733	-0.28404	0.061418	0.07431	0.585106	-0.20973	0.147079	6872543	9.285593	9.57655	9.527763	9.164746	9.4938	9.50843	9.700438	9.816676	9.501994	9.558905	9.67849	9.80416	Ermp1	NM_001081213
6872549	7677	10.02343	9.942456	9.992563	9.931034	0.092397	0.325803	0.011422	0.900236	0.061529	0.505517	-0.05011	0.585843	0.080975	0.385773	0.030869	0.735585	6872549	10.16303	9.946891	9.960373	10.00501	9.931882	9.890476	9.90786	10.17513	9.894698	9.974119	9.957355	9.861628	9930021J	NM_172836
6872551	7678	6.90468	7.235416	7.229336	7.021047	-0.11637	0.442252	0.214369	0.174769	0.20829	0.185935	0.00608	0.967347	-0.33074	0.050665	-0.32466	0.054117	6872551	6.783939	6.815319	7.114783	7.023617	7.434818	7.247813	7.425674	7.096986	7.165349	6.88537	7.020276	7.157494	Ranbp6	NM_177721
6872646	7683	8.741358	9.048134	8.607283	9.135122	-0.39376	0.08365	-0.08699	0.674098	-0.52784	0.02935	0.440851	0.05794	-0.30678	0.162376	0.134075	0.520155	6872646	8.76088	8.955856	8.507339	9.09958	8.939737	9.105085	8.580918	8.395618	8.845314	8.898686	8.960056	9.546624	Asah2	NM_018830
6872651	7684	9.548484	9.738228	9.191478	9.475514	0.07297	0.656663	0.262715	0.135112	-0.28404	0.110098	0.546751	0.008585	-0.18974	0.264363	0.357006	0.053861	6872651	9.408223	9.506891	9.730338	9.580864	9.92865	9.705171	9.327684	8.853509	9.39324	9.446959	9.424273	9.555309	Sgms1	NM_144792
6872704	7685	8.999411	9.125212	9.20614	9.16544	-0.16603	0.137342	-0.04023	0.699606	0.0407	0.696282	-0.08093	0.444216	-0.1258	0.246285	-0.20673	0.073847	6872704	8.954598	9.047947	8.995689	9.043241	9.192983	9.139413	9.387908	9.141999	9.088513	8.992989	9.181446	9.321884	Atad1	NM_026487
6872731	7686	6.488335	6.439195	6.858645	6.612316	-0.12398	0.218068	-0.17312	0.098927	0.246329	0.028989	-0.41945	0.001944	0.049139	0.610624	-0.37031	0.003991	6872731	6.486902	6.44014	6.537962	6.403306	6.470995	6.443285	6.814494	6.843492	6.91795	6.734012	6.736146	6.36679	Rnls	NM_001146342
6872739	7687	6.888744	7.950939	6.388933	7.378876	-0.49013	0.492666	0.572063	0.425792	-0.98994	0.184566	1.562006	0.05118	-1.06219	0.157858	0.499812	0.484425	6872739	7.100661	7.97614	5.589432	7.982112	7.996843	7.873861	7.216434	6.699259	5.251105	6.943124	7.214101	7.979403	AI747699	NM_001013770
6872765	7689	9.190172	9.21713	9.273556	8.265195	0.924977	0.073967	0.951935	0.067391	1.008362	0.055432	-0.05643	0.903345	-0.02696	0.953718	-0.08338	0.857662	6872765	9.966996	8.982897	8.620623	9.503657	9.477866	8.669867	9.610355	9.524403	8.685911	8.179859	8.797209	7.818516	Acta2	NM_007392
6872781	7690	6.451109	6.506171	6.959001	5.179206	1.271903	0.050772	1.326965	0.043475	1.779795	0.012367	-0.45283	0.43731	-0.05506	0.923263	-0.50789	0.385999	6872781	6.948078	6.688065	5.717183	6.450143	7.085014	5.983355	7.126688	6.42541	7.324905	4.525743	6.261375	4.7505	Ch25h	NM_009890
6872783	7691	10.05029	10.27732	10.50126	10.04204	0.008248	0.95202	0.235281	0.114503	0.459224	0.008609	-0.22394	0.13034	-0.22703	0.125833	-0.45098	0.009434	6872783	10.16401	9.987329	9.999521	10.14449	10.48641	10.20106	10.55077	10.29344	10.65958	9.945773	10.23252	9.947824	Lipa	NM_021460
6872797	7693	6.845467	7.006677	6.934742	7.108333	-0.26287	0.022901	-0.10166	0.309094	-0.17359	0.100767	0.071935	0.464258	-0.16121	0.123317	-0.08927	0.368126	6872797	6.898067	6.795733	6.842601	7.120014	6.892935	7.007081	7.060223	6.990004	6.753998	7.046444	7.047377	7.231179	Pank1	NM_001114339
6872834	7696	5.519445	6.355416	5.789896	5.352688	0.166757	0.698053	1.002727	0.041941	0.437207	0.322434	0.56552	0.20969	-0.83597	0.078498	-0.27045	0.532478	6872834	5.064316	6.214046	5.279974	6.911564	6.137054	6.017629	6.295735	5.137456	5.936496	5.170131	5.230031	5.657903	Ankrd1	NM_013468
6872878	7697	8.365142	8.276279	8.204393	7.965434	0.399708	0.103341	0.310845	0.190732	0.238959	0.303781	0.071886	0.749452	0.088863	0.693513	0.160749	0.480889	6872878	8.361549	8.454855	8.279023	8.124127	8.495573	8.209137	8.645066	7.700706	8.267407	7.850581	8.070582	7.97514	Ppp1r3c	NM_016854
6872884	7698	6.176826	5.948183	5.835109	6.23002	-0.05319	0.805784	-0.28184	0.215014	-0.39491	0.095889	0.113074	0.603729	0.228643	0.306443	0.341717	0.141176	6872884	6.12621	6.473495	5.930773	6.026069	5.8922	5.926281	6.028099	5.837421	5.639808	6.07042	5.955378	6.664263	Fgfbp3	NM_028263
6872888	7699	5.856342	5.909231	5.852323	6.296475	-0.44013	0.039404	-0.38724	0.06248	-0.44415	0.038049	0.056908	0.758708	-0.05289	0.775183	0.004019	0.982637	6872888	5.75177	5.98162	5.835637	6.100803	5.804922	5.821967	5.598323	6.201519	5.757127	6.072331	6.283176	6.533919	Cpeb3	NM_198300
6872916	7700	9.948061	9.425101	9.661549	10.27844	-0.33038	0.214622	-0.85334	0.008302	-0.61689	0.03599	-0.23645	0.362971	0.522959	0.065431	0.286511	0.276094	6872916	10.02874	10.32368	9.491762	9.325505	9.560701	9.389098	9.754275	9.581772	9.648601	9.918872	10.70971	10.20674	Ide	NM_031156
6873025	7702	7.430819	7.451823	7.429072	7.323423	0.107395	0.389834	0.128399	0.308712	0.105649	0.39723	0.02275	0.852076	-0.021	0.863292	0.001746	0.988567	6873025	7.402423	7.517289	7.372744	7.253647	7.696131	7.40569	7.380373	7.460865	7.445979	7.243064	7.507384	7.219822	Noc3l	NM_021315
6873066	7707	10.52007	10.14799	10.37423	10.41787	0.102207	0.490091	-0.26987	0.092546	-0.04364	0.76535	-0.22624	0.148026	0.372081	0.030028	0.145843	0.332201	6873066	10.60899	10.34137	10.60986	10.15423	10.3366	9.953147	10.51327	10.36936	10.24006	10.50136	10.56361	10.18863	Pdlim1	NM_016861
6873068	7708	8.449073	8.274859	8.34489	8.432677	0.016396	0.927382	-0.15782	0.391764	-0.08779	0.628136	-0.07003	0.698412	0.174215	0.346897	0.104183	0.566622	6873068	8.469062	8.277978	8.60018	8.356167	8.326021	8.142388	8.668252	8.110542	8.255876	8.286461	8.298497	8.713074	Sorbs1	NM_178362
6873069	7709	7.963439	8.011829	8.101801	8.152026	-0.18859	0.14298	-0.1402	0.261734	-0.05023	0.676749	-0.08997	0.460687	-0.04839	0.687807	-0.13836	0.267542	6873069	7.8599	8.038398	7.99202	7.902643	8.121559	8.011285	8.277061	7.928201	8.10014	8.009703	8.344458	8.101916	Aldh18a1	NM_019698
6873083	7710	6.841789	7.145314	6.970109	6.922728	-0.08094	0.488637	0.222586	0.081011	0.047381	0.682112	0.175205	0.15479	-0.30352	0.026173	-0.12832	0.283082	6873083	6.763096	7.007025	6.755247	7.025313	7.379009	7.03162	7.04588	6.973577	6.890871	6.904218	7.014906	6.84906	Tctn3	NM_026260
6873111	7711	8.034261	7.832345	7.904093	7.792648	0.241613	0.096866	0.039697	0.765269	0.111445	0.411045	-0.07175	0.591881	0.201916	0.154746	0.130168	0.340721	6873111	8.011456	7.966114	8.125212	7.708848	8.13381	7.654376	7.912319	7.817863	7.982097	7.683249	7.759703	7.934991	Blnk	NM_008528
6873129	7714	10.59677	10.57529	10.64547	10.62948	-0.0327	0.706482	-0.05418	0.535936	0.01599	0.8534	-0.07017	0.426597	0.02148	0.804133	-0.04869	0.577126	6873129	10.56267	10.63033	10.59732	10.43641	10.59735	10.69212	10.8174	10.58246	10.53654	10.58644	10.63775	10.66424	Tm9sf3	NM_133352
6873133	7715	8.764207	9.526957	9.09008	8.765638	-0.00143	0.996301	0.761319	0.034452	0.324442	0.309745	0.436877	0.18233	-0.76275	0.034196	-0.32587	0.307754	6873133	8.812044	8.837906	8.642672	9.42499	9.797199	9.358682	9.719813	8.612731	8.937696	8.431904	8.682459	9.182552	Pik3ap1	NM_031376
6873153	7718	5.85132	6.035231	5.725982	6.113912	-0.26259	0.054389	-0.07868	0.518824	-0.38793	0.01043	0.309249	0.029154	-0.18391	0.153379	0.125338	0.313746	6873153	5.877558	5.764131	5.91227	5.974336	6.090405	6.040951	5.89794	5.602466	5.677539	6.053964	5.929654	6.358118	Frat2	NM_177603
6873154	7719	6.644603	6.45614	6.682129	6.562018	0.082585	0.454616	-0.10588	0.343211	0.12011	0.286134	-0.22599	0.063741	0.188463	0.110684	-0.03753	0.730279	6873154	6.738219	6.54321	6.652381	6.447639	6.575533	6.345249	6.724503	6.757762	6.564121	6.459693	6.770758	6.455604	Rrp12	NM_199447
6873158	7720	9.090989	9.031811	8.974932	9.086817	0.004172	0.966237	-0.05501	0.580593	-0.11189	0.275233	0.056879	0.568046	0.059179	0.552846	0.116057	0.259066	6873158	9.125436	8.953674	9.193858	8.885434	9.240654	8.969344	8.934404	9.022466	8.967926	9.108163	9.127708	9.024581	Exosc1	NM_025644
6873160	7721	8.344567	8.416343	8.384401	8.230817	0.11375	0.020821	0.185525	0.001579	0.153583	0.004706	0.031942	0.443545	-0.07178	0.107708	-0.03983	0.344269	6873160	8.360206	8.29151	8.381985	8.40344	8.502797	8.342791	8.403175	8.391077	8.35895	8.217565	8.241221	8.233666	Mms19	NM_028152
6873167	7722	7.738091	7.33713	7.695452	7.719898	0.018193	0.90021	-0.38277	0.026122	-0.02445	0.866252	-0.35832	0.034217	0.400961	0.021395	0.042639	0.769363	6873167	7.887819	7.598769	7.727685	7.477469	7.117395	7.416527	7.507911	7.830843	7.747601	7.710805	7.544825	7.904063	Morn4	NM_198108
6873171	7723	7.584162	7.813749	7.502985	7.727851	-0.14369	0.370524	0.085898	0.586159	-0.22487	0.17591	0.310764	0.074287	-0.22959	0.16801	0.081177	0.606538	6873171	7.616971	7.533592	7.601924	7.749641	8.006609	7.684998	7.690312	7.528924	7.289719	7.431614	7.859584	7.892356	Avpi1	NM_027106
6873194	7726	6.776638	7.323508	6.772709	7.491096	-0.71446	0.016288	-0.16759	0.497321	-0.71839	0.01588	0.550799	0.047668	-0.54687	0.048925	0.003929	0.98711	6873194	6.466695	6.832881	7.030337	7.550741	7.156509	7.263274	6.911336	6.776392	6.630398	7.28357	7.19738	7.992339	Loxl4	NM_053083

6873204	7727	6.887062	6.999547	6.859932	6.902051	-0.01499	0.883617	0.097496	0.354386	-0.04212	0.682262	0.139615	0.196867	-0.11248	0.289569	0.027131	0.791359	6873204	6.810444	6.974153	6.87659	7.054731	7.034311	6.909599	6.904654	7.015369	6.659772	6.795395	6.888891	7.021867	Hps1	NM_019424
6873217	7728	9.865609	9.942207	9.935259	10.95477	-1.08916	0.006594	-1.01257	0.009592	-1.01951	0.009268	0.006948	0.982046	-0.0766	0.804444	-0.06965	0.821809	6873217	9.581491	9.814425	10.20091	10.34612	9.591843	9.888658	9.659284	10.4007	9.745793	10.80249	10.69424	11.36759	Got1	NM_010324
6873230	7729	9.334366	9.366661	9.35359	9.544743	-0.21038	0.044245	-0.17808	0.078262	-0.19115	0.06216	0.013071	0.885893	-0.03229	0.723842	-0.01922	0.832984	6873230	9.336685	9.281443	9.384971	9.375435	9.397424	9.327124	9.329928	9.505534	9.225307	9.68912	9.388602	9.556507	Slc25a28	NM_145156
6873235	7730	7.624494	7.540128	7.48991	7.82057	-0.19608	0.151754	-0.28044	0.053208	-0.33066	0.028276	0.050219	0.695525	0.084365	0.514657	0.134584	0.308478	6873235	7.623191	7.653477	7.596833	7.414486	7.596338	7.609561	7.292129	7.540122	7.637478	7.61957	7.790035	8.052155	Cox15	NM_144874
6873237	7731	6.977466	7.192265	6.942061	6.839311	0.138155	0.230856	0.352954	0.010651	0.10275	0.363063	0.250204	0.046789	-0.2148	0.078521	0.035405	0.748181	6873237	6.905593	6.907736	7.11907	7.10885	7.427468	7.040478	7.032913	6.855552	6.937718	6.783572	6.881089	6.853272	Dnmbp	NM_028029
6873241	7732	6.920507	7.942375	7.596826	7.841752	-0.92125	0.018825	0.100622	0.756669	-0.24493	0.457526	0.345549	0.302814	-1.02187	0.011585	-0.67632	0.06324	6873241	6.519872	7.208519	7.033129	7.707228	8.28545	7.834446	8.002274	7.544463	7.243741	8.3849	7.513704	7.626653	Cpn1	NM_030703
6873246	7734	7.386451	7.517463	7.246207	7.351728	0.034723	0.75025	0.165735	0.154447	-0.10552	0.346014	0.271256	0.032926	-0.13101	0.249007	0.140244	0.219946	6873246	7.395072	7.302441	7.46184	7.37796	7.721408	7.453021	7.416435	7.105036	7.217149	7.307783	7.409793	7.337607	Erlin1	NM_145502
6873249	7735	7.165482	7.279036	7.170823	7.273484	-0.108	0.321207	0.005552	0.957981	-0.10266	0.344272	0.108213	0.320321	-0.11355	0.298529	-0.00534	0.959576	6873249	7.291212	7.163378	7.041856	7.184213	7.380848	7.272047	7.244069	7.285683	6.982718	7.190342	7.243032	7.387077	Chuk	NM_007700
6873252	7736	7.015402	7.031946	7.087587	7.060332	-0.04493	0.640307	-0.02839	0.76686	0.027255	0.775847	-0.05564	0.56429	-0.01654	0.862548	-0.07219	0.457799	6873252	6.981078	6.977591	7.087537	6.972622	7.163795	6.959422	7.120232	6.958489	7.184041	7.150216	7.137098	6.893683	Cwf19l1	NM_001081077
6873271	7738	9.659195	10.10734	9.005518	9.279529	0.379666	0.648784	0.827808	0.33247	-0.27401	0.741566	1.101819	0.207014	-0.44814	0.591837	0.653677	0.438909	6873271	8.613569	9.091586	11.27243	9.883851	10.35827	10.07989	9.917896	9.131525	7.967133	8.814495	10.33231	8.691782	Scd1	NM_009127
6873282	7739	9.22534	9.123627	8.924609	8.988894	0.236445	0.214717	0.134733	0.46462	-0.06429	0.723583	0.199018	0.28953	0.101713	0.578082	0.300731	0.124888	6873282	9.196606	9.069531	9.409882	9.17949	9.079592	9.111799	8.959126	9.231256	8.583445	9.00932	8.763227	9.194136	Ndufb8	NM_026061
6873307	7740	7.875224	7.919424	7.817918	7.877222	-0.002	0.984448	0.042202	0.682239	-0.0593	0.567134	0.101506	0.3369	-0.0442	0.668237	0.057305	0.580001	6873307	7.874393	7.81387	7.937408	7.812957	8.173244	7.772072	7.811128	7.842312	7.800315	7.842751	7.967424	7.821491	Mrpl43	NM_053164
6873309	7741	5.977735	5.87767	5.957235	5.957852	0.019883	0.799357	-0.08018	0.320203	-0.00062	0.993686	-0.07956	0.323714	0.100065	0.222546	0.0205	0.79329	6873309	6.035091	5.912982	5.985132	5.854783	5.790003	5.988225	5.858143	6.031876	5.981685	6.064114	5.842268	5.967175	Pdzd7	BC147399
6873333	7742	5.989721	6.120596	5.875515	6.331173	-0.34145	0.070417	-0.21058	0.234189	-0.45566	0.023771	0.24508	0.17264	-0.13087	0.446994	0.114206	0.50506	6873333	5.823061	5.9206	6.225503	6.281722	6.063231	6.016834	5.861259	6.119406	5.645881	6.358287	6.356604	6.118629	Lbx1	NM_010691
6873360	7743	6.109232	6.196899	6.136924	6.054822	0.05441	0.622865	0.142077	0.218472	0.082102	0.462462	0.059975	0.588383	-0.08767	0.433799	-0.02769	0.80121	6873360	6.136822	6.060744	6.130129	6.272786	6.313766	6.004144	6.103994	6.240933	6.065845	6.243886	5.909655	6.010925	Poll	NM_020032
6873361	7744	7.466608	7.353766	7.375411	7.33369	0.132918	0.130376	0.020076	0.805471	0.04172	0.611129	-0.02164	0.790675	0.112842	0.190325	0.091198	0.280853	6873361	7.445454	7.405901	7.54847	7.445205	7.433668	7.182425	7.438777	7.390447	7.297008	7.336873	7.400228	7.26397	Fbxw4	NM_013907
6873363	7745	6.13736	6.258614	6.096698	6.330539	-0.19318	0.033717	-0.07193	0.368656	-0.23384	0.014727	0.161916	0.064315	-0.12125	0.146938	0.040662	0.60484	6873363	6.149719	6.098957	6.163404	6.331038	6.074792	6.370011	6.171488	6.065704	6.052902	6.37764	6.269229	6.344748	Fgf8	NM_010205
6873364	7746	6.801789	6.679769	6.619294	6.460932	0.340857	0.110832	0.218838	0.283063	0.158363	0.429142	0.060475	0.758621	0.12202	0.539051	0.182495	0.365333	6873364	7.114699	6.889913	6.400755	6.472847	6.865351	6.70111	6.837969	6.439009	6.580905	6.417195	6.432395	6.533205	Npm3-ps1	NR_002702
6873365	7747	8.915462	8.94565	8.871825	8.798404	0.117058	0.200361	0.147247	0.117252	0.073421	0.406906	0.073825	0.404443	-0.03019	0.728234	0.043637	0.616982	6873365	8.958559	8.945313	8.842514	8.830334	9.03544	8.971177	8.907543	8.845892	8.86204	8.624335	8.826907	8.943969	Mgea5	NM_023799
6873369	7749	7.330322	7.214927	7.141811	7.292468	0.037854	0.727585	-0.07754	0.48098	-0.15066	0.188934	0.073116	0.505608	0.115395	0.303375	0.188511	0.110092	6873369	7.322818	7.331037	7.337111	7.275488	7.394271	6.975022	7.104946	7.073832	7.246656	7.179088	7.383383	7.314932	9130011E	NM_198296
6873393	7750	10.06394	10.2701	10.02302	10.13853	-0.07459	0.430152	0.131567	0.180947	-0.11551	0.234201	0.247075	0.024976	-0.20616	0.050757	0.040918	0.660662	6873393	10.07274	10.04848	10.0706	10.3458	10.33215	10.13234	9.91495	10.1841	9.970016	10.0066	10.17948	10.22951	Ldb1	NM_010697
6873396	7751	5.945677	6.111967	6.113341	6.147427	-0.20175	0.024664	-0.03546	0.640576	-0.03409	0.6534	-0.00137	0.985462	-0.16629	0.052468	-0.16766	0.050951	6873396	6.046049	5.919643	5.871338	6.158277	6.108941	6.068683	6.200519	6.195401	5.944103	6.170729	6.133497	6.138054	Pitx3	NM_008852
6873408	7753	7.226273	7.438194	7.254381	7.277359	-0.05109	0.579443	0.160835	0.106517	-0.02298	0.80159	0.183813	0.071319	-0.21192	0.043451	-0.02811	0.758785	6873408	7.12192	7.330742	7.226156	7.329525	7.570427	7.41463	7.321252	7.297338	7.144552	7.275338	7.169185	7.387553	Cuedc2	NM_024192
6873410	7754	9.092085	9.092744	9.105678	8.821109	0.270976	0.036648	0.271636	0.036302	0.284569	0.030141	-0.01293	0.907772	-0.00066	0.995286	-0.01359	0.903098	6873410	9.103062	9.067798	9.105395	9.059798	9.285286	8.933149	9.230021	9.142892	8.944121	8.693137	8.949817	8.820372	Actr1a	NM_016860
6873421	7755	8.516536	8.620713	8.607812	8.806001	-0.28946	0.094657	-0.18529	0.25967	-0.19819	0.230579	0.012901	0.934761	-0.10418	0.51444	-0.09128	0.566617	6873421	8.374426	8.62067	8.554691	8.6648	8.627449	8.569889	8.469894	8.970514	8.383028	8.773108	8.680885	8.964009	Arl3	NM_019718
6873466	7757	8.648964	8.461606	8.533503	8.254651	0.394314	0.003646	0.206956	0.065838	0.278852	0.020849	-0.0719	0.480559	0.187358	0.090011	0.115462	0.268869	6873466	8.714514	8.558484	8.673895	8.261557	8.64416	8.479102	8.600073	8.499896	8.500539	8.1639	8.361567	8.238485	Nt5c2	NM_029810
6873469	7758	6.609142	6.662262	6.540173	6.586221	0.022921	0.844714	0.076041	0.520992	-0.04605	0.695062	0.122089	0.312602	-0.05312	0.651664	0.068969	0.559554	6873469	6.695604	6.555855	6.575968	6.459275	6.901136	6.626376	6.604289	6.545386	6.470845	6.439298	6.630326	6.689039	Pcgf6	NM_027654
6873476	7759	6.228812	6.229763	6.010465	6.011507	0.217305	0.177596	0.218256	0.175915	-0.00104	0.994518	0.219298	0.17409	-0.00095	0.994997	0.218347	0.175755	6873476	6.134335	6.235276	6.316825	6.079155	6.450845	6.159289	6.20039	5.929347	5.901658	5.893913	6.283421	5.857187	Calhm2	NM_133746
6873503	7760	7.580524	7.718786	7.490709	7.439314	0.14121	0.250786	0.279473	0.039917	0.051395	0.66423	0.228078	0.080553	-0.13826	0.260004	0.089815	0.453705	6873503	7.583249	7.575972	7.58235	7.842898	7.77294	7.540521	7.431966	7.672491	7.367669	7.346446	7.6296	7.341895	Sh3pxd2a	NM_008018
6873510	7761	7.574441	7.558012	7.566023	7.336885	0.237556	0.17195	0.221128	0.200102	0.229138	0.185904	-0.00801	0.960893	0.016428	0.919923	0.008418	0.958909	6873510	7.502634	7.611006	7.609682	7.396708	7.688822	7.588507	7.804875	7.18906	7.704134	7.275539	7.479861	7.255254	Obfc1	NM_175360
6873516	7762	5.982426	6.052813	5.978265	6.496686	-0.51426	0.046799	-0.44387	0.07722	-0.51842	0.045431	0.074548	0.742302	-0.07039	0.756126	0.004161	0.985305	6873516	5.982689	6.037417	5.927172	6.105039	6.276481	5.776919	5.916182	6.092937	5.925676	6.623549	6.878558	5.987952	Col17a1	NM_007732
6873520	7763	7.496964	7.544779	7.10491	7.526918	-0.02995	0.843029	0.017861	0.905934	-0.42201	0.020455	0.439869	0.016974	-0.04782	0.752409	0.392054	0.028045	6873520	7.267754	7.527963	7.695174	7.690477	7.561603	7.382257	7.077599	7.052207	7.184924	7.472426	7.780265	7.328064	Itprip	NM_001001738
6873744	7765	8.537093	8.547555	8.511018	8.444373	0.092719	0.537053	0.103182	0.493393	0.066644	0.655338	0.036537	0.805809	-0.01046	0.943777	0.026075	0.860598	6873744	8.67011	8.402439	8.538729	8.501524	8.776896	8.364245	8.74588	8.43536	8.351813	8.302835	8.57693	8.453355	Xpnpep1	NM_133216
6873777	7766	6.627602	6.799462	6.725066	6.575059	0.052542	0.719956	0.224402	0.151317	0.150006	0.319907	0.074396	0.613201	-0.17186	0.259031	-0.09746	0.510306	6873777	6.709404	6.583513	6.589888	6.738915	6.880221	6.779249	6.812822	6.843541	6.518834	6.288807	6.84524	6.591131	Smndc1	NM_172429
6873887	7767	7.318241	7.466515	7.283621	7.368675	-0.05043	0.846657	0.09784	0.708481	-0.08505	0.744879	0.182894	0.489467	-0.14827	0.57322	0.03462	0.894324	6873887	6.98798	7.085573	7.881171	7.7503	7.38535	7.263896	7.432878	7.442793	6.975193	7.273397	7.403567	7.429062	Gpam	NM_008149
6873897	7769	6.934027	6.955149	6.87825	6.943543	-0.00952	0.88744	0.011606	0.862981	-0.06529	0.345411	0.0769	0.271588	-0.02112	0.753998	0.055777	0.416648	6873897	6.921223	6.887775	6.993083	6.842535	7.094614	6.928299	6.914855	6.885309	6.834585	7.019361	6.892779	6.918489	Zdhhc6	NM_001033573
6873944	7770	7.023551	6.758551	6.905735	6.601351	0.4222	0.656604	0.1572	0.867785	0.304384	0.747803	-0.14718	0.876124	0.265	0.779352	0.117815	0.90067	6873944	5.613643	6.56638	8.890629	7.684991	6.756642	5.834019	7.811435	6.569622	6.336149	5.658864	7.229599	6.91559	Nrap	NM_008733
6873960	7771	6.843649	6.87761	6.982317	6.763084	0.080564	0.325751	0.114525	0.175023	0.219232	0.021519	-0.10471	0.210733	-0.03396	0.670679	-0.13867	0.109235	6873960	6.853855	6.849558	6.827533	6.861404	6.975347	6.796078	7.057557	6.8415	7.047893	6.641849	6.789288	6.858116	Dclre1a	NM_018831
6873993	7772	9.053583	9.197967	9.243937	9.29315	-0.23957	0.03106	-0.09518	0.329814	-0.04921	0.606232	-0.04597	0.629792	-0.14438	0.15416	-0.19035	0.071671	6873993	8.990339	9.170725	8.999685	9.128209	9.154051	9.311641	9.211735	9.417009	9.103067	9.236532	9.271453	9.371466	A630007B	NM_170757
6874000	7773	9.694679	9.620268	9.737306	9.639738	0.054941	0.567265	-0.01947	0.837449	0.097568	0.32031	-0.11704	0.239465	0.074411	0.442436	-0.04263	0.655772	6874000	9.708172	9.691904	9.683961	9.677279	9.689732	9.493794	9.761692	9.760002	9.690225	9.498127	9.56225	9.858837	Afap1l2	NM_146102
6874001	7774	7.216492	7.248957	7.13321	7.292099	-0.07561	0.765684	-0.04314	0.864703	-0.15889	0.535118	0.115747	0.649482	-0.03246	0.897932	0.083282	0.742852	6874001	7.15416	7.210752	7.284565	7.420135	7.392088	6.934648	7.201177	7.17133	7.027123	6.802585	7.84305	7.230661	Ablim1	NM_178688
6874057	7775	8.305806	7.316128	7.852646	7.627493	0.678313	0.258907	-0.31136	0.592214	0.225154	0.697229	-0.53652	0.364584	0.989677	0.114142	0.453159	0.440344	6874057	8.918691	7.811321	8.187405	6.857403	8.042439	7.048543	8.086021	7.691568	7.78035	8.634762	7.710039	6.537677	Gfra1	NM_010279
6874085	7778	8.193411	7.796742	8.012578	7.834072	0.359339	0.027632	-0.03733	0.78723	0.178506	0.218707	-0.21584	0.145227	0.396669	0.017982	0.180833	0.21332	6874085	8.432292	8.06198	8.085961	7.849708	7.910317	7.630201	8.231969	7.852466	7.9533	7.886297	7.852363	7.763557	4930506M	NM_001114312
6874090	7779	6.849519	6.901242	6.939904	7.028287	-0.17877	0.181333	-0.12705	0.328543	-0.08838	0.489788	-0.03866	0.759627	-0.05172	0.68302	-0.09038	0.48032	6874090	6.839805	6.831337	6.877416	6.929993	6.831864	6.941869	6.943738	7.218727	6.657247	6.985452	6.978943	7.120467	Vax1	NM_009501
6874173	7780	7.716323	7.858635	7.70477	7.710553	0.00577	0.950507	0.148082	0.138901	-0.00578	0.950405	0.153864	0.126077	-0.14231	0.152883	0.011553	0.90114	6874173	7.702759	7.743482	7.702728	7.856896	7.756358	7.96265	7.858366	7.610913	7.645032	7.666054	7.598471	7.867133	D19Ertd7	NM_029648
6874190	7782	8.132863	8.294292	8.247743	8.147003	-0.01414	0.894852	0.147289	0.193046	0.10074	0.359498	0.046549	0.66524	-0.16143	0.157937	-0.11488	0.299867	6874190	8.131519	8.17951	8.087561	8.112123	8.498516	8.272236	8.335745	8.284758	8.122725	8.041115	8.13783	8.262063	2700078E	BC026363
6874212	7783	10.71563	10.7809	10.76975	10.63603	0.079603	0.262894	0.144873	0.059758	0.133723	0.077689	0.01115	0.870231	-0.06527	0.352343	-0.05412	0.436629	6874212	10.81843	10.69245	10.63601	10.71508	10.77521	10.85241	10.81229	10.7719	10.72506	10.52061	10.72301	10.66446	Eif3a	NM_010123
6874215	7785	9.3842	9.427054	9.220011	9.169451	0.214749	0.112775	0.257603	0.065137	0.05056	0.686013	0.207043	0.124288	-0.04285	0.731467	0.164188	0.2104	6874215	9.406967	9.345786	9.399846	9.324819	9.545399	9.410945	9.448325	9.08617	9.125539	9.022695	9.108226	9.377432	Prdx3	NM_007452
6874542	7786	8.140077	8.189385	8.230345	7.977053	0.163024	0.134724	0.212332	0.062107	0.253291	0.032362	-0.04096	0.686926	-0.04931	0.628365	-0.09027	0.38386	6874542	8.216068	8.106584	8.09758	8.148094	8.339967	8.080095	8.440648	8.167048	8.083338	7.96231	7.986846	7.982004	Nmt2	NM_008708
6874548	7787	6.937848	6.851828	6.92101	6.702255	0.235593	0.048023	0.149573	0.177028	0.218755	0.062284	-0.06918	0.512849	0.08602	0.419298	0.016838	0.871774	6874548	6.96371	6.877326	6.972508	6.826911	6.971292	6.75728	6.821874	6.889282	7.051874	6.72327	6.871337	6.512157	Dclre1c	NM_146114
6874584	7791	8.112843	7.985487	8.08727	7.39503	0.717813	0.005356	0.590457	0.014385	0.69224	0.006501	-0.10178	0.606153	0.127356	0.520882	0.025572	0.896091	6874584	8.287708	8.13527	7.91555	7.92873	8.236816	7.790914	8.13194	7.853981	8.27589	7.174992	7.720585	7.289513	Frmd4a	NM_172475
6874610	7792	8.825628	8.840878	8.791627	8.559051	0.266577	0.010411	0.281827	0.007861	0.232576	0.019776	0.049251	0.555693	-0.01525	0.853737	0.034001	0.682377	6874610	8.788759	8.763625	8.9245	8.787193	8.864877	8.870564	8.905787	8.611877	8.857217	8.620881	8.56192	8.494353	Bend7	NM_178663
6874626	7793	7.613676	7.762087	7.682214	7.632108	-0.01843	0.89256	0.12998	0.354284	0.050106	0.714524	0.079873	0.562442	-0.14841	0.294137	-0.06854	0.618168	6874626	7.629415	7.691898	7.519716	7.693396	7.933183	7.659683	7.911473	7.537217	7.597952	7.478402	7.578752	7.839169	Sephs1	NM_175400
6874631	7794	8.679189	8.803373	8.780306	9.376897	-0.69771	0.013178	-0.57352	0.031302	-0.59659	0.026605	0.023067	0.91909	-0.12418	0.587969	-0.10112	0.658074	6874631	8.527429	8.740903	8.769234	8.990581	8.580331	8.839207	8.788958	9.016547	8.535413	9.187394	9.090693	9.852604	Phyh	NM_010726
6874653	7796	6.983787	7.082506	6.616695	6.984434	-0.00065	0.998479	0.098073	0.773056	-0.36774	0.295763	0.465812	0.194242	-0.09872	0.771609	0.367092	0.296555	6874653	7.066051	7.166648	6.718662	6.979735	7.855707	6.412077	6.671214	6.626178	6.552692	6.818911	7.267491	6.866899	Ccdc3	NM_028804
6874683	7797	7.359468	7.41189	7.382831	7.211342	0.148126	0.181587	0.200548	0.082933	0.171489	0.128741	0.029059	0.781385	-0.05242	0.618614	-0.02336	0.823291	6874683	7.381131	7.346423	7.35085	7.277409	7.618365	7.339895	7.454251	7.361175	7.333066	7.209942	7.367498	7.056585	Nudt5	NM_016918
6874722	7798	8.188993	8.279832	8.286689	8.276399	-0.08741	0.463151	0.003433	0.976599	0.01029	0.929955	-0.00686	0.953284	-0.09084	0.446399	-0.0977	0.414192	6874722	8.310099	8.124681	8.132199	8.374403	8.373116	8.091977	8.330353	8.369912	8.159802	8.104203	8.433398	8.291596	Usp6nl	NM_001080548
6874946	7799	6.561549	6.757777	6.557049	6.65426	-0.09271	0.374996	0.103517	0.324853	-0.09721	0.353449	0.200729	0.076381	-0.19623	0.081976	0.004501	0.964742	6874946	6.628437	6.437412	6.618799	6.674137	6.944467	6.654728	6.457397	6.583284	6.630465	6.534916	6.669969	6.757895	Kin	NM_025280
6874947	7800	6.895727	6.591919	6.239031	6.732623	0.163104	0.701067	-0.1407	0.740215	-0.49359	0.262881	0.352888	0.414286	0.303808	0.479721	0.656696	0.147759	6874947	6.891354	6.808716	6.987112	6.617191	6.93296	6.225607	6.924541	5.942329	5.850223	5.900743	7.17565	7.121477	Itih5	NM_172471
6875039	7804	6.862407	6.831456	6.644409	6.643546	0.218861	0.018028	0.187911	0.034437	0.000863	0.990957	0.187047	0.035069	0.030951	0.686099	0.217998	0.018353	6875039	6.772985	6.91455	6.899686	6.805318	6.865941	6.82311	6.555081	6.6341	6.744046	6.501191	6.753177	6.676269	Il15ra	NM_008358
6875042	7805	7.176059	7.385138	7.096507	7.13603	0.040029	0.771507	0.249108	0.098458	-0.03952	0.774299	0.288631	0.062176	-0.20908	0.155233	0.079552	0.566971	6875042	7.089622	7.137071	7.301483	7.400061	7.532293	7.223061	7.177131	7.001646	7.110744	7.023778	6.962424	7.421888	Ankrd16	NM_177268

6875094	7806	7.337053	7.196348	7.291995	7.075759	0.261294	0.252534	0.120588	0.584913	0.216236	0.337366	-0.09565	0.663695	0.140705	0.525343	0.045058	0.836926	6875094	7.342635	7.375998	7.292526	7.073726	7.432068	7.083249	7.639781	7.058435	7.17777	6.69995	7.424097	7.103231	Pter	NM_008961
6875132	7807	11.06565	11.06183	10.66062	10.32521	0.740439	0.062876	0.736615	0.063977	0.335405	0.356699	0.40121	0.275701	0.003823	0.991378	0.405033	0.271492	6875132	11.26254	11.14138	10.79304	10.92341	11.3225	10.93958	11.14316	10.0667	10.772	10.09303	10.94704	9.935574	Vim	NM_011701
6875173	7808	8.687202	8.647337	8.719425	8.586313	0.100889	0.166259	0.061024	0.383881	0.133111	0.079349	-0.07209	0.308194	0.039865	0.563972	-0.03222	0.639713	6875173	8.725472	8.658616	8.677519	8.55863	8.788407	8.594975	8.754785	8.793705	8.609784	8.608713	8.585835	8.564392	Stam	NM_011484
6875181	7810	8.499733	7.881751	7.843422	7.323721	1.176012	0.009182	0.55803	0.143994	0.519701	0.169913	0.038329	0.914168	0.617982	0.110643	0.656311	0.093281	6875181	8.981004	8.213547	8.304647	7.926902	8.413445	7.304905	8.24247	7.67827	7.609526	7.349324	7.637578	6.984261	Mrc1	NM_008625
6875191	7811	7.529078	7.269442	7.339022	7.408186	0.120892	0.51877	-0.13874	0.460882	-0.06916	0.70948	-0.06958	0.707824	0.259637	0.185256	0.190056	0.319667	6875191	7.812016	7.155499	7.61972	7.301873	7.429801	7.076651	7.574409	7.188951	7.253707	7.481385	7.375764	7.367409	Cacnb2	NM_023116
6875197	7812	8.446896	8.489276	8.332256	8.572109	-0.12521	0.476323	-0.08283	0.634374	-0.23985	0.19022	0.15702	0.376172	-0.04238	0.806723	0.11464	0.513228	6875197	8.365748	8.522843	8.452097	8.333354	8.552474	8.581999	8.252702	8.703716	8.040349	8.555242	8.410625	8.750461	Arl5b	NM_029466
6875214	7813	10.55071	10.52392	10.59415	10.532	0.018707	0.891244	-0.00808	0.952863	0.06215	0.65163	-0.07023	0.610552	0.02679	0.844856	-0.04344	0.751485	6875214	10.52378	10.58895	10.5394	10.46059	10.61024	10.50093	10.71141	10.47905	10.592	10.22066	10.57796	10.79739	Plxdc2	NM_026162
6875325	7814	9.176906	9.179297	9.232995	9.259038	-0.08213	0.226328	-0.07974	0.238911	-0.02604	0.688618	-0.0537	0.416403	-0.00239	0.970501	-0.05609	0.396864	6875325	9.163424	9.184216	9.183078	9.189564	9.152514	9.195812	9.173183	9.387991	9.13781	9.208229	9.233376	9.335508	Dnajc1	BC080300
6875375	7815	8.549464	8.766745	8.581468	8.44261	0.106853	0.47735	0.324134	0.053624	0.138857	0.361078	0.185277	0.232264	-0.21728	0.168049	-0.032	0.828928	6875375	8.583844	8.584095	8.480452	8.713846	8.758703	8.827685	8.730817	8.737132	8.276454	8.24455	8.410393	8.672888	Commd3	NM_147778
6875376	7816	7.335089	7.336592	7.159653	7.42603	-0.09094	0.62853	-0.08944	0.63413	-0.26638	0.178892	0.176939	0.356424	-0.0015	0.993572	0.175437	0.360304	6875376	7.335312	7.407594	7.262362	7.276145	7.367023	7.366608	7.472976	6.822283	7.183699	7.24604	7.276145	7.755904	Bmi1	NM_007552
6875421	7818	7.177923	7.149334	7.168882	7.422668	-0.24474	0.114783	-0.27333	0.083546	-0.25379	0.103868	-0.01955	0.891103	0.02859	0.841411	0.009041	0.949486	6875421	7.037806	7.315282	7.180682	7.104964	7.176761	7.166276	7.235898	7.306006	6.964742	7.256478	7.303689	7.707836	Msrb2	NM_029619
6875459	7820	8.357948	8.302474	8.271621	7.957083	0.400865	0.002163	0.345391	0.005039	0.314537	0.008256	0.030853	0.741255	0.055474	0.555838	0.086328	0.366801	6875459	8.347516	8.370543	8.355786	8.344885	8.368025	8.194512	8.223461	8.333274	8.258127	7.801087	8.170929	7.899234	Etl4	NM_001081006
6875556	7822	6.474762	6.390881	6.668046	6.633692	-0.15893	0.156796	-0.24281	0.044051	0.034354	0.744248	-0.27716	0.02605	0.083881	0.433463	-0.19328	0.093933	6875556	6.437411	6.652203	6.334672	6.447134	6.313671	6.411839	6.580529	6.691563	6.732046	6.814865	6.559508	6.526704	Thnsl1	NM_177588
6875592	7827	7.764527	7.533483	7.72436	6.748611	1.015915	0.01093	0.784872	0.034381	0.975749	0.013287	-0.19088	0.553	0.231044	0.475011	0.040167	0.899545	6875592	8.172104	7.681652	7.439824	7.560095	7.866329	7.174025	7.776588	7.551957	7.844535	6.333398	7.353502	6.558934	Apbb1ip	NM_019456
6875602	7828	8.754187	8.83342	8.841351	8.903866	-0.14968	0.253251	-0.07045	0.578257	-0.06251	0.621026	-0.00793	0.949586	-0.07923	0.53288	-0.08716	0.493823	6875602	8.706023	8.78237	8.774168	8.872013	8.77662	8.851626	9.000937	8.724077	8.79904	8.64973	8.906559	9.15531	Acbd5	NM_001102437
6875608	7829	9.509623	9.560934	9.642871	9.539779	-0.03016	0.803447	0.021155	0.861255	0.103091	0.404742	-0.08194	0.504322	-0.05131	0.673122	-0.13325	0.288503	6875608	9.45038	9.535551	9.542937	9.382878	9.619431	9.680494	9.82103	9.447812	9.65977	9.378757	9.596075	9.644506	Yme1l1	NM_013771
6875638	7833	6.42198	6.497468	6.28819	5.559278	0.862702	0.253761	0.93819	0.217913	0.728912	0.329205	0.209278	0.773079	-0.07549	0.916966	0.13379	0.853513	6875638	7.213236	6.200072	5.852633	6.531135	6.680186	6.281082	6.954484	5.26116	6.648926	4.785182	7.018255	4.874397	Il1f6	NM_019450
6875649	7836	9.25689	9.094884	8.869733	7.005178	2.251712	0.013493	2.089706	0.019053	1.864555	0.031003	0.225151	0.760466	0.162006	0.826112	0.387157	0.60226	6875649	9.597782	9.440609	8.732279	8.683619	9.759842	8.84119	9.289486	7.625939	9.693773	6.599209	8.294783	6.121542	Il1rn	NM_031167
6875650	7837	7.1714	6.992722	6.99033	6.57991	0.59149	0.003152	0.412812	0.019724	0.41042	0.02024	0.002392	0.98698	0.178678	0.244004	0.18107	0.238279	6875650	7.284007	7.088132	7.142061	7.080627	7.10451	6.793028	7.159106	6.867275	6.944608	6.414637	6.856156	6.468936	Psd4	NM_177611
6875659	7838	6.391858	6.415833	6.501662	6.40053	-0.00867	0.960373	0.015304	0.930139	0.101132	0.566476	-0.08583	0.625577	-0.02398	0.890796	-0.1098	0.534453	6875659	6.212717	6.28827	6.674587	6.734804	6.199977	6.312719	6.332889	6.546276	6.62582	6.326391	6.376281	6.498917	Rnf208	NM_176834
6875662	7839	6.505278	6.6933	6.748284	6.696739	-0.19146	0.322434	-0.00344	0.98535	0.051545	0.783692	-0.05498	0.76973	-0.18802	0.330671	-0.24301	0.217536	6875662	6.08769	6.832735	6.59541	6.631294	6.871881	6.576724	6.861032	6.800369	6.58345	6.640441	6.657273	6.792503	Zmynd19	NM_026021
6875664	7840	7.197321	7.298745	7.273903	7.210066	-0.01275	0.846642	0.088679	0.202001	0.063837	0.346335	0.024842	0.707162	-0.10142	0.150564	-0.07658	0.264342	6875664	7.13081	7.193306	7.267846	7.286342	7.329508	7.280385	7.369066	7.202763	7.24988	7.09342	7.306413	7.230366	Mrpl41	NM_001031808
6875666	7841	7.179445	7.217355	7.111853	7.015675	0.16377	0.055866	0.201681	0.024971	0.096179	0.225746	0.105502	0.187895	-0.03791	0.618904	0.067591	0.383295	6875666	7.281806	7.144759	7.111769	7.305225	7.200672	7.146169	7.106739	7.053094	7.175727	7.063957	6.88187	7.101197	Pnpla7	NM_146251
6875680	7842	8.328461	8.479926	8.255305	8.261733	0.066728	0.420594	0.218192	0.024076	-0.00643	0.936825	0.224621	0.021227	-0.15146	0.090141	0.073157	0.379228	6875680	8.386265	8.24608	8.353039	8.473244	8.632888	8.333645	8.266718	8.300511	8.198685	8.257581	8.182686	8.344933	Nelf	NM_001039386
6875695	7844	7.52863	7.768831	7.325821	7.365442	0.163189	0.407367	0.403389	0.06264	-0.03962	0.837181	0.44301	0.044977	-0.2402	0.23405	0.202809	0.30881	6875695	7.4289	7.666514	7.490477	7.576234	8.13589	7.594369	7.299201	7.145239	7.533024	7.261328	7.632845	7.202152	Nrarp	NM_025980
6875697	7845	6.062834	6.062266	6.191314	6.115726	-0.05289	0.637625	-0.05346	0.634067	0.075589	0.504029	-0.12905	0.266557	0.000568	0.995932	-0.12848	0.268507	6875697	6.067435	5.962849	6.158219	6.288364	5.90945	5.988984	6.092117	6.188486	6.29334	6.013604	6.115601	6.217972	Fam166a	BC061039
6875698	7846	5.961417	5.957456	6.030034	6.065514	-0.1041	0.454063	-0.10806	0.43771	-0.03548	0.795346	-0.07258	0.598266	0.003961	0.976849	-0.06862	0.618048	6875698	6.132277	5.776891	5.975082	5.781695	5.911739	6.178933	6.202334	5.989303	5.898464	5.982905	6.05349	6.160148	C730025P	NM_177344
6875702	7847	6.29698	6.504313	6.270481	6.319381	-0.0224	0.794468	0.184932	0.056851	-0.0489	0.572755	0.233832	0.022783	-0.20733	0.037342	0.026499	0.758164	6875702	6.318532	6.231114	6.341294	6.532515	6.634809	6.345615	6.233225	6.407608	6.170611	6.277847	6.324917	6.355379	C430004E	NM_175286
6875703	7848	8.8079	8.720865	8.692527	8.429777	0.378123	0.00958	0.291088	0.031355	0.26275	0.046555	0.028337	0.806177	0.087035	0.4584	0.115372	0.33198	6875703	8.813354	8.762192	8.848153	8.671113	8.927607	8.563874	8.853559	8.673063	8.55096	8.368183	8.570456	8.350692	Anapc2	NM_175300
6875706	7849	5.901808	6.541201	5.852344	6.545276	-0.64347	0.171551	-0.00408	0.992645	-0.69293	0.144491	0.688857	0.146564	-0.63939	0.173972	0.049464	0.910938	6875706	5.517552	5.959517	6.228356	7.270002	6.662247	5.691353	6.029536	5.727052	5.800444	6.66441	5.935125	7.036292	Lrrc26	NM_146117
6875716	7850	6.157688	6.18286	6.143509	6.148812	0.008877	0.879577	0.034049	0.565117	-0.0053	0.927846	0.039352	0.507665	-0.02517	0.669105	0.01418	0.808986	6875716	6.191156	6.070838	6.211071	6.10955	6.152434	6.286597	6.16455	6.130301	6.135675	6.212053	6.076002	6.15838	2010317E	NM_001081085
6875719	7851	7.808865	7.521518	7.476169	7.479338	0.329527	0.046047	0.04218	0.770407	-0.00317	0.982458	0.045349	0.7538	0.287347	0.073721	0.332696	0.044445	6875719	8.042802	7.613204	7.770589	7.562646	7.493412	7.508496	7.68059	7.484883	7.263033	7.357945	7.422384	7.657684	Entpd2	NM_009849
6875720	7852	8.149758	8.047903	7.942194	8.117004	0.032753	0.756216	-0.0691	0.516977	-0.17481	0.124718	0.105709	0.330073	0.101855	0.346972	0.207564	0.076128	6875720	8.202031	8.130616	8.116626	8.058508	8.173395	7.911805	8.08235	8.000349	7.743883	8.106724	8.230793	8.013496	Npdc1	NM_008721
6875722	7854	8.699661	8.493308	8.50244	8.324225	0.375436	0.002197	0.169082	0.081125	0.178214	0.068628	-0.00913	0.916843	0.206354	0.040887	0.197222	0.048375	6875722	8.821859	8.692385	8.58474	8.561853	8.536097	8.381973	8.431296	8.663376	8.412647	8.321065	8.329584	8.322027	Abca2	NM_007379
6875723	7855	10.16377	10.01281	9.987256	9.846456	0.317314	0.174733	0.166352	0.457417	0.1408	0.527282	0.025552	0.907497	0.150962	0.498732	0.176514	0.431426	6875723	10.00709	10.14637	10.33785	10.16623	10.19839	9.673803	10.34253	9.716331	9.902906	9.906226	10.04525	9.587892	Clic3	NM_027085
6875727	7856	9.869203	9.607839	9.855471	10.00446	-0.13525	0.431638	-0.39662	0.041317	-0.14899	0.388355	-0.24763	0.167969	0.261364	0.148233	0.013732	0.935067	6875727	9.820586	9.885316	9.901706	9.650713	9.802488	9.370316	9.586597	10.12138	9.858436	9.808252	10.00364	10.20148	Fbxw5	NM_013908
6875728	7857	10.97248	10.98199	11.09107	10.93599	0.036487	0.797029	0.045993	0.746095	0.15508	0.291127	-0.10909	0.449541	-0.00951	0.946464	-0.11859	0.412582	6875728	10.96615	10.92635	11.02494	10.84544	11.12565	10.97487	11.14578	11.33956	10.78788	10.81796	10.94969	11.04033	Edf1	NM_021519
6875748	7862	8.891845	8.81392	8.984668	8.983785	-0.09194	0.541609	-0.16986	0.272713	0.000883	0.995263	-0.17075	0.270414	0.077925	0.603685	-0.09282	0.537827	6875748	8.906732	8.934689	8.834114	8.686891	9.000682	8.754187	8.744939	9.199514	9.009551	9.048501	8.752576	9.150277	Bmyc	NM_023326
6875789	7865	7.331648	7.419516	7.316421	7.554308	-0.22266	0.069456	-0.13479	0.240332	-0.23789	0.055559	0.103095	0.360408	-0.08787	0.432308	0.015227	0.88961	6875789	7.371088	7.325618	7.298239	7.456121	7.445207	7.357221	7.357888	7.437205	7.154171	7.318207	7.690914	7.653804	Gpsm1	NM_153410
6875792	7866	8.730875	8.690166	8.669088	8.995586	-0.26471	0.014807	-0.30542	0.007299	-0.3265	0.00512	0.021078	0.811623	0.04071	0.646955	0.061788	0.49079	6875792	8.70819	8.683365	8.801071	8.636075	8.741282	8.69314	8.703193	8.660474	8.643596	8.818562	8.970705	9.197491	Pmpca	NM_173180
6875803	7867	8.531104	8.707699	8.450718	8.696371	-0.16527	0.443238	0.011328	0.957268	-0.24565	0.264885	0.256981	0.245193	-0.1766	0.413862	0.080386	0.705076	6875803	8.740559	8.605337	8.247416	8.747174	8.770577	8.605346	8.574128	8.418734	8.359293	8.285255	8.70212	9.101738	Egfl7	NM_198724
6875804	7868	6.251126	6.303616	6.248015	6.655081	-0.40395	0.00213	-0.35146	0.004709	-0.40707	0.002035	0.055602	0.556905	-0.05249	0.578727	0.003112	0.973474	6875804	6.275408	6.337021	6.14095	6.296009	6.241563	6.373277	6.169426	6.349166	6.225452	6.760586	6.4677	6.736957	Fam69b	NM_019833
6875825	7869	8.680981	8.669491	8.728188	8.670056	0.010924	0.916615	-0.00057	0.995673	0.058132	0.581109	-0.0587	0.577509	0.01149	0.912317	-0.04721	0.653003	6875825	8.638253	8.637601	8.767088	8.559922	8.88996	8.55859	8.666839	8.776327	8.741398	8.590771	8.602599	8.816799	Surf2	NM_013678
6875834	7872	8.126392	7.933002	8.103751	8.256414	-0.13002	0.395383	-0.32341	0.055967	-0.15266	0.322488	-0.17075	0.272142	0.19339	0.21839	0.022641	0.879609	6875834	7.93295	8.21876	8.227466	8.024972	7.921914	7.852119	7.831318	8.395812	8.084123	8.37274	8.203382	8.193121	5930434B	NM_029862
6875850	7874	6.400174	6.688114	6.290109	6.960893	-0.56072	0.155269	-0.27278	0.467181	-0.67078	0.097343	0.398005	0.297728	-0.28794	0.443675	0.110065	0.765951	6875850	6.447229	6.473279	6.280013	6.762784	6.675353	6.626205	6.371646	6.316281	6.1824	6.416373	6.513019	7.953287	Adamtsl2	NM_029981
6875865	7876	7.459888	7.632567	7.544757	7.527234	-0.06735	0.47289	0.105333	0.272607	0.017523	0.849509	0.08781	0.354803	-0.17268	0.089534	-0.08487	0.370298	6875865	7.426481	7.443403	7.509781	7.543989	7.810439	7.543273	7.616469	7.613121	7.40468	7.607344	7.434609	7.539749	Wdr5	NM_080848
6875901	7877	8.214523	8.241495	8.186887	8.258938	-0.04441	0.736571	-0.01744	0.894567	-0.07205	0.587502	0.054608	0.679737	-0.02697	0.837754	0.027636	0.833829	6875901	8.117913	8.275386	8.25027	8.307482	8.329568	8.087435	8.150524	8.440475	7.969662	8.134125	8.387438	8.25525	Rxra	NM_011305
6875920	7878	7.68067	7.72945	7.405735	7.539608	0.141062	0.442473	0.189842	0.308551	-0.13387	0.465234	0.323715	0.100834	-0.04878	0.787027	0.274935	0.153963	6875920	7.942826	7.656695	7.442488	7.787726	7.927079	7.473544	7.431613	7.641997	7.143594	7.498851	7.605316	7.514656	Col5a1	NM_015734
6875933	7879	8.528829	7.721067	8.071289	7.465473	1.063755	0.025333	0.255994	0.527735	0.606216	0.15664	-0.35022	0.392867	0.807762	0.070799	0.45754	0.271972	6875933	8.784243	8.359757	8.442486	7.556464	8.244986	7.361751	8.786329	7.468202	7.959336	7.656744	7.775544	6.962932	Olfm1	NM_019498
6875960	7880	6.654411	6.771708	6.666254	6.717343	-0.06293	0.462711	0.054365	0.523978	-0.05109	0.54867	0.105454	0.232292	-0.1173	0.188499	-0.01184	0.888187	6875960	6.517163	6.747914	6.698155	6.749884	6.772318	6.792921	6.523151	6.675666	6.799944	6.70031	6.653069	6.79865	Gm347	BC070440
6875961	7881	7.684237	7.70056	7.693105	7.709866	-0.02563	0.828749	-0.00931	0.937311	-0.01676	0.887402	0.007455	0.949758	-0.01632	0.890329	-0.00887	0.940262	6875961	7.714881	7.704577	7.633254	7.636724	7.902849	7.562107	7.743654	7.871896	7.463764	7.747892	7.67689	7.704817	Mrps2	NM_080452
6875964	7882	6.742782	6.574639	6.813646	6.254121	0.488661	0.000775	0.320517	0.008788	0.559525	0.00032	-0.23901	0.033271	0.168144	0.108542	-0.07086	0.4684	6875964	6.746772	6.699724	6.781851	6.580615	6.650139	6.493162	6.855213	6.707044	6.878682	6.235803	6.450917	6.075644	Gbgt1	NM_139197
6875966	7883	8.531586	8.459197	8.515959	8.492744	0.038842	0.699835	-0.03355	0.738837	0.023216	0.817189	-0.05676	0.575242	0.072389	0.477635	0.015626	0.876236	6875966	8.56419	8.504249	8.526318	8.487275	8.638068	8.252248	8.663795	8.460583	8.4235	8.455924	8.497592	8.524715	Ralgds	NM_009058
6875969	7884	7.29815	7.48144	7.269067	7.562935	-0.26478	0.018801	-0.08149	0.392458	-0.29387	0.011538	0.212372	0.046282	-0.18329	0.076508	0.029082	0.755295	6875969	7.179496	7.307961	7.406992	7.498354	7.35478	7.591185	7.137593	7.330299	7.33931	7.633916	7.45807	7.596818	Tsc1	NM_022887
6875988	7885	6.816228	6.992973	6.845802	6.828878	-0.01265	0.921926	0.164095	0.225908	0.016924	0.895707	0.147171	0.273135	-0.17674	0.195313	-0.02957	0.81902	6875988	6.718874	6.967235	6.762575	6.78655	7.007699	7.184669	6.641591	6.995669	6.900145	6.768388	6.845476	6.87277	Ttf1	NM_009442
6875994	7886	7.439819	7.449074	7.409562	7.371936	0.067882	0.497207	0.077137	0.442402	0.037626	0.703757	0.039512	0.689804	-0.00926	0.925147	0.030257	0.759381	6875994	7.445456	7.462465	7.411535	7.365827	7.665717	7.315677	7.441782	7.46816	7.318744	7.354804	7.488157	7.272848	Setx	NM_198033
6876010	7887	8.770201	8.60334	8.740466	8.762388	0.007812	0.932544	-0.15905	0.113257	-0.02192	0.812551	-0.13713	0.163786	0.16686	0.099079	0.029734	0.748099	6876010	8.73438	8.660998	8.915224	8.566292	8.762852	8.480877	8.790842	8.663164	8.767393	8.693432	8.754595	8.839138	Med27	NM_026896
6876014	7888	8.504392	8.633803	8.445977	8.531728	-0.02734	0.726614	0.102074	0.213264	-0.08575	0.288824	0.187826	0.03762	-0.12941	0.124792	0.058415	0.461255	6876014	8.438292	8.537265	8.537618	8.700855	8.633145	8.567408	8.471274	8.508354	8.358303	8.378641	8.660911	8.555633	Rapgef1	NM_001039087
6876022	7889	7.744128	7.705158	7.732667	7.765193	-0.02107	0.816651	-0.06003	0.513957	-0.03253	0.720998	-0.02751	0.762347	0.03897	0.669306	0.011461	0.899496	6876022	7.690546	7.703671	7.838167	7.676854	7.755535	7.683086	7.718847	7.906416	7.572739	7.675021	7.749403	7.871156	Coq4	NM_178693
6876023	7890	7.87946	7.886391	7.826891	7.816284	0.063176	0.618214	0.070107	0.580929	0.010607	0.932782	0.0595	0.63849	-0.00693	0.956044	0.052569	0.677598	6876023	7.851745	7.931763	7.854873	7.866356	8.052674	7.740143	7.99964	7.900221	7.580813	7.693989	7.934304	7.82056	Slc27a4	NM_011989
6876026	7891	8.424103	8.429414	8.265775	8.119566	0.304537	0.047035	0.309848	0.044127	0.146209	0.292841	0.163639	0.243133	-0.00531	0.968381	0.158328	0.257478	6876026	8.338532	8.562482	8.371295	8.379948	8.465667	8.442626	8.315585	8.148723	8.333016	8.224778	8.323721	7.810198	Cercam	NM_207298

6876028	7892	7.754577	7.880807	7.760798	7.687809	0.066768	0.204012	0.192998	0.003961	0.072989	0.168997	0.120009	0.03776	-0.12623	0.030899	-0.00622	0.900645	6876028	7.726213	7.76886	7.768658	7.834407	7.976503	7.83151	7.821728	7.779817	7.680849	7.664337	7.728918	7.670171	Odf2	NM_001113213
6876029	7893	8.507859	8.537968	8.458966	8.371755	0.136104	0.005223	0.166214	0.00166	0.087212	0.040813	0.079002	0.058376	-0.03011	0.424743	0.048892	0.20919	6876029	8.476933	8.505646	8.540997	8.487604	8.538801	8.5875	8.486161	8.449367	8.441371	8.310523	8.430338	8.374403	Gle1	NM_028923
6876033	7894	7.808948	7.87096	7.659142	7.66148	0.147468	0.462681	0.209481	0.3051	-0.00234	0.990542	0.211819	0.300092	-0.06201	0.753987	0.149806	0.455866	6876033	7.752326	7.669304	8.005214	7.75659	8.085653	7.770638	7.746114	7.671533	7.559778	7.269327	7.684504	8.030608	Set	NM_023871
6876037	7895	6.95741	7.078594	6.882072	7.142413	-0.185	0.09092	-0.06382	0.526083	-0.26034	0.026914	0.196521	0.075554	-0.12118	0.243666	0.075338	0.45649	6876037	6.904007	7.070617	6.897606	7.034497	7.122798	7.078486	6.911917	6.753872	6.980428	6.94351	7.212157	7.271572	Pkn3	NM_153805
6876043	7896	7.848978	7.967222	7.740041	7.80054	0.048438	0.706892	0.166682	0.216687	-0.0605	0.639456	0.227181	0.10496	-0.11824	0.369237	0.108937	0.406281	6876043	7.788734	7.866031	7.892169	7.887129	8.143979	7.870558	7.713599	7.909063	7.597462	7.638455	7.734017	8.029149	Tbc1d13	NM_146252
6876044	7897	6.761577	6.982954	6.516937	6.118541	0.643036	0.011039	0.864413	0.002223	0.398396	0.075944	0.466017	0.044298	-0.22138	0.290305	0.24464	0.246184	6876044	6.823024	6.685218	6.776488	6.927238	7.198235	6.823389	6.349423	6.595221	6.606167	5.665776	6.455185	6.234661	Slc39a1	NM_013901
6876047	7898	5.854732	5.795047	5.988675	6.12395	-0.26922	0.076549	-0.3289	0.037921	-0.13527	0.336992	-0.19363	0.18191	0.059686	0.664221	-0.13394	0.341493	6876047	5.72859	5.920098	5.915509	5.928888	5.589177	5.867075	5.904303	6.265516	5.796207	6.137552	6.123242	6.111055	Endog	NM_007931
6876052	7899	7.818131	7.779013	7.692885	7.661592	0.15654	0.068345	0.117421	0.152901	0.031293	0.684901	0.086128	0.28008	0.039118	0.613044	0.125247	0.13055	6876052	7.923065	7.785332	7.745997	7.761137	7.89106	7.684842	7.65129	7.820667	7.606697	7.647338	7.641294	7.696143	Phyhd1	NM_172267
6876053	7900	8.004738	8.004794	8.017446	8.007707	-0.00297	0.968533	-0.00291	0.969119	0.009739	0.897077	-0.01265	0.866595	-5.5E-05	0.999413	-0.01271	0.866018	6876053	7.996819	8.00536	8.012036	7.995088	8.050128	7.969165	7.870242	8.190784	7.991313	8.037409	8.050249	7.935463	Nup188	NM_198304
6876055	7901	7.765651	7.712227	7.696608	7.903233	-0.13758	0.262111	-0.19101	0.132482	-0.20662	0.107582	0.015619	0.894445	0.053424	0.651946	0.069043	0.561669	6876055	7.801184	7.691461	7.804309	7.784467	7.781225	7.570989	7.660614	7.928138	7.501072	7.887974	7.800441	8.021284	Fam73b	BC051404
6876058	7902	7.659918	7.562927	7.711409	7.534806	0.125112	0.208822	0.028121	0.766507	0.176603	0.08979	-0.14848	0.143405	0.096991	0.32023	-0.05149	0.589139	6876058	7.671939	7.551076	7.756739	7.550813	7.64783	7.490138	7.850986	7.698723	7.584518	7.421893	7.667556	7.514969	Dolpp1	NM_020329
6876062	7903	8.117621	7.992787	7.966048	7.751581	0.36604	0.015263	0.241206	0.077407	0.214467	0.109406	0.026738	0.82798	0.124835	0.325164	0.151573	0.238845	6876062	8.211428	8.123618	8.017818	7.968031	8.236425	7.773904	8.006062	7.985373	7.90671	7.593876	7.849271	7.811596	Ppp2r4	NM_138748
6876079	7905	7.325688	7.353341	7.192232	7.226099	0.099589	0.11004	0.127242	0.050779	-0.03387	0.558067	0.161109	0.019672	-0.02765	0.631208	0.133456	0.042625	6876079	7.335869	7.25294	7.388255	7.299954	7.325061	7.435008	7.251837	7.145512	7.179346	7.226749	7.301006	7.150542	Mettl11a	NM_170592
6876081	7906	7.422425	6.926125	6.924271	6.535427	0.886998	0.000506	0.390698	0.038707	0.388844	0.039421	0.001855	0.990932	0.4963	0.013853	0.498154	0.01361	6876081	7.268701	7.698634	7.29994	6.719532	7.214138	6.844706	6.970571	6.853179	6.949062	6.439541	6.708742	6.457998	Prrx2	NM_009116
6876087	7907	9.174978	8.927621	8.97538	8.80985	0.365129	0.024697	0.117772	0.399417	0.16553	0.246289	-0.04776	0.727497	0.247357	0.098503	0.199598	0.169876	6876087	9.250322	9.152289	9.122324	8.643095	9.058499	9.08127	9.152882	8.821933	8.951325	8.753378	8.935646	8.740525	Tor1b	NM_133673
6876089	7908	7.491127	7.397523	7.432445	7.379633	0.111494	0.100299	0.01789	0.773248	0.052813	0.404563	-0.03492	0.576694	0.093604	0.157489	0.058681	0.356863	6876089	7.471534	7.516085	7.485761	7.47532	7.420916	7.296332	7.451721	7.486276	7.359339	7.394627	7.462699	7.281572	Usp20	NM_028846
6876100	7909	8.829025	8.832144	8.769936	8.729027	0.099999	0.237039	0.103117	0.223994	0.040909	0.615226	0.062208	0.449494	-0.00312	0.969181	0.059089	0.471748	6876100	8.880488	8.799249	8.807339	8.790419	8.893653	8.81236	8.772684	8.900544	8.63658	8.59194	8.8133	8.78184	Gpr107	NM_178760
6876109	7910	7.403716	7.209428	7.228678	7.220765	0.182951	0.418171	-0.01134	0.959114	0.007913	0.971454	-0.01925	0.930644	0.194288	0.39118	0.175038	0.437774	6876109	7.269856	7.528128	7.413165	7.406243	7.370164	6.851877	7.192271	7.528305	6.965459	7.084611	7.026925	7.550759	Freq	NM_019681
6876136	7911	6.706339	6.472888	6.322932	6.272422	0.433917	0.032748	0.200466	0.267885	0.05051	0.771807	0.149956	0.399075	0.233451	0.202959	0.383407	0.052285	6876136	6.773247	6.386895	6.958874	6.59592	6.656879	6.165865	6.340896	6.417908	6.209992	6.226663	6.269512	6.32109	Hmcn2	ENSMUST000000
6876150	7912	7.49786	7.576937	7.625476	7.523772	-0.02591	0.689038	0.053165	0.41926	0.101703	0.14199	-0.04854	0.459298	-0.07908	0.240966	-0.12762	0.075219	6876150	7.510301	7.499539	7.483739	7.487212	7.71925	7.524349	7.65556	7.691771	7.529096	7.523134	7.543666	7.504517	Exosc2	NM_144886
6876154	7913	8.169257	8.319334	8.209859	8.157875	0.011381	0.926873	0.161459	0.215939	0.051983	0.676739	0.109475	0.388912	-0.15008	0.24701	-0.0406	0.744152	6876154	8.25832	8.156129	8.093321	8.312326	8.407639	8.238037	8.251963	8.257403	8.12021	7.86032	8.300364	8.312942	Abl1	NM_001112703
6876159	7914	6.849185	6.816336	6.871054	6.916325	-0.06714	0.543454	-0.09999	0.372335	-0.04527	0.680045	-0.05472	0.619061	0.032849	0.764155	-0.02187	0.841427	6876159	7.002254	6.840766	6.704535	6.883101	6.939432	6.626475	6.928423	6.937025	6.747713	6.990438	6.839413	6.919125	Lamc3	NM_011836
6876161	7915	10.02851	10.37456	10.29437	10.51089	-0.48237	0.188493	-0.13632	0.695194	-0.21652	0.536817	0.080193	0.817118	-0.34605	0.332565	-0.26586	0.451033	6876161	10.24908	9.878812	9.957652	10.41072	10.52557	10.1874	10.33007	10.46175	10.09129	10.17722	9.978316	11.37712	Aif1l	NM_145144
6876162	7916	8.933779	8.853603	8.863161	8.966174	-0.03239	0.698595	-0.11257	0.200513	-0.10301	0.23755	-0.00956	0.908638	0.080176	0.349521	0.070618	0.406999	6876162	8.963404	8.927086	8.910847	8.833459	8.944854	8.782497	8.730485	9.02053	8.838467	8.900077	8.917465	9.080979	Nup214	NM_172268
6876171	7917	6.380911	6.101717	6.059216	6.063767	0.317144	0.184967	0.03795	0.866515	-0.00455	0.983903	0.042501	0.85072	0.279194	0.237439	0.321695	0.179413	6876171	6.142014	6.117392	6.883327	6.0919	6.219494	5.993757	6.323651	6.062649	5.791348	6.145306	5.929623	6.116372	Ppapdc3	NM_145521
6876173	7918	10.34675	10.46381	10.18155	10.26195	0.084797	0.656542	0.201857	0.303641	-0.08041	0.673067	0.282263	0.162824	-0.11706	0.541632	0.165203	0.394587	6876173	10.34222	10.43748	10.26055	10.44207	10.52531	10.42405	9.796921	10.61651	10.13121	10.09185	10.35291	10.3411	Bat2l	NM_001159634
6876175	7919	7.956498	7.862534	7.898397	7.748554	0.207944	0.113814	0.11398	0.359083	0.149843	0.236728	-0.03586	0.767324	0.093964	0.445677	0.058101	0.633264	6876175	7.8688	7.934416	8.066278	7.878439	8.004026	7.705136	8.059912	7.778203	7.857075	7.612806	7.694912	7.937944	Pomt1	NM_145145
6876181	7920	8.430385	8.578312	8.441097	8.701959	-0.27157	0.098403	-0.12365	0.419293	-0.26086	0.11018	0.137215	0.372394	-0.14793	0.338201	-0.01071	0.943011	6876181	8.378607	8.500309	8.412239	8.686917	8.422889	8.625131	8.435327	8.471834	8.41613	8.488573	8.546795	9.070509	Golga2	NM_133852
6876185	7921	8.897842	8.925889	8.895047	8.725834	0.172007	0.034038	0.200055	0.017904	0.169213	0.036308	0.030842	0.659347	-0.02805	0.68819	0.002795	0.967935	6876185	8.878926	8.880279	8.93432	8.924434	9.068336	8.784897	8.82682	8.921909	8.936412	8.686359	8.707519	8.783625	Ciz1	NM_028412
6876188	7922	6.826484	6.975563	7.059666	6.947322	-0.12084	0.319571	0.028242	0.810362	0.112345	0.352716	-0.0841	0.481246	-0.14908	0.2268	-0.23318	0.074755	6876188	6.74911	6.834255	6.896087	6.927583	7.072324	6.926783	7.07166	7.15952	6.947819	6.73366	6.913532	7.194773	Ptges2	NM_133783
6876194	7923	6.037537	6.196866	6.132024	6.178112	-0.14058	0.260809	0.018753	0.875764	-0.04609	0.701952	0.064842	0.592025	-0.15933	0.207468	-0.09449	0.439575	6876194	5.96057	5.958735	6.193305	6.148713	6.382853	6.059031	6.139082	6.060831	6.196158	6.355569	6.170593	6.008175	Naif1	NM_194335
6876205	7924	9.810627	9.672558	9.75785	9.734054	0.076573	0.655984	-0.0615	0.719913	0.023796	0.88925	-0.08529	0.620308	0.138068	0.428435	0.052777	0.75802	6876205	9.977035	9.736529	9.718316	9.780034	9.86807	9.369571	9.695797	10.02104	9.556712	9.628863	9.699012	9.874286	Fam102a	NM_153560
6876206	7925	9.378289	9.200419	9.40977	9.248469	0.12982	0.21461	-0.04805	0.631277	0.1613	0.132511	-0.20935	0.061466	0.17787	0.101975	-0.03148	0.752161	6876206	9.363573	9.341568	9.429726	9.205375	9.40133	8.994553	9.383422	9.49682	9.349067	9.200784	9.339256	9.205368	Dpm2	NM_010073
6876209	7927	7.039391	7.103253	6.973212	6.520413	0.518978	0.029228	0.58284	0.017687	0.452799	0.049487	0.130041	0.525259	-0.06386	0.752667	0.066179	0.744062	6876209	7.069865	7.070694	6.977614	7.25295	7.192684	6.864125	6.935288	6.933686	7.050662	6.517505	6.944777	6.098958	St6galna	NM_011373
6876210	7928	7.163221	7.215632	6.860745	6.419261	0.743961	0.003951	0.796371	0.002682	0.441484	0.044999	0.354887	0.092824	-0.05241	0.785271	0.302476	0.142564	6876210	7.360458	6.993223	7.135983	7.235425	7.453238	6.958233	7.065458	6.721028	6.795749	6.38853	6.714319	6.154933	St6galna	NM_016973
6876211	7929	8.830075	8.497949	8.367671	8.190813	0.639262	0.090268	0.307136	0.381833	0.176858	0.608608	0.130278	0.704924	0.332126	0.346286	0.462404	0.201051	6876211	8.644099	8.463333	9.382793	8.85946	8.683021	7.951367	8.593022	8.406622	8.103369	7.848285	8.568815	8.155339	Ak1	NM_021515
6876212	7930	10.83686	10.88931	10.82284	10.71705	0.11981	0.584515	0.17226	0.436442	0.105783	0.628511	0.066477	0.760014	-0.05245	0.809332	0.014027	0.948459	6876212	11.01554	10.96784	10.52721	11.09908	10.86456	10.7043	11.04197	10.70743	10.71911	10.34983	11.0282	10.77313	Eng	NM_001146350
6876217	7931	7.602699	7.805031	7.564907	7.744101	-0.1414	0.137933	0.06093	0.497759	-0.17919	0.070164	0.240124	0.023239	-0.20233	0.046076	0.037792	0.671233	6876217	7.674782	7.568302	7.565014	7.887263	7.93795	7.589881	7.556498	7.503141	7.635082	7.725729	7.737605	7.768969	Sh2d3c	NM_013781
6876218	7932	7.011704	7.13047	7.068129	6.918758	0.092946	0.562592	0.211712	0.206189	0.149371	0.360161	0.062342	0.696018	-0.11877	0.462444	-0.05642	0.723385	6876218	7.03152	6.93935	7.064242	7.008323	7.452368	6.93072	7.331305	6.871865	7.001216	6.872182	6.972459	6.911633	Tor2a	NM_152800
6876219	7933	6.765468	6.606181	6.59552	6.698119	0.06735	0.591823	-0.09194	0.467746	-0.1026	0.419656	0.010661	0.931731	0.159287	0.223108	0.169949	0.19645	6876219	6.80243	6.707624	6.786351	6.552452	6.738458	6.527633	6.5304	6.74119	6.514969	6.655609	6.951943	6.486804	Ptrh1	NM_178595
6876226	7934	8.660873	8.439171	8.488641	8.03412	0.626754	0.030245	0.405052	0.12779	0.454521	0.093068	-0.04947	0.840825	0.221702	0.379671	0.172233	0.49066	6876226	8.822072	8.570891	8.589657	8.527391	8.637257	8.152866	8.738708	8.343412	8.383802	7.926076	8.53645	7.639833	Fam129b	NM_146119
6876238	7935	9.551423	9.267828	8.90318	8.762594	0.788829	0.036975	0.505234	0.148077	0.140586	0.667801	0.364648	0.281266	0.283595	0.395115	0.648243	0.074049	6876238	9.523698	9.440299	9.690273	9.362133	9.425879	9.015473	9.358177	8.357735	8.993628	8.158954	9.083681	9.048117	Angptl2	NM_011923
6876310	7937	9.074263	9.008537	9.05036	8.865261	0.209002	0.01471	0.143275	0.066437	0.185098	0.025305	-0.04182	0.552545	0.065727	0.358464	0.023904	0.732257	6876310	9.055571	9.068241	9.098978	8.921661	9.117516	8.986433	9.092771	8.986953	9.071355	8.751554	8.98557	8.85866	Mapkap1	NM_177345
6876342	7938	12.33606	12.14552	12.66368	12.35206	-0.016	0.94882	-0.20654	0.417537	0.311617	0.233214	-0.51816	0.064335	0.19054	0.453109	-0.32762	0.212177	6876342	12.34442	12.37188	12.29188	11.76329	12.28593	12.38734	12.77544	12.68235	12.53325	12.52693	12.71035	11.81891	Hspa5	NM_022310
6876357	7939	5.949555	5.95303	5.977672	5.725122	0.224433	0.347531	0.227907	0.340538	0.25255	0.29402	-0.02464	0.915446	-0.00347	0.988053	-0.02812	0.90359	6876357	5.757329	5.755558	6.335779	5.854505	5.701476	6.303108	6.248292	5.933207	5.751518	5.703744	5.561746	5.909877	D730039F	NM_030021
6876369	7940	8.211402	8.031377	8.091904	7.855117	0.356286	0.026353	0.176261	0.215704	0.236787	0.108552	-0.06053	0.656631	0.180025	0.206981	0.119499	0.388706	6876369	8.42534	8.095423	8.113444	7.991525	8.222972	7.879635	8.300279	8.025889	7.949543	7.791627	7.918885	7.854838	Cep110	NM_012018
6876380	7941	10.60063	10.24917	10.05934	9.664194	0.936432	0.028676	0.584976	0.134787	0.395142	0.293745	0.189834	0.604014	0.351457	0.346854	0.541291	0.162305	6876380	10.38165	10.55516	10.86507	10.12671	10.40949	10.21131	10.39354	9.749138	10.03533	9.089724	10.50747	9.395389	Gsn	NM_146120
6876405	7942	8.55397	8.5163	8.454274	8.640091	-0.08612	0.410061	-0.12379	0.246841	-0.18582	0.097595	0.062026	0.548748	0.03767	0.713701	0.099696	0.34378	6876405	8.586791	8.519389	8.55573	8.679818	8.600704	8.268379	8.363604	8.536595	8.462624	8.603956	8.69801	8.618306	Dab2ip	NM_001114124
6876423	7944	8.064143	7.988179	8.097207	7.941523	0.12262	0.432435	0.046656	0.761181	0.155684	0.324631	-0.10903	0.483351	0.075964	0.622433	-0.03306	0.829211	6876423	7.848567	8.28459	8.059272	7.881197	8.207183	7.876157	8.186503	8.130456	7.974662	7.768818	7.909151	8.1466	Mrrf	NM_026422
6876430	7945	10.89741	10.48803	10.11379	10.02527	0.872136	0.116518	0.462759	0.377803	0.088524	0.86269	0.374235	0.471862	0.409377	0.432774	0.783612	0.152535	6876430	11.2917	10.46701	10.93351	10.25446	11.14296	10.06667	11.00512	9.279474	10.05679	10.57062	9.702526	9.802667	Ptgs1	NM_008969
6876486	7963	8.172717	8.479114	8.199649	8.152942	0.019775	0.760984	0.326172	0.00083	0.046707	0.478457	0.279465	0.002144	-0.3064	0.001229	-0.02693	0.679453	6876486	8.205785	8.259104	8.053263	8.404767	8.514844	8.517731	8.29594	8.12326	8.179747	8.132017	8.157146	8.169664	Rabgap1	NM_146121
6876543	7966	8.192858	8.62026	8.219998	8.656637	-0.46378	0.05263	-0.03638	0.862919	-0.43664	0.064762	0.400262	0.085415	-0.4274	0.069491	-0.02714	0.897461	6876543	8.319111	8.219213	8.04025	8.631757	8.685727	8.543295	8.041692	8.401888	8.216413	8.431187	8.376863	9.161862	Nek6	NM_001159631
6876567	7967	7.532005	7.856476	7.266957	7.764897	-0.23289	0.407575	0.091579	0.739937	-0.49794	0.098599	0.589519	0.057862	-0.32447	0.258027	0.265048	0.34901	6876567	7.149816	7.97506	7.47114	7.678809	8.016338	7.874282	7.199813	7.216966	7.384092	7.447488	8.295518	7.551685	Olfml2a	NM_172854
6876570	7969	7.590111	7.617016	7.559216	7.492505	0.097605	0.346876	0.124511	0.238136	0.066711	0.513864	0.0578	0.57032	-0.02691	0.789927	0.030894	0.759851	6876570	7.601909	7.586884	7.581539	7.498732	7.745393	7.606923	7.604681	7.672481	7.400487	7.376922	7.441815	7.658779	Arpc5l	NM_028809
6876740	7971	6.11688	5.968883	6.031365	5.394555	0.722325	0.008019	0.574328	0.023671	0.63681	0.014887	-0.06248	0.769478	0.147997	0.4931	0.085515	0.689076	6876740	6.5802	5.91239	5.858051	5.987533	6.128121	5.790995	6.258008	5.880338	5.955749	5.254312	5.563198	5.366155	Arhgap15	NM_153820
6876914	7972	7.273474	7.469227	7.385885	7.430464	-0.15699	0.16095	0.038764	0.71279	-0.04458	0.672494	0.083342	0.435882	-0.19575	0.090224	-0.11241	0.300785	6876914	7.217024	7.297944	7.305454	7.341085	7.538411	7.528186	7.555569	7.363121	7.238966	7.26511	7.477687	7.548594	Acvr2a	NM_007396
6876937	7973	8.17927	8.436982	8.161288	8.345482	-0.16621	0.146355	0.0915	0.40168	-0.18419	0.112485	0.275694	0.028438	-0.25771	0.037275	0.017982	0.866157	6876937	8.11786	8.225587	8.194364	8.430277	8.45575	8.42492	8.328739	8.058472	8.096653	8.26337	8.201447	8.57163	Epc2	NM_172663
6876943	7974	5.676978	6.474141	5.814382	6.604156	-0.92718	0.014202	-0.13002	0.673191	-0.78977	0.028863	0.659759	0.057091	-0.79716	0.027772	-0.1374	0.656003	6876943	5.753942	5.535899	5.741094	6.917758	6.205257	6.299408	5.760576	6.137197	5.545373	6.573022	6.096086	7.143361	Kif5c	NM_008449
6876944	7975	7.208855	7.478486	7.477138	7.73636	-0.5275	0.084632	-0.25787	0.364236	-0.25922	0.361866	0.001347	0.996113	-0.26963	0.343951	-0.26828	0.346231	6876944	6.897789	7.347054	7.381723	7.810069	6.989661	7.635727	7.427366	7.792692	7.211357	7.523856	7.616182	8.069043	Lypd6b	NM_027990
6876947	7976	6.267813	7.853969	6.196377	7.258386	-0.99057	0.000221	0.595583	0.005109	-1.06201	0.000137	1.657592	5.39E-06	-1.58616	7.5E-06	0.071436	0.659235	6876947	6.348227	6.056068	6.399143	7.995	7.730197	7.836709	6.093371	6.290255	6.205505	7.028848	7.161589	7.58472	Lypd6	NM_177139
6877080	7979	7.487773	7.545865	7.609425	7.623128	-0.13536	0.22437	-0.07726	0.47379	-0.0137	0.897245	-0.06356	0.553538	-0.05809	0.58746	-0.12165	0.27058	6877080	7.384914	7.612815	7.465589	7.368354	7.717992	7.55125	7.698614	7.648784	7.480878	7.536448	7.694895	7.63804	Rif1	NM_175238

6877139	7980	9.311801	9.316487	9.276944	9.361083	-0.04928	0.603525	-0.0446	0.637853	-0.08414	0.383044	0.039543	0.675931	-0.00469	0.960264	0.034857	0.712164	6877139	9.254479	9.344754	9.33617	9.365544	9.217776	9.366142	9.219572	9.366034	9.245226	9.259468	9.249977	9.573804	Fmnl2	NM_172409
6877157	7981	6.924441	7.038643	7.087564	7.357846	-0.4334	0.017923	-0.3192	0.060294	-0.27028	0.101323	-0.04892	0.746217	-0.1142	0.456682	-0.16312	0.296366	6877157	6.731939	7.144269	6.897116	6.844829	7.002149	7.26895	7.161601	7.098638	7.002453	7.474117	7.150258	7.449162	Arl6ip6	NM_022989
6877297	7984	9.030287	8.961863	9.241974	9.596686	-0.5664	0.107932	-0.63482	0.077051	-0.35471	0.289879	-0.28011	0.396912	0.068424	0.832415	-0.21169	0.517873	6877297	8.832398	9.017528	9.240934	9.189962	8.85257	8.843056	9.436152	9.276945	9.012825	9.121927	9.293651	10.37448	Gpd2	NM_001145820
6877356	7985	5.821909	5.690582	6.161375	5.583692	0.238217	0.170226	0.10689	0.517966	0.577683	0.006451	-0.47079	0.017644	0.131327	0.430173	-0.33947	0.064017	6877356	5.700758	5.620046	6.144923	5.829098	5.54677	5.695879	6.265354	6.209862	6.008908	5.769516	5.568127	5.413433	Galnt5	NM_172855
6877428	7987	9.664522	9.603698	9.46307	9.031682	0.63284	0.009994	0.572016	0.016228	0.431388	0.05146	0.140629	0.477171	0.060823	0.755316	0.201452	0.316576	6877428	9.692867	9.578877	9.721821	9.427895	9.931819	9.451381	9.673668	9.118143	9.597398	9.039275	9.217753	8.838018	Pkp4	NM_026361
6877441	7988	8.163701	8.298892	8.225859	8.198479	-0.03478	0.693949	0.100414	0.272605	0.027381	0.756255	0.073033	0.416446	-0.13519	0.151366	-0.06216	0.486628	6877441	8.197397	8.256991	8.036714	8.2105	8.311561	8.374616	8.20045	8.125298	8.35183	8.085424	8.284196	8.225816	Tanc1	NM_198294
6877478	7989	8.310544	8.535802	8.411327	8.353937	-0.04339	0.712301	0.181865	0.147903	0.05739	0.626892	0.124475	0.304883	-0.22526	0.082563	-0.10078	0.400668	6877478	8.382988	8.309623	8.239021	8.476562	8.670702	8.460141	8.585837	8.196831	8.451313	8.194159	8.450707	8.416945	7-Mar	NM_020575
6877584	7990	8.115017	7.878012	8.143191	7.670075	0.444943	0.030139	0.207937	0.253822	0.473116	0.02329	-0.26518	0.155532	0.237005	0.198689	-0.02817	0.871831	6877584	8.427604	8.047934	7.869514	7.698645	8.172799	7.762592	8.239572	8.021552	8.168449	7.652074	7.786679	7.571471	Tank	NM_011529
6877587	7991	9.603977	9.567508	9.651103	9.489756	0.114221	0.449776	0.077752	0.603312	0.161347	0.294231	-0.0836	0.576886	0.036469	0.806124	-0.04713	0.751445	6877587	9.573048	9.604319	9.634563	9.393301	9.742329	9.566893	9.896485	9.506444	9.550381	9.251624	9.675198	9.542446	Psmd14	NM_021526
6877608	7994	9.635665	9.529125	9.659971	9.754825	-0.11916	0.397161	-0.2257	0.12866	-0.09485	0.496671	-0.13085	0.354751	0.10654	0.446937	-0.02431	0.859758	6877608	9.667714	9.514061	9.725219	9.290982	9.701737	9.594656	9.520184	9.842466	9.617263	9.651752	9.688664	9.924058	Gca	NM_145523
6877822	7996	5.798089	6.00192	6.027878	4.744081	1.054007	0.003334	1.257839	0.001165	1.283797	0.001026	-0.02596	0.921636	-0.20383	0.448367	-0.22979	0.395065	6877822	6.08543	5.213752	6.095084	5.740211	6.055559	6.20999	6.207534	5.828148	6.047953	4.718204	4.55195	4.96209	B3galt1	NM_020283
6877895	7999	10.97605	10.86538	11.32363	11.79792	-0.82187	0.004019	-0.93254	0.001938	-0.47429	0.050376	-0.45825	0.056884	0.11067	0.605905	-0.34758	0.130205	6877895	10.91733	11.06474	10.94609	10.97524	10.45177	11.16914	11.24008	11.66505	11.06576	11.68235	11.77808	11.93334	Lass6	NM_172856
6877901	8000	7.546121	7.58576	7.216631	7.512413	0.033708	0.832303	0.073347	0.64677	-0.29578	0.091153	0.369129	0.043462	-0.03964	0.803462	0.32949	0.064927	6877901	7.743176	7.592882	7.302304	7.6843	7.539201	7.533778	7.293758	7.035649	7.320486	7.237341	7.708416	7.591482	Nostrin	NM_181547
6877924	8003	6.21361	6.347039	6.199477	6.164798	0.048812	0.689786	0.182241	0.160808	0.034679	0.776171	0.147562	0.24614	-0.13343	0.290601	0.014133	0.907553	6877924	6.180181	6.34809	6.112559	6.223296	6.538277	6.279545	6.188267	6.401468	6.008696	6.207583	6.121824	6.164987	Bbs5	NM_028284
6877931	8004	7.184686	7.156175	7.418256	7.281541	-0.09685	0.484985	-0.12537	0.371053	0.136715	0.331628	-0.26208	0.082911	0.028511	0.834757	-0.23357	0.115465	6877931	7.029806	7.282814	7.241439	7.025406	7.285829	7.15729	7.445841	7.663008	7.145918	7.230202	7.291737	7.322683	Phospho2	NM_028521
6877940	8006	8.368697	8.452353	8.405304	8.493763	-0.12507	0.24598	-0.04141	0.6894	-0.08846	0.401748	0.047049	0.650256	-0.08366	0.426691	-0.03661	0.723549	6877940	8.334206	8.318177	8.453708	8.468115	8.438935	8.45001	8.617809	8.372744	8.22536	8.394754	8.456481	8.630055	Ubr3	NM_001081548
6877952	8007	8.738904	8.869694	8.716682	8.790708	-0.0518	0.667485	0.078986	0.51577	-0.07403	0.541807	0.153012	0.224292	-0.13079	0.292894	0.022222	0.853076	6877952	8.708719	8.770478	8.737514	8.890674	8.833325	8.885083	8.791064	8.80154	8.557442	8.517519	8.863992	8.990613	Ubr3	NM_001081548
6877958	8009	5.819163	5.889638	5.831254	5.952868	-0.1337	0.349865	-0.06323	0.65123	-0.12161	0.392874	0.058384	0.676076	-0.07047	0.614934	-0.01209	0.930669	6877958	5.92542	5.78461	5.74746	5.829505	5.879159	5.960251	5.979622	6.010263	5.503877	6.039103	5.81253	6.006971	Sp5	NM_022435
6877962	8010	6.150605	6.036085	5.944682	5.284486	0.866119	0.023271	0.751599	0.04133	0.660196	0.065513	0.091403	0.775303	0.11452	0.72102	0.205923	0.524616	6877962	6.391403	5.804673	6.25574	5.977213	6.343129	5.787913	6.533829	5.307349	5.992868	5.45323	5.158147	5.242081	4933404M	NM_025744
6877964	8012	10.43912	10.352	10.51145	10.45478	-0.01567	0.878337	-0.10278	0.330129	0.056663	0.583278	-0.15944	0.1464	0.087113	0.405131	-0.07233	0.486407	6877964	10.38618	10.36855	10.56262	10.31361	10.49198	10.25042	10.47875	10.62546	10.43013	10.41061	10.61809	10.33565	Gorasp2	NR_027343
6877988	8013	7.530953	7.498171	7.536147	7.611121	-0.08017	0.300885	-0.11295	0.157798	-0.07497	0.331241	-0.03798	0.614544	0.032782	0.663102	-0.00519	0.944632	6877988	7.487372	7.664319	7.441168	7.430689	7.45793	7.605895	7.449799	7.53648	7.622163	7.569893	7.623931	7.639538	4833418A	BC031546
6877990	8014	11.34697	10.65866	11.05527	12.00889	-0.66191	0.023519	-1.35022	0.000458	-0.95362	0.003835	-0.3966	0.132996	0.68831	0.019819	0.291707	0.253627	6877990	11.11973	11.32279	11.5984	10.72123	10.47767	10.77709	10.58133	11.55698	11.02749	11.91072	11.96522	12.15072	Cybrd1	NM_028593
6878000	8015	9.578708	9.559156	9.646971	9.364405	0.214303	0.159967	0.194751	0.196868	0.282566	0.075387	-0.08781	0.543318	0.019552	0.891105	-0.06826	0.634979	6878000	9.589081	9.556533	9.590511	9.464911	9.719777	9.49278	9.920386	9.352818	9.667708	9.229214	9.451052	9.412949	Dync1i2	NM_010064
6878012	8016	7.212643	7.422461	7.342895	7.18883	0.023813	0.88351	0.233632	0.176078	0.154065	0.356408	0.079566	0.626886	-0.20982	0.219296	-0.13025	0.431996	6878012	7.220162	7.145514	7.272252	7.199917	7.586355	7.481112	7.671139	7.107686	7.24986	7.048766	7.194468	7.323255	Hat1	NM_026115
6878016	8017	6.816352	6.429893	6.877636	7.113218	-0.29687	0.008137	-0.68333	4.19E-05	-0.23558	0.024162	-0.44774	0.000754	0.386459	0.001875	-0.06128	0.491137	6878016	6.86858	6.855557	6.72492	6.367425	6.383063	6.539191	6.848767	6.999533	6.784609	7.002725	7.08708	7.24985	Metapl1	NM_025633
6878031	8020	10.10769	9.956049	9.945665	9.911375	0.196315	0.185843	0.044674	0.750353	0.03429	0.806804	0.010384	0.940859	0.151641	0.296041	0.162025	0.266499	6878031	10.03575	10.11372	10.1736	9.712867	10.13483	10.02045	10.0603	9.749155	10.02754	9.802405	10.10678	9.824941	Itga6	NM_008397
6878035	8021	7.405361	7.774019	7.446206	7.978462	-0.5731	0.084766	-0.20444	0.502819	-0.53226	0.105183	0.327813	0.29321	-0.36866	0.241418	-0.04084	0.891989	6878035	7.481831	7.331509	7.402744	8.208003	7.380792	7.733263	7.266173	7.753976	7.31847	7.866242	7.534259	8.534886	Pdk1	NM_172665
6878038	8022	6.768644	7.279734	6.858712	7.070232	-0.30159	0.123998	0.209503	0.266717	-0.21152	0.262494	0.421023	0.043225	-0.51109	0.019513	-0.09007	0.621633	6878038	6.714506	6.952714	6.638712	7.290325	7.169322	7.379556	6.776317	6.693145	7.106673	6.70755	7.234883	7.268262	Rapgef4	NM_019688
6878045	8023	6.05716	6.08129	5.528888	6.441694	-0.38453	0.403617	-0.3604	0.432513	-0.91281	0.069677	0.552402	0.240895	-0.02413	0.957231	0.528272	0.260319	6878045	6.17174	6.32599	5.673751	6.217129	5.83558	6.191162	4.517467	5.984394	6.084803	5.973263	6.888283	6.463537	Rapgef4	AK032089
6878049	8024	8.201043	8.409115	8.151728	7.930993	0.270049	0.106863	0.478122	0.012319	0.220734	0.175953	0.257387	0.12168	-0.20807	0.199247	0.049315	0.748657	6878049	8.095384	8.109675	8.398069	8.50773	8.340075	8.37954	8.296174	8.106609	8.0524	7.605599	8.093941	8.09344	B230120H	NM_023057
6878053	8025	6.854215	7.215938	6.94075	6.613399	0.240815	0.038868	0.602538	0.000267	0.32735	0.01002	0.275188	0.022508	-0.36172	0.005989	-0.08654	0.40114	6878053	6.765252	7.030016	6.767376	7.154339	7.211838	7.281636	7.004039	7.042005	6.776205	6.722685	6.57563	6.541883	Cdca7	NM_025866
6878117	8026	8.434417	8.598341	8.480694	8.718712	-0.2843	0.096041	-0.12037	0.447654	-0.23802	0.153024	0.117646	0.45763	-0.16392	0.30855	-0.04628	0.766703	6878117	8.289014	8.367262	8.646975	8.600276	8.571003	8.623743	8.544592	8.63271	8.264781	8.492742	8.673445	8.98995	1700011J	ENSMUST00000
6878138	8027	6.418276	6.505351	6.533373	6.46073	-0.04245	0.500329	0.044621	0.479382	0.072643	0.261635	-0.02802	0.653719	-0.08708	0.185731	-0.1151	0.092023	6878138	6.455551	6.315631	6.483645	6.513428	6.45925	6.543375	6.586046	6.554671	6.459402	6.484553	6.532565	6.365071	Sp9	NM_001005343
6878265	8030	6.161822	6.201466	6.176935	6.258828	-0.09701	0.364067	-0.05736	0.584945	-0.08189	0.440065	0.024531	0.813852	-0.03964	0.704383	-0.01511	0.884534	6878265	6.224193	6.096153	6.16512	6.158453	6.209949	6.235997	6.383959	6.217598	5.929248	6.209718	6.259626	6.307139	Hoxd10	NM_013554
6878296	8035	8.183748	8.323854	8.317468	8.412176	-0.22843	0.046495	-0.08832	0.389604	-0.09471	0.357935	0.006386	0.949178	-0.14011	0.187025	-0.13372	0.205761	6878296	8.090652	8.247762	8.212831	8.238498	8.398561	8.334502	8.431513	8.30896	8.211931	8.212983	8.474283	8.549261	Mtx2	NM_016804
6878329	8036	8.445282	8.406295	8.380982	8.278954	0.166329	0.251073	0.127342	0.371241	0.102028	0.46965	0.025314	0.85533	0.038987	0.779183	0.064301	0.645227	6878329	8.508849	8.381426	8.445572	8.26278	8.559367	8.396739	8.55884	8.246111	8.337994	8.110246	8.550742	8.175873	Agps	NM_172666
6878341	8037	7.991909	8.164005	8.070111	7.843761	0.148148	0.20349	0.320245	0.01724	0.22635	0.067256	0.093894	0.405692	-0.1721	0.146345	-0.0782	0.485627	6878341	7.892248	8.080025	8.003454	8.074153	8.252328	8.165535	8.245359	7.885718	8.079256	7.698977	7.976912	7.855393	Rbm45	NM_153405
6878347	8039	10.01189	9.989262	9.972614	9.99072	0.021165	0.816587	-0.00146	0.987227	-0.01811	0.842644	0.016648	0.855143	0.022623	0.804247	0.039271	0.668276	6878347	10.01167	10.10021	9.923776	9.912428	10.09294	9.962418	10.11658	9.971923	9.829339	9.880321	10.06917	10.02267	Plekha3	NM_031256
6878430	8041	8.796827	8.902883	8.833133	8.694325	0.102502	0.268011	0.208558	0.041714	0.138808	0.145618	0.06975	0.441341	-0.10606	0.253041	-0.03631	0.684389	6878430	8.918148	8.740702	8.73163	8.892368	8.979266	8.837015	8.985456	8.846828	8.667114	8.637096	8.741708	8.704171	Ube2e3	NM_009454
6878448	8042	9.1737	9.037474	8.937618	7.753622	1.420078	0.005481	1.283852	0.00927	1.183997	0.013772	0.099856	0.797742	0.136226	0.727115	0.236081	0.548494	6878448	9.069221	9.252439	9.199439	8.769275	9.214368	9.128779	9.313909	8.270086	9.22886	7.614274	8.484332	7.162259	Itga4	NM_010576
6878469	8043	8.299073	8.153826	8.545295	8.229067	0.070006	0.650223	-0.07524	0.626365	0.316228	0.066046	-0.39147	0.030004	0.145247	0.357093	-0.24622	0.136204	6878469	8.349519	8.379253	8.168447	8.247197	8.178073	8.036209	8.757122	8.665811	8.212952	8.057464	8.345842	8.283894	Ssfa2	NM_080558
6878511	8046	8.886633	9.027038	8.932905	8.710448	0.176185	0.213252	0.31659	0.041201	0.222457	0.126114	0.094133	0.490551	-0.1404	0.312592	-0.04627	0.731647	6878511	8.997894	8.866394	8.795612	8.97214	9.174655	8.934319	9.218522	8.786457	8.793737	8.57674	8.777504	8.7771	Dnajc10	NM_024181
6878548	8047	6.934834	7.125	6.985192	6.786767	0.148067	0.358693	0.338233	0.056792	0.198425	0.228195	0.139808	0.384747	-0.19017	0.24641	-0.05036	0.749019	6878548	6.80903	7.127406	6.868067	7.065372	7.115095	7.194533	6.943871	6.943517	7.068188	6.437135	6.866975	7.056191	Dusp19	NM_024438
6878643	8048	9.139233	8.973086	9.058997	9.082632	0.056601	0.624501	-0.10955	0.353432	-0.02363	0.837004	-0.08591	0.461999	0.166148	0.173512	0.080236	0.491136	6878643	8.974373	9.219087	9.22424	8.86597	9.088572	8.964715	9.109085	9.208193	8.859713	9.045151	9.19122	9.011525	Zc3h15	NM_026934
6878655	8049	10.79223	10.37846	10.60201	10.39858	0.39365	0.083186	-0.02012	0.921924	0.20343	0.33639	-0.22355	0.293679	0.41377	0.071106	0.19022	0.36694	6878655	10.96557	10.88712	10.52399	10.16484	10.68289	10.28764	10.89078	10.38842	10.52682	10.44528	10.57439	10.17606	Itgav	NM_008402
6878694	8051	8.733362	8.649404	8.720108	8.652009	0.081353	0.531571	-0.0026	0.983812	0.068099	0.599107	-0.0707	0.585471	0.083958	0.518856	0.013254	0.917793	6878694	8.669127	8.67152	8.85944	8.455638	8.848319	8.644256	8.713101	8.91798	8.529244	8.660598	8.71503	8.580399	Med19	NM_025885
6878702	8052	6.704359	7.114811	6.563664	6.169598	0.534761	0.122508	0.945213	0.015776	0.394065	0.238983	0.551147	0.113031	-0.41045	0.221683	0.140696	0.661703	6878702	6.929974	6.619021	6.564083	6.886602	7.802886	6.654945	6.573416	6.16567	6.951905	6.117606	6.302683	6.088506	Ypel4	NM_001005342
6878709	8053	8.183765	8.08596	8.014981	7.24476	0.939005	0.027511	0.8412	0.042541	0.770221	0.058426	0.070978	0.84397	0.097805	0.78647	0.168783	0.641725	6878709	8.348558	8.158217	8.044519	7.776367	8.546722	7.93479	8.588782	7.417432	8.03873	7.062202	7.752887	6.919191	Ube2l6	NM_019949
6878710	8054	6.640248	6.843129	6.710263	6.695583	-0.05534	0.764359	0.147545	0.432196	0.014679	0.936444	0.132866	0.47773	-0.20288	0.288368	-0.07001	0.704975	6878710	6.607459	6.913571	6.399714	6.987171	6.86707	6.675145	6.794355	6.953187	6.383246	6.564799	6.767278	6.754673	Timm10	NM_013899
6878713	8056	10.22157	11.15122	10.40294	10.01942	0.202141	0.380268	1.131796	0.000823	0.383519	0.116129	0.748277	0.008858	-0.92965	0.002722	-0.18138	0.428901	6878713	10.66354	10.17262	9.828535	11.24346	10.94379	11.26641	10.4608	10.29816	10.44987	10.02237	10.27785	9.758052	Slc43a3	NM_021398
6878730	8059	8.968036	9.06948	9.017897	8.9206	0.047436	0.584477	0.14888	0.111538	0.097296	0.276184	0.051584	0.552762	-0.10144	0.257752	-0.04986	0.565813	6878730	8.980433	9.01195	8.911725	9.023757	9.153482	9.031202	9.188599	8.911455	8.953636	8.809364	8.930654	9.021783	Ssrp1	NM_001136081
6878739	8060	8.590005	8.374181	8.225105	7.724688	0.865317	0.117717	0.649493	0.224733	0.500417	0.340402	0.149076	0.770366	0.215824	0.673531	0.3649	0.480921	6878739	9.035277	8.593702	8.141037	8.196027	8.584993	8.341523	8.684369	7.457129	8.533817	6.726696	8.39288	8.054489	Aplnr	NM_011784
6878978	8119	7.445485	7.523649	7.511857	7.280834	0.164651	0.223611	0.242815	0.087608	0.231023	0.101327	0.011792	0.927055	-0.07816	0.5486	-0.06637	0.609328	6878978	7.400543	7.432376	7.503537	7.304789	7.779436	7.486722	7.650043	7.379127	7.506402	7.18382	7.418297	7.240385	Nup160	NM_021512
6878984	8120	8.540889	8.603529	8.551053	8.519464	0.021425	0.774379	0.084065	0.278165	0.031589	0.673533	0.052476	0.488372	-0.06264	0.411221	-0.01016	0.891611	6878984	8.552853	8.51671	8.553104	8.568615	8.753428	8.488544	8.51746	8.641098	8.4946	8.433429	8.588308	8.536654	Fnbp4	NM_018828
6878995	8122	9.15617	9.246077	9.253467	9.266805	-0.11064	0.357939	-0.02073	0.859551	-0.01334	0.909295	-0.00739	0.949652	-0.08991	0.450849	-0.0973	0.415963	6878995	9.13765	9.050206	9.280653	9.202415	9.294398	9.241419	9.489865	9.207565	9.062972	9.129356	9.350391	9.320667	Mtch2	NM_019758
6878999	8123	5.880731	5.980382	6.101167	5.977606	-0.09687	0.277611	0.002776	0.974188	0.123562	0.175629	-0.12079	0.184442	-0.09965	0.265092	-0.22044	0.029222	6878999	5.934566	5.919447	5.788181	5.927505	6.067552	5.946088	6.072949	6.279147	5.951406	5.954605	5.951969	6.026243	C1qtnf4	NM_026161
6879003	8124	7.682851	7.760689	7.66132	7.896699	-0.21385	0.03682	-0.13601	0.150189	-0.23538	0.024903	0.099369	0.278473	-0.07784	0.389048	0.021531	0.807452	6879003	7.669574	7.633391	7.745589	7.661406	7.884549	7.736113	7.548969	7.825981	7.609011	7.816589	7.976722	7.896786	Kbtbd4	NM_025991
6879006	8125	8.999008	9.037312	8.955426	8.975277	0.023731	0.761735	0.062035	0.435877	-0.01985	0.79961	0.081886	0.310532	-0.0383	0.626226	0.043582	0.580327	6879006	9.0124	8.938691	9.045934	8.991593	9.12697	8.993373	8.994026	9.066064	8.806189	8.890097	9.060806	8.974928	Cugbp1	NM_017368
[truncated: 2,077,272 more chars]
